# Exercise and BMI in Overweight and Obese Children and Adolescents: A Systematic Review and Trial Sequential Meta-Analysis

**DOI:** 10.1155/2015/704539

**Published:** 2015-10-22

**Authors:** George A. Kelley, Kristi S. Kelley, Russell R. Pate

**Affiliations:** ^1^Department of Biostatistics, School of Public Health, West Virginia University, Morgantown, WV 26506, USA; ^2^Department of Exercise Science, University of South Carolina, Columbia, SC 29208, USA

## Abstract

*Objective*. Determine the effects of exercise on body mass index (BMI in kg·m^−2^) among overweight and obese children and adolescents. *Methods*. Trial sequential meta-analysis of randomized controlled exercise intervention trials ≥ 4 weeks and published up to November 11, 2014. *Results*. Of the 5,436 citations screened, 20 studies representing 971 boys and girls were included. Average length, frequency, and duration of training were 13 weeks, 3 times per week, for 46 minutes per session. Overall, random-effects models showed that exercise decreased BMI by 3.6% (mean: −1.08; 95% CI: −0.52 to −1.64; *Q* = 231.4; *p* < 0.001; *I* ^2^ = 90.9%; 95% CI: 87.6% to 93.4%; *D* ^2^ = 91.5%). Trial sequential meta-analysis showed that changes in BMI crossed the monitoring boundary for a type 1 error in 2010, remaining stable thereafter. The number needed to treat was 5 while the percentile improvement was 26.9. It was estimated that approximately 2.5 million overweight and obese children in the US and 22.0 million overweight and obese children worldwide could reduce their BMI by participating in a regular exercise program. Overall quality of evidence was rated as moderate. *Conclusions*. Exercise is associated with improvements in BMI among overweight and obese children and adolescents. This trial is registered with PROSPERO Trial Registration #CRD42015017586.

## 1. Introduction

The prevalence of overweight and obesity in children and adolescents is a pandemic problem both in the United States (US) and worldwide. Recently, Ogden et al. reported that the prevalence of overweight and obesity in the US, defined as a body mass index (BMI) in kg·m^−2^ ≥ 85th percentile based on Centers for Disease Control Growth Charts, was 31.8% among children and adolescents 2 to 19 years of age, while the prevalence of obesity, defined as a BMI in kg·m^−2^ ≥ 95th percentile, was 16.9% [1]. When compared to 30 years ago, this represents an obesity prevalence that is more than two times higher in US children and more than four times higher in adolescents [1, 2]. From a worldwide perspective, the prevalence of overweight and obesity in 2013 has been reported to be approximately 23% among children and adolescents in developed countries and 13% among children and adolescents from developing countries [3]. Collectively, this represents an approximate 47% increase in the worldwide prevalence of overweight and obesity among children and adolescents between 1980 and 2013 [3].

The economic costs associated with overweight and obesity among children and adolescents are also substantial. For example, Finkelstein et al. estimated that the incremental lifetime medical cost of an obese 10-year-old child in the US, in relation to a normal weight child who maintained normal weight throughout adulthood, was $19,000 [4]. Based on the current number of obese 10-year-olds in the US, the total direct medical costs associated with obesity were estimated at $14 billion for this age only [4].

The negative health consequences of obesity in children and adolescents are both immediate and long-term. For example, in a population-based sample of US children and adolescents 5 to 17 years of age from the Bogalusa Heart Study, approximately 70% of obese youth had at least one cardiovascular disease risk factor [5]. In addition, obese children and adolescents, in relation to their normal weight peers, suffer from a greater prevalence and/or incidence of other conditions that include, but are not necessarily limited to, musculoskeletal pain, injuries and fractures [6], obstructive sleep apnea [7], and poorer self-esteem and quality of life [8]. From a long-term perspective, overweight and obesity during childhood and adolescence have been shown to track into adulthood [9], thereby placing this population group at an increased risk for premature all-cause mortality [10]. This is a major problem since overweight and obesity have been reported to be the third leading cause of preventable death in the US, responsible for 216,000 deaths in 2005 [10]. Globally, the World Health Organization has estimated that approximately 3.4 million adults die each year as a result of being overweight or obese [11]. The issue of obesity has become so problematic that it is now recognized by the American Medical Association as a disease [12].

Exercise has been recommended for the prevention and treatment of overweight and obesity in children and adolescents [13–18]. In a recent systematic review with meta-analysis of studies published until the year 2012, the investigative team reported a statistically significant decrease of approximately 3% in BMI *z*-score in overweight and obese children and adolescents [19]. However, body mass index BMI in kg·m^−2^ continues to be the most commonly assessed and reported metric and is easily recognized and interpreted by practitioners. Unfortunately, the effects of exercise on BMI in kg·m^−2^ have been underwhelming. For example, with the exception of one previous systematic review with meta-analysis that focused on exercise [20], others reported a nonsignificant decrease in BMI in kg·m^−2^ among children and adolescents [17, 21–23]. However, all five suffer from potential limitations. These include (1) the pooling of a small number of exercise-only studies [17, 21, 22], (2) the inclusion of nonrandomized trials [20, 22], (3) inclusion of children and adolescents who were not overweight or obese [20, 22, 23], and (4) overall quality scores ranging from only 45% to 82% when the Assessment of Multiple Systematic Reviews (AMSTAR) instrument was applied to the studies [24]. In addition, none of the studies used trial sequential analysis, an approach that can provide data regarding (1) adequate information size, (2) a threshold for a statistically significant effect, and (3) a threshold for futility [25]. Given the former, the purpose of the current study was to conduct a systematic review and trial sequential meta-analysis of randomized controlled trials addressing the* overall* effects of exercise (aerobic training, strength training, or both) on BMI in kg·m^−2^ among overweight and obese children and adolescents.

## 2. Methods

### 2.1. Registration and General Procedure

This systematic review with trial sequential meta-analysis is registered in PROSPERO (#CRD42015017586), an international prospective registry of systematic reviews. The conduct and reporting of this study was accomplished according to the general guidelines recommended by the Preferred Reporting Items for Systematic Reviews and Meta-Analyses (PRISMA) statement [26].

### 2.2. Study Eligibility

The* a priori* inclusion criteria for this study were as follows: (1) randomized controlled trials (assignment at participant level only), (2) control group (nonintervention, usual care, wait-list control, and attention control), (3) exercise (aerobic training, strength training, or both) ≥ 4 weeks as an independent intervention, (4) overweight and obese children and adolescents, as defined by the authors, (5) boys and/or girls 2 to 18 years of age, (6) studies published in full in any language between January 1, 1990, and November, 11, 2014, and (7) data available for calculating changes in BMI in kg·m^−2^. Studies were excluded based on an inappropriate population, intervention, comparison, outcome, study type, or lack of requisite data for BMI in kg·m^−2^.

### 2.3. Data Sources

The following databases were searched from January 1, 1990, to December 31, 2012: (1) Academic Search Complete, (2) CINAHL, (3) Cochrane Central Register of Controlled Trials (CENTRAL), (4) Education Research Complete, (5) ERIC, (6) LILACS, (7) Medline, (8) Proquest, (9) Scopus, (10) Sport Discus, and (11) Web of Science. In addition, an updated PubMed search was conducted for potentially eligible studies published between August 1, 2012, and November 11, 2014. A brief description of each database is shown in Supplementary File 1 (see Supplementary Material available online at http://dx.doi.org/10.1155/2015/704539) while the updated search strategy for PubMed can be found in Supplementary File 2. Database searches were supplemented by cross-referencing for potentially eligible studies, including reviews, as well as expert review by the third author. All studies were stored in Reference Manager, version 12.0 [27]. Overall precision of the searches was computed by dividing the number of studies included by the total number of studies screened while the number needed to read (NNR) was calculated as the inverse of the precision [28].

### 2.4. Study Selection

Independent, dual-selection of eligible studies was conducted by the first two authors who then met and reviewed their choice for inclusion. Disagreements were resolved by consensus and, if necessary, consultation with the third author.

### 2.5. Data Abstraction

Codebooks were developed in an electronic spreadsheet program [29] that included items that fell within the following four major categories: (1) study characteristics, (2) physical characteristics of participants, (3) training program characteristics, and (4) outcomes and outcome characteristics. Independent, dual-selection of eligible studies was conducted by the first two authors who then met and reviewed their choice for inclusion. Disagreements were resolved by consensus and, if necessary, consultation with the third author. Using Cohen's kappa statistics (*κ*) [30], the overall agreement rate prior to correcting discrepancies was 0.94.

### 2.6. Risk of Bias Assessment

The Cochrane Risk of Bias Assessment Instrument was used to assess potential risk of bias [31]. Items were rated as being at a high, low, or unclear risk for bias [31]. In addition to the six basic items, an additional item about whether the participants were exercising regularly prior to study participation, as defined by the original study authors, was included [31]. Assessment for risk of bias was limited to the primary outcome, BMI in kg·m^−2^. Dual and independent assessment for risk of bias was conducted by the first two authors who subsequently met and reviewed every item for agreement. Disagreements were resolved by consensus and, if necessary, consultation with the third author. Using Cohen's kappa statistics (*κ*) [30], the overall agreement rate prior to correcting discrepancies was 0.70.

### 2.7. Data Synthesis

#### 2.7.1. Calculation of Effect Sizes for BMI in kg·m^−2^

The primary outcome for this study was changes in BMI in kg·m^−2^. Secondary outcomes included body weight, percent body fat, fat mass, fat-free mass, changes in maximum oxygen consumption in mL·kg^−1^ min^−1^ (VO_2max_), and upper and lower body strength and kilocalorie intake. Effect sizes (ES) using the original metrics were calculated by subtracting the change score difference in the exercise group from the change score difference in the control group. Variances were calculated from the pooled standard deviations of change scores in the exercise and control groups. If change score standard deviations were not reported, they were calculated from pre- and poststandard deviations according to procedures developed by Follmann et al. [32]. Each ES was then weighted by the inverse of its variance.

#### 2.7.2. Pooled Estimates for Changes in BMI in kg·m^−2^

Changes in BMI in kg·m^−2^ and all secondary outcomes were pooled using random-effects, method-of-moments models that incorporate between-study heterogeneity into the final estimate [33]. Ninety-five percent confidence intervals (CI) were calculated while *z*-based two-tailed alpha values ≤ 0.05 were considered statistically significant. Heterogeneity was examined using the *Q* statistics [34], with an alpha value ≤ 0.10 representative of statistically significant heterogeneity. Inconsistency was examined using *I* ^2^ [35] and diversity using *D* ^2^ [36]. For both *I* ^2^ and *D* ^2^ values < 25%, 25% to <50%, 50% to <75%, and 75% or greater were considered to represent very low, low, moderate, and large amounts of inconsistency and diversity [37]. Statistically significant outliers were considered to be those with standardized residual alpha values ≤ 0.05. Multiple exercise groups in the same study were analyzed independently as well as collapsing multiple groups so that only one ES represented each study while the sample size for the control group was divided by the number of exercise groups [38]. In addition to 95% CI, 95% prediction intervals (PI) were also calculated [39, 40] for any result that was statistically significant. Based on recent recommendations [41], small-study effects (publication bias, etc.) were examined both qualitatively and quantitatively using funnel plots and Egger's regression intercept test [42]. A one-tailed probability value < 0.05 was considered to be indicative of statistically significant small-study effects. The influence of each result on the overall findings was examined by deleting each result from the model once.* Post hoc*, the fail-safe *N* test was used to estimate the number of studies that would be needed to reverse our finding of a statistically significant, that is, *p* < 0.05, improvement in BMI in kg·m^−2^ [43]. This test was used because four studies that met all of our inclusion criteria except for the provision of sufficient postintervention data were excluded from the meta-analysis.

To enhance practical application, the number needed to treat (NNT) was calculated for changes in BMI in kg·m^−2^ assuming a conservative control group risk of 10% and only if changes in BMI in kg·m^−2^ were statistically significant. If the NNT was calculated, gross estimates were determined for the number of obese children and adolescents in the US as well as worldwide that could potentially benefit from exercise. These estimates were based on 12.5 [1] and 110 million [44, 45] overweight and obese children in the US and worldwide, respectively. In addition to NNT, Cohen's *U*_3_ index, an index used to determine the percentile gain in an intervention group, was calculated for any statistically significant results with respect to BMI in kg·m^−2^ and secondary outcomes [46]. Finally, The Grades of Recommendation, Assessment, Development and Evaluation (GRADE) instrument was used to assess the overall quality of evidence and was limited to the primary outcome, BMI in kg·m^−2^ [47]. Overall quality was categorized as very low, low, moderate, or high [47].

Based on empirical evidence that consideration of information size and adjusted significance thresholds may avoid false statistical inferences due to imprecision and repeated significance testing in meta-analysis [25, 48–50], information size estimates and trial sequential analysis were performed [51] for BMI in kg·m^−2^. Trial sequential analysis is an approach that combines conventional meta-analysis methodology with meta-analytic sample size considerations as well as previously established methods for repeated significance testing on accruing data in randomized trials [51]. Inferences derived from using trial sequential analysis may be more reliable than using conventional meta-analysis procedures [51]. More specifically, previous research suggests that information size considerations as well as adjusted significance thresholds may eliminate early false positive findings due to a lack of precision and repeated significance testing in meta-analyses [25, 48–51].

The* a priori* plan was to estimate the required information size based on previous research suggesting that a 0.1 kg/m^2^ change in BMI in kg·m^−2^ can be clinically important [52]. However, because of the inability to obtain variance statistics, a* post hoc* decision was made to estimate the required information size using the pooled mean difference and variance, adjusted for between-study heterogeneity, from the current study. A two-tailed type 1 error rate of 5% and power of 80% were employed. To control for multiple tests, trial sequential monitoring boundaries for both type 1 (5%) and type 2 (20%) error rates were established using O'Brien-Fleming adjustments [53, 54].

#### 2.7.3. Metaregression Analysis

Simple, random-effects metaregression (method of moments) models were used to examine associations between changes in BMI in kg·m^−2^ and potential predictors [33]. An* a priori* decision was made to not conduct any type of multiple metaregression analyses because of missing data for different variables from different studies. Metaregression analysis was limited to those studies in which there were at least four results for continuous variables or four results per group for categorical variables. Continuous variables, determined* a priori*, included year of publication, percent dropout, age, baseline BMI in kg·m^−2^, and exercise intervention (length, frequency, duration, compliance, minutes per week, unadjusted and adjusted for compliance, and total minutes for the intervention, unadjusted and adjusted for compliance). Categorical variables examined included country, type of control group, funding,* a priori* sample size estimates, adverse events, risk of bias (sequence generation, allocation concealment, blinding of participants and personnel, blinding of outcome assessment, incomplete outcome data, selective reporting, and whether subjects were inactive prior to enrollment), gender, race/ethnicity, changes in exercise and/or physical activity outside the exercise intervention, pubertal stage, type of exercise (aerobic, strength, and both), exercise supervision, setting that exercise took place, type of participation, type of analysis, and exercise intensity (low, moderate, and high) [55].

#### 2.7.4. Reporting and Software Utilization

Changes in primary and secondary outcomes are reported in their natural direction of benefit, that is, negative values for changes in BMI in kg·m^−2^ and positive values for increases in fat-free mass. All statistical analyses were conducted using Comprehensive Meta-Analysis (version 3.3) [56], Microsoft Excel 2010 [57], Trial Sequential Analysis (version 0.9) [51], GradePro (version 3.6) [58], and two add-ins for Microsoft Excel, SSC-stat (version 2.18) [59] and EZ-Analyze (version 3.0) [60].

## 3. Results

### 3.1. Characteristics of Included Studies

**Figure 1:**
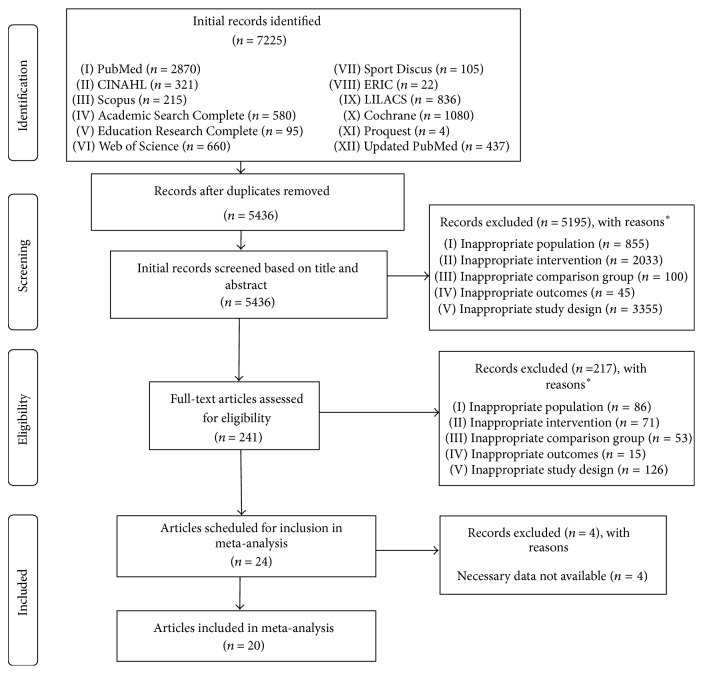
Flow diagram describing the search for relevant literature.  ^*∗*^Number of reasons exceeds the number of records excluded.

After removing duplicates, a total of 5,436 articles were screened. Of these, 20 studies representing 42 groups (22 exercise, 20 control) and final assessment of BMI in kg·m^−2^ in 971 participants (575 exercise, 396 control) met all eligibility criteria [61–80]. Overall precision of the searches was 0.004 while the NNR was 272. The major reasons for exclusion were inappropriate study design (51.6%), intervention (31.2%), population (14.0%), comparison (2.3%), and outcome(s) (0.9%). Another four studies comprising less than 1% of the reasons for exclusion were omitted because data necessary for conducting trial sequential meta-analysis were not available [81–84]. This included (1) lack of both post and change outcome values as well as standard deviations, or data for conversion to standard deviations (e.g., standard error of the mean), for BMI in kg·m^−2^ [81–83] and (2) lack of separate sample sizes for exercise and control groups [84]. A flow diagram of the search process is shown in Figure 1 while a list of excluded studies, including the specific reason(s) for exclusion, can be found in Supplementary File 3.

Table 1 describes the characteristics of each included study. Studies were conducted in 12 different countries and published between 2004 and 2014 [61–80], with all but one [62] published in English-language journals. Assessment of primary and secondary outcomes took place after six [68, 69], eight [63, 67, 77–79], 10 [76], 12 [61, 64, 66, 71, 72, 75, 80], 13 [65], 16 [73], 22 [74], and 24 [62, 70] weeks of exercise.

Two studies used some type of matching procedure, one according to age, gender, and BMI in kg·m^−2^ [67] and another according to sex and degree of overweight [74]. For those studies in which it could be determined, six used the per-protocol approach to analyze their data [61, 65, 69, 70, 73, 75], two used intention-to-treat [76, 77], and three used both [62, 64, 74]. Only five of the studies included sample size estimates [62, 64, 74, 75, 79] while the majority (80%) received some type of funding for their work [61, 63–65, 67–71, 73–76, 78–80]. Nine studies received singular support from either university [69, 70, 75, 80], government [63, 65, 76], or private [78, 79] entities while seven other studies reported multiple sources of support from government and private [67, 71], government and university [64, 68], government, university, and private [73, 74], or private and university [61] entities.

For those studies in which data were available, the dropout rate for studies in which data were available ranged from 0% to 34% in the exercise groups ($\overset{-}{X}\pm SD$, 16.9% ± 14.0, median = 23) and 0% to 26% in the control groups ($\overset{-}{X}\pm SD$, 12.6% ± 12.0, median = 14). Reasons for dropping out included time, lack of interest, unhappiness with group assignment, moving, and medical condition. Four studies reported no serious adverse events during the intervention period [74, 77–79].

Participant characteristics are shown in Tables 1 and 2. Twelve of the 20 studies (60%) included both boys and girls [61, 62, 64, 65, 67, 70, 71, 74, 76–79], seven (35%) were limited to boys [63, 66, 68, 69, 73, 75, 80], and one (5%) was limited to girls [72]. Participants included Whites, Blacks, Asians, and Hispanics. With respect to maturational development, the studies represented boys and girls at the prepubertal, pubertal, and postpubertal stages of development; two studies reported boys and girls at the prepubertal stage [61, 64], one at the postpubertal stage [74], two at the prepubertal and pubertal stage [67, 76], and one each at either the pubertal and postpubertal [73] or prepubertal, pubertal, and postpubertal [63] stages.

Characteristics of the exercise programs from each study are also shown in Table 1. Thirteen of the 22 groups participated in aerobic exercise, two in strength training, and seven in both. For those studies and groups in which data were available, length of training ranged from six to 24 weeks ($\overset{-}{X}\pm SD$, 13.4 ± 5.7, median = 12) and frequency from one to five times per week ($\overset{-}{X}\pm SD$, 3.3 ± 1.1, median = 3). Intensity of training was classified as low for one group, moderate for four groups, moderate to high for two groups, and high for seven groups. When limited to aerobic exercise, duration of training for the 18 groups in which data were available ranged from 20 to 75 minutes per session ($\overset{-}{X}\pm SD$, 45.9 ± 15.4, median = 45). Total minutes of training, per week, ranged from 40 to 224 minutes ($\overset{-}{X}\pm SD$, 148.1 ± 55.0, median = 155) while total minutes of training for the entire intervention period ranged from 480 to 5,040 minutes ($\overset{-}{X}\pm SD$, 1979 ± 1302, median = 1540). When adjusted for compliance to the exercise protocol, total minutes per week for the three groups in which data could be calculated ranged from 39 to 75 minutes ($\overset{-}{X}\pm SD$, 67.3 ± 25.2, median = 75) while total minutes over the entire intervention period ranged from 470 to 896 minutes ($\overset{-}{X}\pm SD$, 749 ± 241, median = 880). Aerobic exercises included walking, jogging, cycling, swimming, jumping rope, stair climbing, aerobic dance, and games (soccer, handball, basketball, volleyball, etc.) as well as other various activities.

For strength training groups, the within-study number of sets for the five groups in which data were provided ranged from one to three while the number of repetitions per set for the six groups in which data were available ranged from three to 25. Two strength training groups reported within-study rest periods between sets that ranged between 60 and 180 seconds. For the five groups that reported data, the number of strength training exercises ranged from seven to 13 ($\overset{-}{X}\pm SD$, 9.2 ± 2.5, median = 9). Types of resistance training equipment used included free weights, machine weights, elastic bands, medicine balls, and the participants' own body weight. Across all exercise groups, 18 participated in supervised exercise, one in unsupervised exercise, and three in both. Compliance to the exercise interventions for the four groups in which data could be calculated ranged from 55% to 98% ($\overset{-}{X}\pm SD$, 83.4 ± 20.2, median = 90).

### 3.2. Risk of Bias Assessment

**Figure 2:**
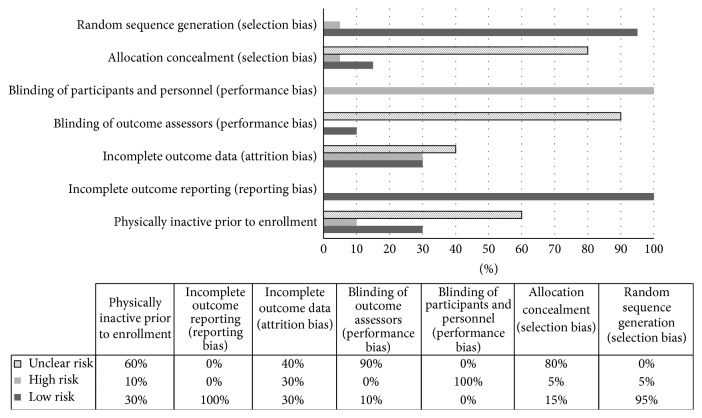
Cochrane risk of bias results.

The results for pooled risk of bias assessment are shown in Figure 2 while study level results are shown in Supplementary File 4. As can be seen, 95% of the included studies adequately described the process for random sequence generation while none of the studies suffered from incomplete outcome reporting. In contrast, more than half of the studies were at a high or unclear risk of bias with respect to allocation concealment (85%), blinding of participants and personnel (100%), blinding of outcome assessors (90%), incomplete outcome data, that is, attrition bias (70%), and boys and girls being physically inactive prior to enrollment (70%).

### 3.3. Data Synthesis

#### 3.3.1. Primary Outcome

**Figure 3:**
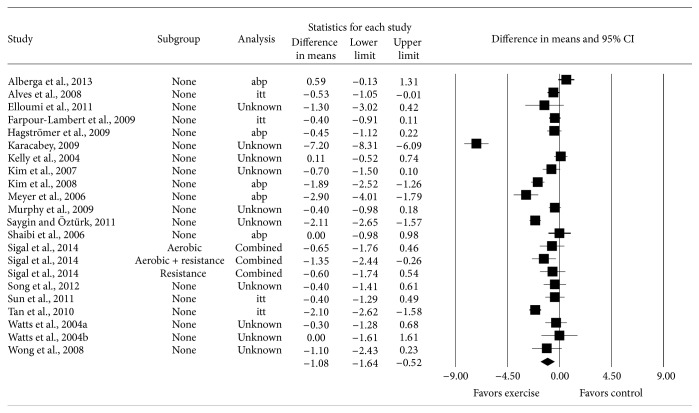
Forest plot for point estimate changes in BMI in kg·m^−2^. The black squares represent the mean difference while the left and right extremes of the squares represent the corresponding 95% confidence intervals. The middle of the black diamond represents the overall mean difference while the left and right extremes of the diamond represent the corresponding 95% confidence intervals.

**Figure 4:**
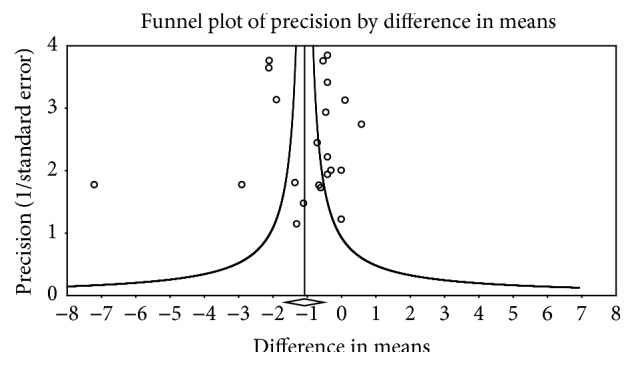
Funnel plot for changes in BMI in kg·m^−2^.

**Figure 5:**
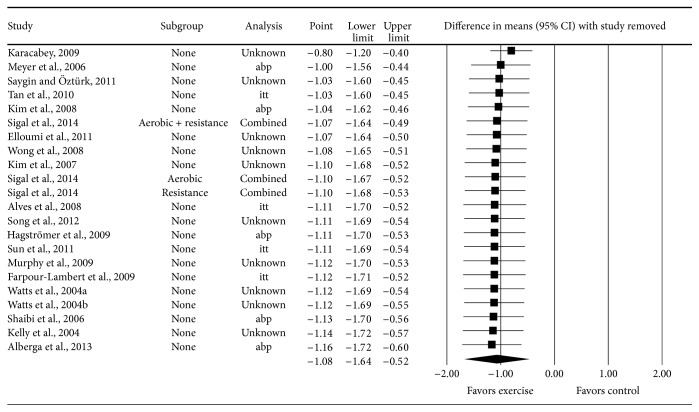
Influence analysis results for point estimate changes in BMI in kg·m^−2^ with each result deleted from the model once. The black squares represent the mean difference while the left and right extremes of the squares represent the corresponding 95% confidence intervals. The middle of the black diamond represents the overall mean difference while the left and right extremes of the diamond represent the corresponding 95% confidence intervals. Results are ordered from smallest to largest reductions.

Pooled results for changes in BMI in kg·m^−2^ are shown in Table 3 and Figure 3. Across all categories, a statistically significant reduction equivalent to 3.6% was found for BMI in kg·m^−2^ along with statistically significant heterogeneity, a large amount of inconsistency and diversity, and overlapping prediction intervals. Changes in BMI in kg·m^−2^ ranged from 0.59 to −7.30 kg/m^2^. With one outlier deleted from the model [66], reductions were not as large (26% difference) but remained statistically significant along with statistically significant heterogeneity as well as a large amount of inconsistency and diversity ($\overset{-}{X}$: −0.80; 95% CI: −0.40 to −1.20; *z* = −3.94; *p* < 0.001; *Q* = 108.1; *p* < 0.001; *I* ^2^ = 81.5%; 95% CI = 72.7 to 87.5; *D* ^2^ = 83.2%). Reductions in BMI in kg·m^−2^ also remained statistically significant along with statistically significant heterogeneity as well as a large amount of inconsistency and diversity when results were collapsed so that only one result represented each study ($\overset{-}{X}$: −1.10; 95% CI: −0.52 to −1.68; *z* = −3.71; *p* < 0.001; *Q* = 230.3; *p* < 0.001; *I* ^2^ = 91.8%; 95% CI = 88.7 to 94.0; *D* ^2^ = 92.2%). No small-study effects were observed as indicated by a lack of funnel plot asymmetry (Figure 4) and Egger's regression-intercept test (*β*_0_: −0.92, *p* = 0.34). With each result deleted from the model once, reductions in BMI in kg·m^−2^ remained statistically significant across all deletions, with changes ranging from −0.80 to −1.16, a difference of 31% (Figure 5). The NNT was 5 (95% CI = 3 to 12) while the percentile improvement was 26.9 (95% CI = 15.3 to 36.0). It was estimated that approximately 2.5 million overweight and obese children in the US (95% CI, 1.0 to 4.2) and 22.0 million overweight and obese children worldwide (95% CI, 9.2 to 36.7) could reduce their BMI in kg·m^−2^ by participating in a regular exercise program.

For the four studies excluded because of insufficient data for BMI in kg·m^−2^ [81–84], one reported a statistically significant exercise minus control group reduction (*p* = 0.02) in BMI in kg·m^−2^ [84]. Another study that did not report results for BMI in kg·m^−2^ did report a statistically significant exercise minus control group reduction in BMI *z*-score (*p* = 0.02) for the high-dose group as well as a trend for improvement (*p* = 0.06) in the low-dose group [81]. The remaining two studies did not report any BMI-related results, although both reported statistically significant reductions of *p* < 0.01 [82] and *p* < 0.001 [83] for percent body fat. In addition, fail-safe *N* results indicated that a total of 774 studies with null findings would be needed to reverse our findings of a statistical significant reduction in BMI in kg·m^−2^.

**Figure 6:**
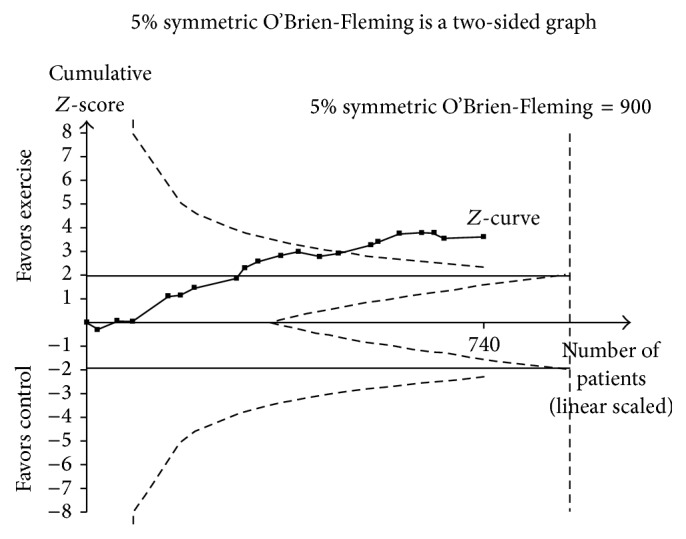
Trial sequential analysis results. Trial sequential meta-analysis of exercise versus control for changes in BMI in kg·m^−2^. The dashed inward sloping lines to the left represent trial sequential monitoring boundaries while the outward dashed sloping lines to the right represent futility boundaries. The solid black line represents the *Z*-curve and the black squares represent the cumulative results with each accumulating study from earliest (2004) to most recent (2014) year. The cumulative *Z*-curve, that is, black solid line with filled squares, crossed the monitoring boundaries in 2010, confirming that exercise reduces BMI in kg·m^−2^ in overweight and obese children and adolescents and is unlikely to be reversed with additional studies in future years.

The results for trial sequential meta-analysis are shown in Figure 6. As can be seen, these findings confirm that the maximum information size has been reached and the stability of findings has been achieved with respect to exercise-induced reductions in BMI in kg·m^−2^ among overweight and obese children and adolescents. More specifically, changes in BMI in kg·m^−2^ crossed the monitoring boundary for a type 1 error in 2010 and have remained stable thereafter. This confirms the statistical significance of exercise-induced reductions in BMI in kg·m^−2^ since 2010 among overweight and obese children and adolescents and suggests that the accumulation of additional studies in future years will not change these findings to one of nonsignificance. Simple metaregression results are shown in Supplementary File 5. No statistically significant associations were observed for those variables in which metaregression analysis was possible. Findings were similar when the one outlier for changes in BMI in kg·m^−2^ was deleted from each of the analyses (results not shown) [66].

The results for GRADE with respect to changes in BMI in kg·m^−2^ are shown in Supplementary File 6. Despite potential biases as well as heterogeneity, inconsistency, diversity, and overlapping prediction intervals, the overall quality of evidence was upgraded from low to moderate based on the magnitude of effect observed, trial sequential analysis results, and lack of adverse events.

#### 3.3.2. Secondary Outcomes

Secondary outcomes are shown in Table 3. Statistically significant improvements were found for body weight, fat mass, percent body fat, VO_2max_ in mL·kg^−1^·min^−1^, and upper and lower body strength. No statistically significant differences were observed for fat-free mass or energy intake. Changes were equivalent to relative improvements of 2.2% (body weight), 3.2% (fat mass), 2.9% (percent body fat), 10.3% (VO_2max_ in mL·kg^−1^·min^−1^), 8.9% (upper body strength), and 52.4% (lower body strength). With the exception of changes in upper and lower body strength, statistically significant heterogeneity as well as moderate to large inconsistency and diversity was observed for body weight, fat mass, percent body fat, and VO_2max_ in mL·kg^−1^·min^−1^. Prediction intervals were overlapping for all outcomes except for upper and lower body strength.

Statistically significant outliers (*p* < 0.05) were identified for changes in body weight [61, 66], fat mass [69], percent body fat [69], and VO_2max_ in mL·kg^−1^·min^−1^ [72]. With two outliers deleted from the model for body weight [61, 66], reductions remained statistically significant along with statistically significant heterogeneity, low inconsistency, and moderate diversity ($\overset{-}{X}$: −1.62; 95% CI: −0.93 to −2.31; *z* = −4.61; *p* < 0.001; *Q* = 33.3; *p* < 0.001; *I* ^2^ = 49.0%; 95% CI = 12.0 to 70.4; *D* ^2^ = 68.1%). For fat mass, decreases remained statistically significant along with no statistically significant heterogeneity as well as very low inconsistency and diversity when one outlier was deleted from the model [69] ($\overset{-}{X}$: −0.69; 95% CI: −0.17 to −1.22; *z* = −2.58; *p* = 0.01; *Q* = 13.9; *p* = 0.31; *I* ^2^ = 13.9%; 95% CI = 0 to 62.5; *D* ^2^ = 21.1%). With the same study deleted [69], reductions in percent body fat remained statistically significant along with statistically significant heterogeneity, moderate inconsistency, and large diversity ($\overset{-}{X}$: −1.01; 95% CI: −0.47 to −1.54; *z* = −3.68; *p* < 0.001; *Q* = 49.6; *p* < 0.001; *I* ^2^ = 69.7%; 95% CI = 49.5 to 81.9; *D* ^2^ = 75.7%). Increases in VO_2max_ in mL·kg^−1^·min^−1^ also remained statistically significant along with statistically significant heterogeneity, moderate inconsistency, and large diversity when one outlier [72] was deleted from the model ($\overset{-}{X}$: 2.35; 95% CI: 1.38 to 3.31; *z* = 4.76; *p* < 0.001; *Q* = 22.3; *p* = 0.004; *I* ^2^ = 64.1%; 95% CI = 26.4 to 82.4; *D* ^2^ = 81.3%). No outliers were identified for changes in lower and upper body muscular strength.

For those secondary outcomes in which statistically significant improvements were found, statistically significant small-study effects were observed for changes in percent body fat (*β*_0_: 1.92, *p* = 0.03). No statistically significant small-study effects were observed for body weight (*β*_0_: −0.54, *p* = 0.22), fat mass (*β*_0_: −0.76, *p* = 0.26), VO_2max_ in mL·kg^−1^·min^−1^ (*β*_0_: −0.01, *p* = 0.50), or upper (*β*_0_, 1.99, *p* = 0.15) and lower (*β*_0_, 4.77, *p* = 0.27) body strength.

With each result deleted from the model once, changes remained statistically significant for all secondary outcomes in which the original findings were statistically significant. Changes ranged from −1.38 to −1.86 kg for body weight (25.3% difference), −0.69 to −1.25 kg for fat mass (44.8% difference), −1.00 to −1.23 for percent body fat (23.0% difference), 2.34 to 3.42 mL·kg·min^−1^ for VO_2max_ (31.6% difference), 6.9 to 9.4 kg for upper body strength (26.6% difference), and 41.5 to 46.0 kg for lower body strength (9.8% difference).

## 4. Discussion

### 4.1. Findings

The overall findings of the current meta-analysis suggest that exercise is associated with reductions in BMI in kg·m^−2^ among overweight and obese children and adolescents. Support for this interpretation is derived from (1) the overall magnitude of effect, (2) nonoverlapping 95% CI, (3) continued significance when each study was deleted from the model once, including the one outlier [66], (4) apparent absence of small-study effects, (5) trial sequential analysis results demonstrating that the maximum information size had been reached and been stable since 2010, (6) the low NNT, and (7) the number of overweight and obese children and adolescents in the US and worldwide who might potentially improve their BMI in kg·m^−2^ from the uptake of regular exercise. In addition, the magnitude of change in BMI in kg·m^−2^ observed in this study (−1.08 kg/m^2^ or 3.6%) may be clinically relevant as previous research has found significant improvements in selected health outcomes with a decrease in BMI in kg·m^−2^ of approximately 4.8% [85]. While the results of the current meta-analysis were 1.2% smaller, they may still be clinically important. Regardless, the observed reductions in BMI in kg·m^−2^ are most likely important at the population level. For example, a recent meta-analysis that reported a reduction of only 0.17 kg·m^−2^ in BMI [20] as a result of school-based interventions suggested that their findings may result in important health benefits at the population level. This suggestion was based on the work of Rose [86] who contended that a small shift in population distribution can be an effective primary preventative strategy because more events occur among the large number of individuals at moderate risk than the small number at high risk. Importantly, the results of the current meta-analysis were more than six times larger than those of Lavelle et al. [20]. However, it is important to realize that whether an intervention should be recommended at the population level depends not only on the size of the effect but also on the costs associated with achieving such an effect as well as society's willingness to pay for this. While the willingness of a society to pay for this most likely varies between countries and there is limited evidence regarding the cost-effectiveness of exercise interventions for the treatment of overweight and obesity in children and adolescents, one cost-effectiveness study found that the number of disability-adjusted life years was greater for a multifaceted school-based intervention that included physical education (8000) versus one without physical education (500) [87]. Clearly, further research in this area is needed.

Finally, considering that the results for GRADE were increased from low to moderate provides justification for recommending exercise for improving BMI in kg·m^−2^ in overweight and obese children and adolescents. This is especially relevant given that a low rating is based on the belief that additional evidence in the future would most likely change the direction of effect, something that the investigative team does not believe will happen, especially given the trial sequential analysis results.

In contrast to the investigative team's findings that support the effects of exercise for reducing BMI in kg·m^−2^ as well as the fact that a random-effects model that incorporates heterogeneity into the analysis was used, no potential sources of heterogeneity were identified as a result of metaregression analyses. Thus, the current results could be compromised. This may be especially important given the large amount of inconsistency and diversity observed for BMI in kg·m^−2^ in the current meta-analysis. However, while such analyses are important, covariate analyses in meta-analysis are considered observational given that studies are not randomly assigned to covariates [88]. As a result, such analyses do not support causal inferences [88]. Thus, while such analyses may generate important findings about potential sources of heterogeneity, they would still need to be tested in adequately powered randomized controlled trials [88]. A second finding that may weaken the BMI in kg·m^−2^ results is the overlapping PI observed for changes in BMI in kg·m^−2^. However, it is important to understand that PI are different compared to CI as the former are based on random-mean effects [35].

While no variables that accounted for heterogeneity with respect to changes in BMI in kg·m^−2^ were found, it may be that factors that were unable to be assessed could account for some or all of the observed heterogeneity between the included studies. These include such things as (1) differences or changes in diet during the exercise intervention [89], (2) physical activity compensation [65, 90], and (3) genetic factors [91].

The results of the current meta-analysis are in agreement with one previous systematic review with meta-analysis that focused on exercise [20] but disagreement with four others that reported a nonsignificant decrease in BMI in kg·m^−2^ among children and adolescents [17, 21–23]. Possible reasons for these discrepancies include (1) the small number of exercise-only studies that were included and pooled in these meta-analyses [17, 21, 22], (2) the inclusion of nonrandomized trials [20, 22], and (3) the inclusion of children and adolescents who were not overweight or obese [20, 22, 23].

The reductions in BMI in kg·m^−2^ observed in the current meta-analysis also compare favorably to orlistat, the only weight-loss medication currently approved by the US Food and Drug Administration for the treatment of obese adolescents. In a recent meta-analysis, changes in BMI in kg·m^−2^ that included two studies representing 579 participants resulted in a statistically significant decrease of −0.76 kg·m^−2^ (95% CI, −1.07, −0.44) as a result of the use of orlistat [92]. These findings are approximately 30% less than those found for BMI in kg·m^−2^ and exercise in the current meta-analysis.

The reductions in BMI in kg·m^−2^ found in the current meta-analysis are also similar to the results reported in a recent systematic review of diet-only interventions in which decreases ranged from 0.8 to 2.7 kg/m^2^ [89]. This suggests that either exercise or diet can reduce BMI in kg·m^−2^ in a similar fashion. In contrast, the results of this previous systematic review when diet and exercise were combined were equivocal, with changes in BMI in kg·m^−2^ ranging from −4.4 to 0.27 kg/m^2^ for aerobic exercise (4 studies), −0.2 to 1.1 kg/m^2^ for resistance training (3 studies), and −0.5 to −2.02 kg/m^2^ for combined aerobic and resistance exercise (3 studies) [89]. However, whether these changes differ significantly according to type of exercise, type of diet, or some other factor(s) is not known.

In addition to changes in BMI in kg·m^−2^, statistically significant and clinically important improvements in body weight, fat mass, percent body fat, relative VO_2max_, and upper and lower body strength were observed. The changes in fat mass as well as percent body fat are particularly noteworthy since both are more relevant than BMI in kg·m^−2^ with respect to improvements in body composition. However, because they are not as practical to assess, BMI in kg·m^−2^ continues to be the preferred method of assessing and classifying overweight and obesity. In addition, the significant changes observed for the six secondary outcomes support the multiple benefits that can be derived from regular participation in exercise. The multiple benefits observed are in contrast to treatments such as pharmacological interventions, approaches that are usually intended to treat one outcome. In addition, orlistat, the only pharmacological intervention currently approved in the United States for the treatment of obesity in children and adolescents [93], has been shown to be less cost-effective than several nonpharmacologic interventions, including exercise [87, 94], and has also been accompanied by side-effects such as gastrointestinal distress [95]. With respect to exercise in the current meta-analysis, four studies that did include information on side-effects reported no serious adverse events [74, 77–79], defined as any intervention that results in death, a life threatening condition, hospitalization (initial or prolonged), disability, or permanent damage [96]. For these same four studies, adverse events, defined as any undesirable experience associated with an intervention, included primarily acute musculoskeletal injury or discomfort in 7.9% of exercise participants in one study [74] and none in the other three [77–79]. However, it is important to realize that 16 [61–73, 75, 76, 80] of the 20 studies in the current meta-analysis did not report adequate information with respect to adverse events.

### 4.2. Implications for Research

The results of this meta-analysis have several implications for both the reporting and conduct of future research. First, it is suggested that future studies report complete information regarding (1) allocation concealment, (2) blinding of outcome assessment, (3) dropouts according to each group, including reasons for dropping out, (4) adverse events, (5) the physical activity levels of participants prior to and during the intervention, (6) intensity of the exercise intervention, and (7) compliance to the exercise intervention. It is also suggested that investigators analyze and report results using both the per-protocol and intention-to-treat results. This will allow one to understand both the efficacy and effectiveness of exercise for improving BMI in kg·m^−2^ as well as other outcomes in overweight and obese children and adolescents. In addition, since both energy intake and energy expenditure are critical in determining weight loss, future studies should collect and report data on energy intake and total daily energy expenditure. Finally, future studies should report complete information on all outcomes assessed, partitioned by group. At a minimum, these data should include pre- and postsample sizes, means, and standard deviations as well as change outcome results along with their standard deviations.

It appears that a need exists for a four-arm randomized controlled trial in overweight and obese children and adolescents that includes an aerobic, strength, and combined aerobic and strength training group as well as a control group. Furthermore, to aid practitioners, a need exists for dose-response studies to determine the optimal exercise program(s) for overweight and obese children and adolescents. This may be especially important since it is currently recommended that children and adolescents participate in 60 or more minutes of moderate to vigorous physical activity per day (420 minutes per week) [97] but the current meta-analysis found statistically significant reductions in BMI in kg·m^−2^ as well as several other outcome variables (body weight, fat mass, percent body fat, VO_2max_, and upper and lower body strength) when the average total minutes per week was less than currently recommended. Finally, since cost is an important factor when deciding what intervention to recommend over another, a need exists for cost-effectiveness studies in overweight and obese children and adolescents.

### 4.3. Implications for Practice

The results of the current meta-analysis suggest that exercise results in important improvements in BMI in kg·m^−2^ as well as body weight, fat mass, percent body fat, VO_2max_ in mL·kg^−1^·min^−1^, and muscular strength in both upper and lower body. Lending further support for this contention is the low NNT, percentile improvement, and the estimated number of overweight and obese children in the United States and worldwide who could potentially benefit. Furthermore, no serious adverse events were reported for the four groups in which sufficient information was available. Unfortunately, the dose-response effects of exercise on BMI in kg·m^−2^ and other outcomes in overweight and obese children and adolescents remain elusive. Thus, in order to not withhold a potentially beneficial and safe intervention and until more definitive evidence is available, it would appear prudent to recommend that practitioners follow the guidelines specific to children and adolescents as denoted in the 2008 Physical Activity Guidelines for Americans [98]. This includes at least 60 minutes per day of moderate to vigorous physical activity, primarily aerobic activity (running, hopping, skipping, jumping rope, swimming, dancing, and bicycling) as well as muscle strengthening activities and bone strengthening activities (running, jumping rope, basketball, tennis, hopscotch, etc.) [98].

While the focus of the current meta-analysis was on the effects of exercise on BMI in kg·m^−2^ in overweight and obese children and adolescents, it would appear plausible to suggest that the addition of reduced caloric intake combined with exercise may result in even greater reductions in BMI in kg·m^−2^_._ However, a recent meta-analysis of randomized controlled trials by Ho et al. found no statistically significant differences in BMI in kg·m^−2^ between exercise and diet versus diet-only groups [89]. Importantly, the authors concluded that further randomized controlled trials with a rigorous design are needed to confirm their findings. Until that time, it would appear plausible to suggest that practitioners follow the recent recommendations that, in addition to exercise, include (1) the avoidance of sugar-sweetened beverages, (2) less food with high caloric density, and (3) increased intake of fruits and vegetables [99].

### 4.4. Strengths and Potential Limitations of Current Study

In the investigative team's opinion, there are at least four* strengths* of the current meta-analysis. First, to the best of the authors' knowledge, this is the first trial sequential meta-analysis that has examined the effects of exercise on BMI in kg·m^−2^ in overweight and obese children and adolescents, something that was not done in previous work by the investigative team [19]. This is important because it suggests that changes in BMI in kg·m^−2^ are stable and not subject to a type 1 or type 2 error. Second, the inclusion of data regarding NNT, percentile improvement, relative improvement, and absolute number of overweight and obese children who might benefit from participation in a regular exercise program provides practical information to decision-makers with respect to what treatment, or combination of treatments, to recommend over others for overweight and obese children and adolescents. Third, the calculation of PI provides future researchers with an estimate of what effect they might expect to find for BMI in kg·m^−2^ and several secondary outcomes (body weight, fat mass, percent body fat, VO_2max_ in mL·kg^−1^·min^−1^, and upper and lower muscular strength) if they were to conduct a randomized controlled exercise intervention trial in overweight and obese children and adolescents. Fourth, this supports previous work by the investigative team in which an exercise minus control group improvement of approximately 3% was found for BMI *z*-score [19]. From the investigative team's perspective, the similar improvements observed for both BMI *z*-score and BMI in kg·m^−2^ are important given the continued controversy regarding which metric is the most valid and reliable for assessing changes in adiposity among children and adolescents. For example, while one study reported that BMI *z*-score is the best BMI measure for assessing adiposity in children and/or adolescents [100], another [101], as well as more recent research [102], suggests that both absolute and relative changes in BMI in kg·m^−2^ are better proxies for changes in adiposity. Thus, regardless of which BMI measure is superior for measuring changes in adiposity, something that is unlikely to be resolved in the near future, the investigative team's previous [19] as well as current findings support similar exercise-induced improvements for both.

As opposed to the strengths of the current meta-analysis, there are at least five* potential limitations*. First, given the statistically significant heterogeneity as well as high inconsistency and diversity of the current findings as well as overlapping PI and GRADE findings, one might conclude that insufficient evidence currently exists to conclude that exercise is associated with statistically significant improvements in BMI in kg·m^−2^ and selected secondary outcomes (body weight, fat mass, percent body fat, VO_2max_ in mL·kg^−1^·min^−1^, and upper and lower muscular strength). Second, the statistically significant findings for increases in upper and lower body strength may need to be viewed with caution given that these findings were limited to three results. Consequently, the generalizability of these findings may be limited. Third, the generalizability of the current findings beyond the populations and intervention protocols included may be limited. Fourth, the results of the current meta-analysis, like any meta-analysis, may suffer from ecological fallacy, phenomena in which incorrect inferences about individual findings are made based upon aggregate statistics [103]. Fifth, since the search for studies focused on BMI in kg·m^−2^ as the primary outcome, the results for all eight secondary outcomes may represent a biased sample.

## 5. Conclusions

The results of the current systematic review of previous meta-analyses suggest that exercise is associated with reductions in BMI in kg·m^−2^ among overweight and obese children and adolescents. A need exists for randomized controlled trials to identify sources of heterogeneity, including dose-response studies.

## Supplementary Material

Supplementary File1: This file provides a brief description of each database that was searched for this meta-analysis.
Supplementary File 2: This file provides the search strategy used for the updated PubMed search.
Supplementary File 3: This file includes a list of excluded references, including the reasons for exclusion.
Supplementary File 4: This file povides a study-level assessment of risk of bias for each item.
Supplementary File 5: This file provides a list of meta-regression analyses for potential predictors for changes in BMI.
Supplementary file 6: This file provides a detailed description of results for the Grades of Recommendation, Assessment, Development and Evaluation (GRADE) Instrument.

## Figures and Tables

**Table 1:** Study characteristics.

Study	Year	Sex M/F	*N* Ex/Con	Age Ex/Con	Exercise intervention								
					Length	Frequency	Intensity (%)				Duration (min)	Mode	Compliance
					(Weeks)	(Days)	MHR	HRR	VO_2max_	1RM	A S	A/S	(%)
Alberga et al. [61]	2013	M/F	12/7	10.0 ± 1.0/10.0 ± 2.0	12	2	65–70	—	—	65–86	20 45	A + S	98%
Alves et al. [62]	2008	M/F	39/39	8.0 ± 1.8/7.9 ± 1.5	24	3	—	—	—	—	50 —	A	—
Elloumi et al. [63]	2011	M	7/8	13.1 ± 1/13.2 ± 0.2	8	4	—	—	—	—	— —	A	—
Farpour-Lambert et al. [64]	2009	M/F	22/22	9.1 ± 1.4/8.8 ± 1.6	12	3	—	—	55–65	—	30 20	A + S	83%
Hagströmer et al. [65]	2009	M/F	16/15	13.7 ± 2.0/13.6 ± 2.2	13	1	—	—	—	50–70	60	A + S	—
Karacabey [66]	2009	M	20/20	11.8 ± 0.5/11.2 ± 0.80	12	3	—	60–65	—	—	20–45 —	A	—
Kelly et al. [67]	2004	M/F	10/10	11.0 ± 0.6/11.0 ± 0.7	8	4	—	—	50–80	—	30–50 —	A	≥80%
Kim et al. [68]	2007	M	14/12	17.0 ± 0.4/17.0 ± 0.4	6	5	—	—	—	—	30 —	A	—
Kim et al. [69]	2008	M	8/9	11.0/11.0	12	2	—	55–75	—	70	30–35 50	A + S	—
Meyer et al. [70]	2006	M/F	33/34	13.7 ± 2.1/14.1 ± 2.4	24	3	—	—	—	—	60–90 —	A	—
Murphy et al. [71]	2009	M/F	23/12	7–12	12	5	—	—	—	—	10–30 —	A	75% ≥ 5x weeks; 15% ≥ 3x weeks
Saygin and Öztürk [72]	2011	F	20/19	10–12	12	3	—	43	—	—	75 —	A	—
Shaibi et al. [73]	2006	M	11/11	15.1 ± 0.5/15.6 ± 0.5	16	2	—	—	—	62–97	— —	S	96%
Sigal et al. [74]^a^	2014	M/F	75 (A)	15.5 ± 1.4 (A)	22	4	65–85	—	—	—	20–50 —	A	62% (A)
			78 (S)	15.9 ± 1.5 (S)			—	—	—	—	— —	S	56% (S)
			75 (A + S)	15.5 ± 1.3 (A ± S)			65–85	—	—	—	20–50	A + S	64% (A + S)
			76 (Con)	15.6 ± 1.3 (Con)									
													
Song et al. [75]	2012	M	12/10	12.7 ± 0.7/12.6 ± 0.6	12	3	60–70	—	—	—	30 —	A	≥80%
Sun et al. [76]	2011	M/F	25/17	13.6 ± 0.7	10	4	—	—	40–60	—	40 —	A	55%
Tan et al. [77]^b^	2010	M/F	30/30	9.4 ± 0.5/9.5 ± 0.5	8	5	—	—	—	—	40 —	A	—
Watts et al. [78]^c^	2004	M/F	19/19	14.3 ± 1.5	8	3	65–85	—	—	55–70	60 —	A + S	≥90%
Watts et al. [79]	2004	M/F	14/14	8.9 ± 1.6	8	3	66–85	—	—	—	60 —	A	≥90%
Wong et al. [80]^c^	2008	M	12/12	13.8 ± 1.1/14.3 ± 1.5	12	2	50–85	—	—	—	55	A + S	—

*Notes.* M/F: males/females; Ex/Con: exercise/control; days: days per week, minutes: minutes per session; A/S: aerobic/strength; compliance: percentage of exercise sessions attended; data reported as mean ± standard deviation; VO_2max_: maximum oxygen consumption in mL·kg^−1^·min^−1^; 1RM: one-repetition maximum; MHRR: maximal heart rate reserve; MHR: maximum heart rate; ^a^compliance based on medians versus means; ^b^participants trained at intensity equivalent to 78% of lactate threshold; ^c^study consisted of circuit training.

**Table 2:** Initial physical characteristics of participants.

Variable	Exercise					Control				
	Groups/participants	Missing data (%)^*∗*^	$\overset{-}{X}$ ± SD	Mdn	Range	Groups/participants	Missing data (%)^*∗*^	$\overset{-}{X}$ ± SD	Mdn	Range
Age (years)	21/489	4.5	12.4 ± 2.5	13	8–17	18/301	10.0	12.0 ± 2.6	12	8–17
Height (cm)	19/443	13.6	155.7 ± 12.5	163	130–174	17/263	15.0	154.7 ± 13.4	154	127–175
Body weight (kg)	20/370	9.1	74.5 ± 18.7	75	35–104	18/312	10.0	73.3 ± 18.5	74	34–99
BMI (kg·m^−2^)	22/433	0.0	29.7 ± 4.0	30	21–36	20/376	0	29.6 ± 3.9	30	21–36
Fat mass (kg)	14/228	36.4	33.4 ± 9.5	32	22–50	12/181	40.0	31.8 ± 7.1	31	22–47
Body fat (%)	17/321	22.7	39.0 ± 6.2	36	31–50	15/265	25.0	38.5 ± 5.0	38	31–48
Fat-free mass (kg)	15/261	31.8	45.2 ± 7.9	47	28–54	13/208	35.0	44.9 ± 9.4	46	26–62
VO_2max_ (mL·kg^−1^·min^−1^)^a^	12/229	40.0	30.2 ± 6.1	31	20–39	10/175	50.0	30.0 ± 6.8	29	20–40
Muscular strength (kg)^b^										
Upper	3	66.7	86.8 ± 84.1	39	37–18	2	75.0	124.4 ± 36.6	124	37–212
Lower	3	66.7	84.3 ± 35.7	100	43–110	2	75.0	80.0 ± 34.2	80	56–104
Energy intake (kcals)	6/108	72.7	2465 ± 577	2319	1813–3278	4/44	81.8	2511 ± 771	2586	1614–3259

*Notes*. Groups/participants: number of groups and participants in which data were available; $\overset{-}{X}$ ± SD: mean ± standard deviation; Mdn: median; BMI: body mass index; VO_2max_: maximum oxygen consumption; kcals: kilocalories; ^a^data limited to those groups in which aerobic exercise was an intervention; ^b^data limited to those groups in which strength training was an intervention; ^*∗*^percentage of missing data calculated based on the premise that (1) all studies should have assessed and reported data for age, height, body weight, BMI, fat mass, percent body fat, fat-free mass, and energy intake, (2) all studies that included an aerobic exercise component should have assessed and reported data on VO_2max_ in mL·kg^−1^·min^−1^, and (3) all studies that included a strength training component should have assessed and reported data on upper and lower body strength.

**Table 3:** Changes in primary and secondary outcomes.

Variable	ES (#)	$\overset{-}{X}$ (95% CI)	*Z* (*p*)	*Q* (*p*)	*I* ^2^% (95% CI)	*D* ^2^%	95% PI
Primary							
BMI (kg·m^−2^)	22	**−1.08 (−0.52, −1.64)** ^*∗*^	−3.81 (<0.001)	**231.4 (<0.001)** ^*∗*^	90.9 (87.6, 93.4)	91.5	−3.74, 1.58
Secondary							
Body weight (kg)	20	**−1.66 (−0.87, −2.45)** ^*∗*^	−4.11 (<0.001)	**57.3 (<0.001)** ^*∗*^	66.8 (47.0, 79.2)	78.0	−4.48, 1.17
Fat mass (kg)	14	**−1.07 (−0.36, −1.79)** ^*∗*^	−2.93 (0.003)	**29.5 (0.006)** ^*∗*^	55.9 (19.8, 75.8)	62.6	−3.33, 1.19
Body fat (%)	17	**−1.13 (−0.58, 1.67)** ^*∗*^	−4.05 (<0.001)	**56.2 (<0.001)** ^*∗*^	71.5 (53.6, 82.6)	77.1	−3.16, 0.91
Fat-free mass (kg)	15	−0.006 (−0.24, 0.22)	−0.05 (0.96)	21.8 (0.08)	35.8 (0, 65.4)	39.2	—
VO_2max_ (mL·kg^−1^·min^−1^)^a^	10	**3.1 (1.1, 5.2)** ^*∗*^	2.95 (<0.001)	**197.8 (<0.001)** ^*∗*^	95.4 (93.3, 96.9)	96.8	−4.53, 10.76
Muscular strength (kg)^b^							
Upper	3	**7.7 (4.4, 10.9)** ^*∗*^	4.58 (<0.001)	0.9 (0.63)	0 (0, 92.7)	0	**6.3, 9.0** ^*∗*^
Lower	3	**44.2 (29.5, 59.0)** ^*∗*^	5.88 (<0.001)	0.3 (0.86)	0 (0, 77.3)	0	**42.9, 45.6** ^*∗*^
Energy intake (kcals)	6	−141 (−294, 13)	−1.80 (0.07)	8.9 (0.11)	43.6 (0, 77.7)	59.4	—

*Notes.* ^#^Number; ES: effect size; $\overset{-}{X}$ (95% CI): mean and 95% confidence interval; *Z*(*p*): *Z* value and alpha value for *Z*; *Q*(*p*): Cochrane's *Q* statistic and alpha value for *Q*; *I* ^2^ (%): *I*-squared; 95% PI: 95% prediction intervals; *D* ^2^: *D*-squared; BMI: body mass index; VO_2max_: maximum oxygen consumption; kcals: kilocalories; ^a^data limited to those groups in which aerobic exercise was an intervention; ^b^data limited to those groups in which strength training was an intervention; ^*∗*^statistically significant; —: not calculated; boldface items indicate statistical significance.

## References

[1] Ogden C. L., Carroll M. D., Kit B. K., Flegal K. M. (2014). Prevalence of childhood and adult obesity in the United States, 2011-2012. *The Journal of the American Medical Association*.

[2] National Center for Health Statistics (2012). *Health, United States, 2011: With Special Feature on Socioeconomic Status and Health*.

[3] Ng M., Fleming T., Robinson M. (2014). Global, regional, and national prevalence of overweight and obesity in children and adults during 1980–2013: a systematic analysis for the Global Burden of Disease Study 2013. *The Lancet*.

[4] Finkelstein E. A., Graham W. C. K., Malhotra R. (2014). Lifetime direct medical costs of childhood obesity. *Pediatrics*.

[5] Freedman D. S., Mei Z., Srinivasan S. R., Berenson G. S., Dietz W. H. (2007). Cardiovascular risk factors and excess adiposity among overweight children and adolescents: the Bogalusa Heart Study. *Journal of Pediatrics*.

[6] Paulis W. D., Silva S., Koes B. W., van Middelkoop M. (2014). Overweight and obesity are associated with musculoskeletal complaints as early as childhood: a systematic review. *Obesity Reviews*.

[7] Arens R., Muzumdar H. (2010). Childhood obesity and obstructive sleep apnea syndrome. *Journal of Applied Physiology*.

[8] Griffiths L. J., Parsons T. J., Hill A. J. (2010). Self-esteem and quality of life in obese children and adolescents: a systematic review. *International Journal of Pediatric Obesity*.

[9] Singh A. S., Mulder C., Twisk J. W. R., van Mechelen W., Chinapaw M. J. M. (2008). Tracking of childhood overweight into adulthood: a systematic review of the literature. *Obesity Reviews*.

[10] Danaei G., Ding E. L., Mozaffarian D. (2009). The preventable causes of death in the United States: comparative risk assessment of dietary, lifestyle, and metabolic risk factors. *PLoS Medicine*.

[11] World Health Organization (2014). *Obesity and Overweight*.

[12] American Medical Association (2013). Obesity as a disease. *Policy Statement*.

[13] Lipnowski S., Leblanc C. M. A. (2012). Healthy active living: physical activity guidelines for children and adolescents. *Paediatrics & Child Health*.

[14] European Commission (2014). *EU Action Plan on Childhood Obesity 2014–2020*.

[15] Hoelscher D. M., Kirk S., Ritchie L., Cunningham-Sabo L. (2013). Position of the Academy of Nutrition and Dietetics: interventions for the prevention and treatment of pediatric overweight and obesity. *Journal of the Academy of Nutrition and Dietetics*.

[16] World Health Organization (2011). *Global Recommendations on Physical Activity for Health: 5–17 Year Olds*.

[17] McGovern L., Johnson J. N., Paulo R. (2008). Treatment of pediatric obesity: a systematic review and meta-analysis of randomized trials. *Journal of Clinical Endocrinology and Metabolism*.

[18] Kamath C. C., Vickers K. S., Ehrlich A. (2008). Behavioral interventions to prevent childhood obesity: a systematic review and metaanalyses of randomized trials. *Journal of Clinical Endocrinology and Metabolism*.

[19] Kelley G. A., Kelley K. S., Pate R. R. (2014). Effects of exercise on BMI z-score in overweight and obese children and adolescents: a systematic review with meta-analysis. *BMC Pediatrics*.

[20] Lavelle H. V., MacKay D. F., Pell J. P. (2012). Systematic review and meta-analysis of school-based interventions to reduce body mass index. *Journal of Public Health*.

[21] Atlantis E., Barnes E. H., Singh M. A. F. (2006). Efficacy of exercise for treating overweight in children and adolescents: a systematic review. *International Journal of Obesity*.

[22] Harris K. C., Kuramoto L. K., Schulzer M., Retallack J. E. (2009). Effect of school-based physical activity interventions on body mass index in children: a meta-analysis. *Canadian Medical Association Journal*.

[23] Guerra P. H., Nobre M. R. C., da Silveira J. A. C., de Aguiar Carrazedo Taddei J. A. (2013). The effect of school-based physical activity interventions on body mass index: a meta-analysis of randomized trials. *Clinics*.

[24] Shea B. J., Hamel C., Wells G. A. (2009). AMSTAR is a reliable and valid measurement tool to assess the methodological quality of systematic reviews. *Journal of Clinical Epidemiology*.

[25] Wetterslev J., Thorlund K., Brok J., Gluud C. (2008). Trial sequential analysis may establish when firm evidence is reached in cumulative meta-analysis. *Journal of Clinical Epidemiology*.

[26] Liberati A., Altman D. G., Tetzlaff J. (2009). The PRISMA statement for reporting systematic reviews and meta-analyses of studies that evaluate health care interventions: explanation and elaboration. *Annals of Internal Medicine*.

[28] Lee E., Dobbins M., Decorby K., McRae L., Tirilis D., Husson H. (2012). An optimal search filter for retrieving systematic reviews and meta-analyses. *BMC Medical Research Methodology*.

[29] Microsoft Corporation (2010). *Microsoft Excel. (2007)*.

[30] Cohen J. (1968). Weighted kappa: nominal scale agreement provision for scaled disagreement or partial credit. *Psychological Bulletin*.

[31] Higgins J. P. T., Altman D. G., Gøtzsche P. C. (2011). The Cochrane Collaboration's tool for assessing risk of bias in randomised trials. *British Medical Journal*.

[32] Follmann D., Elliott P., Suh I., Cutler J. (1992). Variance imputation for overviews of clinical trials with continuous response. *Journal of Clinical Epidemiology*.

[33] Dersimonian R., Laird N. (1986). Meta-analysis in clinical trials. *Controlled Clinical Trials*.

[34] Cochran W. G. (1954). The combination of estimates from different experiments. *Biometrics*.

[35] Higgins J. P. T., Thompson S. G., Deeks J. J., Altman D. G. (2003). Measuring inconsistency in meta-analyses. *British Medical Journal*.

[36] Wetterslev J., Thorlund K., Brok J., Gluud C. (2009). Estimating required information size by quantifying diversity in random-effects model meta-analyses. *BMC Medical Research Methodology*.

[37] Higgins J. P. T., Thompson S. G. (2002). Quantifying heterogeneity in a meta-analysis. *Statistics in Medicine*.

[38] Higgins J. P. T., Green S. (2011). *Cochrane Handbook for Systematic Reviews of Interventions*.

[39] Higgins J. P., Thompson S. G., Spiegelhalter D. J. (2009). A re-evaluation of random-effects meta-analysis. *Journal of the Royal Statistical Society: Series A (Statistics in Society)*.

[40] Kelley G. A., Kelley K. S. (2009). Impact of progressive resistance training on lipids and lipoproteins in adults: another look at a meta-analysis using prediction intervals. *Preventive Medicine*.

[41] Sterne J. A. C., Sutton A. J., Ioannidis J. P. A. (2011). Recommendations for examining and interpreting funnel plot asymmetry in meta-analyses of randomised controlled trials. *British Medical Journal*.

[42] Egger M., Smith G. D., Schneider M., Minder C. (1997). Bias in meta-analysis detected by a simple, graphical test. *British Medical Journal*.

[43] Rosenthal R. (1979). The ‘file drawer’ problem and tolerance for null results. *Psychological Bulletin*.

[44] Cali A. M. G., Caprio S. (2008). Obesity in children and adolescents. *Journal of Clinical Endocrinology and Metabolism*.

[45] Haslam D. W., James W. P. T. (2005). Obesity. *The Lancet*.

[46] Cohen J. (1988). *Statistical Power Analysis for the Behavioral Sciences*.

[47] Guyatt G. H., Oxman A. D., Vist G. E. (2008). GRADE: an emerging consensus on rating quality of evidence and strength of recommendations. *British Medical Journal*.

[48] Thorlund K., Devereaux P. J., Wetterslev J. (2009). Can trial sequential monitoring boundaries reduce spurious inferences from meta-analyses?. *International Journal of Epidemiology*.

[49] Brok J., Thorlund K., Gluud C., Wetterslev J. (2008). Trial sequential analysis reveals insufficient information size and potentially false positive results in many meta-analyses. *Journal of Clinical Epidemiology*.

[50] Brok J., Thorlund K., Wetterslev J., Gluud C. (2009). Apparently conclusive meta-analyses may be inconclusive—trial sequential analysis adjustment of random error risk due to repetitive testing of accumulating data in apparently conclusive neonatal meta-analyses. *International Journal of Epidemiology*.

[51] Thorlund K., Engstrom J., Brok J., Imberger G., Gluud C. (2011). *User Manual for Trial Sequential Analysis (TSA)*.

[52] Lazarus R., Wake M., Hesketh K., Waters E. (2000). Change in body mass index in Australian primary school children, 1985–1997. *International Journal of Obesity*.

[53] Higgins J. P. T., Whitehead A., Simmonds M. (2011). Sequential methods for random-effects meta-analysis. *Statistics in Medicine*.

[54] van der Tweela I., Bollenb C. (2010). Sequential meta-analysis: an efficient decision-making tool. *Clinical Trials*.

[55] American College of Sports Medicine (2006). *ACSM's Guidelines for Exercise Testing and Prescription*.

[56] BioStat (2015). *Comprehensive Meta-Analysis. (3.3)*.

[57] Microsoft Corporation (2010). *Microsoft Excel. (2010)*.

[58] Schunemann H., Brozek J., Oxman A. E. (2009). *GRADE Handbook for Grading Quality of Evidence and Strength of Recommendation*.

[59] Statistical Services Center (2007). *SSC-Stat. (2.18)*.

[60] Poynton T. A. (2007). *EZ Analyze (3.0)*.

[61] Alberga A. S., Farnesi B.-C., Lafleche A., Legault L., Komorowski J. (2013). The effects of resistance exercise training on body composition and strength in obese prepubertal children. *The Physician and Sportsmedicine*.

[62] Alves J. G. B., Galé C. R., Souza E., Batty G. D. (2008). Effect of physical exercise on bodyweight in overweight children: a randomized controlled trial in a Brazilian slum. *Cadernos de Saude Publica*.

[63] Elloumi M., Makni E., Ounis O. B. (2011). Six-minute walking test and the assessment of cardiorespiratory responses during weight-loss programmes in obese children. *Physiotherapy Research International*.

[64] Farpour-Lambert N. J., Aggoun Y., Marchand L. M., Martin X. E., Herrmann F. R., Beghetti M. (2009). Physical activity reduces systemic blood pressure and improves early markers of atherosclerosis in pre-pubertal obese children. *Journal of the American College of Cardiology*.

[65] Hagströmer M., Elmberg K., Mårild S., Sjöström M. (2009). Participation in organized weekly physical exercise in obese adolescents reduced daily physical activity. *Acta Paediatrica*.

[66] Karacabey K. (2009). The effect of exercise on leptin, insulin, cortisol and lipid profiles in obese children. *Journal of International Medical Research*.

[67] Kelly A. S., Wetzsteon R. J., Kaiser D. R., Steinberger J., Bank A. J., Dengel D. R. (2004). Inflammation, insulin, and endothelial function in overweight children and adolescents: the role of exercise. *The Journal of Pediatrics*.

[68] Kim E. S., Im J.-A., Kim K. C. (2007). Improved insulin sensitivity and adiponectin level after exercise training in obese Korean youth. *Obesity*.

[69] Kim H. J., Lee S., Kim T. W. (2008). Effects of exercise-induced weight loss on acylated and unacylated ghrelin in overweight children. *Clinical Endocrinology*.

[70] Meyer A. A., Kundt G., Lenschow U., Schuff-Werner P., Kienast W. (2006). Improvement of early vascular changes and cardiovascular risk factors in obese children after a six-month exercise program. *Journal of the American College of Cardiology*.

[71] Murphy E. C.-S., Carson L., Neal W., Baylis C., Donley D., Yeater R. (2009). Effects of an exercise intervention using Dance Dance Revolution on endothelial function and other risk factors in overweight children. *International Journal of Pediatric Obesity*.

[72] Saygin Ö., Öztürk M. A. (2011). The effect of twelve week aerobic exercise programme on health related physical fitness components and blood lipids in obese girls. *African Journal of Pharmacy and Pharmacology*.

[73] Shaibi G. Q., Cruz M. L., Ball G. D. C. (2006). Effects of resistance training on insulin sensitivity in overweight Latino adolescent males. *Medicine & Science in Sports & Exercise*.

[74] Sigal R. J., Alberga A. S., Goldfield G. S. (2014). Effects of aerobic training, resistance training, or both on percentage body fat and cardiometabolic risk markers in obese adolescents: the healthy eating aerobic and resistance training in youth randomized clinical trial. *JAMA Pediatrics*.

[75] Song J.-K., Stebbins C. L., Kim T.-K., Kim H.-B., Kang H.-J., Chai J.-H. (2012). Effects of 12 weeks of aerobic exercise on body composition and vascular compliance in obese boys. *Journal of Sports Medicine and Physical Fitness*.

[76] Sun M.-X., Huang X.-Q., Yan Y. (2011). One-hour after-school exercise ameliorates central adiposity and lipids in overweight Chinese adolescents: a randomized controlled trial. *Chinese Medical Journal*.

[77] Tan S., Yang C., Wang J. (2010). Physical training of 9- to 10-year-old children with obesity to lactate threshold intensity. *Pediatric Exercise Science*.

[78] Watts K., Beye P., Siafarikas A. (2004). Exercise training normalizes vascular dysfunction and improves central adiposity in obese adolescents. *Journal of the American College of Cardiology*.

[79] Watts K., Beye P., Siafarikas A. (2004). Effects of exercise training on vascular function in obese children. *Journal of Pediatrics*.

[80] Wong P. C. H., Chia M. Y. H., Tsou I. Y. Y. (2008). Effects of a 12-week exercise training programme on aerobic fitness, body composition, blood lipids and C-reactive protein in adolescents with obesity. *Annals of the Academy of Medicine, Singapore*.

[81] Davis C. L., Pollock N. K., Waller J. L. (2012). Exercise dose and diabetes risk in overweight and obese children: a randomized controlled trial. *The Journal of the American Medical Association*.

[82] Gutin B., Owens S., Slavens G., Riggs S., Treiber F. (1997). Effect of physical training on heart-period variability in obese children. *Journal of Pediatrics*.

[83] Gutin B., Owens S., Okuyama T., Riggs S., Ferguson M., Litaker M. (1999). Effect of physical training and its cessation on percent fat and bone density of children with obesity. *Obesity Research*.

[84] Maddison R., Foley L., Ni Mhurchu C. (2011). Effects of active video games on body composition: a randomized controlled trial. *American Journal of Clinical Nutrition*.

[85] Kirk S., Zeller M., Claytor R., Santangelo M., Khoury P. R., Daniels S. R. (2005). The relationship of health outcomes to improvement in BMI in children and adolescents. *Obesity Research*.

[86] Rose G. (1985). Sick individuals and sick populations. *International Journal of Epidemiology*.

[87] Haby M. M., Vos T., Carter R. (2006). A new approach to assessing the health benefit from obesity interventions in children and adolescents: the assessing cost-effectiveness in obesity project. *International Journal of Obesity*.

[88] Littell J. H., Corcoran J., Pillai V. (2008). *Systematic Reviews and Meta-Analysis*.

[89] Ho M., Garnett S. P., Baur L. A. (2013). Impact of dietary and exercise interventions onweight change andmetabolic outcomes in obese children and adolescents: a systematic review and meta-analysis of randomized trials. *JAMA Pediatrics*.

[90] Hagströmer M., Oja P., Sjöström M. (2007). Physical activity and inactivity in an adult population assessed by accelerometry. *Medicine and Science in Sports and Exercise*.

[91] Teran-Garcia M., Rankinen T., Bouchard C. (2008). Genes, exercise, growth, and the sedentary, obese child. *Journal of Applied Physiology*.

[92] Oude Luttikhuis H., Baur L., Jansen H. (2009). Interventions for treating obesity in children. *Cochrane Database of Systematic Reviews*.

[93] Matson K. L., Fallon R. M. (2012). Treatment of obesity in children and adolescents. *Journal of Pediatric Pharmacology and Therapeutics*.

[94] Zohrabian A. (2010). Clinical and economic considerations of antiobesity treatment: a review of orlistat. *ClinicoEconomics and Outcomes Research*.

[95] Viner R. M., Hsia Y., Tomsic T., Wong I. C. K. (2010). Efficacy and safety of anti-obesity drugs in children and adolescents: systematic review and meta-analysis. *Obesity Reviews*.

[96] Food and Drug Administration (2014). *What is a Serious Adverse Event?*.

[97] Centers for Disease Control and Prevention (2013). *How Much Physical Activity Do Children Need?*.

[98] Physical Activity Guidelines Advisory Committee (2008). *Physical Activity Guidelines Advisory Report*.

[99] Daniels S. R., Hassink S. G., Committee on Nutrition (2015). The role of the pediatrician in primary prevention of obesity. *Pediatrics*.

[100] Inokuchi M., Matsuo N., Takayama J. I., Hasegawa T. (2011). BMI z-score is the optimal measure of annual adiposity change in elementary school children. *Annals of Human Biology*.

[101] Cole T. J., Faith M. S., Pietrobelli A., Heo M. (2005). What is the best measure of adiposity change in growing children: BMI, BMI %, BMI z-score or BMI centile?. *European Journal of Clinical Nutrition*.

[102] Kakinami L., Henderson M., Chiolero A., Cole T. J., Paradis G. (2014). Identifying the best body mass index metric to assess adiposity change in children. *Archives of Disease in Childhood*.

[103] Reade M. C., Delaney A., Bailey M. J., Angus D. C. (2008). Bench-to-bedside review: Avoiding pitfalls in critical care meta-analysis—funnel plots, risk estimates, types of heterogeneity, baseline risk and the ecologic fallacy. *Critical Care*.

