# Supplementary material for: Exercise and BMI in Overweight and Obese Children and Adolescents: A Systematic Review and Trial Sequential Meta-Analysis
Source: Biomed Res Int. 2015 Oct 22;2015:704539. doi: 10.1155/2015/704539 (PMC4633529; doi:10.1155/2015/704539)
Supplement: Supplementary file 1 — Supplementary File1: This file provides a brief description of each database that was searched for this meta-analysis. Supplementary File 2: This file provides the search strategy used for the updated PubMed search. Supplementary File 3: This file includes a list of excluded references, including the reasons for exclusion. Supplementary File 4: This file povides a study-level assessment of risk of bias for each item. Supplementary File 5: This file provides a list of meta-regression analyses for potential predictors for changes in BMI. Supplementary file 6: This file provides a detailed description of results for the Grades of Recommendation, Assessment, Development and Evaluation (GRADE) Instrument. [file 704539.f1.pdf]

Supplementary File 1. Description of Databases searched.

Academic Search Complete – This database includes most areas of academic study. It contains the full text for 5,500 periodicals, including 4,600 peer-reviewed publications. In addition, it provides indexing and abstracts for more than 9,500 journals.

CINAHL (Cumulative Index of Nursing and Allied Health Literature) - This database is a major source of research for all areas of nursing and allied health. It includes more than 600,000 full-text articles dating back to 1982 and provides the full text for more than 550 nursing allied health journals.

Cochrane Central Register of Controlled Trials – This is a bibliographic database that provides a focused source for randomized controlled trials. Each record includes (1) a list of authors, (2) the title of the article and (3) the source, volume, issue, page numbers, and, in most cases, the abstract of the article. However, the full text of each article is not available.

Education Research Complete – This database provides an online resource for education research. Indexing and abstracts for more than 1,500 journals as well as the full text for more than 750 journals, including the full text for more than 100 books and monographs. In addition, education-related conference papers are available.

ERIC (Education Resources Information Center) - This database, sponsored by the US Department of Education, provides extensive access to educational-related literature and corresponds to two printed journals, Resources in Education (RIE) and Current Index to Journals in Education (CIJE). Both journals provide access to approximately 14,000 documents and more 20,000 journal articles annually. ERIC also provides coverage of conferences, meetings, government documents, theses, dissertations, reports, audiovisual media, bibliographies, directories, books and monographs.

LILACS (Latin American and Caribbean Health Sciences Literature) – This database contains health science literature from Latin American and Caribbean countries. It includes the indexing of theses, books, books chapters, congresses or conferences annals, scientific-technical reports, journal articles, etc., related to health.

Medline – This database provides medical information on medicine, nursing, dentistry, veterinary medicine, the health care system, pre-clinical sciences, and more. Created by the National Library of Medicine in the United States, it indexes the titles and abstracts from over 4,800 current biomedical journals.

Proquest- This database claims to be the world’s most complete collection of dissertations and theses from around the world, ranging from the year 1743 to the

present. It provides full text for graduate works since 1997 selected full text for works written prior to 1997.

Scopus – This database provides abstracts and citations that include both peer-reviewed research literature as well as quality web sources. It provides researchers with resources to support their research needs in the scientific, technical, medical and social sciences fields as well as the arts and humanities.

Sport Discus – This database provides bibliographic coverage of sport, fitness and related disciplines. It contains over 600,000 records with journal and monograph coverage dating back to the year 1800. It also includes multi-disciplinary, international references from journal and magazine articles, books, book chapters, conference proceedings and more.

Web of Science – This database provides access to current and retrospective multidisciplinary information from approximately 8,700 of high impact research journals from around the world.

PubMed – This database is the United States National Library of Medicine's gateway to MEDLINE, a bibliographic database that includes the fields of medicine, nursing, dentistry, veterinary medicine and pre-clinical sciences. MEDLINE includes bibliographic records and author abstracts from more than 5,200 biomedical journals published in the United States as well as 70 other countries.

Supplementary File 2. Search Strategy (updated) for PubMed.

Translations:

exercise	"exercise"[MeSH Terms] OR "exercise"[All Fields]
physical activity	"motor activity"[MeSH Terms] OR ("motor"[All Fields] AND "activity"[All Fields]) OR "motor activity"[All Fields] OR ("physical"[All Fields] AND "activity"[All Fields]) OR "physical activity"[All Fields]
physical fitness	"physical fitness"[MeSH Terms] OR ("physical"[All Fields] AND "fitness"[All Fields]) OR "physical fitness"[All Fields]
strength training	"resistance training"[MeSH Terms] OR ("resistance"[All Fields] AND "training"[All Fields]) OR "resistance training"[All Fields] OR ("strength"[All Fields] AND "training"[All Fields]) OR "strength training"[All Fields]
weight training	"weight lifting"[MeSH Terms] OR ("weight"[All Fields] AND "lifting"[All Fields]) OR "weight lifting"[All Fields] OR ("weight"[All Fields] AND "training"[All Fields]) OR "weight training"[All Fields]
overweight	"overweight"[MeSH Terms] OR "overweight"[All Fields]
Humans[Mesh]	"humans"[MeSH Terms]

Database:

PubMed

User query:

(((exercise OR physical activity OR physical fitness OR strength training OR weight training) AND (child* OR adolescen* OR boys OR girls) AND (overweight OR obes*) AND random*)) AND ("2012/08/01"[Date - Publication] : "3000"[Date - Publication]) AND (Humans[Mesh])

Supplementary File 3. Studies excluded, including specific reasons for exclusion.

(1) Anon. Habit-reversal behavioral therapy effective for children with Tourette disorder. Journal of the National Medical Association 2010;102:968-9. Not an exercise intervention study
Ref ID: 4650

(2) Escoto-Ponce-de-León-MC, Mancilla-Díaz JM, Camacho-Ruiz EJ. A pilot study of the clinical and statistical significance of a program to reduce eating disorder risk factors in children. Eating and Weight Disorders 2008;13:111-8. Not an exercise intervention study
Ref ID: 4651

(3) Weight gain associated with intensive therapy in the diabetes control and complications trial. The DCCT Research Group. Diabetes Care 1988 July;11(7):567-73. Did not meet all selection criteria
Ref ID: 2329

(4) Noticeboard. Lancet 1992 February 15;339(8790):421. Not an exercise intervention study
Ref ID: 3341

(5) Current awareness in geriatric psychiatry. International Journal of Geriatric Psychiatry 1993 July;8(7):619-26. Not an exercise intervention study
Ref ID: 3342

(6) Current awareness in human psychopharmacology. Human Psychopharmacology: Clinical and Experimental 1994 November;9(6):451-8. Review article
Ref ID: 3343

(7) Progestogen-only contraceptives during lactation: I. Infant growth. World Health Organization Task force for Epidemiological Research on Reproductive Health; Special Programme of Research, Development and Research Training in Human Reproduction. Contraception 1994;50:35-53. Not a randomized controlled trial (RCT)
Ref ID: 1029

(8) Research Digest. Pediatric Exercise Science 1995 February;7(1):5-11. Abstract
Ref ID: 3881

(9) Resãmenes. (Spanish). Resumed 10[1], 9. 1997. Abstract,
Ref ID: 3344

(10) Radiation Oncology. Clinical and Investigative Medicine 1998 December 2;21:S84-S99. Not an exercise intervention study
Ref ID: 3345

(11) American Journal Of Clinical Nutrition. Journal of Human Nutrition and Dietetics 11[4], 347-361. 1998. Abstract,
Ref ID: 3346

(12) [A survey on the physical growth of children under 7 years in the urban and suburban rural areas of nine cities of China in 1995. Coordinating Group of Nine Cities on Physical Growth and Development of Children]. Zhonghua yi xue za zhi 1998;78:187-91. Not a randomized controlled trial (RCT), Not an exercise intervention study
Ref ID: 4648

(13) Poster presentations. Mycoses 1999 March;42(3):165-223. Abstract
Ref ID: 545

(14) Proceedings of the 1st Workshop on insulin-dependent diabetes mellitus in children and adolescents. Amman, Jordan, 26-28 October 1997. Acta Paediatrica Supplement 1999 January 3;88(s427):1-51. Abstract
Ref ID: 3350

(15) 3rd Baltic bone and cartilage conference. Acta Orthopaedica Scandinavica 1999 October 2;70:1. Abstract
Ref ID: 3347

(16) Swedish Orthopedic Society. Acta Orthopaedica Scandinavica 1999 October 2;70:47. Abstract
Ref ID: 3348

(17) Proceedings of the Interplanetary Society Conference. Port Ludlow, Washington, USA. 30 May-2 June 1997. Dedicated to Paul B. Beeson, M.D. Journal of Infectious Diseases 1999 March 15;179:iv-S390. Did not meet all selection criteria
Ref ID: 544

(18) In defence of the Milquetoast phenotype. Lancet 1999 May 29;353(9167):1884-5. Editorial or letter or comment
Ref ID: 3349

(19) Writing Group. British HIV Association (BHIVA) guidelines for the treatment of HIV-infected adults with antiretroviral therapy. HIV Medicine 2000 April;1(2):76-101. Not an exercise intervention study
Ref ID: 3355

(20) Correspondence. Lancet 2000 July 15;356(9225):247. Editorial or letter or comment
Ref ID: 3354

(21) New Millennium Research to Practice - Conference Abstracts: 11th World Congress of the International Association for the Scientific Study of Intellectual

Disabilities (IASSID), 1-6 August 2000 Seattle, Washington, USA. Journal of Intellectual Disability Research 44[3/4], 189-528. 2000. Abstract,
Ref ID: 3793

(22) Current awareness. Human psychopharmacology: Clinical and Experimental 2000 April;15(3):221-6. Abstract
Ref ID: 520

(23) Current literature. Journal of Human Nutrition and Dietetics 13[5], 373-386. 2000. Abstract,
Ref ID: 3351

(24) Subject Index for Volume 3. Nutrition in Clinical Care 2000 November;3(6):407. Abstract
Ref ID: 499

(25) Consensus conference on osteoporosis: Prevention, diagnosis and treatment. Española De Enfermedades Metabólicas Óseas 2000;9(6):231-9. Review article
Ref ID: 3185

(26) Proceedings of the South-Eastern Organ Procurement Foundation meeting. 16 September 1999, Memphis, Tennessee, USA. Clinical Transplantation 2000 August 2;14:365-438. Not an exercise intervention study
Ref ID: 3353

(27) Other topics. Allergy 55, 239-282. 8-2-2000. Abstract,
Ref ID: 3352

(28) Occupational and Environmental Allergy. Allergy 55, 152-170. 8-2-2000. Abstract,
Ref ID: 504

(29) Clinical Practice Guideline: Treatment of the school-aged child with attention-deficit/hyperactivity disorder. Pediatrics 2001 October;108(4):1033. Review article
Ref ID: 3360

(30) Track 5 Physical activity, exercise and sports P130-P151. International Journal of Obesity and Related Metabolic Disorders 2001 May 3;25:S68. Abstract
Ref ID: 490

(31) NASPE Abstracts. Pacing and Clinical Electrophysiology 2001 April 15;24(4P2):539-737. Abstract
Ref ID: 3882

(32) What's new in the journals? Diabetic Medicine 2001 October 15;18:10-3. Abstract
Ref ID: 3359

(33) Brown D. Nutritional management of HIV in the era of highly active antiretroviral therapy: a review of treatment strategies. Australian Journal of Nutrition and Dietetics 2001 December;58(4):224. Review article
Ref ID: 3356

(34) Keyword index to volume 25. International Journal of Obesity and Related Metabolic Disorders 2001 December;25(12):1865. Abstract
Ref ID: 478

(35) Poster Group 1 - Paediatrics. Allergy 56, 173-195. 11-2-2001. Wiley-Blackwell. Abstract,
Ref ID: 480

(36) Pediatrics electronic pages. Pediatrics 2001 November;108(5):1193. Abstract
Ref ID: 3358

(37) Posters Group 2 - Environmental and Occupational Health. Allergy 56, 235-250. 11-2-2001. Wiley-Blackwell. Abstract,
Ref ID: 3357

(38) American Transplant Congress. Day-at-a-Glance, Tuesday, April 30, 2002. American Journal of Transplantation 2002 April 2;2:102-29. Abstract
Ref ID: 3370

(39) Conference Abstracts. Fertility Weekly, 20-27. 2-4-2002. Abstract,
Ref ID: 3371

(40) Friday, 19 April 2002. - Plenary lecture. Acta Psychiatrica Scandinavica 105, 32-52. 4-2-2002. Wiley-Blackwell. Abstract,
Ref ID: 3368

(41) American Transplant Congress. Day-at-a-Glance, Sunday, April 28,2002. American Journal of Transplantation 2002 April 2;2:49-71. Abstract
Ref ID: 3369

(42) New technology program grants from the department of education. Techniques: Connecting Education and Careers 2002 May;77(5):6. Review article
Ref ID: 3795

(43) Policy statements adopted by the Governing Council of the American Public Health Association, October 24, 2001. American Journal of Public Health 2002 March;92(3):451-83. Review article
Ref ID: 3796

(44) Women's health literature watch. Journal of Women's Health and Gender-Based Medicine 2002 January;11(1):89-93. Review article
Ref ID: 473

(45) Education fuels budget debate. Techniques: Connecting Education and Careers 2002 May;77(5):6. Editorial or letter or comment
Ref ID: 3794

(46) Abstracts of the Paediatrics and Child Health Division, RACP Annual Scientific Meeting, May 2002. Journal of Paediatrics and Child Health 2002 October;38(5):A1-A18. Abstract
Ref ID: 3363

(47) Abstracts of the 14th Annual Meeting of the German Society of Andrology (DGA) Jena, Germany, 5-7 September 2002. Andrologia 34[4], 263-287. 2002. Abstract,
Ref ID: 3364

(48) Buchert M, Hennig J. Neurochemical imaging (MR spectroscopy) in neuropsychiatric disorders: Perspectives and limitations. European Archives of Psychiatry and Clinical Neuroscience 2002 October 2;252(5):i1. Not an exercise intervention study
Ref ID: 3362

(49) Study makes strong case for early diabetes intervention. Disease Management Advisor 2002 May;8(5):65-8. Review article
Ref ID: 1819

(50) Abstracts from IMCAS, Paris, January 2002. Journal of Cosmetic and Laser Therapy 4[3/4], 93. 2002. Abstract,
Ref ID: 3361

(51) The XXXIV Nordic Meeting of Gastroenterology. Scandinavian Journal of Gastroenterology Supplement 2002 June 2;37:2-38. Abstract
Ref ID: 3367

(52) Author Index. Clinical Endocrinology 2002 June;56(6):829-38. Abstract
Ref ID: 450

(53) Abstracts 401-450. Journal of Sleep Research 2002 June 2;11:201-24. Abstract
Ref ID: 3366

(54) Poster Discussion Session 12: Asthma mechanisms, treatment and quality of life. Allergy 2002 July 2;57:317-23. Abstract
Ref ID: 3365

(55) Guidelines for Referral to Pediatric Surgical Specialists. Pediatrics 2002 July;110(1):187. Survey or questionnaire
Ref ID: 446

(56) Abstracts of the 24th Annual Scientific Meeting of the High Blood Pressure Research Council of Australia Inc. Clinical and Experimental Pharmacology and Physiology 30[7], A31-A68. 2003. Abstract,
Ref ID: 3382

(57) Chronic daily headache. Headache: The Journal of Head and Face Pain 43[10], 1128. 2003. Review article,
Ref ID: 3381

(58) Pediatric Headache. Headache: The Journal of Head and Face Pain 43[10], 1126. 2003. Not an exercise intervention study,
Ref ID: 3380

(59) Correct answers to multiple choice questions appearing in the European Urology Update Series 2002. BJU International 2003 January 15;91(2):155-73. Not an exercise intervention study
Ref ID: 3385

(60) Physicians poster session: Supportive care and regulatory issues. Bone Marrow Transplantation 2003 March 2;31(5):S216. Abstract
Ref ID: 3384

(61) 16th Annual Meeting of the European Musculo-Skeletal Oncology Society & 4th Symposium of the EMSOS Nurse Group, 7-9 May 2003, Budapest: Abstracts. Sarcoma 7[2], 93. 2003. Abstract,
Ref ID: 3383

(62) Saturday 5 April Respiratory Nurses SIG Oral Presentations (1030-1200). Respirology 8, A1-A65. 11-3-2003. Wiley-Blackwell. Abstract,
Ref ID: 3374

(63) Abstracts of the Annual Meeting of the Thoracic Society of Australia and New Zealand. 4-9 April 2003, Adelaide, Australia. Respirology 2003 November 3;8:A1-A67. Abstract
Ref ID: 3375

(64) Cosmetic Ingredient Review Expert Panel. Safety assessment of salicylic acid, butyloctyl salicylate, calcium salicylate, C12-15 alkyl salicylate, capryloyl salicylic acid, hexyldodecyl salicylate, isocetyl salicylate, isodecyl salicylate, magnesium salicylate, MEA-salicylate, ethylhexyl. International Journal of Toxicology (Taylor & Francis) 2003 November 15;22:1-108. Review article
Ref ID: 3373

(65) Author Index to Volume 64. Kidney International 64[6], 2351-2374. 2003. Nature Publishing Group. Abstract,
Ref ID: 3372

(66) Migraine: Treatment. Headache: The Journal of Head and Face Pain 43[10], 1122. 2003. Review article,
Ref ID: 3378

(67) Migraine: Pathophysiology. Headache: The Journal of Head and Face Pain 43[10], 1125. 2003. Not an exercise intervention study,
Ref ID: 3379

(68) Sister Societies. Journal of the European Academy of Dermatology and Venereology 17, 443. 11-2-2003. Abstract,
Ref ID: 3376

(69) Abstracts. Alternative Medicine Review 8[4], 453-466. 2003. Abstract,
Ref ID: 3377

(70) Citations and clinicians' notes: Micronutrients. Current Medical Literature: Clinical Nutrition 13[2], 31-32. 2004. Abstract,
Ref ID: 3402

(71) Saturday 20 March Respiratory Nurses SIG Oral Session (1030-1230). Respirology 2004 March 3;9:A1-A75. Abstract
Ref ID: 3403

(72) Track 7C: Physical activity. International Journal of Obesity and Related Metabolic Disorders 28, S205-S212. 5-2-2004. Abstract,
Ref ID: 3401

(73) Track 5a: Management - Obesity management. International Journal of Obesity and Related Metabolic Disorders 28, S133-S151. 5-2-2004. Abstract,
Ref ID: 3399

(74) Track 6a: Prevention and etiology - Prevention and methods. International Journal of Obesity and Related Metabolic Disorders 28, S162-S166. 5-2-2004. Abstract,
Ref ID: 3400

(75) Oral rehydration therapy is the mainstay of treatment for infectious diarrhoea in children. Drugs and Therapy Perspectives 2004 January;20(1):7-11. Abstract
Ref ID: 3407

(76) Physical health and profound, multiple disabilities. Journal of Intellectual Disability Research 48[4/5], 340-367. 2004. Abstract,
Ref ID: 3797

(77) Correct answers to multiple choice questions appearing in the European Urology Update Series 2003. BJU International 93[2], 228-246. 1-15-2004. Not an exercise intervention study,
Ref ID: 3406

(78) Carr A, Workman C, Carey D, Rogers G, Martin A, Baker D, Wand H, Law M, Samaras K, Emery S, Cooper DA, Rosey investigators. No effect of rosiglitazone for treatment of HIV-1 lipoatrophy: Randomised, double-blind, placebo-controlled trial. Lancet 2004 February 7;363(9407):429-38. Drug intervention study
Ref ID: 3404

(79) Apulo-Lucania Sections Meeting of the Italian Neurological Society and the Italian Society of Hospital Neurosciences: Foggia, Italy November 13-15, 2003. Neurological Sciences 2004 February;24(6):428-41. Abstract
Ref ID: 3405

(80) Abstracts cont. Clinical Microbiology and Infection 10, 179-273. 5-2-2004. Wiley-Blackwell. Abstract,
Ref ID: 3398

(81) 18th Meeting of the Austrian Society of Transplantation, Transfusion and Genetics. European Surgery: ACA Acta Chirurgica Austriaca 2004 November 3;36:1-42. Abstract
Ref ID: 3389

(82) B. Renal Disease and Failure. Kidney 2004 November;13(6):263-9. Abstract
Ref ID: 3390

(83) Endoscopy. Journal of Gastroenterology and Hepatology 19, A223-A273. 10-2-2004. Wiley-Blackwell. Survey or questionnaire,
Ref ID: 3391

(84) Author Index to Volume 66. Kidney International 66[6], 2493-2515. 2004. Nature Publishing Group. Abstract,
Ref ID: 3387

(85) Index. Alimentary Pharmacology and Therapeutics 2004 December;20(11/12):1391-406. Abstract
Ref ID: 3386

(86) Accumulated Table of Contents. Kidney International 66[6], 2516-2535. 2004. Nature Publishing Group. Abstract,
Ref ID: 3388

(87) American Transplant Journal Day at a Glance. American Journal of Transplantation 4, 51-157. 5-2-2004. Wiley-Blackwell. Abstract,
Ref ID: 3395

(88) Part One - The primary headaches. Cephalalgia (Wiley-Blackwell) 24, 23-136. 5-2-2004. Wiley-Blackwell. Not an exercise intervention study,
Ref ID: 3396

(89) 14th European congress of clinical microbiology and infectious diseases, prague, czech republic, 1-4 may 2004. Clinical Microbiology and Infection 10, 1-86. 5-2-2004. Wiley-Blackwell. Abstract,
Ref ID: 3397

(90) Abstracts of the 40th Annual Scientific Meeting of the Australian and New Zealand Society of Nephrology. Adelaide, Australia, 1-3 September 2004. Nephrology 2004 August 2;9:A1-A46. Abstract
Ref ID: 3392

(91) Citations and clinicians' notes: MIcronutrients. Current Medical Literature: Clinical Nutrition 2004 July;13(3):50-3. Not an exercise intervention study
Ref ID: 3393

(92) Citations and clinicians' notes: Hypertension -- Complications. Current Medical Literature: Nephrology and Hypertension 2004 June;10(2):43-6. Not an exercise intervention study
Ref ID: 3394

(93) Correct answers to multiple choice questions appearing in the European Urology Update Series 2004. BJU International 95[2], 250-269. 1-15-2005. Wiley-Blackwell. Not an exercise intervention study,
Ref ID: 3423

(94) Podium And Moderated Poster Session Abstracts. Journal of Sexual Medicine 2, 3-38. 1-2-2005. Wiley-Blackwell. Abstract,
Ref ID: 3424

(95) L-Carnitine. Alternative Medicine Review 2005 March;10(1):42-50. Review article
Ref ID: 3422

(96) Abstracts of the 26th Annual Scientific Meeting of the High Blood Pressure Research Council of Australia, 24-26 November 2004. Clinical and Experimental Pharmacology and Physiology 2005 July;32(7):A1-A27. Abstract
Ref ID: 3420

(97) Late effects and quality of life. Bone Marrow Transplantation 2005 March 17;35:183-94. Not an exercise intervention study
Ref ID: 3421

(98) Free Communications, Poster Presentations: Student poster award finalists. Journal of Athletic Training 40, S-95. 4-2-2005. Abstract,
Ref ID: 3799

(99) Intense diet, behavior, and physical activity intervention effective for obese children. Journal of Family Practice 2005;54:579. No exercise only group, Diet Intervention Study
Ref ID: 4649

(100) 1st IASSID Asian-Pacific Conference Taipei, Taiwan June 12-15, 2005 Abstracts. Journal of Policy and Practice in Intellectual Disabilities 2005 September;2(3/4):176-220. Abstract
Ref ID: 3798

(101) Unmoderated Poster Session Abstracts. Journal of Sexual Medicine 2, 39-87. 1-2-2005. Wiley-Blackwell. Abstract,
Ref ID: 3425

(102) Macronutrients. 14 ed. Remedica Medical Education and Publishing; 2005. p. 12-5. Not an exercise intervention study
Ref ID: 3426

(103) Chronic obstructive pulmonary disease. Current Medical Literature: Respiratory Medicine 19[4], 86-93. 2005. Abstract,
Ref ID: 3411

(104) 19th Meeting of the Austrian Society of Transplantation, Transfusion, and Genetics, October 26-28, 2005. European Surgery: ACA Acta Chirurgica Austriaca 2005 October 3;37:1-46. Abstract
Ref ID: 3412

(105) Diet, lifestyle, and disease. Current Medical Literature: Clinical Nutrition 2005 October;14(4):76-84. Abstract
Ref ID: 3413

(106) Proceedings of the 2nd International Conference on New Insights in Progression of Chronic Kidney Disease, Barcelona, Spain, May 8-10, 2005. Kidney International Supplement 2005 December 2;(99):S-1. Abstract
Ref ID: 3408

(107) Nutrition and growth in kidney disease. Nephrology 2005 December 2;10:S177-S230. Review article
Ref ID: 3409

(108) Congres Annuel De Recherche Dermatologique (Card) French-Speaking Congress Of Dermatological Research Brest (France), MAY 27-28, 2005. Journal of Investigative Dermatology 2005 Dec;A13-A24. Abstract
Ref ID: 3410

(109) Folic acid. Alternative Medicine Review 2005 September;10(3):222-9. Diet Intervention or Supplement Study
Ref ID: 3417

(110) C. Hypertension and systemic disease. Kidney 2005 September;14(5):224-30. Not an exercise intervention study
Ref ID: 3418

(111) Clinical science. Epilepsia (Series 4) 2005 August;46(8):1158-61. Animal study, Diet Intervention Study
Ref ID: 3419

(112) Oral abstracts. Australian and New Zealand Journal of Obstetrics and Gynaecology 45, A1-A19. 9-2-2005. Wiley-Blackwell. Abstract,
Ref ID: 3414

(113) Colon, rectum and small bowel. Journal of Gastroenterology and Hepatology 2005 September 2;20:A13-A40. Abstract
Ref ID: 3415

(114) Satellite symposia. Journal of the European Academy of Dermatology and Venereology 2005 September 2;19:1-17. Abstract
Ref ID: 3416

(115) Proceedings of the Dutch Society of Clinical Pharmacology and Biopharmacy meeting of October 4th 2005. British Journal of Clinical Pharmacology 61[4], 475-486. 2006. Wiley-Blackwell. Abstract,
Ref ID: 3436

(116) Abstracts of the 10th Meeting of the Italian Peripheral Nerve Study Group. Journal of the Peripheral Nervous System 2006 June;11(2):179-208. Abstract
Ref ID: 3435

(117) Prevention of boar taint in pig production: Abstracts of the 19th Symposium of the Nordic Committee for Veterinary Scientific Cooperation Gardermoen, Norway. 21-22 November 2005. Acta Veterinaria Scandinavica 2006 January 2;48:1-3. Abstract, Animal study
Ref ID: 3437

(118) Speaker abstracts. Isokinetics and Exercise Science 2006 June;14(2):111-52. Abstract
Ref ID: 3883

(119) East-European Symposium Central and Peripheral Synaptic Transmission October 5-9, 2005, Varna, Bulgaria. Autonomic and Autacoid Pharmacology 26[1], 31-120. 2006. Wiley-Blackwell. Abstract,
Ref ID: 3438

(120) Abstracts from the 24th Annual Scientific Meeting. Immunology and Cell Biology 2006 June;84(3):A1-A34. Abstract
Ref ID: 3434

(121) Clinical Renal Pharmacology and Therapeutics. Kidney 15[6], 283-292. 2006. Abstract,
Ref ID: 3429

(122) Oral Communications. Diabetic Medicine 2006 December 2;23:612-9. Not an exercise intervention study
Ref ID: 3427

(123) 5-Methyltetrahydrofolate. Alternative Medicine Review 2006 December;11(4):330-7. Review article
Ref ID: 3428

(124) Hepatology. Journal of Gastroenterology and Hepatology 2006 October 3;21:A300-A334. Abstract
Ref ID: 3430

(125) 47th Annual Meeting of the Austrian Society of Surgery, Vienna, June 15-17, 2006. European Surgery: ACA Acta Chirurgica Austriaca 2006 June 2;38:1-121. Abstract
Ref ID: 3433

(126) SSS abstracts. British Journal of Surgery 93[9], 1153-1176. 2006. Abstract,
Ref ID: 3432

(127) Parallel sessions: oral presentations. Liver International 2006 September 2;26:11-22. Abstract
Ref ID: 3431

(128) Poster abstracts. Transfusion Alternatives in Transfusion Medicine 2007 March 2;9:31-59. Abstract
Ref ID: 3448

(129) Swiss Society of Surgery. British Journal of Surgery 94[6], 761-784. 2007. Abstract,
Ref ID: 3447

(130) Recent abstracts. Alternative Medicine Review 12[2], 174-188. 2007. Abstract,
Ref ID: 3446

(131) Oliphant R. An access-dictionary of internationalist high tech Latinate english. Third Education Group Review 3, 1-123. 2007. Third Education Group. Not an exercise intervention study,
Ref ID: 3800

(132) Murdick DM, Venter WDF, Van Rie A, Feldman C. Immune reconstitution inflammatory syndrome (IRIS): review of common infectious manifestations and treatment options. AIDS Research and Therapy 2007 January;4:9-18. Review

article
Ref ID: 3450

(133) 43rd Workshop for Paediatric Research. European Journal of Pediatrics 2007 March;166(3):273-90. Abstract
Ref ID: 3449

(134) 21st Meeting of the Austrian Society of Transplantation, Transfusion and Genetics. St. Wolfgang, October 17-20, 2007. European Surgery: ACA Acta Chirurgica Austriaca 39, 1-43. 10-3-2007. Abstract,
Ref ID: 3440

(135) Nutrition. Journal of Gastroenterology and Hepatology 2007 October 3;22:A403-A412. Abstract
Ref ID: 3441

(136) Effective dietary interventions for overweight and obese children. Australian Nursing Journal 2007 June;14(11):31-4. Review article
Ref ID: 1210

(137) Subject index. BJU International 2007 December;100(6):1451-63. Abstract
Ref ID: 3439

(138) 41st Annual Meeting of the German Speaking Mycological Society (DMykG). Mycoses 2007 September;50(5):334-82. Abstract
Ref ID: 3444

(139) Circadian rhythms. Sleep and Biological Rhythms 2007 August 2;5:A22-A31. Abstract
Ref ID: 3445

(140) Immune enhancement and cancer prevention of a new strain of probiotic lactic acid bacterium (bifidobacterium lactis hn019). Journal of the Society for Integrative Oncology 2007;5(4):181. Diet Intervention or Supplement Study
Ref ID: 3442

(141) Hot papers in the literature. Journal of Women's Health (15409996) 2007 September;16(7):1076-84. Abstract
Ref ID: 3443

(142) Management -- complications and comorbidities. Current Medical Literature: Diabetes 2008 March;25(1):29-33. Abstract
Ref ID: 3462

(143) International Stroke Conference 2008. Clinician Reviews 2008 April;18(4):47-51. Abstract
Ref ID: 3461

(144) 49th Annual Meeting of the Austrian Society of Surgery, Innsbruck, May 21-23, 2008 (Guest Editors: Beate Neuhauser, Dietmar Ãfner, Elfriede Ruttmann-Ulmer, and Anton Schwabegger). European Surgery: ACA Acta Chirurgica Austriaca 40, 1-131. 4-5-2008. Abstract,
Ref ID: 3460

(145) Block G, Jensen CD, Norkus EP, Hudes M, Crawford PB. Vitamin C in plasma is inversely related to blood pressure and change in blood pressure during the previous year in young Black and White women. Nutrition Journal 2008 January;7:35-43. Cross-sectional study
Ref ID: 3465

(146) Dueñas-González A, García-López P, Herrera LA, Medina-Franco JL, González-Fierro A, Candelaria M. The prince and the pauper. A tale of anticancer targeted agents. Molecular Cancer 2008 January;7:82-114. Review article
Ref ID: 3464

(147) A. diagnosis, pathology, and pathophysiology. Kidney 2008 March;17(2):74-9. Not an exercise intervention study
Ref ID: 3463

(148) Oral program. Nutrition and Dietetics 2008 May 2;65:A1-A24. Abstract
Ref ID: 3459

(149) European Tissue Repair Society: Joint Meeting with the Tissue Viability Unit of Malta. Wound Repair and Regeneration 2008 November;16(6):A66-A82. Abstract
Ref ID: 3453

(150) Contributions from International Congress. High Blood Pressure and Cardiovascular Prevention 15[3], 171-222. 2008. Abstract,
Ref ID: 3454

(151) Ocular and aural disorders. Current Medical Literature: Pediatrics 21[4], 138-141. 2008. Abstract,
Ref ID: 3451

(152) Keyword index. Neurogastroenterology and Motility 2008 November 2;20:154-9. Abstract
Ref ID: 3452

(153) Lectures. Acta Neuropsychiatrica 2008 June 2;20:1-50. Abstract
Ref ID: 3457

(154) GH treatment. Current medical literature: Growth, Growth Hormone, and Metabolism 2008 June;2(2):59-61. Abstract
Ref ID: 3458

(155) Oral presentations. Clinical Microbiology and Infection 2008 July 2;14:S1-S119. Abstract
Ref ID: 3455

(156) Posters. Clinical Microbiology and Infection 2008 July 2;14:S121-S666. Abstract
Ref ID: 3456

(157) 6th Meeting of the American Academy of Veterinary Nutrition, Seattle, WA, USA June 6, 2007 Abstracts. Journal of Animal Physiology and Animal Nutrition 2009;93(2):141-5. Abstract, Animal study
Ref ID: 5107

(158) 23rd Meeting of the Austrian Society of Transplantation, Transfusion and Genetics. Seefeld, October 21-23, 2009. Guest Editor: Walter Mark, Innsbruck, Austria. European Surgery: ACA Acta Chirurgica Austriaca 41, 1-38. 10-2-2009. Abstract,
Ref ID: 3466

(159) Unmoderated Posters June 28, 2009, 1200-June 30, 2009, 1030. Canadian Urological Association Journal 3, S61-S82. 6-2-2009. Abstract,
Ref ID: 3467

(160) Congres Annuel De Recherche Dermatologique (CARD) French-Speaking Congress of Dermatological Research Toulouse (France), 12-13 September 2008. Journal of Investigative Dermatology 129[3], 792-809. 2009. Abstract,
Ref ID: 3468

(161) 43. Jahrestagung Physiologie u. Pathologie der Fortpflanzung, gleichzeitig 35. VeterinÃrHumanmedizinische Gemeinschaftstagung Mãnchen, 24. 26. February 2010. Reproduction in Domestic Animals 2010 February 2;45:1-61. Abstract, Animal study
Ref ID: 3478

(162) Elías-Calles LC, CaleroI TMG. Dislipidemia y virus de inmunodeficiencia adquirida/sida. (Spanish). Revista Cubana de Endocrinologa 2010 May;21(2):202-22. Not an exercise intervention study
Ref ID: 3477

(163) Comunicaciones Pãster. (Spanish): Sociedad Espanola de Nutricion Parenteral y Enteral; 2010 p. 477-94.Abstract
Ref ID: 3476

(164) Temas en Cartel. (Spanish). Revista Cubana de Alimentacion y Nutricion 2010 February;20:S24-S129. Abstract
Ref ID: 3479

(165) Nutrition and cancer: from prevention to nutritional support, 8th October 2010, Milan. Ecancermedicalscience 2010 January;4:1-26. Review article
Ref ID: 3482

(166) Proceedings of the VI National Congress of Pharmacology October 2009 Posters. Autonomic and Autacoid Pharmacology 2010 January;30(1):1-65. Abstract
Ref ID: 3481

(167) von Hurst PR, Stonehouse W, Coad J. Insulin sensitivity is improved with vitamin D supplementation in South Asian women who are vitamin D deficient and insulin resistant - a randomised, placebo-controlled trial. Australasian Medical Journal 2[1], 55-56. 2010. Abstract,
Ref ID: 3480

(168) Recently published abstracts. Alternative Medicine Review 15[4], 369-380. 2010. Abstract,
Ref ID: 3469

(169) Contents / Sommaire. Applied Physiology, Nutrition and Metabolism 2010 December;35(6):C-1. Abstract
Ref ID: 3470

(170) TODAY Study Group. Design of a family-based lifestyle intervention for youth with type 2 diabetes: the TODAY study. International Journal of Obesity (London) 2010 February;34(2):217-26. Descrion versus conduct of study
Ref ID: 91

(171) Other complementary therapies. Focus Alternative and Complementary Therapies 2010;15(2):175-9. Review article
Ref ID: 3186

(172) Widmer N, Meylan P, Ivanyuk A, Aouri M, Decosterd LA, Buclin T. Oseltamivir in seasonal, avian H5N1 and pandemic 2009 A/H1N1 influenza. Clinical Pharmacokinetics 2010 November;49(11):741-65. Not an exercise intervention study
Ref ID: 3471

(173) Laxative abuse. Drugs 2010 August;70(12):1487-503. Review article
Ref ID: 3474

(174) Workshop abstracts. Basic and Clinical Pharmacology and Toxicology 2010 July 2;107:71-111. Abstract
Ref ID: 3475

(175) Clinical Practice Clinical Practice Clinical Practice. Journal of Gastroenterology and Hepatology 2010 October 2;25:A18-A43. Abstract
Ref ID: 3472

(176) Keynote Abstracts. Journal of Intellectual Disability Research 2010 October;54(10):881-4. Abstract
Ref ID: 3473

(177) Blanco-Rojo R, Baeza-Richer C, López-Parra AM, Pérez-Granados AM, Brichs A, Bertoncini S, Buil A, Arroyo-Pardo E, Soria JM, Vaquero MPFour variants in transferrin and HFE genes as potential markers of iron deficiency anaemia risk: an association study in menstruating women. Nutrition and Metabolism 2011 January;8(1):69-76. Cross-sectional study
Ref ID: 3493

(178) Marra M, Sordelli IM, Lombardi A, Lamberti M, Tartantino L, Giudice A, Stiuso P, Abbruzzese A, Sperlongano R, Accardo M, Agresti M, Caraglia M, Sperlongano P. Molecular targets and oxidative stress biomarkers in hepatocellular carcinoma: an overview. Journal of Translational Medicine 2011 January;9(1):171-84. Not an exercise intervention study
Ref ID: 3492

(179) Magnusson KT, Sigurgeirsson I, Sveinsson T, Johannsson E. Assessment of a two-year school-based physical activity intervention among 7-9-year-old children. International Journal of Behavioral Nutrition and Physical Activity 2011 January;8(1):138-50. Diet & Exercise intervention, Not All Participants were Overweight and/or Obese
Ref ID: 3491

(180) de Ronde W, de Jong FH. Aromatase inhibitors in men: effects and therapeutic options. Reproductive Biology and Endocrinology 2011 January;9(1):93-9. Drug intervention study
Ref ID: 3496

(181) Fenton TR, Tough SC, Lyon AW, Eliasziw M, Hanley DA. Causal assessment of dietary acid load and bone disease: A systematic review & meta-analysis applying Hill's epidemiologic criteria for causality. Nutrition Journal 2011 January;10(1):41-63. Review article
Ref ID: 3495

(182) Smitka K, Papezova H, Vondra K, Hill M, Hainer V, Nedvidkova J. A higher response of plasma neuropeptide Y, growth hormone, leptin levels and extracellular glycerol levels in subcutaneous abdominal adipose tissue to Acipimox during exercise in patients with bulimia nervosa: single-blind, randomized, microdialysis study. Nutrition and Metabolism (London) 2011 January;8(1):81-94. Study limited to adults
Ref ID: 3494

(183) Iizuka H. Management of patients with psoriasis in Japan. Advances in Psoriasis and Inflammatory Skin Diseases 2011 June;2(4):126-31. Review

article
Ref ID: 3485

(184) Dietitians Association of Australia 29. Nutrition and Dietetics 2011 May 2;68:1-22. Abstract
Ref ID: 3486

(185) What is New in Preventive Medicine? International Journal of Preventive Medicine 2[3], 190-200. 2011. Abstract,
Ref ID: 3484

(186) Concurrent Session 1: Fatty Acids. Australasian Medical Journal 4[12], 791-794. 2011. Abstract,
Ref ID: 3483

(187) Fukuoka Y, Komatsu J, Suarez L, Vittinghoff E, Haskell W, Noorishad T, Pham K. The mPED randomized controlled clinical trial: applying mobile persuasive technologies to increase physical activity in sedentary women protocol. BMC Public Health 2011 January;11(1):933-40. Study limited to adults
Ref ID: 3489

(188) Ansar S, Koska J, Reaven PD. Postprandial hyperlipidemia, endothelial dysfunction and cardiovascular risk: focus on incretins. Cardiovascular Diabetology 2011 January;10(1):61-71. Review article
Ref ID: 3490

(189) A randomized study of alglucosidase alfa in late-onset Pompes disease. Current Medical Literature: Lysosomal Storage Disease 2011 March;9(1):32-3. Drug intervention study
Ref ID: 3487

(190) Shab-Bidar S, Neyestani TR, Djazayery A, Eshraghian MR, Houshiarrad A, Gharavi A, Kalayi A, Shariatzadeh N, Zahedirad M, Khalaji N, Haidari H. Regular consumption of vitamin D-fortified yogurt drink (Doogh) improved endothelial biomarkers in subjects with type 2 diabetes: a randomized double-blind clinical trial. BMC Medicine 2011 January;9(1):125-34. Diet Intervention or Supplement Study
Ref ID: 3488

(191) Houghton D, Onambele GL. Can a standard dose of eicosapentaenoic acid (EPA) supplementation reduce the symptoms of delayed onset of muscle soreness? Journal of the International Society of Sports Nutrition 2012 January;9(1):2-11. Diet Intervention or Supplement Study
Ref ID: 3884

(192) National Student Conference of the Canadian Society for Epidemiology and Biostatistics, Saskatoon, Canada, May 13-14, 2012 Abstracts. American

Journal of Epidemiology 2012;176(1):80. Abstract
Ref ID: 5108

(193) Thorsen L, Nilsen TS, Raastad T, Courneya KS, Skovlund E, Fossa SD. A randomized controlled trial on the effectiveness of strength training on clinical and muscle cellular outcomes in patients with prostate cancer during androgen deprivation therapy: Rationale and design. BMC Cancer 2012 January;12(1):123-32. Description versus conduct of study
Ref ID: 3498

(194) Ugarte M, Brown M, Hollywood KA, Cooper GJ, Bishop PN, Dunn WB. Metabolomic analysis of rat serum in streptozotocin-induced diabetes and after treatment with oral triethylenetetramine (TETA). Genome Medicine 2012 March;4(3):35-49. Animal study
Ref ID: 3497

(195) Giovannini M, Verduci E, Salvatici E, Paci S, Riva E. Phenylketonuria: Nutritional advances and challenges. Nutrition and Metabolism 2012 January;9(1):7-13. Diet Intervention Study
Ref ID: 3500

(196) Effects of a vildagliptin/metformin combination on markers of atherosclerosis, thrombosis, and inflammation in diabetic patients with coronary artery disease. Cardiovascular Diabetology 2012 January;11(1):60-5. Drug intervention study
Ref ID: 3499

(197) No authors listed. Topiramate + phentermine. An excessively dangerous appetite-suppressant combination. Prescrire International 2013 March;22(136):61-4. Inappropriate Study Design
Ref ID: 5721

(198) Aasvee K, Jauhiainen M, Kurvinen E, Jordania R, Sundvall J, Ehnholm C. Lipoprotein(a), apolipoprotein A-I and B serum levels in young families from Tallinn, Estonia. Relationships with other cardiovascular risk factors and nationality. Scandinavian Journal of Clinical and Laboratory Investigation 1999;59(3):179-89. Cross-sectional study
Ref ID: 5109

(199) Aatola H, Koivistoinen T, Hutri-Kahonen N, Juonala M, Mikkila V, Lehtimaki T, Viikari JS, Raitakari OT, Kahonen M. Lifetime fruit and vegetable consumption and arterial pulse wave velocity in adulthood: the Cardiovascular Risk in Young Finns Study. Circulation 2010 December 14;122(24):2521-8. Cohort Study
Ref ID: 2330

(200) Abd El-Kader MS, Al-Jiffri O, Ashmawy EM. Impact of weight loss on markers of systemic inflammation in obese Saudi children with asthma. African Health Sciences 2013 September;13(3):682-8. Inappropriate Intervention
Ref ID: 5722

(201) Abdel-Rahman SA, Shaheen AAM. Efficacy of weight bearing exercises on balance in children with Down syndrome. Egyptian Journal of Neurology, Psychiatry and Neurosurgery 2010;47:37-42. Inappropriate Outcomes
Ref ID: 4654

(202) Abell TL, Van CE, Abrahamsson H, Huizinga JD, Konturek JW, Galmiche JP, Voeller G, Filez L, Everts B, Waterfall WE, Domschke W, Bruley d, V, Familoni BO, Bourgeois IM, Janssens J, Tougas G. Gastric electrical stimulation in intractable symptomatic gastroparesis. Digestion 2002;66(4):204-12. Not an exercise intervention study
Ref ID: 1784

(203) Abete I, Parra D, Crujeiras AB, Goyenechea E, Martinez JA. Specific insulin sensitivity and leptin responses to a nutritional treatment of obesity via a combination of energy restriction and fatty fish intake. Journal of Human Nutrition and Dietetics 2008 December;21(6):591-600. Diet Intervention Study
Ref ID: 3501

(204) Abood DA, Black DR, Coster DC. Evaluation of a school-based teen obesity prevention minimal intervention. Journal of Nutrition Education and Behavior 2008 May;40(3):168-74. Not an exercise intervention study
Ref ID: 186

(205) Abou-Khalil BW. Lacosamide: What can be expected from the next new antiepileptic drug? Epilepsy Currents 2009 September;9(5):133-4. Drug intervention study
Ref ID: 3502

(206) Aboul-Seoud MA, Aboul-Seoud AL. Estimation of body fat from skinfold thickness. Computer Methods and Programs in Biomedicine 2001;65(3):201-6. Not an exercise intervention study
Ref ID: 5110

(207) Abramczyk A. Body mass, behaviours and social/health situation in diabetes patients at the level of primary medical healthcare: a Polish national study. Kardiologia Polska 2013;71(5):493-501. Inappropriate Population
Ref ID: 5723

(208) Abrams EJ, Matheson PB, Thomas PA, Thea DM, Krasinski K, Lambert G, Shaffer N, Bamji M, Hutson D, Grimm K, Kaul A, Bateman D, Rogers M, Beatrice S, Chiasson MA, Debernardo E, Lawrence K, Mcveigh K, Odonnell R, Oleszko W, Punsalang A, Alford T, Betre A, Cappelli M, Courtland R et al. Neonatal predictors of infection status and early death among 332 infants at risk of HIV-1 infection monitored prospectively from birth. Pediatrics 1995;96(3):451-8. Not an exercise intervention study
Ref ID: 5111

(209) Abreu RNDCd, Costa FLPd, Brito EMd, Vasconcelos SMM, Escudeiro SdS, Moreira TMM, Monteiro MGS. Pessoas em recuperação do alcoolismo: avaliação dos fatores de risco cardiovasculares. Personas en la recuperación del alcoholismo: Evaluación de los factores de riesgo cardiovascular.
People in alcoholism recovery: Assessment of cardiovascular risk factors. SMAD, Revista Eletrônica Saúde Mental Álcool e Drogas 2009 August;5(2):1-14. Not an exercise intervention study
Ref ID: 3919

(210) Abt G, Zhou S, Weatherby R. The effect of a high-carbohydrate diet on the skill performance of midfield soccer players after intermittent treadmill exercise. Journal of Science and Medicine in Sport 1998 December;1(4):203-12. Diet Intervention Study
Ref ID: 2068

(211) Abubakar A, Van d, V, Van BA, Mbonani L, Kalu R, Newton C, Holding P. Socioeconomic status, anthropometric status, and psychomotor development of Kenyan children from resource-limited settings: a path-analytic study. Early Human Development 2008;84:613-21. Cross-sectional study
Ref ID: 4655

(212) Abubakr A, Wambacq I. Long-term outcome of vagus nerve stimulation therapy in patients with refractory epilepsy. Journal of Clinical Neuroscience 2008 February;15(2):127-9. Retrospective study
Ref ID: 1014

(213) Aburto NJ, Ramirez-Zea M, Neufeld LM, Flores-Ayala R. The effect of nutritional supplementation on physical activity and exploratory behavior of Mexican infants aged 8-12 months. European Journal of Clinical Nutrition 2010 June;64(6):644-51. Not an exercise intervention study, Subjects less than 2 years old
Ref ID: 53

(214) Acharya SD, Brooks MM, Evans RW, Linkov F, Burke LE. Weight loss is more important than the diet type in improving adiponectin levels among overweight/obese adults. Journal of the American College of Nutrition 2013;32(4):264-71. Inappropriate Population
Ref ID: 5724

(215) Ackel-D'Elia C, Carnier J, Bueno CR, Jr., Campos RM, Sanches PL, Clemente AP, Tufik S, de Mello MT, Damaso AR. Effects of different physical exercises on leptin concentration in obese adolescents. International Journal of Sports Medicine 2014 February;35(2):164-71. Inappropriate Comparison Group
Ref ID: 5725

(216) Ackerman IL, Karn CA, Denne SC, Ensing GJ, Leitch CA. Total rut not resting energy expenditure is increased in infants with ventricular septal defects.

Pediatrics 1998;102(5):1172-7. Subjects less than 2 years old
Ref ID: 5112

(217) Acosta Garcia E. Vigencia del síndrome metabólicoies vigency of metabolic syndromeien vigencia da sindrome metabólica. Acta Bioquímica Clínica Latinoamericana 2011 September;45(3):423-30. Review article
Ref ID: 3920

(218) Adair LS, Kuzawa CW, Borja J. Maternal energy stores and diet composition during pregnancy program adolescent blood pressure. Circulation 2001;104(9):1034-9. Not an exercise intervention study
Ref ID: 5113

(219) Adam S, Westenhofer J, Rudolphi B, Kraaibeek HK. Effects of a combined inpatient-outpatient treatment of obese children and adolescents. Obesity Facts 2009;2(5):286-93. Not a randomized controlled trial (RCT)
Ref ID: 621

(220) Adamo KB, Rutherford JA, Goldfield GS. Effects of interactive video game cycling on overweight and obese adolescent health. Applied Physiology Nutrition and Metabolism 2010 December;35(6):805-15. No comparative control group
Ref ID: 3

(221) Adamo KB, Sheel AW, Onywera V, Waudo J, Boit M, Tremblay MS. Child obesity and fitness levels among Kenyan and Canadian children from urban and rural environments: a KIDS-CAN Research Alliance Study. International Journal of Pediatric Obesity 2011 June;6(2-2):e225-e232. Not an exercise intervention study
Ref ID: 2332

(222) Adamo KB, Ferraro ZM, Goldfield G, Keely E, Stacey D, Hadjiyannakis S, Jean-Philippe S, Walker M, Barrowman NJ. The Maternal Obesity Management (MOM) Trial Protocol: a lifestyle intervention during pregnancy to minimize downstream obesity. Contemporary Clinical Trials 2013 May;35(1):87-96. Inappropriate Population
Ref ID: 5726

(223) Adams AK, LaRowe TL, Cronin KA, Prince RJ, Wubben DP, Parker T, Jobe JB. The Healthy Children, Strong Families intervention: Design and community participation. Journal of Primary Prevention 2012 August;33(4):175-85. Inappropriate Study Design
Ref ID: 5727

(224) Adams RJ, Piantadosi C, Ettridge K, Miller C, Wilson C, Tucker G, Hill CL. Functional health literacy mediates the relationship between socio-economic status, perceptions and lifestyle behaviors related to cancer risk in an Australian population. Patient Education and Counseling 2013 May;91(2):206-

12. Inappropriate Study Design
Ref ID: 5728

(225) Adams TD, Avelar E, Cloward T, Crosby RD, Farney RJ, Gress R, Halverson RC, Hopkins PN, Kolotkin RL, Lamonte MJ, Litwin S, Nuttall RT, Pendleton R, Rosamond W, Simper SC, Smith SC, Strong M, Walker JM, Wiebke G, Yanowitz FG, Hunt SC. Design and rationale of the Utah obesity study. A study to assess morbidity following gastric bypass surgery. Contemporary Clinical Trials 2005 October;26(5):534-51. Description versus conduct of study
Ref ID: 1474

(226) Adegboye AR, Anderssen SA, Froberg K, Sardinha LB, Heitmann BL, Steene-Johannessen J, Kolle E, Andersen LB. Recommended aerobic fitness level for metabolic health in children and adolescents: a study of diagnostic accuracy. British Journal of Sports Medicine 2011 July;45(9):722-8. Cross-sectional study
Ref ID: 2333

(227) Adiputra IN. The improvement effect of Modern Balinese Baris Dancing Exercise on body composition, blood pressure and heart rate. Journal of Human Ergology 1994 December;23(2):93-9. Study limited to adults
Ref ID: 2219

(228) Adiputra N, Alex P, Sutjana DP, Tirtayasa K, Manuaba A. Balinese dance exercises improve the maximum aerobic capacity. Journal of Human Ergology 1996 June;25(1):25-9. Study limited to adults
Ref ID: 2148

(229) Adjemian D, Bustos P, Amigo H. Nivel socioeconómico y estado nutricional: un estudio en escolares. Archivos Latinoamericanos de Nutrición 2007 June;57(2):125-9. Cross-sectional study
Ref ID: 3921

(230) Adkins S, Sherwood NE, Story M, Davis M. Physical activity among African-American girls: The role of parents and the home environment. Obesity Research 2004 September;12:Suppl-45S. Cross-sectional study
Ref ID: 1579

(231) Adu-Afarwuah S, Lartey A, Brown KH, Zlotkin S, Briend A, Dewey KG. Randomized comparison of 3 types of micronutrient supplements for home fortification of complementary foods in Ghana: effects on growth and motor development. American Journal of Clinical Nutrition 86(2):412-20, 2007 Aug 2007;(2):412-20. Diet Intervention or Supplement Study, Not a randomized controlled trial (RCT)
Ref ID: 2872

(232) Aerts D, Chinazzo H, Santos JAd, Oserow NR. Percepção da imagem corporal de adolescentes escolares brancas e não brancas de escolas públicas do Município de Gravataí, Estado do Rio Grande do Sul, Brasil. Body image

perception of white and non-white female adolescents of city's Public Schools in the Municipality of Gravataí, State of Rio Grande do Sul, Brazil. Epidemiologia e Servicos de Saúde 2011 September;20(3):363-72. Cross-sectional study
Ref ID: 3922

(233) AESGP. Herbal medicinal products in the European Union. Pharmaceuticals Policy and Law 1999 June;2(1):55. Not an exercise intervention study
Ref ID: 3503

(234) Africa EK, Van Deventer KJ, Barnard JG. Health risk behaviours of a selected group of adolescent girls. South African Journal for Research in Sport Physical Education and Recreation 2008;30(2):1-14. Survey or questionnaire
Ref ID: 5114

(235) Agah M, Yahyavi P, Roudneshin F. Comparison between classic laryngeal mask and cobra perilaryngeal airway during mechanical ventilation. Tanaffos 2006;5:13-9. Not an exercise intervention study
Ref ID: 4656

(236) Agarwal S, Swanson S, Murphy A, Yaeger K, Sharek P, Halamek LP. Comparing the utility of a standard pediatric resuscitation cart with a pediatric resuscitation cart based on the Broselow tape: a randomized, controlled, crossover trial involving simulated resuscitation scenarios. Pediatrics 2005;116:e326-e333. Not an exercise intervention study
Ref ID: 4657

(237) Agertoft L, Pedersen S. Effects of long-term treatment with an inhaled corticosteroid on growth and pulmonary function in asthmatic children. Respiratory Medicine 1994;88:373-81. Drug intervention study
Ref ID: 4658

(238) Agiovlasitis S, Pitetti KH, Guerra M, Fernhall B. Prediction of VO_2 peak from the 20-m shuttle-run test in youth with down syndrome. Adapted Physical Activity Quarterly 2011 April 1;28(2):146-56. Not an exercise intervention study
Ref ID: 3897

(239) Agrasada GV, Gustafsson J, Kylberg E, Ewald U. Postnatal peer counselling on exclusive breastfeeding of low-birthweight infants: a randomized, controlled trial. Acta Paediatrica 2005;94:1109-15. Study limited to adults
Ref ID: 4659

(240) Agrasada GV, Kylberg E. When and why Filipino mothers of term low birth weight infants interrupted breastfeeding exclusively. Breastfeeding Review 2009;17:5-10. Study limited to adults
Ref ID: 4660

(241) Aguer C, Gavarry O, Gole Y, Boussuges A, Doyard P, Falgairette G. A 5-month weight-reduction programme has a positive effect on body composition, aerobic fitness, and habitual physical activity of severely obese girls: A pilot evaluation study. Journal of Sports Sciences 2010 February;28(3):281-9. Diet & Exercise intervention
Ref ID: 552

(242) Aguilar-Salinas CA, Gómez-Pérez FJ. Declaración de Acapulco: propuesta para la reducción de la incidencia de la diabetes en México.
The "Declaración de Acapulco": a proposal to reduce incidence of diabetes in Mexico. Revista de Investigación Clínica; Organo del Hospital de Enfermedades de la Nutrición 2006 February;58(1):71-7. Review article
Ref ID: 3923

(243) Aguilar Coronado M, Manrique Rajo L, Tuesta Muñoz M, Musayón Oblitas Y. Depresión y autoestima en adolescentes con obesidad y sobrepeso: un problema que pesa: [revisión]. Depression and self-esteem in teenagers with obesity and overweight: a problem that weighs: [review]. Revista Enfermería Herediana 2010 June;3(1):49-54. Review article
Ref ID: 3924

(244) Aguirre C, Castillo D, Le RO. Desafíos emergentes en la nutrición del adolescente. Emergent challenges in adolescent nutrition. Revista Chilena de Pediatría 2010 December;81(6):488-97. Review article
Ref ID: 3925

(245) Ahmed J, Laghari A, Naseer M, Mehraj V. Prevalence of and factors associated with obesity among Pakistani schoolchildren: a school-based, cross-sectional study. Eastern Mediterranean Health Journal J 2013 March;19(3):242-7. Inappropriate Study Design
Ref ID: 5729

(246) Ahmed T, Garrigo J, Danta I. Preventing bronchoconstriction in exercise-induced asthma with inhaled heparin. New England Journal of Medicine 1993 July 8;329(2):90-5. Drug intervention study, Acute study
Ref ID: 2261

(247) Ahola R, Pyky R, Jamsa T, Mantysaari M, Koskimaki H, Ikaheimo TM, Huotari ML, Roning J, Heikkinen HI, Korpelainen R. Gamified physical activation of young men--a Multidisciplinary Population-Based Randomized Controlled Trial (MOPO study). BMC Public Health 2013;13:32. Inappropriate Population
Ref ID: 5730

(248) Ahrens W, Bammann K, de HS, Halford J, Palou A, Pigeot I, Siani A, Sjostrom M, European Consortium of the IDEFICS Project. Understanding and preventing childhood obesity and related disorders--IDEFICS: a European multilevel epidemiological approach. Nutrition Metabolism and Cardiovascular

Diseases 2006 May;16(4):302-8. Description versus conduct of study
Ref ID: 1393

(249) Ainslie PN, Campbell IT, Frayn KN, Humphreys SM, Maclaren DP, Reilly T. Physiological and metabolic responses to a hill walk. Journal of Applied Physiology 2002 January;92(1):179-87. Acute study
Ref ID: 1860

(250) Aires L, Santos R, Silva P, Santos P, Oliveira J, Ribeiro JC, Rego C, Mota J. Daily differences in patterns of physical activity among overweight/obese children engaged in a physical activity program. American Journal of Human Biology 2007 November;19(6):871-7. Cross-sectional study
Ref ID: 1154

(251) Aitchison TC, Durnin JV, Beckett C, Pollitt E. Effects of an energy and micronutrient supplement on growth and activity, correcting for non-supplemental sources of energy input in undernourished children in Indonesia. European Journal of Clinical Nutrition 2000 May;54:Suppl-73. Diet Intervention or Supplement Study
Ref ID: 1968

(252) Akber A, Portale AA, Johansen KL. Pedometer-assessed physical activity in children and young adults with CKD. Clinical Journal of the American Society of Nephrology 2012;7(5):720-6. Cross-sectional study
Ref ID: 5115

(253) Akimoto-Gunther L, Hubler M, Santos M, Carolino I, Sonoo N, Botti B, Mota D, Takahachi G. Effects of re-education in eating habits and physical activity on the lipid profile of obese teenagers. Clinical Chemistry and Laboratory Medicine 2002 May;40(5):460-2. Diet & Exercise intervention
Ref ID: 1809

(254) Akkari M, Waisberg G, Braga SR, Yamada HH, Lundberg JS, Goiano EdO, Figueiredo MJPSSd, Santili C. Osteocondrite de Van Neck-Odelberg: relato de 4 casos. Van neck-odelberg osteochondritis: report on 4 cases. Revista Brasileira de Ortopedia 2010 December;45(supl):55-8. Case-Control / Case Study
Ref ID: 3926

(255) Al-Awadhi N, Al-Kandari N, Al-Hasan T, Almurjan D, Ali S, Al-Taiar A. Age at menarche and its relationship to body mass index among adolescent girls in Kuwait. BMC Public Health 2013;13:29. Inappropriate Study Design
Ref ID: 5731

(256) Al-Haifi AR, Al-Fayez MA, Al-Athari BI, Al-Ajmi FA, Allafi AR, Al-Hazzaa HM, Musaiger AO. Relative contribution of physical activity, sedentary behaviors, and dietary habits to the prevalence of obesity among Kuwaiti adolescents.

Food and Nutrition Bulletin 2013 March;34(1):6-13. Inappropriate Study Design
Ref ID: 5732

(257) Al-Hazzaa HM, Al-Nakeeb Y, Duncan MJ, Al-Sobayel HI, Abahussain NA, Musaiger AO, Lyons M, Collins P, Nevill A. A cross-cultural comparison of health behaviors between Saudi and British adolescents living in urban areas: gender by country analyses. International Journal of Environmental Research and Public Health 2013 December;10(12):6701-20. Inappropriate Study Design
Ref ID: 5733

(258) Al-Hazzaa HM. Joint associations of body mass index and waist-to-height ratio with sleep duration among Saudi adolescents. Annals of Human Biology 2014 March;41(2):111-7. Inappropriate Study Design
Ref ID: 5734

(259) Al-Mousawi AM, Williams FN, Mlcak RP, Jeschke MG, Herndon DN, Suman OE. Effects of exercise training on resting energy expenditure and lean mass during pediatric burn rehabilitation. Journal of Burn Care and Research 31(3):400-8, 2010 May-Jun 2010;(3):400-8. Not All Participants were Overweight and/or Obese
Ref ID: 2873

(260) Al-Muammar MN, El-Shafie M, Feroze S. Association between dietary habits and body mass index of adolescent females in intermediate schools in Riyadh, Saudi Arabia. Eastern Mediterranean Health Journal 2014 January;20(1):39-45. Inappropriate Study Design
Ref ID: 5735

(261) Al-Turkmani MR, Law T, Kellogg MD. Performance evaluation of a particle-enhanced turbidimetric cystatin C assay on the Hitachi 917 analyzer. Clinica Chimica Acta 2008;398(1-2):75-7. Not an exercise intervention study
Ref ID: 5116

(262) Al JA, Abdulle A, Sabri S, Hag-Ali M, Nagelkerke N. The prevalence and potential determinants of obesity among school children and adolescents in Abu Dhabi, United Arab Emirates. International Journal of Obesity (London) 2013 January;37(1):68-74. Inappropriate Study Design
Ref ID: 5736

(263) Alam S, Afzal K, Maheshwari M, Shukla I. Controlled trial of hypo-osmalar versus World Health Organization oral rehydration solution. Indian Pediatrics 2000;37:952-60. Not an exercise intervention study
Ref ID: 4661

(264) Alarcon OM, Guerrero Y, de Fernandez MR, D'Jesus I, Burguera M, Burguera JL, Di Bernardo ML. Effect of copper supplementation on blood pressure values in patients with stable moderate hypertension. Archivos Latinoamericanos de

Nutricion 2003;53(3):271-6. Case-Control / Case Study
Ref ID: 5117

(265) Alayón AN, Castro-Orozco R, Gaviria-Esquivia L, Fernández-Franco M, Benítez-Peña L. Factores de riesgo cardiovascular en escolares entre 7 y 14 años en Cartagena, Colombia, 2009. Cardiovascular risk factors among 7-and 14-year old schoolchildren in Cartagena, Colombia, 2009. Revista de Salud Pública 2011 April;13(2):196-206. Cross-sectional study
Ref ID: 3927

(266) Albano F, Lo VA, Guarino A. The applicability and efficacy of guidelines for the management of acute gastroenteritis in outpatient children: a field-randomized trial on primary care pediatricians. The Journal of Pediatrics 2010;156:226-30. Not an exercise intervention study
Ref ID: 4662

(267) Alberga AS, Frappier A, Sigal RJ, Prudhomme D, Kenny GP. A review of randomized controlled trials of aerobic exercise training on fitness and cardiometabolic risk factors in obese adolescents. Physician and Sportsmedicine 2013 May;41(2):44-57. Inappropriate Study Design
Ref ID: 5738

(268) Albertson AM, Thompson D, Franko DL, Kleinman RE, Barton BA, Crockett SJ. Consumption of breakfast cereal is associated with positive health outcomes: evidence from the National Heart, Lung, and Blood Institute Growth and Health Study. Nutrition Research 2008;28(11):744-52. Diet Intervention Study
Ref ID: 5118

(269) Albertson AM, Thompson D, Franko DL, Holschuh NM, Bauserman R, Barton BA. Prospective associations among cereal intake in childhood and adiposity, lipid levels, and physical activity during late adolescence. Journal of the American Dietetic Association 2009 October;109(10):1775-80. Prospective Study
Ref ID: 671

(270) Alcântara Neto ODd, Silva RdCR, Assis AMO, Pinto EdJ. Fatores associados à dislipidemia em crianças e adolescentes de escolas públicas de Salvador, Bahia. Factors associated with dyslipidemia in children and adolescents enrolled in public schools of Salvador, Bahia. Revista Brasileira de Eidemiologia 2012 June;15(2):335-45. Cross-sectional study
Ref ID: 3928

(271) Alemu T, Lindtjorn B. Physical activity, illness and nutritional status among adults in a rural Ethiopian community. International Journal of Epidemiology 1995 October;24(5):977-83. Study limited to adults
Ref ID: 2193

(272) Alencar FH, Yuyama LKO, Varejão MdJ, Marinho HA. Determinantes e conseqüências da insegurança alimentar no Amazonas: a influência dos ecossistemas. Acta Amazonica 2007;37(3):413-8. Review article
Ref ID: 574

(273) Alexander D. Prevention of mental retardation: Four decades of research This article is a US Government work and, as such, is in the public domain in the United States of America. Mental Retardation and Developmental Disabilities Research Reviews 1998 February;4(1):50-8. Review article
Ref ID: 3504

(274) Alexander ND, Cousens SN, Yahaya H, Abiose A, Jones BR. Ivermectin dose assessment without weighing scales. Bulletin of the World Health Organization 1993;71:361-6. Drug intervention study
Ref ID: 1039

(275) Alexeeva N, Sames C, Jacobs PL, Hobday L, Distasio MM, Mitchell SA, Calancie B. Comparison of training methods to improve walking in persons with chronic spinal cord injury: a randomized clinical trial. Journal of Spinal Cord Medicine 2011;34(4):362-79. Study limited to adults
Ref ID: 1023

(276) Alfie ME, Treem WR. Nonalcoholic fatty liver disease. Pediatric Annals 2006;35(4):290. Review article
Ref ID: 5119

(277) Alhassan S, Sirard JR, Robinson TN. The effects of increasing outdoor play time on physical activity in Latino preschool children. International Journal of Pediatric Obesity 2007;2(3):153-8. Not All Participants were Overweight and/or Obese
Ref ID: 1135

(278) Ali A, Williams C. Carbohydrate ingestion and soccer skill performance during prolonged intermittent exercise. Journal of Sports Sciences 2009 December;27(14):1499-508. Diet Intervention or Supplement Study
Ref ID: 612

(279) Alkandari JR, Maughan RJ, Roky R, Aziz AR, Karli U. The implications of Ramadan fasting for human health and well-being. Journal of Sports Sciences 2012;30:S9-S19. Diet Intervention Study
Ref ID: 5120

(280) Allan JD. New directions for the study of overweight. Western Journal of Nursing Research 20(1):7-13, 1998 Feb 1998;(1):7-13. Review article
Ref ID: 2874

(281) Allan M, Richardson GM, Jones-Otazo H. Probability density functions describing 24-hour inhalation rates for use in human health risk assessments:

An update and comparison. Human and Ecological Risk Assessment 2008;14(2):372-91. Not an exercise intervention study
Ref ID: 5121

(282) Allor KM, Pivarnik JM, Sam LJ, Perkins CD. Treadmill economy in girls and women matched for height and weight. Journal of Applied Physiology 2000 August;89(2):512-6. Acute study
Ref ID: 1966

(283) Almagiá Flores AA, Rodríguez Rodríguez F, Barrraza Gómez FO, Lizana Arce PJ, Jorquera Aguilera CA. Perfil antropométrico de jugadoras chilenas de fútbol femenino. International Journal of Morphology 2008 December;26(4):817-21. Cross-sectional study
Ref ID: 3929

(284) Almagià Flores AA, Rodríguez Rodríguez F, Barrraza Gómez FO, Lizana Arce PJ, Ivanovic Marincovich D, Binvignat Gutiérrez O. Perfil antropométrico de jugadores profesionales de voleibol sudamericano. International Journal of Morphology 2009 March;27(1):53-7. Not an exercise intervention study
Ref ID: 3930

(285) Almeida A, Roveda G, Valin MR, Almeida NCd, Sartor V, Alves SM. Complicações da técnica de fixação tibial com parafuso e arruela para a reconstrução ligamentar do joelho. Complications of the screw/washer tibial fixation technique for knee ligament reconstruction. Revista Brasileira de Ortopedia 2010;45(5):409-14. Not an exercise intervention study
Ref ID: 3931

(286) Almeida Ed, Gonçalves A, El-Khatib S, Padovani CR. Lesão muscular após diferentes métodos de treinamento de musculação. Fisioterapia em Movimento 2006 December;19(4):17-23. Acute study
Ref ID: 599

(287) Almeida FAd, Konigsfeld HP, Machado LMdO, Canadas AF, Issa EYO, Giordano RH, Cadaval RAdM. Avaliação de influências sociais e econômicas sobre a pressão arterial de adolescentes de escolas públicas e privadas: um estudo epidemiológico. Assessment of social and economic influences on blood pressure of adolescents in public and private schools: an epidemiological study. Jornal Brasileiro de Nefrologia 2011 June;33(2):142-9. Cross-sectional study
Ref ID: 3932

(288) Almeida GPL, Carneiro KKA, Morais HCRd, Oliveira JBBd. Influência do alongamento dos músculos isquiostibial e retofemoral no pico de torque e potência máxima de joelho. Fisioterapia e Pesquisa 2009 December;16(4):346-51. No aerobic exercise or WT intervention
Ref ID: 3933

(289) Almeida MFBd, Guinsburg R, Costa JOd, Anchieta LM, Freire LMS. Material and human resources for neonatal resuscitation in public maternity hospitals in Brazilian state capitals. Recursos materiais e humanos para a reanimação neonatal nas maternidades públicas das capitais brasileiras. São Paulo Medical Journal 2008 May;126(3):156-60. Cross-sectional study
Ref ID: 3934

(290) Almeida TAd, Soares EA. Nutritional and anthropometric profile of adolescent volleyball athletes. Revista Brasileira de Medicina do Esporte 2003 August;9(4):198-203. Cross-sectional study
Ref ID: 3935

(291) Almuzaini KS. Muscle function in Saudi children and adolescents: relationship to anthropometric characteristics during growth. Pediatric Exercise Science 2007 August;19(3):319-33. Cross-sectional study
Ref ID: 1141

(292) Alricsson M, Landstad BJ, Romild U, Gundersen KT. Physical activity, health, BMI and body complaints in high school students. Minerva Pediatrica 2008 February;60(1):19-25. Survey or questionnaire
Ref ID: 999

(293) Als H, Lawhon G, Duffy FH, McAnulty GB, Gibes GR, Blickman JG. Individualized developmental care for the very low-birth-weight preterm infant. Medical and neurofunctional effects. Journal of the American Medical Association 1994;272:853-8. Subjects less than 2 years old
Ref ID: 4663

(294) Althuizen E, van Poppel MN, Seidell JC, van der Wijden C, van MW. Design of the New Life(style) study: a randomised controlled trial to optimise maternal weight development during pregnancy. [ISRCTN85313483]. BMC Public Health 2006;6:168. Study limited to adults
Ref ID: 1365

(295) Alvarado BE, Zunzunegui MV, Beland F, Bamvita JM. Life course social and health conditions linked to frailty in Latin American older men and women. Journals of Gerontology Series A-Biological Sciences and Medical Sciences 2008 December;63(12):1399-406. Cross-sectional study
Ref ID: 835

(296) Alvarado R, López Moreno JM. Hamartoma hipotalámico, una causa de pubertad precoz: caso clínico. Revista médica de Chile 2001 October;129(10):1179-82. Case-Control / Case Study
Ref ID: 760

(297) Alvarez-Jimenez M, Gonzalez-Blanch C, Vazquez-Barquero JL, Perez-Iglesias R, Martinez-Garcia O, Perez-Pardal T, Ramirez-Bonilla ML, Crespo-Facorro B. Attenuation of antipsychotic-induced weight gain with early behavioral

intervention in drug-naive first-episode psychosis patients: A randomized controlled trial. Journal of Clinical Psychiatry 2006 August;67(8):1253-60. Study limited to adults
Ref ID: 269

(298) Alvarez M, Sedano S, Cuadrado G, Redondo JC. Effects of an 18-week strength training program on low-handicap golfers' performance. Journal of Strength and Conditioning Research 2012 April;26(4):1110-21. Study limited to adults
Ref ID: 2335

(299) Alves C, Oliveira AC, Brites C. Lipodystrophic syndrome in children and adolescents infected with the human immunodeficiency virus. Brazilian Journal of Infectious Diseases 2008 August;12(4):342-8. Review article
Ref ID: 3937

(300) Alves C, Lima RVB. Impacto da atividade física e esportes sobre o crescimento e puberdade de crianças e adolescentes: [revisão]. Linear growth and puberty in children and adolescents: effects of physical activity and sports: [revision]. Revista Paulista de Pediatria 2008 December;26(4):383-91. Review article
Ref ID: 3936

(301) Alves E, Henriques A, Correia S, Santos AC, Azevedo A, Barros H. Cardiovascular risk profile of mothers of a Portuguese birth cohort: a survey 4 years after delivery. Preventive Medicine 2013 November;57(5):494-9. Inappropriate Study Design
Ref ID: 5739

(302) Alves JGB, Siqueira PP, Figueiroa JN. Excesso de peso e inatividade física em crianças moradoras de favelas na região metropolitana do Recife, PE. Overweight and physical inactivity in children living in favelas in the metropolitan region of Recife, Brazil. Jornal de Pediatria 2009 February;85(1):67-71. Cross-sectional study
Ref ID: 3939

(303) Alves SS, Silva SRCd, Ribeiro RS, Vertematti AS, Fisberg M. Avaliaçäo de atividade física, estado nutricional e condiçäo social em adolescentes. Folha Medica 2000 March;119(1):26-33. Survey or questionnaire
Ref ID: 3940

(304) Aman J, Skinner TC, de Beaufort CE, Swift PG, Aanstoot HJ, Cameron F, Hvidoere Study Group on Childhood Diabetes. Associations between physical activity, sedentary behavior, and glycemic control in a large cohort of adolescents with type 1 diabetes: the Hvidoere Study Group on Childhood Diabetes. Pediatric Diabetes 2009 June;10(4):234-9. Cross-sectional study
Ref ID: 740

(305) Aman MG, McDougle CJ, Scahill L, Handen B, Arnold LE, Johnson C, Stigler KA, Bearss K, Butter E, Swiezy NB, Sukhodolsky DD, Ramadan Y, Pozdol SL, Nikolov R, Lecavalier L, Kohn AE, Koenig K, Hollway JA, Korzekwa P, Gavaletz A, Mulick JA, Hall KL, Dziura J, Ritz L, Trollinger S et al. Medication and parent training in children with pervasive developmental disorders and serious behavior problems: Results from a randomized clinical trial. Journal of the American Academy of Child and Adolescent Psychiatry 2009;48:1143-54. Drug intervention study
Ref ID: 4664

(306) Amaro S, Viggiano A, Di CA, Madeo I, Viggiano A, Baccari ME, Marchitelli E, Raia M, Viggiano E, Deepak S, Monda M, De LB. Kaledo, a new educational board-game, gives nutritional rudiments and encourages healthy eating in children: A pilot cluster randomized trial. European Journal of Pediatrics 2006 September;165(9):630-5. Not an exercise intervention study
Ref ID: 277

(307) Amaya RA, Kozinetz CA, McMeans A, Schwarzwald H, Kline MW. Lipodystrophy syndrome in human immunodeficiency virus-infected children. Pediatric Infectious Disease Journal 2002;21(5):405-10. Cross-sectional study
Ref ID: 5122

(308) Ambalavanan N, Tyson JE, Kennedy KA, Hansen NI, Vohr BR, Wright LL, Carlo WA, -National-Institute-of-Child-Health-and-Human-Development-Neonatal-Research-Network. Vitamin A supplementation for extremely low birth weight infants: Outcome at 18 to 22 months. Pediatrics 2005;115:e249-e254. Subjects less than 2 years old
Ref ID: 4666

(309) Ambalavanan N, Carlo WA, Bobashev G, Mathias E, Liu B, Poole K, Fanaroff AA, Stoll BJ, Ehrenkranz R, Wright LL. Prediction of death for extremely low birth weight neonates. Pediatrics 2005;116(6):1367-73. Subjects less than 2 years old
Ref ID: 5123

(310) Ambler C, Eliakim A, Brasel JA, Lee WN, Burke G, Cooper DM. Fitness and the effect of exercise training on the dietary intake of healthy adolescents. International Journal of Obesity and Related Metabolic Disorders 1998 April;22(4):354-62. Cross-sectional study, Not All Participants were Overweight and/or Obese
Ref ID: 2094

(311) Amenta M, Cascio MT, Di FP, Venturini I. Diet and chronic constipation. Benefits of oral supplementation with symbiotic zir fos (Bifidobacterium longum W11 + FOS Actilight). Acta Bio-Medica de l Ateneo Parmense 2006 December;77(3):157-62. Drug intervention study
Ref ID: 1303

(312) American Dietetic Association. Position of the American Dietetic Association: individual-, family-, school-, and community-based interventions for pediatric overweight. Journal of the American Dietetic Association 2006 June;106(6):925-45. Review article
Ref ID: 1381

(313) Amigo C, Bustos Muñoz P, Radrigán Kiguel ME, Ureta H. Estado nutricional en escolares de nivel socioeconómico opuesto. Revista médica de Chile 1995 September;123(9):1063-70. Cross-sectional study
Ref ID: 3941

(314) Amigo H, Bustos P, Erazo M, Cumsille P, Silva C. Factores determinantes del exceso de peso en escolares: Un estudio multinivel. Revista médica de Chile 2007 December;135(12):1510-8. Cross-sectional study
Ref ID: 3942

(315) Amigo H, Erazo M, Bustos P, Aguilar C, Taibo M. Vigilancia nutricional en escolares chilenos: validez de la información. Revista médica de Chile 2008 August;136(8):989-95. Cross-sectional study
Ref ID: 3943

(316) Aminian S, Hinckson EA. Examining the validity of the ActivPAL monitor in measuring posture and ambulatory movement in children. International Journal of Behavioral Nutrition and Physical Activity 2012;9:119. Inappropriate Study Design
Ref ID: 5740

(317) Amorim Adegboye AR, Linne YM. Diet or exercise, or both, for weight reduction in women after childbirth. Cochrane Database of Systematic Reviews 2013;7:CD005627. Inappropriate Population
Ref ID: 5741

(318) Amorim AR, Linne YM, Lourenco PM. Diet or exercise, or both, for weight reduction in women after childbirth. [Review] [68 refs]. Cochrane Database of Systematic Reviews (3):CD005627, 2007 2007;(3):CD005627. Review article
Ref ID: 1199

(319) Amorim MMRd, Melo ASdO, Cardoso MAA, Assunção PLd. Atividade física durante a gravidez: revisão e recomendações. Femina 2007 August;35(8):521-7. Review article
Ref ID: 3944

(320) Amorim PG, Mendes TdB, Oliveira LSPd, Guerra-Júnior G, Ribeiro JD. Hormônio de crescimento em crianças e adolescentes com fibrose cística. Growth hormone in children and adolescents with cystic fibrosis. Arquivos Brasileiros de Endocrinologia and Metabologia 2011 December;55(9):671-6. Review article
Ref ID: 3945

(321) Amos A, Currie C, Hunt SM, Martin CJ. Health-related behaviour in a small Scottish community. Public Health 1990 March;104(2):131-40. Survey or questionnaire
Ref ID: 2310

(322) Ampuero S, Bee G. The potential to detect boar tainted carcasses by using an electronic nose based on mass spectrometry. Acta Veterinaria Scandinavica 2006 January 2;48:1-2. Animal study
Ref ID: 3505

(323) An JY, Hayman LL, Park YS, Dusaj TK, Ayres CG. Web-based weight management programs for children and adolescents: a systematic review of randomized controlled trial studies. [Review] [50 refs]. Advances in Nursing Science 32(3):222-40, 2009 Jul-Sep 2009;(3):222-40. Review article
Ref ID: 2875

(324) An P, Perusse L, Rankinen T, Borecki IB, Gagnon J, Leon AS, Skinner JS, Wilmore JH, Bouchard C, Rao DC. Familial aggregation of exercise heart rate and blood pressure in response to 20 weeks of endurance training: the HERITAGE family study. International Journal of Sports Medicine 2003 January;24(1):57-62. Study limited to adults
Ref ID: 1774

(325) An P, Teran-Garcia M, Rice T, Rankinen T, Weisnagel SJ, Bergman RN, Boston RC, Mandel S, Stefanovski D, Leon AS, Skinner JS, Rao DC, Bouchard C, HERITAGE Family. Genome-wide linkage scans for prediabetes phenotypes in response to 20 weeks of endurance exercise training in non-diabetic whites and blacks: the HERITAGE Family Study. Diabetologia 2005 June;48(6):1142-9. Study limited to adults
Ref ID: 1504

(326) An P, Borecki IB, Rankinen T, Despres JP, Leon AS, Skinner JS, Wilmore JH, Bouchard C, Rao DC. Evidence of major genes for plasma HDL, LDL cholesterol and triglyceride levels at baseline and in response to 20 weeks of endurance training: the HERITAGE Family Study. International Journal of Sports Medicine 2005 July;26(6):414-9. Study limited to adults
Ref ID: 1492

(327) Anagnostis P. Metabolic syndrome in the Mediterranean region: Current status. Indian Journal of Endocrinology and Metabolism 2012 January;16(1):72-80. Review article
Ref ID: 3506

(328) Anand SS, Davis AD, Ahmed R, Jacobs R, Xie C, Hill A, Sowden J, Atkinson S, Blimkie C, Brouwers M, Morrison K, de KL, Gerstein H, Yusuf S. A family-based intervention to promote healthy lifestyles in an aboriginal community in Canada. Canadian Journal of Public Health 2007 November;98(6):447-52. No exercise

only group
Ref ID: 147

(329) Andersen JR. Sorting criteria. Methods for on-line/at-line sorting of entire male carcasses with emphasis on the Danish method based on skatole content. Acta Veterinaria Scandinavica 2006 January 2;48:S14-3. Review article
Ref ID: 3507

(330) Andersen LB. Tracking of risk factors for coronary heart disease from adolescence to young adulthood with special emphasis on physical activity and fitness. A longitudinal study. Danish Medical Bulletin 1996;43:407-18. Cohort Study
Ref ID: 988

(331) Andersen LB, Sardinha LB, Froberg K, Riddoch CJ, Page AS, Anderssen SA. Fitness, fatness and clustering of cardiovascular risk factors in children from Denmark, Estonia and Portugal: the European Youth Heart Study. International Journal of Pediatric Obesity 2008;3:Suppl-66. Cross-sectional study
Ref ID: 997

(332) Anderson JW, Greenway FL, Fujioka K, Gadde KM, McKenney J, O'Neil PM. Bupropion SR enhances weight loss: a 48-week double-blind, placebo-controlled trial. Obesity Research 2002 July;10(7):633-41. Study limited to adults
Ref ID: 377

(333) Anderson ML, Foster C, McGuigan MR, Seebach E, Porcari JP. Training vs. body image: does training improve subjective appearance ratings? Journal of Strength and Conditioning Research 2004 May;18(2):255-9. Study limited to adults
Ref ID: 1625

(334) Anderson NA, Raafat A, Shwe KH, Barbara J, Contreras M, Fraser ID, Gunson HH, Martlew V, Mijovic V, Goldie DJ. U.K. multicentre study on blood donors for surrogate markers of non-A non-B hepatitis. Part I: Alanine transferase and anti-HBc testing. Transfusion Medicine 1992 December;2(4):301-10. Not an exercise intervention study
Ref ID: 2274

(335) Anderson SE, Bandini LG, Dietz WH, Must A. Relationship between temperament, nonresting energy expenditure, body composition, and physical activity in girls. International Journal of Obesity 2004;28(2):300-6. Survey or questionnaire
Ref ID: 5124

(336) Anderson SE, Bandini LG, Must A. Child temperament does not predict adolescent body composition in girls. International Journal of Obesity

2005;29(1):47-53. Cross-sectional study
Ref ID: 5125

(337) Ando J, Nonaka K, Ozaki K, Sato N, Fujisawa KK, Suzuki K, Yamagata S, Takahashi Y, Nakajima R, Kato N, Ooki S. The Tokyo Twin Cohort Project: Overview and initial findings. Twin Research and Human Genetics 2006;9(6):817-26. Cohort Study
Ref ID: 5126

(338) Andrade FBd, Caldas Junior AdF, Kitoko PM, Batista JEM, Andrade TBd. Prevalence of overweight and obesity in elderly people from Vitória-ES, Brazil. Prevalência de sobrepeso e obesidade em idosos da cidade de Vitória-ES, Brasil. Ciência and Saúde Coletiva 2012 March;17(3):749-56. Cross-sectional study, Study limited to adults
Ref ID: 3946

(339) Andrade JC, Andrade VS, Buffolo E, Greco OT, Lopes MG, Macedo Júnior A, Menezes Júnior AdS, Moraes AV, Mota NJM, Pachón JC, Schaldach M, Tebexreni AS, Tomas AA. Avaliaçäo do sensor de contratilidade cardíaca em sistema DDDR: estudo multicêntrico. Revista Brasileira de Cirurgia Cardiovascular 1998 December;13(4):340-50. Study limited to adults
Ref ID: 3947

(340) Andrade KC, Souza SB, Szarfarc SC. Desenvolvimento neuromotor e dentição de crianças atendidas em serviços públicos de saúde do Brasil, no primeiro ano de vida. Revista Brasileira de Crescimento e Desenvolvimento Humano 2007 June;17(2):37-44. Cross-sectional study
Ref ID: 3948

(341) Andreasi V, Michelin E, Rinaldi AE, Burini RC. Aptidão física associada às medidas antropométricas de escolares do ensino fundamental.
Physical fitness and associations with anthropometric measurements in 7 to 15-year-old school children. Jornal de Pediatria 2010 December;86(6):497-502. Cross-sectional study
Ref ID: 3949

(342) Andreou E, Philippou C, Papandreou D. Effects of an intervention and maintenance weight loss diet with and without exercise on anthropometric indices in overweight and obese healthy women. Annals of Nutrition and Metabolism 2011;59(2-4):187-92. Study limited to adults
Ref ID: 2336

(343) Andresen Ãy. Boar taint related compounds: Androstenone/skatole/other substances. Acta Veterinaria Scandinavica 2006 January 2;48:S5-4. Animal study
Ref ID: 3508

(344) Andriolo RB, El Dib RP, Ramos L, Atallah AN, da Silva EM. Aerobic exercise training programmes for improving physical and psychosocial health in adults with Down syndrome. [Review] [138 refs][Update of Cochrane Database of Systematic Reviews. 2009;(3):CD005176; PMID: 19588368]. Cochrane Database of Systematic Reviews (5):CD005176, 2010 2010;(5):CD005176. Review article
Ref ID: 535

(345) Angeles-Agdeppa I, Lana RD, Barba CV. A case study on dual forms of malnutrition among selected households in District 1, Tondo, Manila. Asia Pacific Journal of Clinical Nutrition 2003;12(4):438-46. Survey or questionnaire
Ref ID: 1686

(346) Angelini C, Pegoraro E, Turella E, Intino MT, Pini A, Costa C. Deflazacort in Duchenne dystrophy: study of long-term effect.[Erratum appears in Muscle Nerve 1994 Jul;17(7):833]. Muscle and Nerve 1994 April;17(4):386-91. Drug intervention study
Ref ID: 1085

(347) Angelini C, Semplicini C, Ravaglia S, Bembi B, Servidei S, Pegoraro E, Moggio M, Filosto M, Sette E, Crescimanno G, Tonin P, Parini R, Morandi L, Marrosu G, Greco G, Musumeci O, Di Iorio G, Siciliano G, Donati MA, Carubbi F, Ermani M, Mongini T, Toscano A. Observational clinical study in juvenile-adult glycogenosis type 2 patients undergoing enzyme replacement therapy for up to 4 years. Journal of Neurology 2012;259(5):952-8. Observational study
Ref ID: 3188

(348) Angelopoulos PD, Milionis HJ, Grammatikaki E, Moschonis G, Manios Y. Changes in BMI and blood pressure after a school based intervention: the CHILDREN study. European Journal of Public Health 2009;19:319-25. Not All Participants were Overweight and/or Obese
Ref ID: 4667

(349) Angulo BR, Burghardt AR, Lloyd M, Ulrich DA. Physical activity in infants with Down syndrome receiving a treadmill intervention. Infant Behavior and Development 2008;31:255-69. Subjects less than 2 years old
Ref ID: 4668

(350) Annagür, Bilge Burcak. Risk Factors and Impulsivity in Obesity (Turkish). Current Approaches in Psychiatry / Psikiyatride Guncel Yaklasimlar 2010 November;2(4):572-82. Review article
Ref ID: 3509

(351) Annesi JJ, Smith AE, Tennant GA. Effects of a cognitive-behaviorally based physical activity treatment for 4- and 5-year-old children attending US preschools. International Journal of Behavioral Medicine 2013

December;20(4):562-6. Inappropriate Study Design
Ref ID: 5742

(352) Annesi JJ, Smith AE, Tennant GA. Effects of the Start For Life treatment on physical activity in primarily African American preschool children of ages 3-5 years. Psychology, Health and Medicine 2013;18(3):300-9. Inappropriate Population
Ref ID: 5744

(353) Annesi JJ, Smith AE, Tennant GA. Reducing high BMI in African American preschoolers: effects of a behavior-based physical activity intervention on caloric expenditure. Southern Medical Journal 2013 August;106(8):456-9. Inappropriate Population
Ref ID: 5743

(354) Anomasiri W, Sanguanrungsirikul S, Saichandee P. Low dose creatine supplementation enhances sprint phase of 400 meters swimming performance. Journal of the Medical Association of Thailand 2004 September;87:Suppl-32. Diet Intervention or Supplement Study
Ref ID: 1557

(355) Anthes E. Treatment: Marginal gains. Nature 2014 April 17;508(7496):S54-S56. Inappropriate Study Design
Ref ID: 5745

(356) Antonella EDP, Luca S, Emilia DF, Rosaria PM, Annarita C, Giuseppe C, Franco C, Giuliana V, Adriana F, Salvatore DM, Armido R. Familial and Environmental-influences on body-composition and body-fat distribution in childhood in southern Italy. International Journal of Obesity 1994;18(9):596-601. Cross-sectional study
Ref ID: 5127

(357) Antonio J, Sanders MS, Ehler LA, Uelmen J, Raether JB, Stout JR. Effects of exercise training and amino-acid supplementation on body composition and physical performance in untrained women. Nutrition 2000 November;16(11-12):1043-6. Diet Intervention or Supplement Study
Ref ID: 1944

(358) Antonio J, Sanders MS, Van GD. The effects of bovine colostrum supplementation on body composition and exercise performance in active men and women. Nutrition 2001 March;17(3):243-7. Diet Intervention or Supplement Study
Ref ID: 1920

(359) Antoniou EE, Derom C, Thiery E, Fowler T, Southwood TR, Zeegers MP. The Influence of Genetic and Environmental Factors on the Etiology of the Human Umbilical Cord: The East Flanders Prospective Twin Survey. Biology of

Reproduction 2011;85(1):137-43. Not an exercise intervention study
Ref ID: 5128

(360) Apetito L, Vasconcelos K, Marim MMF, Detregiachi CRP. Prática de dietas de emagrecimento por escolares adolescents. Practical diets for weight loss teen. Journal of the Health Sciences Institute 2010 December;28(4):329-33. Survey or questionnaire
Ref ID: 3950

(361) Apolzan JW, Bray GA, Hamilton MT, Zderic TW, Han H, Champagne CM, Shepard D, Martin CK. Short-term overeating results in incomplete energy intake compensation regardless of energy density or macronutrient composition. Obesity (Silver Spring) 2014 January;22(1):119-30. Inappropriate Intervention
Ref ID: 5746

(362) Apostol G, Pakalnis A, Laforet GA, Robieson WZ, Olson E, Abi-Saab WM, Saltarelli M. Safety and tolerability of divalproex sodium extended-release in the prophylaxis of migraine headaches: Results of an open-label extension trial in adolescents. Headache 2009;49:36-44. Drug intervention study
Ref ID: 4670

(363) Apostol G, Lewis DW, Laforet GA, Robieson WZ, Fugate JM, Abi-Saab WM, Saltarelli MD. Divalproex sodium extended-release for the prophylaxis of migraine headache in adolescents: Results of a stand-alone, long-term open-label safety study. Headache 2009;49:45-53. Drug intervention study
Ref ID: 4669

(364) Apovian CM, Bergenstal RM, Cuddihy RM, Qu Y, Lenox S, Lewis MS, Glass LC. Effects of exenatide combined with lifestyle modification in patients with type 2 diabetes. American Journal of Medicine 2010 May;123(5):468-17. Study not limited to children and adolescents
Ref ID: 50

(365) Appel-Dingemanse S. Clinical pharmacokinetics of tegaserod, a serotonin 5-ht[sub 4] receptor partial agonist with promotile activity. Clinical Pharmacokinetics 2002 October;41(13):1021. Drug intervention study
Ref ID: 3510

(366) Aragona J, Cassady J, Drabman RS. Treating overweight children through parental training and contingency contracting. Journal of Applied Behavior Analysis 1975;8(3):269-78. Lifestyle Intervention
Ref ID: 2393

(367) Aranha MFM, Alves MC, Bérzin F, Gavião MBD. Efficacy of electroacupuncture for myofascial pain in the upper trapezius muscle: A case series. Brazilian Journal of Physical Therapy 2011 September;15(5):371-9. Study limited to

adults
Ref ID: 3511

(368) Arauz Boudreau AD, Kurowski DS, Gonzalez WI, Dimond MA, Oreskovic NM. Latino families, primary care, and childhood obesity: A randomized controlled trial. American Journal of Preventive Medicine 2013 March;44(3 Suppl 3):S247-S257. Inappropriate Outcomes
Ref ID: 5747

(369) Araújo CL, Dumith SC, Menezes AM, Hallal PC. Peso medido, peso percebido e fatores associados em adolescents. Measured weight, self-perceived weight, and associated factors in adolescents. Revista Panamericana de Salud Pública 2010 May;27(5):360-7. Cohort Study
Ref ID: 3951

(370) Araújo FL, Monteiro LZ, Pinheiro MHNP, Silva CABd. Prevalência de fatores de risco para hipertensão arterial em escolares do município de Fortaleza, CE. Prevalence of hypertension risk factors in students in the city of Fortaleza, Ceará, Brazil. Revista Brasileira de Hipertensao 2010 December;17(4):203-9. Cross-sectional study
Ref ID: 3952

(371) Araújo MFM, Almeida LSd, Silva PCVd, Vasconcelos HCAd, Lopes MVdO, Damasceno MMC. Sobrepeso entre adolescentes de escolas particulares de Fortaleza, CE, Brasil. Overweight among adolescents from private schools in Fortaleza, CE, Brazil. Sobrepeso entre adolescentes de escuelas privadas de Fortaleza, CE, Brasil. Revista brasileira de Enfermagem 2010 August;63(4):623-8. Cross-sectional study
Ref ID: 3953

(372) Araújo VCd, Konrad LM, Rabacow FM, Graup S, Amboni R, Farias Júnior JCd. Prevalência de excesso de peso em adolescentes brasileiros: Um estudo de revisão sistemática. Revista Brasileira de Atividade Física e Saúde 2007;12(3). Review article
Ref ID: 3954

(373) Aránguiz A, García G, Rojas D, Salas B, Martínez R, Mac MK. Estudio descriptivo, comparativo y correlacional del estado nutricional y condición cardiorrespiratoria en estudiantes universitarios de Chile
Descriptive, comparative and correlational study of nutritional and cardio-respiratory condition of Chilean university students. Revista Chilena de Nutrición 2010 March;37(1):70-8. Cross-sectional study
Ref ID: 3955

(374) Arbeit ML, Johnson CC, Mott DS, Harsha DW, Nicklas TA, Webber LS, Berenson GS. The Heart Smart cardiovascular school health promotion: behavior correlates of risk factor change. Preventive Medicine 1992

January;21(1):18-32. Lifestyle Intervention
Ref ID: 2292

(375) Arbesú Ruiz N, Ríos Fernández Adl. La pubertad y la capacidad de trabajo físico. Revista Cubana de Pediatria 1989 June;61(3):382-92. Not an exercise intervention study
Ref ID: 3956

(376) Arboleda Naranjo LH. Beneficios del Ejercicio. Hacia promoc salud 2003 November;(8):77-84. Review article
Ref ID: 3957

(377) Arcan C, Larson N, Bauer K, Berge J, Story M, Neumark-Sztainer D. Dietary and weight-related behaviors and body mass index among Hispanic, Hmong, Somali, and white adolescents. Journal of the Academy of Nutrition and Dietetics 2014 March;114(3):375-83. Inappropriate Study Design
Ref ID: 5748

(378) Arciero PJ, Bougopoulos CL, Nindl BC, Benowitz NL. Influence of age on the thermic response to caffeine in women. Metabolism: Clinical and Experimental 2000 January;49(1):101-7. Study limited to adults
Ref ID: 1993

(379) Arciero PJ, Hannibal NS, III, Nindl BC, Gentile CL, Hamed J, Vukovich MD. Comparison of creatine ingestion and resistance training on energy expenditure and limb blood flow. Metabolism: Clinical and Experimental 2001 December;50(12):1429-34. Study limited to adults
Ref ID: 1872

(380) Arciero PJ, Ormsbee MJ. Relationship of blood pressure, behavioral mood state, and physical activity following caffeine ingestion in younger and older women. Applied Physiology, Nutrition, and Metabolism = Physiologie Appliquee, Nutrition et Metabolisme 2009 August;34(4):754-62. Study limited to adults
Ref ID: 674

(381) Arcos G, Uarac U, Molina V, Repossi F, Ulloa V. Impacto de la violencia doméstica sobre la salud reproductiva y neonatal. Revista médica de Chile 2001 December;129(12):1413-24. Cohort Study
Ref ID: 3958

(382) Ardoy DN, Fernandez-Rodriguez JM, Chillon P, Artero EG, Espana-Romero V, Jimenez-Pavon D, Ruiz JR, Guirado-Escamez C, Castillo MJ, Ortega FB. [Physical fitness enhancement through education, EDUFIT study: background, design, methodology and dropout analysis]. [Spanish]. Revista Espanola de Salud Publica 2010 March;84(2):151-68. Not All Participants were Overweight and/or Obese
Ref ID: 507

(383) Arens U, Barasi M, Belton L, Burley V, Bussell G, Hood S, McLean L, Watling R, Gatenby S. Current literature. Journal of Human Nutrition and Dietetics 16[3], 201-213. 2003. Abstract,
Ref ID: 3512

(384) Arias JL. Free-throw accuracy and success as a function of ball weight in 9- to 11-year-old male players> Precisão e êxito em função do peso da bola em jogadores de 9 a 11 anos de idade. Motriz Revista de Educação Física (Improv) 2012 June;18(2):338-44. Not an exercise intervention study
Ref ID: 3959

(385) Arif MA, Arif K. Low birthweight babies in the Third World: maternal nursing versus professional nursing careJournal of Tropical Pediatrics 1999;45:278-80. Not an exercise intervention study
Ref ID: 886

(386) Arikawa AY, O'Dougherty M, Kaufman BC, Smith AJ, Thomas W, Warren M, Kurzer MS, Schmitz KH. Women in Steady Exercise Research (WISER): study design and methods. Contemporary Clinical Trials 2010 September;31(5):457-65. Study limited to adults
Ref ID: 2395

(387) Arikawa AY, Kurzer MS, Thomas W, Schmitz KH. No effect of exercise on insulin-like growth factor-I, insulin, and glucose in young women participating in a 16-week randomized controlled trial. Cancer Epidemiology, Biomarkers and Prevention 2010 November;19(11):2987-90. Study limited to adults
Ref ID: 2394

(388) Arikawa AY, Thomas W, Schmitz KH, Kurzer MS. Sixteen weeks of exercise reduces C-reactive protein levels in young women. Medicine and Science in Sports and Exercise 2011 June;43(6):1002-9. Study limited to adults
Ref ID: 2396

(389) Ariza AJ, Laslo KM, Thomson JS, Seshadri R, Binns HJ, Pediatric Practice Research Group. Promoting growth interpretation and lifestyle counseling in primary care. Journal of Pediatrics 2009 April;154(4):596-601. Not an exercise intervention study
Ref ID: 781

(390) Armelagos GJ. The Omnivore'S Dilemma the Evolution of the Brain and the Determinants of Food Choice. Journal of Anthropological Research 2010;66(2):161-86. Review article
Ref ID: 5129

(391) Armstrong LE, Whittlesey MJ, Casa DJ, Elliott TA, Kavouras SA, Keith NR, Maresh CM. No effect of 5% hypohydration on running economy of competitive runners at 23 degrees C. Medicine and Science in Sports and Exercise 2006

October;38(10):1762-9. Not a randomized controlled trial (RCT)
Ref ID: 1343

(392) Armstrong N, Welsman JR. Peak oxygen uptake in relation to growth and maturation in 11-to 17-year-old humans. European Journal of Applied Physiology 2001;85(6):546-51. Cohort Study
Ref ID: 5130

(393) Arnberg K, Larnkjaer A, Michaelsen KF, Molgaard C. Central adiposity and protein intake are associated with arterial stiffness in overweight children. Journal of Nutrition 2012 May;142(5):878-85. Cross-sectional study
Ref ID: 2397

(394) Arnberg K, Molgaard C, Michaelsen KF, Jensen SM, Trolle E, Larnkjaer A. Skim milk, whey, and casein increase body weight and whey and casein increase the plasma C-peptide concentration in overweight adolescents. Journal of Nutrition 2012 December;142(12):2083-90. Inappropriate Intervention
Ref ID: 5749

(395) Arnett MG, Lutz B. Effects of rope-jump training on the os calcis stiffness index of postpubescent girls. Medicine and Science in Sports and Exercise 2002 December;34(12):1913-9. Not All Participants were Overweight and/or Obese
Ref ID: 1790

(396) Arnold LE, Amato A, Bozzolo H, Hollway J, Cook A, Ramadan Y, Crowl L, Zhang D, Thompson S, Testa G, Kliewer V, Wigal T, McBurnett K, Manos M. Acetyl-L-carnitine (ALC) in attention-deficit/hyperactivity disorder: a multi-site, placebo-controlled pilot trial. Journal of Child and Adolescent Psychopharmacology 2007;17:791-802. Diet Intervention or Supplement Study
Ref ID: 4671

(397) Arocha R. Lipoproteinas de alta densidad (HDL-C) y sus relaciones con el proceso aterosclerÃ³tico. (Spanish). Informe Medico 2002 March;4(3):151. Review article
Ref ID: 3513

(398) Arora M, Nazar GP, Gupta VK, Perry CL, Reddy KS, Stigler MH. Association of breakfast intake with obesity, dietary and physical activity behavior among urban school-aged adolescents in Delhi, India: results of a cross-sectional study. BMC Public Health 2012;12:881. Inappropriate Study Design
Ref ID: 5750

(399) Arrebola E, Gomez-Candela C, Fernandez-Fernandez C, Loria V, Munoz-Perez E, Bermejo LM. Evaluation of a lifestyle modification program for treatment of overweight and nonmorbid obesity in primary healthcare and its influence on health-related quality of life. Nutrition in Clinical Practice 2011 June;26(3):316-

21. Study limited to adults
Ref ID: 2398

(400) Arroll B, Beaglehole R. Does physical activity lower blood pressure: a critical review of the clinical trials. [Review] [33 refs]. Journal of Clinical Epidemiology 1992 May;45(5):439-47. Review article
Ref ID: 2289

(401) Arroyave LF, Ramirez AC, Velásquez C, Manrique Hernández RD. Factores de riesgo asociados a estrías atróficas en mujeres adolescentes de un colegio privado, Medellín, 2003 Risk factors associated with strechmarks in teenage girls of a private school, Medellin, 2003. CES Medicine 2009 June;23(1,supl):81-6. Case-Control / Case Study
Ref ID: 3960

(402) Arruda ELMd, Lopes AdS. Gordura corporal, nível de atividade física e hábitos alimentares de adolescentes da região serrana da Santa Catarina, Brasil. Revista Brasileira de Cineantropometria and Desempenho Humano 2007 March;9(1):5-11. Cross-sectional study
Ref ID: 625

(403) Arruda MF. Análise postural computadorizada de alterações musculoesqueléticas decorrentes do sobrepeso em escolares: [revisão]. Evaluation posture computerized in disturbance on musculoskeletal resulting from by overweight schoolchildren: [revision]. Motriz Revista de Educação Física (Improv) 2009 March;15(1):143-50. Cross-sectional study
Ref ID: 3961

(404) Arsenault JE, Havel PJ, López-de RD, Penny ME, Van L, Brown KH. Longitudinal measures of circulating leptin and ghrelin concentrations are associated with the growth of young Peruvian children but are not affected by zinc supplementation. American Journal of Clinical Nutrition 2007;86:1111-9. Diet Intervention or Supplement Study
Ref ID: 4672

(405) Artero EG, Espana-Romero V, Ortega FB, Jimenez-Pavon D, Ruiz JR, Vicente-Rodriguez G, Bueno M, Marcos A, Gomez-Martinez S, Urzanqui A, Gonzalez-Gross M, Moreno LA, Gutierrez A, Castillo MJ. Health-related fitness in adolescents: underweight, and not only overweight, as an influencing factor. The AVENA study. Scandinavian Journal of Medicine and Science in Sports 2010 June;20(3):418-27. Cross-sectional study
Ref ID: 498

(406) Artero EG, Ruiz JR, Ortega FB, Espana-Romero V, Vicente-Rodriguez G, Molnar D, Gottrand F, Gonzalez-Gross M, Breidenassel C, Moreno LA, Gutierrez A, HELENA Study Group. Muscular and cardiorespiratory fitness are independently associated with metabolic risk in adolescents: the HELENA

study. Pediatric Diabetes 2011 December;12(8):704-12. Cross-sectional study
Ref ID: 2399

(407) Artioli GG, Iglesias RT, Franchini E, Gualano B, Kashiwagura DB, Solis MY, Benatti FB, Fuchs M, Lancha Junior AH. Rapid weight loss followed by recovery time does not affect judo-related performance. Journal of Sports Sciences 2010 January;28(1):21-32. Not a randomized controlled trial (RCT)
Ref ID: 553

(408) Artz E, Freemark M. The pathogenesis of insulin resistance in children: Metabolic complications and the roles of diet, exercise and pharmacotherapy in the prevention of type 2 diabetes. Pediatric Endocrinology Reviews 2004;1(3):296-309. Review article
Ref ID: 3189

(409) Aschemeier B, Lange K, Kordonouri O, Danne T. Paediatric obesity and type 2 diabetes: Strategies for prevention and treatment. Pract Diabetes Int 2008;25(9):368-75. Review article
Ref ID: 3190

(410) Ascher B, Goldberg DJ, Polla L, MÃ´le B, Kinney B, Gasperoni C, Salgarello M, Granado PC, Goldberg DJ, Gasparotti M, Sanchez M, Dierickx CC, Bitter P, Kunzi-Rapp K, Wortmann S, Gottlober P, Diebolder R, Rupp M. IMCAS - International Master Course on Ageing Skin. Paris, January 2001. Journal of Cutaneous Laser Therapy 2001 March;3(1):13-49. Abstract
Ref ID: 3514

(411) Asher MI, Douglas C, Airy M, Andrews D, Trenholme A. Effects of chest physical therapy on lung function in children recovering from acute severe asthma. Pediatric Pulmonology 1990;9:146-51. Rehabilitation study, Study less than 4 weeks
Ref ID: 1075

(412) Ashizawa K, Rahmawati NT, Hastuti J. Body size and shape, and its secular change in Javanese-Indonesian adults. Anthropological Science 2009;117(3):165-70. Cross-sectional study
Ref ID: 5131

(413) Ashton CH. Biomedical benefits of cannabinoids? Addiction Biology 1999 April;4(2):111-26. Review article
Ref ID: 3515

(414) Ashwal S, Wycliffe ND, Holshouser BA. Advanced Neuroimaging in Children with Nonaccidental Trauma. Developmental Neuroscience 2010;32(5-6):343-60. Review article
Ref ID: 5132

(415) Ashwood P, Kwong C, Hansen R, Hertz-Picciotto I, Croen L, Krakowiak P, Walker W, Pessah IN, Water J. Brief Report: Plasma Leptin Levels are Elevated in Autism: Association with Early Onset Phenotype? Journal of Autism and Developmental Disorders 2008 January;38(1):169-75. Not an exercise intervention study
Ref ID: 3801

(416) Ashworth A, Khanum S. Cost-effective treatment for severely malnourished children: what is the best approach? Health policy and planning 1997;12:115-21. Diet Intervention Study
Ref ID: 4673

(417) Ashworth A, Shrimpton R, Jamil K. Growth monitoring and promotion: review of evidence of impact. Maternal and Child Nutrition 2008 January 2;4:86-117. Review article
Ref ID: 3516

(418) Aspée A, Sepúlveda C, Amarales O, Acuña L, Olguín C, Rider L, Gross M, Vila C, Enriquez G. Importancia del manejo del ductus arterioso persistente en la XII Región al implementar el diagnóstico ecográfico y tratamiento quirúrgico a nivel local. Revista Chilena de Cardiología 2009 December;28(4):369-74. Retrospective study
Ref ID: 3962

(419) Aspres N, Benítez A, Galindo A, Larguía M. Amamantamiento en recien nacidos prematuros de muy bajo peso al nacer (PMBPN, PN ó1500 grs.) : análisis de una experiencia en una institución pública. Revista del Hospital Materno Infantil Ramón Sardá 1994;13(3):115-22. Subjects less than 2 years old
Ref ID: 3963

(420) Asserhoj M, Nehammer S, Matthiessen J, Michaelsen KF, Lauritzen L. Maternal fish oil supplementation during lactation may adversely affect long-term blood pressure, energy intake, and physical activity of 7-year-old boys. Journal of Nutrition 2009 February;139(2):298-304. Study limited to adults
Ref ID: 142

(421) Assis CMd, Quio VR, Rasseli JG, Cunha FGCd, Salaroli LB. Hábitos alimentares e estado nutricional de jovens: Um estudo comparativoipt. Eating habits and nutritional status of the youth: A comparative study. Nutrire Revista da Sociedade Brasileira de Alimentação e Nutrição 2009 April;34(1):13-27. Diet Intervention Study
Ref ID: 3964

(422) Astrom E, Jorulf H, Soderhall S. Intravenous pamidronate treatment of infants with severe osteogenesis imperfecta. Archives of Disease in Childhood 2007

April;92(4):332-8. Drug intervention study
Ref ID: 1258

(423) Atabek ME, Pirgon O. Use of metformin in obese adolescents with hyperinsulinemia: a 6-month, randomized, double-blind, placebo-controlled clinical trial. J Pediatr Endocrinol Metab 2008 April;21(4):339-48. No exercise only group, Drug intervention study
Ref ID: 178

(424) Atalah Samur E, Urteaga R, Rebolledo Acevedo A, Delfín C, Ramos H. Patrones alimentarios y de actividad física en escolares de la Región de Aysén. Revista Chilena de Pediatría 1999 December;70(6):483-90. Survey or questionnaire
Ref ID: 3965

(425) Athyros VG, Bouloukos VI, Pehlivanidis AN, Papageorgiou AA, Dionysopoulou SG, Symeonidis AN, Petridis DI, Kapousouzi MI, Satsoglou EA, Mikhailidis DP, MetS-Greece Collaborative Group. The prevalence of the metabolic syndrome in Greece: the MetS-Greece Multicentre Study. Diabetes, Obesity and Metabolism 2005 July;7(4):397-405. Cross-sectional study
Ref ID: 1506

(426) Atkin LM, Davies PSW. Diet composition and body composition in preschool children. American Journal of Clinical Nutrition 2000;72(1):15-21. Cross-sectional study
Ref ID: 5133

(427) Atkinson RL, Lee I, Shin HJ, He J. Human adenovirus-36 antibody status is associated with obesity in children. International Journal of Pediatric Obesity 2010;5(2):157-60. Cross-sectional study
Ref ID: 5134

(428) Atlantis E, Barnes EH, Singh MA. Efficacy of exercise for treating overweight in children and adolescents: a systematic review. [Review] [103 refs]. International Journal of Obesity 2006 July;30(7):1027-40. Review article
Ref ID: 1382

(429) Atlantis E, Salmon J, Bauman A. Acute effects of advertisements on children's choices, preferences, and ratings of liking for physical activities and sedentary behaviours: a randomised controlled pilot study. Journal of Science and Medicine in Sport 2008 November;11(6):553-7. Acute study, Primary outcome(s) not assessed
Ref ID: 213

(430) Atsumi T, Iwakura I, Kashiwagi Y, Fujisawa S, Ueha T. Free Radical Scavenging Activity in the Nonenzymatic Fraction of Human Saliva: A Simple DPPH Assay Showing the Effect of Physical Exercise. Antioxidants and Redox

Signaling 1999;1(4):537-46. Not an exercise intervention study
Ref ID: 5135

(431) Attux C, Martini LC, Araujo CM, Roma AM, Reis AF, Bressan RA. The effectiveness of a non-pharmacological intervention for weight gain management in severe mental disorders: results from a national multicentric study. Revista Brasileira de Psiquiatria 2011 June;33(2):117-21. Lifestyle Intervention
Ref ID: 2400

(432) Aucouturier J, Isacco L, Thivel D, Fellmann N, Chardigny JM, Duclos M, Duche P. Effect of time interval between food intake and exercise on substrate oxidation during exercise in obese and lean children. Clinical Nutrition 2011 December;30(6):780-5. Not a randomized controlled trial (RCT)
Ref ID: 2401

(433) August GP, Caprio S, Fennoy I, Freemark M, Kaufman FR, Lustig RH, Silverstein JH, Speiser PW, Styne DM, Montori VM. Prevention and treatment of pediatric obesity: An Endocrine Society clinical practice guideline based on expert opinion. Journal of Clinical Endocrinology and Metabolism 2008;93(12):4576-99. Review article
Ref ID: 3191

(434) Austin GP, Garrett GE, Tiberio D. Effect of added mass on human unipedal hopping. Perceptual and motor skills 2002;94:834-40. Study limited to adults
Ref ID: 4676

(435) Austin SB, Field AE, Wiecha J, Peterson KE, Gortmaker SL. The impact of a school-based obesity prevention trial on disordered weight-control behaviors in early adolescent girls. Arch Pediatr Adolesc Med 2005 March;159(3):225-30. No exercise only group
Ref ID: 313

(436) Austin SB, Kim J, Wiecha J, Troped PJ, Feldman HA, Peterson KE. School-based overweight preventive intervention lowers incidence of disordered weight-control behaviors in early adolescent girls. Arch Pediatr Adolesc Med 2007 September;161(9):865-9. No exercise only group
Ref ID: 219

(437) AuYeung W, Canales RA, Leckie JO. The fraction of total hand surface area involved in young children's outdoor hand-to-object contacts. Environmental Research 2008;108(3):294-9. Not an exercise intervention study
Ref ID: 5136

(438) Avdic D, Jusupovic F, Kudumovic M. Anthropometric values for boys aged 14-15 years who actively train basketball in comparing to boys of same age who do not train any sports. Healthmed 2008;2(4):253-64. Not an exercise

intervention study
Ref ID: 5137

(439) Ayah R, Joshi MD, Wanjiru R, Njau EK, Otieno CF, Njeru EK, Mutai KK. A population-based survey of prevalence of diabetes and correlates in an urban slum community in Nairobi, Kenya. BMC Public Health 2013;13:371. Inappropriate Study Design
Ref ID: 5751

(440) Ayala GX, Elder JP, Campbell NR, Arredondo E, Baquero B, Crespo NC, Slymen DJ. Longitudinal intervention effects on parenting of the Aventuras para Ninos study. American Journal of Preventive Medicine 2010 February;38(2):154-62. No exercise only group, Not an exercise intervention study
Ref ID: 63

(441) Ayoama R, Hiruma E, Sasaki H. Effects of creatine loading on muscular strength and endurance of female softball players. Journal of Sports Medicine and Physical Fitness 2003 December;43(4):481-7. Diet Intervention or Supplement Study
Ref ID: 1679

(442) Azadbakht L, Mirmiran P, Hedayati M, Esmaillzadeh A, Shiva N, Azizi F. Particle size of LDL is affected by the National Cholesterol Education Program (NCEP) step II diet in dyslipidaemic adolescents. BR J NUTR 2007 July;98(1):134-9. Case-Control / Case Study, Diet Intervention Study
Ref ID: 1213

(443) Azevedo BAR, Ribeiro SML. Avaliação do estado nutricional e do balanço energético de um grupo de atletas de ginástica artística. Motriz Revista de Educação Física (Improv) 2007;13(3):165-73. Cross-sectional study
Ref ID: 3967

(444) Azizi F, Bahrainian M, Khamseh ME, Khoshniat M. Intellectual development and thyroid function in children who were breast-fed by thyrotoxic mothers taking methimazole. Journal of Pediatric Endocrinology and Metabolism 2003;16(9):1239-43. Drug intervention study
Ref ID: 5138

(445) Aznar S, Naylor PJ, Silva P, Perez M, Angulo T, Laguna M, Lara MT, Lopez-Chicharro J. Patterns of physical activity in Spanish children: a descriptive pilot study. Child: Care, Health and Development 2011 May;37(3):322-8. Cross-sectional study
Ref ID: 2402

(446) Celik Gl, Tahiroglu A, Avcı A. (Turkish). Metabolic and endocrine side effects of atypical antipsychotic drugs in children and adolescents. Current Approaches in Psychiatry / Psikiyatride Guncel Yaklasimlar 2011 June;3(2):232-50. Review

article
Ref ID: 3517

(447) Babamoto KS, Sey KA, Camilleri AJ, Karlan VJ, Catalasan J, Morisky DE. Improving diabetes care and health measures among hispanics using community health workers: results from a randomized controlled trial. Health Education and Behavior 2009 February;36(1):113-26. Study not limited to children and adolescents
Ref ID: 137

(448) Baby S, Nguyen M, Tran D, Raffa RB. Substance P antagonists: the next breakthrough in treating depression?: REVIEW ARTICLE. Journal of Clinical Pharmacy and Therapeutics 1999 December;24(6):461. Review article
Ref ID: 526

(449) Bacha JM, Appugliese D, Coleman S, Kaciroti N, Bradley RH, Corwyn RF, Lumeng JC. Maternal perception of neighborhood safety as a predictor of child weight status: The moderating effect of gender and assessment of potential mediators. International Journal of Pediatric Obesity 2010;5(1):72-9. Survey or questionnaire
Ref ID: 556

(450) Bacharach DW, von Duvillard SP, Rundell KW, Meng J, Cring MR, Szmedra L, Castle JM. Carbohydrate drinks and cycling performance. Journal of Sports Medicine and Physical Fitness 1994 June;34(2):161-8. Not a randomized controlled trial (RCT)
Ref ID: 2233

(451) Baciuk EP, Pereira RI, Cecatti JG, Braga AF, Cavalcante SR. Water aerobics in pregnancy: cardiovascular response, labor and neonatal outcomes. Reproductive Health 2008;5:10. Study limited to adults
Ref ID: 4678

(452) Badesch DB, Raskob GE, Elliott CG, Krichman AM, Farber HW, Frost AE, Barst RJ, Benza RL, Liou TG, Turner M, Giles S, Feldkircher K, Miller DP, McGoon MD. Pulmonary arterial hypertension: baseline characteristics from the REVEAL Registry. Chest 2010 February;137(2):376-87. Study limited to adults
Ref ID: 589

(453) Badland HM, Schofield GM, Witten K, Schluter PJ, Mavoa S, Kearns RA, Hinckson EA, Oliver M, Kaiwai H, Jensen VG, Ergler C, McGrath L, McPhee J. Understanding the Relationship between Activity and Neighbourhoods (URBAN) Study: research design and methodology. BMC Public Health 2009;9:224. Description of study from review or magazine or etc. (not the actual study)
Ref ID: 705

(454) Baek HS, Kim YD, Shin JH, Kim JH, Oh JW, Lee HB. Serum leptin and adiponectin levels correlate with exercise-induced bronchoconstriction in children with asthma. Annals of Allergy Asthma and Immunology 2011;107(1):14-21. Cross-sectional study
Ref ID: 5139

(455) Baena Diez JM, Atance Yague RM, Escriba Jordana JM, Conesa GA, Rivera CD, Flores Cebria RM. [Perception of medical advice: in all cases and to all patients equally?]. [Spanish]. Gaceta Sanitaria 1999 January;13(1):46-52. Cross-sectional study
Ref ID: 2031

(456) Baggett CD, Stevens J, Catellier DJ, Evenson KR, McMurray RG, He K, Treuth MS. Compensation or displacement of physical activity in middle-school girls: the Trial of Activity for Adolescent Girls. International Journal of Obesity 2010 July;34(7):1193-9. Study less than 4 weeks
Ref ID: 494

(457) Bahl R, Bhandari N, Taneja S, Bhan MK. The impact of vitamin A supplementation on physical growth of children is dependent on season. European Journal of Clinical Nutrition 1997;51:26-9. Diet Intervention or Supplement Study
Ref ID: 4679

(458) Bailey N. Current choices in omega 3 supplementation. Nutrition Bulletin 2009 March;34(1):85-91. Review article
Ref ID: 3518

(459) Baillie-Hamilton PF. Chemical toxins: A hypothesis to explain the global obesity epidemic. Journal of Alternative and Complementary Medicine 2002;8(2):185-92. Review article
Ref ID: 5140

(460) Baker KG, Robertson VJ, Duck FA. A Review of Therapeutic Ultrasound: Biophysical Effects. Physical Therapy 2001 July;81(7):1351. Review article
Ref ID: 3519

(461) Balagopal P, Bayne E, Sager B, Russell L, Patton N, George D. Effect of lifestyle changes on whole-body protein turnover in obese adolescents. International Journal of Obesity and Related Metabolic Disorders 2003 October;27(10):1250-7. No exercise only group
Ref ID: 354

(462) Balagopal P, George D, Patton N, Yarandi H, Roberts WL, Bayne E, Gidding S. Lifestyle-only intervention attenuates the inflammatory state associated with obesity: a randomized controlled study in adolescents. Journal of Pediatrics 2005 March;146(3):342-8. No exercise only group
Ref ID: 312

(463) Balagopal P, George D, Yarandi H, Funanage V, Bayne E. Reversal of obesity-related hypoadiponectinemia by lifestyle intervention: a controlled, randomized study in obese adolescents. Journal of Clinical Endocrinology and Metabolism 2005 November;90(11):6192-7. No exercise only group
Ref ID: 302

(464) Balagopal P, Graham TE, Kahn BB, Altomare A, Funanage V, George D. Reduction of elevated serum retinol binding protein in obese children by lifestyle intervention: association with subclinical inflammation. Journal of Clinical Endocrinology and Metabolism 2007 May;92(5):1971-4. No exercise only group, No comparative control group
Ref ID: 246

(465) Balagopal P, George D, Sweeten S, Mann KJ, Yarandi H, Mauras N, Vaughan DE. Response of fractional synthesis rate (FSR) of fibrinogen, concentration of D-dimer and fibrinolytic balance to physical activity-based intervention in obese children. J Thromb Haemost 2008 August;6(8):1296-303. No comparative control group
Ref ID: 180

(466) Balagopal PB, Gidding SS, Buckloh LM, Yarandi HN, Sylvester JE, George DE, Funanage VL. Changes in circulating satiety hormones in obese children: a randomized controlled physical activity-based intervention study. Obesity (Silver Spring) 2010 September;18(9):1747-53. No exercise only group, Lifestyle Intervention
Ref ID: 69

(467) Balaguer-Fernandez C, Femenia-Font A, Muedra V, Merino V, Lopez-Castellanoa A. Combined strategies for enhancing the transdermal absorption of midazolam through human skin. Journal of Pharmacy and Pharmacology 2010;62(9):1096-102. Drug intervention study
Ref ID: 5141

(468) Balas-Nakash M, Benítez-Arciniega A, Perichart-Perera O, Valdés-Ramos R, Vadillo-Ortega F. The effect of exercise on cardiovascular risk markers in Mexican school-aged children: Comparison between two structured group routines. Efecto del ejercicio sobre marcadores de riesgo cardiovascular en escolares mexicanos: Comparación entre dos rutinas grupales. Salud Pública de México 2010 October;52(5):398-405. Not All Participants were Overweight and/or Obese
Ref ID: 3968

(469) Balbinotti MAA, Capozzoli CJ. Motivação à prática regular de atividade física: Um estudo exploratório com praticantes em academias de ginástica. Revista Brasileira de Educação Física e Esporte 2008 March;22(1):63-80. Study limited to adults
Ref ID: 3969

(470) Balbinotti MAA, Zambonato F, Barbosa MLL, Saldanha RP, Balbinotti CAA. Motivação à prática regular de atividades físicas e esportivas: Um estudo comparativo entre estudantes com sobrepeso, obesos e eutróficos. Motivation of regular physical and sporting activities practice: A comparative study between obeses, overweight, and other students. Motriz Revista de Educação Física (Improv) 2011 September;17(3):384-94. Not an exercise intervention study
Ref ID: 3970

(471) Balcáza M, Pasquet P, Garine Id. Dieta, actividad física y estado de nutrición en escolares tarahumaras, México. Revista Chilena de Salud Pública 2009;13(1):30-7. Observational study
Ref ID: 3971

(472) Baldari C, Di LL, Emerenziani GP, Gallotta MC, Sgro P, Guidetti L. Is explosive performance influenced by androgen concentrations in young male soccer players? British Journal of Sports Medicine 2009 March;43(3):191-4. Observational study
Ref ID: 789

(473) Baldwin CM, Lyseng-Williamson KA, Keam SJ. Meropenem: A Review of its Use in the Treatment of Serious Bacterial Infections. Drugs 2008 March 15;68(6):803. Review article
Ref ID: 3520

(474) Balen AH, Anderson RA. Impact of obesity on female reproductive health: British fertility society, policy and practice guidelines. Hum Fertil 2007;10(4):195-206. Review article
Ref ID: 3192

(475) Balfour JAB, Scott LJ. Cinacalcet Hydrochloride. Drugs 2005 January 15;65(2):271-81. Drug intervention study
Ref ID: 3521

(476) Ball EJ, O'Connor J, Abbott R, Steinbeck KS, Davies PSW, Wishart C, Gaskin KJ, Baur LA. Total energy expenditure, body fatness, and physical activity in children aged 6-9 y. American Journal of Clinical Nutrition 2001;74(4):524-8. Cross-sectional study
Ref ID: 5142

(477) Ball GD, Ambler KA, Keaschuk RA, Rosychuk RJ, Holt NL, Spence JC, Jetha MM, Sharma AM, Newton AS. Parents as agents of change (PAC) in pediatric weight management: the protocol for the PAC randomized clinical trial. BMC Pediatr 2012;12:114. Inappropriate Study Design
Ref ID: 5752

(478) Ballard TL, Clapper JA, Specker BL, Binkley TL, Vukovich MD. Effect of protein supplementation during a 6-mo strength and conditioning program on insulin-

like growth factor I and markers of bone turnover in young adults.[Erratum appears in Am J Clin Nutr. 2006 Mar;83(3):723]. American Journal of Clinical Nutrition 81(6):1442-8, 2005 Jun 2005;(6):1442-8. Study limited to adults
Ref ID: 2882

(479) Ballard TL, Specker BL, Binkley TL, Vukovich MD. Effect of protein supplementation during a 6-month strength and conditioning program on areal and volumetric bone parameters. Bone 2006 June;38(6):898-904. Study limited to adults
Ref ID: 1385

(480) Ballard TP, Melby CL, Camus H, Cianciulli M, Pitts J, Schmidt S, Hickey MS. Effect of resistance exercise, with or without carbohydrate supplementation, on plasma ghrelin concentrations and postexercise hunger and food intake. Metabolism: Clinical and Experimental 2009 August;58(8):1191-9. Not a randomized controlled trial (RCT)
Ref ID: 717

(481) Ballew C, Khan LK, Kaufmann R, Mokdad A, Miller DT, Gunter EW. Blood lead concentration and children's anthropometric dimensions in the Third National Health and Nutrition Examination Survey (NHANES III), 1988-1994. Journal of Pediatrics 1999;134(5):623-30. Survey or questionnaire
Ref ID: 5143

(482) Ballin AC, Koerner HN, Ballin CH, Pereira R, Alcântara LJL, Taques GR, Mocellin M. Assimetria de tonsilas palatinas: experiência de 10 anos do serviço de otorrinolaringologia do Hospital de Clínicas da Universidade Federal do Paraná. Palatine tonsils asymmetry: 10 years experience of the otorhinolaryngology service of the Clinical Hospital of the Federal University of Paraná. Arq int otorrinolaringol (Impr) 2011 March;15(1):67-71. Retrospective study
Ref ID: 3972

(483) Bammann K, Peplies J, Pigeot I, Ahrens W. [IDEFICS: a multicenter European project on diet- and lifestyle-related disorders in children]. [German]. Medizinische Klinik 2007 March 15;102(3):230-5. Description of study from review or magazine or etc. (not the actual study)
Ref ID: 1263

(484) Bangirana P, Giordani B, John CC, Page C, Opoka RO, Boivin MJ. Immediate neuropsychological and behavioral benefits of computerized cognitive rehabilitation in Ugandan pediatric cerebral malaria survivors. Journal of Developmental and Behavioral Pediatrics 2009;30:310-8. Rehabilitation study
Ref ID: 4680

(485) Bangirana P, Allebeck P, Boivin MJ, John CC, Page C, Ehnvall A, Musisi S. Cognition, behaviour and academic skills after cognitive rehabilitation in

Ugandan children surviving severe malaria: a randomised trial. BMC neurology 2011;11:96. Rehabilitation study
Ref ID: 4681

(486) Banks J, Williams J, Cumberlidge T, Cimonetti T, Sharp DJ, Shield JP. Is healthy eating for obese children necessarily more costly for families? British Journal of General Practice 2012 January;62(594):e1-e5. Diet Intervention Study
Ref ID: 2405

(487) Banks J, Sharp DJ, Hunt LP, Shield JP. Evaluating the transferability of a hospital-based childhood obesity clinic to primary care: a randomised controlled trial. British Journal of General Practice 2012 January;62(594):e6-12. Lifestyle Intervention
Ref ID: 2404

(488) Baptista MN, Vargas JF, Baptista ASD. Depressão e qualidade de vida em uma amostra brasileira de obesos mórbidos. Depression and quality of life in a morbid obese brazilian sample. Avaliação Psicológica 2008 August;7(2):235-47. Survey or questionnaire
Ref ID: 3973

(489) Bar-Or O, Blimkie CJ, Hay JA, MacDougall JD, Ward DS, Wilson WM. Voluntary dehydration and heat intolerance in cystic fibrosis. Lancet 1992 March 21;339(8795):696-9. Study less than 4 weeks
Ref ID: 2291

(490) Baranowski T, Simons-Morton B, Hooks P, Henske J, Tiernan K, Dunn JK, Burkhalter H, Harper J, Palmer J. A center-based program for exercise change among black-American families. Health Education Quarterly 1990;17(2):179-96. Not All Participants were Overweight and/or Obese
Ref ID: 2312

(491) Baranowski T, Baranowski JC, Cullen KW, Thompson DI, Nicklas T, Zakeri IE, Rochon J. The Fun, Food, and Fitness Project (FFFP): the Baylor GEMS pilot study. Ethnicity and Disease 2003;13(1 Suppl 1):S30-S39. No exercise only group
Ref ID: 368

(492) Baranowski T, Baranowski J, Thompson D, Buday R, Jago R, Griffith MJ, Islam N, Nguyen N, Watson KB. Video game play, child diet, and physical activity behavior change a randomized clinical trial. American Journal of Preventive Medicine 2011 January;40(1):33-8. Not All Participants were Overweight and/or Obese
Ref ID: 1101

(493) Baranowski T, Abdelsamad D, Baranowski J, O'Connor TM, Thompson D, Barnett A, Cerin E, Chen TA. Impact of an active video game on healthy

children's physical activity. Pediatrics 2012 March;129(3):e636-e642. Not All Participants were Overweight and/or Obese
Ref ID: 2407

(494) Baranowski T, Chen TA, Mendoza JA, O'Connor T, Baranowski J, Jago R. Prospective BMI category change associated with cardiovascular fitness change. Med Sci Sports Exerc 2013 February;45(2):294-8. Inappropriate Study Design
Ref ID: 5753

(495) Barba G, Troiano E, Russo P, Venezia A, Siani A. Inverse association between body mass and frequency of milk consumption in children. BR J NUTR 2005;93(1):15-9. Survey or questionnaire
Ref ID: 5144

(496) Barbeau P, Gutin B, Litaker M, Owens S, Riggs S, Okuyama T. Correlates of individual differences in body-composition changes resulting from physical training in obese children. Am J Clin Nutr 1999 April;69(4):705-11. No comparative control group
Ref ID: 412

(497) Barbeau P, Litaker MS, Woods KF, Lemmon CR, Humphries MC, Owens S, Gutin B. Hemostatic and inflammatory markers in obese youths: effects of exercise and adiposity. Journal of Pediatrics 2002 September;141(3):415-20. No exercise only group
Ref ID: 372

(498) Barbeau P, Gutin B, Litaker MS, Ramsey LT, Cannady WE, Allison J, Lemmon CR, Owens S. Influence of physical training on plasma leptin in obese youths. Can J Appl Physiol 2003 June;28(3):382-96. No exercise only group
Ref ID: 357

(499) Barbeau P, Johnson MH, Howe CA, Allison J, Davis CL, Gutin B, Lemmon CR. Ten months of exercise improves general and visceral adiposity, bone, and fitness in black girls. Obesity 2007 August;15(8):2077-85. Not All Participants were Overweight and/or Obese
Ref ID: 1178

(500) Barbieri RL, Gargiulo AR. Metformin for the treatment of the polycystic ovary syndrome. [Review] [118 refs]. Minerva Ginecologica 2004 February;56(1):63-79. Review article
Ref ID: 1665

(501) Barbosa Filho VC, Reges LAG, Souza EAd, Ribeiro EAG, Lima AB. Práticas esportivas e recreativas em adolescentes com excesso de peso: Análise da composição corporal e do desempenho motor. Sports and recreational activities in overweight adolescents: Analyze of body composition and motor performance. Motriz Revista de Educação Física (Improv) 2011

June;17(2):264-73. Not a randomized controlled trial (RCT)
Ref ID: 3974

(502) Barbosa FP, Oliveira HB, Fernandes PR, Fernandes Filho J. Comparação de equaçoes de estimativa do consumo máiximo de oxigênio em indivíduos jovens. Acta Cirurgica Brasileira 2005;20(supl.1):82-7. Not a randomized controlled trial (RCT)
Ref ID: 3975

(503) Barbosa VLP, Cézar C, Vítolo MR, Lopez FA. Atuação ambulatorial do profissional de educação física no atendimento a crianças e adolescentes obesos. Outpatient performance of the physical education professional caring for the obese children and adolescents. Revista Brasileira de Medicina do Esporte 1999 February;5(1):31-4. Not an exercise intervention study
Ref ID: 695

(504) Bardsley-Elliot A, Plosker GL. Nelfinavir: An Update on its Use in HIV Infection. Drugs 2000 March;59(3):581-620. Review article
Ref ID: 3522

(505) Bardwell G, Mujuru P, Fitch C, Seidel G, Hu W, Sogodogo K, Chester A. Engaging Youth to Examine Lifestyle Behaviors through Authentic Research with University Partnerships. International Electronic Journal of Health Education 2007 January 1;10:95-103. Cross-sectional study
Ref ID: 3898

(506) Baria F, Kamimura MA, Aoike DT, Ammirati A, Rocha ML, de Mello MT, Cuppari L. Randomized controlled trial to evaluate the impact of aerobic exercise on visceral fat in overweight chronic kidney disease patients. Nephrol Dial Transplant 2014 April;29(4):857-64. Inappropriate Population
Ref ID: 5754

(507) Barkin SL, Gesell SB, Poe EK, Ip EH. Changing overweight Latino preadolescent body mass index: the effect of the parent-child dyad. Clinical Pediatrics 2011 January;50(1):29-36. Not All Participants were Overweight and/or Obese
Ref ID: 2408

(508) Barkin SL, Gesell SB, Po'e EK, Escarfuller J, Tempesti T. Culturally tailored, family-centered, behavioral obesity intervention for Latino-American preschool-aged children. Pediatrics 2012 September;130(3):445-56. Inappropriate Intervention
Ref ID: 5755

(509) Barlow SE, Dietz WH. Obesity evaluation and treatment: Expert committee recommendations. Pediatrics 1998;102(3). Review article
Ref ID: 5145

(510) Barnes MJ, Mundel T, Stannard SR. The effects of acute alcohol consumption and eccentric muscle damage on neuromuscular function. Applied Physiology, Nutrition, and Metabolism = Physiologie Appliquee, Nutrition et Metabolisme 2012 February;37(1):63-71. Not an exercise intervention study
Ref ID: 2410

(511) Barnett TA, O'Loughlin J, Gauvin L, Paradis G, Hanley J. Opportunities for student physical activity in elementary schools: A cross-sectional survey of frequency and correlates. Health Education and Behavior 2006;33(2):215-32. Survey or questionnaire
Ref ID: 5146

(512) Barnow S, Bernheim D, Schroder C, Lauffer H, Fusch C, Freyberger HJ. [Obesity in childhood and adolescence--first results of a multimodal intervention study in Mecklenburg-Vorpommern]. [German]. Psychotherapie, Psychosomatik, Medizinische Psychologie 2003 January;53(1):7-14. Not All Participants were Overweight and/or Obese, Lifestyle Intervention
Ref ID: 1779

(513) Barondess JA. Health through the urban lens. Journal of Urban Health-Bulletin of the New York Academy of Medicine 2008;85(5):787-801. Not an exercise intervention study
Ref ID: 5147

(514) Barr-Anderson DJ, Neumark-Sztainer D, Schmitz KH, Ward DS, Conway TL, Pratt C, Baggett CD, Lytle L, Pate RR. But I like PE: factors associated with enjoyment of physical education class in middle school girls. Research Quarterly for Exercise and Sport 2008 March;79(1):18-27. Cross-sectional study
Ref ID: 966

(515) Barr-Anderson DJ, Adams-Wynn AW, DiSantis KI, Kumanyika S. Family-focused physical activity, diet and obesity interventions in African-American girls: a systematic review. Obesity Reviews 2013 January;14(1):29-51. Inappropriate Study Design
Ref ID: 5756

(516) Barrack MT, Van Loan MD, Rauh MJ, Nichols JF. Physiologic and behavioral indicators of energy deficiency in female adolescent runners with elevated bone turnover. American Journal of Clinical Nutrition 2010 September;92(3):652-9. Cross-sectional study
Ref ID: 471

(517) Barreto SM, Passos VMA, Firmo JOA, Guerra HL, Vidigal PG, Lima-Costa MFF. Hypertension and clustering of cardiovascular risk factors in a community in Southeast Brazil - The Bambuí Health and Ageing Study. Arq bras cardiol

2001 December;77(6):576-81. Study limited to adults
Ref ID: 3976

(518) Barrett LA, Morris JG, Stensel DJ, Nevill ME. Exercise and postprandial plasma triacylglycerol concentrations in healthy adolescent boys. Medicine and Science in Sports and Exercise 2007 January;39(1):116-22. Study less than 4 weeks
Ref ID: 1284

(519) Barrett ML, Udani JK. A proprietary alpha-amylase inhibitor from white bean (Phaseolus vulgaris): a review of clinical studies on weight loss and glycemic control. [Review]. Nutrition Journal 2011;10(1):24-33. Review article
Ref ID: 2411

(520) Barría P, Amigo C. Transición Nutricional: una revisión del perfil latinoamericano. Archivos Latinoamericanos de Nutrición 2006 March;56(1):3-11. Review article
Ref ID: 3977

(521) Barros C, Araújo T, Andrade E, Cruciani F, Matsudo V. Avaliação das variáveis de força muscular, agilidade e composição corporal em crianças vivendo com HIV/AIDS. Revista Brasileira de Ciência e Movimento 2006;14(4):47-54. Cross-sectional study
Ref ID: 3978

(522) Barros JWO, de Almeida MB, dos Santos MAM, de Santana PR, Campos FDCE, Leandro CG. Can birth weight influence nutritional status, physical activity levels and health-related physical fitness levels of children and adolescents? Revista de Nutricao-Brazilian Journal of Nutrition 2011;24(5):777-84. Review article
Ref ID: 5148

(523) Barros MV, Ritti-Dias RM, Honda Barros SS, Mota J, Andersen LB. Does self-reported physical activity associate with high blood pressure in adolescents when adiposity is adjusted for? Journal of Sports Sciences 2013;31(4):387-95. Inappropriate Study Design
Ref ID: 5757

(524) Barros MBdA, Zanchetta LM, Moura ECd, Malta DC. Auto-avaliação da saúde e fatores associados, Brasil, 2006. Auto-evaluación de la salud y factores asociados, Brasil, 2006. Self-rated health and associated factors, Brazil, 2006. Revista de Saúde Pública 2009 November;43(supl.2):27-37. Study limited to adults
Ref ID: 3980

(525) Barros SSH, Lopes AdS, Barros MVGd. Prevalência de baixo nivel de atividade física em crianças pré-escolares. Prevalence of low physical activity level among preschool children. Revista Brasileira de Cineantropometria e

Desempenho Humano 2012;14(4):390-400. Cross-sectional study
Ref ID: 3981

(526) Barry JJ. The recognition and management of mood disorders as a comorbidity of epilepsy. Epilepsia (Series 4) 2003 April 2;44:30-40. Review article
Ref ID: 3524

(527) Barshop NJ, Sirlin CB, Schwimmer JB, Lavine JE. Review article: Epidemiology, pathogenesis and potential treatments of paediatric non-alcoholic fatty liver disease. Aliment Pharmacol Ther 2008;28(1):13-24. Review article
Ref ID: 3193

(528) Barst RJ, Ivy D, Dingemanse J, Widlitz A, Schmitt K, Doran A, Bingaman D, Nguyen N, Gaitonde M, van Giersbergen PL. Pharmacokinetics, safety, and efficacy of bosentan in pediatric patients with pulmonary arterial hypertension. Clinical Pharmacology and Therapeutics 2003 April;73(4):372-82. Drug intervention study
Ref ID: 1756

(529) Bartlett AV, Torun B, Morales C, Cano F, Cruz JR. Oral gentamicin is not effective treatment for persistent diarrhea. Acta Paediatrica Supplement 1992 September;381:149-54. Drug intervention study
Ref ID: 2282

(530) Bartolucci G, Younger J. Tentative classification of neuropsychiatric disturbances in Prader-Willi syndrome. Journal of Intellectual Disability Research 1994 December;38(6):621-9. Review article
Ref ID: 3802

(531) Baruki SBS, Rosado LEFPdL, Rosado GP, Ribeiro RdCL. Associação entre estado nutricional e atividade física em escolares da Rede Municipal de Ensino em Corumbá - MS. Revista Brasileira de Medicina do Esporte 2006 April;12(2):90-4. Cross-sectional study
Ref ID: 3982

(532) Bas P, Romagnoli M, Gomez-Cabrera MC, Bas JL, Aura JV, Franco N, Bas T. Beneficial effects of aerobic training in adolescent patients with moderate idiopathic scoliosis. European Spine Journal 2011 August;20:Suppl-9. Not a randomized controlled trial (RCT)
Ref ID: 2412

(533) Bass MM, Duchowny CA, Llabre MM. The effect of therapeutic horseback riding on social functioning in children with autism. Journal of autism and developmental disorders 2009;39:1261-7. Not a randomized controlled trial (RCT)
Ref ID: 4683

(534) Bass SL, Naughton G, Saxon L, Iuliano BS, Daly R, Briganti EM, Hume C, Nowson C. Exercise and calcium combined results in a greater osteogenic effect than either factor alone: a blinded randomized placebo-controlled trial in boys. Journal of Bone and Mineral Research 2007;22:458-64. Inappropriate Comparison Group
Ref ID: 4684

(535) Bassan H, Bassan M, Pinhasov A, Kariv N, Giladi E, Gozes I, Harel S. The pregnant spontaneously hypertensive rat as a model of asymmetric intrauterine growth retardation and neurodevelopmental delay. Hypertension in Pregnancy 2005;24(3):201-11. Animal study
Ref ID: 5149

(536) Bassett DR. Physical activity of Canadian and American children: a focus on youth in Amish, Mennonite, and modern cultures. Applied Physiology Nutrition and Metabolism-Physiologie Appliquee Nutrition et Metabolisme 2008;33(4):831-5. Review article
Ref ID: 5150

(537) Basso RP, Jamami M, Pessoa BV, Labadessa IG, Regueiro EMG, Di Lorenzo VAP. Avaliação da capacidade de exercício em adolescentes asmáticos e saudáveis. Assessment of exercise capacity among asthmatic and healthy adolescents. Revista Brasileira de Fisioterapia 2010 June;14(3):252-8. Review article
Ref ID: 3983

(538) Bassols J, Prats-Puig A, Vazquez-Ruiz M, Garcia-Gonzalez MM, Martinez-Pascual M, Avelli P, Martinez-Martinez R, Fabrega R, Colomer-Virosta C, Soriano-Rodriguez P, Diaz M, de Zegher F, Ibanez L, Lopez-Bermejo A. Placental FTO expression relates to fetal growth. International Journal of Obesity 2010;34(9):1365-70. Not an exercise intervention study
Ref ID: 5151

(539) Bastani F, Hidarnia A, Montgomery KS, Aguilar-Vafaei ME, Kazemnejad A. Does relaxation education in anxious primigravid Iranian women influence adverse pregnancy outcomes?: a randomized controlled trial. Journal of Perinatal and Neonatal Nursing 2006;20:138-46. Study limited to adults
Ref ID: 4685

(540) Bateman A, Culpan FJ, Pickering AD, Powell JH, Scott OM, Greenwood RJ. The effect of aerobic training on rehabilitation outcomes after recent severe brain injury: a randomized controlled evaluation. Archives of Physical Medicine and Rehabilitation 2001 February;82(2):174-82. Not All Participants were Overweight and/or Obese
Ref ID: 1928

(541) Bateman LA, Slentz CA, Willis LH, Shields AT, Piner LW, Bales CW, Houmard JA, Kraus WE. Comparison of aerobic versus resistance exercise training effects on metabolic syndrome (from the Studies of a Targeted Risk Reduction Intervention Through Defined Exercise - STRRIDE-AT/RT). American Journal of Cardiology 2011 September 15;108(6):838-44. Study limited to adults
Ref ID: 1087

(542) Batista Júnior ML, Franchini E, Uchida MC, Rosa LFBPC. Efeito da suplementação de creatina sobre o desempenho na velocidade do swing e no tempo para percorrer três bases (home base - terceira base) em atletas da seleção brasileira de beisebol juvenil (16 a 18 anos). Revista Brasileira de Ciência e Movimento 2005;13(4):85-92. Diet Intervention or Supplement Study
Ref ID: 3984

(543) Batista EdS, Sabarense CM, Priore SE, Rosa DD, Montezano IM, Peluzio MdCG. Hábito alimentar, níveis de lipídios sangüíneos e o status antioxidante de adultos jovens fumantes e não fumantes. Revista de Nutrição 2009 June;22(3):377-88. Cross-sectional study
Ref ID: 3985

(544) Batrouni L, Navarro A, Sabulsky J, Fanto S, Rodriguez A. Situación alimentaria de escolares en relación con su condición social: Córdoba, República Argentina. Archivos Latinoamericanos de Nutrición 1993 March;43(1):12-9. Diet Intervention or Supplement Study
Ref ID: 3986

(545) Battaglini CL, Hackney AC, Garcia R, Groff D, Evans E, Shea T. The effects of an exercise program in leukemia patients. Integrative Cancer Therapies 2009 June;8(2):130-8. Study limited to adults
Ref ID: 699

(546) Battistella PA, Ruffilli R, Moro R, Fabiani M, Bertoli S, Antolini A, Zacchello F. A placebo-controlled crossover trial of nimodipine in pediatric migraine. Headache 1990;30(5):264-8. Drug intervention study
Ref ID: 3194

(547) Bauer CR, Langer JC, Shankaran S, Bada HS, Lester B, Wright LL, Krause SH, Smeriglio VL, Finnegan LP, Maza PL, Verter J. Acute neonatal effects of cocaine exposure during pregnancy. Archives of Pediatrics and Adolescent Medicine 2005;159:824-34. Not an exercise intervention study
Ref ID: 4686

(548) Baum M. Ask the expert. Pediatric Nephrology 2000 January 15;14(2):184-5. Abstract
Ref ID: 524

(549) Bautista-Castano I, Sangil-Monroy M, Serra-Majem L, Comite dN. [Knowledge and gaps on the role of nutrition and physical activity on the onset of childhood

obesity]. [Spanish]. Medicina Clinica 2004 December 4;123(20):782-93. Review article
Ref ID: 1565

(550) Baynard T, Miller WC, Fernhall B. Effects of exercise on vasodilatory capacity in endurance- and resistance-trained men. European Journal of Applied Physiology 2003 March;89(1):69-73. Not a randomized controlled trial (RCT)
Ref ID: 1768

(551) Bayne-Smith M, Fardy PS, Azzollini A, Magel J, Schmitz KH, Agin D. Improvements in heart health behaviors and reduction in coronary artery disease risk factors in urban teenaged girls through a school-based intervention: the PATH program. American Journal of Public Health 2004 September;94(9):1538-43. Lifestyle Intervention
Ref ID: 1593

(552) Bárzaga Arencibia ZM, Barranco Pedraza LM, López Leyva A, de la Torre Rosés M. La moxibustión en el tratamiento de la infertilidad femenina asociada a la insuficiencia de Yang de riñón. Moxibustion in the treatment of feminine infertility associated to Yang insuffiency of kidney. Archivo Médico de Camagüey 2009;13(1). Drug intervention study
Ref ID: 3987

(553) Berard E, Crosnier H, Six-Beneton A, Chevallier T, Cochat P, Broyer M. Recombinant human growth hormone treatment of children on hemodialysis. Pediatric Nephrology 1998 May;12(4):304-10. Drug intervention study
Ref ID: 3525

(554) Bean MK, Mazzeo SE, Stern M, Bowen D, Ingersoll K. A values-based Motivational Interviewing (MI) intervention for pediatric obesity: study design and methods for MI Values. Contemporary Clinical Trials 2011 September;32(5):667-74. Description of study from review or magazine or etc. (not the actual study)
Ref ID: 1091

(555) Beauchamp MR, Rhodes RE, Hua S, Morton KL, Kreutzer C, Liang JA, Khou KY, Dominelli PB, Daoud DM, Sherman MF, Dunlop WL, Sheel AW. Testing the effects of an expectancy-based intervention among adolescents: Can placebos be used to enhance physical health? Psychology Health and Medicine 2011 August;16(4):405-17. Not a randomized controlled trial (RCT)
Ref ID: 2414

(556) Becerra C, Gonzales GF, Villena A, De la Cruz D, Florián A. Prevalencia de anemia en gestantes, Hospital Regional de Pucallpa, Perú. Revista Panamericana de Salud Pública 1998 May;3(5):285-92. Cross-sectional study
Ref ID: 3988

(557) Beck CC, Lopes AdS, Giuliano IdCB, Borgatto AF. Fatores de risco cardiovascular em adolescentes de município do sul do Brasil: Prevalência e associações com variáveis sociodemográficas. Cardiovacular risk factors in adolescents from a town in the Brazilian South: Prevalence and association with sociodemographic variables. Revista Brasileira de Eidemiologia 2011 March;14(1):36-49. Cross-sectional study
Ref ID: 3989

(558) Becker AJ, Uckert S, Stief CG, Jonas U. Growth hormone, somatomedins and men's health. The Aging Male 2002;5:258-62. Review article
Ref ID: 4687

(559) Becker DJ, Gordon RY, Morris PB, Yorko J, Gordon YJ, Li M, Iqbal N. Simvastatin vs therapeutic lifestyle changes and supplements: randomized primary prevention trial. Mayo Clinic Proceedings 2008 July;83(7):758-64. Drug intervention study
Ref ID: 920

(560) Becker MdMC, Silva OB, Moreira IEG, Victor EG. Pressão arterial em adolescentes durante teste ergométrico. Arquivos Brasileiros de Cardiologia 2007 March;88(3):329-33. Cross-sectional study
Ref ID: 3990

(561) Beckett C, Durnin JV, Aitchison TC, Pollitt E. Effects of an energy and micronutrient supplement on anthropometry in undernourished children in Indonesia. European Journal of Clinical Nutrition 2000 May;54:Suppl-9. Diet Intervention or Supplement Study
Ref ID: 1969

(562) Beckham SG, Earnest CP. Metabolic cost of free weight circuit weight training. Journal of Sports Medicine and Physical Fitness 2000 June;40(2):118-25. Not a randomized controlled trial (RCT)
Ref ID: 1955

(563) Becque MD, Katch VL, Rocchini AP, Marks CR, Moorehead C. Coronary risk incidence of obese adolescents: reduction by exercise plus diet intervention. Pediatrics 1988 May;81(5):605-12. No exercise only group
Ref ID: 447

(564) Beech BM, Klesges RC, Kumanyika SK, Murray DM, Klesges L, McClanahan B, Slawson D, Nunnally C, Rochon J, McLain-Allen B, Pree-Cary J. Child- and parent-targeted interventions: the Memphis GEMS pilot study. Ethnicity and Disease 2003;13(1 Suppl 1):S40-S53. No exercise only group
Ref ID: 367

(565) Beech BM, Kumanyika SK, Baranowski T, Davis M, Robinson TN, Sherwood NE, Taylor WC, Relyea G, Zhou A, Pratt C, Owens A, Thompson NS. Parental cultural perspectives in relation to weight-related behaviors and concerns of

African-American girls. Obesity Research 2004 September;12:Suppl-19S. No exercise only group
Ref ID: 1580

(566) Behar Astudillo R, Hernández T. Deportes y trastornos de la conducta alimentaria. Revista médica de Chile 2002 March;130(3):287-94. Survey or questionnaire
Ref ID: 751

(567) Behar A. Trastornos de la conducta alimentaria no especificados, síndromes parciales y cuadros subclínicos: una alerta para la atención primaria: [revisión]. Revista médica de Chile 2008 December;136(12):1589-98. Review article
Ref ID: 3991

(568) Beilin L, Burke V, Milligan R. Strategies for prevention of adult hypertension and cardiovascular risk behaviour in childhood. An Australian perspective. Journal of Human Hypertension 1996 February;10:Suppl-4. Cross-sectional study
Ref ID: 2180

(569) Belcher JD, Ellison RC, Shepard WE, Bigelow C, Webber LS, Wilmore JH, Parcel GS, Zucker DM, Luepker RV. Lipid and lipoprotein distributions in children by ethnic group, gender, and geographic location--preliminary findings of the Child and Adolescent Trial for Cardiovascular Health (CATCH). Preventive Medicine 1993 March;22(2):143-53. Lifestyle Intervention
Ref ID: 2268

(570) Belenchia AM, Tosh AK, Hillman LS, Peterson CA. Correcting vitamin D insufficiency improves insulin sensitivity in obese adolescents: a randomized controlled trial. Am J Clin Nutr 2013 April;97(4):774-81. Inappropriate Intervention
Ref ID: 5758

(571) Bell AC, Wolfenden L, Sutherland R, Coggan L, Young K, Fitzgerald M, Hodder R, Orr N, Milat AJ, Wiggers J. Harnessing the power of advertising to prevent childhood obesity. International Journal of Behavioral Nutrition and Physical Activity 2013;10:114. Inappropriate Study Design
Ref ID: 5759

(572) Bell C, Seals DR, Monroe MB, Day DS, Shapiro LF, Johnson DG, Jones PP. Tonic sympathetic support of metabolic rate is attenuated with age, sedentary lifestyle, and female sex in healthy adults. Journal of Clinical Endocrinology and Metabolism 2001 September;86(9):4440-4. Study limited to adults
Ref ID: 1896

(573) Bell C, Stob NR, Seals DR. Thermogenic responsiveness to beta-adrenergic stimulation is augmented in exercising versus sedentary adults: role of oxidative stress. Journal of Physiology 2006 February 1;570(Pt:3):3-35. Study

limited to adults
Ref ID: 1426

(574) Bell EF, Hansen NI, Morriss FH, Stoll BJ, Ambalavanan N, Gould JB, Laptook AR, Walsh MC, Carlo WA, Shankaran S, Das A, Higgins RD. Impact of Timing of Birth and Resident Duty-Hour Restrictions on Outcomes for Small Preterm Infants. Pediatrics 2010;126(2):222-31. Not an exercise intervention study
Ref ID: 5152

(575) Bellissimo N, Thomas SG, Goode RC, Anderson GH. Effect of short-duration physical activity and ventilation threshold on subjective appetite and short-term energy intake in boys. Appetite 2007;49:644-51. Study less than 4 weeks
Ref ID: 4688

(576) Bellows LL, Davies PL, Anderson J, Kennedy C. Effectiveness of a physical activity intervention for Head Start preschoolers: a randomized intervention study. American Journal of Occupational Therapy 2013 January;67(1):28-36. Inappropriate Intervention
Ref ID: 5760

(577) Belury MA. Dietary conjugated linoleic acid in health: Physiological effects and mechanisms of action. Annual Review of Nutrition 2002 August;22(1):505. Review article
Ref ID: 3526

(578) Ben-Menachem E. Vigabatrin's Complicated Journeyâ€”To Be or Not to Be? Epilepsy Currents 2009 September;9(5):130-2. Retrospective study
Ref ID: 3527

(579) Ben Ounis OF, Elloumi MF, Ben C, I, Zbidi AF, Amri MF, Lac GF, Tabka Z. Effects of two-month physical-endurance and diet-restriction programmes on lipid profiles and insulin resistance in obese adolescent boys. Diabetes and Metabolism 2008;34:595-600. No comparative control group
Ref ID: 462

(580) Ben AN, Chaouachi A, Chamari K, Chtara M, Castagna C. Positional role and competitive-level differences in elite-level men's basketball players. Journal of Strength and Conditioning Research 2010 May;24(5):1346-55. Cross-sectional study
Ref ID: 538

(581) Ben OO, Elloumi M, Amri M, Zbidi A, Tabka Z. Impact of diet, exercise and diet combined with exercise programs on plasma lipoprotein and adiponectin levels in obese girls. J Sports Sci Med 2008;7(4):437-45. No comparative control group
Ref ID: 461

(582) Ben OO, Elloumi M, Zouhal H, Makni E, Denguezli M, Amri M, Lac G, Tabka Z. Effect of individualized exercise training combined with diet restriction on inflammatory markers and IGF-1/IGFBP-3 in obese children. Annals of Nutrition and Metabolism 2010;56(4):260-6. No exercise only group
Ref ID: 47

(583) Ben SH, Gaigi I, El FH, Gaigi S, El AJ. [Bulimia and anorexia among the teenagers]. [French]. Tunisie Medicale 2011 November;89(11):820-4. Not a randomized controlled trial (RCT)
Ref ID: 2416

(584) Bender BG, Fuhlbrigge A, Walders N, Zhang L. Overweight, race, and psychological distress in children in the Childhood Asthma Management Program. Pediatrics 2007 October;120(4):805-13. Not a randomized controlled trial (RCT)
Ref ID: 214

(585) Beneke R, Hutler M, Jung M, Leithauser RM. Modeling the blood lactate kinetics at maximal short-term exercise conditions in children, adolescents, and adults. Journal of Applied Physiology 2005 August;99(2):499-504. Not an exercise intervention study
Ref ID: 1496

(586) Beneke R, Hutler M, Leithauser RM. Carbohydrate and fat metabolism related to blood lactate in boys and male adolescents. European Journal of Applied Physiology 2009;105(2):257-63. Not an exercise intervention study
Ref ID: 5153

(587) Benevento BT, Sipski ML. Neurogenic Bladder, Neurogenic Bowel, and Sexual Dysfunction in People With Spinal Cord Injury. Physical Therapy 2002 June;82(6):601-12. Review article
Ref ID: 3528

(588) Bengmark S, Gil A. Productos finales de la glicaciÃ³n y de la lipoxidaciÃ³n como amplificadores de la inflamaciÃ³n: papel de los alimentos. (Spanish). Nutricion Hospitalaria 2007 November;22(6):625-40. Review article
Ref ID: 3529

(589) Benini J, Karolczak APB. Benefícios de um programa de educação postural para alunos de uma escola municipal de Garibaldi, RS. Benefits of a posture education program for schoolchildren in the city of Garibaldi, RS. Fisioterapia e Pesquisa 2010 December;17(4):346-51. Educational intervention
Ref ID: 3992

(590) Benjamin SE, Ammerman A, Sommers J, Dodds J, Neelon B, Ward DS. Nutrition and physical activity self-assessment for child care (NAP SACC): results from a pilot intervention. Journal Of Nutrition Education And Behavior

2007;39:142-9. Lifestyle Intervention
Ref ID: 4689

(591) Benjamin SE, Tate DF, Bangdiwala SI, Neelon BH, Ammerman AS, Dodds JM, Ward DS. Preparing Child Care Health Consultants to address childhood overweight: a randomized controlled trial comparing web to in-person training. Maternal and Child Health Journal 2008 September;12(5):662-9. Not an exercise intervention study
Ref ID: 221

(592) Bennell K, Khan KM, Matthews B, De GM, Cook E, Holzer K, Wark JD. Hip and ankle range of motion and hip muscle strength in young female ballet dancers and controls. British Journal of Sports Medicine 1999 October;33(5):340-6. Cross-sectional study
Ref ID: 1994

(593) Bensch GW, Greos LS, Gawchik S, Kpamegan E, Newman KB. Linear growth and bone maturation are unaffected by 1 year of therapy with inhaled flunisolide hydrofluoroalkane in prepubescent children with mild persistent asthma: a randomized, double-blind, placebo-controlled trial. Annals of Allergy, Asthma and Immunology 2011;107:323-9. Drug intervention study
Ref ID: 4690

(594) Benson AC, Torode ME, Singh MA. Muscular strength and cardiorespiratory fitness is associated with higher insulin sensitivity in children and adolescents. International Journal of Pediatric Obesity 2006;1(4):222-31. Cross-sectional study
Ref ID: 1299

(595) Benson AC, Torode ME, Fiatarone Singh MA. A rationale and method for high-intensity progressive resistance training with children and adolescents. Contemporary Clinical Trials 2007 July;28(4):442-50. Description versus conduct of study
Ref ID: 255

(596) Benson AC, Torode ME, Fiatarone Singh MA. The effect of high-intensity progressive resistance training on adiposity in children: a randomized controlled trial. International Journal of Obesity (London) 2008 June;32(6):1016-27. Not All Participants were Overweight and/or Obese
Ref ID: 194

(597) Benson JE, Geiger CJ, Eiserman PA, Wardlaw GM. Relationship between nutrient intake, body mass index, menstrual function, and ballet injury. Journal of the American Dietetic Association 1989 January;89(1):58-63. Diet Intervention or Supplement Study
Ref ID: 2326

(598) Bentley GF, Goodred JK, Jago R, Sebire SJ, Lucas PJ, Fox KR, Stewart-Brown S, Turner KM. Parents' views on child physical activity and their implications for physical activity parenting interventions: a qualitative study. BMC Pediatr 2012;12:180. Inappropriate Study Design
Ref ID: 5761

(599) Bentley ME, Caulfield LE, Ram M, Santizo MC, Hurtado E, Rivera JA, Ruel MT, Brown KH. Zinc supplementation affects the activity patterns of rural Guatemalan infants. Journal of Nutrition 1997;127(7):1333-8. Subjects less than 2 years old
Ref ID: 5154

(600) Berenson AB, Radecki CM, Grady JJ, Rickert VI, Thomas A. A prospective, controlled study of the effects of hormonal contraception on bone mineral density. Obstetrics and Gynecology 2001 October;98(4):576-82. Study limited to adults
Ref ID: 1892

(601) Berenson AB, Breitkopf CR, Grady JJ, Rickert VI, Thomas A. Effects of hormonal contraception on bone mineral density after 24 months of use. Obstetrics and Gynecology 2004 May;103(5:Pt 1):t-906. Study limited to adults
Ref ID: 1635

(602) Berenson GS, Shear CL, Chiang YK, Webber LS, Voors AW. Combined low-dose medication and primary intervention over a 30-month period for sustained high blood pressure in childhood. The American Journal of the Medical Sciences 1990;299:79-86. Not an exercise intervention study
Ref ID: 1073

(603) Beresford SA, Locke E, Bishop S, West B, McGregor BA, Bruemmer B, Duncan GE, Thompson B. Worksite study promoting activity and changes in eating (PACE): design and baseline results. Obesity 2007 November;15:Suppl-15S. Study limited to adults
Ref ID: 1127

(604) Bergamo VR. Estabilidade: aspecto significativo na previsão do talento no basquetebol feminino. Revista Brasileira de Ciência e Movimento 2004;12(2):51-6. Not an exercise intervention study
Ref ID: 3994

(605) Bergendal A, Bremme K, Hedenmalm K, Larfars G, Odeberg J, Persson I, Sundstrom A, Kieler H. Risk factors for venous thromboembolism in pre-and postmenopausal women. Thromb Res 2012 October;130(4):596-601. Inappropriate Population
Ref ID: 5762

(606) Bergh C, Brodin U, Lindberg G, Sodersten P. Randomized controlled trial of a treatment for anorexia and bulimia nervosa. Proceedings of the National

Academy of Sciences of the United States of America 2002 July 9;99(14):9486-91. Not an exercise intervention study
Ref ID: 1813

(607) Bergh IH, van Stralen MM, Grydeland M, Bjelland M, Lien N, Andersen LF, Anderssen SA, Ommundsen Y. Exploring mediators of accelerometer assessed physical activity in young adolescents in the Health In Adolescents Study - a group randomized controlled trial. BMC Public Health 2012;12:814. Inappropriate Population
Ref ID: 5763

(608) Bergmann GG, de Araujo Bergmann ML, Hallal PC. Independent and combined associations of cardiorespiratory fitness and fatness with cardiovascular risk factors in Brazilian youth. Journal of Physical Activity and Health 2014 February;11(2):375-83. Inappropriate Study Design
Ref ID: 5764

(609) Bergmann MLdA, Bergmann GG, Halpern R, Rech RR, Constanzi CB, Alli LR. Colesterol total e fatores associados: Estudo de base escolar no sul do Brasil. Associated factors to total cholesterol: School based study in southern Brazil. Colesterol total y factores asociados: Estudio de base escolar en el sur del brasil. Arquivos Brasileiros de Cardiologia 2011 July;97(1):17-25. Cross-sectional study
Ref ID: 3995

(610) Bergstrom J, Hultman E. Synthesis of muscle glycogen in man after glucose and fructose infusion. Acta Medica Scandinavica 1967 July;182(1):93-107. Not an exercise intervention study
Ref ID: 2417

(611) Berkey CS, Rockett HR, Gillman MW, Colditz GA. One-year changes in activity and in inactivity among 10- to 15-year-old boys and girls: relationship to change in body mass index. Pediatrics 2003 April;111(4:Pt 1):t-43. Cross-sectional study
Ref ID: 1762

(612) Berleze A, Haeffner LSB, Valentini NC. Desempenho motor de crianças obesas: uma investigação do processo e produto de habilidades motoras fundamentais. Revista Brasileira de Cineantropometria e Desempenho Humano 2007 June;9(2). Cross-sectional study
Ref ID: 595

(613) BermÃºdez-HumarÃ¡n LG, Kharrat P, Chatel JM, Langella P. Lactococci and lactobacilli as mucosal delivery vectors for therapeutic proteins and DNA vaccines. Microbial Cell Factories 2011 January 2;10(Suppl 1):1-10. Review article
Ref ID: 3530

(614) Bernbaum JC, Umbach DM, Ragan NB, Ballard JL, Archer JL, Schmidt-Davis H, Rogan WJ. Pilot studies of estrogen-related physical findings in wants. Environmental Health Perspectives 2008;116(3):416-20. Subjects less than 2 years old
Ref ID: 5155

(615) Bernhoft A, Nafstad I, Engen P, Skaare JU. Effects of Prenatal and Postnatal Exposure to 3,3',4,4',5-Pentachlorobiphenyl on Physical Development, Neurobehavior and Xenobiotic-Metabolizing Enzymes in Rats. Environmental Toxicology and Chemistry 1994;13(10):1589-97. Animal study
Ref ID: 5156

(616) Berntsen S, Mowinckel P, Carlsen KH, Lodrup Carlsen KC, Pollestad Kolsgaard ML, Joner G, Anderssen SA. Obese children playing towards an active lifestyle. International Jorunal of Pediatric Obesity 2010;5(1):64-71. No exercise only group, No comparative control group
Ref ID: 118

(617) Berry DC, Neal M, Hall EG, McMurray RG, Schwartz TA, Skelly AH, Smith-Miller C. Recruitment and retention strategies for a community-based weight management study for multi-ethnic elementary school children and their parents. Public Health Nursing 2013 January;30(1):80-6. Inappropriate Study Design
Ref ID: 5765

(618) Bertakis KD, Azari R. The impact of obesity on primary care visits. Obesity Research 2005 September;13(9):1615-23. Not an exercise intervention study
Ref ID: 295

(619) Bertapelli F, Gorla JI, Costa LT, Freire F. Composição corporal em jovens com síndrome de down: Aspectos genéticos, ambientais e fisiológicos.
Body composition in down syndrome youngters: Genetic, environmental and physiologic aspects. Arquivos de Ciências da Saúde da UNIPAR 2012 August;15(2). Review article
Ref ID: 3996

(620) Bertrais S, Preziosi P, Mermen L, Galan P, Hercberg S, Oppert JM. Sociodemographic and geographic correlates of meeting current recommendations for physical activity in middle-aged french adults: the supplémentation en vitamines et minéraux antioxydants (suvimax) study. American Journal of Public Health 2004 September;94(9):1560-6. Cross-sectional study
Ref ID: 3803

(621) Besier TF, Lloyd DG, Ackland TR, Cochrane JL. Anticipatory effects on knee joint loading during running and cutting maneuvers. Medicine and Science in Sports and Exercise 2001 July;33(7):1176-81. Not an exercise intervention

study
Ref ID: 1906

(622) Beske SD, Alvarez GE, Ballard TP, Davy KP. Gender difference in cardiovagal baroreflex gain in humans. Journal of Applied Physiology 2001 November;91(5):2088-92. Study limited to adults
Ref ID: 1887

(623) Bessesen DH. Update on obesity. Journal of Clinical Endocrinology and Metabolism 2008;93(6):2027-34. Review article
Ref ID: 3195

(624) Betancourt León H, Díaz ME. Análisis longitudinal de los indicadores peso-edad, talla-edad y peso–talla en adolescentes de la Escuela Nacional de Ballet de Cuba. An venez nutr 2005;18(2):177-85. Cohort Study
Ref ID: 3997

(625) Betancourt León H, Aréchiga Viramontes J, Ramírez García CM, Díaz Sánchez ME. Determinación del peso corporal para la estatura de bailarines de ballet y danza moderna y folclórica de Cuba. An venez nutr 2009;22(2):69-75. Cross-sectional study
Ref ID: 3998

(626) Betzig L. Means, variances, and ranges in reproductive success: comparative evidence. Evolution and Human Behavior 2012;33(4):309-17. Not an exercise intervention study
Ref ID: 5157

(627) Bevan JC, Veall GR, Macnab AJ, Ries CR, Marsland C. Midazolam premedication delays recovery after propofol without modifying involuntary movements. Anesthesia and analgesia 1997;85:50-4. Drug intervention study
Ref ID: 4691

(628) Beyrouty P, Chan HM. Co-consumption of selenium and vitamin E altered the reproductive and developmental toxicity of methylmercury in rats. Neurotoxicology and Teratology 2006;28(1):49-58. Animal study
Ref ID: 5158

(629) Bhandari N, Mazumder S, Bahl R, Martines J, Black RE, Bhan MK, -Infant-Feeding-Study-Group. An educational intervention to promote appropriate complementary feeding practices and physical growth in infants and young children in rural Haryana, India. Journal of Nutrition 2004;134:2342-8. Educational intervention, Diet Intervention or Supplement Study
Ref ID: 4692

(630) Bhandari N, Taneja S, Mazumder S, Bahl R, Fontaine O, Bhan MK, Zinc Study Group. Adding zinc to supplemental iron and folic acid does not affect mortality and severe morbidity in young children. Journal of Nutrition 2007

January;137(1):112-7. Diet Intervention or Supplement Study
Ref ID: 1289

(631) Bhugra D, Mastrogianni A, Maharajh H, Harvey S. Prevalence of bulimic behaviours and eating attitudes in schoolgirls from Trinidad and Barbados. Transcultural Psychiatry 2003 September;40(3):409-28. Not an exercise intervention study
Ref ID: 1693

(632) Bhutta ZA, Nizami SQ, Isani Z. Zinc supplementation in malnourished children with persistent diarrhea in Pakistan. Pediatrics 1999;103:e42. Diet Intervention or Supplement Study
Ref ID: 4693

(633) Biassio LG, Matsudo SMM, Matsudo VKR. Impacto da menarca nas variáveis antropométricas e neuromotoras da aptidão física, analisado longitudinalmente. Revista Brasileira de Ciência e Movimento 2004;12(2):97-101. Not an exercise intervention study
Ref ID: 3999

(634) Biban P, Zangardi T, Baraldi E, Dussini N, Chiandetti L, Zacchello F. Mixed exhaled nitric oxide and plasma nitrites and nitrates ire newborn infants. Life Sciences 2001;68(25):2789-97. Subjects less than 2 years old
Ref ID: 5159

(635) Biddle MG, Vincent G, McCambridge A, Britton G, Dewes O, Elley CR, Moyes SA, Edge J. Randomised controlled trial of informal team sports for cardiorespiratory fitness and health benefit in Pacific adults. Journal of Primary Health Care 2011 December;3(4):269-77. Study limited to adults
Ref ID: 2418

(636) Biddle SJ, Gorely T, Stensel DJ. Health-enhancing physical activity and sedentary behaviour in children and adolescents. [Review] [146 refs]. Journal of Sports Sciences 2004 August;22(8):679-701. Review article
Ref ID: 1588

(637) Bielen E, Fagard R, Amery A. Inheritance of heart structure and physical exercise capacity: a study of left ventricular structure and exercise capacity in 7-year-old twins. European Heart Journal 1990 January;11(1):7-16. Not an exercise intervention study
Ref ID: 2316

(638) Bier ID, Wilson J, Studt P, Shakleton M. Auricular Acupuncture, Education, and Smoking Cessation: A Randomized, Sham-Controlled Trial. American Journal of Public Health 2002 October;92(10):1642-7. Not an exercise intervention study
Ref ID: 3804

(639) Bilaceroglu S, Perim K, Buyuksirin M, Celikten E. Prednisolone: a beneficial and safe adjunct to antituberculosis treatment? A randomized controlled trial. International Journal of Tuberculosis and Lung Disease 1999 January;3(1):47-54. Drug intervention study
Ref ID: 2043

(640) Bilger M, Speraw S, LaFranchi SH, Hanna CE. Androgen replacement in adolescents and young women with hypopituitarism. Journal of Pediatric Endocrinology 2005 April;18(4):355-62. Drug intervention study
Ref ID: 1523

(641) Billoo AG, Murtaza G, Memon MA, Khaskheli SA, Iqbal K, Rao MH. Comparison of oral versus injectable vitamin-D for the treatment of nutritional vitamin-D deficiency rickets. Journal of the College of Physicians and Surgeons Pakistan 2009;19:428-31. Diet Intervention or Supplement Study
Ref ID: 4694

(642) Binesh MT, Adeli K. Pharmacological management of metabolic syndrome and its lipid complications. DARU 2010;18(3):146-54. Drug intervention study
Ref ID: 3531

(643) Binks M, van MT. Utilization patterns and user characteristics of an ad libitum Internet weight loss program. Journal of Medical Internet Research 2010;12(1):e9. Diet Intervention Study
Ref ID: 561

(644) Bird SR, Wiles J, Robbins J. The effect of sodium bicarbonate ingestion on 1500-m racing time. Journal of Sports Sciences 1995 October;13(5):399-403. Diet Intervention or Supplement Study
Ref ID: 2192

(645) Birketvedt GS, Thom E, Bernersen B, Florholmen J. Combination of diet, exercise and intermittent treatment of cimetidine on body weight and maintenance of weight loss. A 42 months follow-up study. Med Sci Monit 2000 July;6(4):699-703. Study limited to adults
Ref ID: 400

(646) Birks EJ, Tansley PD, Hardy J, George RS, Bowles CT, Burke M, Banner NR, Khaghani A, Yacoub MH. Left ventricular assist device and drug therapy for the reversal of heart failure. New England Journal of Medicine 2006 November 2;355(18):1873-84. Drug intervention study
Ref ID: 1334

(647) Bischoff SC, Damms-Machado A, Betz C, Herpertz S, Legenbauer T, Low T, Wechsler JG, Bischoff G, Austel A, Ellrott T. Multicenter evaluation of an interdisciplinary 52-week weight loss program for obesity with regard to body weight, comorbidities and quality of life--a prospective study. International

Journal of Obesity 2012 April;36(4):614-24. Observational study
Ref ID: 2419

(648) Bishop C, Hudson VM, Hilton SC, Wilde C. A pilot study of the effect of inhaled buffered reduced glutathione on the clinical status of patients with cystic fibrosis. Chest 2005 January;127(1):308-17. Drug intervention study
Ref ID: 1551

(649) Bispo Júnior RZ, Kawano CT, Guedes AV. Chronic multiple knee ligament injuries: epidemiological analysis of more than one hundred cases. Clinics 2008;63(1):3-8. Retrospective study
Ref ID: 4000

(650) Bissonnette B, Sessler DI. Mild Hypothermia Does Not Impair Postanesthetic Recovery in Infants and Children. Anesthesia and analgesia 1993;76(1):168-72. Not an exercise intervention study
Ref ID: 5160

(651) Bjelland M, Bergh IH, Grydeland M, Klepp KI, Andersen LF, Anderssen SA, Ommundsen Y, Lien N. Changes in adolescents' intake of sugar-sweetened beverages and sedentary behaviour: results at 8 month mid-way assessment of the HEIA study--a comprehensive, multi-component school-based randomized trial. International Journal of Behavioral Nutrition and Physical Activity 2011;8:63. Survey or questionnaire
Ref ID: 2420

(652) Bjork J, Albin M, Grahn P, Jacobsson H, Ardo J, Wadbro J, Ostergren PO. Recreational values of the natural environment in relation to neighbourhood satisfaction, physical activity, obesity and wellbeing. Journal of Epidemiology and Community Health 2008 April;62(4):e2. Survey or questionnaire
Ref ID: 981

(653) Black LE, Swan PD, Alvar BA. Effects of intensity and volume on insulin sensitivity during acute bouts of resistance training. Journal of Strength and Conditioning Research 2010 April;24(4):1109-16. Acute study
Ref ID: 555

(654) Black MA, Cable NT, Thijssen DHJ, Green DJ. Importance of measuring the time course of flow-mediated dilatation in humans. Hypertension 2008;51(2):203-10. Not a randomized controlled trial (RCT)
Ref ID: 5161

(655) Black MM, Dubowitz H, Hutcheson J, Berenson HJ, Starr RH. A randomized clinical trial of home intervention for children with failure to thrive. Pediatrics 1995;95:807-14. Subjects less than 2 years old
Ref ID: 4695

(656) Black MM, Hager ER, Le K, Anliker J, Arteaga SS, Diclemente C, Gittelsohn J, Magder L, Papas M, Snitker S, Treuth MS, Wang Y. Challenge! Health promotion/obesity prevention mentorship model among urban, black adolescents. Pediatrics 2010 August;126(2):280-8. No exercise only group
Ref ID: 28

(657) Blackwell DL, Lucas JW, Clarke TC. Summary health statistics for U.S. adults: national health interview survey, 2012. Vital Health Stat 10 2014 February;(260):1-161. Inappropriate Population
Ref ID: 5766

(658) Blackwell PL. The influence of touch on child development: Implications for intervention. Infants and Young Children 2000;13(1):25-39. Review article
Ref ID: 5162

(659) Blair D, Buskirk ER. Habitual daily energy expenditure and activity levels of lean and adult-onset and child-onset obese women. American Journal of Clinical Nutrition 1987 March;45(3):540-50. Acute study
Ref ID: 2342

(660) Blair SN, Chandler JV, Ellisor DB, Langley T. Improving physical fitness by exercise training programs. Southern Medical Journal 1980 December;73(12):1594-6. Not a randomized controlled trial (RCT)
Ref ID: 2376

(661) Blanc S, Normand S, Pachiaudi C, Duvareille M, Gharib C. Leptin responses to physical inactivity induced by simulated weightlessness. American Journal of Physiology-Regulatory Integrative and Comparative Physiology 2000;279(3):R891-R898. Acute study
Ref ID: 5163

(662) Bleich SN, Segal J, Wu Y, Wilson R, Wang Y. Systematic review of community-based childhood obesity prevention studies. Pediatrics 2013 July;132(1):e201-e210. Inappropriate Study Design
Ref ID: 5767

(663) Blevins T, Pullman J, Malloy J, Yan P, Taylor K, Schulteis C, Trautmann M, Porter L. DURATION-5: exenatide once weekly resulted in greater improvements in glycemic control compared with exenatide twice daily in patients with type 2 diabetes. Journal of Clinical Endocrinology and Metabolism 2011 May;96(5):1301-10. Drug intervention study
Ref ID: 2421

(664) Bleyenheuft Y, Thonnard JL. Grip Control in Children before, during, and after Impulsive Loading. Journal of Motor Behavior 2010;42(3):169-77. Not All Participants were Overweight and/or Obese
Ref ID: 5164

(665) Block KI, Block P, Gyllenhaal C. The Role of Optimal Healing Environments in Patients Undergoing Cancer Treatment: Clinical Research Protocol Guidelines. Journal of Alternative and Complementary Medicine 2004 October 2;10:S-157. Review article
Ref ID: 3532

(666) Blohm D, Ploch T, Apelt S. [Efficacy of exercise therapy to reduce cardiometabolic risk factors in overweight and obese children and adolescents: a systematic review]. Deutsche medizinische Wochenschrift 2012 December;137(50):2631-6. Inappropriate Study Design
Ref ID: 5768

(667) Bloom DE, Canning D, Shenoy ES. The effect of vaccination on children's physical and cognitive development in the Philippines. Applied Economics 2012;44(21):2777-83. Survey or questionnaire
Ref ID: 5165

(668) Bloom T, Sharpe L, Mullan B, Zucker N. A pilot evaluation of appetite-awareness training in the treatment of childhood overweight and obesity: a preliminary investigation. International Journal of Eating Disorders 2013 January;46(1):47-51. Inappropriate Intervention
Ref ID: 5769

(669) Bloomfield SA. Contributions of physical activity to bone health over the lifespan. Topics in Geriatric Rehabilitation 2005;21(1):68-76. Review article
Ref ID: 5166

(670) Boa-Sorte N, Neri LA, Leite ME, Brito SM, Meirelles AR, Luduvice FBS, Santos JP, Viveiros MR, Ribeiro-Júnior HC. Percepção materna e autopercepção do estado nutricional de crianças e adolescentes de escolas privadas. Jornal de Pediatria 2007 August;83(4):349-56. Cross-sectional study
Ref ID: 4001

(671) Bocca G, Corpeleijn E, Stolk RP, Sauer PJ. Results of a multidisciplinary treatment program in 3-year-old to 5-year-old overweight or obese children: a randomized controlled clinical trial. Arch Pediatr Adolesc Med 2012 December;166(12):1109-15. Inappropriate Intervention
Ref ID: 5770

(672) Boddy LM, Knowles ZR, Davies IG, Warburton GL, Mackintosh KA, Houghton L, Fairclough SJ. Using formative research to develop the healthy eating component of the CHANGE! school-based curriculum intervention. BMC Public Health 2012;12:710. Inappropriate Study Design
Ref ID: 5771

(673) Bodinier M, Brossard C, Triballeau S, Morisset M, Guerin-Marchand C, Pineau F, de Coppet P, Moneret-Vautrin DA, Blank U, Denery-Papini S. Evaluation of an in vitro mast cell degranulation test in the context of food allergy to wheat.

International Archives of Allergy and Immunology 2008;146(4):307-20. Not an exercise intervention study
Ref ID: 5167

(674) Boellner SW, Earl CQ, Arora S. Modafinil in children and adolescents with attention-deficit/hyperactivity disorder: a preliminary 8-week, open-label study. Curr Med Res Opin 2006;22:2457-65. Drug intervention study
Ref ID: 4696

(675) Boellner SW, Stark JG, Krishnan S, Zhang Y. Pharmacokinetics of lisdexamfetamine dimesylate and its active metabolite, d-amphetamine, with increasing oral doses of lisdexamfetamine dimesylate in children with attention-deficit/hyperactivity disorder: a single-dose, randomized, open-label, crossover study. Clinical Therapeutics 2010;32:252-64. Drug intervention study
Ref ID: 4697

(676) Boer JM, Ehnholm C, Menzel HJ, Havekes LM, Rosseneu M, O'Reilly DS, Tiret L. Interactions between lifestyle-related factors and the ApoE polymorphism on plasma lipids and apolipoproteins. The EARS Study. European Atherosclerosis Research Study. Arteriosclerosis, Thrombosis and Vascular Biology 1997 September;17(9):1675-81. Study limited to adults
Ref ID: 2123

(677) Bogarin R, Chanoine JP. Efficacy, safety and tolerability of orlistat, a lipase inhibitor, in the treatment of adolescent weight excess. Therapy 2009;6(1):23-30. Review article
Ref ID: 3196

(678) Bogin B, Varela-Silva MI. Leg length, proportion, health and beauty: a review. Anthropologischer Anzeiger 2009;67(4):439-59. Review article
Ref ID: 5168

(679) Bohdjalian A, Prager G, Rosak C, Weiner R, Jung R, Schramm M, Aviv R, Schindler K, Haddad W, Rosenthal N, Ludvik B. Improvement in glycemic control in morbidly obese type 2 diabetic subjects by gastric stimulation. Obesity Surgery 2009 September;19(9):1221-7. Not an exercise intervention study
Ref ID: 693

(680) Bohler T, Alex C, Becker E, Becker R, Hoffmann S, Hutzler D, Jung C, Laufersweiler-Lochmann F, Radu C. [Quality indicators for ambulatory health education programme for overweight and obese children and adolescents]. [German]. Gesundheitswesen 2004 November;66(11):748-53. Review article
Ref ID: 1569

(681) Bohman B, Forsberg L, Ghaderi A, Rasmussen F. An evaluation of training in motivational interviewing for nurses in child health services. Behav Cogn

Psychother 2013 May;41(3):329-43. Inappropriate Study Design
Ref ID: 5773

(682) Bohman B, Eriksson M, Lind M, Ghaderi A, Forsberg L, Rasmussen F. Infrequent attention to dietary and physical activity behaviours in conversations in Swedish child health services. Acta Paediatr 2013 May;102(5):520-4. Inappropriate Study Design
Ref ID: 5772

(683) Bohnert A, Burdette K, Dugas L, Travers L, Randall E, Richards M, Luke A. Multimethod analyses of discretionary time use and health behaviors among urban low-income African-American adolescents: a pilot study. J Dev Behav Pediatr 2013 October;34(8):589-98. Inappropriate Population
Ref ID: 5774

(684) Bohórquez IM, Caballero S, Carrera L, Chávez R, Espinoza R, Flores L, Llanos M, Luna E, Vega J, Vera J, Salvatierra H, Pereyra H. Factores asociados a síntomas depresivos en trabajadoras sexuales. Factors associated to depression symptomatology in sex workers. An Fac Med (Perú) 2010 December;71(4):277-82. Cross-sectional study
Ref ID: 4002

(685) Boisseau N, Vermorel M, Rance M, Duche P, Patureau-Mirand P. Protein requirements in male adolescent soccer players. European Journal of Applied Physiology 2007;100(1):27-33. Not a randomized controlled trial (RCT)
Ref ID: 5169

(686) Boivin MJ, Busman RA, Parikh SM, Bangirana P, Page CF, Opoka RO, Giordani B. A pilot study of the neuropsychological benefits of computerized cognitive rehabilitation in Ugandan children with HIV. Neuropsychology 2010;24:667-73. Not an exercise intervention study
Ref ID: 4698

(687) Bolgla LA, Uhl TL. Electromyographic analysis of hip rehabilitation exercises in a group of healthy subjects. Journal of Orthopaedic and Sports Physical Therapy 2005 August;35(8):487-94. Study limited to adults
Ref ID: 1470

(688) Bolotova NV, Lazebnikova SV, Chicheva GV, Raigorodskaia NI. [The effectiveness of transcranial treatment using the AMO-ATOS-E apparatus for the correction of the reproductive system disorders in adolescent girls]. [Russian]. Voprosy Kurortologii, Fizioterapii i Lechebnoi Fizicheskoi Kultury (6):30-3, 2010 Nov-Dec 2010 November;(6):30-3. Not an exercise intervention study
Ref ID: 2422

(689) Bolster DR, Pikosky MA, McCarthy LM, Rodriguez NR. Exercise affects protein utilization in healthy children. Journal of Nutrition 2001 October;131(10):2659-

63. Not a randomized controlled trial (RCT)
Ref ID: 1891

(690) Boman K, Hellsten G, Bruce A, Hallmans G, Nilsson TK. Endurance physical activity, diet and fibrinolysis. Atherosclerosis 1994 March;106(1):65-74. Study limited to adults
Ref ID: 2242

(691) Bond CM, Bonci LJ, Granger LR, Johnson CL, Malina RM, Milne LW, Ryan RA, Vanderbunt EM. National athletic trainers' association position statement: preventing, detecting, and managing disordered eating in athletes. Journal of Athletic Training 2008 January;43(1):80-108. Review article
Ref ID: 3805

(692) Bond M, Wyatt K, Lloyd J, Welch K, Taylor R. Systematic review of the effectiveness and cost-effectiveness of weight management schemes for the under fives: a short report. [Review] [95 refs]. Health Technology Assessment (Winchester, England) 2009 December 20;13(61):1-75. Review article
Ref ID: 624

(693) Bondi M, Grugni G, Velardo A, Biella O, Venneri MG, Morabito F, Menozzi R, Del Rio G. Adrenomedullary response to caffeine in prepubertal and pubertal obese subjects. International Journal of Obesity 1999;23(9):992-6. Diet Intervention or Supplement Study
Ref ID: 5170

(694) Bondy CA. Congenital Cardiovascular Disease in Turner Syndrome. Congenital Heart Disease 2008 January;3(1):2-15. Review article
Ref ID: 3533

(695) Bonifati MD, Ruzza G, Bonometto P, Berardinelli A, Gorni K, Orcesi S, Lanzi G, Angelini C. A multicenter, double-blind, randomized trial of deflazacort versus prednisone in duchenne muscular dystrophy. Muscle Nerve 2000;23(9):1344-7. Drug intervention study
Ref ID: 3197

(696) Bonifazi M, Bela E, Carli G, Lodi L, Martelli G, Zhu B, Lupo C. Influence of training on the response of androgen plasma concentrations to exercise in swimmers. European Journal of Applied Physiology and Occupational Physiology 1995;70(2):109-14. Acute study
Ref ID: 2209

(697) Bonneau M. Factors affecting the level of androstenone. Acta Veterinaria Scandinavica 2006 January 2;48:S7-3. Animal study
Ref ID: 3534

(698) Bonnefoy XR, Braubach M, Moissonnier B, Monolbaev K, Robbel N. Housing and Health in Europe: Preliminary Results of a Pan-European Study. American

Journal of Public Health 93[9], 1559-1563. 2003. Survey or questionnaire,
Ref ID: 3535

(699) Bonofiglio D, Maggiolini M, Marsico S, Giorno A, Catalano S, Aquila S, Andò S. Critical years and stages of puberty for radial bone mass apposition during adolescence. Hormone and metabolic research = Hormon und Stoffwechselforschung = Hormones et métabolisme 1999;31:478-82. Cross-sectional study
Ref ID: 4699

(700) Bonsergent E, Agrinier N, Thilly N, Tessier S, Legrand K, Lecomte E, Aptel E, Hercberg S, Collin JF, Briancon S. Overweight and obesity prevention for adolescents: a cluster randomized controlled trial in a school setting. American Journal of Preventive Medicine 2013 January;44(1):30-9. Inappropriate Population
Ref ID: 5775

(701) Boot AM, Nauta J, de Jong MCJW, Groothoff JW, Lilien MR, van Wijk JA, Kist-van Holthe JE, Hokken-Koelega S, Pols HAP, de Muinck Keizer-Schrama S, Boot. Bone mineral density, bone metabolism and body composition of children with chronic renal failure, with and without growth hormone treatment. Clinical Endocrinology 1998 November;49(5):665-72. Diet Intervention or Supplement Study
Ref ID: 548

(702) Booth CK, Coad RA, Forbes-Ewan CH, Thomson GF, Niro PJ. The physiological and psychological effects of combat ration feeding during a 12-day training exercise in the tropics. Military Medicine 2003 January;168(1):63-70. Diet Intervention Study
Ref ID: 1776

(703) Booth ML, Macaskill P, Lazarus R, Baur LA. Sociodemographic distribution of measures of body fatness among children and adolescents in New South Wales, Australia. International Journal of Obesity and Related Metabolic Disorders 1999 May;23(5):456-62. Not an exercise intervention study, Observational study
Ref ID: 409

(704) Bopp CM, Townsend DK, Barstow TJ. Characterizing near-infrared spectroscopy responses to forearm post-occlusive reactive hyperemia in healthy subjects. European Journal of Applied Physiology 2011 November;111(11):2753-61. Not an exercise intervention study
Ref ID: 2424

(705) Boreham C, Savage JM, Primrose D, Cran G, Strain J. Coronary risk factors in schoolchildren. Archives of Disease in Childhood 1993 February;68(2):182-6.

Cross-sectional study
Ref ID: 2269

(706) Boreham CA, Kennedy RA, Murphy MH, Tully M, Wallace WF, Young I. Training effects of short bouts of stair climbing on cardiorespiratory fitness, blood lipids, and homocysteine in sedentary young women. British Journal of Sports Medicine 2005 September;39(9):590-3. Not All Participants were Overweight and/or Obese
Ref ID: 1481

(707) Borges AF, Borin JP, De Marco A. Avaliação de indicadores antropométricos e neuromusculares de jovens escolares do ensino fundamental do interior paulista. Motriz Revista de Educação Física (Improv) 2010 June;16(2):326-37. Not a randomized controlled trial (RCT)
Ref ID: 4003

(708) Borges CR, Kõhler MLK, Leite MdL, Silva ABF, Camargo ATD, Kanunfre CC. Influência da televisão na prevalência de obesidade infantil em Ponta Grossa, Paraná. Ciência Cuidado e Saúde 2007 September;6(3):305-11. Cross-sectional study
Ref ID: 4004

(709) Borggraefe I, Schaefer JS, Klaiber M, Dabrowski E, Ammann-Reiffer C, Knecht B, Berweck S, Heinen F, Meyer-Heim A. Robotic-assisted treadmill therapy improves walking and standing performance in children and adolescents with cerebral palsy. European Journal of Paediatric Neurology 2010 November;14(6):496-502. Not a randomized controlled trial (RCT)
Ref ID: 2425

(710) Borovsky J, Kersz MJ, Kuper E. Valor de un examen médico de ingreso a una institución deportiva. Revista Argentina de Medicina del Deporte 1996;18(61):106-12. Not an exercise intervention study
Ref ID: 792

(711) Borradaile KE, Foster GD, May H, Karpyn A, Sherman S, Grundy K, Nachmani J, Vander VS, Boruch RF. Associations between the Youth/Adolescent Questionnaire, the Youth/Adolescent Activity Questionnaire, and body mass index z score in low-income inner-city fourth through sixth grade children. American Journal of Clinical Nutrition 2008 June;87(6):1650-5. Cross-sectional study
Ref ID: 940

(712) Borrelli A, Mattiazzi L, Capucchio MT, Biolatti C, Cagnasso A, Gianella P, D'Angelo A. Cachexia secondary to intracranial anaplastic (malignant) ependymoma in a boxer dog. Journal of Small Animal Practice 2009;50(10):554-7. Animal study
Ref ID: 5171

(713) Borsheim E, Kien CL, Pearl WM. Differential effects of dietary intake of palmitic acid and oleic acid on oxygen consumption during and after exercise. Metabolism: Clinical and Experimental 2006 September;55(9):1215-21. Diet Intervention or Supplement Study
Ref ID: 1358

(714) Bortman M. Factores de riesgo de bajo peso al nacer. Revista Panamericana de Salud Pública 1998 May;3(5):314-21. Cross-sectional study
Ref ID: 601

(715) Bortsov AV, Liese AD, Bell RA, Dabelea D, D'Agostino RB, Jr., Hamman RF, Klingensmith GJ, Lawrence JM, Maahs DM, McKeown R, Marcovina SM, Thomas J, Williams DE, Mayer-Davis EJ. Sugar-sweetened and diet beverage consumption is associated with cardiovascular risk factor profile in youth with type 1 diabetes. Acta Diabetologica 2011 December;48(4):275-82. Cross-sectional study
Ref ID: 2426

(716) Bosa VL, Mello EDd, Mocelin HT, Benedetti FJ, Fischer GB. Avaliação do estado nutricional de crianças e adolescentes com bronquiolite obliterante pós-infecciosa. Assessment of nutritional status in children and adolescents with post-infectious bronchiolitis obliterans. Jornal de Pediatria 2008 August;84(4):323-30. Cross-sectional study
Ref ID: 4005

(717) Boschi V, Siervo M, Nasti G, Trapanese E, D'Orsi P, Augelli E, Papa A, Margiotta N, Bellini O, Falconi C. Interdisciplinary treatment of a female outpatient population. Organizational model and preliminary results. Eating And Weight Disorders 2002 December;7(4):268-75. Diet Intervention Study
Ref ID: 1785

(718) Botvin GJ, Cantlon A, Carter BJ, Williams CL. Reducing adolescent obesity through a school health program. Journal of Pediatrics 1979 December;95(6):1060-3. Lifestyle Intervention
Ref ID: 2381

(719) Bouhlel E, Denguezli M, Zaouali M, Tabka Z, Shephard RJ. Ramadan fastings effect on plasma leptin, adiponectin concentrations, and body composition in trained young men. International Journal of Sport Nutrition and Exercise Metabolism 2008 December;18(6):617-27. Study limited to adults
Ref ID: 830

(720) Bourgeois JM, Nagel K, Pearce E, Wright M, Barr RD, Tarnopolsky MA. Creatine monohydrate attenuates body fat accumulation in children with acute lymphoblastic leukemia during maintenance chemotherapy. Pediatric Blood and Cancer 2008;51(2):183-7. Diet Intervention or Supplement Study
Ref ID: 5172

(721) Bourgois J, Vrijens J. The Conconi test: a controversial concept for the determination of the anaerobic threshold in young rowers. International Journal of Sports Medicine 1998 November;19(8):553-9. Not a randomized controlled trial (RCT)
Ref ID: 2072

(722) Boury JM, Larkin KT, Krummel DA. Factors related to postpartum depressive symptoms in low-income women. Women and Health 2004;39(3):19-34. Study limited to adults
Ref ID: 1607

(723) Boutelle KN, Cafri G, Crow SJ. Parent-only treatment for childhood obesity: a randomized controlled trial. Obesity 2011 March;19(3):574-80. Lifestyle Intervention
Ref ID: 2427

(724) Boutelle KN, Norman GJ, Rock CL, Rhee KE, Crow SJ. Guided self-help for the treatment of pediatric obesity. Pediatrics 2013 May;131(5):e1435-e1442. Inappropriate Intervention
Ref ID: 5776

(725) Bouten CVC, Koekkoek KTM, Verduin M, Kodde R, Janssen JD. A triaxial accelerometer and portable data processing unit for the assessment of daily physical activity. Ieee Transactions on Biomedical Engineering 1997;44(3):136-47. Not an exercise intervention study
Ref ID: 5173

(726) Bouza E, Munoz P. Linezolid: pharmacokinetic characteristics and clinical studies. Clinical Microbiology and Infection 2001 August 3;7:75-82. Drug intervention study
Ref ID: 3536

(727) Bowden RG, Lanning BA, Doyle EI, Slonaker B, Johnston HM, Scanes G. Systemic glucose level changes with a carbohydrate-restricted and higher protein diet combined with exercise. Journal of American College Health 2007 September;56(2):147-52. Diet Intervention Study
Ref ID: 1146

(728) Bower M, Collins S, Cottrill C, Cwynarski K, Montoto S, Nelson M, Nwokolo N, Powles T, Stebbing J, Wales N, Webb A. British HIV Association guidelines for HIV-associated malignancies 2008. HIV Medicine 2008 July;9(6):336-88. Review article
Ref ID: 3537

(729) Bowling FG, Munce TB. Abnormal protein glycoforms in Prader-Willi syndrome. Journal of Intellectual Disability Research 2008 October;52(10):812. Not an exercise intervention study
Ref ID: 3806

(730) Boyd RN, Dobson F, Parrott J, Love S, Oates J, Larson A, Burchall G, Chondros P, Carlin J, Nattrass G, Graham HK. The effect of botulinum toxin type A and a variable hip abduction orthosis on gross motor function: a randomized controlled trial. European Journal of Neurology 2001 November;8:Suppl-19. Drug intervention study
Ref ID: 1863

(731) Boyle CA. Surveillance of developmental disabilities with an emphasis on special studies. Reproductive Toxicology 1997;11(2-3):271-4. Not an exercise intervention study
Ref ID: 5174

(732) Boyle MH, Willms JD. Multilevel Modelling of Hierarchical Data in Developmental Studies. Journal of Child Psychology and Psychiatry and Allied Disciplines 2001 January;42(1):141. Not an exercise intervention study
Ref ID: 496

(733) Boynton JR, Thomas TN, Peterson KE, Wiecha J, Sobol AM, Gortmaker SL. Impact of television viewing patterns on fruit and vegetable consumption among adolescents. Pediatrics in review / American Academy of Pediatrics 2003;112:1321-6. Prospective Study
Ref ID: 4700

(734) Bozza R, Stabelini Neto A, Ulbrich AZ, Vasconcelos ÍQAd, Mascarenhas LPG, Brito LMS, Campos Wd. Circunferência da cintura, índice de massa corporal e fatores de risco cardiovascular na adolescência. Waist circumference, body mass index and cardiovascular risk factors in adolescence. Revista Brasileira de Cineantropometria e Desempenho Humano 2009;11(3):286-91. Cross-sectional study
Ref ID: 4006

(735) Braam W, Smits MG, Didden R, Curfs LMG. Melatonin is effective in treating sleep problems in Angelman syndrome but problems in metabolising melatonin may be part of the Angelman phenotype. Journal of Intellectual Disability Research 2008 October;52(10):814. Diet Intervention or Supplement Study
Ref ID: 3807

(736) Braamskamp MJ, Wijburg FA, Wiegman A. Drug therapy of hypercholesterolaemia in children and adolescents. Drugs 2012 April 16;72(6):759-72. Review article
Ref ID: 2428

(737) Bracco MM, Colugnati FAB, Pratt M, Taddei JAAC. Multivariate hierarchical model for physical inactivity among public school children. Jornal de Pediatria 2006 August;82(4):302-7. Survey or questionnaire
Ref ID: 4007

(738) Bracken RM, Linnane DM, Brooks S. Alkalosis and the plasma catecholamine response to high-intensity exercise in man. Medicine and Science in Sports and Exercise 2005 February;37(2):227-33. Acute study
Ref ID: 1547

(739) Bracko MR. On-ice performance characteristics of elite and non-elite women's ice hockey players. Journal of Strength and Conditioning Research 2001 February;15(1):42-7. Not an exercise intervention study
Ref ID: 1880

(740) Bracko MR, George JD. Prediction of ice skating performance with off-ice testing in women's ice hockey players. Journal of Strength and Conditioning Research 2001 February;15(1):116-22. Cross-sectional study
Ref ID: 1879

(741) Bradley RH, McRitchie S, Houts RM, Nader P, O'Brien M, NICHD Early Child Care Research Network. Parenting and the decline of physical activity from age 9 to 15. International Journal of Behavioral Nutrition and Physical Activity 2011;8:33. Cross-sectional study
Ref ID: 2429

(742) Bradney M, Pearce G, Naughton G, Sullivan C, Bass S, Beck T, Carlson J, Seeman E. Moderate exercise during growth in prepubertal boys: changes in bone mass, size, volumetric density, and bone strength: a controlled prospective study. Journal of Bone and Mineral Research 1998;13:1814-21. Not All Participants were Overweight and/or Obese
Ref ID: 4701

(743) Bradshaw B. The role of the family in managing therapy in minority children with type 2 diabetes mellitus. Journal of Pediatric Endocrinology 2002 April;15:Suppl-51. Not an exercise intervention study
Ref ID: 1825

(744) Brady ML, Allan AM, Caldwell KK. A Limited Access Mouse Model of Prenatal Alcohol Exposure that Produces Long-Lasting Deficits in Hippocampal-Dependent Learning and Memory. Alcoholism-Clinical and Experimental Research 2012;36(3):457-66. Animal study
Ref ID: 5175

(745) Braga PD, Molina MdCB, Cade NV. Expectativas de adolescentes em relação a mudanças do perfil nutricional. Ciência and Saúde Coletiva 2007 October;12(5):1221-8. Cross-sectional study
Ref ID: 4008

(746) Braham R, Finch CF, McIntosh A, McCrory P. Community level Australian Football: a profile of injuries. Journal of Science and Medicine in Sport 2004 March;7(1):96-105. Not an exercise intervention study
Ref ID: 1631

(747) Branca F, Popkin BM, Simopoulos AP. Preface by guest editors. International Journal of Obesity 2008 November;32:Suppl-3. Editorial or letter or comment
Ref ID: 858

(748) Brandäo CMA, Lombardi MT, Nishida SK, Hauache OM, Vieira JGH. Serum leptin concentration during puberty in healthy nonobese adolescents. Brazilian Journal of Medical and Biological Research 2003 October;36(10):1293-6. Not All Participants were Overweight and/or Obese
Ref ID: 724

(749) Brandes JM, Itskovitz J, Scher A, Gershonibaruch R. The Physical and Mental-Development of Co-Sibs Surviving Selective Reduction of Multifetal Pregnancies. Human Reproduction 1990;5(8):1014-7. Not an exercise intervention study
Ref ID: 5177

(750) Brandes JM, Scher A, Itzkovits J, Thaler I, Sarid M, Gershoni BR. Growth and development of children conceived by in vitro fertilization. Pediatrics 1992;90:424-9. Not an exercise intervention study
Ref ID: 1055

(751) Brandes M. The importance of physical activity and fitness for human health. Bundesgesundheitsblatt-Gesundheitsforschung-Gesundheitsschutz 2012;55(1):96-101. Review article
Ref ID: 5178

(752) Brandou F, Dumortier M, Garandeau P, Mercier J, Brun JF. Effects of a two-month rehabilitation program on substrate utilization during exercise in obese adolescents. Diabetes and Metabolism 2003 February;29(1):20-7. Not a randomized controlled trial (RCT)
Ref ID: 1767

(753) Brandou F, Savy-Pacaux AM, Marie J, Bauloz M, Maret-Fleuret I, Borrocoso S, Mercier J, Brun JF. Impact of high- and low-intensity targeted exercise training on the type of substrate utilization in obese boys submitted to a hypocaloric diet. Diabetes and Metabolism 2005 September;31(4:Pt 1):t-35. Diet Intervention Study
Ref ID: 1439

(754) Brandstetter S, Klenk J, Berg S, Galm C, Fritz M, Peter R, Prokopchuk D, Steiner RP, Wartha O, Steinacker J, Wabitsch M. Overweight prevention implemented by primary school teachers: a randomised controlled trial. Obesity Facts 2012;5(1):1-11. Not All Participants were Overweight and/or Obese
Ref ID: 2430

(755) Branger B, Cadudal JL, Delobel M, Ouoba H, Yameogo P, Ouedraogo D, Guerin D, Valea A, Zombre C, Ancel P, personnels-des CREN. [Spiruline as a food supplement in case of infant malnutrition in Burkina-Faso]. Archives de

pédiatrie : organe officiel de la Sociéte française de pédiatrie 2003;10:424-31. Diet Intervention or Supplement Study
Ref ID: 4702

(756) Branscum P, Sharma M, Wang LL, Wilson BR, Rojas-Guyler L. A true challenge for any superhero: an evaluation of a comic book obesity prevention program. Fam Community Health 2013 January;36(1):63-76. Inappropriate Intervention
Ref ID: 5777

(757) Branski LK, Herndon DN, Barrow RE, Kulp GA, Klein GL, Suman OE, Przkora R, Meyer W, Huang T, Lee JO, Chinkes DL, Mlcak RP, Jeschke MG. Randomized controlled trial to determine the efficacy of long-term growth hormone treatment in severely burned children. Annals of Surgery 2009;250(4):514-22. Drug intervention study
Ref ID: 3199

(758) Bratland-Sanda S, Rosenvinge JH, Vrabel KA, Norring C, Sundgot-Borgen J, Ro O, Martinsen EW. Physical activity in treatment units for eating disorders: clinical practice and attitudes. Eating And Weight Disorders 2009 June;14(2-3):e106-e112. Survey or questionnaire
Ref ID: 640

(759) Braun B, Gerson L, Hagobian T, Grow D, Chipkin SR. No effect of short-term testosterone manipulation on exercise substrate metabolism in men. Journal of Applied Physiology 2005 November;99(5):1930-7. Study limited to adults
Ref ID: 1462

(760) Bravender T, Russell A, Chung RJ, Armstrong SC. A "novel" intervention: a pilot study of children's literature and healthy lifestyles. Pediatrics 2010 March;125(3):e513-e517. Not an exercise intervention study
Ref ID: 60

(761) Breman JG, Holloway CN. Malaria surveillance counts. American Journal of Tropical Medicine and Hygiene 2007;77(6):36-47. Not an exercise intervention study
Ref ID: 5179

(762) Brennan L, Walkley J, Fraser SF, Greenway K, Wilks R. Motivational interviewing and cognitive behaviour therapy in the treatment of adolescent overweight and obesity: study design and methodology. Contemporary Clinical Trials 2008 May;29(3):359-75. Description of study from review or magazine or etc. (not the actual study)
Ref ID: 968

(763) Brennan L, Walkley J, Wilks R, Fraser SF, Greenway K. Physiological and behavioural outcomes of a randomised controlled trial of a cognitive behavioural lifestyle intervention for overweight and obese adolescents.

Obesity Research Clin Pract 2013 January;7(1):e23-e41. Inappropriate Intervention
Ref ID: 5778

(764) Brent RL, Tanski S, Weitzman M. A pediatric perspective on the unique vulnerability and resilience of the embryo and the child to environmental toxicants: The importance of rigorous research concerning age and agent. Pediatrics 2004;113(4):935-44. Not an exercise intervention study
Ref ID: 5180

(765) Brentano MA, Cadore EL, da Silva EM, Ambrosini AB, Coertjens M, Petkowicz R, Viero I, Kruel LF. Physiological adaptations to strength and circuit training in postmenopausal women with bone loss. Journal of Strength and Conditioning Research 2008 November;22(6):1816-25. Study limited to adults
Ref ID: 864

(766) Briancon S, Bonsergent E, Agrinier N, Tessier S, Legrand K, Lecomte E, Aptel E, Hercberg S, Collin JF, PRALIMAP Trial Group. PRALIMAP: study protocol for a high school-based, factorial cluster randomised interventional trial of three overweight and obesity prevention strategies. Trials [Electronic Resource] 2010;11:119. Lifestyle Intervention
Ref ID: 2431

(767) Briceño Y, Chirinos J, Paoli M, Zerpa Y. Panhipopituitarismo secundario a macroadenoma hipofisario no funcionante en la adolescencia. Revista Venezolana de Endocrinología y Metabolismo 2009 February;7(1):35-40. Case-Control / Case Study
Ref ID: 4009

(768) Bricout VA, Guinot M, Faure P, Flore P, Eberhard Y, Garnier P, Favre Juvin A. Are Hormonal Responses to Exercise in Young Men with Downâ€™s Syndrome Related to Reduced Endurance Performance? Journal of Neuroendocrinology 2008 May;20(5):558-65. Not an exercise intervention study
Ref ID: 3538

(769) Briley AL, Barr S, Badger S, Bell R, Croker H, Godfrey KM, Holmes B, Kinnunen TI, Nelson SM, Oteng-Ntim E, Patel N, Robson SC, Sandall J, Sanders T, Sattar N, Seed PT, Wardle J, Poston L. A complex intervention to improve pregnancy outcome in obese women; the UPBEAT randomised controlled trial. BMC Pregnancy Childbirth 2014;14:74. Inappropriate Population
Ref ID: 5779

(770) Brilla LR, Haley TF. Effect of magnesium supplementation on strength training in humans. Journal of the American College of Nutrition 1992 June;11(3):326-9. Diet Intervention or Supplement Study
Ref ID: 2288

(771) Brinkworth GD, Buckley JD, Slavotinek JP, Kurmis AP. Effect of bovine colostrum supplementation on the composition of resistance trained and untrained limbs in healthy young men. European Journal of Applied Physiology 2004 January;91(1):53-60. Diet Intervention or Supplement Study
Ref ID: 1674

(772) Brisch KH, Buchheim A, Kohntop B, Kunzke D, Schmucker G, Kachele H, Pohlandt F. Early preventive psychotherapeutic intervention for parents of a premature infant with very low birth weight: The Ulm Study. PRAVENTIVES PSYCHOTHERAPEUTISCHES INTERVENTIONSPROGRAMM FUR ELTERN NACH DER GEBURT EINES SEHR KLEINEN FRUHGEBORENEN - ULMER MODELL. RANDOMISIERTE LANGSSCHNITTSTUDIE. Monatsschrift Fur Kinderheilkunde 1996;144:1206-12. Not an exercise intervention study
Ref ID: 4703

(773) Brisch KH, Bechinger D, Betzler S, Heinemann H. Early preventive attachment-oriented psychotherapeutic intervention program with parents of a very low birthweight premature infant: results of attachment and neurological development. Attachment and Human Development 2003;5:120-35. Subjects less than 2 years old
Ref ID: 4704

(774) Brismar T, Maurex L, Cooray G, Juntti-Berggren L, Lindstrom P, Ekberg K, Adner N, Andersson S. Predictors of cognitive impairment in type 1 diabetes. Psychoneuroendocrinology 2007;32(8-10):1041-51. Cross-sectional study
Ref ID: 5181

(775) Brito EC, Vimaleswaran KS, Brage S, Andersen LB, Sardinha LB, Wareham NJ, Ekelund U, Loos RJ, Franks PW. PPARGC1A sequence variation and cardiovascular risk-factor levels: a study of the main genetic effects and gene x environment interactions in children from the European Youth Heart Study. Diabetologia 2009 April;52(4):609-13. Cross-sectional study
Ref ID: 793

(776) Brockmann V, Caussade L, Holmgren P, Prado A, Reyes M, Viviani G, Bertrand N. Actividad física y obesidad en niños con asma. Revista Chilena de Pediatría 2007 October;78(5):482-8. Not an exercise intervention study
Ref ID: 4010

(777) Brodersen NH, Steptoe A, Boniface DR, Wardle J. Trends in physical activity and sedentary behaviour in adolescence: ethnic and socioeconomic differences. British Journal of Sports Medicine 2007 March;41(3):140-4. Cohort Study
Ref ID: 1265

(778) Broeder CE, Burrhus KA, Svanevik LS, Wilmore JH. The effects of either high-intensity resistance or endurance training on resting metabolic rate. American

Journal of Clinical Nutrition 1992 April;55(4):802-10. Study limited to adults
Ref ID: 2290

(779) Brondel L, Romer MA, Nougues PM, Touyarou P, Davenne D. Acute partial sleep deprivation increases food intake in healthy men. Am J Clin Nutr 2010 June;91(6):1550-9. Not an exercise intervention study
Ref ID: 52

(780) Bronhara B, Vieira VCR. Proporcionalidade corporal na avaliação antropométrica de adolescentes pós-menarca. Revista de Nutrição 2007 February;20(1):27-37. Cross-sectional study
Ref ID: 4011

(781) Bronner YL, Paige DM. Current Concepts in Infant Nutrition. Journal of Nurse-Midwifery 1992;37(2):S43-S58. Review article
Ref ID: 5182

(782) Brosnan M, Walker I. A Preliminary Investigation into the Potential Role of Waist Hip Ratio (WHR) Preference within the Assortative Mating Hypothesis of Autistic Spectrum Disorders. Journal of Autism and Developmental Disorders 2009 January;39(1):164-71. Not an exercise intervention study
Ref ID: 3808

(783) Brotman LM, Dawson-McClure S, Huang KY, Theise R, Kamboukos D, Wang J, Petkova E, Ogedegbe G. Early childhood family intervention and long-term obesity prevention among high-risk minority youth. Pediatrics 2012 March;129(3):e621-e628. Not an exercise intervention study
Ref ID: 2432

(784) Broussard JL, Ehrmann DA, Van CE, Tasali E, Brady MJ. Impaired insulin signaling in human adipocytes after experimental sleep restriction: a randomized, crossover study. Annals of Internal Medicine 2012 October 16;157(8):549-57. Inappropriate Population
Ref ID: 5780

(785) Brown B, Noonan C, Harris KJ, Parker M, Gaskill S, Ricci C, Cobbs G, Gress S. Developing and piloting the Journey to Native Youth Health program in Northern Plains Indian communities. Diabetes Educ 2013 January;39(1):109-18. Inappropriate Intervention
Ref ID: 5781

(786) Brown BD, Harris KJ, Harris JL, Parker M, Ricci C, Noonan C. Translating the diabetes prevention program for Northern Plains Indian youth through community-based participatory research methods. Diabetes Educator 2010 November;36(6):924-35. Not an exercise intervention study
Ref ID: 1084

(787) Brown HE, Pearson N, Braithwaite RE, Brown WJ, Biddle SJ. Physical activity interventions and depression in children and adolescents : a systematic review and meta-analysis. Sports Med 2013 March;43(3):195-206. Inappropriate Study Design
Ref ID: 5782

(788) Brown KH, López-de RD, Arsenault JE, Peerson JM, Penny ME. Comparison of the effects of zinc delivered in a fortified food or a liquid supplement on the growth, morbidity, and plasma zinc concentrations of young Peruvian children. American Journal of Clinical Nutrition 2007;85:538-47. Diet Intervention or Supplement Study
Ref ID: 4705

(789) Brown LD, Heermann JA. The effect of developmental care on preterm infant outcome. Applied nursing research : ANR 1997;10:190-7. Retrospective study
Ref ID: 952

(790) Brown NA, Jensen JL. The development of contact force construction in the dynamic-contact task of cycling [corrected].[Erratum appears in J Biomech. 2003 Apr;36(4):619]. Journal of Biomechanics 2003 January;36(1):1-8. Not a randomized controlled trial (RCT)
Ref ID: 1781

(791) Brown T, Avenell A, Edmunds LD, Moore H, Whittaker V, Avery L, Summerbell C. Systematic review of long-term lifestyle interventions to prevent weight gain and morbidity in adults. [Review] [68 refs]. Obesity Reviews 2009 November;10(6):627-38. Review article
Ref ID: 652

(792) Brownell KD, Kelman JH, Stunkard AJ. Treatment of obese children with and without their mothers: changes in weight and blood pressure. Pediatrics 1983 April;71(4):515-23. Lifestyle Intervention
Ref ID: 2367

(793) Brownson RC, Chriqui JF, Burgeson CR, Fisher MC, Ness RB. Translating Epidemiology Into Policy to Prevent Childhood Obesity: The Case for Promoting Physical Activity in School Settings. Annals of Epidemiology 2010;20(6):436-44. Review article
Ref ID: 5183

(794) Brunstrom JM, Mitchell GL. Flavor-nutrient learning in restrained and unrestrained eaters. Physiology and Behavior 2007;90(1):133-41. Diet Intervention or Supplement Study
Ref ID: 5184

(795) Brutsaert TD, Spielvogel H, Soria R, Caceres E, Buzenet G, Haas JD. Effect of developmental and ancestral high-altitude exposure on VO(2)peak of Andean and European/North American natives. American Journal of Physical

Anthropology 1999;110(4):435-55. Not an exercise intervention study
Ref ID: 5185

(796) Bryner RW, Toffle RC, Ullrich IH, Yeater RA. The effects of exercise intensity on body composition, weight loss, and dietary composition in women. Journal of the American College of Nutrition 1997 February;16(1):68-73. Study limited to adults
Ref ID: 2147

(797) Brzycki M. Pills, Powders & potions. Coach and Athletic Director 2007 March;76(8):63-5. Review article
Ref ID: 3809

(798) Buchan DS, Ollis S, Thomas NE, Baker JS. The influence of a high intensity physical activity intervention on a selection of health related outcomes: an ecological approach. BMC Public Health 2010;10:8. Description of study from review or magazine or etc. (not the actual study)
Ref ID: 575

(799) Buchan DS, Ollis S, Thomas NE, Buchanan N, Cooper SM, Malina RM, Baker JS. Physical activity interventions: effects of duration and intensity. Scandinavian Journal of Medicine and Science in Sports 2011 December;21(6):e341-e350. Not All Participants were Overweight and/or Obese
Ref ID: 2434

(800) Buchan DS, Ollis S, Young JD, Cooper SM, Shield JP, Baker JS. High intensity interval running enhances measures of physical fitness but not metabolic measures of cardiovascular disease risk in healthy adolescents. BMC Public Health 2013;13:498. Inappropriate Population
Ref ID: 5783

(801) Buchheit M, Horobeanu C, Mendez-Villanueva A, Simpson BM, Bourdon PC. Effects of age and spa treatment on match running performance over two consecutive games in highly trained young soccer players. Journal of Sports Sciences 2011 March 15;29(6):591-8. Acute study
Ref ID: 3885

(802) Buckley JD, Brinkworth GD, Abbott MJ. Effect of bovine colostrum on anaerobic exercise performance and plasma insulin-like growth factor I. Journal of Sports Sciences 2003 July;21(7):577-88. Diet Intervention or Supplement Study
Ref ID: 1734

(803) Buckley JM, Souhrada JF. A comparison of pulmonary function tests in detecting exercise-induced bronchoconstriction. Pediatrics 1975 November;56(5:pt-2 suppl):t-2. Acute study
Ref ID: 2392

(804) Bueno N, Fletcher BJ, Fletcher GF, Serra S, Cruz PM, Kelly D, Meirelles L, Atkinson E, Tabor LA, Ramos A, Castro I. Coronary risk factors in adult children of parents with coronary heart disease: a comparison survey of southeastern Brazil and southeastern United States. Preventive Cardiology 2005;8(3):149-54. Survey or questionnaire
Ref ID: 1493

(805) Bueno O, Bueno G, Moreno LA, Nuviala RJ, Perez-Gonzalez JM, Bueno M. Zinc supplementation in infants with asymmetric intra uterine growth retardation; effect on growth, nutritional status and leptin secretion. Nutricion Hospitalaria 2008 May;23(3):212-9. Diet Intervention or Supplement Study
Ref ID: 3539

(806) Bueno VC, Lombardi Júnior I, Medeiros WM, Azevedo MMA, Len CA, Terreri MT, Natour J, Hilário MOE. Reabilitação em artrite idiopática juvenil. Revista Brasileira de Reumatologia 2007 June;47(3):197-203. Review article
Ref ID: 4012

(807) Buff CdG, Ramos E, Souza FI, Sarni RO. Freqüência de síndrome metabólica em crianças e adolescentes com sobrepeso e obesidade. Frequency of metabolic syndrome in overweight and obese children and adolescents. Revista Paulista de Pediatria 2007 September;25(3):221-6. Cross-sectional study
Ref ID: 4013

(808) Buffart LM, van den Berg-Emons RJ, van Wijlen-Hempel MS, Stam HJ, Roebroeck ME. Health-related physical fitness of adolescents and young adults with myelomeningocele. European Journal of Applied Physiology 2008 May;103(2):181-8. Cross-sectional study
Ref ID: 960

(809) Buhring B, Oliva M, Bravo C. Determinación no experimental de la conducta sedentaria en escolares. Revista Chilena de Nutrición 2009 March;36(1):23-30. Review article
Ref ID: 4014

(810) Bullock N, Comfort P. An investigation into the acute effects of depth jumps on maximal strength performance. Journal of Strength and Conditioning Research 2011 November;25(11):3137-41. Acute study
Ref ID: 2435

(811) Bundy AC, Naughton G, Tranter P, Wyver S, Baur L, Schiller W, Bauman A, Engelen L, Ragen J, Luckett T, Niehues A, Stewart G, Jessup G, Brentnall J. The Sydney playground project: popping the bubblewrap--unleashing the power of play: a cluster randomized controlled trial of a primary school playground-based intervention aiming to increase children's physical activity and social skills. BMC Public Health 2011;11:680. Description of study from review or

magazine or etc. (not the actual study)
Ref ID: 2436

(812) Buntain HM, Greer RM, Schluter PJ, Wong JC, Batch JA, Potter JM, Lewindon PJ, Powell E, Wainwright CE, Bell SC. Bone mineral density in Australian children, adolescents and adults with cystic fibrosis: a controlled cross sectional study. Thorax 2004 February;59(2):149-55. Cross-sectional study
Ref ID: 1669

(813) Buonani C, Fernandes RA, Silveira LS, Bastos KdN, Monteiro PA, Viotto Filho I, Júnior F. Prevenção da síndrome metabólica em crianças obesas: uma proposta de intervenção. Prevention of metabolic syndrome in obese children: a proposal of intervention. Revista Paulista de Pediatria 2011 June;29(2):186-92. Not a randomized controlled trial (RCT)
Ref ID: 4015

(814) Burbano JC, Fornasini M, Acosta M. Prevalencia y factores de riesgo de sobrepeso en colegialas de 12 a 19 años en una región semiurbana del Ecuador. Revista Panamericana de Salud Pública 2003 May;13(5):277-84. Cross-sectional study
Ref ID: 4017

(815) Burbano JC, Fornasini M, Acosta M. Fe de errata: prevalencia y factores de riesgo de sobrepeso en colegialas de 12 a 19 años en una región semiurbana del Ecuador. Revista Panamericana de Salud Pública 2003 August;14(2):96. Cross-sectional study
Ref ID: 4016

(816) Burdette HL, Wadden TA, Whitaker RC. Neighborhood safety, collective efficacy, and obesity in women with young children. Obesity 2006;14(3):518-25. Cross-sectional study
Ref ID: 5186

(817) Burgert TS, Duran EJ, Goldberg-Gell R, Dziura J, Yeckel CW, Katz S, Tamborlane WV, Caprio S. Short-term metabolic and cardiovascular effects of metformin in markedly obese adolescents with normal glucose tolerance. Pediatric Diabetes 2008 December;9(6):567-76. No exercise only group, Drug intervention study
Ref ID: 159

(818) Burgos MS, Reuter CP, Burgos LT, Pohl HH, Pauli LTS, Horta JA, Reckziegel MB, Franke SIR, Prá D, Camargo M. Uma análise entre índices pressóricos, obesidade e capacidade cardiorrespiratória em escolares. Comparison analysis of blood pressure, obesity, and cardio-respiratory fitness in schoolchildren. Arq bras cardiol 2010 June;94(6):788-93. Cross-sectional study
Ref ID: 4018

(819) Burk A, Timpmann S, Medijainen L, Vahi M, Oopik V. Time-divided ingestion pattern of casein-based protein supplement stimulates an increase in fat-free body mass during resistance training in young untrained men. Nutrition Research 2009 June;29(6):405-13. Diet Intervention or Supplement Study
Ref ID: 706

(820) Burke DG, Smith-Palmer T, Holt LE, Head B, Chilibeck PD. The effect of 7 days of creatine supplementation on 24-hour urinary creatine excretion. Journal of Strength and Conditioning Research 15(1):59-62, 2001 Feb 2001;(1):59-62. Study limited to adults
Ref ID: 2902

(821) Burke DG, Chilibeck PD, Parise G, Tarnopolsky MA, Candow DG. Effect of alpha-lipoic acid combined with creatine monohydrate on human skeletal muscle creatine and phosphagen concentration. International Journal of Sport Nutrition and Exercise Metabolism 2003 September;13(3):294-302. Diet Intervention or Supplement Study
Ref ID: 1690

(822) Burke LE, Warziski M, Styn MA, Music E, Hudson AG, Sereika SM. A randomized clinical trial of a standard versus vegetarian diet for weight loss: the impact of treatment preference. International Journal of Obesity (London) 2008 January;32(1):166-76. Study limited to adults
Ref ID: 222

(823) Burke LE, Conroy MB, Sereika SM, Elci OU, Styn MA, Acharya SD, Sevick MA, Ewing LJ, Glanz K. The effect of electronic self-monitoring on weight loss and dietary intake: a randomized behavioral weight loss trial. Obesity 2011 February;19(2):338-44. Study limited to adults
Ref ID: 1061

(824) Burke V, Beilin LJ, Milligan R, Thompson C. Assessment of nutrition and physical activity education programmes in children. [Review] [33 refs]. Clinical and Experimental Pharmacology and Physiology 1995 March;22(3):212-6. Review article
Ref ID: 2199

(825) Burke V, Thompson C, Taggart AC, Spickett EE, Beilin LJ, Vandongen R, Milligan RA, Dunbar DL. Differences in response to nutrition and fitness education programmes in relation to baseline levels of cardiovascular risk in 10 to 12-year-old children. Journal of Human Hypertension 1996 September;10:Suppl-106. Not All Participants were Overweight and/or Obese
Ref ID: 2159

(826) Burke V, Milligan RA, Thompson C, Taggart AC, Dunbar DL, Spencer MJ, Medland A, Gracey MP, Vandongen R, Beilin LJ. A controlled trial of health promotion programs in 11-year-olds using physical activity "enrichment" for

higher risk children. Journal of Pediatrics 1998 May;132(5):840-8. Not All Participants were Overweight and/or Obese
Ref ID: 2091

(827) Burke V, Beilin LJ, Dunbar D. Family lifestyle and parental body mass index as predictors of body mass index in Australian children: a longitudinal study. International Journal of Obesity 2001;25(2):147-57. Not a randomized controlled trial (RCT)
Ref ID: 5187

(828) Burke V, Giangiulio N, Gillam HF, Beilin LJ, Houghton S. Physical activity and nutrition programs for couples: a randomized controlled trial. J Clin Epidemiol 2003 May;56(5):421-32. Study limited to adults
Ref ID: 360

(829) Burke V, Beilin LJ, Durkin K, Stritzke WG, Houghton S, Cameron CA. Television, computer use, physical activity, diet and fatness in Australian adolescents. International Journal of Pediatric Obesity 2006;1(4):248-55. Cross-sectional study
Ref ID: 1298

(830) Burman P, Ritzen EM, Lindgren AC. Endocrine dysfunction in Prader-Willi syndrome: a review with special reference to GH. [Review] [119 refs]. Endocrine Reviews 2001 December;22(6):787-99. Review article
Ref ID: 1869

(831) Burnier D, Dubois L, Girard M. Arguments at Mealtime and Child Energy Intake. Journal Of Nutrition Education And Behavior 2011;43(6):473-81. Cross-sectional study
Ref ID: 5188

(832) Burns JS, Williams PL, Sergeyev O, Korrick SA, Lee MM, Revich B, Altshul L, Del Prato JT, Humblet O, Patterson DG, Turner WE, Starovoytov M, Hauser R. Serum Concentrations of Organochlorine Pesticides and Growth among Russian Boys. Environmental Health Perspectives 2012;120(2):303-8. Cohort Study
Ref ID: 5189

(833) Burns SF, Corrie H, Holder E, Nightingale T, Stensel DJ. A single session of resistance exercise does not reduce postprandial lipaemia. Journal of Sports Sciences 2005 March;23(3):251-60. Acute study
Ref ID: 1501

(834) Burns SF, Oo HH, Tran AT. Effect of sprint interval exercise on postexercise metabolism and blood pressure in adolescents. International Journal of Sport Nutrition and Exercise Metabolism 2012 February;22(1):47-54. No control group (NC)
Ref ID: 2438

(835) Burr JF, Jamnik VK, Gledhill N. Physiological fitness and health adaptations from purposeful training using off-road vehicles. European Journal of Applied Physiology 2011 August;111(8):1841-50. Not an exercise intervention study
Ref ID: 2439

(836) Burri BJ, Neidlinger TR, Clifford AJ. Serum carotenoid depletion follows first-order kinetics in healthy adult women fed naturally low carotenoid diets. Journal of Nutrition 2001 August;131(8):2096-100. Diet Intervention or Supplement Study
Ref ID: 1903

(837) Burrows Argote R. Prevención, diagnóstico y tratamiento de la obesidad infantil y juvenil: recomendaciones actuales. Revista Chilena de Nutrición 2000 April;27(1):31-5. Review article
Ref ID: 4020

(838) Burrows Argote R, Burgueño A, Díaz Bustos E, Gattás Z, SALAS A, De la Maza C. Protocolos de tratamiento. In: Albala Brevis C, Kain B, Burrows Argote R, Díaz Bustos E, editors. Obesidad: un desafío pendiente.Santiago de Chile: Universitaria; 2000. p. 221-8. Not a randomized controlled trial (RCT)
Ref ID: 4019

(839) Burrows Argote R. Prevención y tratamiento de la obesidad desde la niñez: la estrategia para disminuir las enfermedades crónicas no transmisibles del adulto. Revista médica de Chile 2000 January;128(1):105-10. Review article
Ref ID: 4021

(840) Burrows Argote R, GATTAS Z, Leiva B, Barrera A, Burgueño A. Características biológicas, familiares y metabólicas de la obesidad infantil y juvenil. Revista médica de Chile 2001 October;129(10):1155-62. Retrospective study
Ref ID: 4022

(841) Burrows A, Díaz B, Sciaraffia M, GATTAS Z, Montoya C, Lera M. Hábitos de ingesta y actividad física en escolares, según tipo de establecimiento al que asisten. Revista médica de Chile 2008 January;136(1):53-63. Survey or questionnaire
Ref ID: 4023

(842) Burrows R, Ceballos X, Burgueno M, Muzzo S. Trends in puberal development of school age children living in the Metropolitan Region of Chile. Revista Medica de Chile 2010;138(1):61-7. Cohort Study
Ref ID: 5190

(843) Burrows T, Warren JM, Baur LA, Collins CE. Impact of a child obesity intervention on dietary intake and behaviors. International Journal of Obesity (London) 2008 October;32(10):1481-8. No comparative control group
Ref ID: 174

(844) Burrows T, Warren JM, Collins CE. The impact of a child obesity treatment intervention on parent child-feeding practices. International Journal of Pediatric Obesity 2010;5(1):43-50. No comparative control group
Ref ID: 119

(845) Burton E, Stice E. Evaluation of a healthy-weight treatment program for bulimia nervosa: a preliminary randomized trial. Behaviour Research and Therapy 2006 December;44(12):1727-38. Diet Intervention or Supplement Study
Ref ID: 1338

(846) Burtscher M, Gatterer H, Faulhaber M, Gerstgrasser W, Schenk K. Effects of intermittent hypoxia on running economy. International Journal of Sports Medicine 2010 September;31(9):644-50. Not All Participants were Overweight and/or Obese
Ref ID: 2440

(847) Bush CL, Pittman S, McKay S, Ortiz T, Wong WW, Klish WJ. Park-based obesity intervention program for inner-city minority children. Journal of Pediatrics 517;151(5):513-7. Diet & Exercise intervention
Ref ID: 1148

(848) Busnello FM, Bodanese LC, Pellanda LC, Santos ZE. Nutritional intervention and the impact on adherence to treatment in patients with metabolic syndrome. Arq Bras Cardiol 2011 September;97(3):217-24. Diet Intervention or Supplement Study
Ref ID: 2441

(849) Bussau VA, Fairchild TJ, Rao A, Steele P, Fournier PA. Carbohydrate loading in human muscle: an improved 1 day protocol. European Journal of Applied Physiology 2002 July;87(3):290-5. Diet Intervention or Supplement Study
Ref ID: 1810

(850) Bustamante Valdivia A, Seabra AFT, Silva RMGd, Maia JAR. Efectos de la actividad física y del nivel socioecómico en el sobrepeso y obesidad de escolares, Lima Este 2005. Revista Peruana de Medicina Experimental y Salud Publica 2007 June;24(2):121-8. Survey or questionnaire
Ref ID: 4025

(851) Bustamante C, Ulloa R, Melo C, Muñoz M, Sanhueza X. Evaluaciones de aptitud laboral para trabajos en gran altitud. Bol Cient Asoc Chil Segur 2000 December;2(4):62-5. Not an exercise intervention study
Ref ID: 4026

(852) Butler D. Science of dieting: slim pickings. Nature 2004 March 18;428(6980):252-4. Diet Intervention or Supplement Study
Ref ID: 1659

(853) Butte NE, Wong WW, Adolph AL, Puyau MR, Vohra FA, Zakeri IF. Validation of Cross-Sectional Time Series and Multivariate Adaptive Regression Splines Models for the Prediction of Energy Expenditure in Children and Adolescents Using Doubly Labeled Water. Journal of Nutrition 2010;140(8):1516-23. Not an exercise intervention study
Ref ID: 5191

(854) Butte NF, Wong WW, Hopkinson JM, Heinz CJ, Mehta NR, Smith EO. Energy requirements derived from total energy expenditure and energy deposition during the first 2 y of life. American Journal of Clinical Nutrition 2000 December;72(6):1558-69. Subjects less than 2 years old
Ref ID: 1947

(855) Butte NF, Christiansen E, Sorensen TIA. Energy imbalance underlying the development of childhood obesity. Obesity 2007;15(12):3056-66. Not an exercise intervention study
Ref ID: 5192

(856) Buyken AE, Goletzke J, Joslowski G, Felbick A, Cheng G, Herder C, Brand-Miller JC. Association between carbohydrate quality and inflammatory markers: systematic review of observational and interventional studies. Am J Clin Nutr 2014 April;99(4):813-33. Inappropriate Study Design
Ref ID: 5784

(857) Buzi F, Corna A, Pilotta A, Negrini F, Lombardi A, Re T, Ambrosi B. Loperamide test: a simple and highly specific screening test for hypercortisolism in children and adolescents. Acta Paediatrica 1997 November;86(11):1177-80. Drug intervention study
Ref ID: 2119

(858) Byrd-Williams CE, Belcher BR, Spruijt-Metz D, Davis JN, Ventura EE, Kelly L, Berhane K, Azen S, Goran MI. Increased physical activity and reduced adiposity in overweight Hispanic adolescents. Med Sci Sports Exerc 2010 March;42(3):478-84. No exercise only group, No comparative control group
Ref ID: 76

(859) Byrne NM, Hills AP, Hunter GR, Weinsier RL, Schutz Y. Metabolic equivalent: one size does not fit all. Journal of Applied Physiology 2005 September;99(3):1112-9. Study limited to adults
Ref ID: 1484

(860) Caan BJ, Flatt SW, Rock CL, Ritenbaugh C, Newman V, Pierce JP. Low-energy reporting in women at risk for breast cancer recurrence. Women's Healthy Eating and Living Group. Cancer Epidemiology, Biomarkers and Prevention 2000 October;9(10):1091-7. Not an exercise intervention study
Ref ID: 1954

(861) Caballero B, Clay T, Davis SM, Ethelbah B, Rock BH, Lohman T, Norman J, Story M, Stone EJ, Stephenson L, Stevens J. Pathways: a school-based, randomized controlled trial for the prevention of obesity in American Indian schoolchildren. Am J Clin Nutr 2003 November;78(5):1030-8. No exercise only group
Ref ID: 351

(862) Caballero C, Hernández B, Moreno H, Hernández-Girón C, Campero L, Cruz A, Lazcano-Ponce E. Obesidad, actividad e inactividad física en adolescentes de Morelos, México: un estudio longitudinal. Archivos Latinoamericanos de Nutrición 2007 September;57(3):231-7. Cohort Study
Ref ID: 4027

(863) Cabral Sd, Barbosa FP, Cabral BG, Knackfuss MI, Medeiros HJ, Fernandes Filho J. A seleção brasileira de voleibol infanto-juvenil feminina e o seu perfil dermatoglífico. Acta Cirurgica Brasileira 2005;20(supl.1):22-6. No control group (NC)
Ref ID: 4028

(864) Cade WT, Reeds DN, Mondy KE, Overton ET, Grassino J, Tucker S, Bopp C, Laciny E, Hubert S, Lassa-Claxton S, Yarasheski KE. Yoga lifestyle intervention reduces blood pressure in HIV-infected adults with cardiovascular disease risk factors. HIV Medicine 2010 July 1;11(6):379-88. Study limited to adults
Ref ID: 488

(865) Cadepond P, Ulmann MD, Baulieu MD. RU486 (MIFEPRISTONE): Mechanisms of Action and Clinical Uses. Annual Review of Medicine 1997 February;48(1):129. Review article
Ref ID: 3540

(866) Caggiani Malzone M, Farré Silva Y. Consenso Uruguayo de Hipertensión Arterial en el Niño y el Adolescente. Arch pediatr Urug 2006 October;77(3):300-7. Review article
Ref ID: 4029

(867) Cagnacci A, Cannoletta M, Volpe A. High-dose short-term folate administration modifies ambulatory blood pressure in postmenopausal women. A placebo-controlled study. European Journal of Clinical Nutrition 2009 October;63(10):1266-8. Study limited to adults
Ref ID: 3541

(868) Cagney KA, Browning CR. Exploring neighborhood-level variation in asthma and other respiratory diseases - The contribution of neighborhood social context. Journal of General Internal Medicine 2004;19(3):229-36. Survey or questionnaire
Ref ID: 5193

(869) Cai GW, Cole SA, Butte N, Bacino C, Diego V, Tan K, Goring HH, O'Rahilly S, Farooqi IS, Comuzzie AG. A quantitative trait locus on chromosome 18q for physical activity and dietary intake in Hispanic children. Obesity 2006;14(9):1596-604. Not an exercise intervention study
Ref ID: 5194

(870) Cai L, Wu Y, Wilson RF, Segal JB, Kim MT, Wang Y. Effect of childhood obesity prevention programs on blood pressure: a systematic review and meta-analysis. Circulation 2014 May 6;129(18):1832-9. Inappropriate Study Design
Ref ID: 5785

(871) Cain PA, Ahl R, Hedstrom E, Ugander M, Allansdotter-Johnsson A, Friberg P, Marild S, Arheden H. Physiological determinants of the variation in left ventricular mass from early adolescence to late adulthood in healthy subjects.[Erratum appears in Clin Physiol Funct Imaging. 2007 Jul;27(4):254], [Republished in Clin Physiol Funct Imaging. 2007 Jul;27(4):255-62; PMID: 17564676]. Clinical Physiology and Functional Imaging 2005 November;25(6):332-9. Not an exercise intervention study
Ref ID: 1456

(872) Calanas-Continente A, Arrizabalaga JJ, Caixas A, Cuatrecasas G, Diaz-Fernandez MJ, Garcia-Luna PP, Goday A, Masmiquel L, Monereo S, Morales MJ, Moreiro J, Moreno B, Ricart W, Vidal J, Cordido F. Strategies for treating overweight in adolescents and their families. Endocrinology and Nutrition 2008;55(SUPPL. 4):60-77. Review article
Ref ID: 3200

(873) Calabro MA, Stewart JM, Welk GJ. Validation of pattern-recognition monitors in children using doubly labeled water. Medicine and Science in Sports and Exercise 2013 July;45(7):1313-22. Inappropriate Outcomes
Ref ID: 5786

(874) Calders P, Elmahgoub S, de Mettelinge TR, Vandenbroeck C, Dewandele I, Rombaut L, Vandevelde A, Cambier D. Effect of combined exercise training on physical and metabolic fitness in adults with intellectual disability: a controlled trial. Clinical Rehabilitation 25(12):1097-108, 2011 Dec 2011;(12):1097-108. Study limited to adults
Ref ID: 2906

(875) Caliandro P, Grugni G, Padua L, Kodra Y, Tonali P, Gargantini L, Ragusa L, Crino A, Taruscio D. Quality of life assessment in a sample of patients affected by Prader-Willi syndrome. Journal of Paediatrics and Child Health 2007 December;43(12):826-30. Survey or questionnaire
Ref ID: 1137

(876) Calles-Escandon J, Arciero PJ, Gardner AW, Bauman C, Poehlman ET. Basal fat oxidation decreases with aging in women. Journal of Applied Physiology

1995 January;78(1):266-71. Study limited to adults
Ref ID: 2214

(877) Calvin AD, Carter RE, Adachi T, Macedo PG, Albuquerque FN, van der Walt C, Bukartyk J, Davison DE, Levine JA, Somers VK. Effects of experimental sleep restriction on caloric intake and activity energy expenditure. Chest 2013 July;144(1):79-86. Inappropriate Outcomes
Ref ID: 5787

(878) Camargo Lemos DM. Factores asociados a la calidad de vida en adolescentes de Bucaramanga. Factors associated to living standards for teenagers in Bucaramanga. Review Universidad Industrial de Santander, Salud 2009 April 30;41(1):33-42. Cross-sectional study
Ref ID: 4030

(879) Cameron AJ, Ball K, Hesketh KD, McNaughton SA, Salmon J, Crawford DA, Lioret S, Campbell KJ. Variation in outcomes of the Melbourne Infant, Feeding, Activity and Nutrition Trial (InFANT) Program according to maternal education and age. Preventive Medicine 2014 January;58:58-63. Inappropriate Population
Ref ID: 5788

(880) Cameron EC, Maehle V, Reid J. The effects of an early physical therapy intervention for very preterm, very low birth weight infants: a randomized controlled clinical trial. Pediatric Physical Therapy 2005;17:107-19. Subjects less than 2 years old
Ref ID: 4706

(881) Cameron JW. Self-esteem changes in children enrolled in weight management programs. Issues in Comprehensive Pediatric Nursing 22(2-3):75-85, 1999 Apr-Sep 1999;(2-3):75-85. Survey or questionnaire
Ref ID: 2907

(882) Campbell-Yeo ML, Allen AC, Joseph KS, Ledwidge JM, Allen VM, Dooley KC. Study protocol: A double blind placebo controlled trial examining the effect of domperidone on the composition of breast milk [NCT00308334]. BMC Pregnancy and Childbirth 2006;6:17. Drug intervention study
Ref ID: 4707

(883) Campbell DD, Meckling KA. Effect of the protein:carbohydrate ratio in hypoenergetic diets on metabolic syndrome risk factors in exercising overweight and obese women. British Journal of Nutrition 2012 November 14;108(9):1658-71. Inappropriate Population
Ref ID: 5789

(884) Campbell K, Waters E, O'Meara S, Summerbell C. Interventions for preventing obesity in childhood. A systematic review. [Review] [31 refs]. Obesity Reviews 2001 August;2(3):149-57. Review article
Ref ID: 1862

(885) Campbell K, Hesketh K, Crawford D, Salmon J, Ball K, McCallum Z. The Infant Feeding Activity and Nutrition Trial (INFANT) an early intervention to prevent childhood obesity: cluster-randomised controlled trial. BMC Public Health 2008 March 31;8:103.:103. Description versus conduct of study
Ref ID: 187

(886) Campbell KJ, Lioret S, McNaughton SA, Crawford DA, Salmon J, Ball K, McCallum Z, Gerner BE, Spence AC, Cameron AJ, Hnatiuk JA, Ukoumunne OC, Gold L, Abbott G, Hesketh KD. A parent-focused intervention to reduce infant obesity risk behaviors: a randomized trial. Pediatrics 2013 April;131(4):652-60. Inappropriate Population
Ref ID: 5790

(887) Campbell ML. Administering Child Protection - A feminist analysis of the conceptual practices of organizational power. Canadian Public Administration-Administration Publique du Canada 1992;35(4):501-18. Case-Control / Case Study
Ref ID: 5195

(888) Campbell VS, Sinha DP, Patterson AW. KAP study on project lifestyle (Jamaica). Cajanus 1992;25(1):25-48. Survey or questionnaire
Ref ID: 698

(889) Campo Osaba MA, Del Val JL, Lapena C, Laguna V, Garcia A, Lozano O, Martin Z, Rodriguez R, Borras E, Orfila F, Tierno MT. The effectiveness of a health promotion with group intervention by clinical trial. Study protocol. BMC Public Health 2012;12:209. Description of study from review or magazine or etc. (not the actual study)
Ref ID: 2443

(890) Campos Cavada I. Factores de riesgo modificables para enfermedad cardiovascular en niñosies Modifiable risk factors for cardiovascular diseases in children. Anales venezolanos de nutrición 2010 December;23(2):100-7. Review article
Ref ID: 4031

(891) Campos Wd, Stabelini Neto A, Bozza R, Ulbrich AZ, Bertin RL, Mascarenhas LPG, Silva SGd, Sasaki JE. Atividade física, consumo de lipídios e fatores de risco para aterosclerose em adolescentes. Physical activity, lipid consumption and risk factors for atherosclerosis in adolescents. Arquivos Brasileiros de Cardiologia 2010 May;94(5):601-7. Cross-sectional study
Ref ID: 4032

(892) Can F, Yilmaz I, Erden Z. Morphological characteristics and performance variables of women soccer players. Journal of Strength and Conditioning Research 2004 August;18(3):480-5. Not an exercise intervention study
Ref ID: 1599

(893) Candow DG, Burke NC, Smith-Palmer T, Burke DG. Effect of whey and soy protein supplementation combined with resistance training in young adults. International Journal of Sport Nutrition and Exercise Metabolism 2006 June;16(3):233-44. Diet Intervention or Supplement Study
Ref ID: 1353

(894) Canessa E. Modeling of body mass index by Newton's second law. Journal of Theoretical Biology 2007;248(4):646-56. Not an exercise intervention study
Ref ID: 5196

(895) Canessa F, Santullo B, López A. Rendimiento en la carrera de 30 metros en niños sanos de 3 a 4 años. Kinesiologia 2002 March;(66):16-20. Not an exercise intervention study
Ref ID: 745

(896) Canhadas IL, Silva RLP, Chaves CR, Portes LA. Anthropometric and physical fitness characteristics of young male soccer players. Revista Brasileira de Cineantropometria e Desempenho Humano 2010 August;12(4). Cross-sectional study
Ref ID: 4033

(897) Canuto KJ, McDermott RA, Cargo M, Esterman AJ. Study protocol: a pragmatic randomised controlled trial of a 12-week physical activity and nutritional education program for overweight Aboriginal and Torres Strait Islander women. BMC Public Health 2011;11:655. Study limited to adults
Ref ID: 1052

(898) Capozza RF, Cointry GR, Cure-Ramirez P, Ferretti JL, Cure-Cure C. A DXA study of muscle-bone relationships in the whole body and limbs of 2512 normal men and pre- and post-menopausal women. Bone 2004;35(1):283-95. Study limited to adults
Ref ID: 5197

(899) Caranti DA, de Mello MT, Prado WL, Tock L, Siqueira KO, de PA, Lofrano MC, Cristofalo DM, Lederman H, Tufik S, Damaso AR. Short- and long-term beneficial effects of a multidisciplinary therapy for the control of metabolic syndrome in obese adolescents. Metabolism: Clinical and Experimental 2007 September;56(9):1293-300. Lifestyle Intervention, Not a randomized controlled trial (RCT)
Ref ID: 1182

(900) Cardon G, De B, I, De CD. Knowledge and perceptions about back education among elementary school students, teachers, and parents in Belgium. The Journal of School Health 2002;72:100-6. Educational intervention
Ref ID: 4708

(901) Cardon G, Labarque V, Smits D, De B, I. Promoting physical activity at the pre-school playground: the effects of providing markings and play equipment.

Preventive Medicine 2009;48:335-40. Not All Participants were Overweight and/or Obese
Ref ID: 4709

(902) Cardoso AT, Oliveira YRDd, Carvalho Jd, Nahas MV. Efeitos da atividade física de baixo teor metabólico sobre alguns componentes corporais: % G, Gord. Total e massa metabólica magra. Revista Brasileira de Ciências do Esporte 1987 May;8(2/3):163-5. Study limited to adults
Ref ID: 4034

(903) Cardoso LD, de Castro IRR, Gomes FD, Leite ID. Individual and school environment factors associated with overweight in adolescents of the municipality of Rio de Janeiro, Brazil. Public Health Nutrition 2011;14(5):914-22. Cross-sectional study
Ref ID: 5198

(904) Carei TR, Fyfe-Johnson AL, Breuner CC, Brown MA. Randomized controlled clinical trial of yoga in the treatment of eating disorders. Journal of Adolescent Health 2010 April;46(4):346-51. Not All Participants were Overweight and/or Obese
Ref ID: 566

(905) Carey VJ, Yong FH, Frenkel LM, McKinney RE. Pediatric AIDS prognosis using somatic growth velocity. Aids 1998;12(11):1361-9. Not an exercise intervention study
Ref ID: 5199

(906) Carletti L, Rodrigues AN, Perez AJ, Vassallo DV. Resposta da pressão arterial ao esforço em adolescentes: influência do sobrepeso e obesidade. Arquivos Brasileiros de Cardiologia 2008 July;91(1):25-30. Not a randomized controlled trial (RCT)
Ref ID: 4035

(907) Carlo WA, Goudar SS, Jehan I, Chomba E, Tshefu A, Garces A, Parida S, Althabe F, McClure EM, Derman RJ, Goldenberg RL, Bose C, Hambidge M, Panigrahi P, Buekens P, Chakraborty H, Hartwell TD, Moore J, Wright LL, -First-Breath-Study-Group. High mortality rates for very low birth weight infants in developing countries despite training. Pediatrics 2010;126:e1072-e1080. Subjects less than 2 years old
Ref ID: 4710

(908) Carlock JM, Smith SL, Hartman MJ, Morris RT, Ciroslan DA, Pierce KC, Newton RU, Harman EA, Sands WA, Stone MH. The relationship between vertical jump power estimates and weightlifting ability: a field-test approach. Journal of Strength and Conditioning Research 2004 August;18(3):534-9. Acute study
Ref ID: 1596

(909) Carlsen EM, Kyhnaeb A, Renault KM, Cortes D, Michaelsen KF, Pryds O. Telephone-based support prolongs breastfeeding duration in obese women: a randomized trial. American Journal of Clinical Nutrition 2013 November;98(5):1226-32. Inappropriate Population
Ref ID: 5791

(910) Carlson JA, Sallis JF, Ramirez ER, Patrick K, Norman GJ. Physical activity and dietary behavior change in Internet-based weight loss interventions: comparing two multiple-behavior change indices. Preventive Medicine 2012 January;54(1):50-4. Study limited to adults
Ref ID: 1033

(911) Carlson MC, Saczynski JS, Rebok GW, Seeman T, Glass TA, McGill S, Tielsch J, Frick KD, Hill J, Fried LP. Exploring the effects of an "everyday" activity program on executive function and memory in older adults: Experience Corps. The Gerontologist 2008;48:793-801. Study limited to adults
Ref ID: 4711

(912) Carlson SE, Werkman SH, Peeples JM, Cooke RJ, Tolley EA. Arachidonic-acid status correlates with 1st year growth in preterm infants. Proceedings of the National Academy of Sciences of the United States of America 1993;90(3):1073-7. Subjects less than 2 years old
Ref ID: 5200

(913) Carneiro O, Jardim PC. Pressäo arterial em tribo Xavante: comparaçäo 15 anos depois. Arquivos brasileiros de cardiologia 1993 November;61(5):279-82. Study limited to adults
Ref ID: 4036

(914) Carnelosso ML, Barbosa MA, Porto CC, Silva SA, Carvalho MMd, Oliveira ALI. Prevalência de fatores de risco para doenças cardiovasculares na região leste de Goiânia (GO). Prevalence of risk factors for cardiovascular diseases in the east region of Goiânia, Goiás State. Ciência and Saúde Coletiva 2010 June;15(supl.1):1073-80. Cross-sectional study
Ref ID: 4037

(915) Caronia LM, Martin C, Welt CK, Sykiotis GP, Quinton R, Thambundit A, Avbelj M, Dhruvakumar S, Plummer L, Hughes VA, Seminara SB, Boepple PA, Sidis Y, Crowley WF, Martin KA, Hall JE, Pitteloud N. A genetic basis for functional hypothalamic amenorrhea. New England Journal of Medicine 2011;364(3):215-25. Study limited to adults
Ref ID: 5201

(916) Carpenter WH, Poehlman ET, Oconnell M, Goran MI. influence of body-composition and resting metabolic-rate on variation in total-energy expenditure - A Meta-analysis. American Journal of Clinical Nutrition 1995;61(1):4-10.

Review article
Ref ID: 5202

(917) Carpentier MY, Mullins LL, Elkin TD, Wolfe-Christensen C. Prevalence of multiple health-related behaviors in adolescents with cancer. Journal of Pediatric Hematology/Oncology 2008 December;30(12):902-7. Survey or questionnaire
Ref ID: 833

(918) Carr A, Cooper DA. Adverse effects of antiretroviral therapy. Lancet 2000 October 21;356(9239):1423. Review article
Ref ID: 3543

(919) Carrasco F, Moreno M, Irribarra V, Rodríguez L, Martin MA, Alarcón A, Mizón C, Echenique C, Saavedra V, Pizarra T, Atalah E. Evaluación de un programa piloto de intervención en adultos con sobrepeso u obesidad, en riesgo de diabetes. Revista médica de Chile 2008 January;136(1):13-21. Study limited to adults
Ref ID: 4038

(920) Carrasco P, Pérez B, Angel B, Albala B, Santos M, Larenas Y, Montalvo V. Prevalencia de diabetes tipo 2 y obesidad en dos poblaciones aborígenes de Chile en ambiente urbano. Revista médica de Chile 2004 October;132(10):1189-97. Study limited to adults
Ref ID: 4039

(921) Carrel AL, Myers SE, Whitman BY, Allen DB. Growth hormone improves body composition, fat utilization, physical strength and agility, and growth in Prader-Willi syndrome: A controlled study. Journal of Pediatrics 1999 February;134(2):215-21. Drug intervention study
Ref ID: 2050

(922) Carrel AL, Myers SE, Whitman BY, Allen DB. Sustained benefits of growth hormone on body composition, fat utilization, physical strength and agility, and growth in Prader-Willi syndrome are dose-dependent. Journal of Pediatric Endocrinology 2001 September;14(8):1097-105. Drug intervention study
Ref ID: 1890

(923) Carrel AL, Clark RR, Peterson SE, Nemeth BA, Sullivan J, Allen DB. Improvement of fitness, body composition, and insulin sensitivity in overweight children in a school-based exercise program: a randomized, controlled study. Archives of Pediatric Adolescent Medicine 2005 October;159(10):963-8. No exercise only group, Lifestyle Intervention
Ref ID: 298

(924) Carrel AL, Clark RR, Peterson S, Eickhoff J, Allen DB. School-based fitness changes are lost during the summer vacation. Archives of Pediatrics and Adolescent Medicine 2007 June;161(6):561-4. Not an exercise intervention

study
Ref ID: 1224

(925) Carrel AL, McVean JJ, Clark RR, Peterson SE, Eickhoff JC, Allen DB. School-based exercise improves fitness, body composition, insulin sensitivity, and markers of inflammation in non-obese children. Journal of Pediatric Endocrinology 2009 May;22(5):409-15. Not All Participants were Overweight and/or Obese
Ref ID: 716

(926) Carrel AL, Myers SE, Whitman BY, Eickhoff J, Allen DB. Long-term growth hormone therapy changes the natural history of body composition and motor function in children with Prader-Willi Syndrome. Journal of Clinical Endocrinology and Metabolism 2010;95(3):1131-6. Drug intervention study
Ref ID: 5203

(927) Carrel AL. Randomised trial of exercise dose in children reduces diabetes risk (as measured by insulin resistance) in both 20-min and 40-min doses. Evidence Based Medicine 2013 August;18(4):147-8. Inappropriate Study Design
Ref ID: 5792

(928) Carrera F, Ramos K, Velasco CA. Manejo de la obesidad infantil. Revista Gastrohnup 2010 January 15;12(1):S31-S37. Survey or questionnaire
Ref ID: 4040

(929) Carruth BR, Skinner JD. Revisiting the picky eater phenomenon: Neophobic behaviors of young children. Journal of the American College of Nutrition 2000;19(6):771-80. Diet Intervention Study
Ref ID: 5204

(930) Carter FA, Bulik CM. Childhood obesity prevention programs: how do they affect eating pathology and other psychological measures?. [Review] [56 refs]. Psychosomatic Medicine 2008 April;70(3):363-71. Review article
Ref ID: 974

(931) Carter GT, Weiss MD, Chamberlain JR, Han JJ, Abresch RT, MirÃ³ J, Jensen MP. Aging with muscular dystrophy: Pathophysiology and clinical management. Physical Medicine and Rehabilitation Clinics of North America 2010;21(2):429-50. Review article
Ref ID: 3201

(932) Carter NJ, Keating GM. Bosentan: In pediatric patients with pulmonary arterial hypertension. Pediatrics Drugs 2010;12(1):63-73. Drug intervention study
Ref ID: 3202

(933) Caruso-Davis MK, Guillot TS, Podichetty VK, Mashtalir N, Dhurandhar NV, Dubuisson O, Yu Y, Greenway FL. Efficacy of low-level laser therapy for body contouring and spot fat reduction. Obesity Surgery 2011 June;21(6):722-9.

Study limited to adults
Ref ID: 2446

(934) Caruso JF, Signorile JF, Perry AC, Leblanc B, Williams R, Clark M, Bamman MM. The effects of albuterol and isokinetic exercise on the quadriceps muscle group. Medicine and Science in Sports and Exercise 1995 November;27(11):1471-6. Drug intervention study
Ref ID: 2191

(935) Caruso JF, Hamill JL, De GN. Oral albuterol dosing during the latter stages of a resistance exercise program. Journal of Strength and Conditioning Research 2005 February;19(1):102-7. Drug intervention study
Ref ID: 1545

(936) Carvalhaes MA, Martiniano AC, Malta MB, Takito MY, Benicio MH. [Physical activity in pregnant women receiving care in primary health care units]. Revista de Saúde Pública 2013 October;47(5):958-67. Inappropriate Population
Ref ID: 5793

(937) Carvalho Filho G, Chueire AG, Ignácio H, Carneiro MdO, Francese Neto J, Canesin AC. Tratamento cirúrgico de luxação congênita do quadril pós marcha: redução aberta e osteotomia de Salter. Surgical Treatment of the congenital dislocation of the hip after walking age: open reduction and Salter´s osteotomy. Acta Ortopédica Brasileira 2003 March;11(1):42-7. Not an exercise intervention study
Ref ID: 4041

(938) Carvalho JFd, Caleiro MTC. Lipoprotein(a) in primary antiphospholipid syndromeien Lipoproteína(a) na síndrome antifosfolípide primária. Revista Brasileira de Reumatologia 2009 June;49(3). Cross-sectional study
Ref ID: 4043

(939) Carvalho JFd, Caleiro MTC, Bonfá E. Hyperhomocysteinemia and primary antiphospholipid syndrome. Hiper-homocisteinemia e síndrome antifosfolípide primária. Revista brasileira de reumatologia 2009 August;49(4). Cross-sectional study
Ref ID: 4042

(940) Carvalho LAPd, Rodacki ALF. A influência de duas mochilas sobre a cinemática da coluna de crianças. Revista Brasileira de Educação Física e Esporte 2008 March;22(1):44-52. Not an exercise intervention study
Ref ID: 4044

(941) Carvalho P, Oliveira B, Barros R, Padrao P, Moreira P, Teixeira VH. Impact of fluid restriction and ad libitum water intake or an 8% carbohydrate-electrolyte beverage on skill performance of elite adolescent basketball players. International Journal of Sport Nutrition and Exercise Metabolism 2011

June;21(3):214-21. Diet Intervention or Supplement Study
Ref ID: 2447

(942) Casanueva E. V, Milos G, Chiang S. MT, Espejo G, Cid C, Riquelme M. Efectos de un programa de entrenamiento aeróbico de dos años de duración sobre el perfil lipídico de escolares obesos. Revista Chilena de Pediatría 1992 December;63(6):312-5. Lifestyle Intervention
Ref ID: 815

(943) Casazza K, Ciccazzo M. Improving the dietary patterns of adolescents using a computer-based approach. Journal of School Health 2006 February;76(2):43-6. Diet Intervention Study
Ref ID: 1420

(944) Casazza K, Ciccazzo M. The method of delivery of nutrition and physical activity information may play a role in eliciting behavior changes in adolescents. Eating Behaviors 2007 January;8(1):73-82. Lifestyle Intervention
Ref ID: 1292

(945) Casazza K, Hanks LJ, Beasley TM, Fernandez JR. Beyond Thriftiness: Independent and interactive effects of genetic and dietary factors on variations in fat deposition and distribution across populations. American Journal of Physical Anthropology 2011;145(2):181-91. Not an exercise intervention study
Ref ID: 5205

(946) Casey AA, Elliott M, Glanz K, Haire-Joshu D, Lovegreen SL, Saelens BE, Sallis JF, Brownson RC. Impact of the food environment and physical activity environment on behaviors and weight status in rural U.S. communities. Preventive Medicine 2008 December;47(6):600-4. Cross-sectional study
Ref ID: 843

(947) Casiro O, Bingham W, MacMurray B, Whitfield M, Saigal S, Vincer M, Long W. One-year follow-up of 89 infants with birth weights of 500 to 749 grams and respiratory distress syndrome randomized to two rescue doses of synthetic surfactant or air placebo. Canadian Exosurf Neonatal Study Group. Canadian Exosurf Neonatal Follow-Up Group. The Journal of Pediatrics 1995;126:S53-S60. Subjects less than 2 years old
Ref ID: 4712

(948) Castelli DM, Hillman CH, Hirsch J, Hirsch A, Drollette E. FIT Kids: Time in target heart zone and cognitive performance. Preventive Medicine 2011 June;52:Suppl-9. Not a randomized controlled trial (RCT)
Ref ID: 1043

(949) Castelnuovo G, Manzoni GM, Villa V, Cesa GL, Pietrabissa G, Molinari E. The STRATOB study: design of a randomized controlled clinical trial of Cognitive Behavioral Therapy and Brief Strategic Therapy with telecare in patients with obesity and binge-eating disorder referred to residential nutritional

rehabilitation. Trials [Electronic Resource] 2011;12:114. Description of study from review or magazine or etc. (not the actual study)
Ref ID: 2449

(950) Castillo C, Kain B. Consejería en vida sana y cambio de conductas en escolares obesos: Intervención controlada en madres/cuidadoras. Counseling on healthy lifestyles and behavioral change in obese school children: Controlled intervention in mothers/caretakers. Revista Chilena de Nutrición 2010 June;37(2):155-63. Not a randomized controlled trial (RCT)
Ref ID: 4045

(951) Castillo I, Molina-García J. Adiposidad corporal y bienestar psicológico: Efectos de la actividad física en universitarios de Valencia, España. Adiposity and psychological well-being: Effects of physical activity on university students in Valencia, Spain. Revista Panamericana de Salud Pública 2009 October;26(4):334-40. Study limited to adults
Ref ID: 4046

(952) Castro IRRd, Cardoso LO, Engstrom EM, Levy RB, Monteiro CA. Vigilância de fatores de risco para doenças não transmissíveis entre adolescentes: A experiência da cidade do Rio de Janeiro, Brasil. Cadernos de Saúde Pública 2008 October;24(10):2279-88. Survey or questionnaire
Ref ID: 4047

(953) Catellier DJ, Hannan PJ, Murray DM, Addy CL, Conway TL, Yang S, Rice JC. Imputation of missing data when measuring physical activity by accelerometry. Medicine and Science in Sports and Exercise 2005 November;37(11:Suppl):Suppl-62. Not an exercise intervention study
Ref ID: 1447

(954) Catenacci VA, Barrett C, Odgen L, Browning R, Schaefer CA, Hill J, Wyatt H. Changes in physical activity and sedentary behavior in a randomized trial of an internet-based versus workbook-based family intervention study. Journal of Physical Activity and Health 2014 February;11(2):348-58. Inappropriate Comparison Group
Ref ID: 5794

(955) Catenassi FZ, Marques I, Bastos CB, Basso L, Ronque ERV, Gerage AM. Relação entre índice de massa corporal e habilidade motora grossa em crianças de quatro a seis anos. Revista Brasileira de Medicina do Esporte 2007 August;13(4):227-30. Not a randomized controlled trial (RCT)
Ref ID: 4048

(956) Catina P. Psychological modeling and adaptations in cognitive representations with increased resistance during motor skill acquisition. Journal of Strength and Conditioning Research 2009 March;23(2):668-76. Study limited to adults
Ref ID: 791

(957) Cattai GB, Hintze LJ, Nardo Junior N. Validação interna do questionário de estágio de prontidão para mudança do comportamento alimentar e de atividade física. Internal validation of the stage of change questionnaire for alimentary and physical activity behaviors. Revista Paulista de Pediatria 2010 June;28(2):194-9. Not an exercise intervention study
Ref ID: 4049

(958) Cattai GBP, Rocha FA, Hintze LJ, Pagan BGM, Junior NN. Programa de tratamento multiprofissional da obesidade: os desafios da prática. Ciência Cuidado e Saúde 2008 May;7(supl.1):121-6. Lifestyle Intervention
Ref ID: 4050

(959) Cauchi S, Stutzmann F, Cavalcanti-Proenca C, Durand E, Pouta A, Hartikainen AL, Marre M, Vol S, Tammelin T, Laitinen J, Gonzalez-Izquierdo A, Blakemore AI, Elliott P, Meyre D, Balkau B, Jarvelin MR, Froguel P. Combined effects of MC4R and FTO common genetic variants on obesity in European general populations. Journal of Molecular Medicine 2009 May;87(5):537-46. Cohort Study
Ref ID: 766

(960) Caumo W, Hidalgo MPL, Schmidt AP, Iwamoto CW, Adamatti LC, Bergmann J, Ferreira MBC. Effect of pre-operative anxiolysis on postoperative pain response in patients undergoing total abdominal hysterectomy. Anaesthesia 2002 August;57(8):740-6. Drug intervention study
Ref ID: 3544

(961) Cavalcanti CB, Barros MV, Menêses AL, Santos CM, Azevedo AM, Guimarães FJ. Obesidade abdominal em adolescentes: Prevalência e associação com atividade física e hábitos alimentares. Abdominal obesity in adolescents: Prevalence and association with physical activity and eating habits. Arquivos brasileiros de cardiologia 2010 March;94(3):371-7. Survey or questionnaire
Ref ID: 4051

(962) Cawley MM, Benson LM. Current trends in managing oral mucositis. Clinical Journal of Oncology Nursing 2005 October;9(5):584-92. Not an exercise intervention study
Ref ID: 3545

(963) Cebulj Navarrete D, Vildoso Castillo JF, Quezada Donoso E, Figueroa Mellado F, Prieto Correa MJ, Díaz Narváez VP, Maturana Aracena P, Orellana Campos B. Función pulmonar en niños sanos de 7 y 8 años de las comunas de Cerro Navia y Los Andes expuestos a diferentes niveles de contaminación por MP10. Lung function in healthy children aged 7 and 8 of the comunes of Cerro Navia and Los Andes exposes to different levels of pollution by MP10. Salud Uninorte 2011 December;27(2):198-209. Not an exercise intervention study
Ref ID: 4052

(964) Cecil JE, Tavendale R, Watt P, Hetherington MM, Palmer CNA. An obesity-associated FTO gene variant and increased energy intake in children. New England Journal of Medicine 2008;359(24):2558-66. Cross-sectional study
Ref ID: 5206

(965) Cederborg J, Freinkel S, John F, Motte P, Spence E, Spitz T, Weaver A, Wong K, Wrightson C. healthy news. Health (Time Inc Health) 2000 September;14(7):19. Description of study from review or magazine or etc. (not the actual study)
Ref ID: 3887

(966) Cejudo P, Bautista J, Montemayor T, Villagomez R, Jimenez L, Ortega F, Campos Y, Sanchez H, Arenas J. Exercise training in mitochondrial myopathy: a randomized controlled trial. Muscle and Nerve 2005 September;32(3):342-50. Not a randomized controlled trial (RCT)
Ref ID: 1479

(967) Celermajer DS. Wait for weight or "waste" the waist: the benefits of early intervention in childhood obesity. Journal of the American College of Cardiology 2009 December 15;54(25):2407-8. Editorial or letter or comment
Ref ID: 619

(968) Celio AA. Early intervention of eating- and weight-related problems via the Internet in overweight adolescents: A randomized controlled trial. United States -- California: University of California, San Diego and San Diego State University; 2005. Lifestyle Intervention

Ref ID: 5103

(969) Cerezo Correa MdP, Vergara Quintero MdC, Nieto Murillo E, Cifuentes Aguirre OL, Parra Sánchez JH. Características de salud pública de estudiantes de una universidad privada de la ciudad de Manizales. Public health characteristics of a private university students in Manizales. Carateristicas de saúde publica de estudantes duma universidade privada da cidade de Manizales. Hacia Promocion de la salud 2011 June;16(1):73-86. Study limited to adults
Ref ID: 4053

(970) Cesar CMPdCdS, Oliveira TA, Santos JFKd, Souza Ed, Camano L. Uso da monitorizaçäo da contraçäo uterina e da autopalpaçäo materna para prevenir a prematuridade. Femina 1998 October;26(9):743-6. Subjects less than 2 years old
Ref ID: 4054

(971) Cesar JA, Mendoza-Sassi R, Horta BL, Ribeiro PRP, D'Avila AC, Santos FM, Martins PB, Brandolt RR. Basic indicators of child health in an urban area in southern Brazil: estimating prevalence rates and evaluating differentials. Jornal de Pediatria 2006 December;82(6):437-44. Cross-sectional study
Ref ID: 651

(972) Cesar MC, Borin JP, Gonelli PR, Simoes RA, de Souza TM, Montebelo MI. The effect of local muscle endurance training on cardiorespiratory capacity in young women. Journal of Strength and Conditioning Research 2009 September;23(6):1637-43. Study limited to adults
Ref ID: 680

(973) Cespedes EM, McDonald J, Haines J, Bottino CJ, Schmidt ME, Taveras EM. Obesity-related behaviors of US- and non-US-born parents and children in low-income households. Journal of Developmental and Behavioral Pediatrics 2013 October;34(8):541-8. Inappropriate Study Design
Ref ID: 5795

(974) Cezar C. O tratamento da obesidade estruturado em terapêutica multiprofissional. Pediatria Mod 2000 March;36(3):140-6. Review article
Ref ID: 4055

(975) Chad KE, Bailey DA, McKay HA, Zello GA, Snyder RE. The effect of a weight-bearing physical activity program on bone mineral content and estimated volumetric density in children with spastic cerebral palsy. The Journal of Pediatrics 1999;135:115-7. Not a randomized controlled trial (RCT), Not All Participants were Overweight and/or Obese
Ref ID: 4713

(976) Chaddock L, Erickson KI, Prakash RS, Kim JS, Voss MW, VanPatter M, Pontifex MB, Raine LB, Konkel A, Hillman CH, Cohen NJ, Kramer AF. A neuroimaging investigation of the association between aerobic fitness, hippocampal volume, and memory performance in preadolescent children. Brain Research 2010;1358:172-83. Not a randomized controlled trial (RCT)
Ref ID: 5207

(977) Chaddock L, Hillman CH, Buck SM, Cohen NJ. Aerobic fitness and executive control of relational memory in preadolescent children. Medicine and Science in Sports and Exercise 2011;43(2):344-9. Not a randomized controlled trial (RCT)
Ref ID: 5208

(978) Chae HW, Kwon YN, Rhie YJ, Kim HS, Kim YS, Paik IY, Suh SH, Kim DH. Effects of a structured exercise program on insulin resistance, inflammatory markers and physical fitness in obese Korean children. Journal of Pediatric Endocrinology and Metabolism 2010 October;23(10):1065-72. No exercise only group
Ref ID: 4

(979) Chagas PSdCC, Soares TBdC, Mancini MC, Fonseca STd, Vaz DV, Gontijo APB. Mudanças antropométricas e habilidade motora em crianças no início da marcha independente. Fisioterapia e Pesquisa 2006;13(2):43-9. Subjects less than 2 years old
Ref ID: 654

(980) Chagnon YC, Rice T, Perusse L, Borecki IB, Ho-Kim MA, Lacaille M, Pare C, Bouchard L, Gagnon J, Leon AS, Skinner JS, Wilmore JH, Rao DC, Bouchard C, HERITAGE Family. Genomic scan for genes affecting body composition before and after training in Caucasians from HERITAGE. Journal of Applied Physiology 2001 May;90(5):1777-87. Not an exercise intervention study
Ref ID: 1921

(981) Chahal H, Fung C, Kuhle S, Veugelers PJ. Availability and night-time use of electronic entertainment and communication devices are associated with short sleep duration and obesity among Canadian children. Pediatric Obesity 2013 February;8(1):42-51. Inappropriate Study Design
Ref ID: 5796

(982) Chamorro-Vina C, Ruiz JR, Santana-Sosa E, Gonzalez VM, Madero L, Perez M, Fleck SJ, Perez A, Ramirez M, Lucia A. Exercise during hematopoietic stem cell transplant hospitalization in children. Medicine and Science in Sports and Exercise 2010 June;42(6):1045-53. Study less than 4 weeks
Ref ID: 529

(983) Champagne CM, Bray GA, Kurtz AA, Monteiro JBR, Tucker E, Volaufova J, DeLany JP. Energy intake and energy expenditure: A controlled study comparing dietitians and non-dietitians. Journal of the American Dietetic Association 2002;102(10):1428-32. Not an exercise intervention study
Ref ID: 5209

(984) Champkin J. Making information beautiful - and clear. Significance 2011 March;8(1):39-41. Not an exercise intervention study
Ref ID: 3546

(985) Chan Osilla K, Van Busum K, Schnyer C, Wozar Larkin J, Eibner C, Mattke S. Systematic review of the impact of worksite wellness programs. American Journal of Managed Care 2012 February;18(2):e68-e81. Review article
Ref ID: 3547

(986) Chang CY, Schiano TD. Review article: drug hepatotoxicity. Alimentary Pharmacology and Therapeutics 2007 May 15;25(10):1135-51. Review article
Ref ID: 3548

(987) Chang KW. Randomized controlled trial of Coblation versus electrocautery tonsillectomy. Otolaryngology Head and Neck Surgery 2005;132(2):273-80. Not an exercise intervention study
Ref ID: 3203

(988) Chang MW, Brown R, Nitzke S. Participant recruitment and retention in a pilot program to prevent weight gain in low-income overweight and obese mothers. BMC Public Health 2009 November 21;9:424.:424. Study not limited to children and adolescents
Ref ID: 80

(989) Chang MW, Nitzke S, Brown R. Design and outcomes of a Mothers In Motion behavioral intervention pilot study. Journal of Nutrition Education and Behavior 2010 May;42(3 Suppl):S11-S21. Study not limited to children and adolescents
Ref ID: 46

(990) Chang YY, Chiou WB. Taking weight-loss supplements may elicit liberation from dietary control. A laboratory experiment. Appetite 2014 January;72:8-12. Inappropriate Intervention
Ref ID: 5797

(991) Chanoine JP, Hampl S, Jensen C, Boldrin M, Hauptman J. Effect of orlistat on weight and body composition in obese adolescents: a randomized controlled trial. Journal of the American Medical Association 2005 June 15;293(23):2873-83. No exercise only group, Drug intervention study
Ref ID: 309

(992) Chaparro CM, Dewey KG. Use of lipid-based nutrient supplements (LNS) to improve the nutrient adequacy of general food distribution rations for vulnerable sub-groups in emergency settings. Maternal and Child Nutrition 2010 January 2;6:1-69. Diet Intervention or Supplement Study
Ref ID: 3549

(993) Chapelot D. The role of snacking in energy balance: A biobehavioral approach. Journal of Nutrition 2011;141(1):158-62. Diet Intervention or Supplement Study
Ref ID: 5210

(994) Chapman P, Toma RB, Tuveson RV, Jacob M. Nutrition knowledge among adolescent high school female athletes. Adolescence 1997;32(126):437-46. Diet Intervention or Supplement Study
Ref ID: 2140

(995) Chaput JP, Visby T, Nyby S, Klingenberg L, Gregersen NT, Tremblay A, Astrup A, Sjodin A. Video game playing increases food intake in adolescents: a randomized crossover study. American Journal of Clinical Nutrition 2011 June;93(6):1196-203. Acute study
Ref ID: 2451

(996) Charbonnel B, Karasik A, Liu J, Wu M, Meininger G, Sitagliptin S. Efficacy and safety of the dipeptidyl peptidase-4 inhibitor sitagliptin added to ongoing metformin therapy in patients with type 2 diabetes inadequately controlled with metformin alone. Diabetes Care 2006 December;29(12):2638-43. Drug intervention study
Ref ID: 1322

(997) Chasan-Taber L, Marcus BH, Rosal MC, Tucker KL, Hartman SJ, Pekow P, Braun B, Moore Simas TA, Solomon CG, Manson JE, Markenson G. Estudio Parto: postpartum diabetes prevention program for hispanic women with abnormal glucose tolerance in pregnancy: a randomised controlled trial - study

protocol. BMC Pregnancy Childbirth 2014;14:100. Inappropriate Population
Ref ID: 5798

(998) Chateauvieux SÃ, Morceau F, Dicato M, Diederich M. Molecular and Therapeutic potential and toxicity of valproic acid. Journal of Biomedicine and Biotechnology 2010 January;1-18. Drug intervention study
Ref ID: 3550

(999) Chavarro JE, Peterson KE, Sobol AM, Wiecha JL, Gortmaker SL. Effects of a school-based obesity-prevention intervention on menarche (United States). Cancer Causes Control 2005 December;16(10):1245-52. No exercise only group
Ref ID: 296

(1000) Chaves CRMdM, Oliveira CQd, Britto JAAd, Elsas MICG. Exercício aeróbico, treinamento de força muscular e testes de aptidão física para adolescentes com fibrose cística: revisão da literatura. Exercise testing, aerobic and strength training for adolescents with cystic fibrosis: a literature review. Revista Brasileira de Saúde Materno Infantil 2007 September;7(3):245-50. Review article
Ref ID: 4056

(1001) Chaves ES, Silva VMd, Costa FBC, Araujo TLd. Avaliação do índice de massa corporal em crianças e adolescentes de uma Escola Pública de Fortaleza – Ceará. Evaluación del índice de masa corpórea en niños y adolescentes de una escuela pública de Fortaleza-Ceará. Revista Paulista de Enfermagem 2004 March;23(1):37-42. Not All Participants were Overweight and/or Obese
Ref ID: 487

(1002) Chavez-Tapia NC, Sanchez-Avila F, Vasquez-Fernandez F, Torres-Machorro A, Tellez-Avila FI, Uribe M. Non-alcoholic fatty-liver disease in pediatric populations. Journal of Pediatric Endocrinology and Metabolism 2007;20(10):1059-73. Review article
Ref ID: 3204

(1003) Chawla K, Mishra R, Sachdeva V, Beenu. Correlation of antioxidants and fitness levels in undergraduate medical students. Indian Journal of Physiology and Pharmacology 2007 July;51(3):293-5. Study limited to adults
Ref ID: 1114

(1004) Checon K, Fonseca VM, Faria CPd, Carletti L, Molina MdCB. Reprodutibilidade do questionário de avaliação de atividade física para crianças aplicado no Estudo Saúdes: Vitória. The reproducibility of the Saúdes: Vitória Study's physical activity assessment questionnaire for children. Revista Brasileira de Saúde Materno Infantil 2011 June;11(2):173-80. Survey or questionnaire, Cross-sectional study
Ref ID: 4057

(1005) Chehuen MdR, Bezerra AIL, Bartholomeu T, Junqueira NO, Rezende JAS, Basso L, Oliveira JA, Lemos WP, Tani G, Prista A, Maia JAR, Forjaz CLdM. Risco cardiovascular e prática de atividade física em crianças e adolescentes de Muzambinho/MG: Influência do gênero e da idade. Cardiovascular risk and physical activity practice in children and adolescents of Muzambinho/MG: Influence of gender and age. Revista Brasileira de Medicina do Esporte 2011 August;17(4):232-6. Cross-sectional study
Ref ID: 4058

(1006) Chellini E, Talassi F, Corbo G, Berti G, De SM, Rusconi F, Piffer S, Caranci N, Petronio MG, Sestini P, Dell'Orco V, Bonci E, Armenio L, La GS, Gruppo Collaborativo SIDR. [Environmental, social and demographic characteristics of children and adolescents, resident in different Italian areas]. [Italian]. Epidemiologia e Prevenzione 2005 March;29(2:Suppl):Suppl-23. Cross-sectional study
Ref ID: 1477

(1007) Chen CC, Lin SY. The impact of rope jumping exercise on physical fitness of visually impaired students. Research in Developmental Disabilities 2011 January;32(1):25-9. Not All Participants were Overweight and/or Obese, Not a randomized controlled trial (RCT)
Ref ID: 2452

(1008) Chen HL, Lee CL, Tseng HI, Yang SN, Yang RC, Jao HC. Assisted exercise improves bone strength in very low birthweight infants by bone quantitative ultrasound. Journal of Paediatrics and Child Health 2010;46:653-9. Subjects less than 2 years old
Ref ID: 4714

(1009) Chen J, Sadakata M, Ishida M, Sekizuka N, Sayama M. Baby massage ameliorates neonatal jaundice in full-term newborn infants. The Tohoku Journal of Experimental Medicine 2011;223:97-102. Subjects less than 2 years old
Ref ID: 4715

(1010) Chen JL, Weiss S, Heyman MB, Lustig RH. Efficacy of a child-centred and family-based program in promoting healthy weight and healthy behaviors in Chinese American children: a randomized controlled study. Journal of Public Health (Oxf) 2010 June;32(2):219-29. No exercise only group
Ref ID: 78

(1011) Chen JL, Weiss SJ, Heyman MB, Cooper B, Lustig RH. The Active Balance Childhood program for improving coping and quality of life in Chinese American children. Nursing Research 2010;59:270-9. No exercise only group
Ref ID: 4716

(1012) Chen JL, Weiss S, Heyman MB, Cooper B, Lustig RH. The efficacy of the web-based childhood obesity prevention program in Chinese American adolescents

(Web ABC study). Journal of Adolescent Health 2011 August;49(2):148-54. Lifestyle Intervention
Ref ID: 2453

(1013) Chen KT, Chen YY, Wu HJ, Chang CK, Lee WT, Lu YY, Liu CC, Yang RS, Lin JC. Decreased anaerobic performance and hormone adaptation after expedition to Peak Lenin. Chinese Medical Journal 2008 November 20;121(22):2229-33. Not a randomized controlled trial (RCT)
Ref ID: 840

(1014) Chen LL, Su YC, Su CH, Lin HC, Kuo HW. Acupressure and meridian massage: combined effects on increasing body weight in premature infants.[Erratum appears in J Clin Nurs. 2008 Aug;17(15):2089]. Journal of Clinical Nursing 17(9):1174-81, 2008 May 2008;(9):1174-81. Subjects less than 2 years old
Ref ID: 2918

(1015) Chen SL, Li LL, Li HL. [Effect of massage in traditional Chinese medicine on short penis in male obese children]. Chinese Journal of Clinical Rehabilitation 2006;10:168-9. Not an exercise intervention study
Ref ID: 4717

(1016) Chen TL, Mao HC, Lai CH, Li CY, Kuo CH. [The effect of yoga exercise intervention on health related physical fitness in school-age asthmatic children]. [Chinese]. Hu Li Tsa Chih Journal of Nursing 2009;56:42-52. Not a randomized controlled trial (RCT)
Ref ID: 4718

(1017) Chen X, Wang Y. Is ideal body image related to obesity and lifestyle behaviours in African American adolescents? Child: Care, Health and Development 2012 March;38(2):219-28. Survey or questionnaire
Ref ID: 2454

(1018) Chen YC, Chen PC, Hsieh WS, Portnov BA, Chen YA, Lee YL. Environmental factors associated with overweight and obesity in taiwanese children. Paediatric and Perinatal Epidemiology 2012 November;26(6):561-71. Inappropriate Study Design
Ref ID: 5799

(1019) Cheng-Mayer C, Watkins D, Marthas M, Picker LJ. 20[sup th] Annual Symposium on Nonhuman Primate Models for AIDS. Journal of Medical Primatology 32[4/5], 265. 2003. Abstract,
Ref ID: 3551

(1020) Cheng HL, Griffin HJ, Bryant CE, Rooney KB, Steinbeck KS, O'Connor HT. Impact of diet and weight loss on iron and zinc status in overweight and obese young women. Asia Pacific Journal of Clinical Nutrition 2013;22(4):574-82.

Inappropriate Population
Ref ID: 5800

(1021) Cherkas LF, Hunkin JL, Kato BS, Richards JB, Gardner JP, Surdulescu GL, Kimura M, Lu X, Spector TD, Aviv A. The association between physical activity in leisure time and leukocyte telomere length. Archives of Internal Medicine 2008 January 28;168(2):154-8. Survey or questionnaire
Ref ID: 1005

(1022) Cherng RJ, Liu CF, Lau TW, Hong RB. Effect of treadmill training with body weight support on gait and gross motor function in children with spastic cerebral palsy. American Journal of Physical Medicine and Rehabilitation 2007 July;86(7):548-55. Not All Participants were Overweight and/or Obese
Ref ID: 1211

(1023) Cheung BM. Drug treatment for obesity in the post-sibutramine era. Drug Safety 2011 August 1;34(8):641-50. Drug intervention study
Ref ID: 2455

(1024) Cheung SS, Sleivert GG. Lowering of skin temperature decreases isokinetic maximal force production independent of core temperature. European Journal of Applied Physiology 2004 May;91(5-6):723-8. Not an exercise intervention study
Ref ID: 1642

(1025) Chevallier JM, Paita M, Rodde-Dunet MH, Marty M, Nogues F, Slim K, Basdevant A. Predictive factors of outcome after gastric banding: a nationwide survey on the role of center activity and patients' behavior. Annals of Surgery 2007 December;246(6):1034-9. Survey or questionnaire
Ref ID: 1134

(1026) Chiappetta DA, Alvarez-Lorenzo C, Rey-Rico A, Taboada P, Concheiro A, Sosnik A. N-alkylation of poloxamines modulates micellar assembly and encapsulation and release of the antiretroviral efavirenz. European Journal of Pharmaceutics and Biopharmaceutics 2010;76(1):24-37. Drug intervention study
Ref ID: 5211

(1027) Chiaratti PS, Sprocatti R, Piovesana AM. Crianças de alto risco: evoluçäo dos aspectos linguísticos e cognitivos. Temas sobre Desenvolvimento 2001 August;10(57):19-23. Not an exercise intervention study
Ref ID: 731

(1028) Childs JD, Teyhen DS, Van Wyngaarden JJ, Dougherty BF, Ladislas BJ, Helton GL, Robinson ME, Wu SS, George SZ. Predictors of web-based follow-up response in the Prevention Of Low Back Pain In The Military Trial (POLM). BMC Musculoskeletal Disorders 2011;12:132. Study limited to adults
Ref ID: 2456

(1029) Chinapaw MJ, Proper KI, Brug J, van MW, Singh AS. Relationship between young peoples' sedentary behaviour and biomedical health indicators: a systematic review of prospective studies. [Review]. Obesity Reviews 2011 July;12(7):e621-e632. Review article
Ref ID: 2457

(1030) Chipps BE. Asthma in Infants and Children. Clin Cornerstone 2008;8(4):44-61. Review article
Ref ID: 3205

(1031) Cho AH, Killeya-Jones LA, O'Daniel JM, Kawamoto K, Gallagher P, Haga S, Lucas JE, Trujillo GM, Joy SV, Ginsburg GS. Effect of genetic testing for risk of type 2 diabetes mellitus on health behaviors and outcomes: study rationale, development and design. BMC Health Services Research 2012;12:16. Description of study from review or magazine or etc. (not the actual study)
Ref ID: 2458

(1032) Chomitz VR, Collins J, Kim J, Kramer E, McGowan R. Promoting healthy weight among elementary school children via a health report card approach. Archives of Pediatrics and Adolescent Medicine 2003;157:765-72. Not a randomized controlled trial (RCT)
Ref ID: 4719

(1033) Chomtho S, Wells JC, Williams JE, Davies PS, Lucas A, Fewtrell MS. Infant growth and later body composition: evidence from the 4-component model. American Journal of Clinical Nutrition 2008 June;87(6):1776-84. Cross-sectional study
Ref ID: 939

(1034) Choudhuri D, Choudhuri S, Kulkarni VA. Physical fitness: a comparative study between students of residential (Sainik) and non-residential schools (aged 12-14 years). Indian Journal of Physiology and Pharmacology 2002 July;46(3):328-32. Cross-sectional study
Ref ID: 1783

(1035) Chowdhury PP, Balluz LS, Zhao G, Town M. Health behaviors and obesity among Hispanics with depression, United States 2006. Ethnicity and Disease 2014;24(1):92-6. Inappropriate Study Design
Ref ID: 5801

(1036) Chrisoulidou A, Kousta E, Beshyah SA, Robinson S, Johnston DG. How much, and by what mechanisms, does growth hormone replacement improve the quality of life in GH-deficient adults?. [Review] [52 refs]. Baillieres Clinical Endocrinology and Metabolism 1998 July;12(2):261-79. Review article
Ref ID: 2065

(1037) Christ ER, Cummings MH, Westwood NB, Sawyer BM, Pearson TC, Sonksen PH, Russell-Jones DL. The importance of growth hormone in the regulation of

erythropoiesis, red cell mass, and plasma volume in adults with growth hormone deficiency. Journal of Clinical Endocrinology and Metabolism 1997 September;82(9):2985-90. Study limited to adults
Ref ID: 2127

(1038) Christensen JR, Overgaard K, Carneiro IG, Holtermann A, Sogaard K. Weight loss among female health care workers--a 1-year workplace based randomized controlled trial in the FINALE-health study. BMC Public Health 2012;12:625. Inappropriate Population
Ref ID: 5802

(1039) Christensen JR, Overgaard K, Hansen K, Sogaard K, Holtermann A. Effects on presenteeism and absenteeism from a 1-year workplace randomized controlled trial among health care workers. Journal of Occupational and Environmental Medicine 2013 October;55(10):1186-90. Inappropriate Population
Ref ID: 5803

(1040) Christian JG, Byers TE, Christian KK, Goldstein MG, Bock BC, Prioreschi B, Bessesen DH. A computer support program that helps clinicians provide patients with metabolic syndrome tailored counseling to promote weight loss. Journal of the American Dietetic Association 2011 January;111(1):75-83. Study limited to adults
Ref ID: 2459

(1041) Christiansen LB, Toftager M, Boyle E, Kristensen PL, Troelsen J. Effect of a school environment intervention on adolescent adiposity and physical fitness. Scandinavian Journal of Medicine and Science in Sports 2013 December;23(6):e381-e389. Inappropriate Population
Ref ID: 5804

(1042) Christiansen LI, LÃ¤hteenmÃ¤ki PLA, Mannelin MR, SeppÃ¤nen-Laakso TE, Hiltunen RVK, Yliruusi JK. Cholesterol-lowering effect of spreads enriched with microcrystalline plant sterols in hypercholesterolemic subjects. European Journal of Nutrition 2001 April;40(2):66. Diet Intervention or Supplement Study
Ref ID: 491

(1043) Christiansen T, Paulsen SK, Bruun JM, Overgaard K, Ringgaard S, Pedersen SB, Positano V, Richelsen B. Comparable reduction of the visceral adipose tissue depot after a diet-induced weight loss with or without aerobic exercise in obese subjects: a 12-week randomized intervention study. European Journal of Endocrinology 2009 May;160(5):759-67. No comparative control group
Ref ID: 135

(1044) Christiansen T, Paulsen SK, Bruun JM, Ploug T, Pedersen SB, Richelsen B. Diet-induced weight loss and exercise alone and in combination enhance the expression of adiponectin receptors in adipose tissue and skeletal muscle, but only diet-induced weight loss enhanced circulating adiponectin. Journal of

Clinical Endocrinology and Metabolism 2010 February;95(2):911-9. Study not limited to children and adolescents, No comparative control group
Ref ID: 74

(1045) Christiansen T, Paulsen SK, Bruun JM, Pedersen SB, Richelsen B. Exercise training versus diet-induced weight-loss on metabolic risk factors and inflammatory markers in obese subjects: a 12-week randomized intervention study. American Journal of Physiology, Endocrinology and Metabolism 2010 April;298(4):E824-E831. No comparative control group
Ref ID: 70

(1046) Christison A, Khan HA. Exergaming for health: a community-based pediatric weight management program using active video gaming. Clinical Pediatrics 2012 April;51(4):382-8. Not a randomized controlled trial (RCT)
Ref ID: 2460

(1047) Christofaro DGD, Andrade SMd, Fernandes RA, Ohara D, Dias DF, Freitas Júnior IF, Oliveira DRd. Prevalência de fatores de risco para doenças cardiovasculares entre escolares em Londrina - PR: Diferenças entre classes econômicas. Prevalence of risk factors for cardiovascular diseases among students of Londrina - PR: Differences between economic classes. Revista Brasileira de Eidemiologia 2011 March;14(1):27-35. Cross-sectional study
Ref ID: 4059

(1048) Chromiak JA, Smedley B, Carpenter W, Brown R, Koh YS, Lamberth JG, Joe LA, Abadie BR, Altorfer G. Effect of a 10-week strength training program and recovery drink on body composition, muscular strength and endurance, and anaerobic power and capacity. Nutrition 2004 May;20(5):420-7. Diet Intervention or Supplement Study
Ref ID: 1640

(1049) Chu L, Riddell MC, Takken T, Timmons BW. Carbohydrate intake reduces fat oxidation during exercise in obese boys. European Journal of Applied Physiology 2011 December;111(12):3135-41. Diet Intervention or Supplement Study
Ref ID: 2461

(1050) Chuang SC, Gallo V, Michaud D, Overvad K, Tjonneland A, Clavel-Chapelon F, Romieu I, Straif K, Palli D, Pala V, Tumino R, Sacerdote C, Panico S, Peeters PH, Lund E, Gram IT, Manjer J, Borgquist S, Riboli E, Vineis P. Exposure to environmental tobacco smoke in childhood and incidence of cancer in adulthood in never smokers in the European Prospective Investigation into Cancer and Nutrition. Cancer Causes and Control 2011 March;22(3):487-94. Not an exercise intervention study
Ref ID: 2462

(1051) Chubak J, Tworoger SS, Yasui Y, Ulrich CM, Stanczyk FZ, McTiernan A. Associations between reproductive and menstrual factors and postmenopausal sex hormone concentrations. Cancer Epidemiology, Biomarkers and Prevention 2004 August;13(8):1296-301. Study limited to adults
Ref ID: 1603

(1052) Chumnijarakij T, Nuchprayoon T, Chitinand S, Onthuam Y, Quamkul N, Dusitsin N, Viputsiri OA, Chotiwan P, Limpongsanurak S, Sukomol P. Maternal risk factors for low birth weight newborn in Thailand. Journal of the Medical Association of Thailand 1992 August;75(8):445-52. Not an exercise intervention study
Ref ID: 2275

(1053) Church C, Lee S, Bagg EAL, McTaggart JS, Deacon R, Gerken T, Lee A, Moir L, Mecinovic J, Quwailid MM, Schofield CJ, Ashcroft FM, Cox RD. A Mouse Model for the Metabolic Effects of the Human Fat Mass and Obesity Associated FTO Gene. Plos Genetics 2009;5(8). Animal study
Ref ID: 5212

(1054) Church C, Moir L, McMurray F, Girard C, Banks GT, Teboul L, Wells S, Bruning JC, Nolan PM, Ashcroft FM, Cox RD. Overexpression of Fto leads to increased food intake and results in obesity. Nature Genetics 2010;42(12):1086-U147. Animal study
Ref ID: 5213

(1055) Ciampo LAD, Rodrigues DMS, Ciampo IRLD, Cardoso VC, Bettiol H, Barbieri MA. Percepção corporal e atividade física em uma coorte de adultos jovens brasileiros. Body image and physical activity among a brazilian youth cohort. Revista Brasileira de Crescimento e Desenvolvimento Humano 2010;20(3):671-9. Cohort Study
Ref ID: 4060

(1056) Ciampolini M, Bini S, Giommi A, Vicarelli D, Giannellini V. Same growth and different energy intake over four years in children suffering from chronic non-specific diarrhoea. International Journal of Obesity and Related Metabolic Disorders 1994;18:17-23. Not an exercise intervention study
Ref ID: 4720

(1057) Ciccolo JT, Dunsiger SI, Williams DM, Bartholomew JB, Jennings EG, Ussher MH, Kraemer WJ, Marcus BH. Resistance training as an aid to standard smoking cessation treatment: A pilot study. Nicotine and Tobacco Research 2011 August;13(8):756-60. Study limited to adults
Ref ID: 2463

(1058) Cieslak TJ, Frost G, Klentrou P. Effects of physical activity, body fat, and salivary cortisol on mucosal immunity in children. Journal of Applied Physiology

2003 December;95(6):2315-20. Cross-sectional study
Ref ID: 1706

(1059) Cimadon HMS, Geremia R, Pellanda LC. Hábitos alimentares e fatores de risco para aterosclerose em estudantes de Bento Gonçalves (RS). Dietary habits and risk factors for atherosclerosis in students from Bento Gonçalves (state of Rio Grande do Sul). Arquivos brasileiros de cardiologia 2010 August;95(2):166-72. Cross-sectional study
Ref ID: 4061

(1060) Cimolin V, Galli M, Grugni G, Vismara L, Albertini G, Rigoldi C, Capodaglio P. Gait patterns in Prader-Willi and Down syndrome patients. Journal of Neuroengineering and Rehabilitation 2010;7:28. Not an exercise intervention study
Ref ID: 495

(1061) Cinar V, Mogulkoc R, Baltaci AK, Polat Y. Adrenocorticotropic hormone and cortisol levels in athletes and sedentary subjects at rest and exhaustion: effects of magnesium supplementation. Biological Trace Element Research 2008 March;121(3):215-20. Diet Intervention or Supplement Study
Ref ID: 1002

(1062) Cinar V, Polat Y, Baltaci AK, Mogulkoc R. Effects of magnesium supplementation on testosterone levels of athletes and sedentary subjects at rest and after exhaustion. Biological Trace Element Research 2011 April;140(1):18-23. Diet Intervention or Supplement Study
Ref ID: 2464

(1063) Citrome L, Vreeland B. Schizophrenia, obesity, and antipsychotic medications: What can we do? Postgraduate Medicine 2008;120(2):18-33. Review article
Ref ID: 3206

(1064) Claeyssens S, Peynet J, Gazengel C, Alcalay M, Bertrand MA, Briquel ME, Derlon A, Guerois C, D'Oiron R, Pautard B, Stieltjes N, Sultan Y, Bridey F, Goudemand J. FACTEUR IX-LFB(TM): Experiment in surgery. Sang Thrombose Vaisseaux 1998;10:52-7. Drug intervention study
Ref ID: 919

(1065) Clapp JF, Kiess W. Cord blood leptin reflects fetal fat mass. Journal of the Society for Gynecologic Investigation 1998;5(6):300-3. Not an exercise intervention study
Ref ID: 5214

(1066) Clapp JF, III, Simonian S, Lopez B, Appleby-Wineberg S, Harcar-Sevcik R. The one-year morphometric and neurodevelopmental outcome of the offspring of women who continued to exercise regularly throughout pregnancy. American Journal of Obstetrics and Gynecology 1998 March;178(3):594-9. Subjects less

than 2 years old
Ref ID: 2098

(1067) Clar D, Cruciani D, Molinari J. Hidratación en la alta competencia: debito hidrosalino del tenista de alta competición. Revista Argentina de Medicina del Deporte 1995;17(56):24-30. Not an exercise intervention study
Ref ID: 4062

(1068) Clark EN, Dewey AM, Temple JL. Effects of daily snack food intake on food reinforcement depend on body mass index and energy density. American Journal of Clinical Nutrition 2010 February;91(2):300-8. Not an exercise intervention study
Ref ID: 73

(1069) Clark T, Fleming T, Bullen P, Crengle S, Denny S, Dyson B, Peiris-John R, Robinson E, Rossen F, Sheridan J, Teevale T, Utter J, Lewycka S. Health and well-being of secondary school students in New Zealand: trends between 2001, 2007 and 2012. Journal of Paediatrics and Child Health 2013 November;49(11):925-34. Inappropriate Study Design
Ref ID: 5805

(1070) Clarkson PM, Devaney JM, Gordish-Dressman H, Thompson PD, Hubal MJ, Urso M, Price TB, Angelopoulos TJ, Gordon PM, Moyna NM, Pescatello LS, Visich PS, Zoeller RF, Seip RL, Hoffman EP. ACTN3 genotype is associated with increases in muscle strength in response to resistance training in women. Journal of Applied Physiology 2005 July;99(1):154-63. Study limited to adults
Ref ID: 1490

(1071) Clarson CL, Mahmud FH, Baker JE, Clark HE, McKay WM, Schauteet VD, Hill DJ. Metformin in combination with structured lifestyle intervention improved body mass index in obese adolescents, but did not improve insulin resistance. Endocrine 2009 August;36(1):141-6. No exercise only group, No comparative control group
Ref ID: 125

(1072) Claudi-Magnussen C. The consumers' view/reaction. Acta Veterinaria Scandinavica 2006 January 2;48:S4-2. Abstract
Ref ID: 3552

(1073) Clauss SB, Holmes KW, Hopkins P, Stein E, Cho M, Tate A, Johnson-Levonas AO, Kwiterovich PO. Efficacy and safety of lovastatin therapy in adolescent girls with heterozygous familial hypercholesterolemia. Pediatrics 2005;116(3):682-8. Diet Intervention Study, Drug intervention study
Ref ID: 3207

(1074) Cleland V, Crawford D, Baur LA, Hume C, Timperio A, Salmon J. A prospective examination of children's time spent outdoors, objectively measured physical activity and overweight. International Journal of Obesity 2008

November;32(11):1685-93. Cross-sectional study
Ref ID: 859

(1075) Cleland V, Granados A, Crawford D, Winzenberg T, Ball K. Effectiveness of interventions to promote physical activity among socioeconomically disadvantaged women: a systematic review and meta-analysis. Obesity Reviews 2013 March;14(3):197-212. Inappropriate Study Design
Ref ID: 5806

(1076) Clemmens D, Hayman LL. Increasing activity to reduce obesity in adolescent girls: a research review. [Review] [39 refs]. Journal of Obstetric, Gynecologic, and Neonatal Nursing 33(6):801-8, 2004 Nov-Dec 2004;(6):801-8. Review article
Ref ID: 2921

(1077) Cliff DP, Wilson A, Okely AD, Mickle KJ, Steele JR. Feasibility of SHARK: a physical activity skill-development program for overweight and obese children. Journal of Science and Medicine in Sport 2007 August;10(4):263-7. Not a randomized controlled trial (RCT)
Ref ID: 1226

(1078) Cliff DP, Okely AD, Smith LM, McKeen K. Relationships between fundamental movement skills and objectively measured physical activity in preschool children. Pediatric Exercise Science 2009 November;21(4):436-49. Cross-sectional study
Ref ID: 614

(1079) Cliff DP, Okely AD, Morgan PJ, Steele JR, Jones RA, Colyvas K, Baur LA. Movement skills and physical activity in obese children: randomized controlled trial. Medicine and Science in Sports and Exercise 43(1):90-100, 2011 Jan 2011;(1):90-100. No control group (NC)
Ref ID: 2922

(1080) Clifton P. Dietary fatty acids and inflammation. Nutrition and Dietetics 2009 March;66(1):7-11. Review article
Ref ID: 3553

(1081) Clough GF, Turzyniecka M, Walter L, Krentz AJ, Wild SH, Chipperfield AJ, Gamble J, Byrne CD. Muscle microvascular dysfunction in central obesity is related to muscle insulin insensitivity but is not reversed by high-dose statin treatment. Diabetes 2009 May;58(5):1185-91. Study limited to adults, No exercise only group
Ref ID: 136

(1082) Coelho CM, Silva RCd, Egashira EM, Ribeiro SML. Evolução do estado nutricional de crianças com mielomeningoceleem período de três anos. Tree-year period evolution of the nutritional condition of children with mielomeningocelis. Evolución en tres años de la condición nutricional de niños

con mielomeningocele. Mundo Saúde (Impr) (1995) 2009 September;33(3):347-51. Cross-sectional study
Ref ID: 4063

(1083) Coffey VG, Jemiolo B, Edge J, Garnham AP, Trappe SW, Hawley JA. Effect of consecutive repeated sprint and resistance exercise bouts on acute adaptive responses in human skeletal muscle. American Journal of Physiology - Regulatory Integrative and Comparative Physiology 2009 November;297(5):R1441-R1451. Acute study
Ref ID: 653

(1084) Cohen D, Scott M, Wang FZ, McKenzie TL, Porter D. School design and physical activity among middle school girls. Journal of Physical Activity and Health 2008;5:719-31. Cross-sectional study
Ref ID: 4721

(1085) Cohen MS, Zak V, Atz AM, Printz BF, Pinto N, Lambert L, Pemberton V, Li JS, Margossian R, Dunbar-Masterson C, McCrindle BW. Anthropometric measures after Fontan procedure: implications for suboptimal functional outcome. American Heart Journal 2010 December;160(6):1092-8, 1098. Not a randomized controlled trial (RCT), Not an exercise intervention study
Ref ID: 6

(1086) Colado JC, Garcia-Masso X, Gonzalez LM, Triplett NT, Mayo C, Merce J. Two-leg squat jumps in water: an effective alternative to dry land jumps. International Journal of Sports Medicine 2010 February;31(2):118-22. Not a randomized controlled trial (RCT)
Ref ID: 572

(1087) Colak R, Ozcelik O. Effects of short-period exercise training and orlistat therapy on body composition and maximal power production capacity in obese patients. Physiological Research 2004;53(1):53-60. Diet Intervention Study, Drug intervention study
Ref ID: 1664

(1088) Cole CR, Rising R, Hakim A, Danon M, Mehta R, Choudhury S, Sundaresh M, Lifshitz F. Comprehensive assessment of the components of energy expenditure in infants using a new infant respiratory chamber. Journal of the American College of Nutrition 1999;18(3):233-41. Subjects less than 2 years old
Ref ID: 5215

(1089) Coledam DHC, Arruda GAd, Oliveira ARd. Efeito crônico do alongamento estático realizado durante o aquecimento sobre a flexibilidade de crianças. Chronic effect of static stretching performed during warm-up on flexibility in children. Revista Brasileira de Cineantropometria e Desempenho Humano

2012;14(3):296-304. Not a randomized controlled trial (RCT)
Ref ID: 4064

(1090) Coleman KJ, Raynor HR, Mueller DM, Cerny FJ, Dorn JM, Epstein LH. Providing sedentary adults with choices for meeting their walking goals. Preventive Medicine 1999 May;28(5):510-9. Study limited to adults
Ref ID: 2027

(1091) Coleman KJ, Heath EM, Alcalá IS. Overweight and aerobic fitness in children in the United States/Mexico border region. Revista Panamericana de Salud Pública 2004 April;15(4). Cross-sectional study
Ref ID: 4065

(1092) Colín RE, Castillo ML, Orea TA, Vergara CA, Keirns DC, Villa RA. Outcomes of a school-based intervention (RESCATE) to improve physical activity patterns in Mexican children aged 8-10 years. Health Education Research 2010;25:1042-9. Not All Participants were Overweight and/or Obese
Ref ID: 4722

(1093) Coll CdVN, Amorim TC, Hallal PC. Percepção de adolescentes e adultos referente à influência da mídia sobre o estilo de vida. Perception of adolescents and adults on the influence of media on lifestyle. Revista Brasileira de Atividade Física e Saúde 2010 June;15(2). Cross-sectional study
Ref ID: 4066

(1094) Collaer ML, Brook CG, Conway GS, Hindmarsh PC, Hines M. Motor development in individuals with congenital adrenal hyperplasia: strength, targeting, and fine motor skill. Psychoneuroendocrinology 2009;34:249-58. Not an exercise intervention study
Ref ID: 4723

(1095) Collaku A, Rankinen T, Rice T, Leon AS, Rao DC, Skinner JS, Wilmore JH, Bouchard C. A genome-wide linkage scan for dietary energy and nutrient intakes: The Health, Risk Factors, Exercise Training, and Genetics (HERITAGE) Family Study. American Journal of Clinical Nutrition 2004 May;79(5):881-6. Not an exercise intervention study
Ref ID: 1637

(1096) Collard DC, Verhagen EA, Chinapaw MJ, Knol DL, van MW. Effectiveness of a school-based physical activity injury prevention program: a cluster randomized controlled trial. Archives of Pediatrics and Adolescent Medicine 2010 February;164(2):145-50. Not All Participants were Overweight and/or Obese
Ref ID: 592

(1097) Collins AL, McCarthy HD. Evaluation of factors determining the precision of body composition measurements by air displacement plethysmography. European Journal of Clinical Nutrition 2003;57(6):770-6. Not an exercise

intervention study
Ref ID: 5216

(1098) Collins CE, Morgan PJ, Jones P, Fletcher K, Martin J, Aguiar EJ, Lucas A, Neve M, McElduff P, Callister R. Evaluation of a commercial web-based weight loss and weight loss maintenance program in overweight and obese adults: a randomized controlled trial. BMC Public Health 2010 November 3;10:669.:669. Study limited to adults
Ref ID: 14

(1099) Collins CE, Okely AD, Morgan PJ, Jones RA, Burrows TL, Cliff DP, Colyvas K, Warren JM, Steele JR, Baur LA. Parent diet modification, child activity, or both in obese children: an RCT. Pediatrics 2011 April;127(4):619-27. No non-intervention control group
Ref ID: 1083

(1100) Collins CE, Dewar DL, Schumacher TL, Finn T, Morgan PJ, Lubans DR. 12 month changes in dietary intake of adolescent girls attending schools in low-income communities following the NEAT Girls cluster randomized controlled trial. Appetite 2014 February;73:147-55. Inappropriate Intervention
Ref ID: 5807

(1101) Collins DL, Evans JM, Grundy RH. The efficiency of multiple impulse therapy for musculoskeletal complaints. Journal of Manipulative and Physiological Therapeutics 2006 February;29(2):162. Not an exercise intervention study
Ref ID: 1422

(1102) Colton P, Olmsted M, Daneman D, Rydall A, Rodin G. Disturbed eating behavior and eating disorders in preteen and early teenage girls with type 1 diabetes: a case-controlled study. Diabetes Care 2004 July;27(7):1654-9. Cross-sectional study
Ref ID: 1614

(1103) Comfort P, Pearson SJ, Mather D. An electromyographical comparison of trunk muscle activity during isometric trunk and dynamic strengthening exercises. Journal of Strength and Conditioning Research 2011 January;25(1):149-54. Study limited to adults
Ref ID: 2469

(1104) Comité Nacional de Medicina del Deporte Infanto-Juvenil.Subcomité de Emidemiología. Consenso sobre factores de riesgo de enfermedad cardiovascular en pediatría. Sedentarismo. Archivos Argentinos de Pediatría 2005 May;103(5):450-63. Review article
Ref ID: 4067

(1105) Conde WL, Borges C. O risco de incidência e persistência da obesidade entre adultos Brasileiros segundo seu estado nutricional ao final da adolescência. The risk of incidence and persistence of obesity among Brazilian adults

according to their nutritional status at the end of adolescence. Revista Brasileira de Eidemiologia 2011 September;14(supl.1):71-9. Not an exercise intervention study
Ref ID: 4068

(1106) Conkin J, Powell MR, Gernhardt ML. Age affects severity of venous gas emboli on decompression from 14.7 to 4.3 psia. Aviation Space and Environmental Medicine 2003 November;74(11):1142-50. Study limited to adults
Ref ID: 1702

(1107) Conlon BA, Beasley JM, Aebersold K, Jhangiani SS, Wylie-Rosett J. Nutritional management of insulin resistance in nonalcoholic fatty liver disease (NAFLD). Nutrients 2013 October;5(10):4093-114. Inappropriate Outcomes
Ref ID: 5808

(1108) Connelly JB, Duaso MJ, Butler G. A systematic review of controlled trials of interventions to prevent childhood obesity and overweight: a realistic synthesis of the evidence. [Review] [43 refs]. Public Health 2007 July;121(7):510-7. Review article
Ref ID: 1212

(1109) Conners CK, Casat CD, Gualtieri CT, Weller E, Reader M, Reiss A, Weller RA, Khayrallah M, Ascher J. Bupropion hydrochloride in attention deficit disorder with hyperactivity. Journal of the American Academy of Child and Adolescent Psychiatry 1996;35:1314-21. Drug intervention study
Ref ID: 4724

(1110) Connolly AM, Schierbecker J, Renna R, Florence J. High dose weekly oral prednisone improves strength in boys with Duchenne muscular dystrophy. Neuromuscular Disorders 2002 December;12(10):917-25. Drug intervention study
Ref ID: 1791

(1111) Conrod PJ, Peterson JB, Pihl RO. Reliability and validity of alcohol-induced heart rate increase as a measure of sensitivity to the stimulant properties of alcohol. Psychopharmacology 2001 July 15;157(1):20. Drug intervention study
Ref ID: 486

(1112) Conte M, Gonçalves A, Aragon FF, Padovani CR. Influência da massa corporal sobre a aptidão física em adolescentes: estudo a partir de escolares do ensino fundamental e médio de Sorocaba/SP. Revista Brasileira de Medicina do Esporte 2000 April;6(2):44-9. Cohort Study
Ref ID: 4069

(1113) Contento IR, Koch PA, Lee H, Calabrese-Barton A. Adolescents demonstrate improvement in obesity risk behaviors after completion of choice, control & change, a curriculum addressing personal agency and autonomous motivation. Journal of the American Dietetic Association 2010 December;110(12):1830-9.

No exercise only group, Not an exercise intervention study
Ref ID: 8

(1114) Contopoulos-Ioannidis DG, Seto I, Hamm MP, Thomson D, Hartling L, Ioannidis JPA, Curtis S, Constantin E, Batmanabane G, Klassen T, Williams K. Empirical evaluation of age groups and age-subgroup analyses in pediatric randomized trials and pediatric meta-analyses. Pediatrics 2012;129(SUPPL. 3):S161-S184. Review article
Ref ID: 3208

(1115) Conwell LS, Trost SG, Spence L, Brown WJ, Batch JA. The feasibility of a home-based moderate-intensity physical activity intervention in obese children and adolescents. British Journal of Sports Medicine 2010 March;44(4):250-5. Not a randomized controlled trial (RCT)
Ref ID: 563

(1116) Cooper JA, Watras AC, Shriver T, Adams AK, Schoeller DA. Influence of dietary fatty acid composition and exercise on changes in fat oxidation from a high-fat diet. Journal of Applied Physiology 2010 October;109(4):1011-8. Study limited to adults
Ref ID: 2471

(1117) Cooper JA, Watras AC, Paton CM, Wegner FH, Adams AK, Schoeller DA. Impact of exercise and dietary fatty acid composition from a high-fat diet on markers of hunger and satiety. Appetite 2011 February;56(1):171-8. Study limited to adults
Ref ID: 2472

(1118) Cooper JL. Dietary lipids in the aetiology of Alzheimers disease: Implications for therapy. Drugs and Aging 2003 April;20(6):399-418. Review article
Ref ID: 3554

(1119) Cooper L, Lockwood B. Difficulty breathing? Just get eating. Pharmaceutical Journal 2006;276(7402):629-36. Not an exercise intervention study
Ref ID: 3209

(1120) Cooper R, Hypponen E, Berry D, Power C. Associations between parental and offspring adiposity up to midlife: the contribution of adult lifestyle factors in the 1958 British Birth Cohort Study. American Journal of Clinical Nutrition 2010;92(4):946-53. Cohort Study
Ref ID: 5217

(1121) Copeland KC, Zeitler P, Geffner M, Guandalini C, Higgins J, Hirst K, Kaufman FR, Linder B, Marcovina S, McGuigan P, Pyle L, Tamborlane W, Willi S, -TODAY-Study-Group. Characteristics of adolescents and youth with recent-onset type 2 diabetes: the TODAY cohort at baseline. The Journal of Clinical Endocrinology and Metabolism 2011;96:159-67. Cohort Study
Ref ID: 4725

(1122) Copetti J, Neutzling MB, Silva MCd. Barreiras à prática de atividades físicas em adolescentes de uma cidade do sul do Brasil. Barriers to physical activity practice in adolescents of southern Brazilian city. Revista Brasileira de Atividade Física e Saúde 2010 June;15(2). Cross-sectional study
Ref ID: 4070

(1123) Coppins DF, Margetts BM, Fa JL, Brown M, Garrett F, Huelin S. Effectiveness of a multi-disciplinary family-based programme for treating childhood obesity (the Family Project). European Journal of Clinical Nutrition 2011 August;65(8):903-9. Lifestyle Intervention
Ref ID: 2473

(1124) Corapcioglu F, Guvenc BH, Sarper N, Aydogan A, Akansel G, Arisoy ES. Peritoneal tuberculosis with elevated serum CA 125 level mimicking advanced ovarian carcinoma in an adolescent. Turkish Journal of Pediatrics 2006;48(1):69-72. Not an exercise intervention study
Ref ID: 5218

(1125) Corbo GM, Forastiere F, Rusconi F, De SM, Biggeri A, Russo A, Chellini E, Brunetti L, Gruppo Collaborativo SIDR. [Dietary habits, life styles and respiratory symptoms in childhood]. [Italian]. Epidemiologia e Prevenzione 2005 March;29(2:Suppl):Suppl-6. Cross-sectional study
Ref ID: 1476

(1126) Corbo GM, Forastiere F, De SM, Brunetti L, Bonci E, Bugiani M, Chellini E, La GS, Migliore E, Pistelli R, Rusconi F, Russo A, Simoni M, Talassi F, Galassi C, Collaborative Group. Wheeze and asthma in children: associations with body mass index, sports, television viewing, and diet. Epidemiology 2008 September;19(5):747-55. Survey or questionnaire
Ref ID: 899

(1127) Corella D, Carrasco P, Sorli JV, Coltell O, Ortega-Azorin C, Guillen M, Gonzalez JI, Saiz C, Estruch R, Ordovas JM. Education modulates the association of the FTO rs9939609 polymorphism with body mass index and obesity risk in the Mediterranean population. Nutrition, Metabolism and Cardiovascular Diseases 2012 August;22(8):651-8. Inappropriate Study Design
Ref ID: 5809

(1128) Cornish SM, Chilibeck PD, Burke DG. The effect of creatine monohydrate supplementation on sprint skating in ice-hockey players. Journal of Sports Medicine and Physical Fitness 2006 March;46(1):90-8. Diet Intervention or Supplement Study
Ref ID: 1401

(1129) Corr M, De Souza MJ, Toombs RJ, Williams NI. Circulating leptin concentrations do not distinguish menstrual status in exercising women.

Human Reproduction 2011 March;26(3):685-94. Study limited to adults
Ref ID: 2474

(1130) Correa B. Determinación del perfil antropométrico y cualidades físicas de niños futbolistas de Bogotá. Determination of the anthropometric and physical qualities profile in footballers children of Bogotá. Revista Ciencias de la Salud (Bogota) 2008 August;6(2):74-84. Cross-sectional study
Ref ID: 4071

(1131) Correia CT, Almeida JP, Santos PE, Sequeira AF, Marques CE, Miguel TS, Abreu RL, Oliveira GG, Vicente AM. Pharmacogenetics of risperidone therapy in autism: association analysis of eight candidate genes with drug efficacy and adverse drug reactions. Pharmacogenomics Journal 2010 October;10(5):418-30. Drug intervention study
Ref ID: 3555

(1132) Corsino L, Lin PH, Batch BC, Intille S, Grambow SC, Bosworth HB, Bennett GG, Tyson C, Svetkey LP, Voils CI. Recruiting young adults into a weight loss trial: report of protocol development and recruitment results. Contemporary Clinical Trials 2013 July;35(2):1-7. Inappropriate Population
Ref ID: 5810

(1133) Corso ACT, Caldeira GV, Fiates GMR, Schmitz BdAS, Ricardo GD, Vasconcelos FdAGd. Fatores comportamentais associados ao sobrepeso e à obesidade em escolares do Estado de Santa Catarina. Behavioral factors associated with overweight and with obesity in students in the State of Santa Catarina. Factores de comportamiento asociados al sobrepeso y a la obesidad en escolares del Estado de Santa Catarina. Revista Brasileira de Estudos dePopulacao 2012 June;29(1):117-31. Cross-sectional study
Ref ID: 4072

(1134) Corte de Araujo AC, Roschel H, Picanco AR, do Prado DM, Villares SM, de Sa Pinto AL, Gualano B. Similar health benefits of endurance and high-intensity interval training in obese children. PLoS ONE 2012;7(8):e42747. Inappropriate Comparison Group
Ref ID: 5811

(1135) Cosgrove MJ, Wilson J, Watt D, Grant SF. The relationship between selected physiological variables of rowers and rowing performance as determined by a 2000 m ergometer test. Journal of Sports Sciences 1999 November;17(11):845-52. Not a randomized controlled trial (RCT)
Ref ID: 2060

(1136) Cossio-Bolaños M, Figueroa P, Cossio-Bolaños WJ, Lázari E, Arruda M. Parámetros del crecimiento físico de niños que viven a moderada altitud. Revista Médica Herediana 2012 June;23(2):96-105. Cross-sectional study
Ref ID: 4073

(1137) Costa ARCD, Teodoro TN, Araújo MDFMD. Análise dos conhecimentos e da prática de profissionais de saúde na promoção e no apoio à amamentação: Estudo de revisão. Comunicação em Ciências da Saúde 2009 March;20(1):55-63. Review article
Ref ID: 4074

(1138) Costa FFd, Assis MAAd. Nível de atividade física e comportamentos sedentários de escolares de sete a dez anos de Florianópolis-SC. Physical activity and sedentary behaviour of schoolchildren aged 7 to 10 in Florianópolis-SC. Revista Brasileira de Atividade Física e Saúde 2011 March;16(1). Survey or questionnaire
Ref ID: 4075

(1139) Costa MF, Barbosa SCT, Barletta M, Dantas DV, Kehrig HA, Seixas TG, Malm O. Seasonal differences in mercury accumulation in Trichiurus lepturus (Cutlassfish) in relation to length and weight in a Northeast Brazilian estuary. Environmental Science and Pollution Research 2009;16(4):423-30. Not an exercise intervention study
Ref ID: 5219

(1140) Costa MCD, Cordoni Junior L, Matsuo T. Sobrepeso em adolescentes de 14 a 19 anos em um município da região Sul do Brasil. Overweight in adolescents aged 14 to 19 years old in a Southern Brazilian city. Revista Brasileira de Saúde Materno Infantil 2007 September;7(3):263-70. Cross-sectional study
Ref ID: 4076

(1141) Costacurta M, Di Renzo L, Bianchi A, Fabiocchi F, De Lorenzo A, Docimo R. Obesity and dental caries in paediatric patients. A cross-sectional study. European Journal of Paediatric Dentistry 2011;12(2):112-6. Cross-sectional study
Ref ID: 5220

(1142) Costanzi CB, Halpern R, Rech RR, Bergmann MLdA, Alli LR, Mattos APd. Fatores associados a níveis pressóricos elevados em escolares de uma cidade de porte médio do sul do Brasil. Associated factors in high blood pressure among schoolchildren in a middle size city, southern Brazil. Jornal de Pediatria 2009 August;85(4):335-40. Cross-sectional study
Ref ID: 4077

(1143) Coté CJ, Rolf N, Liu LM, Goudsouzian NG, Ryan JF, Zaslavsky A, Gore R, Todres TD, Vassallo S, Polaner D. A single-blind study of combined pulse oximetry and capnography in children. Anesthesiology 1991;74:980-7. Drug intervention study
Ref ID: 4726

(1144) Cotton B, Smith A, Hansen I, Davis C, Doyle A, Walsh A. Physician-directed primary care intervention to reduce risk factors for type 2 diabetes in high-risk

youth. American Journal of the Medical Sciences 2006 September;332(3):108-11. No control group (NC)
Ref ID: 1349

(1145) Cottrell L, Spangler-Murphy E, Minor V, Downes A, Nicholson P, Neal WA. A kindergarten cardiovascular risk surveillance study: CARDIAC-Kinder. American Journal of Health Behavior 2005 November;29(6):595-606. Lifestyle Intervention
Ref ID: 1443

(1146) Coudreau SK, Tounian P, Bonhomme G, Froguel P, Girardet JP, Guy-Grand B, Basdevant A, Clement K. Role of the DGAT gene C79T single-nucleotide polymorphism in french obese subjects. Obesity Research 2003;11(10):1163-7. Not an exercise intervention study
Ref ID: 5221

(1147) Coukell AJ, Brogden RN. Liposomal Amphotericin B: Therapeutic Use in the Management of Fungal Infections and Visceral Leishmaniasis. Drugs 1998 April;55(4):585-612. Drug intervention study
Ref ID: 558

(1148) Coulon SM, Wilson DK, Egan BM. Associations among environmental supports, physical activity, and blood pressure in African-American adults in the PATH trial. Social Science and Medicine 2013 June;87:108-15. Inappropriate Population
Ref ID: 5812

(1149) Counterweight Project Team. Evaluation of the Counterweight Programme for obesity management in primary care: a starting point for continuous improvement. British Journal of General Practice 2008 August;58(553):548-54. Not a randomized controlled trial (RCT)
Ref ID: 903

(1150) Courneya KS, Sellar CM, Stevinson C, McNeely ML, Peddle CJ, Friedenreich CM, Tankel K, Basi S, Chua N, Mazurek A, Reiman T. Randomized controlled trial of the effects of aerobic exercise on physical functioning and quality of life in lymphoma patients. Journal of Clinical Oncology 2009 September 20;27(27):4605-12. Study limited to adults
Ref ID: 666

(1151) Courneya KS, Stevinson C, McNeely ML, Sellar CM, Peddle CJ, Friedenreich CM, Mazurek A, Chua N, Tankel K, Basi S, Reiman T. Predictors of adherence to supervised exercise in lymphoma patients participating in a randomized controlled trial. Annals of Behavioral Medicine 2010 August;40(1):30-9. Study not limited to children and adolescents
Ref ID: 36

(1152) Courteix D, Lespessailles E, Loiseau-Peres S, Obert P, Ferry B, Benhamou CL. Lean tissue mass is a better predictor of bone mineral content and density than body weight in prepubertal girls. Revue du Rhumatisme (English Edition) 1998 May;65(5):328-36. Not a randomized controlled trial (RCT)
Ref ID: 2088

(1153) Courteix D, Jaffre C, Lespessailles E, Benhamou L. Cumulative effects of calcium supplementation and physical activity on bone accretion in premenarchal children: a double-blind randomised placebo-controlled trial. International Journal of Sports Medicine 2005 June;26(5):332-8. Not All Participants were Overweight and/or Obese
Ref ID: 1519

(1154) Couser RJ, Hoekstra RE, Ferrara TB, Wright GB, Cabalka AK, Connett JE. Neurodevelopmental follow-up at 36 months' corrected age of preterm infants treated with prophylactic indomethacin. Archives of Pediatrics and Adolescent Medicine 2000;154:598-602. Subjects less than 2 years old
Ref ID: 4727

(1155) Cousins JH, Rubovits DS, Dunn JK, Reeves RS, Ramirez AG, Foreyt JP. Family versus individually oriented intervention for weight loss in Mexican American women. Public Health Reports 1992 September;107(5):549-55. Study limited to adults
Ref ID: 437

(1156) Cousins JM, Langer SM, Rhew LK, Thomas C. The role of state health departments in supporting community-based obesity prevention. Preventing Chronic Disease 2011 July;8(4):A87. Not an exercise intervention study
Ref ID: 2475

(1157) Coutant R, Carel JC, Timsit J, Boitard C, Bougneres P. Insulin and the prevention of insulin-dependent diabetes mellitus. [Review] [20 refs]. Diabetes and Metabolism 1997 September;23:Suppl-8. Review article
Ref ID: 2122

(1158) Coutinho W. The first decade of sibutramine and orlistat: a reappraisal of their expanding roles in the treatment of obesity and associated conditions. [Review] [60 refs]. Arquivos Brasileiros de Endocrinologia e Metabologia 2009 March;53(2):262-70. Review article
Ref ID: 746

(1159) Coutinho W. The first decade of sibutramine and orlistat: a reappraisal of their expanding roles in the treatment of obesity and associated conditions.
A primeira década da sibutramina e do orlistate: reavaliação do seu crescente papel no tratamento da obesidade e condições associadas. Arquivos Brasileiros de Endocrinologia and Metabologia 2009 March;53(2):262-70. Drug

intervention study
Ref ID: 4078

(1160) Coutts AJ, Murphy AJ, Dascombe BJ. Effect of direct supervision of a strength coach on measures of muscular strength and power in young rugby league players. Journal of Strength and Conditioning Research 2004 May;18(2):316-23. Not a randomized controlled trial (RCT)
Ref ID: 1629

(1161) Cowie RL, Boulet LP, Keith PK, Scott-Wilson CA, House KW, Dorinsky PM. Tolerability of a salmeterol xinafoate/fluticasone propionate hydrofluoroalkane metered-dose inhaler in adolescent and adult patients with persistent asthma: a 52-week, open-label, stratified, parallel-group, multicenter study. Clinical Therapeutics 2007;29:1390-402. Drug intervention study
Ref ID: 4728

(1162) Cox G, Jenkins DG. The physiological and ventilatory responses to repeated 60 s sprints following sodium citrate ingestion. Journal of Sports Sciences 1994 October;12(5):469-75. Diet Intervention or Supplement Study
Ref ID: 2227

(1163) Cox JH, Cortright RN, Dohm GL, Houmard JA. Effect of aging on response to exercise training in humans: skeletal muscle GLUT-4 and insulin sensitivity. Journal of Applied Physiology 1999 June;86(6):2019-25. Study limited to adults
Ref ID: 2020

(1164) Coyne KS, Kaplan SA, Chapple CR, Sexton CC, Kopp ZS, Bush EN, Aiyer LP, EpiLUTS Team. Risk factors and comorbid conditions associated with lower urinary tract symptoms: EpiLUTS. BJU International 2009 April;103:Suppl-32. Study limited to adults
Ref ID: 783

(1165) Coyote-Estrada N, Liliana Miranda-Lora Ar. Tratamiento farmacologico de la obesidad en niÃ±os. (Spanish). Boletin Medico del Hospital Infantil de Mexico 2008 November;65(6):547-67. Drug intervention study
Ref ID: 3556

(1166) Cradock AL, Kawachi I, Colditz GA, Gortmaker SL, Buka SL. Neighborhood social cohesion and youth participation in physical activity in Chicago. Social Science and Medicine 2009;68(3):427-35. Not a randomized controlled trial (RCT)
Ref ID: 5222

(1167) Cradock AL, Melly SJ, Allen JG, Morris JS, Gortmaker SL. Youth destinations associated with objective measures of physical activity in adolescents. Journal of Adolescent Health 2009 September;45(3:Suppl):Suppl-8. Not a randomized controlled trial (RCT)
Ref ID: 692

(1168) Craig IS, Morgan DW. Relationship between 800-m running performance and accumulated oxygen deficit in middle-distance runners. Medicine and Science in Sports and Exercise 1998 November;30(11):1631-6. Study limited to adults
Ref ID: 2075

(1169) Craigie AM, Macleod M, Barton KL, Treweek S, Anderson AS, WeighWell team. Supporting postpartum weight loss in women living in deprived communities: design implications for a randomised control trial. European Journal of Clinical Nutrition 2011 August;65(8):952-8. Study limited to adults
Ref ID: 1093

(1170) Crawford D, Cleland V, Timperio A, Salmon J, Andrianopoulos N, Roberts R, Giles-Corti B, Baur L, Ball K. The longitudinal influence of home and neighbourhood environments on children's body mass index and physical activity over 5 years: the CLAN study. International Journal of Obesity 2010 July;34(7):1177-87. Not an exercise intervention study
Ref ID: 493

(1171) Crawford PB, Gosliner W, Strode P, Samuels SE, Burnett C, Craypo L, Yancey AK. Walking the talk: Fit WIC wellness programs improve self-efficacy in pediatric obesity prevention counseling. American Journal of Public Health 2004 September;94(9):1480-5. Survey or questionnaire
Ref ID: 1594

(1172) Cremonini F, Camilleri M, Clark MM, Beebe TJ, Locke GR, Zinsmeister AR, Herrick LM, Talley NJ. Associations among binge eating behavior patterns and gastrointestinal symptoms: a population-based study.[Erratum appears in International Journal of Obesity (London). 2010 Jan;34(1):214]. International Journal of Obesity 2009 March;33(3):342-53. Diet Intervention Study
Ref ID: 787

(1173) Crespo NC, Corder K, Marshall S, Norman GJ, Patrick K, Sallis JF, Elder JP. An examination of multilevel factors that may explain gender differences in children's physical activity. Journal of Physical Activity and Health 2013 September;10(7):982-92. Inappropriate Study Design
Ref ID: 5813

(1174) Crider LB, Hall AK. Street Wise Part 1: Promoting Safe Bicycling and Walking to School. Teaching Elementary Physical Education 2005 May 1;16(3):8-11. Editorial or letter or comment
Ref ID: 3899

(1175) Criswell D, Powers S, Lawler J, Tew J, Dodd S, Iryiboz Y, Tulley R, Wheeler K. Influence of a carbohydrate-electrolyte beverage on performance and blood homeostasis during recovery from football. International Journal of Sport Nutrition 1991 June;1(2):178-91. Diet Intervention or Supplement Study
Ref ID: 2294

(1176) Crocker MK, Yanovski JA. Pediatric Obesity: Etiology and Treatment. Endocrinol Metab Clin North Am 2009;38(3):525-48. Review article
Ref ID: 3210

(1177) Croti UA, Beani L, Moscardini AC, Souza Júnior AS, Souza AS, Sobrinho SH, De Marchi CH, Godoy MFd, Braile DM. Tomografia computadorizada na avaliação tardia do tratamento cirúrgico da conexão anômala total de veias pulmonares. Computed tomography in late evaluation of surgical treatment of pulmonary veins total anomalous connection. Revista Brasileira de Cirurgia Cardiovascular 2011 December;26(4):532-43. Not an exercise intervention study
Ref ID: 4079

(1178) Crova C, Struzzolino I, Marchetti R, Masci I, Vannozzi G, Forte R, Pesce C. Cognitively challenging physical activity benefits executive function in overweight children. Journal of Sports Sciences 2014 February;32(3):201-11. Inappropriate Population
Ref ID: 5814

(1179) Crum AJ, Langer EJ. Mind-set matters: exercise and the placebo effect. Psychological Science 2007 February;18(2):165-71. Study limited to adults
Ref ID: 1248

(1180) Cruvinel MGC, Bittencourt PFS, Costa JRdR, Barbosa PRV. Volume gástrico residual e risco de aspiração pulmonar em crianças com refluxo gastroesofágico: estudo comparative. Residual gastric volume and risk for pulmonary aspiration in children with gastroesophageal reflux: comparative study. Revista Brasileira de Anestesiologia 2004 February;54(1):37-42. Not an exercise intervention study
Ref ID: 713

(1181) Cullberg KB, Christiansen T, Paulsen SK, Bruun JM, Pedersen SB, Richelsen B. Effect of weight loss and exercise on angiogenic factors in the circulation and in adipose tissue in obese subjects. Obesity (Silver Spring) 2013 March;21(3):454-60. Inappropriate Intervention
Ref ID: 5815

(1182) Culnane M, Fowler M, Lee SS, McSherry G, Brady M, O'Donnell K, Mofenson L, Gortmaker SL, Shapiro DE, Scott G, Jimenez E, Moore EC, Diaz C, Flynn PM, Cunningham B, Oleske J. Lack of long-term effects of in utero exposure to zidovudine among uninfected children born to HIV-infected women. Pediatric AIDS Clinical Trials Group Protocol 219/076 Teams. Journal of the American Medical Association 1999;281:151-7. Cohort Study
Ref ID: 4729

(1183) Cumbá Abréu Cdl, Betancourt Vega CO, Díaz Castrillo O, Pommerenck Martínez CW. Capacidad física de trabajo en niños asmáticos. Revista Cubana

de Higiene y Epidemiología 1986 September;24(3):353-61. Not an exercise intervention study
Ref ID: 4080

(1184) Cummings DE, Merriam GR. Growth Hormone Therapy in Adults. 54, 513-533. 2003. Review article,
Ref ID: 3211

(1185) Cundiff DK, Nigg CR. Diet and diabetic retinopathy: insights from the Diabetes Control and Complications Trial (DCCT). Medgenmed [Computer File]: Medscape General Medicine 2005;7(1):3. Lifestyle Intervention
Ref ID: 1438

(1186) Cuneo RC, Salomon F, Wiles CM, Hesp R, Sonksen PH. Growth hormone treatment in growth hormone-deficient adults. II. Effects on exercise performance. Journal of Applied Physiology 1991 February;70(2):695-700. Study limited to adults
Ref ID: 2301

(1187) Cunha AMRd, Lemônica L. Incidência da síndrome da mama fantasma e suas características clínicas. Revista Brasileira de Mastologia 2002 December;12(4):29-38. Retrospective study
Ref ID: 4081

(1188) Cunha MT, Santos ACd, Silva GFC, Oehlmeyer KD, Baldo TMI. Teste de caminhada de seis minutos (TC6') em criança obesa: relato de caso. Pediatria (São Paulo) 2009 September;31(3):214-8. Case-Control / Case Study
Ref ID: 4082

(1189) Cunningham PM, Brennan D, O'Connell M, MacMahon P, O'Neill P, Eustace S. Patterns of bone and soft-tissue injury at the symphysis pubis in soccer players: observations at MRI. AJR American Journal of Roentgenology 2007 March;188(3):W291-W296. Not an exercise intervention study
Ref ID: 1272

(1190) Cusick A, McIntyre S, Novak I, Lannin N, Lowe K. A comparison of goal attainment scaling and the Canadian Occupational Performance Measure for paediatric rehabilitation research. Pediatric rehabilitation 2006;9:149-57. No non-intervention control group
Ref ID: 4730

(1191) Cutler JA. Randomized clinical trials of weight reduction in nonhypertensive persons. Annals of Epidemiology 1991;1:363-70. Review article
Ref ID: 4731

(1192) D'Adamo E, Chiarelli F, Mohn A. Treatment of non-alcoholic fatty liver disease (NAFLD) in the paediatric population. Recent Pat Endocr Metab Immune Drug

Discov 2009;3(2):94-101. Review article
Ref ID: 3212

(1193) D'Anci KE. Nutrition Updates. Nutrition Reviews 2008 January;66(1):60-3. Abstract
Ref ID: 3557

(1194) D'Argent J. Gastric electrical stimulation as therapy of morbid obesity: preliminary results from the French study. Obesity Surgery 2002 April;12:Suppl-25S. Not an exercise intervention study
Ref ID: 1839

(1195) D'Avanzo B, Nanni O, La VC, Franceschi S, Negri E, Giacosa A, Conti E, Montella M, Talamini R, Decarli A. Physical activity and breast cancer risk. Cancer Epidemiology, Biomarkers and Prevention 1996 March;5(3):155-60. Case-Control / Case Study
Ref ID: 2163

(1196) D'Hondt E, Segers V, Deforche B, Shultz SP, Tanghe A, Gentier I, De Bourdeaudhuij I, De Clercq D, Lenoir M. The role of vision in obese and normal-weight children's gait control. Gait and Posture 2011;33(2):179-84. Not All Participants were Overweight and/or Obese
Ref ID: 5224

(1197) D'Hondt E, Deforche B, Gentier I, De B, I, Vaeyens R, Philippaerts R, Lenoir M. A longitudinal analysis of gross motor coordination in overweight and obese children versus normal-weight peers. International Journal of Obesity (London) 2013 January;37(1):61-7. Inappropriate Study Design
Ref ID: 5816

(1198) D'hooge R, Hellinckx T, Van LC, Stegen S, De SJ, Van AS, Dewolf D, Calders P. Influence of combined aerobic and resistance training on metabolic control, cardiovascular fitness and quality of life in adolescents with type 1 diabetes: a randomized controlled trial. Clinical Rehabilitation 25(4):349-59, 2011 Apr 2011;(4):349-59. Not All Participants were Overweight and/or Obese
Ref ID: 2927

(1199) Da Silva ME, Fernandez JM, Castillo E, Nunez VM, Vaamonde DM, Poblador MS, Lancho JL. Influence of vibration training on energy expenditure in active men. Journal of Strength and Conditioning Research 21(2):470-5, 2007 May 2007;(2):470-5. No control group (NC)
Ref ID: 2928

(1200) Dahlkoetter J, Callahan EJ, Linton J. Obesity and the unbalanced energy equation: exercise versus eating habit change. Journal of Consulting and Clinical Psychology 1979 October;47(5):898-905. Not All Participants were Overweight and/or Obese
Ref ID: 2380

(1201) Dai J, Jiang Z, Zhang B. [Exercise and nutrition therapy for simple obesity in children]. Chinese Journal of Clinical Rehabilitation 2006;10:20-2. Primary outcome(s) not assessed
Ref ID: 4732

(1202) Dale D, Corbin CB, Dale KS. Restricting opportunities to be active during school time: do children compensate by increasing physical activity levels after school? Research Quarterly for Exercise and Sport 2000;71:240-8. Not a randomized controlled trial (RCT)
Ref ID: 4733

(1203) Daley AJ, Mutrie N, Crank H, Coleman R, Saxton J. Exercise therapy in women who have had breast cancer: design of the Sheffield women's exercise and well-being project. Health Education Research 2004 December;19(6):686-97. Study limited to adults
Ref ID: 1570

(1204) Daley AJ, Copeland RJ, Wright NP, Wales JK. Protocol for: Sheffield Obesity Trial (SHOT): a randomised controlled trial of exercise therapy and mental health outcomes in obese adolescents [ISRCNT83888112]. BMC Public Health 2005;5:113. Description of study from review or magazine or etc. (not the actual study)
Ref ID: 1440

(1205) Daley AJ, Copeland RJ, Wright NP, Roalfe A, Wales JK. Exercise therapy as a treatment for psychopathologic conditions in obese and morbidly obese adolescents: a randomized, controlled trial. Pediatrics 2006 November;118(5):2126-34. Inappropriate Outcomes
Ref ID: 264

(1206) Daley AJ, Copeland RJ, Wright NP, Wales JK. 'I can actually exercise if I want to; it isn't as hard as I thought': a qualitative study of the experiences and views of obese adolescents participating in an exercise therapy intervention. Journal of Health Psychology 2008 September;13(6):810-9. Not a randomized controlled trial (RCT)
Ref ID: 162

(1207) Dallocchio C, Arbasino C, Klersy C, Marchioni E. The effects of physical activity on psychogenic movement disorders. Movement Disorders 2010;25(4):421-5. Study limited to adults
Ref ID: 3213

(1208) Dalskov SM, Muller M, Ritz C, Damsgaard CT, Papadaki A, Saris WH, Astrup A, Michaelsen KF, Molgaard C. Effects of dietary protein and glycaemic index on biomarkers of bone turnover in children. British Journal of Nutrition 2014 April 14;111(7):1253-62. Inappropriate Intervention
Ref ID: 5817

(1209) Daly HB. Laboratory Rat Experiments Show Consumption of Lake-Ontario Salmon Causes Behavioral-Changes - Support for Wildlife and Human Research Results. Journal of Great Lakes Research 1993;19(4):784-8. Animal study
Ref ID: 5225

(1210) Damiani D. Uso de hormônio de crescimento na síndrome de Prader-Willi: [revisão]. Arquivos Brasileiros de Endocrinologia and Metabologia 2008 July;52(5):833-8. Review article
Ref ID: 4083

(1211) Damiano DL, Dodd K, Taylor NF. Should we be testing and training muscle strength in cerebral palsy? Developmental Medicine and Child Neurology 2002;44:68-72. Review article
Ref ID: 4734

(1212) Damsgaard CT, Dalskov SM, Petersen RA, Sorensen LB, Molgaard C, Biltoft-Jensen A, Andersen R, Thorsen AV, Tetens I, Sjodin A, Hjorth MF, Vassard D, Jensen JD, Egelund N, Dyssegaard CB, Skovgaard I, Astrup A, Michaelsen KF. Design of the OPUS School Meal Study: a randomised controlled trial assessing the impact of serving school meals based on the New Nordic Diet. Scandinavian Journal of Public Health 2012 December;40(8):693-703. Inappropriate Intervention
Ref ID: 5818

(1213) Damsgaard CT, Molgaard C, Matthiessen J, Gyldenlove SN, Lauritzen L. The effects of n-3 long-chain polyunsaturated fatty acids on bone formation and growth factors in adolescent boys. Pediatric Research 2012;71(6):713-9. Diet Intervention or Supplement Study
Ref ID: 5226

(1214) Damsgaard CT, Papadaki A, Jensen SM, Ritz C, Dalskov SM, Hlavaty P, Saris WH, Martinez JA, Handjieva-Darlenska T, Andersen MR, Stender S, Larsen TM, Astrup A, Molgaard C, Michaelsen KF. Higher protein diets consumed ad libitum improve cardiovascular risk markers in children of overweight parents from eight European countries. Journal of Nutrition 2013 June;143(6):810-7. Inappropriate Intervention
Ref ID: 5819

(1215) Danda AK, -S-R, Chinnaswami R. Comparison of gap arthroplasty with and without a temporalis muscle flap for the treatment of ankylosis. Journal of Oral and Maxillofacial Surgery 2009;67:1425-31. Not an exercise intervention study
Ref ID: 4735

(1216) Danel C, Moh R, Minga A, Anzian A, Ba-Gomis O, Kanga C, Nzunetu G, Gabillard D, Rouet Fo, Sorho S, Chaix ML, Eholie S, Menan H, Sauvageot D, Bissagnene E, Salamon R, Anglaret X. CD4-guided structured antiretroviral

treatment interruption strategy in HIV-infected adults in west Africa (Trivacan ANRS 1269 trial): a randomised trial. Lancet 2006 June 17;367(9527):1981-9. Study limited to adults
Ref ID: 3558

(1217) Dangi CBS, Firodiya A. Triple-Negative Breast Cancer and it's Therapeutic Options. International Journal of Pharmaceutical and BioSciences 2012 April 13;3(2):B. Not an exercise intervention study
Ref ID: 3559

(1218) Daniels LA, Wilson JL, Mallan KM, Mihrshahi S, Perry R, Nicholson JM, Magarey A. Recruiting and engaging new mothers in nutrition research studies: lessons from the Australian NOURISH randomised controlled trial. International Journal of Behavioral Nutrition and Physical Activity 2012;9:129. Inappropriate Population
Ref ID: 5820

(1219) Daniels S. Pharmacological treatment of obesity in paediatric patients. [Review] [33 refs]. Paediatric Drugs 2001;3(6):405-10. Review article
Ref ID: 1908

(1220) Daniels SR, Greer FR. Lipid screening and cardiovascular health in childhood. Pediatrics 2008;122(1):198-208. Review article
Ref ID: 3214

(1221) Danis A, Kyriazis Y, Klissouras V. The effect of training in male prepubertal and pubertal monozygotic twins. European Journal of Applied Physiology 2003 May;89(3-4):309-18. Not a randomized controlled trial (RCT)
Ref ID: 1749

(1222) Dantas PROF, Lira FAS, Borba VVL, Costa MJC, Trombetta IC, Santos MSB, Santos AC. Vitamin C restores blood pressure and vasodilator response during mental stress in obese children. Arquivos brasileiros de cardiologia 2011;96(6):490-7. Diet Intervention or Supplement Study
Ref ID: 3215

(1223) Dapi LN, Hornell A, Janlert U, Stenlund H, Larsson C. Energy and nutrient intakes in relation to sex and socio-economic status among school adolescents in urban Cameroon, Africa. Public Health Nutrition 2011 May;14(5):904-13. Cross-sectional study
Ref ID: 2477

(1224) Daray LA, Henagan TM, Zanovec M, Earnest CP, Johnson LG, Winchester J, Tuuri G, Stewart LK. Endurance and resistance training lowers C-reactive protein in young, healthy females. Applied Physiology, Nutrition, and Metabolism = Physiologie Appliquee, Nutrition et Metabolisme 2011 October;36(5):660-70. Study limited to adults
Ref ID: 2478

(1225) Datar A, Sturm R. Physical education in elementary school and body mass index: Evidence from the early childhood longitudinal study. American Journal of Public Health 2004;94:1501-6. Not All Participants were Overweight and/or Obese
Ref ID: 4736

(1226) Davidson ZE, Truby H. A review of nutrition in Duchenne muscular dystrophyJournal of Human Nutrition and Dietetics 2009;22(5):383-93. Review article
Ref ID: 3216

(1227) Davis AM, James RL, Boles RE, Goetz JR, Belmont J, Malone B. The use of TeleMedicine in the treatment of paediatric obesity: feasibility and acceptability. Maternal and Child Nutrition 2011 January;7(1):71-9. No exercise only group
Ref ID: 9

(1228) Davis AM, Sampilo M, Gallagher KS, Landrum Y, Malone B. Treating rural pediatric obesity through telemedicine: outcomes from a small randomized controlled trial. Journal of Pediatric Psychology 2013 October;38(9):932-43. Inappropriate Intervention
Ref ID: 5821

(1229) Davis B, Carpenter C. Proximity of Fast-Food Restaurants to Schools and Adolescent Obesity. American Journal of Public Health 2009 March;99(3):505-10. Diet Intervention or Supplement Study
Ref ID: 3810

(1230) Davis C, Kennedy SH, Ravelski E, Dionne M. The role of physical activity in the development and maintenance of eating disorders. Psychological Medicine 1994 November;24(4):957-67. Not a randomized controlled trial (RCT)
Ref ID: 2221

(1231) Davis CE, Hunsberger S, Murray DM, Fabsitz RR, Himes JH, Stephenson LK, Caballero B, Skipper B. Design and statistical analysis for the Pathways study. American Journal of Clinical Nutrition 1999 April;69(4:Suppl):Suppl-763S. Description of study from review or magazine or etc. (not the actual study)
Ref ID: 2037

(1232) Davis CL, Tkacz J, Gregoski M, Boyle CA, Lovrekovic G. Aerobic exercise and snoring in overweight children: a randomized controlled trial. Obesity (Silver Spring) 2006 November;14(11):1985-91. Same subjects as another study already included
Ref ID: 259

(1233) Davis CL, Tomporowski PD, Boyle CA, Waller JL, Miller PH, Naglieri JA, Gregoski M. Effects of aerobic exercise on overweight children's cognitive functioning: a randomized controlled trial. Research Quarterly for Exercise and Sport 2007 December;78(5):510-9. Same subjects as another study already

included
Ref ID: 196

(1234) Davis CL, Cooper S. Fitness, fatness, cognition, behavior, and academic achievement among overweight children: do cross-sectional associations correspond to exercise trial outcomes? Preventive Medicine 2011 June;52:Suppl-9. Cross-sectional study
Ref ID: 1032

(1235) Davis CL, Tomporowski PD, McDowell JE, Austin BP, Miller PH, Yanasak NE, Allison JD, Naglieri JA. Exercise improves executive function and achievement and alters brain activation in overweight children: a randomized, controlled trial. Health Psychology 2011 January;30(1):91-8. Same subjects as another study already included
Ref ID: 442

(1236) Davis CL, Pollock NK, Waller JL, Allison JD, Dennis BA, Bassali R, Melendez A, Boyle CA, Gower BA. Exercise dose and diabetes risk in overweight and obese children: a randomized controlled trial. Journal of the American Medical Association 2012 September 19;308(11):1103-12. Met criteria but could not retrieve data
Ref ID: 5720

(1237) Davis JN, Kelly LA, Lane CJ, Ventura EE, Byrd-Williams CE, Alexandar KA, Azen SP, Chou CP, Spruijt-Metz D, Weigensberg MJ, Berhane K, Goran MI. Randomized control trial to improve adiposity and insulin resistance in overweight Latino adolescents. Obesity 2009 August;17(8):1542-8. No exercise only group
Ref ID: 704

(1238) Davis JN, Tung A, Chak SS, Ventura EE, Byrd-Williams CE, Alexander KE, Lane CJ, Weigensberg MJ, Spruijt-Metz D, Goran MI. Aerobic and strength training reduces adiposity in overweight Latina adolescents. Medicine and Science in Sports and Exercise 2009 July;41(7):1494-503. No exercise only group
Ref ID: 113

(1239) Davis JN, Ventura EE, Shaibi GQ, Byrd-Williams CE, Alexander KE, Vanni AK, Meija MR, Weigensberg MJ, Spruijt-Metz D, Goran MI. Interventions for improving metabolic risk in overweight Latino youth. [Review]. International Journal of Pediatric Obesity 2010 October;5(5):451-5. Review article
Ref ID: 2481

(1240) Davis JN, Gyllenhammer LE, Vanni AA, Meija M, Tung A, Schroeder ET, Spruijt-Metz D, Goran MI. Startup circuit training program reduces metabolic risk in Latino adolescents. Medicine and Science in Sports and Exercise 2011

November;43(11):2195-203. Primary outcome(s) not assessed
Ref ID: 2482

(1241) Davis JN, Ventura EE, Tung A, Munevar MA, Hasson RE, Byrd-Williams C, Vanni AK, Spruijt-Metz D, Weigensberg M, Goran MI. Effects of a randomized maintenance intervention on adiposity and metabolic risk factors in overweight minority adolescents. Pediatric Obesity 2012 February;7(1):16-27. Follow-up Study
Ref ID: 2483

(1242) Davis KL, Kang M, Boswell BB, DuBose KD, Altman SR, Binkley HM. Validity and reliability of the medicine ball throw for kindergarten children. Journal of Strength and Conditioning Research 2008 November;22(6):1958-63. Not an exercise intervention study
Ref ID: 866

(1243) Davis PJ, McGowan FX, Landsman I, Maloney K, Hoffmann P. Effect of antiemetic therapy on recovery and hospital discharge time. A double-blind assessment of ondansetron, droperidol, and placebo in pediatric patients undergoing ambulatory surgery. Anesthesiology 1995;83:956-60. Drug intervention study
Ref ID: 4737

(1244) Davis PJ, Greenberg JA, Gendelman M, Fertal K. Recovery characteristics of sevoflurane and halothane in preschool-aged children undergoing bilateral myringotomy and pressure equalization tube insertion. Anesthesia and Analgesia 1999;88:34-8. Drug intervention study
Ref ID: 892

(1245) Davis SM, Clay T, Smyth M, Gittelsohn J, Arviso V, Flint-Wagner H, Rock BH, Brice RA, Metcalfe L, Stewart D, Vu M, Stone EJ. Pathways curriculum and family interventions to promote healthful eating and physical activity in American Indian schoolchildren. Preventive Medicine 2003 December;37(6 Pt 2):S24-S34. Primary outcome(s) not assessed
Ref ID: 348

(1246) Davison GW, Ashton T, George L, Young IS, McEneny J, Davies B, Jackson SK, Peters JR, Bailey DM. Molecular detection of exercise-induced free radicals following ascorbate prophylaxis in type 1 diabetes mellitus: a randomised controlled trial. Diabetologia 2008 November;51(11):2049-59. Diet Intervention or Supplement Study
Ref ID: 882

(1247) Davison K, Coates AM, Buckley JD, Howe PR. Effect of cocoa flavanols and exercise on cardiometabolic risk factors in overweight and obese subjects. International Journal of Obesity (London) 2008 August;32(8):1289-96. Study

limited to adults
Ref ID: 181

(1248) Davy BM, Harrell K, Stewart J, King DS. Body weight status, dietary habits, and physical activity levels of middle school-aged children in rural Mississippi. Southern Medical Journal 2004 June;97(6):571-7. Cross-sectional study
Ref ID: 1608

(1249) Dawson B, Vladich T, Blanksby BA. Effects of 4 weeks of creatine supplementation in junior swimmers on freestyle sprint and swim bench performance. Journal of Strength and Conditioning Research 2002 November;16(4):485-90. Diet Intervention or Supplement Study
Ref ID: 1794

(1250) Dâmaso AR, Teixeira LR, Nascimento CMO. Obesidade: subsídios para o desenvolvimento de atividades motoras. Revista Paulo de Educação Física 1994 June;8(1):98-111. Review article
Ref ID: 4084

(1251) Dâmaso AR, Teixeira LR, Curi CMOdN. Atividades motoras na obesidade. In: Fisberg M, editor. Obesidade na infância e adolescência.Säo Paulo: Fundo Editorial Byk; 1995. p. 91-9. Review article
Ref ID: 4085

(1252) Dämon S, Dietwch S, Widhalm K. PRESTO--Prevention Study of Obesity: a project to prevent obesity during childhood and adolescence. Acta Paediatrica Supplement 2005;94:47-8. Lifestyle Intervention
Ref ID: 4738

(1253) De-Windt AC, Asehnoune K, Roquilly A, Guillaud C, Le RC, Pinaud M, Lejus C. An opioid-free anaesthetic using nerve blocks enhances rapid recovery after minor hand surgery in children. European Journal of Anaesthesiology 2010;27:521-5. Drug intervention study
Ref ID: 4739

(1254) de Alwis NMW, Day CP. Current and future therapeutic strategies in NAFLD. Current Pharmaceutical Design 2010;16(17):1958-62. Review article
Ref ID: 3217

(1255) de Backer TLM, Smedema JP, Carlier SG. Current Management of Primary Pulmonary Hypertension. BioDrugs 2001 December;15(12):801-17. Review article
Ref ID: 3560

(1256) De Bruyne RML, Fitzpatrick E, Dhawan A. Fatty liver disease in children: Eat now pay later. Hepatology International 2010;4(1):375-85. Review article
Ref ID: 3218

(1257) de Carvalho MV, Marins JC, Silami-Garcia E. The influence of water versus carbohydrate-electrolyte hydration on blood components during a 16-km military march. Military Medicine 2007 January;172(1):79-82. Diet Intervention or Supplement Study
Ref ID: 1281

(1258) De Gáspari JC, Schwartz GM. Vivências em arte circense: Motivos de aderência e expectativas. Motriz Revista de Educação Física (Improv) 2007;13(3):158-64. Not a randomized controlled trial (RCT)
Ref ID: 4086

(1259) de Groot JF, Takken T, van BM, Gooskens R, Schoenmakers M, Versteeg C, Vanhees L, Helders P. Randomized controlled study of home-based treadmill training for ambulatory children with spina bifida. Neurorehabilitation and Neural Repair 2011 September;25(7):597-606. Not All Participants were Overweight and/or Obese
Ref ID: 2484

(1260) de Heer HD, Koehly L, Pederson R, Morera O. Effectiveness and spillover of an after-school health promotion program for Hispanic elementary school children. American Journal of Public Health 2011 October;101(10):1907-13. Not All Participants were Overweight and/or Obese
Ref ID: 2485

(1261) De Hert M, Dobbelaere M, Sheridan EM, Cohen D, Correll CU. Metabolic and endocrine adverse effects of second-generation antipsychotics in children and adolescents: A systematic review of randomized, placebo controlled trials and guidelines for clinical practice. European Psychiatry 2011;26(3):144-58. Review article
Ref ID: 3219

(1262) De Jongh S, Ose L, Szamosi T, Gagne C, Lambert M, Scott R, Perron P, Dobbelaere D, Saborio M, Tuohy MB, Stepanavage M, Sapre A, Gumbiner B, Mercuri M, Van Trotsenburg ASP, Bakker HD, Kastelein JJP. Efficacy and safety of statin therapy in children with familial hypercholesterolemia: A randomized, double-blind, placebo-controlled trial with simvastatin. Circulation 2002;106(17):2231-7. Drug intervention study
Ref ID: 3220

(1263) de la Torre A, Sadeghi B, Green RD, Kaiser LL, Flores YG, Jackson CF, Shaikh U, Whent L, Schaefer SE. Ninos Sanos, Familia Sana: Mexican immigrant study protocol for a multifaceted CBPR intervention to combat childhood obesity in two rural California towns. BMC Public Health 2013;13:1033. Inappropriate Study Design
Ref ID: 5822

(1264) de Lima SP, de Mello MT, Elias N, Fonseca FA, de PA, Carnier J, Oyama LM, Tock L, Tufik S, Damaso AR. Improvement in HOMA-IR is an independent predictor of reduced carotid intima-media thickness in obese adolescents participating in an interdisciplinary weight-loss program. Hypertension Research - Clinical and Experimental 2011 February;34(2):232-8. Lifestyle Intervention
Ref ID: 2486

(1265) De Lorenzo A, Bertini I, Iacopino L, Pagliato E, Testolin C, Testolin G. Body composition measurement in highly trained male athletes - A comparison of three methods. Journal of Sports Medicine and Physical Fitness 2000;40(2):178-83. Not All Participants were Overweight and/or Obese
Ref ID: 5227

(1266) de Meij JS, Chinapaw MJ, van Stralen MM, van der Wal MF, van DL, van MW. Effectiveness of JUMP-in, a Dutch primary school-based community intervention aimed at the promotion of physical activity. British Journal of Sports Medicine 2011 October;45(13):1052-7. Not a randomized controlled trial (RCT)
Ref ID: 2487

(1267) de Mello ED, Luft VC, Meyer F. [Individual outpatient care versus group education programs. Which leads to greater change in dietary and physical activity habits for obese children?]. Jornal de Pediatria (Rio J) 2004 November;80(6):468-74. No exercise only group, No comparative control group
Ref ID: 317

(1268) de Mello MT, de PA, Carnier J, Sanches PL, Correa FA, Tock L, Ernandes RM, Tufik S, Damaso AR. Long-term effects of aerobic plus resistance training on the metabolic syndrome and adiponectinemia in obese adolescents. Journal of Clinical Hypertension 2011 May;13(5):343-50. No non-intervention control group
Ref ID: 2488

(1269) De Miguel-Etayo P, Bueno G, Garagorri JM, Moreno LA. Interventions for treating obesity in children. World Review of Nutrition and Dietetics 2013;108:98-106. Inappropriate Study Design
Ref ID: 5823

(1270) De Miguel-Etayo P, Moreno LA, Iglesia I, Bel-Serrat S, Mouratidou T, Garagorri JM. Body composition changes during interventions to treat overweight and obesity in children and adolescents; a descriptive review. Nutricion Hospitalaria 2013 January;28(1):52-62. Inappropriate Study Design
Ref ID: 5824

(1271) de Onis M, Garza C, Onyango AW, Rolland-Cachera MF. WHO growth standards for infants and young children. Archives de Pediatrie 2009;16(1):47-

53. Not an exercise intervention study
Ref ID: 5228

(1272) De Palo EF, Gatti R, Lancerin F, Cappellin E, Solda G, De Palo CB, Spinella P. Urinary insulin-like growth factor I in athletes, before and after physical exercise, and in sedentary subjects. Clinica Chimica Acta 2002;322(1-2):51-7. Not an exercise intervention study
Ref ID: 5229

(1273) de Ramirez SS, Enquobahrie DA, Nyadzi G, Mjungu D, Magombo F, Ramirez M, Sachs SE, Willett W. Prevalence and correlates of hypertension: a cross-sectional study among rural populations in sub-Saharan Africa. Journal of Human Hypertension 2010 December;24(12):786-95. Cross-sectional study
Ref ID: 2489

(1274) De Ravel TJL, Swillen A, Willekens D, Descheemaeker MJ, Govers V, Borghgraef M, Vermeesch JR, Fryns JP. Molecular karyotyping is important in determining the cause of behavioural phenotypes. Journal of Intellectual Disability Research 2008 October;52(10):813. Case-Control / Case Study
Ref ID: 3811

(1275) de Silva-Sanigorski AM, Bell AC, Kremer P, Nichols M, Crellin M, Smith M, Sharp S, de GF, Carpenter L, Boak R, Robertson N, Swinburn BA. Reducing obesity in early childhood: results from Romp & Chomp, an Australian community-wide intervention program. American Journal of Clinical Nutrition 2010 April;91(4):831-40. Not a randomized controlled trial (RCT)
Ref ID: 568

(1276) De Souza FMB, Pereira RP, Minuque NP, Do Carmo CM, De Mello MHM, Villaca P, Tanaka C. Postural adjustment after an unexpected perturbation in children with haemophilia. Haemophilia 2012;18(3):e311-e315. Not a randomized controlled trial (RCT)
Ref ID: 5230

(1277) De Ste Croix MB, Armstrong N, Chia MY, Welsman JR, Parsons G, Sharpe P. Changes in short-term power output in 10- to 12-year-olds. Journal of Sports Sciences 2001 February;19(2):141-8. Not a randomized controlled trial (RCT)
Ref ID: 1933

(1278) De Vitta A, Madrigal C, Sales VS. Peso corporal e peso do material escolar transportado por crianças em idade escolar. Fisioterapia em Movimento 2003 June;16(2):55-60. Cross-sectional study
Ref ID: 709

(1279) De Vitta A, Martinez MG, Piza NT, Simeão SFdAP, Ferreira NP. Prevalência e fatores associados à dor lombar em escolares. Prevalence of lower back pain and associated factors in students. Cadernos de Saúde Pública 2011

August;27(8):1520-8. Cross-sectional study
Ref ID: 4087

(1280) de Vries C, Garneau CJ, Nadadur G, Parkinson MB. Considering Secular and Demographic Trends in Designing Long Lifetime Products for Target User Populations. Journal of Mechanical Design 2011;133(8). Not an exercise intervention study
Ref ID: 5231

(1281) de Zoysa NS, Jayaweera KK, Vaithianathan T. Manual plasmapheresis in the treatment of Guillain-Barre syndrome. Journal of Clinical Apheresis 1994;9(2):147-50. Drug intervention study
Ref ID: 2228

(1282) De BF, Fischer JE, Hoffmann K, Renz-Polster H. A participatory parent-focused intervention promoting physical activity in preschools: design of a cluster-randomized trial. BMC Public Health 2010;10:49. Description of study from review or magazine or etc. (not the actual study)
Ref ID: 573

(1283) de BS, Mathern GW, Bookheimer S, Dobkin B. Locomotor training remodels fMRI sensorimotor cortical activations in children after cerebral hemispherectomy. Neurorehabilitation and Neural Repair 2007 November;21(6):497-508. Study less than 4 weeks
Ref ID: 1152

(1284) De CK, Ottevaere C, Sjostrom M, Moreno LA, Warnberg J, Valtuena J, Manios Y, Dietrich S, Mauro B, Artero EG, Molnar D, Hagstromer M, Ruiz JR, Sarri K, Kafatos A, Gottrand F, de HS, Maes L, De B, I, HELENA Study Group. Self-reported physical activity in European adolescents: results from the HELENA (Healthy Lifestyle in Europe by Nutrition in Adolescence) study. Public Health Nutrition 2011 February;14(2):246-54. Cross-sectional study
Ref ID: 2490

(1285) De C, V, De B, I, Vereecken C, Verbestel V, Haerens L, Huybrechts I, Van LW, Maes L. Effects of a 2-year healthy eating and physical activity intervention for 3-6-year-olds in communities of high and low socio-economic status: the POP (Prevention of Overweight among Pre-school and school children) project. Public Health Nutrition 2012 September;15(9):1737-45. Inappropriate Intervention
Ref ID: 5825

(1286) De CC, Malinow MR, van Kranenburg GP, Geurten PG, Longford NT, Keizer HA. Influence of exercise and menstrual cycle phase on plasma homocyst(e)ine levels in young women--a prospective study. Scandinavian Journal of Medicine and Science in Sports 1999 October;9(5):272-8. Acute

study
Ref ID: 1998

(1287) de GL, de-Groot CJ, Hopkins B. An instrument to measure independent walking: are there differences between preterm and fullterm infants? Journal of Child Neurology 1997;12:37-41. Subjects less than 2 years old
Ref ID: 934

(1288) de GS, Dallmeijer AJ, Post MW, Angenot EL, van den Berg-Emons RJ, van der Woude LH. Prospective analysis of lipid profiles in persons with a spinal cord injury during and 1 year after inpatient rehabilitation. Archives of Physical Medicine and Rehabilitation 2008 March;89(3):531-7. Not an exercise intervention study
Ref ID: 994

(1289) de GS, van der Woude LH, Niezen A, Smit CA, Post MW. Evaluation of the physical activity scale for individuals with physical disabilities in people with spinal cord injury. Spinal Cord 2010 July;48(7):542-7. Cross-sectional study
Ref ID: 501

(1290) de JW, van-Aalderen WM, Kraan J, Koëter GH, van der Schans CP. Inspiratory muscle training in patients with cystic fibrosis. Respiratory Medicine 2001;95:31-6. Not a randomized controlled trial (RCT)
Ref ID: 4740

(1291) De LM, Segato G, Busetto L, Favretti F, Aigner F, Weiss H, de GC, Gaggiotti G, Himpens J, Limao J, Scheyer M, Toppino M, Zurmeyer EL, Bottani G, Penthaler H. Progress in implantable gastric stimulation: summary of results of the European multi-center study. Obesity Surgery 2004 September;14:Suppl-9. Study limited to adults
Ref ID: 1582

(1292) de NJ, Timman R, Bauer S, van den Akker E, de KC, Kordy H, Passchier J. Short message service reduces dropout in childhood obesity treatment: a randomized controlled trial. Health Psychology 2012 November;31(6):797-805. Inappropriate Intervention
Ref ID: 5826

(1293) de PA, de Mello MT, Sanches PL, da Silva PL, Campos RM, Carnier J, Corgosinho F, Foschini D, Masquio DL, Tock L, Oyama LM, do Nascimento CM, Tufik S, Damaso AR. Long-term effects of aerobic plus resistance training on the adipokines and neuropeptides in nonalcoholic fatty liver disease obese adolescents. European Journal of Gastroenterology and Hepatology 2012 November;24(11):1313-24. Inappropriate Comparison Group
Ref ID: 5827

(1294) De SJ, Van den Broeck M, Jonckheer MH. Study of lumbar spine bone mineral density in obese children. Acta Paediatrica 1995 March;84(3):313-5. Not an

exercise intervention study
Ref ID: 2207

(1295) Debanne T, Laffaye G. Predicting the throwing velocity of the ball in handball with anthropometric variables and isotonic tests. Journal of Sports Sciences 2011 April;29(7):705-13. Study limited to adults
Ref ID: 2491

(1296) DeBar LL, Ritenbaugh C, Vuckovic N, Stevens VJ, Aickin M, Elliot D, Moe E, Orwoll E, Ernst D, Irving LM. YOUTH: decisions and challenges in designing an osteoporosis prevention intervention for teen girls. Preventive Medicine 2004 November;39(5):1047-55. Description of study from review or magazine or etc. (not the actual study)
Ref ID: 1583

(1297) DeBar LL, Ritenbaugh C, Aickin M, Orwoll E, Elliot D, Dickerson J, Vuckovic N, Stevens VJ, Moe E, Irving LM. Youth: a health plan-based lifestyle intervention increases bone mineral density in adolescent girls.[Erratum appears in Archives of Pediatric Adolescent Medicine. 2007 Feb;161(2):130]. Archives of Pediatrics and Adolescent Medicine 2006 December;160(12):1269-76. Not All Participants were Overweight and/or Obese
Ref ID: 1316

(1298) DeBar LL, Dickerson J, Clarke G, Stevens VJ, Ritenbaugh C, Aickin M. Using a website to build community and enhance outcomes in a group, multi-component intervention promoting healthy diet and exercise in adolescents. Journal of Pediatric Psychology 2009 June;34(5):539-50. Lifestyle Intervention
Ref ID: 748

(1299) DeBar LL, Schneider M, Ford EG, Hernandez AE, Showell B, Drews KL, Moe EL, Gillis B, Jessup AN, Stadler DD, White M, HEALTHY study group. Social marketing-based communications to integrate and support the HEALTHY study intervention. International Journal of Obesity 2009 August;33:Suppl-9. Cohort Study
Ref ID: 710

(1300) DeBar LL, Schneider M, Drews KL, Ford EG, Stadler DD, Moe EL, White M, Hernandez AE, Solomon S, Jessup A, Venditti EM, HEALTHY study group. Student public commitment in a school-based diabetes prevention project: impact on physical health and health behavior. BMC Public Health 2011;11:711. Review article
Ref ID: 2492

(1301) DeBar LL, Stevens VJ, Perrin N, Wu P, Pearson J, Yarborough BJ, Dickerson J, Lynch F. A primary care-based, multicomponent lifestyle intervention for overweight adolescent females. Pediatrics 2012 March;129(3):e611-e620.

Lifestyle Intervention
Ref ID: 1042

(1302) Deconinck FJA, De Clercq D, Van Coster R, Oostra A, Dewitte G, Savelsbergh GJR, Cambier D, Lenoir M. Sensory contributions to balance in boys with developmental coordination disorder. Adapted Physical Activity Quarterly 2008;25(1):17-35. Not a randomized controlled trial (RCT)
Ref ID: 5232

(1303) Dedoussis GV, Yannakoulia M, Timpson NJ, Manios Y, Kanoni S, Scott RA, Papoutsakis C, Deloukas P, Pitsiladis YP, Davey-Smith G, Hirschhorn JN, Lyon HN. Does a short breastfeeding period protect from FTO-induced adiposity in children? International Journal of Pediatric Obesity 2011 June;6(2-2):e326-e335. Cohort Study
Ref ID: 2494

(1304) Deforche B, De B, I, Tanghe A, Hills AP, De BP. Changes in physical activity and psychosocial determinants of physical activity in children and adolescents treated for obesity. Patient Education and Counseling 2004 December;55(3):407-15. Not a randomized controlled trial (RCT)
Ref ID: 1566

(1305) Deforche B, De B, I, Tanghe A, Debode P, Hills AP, Bouckaert J. Post-treatment phone contact: a weight maintenance strategy in obese youngsters. International Journal of Obesity (London) 2005 May;29(5):543-6. No exercise only group
Ref ID: 314

(1306) Degoricija V, Zjacic-Rotkvic V, Marout J, Sefer S, Troskot B. Clinical and neurohumoral response to posture, physical exercise, and ascites treatment in Child-Pugh C liver cirrhosis: randomized prospective trial. Croatian Medical Journal 2003 April;44(2):178-86. Not an exercise intervention study
Ref ID: 1758

(1307) DeJongh ED, Binkley TL, Specker BL. Fat mass gain is lower in calcium-supplemented than in unsupplemented preschool children with low dietary calcium intakes. American Journal of Clinical Nutrition 2006 November;84(5):1123-7. Secondary analysis, No exercise only group
Ref ID: 261

(1308) del RÃ-o-Navarro BE, Hidalgo-Castro EMa, Luis Sienra-Monge JJ. Asma. (Spanish). Boletin Medico del Hospital Infantil de Mexico 2009 January;66(1):3-33. Review article
Ref ID: 3561

(1309) del Rio-Navarro B, Cisneros-Rivero M, Berber-Eslava A, Espinola-Reyna G, Sienra-Monge J. Exercise induced bronchospasm in asthmatic and non-asthmatic obese children. Allergologia et Immunopathologia 2000

January;28(1):5-11. Acute study
Ref ID: 1984

(1310) del Valle MF, Perez M, Santana-Sosa E, Fiuza-Luces C, Bustamante-Ara N, Gallardo C, Villasenor A, Graell M, Morande G, Romo GR, Lopez-Mojares LM, Ruiz JR, Lucia A. Does resistance training improve the functional capacity and well being of very young anorexic patients? A randomized controlled trial. Journal of Adolescent Health 2010 April;46(4):352-8. Not All Participants were Overweight and/or Obese
Ref ID: 565

(1311) Delahanty LM, Nathan DM, Lachin JM, Hu FB, Cleary PA, Ziegler GK, Wylie-Rosett J, Wexler DJ, Diabetes Control and Complications Trial/Epidemiology of Diabetes. Association of diet with glycated hemoglobin during intensive treatment of type 1 diabetes in the Diabetes Control and Complications Trial. American Journal of Clinical Nutrition 2009 February;89(2):518-24. Cross-sectional study
Ref ID: 812

(1312) DeLany JP, Bray GA, Harsha DW, Volaufova J. Energy expenditure in preadolescent African American and white boys and girls: the Baton Rouge Children's Study. American Journal of Clinical Nutrition 2002;75(4):705-13. Cohort Study
Ref ID: 5233

(1313) Delgado HL, Hurtado E. Crecimiento físico y menarquia en adolescentes de Guatemala. Archivos Latinoamericanos de Nutrición 1990 December;40(4):503-17. Cross-sectional study
Ref ID: 817

(1314) Delisle H, Agueh V, Fayomi B. Partnership research on nutrition transition and chronic diseases in West Africa - trends, outcomes and impacts. BMC International Health and Human Rights 2011;11. Cross-sectional study
Ref ID: 5234

(1315) Dell'Agnello G, Maschietto D, Bravaccio C, Calamoneri F, Masi G, Curatolo P, Besana D, Mancini F, Rossi A, Poole L, Escobar R, Zuddas A, -LYCY-Study-Group. Atomoxetine hydrochloride in the treatment of children and adolescents with attention-deficit/hyperactivity disorder and comorbid oppositional defiant disorder: A placebo-controlled Italian study. European Neuropsychopharmacology 2009;19:822-34. Drug intervention study
Ref ID: 4741

(1316) Della CC, Alisi A, Iorio R, Alterio A, Nobili V. Expert opinion on current therapies for nonalcoholic fatty liver disease. [Review]. Expert Opinion on Pharmacotherapy 2011 August;12(12):1901-11. Review article
Ref ID: 2495

(1317) Dellagrana RA, Silva MPd, Smolarek AdC, Bozza R, Stabelini Neto A, Campos Wd. Composição corporal, maturação sexual e desempenho motor de jovens praticantes de handebol. Body composition, sexual maturation and motor performance the young practitioners handball. Motriz Revista de Educação Física (Improv) 2010 December;16(4):880-8. Cross-sectional study
Ref ID: 4088

(1318) Delva J, OMalley PM, Johnston LD. Health-related behaviors and overweight: a study of Latino adolescents in the United States of America. Revista Panamericana de Salud Pública 2007 January;21(1):11-20. Cross-sectional study
Ref ID: 639

(1319) DeMattia L, Lemont L, Meurer L. Do interventions to limit sedentary behaviours change behaviour and reduce childhood obesity? A critical review of the literature. [Review] [32 refs]. Obesity Reviews 2007 January;8(1):69-81. Review article
Ref ID: 1286

(1320) Demeer K, Bergman R, Kusner JS, Voorhoeve HWA. Differences in Physical Growth of Aymara and Quechua Children Living at High-Altitude in Peru. American Journal of Physical Anthropology 1993;90(1):59-75. Cross-sectional study
Ref ID: 5235

(1321) Demke DM, Peters GR, Linet OI, Metzler CM, Klott KA. Effects of a fish oil concentrate in patients with hypercholesterolemia. Atherosclerosis 1988 March;70(1-2):73-80. Diet Intervention or Supplement Study
Ref ID: 2496

(1322) Demling RH, DeSanti L. The rate of restoration of body weight after burn injury, using the anabolic agent oxandrolone, is not age dependent. Burns 2001 February;27(1):46-51. No non-intervention control group
Ref ID: 1931

(1323) Demo R, Senestrari D, Ferreyra JE. [Young football players aerobic perfomance in sub-maximum exercise with exhaustion at a moderate altitude without acclimation: experience in El Condor]. [Spanish]. Revista de la Facultad de Ciencias Medicas de Cordoba 2007;64(1):8-17. Not a randomized controlled trial (RCT)
Ref ID: 1113

(1324) Dempsey RL, Mazzone MF, Meurer LN. Does oral creatine supplementation improve strength? A meta-analysis. Journal of Family Practice 2002 November;51(11):945-51. Review article
Ref ID: 1787

(1325) Demura S, Yamaji S, Goshi F, Nagasawa Y. The influence of transient change of total body water on relative body fats based on three bioelectrical impedance analyses methods. Comparison between before and after exercise with sweat loss, and after drinking. Journal of Sports Medicine and Physical Fitness 2002 March;42(1):38-44. Diet Intervention or Supplement Study
Ref ID: 1851

(1326) den Hoed M, Westerterp KR. Body composition is associated with physical activity in daily life as measured using a triaxial accelerometer in both men and women. International Journal of Obesity 2008;32(8):1264-70. Cross-sectional study
Ref ID: 5236

(1327) den HM, Brage S, Zhao JH, Westgate K, Nessa A, Ekelund U, Spector TD, Wareham NJ, Loos RJ. Heritability of objectively assessed daily physical activity and sedentary behavior. American Journal of Clinical Nutrition 2013 November;98(5):1317-25. Inappropriate Outcomes
Ref ID: 5828

(1328) Denadai RC, Sigulem DM, Vítolo MR, Fisberg M, Dâmaso AR. Efeito da atividade motora sobre a composiçäo corporal, taxa metabólica basal e diária de adolescentes obesos. Revista Paulista de Pediatria 1996 December;14(4):163-8. Not a randomized controlled trial (RCT)
Ref ID: 4089

(1329) Denadai RC, Vítolo MR, Macedo AS, Teixeira L, Cezar C, Dâmaso AR. Efeitos do exercício moderado e da orientaçäo nutricional sobre a composiçäo corporal de adolescentes obesos avaliados por densitometria óssea (DEXA). Revista Paulo de Educação Física 1998 December;12(2):210-8. Lifestyle Intervention
Ref ID: 779

(1330) Dennis BA, Ergul A, Gower BA, Allison JD, Davis CL. Oxidative stress and cardiovascular risk in overweight children in an exercise intervention program. Childhood Obesity 2013 February;9(1):15-21. Inappropriate Outcomes
Ref ID: 5829

(1331) Derave W, Eijnde BO, Verbessem P, Ramaekers M, Van LM, Richter EA, Hespel P. Combined creatine and protein supplementation in conjunction with resistance training promotes muscle GLUT-4 content and glucose tolerance in humans. Journal of Applied Physiology 2003 May;94(5):1910-6. Diet Intervention or Supplement Study
Ref ID: 1761

(1332) Derman O, Cinemre A, Kanbur N, Dogan M, Kilic M, Karaduman E. Effect of swimming on bone metabolism in adolescents. Turkish Journal of Pediatrics

2008 March;50(2):149-54. Not a randomized controlled trial (RCT)
Ref ID: 908

(1333) Deruelle F, Baron B. Vitamin C: Is Supplementation Necessary for Optimal Health? Journal of Alternative and Complementary Medicine 2008 December;14(10):1291-8. Review article
Ref ID: 3562

(1334) Detsch C, Luz AMH, Candotti CT, Oliveira DSd, Lazaron F, Guimarães LK, Schimanoski P. Prevalência de alterações posturais em escolares do ensino médio em uma cidade no Sul do Brasil. Revista Panamericana de Salud Pública 2007 April;21(4):231-8. Survey or questionnaire
Ref ID: 4090

(1335) Deus RKBCd, Bustamante A, Lopes VP, Seabra AT, Silva RMGd, Maia JAR. Modelação longitudinal dos níveis de coordenação motora de crianças dos seis aos 10 anos de idade da Região Autônoma dos Açores, Portugal. Longetudinal modeling of motor coordination levels of children aged six to 10 years of age from the Autonomous Region of Azores, Portugal. Revista Brasileira de Educação Física e Esporte 2010 June;24(2):259-73. Survey or questionnaire
Ref ID: 4091

(1336) Deutschbein T, Unger N, Jaeger A, Broecker-Preuss M, Mann K, Petersenn S. Influence of various confounding variables and storage conditions on metanephrine and normetanephrine levels in plasma. Clinical Endocrinology 2010 August;73(2):153-60. Not a randomized controlled trial (RCT)
Ref ID: 484

(1337) Devaney JM, Tosi LL, Fritz DT, Gordish-Dressman HA, Jiang S, Orkunoglu-Suer FE, Gordon AH, Harmon BT, Thompson PD, Clarkson PM, Angelopoulos TJ, Gordon PM, Moyna NM, Pescatello LS, Visich PS, Zoeller RF, Brandoli C, Hoffman EP, Rogers MB. Differences in fat and muscle mass associated with a functional human polymorphism in a post-transcriptional BMP2 gene regulatory element. Journal of Cellular Biochemistry 2009 August 15;107(6):1073-82. Not an exercise intervention study
Ref ID: 703

(1338) DeVault N, Kennedy T, Hermann J, Mwavita M, Rask P, Jaworsky A. It's all about kids: preventing overweight in elementary school children in Tulsa, OK. Journal of the American Dietetic Association 2009 April;109(4):680-7. No exercise only group
Ref ID: 131

(1339) Dewan B, Balasubramanian A. Troxipide in the Management of Gastritis: A Randomized Comparative Trial in General Practice. Gastroenterology

Research and Practice 2010 January;2010:1-7. Drug intervention study
Ref ID: 3563

(1340) Dewar DL, Morgan PJ, Plotnikoff RC, Okely AD, Collins CE, Batterham M, Callister R, Lubans DR. The nutrition and enjoyable activity for teen girls study: a cluster randomized controlled trial. American Journal of Preventive Medicine 2013 September;45(3):313-7. Inappropriate Intervention
Ref ID: 5830

(1341) Dewar DL, Morgan PJ, Plotnikoff RC, Okely AD, Batterham M, Lubans DR. Exploring changes in physical activity, sedentary behaviors and hypothesized mediators in the NEAT girls group randomized controlled trial. Journal of Science and Medicine in Sport 2014 January;17(1):39-46. Inappropriate Intervention
Ref ID: 5831

(1342) Dewey KG, Cohen RJ, Brown KH, Rivera LL. Effects of exclusive breastfeeding for four versus six months on maternal nutritional status and infant motor development: results of two randomized trials in Honduras. Journal of Nutrition 2001 February;131(2):262-7. Subjects less than 2 years old
Ref ID: 1929

(1343) DeWolfe JA, Jack E. Weight control in adolescent girls: a comparison of the effectiveness of three approaches to follow-up. Journal of School Health 1984 October;54(9):347-9. Follow-up Study
Ref ID: 2361

(1344) Di Marzio D, Mohn A, De Martino M, Chiarelli F. Macroangiopathy in adults and children with diabetes: Risk factors (Part 2). Hormone and Metabolic Research 2006;38(11):706-20. Review article
Ref ID: 3221

(1345) Diallo O, Dore E, Duche P, Van PE. Effects of plyometric training followed by a reduced training programme on physical performance in prepubescent soccer players. Journal of Sports Medicine and Physical Fitness 2001;41:342-8. Not a randomized controlled trial (RCT)
Ref ID: 4742

(1346) Dias RG, Alves MJNN, Pereira AC, Rondon MUPB, dos Santos MR, Krieger JE, Krieger MH, Negrao CE. Glu298Asp eNOS gene polymorphism causes attenuation in nonexercising muscle vasodilatation. Physiological Genomics 2009;37(2):99-107. Study limited to adults
Ref ID: 5237

(1347) Dias RMR, Carvalho FO, Souza CFd, Avelar A, Altimari LR, Cyrino ES. Características antropométricas e de desempenho motor de atletas de futsal em diferentes categorias. Revista Brasileira de Cineantropometria e

Desempenho Humano 2007 September;9(3). Cross-sectional study
Ref ID: 4092

(1348) Diaz-Gomez NM, Domenech E, Cortabarria C, Barroso F, Castells S, Jimenez A. The Effect of Zinc Supplementation on Linear Growth, Body Composition, and Growth Factors in Preterm Infants. Pediatrics 2003 May;111(5):1002. Diet Intervention or Supplement Study
Ref ID: 3564

(1349) Diaz A, Vogiatzi MG, Sanz MM, German J. Evaluation of short stature, carbohydrate metabolism and other endocrinopath. in Bloom's syndrome. Hormone Research 2006;66:111-7. Not an exercise intervention study
Ref ID: 4743

(1350) Diaz RG, Esparza-Romero J, Moya-Camarena SY, Robles-Sardin AE, Valencia ME. Lifestyle intervention in primary care settings improves obesity parameters among Mexican youth. Journal of the American Dietetic Association 2010 February;110(2):285-90. Lifestyle Intervention
Ref ID: 602

(1351) Dib SA. Resistência à insulina e síndrome metabólica no diabetes melito do tipo 1. Arquivos Brasileiros de Endocrinologia and Metabologia 2006 April;50(2):250-63. Review article
Ref ID: 659

(1352) Dicken-Kano R, Bell MM. Pedometers as a means to increase walking and achieve weight loss. Journal of the American Board of Family Medicine 2006 September;19(5):524-5. Not an exercise intervention study
Ref ID: 270

(1353) Dickinson S, Hancock DP, Petocz P, Ceriello A, Brand-Miller J. High-glycemic index carbohydrate increases nuclear factor-kappaB activation in mononuclear cells of young, lean healthy subjects. American Journal of Clinical Nutrition 2008 May;87(5):1188-93. Not an exercise intervention study
Ref ID: 184

(1354) Dickson-Parnell BE, Zeichner A. Effects of a short-term exercise program on caloric consumption. Health Psychology 1985;4(5):437-48. Study limited to adults
Ref ID: 2355

(1355) Dickson JM, Weavers HM, Mitchell N, Winter EM, Wilkinson ID, Van Beek EJ, Wild JM, Griffiths PD. The effects of dehydration on brain volume -- preliminary results. International Journal of Sports Medicine 2005 July;26(6):481-5. Not an exercise intervention study
Ref ID: 1491

(1356) Dieruf K, Burtner PA, Provost B, Phillips J, Bernitsky-Beddingfield A, Sullivan KJ. A Pilot Study of Quality of Life in Children with Cerebral Palsy After Intensive Body Weight-Supported Treadmill Training. Pediatric Physical Therapy 2009;21(1):45-52. Study less than 4 weeks
Ref ID: 5238

(1357) Dikel W, Olness K. Self-hypnosis, biofeedback, and voluntary peripheral temperature control in children. Pediatrics 1980 September;66(3):335-40. Not an exercise intervention study
Ref ID: 2378

(1358) Dimatos SC, Souza JAd, Dimatos OC, Araújo EJ, Dimatos DC, Pereima MJL. Tricobezoar na infância: Relato de três casos e revisão da literatura. ACM Arquivos Catarinense de Medicina 2009 July;38(2):112-5. Case-Control / Case Study
Ref ID: 4093

(1359) Dimkpa U, Oji JO. Association of heart rate recovery after exercise with indices of obesity in healthy, non-obese adults. European Journal of Applied Physiology 2010 March;108(4):695-9. Study limited to adults, Not an exercise intervention study
Ref ID: 84

(1360) Dimkpa U, Oji JO. Relationship of body mass index with haemodynamic variables and abnormalities in young adults. Journal of Human Hypertension 2010;24(4):230-6. Cross-sectional study
Ref ID: 5239

(1361) DiNapoli PP, Lewis JB. Understanding school-age obesity: through participatory action research. MCN, American Journal of Maternal Child Nursing 2008 March;33(2):104-10. Cross-sectional study
Ref ID: 985

(1362) Dinis-Oliveira RJ, Duarte JA, Sanchez-Navarro A, Remiao F, Bastos ML, Carvalho F. Paraquat Poisonings: Mechanisms of Lung Toxicity, Clinical Features, and Treatment. Critical Reviews in Toxicology 2008 January;38(1):13-71. Review article
Ref ID: 3565

(1363) Diniz IMS, Lopes AdS, Dummel CCB, Rieger T. Crescimento físico e adiposidade corporal de escolares. Revista Brasileira de Cineantropometria e Desempenho Humano 2006 June;8(2). Cross-sectional study
Ref ID: 4094

(1364) Diniz IMS, Lopes AdS, Borgatto AF. Crescimento físico e composição corporal de escolares de diferentes grupos étnicos do Estado do Rio Grande do Sul, Brasil. Revista Brasileira de Cineantropometria e Desempenho Humano 2008

March;10(1). Cross-sectional study
Ref ID: 4095

(1365) Diniz MB, Coldebella CR, Zuanon AC, Cordeiro RdC. Alterações orais em crianças prematuras e de baixo peso ao nascer: a importância da relação entre pediatras e odontopediatras. Oral abnormalities in preterm and low birth weight infants: the importance of the relationship between pediatricians and pediatric dentists. Revista Paulista de Pediatria 2011 September;29(3):440-53. Review article
Ref ID: 4096

(1366) Ditunno JF, Jr., Barbeau H, Dobkin BH, Elashoff R, Harkema S, Marino RJ, Hauck WW, Apple D, Basso DM, Behrman A, Deforge D, Fugate L, Saulino M, Scott M, Chung J, Spinal Cord Injury Locomotor Trial Group. Validity of the walking scale for spinal cord injury and other domains of function in a multicenter clinical trial. Neurorehabilitation and Neural Repair 2007 November;21(6):539-50. Not All Participants were Overweight and/or Obese
Ref ID: 1151

(1367) Dixon JB, Jones K, Dixon M. Medical versus surgical interventions for the metabolic complications of obesity in children. Seminars in Pediatric Surgery 2009 August;18(3):168-75. Review article
Ref ID: 726

(1368) Díaz franco MB, Duran reina MC, Ramírez Pérez MF. Perfil funcional después de un entrenamiento con pesas en jugadores de futbol entre 15 y 19 años de edad de las divisiones menores del club Santa Fe C. D. Revista Colombiana de Rehabilitación 2004 October;1(3):49-56. No control group (NC)
Ref ID: 4097

(1369) Díaz RG, Esparza RJ, Moya-Camarena SY, Robles-Sardín AE, Valencia ME. Lifestyle intervention in primary care settings improves obesity parameters among Mexican youth. Journal of the American Dietetic Association 2010;110:285-90. Lifestyle Intervention
Ref ID: 4744

(1370) Djuric Z, DiLaura NM, Jenkins I, Darga L, Jen CK, Mood D, Bradley E, Hryniuk WM. Combining weight-loss counseling with the weight watchers plan for obese breast cancer survivors. Obesity Research 2002 July;10(7):657-65. Study limited to adults
Ref ID: 376

(1371) Dobbins M, De CK, Robeson P, Husson H, Tirilis D. School-based physical activity programs for promoting physical activity and fitness in children and adolescents aged 6-18. [Review] [408 refs]. Cochrane Database of Systematic Reviews (1):CD007651, 2009 2009;(1):CD007651. Review article
Ref ID: 807

(1372) Dobbins M, Husson H, DeCorby K, LaRocca RL. School-based physical activity programs for promoting physical activity and fitness in children and adolescents aged 6 to 18. Cochrane Database of Systematic Reviews 2013;2:CD007651. Inappropriate Study Design
Ref ID: 5832

(1373) Doberenz J, Birkenfeld C, Kluge H, Eder K. Effects ofL-carnitine supplementation in pregnant sows on plasma concentrations of insulin-like growth factors, various hormones and metabolites and chorion characteristics. Journal of Animal Physiology and Animal Nutrition 2006 December;90(11/12):487-99. Animal study
Ref ID: 3566

(1374) Dobkin B, Apple D, Barbeau H, Basso M, Behrman A, Deforge D, Ditunno J, Dudley G, Elashoff R, Fugate L, Harkema S, Saulino M, Scott M, Spinal Cord Injury Locomotor Trial Group. Weight-supported treadmill vs over-ground training for walking after acute incomplete SCI. Neurology 2006 February 28;66(4):484-93. Not All Participants were Overweight and/or Obese
Ref ID: 1413

(1375) Dodd CJ, Welsman JR, Armstrong N. Energy intake and appetite following exercise in lean and overweight girls. Appetite 2008 November;51(3):482-8. Acute study
Ref ID: 183

(1376) Dodd JM, Grivell RM, Crowther CA, Robinson JS. Antenatal interventions for overweight or obese pregnant women: a systematic review of randomised trials. [Review]. International Journal of Obstetrics and Gynaecology 117(11):1316-26, 2010 Oct 2010;(11):1316-26. Review article
Ref ID: 2943

(1377) Dodd KJ, Foley S. Partial body-weight-supported treadmill training can improve walking in children with cerebral palsy: a clinical controlled trial. Developmental Medicine and Child Neurology 2007 February;49(2):101-5. Not a randomized controlled trial (RCT)
Ref ID: 1282

(1378) Dodd SL, Brooks E, Powers SK, Tulley R. The effects of caffeine on graded exercise performance in caffeine naive versus habituated subjects. European Journal of Applied Physiology and Occupational Physiology 1991;62(6):424-9. Diet Intervention or Supplement Study
Ref ID: 2299

(1379) Doherty M, Dimitriou L. Comparison of lung volume in Greek swimmers, land based athletes, and sedentary controls using allometric scaling. British Journal of Sports Medicine 1997 December;31(4):337-41. Cross-sectional study
Ref ID: 2115

(1380) Dolev E, Burstein R, Lubin F, Wishnizer R, Chetrit A, Shefi M, Deuster PA. Interpretation of zinc status indicators in a strenuously exercising population. Journal of the American Dietetic Association 1995 April;95(4):482-4. Review article
Ref ID: 2215

(1381) Dolinsky DH, Brouwer RJ, Evenson KR, Siega-Riz AM, Ostbye T. Correlates of sedentary time and physical activity among preschool-aged children. Preventing Chronic Disease 2011 November;8(6):A131. Cross-sectional study
Ref ID: 2497

(1382) Dollfus C, Blanche S, Trocme N, Funck-Brentano I, Bonnet F, Levan P. Correction of facial lipoatrophy using autologous fat transplants in HIV-infected adolescents. HIV Medicine 2009;10(5):263-8. Not an exercise intervention study
Ref ID: 5240

(1383) Domecq JP, Prutsky G, Mullan RJ, Hazem A, Sundaresh V, Elamin MB, Phung OJ, Wang A, Hoeger K, Pasquali R, Erwin P, Bodde A, Montori VM, Murad MH. Lifestyle modification programs in polycystic ovary syndrome: systematic review and meta-analysis. Journal of Clinical Endocrinology and Metabolism 2013 December;98(12):4655-63. Inappropriate Study Design
Ref ID: 5833

(1384) Domingues RB, Teixeira AL, Domingues SA. Physical practice is associated with less functional disability in medical students with migraine.
A prática de exercícios físicos está associada a menor comprometimento funcional da migrânea entre estudantes de medicina. Arquivos de Neuro-Psiquiatria 2011 February;69(1):39-43. Survey or questionnaire
Ref ID: 4098

(1385) Dominici N, Ivanenko YP, Lacquaniti F. Control of foot trajectory in walking toddlers: Adaptation to load changes. Journal of Neurophysiology 2007;97(4):2790-801. Not an exercise intervention study
Ref ID: 5241

(1386) Donaghue KC, Pena MM, Chan AK, Blades BL, King J, Storlien LH, Silink M. Beneficial effects of increasing monounsaturated fat intake in adolescents with type 1 diabetes. Diabetes Research and Clinical Practice 2000 June;48(3):193-9. Diet Intervention Study
Ref ID: 1979

(1387) Donahue RP, Prineas RJ, Gomez O, Hong CP. Familial Resemblance of Body-Fat Distribution - the Minneapolis-Childrens-Blood-Pressure-Study. International Journal of Obesity 1992;16(3):161-7. Cross-sectional study
Ref ID: 5242

(1388) Dong W, Colhoun HM, Poulter NR. Blood pressure in women using oral contraceptives: results from the Health Survey for England 1994. Journal of Hypertension 1997 October;15(10):1063-8. Cross-sectional study
Ref ID: 2121

(1389) Dong Y, Pollock N, Stallmann-Jorgensen IS, Gutin B, Lan L, Chen TC, Keeton D, Petty K, Holick MF, Zhu H. Low 25-hydroxyvitamin D levels in adolescents: race, season, adiposity, physical activity, and fitness. Pediatrics 2010 June;125(6):1104-11. Cross-sectional study
Ref ID: 519

(1390) Donma MM, Donma O. The influence of feeding patterns on head circumference among Turkish infants during the first 6 months of life. Brain and Development 1997;19(6):393-7. Subjects less than 2 years old
Ref ID: 5243

(1391) Donma MM, Donma O. Infant feeding and growth: A study on Turkish infants from birth to 6 months. Pediatrics International 1999;41(5):542-8. Subjects less than 2 years old
Ref ID: 5244

(1392) Donnelly JE, Kirk EP, Jacobsen DJ, Hill JO, Sullivan DK, Johnson SL. Effects of 16 mo of verified, supervised aerobic exercise on macronutrient intake in overweight men and women: the Midwest Exercise Trial. American Journal of Clinical Nutrition 2003 November;78(5):950-6. Study limited to adults
Ref ID: 352

(1393) Donnelly JE, Hill JO, Jacobsen DJ, Potteiger J, Sullivan DK, Johnson SL, Heelan K, Hise M, Fennessey PV, Sonko B, Sharp T, Jakicic JM, Blair SN, Tran ZV, Mayo M, Gibson C, Washburn RA. Effects of a 16-month randomized controlled exercise trial on body weight and composition in young, overweight men and women: the Midwest Exercise Trial. Archives of Internal Medicine 2003 June 9;163(11):1343-50. Study limited to adults
Ref ID: 362

(1394) Donnelly JE, Sullivan DK, Smith BK, Jacobsen DJ, Washburn RA, Johnson SL, Hill JO, Mayo MS, Spaeth KR, Gibson C. Alteration of dietary fat intake to prevent weight gain: Jayhawk Observed Eating Trial. Obesity (Silver Spring) 2008 January;16(1):107-12. Not an exercise intervention study
Ref ID: 202

(1395) Donnelly JE, Greene JL, Gibson CA, Smith BK, Washburn RA, Sullivan DK, DuBose K, Mayo MS, Schmelzle KH, Ryan JJ, Jacobsen DJ, Williams SL. Physical Activity Across the Curriculum (PAAC): a randomized controlled trial to promote physical activity and diminish overweight and obesity in elementary school children. Preventive Medicine 2009 October;49(4):336-41. Not All

Participants were Overweight and/or Obese
Ref ID: 101

(1396) Donnelly JE, Lambourne K. Classroom-based physical activity, cognition, and academic achievement. [Review]. Preventive Medicine 2011 June;52:Suppl-42. Review article
Ref ID: 1044

(1397) Donnelly JE, Greene JL, Gibson CA, Sullivan DK, Hansen DM, Hillman CH, Poggio J, Mayo MS, Smith BK, Lambourne K, Herrmann SD, Scudder M, Betts JL, Honas JJ, Washburn RA. Physical activity and academic achievement across the curriculum (A + PAAC): rationale and design of a 3-year, cluster-randomized trial. BMC Public Health 2013;13:307. Inappropriate Study Design
Ref ID: 5835

(1398) Donnelly JE, Honas JJ, Smith BK, Mayo MS, Gibson CA, Sullivan DK, Lee J, Herrmann SD, Lambourne K, Washburn RA. Aerobic exercise alone results in clinically significant weight loss for men and women: midwest exercise trial 2. Obesity (Silver Spring) 2013 March;21(3):E219-E228. Inappropriate Population
Ref ID: 5834

(1399) Dore E, Martin R, Ratel S, Duche P, Bedu M, Van PE. Gender differences in peak muscle performance during growth. International Journal of Sports Medicine 2005 May;26(4):274-80. Cross-sectional study
Ref ID: 1532

(1400) Dorofeyeva EE, Dorofeyev AE. Biochemical and heart adaptations to physical training and supplementation with amino acids. Journal of Strength and Conditioning Research 2004 November;18(4):738-40. Diet Intervention or Supplement Study
Ref ID: 1568

(1401) Dorosko SM. Vitamin A, Mastitis, and Mother-to-Child Transmission of HIV-1 through Breast-feeding: Current Information and Gaps in Knowledge. Nutrition Reviews 2005 October;63(10):332-46. Review article
Ref ID: 3567

(1402) Dotan R, Ohana S, Bediz C, Falk B. Blood lactate disappearance dynamics in boys and men following exercise of similar and dissimilar peak-lactate concentrations. Journal of Pediatric Endocrinology 2003 March;16(3):419-29. Acute study
Ref ID: 1757

(1403) Dougherty KA, Baker LB, Chow M, Kenney WL. Two percent dehydration impairs and six percent carbohydrate drink improves boys basketball skills. Medicine and Science in Sports and Exercise 2006;38:1650-8. Acute study
Ref ID: 4745

(1404) Dougherty KA, Chow M, Kenney WL. Critical environmental limits for exercising heat-acclimated lean and obese boys. European Journal of Applied Physiology 2010 March;108(4):779-89. Not an exercise intervention study
Ref ID: 56

(1405) Douma-van Riet DC, Engelbert RH, van Genderen FR, Ter Horst-De Ronde MT, de Goede-Bolder A, Hartman A. Physical fitness in children with haemophilia and the effect of overweight. Haemophilia 2009 March;15(2):519-27. Cross-sectional study
Ref ID: 776

(1406) Dour CA, Horacek TM, Schembre SM, Lohse B, Hoerr S, Kattelmann K, White AA, Shoff S, Phillips B, Greene G. Process evaluation of Project WebHealth: a nondieting Web-based intervention for obesity prevention in college students. Journal of Nutrition Education and Behavior 2013 July;45(4):288-95. Inappropriate Population
Ref ID: 5836

(1407) Dow CB. Young Children and Movement: The Power of Creative Dance. Young Children 2010 March 1;65(2):30-5. Review article
Ref ID: 3900

(1408) Dowda M, McKenzie TL, Cohen DA, Scott MM, Evenson KR, Bedimo-Rung AL, Voorhees CC, Almeida MJ. Commercial venues as supports for physical activity in adolescent girls. Preventive Medicine 2007;45:163-8. Cross-sectional study
Ref ID: 4746

(1409) Downs DS, Feinberg M, Hillemeier MM, Weisman CS, Chase GA, Chuang CH, Parrott R, Francis LA. Design of the Central Pennsylvania Women's Health Study (CePAWHS) strong healthy women intervention: improving preconceptional health. Maternal and Child Health Journal 2009 January;13(1):18-28. Study limited to adults, Description versus conduct of study
Ref ID: 198

(1410) Downs SM, Farmer A, Quintanilha M, Berry TR, Mager DR, Willows ND, McCargar LJ. From Paper to Practice: Barriers to Adopting Nutrition Guidelines in Schools. Journal of Nutrition Education and Behavior 2012;44(2):114-22. Cross-sectional study
Ref ID: 5245

(1411) Dowson JH. Pharmacological treatment for attention-deficit/hyperactivity disorder (ADHD) in adults. Current Psychiatry Reviews 2006;2(3):317-31. Review article
Ref ID: 3222

(1412) Doyle-Baker PK, Venner AA, Lyon ME, Fung T. Impact of a combined diet and progressive exercise intervention for overweight and obese children: the B.E. H.I.P. study. Applied Physiology, Nutrition, and Metabolism = Physiologie Appliquee, Nutrition et Metabolisme 2011 August;36(4):515-25. Multiple interventions
Ref ID: 2499

(1413) Doyle-Lucas AF, Akers JD, Davy BM. Energetic efficiency, menstrual irregularity, and bone mineral density in elite professional female ballet dancers. Journal of Dance Medicine and Science 2010;14(4):146-54. Not a randomized controlled trial (RCT)
Ref ID: 2500

(1414) Doyle AC, Goldschmidt A, Huang C, Winzelberg AJ, Taylor CB, Wilfley DE. Reduction of overweight and eating disorder symptoms via the Internet in adolescents: a randomized controlled trial. Journal of Adolescent Health 2008 August;43(2):172-9. No exercise only group
Ref ID: 168

(1415) Dórea V, Ronque ERV, Cyrino ES, Serassuelo Junior H, Gobbo LA, Carvalho FO, Souza CFD, Melo JCD, Gaion PA. Aptidão física relacionada à saúde em escolares de Jequié, BA, Brasil. Revista Brasileira de Medicina do Esporte 2008 December;14(6):494-9. Cross-sectional study
Ref ID: 4099

(1416) Dreifuss FE, Rosman NP, Cloyd JC, Pellock JM, Kuzniecky RI, Lo WD, Matsuo F, Sharp GB, Conry JA, Bergen DC, Bell WE. A comparison of rectal diazepam gel and placebo for acute repetitive seizures. New England Journal of Medicine 1998;338:1869-75. Drug intervention study
Ref ID: 912

(1417) Dresser R. Wanted. Hastings Center Report 1992 January;22(1):24. Editorial or letter or comment
Ref ID: 579

(1418) Dreyhaupt J, Koch B, Wirt T, Schreiber A, Brandstetter S, Kesztyus D, Wartha O, Kobel S, Kettner S, Prokopchuk D, Hundsdorfer V, Klepsch M, Wiedom M, Sufeida S, Fischbach N, Muche R, Seufert T, Steinacker JM. Evaluation of a health promotion program in children: Study protocol and design of the cluster-randomized Baden-Wurttemberg primary school study [DRKS-ID: DRKS00000494]. BMC Public Health 2012;12:157. Description of study from review or magazine or etc. (not the actual study)
Ref ID: 2501

(1419) Drieling RL, Ma J, Stafford RS. Evaluating clinic and community-based lifestyle interventions for obesity reduction in a low-income Latino neighborhood: Vivamos Activos Fair Oaks Program. BMC Public Health 2011;11:98. Multiple

interventions
Ref ID: 2502

(1420) du AS, Lartey A, Brown KH, Zlotkin S, Briend A, Dewey KG, Osborn DA, Evans N, Kluckow M, Bowen JR, Rieger I. Randomized comparison of 3 types of micronutrient supplements for home fortification of complementary foods in Ghana: effects on growth and motor development Low superior vena cava flow and effect of inotropes on neurodevelopment to 3 years in preterm infants. American Journal of Clinical Nutrition 2007;86:412-20. Diet Intervention or Supplement Study
Ref ID: 4747

(1421) Du H, Xu X, Yao T, Wu X. [Application of combined epidural-spinal anesthesia in pediatric surgery and postoperative analgesia]. Beijing da xue xue bao Yi xue ban = Journal of Peking University Health Sciences 2003;35:642-4. Drug intervention study
Ref ID: 4748

(1422) Du X, Zhu K, Trube A, Zhang Q, Ma G, Hu X, Fraser DR, Greenfield H. School-milk intervention trial enhances growth and bone mineral accretion in Chinese girls aged 10-12 years in Beijing. The British Journal of Nutrition 2004;92:159-68. Diet Intervention or Supplement Study
Ref ID: 4749

(1423) Du X, Zhu K, Trube A, Zhang Q, Ma G, Hu X, Fraser DR, Greenfield H. School-milk intervention trial enhances growth and bone mineral accretion in Chinese girls aged 10-12 years in Beijing.[Erratum appears in British Journal of Nutrition. 2005 Apr;93(4):571-2]. British Journal of Nutrition 2004 July;92(1):159-68. Diet Intervention or Supplement Study
Ref ID: 1613

(1424) Du XQ, Greenfield H, Fraser DR, Ge KY, Liu ZH, He W. Milk consumption and bone mineral content in Chinese adolescent girls. Bone 2002;30:521-8. Cross-sectional study
Ref ID: 4750

(1425) Dua JS, Cooper AR, Fox KR, Graham SA. Exercise training in adults with congenital heart disease: feasibility and benefits. International Journal of Cardiology 2010 January 21;138(2):196-205. Study limited to adults
Ref ID: 594

(1426) Duarte CM, Nascimento VBd, Akerman M. Gravidez na adolescência e exclusão social: análise de disparidades intra-urbanas. Revista Panamericana de Salud Pública 2006 April;19(4):236-43. Cross-sectional study
Ref ID: 4100

(1427) DuBose KD, Mayo MS, Gibson CA, Green JL, Hill JO, Jacobsen DJ, Smith BK, Sullivan DK, Washburn RA, Donnelly JE. Physical activity across the curriculum

(PAAC): rationale and design. Contemporary Clinical Trials 2008 January;29(1):83-93. Description versus conduct of study
Ref ID: 227

(1428) Duckworth LC, Gately PJ, Radley D, Cooke CB, King RF, Hill AJ. RCT of a high-protein diet on hunger motivation and weight-loss in obese children: an extension and replication. Obesity (Silver Spring) 2009 September;17(9):1808-10. No exercise only group, No comparative control group
Ref ID: 129

(1429) Ducro-Steverink D. Selection against boar taint: a simulation study. Acta Veterinaria Scandinavica 2006 January 2;48:6-3. Animal study
Ref ID: 3568

(1430) Dudley DA, Okely AD, Pearson P, Cotton WG, Caputi P. Changes in physical activity levels, lesson context, and teacher interaction during physical education in culturally and linguistically diverse Australian schools. International Journal of Behavioral Nutrition and Physical Activity 2012;9:114. Inappropriate Study Design
Ref ID: 5837

(1431) Due A, Larsen TM, Mu H, Hermansen K, Stender S, Astrup A. Comparison of 3 ad libitum diets for weight-loss maintenance, risk of cardiovascular disease, and diabetes: a 6-mo randomized, controlled trial. American Journal of Clinical Nutrition 2008 November;88(5):1232-41. Study limited to adults, No exercise only group
Ref ID: 152

(1432) Duff SV, Gordon AM. Learning of grasp control in children with hemiplegic cerebral palsy. Developmental Medicine and Child Neurology 2003;45:746-57. Not a randomized controlled trial (RCT)
Ref ID: 4751

(1433) Duffy FH, McAnulty GB, McCreary MC, Cuchural GJ, Komaroff AL. EEG spectral coherence data distinguish chronic fatigue syndrome patients from healthy controls and depressed patients-A case control study. 11[1], 82-94. 2011. BioMed Central. Case-Control / Case Study,
Ref ID: 3569

(1434) Duffy G, Spence SH. The effectiveness of cognitive self-management as an adjunct to a behavioural intervention for childhood obesity: a research note. Journal of Child Psychology and Psychiatry, and Allied Disciplines 1993;34:1043-50. Behavior Modification Intervention
Ref ID: 4752

(1435) Dugas LR, Ebersole K, Schoeller D, Yanovski JA, Barquera S, Rivera J, Durazo-Arzivu R, Luke A. Very low levels of energy expenditure among pre-adolescent Mexican-American girls. International Journal of Pediatric Obesity

2008;3(2):123-6. Cross-sectional study
Ref ID: 957

(1436) Duggins M, Cherven P, Carrithers J, Messamore J, Harvey A. Impact of family YMCA membership on childhood obesity: a randomized controlled effectiveness trial. Journal of the American Board of Family Medicine 2010 May;23(3):323-33. No exercise only group, No comparative control group
Ref ID: 44

(1437) Duijkers IJM, Klipping C, Grob P, Korver T. Effects of a monophasic combined oral contraceptive containing nomegestrol acetate and 17Î²-oestradiol on ovarian function in comparison to a monophasic combined oral contraceptive containing drospirenone and ethinylestradiol. European Journal of Contraception and Reproductive Health Care 2010 October;15(5):314-25. Drug intervention study
Ref ID: 3570

(1438) Dujovne CA. Treatment of familial hypercholesterolemia and other genetic dyslipidemias. Current Treatment Options in Cardiovascular Medicine 2004;6(4):269-78. Review article
Ref ID: 3223

(1439) Dumith SC. Physical activity in Brazil: a systematic review. Atividade física no Brasil: uma revisão sistemática. Cadernos de Saúde Pública 2009;25(supl.3):S415-S426. Review article
Ref ID: 4101

(1440) Dumith SC, Farias Júnior JC. Sobrepeso e obesidade em crianças e adolescentes: comparação de três critérios de classificação baseados no índice de massa corporal. Overweight and obesity in children and adolescents: comparison of three classification criteria based on body mass index. Revista Panamericana de Salud Pública 2010 July;28(1):30-5. Cohort Study
Ref ID: 4103

(1441) Dumith SC, Hallal PC, Menezes AMB, Araújo CL. Sedentary behavior in adolescents: the 11-year follow-up of the 1993 Pelotas (Brazil) birth cohort study. Comportamento sedentário em adolescentes: a visita de 11 anos da coorte de nascimentos de Pelotas, Rio Grande do Sul, Brasil, 1993. Cadernos de Saúde Pública 2010 October;26(10):1928-36. Survey or questionnaire
Ref ID: 4102

(1442) Dumith SC, Domingues MR, Mendoza-Sassi RA, Cesar JA. Atividade física durante a gestação e associação com indicadores de saúde materno-infantil. Physical activity during pregnancy and its association with maternal and child health indicators. Actividad física durante la gestación y asociación con indicadores de salud materno-infantil. Revista de Saúde Pública 2012

April;46(2):327-33. Cross-sectional study
Ref ID: 4104

(1443) Dumith SdC, Azevedo Júnior MR, Rombaldi AJ. Aptidão física relacionada à saúde de alunos do ensino fundamental do município de Rio Grande, RS, Brasil. Revista Brasileira de Medicina do Esporte 2008 October;14(5):454-9. Cross-sectional study
Ref ID: 4105

(1444) Dumoulin C, Bourbonnais D, Morin M, Gravel D, Lemieux MC. Predictors of success for physiotherapy treatment in women with persistent postpartum stress urinary incontinence. Archives of Physical Medicine and Rehabilitation 2010;91:1059-63. Study limited to adults
Ref ID: 4754

(1445) Duncan BB, Schmidt MI, Polanczyk CA, Homrich CS, Rosa RdS, Achutti AC. Fatores de risco para doenças näo-transmissíveis em área metropolitana na regiäo sul do Brasil: prevalência e simultaneidade. Revista de Saúde Pública 1993 February;27(1):43-8. Survey or questionnaire
Ref ID: 814

(1446) Duncan MJ, Al-Nakeeb Y, Nevill AM. Effects of a 6-week circuit training intervention on body esteem and body mass index in British primary school children. Body Image 2009 June;6(3):216-20. Not All Participants were Overweight and/or Obese
Ref ID: 117

(1447) Dunn AL, Andersen RE, Jakicic JM. Lifestyle physical activity interventions. History, short- and long-term effects, and recommendations. [Review] [77 refs]. American Journal of Preventive Medicine 1998 November;15(4):398-412. Review article
Ref ID: 2073

(1448) Dunshea FR, Cox ML. Effect of dietary protein on body composition and insulin resistance using a pig model of the child and adolescent. Nutrition and Dietetics 2008;65:S60-S65. Animal study
Ref ID: 5246

(1449) Dunton GF, Liao Y, Intille SS, Spruijt-Metz D, Pentz M. Investigating children's physical activity and sedentary behavior using ecological momentary assessment with mobile phones. Obesity 2011 June;19(6):1205-12. Not All Participants were Overweight and/or Obese
Ref ID: 2503

(1450) Dupuis JM, Vivant JF, Daudet G, Bouvet A, Clement M, Dazord A, Dumet N, David M, Bellon G. [Personal sports training in the management of obese boys aged 12 to 16 years]. [French]. Archives de Pediatrie 2000

November;7(11):1185-93. Not a randomized controlled trial (RCT)
Ref ID: 1945

(1451) Duquia RP, Dumith SdC, Reichert FF, Madruga SW, Duro LN, Menezes AMB, Araújo CL. Epidemiologia das pregas cutâneas triciptal e subescapular elevadas em adolescentes. Cadernos de Saúde Pública 2008 January;24(1):113-21. Cross-sectional study, Cohort Study
Ref ID: 4106

(1452) Durant N, Harris SK, Doyle S, Person S, Saelens BE, Kerr J, Norman GJ, Sallis JF. Relation of school environment and policy to adolescent physical activity. Journal of School Health 205 June;79(4):153-9. Survey or questionnaire
Ref ID: 786

(1453) Durant RH, Baranowski T, Johnson M, Thompson WO. The relationship among television watching, physical activity, and body composition of young children. Pediatrics 1994 October;94(4:Pt 1):t-55. Observational study
Ref ID: 2235

(1454) Durnin JV, Aitchison TC, Beckett C, Husaini M, Pollitt E. Nutritional intake of an undernourished infant population receiving an energy and micronutrient supplement in Indonesia. European Journal of Clinical Nutrition 2000;54 Suppl 2:S43-S51. Subjects less than 2 years old
Ref ID: 848

(1455) Duruz J. Food As Nostalgia: Eating the Fifties and Sixties. Australian Historical Studies 1999 October;30(113):231. Editorial or letter or comment
Ref ID: 3571

(1456) Dutton GR, Davis MP, Welsch MA, Brantley PJ. Promoting physical activity for low-income minority women in primary care. American Journal of Health Behavior 2007 November;31(6):622-31. Study limited to adults
Ref ID: 223

(1457) Duvall J. A comparison of engagement strategies for encouraging outdoor walking. Journal of Physical Activity and Health 2012 January;9(1):62-70. Study limited to adults
Ref ID: 2504

(1458) Dvorchik B. Moderate liver impairment has no influence on daptomycin pharmacokinetics. Journal of Clinical Pharmacology 2004 July;44(7):715-22. Study limited to adults
Ref ID: 1617

(1459) Dwyer JT, Feldman HA, Yang M, Webber LS, Must A, Perry CL, Nader PR, Parcel GS. Maintenance of lightweight correlates with decreased cardiovascular risk factors in early adolescence. Journal of Adolescent Health

2002 August;31(2):117-24. Not an exercise intervention study, Cohort Study
Ref ID: 375

(1460) Dwyer T, Coonan WE, Leitch DR, Hetzel BS, Baghurst RA. An investigation of the effects of daily physical activity on the health of primary school students in South Australia. International Journal of Epidemiology 1983 September;12(3):308-13. Not a randomized controlled trial (RCT)
Ref ID: 2366

(1461) Dybdahl R. Children and mothers in war: an outcome study of a psychosocial intervention program. Child Development 2001;72:1214-30. Not an exercise intervention study
Ref ID: 4755

(1462) Dyer AR, Cutter GR, Liu KQ, Armstrong MA, Friedman GD, Hughes GH, Dolce JJ, Raczynski J, Burke G, Manolio T. Alcohol intake and blood pressure in young adults: the CARDIA Study. Journal of Clinical Epidemiology 1990;43(1):1-13. Study limited to adults
Ref ID: 2313

(1463) Dyke JV, Kirk AB, Martinelango K, Dasgupta PK. Sample processing method for the determination of perchlorate in milk. Analytica Chimica Acta 2006;567(1):73-8. Diet Intervention or Supplement Study
Ref ID: 5247

(1464) Dzewaltowski DA, Rosenkranz RR, Geller KS, Coleman KJ, Welk GJ, Hastmann TJ, Milliken GA. HOP'N after-school project: an obesity prevention randomized controlled trial. International Journal of Behavioral Nutrition and Physical Activity 2010;7. Diet & Exercise intervention
Ref ID: 5248

(1465) Eagle TF, Gurm R, Smith CA, Corriveau N, DuRussell-Weston J, Palma-Davis L, Aaronson S, Goldberg C, Kline-Rogers E, Cotts T, Jackson EA, Eagle KA. A middle school intervention to improve health behaviors and reduce cardiac risk factors. American Journal of Medicine 2013 October;126(10):903-8. Inappropriate Study Design
Ref ID: 5838

(1466) Eakin EG, Youlden DR, Baade PD, Lawler SP, Reeves MM, Heyworth JS, Fritschi L. Health behaviors of cancer survivors: data from an Australian population-based survey. Cancer Causes Control 2007 October;18(8):881-94. Not an exercise intervention study, Observational study
Ref ID: 226

(1467) Eaton DK, Kann L, Kinchen S, Ross J, Hawkins J, Harris WA, Lowry R, McManus T, Chyen D, Shanklin S, Lim C, Grunbaum JA, Wechsler H. Youth risk behavior surveillance - United States, 2005. Journal of School Health

2006;76(7):353-72. Review article
Ref ID: 5249

(1468) Eaton DK, Foti K, Brener ND, Crosby AE, Flores G, Kann L. Associations Between Risk Behaviors and Suicidal Ideation and Suicide Attempts: Do Racial/Ethnic Variations in Associations Account for Increased Risk of Suicidal Behaviors Among Hispanic/Latina 9th- to 12th-Grade Female Students? Archives of Suicide Research 2011;15(2):113-26. Survey or questionnaire
Ref ID: 5250

(1469) Eaton DK, Kann L, Kinchen S, Shanklin S, Flint KH, Hawkins J, Harris WA, Lowry R, McManus T, Chyen D, Whittle L, Lim C, Wechsler H, Centers for Disease Control and Prevention (DHHS/PHS). Youth Risk Behavior Surveillance--United States, 2011. Morbidity and Mortality Weekly Report. Surveillance Summaries. Volume 61, Number 4. Centers for Disease Control and Prevention; 2012 Jun 8.
Review article
Ref ID: 3901

(1470) Ebbeling CB, Rodriguez NR. Effects of exercise combined with diet therapy on protein utilization in obese children. Medicine and Science in Sports and Exercise 1999 March;31(3):378-85. Diet & Exercise intervention
Ref ID: 2039

(1471) Ebbeling CB, Feldman HA, Osganian SK, Chomitz VR, Ellenbogen SJ, Ludwig DS. Effects of decreasing sugar-sweetened beverage consumption on body weight in adolescents: a randomized, controlled pilot study. Pediatrics 2006 March;117(3):673-80. Not an exercise intervention study
Ref ID: 285

(1472) Ebbeling CB, Ludwig DS. Pediatric obesity prevention initiatives: more questions than answers. Archives of Pediatrics and Adolescent Medicine 2010 November;164(11):1067-9. Editorial or letter or comment
Ref ID: 2505

(1473) Ebbeling CB, Swain JF, Feldman HA, Wong WW, Hachey DL, Garcia LE, Ludwig DS. Effects of dietary composition on energy expenditure during weight-loss maintenance. Journal of the American Medical Association 2012;307:2627-34. Diet Intervention Study
Ref ID: 4756

(1474) Ebbeling CB, Pawlak DB, Ludwig DS. Childhood obesity: public-health crisis, common sense cure. Lancet 2002 August 10;360(9331):473. Review article
Ref ID: 3572

(1475) Ebben WP, Wurm B, VanderZanden TL, Spadavecchia ML, Durocher JJ, Bickham CT, Petushek EJ. Kinetic analysis of several variations of push-ups. Journal of Strength and Conditioning Research 2011 October;25(10):2891-4.

Not a randomized controlled trial (RCT)
Ref ID: 2506

(1476) Ebrahim S, Smith GD. Systematic review of randomised controlled trials of multiple risk factor interventions for preventing coronary heart disease. British Medical Journal 1997 June 7;314(7095):1666-74. Review article
Ref ID: 2136

(1477) Ebrahimi S, Pormahmodi A, Kamkar A. Study of zinc supplementation on growth of schoolchildren in Yasuj, Southwest of Iran. Pakistan Journal of Nutrition 2006;5:341-2. Diet Intervention or Supplement Study
Ref ID: 4757

(1478) Economos CD, Hyatt RR, Must A, Goldberg JP, Kuder J, Naumova EN, Collins JJ, Nelson ME. Shape Up Somerville two-year results: a community-based environmental change intervention sustains weight reduction in children. Preventive Medicine 2013 October;57(4):322-7. Inappropriate Study Design
Ref ID: 5839

(1479) Edgar TS. Clinical Utility Botulinum Toxin in the Treatment of Cerebral Palsy: Comprehensive Review. Journal of Child Neurology 2001 January;16(1):37. Review article
Ref ID: 3573

(1480) Edouard T, Deal C, Van VG, Gaulin N, Moreau A, Rauch F, Alos N. Muscle-bone characteristics in children with Prader-Willi syndrome. Journal of Clinical Endocrinology and Metabolism 2012 February;97(2):E275-E281. Not an exercise intervention study
Ref ID: 2507

(1481) Eek MN, Tranberg R, Zugner R, Alkema K, Beckung E. Muscle strength training to improve gait function in children with cerebral palsy. Developmental Medicine and Child Neurology 2008 October;50(10):759-64. Not a randomized controlled trial (RCT)
Ref ID: 884

(1482) Efrat MW. Exploring effective strategies for increasing the amount of moderate-to-vigorous physical activity children accumulate during recess: a quasi-experimental intervention study. Journal of School Health 2013 April;83(4):265-72. Inappropriate Study Design
Ref ID: 5840

(1483) Egede LE. Lifestyle modification to improve blood pressure control in individuals with diabetes: is physician advice effective? Diabetes Care 2003 March;26(3):602-7. Study limited to adults
Ref ID: 1772

(1484) Eguchi R, Cheik NC, Oyama LM, do Nascimento CMO, de Mello MT, Tufik S, Damaso A. Effects of the Chronic Exercise on the Circulating Concentration of Leptin and Ghrelin in Rats With Diet-induced Obesity. Revista Brasileira de Medicina do Esporte 2008;14(3):182-7. Animal study
Ref ID: 5251

(1485) Ehlers DK, Huberty JL, Beseler CL. Changes in community readiness among key school stakeholders after Ready for Recess. Health Education Research 2013 December;28(6):943-53. Inappropriate Intervention
Ref ID: 5841

(1486) Eiben G, Lissner L. Health Hunters--an intervention to prevent overweight and obesity in young high-risk women. International Journal of Obesity (London) 2006 April;30(4):691-6. Study limited to adults
Ref ID: 294

(1487) Eiholzer U, Gisin R, Weinmann C, Kriemler S, Steinert H, Torresani T, Zachmann M, Prader A. Treatment with human growth hormone in patients with Prader-Labhart-Willi syndrome reduces body fat and increases muscle mass and physical performance. European Journal of Pediatrics 1998 May;157(5):368-77. Drug intervention study
Ref ID: 2089

(1488) Eiholzer U, l'Allemand D, Schlumpf M, Rousson V, Gasser T, Fusch C. Growth hormone and body composition in children younger than 2 years with Prader-Willi syndrome. The Journal of Pediatrics 2004;144:753-8. Subjects less than 2 years old
Ref ID: 4758

(1489) Eiholzer U, Grieser J, Schlumpf M, l'Allemand D. Clinical effects of treatment for hypogonadism in male adolescents with prader-labhart-willi syndrome. Hormone Research 2007;68(4):178-84. Drug intervention study
Ref ID: 5252

(1490) Eiholzer U, Meinhardt U, Petro R, Witassek F, Gutzwiller F, Gasser T. High-intensity training increases spontaneous physical activity in children: a randomized controlled study. Journal of Pediatrics 2010 February;156(2):242-6. No comparative control group
Ref ID: 88

(1491) Einfeld S, GÃ¶tz J, Holsinger RMD. Pathogenetic mechanisms in Down Syndrome. Journal of Intellectual Disability Research 2008 October;52(10):813. Review article
Ref ID: 3812

(1492) Eisele R, Maier E, Kinzl L, Gude U. [Stationary thromboprophylaxis in casualty surgery. Relevance of postoperative mobility and preexisting risk factors]. [German]. Unfallchirurg 2004 April;107(4):294-9. Not an exercise intervention

study
Ref ID: 1638

(1493) Eisenmann JC, Pivarnik JM, Malina RM. Scaling peak VO2 to body mass in young male and female distance runners. Journal of Applied Physiology 2001 June;90(6):2172-80. Not a randomized controlled trial (RCT)
Ref ID: 1918

(1494) Eisenmann JC, Welk GJ, Wickel EE, Blair SN. Combined influence of cardiorespiratory fitness and body mass index on cardiovascular disease risk factors among 8-18 year old youth: The Aerobics Center Longitudinal Study. International Journal of Pediatric Obesity 2007;2(2):66-72. Not a randomized controlled trial (RCT)
Ref ID: 1173

(1495) Eisenmann JC, Gentile DA, Welk GJ, Callahan R, Strickland S, Walsh M, Walsh DA. SWITCH: rationale, design, and implementation of a community, school, and family-based intervention to modify behaviors related to childhood obesity. BMC Public Health 2008 June 29;8:223.:223. Description versus conduct of study
Ref ID: 175

(1496) Eisenmann JC, Subcommittee on Assessment in Pediatric Obesity Management Programs NAoCHaRI. Assessment of obese children and adolescents: a survey of pediatric obesity-management programs. Pediatrics 2011 September;128:Suppl-8. Survey or questionnaire
Ref ID: 2508

(1497) Ek KL, Wang S, Copeland L, Brand-Miller JC. Discovery of a low-glycaemic index potato and relationship with starch digestion in vitro. British Journal of Nutrition 2014 February;111(4):699-705. Inappropriate Intervention
Ref ID: 5842

(1498) Ekblom O, Oddsson K, Ekblom B. Health-related fitness in Swedish adolescents between 1987 and 2001. Acta Paediatrica 2004 May;93(5):681-6. Cross-sectional study
Ref ID: 1620

(1499) Ekelund U, Anderssen SA, Froberg K, Sardinha LB, Andersen LB, Brage S, European Youth Heart Study Group. Independent associations of physical activity and cardiorespiratory fitness with metabolic risk factors in children: the European youth heart study. Diabetologia 2007 September;50(9):1832-40. Cross-sectional study
Ref ID: 1188

(1500) El-Hajj FG, Nabulsi M, Tamim H, Maalouf J, Salamoun M, Khalife H, Choucair M, Arabi A, Vieth R. Effect of vitamin D replacement on musculoskeletal parameters in school children: a randomized controlled trial. Journal of Clinical

Endocrinology and Metabolism 2006 February;91(2):405-12. Diet Intervention or Supplement Study
Ref ID: 1423

(1501) El AW, El AS, Moseley L. Associations between physical activity and health parameters in adolescent pupils in Egypt. International Journal of Environmental Research and Public Health 2010;7:1649-69. Not All Participants were Overweight and/or Obese
Ref ID: 4759

(1502) Elbl L, Hrstkova H, Tomaskova I, Blazek B, Michalek J. Long-term serial echocardiographic examination of late anthracycline cardiotoxicity and its prevention by dexrazoxane in paediatric patients. European Journal of Pediatrics 2005 November;164(11):678-84. Drug intervention study
Ref ID: 1461

(1503) Elder JP, McGraw SA, Stone EJ, Reed DB, Harsha DW, Greene T, Wambsgans KC. CATCH: process evaluation of environmental factors and programs. Health Education Quarterly Supplement 2:S107-27, 1994 1994;S107-S127. Description of study from review or magazine or etc. (not the actual study)
Ref ID: 2248

(1504) Elder JP, McKenzie TL, Arredondo EM, Crespo NC, Ayala GX. Effects of a multi-pronged intervention on children's activity levels at recess: the Aventuras para Ninos study. Advances in Nutrition 2011 March;2(2):171S-6S. Not All Participants were Overweight and/or Obese
Ref ID: 2509

(1505) Elferink-Gemser MT, Visscher C, Lemmink KA, Mulder TW. Relation between multidimensional performance characteristics and level of performance in talented youth field hockey players. Journal of Sports Sciences 2004 November;22(11-12):1053-63. Not a randomized controlled trial (RCT)
Ref ID: 1558

(1506) Elia M, Ritz P. Total energy expenditure in the elderly. European Journal of Clinical Nutrition 2000 June 2;54(6):S92. Abstract
Ref ID: 511

(1507) Elia M. Changing concepts of nutrient requirements in disease: Implications for artificial nutritional. Lancet 1995 May 20;345(8960):1279. Review article
Ref ID: 570

(1508) Eliakim A, Barstow TJ, Brasel JA, Ajie H, Lee WN, Renslo R, Berman N, Cooper DM. Effect of exercise training on energy expenditure, muscle volume, and maximal oxygen uptake in female adolescents. Journal of Pediatrics 1996 October;129(4):537-43. Not All Participants were Overweight and/or Obese
Ref ID: 2160

(1509) Eliakim A, Burke GS, Cooper DM. Fitness, fatness, and the effect of training assessed by magnetic resonance imaging and skinfold-thickness measurements in healthy adolescent females. American Journal of Clinical Nutrition 1997 August;66(2):223-31. Cross-sectional study
Ref ID: 2130

(1510) Eliakim A, Brasel JA, Mohan S, Wong WL, Cooper DM. Increased physical activity and the growth hormone-IGF-I axis in adolescent males. American Journal of Physiology 1998 July;275(1:Pt 2):t-14. Not All Participants were Overweight and/or Obese
Ref ID: 2085

(1511) Eliakim A, Makowski GS, Brasel JA, Cooper DM. Adiposity, lipid levels, and brief endurance training in nonobese adolescent males. International Journal of Sports Medicine 2000 July;21(5):332-7. Not All Participants were Overweight and/or Obese
Ref ID: 1963

(1512) Eliakim A, Scheett T, Allmendinger N, Brasel JA, Cooper DM. Training, muscle volume, and energy expenditure in nonobese American girls. Journal of Applied Physiology 2001 January;90(1):35-44. Not All Participants were Overweight and/or Obese
Ref ID: 1937

(1513) Eliakim A, Scheett TP, Newcomb R, Mohan S, Cooper DM. Fitness, training, and the growth hormone-->insulin-like growth factor I axis in prepubertal girls. Journal of Clinical Endocrinology and Metabolism 2001 June;86(6):2797-802. Not All Participants were Overweight and/or Obese
Ref ID: 1915

(1514) Eliakim A, Kaven G, Berger I, Friedland O, Wolach B, Nemet D. The effect of a combined intervention on body mass index and fitness in obese children and adolescents - a clinical experience. European Journal of Pediatrics 2002 August;161(8):449-54. Not a randomized controlled trial (RCT)
Ref ID: 1805

(1515) Eliakim A, Friedland O, Kowen G, Wolach B, Nemet D. Parental obesity and higher pre-intervention BMI reduce the likelihood of a multidisciplinary childhood obesity program to succeed--a clinical observation. Journal of Pediatric Endocrinology 2004 August;17(8):1055-61. Multiple interventions
Ref ID: 1586

(1516) Eliakim A, Barzilai M, Wolach B, Nemet D. Should we treat elevated thyroid stimulating hormone levels in obese children and adolescents? International Journal of Pediatric Obesity 2006;1(4):217-21. Inappropriate Outcomes
Ref ID: 1300

(1517) Eliakim A, Nemet D, Balakirski Y, Epstein Y. The effects of nutritional-physical activity school-based intervention on fatness and fitness in preschool children. Journal of Pediatric Endocrinology and Metabolism 2007 June;20(6):711-8. No exercise only group
Ref ID: 224

(1518) Eliasson AC, Forssberg H, Ikuta K, Apel I, Westling G, Johansson R. Development of Human Precision Grip .5. Anticipatory and Triggered Grip Actions During Sudden Loading. Experimental Brain Research 1995;106(3):425-33. Acute study
Ref ID: 5254

(1519) Eliasson K, Elfving B, Nordgren B, Mattsson E. Urinary incontinence in women with low back pain. Manual Therapy 2008 June;13(3):206-12. Survey or questionnaire
Ref ID: 975

(1520) Ellenbecker TS, Roetert EP. An isokinetic profile of trunk rotation strength in elite tennis players. Medicine and Science in Sports and Exercise 2004 November;36(11):1959-63. Not a randomized controlled trial (RCT)
Ref ID: 1575

(1521) Elliot DL, Moe EL, Goldberg L, DeFrancesco CA, Durham MB, Hix-Small H. Definition and outcome of a curriculum to prevent disordered eating and body-shaping drug use. Journal of School Health 2006 February;76(2):67-73. Survey or questionnaire
Ref ID: 1419

(1522) Elliott JG. Practitioner Review: School Refusal: Issues of Conceptualisation, Assessment, and Treatment. Journal of Child Psychology and Psychiatry and Allied Disciplines 1999 October;40(7):1001. Review article
Ref ID: 3574

(1523) Ellis DA, Janisse H, Naar-King S, Kolmodin K, Jen KL, Cunningham P, Marshall S. The effects of multisystemic therapy on family support for weight loss among obese African-American adolescents: findings from a randomized controlled trial. Journal of Developmental and Behavioral Pediatrics 2010 July;31(6):461-8. No exercise only group
Ref ID: 34

(1524) Ellis JA, Ponsonby AL, Pezic A, Williamson E, Cochrane JA, Dickinson JL, Dwyer T. APOE Genotype and Cardio-Respiratory Fitness Interact to Determine Adiposity in 8-Year-Old Children from the Tasmanian Infant Health Survey. PLoS ONE 2011;6(11). Cohort Study
Ref ID: 5255

(1525) Elloumi M, Ben Ounis O, Makni E, Van Praagh E, Tabka Z, Lac G. Effect of individualized weight-loss programmes on adiponectin, leptin and resistin levels

in obese adolescent boys. Acta Paediatrica 2009;98(9):1487-93. No comparative control group
Ref ID: 5256

(1526) Ells LJ, Hillier FC, Shucksmith J, Crawley H, Harbige L, Shield J, Wiggins A, Summerbell CD. A systematic review of the effect of dietary exposure that could be achieved through normal dietary intake on learning and performance of school-aged children of relevance to UK schools. [Review] [50 refs]. British Journal of Nutrition 2008 November;100(5):927-36. Review article
Ref ID: 883

(1527) Ellsworth DL, Coady SA, Chen W, Srinivasan SR, Elkasabany A, Gustat J, Boerwinkle E, Berenson GS. Influence of the beta 2-adrenergic receptor Arg16Gly polymorphism on longitudinal changes in obesity from childhood through young adulthood in a biracial cohort: the Bogalusa Heart Study. International Journal of Obesity 2002;26(7):928-37. Cross-sectional study
Ref ID: 5257

(1528) Elmahgoub SM, Lambers S, Stegen S, Van LC, Cambier D, Calders P. The influence of combined exercise training on indices of obesity, physical fitness and lipid profile in overweight and obese adolescents with mental retardation. European Journal of Pediatrics 2009 November;168(11):1327-33. Not a randomized controlled trial (RCT)
Ref ID: 675

(1529) Elmahgoub SS, Calders P, Lambers S, Stegen SM, Van LC, Cambier DC. The effect of combined exercise training in adolescents who are overweight or obese with intellectual disability: the role of training frequency. Journal of Strength and Conditioning Research 2011 August;25(8):2274-82. Not a randomized controlled trial (RCT)
Ref ID: 2510

(1530) Elnour A, Hambraeus L, Eltom M, Dramaix M, Bourdoux P, Caccetta RA-A, Kroft KD, Beilin LJ, Puddey IB, Jooste PL, Weight MJ, Lombard CJ, Zimmermann M, Adou P, Torresani T, Zeder C, Hurrell R, Hunt J, Roughead ZK. Current literature. Journal of Human Nutrition and Dietetics 13[3], 225-239. 2000. Abstract,
Ref ID: 3575

(1531) Elster J. Don't Burn Your Bridge Before You Come To It: Some Ambiguities and Complexities of Precommitment. Texas Law Review 2003 June;81(7):1751. Editorial or letter or comment
Ref ID: 3576

(1532) Emes C, Velde B, Moreau M, Murdoch DD, Trussell R. An activity based weight control program. Adapted Physical Activity Quarterly 1990;7:314-24. No

exercise only group
Ref ID: 452

(1533) Eneli IU, Skybo T, Camargo CA, Jr. Weight loss and asthma: a systematic review. [Review] [68 refs]. Thorax 2008 August;63(8):671-6. Review article
Ref ID: 910

(1534) Enes CC, Slater B. Obesidade na adolescência e seus principais fatores determinantes. Obesity in adolescence and its main determinants. Revista Brasileira de Eidemiologia 2010 March;13(1):163-71. Review article
Ref ID: 4107

(1535) Engel SM, Zhu C, Berkowitz GS, Calafat AM, Silva MJ, Miodovnik A, Wolff MS. Prenatal phthalate exposure and performance on the Neonatal Behavioral Assessment Scale in a multiethnic birth cohort. Neurotoxicology 2009;30(4):522-8. Cohort Study
Ref ID: 5258

(1536) Engstrom C, Davidson D. Hot Flashes. 10[4], 533-535. 2006. Oncology Nursing Society. Case-Control / Case Study,
Ref ID: 3577

(1537) Entin PL, Gest C, Trancik S, Richard CJ. Fuel oxidation in relation to walking speed: influence of gradient and external load. European Journal of Applied Physiology 2010 October;110(3):515-21. Not a randomized controlled trial (RCT)
Ref ID: 2511

(1538) Epstein LH, Wing RR, Koeske R, Valoski A. Effects of diet plus exercise on weight change in parents and children. Journal of Consulting and Clinical Psychology 1984 June;52(3):429-37. No exercise only group
Ref ID: 443

(1539) Epstein LH, Wing RR, Penner BC, Kress MJ. Effect of diet and controlled exercise on weight loss in obese children. Journal of Pediatrics 1985 September;107(3):358-61. No exercise only group
Ref ID: 448

(1540) Epstein LH, Kuller LH, Wing RR, Valoski A, McCurley J. The effect of weight control on lipid changes in obese children. American Journal of Diseases of Children 1989 April;143(4):454-7. Behavior Modification Intervention
Ref ID: 2325

(1541) Epstein LH, McCurley J, Wing RR, Valoski A. Five-year follow-up of family-based behavioral treatments for childhood obesity. Journal of Consulting and Clinical Psychology 1990 October;58(5):661-4. No exercise only group
Ref ID: 440

(1542) Epstein LH, Valoski A, Wing RR, McCurley J. Ten-year follow-up of behavioral, family-based treatment for obese children. Journal of the American Medical Association 1990 November 21;264(19):2519-23. No exercise only group
Ref ID: 439

(1543) Epstein LH, McKenzie SJ, Valoski A, Klein KR, Wing RR. Effects of mastery criteria and contingent reinforcement for family-based child weight control. Addictive Behaviors 1994 March;19(2):135-45. No exercise only group
Ref ID: 434

(1544) Epstein LH, Valoski A, Wing RR, McCurley J. Ten-year outcomes of behavioral family-based treatment for childhood obesity. Health Psychology 1994 September;13(5):373-83. No exercise only group
Ref ID: 432

(1545) Epstein LH, Valoski AM, Vara LS, McCurley J, Wisniewski L, Kalarchian MA, Klein KR, Shrager LR. Effects of decreasing sedentary behavior and increasing activity on weight change in obese children. Health Psychology 1995 March;14(2):109-15. No comparative control group
Ref ID: 431

(1546) Epstein LH, Valoski AM, Kalarchian MA, McCurley J. Do children lose and maintain weight easier than adults: a comparison of child and parent weight changes from six months to ten years. Obesity Research 1995 September;3(5):411-7. No exercise only group
Ref ID: 430

(1547) Epstein LH, Coleman KJ, Myers MD. Exercise in treating obesity in children and adolescents. [Review] [52 refs]. Medicine and Science in Sports and Exercise 1996 April;28(4):428-35. Review article
Ref ID: 2179

(1548) Epstein LH, Saelens BE, Myers MD, Vito D. Effects of decreasing sedentary behaviors on activity choice in obese children. Health Psychology 1997 March;16(2):107-13. Not an exercise intervention study
Ref ID: 426

(1549) Epstein LH. Integrating theoretical approaches to promote physical activity. American Journal of Preventive Medicine 1998;15(4):257-65. Review article
Ref ID: 5259

(1550) Epstein LH, Myers MD, Raynor HA, Saelens BE. Treatment of pediatric obesity. [Review] [135 refs]. Pediatrics 1998 March;101(3:Pt 2):t-70. Review article
Ref ID: 2108

(1551) Epstein LH, Paluch RA, Gordy CC, Dorn J. Decreasing sedentary behaviors in treating pediatric obesity. Archives of Pediatric Adolescent Medicine 2000

March;154(3):220-6. No exercise only group, No comparative control group
Ref ID: 403

(1552) Epstein LH, Paluch RA, Raynor HA. Sex differences in obese children and siblings in family-based obesity treatment. Obesity Research 2001 December;9(12):746-53. No comparative control group
Ref ID: 390

(1553) Epstein LH, Paluch RA, Consalvi A, Riordan K, Scholl T. Effects of manipulating sedentary behavior on physical activity and food intake. Journal of Pediatrics 2002 March;140(3):334-9. Study less than 4 weeks
Ref ID: 1841

(1554) Epstein LH, Roemmich JN, Saad FG, Handley EA. The value of sedentary alternatives influences child physical activity choice. International Journal of Behavioral Medicine 2004;11:236-42. Not All Participants were Overweight and/or Obese
Ref ID: 4762

(1555) Epstein LH, Paluch RA, Kilanowski CK, Raynor HA. The effect of reinforcement or stimulus control to reduce sedentary behavior in the treatment of pediatric obesity. Health Psychology 2004 July;23(4):371-80. No exercise only group, No comparative control group
Ref ID: 327

(1556) Epstein LH, Roemmich JN, Paluch RA, Raynor HA. Influence of changes in sedentary behavior on energy and macronutrient intake in youth. American Journal of Clinical Nutrition 2005 February;81(2):361-6. Not All Participants were Overweight and/or Obese
Ref ID: 1546

(1557) Epstein LH, Roemmich JN, Paluch RA, Raynor HA. Physical activity as a substitute for sedentary behavior in youth. Annals of Behavioral Medicine 2005 June;29(3):200-9. Study less than 4 weeks
Ref ID: 1507

(1558) Epstein LH, Roemmich JN, Stein RI, Paluch RA, Kilanowski CK. The challenge of identifying behavioral alternatives to food: clinic and field studies. Annals of Behavioral Medicine 2005 December;30(3):201-9. No exercise only group, No comparative control group
Ref ID: 292

(1559) Epstein LH, Roemmich JN, Robinson JL, Paluch RA, Winiewicz DD, Fuerch JH, Robinson TN. A randomized trial of the effects of reducing television viewing and computer use on body mass index in young children. Archives of Pediatric Adolescent Medicine 2008 March;162(3):239-45. Not an exercise intervention study
Ref ID: 190

(1560) Epstein LH, Roemmich JN, Cavanaugh MD, Paluch RA. The motivation to be sedentary predicts weight change when sedentary behaviors are reduced. International Journal of Behavioral Nutrition and Physical Activity 2011;8:13. Study less than 4 weeks
Ref ID: 2512

(1561) Epstein LH, Raja S, Daniel TO, Paluch RA, Wilfley DE, Saelens BE, Roemmich JN. The built environment moderates effects of family-based childhood obesity treatment over 2 years. Annals of Behavioral Medicine 2012 October;44(2):248-58. Inappropriate Intervention
Ref ID: 5843

(1562) Erickson C, Stigler K, Wink L, Mullett J, Kohn A, Posey D, McDougle C. A prospective open-label study of aripiprazole in fragile X syndrome. Psychopharmacology 2011 July;216(1):85-90. Drug intervention study
Ref ID: 3578

(1563) Eriksen J, Mujinja P, Warsame M, Nsimba S, Kouyaté B, Gustafsson LL, Jahn A, Müller O, Sauerborn R, Tomson G. Effectiveness of a community intervention on malaria in rural Tanzania - a randomised controlled trial. African Health Sciences 2010;10:332-40. Survey or questionnaire
Ref ID: 4763

(1564) Erwin HE, Woods AM, Woods MK, Castelli DM. Chapter 6: Children's Environmental Access in Relation to Motor Competence, Physical Activity, and Fitness. Journal of Teaching in Physical Education 2007 October 1;26(4):404-15. Survey or questionnaire
Ref ID: 3902

(1565) Esco MR, Olson MS, Williford H. Relationship of push-ups and sit-ups tests to selected anthropometric variables and performance results: a multiple regression study. Journal of Strength and Conditioning Research 2008 November;22(6):1862-8. Cohort Study
Ref ID: 865

(1566) Escobar-Chaves SL, Markham CM, Addy RC, Greisinger A, Murray NG, Brehm B. The Fun Families Study: intervention to reduce children's TV viewing. Obesity (Silver Spring) 2010 February;18 Suppl 1:S99-101.:S99-101. No exercise only group
Ref ID: 64

(1567) Espana-Romero V, Ortega FB, Ruiz JR, Artero EG, Martinez-Gomez D, Vicente-Rodriguez G, Moliner-Urdiales D, Gracia-Marco L, Ciarapica D, Widhalm K, Castillo Garzon MJ, Sjostrom M, Moreno LA. Role of cardiorespiratory fitness on the association between physical activity and abdominal fat content in adolescents: the HELENA study. International Journal

of Sports Medicine 2010 October;31(10):679-82. Cross-sectional study
Ref ID: 2513

(1568) Espinoza-Navarro O, Vega C, Urrutia A, Moreno A, Rodríguez H. Patrones antropométricos y consumo máximo de oxígeno (VO2) entre niños escolares chilenos Aymaras y no Aymaras de 10 a 12 años, que viven en altura (3.500 msnm) y en la planicie (500 msnm). Anthropometric patterns and oxygen consumption (VO2) of school-children Aymara and non-Aymara of 10-12 years, living in high altitude (3500m) and the plain (500 m), from Chile. International Journal of Morphology 2009 December;27(4):1313-8. Cross-sectional study
Ref ID: 4108

(1569) Esquivel Lauzurique M, González Fernández C. Desarrollo físico y nutrición de preescolares habaneros según nuevos patrones de crecimiento de la OMS. Physical development and nutritional status of preschool-age children in Havana according to the new WHO Growth References. Revista Cubana de Salud Pública 2009 March;35(1):0. Cross-sectional study
Ref ID: 4109

(1570) Esquivel Solis V. Factores asociados a la obesidad en mujeres en edad fértil. Revista Costarricense de Salud Pública 2004 December;13(25):42-7. Cross-sectional study
Ref ID: 4110

(1571) Esquivel Solís V, Alvarado MV. Estado nutricional de mujeres con sobrepeso y obesidad del área de cobertura del programa de atención integral en salud (PAIS) 2006ies. Acta Médica Costarricense 2009 December;51(4):222-8. Study limited to adults
Ref ID: 4111

(1572) Estabrooks PA, Shoup JA, Gattshall M, Dandamudi P, Shetterly S, Xu S. Automated telephone counseling for parents of overweight children: a randomized controlled trial. American Journal of Preventive Medicine 2009 January;36(1):35-42. No exercise only group
Ref ID: 141

(1573) Estalella I, Rica I, Perez de NG, Bilbao JR, Vazquez JA, San Pedro JI, Busturia MA, Castano L, Spanish MODY Group. Mutations in GCK and HNF-1alpha explain the majority of cases with clinical diagnosis of MODY in Spain. Clinical Endocrinology 2007 October;67(4):538-46. Not an exercise intervention study
Ref ID: 1164

(1574) Eston RG, Rowlands AV, Ingledew DK. Validity of heart rate, pedometry, and accelerometry for predicting the energy cost of children's activities. Journal of Applied Physiology 1998 January;84(1):362-71. Cross-sectional study
Ref ID: 2104

(1575) Estrada MC, Velásquez MI, Orrego M. Neumomediastino espontáneo: Reporte de un caso y revisión de la literatura. Spontaneous pneumomediastinum: A case report and literature review. CES Medicine 2009 December;23(2):47-53. Case-Control / Case Study
Ref ID: 4112

(1576) Evangelista LS, Doering LV, Lennie T, Moser DK, Hamilton MA, Fonarow GC, Dracup K. Usefulness of a home-based exercise program for overweight and obese patients with advanced heart failure. American Journal of Cardiology 2006 March 15;97(6):886-90. Study limited to adults
Ref ID: 284

(1577) Evans BW, Claiborne JM. Health Related Physical Fitness: Who, What, Why, and How. 1982 Jan 1. Review article
Ref ID: 3903

(1578) Evenson KR, Scott MM, Cohen DA, Voorhees CC. Girls' perception of neighborhood factors on physical activity, sedentary behavior, and BMI. Obesity 2007 February;15(2):430-45. Survey or questionnaire
Ref ID: 1276

(1579) Evenson KR, Murray DM, Birnbaum AS, Cohen DA. Examination of perceived neighborhood characteristics and transportation on changes in physical activity and sedentary behavior: The Trial of Activity in Adolescent Girls. Health and Place 2010;16:977-85. Not a randomized controlled trial (RCT)
Ref ID: 4764

(1580) Everitt AV, Hilmer SN, Brand-Miller JC, Jamieson HA, Truswell AS, Sharma AP, Mason RS, Morris BJ, Le Couteur DG. Dietary approaches that delay age-related diseases. [Review] [336 refs]. Clinical Interventions in Aging 2006;1(1):11-31. Review article
Ref ID: 1297

(1581) Exl BM, Deland U, Wall M, Preysch U, Secretin MC, Shmerling DH. Zug-Frauenfeld nutritional survey ('Zuff Study'): Allergen-reduced nutrition in a normal infant population and its health-related effects: Results at the age of six months. Nutrition Research 1998;18:1443-62. Subjects less than 2 years old
Ref ID: 4765

(1582) Eyler AA, Brownson RC, Doescher MP, Evenson KR, Fesperman CE, Litt JS, Pluto D, Steinman LE, Terpstra JL, Troped PJ, Schmid TL. Policies related to active transport to and from school: a multisite case study. Health Education Research 2008 December;23(6):963-75. Case-Control / Case Study
Ref ID: 860

(1583) Ezendam NP, Oenema A, van de Looij-Jansen PM, Brug J. Design and evaluation protocol of "FATaintPHAT", a computer-tailored intervention to

prevent excessive weight gain in adolescents. BMC Public Health 2007 November 12;7:324.:324. Description versus conduct of study
Ref ID: 212

(1584) Ezendam NP, Brug J, Oenema A. Evaluation of the Web-based computer-tailored FATaintPHAT intervention to promote energy balance among adolescents: results from a school cluster randomized trial. Archives of Pediatrics and Adolescent Medicine 2012 March;166(3):248-55. Multiple interventions
Ref ID: 2514

(1585) Ezendam NP, Noordegraaf VS, Kroeze W, Brug J, Oenema A. Process evaluation of FATaintPHAT, a computer-tailored intervention to prevent excessive weight gain among Dutch adolescents. Health Promotion International 2013 March;28(1):26-35. Inappropriate Study Design
Ref ID: 5844

(1586) Fabricatore AN, Wadden TA, Ebbeling CB, Thomas JG, Stallings VA, Schwartz S, Ludwig DS. Targeting dietary fat or glycemic load in the treatment of obesity and type 2 diabetes: a randomized controlled trial. Diabetes Research and Clinical Practice 2011 April;92(1):37-45. Study limited to adults
Ref ID: 1059

(1587) Fabricatore AN, Wadden TA, Higginbotham AJ, Faulconbridge LF, Nguyen AM, Heymsfield SB, Faith MS. Intentional weight loss and changes in symptoms of depression: a systematic review and meta-analysis. [Review]. International Journal of Obesity 2011 November;35(11):1363-76. Review article
Ref ID: 1047

(1588) Fagondes SC, Moreira GA. Apneia obstrutiva do sono em crianças. Obstructive sleep apnea in children. Jornal Brasileiro de Pneumologia 2010 June;36(supl.2):57-61. Review article
Ref ID: 4113

(1589) Fagundes AL, Ribeiro DC, Naspitz L, Garbelini LE, Vieira JK, Silva APd, Lima VdO, Fagundes DJ, Compri PC, Juliano Y. Prevalência de sobrepeso e obesidade em escolares da região de Parelheiros do município de São Paulo. Revista Paulista de Pediatria 2008 September;26(3):212-7. Cross-sectional study
Ref ID: 4114

(1590) Fahrenwald NL, Atwood JR, Walker SN, Johnson DR, Berg K. A randomized pilot test of "Moms on the Move": a physical activity intervention for WIC mothers. Annals of Behavioral Medicine 2004;27:82-90. Study limited to adults
Ref ID: 4766

(1591) Fahrenwald NL, Atwood JR, Johnson DR. Mediator analysis of Moms on the move. Western journal of nursing research 2005;27:271-91. Study limited to

adults
Ref ID: 4767

(1592) Faigenbaum AD, Westcott WL, Loud RL, Long C. The effects of different resistance training protocols on muscular strength and endurance development in children. Pediatrics 1999;104:e5. Not All Participants were Overweight and/or Obese
Ref ID: 900

(1593) Faigenbaum AD, Loud RL, O'Connell J, Glover S, O'Connell J, Westcott WL. Effects of different resistance training protocols on upper-body strength and endurance development in children. Journal of Strength and Conditioning Research 2001;15:459-65. Not a randomized controlled trial (RCT)
Ref ID: 795

(1594) Faigenbaum AD, Milliken LA, Loud RL, Burak BT, Doherty CL, Westcott WL. Comparison of 1 and 2 days per week of strength training in children. Research Quarterly for Exercise and Sport 2002;73:416-24. Not a randomized controlled trial (RCT)
Ref ID: 4768

(1595) Faigenbaum AD, Farrell A, Fabiano M, Radler T, Naclerio F, Ratamess NA, Kang J, Myer GD. Effects of integrative neuromuscular training on fitness performance in children. Pediatric Exercise Science 2011 November;23(4):573-84. Not All Participants were Overweight and/or Obese
Ref ID: 1034

(1596) Faintuch J, Souza SAF, Valezi AC, Sant'Anna AF, Gama-Rodrigues JJ. Pulmonary function and aerobic capacity in asymptomatic bariatric candidates with very severe morbid obesity. Revista do Hospital das Clínicas Universidade de São Paulo 2004 August;59(4):181-6. Prospective Study
Ref ID: 4115

(1597) Fairclough SJ, Hackett AF, Davies IG, Gobbi R, Mackintosh KA, Warburton GL, Stratton G, van Sluijs EM, Boddy LM. Promoting healthy weight in primary school children through physical activity and nutrition education: a pragmatic evaluation of the CHANGE! randomised intervention study. BMC Public Health 2013;13:626. Inappropriate Intervention
Ref ID: 5845

(1598) Fairley JA, Sejdic E, Chau T. The effect of treadmill walking on the stride interval dynamics of children. Human Movement Science 2010;29(6):987-98. Acute study
Ref ID: 5261

(1599) Faith MS, Berman N, Heo M, Pietrobelli A, Gallagher D, Epstein LH, Eiden MT, Allison DB. Effects of contingent television on physical activity and television viewing in obese children. Pediatrics 2001 May;107(5):1043-8. Behavior

Modification Intervention, No comparative control group
Ref ID: 397

(1600) Faith MS, Van HL, Appel LJ, Burke LE, Carson JA, Franch HA, Jakicic JM, Kral TV, Odoms-Young A, Wansink B, Wylie-Rosett J, American Heart Association Nutrition and Obesity Committees of the Council on Nutrition, Physical Activity and Metabolism, Council on Clinical Cardiology, Council on Cardiovascular Disease in the Young, Council on Cardiovascular Nursing, Council on Epidemiology and Prevention aCotKiCD. Evaluating parents and adult caregivers as "agents of change" for treating obese children: evidence for parent behavior change strategies and research gaps: A scientific statement from the American Heart Association. Circulation 2012 March 6;125(9):1186-207. Review article
Ref ID: 2517

(1601) Falvo DR, Parker RM. Ethics in Rehabilitation Education and Research. Rehabilitation Counseling Bulletin 2000;43(4):197-214. Review article
Ref ID: 3579

(1602) Fan H, Zhang XQ, Li J. [Effects of life style intervention on obesity-related vascular dysfunction in children]. Zhonghua Liu Xing Bing Xue Za Zhi 2008 July;29(7):672-5. No exercise only group, No comparative control group
Ref ID: 148

(1603) Fan JG, Zhu J, Li XJ, Chen L, Li L, Dai F, Li F, Chen SY. Prevalence of and risk factors for fatty liver in a general population of Shanghai, China. Journal of Hepatology 2005 September;43(3):508-14. Study limited to adults
Ref ID: 1487

(1604) Fan YF, Fan YB, Li ZY, Lv CS, Luo DL. Natural Gaits of the Non-Pathological Flat Foot and High-Arched Foot. PLoS ONE 2011;6(3). Not an exercise intervention study
Ref ID: 5262

(1605) Fang PC, Kuo HK, Huang CB, Ko TY, Chen CC, Chung MY. The effect of supplementation of docosahexaenoic acid and arachidonic acid on visual acuity and neurodevelopment in larger preterm infants. Chang Gung Medical Journal 2005;28:708-15. Subjects less than 2 years old
Ref ID: 4769

(1606) Fantino M. Is the energy supplied by caloric sweetened beverages regulated? Sciences des Aliments 2007;27(4-5):301-10. Review article
Ref ID: 5263

(1607) Faria AMC, Weiner HL. Oral tolerance. Immunological Reviews 2005 August;206(1):232-59. Review article
Ref ID: 3580

(1608) Faria ERd, Franceschini SdCC, Peluzio MdCG, Priore SE. Síndrome Metabólica em adolescentes: uma atualização. Metabolic Syndrome in adolescents: an update. Nutrire Revista da Sociedade Brasileira de Alimentação e Nutrição 2009 August;34(2):179-94. Review article
Ref ID: 4116

(1609) Farias Júnior JCd, Mendes JKF, Barbosa DBM, Lopes AdS. Fatores de risco cardiovascular em adolescentes: Prevalência e associação com fatores sociodemográficos. Cardiovascular risk factors for adolescents: Prevalence and association with sociodemographic factors. Revista Brasileira de Eidemiologia 2011 March;14(1):50-62. Not a randomized controlled trial (RCT)
Ref ID: 4117

(1610) Farias Júnior JCd, Lopes AdS, Mota J, Hallal PC. Prática de atividade física e fatores associados em adolescentes no Nordeste do Brasil. Physical activity practice and associated factors in adolescents in Northeastern Brazil. Práctica de actividad física y factores asociados en adolescentes en el Noreste de Brasil. Revista de Saúde Pública 2012 June;46(3):505-15. Survey or questionnaire
Ref ID: 4118

(1611) Farias ES, Paula F, Carvalho WR, Goncalves EM, Baldin AD, Guerra-Junior G. Influence of programmed physical activity on body composition among adolescent students. Jornal de Pediatria 2009 January;85(1):28-34. Not a randomized controlled trial (RCT)
Ref ID: 800

(1612) Farias EdS, Petroski EL. Estado nutricional e atividade física de escolares da cidade de Porto Velho, RO. Revista Brasileira de Cineantropometria e Desempenho Humano 2003;5(1). Survey or questionnaire
Ref ID: 4119

(1613) Faude O, Meyer T, Scharhag J, Weins F, Urhausen A, Kindermann W. Volume vs. intensity in the training of competitive swimmers. International Journal of Sports Medicine 2008 November;29(11):906-12. Not All Participants were Overweight and/or Obese
Ref ID: 873

(1614) Faude O, Kerper O, Multhaupt M, Winter C, Beziel K, Junge A, Meyer T. Football to tackle overweight in children. Scandinavian Journal of Medicine and Science in Sports 2010 April;20 Suppl 1:103-10. Epub;2010 Feb 2.:103-10. No comparative control group
Ref ID: 62

(1615) Faulkner RA, Forwood MR, Beck TJ, Mafukidze JC, Russell K, Wallace W. Strength indices of the proximal femur and shaft in prepubertal female gymnasts. Medicine and Science in Sports and Exercise 2003

March;35(3):513-8. Cross-sectional study
Ref ID: 1770

(1616) Favier R, Caceres E, Koubi H, Sempore B, Sauvain M, Spielvogel H. Effects of coca chewing on hormonal and metabolic responses during prolonged submaximal exercise. Journal of Applied Physiology 1996 February;80(2):650-5. Not an exercise intervention study
Ref ID: 2155

(1617) Fechner H, Pinkert S, Geisler A, Poller W, Kurreck J. Pharmacological and Biological Antiviral Therapeutics for Cardiac Coxsackievirus Infections. Molecules 2011 October;16(10):8475-503. Review article
Ref ID: 3581

(1618) Fedewa MV, Gist NH, Evans EM, Dishman RK. Exercise and insulin resistance in youth: a meta-analysis. Pediatrics 2014 January;133(1):e163-e174. Inappropriate Study Design
Ref ID: 5846

(1619) Fehily AM, Coles RJ, Evans WD, Elwood PC. Factors affecting bone density in young adults. American Journal of Clinical Nutrition 56(3):579-86, 1992 Sep 1992;(3):579-86. Follow-up Study
Ref ID: 2961

(1620) Feinberg I, Higgins LM, Khaw WY, Campbell IG. The adolescent decline of NREM delta, an indicator of brain maturation, is linked to age and sex but not to pubertal stage. American Journal of Physiology Regulatory, Integrative and Comparative Physiology 2006;291:R1724-R1729. Not an exercise intervention study
Ref ID: 4770

(1621) Feinstein S, Rinat C, Becker-Cohen R, Ben-Shalom E, Schwartz SB, Frishberg Y. The outcome of chronic dialysis in infants and toddlers - advantages and drawbacks of haemodialysis. Nephrology Dialysis Transplantation 2008;23(4):1336-45. Not an exercise intervention study
Ref ID: 5264

(1622) Feitosa MF, Rice T, Rosmond R, Borecki IB, An P, Gagnon J, Leon AS, Skinner JS, Wilmore JH, Bouchard C, Rao DC. A genetic study of cortisol measured before and after endurance training: the HERITAGE Family Study. Metabolism: Clinical and Experimental 2002 March;51(3):360-5. Inappropriate Outcomes
Ref ID: 1844

(1623) Feitosa MF, Borecki IB, Rankinen T, Leon AS, Skinner JS, Wilmore JH, Bouchard C, Rao DC. Lack of pleiotropic genetic effects between adiposity and sex hormone-binding globulin concentrations before and after 20 weeks of exercise training: the HERITAGE family study. Metabolism: Clinical and

Experimental 2003 January;52(1):35-41. Inappropriate Outcomes
Ref ID: 1778

(1624) Feld JJ, Hoofnagle JH. Mechanism of action of interferon and ribavirin in treatment of hepatitis C. Nature 2005 August 18;436(7053):967-72. Drug intervention study
Ref ID: 3582

(1625) Feldman NT. Narcolepsy. Southern Medical Journal 2003 March;96(3):277. Review article
Ref ID: 3583

(1626) Fenichel GM, Florence JM, Pestronk A, Mendell JR, Moxley RT, Griggs RC, Brooke MH, Miller JP, Robison J, King W. Long-term benefit from prednisone therapy in Duchenne muscular dystrophy. Neurology 1991;41:1874-7. Drug intervention study
Ref ID: 4771

(1627) Fenn P. Assessment and management of abdominal obesity in patients with type 2 diabetes. (Cover story). Nursing Standard 2007 February 28;21(25):37-44. Review article
Ref ID: 3584

(1628) Ferber SG, Kuint J, Weller A, Feldman R, Dollberg S, Arbel E, Kohelet D. Massage therapy by mothers and trained professionals enhances weight gain in preterm infants. Early Human Development 2002;67:37-45. Subjects less than 2 years old
Ref ID: 770

(1629) Ferguson MA, Gutin B, Owens S, Barbeau P, Tracy RP, Litaker M. Effects of physical training and its cessation on the hemostatic system of obese children. American Journal of Clinical Nutrition 1999 June;69(6):1130-4. Same subjects as another study already included
Ref ID: 2024

(1630) Ferguson MA, Gutin B, Le NA, Karp W, Litaker M, Humphries M, Okuyama T, Riggs S, Owens S. Effects of exercise training and its cessation on components of the insulin resistance syndrome in obese children. International Journal of Obesity and Related Metabolic Disorders 1999 August;23(8):889-95. Same subjects as another study already included
Ref ID: 408

(1631) Ferguson TB, Syrotuik DG. Effects of creatine monohydrate supplementation on body composition and strength indices in experienced resistance trained women. Journal of Strength and Conditioning Research 2006 November;20(4):939-46. Diet Intervention or Supplement Study
Ref ID: 1311

(1632) Fermino RC, Rech CR, Hino AAF, Rodriguez Añez CR, Reis RS. Atividade física e fatores associados em adolescentes do ensino médio de Curitiba, Brasil. Physical activity and associated factors in high-school adolescents in Southern Brazil. Actividad física y factores asociados en adolescentes de enseñanza secundaria de Curitiba, Sur de Brasil. Revista de Saúde Pública 2010 December;44(6):986-95. Cross-sectional study
Ref ID: 4121

(1633) Fernald LC, Gertler PJ, Neufeld LM. 10-year effect of Oportunidades, Mexico's conditional cash transfer programme, on child growth, cognition, language, and behaviour: a longitudinal follow-up study. Lancet 2009;374:1997-2005. Follow-up Study
Ref ID: 4772

(1634) Fernandes PS, Bernardo CDO, Campos RMMB, De-Vasconcelos FDAG. Evaluating the effect of nutritional education on the prevalence of overweight/obesity and on foods eaten at primary schools. Jornal de Pediatria 2009;85:315-21. Diet Intervention Study
Ref ID: 4773

(1635) Fernandes RA, Codogno JS, Cardoso JR, Ronque ERV, Freitas Júnior IF, Oliveira AR. Fatores associados ao excesso de peso entre adolescentes de diferentes redes de ensino do município de Presidente Prudente, São Paulo. Factors associated with overweight among adolescents in different school systems in the municipality of Presidente Prudente in the State of São Paulo. Revista Brasileira de Saúde Materno Infantil 2009 December;9(4):443-9. Cross-sectional study
Ref ID: 4123

(1636) Fernandes RA, Oliveira ARd, Freitas Júnior IF. Correlação entre diferentes indicadores de adiposidade corporal e atividade física habitual em jovens do sexo masculino. Revista Brasileira de Cineantropometria e Desempenho Humano 2006 December;8(4). Survey or questionnaire
Ref ID: 598

(1637) Fernandes RA, Christofaro DGD, Cucato GG, Agostini L, Oliveira ARd, Freitas Júnior IF. Nutritional status, physical activity level, waist circumference, and flexibility in brazilian boys. Revista Brasileira de Cineantropometria e Desempenho Humano 2007 September;9(4). Cross-sectional study
Ref ID: 4124

(1638) Fernandes RA, Casonatto J, Christofaro DGD, Ronque ERV, Oliveira ARd, Freitas Júnior IF. Riscos para o excesso de peso entre adolescentes de diferentes classes socioeconômicas. Revista da Associação Médica Brasileira (1992) 2008 August;54(4):334-8. Cross-sectional study
Ref ID: 4125

(1639) Fernandes RA, Casonatto J, Christofaro DGD, Buonani C, Oliveira ARd, Freitas Júnior IF. Influência da atividade e inatividade física na composição corporal e adiposidade central. Motriz Revista de Educação Física (Improv) 2010 March;16(1):43-9. Cross-sectional study
Ref ID: 4127

(1640) Fernandes RA, Casonatto J, Christofaro DGD, Cucato GG, Romanzini M, Ronque ERV. Aptidão cardiorrespiratória, excesso de peso e pressão arterial elevada em adolescentes. Cardiorespiratory fitness, surplus weight and high blood pressure in adolescents. Revista Brasileira de Medicina do Esporte 2010 December;16(6):404-7. Cross-sectional study
Ref ID: 4126

(1641) Fernandes RA, Christofaro DGD, Casonatto J, Codogno JS, Rodrigues E, Cardoso M, Kawaguti S, Zanesco A. Prevalência de dislipidemia em indivíduos fisicamente ativos durante a infância, adolescência e idade adulta. Prevalence of dyslipidemia in individuals physically active during childhood, adolescence and adult age. Arquivos brasileiros de cardiologia 2011 October;97(4):317-23. Cross-sectional study
Ref ID: 4128

(1642) Fernandes TL, Protta TR, Fregni F, Neto RB, Pedrinelli A, Camanho GL, Hernandez AJ. Isokinetic muscle strength and knee function associated with double femoral pin fixation and fixation with interference screw in anterior cruciate ligament reconstruction. Knee Surgery, Sports Traumatology, Arthroscopy 2012 February;20(2):275-80. Not an exercise intervention study
Ref ID: 2518

(1643) Fernandez-de-las-Penas C, Hernandez-Barrera V, Alonso-Blanco C, Palacios-Cena D, Carrasco-Garrido P, Jimenez-Sanchez S, Jimenez-Garcia R. Prevalence of neck and low back pain in community-dwelling adults in Spain: a population-based national study. Spine 2011 February 1;36(3):E213-E219. Study limited to adults
Ref ID: 2519

(1644) Fernandez AC, Cintra IP, Sawaya AL, Fisberg M, Silva AC. Respostas metabólicas e cardiorrespiratórias ao exercício máximo e submáximo em meninas eutróficas e com desnutriçäo pregressa. Revista da Associação Médica Brasileira (1992) 2000 December;46(4):312-9. Not a randomized controlled trial (RCT)
Ref ID: 4129

(1645) Fernandez AC, Mello MTd, Tufik S, Castro PMd, Fisberg M. Influência do treinamento aeróbio e anaeróbio na massa de gordura corporal de adolescentes obesos. Influence of the aerobic and anaerobic training on the body fat mass in obese adolescents. Revista Brasileira de Medicina do Esporte

2004 June;10(3):152-64. Diet & Exercise intervention
Ref ID: 4130

(1646) Fernandez F, I, Pascual dlP, Investigadores del Pg. [Predictive value of metabolic syndrome in pregnancy for the development of diabetes mellitus and factors of short-term vascular risk for mother and child after birth (gestaMET)]. [Spanish]. Atencion Primaria 2006 May 31;37(9):517-21. Study limited to adults
Ref ID: 1386

(1647) Fernández Ortega JA, González M, Martha Farfán F. Modelo teórico, agentes, estrategias, duración, escenarios para la implementación, grado escolar e indicadores de resultados utilizados en los programas de promoción de la actividad física en el contexto escolar. Theoretical model, agents, strategies, duration, spaces of implementation, school grades and performance indicators, used in programs to promote physical activity in school context. MedUNAB 2011 August;14(2):121-31. Review article
Ref ID: 4131

(1648) Fernhall B, Otterstetter M. Attenuated responses to sympathoexcitation in individuals with Down syndrome. Journal of Applied Physiology 2003 June;94(6):2158-65. Acute study
Ref ID: 370

(1649) Ferns SJ, Wehrmacher WH, Serratto M. Effects of obesity and gender on exercise capacity in urban children.[Erratum appears in Gend Med. 2011 Oct;8(5):342]. Gender Medicine 2011 August;8(4):224-30. Observational study
Ref ID: 2520

(1650) Ferrara A, Ehrlich SF. Strategies for diabetes prevention before and after pregnancy in women with GDM. [Review]. Current Diabetes Reviews 2011 March;7(2):75-83. Review article
Ref ID: 2521

(1651) Ferrara P, Del BF, Ianniello F, Franceschini A, Paolini PF, Massart F, Saggese G. Diet and physical activity "defeated" Tuberil(R) in treatment of childhood obesity. Minerva Endocrinologica 2013 June;38(2):181-5. Inappropriate Intervention
Ref ID: 5847

(1652) Ferrari GLd, Silva LJ, Ceschini FL, Oliveira LC, Douglas R, Matsudo VKR. Influência da maturação sexual na aptidão física de escolares do município de Ilhabela: Um estudo longitudinal. Revista Brasileira de Atividade Física e Saúde 2008;13(3). Not an exercise intervention study
Ref ID: 4132

(1653) Ferrari TK, Ferrari GLd, Silva Júnior JPd, Silva LJd, Oliveira LC, Matsudo VKR. Modificações da adiposidade em escolares de acordo com o estado nutricional: análise de 20 anos. Modifications of adiposity in school-age children according

to nutritional status: a 20-year analysis. Jornal de Pediatria 2012 June;88(3):239-45. Not an exercise intervention study
Ref ID: 4133

(1654) Ferraz ST, Frônio JdS, Neves LAT, Demarchi RS, Vargas ALdA, Ghetti FdF, Filgueiras MST. Programa de follow-up de recém-nascidos de alto risco: Relato da experiência de uma equipe interdisciplinar. Follow-up program of high risk neonates: Report of the experience of an interdisciplinary team. Revista de APS 2010 March;13(1). Follow-up Study
Ref ID: 4134

(1655) Ferreira AD, César CC, Malta DC, Souza Andrade ACd, Ramos CGC, Proietti FA, Bernal RTI, Caiaffa WT. Validade de estimativas obtidas por inquérito telefônico: Comparação entre VIGITEL 2008 e inquérito Saúde em Beagá. Validity of data collected by telephone survey: A comparison of VIGITEL 2008 and 'Saúde em Beagá' survey. Revista Brasileira de Eidemiologia 2011 September;14(supl.1):16-30. Survey or questionnaire
Ref ID: 4135

(1656) Ferreira AMd, Yonamine CY, Fujisawa DS, Lavado EL. A criança com paralisia cerebral: Características clínicas e fisioterapia. Temas Sobre Desenvolvimento 2008 August;16(93):113-7. Retrospective study
Ref ID: 4136

(1657) Ferreira AM, Bergamasco NHP. Análise comportamental de recém-nascidos pré-termos incluídos em um programa de estimulação tátil-cinestésica durante a internação hospitalar. Behavioral analysis of preterm neonates included in a tactile and kinesthetic stimulation program during hospitalization. Revista Brasileira de Fisioterapia 2010 April;14(2):141-8. Subjects less than 2 years old
Ref ID: 4137

(1658) Ferreira PL, Coelho VACC, Cesar MdC, Tolocka RE. Avaliação da saúde, fatores de risco e estado nutricional de crianças e adultos freqüentadores de um programa de natação. Revista Brasileira de Atividade Física e Saúde 2006 December;11(3). Cross-sectional study
Ref ID: 4138

(1659) Ferreira S, Marins JCB, Silva LCd, Lunz W, Pimentel GGdA, Migliorini EM. Determinação de perfil de repetições máximas no exercício de extensão de pernas e supino reto com diferentes percentuais de força. Revista da Educação Física 2006 December;17(2):149-59. Not a randomized controlled trial (RCT)
Ref ID: 4139

(1660) Ferretti G, Narici MV, Binzoni T, Gariod L, Lebas JF, Reutenauer H, Cerretelli P. Determinants of Peak Muscle Power - Effects of Age and Physical Conditioning. European Journal of Applied Physiology and Occupational

Physiology 1994;68(2):111-5. Not a randomized controlled trial (RCT)
Ref ID: 5265

(1661) Ferriani MdGC, Dechen S, Dias TS, Iossi MA. A percepção de saúde para adolescentes obesos. Revista brasileira de Enfermagem 2000 December;53(4):537-43. Not a randomized controlled trial (RCT)
Ref ID: 4140

(1662) Fessler DMT, Stieger S, Asaridou SS, Bahia U, Cravalho M, de Barros P, Delgado T, Fisher ML, Frederick D, Perez PG, Goetz C, Haley K, Jackson J, Kushnick G, Lew K, Pain E, Florindo PP, Pisor A, Sinaga E, Sinaga L, Smolich L, Sun DM, Voracek M. Testing a postulated case of intersexual selection in humans: The role of foot size in judgments of physical attractiveness and age. Evolution and Human Behavior 2012;33(2):147-64. Not a randomized controlled trial (RCT)
Ref ID: 5266

(1663) Festi D, Colecchia A, Sacco T, Bondi M, Roda E, Marchesini G. Hepatic steatosis in obese patients: clinical aspects and prognostic significance. [Review] [155 refs]. Obesity Reviews 2004 February;5(1):27-42. Review article
Ref ID: 1666

(1664) Fett CA, Fett WCR, Marchini JS, Ribeiro RPP. Estilo de vida e fatores de risco associados ao aumento da gordura corporal de mulheres. Lifestyle and risk factors associated to body fat increase in women. Ciência and Saúde Coletiva 2010 January;15(1):131-40. Study limited to adults
Ref ID: 4141

(1665) Fideleff HL, Boquete HR, Saskyn N, Pagano SM, Holland M. [Variability of growth hormone response to repeated stimulation tests in children of normal height]. [Spanish]. Medicina 1994;54(6):630-4. Not an exercise intervention study
Ref ID: 2218

(1666) Fidler JA, West R, Van Jaarsveld CH, Jarvis MJ, Wardle J. Does smoking in adolescence affect body mass index, waist or height? Findings from a longitudinal study. Addiction 2007 September;102(9):1493-501. Not an exercise intervention study
Ref ID: 1184

(1667) Fidler MC, Sanchez M, Raether B, Weissman NJ, Smith SR, Shanahan WR, Anderson CM, BLOSSOM Clinical Trial Group. A one-year randomized trial of lorcaserin for weight loss in obese and overweight adults: the BLOSSOM trial. Journal of Clinical Endocrinology and Metabolism 2011 October;96(10):3067-77. Drug intervention study
Ref ID: 2522

(1668) Figgitt DP, Plosker GL. Saquinavir Soft-Gel Capsule: An Updated Review of its Use in the Management of HIV Infection. Drugs 2000 August;60(2):481-516. Review article
Ref ID: 3585

(1669) Figueira Junior AJ, Matsudo VKR, Pereira MHN, Duarte CR. Tendência secular de variaveis antropométrica e de força muscular: visão durante uma década. Revista Brasileira de Ciência e Movimento 1988 April;2(2):17-23. Cross-sectional study
Ref ID: 4142

(1670) Figueroa-Colon R, Franklin FA, Lee JY, von Almen TK, Suskind RM. Feasibility of a clinic-based hypocaloric dietary intervention implemented in a school setting for obese children. Obesity Research 1996 September;4(5):419-29. No exercise only group
Ref ID: 427

(1671) Filaire E, Lac G. Nutritional status and body composition of juvenile elite female gymnasts. Journal of Sports Medicine and Physical Fitness 2002 March;42(1):65-70. Not a randomized controlled trial (RCT)
Ref ID: 1850

(1672) Filardo RD, Rodriguez-Añez CR, Pires Neto CS. Antropometria e composição corporal de jovens do sexo feminino entre 13 e 17 anos de idade. Revista Brasileira de Cineantropometria e Desempenho Humano 2000;2(1). Not a randomized controlled trial (RCT)
Ref ID: 4143

(1673) Filippin NT, Barbosa VLP, Sacco ICN, Lobo da Costa PH. Efeitos da obesidade na distribuição de pressão plantar em crianças. Revista Brasileira de Fisioterapia 2007 December;11(6):495-501. Not a randomized controlled trial (RCT)
Ref ID: 583

(1674) Filippin NT, Murazo CF, Rigotti M, Bonamigo ECB. A influência do treinamento da marcha com suporte parcial de peso corporal na diplegia espástica. Fisioterapia Brasileira 2007 June;8(3):214-7. Case-Control / Case Study
Ref ID: 4144

(1675) Filozof CM, Murua C, Sanchez MP, Brailovsky C, Perman M, Gonzalez CD, Ravussin E. Low plasma leptin concentration and low rates of fat oxidation in weight-stable post-obese subjects. Obesity Research 2000;8(3):205-10. Study limited to adults
Ref ID: 5267

(1676) Finch M, Wolfenden L, Morgan PJ, Freund M, Wyse R, Wiggers J. A cluster randomised trial to evaluate a physical activity intervention among 3-5 year old children attending long day care services: study protocol. BMC Public Health

2010;10:534. Description of study from review or magazine or etc. (not the actual study)
Ref ID: 4775

(1677) Finch M, Wolfenden L, Falkiner M, Edenden D, Pond N, Hardy LL, Milat AJ, Wiggers J. Impact of a population based intervention to increase the adoption of multiple physical activity practices in centre based childcare services: a quasi experimental, effectiveness study. International Journal of Behavioral Nutrition and Physical Activity 2012;9:101. Inappropriate Study Design
Ref ID: 5848

(1678) Fink B, Thanzami V, Seydel H, Manning JT. Digit ratio and hand-grip strength in German and Mizos men: Cross-cultural evidence for an organizing effect of prenatal testosterone on strength. American Journal of Human Biology 2006;18(6):776-82. Study limited to adults
Ref ID: 5268

(1679) Fink B, Neave N, Seydel H. Male facial appearance signals physical strength to women. American Journal of Human Biology 2007 January;19(1):82-7. Study limited to adults
Ref ID: 1295

(1680) Fink B, Seydel H, Manning JT, Kappeler PM. A preliminary investigation of the associations between digit ratio and women's perception of men's dance. Personality and Individual Differences 2007;42(2):381-90. Study limited to adults
Ref ID: 5269

(1681) Finlayson G, Bryant E, Blundell JE, King NA. Acute compensatory eating following exercise is associated with implicit hedonic wanting for food. Physiology and Behavior 2009 April 20;97(1):62-7. Acute study
Ref ID: 755

(1682) Finne E, Reinehr T, Schaefer A, Winkel K, Kolip P. Overweight children and adolescents--is there a subjective need for treatment? International Journal of Public Health 2009;54(2):112-6. Description of study from review or magazine or etc. (not the actual study)
Ref ID: 782

(1683) Finne E, Reinehr T, Schaefer A, Winkel K, Kolip P. Changes in self-reported and parent-reported health-related quality of life in overweight children and adolescents participating in an outpatient training: findings from a 12-month follow-up study. Health and Quality of Life Outcomes 2013;11:1. Inappropriate Study Design
Ref ID: 5849

(1684) Finni T, Saakslahti A, Laukkanen A, Pesola A, Sipila S. A family based tailored counselling to increase non-exercise physical activity in adults with a sedentary

job and physical activity in their young children: design and methods of a year-long randomized controlled trial. BMC Public Health 2011;11:944. Description of study from review or magazine or etc. (not the actual study)
Ref ID: 2523

(1685) Finsterer J. Mitochondriopathies. European Journal of Neurology 2004 March;11(3):163-86. Review article
Ref ID: 3586

(1686) Fiorillo L. Therapy of pediatric genital diseases. Dermatologic Therapy 2004 March;17(1):117-28. Not an exercise intervention study
Ref ID: 3587

(1687) Firrincieli V, Keller A, Ehrensberger R, Platts-Mills J, Shufflebarger C, Geldmaker B, Platts-Mills T. Decreased physical activity among Head Start children with a history of wheezing: use of an accelerometer to measure activity. Pediatric Pulmonology 2005 July;40(1):57-63. Not an exercise intervention study
Ref ID: 1509

(1688) Fisch GS, Carey J, Youngblom J, Simensen R, Battaglia A. Cognitive-behavioural and autistic features of children with subtelomeric deletions. Journal of Intellectual Disability Research 2008 October;52(10):813. Not an exercise intervention study
Ref ID: 3813

(1689) Fischer R, Shneider B. Treatment of non-alcoholic fatty liver disease in children: swim at your own risk. Pediatric Diabetes 2009 February;10(1):1-4. Editorial or letter or comment
Ref ID: 806

(1690) Fisher A, Hill C, Webber L, Purslow L, Wardle J. MVPA is associated with lower weight gain in 8-10 year old children: a prospective study with 1 year follow-up. PLoS ONE [Electronic Resource] 2011;6(4):e18576. Prospective Study
Ref ID: 2524

(1691) Fisher EB, Brownson CA, O'Toole ML, Sherry G, Anwuri VV, Glasgow RE. Ecological Approaches to Self-Management: The Case of Diabetes. American Journal of Public Health 2005 September;95(9):1523-35. Review article
Ref ID: 3814

(1692) Fitch KD, Morton AR, Blanksby BA. Effects of swimming training on children with asthma. Archives of Disease in Childhood 1976 March;51(3):190-4. Not a randomized controlled trial (RCT)
Ref ID: 2390

(1693) Fitch RC, Harnack LJ, Neumark-Sztainer DR, Story MT, French SA, Oakes JM, Rydell SA. Providing calorie information on fast-food restaurant menu boards:

consumer views. American Journal of Health Promotion 2009 November;24(2):129-32. Not an exercise intervention study
Ref ID: 641

(1694) Fitzgerald MD, Tanaka H, Tran ZV, Seals DR. Age-related declines in maximal aerobic capacity in regularly exercising vs. sedentary women: a meta-analysis. Journal of Applied Physiology 1997 July;83(1):160-5. Review article
Ref ID: 2132

(1695) Fitzgerald PJ. Is elevated noradrenaline an aetiological factor in a number of diseases? Autonomic and Autacoid Pharmacology 2009 October;29(4):143-56. Not an exercise intervention study
Ref ID: 3588

(1696) Fitzgibbon ML, Prewitt TE, Blackman LR, Simon P, Luke A, Keys LC, Avellone ME, Singh V. Quantitative assessment of recruitment efforts for prevention trials in two diverse black populations. Preventive Medicine 1998 November;27(6):838-45. Description of study from review or magazine or etc. (not the actual study)
Ref ID: 2069

(1697) Fitzgibbon ML, Stolley MR, Dyer AR, VanHorn L, KauferChristoffel K. A community-based obesity prevention program for minority children: rationale and study design for Hip-Hop to Health Jr. Preventive Medicine 2002 February;34(2):289-97. Description versus conduct of study, No exercise only group
Ref ID: 385

(1698) Fitzgibbon ML, Stolley MR, Schiffer L, Van HL, KauferChristoffel K, Dyer A. Two-year follow-up results for Hip-Hop to Health Jr.: a randomized controlled trial for overweight prevention in preschool minority children. Journal of Pediatrics 2005;146:618-25. Follow-up Study
Ref ID: 4776

(1699) Fitzgibbon ML, Stolley MR, Schiffer L, Van HL, KauferChristoffel K, Dyer A. Hip-Hop to Health Jr. for Latino preschool children. Obesity (Silver Spring) 2006 September;14(9):1616-25. No exercise only group
Ref ID: 266

(1700) Fitzgibbon ML, Stolley MR, Schiffer LA, Braunschweig CL, Gomez SL, Van HL, Dyer AR. Hip-Hop to Health Jr. Obesity Prevention Effectiveness Trial: postintervention results. Obesity 2011 May;19(5):994-1003. No exercise only group
Ref ID: 2525

(1701) Fitzgibbon ML, Stolley MR, Schiffer L, Kong A, Braunschweig CL, Gomez-Perez SL, Odoms-Young A, Van HL, Christoffel KK, Dyer AR. Family-based hip-hop to health: outcome results. Obesity (Silver Spring) 2013

February;21(2):274-83. Inappropriate Population
Ref ID: 5850

(1702) Flack JM, Gardin JM, Yunis C, Liu K. Static and pulsatile blood pressure correlates of left ventricular structure and function in black and white young adults: the CARDIA study. American Heart Journal 1999 November;138(5:Pt 1):t-64. Study limited to adults
Ref ID: 2063

(1703) Flakoll PJ, Judy T, Flinn K, Carr C, Flinn S. Postexercise protein supplementation improves health and muscle soreness during basic military training in Marine recruits. Journal of Applied Physiology 2004 March;96(3):951-6. Diet Intervention or Supplement Study
Ref ID: 1668

(1704) Fleischman A, Rhodes ET. Management of obesity, insulin resistance and type 2 diabetes in children: Consensus and controversy. Diabetes, Metabolic Syndrome and Obesity: Targets and Therapy 2009;2:185-202. Review article
Ref ID: 3224

(1705) Fleischman A, Makimura H, Stanley TL, McCarthy MA, Kron M, Sun N, Chuzi S, Hrovat MI, Systrom DM, Grinspoon SK. Skeletal muscle phosphocreatine recovery after submaximal exercise in children and young and middle-aged adults. The Journal of Clinical Endocrinology and Metabolism 2010;95:E69-E74. Cross-sectional study
Ref ID: 4777

(1706) Fletcher A, Cooper JR, Helms P, Northington L, Winters K. Stemming the tide of childhood obesity in an underserved urban African American population: a pilot study. ABNF Journal 2009;20(2):44-8. Lifestyle Intervention
Ref ID: 741

(1707) Fletcher JM, Green JC, Neidell MJ. Long term effects of childhood asthma on adult health. Journal of Health Economics 2010;29(3):377-87. Review article
Ref ID: 5270

(1708) Fletcher K, Stone E, Mohamad MW, Faulder GC, Faulder RM, Jones K, Morgan D, Wegerdt J, Kelly M, Chick J. A breath test to assess compliance with disulfiram. Addiction 2006 December;101(12):1705-10. Not an exercise intervention study
Ref ID: 3589

(1709) Flodmark CE, Ohlsson T, Ryden O, Sveger T. Prevention of progression to severe obesity in a group of obese schoolchildren treated with family therapy. Pediatrics 1993 May;91(5):880-4. Diet Intervention Study
Ref ID: 2270

(1710) Florence JM, Pandya S, King WM, Robison JD, Baty J, Miller JP, Schierbecker J, Signore LC. Intrarater reliability of manual muscle test (Medical Research Council scale) grades in Duchenne's muscular dystrophy. Physical Therapy 1992;72:115-22. Not an exercise intervention study
Ref ID: 4778

(1711) Florentin M, Liberopoulos EN, Mikhailidis DP, Elisaf MS. Sitagliptin in clinical practice: A new approach in the treatment of type 2 diabetes. Expert Opinion on Pharmacotherapy 2008;9(10):1705-20. Review article
Ref ID: 3226

(1712) Florentin M, Liberopoulos EN, Elisaf MS. Sibutramine-associated adverse effects: A practical guide for its safe use. Obesity Reviews 2008;9(4):378-87. Diet Intervention Study
Ref ID: 3225

(1713) Flores-Peña Y, Trejo-Ortiz PM, Gallegos-Cabriales EC, Cerda-Flores RM. Validez de dos pruebas para evaluar la percepción materna del peso del hijo. Validity of two tests to evaluate maternal perception of child's weight. Salud Pública de México 2009 December;51(6):489-95. Not an exercise intervention study
Ref ID: 4145

(1714) Flores C, Eyzaguirre C, García F, Vega N, Cosentino A, Silva A, Román R, Cavada C, Tapia J, García B. Evaluación de un programa integral de tratamiento de obesidad y sobrepeso en niños escolares y adolescentes en Santiago de Chile (1999-2006). Revista Chilena de Edocrinología y Diabetes 2009 January;2(1):5-12. Multiple interventions
Ref ID: 4146

(1715) Flores R. Dance for health: improving fitness in African American and Hispanic adolescents. Public Health Reports 1995 March;110(2):189-93. Not All Participants were Overweight and/or Obese
Ref ID: 2203

(1716) Florin TA, Fryer GE, Miyoshi T, Weitzman M, Mertens AC, Hudson MM, Sklar CA, Emmons K, Hinkle A, Whitton J, Stovall M, Robison LL, Oeffinger KC. Physical inactivity in adult survivors of childhood acute lymphoblastic leukemia: a report from the childhood cancer survivor study. Cancer Epidemiology, Biomarkers and Prevention 2007 July;16(7):1356-63. Not an exercise intervention study
Ref ID: 1200

(1717) Floyd MF, Spengler JO, Maddock JE, Gobster PH, Suau LJ. Park-based physical activity in diverse communities of two US cities - An observational study. American Journal of Preventive Medicine 2008;34(4):299-305.

Observational study
Ref ID: 5271

(1718) Flynn MG, Mackinnon L, Gedge V, Fahlman M, Brickman T. Influence of iron status and iron supplements on natural killer cell activity in trained women runners. International Journal of Sports Medicine 2003 April;24(3):217-22. Diet Intervention or Supplement Study
Ref ID: 1745

(1719) Foger M, Bart G, Rathner G, Jager B, Fischer H, Zollner-Neussl D. [Physical activity, nutritional counseling and psychological guidance in treatment of obese children. A controlled follow-up study over six months]. Monatsschrift Kinderheilkunde 1993 June;141(6):491-7. No exercise only group
Ref ID: 435

(1720) Fogt DL, Brosch LC, Dacey DC, Kalns JE, Ketchum NS, Rohrbeck P, Venuto MM, Tchandja JB, Bunning ML. Hydration status of Air Force military basic trainees after implementation of the back-mounted hydration system. Military Medicine 2009 August;174(8):821-7. Study limited to adults
Ref ID: 678

(1721) Foley L, Jiang Y, Ni MC, Jull A, Prapavessis H, Rodgers A, Maddison R. The effect of active video games by ethnicity, sex and fitness: subgroup analysis from a randomised controlled trial. International Journal of Behavioral Nutrition and Physical Activity 2014;11(1):46. Inappropriate Population
Ref ID: 5851

(1722) Follansbee JK, Janicke DM, Sallinen BJ. The influence of a behavioral weight management program on disordered eating attitudes and behaviors in children with overweight. Journal of the American Dietetic Association 2010;110:1653-9. Behavior Modification Intervention
Ref ID: 4779

(1723) Folsom AR, Qamhieh HT, Flack JM, Hilner JE, Liu K, Howard BV, Tracy RP. Plasma fibrinogen: levels and correlates in young adults. The Coronary Artery Risk Development in Young Adults (CARDIA) Study. American Journal of Epidemiology 1993 December 15;138(12):1023-36. Cross-sectional study
Ref ID: 2253

(1724) Folsom AR, Jacobs DR, Jr., Wagenknecht LE, Winkhart SP, Yunis C, Hilner JE, Savage PJ, Smith DE, Flack JM. Increase in fasting insulin and glucose over seven years with increasing weight and inactivity of young adults. The CARDIA Study. Coronary Artery Risk Development in Young Adults. American Journal of Epidemiology 1996 August 1;144(3):235-46. Study limited to adults
Ref ID: 2177

(1725) Folta SC, Goldberg JP, Economos C, Bell R, Landers S, Hyatt R. Assessing the use of school public address systems to deliver nutrition messages to

children: Shape up Somerville--audio adventures. Journal of School Health 2006;76:459-64. Diet Intervention Study
Ref ID: 4780

(1726) Folta SC, Kuder JF, Goldberg JP, Hyatt RR, Must A, Naumova EN, Nelson ME, Economos CD. Changes in diet and physical activity resulting from the Shape Up Somerville community intervention. BMC Pediatrics 2013;13:157. Inappropriate Study Design
Ref ID: 5852

(1727) Fong DTP, Hong YL, Li JX. Cushioning and lateral stability functions of cloth sport shoes. Sports Biomechanics 2007;6(3):407-17. Not an exercise intervention study
Ref ID: 5272

(1728) Fonseca-Toledo C, Roquetti P, Fernandes-Filho J. Perfil antropométrico de atletas brasileiros de voleibol infanto juvenil em diferentes níveis de qualificação esportiva. Anthropometrical profile of Brazilian junior volleyball players for different sports requirement levels. Perfil antropométrico de jugadores brasileños infantiles y juveniles de voleibol, en diferentes niveles de calificación deportiva. Revista de Salud Pública 2010 December;12(6):915-28. Cross-sectional study
Ref ID: 4147

(1729) Fonseca H, Palmeira AL, Martins SC, Falcato L, Quaresma A. Managing paediatric obesity: a multidisciplinary intervention including peers in the therapeutic process. BMC Pediatrics 2014;14:89. Inappropriate Study Design
Ref ID: 5853

(1730) Fonseca HARd, Dellagrana RA, Lima LRAd, Kaminagakura EI. Aptidão física relacionada à saúde de escolares de escola pública de tempo integral. Acta Scientiarum. Health Sciences 2010 December;32(2). Cross-sectional study
Ref ID: 4148

(1731) Fonseca LS, Kirsten VR. Fatores de risco para a elevação da pressão arterial em adolescentes. Risk factors for high blood pressure in adolescents. Medicina (Ribeirão Preto) 2010 December;43(4):400-7. Cross-sectional study
Ref ID: 4149

(1732) Fontana K, Aldrovani M, de Paoli F, Oliveira HCF, Vidal BD, da Cruz-Hofling MA. Hepatocyte nuclear phenotype: the cross-talk between anabolic androgenic steroids and exercise in transgenic mice. Histology and Histopathology 2008;23(11):1367-77. Animal study
Ref ID: 5273

(1733) Fontoura ASd, Schneider P, Meyer F. O efeito do destreinamento de força muscular em meninos pré-púberes. Effect of the muscular strength detraining in prepubertal boys. Revista Brasileira de Medicina do Esporte 2004

August;10(4):281-8. Not a randomized controlled trial (RCT)
Ref ID: 4150

(1734) Fontvieille AM, Harper IT, Ferraro RT, Spraul M, Ravussin E. Daily Energy-Expenditure by 5-Year-Old Children, Measured by Doubly Labeled Water. Journal of Pediatrics 1993;123(2):200-7. Cross-sectional study
Ref ID: 5274

(1735) Foricher JM, Ville N, Gratas-Delamarche A, Delamarche P. Effects of submaximal intensity cycle ergometry for one hour on substrate utilisation in trained prepubertal boys versus trained adults. Journal of Sports Medicine and Physical Fitness 2003 March;43(1):36-43. Acute study
Ref ID: 1766

(1736) Forman EM, Butryn ML, Juarascio AS, Bradley LE, Lowe MR, Herbert JD, Shaw JA. The mind your health project: a randomized controlled trial of an innovative behavioral treatment for obesity. Obesity (Silver Spring) 2013 June;21(6):1119-26. Inappropriate Intervention
Ref ID: 5854

(1737) Formiga CKMR, Cezar MEN, Linhares MBM. Avaliação longitudinal do desenvolvimento motor e da habilidade de sentarem crianças nascidas prematuras. Longitudinal assessment of motor development and sitting skill in preterm infants. Fisioterapia e Pesquisa 2010 June;17(2):102-7. Subjects less than 2 years old
Ref ID: 4151

(1738) Forsander G, Malmodin B, Eklund C, Persson B. Relationship between dietary intake in children with diabetes mellitus type I, their management at diagnosis, social factors, anthropometry and glycaemic control. Scandinavian Journal of Nutrition 2003;47:75-84. Diet Intervention Study
Ref ID: 4781

(1739) Forssberg H, Kinoshita H, Eliasson AC, Johansson RS, Westling G, Gordon AM. Development of Human Precision Grip .2. Anticipatory Control of Isometric Forces Targeted for Objects Weight. Experimental Brain Research 1992;90(2):393-8. Not a randomized controlled trial (RCT)
Ref ID: 644

(1740) Forssberg H, Eliasson AC, Kinoshita H, Westling G, Johansson RS. Development of Human Precision Grip .4. Tactile Adaptation of Isometric Finger Forces to the Frictional Condition. Experimental Brain Research 1995;104(2):323-30. Not a randomized controlled trial (RCT)
Ref ID: 5275

(1741) Fortes LdS, Miranda VPN, Amaral ACS, Ferreira MEC. Insatisfação corporal de adolescentes atletas e não atletas. Body dissatisfaction of teen athletes and non-athletes. Jornal Brasileiro de Psiquiatria 2011;60(4):309-14. Cross-

sectional study
Ref ID: 4152

(1742) Fortes MB, Diment BC, Di FU, Gunn AE, Kendall JL, Esmaeelpour M, Walsh NP. Tear fluid osmolarity as a potential marker of hydration status. Medicine and Science in Sports and Exercise 2011 August;43(8):1590-7. Acute study
Ref ID: 2526

(1743) Fortier MS, Hogg W, O'Sullivan TL, Blanchard C, Reid RD, Sigal RJ, Boulay P, Doucet E, Sweet S, Bisson E, Beaulac J. The physical activity counselling (PAC) randomized controlled trial: rationale, methods, and interventions. Applied Physiology, Nutrition, and Metabolism = Physiologie Appliquee, Nutrition et Metabolisme 2007 December;32(6):1170-85. Description of study from review or magazine or etc. (not the actual study)
Ref ID: 1130

(1744) Fortier MS, Hogg W, O'Sullivan TL, Blanchard C, Sigal RJ, Reid RD, Boulay P, Doucet E, Bisson E, Beaulac J, Culver D. Impact of integrating a physical activity counsellor into the primary health care team: physical activity and health outcomes of the Physical Activity Counselling randomized controlled trial. Applied Physiology, Nutrition, and Metabolism = Physiologie Appliquee, Nutrition et Metabolisme 2011 August;36(4):503-14. Study limited to adults
Ref ID: 2527

(1745) Foster GD, Wyatt HR, Hill JO, Makris AP, Rosenbaum DL, Brill C, Stein RI, Mohammed BS, Miller B, Rader DJ, Zemel B, Wadden TA, Tenhave T, Newcomb CW, Klein S. Weight and metabolic outcomes after 2 years on a low-carbohydrate versus low-fat diet: a randomized trial. Annals of Internal Medicine 2010 August 3;153(3):147-57. Study limited to adults
Ref ID: 27

(1746) Foster KE, Behrens TK, Jager AL, Dzewaltowski DA. Effect of elimination games on physical activity and psychosocial responses in children. Journal of Physical Activity and Health 2010;7:475-83. Not a randomized controlled trial (RCT)
Ref ID: 4782

(1747) Fotiadou E, Giagazoglou P, Kokaridas D, Angelopoulou N, Tsimaras V, Tsorbatzoudis C. Effect of rhythmic gymnastics on the dynamic balance of children with deafness. European Journal of Special Needs Education 2002;17:301-9. Not All Participants were Overweight and/or Obese
Ref ID: 4783

(1748) Fountaine RJ, Taylor AE, Mancuso JP, Greenway FL, Byerley LO, Smith SR, Most MM, Fryburg DA. Increased food intake and energy expenditure following administration of olanzapine to healthy men. Obesity 2010 August;18(8):1646-

51. Study limited to adults
Ref ID: 482

(1749) Foxcroft KF, Rowlands IJ, Byrne NM, McIntyre HD, Callaway LK, BAMBINO group. Exercise in obese pregnant women: the role of social factors, lifestyle and pregnancy symptoms. BMC Pregnancy and Childbirth 2011;11:4. Study limited to adults
Ref ID: 2528

(1750) Föger M, Bart G, Rathner G, Jäger B, Fischer H, Zollner ND. [Physical activity, nutritional counseling and psychological guidance in treatment of obese children. A controlled follow-up study over six months]. Monatsschrift Kinderheilkunde: Organ der Deutschen Gesellschaft für Kinderheilkunde 1993;141:491-7. Diet & Exercise intervention
Ref ID: 4784

(1751) Fragala MS, Kraemer WJ, Mastro AM, Denegar CR, Volek JS, Hakkinen K, Anderson JM, Lee EC, Maresh CM. Leukocyte beta2-adrenergic receptor expression in response to resistance exercise. Medicine and Science in Sports and Exercise 2011 August;43(8):1422-32. Study limited to adults
Ref ID: 2529

(1752) Frainer DES, Oliveira FRd, Pazin J. Influência da maturação sexual, idade cronológica e índices de crescimento no limiar de lactato e no desempenho da corrida de 20 minutos. Revista Brasileira de Medicina do Esporte 2006 June;12(3):139-44. No control group (NC)
Ref ID: 4153

(1753) Frainer DES, Silva MdCMd, Santana MLPd, Santos NSd, Oliveira LPMd, Barreto ML, Assis AM. Prevalência e fatores associados ao excesso de peso em adolescentes de Salvador, Bahia, Brasil. Prevalence and associated factors of surplus weight in adolescents from Salvador, Bahia, Brazil. Revista Brasileira de Medicina do Esporte 2011 April;17(2):102-6. Cross-sectional study
Ref ID: 4154

(1754) Francescato MP, Puntel I. Does a pre-exercise carbohydrate feeding improve a 20-km cross-country ski performance? Journal of Sports Medicine and Physical Fitness 2006 June;46(2):248-56. Acute study
Ref ID: 1376

(1755) Francis DJ, Fletcher JM, Stuebing KK, Lyon GR, Shaywitz BA, Shaywitz SE. Psychometric approaches to the identification of ld: iq and achievement scores are not sufficient. Journal of Learning Disabilities 2005 March;38(2):98-108. Not an exercise intervention study
Ref ID: 3815

(1756) Francis M, Nichols SS, Dalrymple N. The effects of a school-based intervention programme on dietary intakes and physical activity among primary-school

children in Trinidad and Tobago. Public Health Nutrition 2010 May;13(5):738-47. No exercise only group
Ref ID: 59

(1757) Francis PW, Krastins IR, Levison H. Oral and inhaled salbutamol in the prevention of exercise-induced bronchospasm. Pediatrics 1980 July;66(1):103-8. Drug intervention study
Ref ID: 2379

(1758) Franco CD, Vieira ZE. 1,001 subclavian perivascular brachial plexus blocks: success with a nerve stimulator. Regional Anesthesia and Pain Medicine 2000 January;25(1):41-6. Not an exercise intervention study
Ref ID: 1991

(1759) Frank I, Briggs R, Spengler CM. Respiratory muscles, exercise performance, and health in overweight and obese subjects. Medicine and Science in Sports and Exercise 2011 April;43(4):714-27. Study limited to adults
Ref ID: 2530

(1760) Frank LM, Enlow T, Holmes GL, Manasco P, Concannon S, Chen C, Womble G, Casale EJ. Lamictal (lamotrigine) monotherapy for typical absence seizures in children. Epilepsia 1999;40:973-9. Drug intervention study
Ref ID: 4785

(1761) Franks ME, Macpherson GR, Figg WD. Thalidomide. Lancet 2004 May 29;363(9423):1802-11. Review article
Ref ID: 3590

(1762) Franks PW, Jablonski KA, Delahanty LM, McAteer JB, Kahn SE, Knowler WC, Florez JC. Assessing gene-treatment interactions at the FTO and INSIG2 loci on obesity-related traits in the Diabetes Prevention Program. Diabetologia 2008;51(12):2214-23. Multiple interventions
Ref ID: 5276

(1763) Frazier B, Hsiao CW, Deuster P, Poth M. African Americans and Caucasian Americans: differences in glucocorticoid-induced insulin resistance. Hormone and Metabolic Research 2010 November;42(12):887-91. Not an exercise intervention study
Ref ID: 2531

(1764) Frederick MJ. Birth Weight Predicts Scores on the ADHD Self-Report Scale and Attitudes Towards Casual Sex in College Men: A Short-Term Life History Strategy? Evolutionary Psychology 2012;10(2):342-51. Survey or questionnaire
Ref ID: 5277

(1765) Fredriksen B, Nafstad O, Lium BM, Marka CH, Dahl E, Choinski JU. Artificial light programmes in entire male pig production -- effects on androstenone, skatole and animal welfare. Acta Veterinaria Scandinavica 2006 January

2;48:3-2. Animal study
Ref ID: 3591

(1766) Freed DL, Banks AJ, Longson D, Burley DM. Anabolic steroids in athelics: crossover double-blind trial on weightlifters. British Medical Journal 1975 May 31;2(5969):471-3. Drug intervention study
Ref ID: 2532

(1767) Freeland-Graves J, Nitzke S, Denny S, Askew EW, McMahon KE, Owen AL, Evers W, Watkins S, Heins J, Kubena K, Duffy V, Geiger C. Position of the American Dietetic Association: Total diet approach to communicating food and nutrition information. Journal of the American Dietetic Association 2002;102(1):100-8. Review article
Ref ID: 5278

(1768) Freeman III BB, Daw NC, Geyer JR, Furman WL, Stewart CF. Evaluation of Gefitinib for Treatment of Refractory Solid Tumors and Central Nervous System Malignancies in Pediatric Patients. Cancer Investigation 2006 April;24(3):310-7. Drug intervention study
Ref ID: 3592

(1769) Freeman D, McManus S, Brugha T, Meltzer H, Jenkins R, Bebbington P. Concomitants of paranoia in the general population. Psychological Medicine 2011;41(5):923-36. Study limited to adults
Ref ID: 5279

(1770) Freeman JM, Vining EPG, Cost S, Singhi P. Does carnitine administration improve the symptoms attributed to anticonvulsant medications?: A double-blinded, crossover study. Pediatrics 1994;93(6 I):893-5. Drug intervention study
Ref ID: 3227

(1771) Freemark M, Bursey D. The effects of metformin on body mass index and glucose tolerance in obese adolescents with fasting hyperinsulinemia and a family history of type 2 diabetes. Pediatrics 2001 April;107(4):E55. Drug intervention study
Ref ID: 1917

(1772) Freemark M. Pharmacotherapy of childhood obesity: An evidence-based, conceptual approach. Diabetes Care 2007;30(2):395-402. Review article
Ref ID: 3228

(1773) Freiling D, Lobenhoffer P. [The surgical treatment of chronic extension deficits of the knee]. [German]. Operative Orthopadie und Traumatologie 2009 December;21(6):545-56. Not an exercise intervention study
Ref ID: 618

(1774) Freitas Júnior IF. Sobrepeso e obesidade em crianças e adolescentes brasileiros. Salusvita 2007;26(2):125-52. Review article
Ref ID: 4155

(1775) Freitas RWJFd, Silva ARVd, Araújo MFMd, Marinho NBP, Damasceno MMC, Oliveira MRd. Prática de atividade física por adolescentes de Fortaleza, CE, Brasil. Práctica de actividad física por adolescentes de Fortaleza, CE, Brasil. Physical activity practice by adolescents from Fortaleza, CE, Brazil. Revista brasileira de Enfermagem 2010 June;63(3):410-5. Cross-sectional study
Ref ID: 4156

(1776) Fremeaux AE, Mallam KM, Metcalf BS, Hosking J, Voss LD, Wilkin TJ. The impact of school-time activity on total physical activity: the activitystat hypothesis (EarlyBird 46). International Journal of Obesity 2011 October;35(10):1277-83. Observational study
Ref ID: 2533

(1777) French SA, Neumark-Sztainer D, Story M, Jeffery RW. Reducing barriers to participation in weight-loss programs in low-income women. Journal of the American Dietetic Association 1998 February;98(2):198-200. Study limited to adults
Ref ID: 2107

(1778) French SA, Story M, Fulkerson JA, Himes JH, Hannan P, Neumark SD, Ensrud K. Increasing weight-bearing physical activity and calcium-rich foods to promote bone mass gains among 9-11 years old girls: outcomes of the Cal-Girls study. International Journal of Behavioral Nutrition and Physical Activity 2005;2:11. Behavior Modification Intervention
Ref ID: 4786

(1779) French SA, Gerlach AF, Mitchell NR, Hannan PJ, Welsh EM. Household obesity prevention: Take Action--a group-randomized trial. Obesity 2011 October;19(10):2082-8. Not All Participants were Overweight and/or Obese
Ref ID: 2534

(1780) French SA, Mitchell NR, Hannan PJ. Decrease in television viewing predicts lower body mass index at 1-year follow-up in adolescents, but not adults. Journal of Nutrition Education and Behavior 2012 September;44(5):415-22. Inappropriate Study Design
Ref ID: 5855

(1781) Frenn M, Malin S, Bansal NK. Stage-based interventions for low-fat diet with middle school students. Journal of Pediatric Nursing 2003 February;18(1):36-45. No exercise only group
Ref ID: 369

(1782) Frenn M, Malin S, Brown RL, Greer Y, Fox J, Greer J, Smyczek S. Changing the tide: an Internet/video exercise and low-fat diet intervention with middle-

school students. Applied Nursing Research 2005 February;18(1):13-21. Not a randomized controlled trial (RCT)
Ref ID: 1529

(1783) Frenn M, Pruszynski JE, Felzer H, Zhang J. Authoritative feeding behaviors to reduce child BMI through online interventions. Journal for Specialists in Pediatric Nursing 2013 January;18(1):65-77. Inappropriate Study Design
Ref ID: 5856

(1784) Frery N, Maury-Brachet R, Maillot E, Deheeger M, de Merona B, Boudou A. Gold-mining activities and mercury contamination of native Amerindian communities in French Guiana: Key role of fish in dietary uptake. Environmental Health Perspectives 2001;109(5):449-56. Diet Intervention Study
Ref ID: 5280

(1785) Frey DJ, Ortega JD, Wiseman C, Farley CT, Wright KP. Influence of Zolpidem and Sleep Inertia on Balance and Cognition During Nighttime Awakening: A Randomized Placebo-Controlled Trial. Journal of the American Geriatrics Society 2011 January;59(1):73-81. Drug intervention study
Ref ID: 3593

(1786) Fridman C, Varela MC, Kok F, Diament A, Koiffmann CP. Paternal UPD15: Further genetic and clinical studies in four Angelman syndrome patients. American Journal of Medical Genetics 2000;92(5):322-7. Not a randomized controlled trial (RCT)
Ref ID: 5281

(1787) Fridman C, Varela MC, Valente K, Marques-Dias MJ, Koiffmann CP. Phenotypic and behavioral variability within Angelman Syndrome group with UPD. Genetics and Molecular Biology 2002 June;25(2):127-30. Not a randomized controlled trial (RCT)
Ref ID: 4157

(1788) Friebe D, Neef M, Kratzsch J, Erbs S, Dittrich K, Garten A, Petzold-Quinque S, Bluher S, Reinehr T, Stumvoll M, Bluher M, Kiess W, Korner A. Leucocytes are a major source of circulating nicotinamide phosphoribosyltransferase (NAMPT)/pre-B cell colony (PBEF)/visfatin linking obesity and inflammation in humans. Diabetologia 2011 May;54(5):1200-11. Cohort Study
Ref ID: 2535

(1789) Friedl KE. Biomedical Research on Health and Performance of Military Women: Accomplishments of the Defense Women’s Health Research Program (DWHRP). Journal of Women's Health (15409996) 2005 November;14(9):764-802. Review article
Ref ID: 3594

(1790) Friedlander AL, Casazza GA, Horning MA, Huie MJ, Brooks GA. Training-induced alterations of glucose flux in men. Journal of Applied Physiology 1997 April;82(4):1360-9. No control group (NC)
Ref ID: 2145

(1791) Friedlander AL, Casazza GA, Horning MA, Huie MJ, Piacentini MF, Trimmer JK, Brooks GA. Training-induced alterations of carbohydrate metabolism in women: women respond differently from men. Journal of Applied Physiology 1998 September;85(3):1175-86. Study limited to adults
Ref ID: 2081

(1792) Friedlander AL, Casazza GA, Horning MA, Usaj A, Brooks GA. Endurance training increases fatty acid turnover, but not fat oxidation, in young men. Journal of Applied Physiology 1999 June;86(6):2097-105. Study limited to adults
Ref ID: 2019

(1793) Friedlander EA, Pallentino J, Miller SK, VanBeuge SS. The evolution of proton pump inhibitors for the treatment of gastroesophageal reflux disease. Journal of the American Academy of Nurse Practitioners 2010 December;22(12):674-83. Review article
Ref ID: 3595

(1794) Friedrich C, Port A, Ring A, Graefe-Mody U, Giessmann T, Iovino M, Woerle HJ. Effect of Multiple Oral Doses of Linagliptin on the Steady-State Pharmacokinetics of a Combination Oral Contraceptive in Healthy Female Adults. Clinical Drug Investigation 2011 September;31(9):643-53. Drug intervention study
Ref ID: 3596

(1795) Friedrich RR, Schuch I, Wagner MB. Efeito de intervenções sobre o índice de massa corporal em escolares. Effect of interventions on the body mass index of school-age students. Efecto de las intervenciones con actividad física y educación nutricional sobre el índice de masa corporal en escolares. Revista de Saúde Pública 2012 June;46(3):551-60. Review article
Ref ID: 4158

(1796) Frisch H. Growth hormone and body composition in athletes. [Review] [24 refs]. Journal of Endocrinological Investigation 1999;22(5:Suppl):Suppl-9. Review article
Ref ID: 2007

(1797) Fritsch P, Kleber M, Schlagenhauf A, Laschnik B, Fritsch M, Muntean W, Mangge H, Reinehr T. Normalization of haemostatic alterations in overweight children with weight loss due to lifestyle intervention. Atherosclerosis 2011 May;216(1):170-3. Multiple interventions
Ref ID: 2536

(1798) Frutoso MFP, Bovi TG, Gambardella AMD. Adiposidade em adolescentes e obesidade materna. Relationship between maternal obesity and adiposity in adolescents. Revista de Nutrição 2011 February;24(1):5-15. Cross-sectional study
Ref ID: 4159

(1799) Fuchs RK, Bauer JJ, Snow CM. Jumping improves hip and lumbar spine bone mass in prepubescent children: a randomized controlled trial. Journal of Bone and Mineral Research 2001 January;16(1):148-56. Not All Participants were Overweight and/or Obese
Ref ID: 1935

(1800) Fuchs SC, Moreira LB, Camey SA, Moreira MB, Fuchs FD. Clustering of risk factors for cardiovascular disease among women in Southern Brazil: A population-based study. Agregação de fatores de risco para doenças cardiovasculares em mulheres no Sul do Brasil: Um estudo de base populacional. Cadernos de Saúde Pública 2008;24(supl.2):s285-s293. Study limited to adults
Ref ID: 4160

(1801) Fuentes-Nucamendi MA, Carrillo-Muniz H, Bonfil-Ojeda JR, Frias-Austria R. [Growing pains. Simplified treatment regimen]. [Spanish]. Acta Ortopedica Mexicana 2011 March;25(2):79-86. Not a randomized controlled trial (RCT)
Ref ID: 2537

(1802) Fuentes Díaz Z, Rodríguez Salazar O, Salazar Diez M, Rodríguez Hernández O. Factores de riesgo de las enfermedades diarreicas agudas en menores de cinco años. Risk factors of the acute diarrheal diseases in children less than five years. Archivo Médico de Camagüey 2008 April;12(2). Not an exercise intervention study
Ref ID: 4161

(1803) Fuentes RM, Notkola IL, Shemeikka S, Tuomilehto J, Nissinen A. Familial aggregation of body mass index: A population-based family study in eastern Finland. Hormone and Metabolic Research 2002;34(7):406-10. Not an exercise intervention study
Ref ID: 5282

(1804) Fugh-Berman A, Kronenberg F. Complementary and alternative medicine (CAM) in reproductive-age women: A review of randomized controlled trials. Reproductive Toxicology 2003;17(2):137-52. Review article
Ref ID: 3229

(1805) Fujihara T, Gervais P. Circles with a suspended aid: reducing pommel reaction forces. Sports Biomechanics 2012 March;11(1):34-47. Not a randomized controlled trial (RCT)
Ref ID: 2538

(1806) Fuleihan GEH, Nabulsi M, Tamim H, Maalouf J, Salamoun M, Khalife H, Choucair M, Arabi A, Vieth R. Effect of vitamin D replacement on musculoskeletal parameters in school children: A randomized controlled trial. Journal of Clinical Endocrinology and Metabolism 2006;91(2):405-12. Diet Intervention or Supplement Study
Ref ID: 3230

(1807) Fulford GE, Lunn PG, Macnicol MF. A prospective study of nonoperative and operative management for Perthes' disease. Journal of Pediatric Orthopedics 1993;13:281-5. Not an exercise intervention study
Ref ID: 4787

(1808) Fullerton G, Tyler C, Johnston CA, Vincent JP, Harris GE, Foreyt JP. Quality of life in Mexican-American children following a weight management program. Obesity (Silver Spring) 2007 November;15(11):2553-6. No exercise only group
Ref ID: 208

(1809) Fulton JE, McGuire MT, Caspersen CJ, Dietz WH. Interventions for weight loss and weight gain prevention among youth: current issues. [Review] [83 refs]. Sports Medicine 2001;31(3):153-65. Review article
Ref ID: 1923

(1810) Fung EB, Xu Y, Kwiatkowski JL, Vogiatzi MG, Neufeld E, Olivieri N, Vichinsky EP, Giardina PJ, Thalassemia Clinical RN. Relationship between chronic transfusion therapy and body composition in subjects with thalassemia. Journal of Pediatrics 647;157(4):641-7. Cross-sectional study
Ref ID: 1041

(1811) Funk W, Jakob W, Riedl T, Taeger K. Oral preanaesthetic medication for children: double-blind randomized study of a combination of midazolam and ketamine vs midazolam or ketamine alone. British Journal of Anaesthesia 2000;84:335-40. Drug intervention study
Ref ID: 4788

(1812) Gabbett TJ. Physiological and anthropometric characteristics of junior rugby league players over a competitive season. Journal of Strength and Conditioning Research 2005 November;19(4):764-71. Not All Participants were Overweight and/or Obese
Ref ID: 1450

(1813) Gademan MG, Deutekom M, Hosper K, Stronks K. The effect of exercise on prescription on physical activity and wellbeing in a multi-ethnic female population: A controlled trial. BMC Public Health 2012;12:758. Inappropriate Population
Ref ID: 5857

(1814) Gajewska J, Klemarczyk W, Ambroszkiewicz J, Chelchowska M, Riahi A, Zielinska A, Oltarzewski M, Laskowska-Klita T. [Effect of weight reduction

programme on C-peptide concentration and lipid profile in obese children aged 4 to 10 years]. [Polish]. Medycyna Wieku Rozwojowego 2010 October;14(4):357-64. Lifestyle Intervention
Ref ID: 2540

(1815) Galassetti P, Larson J, Iwanaga K, Salsberg SL, Eliakim A, Pontello A. Effect of a high-fat meal on the growth hormone response to exercise in children. Journal of Pediatric Endocrinology 2006 June;19(6):777-86. Acute study
Ref ID: 1362

(1816) Gall SL, Jamrozik K, Blizzard L, Dwyer T, Venn A. Healthy lifestyles and cardiovascular risk profiles in young Australian adults: the Childhood Determinants of Adult Health Study. European Journal of Cardiovascular Prevention and Rehabilitation 2009 December;16(6):684-9. Cross-sectional study
Ref ID: 628

(1817) Gallagher KS, Davis AM, Malone B, Landrum Y, Black W. Treating rural pediatric obesity through telemedicine: baseline data from a randomized controlled trial. Journal of Pediatric Psychology 2011 July;36(6):687-95. Behavior Modification Intervention
Ref ID: 1024

(1818) Gallois KM, de HS, Hassel H, Hebestreit A, Pigeot I, Zeeb H. [Standardized development of the IDEFICS intervention and its implementation in Germany]. [German]. Bundesgesundheitsblatt, Gesundheitsforschung, Gesundheitsschutz 2011 March;54(3):330-8. Description of study from review or magazine or etc. (not the actual study)
Ref ID: 2542

(1819) Galloway SD, Craig TP, Cleland SJ. Effects of oral L-carnitine supplementation on insulin sensitivity indices in response to glucose feeding in lean and overweight/obese males. Amino Acids 2011 July;41(2):507-15. Diet Intervention Study
Ref ID: 2543

(1820) Gama SR, Carvalho MS, Cardoso LdO, Chaves CRMdM, Engstrom EM. Cohort study for monitoring cardiovascular risk factors in children using a primary health care service: Methods and initial results. Estudo de coorte para vigilância dos fatores de risco cardiovascular em crianças na atenção básica de saúde: Métodos e primeiros resultados. Cadernos de Saúde Pública 2011 March;27(3):510-20. Description of study from review or magazine or etc. (not the actual study)
Ref ID: 4162

(1821) Gamba YSS. Análise quantitativa dos benefícios da fisioterapia pré-natal na qualidade de vida de gestantes sedentárias. Reabilitar 2004 June;6(23):36-44.

Study limited to adults
Ref ID: 4163

(1822) Gambardella AMD, Bismarck-Nasi EM. Televisão e predisposição à obesidade em adolescentes. Revista Paulista de Pediatria 2000 March;18(1):18-21. Cross-sectional study
Ref ID: 4164

(1823) Gamboa-Delgado EM, López-Barbosa N, Vera-Cala LM, Prada-Gómez GE. Patrón Alimentario y Estado Nutricional en Niños Desplazados en Piedecuesta, Colombia. Revista de Salud Pública 2007 March 17;9(1):129-39. Survey or questionnaire
Ref ID: 635

(1824) Gance-Cleveland B, Sidora-Arcoleo K, Keesing H, Gottesman MM, Brady M. Changes in nurse practitioners' knowledge and behaviors following brief training on the healthy eating and activity together (HEAT) guidelines. Journal of Pediatric Health Care 2009 July;23(4):222-30. Study limited to adults
Ref ID: 733

(1825) Ganem EM, Módolo NSP, Vianna PTG, Castiglia YMM. Influência da medicação pré-anestésica com midazolam e clonidina no nível de hipnose após indução anestésica com propofol e alfentanil em crianças: Monitorização pelo índice bispectral. Influence of midazolam and clonidine premedication on hypnosis level after anesthetic induction with propofol and alfentanil in children: Bispectral index monitoring. Influencia de la medicación pré-anestésica con midazolam y clonidina en el nivel de hipnosis después de inducción anestésica con propofol y alfentanil en niños: Monitorización por el índice bispectral. Revista Brasileira de Anestesiologia 2002 February;52(1):19-23. Drug intervention study
Ref ID: 4165

(1826) Ganio MS, Johnson EC, Klau JF, Anderson JM, Casa DJ, Maresh CM, Volek JS, Armstrong LE. Effect of ambient temperature on caffeine ergogenicity during endurance exercise. European Journal of Applied Physiology 2011 June;111(6):1135-46. Study limited to adults
Ref ID: 1037

(1827) Gant N, Ali A, Foskett A. The influence of caffeine and carbohydrate coingestion on simulated soccer performance. International Journal of Sport Nutrition and Exercise Metabolism 2010 June;20(3):191-7. Diet Intervention or Supplement Study
Ref ID: 500

(1828) Gantz I, Erondu N, Mallick M, Musser B, Krishna R, Tanaka WK, Snyder K, Stevens C, Stroh MA, Zhu H, Wagner JA, Macneil DJ, Heymsfield SB, Amatruda JM. Efficacy and safety of intranasal peptide YY3-36 for weight

reduction in obese adults. Journal of Clinical Endocrinology and Metabolism 2007 May;92(5):1754-7. Study limited to adults
Ref ID: 245

(1829) Garaulet M, Ortega FB, Ruiz JR, Rey-Lopez JP, Beghin L, Manios Y, Cuenca-Garcia M, Plada M, Diethelm K, Kafatos A, Molnar D, Al-Tahan J, Moreno LA. Short sleep duration is associated with increased obesity markers in European adolescents: effect of physical activity and dietary habits. The HELENA study. International Journal of Obesity 2011 October;35(10):1308-17. Cross-sectional study
Ref ID: 2545

(1830) GarcÃ-a MDM, GarcÃ-a CMA, HernÃ¡ndez AG. Importancia de los lÃ-pidos en el tratamiento nutricional de las patologÃ-as de base inflamatoria. (Spanish). Nutricion Hospitalaria 2006 May 3;21:30-43. Review article
Ref ID: 3597

(1831) Garcia-Hermoso A, Saavedra JM, Escalante Y. Effects of exercise on resting blood pressure in obese children: a meta-analysis of randomized controlled trials. Obesity Reviews 2013 November;14(11):919-28. Inappropriate Study Design
Ref ID: 5858

(1832) Garcia-Hermoso A, Saavedra JM, Escalante Y, Sanchez-Lopez M, Martinez-Vizcaino V. Endocrinology and Adolescence: aerobic exercise reduces insulin resistance markers in obese youth: a meta-analysis of randomized controlled trials. European Journal of Endocrinology 2014 October;171(4):R163-R171. Inappropriate Study Design
Ref ID: 5859

(1833) Garcia-Hernandez P, Arechavaleta-Granell MR, Yamamoto J, Falahati A, Gonzalez-Galvez G, Grupo de Investigadores de LEAD-. [Liraglutide and glimepiride on glycaemic control in type 2 diabetes in the Mexican cohort (LEAD 3)]. [Spanish]. Revista Medica del Instituto Mexicano del Seguro Social 2010 September;48(5):543-8. Drug intervention study
Ref ID: 2546

(1834) Garcia-Marcos L, Canflanca IM, Garrido JB, Varela AL, Garcia-Hernandez G, Guillen GF, Gonzalez-Diaz C, Carvajal-Uruena I, Arnedo-Pena A, Busquets-Monge RM, Morales Suarez-Varela M, Blanco-Quiros A. Relationship of asthma and rhinoconjunctivitis with obesity, exercise and Mediterranean diet in Spanish schoolchildren. Thorax 2007 June;62(6):503-8. Cross-sectional study
Ref ID: 1227

(1835) Garcia-Morales LM, Berber A, Macias-Lara CC, Lucio-Ortiz C, Del-Rio-Navarro BE, Dorantes-Alvarez LM. Use of sibutramine in obese mexican adolescents: a 6-month, randomized, double-blind, placebo-controlled, parallel-group trial.

Clinical Therapeutics 2006 May;28(5):770-82. No exercise only group
Ref ID: 274

(1836) Garcia-Pallares J, Lopez-Gullon JM, Muriel X, Diaz A, Izquierdo M. Physical fitness factors to predict male Olympic wrestling performance. European Journal of Applied Physiology 2011 August;111(8):1747-58. Not an exercise intervention study
Ref ID: 2547

(1837) Garcia LMT, Freire CC, Pereira DZ, Oliveira JLd, Vitalle MSdS. Do diagnostico a acao: Programa de Atividades para o Paciente obeso (PAPo) - uma abordagem interdisciplinar com adolescentes. From diagnosis to action: Program of Activities for the obese Patient (PAPo) - an interdisciplinary approach with adolescents. Revista Brasileira de Atividade Física e Saúde 2010 September;15(3). Description of study from review or magazine or etc. (not the actual study)
Ref ID: 4166

(1838) Garcinuno AC, Garcia IP, Alonso IC, Lopez SA. Determining factors of physical activity level in school children and adolescents: The OPACA study. Anales de Pediatria 2011;74(1):15-24. Cross-sectional study
Ref ID: 5283

(1839) García-Londoño G, Liévano-Fiesco MC, Leclercq-Barriga MH, Liévano de Lombo G, Cuervo Lozada NA. Caracterización de los hábitos alimentarios y estilos de vida de los niños, de la institución obra misionera Jesús y María, Jardín Sol - Solecito, localidad de Suba, Bogotá D. C. University Science 2008 December;13(3):290-7. Cross-sectional study
Ref ID: 4167

(1840) Gardner B, Wardle J, Poston L, Croker H. Changing diet and physical activity to reduce gestational weight gain: a meta-analysis. [Review]. Obesity Reviews 2011 July;12(7):e602-e620. Review article
Ref ID: 2548

(1841) Gardner J, Wilkinson P. Is family therapy the most effective treatment for anorexia nervosa?. [Review]. Psychiatria Danubina 2011 September;23:Suppl-7. Review article
Ref ID: 2549

(1842) Gariod L, Binzoni T, Ferretti G, Lebas JF, Reutenauer H, Cerretelli P. Standardization of (31)Phosphorus-Nuclear Magnetic-Resonance Spectroscopy Determinations of High-Energy Phosphates in Humans. European Journal of Applied Physiology and Occupational Physiology 1994;68(2):107-10. Not an exercise intervention study
Ref ID: 5284

(1843) Garnett SP, Baur LA, Noakes M, Steinbeck K, Woodhead HJ, Burrell S, Chisholm K, Broderick CR, Parker R, De S, Shrinivasan S, Hopley L, Hendrie G, Ambler GR, Kohn MR, Cowell CT. Researching Effective Strategies to Improve Insulin Sensitivity in Children and Teenagers - RESIST. A randomised control trial investigating the effects of two different diets on insulin sensitivity in young people with insulin resistance and/or pre-diabetes. BMC Public Health 2010 September 25;10:575.:575. Description versus conduct of study, No exercise only group
Ref ID: 21

(1844) Garnett SP, Gow M, Ho M, Baur LA, Noakes M, Woodhead HJ, Broderick CR, Burrell S, Chisholm K, Halim J, De S, Steinbeck K, Srinivasan S, Ambler GR, Kohn MR, Cowell CT. Optimal macronutrient content of the diet for adolescents with prediabetes; RESIST a randomised control trial. Journal of Clinical Endocrinology and Metabolism 2013 May;98(5):2116-25. Inappropriate Intervention
Ref ID: 5860

(1845) Garnier D, Benefice E. Reliable method to estimate characteristics of sleep and physical inactivity in free-living conditions using accelerometry. Annals of Epidemiology 2006;16(5):364-9. Not a randomized controlled trial (RCT)
Ref ID: 5285

(1846) Garnock-Jones KP, Keating GM. Atomoxetine. Pediatrics Drugs 2009 June;11(3):203-26. Drug intervention study
Ref ID: 3598

(1847) Garthe I, Raastad T, Refsnes PE, Koivisto A, Sundgot-Borgen J. Effect of two different weight-loss rates on body composition and strength and power-related performance in elite athletes. International Journal of Sport Nutrition and Exercise Metabolism 2011 April;21(2):97-104. Diet Intervention Study
Ref ID: 2553

(1848) Garthe I, Raastad T, Sundgot-Borgen J. Long-term effect of nutritional counselling on desired gain in body mass and lean body mass in elite athletes. Applied Physiology, Nutrition, and Metabolism = Physiologie Appliquee, Nutrition et Metabolisme 2011 August;36(4):547-54. Diet Intervention Study
Ref ID: 2552

(1849) Garthe I, Raastad T, Sundgot-Borgen J. Long-term effect of weight loss on body composition and performance in elite athletes. International Journal of Sport Nutrition and Exercise Metabolism 2011 October;21(5):426-35. Multiple interventions
Ref ID: 2551

(1850) Gasperin D, Fensterseifer LM. [Changes in the lifestyle of hypertensive patients]. [Review] [37 refs] [Portuguese]. Revista Gaucha de Enfermagem

2006 September;27(3):372-8. Review article
Ref ID: 1305

(1851) Gassmann B. Dietary Reference Intakes (DRI), Report 6, Part 1: Energy, carbohydrates, and fiber. Ernahrungs-Umschau 2003;50(3):96-+. Review article
Ref ID: 5286

(1852) Gately PJ, King NA, Greatwood HC, Humphrey LC, Radley D, Cooke CB, Hill AJ. Does a high-protein diet improve weight loss in overweight and obese children? Obesity (Silver Spring) 2007 June;15(6):1527-34. No exercise only group
Ref ID: 236

(1853) Gates D, Brehm B, Hutton S, Singler M, Poeppelman A. Changing the work environment to promote wellness: a focus group study. AAOHN Journal 2006 December;54(12):515-20. Not a randomized controlled trial (RCT)
Ref ID: 1312

(1854) Gates PE, Campbell IG, George KP. Absence of training-specific cardiac adaptation in paraplegic athletes. Medicine and Science in Sports and Exercise 2002 November;34(11):1699-704. Not a randomized controlled trial (RCT)
Ref ID: 1792

(1855) Gates PE, Strain WD, Shore AC. Human endothelial function and microvascular ageing. Experimental Physiology 2009;94(3):311-6. Review article
Ref ID: 5287

(1856) Gates PE, Banks D, Johnston TE, Campbell SR, Gaughan JP, Ross SA, Engsberg JR, Tucker C. Randomized controlled trial assessing participation and quality of life in a supported speed treadmill training exercise program vs. a strengthening program for children with cerebral palsy. Journal of Pediatric Rehabilitation Medicine 2012;5(2):75-88. No non-intervention control group
Ref ID: 3232

(1857) Gathwala G, Singh B, Balhara B. KMC facilitates mother baby attachment in low birth weight infants. Indian Journal of Pediatrics 2008;75:43-7. Subjects less than 2 years old
Ref ID: 4791

(1858) Gathwala G, Singh B, Singh J. Effect of Kangaroo Mother Care on physical growth, breastfeeding and its acceptability. Tropical Doctor 2010;40:199-202. Subjects less than 2 years old
Ref ID: 4792

(1859) Gaullier JM, Halse J, Hoivik HO, Hoye K, Syvertsen C, Nurminiemi M, Hassfeld C, Einerhand A, O'Shea M, Gudmundsen O. Six months supplementation with

conjugated linoleic acid induces regional-specific fat mass decreases in overweight and obese. British Journal of Nutrition 2007 March;97(3):550-60. Study limited to adults
Ref ID: 249

(1860) Gaya AR, Matzenbacher J, Martins C, Gaya A, Mansilha H, Mota J. Resistência à insulina e excesso de peso corporal. Revista Brasileira de Atividade Física e Saúde 2008;13(3). Cross-sectional study
Ref ID: 4168

(1861) Geerling BJ, StockbrÃ¼gger RW, Brummer R-JM. Nutrition and Inflammatory Bowel Disease: An Update. Scandinavian Journal of Gastroenterology Supplement 1999 September 7;34:95-105. Review article
Ref ID: 534

(1862) Geleijnse JM, Kok FJ, Grobbee DE. Impact of dietary and lifestyle factors on the prevalence of hypertension in Western populations. European Journal of Public Health 2004 September;14(3):235-9. Survey or questionnaire
Ref ID: 1589

(1863) Gendall KA, Joyce PR, Carter FA, McIntosh VV, Jordan J, Bulik CM. The psychobiology and diagnostic significance of amenorrhea in patients with anorexia nervosa. Fertility and Sterility 2006 May;85(5):1531-5. Study limited to adults
Ref ID: 1394

(1864) Gentile DA, Welk G, Eisenmann JC, Reimer RA, Walsh DA, Russell DW, Callahan R, Walsh M, Strickland S, Fritz K. Evaluation of a multiple ecological level child obesity prevention program: Switch what you Do, View, and Chew. BMC Medicine 2009 September 18;7:49.:49. No exercise only group
Ref ID: 94

(1865) George A, Bhaduri A, Sen S, Choudhry VP. Physical growth parameters in thalassemic children. Indian Journal of Pediatrics 1997;64:861-71. Not a randomized controlled trial (RCT)
Ref ID: 4793

(1866) George CL, Oriel KN, Blatt PJ, Marchese V. Impact of a community-based exercise program on children and adolescents with disabilities. Journal of Allied Health 2011;40(4):e55-e60. Not All Participants were Overweight and/or Obese
Ref ID: 2554

(1867) George E, Noel F, Bois G, Cassagnol R, Estavien L, Rouzier PD, Verdier RI, Johnson WD, Pape JW, Fitzgerald DW, Wright PF. Antiretroviral therapy for HIV-1-infected children in Haiti. Journal of Infectious Diseases 2007;195(10):1411-8. Not an exercise intervention study
Ref ID: 5288

(1868) George K, Oxborough D, Forster J, Whyte G, Shave R, Dawson E, Stephenson C, Dugdill L, Edwards B, Gaze D. Mitral annular myocardial velocity assessment of segmental left ventricular diastolic function after prolonged exercise in humans. Journal of Physiology 2005 November 15;569(Pt:1):1-13. Not a randomized controlled trial (RCT)
Ref ID: 1446

(1869) George SZ, Childs JD, Teyhen DS, Wu SS, Wright AC, Dugan JL, Robinson ME. Predictors of occurrence and severity of first time low back pain episodes: findings from a military inception cohort. PLoS ONE [Electronic Resource] 2012;7(2):e30597. Cohort Study
Ref ID: 2555

(1870) Georgiou C, Betts N, Hoos T, Glenn M. Young adult exercisers and nonexercisers differ in food attitudes, perceived dietary changes, and food choices. International Journal of Sport Nutrition 1996 December;6(4):402-13. Study limited to adults
Ref ID: 2154

(1871) Georgopoulos NA, Markou KB, Theodoropoulou A, Vagenakis GA, Benardot D, Leglise M, Dimopoulos JC, Vagenakis AG. Height velocity and skeletal maturation in elite female rhythmic gymnasts. Journal of Clinical Endocrinology and Metabolism 2001 November;86(11):5159-64. Prospective Study
Ref ID: 1881

(1872) Gernaat HBPE, Dechering WHJC, Voorhoeve HWA. Physical growth of children under five years of age in Nchelenge, Zambia: Results from a district survey. American Journal of Physical Anthropology 1996;100(4):473-85. Not an exercise intervention study
Ref ID: 5289

(1873) Gerstenberger SL, Martinson A, Kramer JL. An Evaluation of Mercury Concentrations in Three Brands of Canned Tuna. Environmental Toxicology and Chemistry 2010;29(2):237-42. Not an exercise intervention study
Ref ID: 5290

(1874) Gerster H. N-3 fish oil polyunsaturated fatty acids and bleeding. Journal of Nutritional and Environmental Medicine 1995 June;5(3):281. Review article
Ref ID: 3599

(1875) Gesell SB, Scott TA, Barkin SL. Accuracy of perception of body size among overweight Latino preadolescents after a 6-month physical activity skills building intervention. Clinical Pediatrics (Philadelphia) 2010 April;49(4):323-9. Behavior Modification Intervention
Ref ID: 109

(1876) Gesell SB, Tesdahl E, Ruchman E. The Distribution of Physical Activity in an After-school Friendship Network. Pediatrics 2012;129(6):1064-71. Not an

exercise intervention study
Ref ID: 5291

(1877) Getz L, Nilsson PM, Hetlevik I. A matter of heart: the general practitioner consultation in an evidence-based world. Scandinavian Journal of Primary Health Care 2003 March;21(1):3. Not an exercise intervention study
Ref ID: 3600

(1878) Gey KF. Vitamins E plus C andd interacting conutrients required for optimal health. Biofactors 1998 January;7(1/2):113. Diet Intervention or Supplement Study
Ref ID: 3601

(1879) Ghavamzadeh S, Khalkhali HR, Alizadeh M. TV viewing, independent of physical activity and obesogenic foods, increases overweight and obesity in adolescents. Journal of Health, Population, and Nutrition 2013 September;31(3):334-42. Inappropriate Study Design
Ref ID: 5861

(1880) Ghio S, Scelsi L, Latini R, Masson S, Eleuteri E, Palvarini M, Vriz O, Pasotti M, Gorini M, Marchioli R, Maggioni A, Tavazzi L. Effects of n-3 polyunsaturated fatty acids and of rosuvastatin on left ventricular function in chronic heart failure: a substudy of GISSI-HF trial. European Journal of Heart Failure 2010 December;12(12):1345-53. Diet Intervention or Supplement Study
Ref ID: 3602

(1881) Ghroubi S, Elleuch H, Chikh T, Kaffel N, Abid M, Elleuch MH. Physical training combined with dietary measures in the treatment of adult obesity. A comparison of two protocols. Annals of Physical and Rehabilitation Medicine 2009 June;52(5):394-413. Study limited to adults
Ref ID: 104

(1882) Giardini A, Balducci A, Specchia S, Gargiulo G, Bonvicini M, Picchio FM. Effect of sildenafil on haemodynamic response to exercise and exercise capacity in Fontan patients. European Heart Journal 2008 July;29(13):1681-7. Drug intervention study
Ref ID: 925

(1883) Gibbs L, O'Connor T, Waters E, Booth M, Walsh O, Green J, Bartlett J, Swinburn B. Addressing the potential adverse effects of school-based BMI assessments on children's wellbeing. International Journal of Pediatric Obesity 2008;3(1):52-7. Not All Participants were Overweight and/or Obese
Ref ID: 2556

(1884) Gidding SS, Barton BA, Dorgan JA, Kimm SY, Kwiterovich PO, Lasser NL, Robson AM, Stevens VJ, Van HL, Simons-Morton DG. Higher self-reported physical activity is associated with lower systolic blood pressure: the Dietary Intervention Study in Childhood (DISC). Pediatrics 2006

December;118(6):2388-93. Diet Intervention Study
Ref ID: 1319

(1885) Gieck DJ, Olsen S. Holistic wellness as a means to developing a lifestyle approach to health behavior among college students. Journal of American College Health 2007 July;56(1):29-36. Study limited to adults
Ref ID: 3816

(1886) Gilbert ME, MacPhail R, Baldwin J, Moser VC, Chernoff N. Moderate developmental undernutrition: Impact on growth and cognitive function in youth and old age. Neurotoxicology and Teratology 2010;32(3):362-72. Animal study
Ref ID: 5292

(1887) Gilbert MJ, Fleming MF. Use of enhanced body mass index charts during the pediatric health supervision visit increases physician recognition of overweight patients. Clinical Pediatrics 2007 October;46(8):689-97. Not an exercise intervention study
Ref ID: 1157

(1888) Gilchrist NL, Frampton CM, Acland RH, Nicholls MG, March RL, Maguire P, Heard A, Reilly P, Marshall K. Alendronate prevents bone loss in patients with acute spinal cord injury: a randomized, double-blind, placebo-controlled study. Journal of Clinical Endocrinology and Metabolism 2007 April;92(4):1385-90. Drug intervention study
Ref ID: 1254

(1889) Giles BE, Walker JS. Sex differences in pain and analgesia. Pain Reviews 2000 October;7(3/4):181-93. Review article
Ref ID: 3603

(1890) Gilger MA, Tolia V, Vandenplas Y, Youssef NN, Traxler B, Illueca M. Safety and tolerability of esomeprazole in children with gastroesophageal reflux disease. Journal of Pediatric Gastroenterology and Nutrition 2008;46:524-33. Drug intervention study
Ref ID: 4794

(1891) Gillett M, Royle P, Snaith A, Scotland G, Poobalan A, Imamura M, Black C, Boroujerdi M, Jick S, Wyness L, McNamee P, Brennan A, Waugh N. Non-pharmacological interventions to reduce the risk of diabetes in people with impaired glucose regulation: a systematic review and economic evaluation. Health Technology Assessment 2012 August;16(33):1-iv. Inappropriate Study Design
Ref ID: 5862

(1892) Gillett RM, Tobias PV. Human growth in southern Zambia: A first study of Tonga children predating the Kariba Dam (1957-1958). American Journal of Human Biology 2002;14(1):50-60. Not an exercise intervention study
Ref ID: 5293

(1893) Gillingham MB, Scott B, Elliott D, Harding CO. Metabolic control during exercise with and without medium-chain triglycerides (MCT) in children with long-chain 3-hydroxy acyl-CoA dehydrogenase (LCHAD) or trifunctional protein (TFP) deficiency. Molecular Genetics and Metabolism 2006;89:58-63. Diet Intervention or Supplement Study
Ref ID: 4795

(1894) Gillis B, Mobley C, Stadler DD, Hartstein J, Virus A, Volpe SL, El GL, Staten MA, Bridgman J, McCormick S, HEALTHY study group. Rationale, design and methods of the HEALTHY study nutrition intervention component. International Journal of Obesity 2009 August;33:Suppl-36. Description of study from review or magazine or etc. (not the actual study)
Ref ID: 714

(1895) Gillis D, Brauner M, Granot E. A community-based behavior modification intervention for childhood obesity. Journal of Pediatric Endocrinology and Metabolism 2007 February;20(2):197-203. No exercise only group
Ref ID: 244

(1896) Gillman MW, Oakey H, Baghurst PA, Volkmer RE, Robinson JS, Crowther CA. Effect of treatment of gestational diabetes mellitus on obesity in the next generation. Diabetes Care 2010;33:964-8. Not an exercise intervention study
Ref ID: 4796

(1897) Gilsanz V, Wren TA, Sanchez M, Dorey F, Judex S, Rubin C. Low-level, high-frequency mechanical signals enhance musculoskeletal development of young women with low BMD. Journal of Bone and Mineral Research 2006 September;21(9):1464-74. Inappropriate Intervention
Ref ID: 1354

(1898) Gin S, Philip B, Caterson I. Why getting fat, Doc? Weight gain and psychotropic medications. Australian and New Zealand Journal of Psychiatry 2001 June;35(3):315-21. Review article
Ref ID: 3604

(1899) Ginde SR, Geliebter A, Rubiano F, Silva AM, Wang J, Heshka S, Heymsfield SB. Air displacement plethysmography: Validation in overweight and obese subjects. Obesity Research 2005;13(7):1232-7. Not an exercise intervention study
Ref ID: 5294

(1900) Giraldo D, Poveda E, Yibby F, Mendivil C, Castro L. Actividad física autorreportada, comparación con indicadores antropométricos de grasa corporal en un grupo de escolares de Bogotá y de cinco departamentos del centro-oriente, Colombia 2000-2002. Biomédica (Bogotá) 2008 September;28(3):386-95. Cross-sectional study
Ref ID: 4169

(1901) Giralt M, Albaladejo R, Tarro L, Moriña D, Arija V, Solà R. A primary-school-based study to reduce prevalence of childhood obesity in Catalunya (Spain)--EDAL-Educació en alimentació: study protocol for a randomised controlled trial. Trials 2011;12:54. Description of study from review or magazine or etc. (not the actual study)
Ref ID: 4797

(1902) Giraud DW, Kim YN, Cho YO, Driskell JA. Vitamin E Inadequacy Observed in a Group of 2-to 6-Year-Old Children Living in Kwangju, Republic of Korea. International Journal for Vitamin and Nutrition Research 2008;78(3):148-55. Not an exercise intervention study
Ref ID: 5295

(1903) Girotto CA, Vacchino MN, Spillmann CA, Soria JA. Prevalencia de factores de riesgo cardiovascular en ingresantes universitarios. Revista de Saúde Pública 1996 December;30(6):576-86. Not an exercise intervention study
Ref ID: 797

(1904) Gisel EG. Effect of oral sensorimotor treatment on measures of growth and efficiency of eating in the moderately eating-impaired child with cerebral palsy. Dysphagia 1996;11(1):48-58. Diet Intervention Study
Ref ID: 2183

(1905) Gittelsohn J, Merkle S, Story M, Stone EJ, Steckler A, Noel J, Davis S, Martin CJ, Ethelbah B. School climate and implementation of the Pathways study. Preventive Medicine 2003 December;37(6:Pt 2):t-106. Multiple interventions
Ref ID: 1694

(1906) Giunta M, Cardinale M, Agosti F, Patrizi A, Compri E, Rigamonti AE, Sartorio A. Growth hormone-releasing effects of whole body vibration alone or combined with squatting plus external load in severely obese female subjects. Obesity Facts 2012;5(4):567-74. Inappropriate Population
Ref ID: 5863

(1907) Giusti, Meineri, Malagamba, Cuttica, Fattacciu, Menichini, Rasore, Giordano, Giusti. Impact of recombinant human growth hormone treatment on psychological profiles in hypopituitary patients with adult-onset growth hormone deficiency. European Journal of Clinical Investigation 1998 January;28(1):13-9. Study limited to adults
Ref ID: 3605

(1908) Giustina A, Malerba M, Bresciani E, Desenzani P, Licini M, Zaltieri G, Grassi V. Effect of two beta 2-agonist drugs, salbutamol and broxaterol, on the growth hormone response to exercise in adult patients with asthmatic bronchitis. Journal of Endocrinological Investigation 1995 December;18(11):847-52. Drug intervention study
Ref ID: 2189

(1909) Glaner MF. Crescimento físico em adolescentes do norte gaúcho e oeste catarinense. Revista Brasileira de Ciência e Movimento 2005;13(2):15-26. Cross-sectional study
Ref ID: 4170

(1910) Glazebrook C, Batty MJ, Mullan N, Macdonald I, Nathan D, Sayal K, Smyth A, Yang M, Guo B, Hollis C. Evaluating the effectiveness of a schools-based programme to promote exercise self-efficacy in children and young people with risk factors for obesity: steps to active kids (STAK). BMC Public Health 2011;11:830. Description of study from review or magazine or etc. (not the actual study)
Ref ID: 1068

(1911) Glenny AM, O'Meara S, Melville A, Sheldon TA, Wilson C. The treatment and prevention of obesity: a systematic review of the literature. [Review] [121 refs]. International Journal of Obesity and Related Metabolic Disorders 1997 September;21(9):715-37. Review article
Ref ID: 2120

(1912) Godard M, Rodríguez N, Díaz N, Lera M, Salazar R, BURROWS A. Valor de un test clínico para evaluar actividad física en niños. Revista médica de Chile 2008 September;136(9):1155-62. Survey or questionnaire
Ref ID: 4171

(1913) Godfrey S. Problems peculiar to the diagnosis and management of asthma in children. [Review] [53 refs]. BTTA Review 1974 March;4(1):1-16. Review article
Ref ID: 2558

(1914) Godin G, Belanger-Gravel A, Amireault S, Gallani MC, Vohl MC, Perusse L. Effect of implementation intentions to change behaviour: moderation by intention stability. Psychological Reports 2010 February;106(1):147-59. Primary outcome(s) not assessed
Ref ID: 49

(1915) Godoy-Matos A, Carraro L, Vieira A, Oliveira J, Guedes EP, Mattos L, Rangel C, Moreira RO, Coutinho W, Appolinario JC. Treatment of obese adolescents with sibutramine: a randomized, double-blind, controlled study. Journal of Clinical Endocrinology and Metabolism 2005 March;90(3):1460-5. No exercise only group, Drug intervention study
Ref ID: 318

(1916) Godoy-Matos AF, Guedes EP, Souza LLd, Martins MF. Management of obesity in adolescents: State of art. Controle da obesidade em adolescentes: atualização. Arquivos Brasileiros de Endocrinologia and Metabologia 2009 March;53(2):252-61. Review article
Ref ID: 4172

(1917) Goemans NM, Tulinius M, van den Akker JT, Burm BE, Ekhart PF, Heuvelmans N, Holling T, Janson AA, Platenburg GJ, Sipkens JA, Sitsen JM, Aartsma-Rus A, van Ommen GJ, Buyse G, Darin N, Verschuuren JJ, Campion GV, de Kimpe SJ, van Deutekom JC. Systemic administration of PRO051 in Duchenne's muscular dystrophy.[Erratum appears in New England Journal of Medicine. 2011 Oct 6;365(14):1361]. New England Journal of Medicine 2011 April 21;364(16):1513-22. Drug intervention study
Ref ID: 2559

(1918) Goff DC, Sullivan LM, McEvoy JP, Meyer JM, Nasrallah HA, Daumit GL, Lamberti S, D'Agostino RB, Stroup TS, Davis S, Lieberman JA. A comparison of ten-year cardiac risk estimates in schizophrenia patients from the CATIE study and matched controls. Schizophrenia Research 2005 December 1;80(1):45-53. Not an exercise intervention study
Ref ID: 1448

(1919) Gogakos A, Tzotzas T, Krassas GE. Recent concepts of pharmacotherapy and bariatric surgery for childhood obesity: An overview. Pediatric Endocrinology Reviews 2009;7(2):3-14. Review article
Ref ID: 3233

(1920) Going S, Thompson J, Cano S, Stewart D, Stone E, Harnack L, Hastings C, Norman J, Corbin C. The effects of the Pathways Obesity Prevention Program on physical activity in American Indian children. Preventive Medicine 2003 December;37(6 Pt 2):S62-S69. Not All Participants were Overweight and/or Obese
Ref ID: 346

(1921) Gokee-LaRose J, Gorin AA, Raynor HA, Laska MN, Jeffery RW, Levy RL, Wing RR. Are standard behavioral weight loss programs effective for young adults? International Journal of Obesity (London) 2009 December;33(12):1374-80. Study limited to adults
Ref ID: 92

(1922) Gokee LJ, Tate DF, Gorin AA, Wing RR. Preventing weight gain in young adults: a randomized controlled pilot study. American Journal of Preventive Medicine 2010 July;39(1):63-8. Study not limited to children and adolescents
Ref ID: 40

(1923) Golan M, Fainaru M, Weizman A. Role of behaviour modification in the treatment of childhood obesity with the parents as the exclusive agents of change. International Journal of Obesity and Related Metabolic Disorders 1998 December;22(12):1217-24. No exercise only group, No comparative control group
Ref ID: 418

(1924) Golan M, Weizman A, Fainaru M. Impact of treatment for childhood obesity on parental risk factors for cardiovascular disease. Preventive Medicine 1999 December;29(6 Pt 1):519-26. No exercise only group, No comparative control group
Ref ID: 405

(1925) Golan M, Kaufman V, Shahar DR. Childhood obesity treatment: targeting parents exclusively v. parents and children. British Journal of Nutrition 2006 May;95(5):1008-15. No exercise only group, No comparative control group
Ref ID: 280

(1926) Gold DR, Wright R. Population Disparities in Asthma. Annual Review of Public Health 2005 April;26(1):89-113. Review article
Ref ID: 3606

(1927) Gold EB, Colvin A, Avis N, Bromberger J, Greendale GA, Powell L, Sternfeld B, Matthews K. Longitudinal Analysis of the Association Between Vasomotor Symptoms and Race/Ethnicity Across the Menopausal Transition: Study of Women's Health Across the Nation. American Journal of Public Health 2006 July;96(7):1226-35. Study limited to adults
Ref ID: 3817

(1928) Gold PW, Chrousos GP. Organization of the stress system and its dysregulation in melancholic and atypical depression: high vs low CRH/NE states. Molecular Psychiatry 2002 March;7(3):254. Not an exercise intervention study
Ref ID: 3607

(1929) Goldberg G. Review Flair-Flow 4: Synthesis report on obesity for health professionals. Nutrition Bulletin 2003 December;28(4):343-54. Review article
Ref ID: 3608

(1930) Golden NH, Iglesias EA, Jacobson MS, Carey D, Meyer W, Schebendach J, Hertz S, Shenker IR. Alendronate for the treatment of osteopenia in anorexia nervosa: a randomized, double-blind, placebo-controlled trial. Journal of Clinical Endocrinology and Metabolism 2005 June;90(6):3179-85. Drug intervention study
Ref ID: 1511

(1931) Goldfield GS, Kalakanis LE, Ernst MM, Epstein LH. Open-loop feedback to increase physical activity in obese children. International Journal of Obesity and Related Metabolic Disorders 2000 July;24(7):888-92. Reward given for exercise, No comparative control group
Ref ID: 402

(1932) Goldfield GS, Epstein LH, Kilanowski CK, Paluch RA, Kogut-Bossler B. Cost-effectiveness of group and mixed family-based treatment for childhood obesity. International Journal of Obesity and Related Metabolic Disorders 2001

December;25(12):1843-9. No exercise only group, No comparative control group
Ref ID: 387

(1933) Goldfield GS, Mallory R, Parker T, Cunningham T, Legg C, Lumb A, Parker K, Prud'homme D, Gaboury I, Adamo KB. Effects of open-loop feedback on physical activity and television viewing in overweight and obese children: a randomized, controlled trial. Pediatrics 2006 July;118(1):e157-e166. No comparative control group
Ref ID: 276

(1934) Goldfield GS, Mallory R, Parker T, Cunningham T, Legg C, Lumb A, Parker K, Prud'homme D, Adamo KB. Effects of modifying physical activity and sedentary behavior on psychosocial adjustment in overweight/obese children. Journal of Pediatric Psychology 2007 August;32(7):783-93. Secondary analysis
Ref ID: 240

(1935) Goldfield GS, Mallory R, Prud'homme D, Adamo KB. Gender differences in response to a physical activity intervention in overweight and obese children. Journal of Physical Activity and Health 2008 July;5(4):592-606. Reward given for exercise, No comparative control group
Ref ID: 166

(1936) Goldfield GS. Predictors of response to an intervention modifying physical activity and sedentary behavior in overweight/obese children: attitudes vs. behavior. Journal of Physical Activity and Health 2009 July;6(4):463-6. Secondary analysis
Ref ID: 90

(1937) Goldfield GS, Adamo KB, Rutherford J, Murray M. The effects of aerobic exercise on psychosocial functioning of adolescents who are overweight or obese. Journal of Pediatric Psychology 2012 November;37(10):1136-47. Inappropriate Comparison Group
Ref ID: 5864

(1938) Goldfield GS. Making access to TV contingent on physical activity: effects on liking and relative reinforcing value of TV and physical activity in overweight and obese children. Journal of Behavioral Medicine 2012 February;35(1):1-7. Secondary analysis
Ref ID: 2560

(1939) Goldfine BD, Nahas MV. Incorporating health-fitness concepts in secondary physical education curricula. Journal of School Health 1993 March;63(3):142-6. Not All Participants were Overweight and/or Obese
Ref ID: 2264

(1940) Goldman LR. New approaches for assessing the etiology and risks of developmental abnormalities from chemical exposure. Reproductive Toxicology

1997;11(2-3):443-51. Not an exercise intervention study
Ref ID: 5296

(1941) Goldrosen MH, Straus SE. Science and society: Complementary and alternative medicine: assessing the evidence for immunological benefits. Nature Reviews Immunology 2004 November;4(11):912-21. Review article
Ref ID: 3609

(1942) Goldsmith DR, Wagstaff AJ. Mammalian Cell-Derived Somatropin: A Review of its Use in the Management of HIV-Associated Wasting. Drugs 2006 February;66(3):387. Review article
Ref ID: 3610

(1943) Goldstein I. Current Management Strategies of the Postmenopausal Patient with Sexual Health Problems. Journal of Sexual Medicine 2007 January 4;4:235-53. Study limited to adults
Ref ID: 3611

(1944) Golebiowska M, Chlebna-Sokol D, Mastalska A, Zwaigzne-Raczynska J. [The clinical evaluation of teronac (Mazindol) in the treatment of children with obesity. Part I. Effect of the drug on somatic patterns and exercise capacity (author's transl)]. [Polish]. Przeglad Lekarski 1981;38(2):311-4. Drug intervention study
Ref ID: 2372

(1945) Golikov V, Wallstrom E, Wohni T, Tanaka K, Endo S, Hoshi M. Evaluation of conversion coefficients from measurable to risk quantities for external exposure over contaminated soil by use of physical human phantoms. Radiation and Environmental Biophysics 2007;46(4):375-82. Not an exercise intervention study
Ref ID: 5297

(1946) Golley RK, Magarey AM, Baur LA, Steinbeck KS, Daniels LA. Twelve-month effectiveness of a parent-led, family-focused weight-management program for prepubertal children: a randomized, controlled trial. Pediatrics 2007;119:517-25. Behavior Modification Intervention
Ref ID: 4798

(1947) Golley RK, Magarey AM, Daniels LA. Children's food and activity patterns following a six-month child weight management program. International Journal of Pediatric Obesity 2011 October;6(5-6):409-14. Behavior Modification Intervention
Ref ID: 2561

(1948) Gomes BdMR, Alves JGB. Prevalência de hipertensão arterial e fatores associados em estudantes de Ensino Médio de escolas públicas da Região Metropolitana do Recife, Pernambuco, Brasil, 2006t. Cadernos de Saúde

Pública 2009 February;25(2):375-81. Cross-sectional study
Ref ID: 479

(1949) Gomes RV, Ribeiro SM, Veibig RF, Aoki MS. Consumo alimentar e perfil antropométrico de tenistas amadores e profissionais. Food intake and anthropometric profile of amateur and professionals tennis players. Revista Brasileira de Medicina do Esporte 2009 December;15(6):436-40. Cross-sectional study
Ref ID: 4173

(1950) Gomez CC, Olivar RJ, Garcia M, Marin M, Madero R, Perez-Portabella C, Planas M, Mokoroa A, Pereyra F, Martin PA. [Assessment of a malnutrition screening tool in cancer patients]. [Spanish]. Nutricion Hospitalaria 2010 May;25(3):400-5. Not an exercise intervention study
Ref ID: 503

(1951) Gomez L, Jacoby E, Ibarra L, Lucumi D, Hernandez A, Parra D, Florindo A, Hallal P. Sponsorship of physical activity programs by the sweetened beverages industry: public health or public relations? Revista de Saude Publica 2011;45(2):423-7. Editorial or letter or comment
Ref ID: 5298

(1952) Gonçalves H, Hallal PC, Amorim TC, Araújo CLP, Menezes AMB. Fatores socioculturais e nível de atividade física no início da adolescência. Revista Panamericana de Salud Pública 2007 October;22(4):246-53. Cohort Study
Ref ID: 4174

(1953) Gonçalves H, Dumith SC, González DA, Menezes AMB, Araújo CLP, Hallal PC, Bastos JL. Discriminação autorrelatada por adolescentes de uma coorte de nascimentos brasileira: Prevalência e associações. Self-reported discrimination by adolescents in a Brazilian birth cohort: Prevalence and associations. Revista Panamericana de Salud Pública 2012 March;31(3):204-10. Cross-sectional study, Description of study from review or magazine or etc. (not the actual study)
Ref ID: 4175

(1954) Gonçalves HR, Arruda M, Barros Filho AdA, Gonçalves LAP. Composição corporal em escolares de 7 a 14 anos de ambos os sexos de alto nível sócio-econômico. Arquivos de Ciências da Saúde da UNIPAR 2002 December;6(3):119-26. Cross-sectional study
Ref ID: 4176

(1955) Gong L, Yuan F, Teng J, Li X, Zheng S, Lin L, Deng H, Ma G, Sun C, Li Y. Weight loss, inflammatory markers, and improvements of iron status in overweight and obese children. Journal of Pediatrics 2014 April;164(4):795-800. Inappropriate Intervention
Ref ID: 5865

(1956) Gonzales JU, Thistlethwaite JR, Thompson BC, Scheuermann BW. Exercise-induced shear stress is associated with changes in plasma von Willebrand factor in older humans. European Journal of Applied Physiology 2009 July;106(5):779-84. Study limited to adults
Ref ID: 727

(1957) Gonzalez-Aguero A, Vicente-Rodriguez G, Gomez-Cabello A, Ara I, Moreno LA, Casajus JA. A combined training intervention programme increases lean mass in youths with Down syndrome. Research in Developmental Disabilities 2011 November;32(6):2383-8. Not All Participants were Overweight and/or Obese
Ref ID: 2562

(1958) Gonzalez-Aguero A, Vicente-Rodriguez G, Gomez-Cabello A, Ara I, Moreno LA, Casajus JA. A 21-week bone deposition promoting exercise programme increases bone mass in young people with Down syndrome. Developmental Medicine and Child Neurology 2012 June;54(6):552-6. Inappropriate Outcomes
Ref ID: 2563

(1959) Gonzalez-Barcala FJ, Pertega S, Perez-Castro T, Sampedro M, Sanchez-Lastres J, San-Jose-Gonzalez MA, Bamonde L, Garnelo L, Valdes-Cuadrado L, Moure JD, Carreira JM, Lopez-Silvarrey A. Obesity and asthma: an association modified by age. Allergologia et immunopathologia (Madrid) 2013 May;41(3):176-80. Inappropriate Study Design
Ref ID: 5866

(1960) Gonzalez AP, Vasquez-Mendoza G, Garcia-Vela A, Guzman-Ramirez A, Salazar-Torres M, Romero-Gutierrez G. Weight gain in preterm infants following parent-administered Vimala massage: a randomized controlled trial. American Journal of Perinatology 2009 April;26(4):247-52. Subjects less than 2 years old
Ref ID: 784

(1961) Gonzalez M, del Mar BM, Pons A, Llompart I, Tur JA. Inflammatory markers and metabolic syndrome among adolescents. European Journal of Clinical Nutrition 2012 October;66(10):1141-5. Inappropriate Intervention
Ref ID: 5867

(1962) Gonzalez MJ, Jodar E, Munoz M, Diez PA, Guanabens N, Fuster E. [Risk factors for osteoporosis in osteoporotic women followed in Primary Care and in Hospitals. OPINHO-PC study]. [Spanish]. Revista Clinica Espanola 2009 July;209(7):319-24. Study limited to adults
Ref ID: 686

(1963) González Fernández P, Cabrera Rode E, Oti Gil MA. Resistencia a la insulina e historia familiar de diabetes en niños y adolescentes obesos con acantosis nigricans y sin ella. Insulin resistance and family history of diabetes in obese

children and with and without acanthosis nigricans. Revista Cubana de Endocrinología 2011 December;22(3):210-24. Cross-sectional study
Ref ID: 4177

(1964) González Santiesteban A, Sánchez González P, Castillo Núñez J. Incidencia de factores de riesgo cardiovasculares en niños y adolescentes con hipertensión arterial esencial en Artemisa. Incidence of cardiovascular risk factors in children and adolescent presenting with essential high blood pressure in Artemisa province. Revista Cubana de Enfermería 2011 June;27(2):151-8. Cross-sectional study
Ref ID: 4178

(1965) González Sánchez R, Llapur Milián R, Rubio Olivares D. Caracterización de la obesidad en los adolescentes. Revista Cubana de Pediatria 2009 June;81(2). Cross-sectional study
Ref ID: 4180

(1966) González Sánchez R, Llapur Milián R, Jiménez Hernández JM, Llapur González A, Fernández Morales D. Percepción de riesgo de hipertensión arterial infantil en familiares de niños y adolescentes. Relatives's perception of arterial hypertension in children and adolescents. Revista Cubana de Pediatria 2011 March;83(1):65-73. Not an exercise intervention study
Ref ID: 4181

(1967) Goodarzi M, Shier NH, Ogden JA. Epidural versus patient-controlled analgesia with morphine for postoperative pain after orthopaedic procedures in children. Journal of Pediatric Orthopedics 1993;13:663-7. Drug intervention study
Ref ID: 4799

(1968) Goodman A, Koupil I. The effect of school performance upon marriage and long-term reproductive success in 10,000 Swedish males and females born 1915-1929. Evolution and Human Behavior 2010;31(6):425-35. Cohort Study
Ref ID: 5299

(1969) Goodman E, Mcewen BS, Huang B, Dolan LM, Adler NE. Social inequalities in biomarkers of cardiovascular risk in adolescence. Psychosomatic Medicine 2005;67(1):9-15. Cross-sectional study
Ref ID: 5300

(1970) Gopinath B, Baur LA, Hardy LL, Kifley A, Rose KA, Wong TY, Mitchell P. Relationship between a range of sedentary behaviours and blood pressure during early adolescence. Journal of Human Hypertension 2012;26(6):350-6. Cross-sectional study
Ref ID: 5301

(1971) Goran MI. Variation in Total-Energy Expenditure in Humans. Obesity Research 1995;3:59-66. Review article
Ref ID: 5302

(1972) Goran MI. Genetic influences on human energy expenditure and substrate utilization. Behavior Genetics 1997;27(4):389-99. Not an exercise intervention study
Ref ID: 5303

(1973) Goran MI, Nagy TR, Gower BA, Mazariegos A, Solomons N, Hood V, Johnson R. Influence of sex, seasonality, ethnicity, and geographic location on the components of total energy expenditure in young children: implications for energy requirements. American Journal of Clinical Nutrition 1998;68(3):675-82. Cross-sectional study
Ref ID: 5304

(1974) Goran MI. Energy metabolism and obesity. Medical Clinics of North America 2000;84(2):347-+. Review article
Ref ID: 5305

(1975) Goran MI. Metabolic precursors and effects of obesity in children: a decade of progress, 1990-1999. [Review] [84 refs]. American Journal of Clinical Nutrition 2001 February;73(2):158-71. Review article
Ref ID: 1932

(1976) Gordia AP, Quadros TMB, Campos Wd, Petroski ÉL. Domínio físico da qualidade de vida entre adolescentes: Associação com atividade física e sexo. Dominio físico de la calidad de vida entre adolescentes: Asociación con el sexo y la actividad física. Adolescents' physical quality of life: associations with physical activity and sex. Revista de Salud Pública 2009 February;11(1):50-61. Cross-sectional study
Ref ID: 4182

(1977) Gordia AP, Silva RC, Quadros TM, Campos Wd. Variáveis comportamentais e sociodemográficas estão associadas ao domínio psicológico da qualidade de vida de adolescentes. Behavioral and sociodemographic variables are associated with the psychological domain of adolescents' quality of life. Revista Paulista de Pediatria 2010 March;28(1):29-35. Survey or questionnaire
Ref ID: 4183

(1978) Gordon-Larsen P, Boone-Heinonen J, Sidney S, Sternfeld B, Jacobs DR, Jr., Lewis CE. Active commuting and cardiovascular disease risk: the CARDIA study. Archives of Internal Medicine 2009 July 13;169(13):1216-23. Cross-sectional study
Ref ID: 719

(1979) Gordon AM, Forssberg H, Johansson RS, Eliasson AC, Westling G. Development of Human Precision Grip .3. Integration of Visual Size Cues During the Programming of Isometric Forces. Experimental Brain Research 1992;90(2):399-403. Not a randomized controlled trial (RCT)
Ref ID: 5306

(1980) Gordon AM, Charles J, Duff SV. Fingertip forces during object manipulation in children with hemiplegic cerebral palsy. II: Bilateral coordination. Developmental Medicine and Child Neurology 1999;41(3):176-85. Not a randomized controlled trial (RCT)
Ref ID: 5308

(1981) Gordon AM, Duff SV. Fingertip forces during object manipulation in children with hemiplegic cerebral palsy. I: Anticipatory scaling. Developmental Medicine and Child Neurology 1999;41(3):166-75. Not a randomized controlled trial (RCT)
Ref ID: 5307

(1982) Gordon CM, Grace E, Emans SJ, Feldman HA, Goodman E, Becker KA, Rosen CJ, Gundberg CM, LeBoff MS. Effects of oral dehydroepiandrosterone on bone density in young women with anorexia nervosa: a randomized trial. Journal of Clinical Endocrinology and Metabolism 2002 November;87(11):4935-41. Drug intervention study
Ref ID: 1795

(1983) Gordon NF, Myburgh JL, Kruger PE, Kempff PG, Cilliers JF, Moolman J, Grobler HC. Effects of caffeine ingestion on thermoregulatory and myocardial function during endurance performance. South African Medical Journal Suid-Afrikaanse Tydskrif Vir Geneeskunde 1982 October 23;62(18):644-7. Diet Intervention or Supplement Study
Ref ID: 2370

(1984) Goris ML, Hotz B, Thirion JP, Similon P. Factors affecting and computation of myocardial perfusion reference images. Nuclear Medicine Communications 1999 July;20(7):627-35. Review article
Ref ID: 2009

(1985) Gorla JI, Duarte E, Costa LT, Freire F. Crescimento de crianças e adolescentes com Síndrome de Down: uma breve revisão de literatura. Growth of children and adolescents with Down's syndrome: a brief review of the literature. Revista Brasileira de Crescimento e Desenvolvimento Humano 2011 June;13(3):230-7. Review article
Ref ID: 4184

(1986) Gortmaker SL, Cheung LW, Peterson KE, Chomitz G, Cradle JH, Dart H, Fox MK, Bullock RB, Sobol AM, Colditz G, Field AE, Laird N. Impact of a school-based interdisciplinary intervention on diet and physical activity among urban primary school children: eat well and keep moving. Archives of Pediatrics and Adolescent Medicine 1999 September;153(9):975-83. Not a randomized controlled trial (RCT)
Ref ID: 2004

(1987) Gortmaker SL, Peterson K, Wiecha J, Sobol AM, Dixit S, Fox MK, Laird N. Reducing obesity via a school-based interdisciplinary intervention among youth: Planet Health. Archives of Pediatric Adolescent Medicine 1999 April;153(4):409-18. No exercise only group
Ref ID: 411

(1988) Gosline A. When kids go bad. New Scientist 2008;198(2651):38-41. Editorial or letter or comment
Ref ID: 5309

(1989) Gothelf D, Falk B, Singer P, Kairi M, Phillip M, Zigel L, Poraz I, Frishman S, Constantini N, Zalsman G, Weizman A, Apter A. Weight gain associated with increased food intake and low habitual activity levels in male adolescent schizophrenic inpatients treated with olanzapine. American Journal of Psychiatry 2002 June;159(6):1055-7. Not a randomized controlled trial (RCT)
Ref ID: 1821

(1990) Gotthelf SJ, Jubany LL. Prevalencia de factores de riesgo cardiovascular en adolescentes de escuelas públicas y privadasde la ciudad de Salta, año 2009. Prevalence of cardiovascular risk factors in adolescents of public and private schools: Salta City, Argentina, 2009. Archivos Argentinos de Pediatría 2010 October;108(5):418-26. Cross-sectional study
Ref ID: 4186

(1991) Gottschalk M, Danne T, Vlajnic A, Cara JF. Glimepiride versus metformin as monotherapy in pediatric patients with type 2 diabetes: a randomized, single-blind comparative study. Diabetes Care 2007 April;30(4):790-4. Drug intervention study
Ref ID: 1256

(1992) Gourlan M, Sarrazin P, Trouilloud D. Motivational interviewing as a way to promote physical activity in obese adolescents: a randomised-controlled trial using self-determination theory as an explanatory framework. Psychology and Health 2013 November;28(11):1265-86. Inappropriate Comparison Group
Ref ID: 5868

(1993) Gouveia ÉR, Freitas DLd, Maia JA, Beunen GP, Claessens AL, Marques AT, Thomis MA, Almeida SM, Sousa AM, Lefevre JA. Atividade física, aptidão e sobrepeso em crianças e adolescentes: O estudo de crescimento da Madeira. Revista Brasileira de Educação Física e Esporte 2007 June;21(2):95-106. Cross-sectional study
Ref ID: 4187

(1994) Govindan M, Gurm R, Mohan S, Kline-Rogers E, Corriveau N, Goldberg C, Durussel-Weston J, Eagle KA, Jackson EA. Gender differences in physiologic markers and health behaviors associated with childhood obesity. Pediatrics

2013 September;132(3):468-74. Inappropriate Study Design
Ref ID: 5869

(1995) Govindarajan R, Ghosh B, Sathyamoorthy MK, Kodali NS, Raza A, Aronsohn J, Rajpal S, Ramaswamy C, Abadir A. Efficacy of ketorolac in lieu of narcotics in the operative management of laparoscopic surgery for morbid obesity. Surgery for Obesity and Related Diseases 535 June;1(6):530-5. Drug intervention study
Ref ID: 1434

(1996) Goviridji A. The role of carbohydrates in a healthy diet. (Cover story). Nursing Standard 2006 September 27;21(3):56-64. Diet Intervention Study
Ref ID: 3612

(1997) Gowda C, Hadley C, Aiello AE. The Association Between Food Insecurity and Inflammation in the US Adult Population. American Journal of Public Health 2012 August;102(8):1579-86. Study limited to adults
Ref ID: 3818

(1998) Gowers SG, Clark AF, Roberts C, Byford S, Barrett B, Griffiths A, Edwards V, Bryan C, Smethurst N, Rowlands L, Roots P. A randomised controlled multicentre trial of treatments for adolescent anorexia nervosa including assessment of cost-effectiveness and patient acceptability - the TOuCAN trial. Health Technology Assessment 2010;14:1-98. Not an exercise intervention study
Ref ID: 4800

(1999) Goya KM, Siqueira LT, Costa RA, Gallinaro AL, Gonçalves CR, Carvalho JFd. Atividade física regular preserva a função pulmonar em pacientes com espondilite anquilosante sem doença pulmonar prévia. Regular physical activity preserves the lung function in patients with ankylosing spondylitis without previous lung alterations. Revista brasileira de reumatologia 2009 April;49(2). Not an exercise intervention study
Ref ID: 4188

(2000) Gozal D, Thiriet P. Respiratory muscle training in neuromuscular disease: long-term effects on strength and load perception. Medicine and Science in Sports and Exercise 1999;31:1522-7. Not an exercise intervention study
Ref ID: 4801

(2001) Gómez LF, Lucumí DI, Parra DC, Lobelo F. Niveles de urbanización, uso de televisión y video-juegos en niños colombianos: Posibles implicaciones en salud pública. Possible public health implications regarding associations between the degree of urbanisation and electronic media exposure amongst Colombian children. Revista de Salud Pública 2008 October;10(4):505-16. Cross-sectional study
Ref ID: 4189

(2002) Grace ND, Waghorn GC. Impact of iodine supplementation of dairy cows on milk production and iodine concentrations in milk. New Zealand Veterinary Journal 2005;53(1):10-3. Animal study
Ref ID: 5310

(2003) Gracey M. The pediatrician's role in the twenty-first century. Acta Paediatrica Japonica 1998;40(5):393-9. Review article
Ref ID: 5311

(2004) Gracia-Marco L, Ortega FB, Jimenez-Pavon D, Rodriguez G, Castillo MJ, Vicente-Rodriguez G, Moreno LA. Adiposity and bone health in Spanish adolescents. The HELENA study. Osteoporosis International 2012 March;23(3):937-47. Cross-sectional study
Ref ID: 2564

(2005) Graf C, Dordel S, Tokarski W, Predel HG. [Overweight and obesity in childhood and adolescence. Is prevention possible?]. [German]. Herz 2006 September;31(6):507-13. Review article
Ref ID: 1340

(2006) Graf C, Koch B, Bjarnason-Wehrens B, Sreeram N, Brockmeier K, Tokarski W, Dordel S, Predel HG. Who benefits from intervention in, as opposed to screening of, overweight and obese children? Cardiology in the Young 2006 October;16(5):474-80. No exercise only group
Ref ID: 268

(2007) Graf C, Koch B, Falkowski G, Jouck S, Christ H, Staudenmaier K, Tokarski W, Gerber A, Predel HG, Dordel S. School-based prevention: effects on obesity and physical performance after 4 years. Journal of Sports Sciences 2008 August;26(10):987-94. No exercise only group
Ref ID: 172

(2008) Graf C, Dordel S. [The CHILT I project (Children's Health Interventional Trial). A multicomponent intervention to prevent physical inactivity and overweight in primary schools]. [German]. Bundesgesundheitsblatt, Gesundheitsforschung, Gesundheitsschutz 2011 March;54(3):313-21. Not All Participants were Overweight and/or Obese
Ref ID: 2565

(2009) Graff GR, Maguiness K, McNamara J, Morton R, Boyd D, Beckmann K, Bennett D. Efficacy and tolerability of a new formulation of pancrelipase delayed-release capsules in children aged 7 to 11 years with exocrine pancreatic insufficiency and cystic fibrosis: a multicenter, randomized, double-blind, placebo-controlled, two-period crossover, superiority study. Clinical Therapeutics 2010;32:89-103. Drug intervention study
Ref ID: 4802

(2010) Graham D, Appleton S, Rush E, McLennan S, Reed P, Simmons D. Increasing activity and improving nutrition through a schools-based programme: Project Energize. 1. Design, programme, randomisation and evaluation methodology. Public Health Nutrition 2008 October;11(10):1076-84. Description of study from review or magazine or etc. (not the actual study)
Ref ID: 893

(2011) Graham DJ, Schneider M, Cooper DM. Television viewing: moderator or mediator of an adolescent physical activity intervention? American Journal of Health Promotion 2008 November;23(2):88-91. Not a randomized controlled trial (RCT)
Ref ID: 861

(2012) Graham DJ, Bauer KW, Friend S, Barr-Anderson DJ, Nuemark-Sztainer D. Personal, behavioral, and socio-environmental correlates of physical activity among adolescent girls: cross-sectional and longitudinal associations. Journal of Physical Activity and Health 2014 January;11(1):51-61. Inappropriate Study Design
Ref ID: 5870

(2013) Graham HK, Boyd R, Carlin JB, Dobson F, Lowe K, Nattrass G, Thomason P, Wolfe R, Reddihough D. Does botulinum toxin a combined with bracing prevent hip displacement in children with cerebral palsy and "hips at risk"? A randomized, controlled trial. The Journal of Bone and Joint Surgery American volume 2008;90:23-33. Drug intervention study
Ref ID: 4803

(2014) Graham MR, Baker JS, Evans P, Kicman A, Cowan D, Hullin D, Davies B. Short-term recombinant human growth hormone administration improves respiratory function in abstinent anabolic-androgenic steroid users. Growth Hormone and Igf Research 2007;17(4):328-35. Drug intervention study
Ref ID: 5312

(2015) Gramsbergen A, Mulder EJH. The influence of betamethasone and dexamethasone on motor development in young rats. Pediatric Research 1998;44(1):105-10. Animal study
Ref ID: 5313

(2016) Granacher U, Goesele A, Roggo K, Wischer T, Fischer S, Zuerny C, Gollhofer A, Kriemler S. Effects and mechanisms of strength training in children. International Journal of Sports Medicine 2011;32:357-64. Not All Participants were Overweight and/or Obese
Ref ID: 4804

(2017) Granacher U, Muehlbauer T, Doerflinger B, Strohmeier R, Gollhofer A. Promoting strength and balance in adolescents during physical education: effects of a short-term resistance training. Journal of Strength and Conditioning

Research 2011 April;25(4):940-9. Not All Participants were Overweight and/or Obese
Ref ID: 2566

(2018) Granger DA, Whalen CK, Henker B. Malleability of social impressions of hyperactive children. Journal of Abnormal Child Psychology 1993;21:631-47. Observational study
Ref ID: 4805

(2019) Grant S, Craig I, Wilson J, Aitchison T. The relationship between 3 km running performance and selected physiological variables. Journal of Sports Sciences 1997 August;15(4):403-10. Cross-sectional study
Ref ID: 2125

(2020) Grant S, Davidson W, Aitchison T, Wilson J. A comparison of physiological responses and rating of perceived exertion between high-impact and low-impact aerobic dance sessions. European Journal of Applied Physiology and Occupational Physiology 1998 September;78(4):324-32. Study not limited to children and adolescents
Ref ID: 421

(2021) Grasso R, Ivanenko YP, Zago M, Molinari M, Scivoletto G, Lacquaniti F. Recovery of forward stepping in spinal cord injured patients does not transfer to untrained backward stepping. Experimental Brain Research 2004 August;157(3):377-82. Not a randomized controlled trial (RCT)
Ref ID: 1609

(2022) Graves LE, Ridgers ND, Atkinson G, Stratton G. The effect of active video gaming on children's physical activity, behavior preferences and body composition. Pediatric Exercise Science 2010 November;22(4):535-46. Not All Participants were Overweight and/or Obese
Ref ID: 2567

(2023) Gravholt CH, Jorgensen JOL, Christiansen JS, Naeraa RW, Brixen K, Kastrup KW, Mosekilde L. Short-Term Growth Hormone Treatment in Girls With Turner Syndrome Decreases Fat Mass and Insulin Sensitivity: A Randomized, Double-Blind, Placebo-Controlled, Crossover Study. Pediatrics 2002 November;110(5):889. Drug intervention study
Ref ID: 3613

(2024) Gray SJ, Wiebusch B, Akol HA. Cross-sectional growth of pastoralist Karimojong and Turkana children. American Journal of Physical Anthropology 2004;125(2):193-202. Cross-sectional study
Ref ID: 5314

(2025) Graziadio C, Bernardi P, Rosa RFM, Zen PRG, Paskulin GA. Type 1 diabetes in a patient with Ellis-van Creveld syndrome. Diabetes tipo 1 em um paciente com a síndrome de Ellis-van Creveld. São Paulo Medical Journal

2012;130(1):53-6. Case-Control / Case Study
Ref ID: 4190

(2026) Green DJ, Swart A, Exterkate A, Naylor LH, Black MA, Cable NT, Thijssen DHJ. Impact of age, sex and exercise on brachial and popliteal artery remodelling in humans. Atherosclerosis 2010;210(2):525-30. Cross-sectional study
Ref ID: 5315

(2027) Greenberg RS, Ariza AJ, Binns HJ. Activity and dietary habits of mothers and children: close ties. Clinical Pediatrics 2010 November;49(11):1026-32. Cross-sectional study
Ref ID: 2568

(2028) Greene DA, Wiebe PN, Naughton GA. Influence of drop-landing exercises on bone geometry and biomechanical properties in prepubertal girls: a randomized controlled study. Calcified Tissue International 2009 August;85(2):94-103. Not All Participants were Overweight and/or Obese
Ref ID: 694

(2029) Greene GW, White AA, Hoerr SL, Lohse B, Schembre SM, Riebe D, Patterson J, Kattelmann KK, Shoff S, Horacek T, Blissmer B, Phillips BW. Impact of an online healthful eating and physical activity program for college students. American Journal of Health Promotion 2012 November;27(2):e47-e58. Inappropriate Population
Ref ID: 5871

(2030) Greening L, Harrell KT, Low AK, Fielder CE. Efficacy of a school-based childhood obesity intervention program in a rural southern community: TEAM Mississippi Project. Obesity 2011 June;19(6):1213-9. Diet & Exercise intervention
Ref ID: 2569

(2031) Greenway FL, Dunayevich E, Tollefson G, Erickson J, Guttadauria M, Fujioka K, Cowley MA. Comparison of combined bupropion and naltrexone therapy for obesity with monotherapy and placebo. Journal of Clinical Endocrinology and Metabolism 2009 December;94(12):4898-906. Study limited to adults, No exercise only group
Ref ID: 87

(2032) Grego LG, Luiz MH, Gonçalves A, Padovani CR. Aptidão física e saúde de praticantes de dança e de escolares. Physical condition and health of practicing dancers and school-girls. Salusvita 2006;25(2):81-112. Not an exercise intervention study
Ref ID: 4191

(2033) Gregory J, Robling M, Bennert K, Channon S, Cohen D, Crowne E, Hambly H, Hawthorne K, Hood K, Longo M, Lowes L, McNamara R, Pickles T, Playle R,

Rollnick S, Thomas JE. Development and evaluation by a cluster randomised trial of a psychosocial intervention in children and teenagers experiencing diabetes: the DEPICTED study. Health Technology Assessment 2011;15:1-202. Not an exercise intervention study
Ref ID: 4806

(2034) Gregson CL, Steel SA, O'Rourke KP, Allan K, Ayuk J, Bhalla A, Clunie G, Crabtree N, Fogelman I, Goodby A, Langman CM, Linton S, Marriott E, McCloskey E, Moss KE, Palferman T, Panthakalam S, Poole KE, Stone MD, Turton J, Wallis D, Warburton S, Wass J, Duncan EL, Brown MA et al. 'Sink or swim': an evaluation of the clinical characteristics of individuals with high bone mass. Osteoporosis International 2012 February;23(2):643-54. Study limited to adults
Ref ID: 2570

(2035) Grey M, Boland EA, Davidson M, Yu C, Sullivan BS, Tamborlane WV. Short-term effects of coping skills training as adjunct to intensive therapy in adolescents. Diabetes Care 1998;21:902-8. Behavior Modification Intervention
Ref ID: 4807

(2036) Grey M, Boland EA, Davidson M, Li J, Tamborlane WV. Coping skills training for youth with diabetes mellitus has long-lasting effects on metabolic control and quality of life. The Journal of Pediatrics 2000;137:107-13. Behavior Modification Intervention
Ref ID: 4808

(2037) Grey M, Berry D, Davidson M, Galasso P, Gustafson E, Melkus G. Preliminary testing of a program to prevent type 2 diabetes among high-risk youth. Journal of School Health 2004 January;74(1):10-5. No exercise only group, No comparative control group
Ref ID: 339

(2038) Grey M, Jaser SS, Holl MG, Jefferson V, Dziura J, Northrup V. A multifaceted school-based intervention to reduce risk for type 2 diabetes in at-risk youth. Preventive Medicine 2009;49:122-8. Educational intervention
Ref ID: 4809

(2039) Grieco LA, Jowers EM, Bartholomew JB. Physically active academic lessons and time on task: the moderating effect of body mass index. Medicine and Science in Sports and Exercise 2009 October;41(10):1921-6. Study less than 4 weeks
Ref ID: 610

(2040) Grier TD, Lloyd LK, Walker JL, Murray TD. Metabolic cost of aerobic dance bench stepping at varying cadences and bench heights. Journal of Strength and Conditioning Research 2002 May;16(2):242-9. Study limited to adults
Ref ID: 1834

(2041) Griese M, Wiesener A, Lottspeich F, von Bredow C. Limited proteolysis of surfactant protein D causes a loss of its calcium-dependent lectin functions. Biochimica et Biophysica Acta-Molecular Basis of Disease 2003;1638(2):157-63. Drug intervention study
Ref ID: 5316

(2042) Griffin HJ, Cheng HL, O'Connor HT, Rooney KB, Petocz P, Steinbeck KS. Higher protein diet for weight management in young overweight women: a 12-month randomized controlled trial. Diabetes, Obesity and Metabolism 2013 June;15(6):572-5. Inappropriate Population
Ref ID: 5872

(2043) Grindstaff PD, Kreider R, Bishop R, Wilson M, Wood L, Alexander C, Almada A. Effects of creatine supplementation on repetitive sprint performance and body composition in competitive swimmers. International Journal of Sport Nutrition 1997 December;7(4):330-46. Diet Intervention or Supplement Study
Ref ID: 2116

(2044) Grinpoon S, Mulligan K. Weight Loss and Wasting in Patients Infected with Human Immunodeficiency Virus. Clinical Infectious Diseases 2003 April 2;36:S69. Review article
Ref ID: 3614

(2045) Grivell R, Dodd J, Robinson J. The prevention and treatment of intrauterine growth restriction. Best Practice and Research Clinical Obstetrics and Gynaecology 2009;23(6):795-807. Review article
Ref ID: 3234

(2046) Griz LHM, Viégas M, Barros M, Griz AL, Freese E, Bandeira F. Prevalence of central obesity in a large sample of adolescents from public schools in Recife, Brazil. Prevalência de obesidade central em grande amostra de adolescentes de escolas públicas em Recife, Brasil. Arquivos Brasileiros de Endocrinologia and Metabologia 2010 October;54(7):607-11. Cross-sectional study
Ref ID: 4192

(2047) Groeneveld IF, Proper KI, van der Beek AJ, van DC, van MW. Design of a RCT evaluating the (cost-) effectiveness of a lifestyle intervention for male construction workers at risk for cardiovascular disease: the health under construction study. BMC Public Health 2008;8:1. Description of study from review or magazine or etc. (not the actual study)
Ref ID: 992

(2048) Groeneveld IF, Proper KI, van der Beek AJ, Hildebrandt VH, van MW. Short and long term effects of a lifestyle intervention for construction workers at risk for cardiovascular disease: a randomized controlled trial. BMC Public Health 2011;11:836. Study limited to adults
Ref ID: 2571

(2049) Grossman P, Spoerle M, Wilhelm FH. Reliability of respiratory tidal volume estimation by means of ambulatory inductive plethysmography. Biomedical Sciences Instrumentation 2006;42:193-8. No control group (NC)
Ref ID: 1380

(2050) Grotenhermen F. Pharmacokinetics and Pharmacodynamics of Cannabinoids. Clinical Pharmacokinetics 2003 February 15;42(4):327-60. Review article
Ref ID: 3615

(2051) Grund A, Dilba B, Forberger K, Krause H, Siewers M, Rieckert H, Muller MJ. Relationships between physical activity, physical fitness, muscle strength and nutritional state in 5- to 11-year-old children. European Journal of Applied Physiology 2000 August;82(5-6):425-38. Cross-sectional study
Ref ID: 1960

(2052) Grunewald TGP, von Luettichau I, Welsch U, Dorr HG, Hopner F, Kovacs K, Burdach S, Rabl W. First report of ectopic ACTH syndrome and PTHrP-induced hypercalcemia due to a hepatoblastoma in a child. European Journal of Endocrinology 2010;162(4):813-8. Case-Control / Case Study
Ref ID: 5317

(2053) Grunseit AC, Taylor AJ, Hardy LL, King L. Composite measures quantify households' obesogenic potential and adolescents' risk behaviors. Pediatrics 2011 August;128(2):e308-e316. Survey or questionnaire
Ref ID: 2572

(2054) Grydeland M, Bergh IH, Bjelland M, Lien N, Andersen LF, Ommundsen Y, Klepp KI, Anderssen SA. Intervention effects on physical activity: the HEIA study - a cluster randomized controlled trial. International Journal of Behavioral Nutrition and Physical Activity 2013;10:17. Inappropriate Population
Ref ID: 5873

(2055) Grzanna MW, Ownby SL, Heinecke LF, Au AY, Frondoza CG. Inhibition of Cytokine Expression and Prostaglandin E2 Production in Monocyte/Macrophage-Like Cells by Avocado/Soybean Unsaponifiables and Chondroitin Sulfate. Journal of Complementary and Integrative Medicine 2010 January;7(1):1-16. Not an exercise intervention study
Ref ID: 3888

(2056) Gschwend S, Ebert W, Schultze-Mosgau M, Breuer J. Pharmacokinetics and Imaging Properties of Gd-EOB-DTPA in Patients With Hepatic and Renal Impairment. Investigative Radiology 2011;46(9):556-66. Not an exercise intervention study
Ref ID: 5318

(2057) Guagliano JM, Rosenkranz RR. Physical activity promotion and obesity prevention in Girl Scouts: Scouting Nutrition and Activity Program+. Pediatrics

International 2012 December;54(6):810-5. Inappropriate Intervention
Ref ID: 5874

(2058) Guedes DP, Guedes JERP. Associaçäo entre variáveis do aspecto morfológico e desempenho motor em crianças e adolescentes. Revista Paulo de Educação Física 1996 December;10(2):99-112. Cross-sectional study
Ref ID: 4193

(2059) Guedes DP, Martini FAN, Borges MB, Bernardelli Júnior R. Crescimento físico de escolares: Comparação com a referência do Centers for Disease Control and Prevention mediante o método LMS. Physical growth of schoolchildren: A comparison with the Centers for Disease Control and Prevention reference using the LMS method. Revista Brasileira de Saúde Materno Infantil 2009 March;9(1):39-48. Cross-sectional study
Ref ID: 4194

(2060) Guedes DP, Miranda Neto JT, Almeida MJ, Silva AJRM. Impacto de fatores sociodemográficos e comportamentais na prevalência de sobrepeso e obesidade de escolares. Revista Brasileira de Cineantropometria e Desempenho Humano 2010 August;12(4). Cross-sectional study
Ref ID: 4195

(2061) Guedes DP, Rocha GD, Silva AJRM, Carvalhal IM, Coelho EM. Effects of social and environmental determinants on overweight and obesity among Brazilian schoolchildren from a developing region. Efectos de los determinantes sociales y ambientales sobre la obesidad y el sobrepeso en escolares de una región en desarrollo del Brasil. Revista Panamericana de Salud Pública 2011 October;30(4):295-302. Survey or questionnaire
Ref ID: 4196

(2062) Guedes DP, Mendes RR. Crescimento físico e estado nutricional de escolares do Vale do Jequitinhonha, Minas Gerais, Brasil. Physical growth and nutritional status of schoolchildren from Valley of the Jequitinhonha, Minas Gerais, Brazil. Revista Brasileira de Cineantropometria e Desempenho Humano 2012;14(4):363-76. Cross-sectional study
Ref ID: 4197

(2063) Guedes NG, Moreira RP, Cavalcante TF, Araujo TLd, Ximenes LB. Atividade física de escolares: Análise segundo o modelo teórico de promoção da saúde de Pender. La actividad física de escolares: Análisis según el modelo teórico de promoción de la salud de Pender. Revista da Escola de Enfermagem da USP 2009 December;43(4):744-80. Cross-sectional study
Ref ID: 4198

(2064) Guelinckx I, Devlieger R, Mullie P, Vansant G. Effect of lifestyle intervention on dietary habits, physical activity, and gestational weight gain in obese pregnant women: a randomized controlled trial. American Journal of Clinical Nutrition

2010 February;91(2):373-80. Study limited to adults
Ref ID: 75

(2065) Guerra-Juárez R, Gallegos EC, Cerda-Flores RM. Lifestyle changes in descendants of parents with diabetes type 2. Cambio en los patrones de vida en descendientes de progenitores con diabetes mellitus tipo 2 del noreste de Mexico. Mudança em os padrões de vida em descendentes de progenitores com diabetes mellitus tipo 2 do nordeste do México. Revista Latino-Americana de Enfermagem 2007 October;15(5):909-13. Not an exercise intervention study
Ref ID: 588

(2066) Guerra A, Rego C, Laires MJ, Castro EMB, Silva D, Monteiro C, Silva Z, Lebre E, Bicho M. Lipid profile and redox status in high performance rhythmic female teenagers gymnasts. Journal of Sports Medicine and Physical Fitness 2001;41(4):505-12. Not a randomized controlled trial (RCT)
Ref ID: 5319

(2067) Guerra PH, Nobre MR, Silveira JA, Taddei JA. The effect of school-based physical activity interventions on body mass index: a meta-analysis of randomized trials. Clinics (Sao Paulo) 2013 September;68(9):1263-73. Inappropriate Study Design
Ref ID: 5875

(2068) Guest G, BÃ©rard E, Crosnier H, Chevallier T, Rappaport R, Broyer M. Effects of growth hormone in short children after renal transplantation. Pediatric Nephrology 1998 August;12(6):437-46. Drug intervention study
Ref ID: 3616

(2069) Guglielmi FW, Panella C, Buda A, Budillon G, Caregaro L, Clerici C, Conte D, Federico A, Gasbarrini G, Guglielmi A, Loguercio C, Losco A, Martines D, Mazzuoli S, Merli M, Mingrone G, Morelli A, Nardone G, Zoli G, Francavilla A. Nutritional state and energy balance in cirrhotic patients with or without hypermetabolism. Multicentre prospective study by the 'Nutritional Problems in Gastroenterology' Section of the Italian Society of Gastroenterology (SIGE). Digestive and Liver Disease 2005 September;37(9):681-8. Prospective Study
Ref ID: 1483

(2070) Guimarães ACdA, Pinto EC, Soares A, Azevedo SFd, Machado Z. Atividade física de mães e de escolares com excesso de peso e obesidade. Physical activity of mothers and schoolchildren with weight excess and obesity. Revista Brasileira de Ciências da Saúde 2012 May;16(2). Cross-sectional study
Ref ID: 4199

(2071) Guimarey LM, Piedrabuena AE, Barros Fillho AA. Treinamento e padronizacao do pessoal para a realizacao de um estudo antropometrico em escolares;

Standardization and training of personnel for the realization of a study on school antropometrico. Archivos Latinoamericanos de Nutrición 1981;31(2):303-13. Not an exercise intervention study
Ref ID: 4200

(2072) Guizar-Mendoza JM, Amador-Licona N, Flores-Martinez SE, Lopez-Cardona MG, Ahuatzin-Tremary R, Sanchez-Corona J. Association analysis of the Gln223Arg polymorphism in the human leptin receptor gene, and traits related to obesity in Mexican adolescents. Journal of Human Hypertension 2005;19(5):341-6. Cross-sectional study
Ref ID: 5320

(2073) Gullestad L, Birkeland K, Bjonerheim R, Djoseland O, Trygstad O, Simonsen S. Exercise capacity and hormonal response in adults with childhood onset growth hormone deficiency during long-term somatropin treatment. Growth Hormone and Igf Research 1998 October;8(5):377-84. Study limited to adults
Ref ID: 2111

(2074) Gulmans VA, de MK, Brackel HJ, Faber JA, Berger R, Helders PJ. Outpatient exercise training in children with cystic fibrosis: physiological effects, perceived competence, and acceptability. Pediatric Pulmonology 1999 July;28(1):39-46. No control group (NC)
Ref ID: 2011

(2075) Gunderson EP, Lewis CE, Murtaugh MA, Quesenberry CP, Smith WD, Sidney S. Long-term plasma lipid changes associated with a first birth: the Coronary Artery Risk Development in Young Adults study. American Journal of Epidemiology 2004 June 1;159(11):1028-39. Prospective Study
Ref ID: 1622

(2076) Gunderson EP, Quesenberry CP, Jr., Lewis CE, Tsai AL, Sternfeld B, Smith WD, Sidney S. Development of overweight associated with childbearing depends on smoking habit: The Coronary Artery Risk Development in Young Adults (CARDIA) Study. Obesity Research 2004 December;12(12):2041-53. Prospective Study
Ref ID: 1560

(2077) Gunderson EP, Lewis CE, Tsai AL, Chiang V, Carnethon M, Quesenberry CP, Jr., Sidney S. A 20-year prospective study of childbearing and incidence of diabetes in young women, controlling for glycemia before conception: the Coronary Artery Risk Development in Young Adults (CARDIA) Study. Diabetes 2007 December;56(12):2990-6. Prospective Study
Ref ID: 1133

(2078) Gunderson EP, Jacobs DR, Jr., Chiang V, Lewis CE, Feng J, Quesenberry CP, Jr., Sidney S. Duration of lactation and incidence of the metabolic syndrome in women of reproductive age according to gestational diabetes mellitus status: a

20-Year prospective study in CARDIA (Coronary Artery Risk Development in Young Adults). Diabetes 2010 February;59(2):495-504. Prospective Study
Ref ID: 604

(2079) Gunter K, Baxter-Jones AD, Mirwald RL, Almstedt H, Fuchs RK, Durski S, Snow C. Impact exercise increases BMC during growth: an 8-year longitudinal study. Journal of Bone and Mineral Research 2008;23:986-93. Not a randomized controlled trial (RCT)
Ref ID: 4810

(2080) Guo G, North KE, Gorden-Larsen P, Bulik CM, Choi S. Body mass, DRD4, physical activity, sedentary behavior, and family socioeconomic status: the add health study. Obesity 2007 May;15(5):1199-206. Longitudinal Study
Ref ID: 1233

(2081) Gupta R. Lifestyle risk factors and coronary heart disease prevalence in Indian men. Journal of the Association of Physicians of India 1996 October;44(10):689-93. Study limited to adults
Ref ID: 2149

(2082) Gupta V, Lee M. Growth hormone in chronic renal disease. Indian Journal of Endocrinology and Metabolism 2012 March;16(2):195-203. Review article
Ref ID: 3617

(2083) Gurd B, Klentrou P. Physical and pubertal development in young male gymnasts. Journal of Applied Physiology 2003 September;95(3):1011-5. Survey or questionnaire
Ref ID: 1725

(2084) Gurjão ALD, Cyrino ES, Caldeira LFS, Nakamura FY, Oliveira ARd, Salvador EP, Dias RMR. Variação da força muscular em testes repetitivos de 1-RM em crianças pré-púberes. Revista Brasileira de Medicina do Esporte 2005 December;11(6):319-24. Not a randomized controlled trial (RCT)
Ref ID: 629

(2085) Gutiérrez R, Correa R. Operaciones discursivas y contextos argumentativos: sobre la comprensión del fenómeno físico de rebotar. Discursive operations and argumentative contexts:Understanding the physical phenomenon of bouncing. Operações discursivas e contextos argumentativos: Compreensão do fenômeno físico de rebote. Acta colomb psicol 2009 June;12(2):85-95. Not an exercise intervention study
Ref ID: 4201

(2086) Gutin B, Cucuzzo N, Islam S, Smith C, Stachura ME. Physical training, lifestyle education, and coronary risk factors in obese girls. Medicine and Science in Sports and Exercise 1996 January;28(1):19-23. No non-intervention control group
Ref ID: 449

(2087) Gutin B, Owens S, Slavens G, Riggs S, Treiber F. Effect of physical training on heart-period variability in obese children. Journal of Pediatrics 1997 June;130(6):938-43. Met criteria but could not retrieve data
Ref ID: 424

(2088) Gutin B, Riggs S, Ferguson M, Owens S. Description and process evaluation of a physical training program for obese children. Research Quarterly for Exercise and Sport 1999 March;70(1):65-9. Description of study from review or magazine or etc. (not the actual study)
Ref ID: 414

(2089) Gutin B, Ramsey L, Barbeau P, Cannady W, Ferguson M, Litaker M, Owens S. Plasma leptin concentrations in obese children: changes during 4-mo periods with and without physical training. American Journal of Clinical Nutrition 1999 March;69(3):388-94. Primary outcome(s) not assessed, Same subjects as another study already included
Ref ID: 416

(2090) Gutin B, Owens S, Okuyama T, Riggs S, Ferguson M, Litaker M. Effect of physical training and its cessation on percent fat and bone density of children with obesity. Obesity Research 1999 March;7(2):208-14. Met criteria but could not retrieve data
Ref ID: 413

(2091) Gutin B, Barbeau P, Litaker MS, Ferguson M, Owens S. Heart rate variability in obese children: relations to total body and visceral adiposity, and changes with physical training and detraining. Obesity Research 2000 January;8(1):12-9. Same subjects as another study already included
Ref ID: 1988

(2092) Gutin B, Barbeau P, Owens S, Lemmon CR, Bauman M, Allison J, Kang HS, Litaker MS. Effects of exercise intensity on cardiovascular fitness, total body composition, and visceral adiposity of obese adolescents. American Journal of Clinical Nutrition 2002 May;75(5):818-26. No exercise only group, No comparative control group
Ref ID: 383

(2093) Gutin B, Yin Z, Humphries MC, Barbeau P. Relations of moderate and vigorous physical activity to fitness and fatness in adolescents. American Journal of Clinical Nutrition 2005 April;81(4):746-50. Cross-sectional study
Ref ID: 1527

(2094) Gutin B. Child obesity can be reduced with vigorous activity rather than restriction of energy intake. Obesity 2008 October;16(10):2193-6. Editorial or letter or comment
Ref ID: 875

(2095) Gutin B, Yin Z, Johnson M, Barbeau P. Preliminary findings of the effect of a 3-year after-school physical activity intervention on fitness and body fat: the Medical College of Georgia Fitkid Project. International Journal of Pediatric Obesity 2008;3 Suppl 1:3-9.:3-9. No exercise only group
Ref ID: 195

(2096) Guzzetta A, Baldini S, Bancale A, Baroncelli L, Ciucci F, Ghirri P, Putignano E, Sale A, Viegi A, Berardi N, Boldrini A, Cioni G, Maffei L. Massage accelerates brain development and the maturation of visual function. Journal of Neuroscience 2009 May 6;29(18):6042-51. Subjects less than 2 years old
Ref ID: 758

(2097) Haack A, Garbi Novaes M. Multidisciplinary care in cystic fibrosis; a clinical-nutrition review. Nutricion Hospitalaria 2012 March;27(2):362-71. Review article
Ref ID: 3618

(2098) Haack A, Carvalho KMB. Risco metabólico e estado nutricional de adolescentes atendidos em unidade básica de saúde. Comunicação em Ciências da Saúde 2009 September;20(3):203-10. Cross-sectional study
Ref ID: 4202

(2099) Haakstad LA, Bo K. Exercise in pregnant women and birth weight: a randomized controlled trial. BMC Pregnancy and Childbirth 2011;11:66. Study limited to adults
Ref ID: 2573

(2100) Haas AN, Plaza MR, Rose EHD. Estudo antropométrico comparativo entre meninas espanholas e brasileiras praticantes de dança. Revista Brasileira de Cineantropometria e Desempenho Humano 2000;2(1). Cross-sectional study
Ref ID: 4203

(2101) Haas JD, Martinez EJ, Murdoch S, Conlisk E, Rivera JA, Martorell R. Nutritional supplementation during the preschool years and physical work capacity in adolescent and young adult Guatemalans. Journal of Nutrition 1995;125:1078S-89S. Diet Intervention or Supplement Study
Ref ID: 4811

(2102) Habte HH, de Beer C, Lotz ZE, Tyler MG, Kahn D, Mall AS. Inhibition of human immunodeficiency virus type 1 activity by purified human breast milk mucin (MUC1) in an inhibition assay. Neonatology 2008;93(3):162-70. Not an exercise intervention study
Ref ID: 5321

(2103) Haerens L, Deforche B, Maes L, Stevens V, Cardon G, De B, I. Body mass effects of a physical activity and healthy food intervention in middle schools. Obesity 2006 May;14(5):847-54. Multiple interventions
Ref ID: 1371

(2104) Haerens L, Deforche B, Maes L, Cardon G, Stevens V, De B, I. Evaluation of a 2-year physical activity and healthy eating intervention in middle school children. Health Education Research 2006 December;21(6):911-21. Multiple interventions
Ref ID: 1324

(2105) Haerens L, De B, I, Maes L, Vereecken C, Brug J, Deforche B. The effects of a middle-school healthy eating intervention on adolescents' fat and fruit intake and soft drinks consumption. Public Health Nutrition 2007;10:443-9. Multiple interventions
Ref ID: 4812

(2106) Hafezi R, Mirmohammadi SJ, Mehrparvar AH, Akbari H, Akbari H. An Analysis of Anthropometric Data on Iranian Primary School Children. Iranian Journal of Public Health 2010;39(4):78-86. Cross-sectional study
Ref ID: 5322

(2107) Hagen EH, Barrett HC, Price ME. Do human parents face a quantity-quality tradeoff?: Evidence from a Shuar community. American Journal of Physical Anthropology 2006;130(3):405-18. Off topic
Ref ID: 5323

(2108) Hager ER, Witherspoon DO, Gormley C, Latta LW, Pepper MR, Black MM. The perceived and built environment surrounding urban schools and physical activity among adolescent girls. Annals of Behavioral Medicine 2013 February;45 Suppl 1:S68-S75. Inappropriate Study Design
Ref ID: 5876

(2109) Hagerman R, Berry-Kravis E, Hessl D, Coffey S, Schneider A, Nguyen D, Hervey C, Hutchison J, Snape M. Trial of fenobam, an mGluR5 antagonist, in adults with Fragile X Syndrome. Journal of Intellectual Disability Research 2008 October;52(10):814. Drug intervention study
Ref ID: 3819

(2110) Haghdoost AA, Poorranjbar M. The interaction between physical activity and fasting on the serum lipid profile during Ramadan. Singapore Medical Journal 2009 September;50(9):897-901. Inappropriate Outcomes
Ref ID: 668

(2111) Hainer V, Kunesova M, Bellisle F, Hill M, Braunerova R, Wagenknecht M. Psychobehavioral and nutritional predictors of weight loss in obese women treated with sibutramine. International Journal of Obesity (London) 2005 February;29(2):208-16. Study limited to adults, Drug intervention study
Ref ID: 319

(2112) Haire-Joshu D, Nanney MS, Elliott M, Davey C, Caito N, Loman D, Brownson RC, Kreuter MW. The use of mentoring programs to improve energy balance behaviors in high-risk children. Obesity (Silver Spring) 2010 February;18 Suppl

1:S75-83.:S75-S83. No exercise only group
Ref ID: 65

(2113) Hakanen M, Lagstrom H, Kaitosaari T, Niinikoski H, Nanto-Salonen K, Jokinen E, Sillanmaki L, Viikari J, Ronnemaa T, Simell O. Development of overweight in an atherosclerosis prevention trial starting in early childhood. The STRIP study. International Journal of Obesity (London) 2006 April;30(4):618-26. No exercise only group
Ref ID: 288

(2114) Hakanen M, Raitakari OT, Lehtimaki T, Peltonen N, Pahkala K, Sillanmaki L, Lagstrom H, Viikari J, Simell O, Ronnemaa T. FTO genotype is associated with body mass index after the age of seven years but not with energy intake or leisure-time physical activity. Journal of Clinical Endocrinology and Metabolism 2009 April;94(4):1281-7. Lifestyle Intervention
Ref ID: 772

(2115) Hale DE. Type 2 diabetes and diabetes risk factors in children and adolescents. Clin Cornerstone 2004;6(2):17-27. Review article
Ref ID: 3235

(2116) Halimi S. [Benefits of blood glucose self-monitoring in the management of insulin-dependent (IDDM) and non-insulin-dependent diabetes (NIDDM). Analysis of the literature: mixed results]. [Review] [28 refs] [French]. Diabetes and Metabolism 1998 November;24:Suppl-41. Review article
Ref ID: 2070

(2117) Hall EH, Crowe SE. Environmental and Lifestyle Influences on Disorders of the Large and Small Intestine: Implications for Treatment. Digestive Diseases 2011;29(2):249-54. Off topic
Ref ID: 5324

(2118) Hallal PC, Bertoldi AD, Gonçalves H, Victora CG. Prevalência de sedentarismo e fatores associados em adolescentes de 10-12 anos de idade. Cadernos de Saúde Pública 2006 June;22(6):1277-87. Prevalence study
Ref ID: 4204

(2119) Hallal PC, Knuth AG, Cruz DKA, Mendes MI, Malta DC. Prática de atividade física em adolescentes brasileiros. Physical activity practice among brazilian adolescents. Ciência and Saúde Coletiva 2010 October;15(supl.2):3035-42. Cross-sectional study
Ref ID: 4205

(2120) Halpern A. Tratamento medicamentoso da obesidade na infância e na adolescência. In: Fisberg M, editor. Obesidade na infância e adolescência.Säo Paulo: Fundo Editorial Byk; 1995. p. 149-55.
Drug intervention study
Ref ID: 803

(2121) Halsall DJ, Luan J, Saker P, Huxtable S, Farooqi IS, Keogh J, Wareham NJ, O'Rahilly S. Uncoupling protein 3 genetic variants in human obesity: the c-55t promoter polymorphism is negatively correlated with body mass index in a UK Caucasian population. International Journal of Obesity 2001;25(4):472-7. Off topic
Ref ID: 5325

(2122) Hamadani JD, Fuchs GJ, Osendarp SJ, Khatun F, Huda SN, Grantham-McGregor SM. Randomized controlled trial of the effect of zinc supplementation on the mental development of Bangladeshi infants. American Journal of Clinical Nutrition 2001;74:381-6. Diet Intervention or Supplement Study
Ref ID: 4815

(2123) Hamel LM, Robbins LB, Wilbur J. Computer- and web-based interventions to increase preadolescent and adolescent physical activity: a systematic review. [Review]. Journal of Advanced Nursing 2011 February;67(2):251-68. Review article
Ref ID: 2574

(2124) Hamer M, Boutcher Y, Boutcher SH. Cardiovascular and renal responses to mental challenge in highly and moderately active males with a family history of hypertension. Journal of Human Hypertension 2002 May;16(5):319. Off topic
Ref ID: 3619

(2125) Hammett JB, Hey WT. Neuromuscular adaptation to short-term (4 weeks) ballistic training in trained high school athletes. Journal of Strength and Conditioning Research 2003 August;17(3):556-60. Not All Participants were Overweight and/or Obese
Ref ID: 1720

(2126) Hampel FC, Kittner B, van-Bavel JH. Safety and tolerability of fexofenadine hydrochloride, 15 and 30 mg, twice daily in children aged 6 months to 2 years with allergic rhinitis. Annals of Allergy, Asthma and Immunology 2007;99:549-54. Drug intervention study
Ref ID: 4816

(2127) Hamra ST. The role of the septal reset in creating a youthful eyelid-cheek complex in facial rejuvenation. Plastic and Reconstructive Surgery 2004;113(7):2124-41. Off topic
Ref ID: 5326

(2128) Han JC, Lawlor DA, Kimm SY. Childhood obesity. The Lancet 2010;375(9727):1737-48. Review article
Ref ID: 3236

(2129) Hanai T, Takada H, Nagashima M, Kuwano T, Iwata H. Effects of exercise for 1 month on serum lipids in adolescent females. Pediatrics International 1999

June;41(3):253-9. Not All Participants were Overweight and/or Obese
Ref ID: 2022

(2130) Hanevold CD. Concepts guiding therapy for hypertension in children. Expert Review of Cardiovascular Therapy 2009;7(6):647-57. Review article
Ref ID: 3237

(2131) Hanisch F, Zierz S. Only transient increase of serum CoQ subset 10 during long-term CoQ10 therapy in mitochondrial ophthalmoplegia. European Journal of Medical Research 2003 November 12;8(11):485-91. Off topic
Ref ID: 1692

(2132) Hanna MG, Refaie A, Gouda N, Obaya G. Reduction of peri-operative bleeding in craniofacial surgeries in pediatrics. Comparison between recombinant factor VII and tranexamic acid. Egyptian Journal of Anaesthesia 2010;26:53-61. Off topic
Ref ID: 4817

(2133) Hanning RM, Blimkie CJ, Bar-Or O, Lands LC, Moss LA, Wilson WM. Relationships among nutritional status and skeletal and respiratory muscle function in cystic fibrosis: does early dietary supplementation make a difference? American Journal of Clinical Nutrition 1993 April;57(4):580-7. Off topic
Ref ID: 2271

(2134) Hansel TT, Benezet O, Kafé H, Ponitz HH, Cheung D, Engelstätter R, Barnes PJ. A multinational, 12-week, randomized study comparing the efficacy and tolerability of ciclesonide and budesonide in patients with asthma. Clinical Therapeutics 2006;28:906-20. Drug intervention study
Ref ID: 4818

(2135) Hansen HS, Froberg K, Hyldebrandt N, Nielsen JR. A controlled study of eight months of physical training and reduction of blood pressure in children: the Odense schoolchild study. BRITISH MEDICAL JOURNAL 1991;303:682-5. Inappropriate Outcomes
Ref ID: 4819

(2136) Hansen L, Bangsbo J, Twisk J, Klausen K. Development of muscle strength in relation to training level and testosterone in young male soccer players. Journal of Applied Physiology 1999 September;87(3):1141-7. Off topic
Ref ID: 2003

(2137) Hansen M, Morthorst R, Larsson B, Dall R, Flyvbjerg A, Rasmussen MH, Orskov H, Kjaer M, Lange KH. No effect of growth hormone administration on substrate oxidation during exercise in young, lean men. Journal of Physiology 2005 September 15;567(Pt:3):3-45. Study limited to adults
Ref ID: 1472

(2138) Hanson H, Jawad AF, Ryan T, Silver J. Factors Influencing Gross Motor Development in Young Children in an Urban Child Welfare System. Pediatric Physical Therapy 2011;23(4):335-46. Off topic
Ref ID: 5327

(2139) Harahap H, Jahari AB, Husaini MA, Saco PC, Pollitt E. Effects of an energy and micronutrient supplement on iron deficiency anemia, physical activity and motor and mental development in undernourished children in Indonesia. European Journal of Clinical Nutrition 2000;54 Suppl 2:S114-S119. Diet Intervention or Supplement Study
Ref ID: 4820

(2140) Harambat JÃ, Cochat P. Growth after renal transplantation. Pediatric Nephrology 2009 July;24(7):1297-306. Off topic
Ref ID: 3620

(2141) Hardee JP, Porter RR, Sui X, Archer E, Lee IM, Lavie CJ, Blair SN. The effect of resistance exercise on all-cause mortality in cancer survivors. Mayo Clinic Proceedings 2014 August;89(8):1108-15. Inappropriate Population
Ref ID: 5877

(2142) Hardin DS, Hebert JD, Bayden T, Dehart M, Mazur L. Treatment of childhood syndrome X. Pediatrics 1997 August;100(2):E5. Not a randomized controlled trial (RCT)
Ref ID: 2113

(2143) Hardin DS. GH improves growth and clinical status in children with cystic fibrosis -- a review of published studies. [Review] [18 refs]. European Journal of Endocrinology 2004 August;151(Suppl 5):S81-S85. Review article
Ref ID: 1592

(2144) Hardman AE, Lawrence JE, Herd SL. Postprandial lipemia in endurance-trained people during a short interruption to training. Journal of Applied Physiology 1998 June;84(6):1895-901. Off topic
Ref ID: 2090

(2145) Hardy LL, King L, Kelly B, Farrell L, Howlett S. Munch and Move: evaluation of a preschool healthy eating and movement skill program. International Journal of Behavioral Nutrition and Physical Activity 2010;7:80. Off topic
Ref ID: 4821

(2146) Hardy LL, Grunseit A, Khambalia A, Bell C, Wolfenden L, Milat AJ. Co-occurrence of obesogenic risk factors among adolescents. Journal of Adolescent Health 2012 September;51(3):265-71. Inappropriate Study Design
Ref ID: 5878

(2147) Hare ME, Coday M, Williams NA, Richey PA, Tylavsky FA, Bush AJ. Methods and baseline characteristics of a randomized trial treating early childhood

obesity: the Positive Lifestyles for Active Youngsters (Team PLAY) trial. Contemporary Clinical Trials 2012 May;33(3):534-49. Description of study from review or magazine or etc. (not the actual study)
Ref ID: 2575

(2148) Harju J, Juvonen P, Eskelinen M, Miettinen P, Paakkonen M. Minilaparotomy cholecystectomy versus laparoscopic cholecystectomy: a randomized study with special reference to obesity. Surgical Endoscopy 2006 April;20(4):583-6. Off topic
Ref ID: 1398

(2149) Harke H, Gretenkort P, Schmidt K, Hommerich P, Deutschmann S, Eckes C, Hense W, Kleemann A, Rehorn W, Stocker H, Leffers B, Blazejak J, Felix S, Fischer A, Fuhrmeister O, Gerecht A, Hackstein N, Knops HJ, Kostecka D, et a. A comparison of neurolept-, balanced, and intravenous propofol anaesthesia. Part 1: Design and patient demographics. Anaesthesist 1995;44:531-7. Off topic
Ref ID: 4822

(2150) Harke H, Gretenkort P, Schmidt K, Hommerich P, Deutschmann S, Eckes C, Hense W, Kleemann A, Rehorn W, Stöcker H. [Quality comparison of modified neurolept-, balanced and intravenous anesthesia. 1. Study design and patient analysis of the Krefelder study 1992]. Der Anaesthesist 1995;44:531-7. Off topic
Ref ID: 4823

(2151) Harrell JS, McMurray RG, Bangdiwala SI, Frauman AC, Gansky SA, Bradley CB. Effects of a school-based intervention to reduce cardiovascular disease risk factors in elementary-school children: the Cardiovascular Health in Children (CHIC) study. Journal of Pediatrics 1996 June;128(6):797-805. Not All Participants were Overweight and/or Obese
Ref ID: 2181

(2152) Harrell JS, Johnston LF, Griggs TR, Schaefer P, Carr EG, Jr., McMurray RG, Meibohm AR, Munoz S, Raines BN, Williams OD. An occupation based physical activity intervention program: improving fitness and decreasing obesity. AAOHN J 1996 August;44(8):377-84. Study limited to adults
Ref ID: 428

(2153) Harrell JS, Gansky SA, McMurray RG, Bangdiwala SI, Frauman AC, Bradley CB. School-based interventions improve heart health in children with multiple cardiovascular disease risk factors. Pediatrics 1998 August;102(2 Pt 1):371-80. No exercise only group
Ref ID: 422

(2154) Harrell JS, McMurray RG, Gansky SA, Bangdiwala SI, Bradley CB. A public health vs a risk-based intervention to improve cardiovascular health in

elementary school children: the Cardiovascular Health in Children Study. American Journal of Public Health 1999 October;89(10):1529-35. No control group (NC)
Ref ID: 1997

(2155) Harris KC, Kuramoto LK, Schulzer M, Retallack JE. Effect of school-based physical activity interventions on body mass index in children: a meta-analysis. [Review] [76 refs]. CMAJ Canadian Medical Association Journal 2009 March 31;180(7):719-26. Review article
Ref ID: 778

(2156) Harris MB, Hallbauer ES. Self-directed weight control through eating and exercise. Behaviour Research and Therapy 1973 November;11(4):523-9. Diet Intervention Study
Ref ID: 2576

(2157) Harrison A, Pierre RB, Palmer P, Moore J, Davis D, Dunkley-Thompson J, Figueroa JP, Christie CDC. Clinical Manifestations of Adolescents with HIV/AIDS in Jamaica. West Indian Medical Journal 2008;57(3):257-64. Off topic
Ref ID: 5329

(2158) Harrison M, Burns CF, McGuinness M, Heslin J, Murphy NM. Influence of a health education intervention on physical activity and screen time in primary school children: 'Switch Off--Get Active'. Journal of Science and Medicine in Sport 2006 October;9(5):388-94. Not a randomized controlled trial (RCT)
Ref ID: 1344

(2159) Harrison RA, Roberts C, Elton PJ. Does primary care referral to an exercise programme increase physical activity one year later? A randomized controlled trial. Journal of Public Health (Oxford) 2005 March;27(1):25-32. Study not limited to children and adolescents
Ref ID: 321

(2160) Harsha DW, Bray GA. Body composition and childhood obesity. Endocrinol Metab Clin North Am 1996;25(4):871-. Review article
Ref ID: 5330

(2161) Hart N, Tounian P, Clement A, Boule M, Polkey MI, Lofaso F, Fauroux B. Nutritional status is an important predictor of diaphragm strength in young patients with cystic fibrosis. American Journal of Clinical Nutrition 80(5):1201-6, 2004 Nov 2004;(5):1201-6. Off topic
Ref ID: 2992

(2162) Harten N, Olds T, Dollman J. The effects of gender, motor skills and play area on the free play activities of 8-11 year old school children. Health and Place 2008 September;14(3):386-93. Cross-sectional study
Ref ID: 980

(2163) Hartman A, te Winkel ML, van Beek RD, de Muinck Keizer-Schrama SM, Kemper HC, Hop WC, van den Heuvel-Eibrink MM, Pieters R. A randomized trial investigating an exercise program to prevent reduction of bone mineral density and impairment of motor performance during treatment for childhood acute lymphoblastic leukemia. Pediatric Blood and Cancer 2009 July;53(1):64-71. Off topic
Ref ID: 753

(2164) Hartman D, Crisp A, Rooney B, Rackow C, Atkinson R, Patel S. Bone density of women who have recovered from anorexia nervosa. International Journal of Eating Disorders 2000 July;28(1):107-12. Off topic
Ref ID: 1980

(2165) Hartman JW, Tang JE, Wilkinson SB, Tarnopolsky MA, Lawrence RL, Fullerton AV, Phillips SM. Consumption of fat-free fluid milk after resistance exercise promotes greater lean mass accretion than does consumption of soy or carbohydrate in young, novice, male weightlifters. American Journal of Clinical Nutrition 2007 August;86(2):373-81. Diet Intervention or Supplement Study
Ref ID: 1187

(2166) Hartmann T, Zahner L, Puhse U, Puder JJ, Kriemler S. Effects of a school-based physical activity program on physical and psychosocial quality of life in elementary school children: a cluster-randomized trial. Pediatric Exercise Science 2010 November;22(4):511-22. Primary outcome(s) not assessed
Ref ID: 2

(2167) Hartmann T, Zahner L, Puhse U, Schneider S, Puder JJ, Kriemler S. Physical activity, bodyweight, health and fear of negative evaluation in primary school children. Scandinavian Journal of Medicine and Science in Sports 2010 February;20(1):e27-e34. Not All Participants were Overweight and/or Obese
Ref ID: 121

(2168) Harvey-Berino J, Rourke J. Obesity prevention in preschool native-american children: a pilot study using home visiting. Obesity Research 2003 May;11(5):606-11. No exercise only group, No comparative control group
Ref ID: 364

(2169) Harvey EM, Dobson V, Tung B, Quinn GE, Hardy RJ. Interobserver agreement for grating acuity and letter acuity assessment in 1- to 5.5-year-olds with severe retinopathy of prematurity. Investigative Ophthalmology and Visual Science 1999;40:1565-76. Off topic
Ref ID: 4825

(2170) Harvey NC, Javaid K, Bishop N, Kennedy S, Papageorghiou AT, Fraser R, Gandhi SV, Schoenmakers I, Prentice A, Cooper C. MAVIDOS Maternal Vitamin D Osteoporosis Study: study protocol for a randomized controlled trial. The MAVIDOS Study Group. Trials 2012;13. Description of study from review

or magazine or etc. (not the actual study)
Ref ID: 5331

(2171) Harwood K, Vuguin P, DiMartino-Nardi J. Current approaches to the diagnosis and treatment of polycystic ovarian syndrome in youth. Hormone Research 2007;68(5):209-17. Off topic
Ref ID: 3238

(2172) Harz KJ, Muller HL, Waldeck E, Pudel V, Roth C. Obesity in patients with craniopharyngioma: assessment of food intake and movement counts indicating physical activity. Journal of Clinical Endocrinology and Metabolism 2003 November;88(11):5227-31. Cross-sectional study
Ref ID: 1704

(2173) Hasik J, Gawlak E, Tycowa M. [Treatment of simple obesity with mazindol under sanatorium conditions]. [Polish]. Polski Tygodnik Lekarski 1980 November 10;35(45):1729-31. Drug intervention study
Ref ID: 2375

(2174) Hassan J, van der Net J, Helders PJ, Prakken BJ, Takken T. Six-minute walk test in children with chronic conditions. British Journal of Sports Medicine 2010 March;44(4):270-4. Cross-sectional study
Ref ID: 562

(2175) Hasson RE, Granados K, Chipkin S, Freedson PS, Braun B. Effects of a single exercise bout on insulin sensitivity in black and white individuals. Journal of Clinical Endocrinology and Metabolism 2010 October;95(10):E219-E223. Acute study
Ref ID: 2578

(2176) Hasson RE, Adam TC, Davis JN, Kelly LA, Ventura EE, Byrd-Williams CE, Toledo-Corral CM, Roberts CK, Lane CJ, Azen SP, Chou CP, Spruijt-Metz D, Weigensberg MJ, Berhane K, Goran MI. Randomized controlled trial to improve adiposity, inflammation, and insulin resistance in obese African-American and Latino youth. Obesity 2012 April;20(4):811-8. Diet & Exercise intervention
Ref ID: 2579

(2177) Hattori H, Matsuzaki A, Suminoe A, Ihara K, Eguchi M, Tajiri T, Suita S, Ishii E, Hara T. Genomic imprinting of insulin-like growth factor-2 in infant leukemia and childhood neuroblastoma. Cancer 2000;88(10):2372-7. Off topic
Ref ID: 5332

(2178) Haug LS, Thomsen C, Brantsaeter AL, Kvalem HE, Haugen M, Becher G, Alexander J, Meltzer HM, Knutsen HK. Diet and particularly seafood are major sources of perfluorinated compounds in humans. Environment International 2010;36(7):772-8. Off topic
Ref ID: 5333

(2179) Haugen JE. The use of chemical sensor array technology, the electronic nose, for detection of boar taint. Acta Veterinaria Scandinavica 2006 January 2;48:S15-3. Animal study
Ref ID: 3621

(2180) Haupenthal A, Fontana HdB, Ruschel C, Roesler H, Borgatto AF. Predição da força de reação do solo durante a corrida na água. Fisioterapia e Pesquisa 2010 September;17(3):253-8. Off topic
Ref ID: 4206

(2181) Haut S. Psychiatric History and Temporal Lobectomy Outcome: Looking to the Past to Predict the Future. Epilepsy Currents 2009 September;9(5):135-6. Off topic
Ref ID: 3622

(2182) Hawkins SS, Cole TJ, Law C, Millennium Cohort Study Child Health Group. Examining the relationship between maternal employment and health behaviours in 5-year-old British children. Journal of Epidemiology and Community Health 2009 December;63(12):999-1004. Off topic
Ref ID: 2580

(2183) Haymond M, Anderson B, Barrera P, Brosnan P, Bush C, Green T, Holden H, Jeha G, Jones M, McGirk S, McKay S, Miller D, Schreiner B, Zarate M, Dahms W, Casey T, Cuttler L, Drotar D, Frieson E, Levers-Landis C, McGuigan P, Palmert M, Sundararajan S, Witherspoon D, Geffner M et al. Treatment options for type 2 diabetes in adolescents and youth: A study of the comparative efficacy of metformin alone or in combination with rosiglitazone or lifestyle intervention in adolescents with type 2 diabetes. Pediatric Diabetes 2007;8(2):74-87. Review article
Ref ID: 3239

(2184) HÃ©brard GÃ, Hoffart Vr, Beyssac E, Cardot JM, Alric M, Subirade M. Coated whey protein/alginate microparticles as oral controlled delivery systems for probiotic yeast. Journal of Microencapsulation 2010 June;27(4):292-302. Off topic
Ref ID: 3623

(2185) HÃ¶ybye C, Hilding A, Jacobsson H, ThorÃ©n M. Growth hormone treatment improves body composition in adults with Praderâ€“Willi syndrome. Clinical Endocrinology 2003 May;58(5):653-61. Off topic
Ref ID: 3624

(2186) He FJ, MacGregor GA. Effect of modest salt reduction on blood pressure: a meta-analysis of randomized trials. Implications for public health. Journal of Human Hypertension 2002 November;16(11):761. Review article
Ref ID: 3625

(2187) He JP, Stein AD, Humphrey HEB, Paneth N, Courval JM. Time trends in sport-caught Great Lakes fish consumption and serum polychlorinated biphenyl levels among Michigan anglers, 1973-1993. Environmental Science and Technology 2001;35(3):435-40. Off topic
Ref ID: 5334

(2188) He ZH, Ma LH. The aerobic fitness (VO2 peak) and alpha-fibrinogen genetic polymorphism in obese and non-obese Chinese boys. International Journal of Sports Medicine 2005 May;26(4):253-7. Cohort Study
Ref ID: 1531

(2189) Head K, Jurenka J. Inflammatory Bowel Disease Part II: Crohn's Disease - Pathophysiology and Conventional and Alternative Treatment Options. Alternative Medicine Review 2004 December;9(4):360-401. Off topic
Ref ID: 3626

(2190) Heal DJ, Gosden J, Smith SL. Regulatory challenges for new drugs to treat obesity and comorbid metabolic disorders. British Journal of Clinical Pharmacology 2009 December;68(6):861-74. Off topic
Ref ID: 626

(2191) HEALTHY study group, Foster GD, Linder B, Baranowski T, Cooper DM, Goldberg L, Harrell JS, Kaufman F, Marcus MD, Trevino RP, Hirst K. A school-based intervention for diabetes risk reduction. New England Journal of Medicine 2010 July 29;363(5):443-53. Not All Participants were Overweight and/or Obese
Ref ID: 481

(2192) Heathcock JC, Lobo M, Galloway JC. Movement training advances the emergence of reaching in infants born at less than 33 weeks of gestational age: a randomized clinical trial. Physical Therapy 2008;88:310-22. Subjects less than 2 years old
Ref ID: 4826

(2193) Heathcock JC, Galloway JC. Exploring objects with feet advances movement in infants born preterm: a randomized controlled trial. Physical Therapy 2009;89:1027-38. Subjects less than 2 years old
Ref ID: 4827

(2194) Hebden L, Chey T, Allman-Farinelli M. Lifestyle intervention for preventing weight gain in young adults: a systematic review and meta-analysis of RCTs. Obesity Reviews 2012 August;13(8):692-710. Inappropriate Study Design
Ref ID: 5879

(2195) Hebden L, Balestracci K, McGeechan K, Denney-Wilson E, Harris M, Bauman A, Allman-Farinelli M. 'TXT2BFiT' a mobile phone-based healthy lifestyle program for preventing unhealthy weight gain in young adults: study protocol

for a randomized controlled trial. Trials 2013;14:75. Inappropriate Study Design
Ref ID: 5880

(2196) Hebestreit H, Mimura K, Baror O. Recovery of Muscle Power After High-Intensity Short-Term Exercise - Comparing Boys and Men. Journal of Applied Physiology 1993;74(6):2875-80. Off topic
Ref ID: 5335

(2197) Hebestreit H, Dunstheimer D, Staschen B, Strassburg HM. Single-leg Wingate Test in children: reliability and optimal braking force. Medicine and Science in Sports and Exercise 1999 August;31(8):1218-25. Acute study
Ref ID: 2006

(2198) Hebestreit H, Hebestreit A, Trusen A, Hughson RL. Oxygen uptake kinetics are slowed in cystic fibrosis. Medicine and Science in Sports and Exercise 2005 January;37(1):10-7. Not a randomized controlled trial (RCT)
Ref ID: 1553

(2199) Hecker TM, Aris RM. Management of osteoporosis in adults with cystic fibrosis. [Review] [74 refs]. Drugs 2004;64(2):133-47. Review article
Ref ID: 1671

(2200) Hediger ML, England LJ, Molloy CA, Yu KF, Manning-Court, Mills JL. Reduced Bone Cortical Thickness in Boys with Autism or Autism Spectrum Disorder. Journal of Autism and Developmental Disorders 2008 May;38(5):848-56. Off topic
Ref ID: 3820

(2201) Heinonen A, Sievänen H, Kannus P, Oja P, Pasanen M, Vuori I. High-impact exercise and bones of growing girls: a 9-month controlled trial. Osteoporosis international : a journal established as result of cooperation between the European Foundation for Osteoporosis and the National Osteoporosis Foundation of the USA 2000;11:1010-7. Not a randomized controlled trial (RCT)
Ref ID: 4828

(2202) Helge EW, Kanstrup IL. Bone density in female elite gymnasts: impact of muscle strength and sex hormones. Medicine and Science in Sports and Exercise 2002 January;34(1):174-80. Off topic
Ref ID: 1857

(2203) Hellings JA, Zarcone JR, Crandall K, Wallace D, Schroeder SR. Weight gain in a controlled study of risperidone in children, adolescents and adults with mental retardation and autism. Journal of Child and Adolescent Psychopharmacology 2001;11(3):229-38. Drug intervention study
Ref ID: 3240

(2204) Henck JW, Mattsson JL, Rezabek DH, Carlson CL, Rech RH. Developmental Neurotoxicity of Polybrominated Biphenyls. Neurotoxicology and Teratology 1994;16(4):391-9. Animal study
Ref ID: 5336

(2205) Hendelman DL, Ornstein K, Debold EP, Volpe SL, Freedson PS. Preexercise feeding in untrained adolescent boys does not affect responses to endurance exercise or performance. International Journal of Sport Nutrition 1997 September;7(3):207-18. Diet Intervention Study
Ref ID: 2126

(2206) Henderson RC, Kairalla J, Abbas A, Stevenson RD. Predicting low bone density in children and young adults with quadriplegic cerebral palsy. Developmental Medicine and Child Neurology 46(6):416-9, 2004 Jun 2004;(6):416-9. Off topic
Ref ID: 2998

(2207) Henderson VR. Longitudinal associations between television viewing and body mass index among white and black girls. Journal of Adolescent Health 2007 December;41(6):544-50. Longitudinal Study
Ref ID: 1140

(2208) Henness S, Perry CM. Orlistat: a review of its use in the management of obesity. [Review] [115 refs]. Drugs 2006;66(12):1625-56. Review article
Ref ID: 1352

(2209) Henney HR, III, Faust B, Blight AR. Effect of food on the single-dose pharmacokinetics and tolerability of dalfampridine extended-release tablets in healthy volunteers. American Journal of Health-System Pharmacy 2011 November 15;68(22):2148-54. Off topic
Ref ID: 1036

(2210) Hennig EM. The evolution and biomechanics of the human foot - applied research for footwear. Revista Brasileira de Biomecânica 2003;4(1):7-14. Off topic
Ref ID: 4207

(2211) Hennig MdAeS, Gomes MAdSM, Gianini NOM. Conhecimentos e práticas dos profissionais de saúde sobre a "atenção humanizada ao recém-nascido de baixo peso - Método canguru. Revista Brasileira de Saúde Materno Infantil 2006;6(4):427-36. Survey or questionnaire
Ref ID: 4208

(2212) Herbert WJ, Heiss DG, Basso DM. Influence of feedback schedule in motor performance and learning of a lumbar multifidus muscle task using rehabilitative ultrasound imaging: a randomized clinical trial. Physical Therapy 2008 February;88(2):261-9. Off topic
Ref ID: 1004

(2213) Herd SL, Hardman AE, Boobis LH, Cairns CJ. The effect of 13 weeks of running training followed by 9 d of detraining on postprandial lipaemia. BRITISH JOURNAL OF NUTRITION 1998 July;80(1):57-66. Study limited to adults
Ref ID: 2077

(2214) Herd SL, Lawrence JE, Malkova D, Murphy MH, Mastana S, Hardman AE. Postprandial lipemia in young men and women of contrasting training status. Journal of Applied Physiology 2000 November;89(5):2049-56. Off topic
Ref ID: 1953

(2215) Herdy AH, Uhlendorf D. Valores de referência para o teste cardiopulmonar para homens e mulheres sedentários e ativos. Reference values for cardiopulmonary exercise testing for sedentary and active men and women. Valores de referencia para el test cardiopulmonar para hombres y mujeres sedentarios y activos. Arquivos Brasileiros de Cardiologia 2011 January;96(1):54-9. Off topic
Ref ID: 4209

(2216) Hergenroeder AC, Schoene RB. Predicting maximum oxygen uptake in adolescents. American Journal of Diseases of Children 1989 June;143(6):673-7. Off topic
Ref ID: 2321

(2217) Hergenroeder AC. Bone mineralization, hypothalamic amenorrhea, and sex steroid therapy in female adolescents and young adults. [Review] [90 refs]. Journal of Pediatrics 1995 May;126(5:Pt 1):t-9. Review article
Ref ID: 2210

(2218) Hermansen K, Kipnes M, Luo E, Fanurik D, Khatami H, Stein P, Sitagliptin S. Efficacy and safety of the dipeptidyl peptidase-4 inhibitor, sitagliptin, in patients with type 2 diabetes mellitus inadequately controlled on glimepiride alone or on glimepiride and metformin. Diabetes, Obesity and Metabolism 2007 September;9(5):733-45. Drug intervention study
Ref ID: 1183

(2219) Hermelo M, Amador M, Alvarez R, Alonso A. Pérfil lipídico de adolescentes obesos antes y después de dos semanas con tratamiento reductor. Revista Cubana de Pediatria 1985 June;57(3):273-82. Diet & Exercise intervention
Ref ID: 4210

(2220) Hernan JO, Ramirez-Velez R. [Strength training improves insulin sensitivity and plasma lipid levels without altering body composition in overweight and obese subjects]. [Spanish]. Endocrinology and Nutrition 2011 April;58(4):169-74. Study limited to adults
Ref ID: 2582

(2221) Hernández-Escolar J, Herazo-Beltrán Y, Valero MV. Frecuencia de factores de riesgo asociados a enfermedades cardiovasculares en población universitaria

joven. The frequency of cardiovascular disease-associated risk factors in a university student population. Revista de Salud Pública 2010 October;12(5):852-64. Cross-sectional study
Ref ID: 4211

(2222) Hernández Hernández RA, Hernández de Valera Y. Contextura en grupo de niños venezolanos. Anales venezolanos de nutrición 1999;12(1):5-9. Cross-sectional study
Ref ID: 4212

(2223) Hernández B, Haene Jd, Barquera S, Monterrubio E, Rivera J, Shamah T, Sepúlveda J, Haas J, Campirano F. Factores asociados con la actividad física en mujeres mexicanas en edad reproductiva. Revista Panamericana de Salud Pública 2003 October;14(4):235-45. Secondary analysis
Ref ID: 715

(2224) Hernández V. Manejo nutricional del niño obeso. Revista Gastrohnup 2011 August;13(2, Supl.1):S20-S26. Review article
Ref ID: 4213

(2225) Herrera Lucena J, Isaacura C, Martinez I. Caracteristicas del crecimiento fisico durante las primeras seis semanas de vida en 272 ninos de bajo peso. Barquisimeto 1976-82. Archivos Venezolanos de Puericultura y Pediatría 1984;47(1/2):11-5. Off topic
Ref ID: 4214

(2226) Herron KL, Vega-Lopez S, Conde K, Ramjiganesh T, Roy S, Shachter NS, Fernandez ML. Pre-menopausal women, classified as hypo- or hyperresponders, do not alter their LDL/HDL ratio following a high dietary cholesterol challenge. Journal of the American College of Nutrition 2002 June;21(3):250-8. Study limited to adults
Ref ID: 378

(2227) Hersch EC, Merriam GR. Growth hormone (GH)-releasing hormone and GH secretagogues in normal aging: Fountain of Youth or Pool of Tantalus? Clinical Interventions in Aging 2008;3(1):121-9. Off topic
Ref ID: 3241

(2228) Hertzrnan-Miller RP, Morgenstern H, Hurwitz EL, Fei Y, Adams AH, Harber P, Kominski GF. Comparing the Satisfaction of Low Back Pain Patients Randomized to Receive Medical or Chiropractic Care: Results From the UCLA Low-Back Pain Study. American Journal of Public Health 2002 October;92(10):1628-33. Off topic
Ref ID: 3821

(2229) Hesketh K, Waters E, Green J, Salmon L, Williams J. Healthy eating, activity and obesity prevention: a qualitative study of parent and child perceptions in Australia. Health Promotion International 2005 March;20(1):19-26. Not a

randomized controlled trial (RCT)
Ref ID: 1543

(2230) Hesketh KD, Campbell K, Salmon J, McNaughton SA, McCallum Z, Cameron A, Ball K, Gold L, Andrianopoulos N, Crawford D. The Melbourne Infant Feeding, Activity and Nutrition Trial (InFANT) Program follow-up. Contemporary Clinical Trials 2013 January;34(1):145-51. Inappropriate Population
Ref ID: 5881

(2231) Heuser L, Herbig S. The childhood obesity challenge--Tap into Fitness: program overview and results analysis. Journal of the Kentucky Medical Association 2008 March;106(3):118-22. Case-Control / Case Study
Ref ID: 953

(2232) Heussler H, Harris M, Cooper D, Dakin C, Suresh S, Williams G. Hypersomnolence in Prader Willi Syndrome. Journal of Intellectual Disability Research 2008 October;52(10):814. Off topic
Ref ID: 3822

(2233) Heussler H, Suresh S, Harris M, Cooper D, Dakin C, Williams G, Wilson S. Developmental aspects of sleep in Prader Willi Syndrome. Journal of Intellectual Disability Research 2008 October;52(10):815. Off topic
Ref ID: 3823

(2234) Heydari M, Boutcher YN, Boutcher SH. The effects of high-intensity intermittent exercise training on cardiovascular response to mental and physical challenge. Int J Psychophysiol 2013 February;87(2):141-6. Inappropriate Population
Ref ID: 5882

(2235) Heyman E, Toutain C, Delamarche P, Berthon P, Briard D, Youssef H, Dekerdanet M, Gratas-Delamarche A. Exercise training and cardiovascular risk factors in type 1 diabetic adolescent girls. Pediatric Exercise Science 2007 November;19(4):408-19. Not All Participants were Overweight and/or Obese
Ref ID: 1124

(2236) Hickey MS, Calsbeek DJ. Plasma leptin and exercise - Recent findings. Sports Medicine 2001;31(8):583-9. Review article
Ref ID: 5337

(2237) Hickson JF, Jr., Hinkelmann K. Exercise and protein intake effects on urinary 3-methylhistidine excretion. American Journal of Clinical Nutrition 1985 February;41(2):246-53. Off topic
Ref ID: 2359

(2238) Hidaka S, Kaneko O, Shirai M, Kojima K, Igarashi Y, Oda K, Chimata M, Nakamura K, Nagase M. Do obesity and non-insulin dependent diabetes mellitus aggravate exercise-induced microproteinuria? Clinica Chimica Acta 1998 July 28;275(2):115-26. Not All Participants were Overweight and/or

Obese
Ref ID: 2083

(2239) Hilgartner MW, Donfield SM, Lynn HS, Hoots WK, Gomperts ED, Daar ES, Chernoff D, Pearson SK. The effect of plasma human immunodeficiency virus RNA and CD4(+) T lymphocytes on growth measurements of hemophilic boys and adolescents. Pediatrics 2001;107(4):art-e56. Off topic
Ref ID: 5338

(2240) Hill C, Llewellyn CH, Saxton J, Webber L, Semmler C, Carnell S, van Jaarsveld CHM, Boniface D, Wardle J. Adiposity and 'eating in the absence of hunger' in children. International Journal of Obesity 2008;32(10):1499-505. Cross-sectional study
Ref ID: 5339

(2241) Hill C, Wardle J, Cooke L. Adiposity is not associated with children's reported liking for selected foods. Appetite 2009;52(3):603-8. Cross-sectional study
Ref ID: 5340

(2242) Hill JM, Kornblith AB, Jones D, Freeman A, Holland JF, Glicksman AS, Boyett JM, Lenherr B, Brecher ML, Dubowy R, Kung F, Maurer H, Holland JC. A comparative study of the long term psychosocial functioning of childhood acute lymphoblastic leukemia survivors treated by intrathecal methotrexate with or without cranial radiation. Cancer 1998;82:208-18. Off topic
Ref ID: 911

(2243) Hill K, Bucuvalas J, McClain C, Kryscio R, Martini RT, Alfaro MP, Maloney M. Pilot study of growth hormone administration during the refeeding of malnourished anorexia nervosa patients. Journal of Child and Adolescent Psychopharmacology 2000;10:3-8. Off topic
Ref ID: 4829

(2244) Hill LJ, Williams JH, Aucott L, Thomson J, Mon-Williams M. How does exercise benefit performance on cognitive tests in primary-school pupils? Developmental Medicine and Child Neurology 2011 July;53(7):630-5. Study less than 4 weeks
Ref ID: 2583

(2245) Hillebrand JJ, van Elburg AA, Kas MJ, van EH, Adan RA. Olanzapine reduces physical activity in rats exposed to activity-based anorexia: possible implications for treatment of anorexia nervosa? Biological Psychiatry 2005 October 15;58(8):651-7. Animal study
Ref ID: 1455

(2246) Hillenbrand JM, Houde RA. A narrow band pattern-matching model of vowel perception. Journal of the Acoustical Society of America 2003;113(2):1044-55. Off topic
Ref ID: 5341

(2247) Hillier F, Pedley C, Summerbell C. Evidence base for primary prevention of obesity in children and adolescents. [Review]. Bundesgesundheitsblatt, Gesundheitsforschung, Gesundheitsschutz 2011 March;54(3):259-64. Review article
Ref ID: 2584

(2248) Hillier FC, Batterham AM, Nixon CA, Crayton AM, Pedley CL, Summerbell CD. A community-based health promotion intervention using brief negotiation techniques and a pledge on dietary intake, physical activity levels and weight outcomes: lessons learnt from an exploratory trial. Public Health Nutrition 2012 August;15(8):1446-55. Inappropriate Intervention
Ref ID: 5883

(2249) Hills AP, Parker AW. Gait characteristics of obese pre-pubertal children: effects of diet and exercise on parameters. International Journal of Rehabilitation Research 1991;14:348-9. Diet & Exercise intervention
Ref ID: 4830

(2250) Himes JH, Ring K, Gittelsohn J, Cunningham-Sabo L, Weber J, Thompson J, Harnack L, Suchindran C. Impact of the Pathways intervention on dietary intakes of American Indian schoolchildren. Preventive Medicine 2003 December;37(6 Pt 2):S55-S61. Primary outcome(s) not assessed
Ref ID: 347

(2251) Hind K, Burrows M. Weight-bearing exercise and bone mineral accrual in children and adolescents: a review of controlled trials. [Review] [46 refs]. Bone 2007 January;40(1):14-27. Review article
Ref ID: 1296

(2252) Hinderliter PM, Price PS, Bartels MJ, Timchalk C, Poet TS. Development of a source-to-outcome model for dietary exposures to insecticide residues: An example using chlorpyrifos. Regulatory Toxicology and Pharmacology 2011;61(1):82-92. Off topic
Ref ID: 5342

(2253) Hino AAF, Reis RS, Rodriguez-Añez CR. Observação dos níveis de atividade física, contexto das aulas e comportamento do professor em aulas de educação física do ensino médio da rede pública. Revista Brasileira de Atividade Física e Saúde 2007;12(3). Not a randomized controlled trial (RCT)
Ref ID: 4215

(2254) Hintz SR, Kendrick DE, Vohr BR, Poole WK, Higgins RD. Community supports after surviving extremely low-birth-weight, extremely preterm birth - Special outpatient services in early childhood. Archives of Pediatrics and Adolescent Medicine 2008;162(8):748-55. Off topic
Ref ID: 5343

(2255) Hirsch LJ. Breathing New Life into the Fight against Sudden Death in Epilepsy. Epilepsy Currents 2009 September;9(5):137-9. Off topic
Ref ID: 3627

(2256) Hirst K, Baranowski T, DeBar L, Foster GD, Kaufman F, Kennel P, Linder B, Schneider M, Venditti EM, Yin Z. HEALTHY study rationale, design and methods: moderating risk of type 2 diabetes in multi-ethnic middle school students. International Journal of Obesity (London) 2009 August;33 Suppl 4:S4-20.:S4-20. No exercise only group
Ref ID: 107

(2257) Hitos K, Cannon M, Cannon S, Garth S, Fletcher JP. Effect of leg exercises on popliteal venous blood flow during prolonged immobility of seated subjects: implications for prevention of travel-related deep vein thrombosis. Journal of Thrombosis and Haemostasis 2007 September;5(9):1890-5. Off topic
Ref ID: 1175

(2258) Hivert MF, Langlois MF, Carpentier AC. The entero-insular axis and adipose tissue-related factors in the prediction of weight gain in humans. [Review] [77 refs]. International Journal of Obesity 2007 May;31(5):731-42. Review article
Ref ID: 1243

(2259) Ho M, Garnett SP, Baur L, Burrows T, Stewart L, Neve M, Collins C. Effectiveness of lifestyle interventions in Childhood Obesityity: systematic review with meta-analysis. Pediatrics 2012 December;130(6):e1647-e1671. Inappropriate Study Design
Ref ID: 5884

(2260) Ho M, Garnett SP, Baur LA, Burrows T, Stewart L, Neve M, Collins C. Impact of dietary and exercise interventions on weight change and metabolic outcomes in obese children and adolescents: a systematic review and meta-analysis of randomized trials. JAMA Pediatrics 2013 August 1;167(8):759-68. Inappropriate Study Design
Ref ID: 5886

(2261) Ho M, Gow M, Halim J, Chisholm K, Baur LA, Noakes M, Steinbeck K, Kohn MR, Cowell CT, Garnett SP. Effect of a prescriptive dietary intervention on psychological dimensions of eating behavior in obese adolescents. International Journal of Behavioral Nutrition and Physical Activity 2013;10:119. Inappropriate Intervention
Ref ID: 5885

(2262) Ho RC, Davy KP, Hickey MS, Summers SA, Melby CL. Behavioral, metabolic, and molecular correlates of lower insulin sensitivity in Mexican-Americans. American Journal of Physiology-Endocrinology and Metabolism 2002;283(4):E799-E808. Study limited to adults
Ref ID: 5344

(2263) Hoban TF. Sleep disorders in children. Johnson RT, editor. 1184, 1-14. 2010. Review article,
Ref ID: 3242

(2264) Hochberg I, Hochberg Z. Expanding the definition of hypothalamic obesity. Obesity Reviews 2010 October;11(10):709-21. Review article
Ref ID: 3628

(2265) Hochstenbach-Waelen A, Westerterp KR, Soenen S, Westerterp-Plantenga MS. No long-term weight maintenance effects of gelatin in a supra-sustained protein diet. Physiology and Behavior 2010 September 1;101(2):237-44. No exercise only group
Ref ID: 43

(2266) Hodapp M, Vry J, Mall V, Faist M. Changes in soleus H-reflex modulation after treadmill training in children with cerebral palsy. Brain 2009;132:37-44. Off topic
Ref ID: 5345

(2267) Hoegerman GS, Lewis CE, Flack J, Raczynski JM, Caveny J, Gardin JM. Lack of association of recreational cocaine and alcohol use with left ventricular mass in young adults. The Coronary Artery Risk Development in Young Adults (CARDIA) study. Journal of the American College of Cardiology 1995 March 15;25(4):895-900. Off topic
Ref ID: 2216

(2268) Hoelscher DM, Feldman HA, Johnson CC, Lytle LA, Osganian SK, Parcel GS, Kelder SH, Stone EJ, Nader PR. School-based health education programs can be maintained over time: results from the CATCH Institutionalization study. Preventive Medicine 2004;38:594-606. Follow-up Study
Ref ID: 4831

(2269) Hoelscher DM, Kirk S, Ritchie L, Cunningham-Sabo L. Position of the Academy of Nutrition and Dietetics: interventions for the prevention and treatment of pediatric overweight and obesity. Journal of the Academy of Nutrition and Dietetics 2013 October;113(10):1375-94. Inappropriate Study Design
Ref ID: 5887

(2270) Hoffman DJ, Roberts SB, Verreschi I, Martins PA, de Nascimento C, Tucker KL, Sawaya AL. Regulation of energy intake may be impaired in nutritionally stunted children from the shantytowns of Sao Paulo, Brazil. Journal of Nutrition 2000;130(9):2265-70. Off topic
Ref ID: 5346

(2271) Hoffmann-Streb A, Niggemann B, Buttner P, Wahn U. [The protective effect of theophylline in exercise-induced asthma in childhood]. [German]. Klinische Padiatrie 1993 March;205(2):99-102. Off topic
Ref ID: 2265

(2272) Hoffmann M, Silva ACPd, Siviero J. Prevalência de hipertensão arterial sistêmica e interrelações com sobrepeso, obesidade, consumo alimentar e atividade física, em estudantes de escolas municipais de Caxias do Sul. Prevalence of hypertension and inter-relations with overweight, obesity, food intake and physical activity in students of municipal schools of Caxias do Sul. Pediatria (São Paulo) 2010 September;32(3):163-72. Prevalence study
Ref ID: 4216

(2273) Hoffmeister U, Bullinger M, Egmond-Frohlich A, Goldapp C, Mann R, Ravens-Sieberer U, Reinehr T, Westenhofer J, Holl RW. [Treatment of obesity in pediatric patients in Germany: anthropometry, comorbidity and socioeconomic gradients based on the BZgA Observational Study]. [German]. Klinische Padiatrie 2010 July;222(4):274-8. Not a randomized controlled trial (RCT)
Ref ID: 483

(2274) Hofman A, Grobbee DE. Non-pharmacological intervention in primary hypertension in childhood. [Review] [19 refs]. Clinical and Experimental Hypertension - Part A, Theory and Practice 1986;8(4-5):813-22. Review article
Ref ID: 2348

(2275) Hofmo PO. Sperm sorting and low-dose insemination in the pig -- An update. Acta Veterinaria Scandinavica 2006 January 2;48:S11-S13. Animal study
Ref ID: 3629

(2276) Hofsteenge GH, Chinapaw MJ, Weijs PJ, van Tulder MW, Delemarre-van de Waal HA. Go4it; Study design of a randomised controlled trial and economic evaluation of a multidisciplinary group intervention for obese adolescents for prevention of diabetes mellitus type 2. BMC Public Health 2008 December 16;8:410.:410. No exercise only group
Ref ID: 144

(2277) Hogan SP, Rosenberg HF, Moqbel REDW, Phipps SIMO, Foster PS, Lacy PAIG, Kay AB, Rothenberg ME. Eosinophils: Biological properties and role in health and disease. Clinical & Experimental Allergy 2008 May;709-750. Off topic
Ref ID: 3630

(2278) Hogg RJ, Portman RJ, Milliner D, Lemley KV, Eddy A, Ingelfinger J. Evaluation and management of proteinuria and nephrotic syndrome in children: Recommendations from a Pediatric Nephrology Panel established at the National Kidney Foundation Conference on Proteinuria. Pediatrics 2000 June;105(6):1242. Off topic
Ref ID: 514

(2279) Hoglund K, Normen L. A high exercise load is linked to pathological weight control behavior and eating disorders in female fitness instructors. Scandinavian Journal of Medicine and Science in Sports 2002

October;12(5):261-75. Survey or questionnaire
Ref ID: 1797

(2280) Hoglund P, Holmberg C, Delachapelle A, Kere J. Paternal Isodisomy for Chromosome-7 Is Compatible with Normal Growth and Development in A Patient with Congenital Chloride Diarrhea. American Journal of Human Genetics 1994;55(4):747-52. Off topic
Ref ID: 5347

(2281) Hoie LH, Bruusgaard D. Compliance, clinical effects, and factors predicting weight reduction during a very low calorie diet regime. Scandinavian Journal of Primary Health Care 1995 March;13(1):13-20. Diet Intervention Study
Ref ID: 2208

(2282) Holanda NMV, Andrade ISNd. Dinâmica familiar na alimentação de crianças com paralisia cerebral. Family dynamics in the process of feeding of children with cerebral palsy. Revista Brasileira em Promoção da Saúde (Impr) 2010 December;23(4). Off topic
Ref ID: 4217

(2283) Holbrook JM, Cohen PG. Aromatase Inhibition for the Treatment of Idiopathic Hypogonadotropic Hypogonadism in Men with Premature Ejaculation. Southern Medical Journal 2003 June;96(6):544-7. Off topic
Ref ID: 3631

(2284) Holene E, Nafstad I, Skaare JU, Bernhoft A, Engen P, Sagvolden T. Behavioral-Effects of Prenatal and Postnatal Exposure to Individual Polychlorinated Biphenyl Congeners in Rats. Environmental Toxicology and Chemistry 1995;14(6):967-76. Animal study
Ref ID: 5348

(2285) Hollar D, Messiah SE, Lopez-Mitnik G, Hollar TL, Almon M, Agatston AS. Healthier options for public schoolchildren program improves weight and blood pressure in 6- to 13-year-olds. Journal of the American Dietetic Association 2010 February;110(2):261-7. Not a randomized controlled trial (RCT)
Ref ID: 603

(2286) Hollinghurst S, Hunt LP, Banks J, Sharp DJ, Shield JP. Cost and effectiveness of treatment options for childhood obesity. Pediatric Obesity 2014 February;9(1):e26-e34. Inappropriate Outcomes
Ref ID: 5888

(2287) Hollywood E, Comiskey C, Begley T, Snel A, O'Sullivan K, Quirke M, Wynne C. Measuring and modelling body mass index among a cohort of urban children living with disadvantage. J Adv Nurs 2013 April;69(4):851-61. Inappropriate Study Design
Ref ID: 5889

(2288) Holmstrup ME, Fairchild TJ, Keslacy S, Weinstock RS, Kanaley JA. Satiety, but not total PYY, Is increased with continuous and intermittent exercise. Obesity (Silver Spring) 2013 October;21(10):2014-20. Inappropriate Intervention
Ref ID: 5890

(2289) Holt KG, Fonseca ST, LaFiandra ME. The dynamics of gait in children with spastic hemiplegic cerebral palsy: Theoretical and clinical implications. Human Movement Science 2000;19(3):375-405. Off topic
Ref ID: 5349

(2290) Holt N, Schetzina KE, Dalton WT, III, Tudiver F, Fulton-Robinson H, Wu T. Primary care practice addressing child overweight and obesity: a survey of primary care physicians at four clinics in southern Appalachia. Southern Medical Journal 2011 January;104(1):14-9. Survey or questionnaire
Ref ID: 1099

(2291) Holtz H, Heinrich J, Duck KD, Ruhling K, Heller R, Schauer I. [Effectiveness of different intervention measures in children and adolescents with hypertension and lipid metabolism disorders]. [German]. Zeitschrift fur die Gesamte Innere Medizin und Ihre Grenzgebiete 1983 December 1;38(23):644-9. No non-intervention control group
Ref ID: 2365

(2292) Holzman RS, van der Velde ME, Kaus SJ, Body SC, Colan SD, Sullivan LJ, Soriano SG. Sevoflurane depresses myocardial contractility less than halothane during induction of anesthesia in children. Anesthesiology 1996;85:1260-7. Drug intervention study
Ref ID: 4832

(2293) Honas JJ, Washburn RA, Smith BK, Greene JL, Cook-Wiens G, Donnelly JE. The System for Observing Fitness Instruction Time (SOFIT) as a measure of energy expenditure during classroom-based physical activity. Pediatric Exercise Science 2008 November;20(4):439-45. Not an exercise intervention study
Ref ID: 828

(2294) Hong TS, Shammas A, Charron M, Zukotynski KA, Drubach LA, Lim R. Brown adipose tissue F-18-FDG uptake in pediatric PET/CT imaging. Pediatric Radiology 2011;41(6):759-68. Off topic
Ref ID: 5350

(2295) Hong Wd, Dong Lm, Jiang Zc, Zhu Qh, Jin SQ. Prediction of large esophageal varices in cirrhotic patients using classification and regression tree analysis. Clinics 2011;66(1):119-24. Off topic
Ref ID: 4218

(2296) Hopkins ME, Davis FC, Vantieghem MR, Whalen PJ, Bucci DJ. Differential Effects of Acute and Regular Physical Exercise on Cognition and Affect.

Neuroscience 2012;215:59-68. Study limited to adults
Ref ID: 5351

(2297) Hopkins N, Stratton G, Maia J, Tinken TM, Graves LE, Cable TN, Green DJ. Heritability of Arterial Function, Fitness, and Physical Activity in Youth: A Study of Monozygotic and Dizygotic Twins. Journal of Pediatrics 2010;157(6):943-8. Inappropriate Outcomes
Ref ID: 5352

(2298) Hopkins ND, Green DJ, Tinken TM, Sutton L, Mcwhannell N, Thijssen DHJ, Cable NT, Stratton G, George K. Does conduit artery diameter vary according to the anthropometric characteristics of children or men? American Journal of Physiology-Heart and Circulatory Physiology 2009;297(6):H2182-H2187. Not an exercise intervention study
Ref ID: 5353

(2299) Hopkins ND, Stratton G, Tinken TM, Ridgers ND, Graves LE, Mcwhannell N, Cable NT, Green DJ. Seasonal Reduction in Physical Activity and Flow-Mediated Dilation in Children. Medicine and Science In Sports and Exercise 2011;43(2):232-8. Not an exercise intervention study
Ref ID: 5354

(2300) Hopkins ND, Stratton G, Cable NT, Tinken TM, Graves LEF, Green DJ. Impact of exercise training on endothelial function and body composition in young people: a study of mono- and di-zygotic twins. European Journal of Applied Physiology 2012;112(2):421-7. No control group (NC)
Ref ID: 5355

(2301) Hoppel AM. The mandate debate. (Cover story). Clinician Reviews 2010 October;20(10):1-30. Review article
Ref ID: 3632

(2302) Hopper CA, Gruber MB, Munoz KD, Herb RA. Effect of including parents in a school-based exercise and nutrition program for children. Research Quarterly for Exercise and Sport 1992 September;63(3):315-21. Diet & Exercise intervention
Ref ID: 2287

(2303) Hopper CA, Munoz KD, Gruber MB, Nguyen KP. The effects of a family fitness program on the physical activity and nutrition behaviors of third-grade children. Research Quarterly for Exercise and Sport 2005 June;76(2):130-9. Diet & Exercise intervention
Ref ID: 1478

(2304) Hordern MD, Cooney LM, Beller EM, Prins JB, Marwick TH, Coombes JS. Determinants of changes in blood glucose response to short-term exercise training in patients with Type 2 diabetes. Clinical Science 2008

November;115(9):273-81. Study limited to adults
Ref ID: 885

(2305) Hordern MD, Marwick TH, Wood P, Cooney LM, Prins JB, Coombes JS. Acute response of blood glucose to short-term exercise training in patients with type 2 diabetes. Journal of Science and Medicine in Sport 2011 May;14(3):238-42. Not a randomized controlled trial (RCT)
Ref ID: 2586

(2306) Horgan R. Piglet castration and EU animal welfare legislation. Acta Veterinaria Scandinavica 2006 January 2;48:S2-S4. Animal study
Ref ID: 3633

(2307) Horn OK, Paradis G, Potvin L, Macaulay AC, Desrosiers S. Correlates and predictors of adiposity among Mohawk children. Preventive Medicine 2001 October;33(4):274-81. Cross-sectional study
Ref ID: 1894

(2308) Horner FE, Rayson MP, Bilzon JLJ. Reliability and validity of the 3DNX accelerometer during mechanical and human treadmill exercise testing. International Journal of Obesity 2011;35:S88-S97. Off topic
Ref ID: 5356

(2309) Hornery DJ, Farrow D, Mujika I, Young WB. Caffeine, carbohydrate, and cooling use during prolonged simulated tennis. International Journal of Sports Physiology and Performance 2007 December;2(4):423-38. Off topic
Ref ID: 1111

(2310) Horodynski MA, Baker S, Coleman G, Auld G, Lindau J. The Healthy Toddlers Trial Protocol: an intervention to reduce risk factors for childhood obesity in economically and educationally disadvantaged populations. BMC Public Health 2011;11:581. Diet Intervention Study
Ref ID: 4834

(2311) Horodynski MA, Olson B, Baker S, Brophy HH, Auld G, Van EL, Lindau J, Singleterry L. Healthy babies through infant-centered feeding protocol: an intervention targeting early childhood obesity in vulnerable populations. BMC Public Health 2011;11:868. Subjects less than 2 years old
Ref ID: 4833

(2312) Horvath SM, Agnew JW, Wagner JA, Bedi JF. Maximal aerobic capacity at several ambient concentrations of carbon monoxide at several altitudes. Research Report - Health Effects Institute (21):1-21, 1988 Dec 1988 December;(21):1-21. Off topic
Ref ID: 2327

(2313) Horvath V, Majlij D, Seguel B, Whittle P, Mackinnon D, Niedmann E. JP, Baldassare P, González M, Soffia C. Apendicitis epiploica primaria: diagnóstico

clínico y radiológico. Revista médica de Chile 2000 June;128(6):601-7. Off topic
Ref ID: 4219

(2314) Hostetter MK, Iverson S, Thomas W, Mckenzie D, Dole K, Johnson DE. Medical Evaluation of Internationally Adopted-Children. New England Journal of Medicine 1991;325(7):479-85. Off topic
Ref ID: 5357

(2315) Hostmark AT, Ekeland GS, Beckstrom AC, Meen HD. Postprandial light physical activity blunts the blood glucose increase. Preventive Medicine 2006 May;42(5):369-71. Study limited to adults
Ref ID: 1389

(2316) Hoti SL, Pani SP, Vanamail P, Athisaya MK, Das LK, Das PK. Effect of a single dose of diethylcarbamazine, albendazole or both on the clearance of Wuchereria bancrofti microfilariae and antigenaemia among microfilaria carriers: a randomized trial. National Medical Journal of India 2010 March;23(2):72-6. Drug intervention study
Ref ID: 2587

(2317) Houde S, Filiatrault M, Fournier A, Dube J, D'Arcy S, Berube D, Brousseau Y, Lapierre G, Vanasse M. Deflazacort use in Duchenne muscular dystrophy: an 8-year follow-up. Pediatric Neurology 2008 March;38(3):200-6. Drug intervention study
Ref ID: 996

(2318) Hovorka R, Allen JM, Elleri D, Chassin LJ, Harris J, Xing D, Kollman C, Hovorka T, Larsen AM, Nodale M, De PA, Wilinska ME, Acerini CL, Dunger DB. Manual closed-loop insulin delivery in children and adolescents with type 1 diabetes: a phase 2 randomised crossover trial. Lancet 2010 February 27;375(9716):743-51. Not an exercise intervention study
Ref ID: 61

(2319) Howard BV. Insulin resistance and lipid metabolism. [Review] [25 refs]. American Journal of Cardiology 1999 July 8;84(1A):28J-32J. Review article
Ref ID: 2010

(2320) Hoybye C. Endocrine and metabolic aspects of adult Prader-Willi syndrome with special emphasis on the effect of growth hormone treatment. Growth Horm IGF Res 2004 February;14(1):1-15. Not an exercise intervention study
Ref ID: 343

(2321) Hoyer E, Jahnsen R, Stanghelle JK, Strand LI. Body weight supported treadmill training versus traditional training in patients dependent on walking assistance after stroke: a randomized controlled trial. Disability and Rehabilitation 2012;34(3):210-9. Off topic
Ref ID: 2588

(2322) Hoyos C, I, Jago R, Sebire S. Individual and social predictors of screen-viewing among Spanish school children. European Journal of Pediatrics 2011 January;170(1):93-102. Off topic
Ref ID: 2589

(2323) Hoyt RW, Knapik JJ, Lanza JF, Jones BH, Staab JS. Ambulatory Foot Contact Monitor to Estimate Metabolic Cost of Human Locomotion. Journal of Applied Physiology 1994;76(4):1818-22. Off topic
Ref ID: 5358

(2324) Hsieh CH. The effects of auricular acupressure on weight loss and serum lipid levels in overweight adolescents. Am J Chin Med 2010;38(4):675-82. Not an exercise intervention study
Ref ID: 33

(2325) Hsieh CH, Su TJ, Fang YW, Chou PH. Effects of auricular acupressure on weight reduction and abdominal obesity in Asian young adults: a randomized controlled trial. American Journal of Chinese Medicine 2011;39(3):433-40. Off topic
Ref ID: 2590

(2326) Hsu CH, Hwang KC, Chao CL, Chang HH, Chou P. Electroacupuncture in obese women: a randomized, controlled pilot study. J Womens Health (Larchmt) 2005 June;14(5):434-40. Study limited to adults
Ref ID: 308

(2327) Hsu PC, Lai TJ, Guo NW, Lambert GH, Guo YLL. Serum hormones in boys prenatally exposed to polychlorinated biphenyls and dibenzofurans. Journal of Toxicology and Environmental Health-Part A-Current Issues 2005;68(17-18):1447-56. Off topic
Ref ID: 5359

(2328) Hu JF, Zhao XH, Parpia B, Chen JS, Campbell TC. Assessment of a modified household food weighing method in a study of bone health in China. European Journal of Clinical Nutrition 1994 June;48(6):442-52. Off topic
Ref ID: 2236

(2329) Hu M, Wang B, Zhao HB, Liang HS, Zhang CQ, Zhao XL. [Prophylactic medication of Osteoking for the incidence of femoral head necrosis and the degree of weight loading-induced hip ache half a year after the operation of femoral neck fracture]. Zhongguo Linchuang Kangfu 2005;9:158-9. Off topic
Ref ID: 4836

(2330) Huang JS, Norman GJ, Zabinski MF, Calfas K, Patrick K. Body image and self-esteem among adolescents undergoing an intervention targeting dietary and physical activity behaviors. Journal of Adolescent Health 2007 March;40(3):245-51. No exercise only group
Ref ID: 247

(2331) Huang JS, Dillon L, Terrones L, Schubert L, Roberts W, Finklestein J, Swartz MC, Norman GJ, Patrick K. Fit4Life: a weight loss intervention for children who have survived childhood leukemia. Pediatr Blood Cancer 2014 May;61(5):894-900. Inappropriate Intervention
Ref ID: 5891

(2332) Huang LC, Pan WY. [Comparation of effect and cost-benefit analysis between acupoint catgut-embedding and electroacupuncture on simple obesity]. [Chinese]. Zhongguo Zhenjiu 2011 October;31(10):883-6. Inappropriate Outcomes
Ref ID: 2591

(2333) Huang R, Ho SY, Lo WS, Lam TH. Assessment of weight-related factors of adolescents by private practitioners. BMC Family Practice 2013;14:141. Inappropriate Study Design
Ref ID: 5892

(2334) Huang R, Ho SY, Lo WS, Lam TH. Physical activity and constipation in Hong Kong adolescents. PLoS ONE 2014;9(2):e90193. Inappropriate Study Design
Ref ID: 5893

(2335) Huang SH, Weng KP, Hsieh KS, Ou SF, Lin CC, Chien KJ, Liu PY, Ho TY. Effects of a classroom-based weight-control intervention on cardiovascular disease in elementary-school obese children. Acta Paediatrica Taiwanica 2007 July;48(4):201-6. No exercise only group
Ref ID: 199

(2336) Huang TT, Yeh CY, Tsai YC. A diet and physical activity intervention for preventing weight retention among Taiwanese childbearing women: a randomised controlled trial. Midwifery 2011 April;27(2):257-64. Diet & Exercise intervention
Ref ID: 2592

(2337) Huey SJ, Polo AJ. Evidence-Based Psychosocial Treatments for Ethnic Minority Youth. Journal of Clinical Child and Adolescent Psychology 2008 January;37(1):262-301. Review article
Ref ID: 3824

(2338) Hughes AR, Henderson A, Ortiz-Rodriguez V, Artinou ML, Reilly JJ. Habitual physical activity and sedentary behaviour in a clinical sample of obese children. International Journal of Obesity 2006 October;30(10):1494-500. Not All Participants were Overweight and/or Obese
Ref ID: 1345

(2339) Hughes AR, Stewart L, Chapple J, McColl JH, Donaldson MD, Kelnar CJ, Zabihollah M, Ahmed F, Reilly JJ. Randomized, controlled trial of a best-practice individualized behavioral program for treatment of childhood overweight: Scottish Childhood Overweight Treatment Trial (SCOTT).

Pediatrics 2008 March;121(3):e539-e546. No exercise only group
Ref ID: 192

(2340) Hujova Z, Alberty R, Ahlers I, Ahlersova E, Paulikova E, Desatnikova J, Gabor D, Hruba F. Cardiovascular risk predictors in central Slovakian Roma children and adolescents: regional differences. Central European Journal of Public Health 2010 September;18(3):139-44. Cross-sectional study
Ref ID: 2593

(2341) Hultquist CM, Meyers AW, Whelan JP, Klesges RC, Peacher-Ryan H, DeBon MW. The effect of smoking and light activity on metabolism in men. Health Psychology 1995 March;14(2):124-31. Off topic
Ref ID: 2205

(2342) Hume C, Okely A, Bagley S, Telford A, Booth M, Crawford D, Salmon J. Does weight status influence associations between children's fundamental movement skills and physical activity? Research Quarterly for Exercise and Sport 2008 June;79(2):158-65. Cross-sectional study
Ref ID: 909

(2343) Hume C, Singh A, Brug J, Mechelen W, Chinapaw M. Dose-response associations between screen time and overweight among youth. International Journal of Pediatric Obesity 2009;4(1):61-4. Not a randomized controlled trial (RCT), Observational study
Ref ID: 173

(2344) Humenikova L, Gates GE. Dietary intakes, physical activity, and predictors of child obesity among 4-6th graders in the Czech Republic. Central European Journal of Public Health 2007 March;15(1):23-8. Cross-sectional study
Ref ID: 1235

(2345) Humphries B, Fenning A, Dugan E, Guinane J, MacRae K. Whole-body vibration effects on bone mineral density in women with or without resistance training. Aviation Space and Environmental Medicine 2009 December;80(12):1025-31. Off topic
Ref ID: 623

(2346) Humphries MC, Gutin B, Barbeau P, Vemulapalli S, Allison J, Owens S. Relations of adiposity and effects of training on the left ventricle in obese youths. Medicine and Science in Sports and Exercise 2002 September;34(9):1428-35. No comparative control group
Ref ID: 373

(2347) Hunter GW, Xu JC, Biaggi-Labiosa AM, Laskowski D, Dutta PK, Mondal SP, Ward BJ, Makel DB, Liu CC, Chang CW, Dweik RA. Smart sensor systems for human health breath monitoring applications. Journal of Breath Research 2011;5(3). Off topic
Ref ID: 5360

(2348) Hunter RP, Isaza R, Carpenter JW, Koch DE. Clinical effects and plasma concentrations of fentanyl after transmucosal administration in three species of great ape. Journal of Zoo and Wildlife Medicine 2004;35(2):162-6. Animal study
Ref ID: 5361

(2349) Hurst DL, Lajara-Nanson W. Use of modafinil in spastic cerebral palsy. Journal of Child Neurology 2002 March;17(3):169-72. Drug intervention study
Ref ID: 1822

(2350) Hursting SD, Lashinger LM, Wheatley KW, Rogers CJ, Colbert LH, Nunez NP, Perkins SN. Reducing the weight of cancer: Mechanistic targets for breaking the obesity-carcinogenesis link. Best Practice and Research Clinical Endocrinology and Metabolism 2008;22(4):659-69. Off topic
Ref ID: 5362

(2351) Hussey J, Bell C, Bennett K, O'Dwyer J, Gormley J. Relationship between the intensity of physical activity, inactivity, cardiorespiratory fitness and body composition in 7-10-year-old Dublin children. British Journal of Sports Medicine 2007 May;41(5):311-6. Cross-sectional study
Ref ID: 1244

(2352) Hutchesson MJ, Hulst J, Collins CE. Weight management interventions targeting young women: a systematic review. Journal of the Academy of Nutrition and Dietetics 2013 June;113(6):795-802. Inappropriate Study Design
Ref ID: 5894

(2353) Hutchesson MJ, Collins CE, Morgan PJ, Watson JF, Guest M, Callister R. Changes to dietary intake during a 12-week commercial web-based weight loss program: a randomized controlled trial. European Journal of Clinical Nutrition 2014 January;68(1):64-70. Inappropriate Intervention
Ref ID: 5895

(2354) Hutler M, Schnabel D, Staab D, Tacke A, Wahn U, Boning D, Beneke R. Effect of growth hormone on exercise tolerance in children with cystic fibrosis. Medicine and Science in Sports and Exercise 2002 April;34(4):567-72. Drug intervention study
Ref ID: 1843

(2355) Hübner ME, Ramírez R. Sobrevida, viabilidad y pronóstico del prematuro. Revista médica de Chile 2002 August;130(8):931-8. Subjects less than 2 years old
Ref ID: 711

(2356) Hwang J, Kim YH. Physical activity and its related motivational attributes in adolescents with different BMI. International Journal of Behavioral Medicine 2013 March;20(1):106-13. Inappropriate Study Design
Ref ID: 5896

(2357) Hystad HT, Steinsbekk S, Odegard R, Wichstrom L, Gudbrandsen OA. A randomised study on the effectiveness of therapist-led v. self-help parental intervention for treating childhood obesity. British Journal of Nutrition 2013 September 28;110(6):1143-50. Inappropriate Intervention
Ref ID: 5897

(2358) Hyzyk AK, Regula J, Jeszka J. [Evaluation of total energy balance and food habits of obese children]. [Polish]. Medycyna Wieku Rozwojowego 2000 April;4(2):109-18. Not All Participants were Overweight and/or Obese
Ref ID: 1957

(2359) Iannaccone ST. Meeting Abstracts. Journal of Child Neurology 2002 March;17(3):206. Abstract
Ref ID: 3634

(2360) Iannitti T, Palmieri B. The obese patient: clinical effectiveness of a high-protein low-calorie diet and its usefulness in the field of surgery. [Review] [194 refs]. Minerva Gastroenterologica e Dietologica 2010 June;56(2:Suppl 1):Suppl-65. Review article
Ref ID: 542

(2361) Ibarra P, Alarcón R. Mal nutrición por exceso en escolares.
Malnutrition by excess among school-age children. Revista Chilena de Pediatría 2010 December;81(6):506-14. Diet Intervention Study
Ref ID: 4220

(2362) Ibáñez L, Baar Z, Gana A. Cambios fisiológicos de la rotación de la marcha durante el desarrollo. Revista Chilena de Pediatría 2008 February;79(1):45-9. Off topic
Ref ID: 4221

(2363) Ibrahim AI, Hawamdeh ZM, Alsharif AA. Evaluation of bone mineral density in children with perinatal brachial plexus palsy: effectiveness of weight bearing and traditional exercises. Bone 2011 September;49(3):499-505. Not All Participants were Overweight and/or Obese
Ref ID: 2594

(2364) Ickovics JR, Carroll-Scott A, Peters SM, Schwartz M, Gilstad-Hayden K, McCaslin C. Health and academic achievement: cumulative effects of health assets on standardized test scores among urban youth in the United States. Journal of School Health 2014 January;84(1):40-8. Inappropriate Study Design
Ref ID: 5898

(2365) Ievers-Landis CE, Burant C, Drotar D, Morgan L, Trapl ES, Kent KC. Social support, knowledge, and self-efficacy as correlates of osteoporosis preventive behaviors among preadolescent females. Journal of Pediatric Psychology 2003;28:335-45. Behavior Modification Intervention
Ref ID: 4838

(2366) Ildiko V, Zsofia M, Janos M, Andreas P, Dora NE, Andras P, Agnes S, Zsolt S, Kumagai S. Activity-related changes of body fat and motor performance in obese seven-year-old boys. J Physiol Anthropol 2007 May;26(3):333-7. Not a randomized controlled trial (RCT)
Ref ID: 225

(2367) Im HS. [Yakson vs. GHT therapy effects on growth and physical response of preterm infants and on maternal attachment]. Taehan Kanho Hakhoe chi 2006;36:255-64. Drug intervention study
Ref ID: 4840

(2368) Impellizzeri FM, Rampinini E, Maffiuletti NA, Castagna C, Bizzini M, Wisloff U. Effects of aerobic training on the exercise-induced decline in short-passing ability in junior soccer players. Applied Physiology, Nutrition, and Metabolism = Physiologie Appliquee, Nutrition et Metabolisme 2008 December;33(6):1192-8. Off topic
Ref ID: 837

(2369) Inelmen EM, Toffanello ED, Enzi G, Gasparini G, Miotto F, Sergi G, Busetto L. Predictors of drop-out in overweight and obese outpatients. International Journal of Obesity 2005 January;29(1):122-8. Retrospective study
Ref ID: 1556

(2370) Ingle L, Sleap M, Tolfrey K. The effect of a complex training and detraining programme on selected strength and power variables in early pubertal boys. Journal of Sports Sciences 2006 September;24(9):987-97. Not All Participants were Overweight and/or Obese
Ref ID: 1363

(2371) Interdonato GC, Greguol M. Qualidade de vida e prática habitual de atividade física em adolescentes com deficiência. Quality of life and physical activity in adolescents with disabilities. Revista Brasileira de Crescimento e Desenvolvimento Humano 2011;21(2):282-95. Survey or questionnaire
Ref ID: 4222

(2372) Iotti Neto L. Obesidade: relaçöes de causas e efeitos; Relaçöes of obesity: causes and effects. J bras med 1996 August;71(2):100, 102, 104. Review article
Ref ID: 798

(2373) Ip WY, Tang CS, Goggins WB. An educational intervention to improve womenâ€™s ability to cope with childbirth. Journal of Clinical Nursing 2009 August;18(15):2125-35. Educational intervention
Ref ID: 3635

(2374) Irani BG, Xiang Z, Moore MC, Mandel RJ, Haskell-Luevano C. Voluntary exercise delays monogenetic obesity and overcomes reproductive dysfunction of the melanocortin-4 receptor knockout mouse. Biochemical and Biophysical

Research Communications 2005;326(3):638-44. Animal study
Ref ID: 5363

(2375) Iriart JAB, Chaves JC, Orleans RGd. Culto ao corpo e uso de anabolizantes entre praticantes de musculação. Cadernos de Saúde Pública 2009 April;25(4):773-82. Off topic
Ref ID: 4223

(2376) Iser BP, Yokota RT, de Sa NN, de ML, Malta DC. [Protection from chronic diseases and the prevalence of risk factors in Brazilian state capitals--main results from Vigitel 2010]. Cien Saude Colet 2012 September;17(9):2343-56. Inappropriate Study Design
Ref ID: 5899

(2377) Iser BPM, Claro RM, Moura ECd, Malta DC, Morais Neto OL. Fatores de risco e proteção para doenças crônicas não transmissíveis obtidos por inquérito telefônico - VIGITEL Brasil – 2009. Risk and protection factors for chronic non communicable diseases by telephone survey - VIGITEL - 2009. Revista Brasileira de Eidemiologia 2011 September;14(supl.1):90-102. Off topic
Ref ID: 4224

(2378) Isfort M, Brühl A, Bünte A, Jorch G, Kray A. [Contributions and effects of parental basal stimulation contact care within the scope of gentle neonatal nursing care--II]. Kinderkrankenschwester : Organ der Sektion Kinderkrankenpflege / Deutsche Gesellschaft für Sozialpädiatrie und Deutsche Gesellschaft für Kinderheilkunde 2008;27:272-82. Off topic
Ref ID: 4841

(2379) Isharwal S, Misra A, Wasir JS, Nigam P. Diet & insulin resistance: A review & Asian Indian perspective. Indian Journal of Medical Research 2009 May;129(5):485-99. Review article
Ref ID: 3636

(2380) Ishibashi M, Maurino Junior D, Junior L. Glicemia e insulinemia em crianças e adolescentes obesos após 12 semanas de treinamento físico. Revista Paulista de Pediatria 2007 March;25(1):33-7. No non-intervention control group
Ref ID: 4225

(2381) Islam S, Gutin B, Treiber F, Hobbs G, Kamboh I, Lopes-Virella M. Association of apolipoprotein A phenotypes and oxidized low-density lipoprotein immune complexes in children. Archives of Pediatrics and Adolescent Medicine 1999 January;153(1):57-62. Survey or questionnaire
Ref ID: 2052

(2382) Israel AC, Guile CA, Baker JE, Silverman WK. An evaluation of enhanced self-regulation training in the treatment of childhood obesity. Journal of Pediatric Psychology 1994;19:737-49. Behavior Modification Intervention
Ref ID: 4843

(2383) Israsena T, Israngkura M, Srivuthana S. Treatment of childhood obesity. Journal of the Medical Association of Thailand 1980 August;63(8):433-7. Review article
Ref ID: 2377

(2384) Ito PS, Saffar RDVP, Oliveira TA, Souza Ed. Associaçäo entre parto pré-termo com atividade física e sexual; Associaçäo between pre-term labor with physical and sexual activity. Femina 2002 May;30(4):227-30. Off topic
Ref ID: 4226

(2385) Itomura M, Hamazaki K, Sawazaki S, Kobayashi M, Terasawa K, Watanabe S, Hamazaki T. The effect of fish oil on physical aggression in schoolchildren--a randomized, double-blind, placebo-controlled trial. The Journal of nutritional biochemistry 2005;16:163-71. Diet Intervention or Supplement Study
Ref ID: 4844

(2386) Itou M, Kawaguchi T, Taniguchi E, Oriishi T, Suetsugu T, Hano R, Yoshihara M, Takishita M, Eguchi M, Ogiwara Y, Otsuyama J, Mutou M, Ibi R, Akiyama R, Ono M, Otsuka M, Nagamatsu A, Tanaka S, Miyakoda K, Emori K, Morita Y, Kumamoto M, Tsuruta O, Sata M. Supplementation before endoscopic therapy for esophageal varices reduces mental stress in patients with liver cirrhosis. Hepato-Gastroenterology 2011 May;58(107-108):814-8. Diet Intervention or Supplement Study
Ref ID: 2595

(2387) Iughetti L, Predieri B, Bruzzi P, Balli F. Approaches to dyslipidemia treatment in children and adolescents. Expert Review of Endocrinology and Metabolism 2008;3(5):615-33. Review article
Ref ID: 3243

(2388) Ivanovic Marincovich D. Nutrition and Education: IV. Clinical signs of malnutrition and its relationship with socioeconomic, anthropometric, dietetic and educational achievement parameters. Archivos Latinoamericanos de Nutrición 1992 March;42(1):15-25. Diet Intervention Study
Ref ID: 4227

(2389) Ivanovic Marincovich D, Ivanovic Marincovich R, Durán Santana MC, Hazbún Game J. Ingesta alimentaria de escolares rurales de la región metropolitana de Chile: un estudio comparativo: 1989. Archivos Latinoamericanos de Nutrición 1992 September;42(4):374-88. Diet Intervention Study
Ref ID: 810

(2390) Iwamoto J. [Effect of exercise on developing bone mass and cortical bone geometry]. [Review] [Japanese]. Clinical Calcium 2011 September;21(9):1323-8. Review article
Ref ID: 2596

(2391) Jaber R. Respiratory and allergic diseases: from upper respiratory tract infections to asthma. [Review] [216 refs]. Primary Care; Clinics in Office Practice 2002 June;29(2):231-61. Review article
Ref ID: 1796

(2392) Jackson EM, Dishman RK. Hemodynamic responses to stress among black women: fitness and parental hypertension. Medicine and Science in Sports and Exercise 2 A.D.;34(7):1097-104. Study limited to adults
Ref ID: 1806

(2393) Jackson KA, Berg JM, Murray JD, Maga EA. Evaluating the fitness of human lysozyme transgenic dairy goats: growth and reproductive traits. Transgenic Research 2010;19(6):977-86. Animal study
Ref ID: 5364

(2394) Jacobs KA, Krauss RM, Fattor JA, Horning MA, Friedlander AL, Bauer TA, Hagobian TA, Wolfel EE, Brooks GA. Endurance training has little effect on active muscle free fatty acid, lipoprotein cholesterol, or triglyceride net balances. American Journal of Physiology - Endocrinology and Metabolism 2006 September;291(3):E656-E665. Study limited to adults
Ref ID: 1359

(2395) Jacobson JL, Jacobson SW. A 4-Year Follow-Up-Study of Children Born to Consumers of Lake-Michigan Fish. Journal of Great Lakes Research 1993;19(4):776-83. Off topic
Ref ID: 5365

(2396) Jacqueminet S, Jannot-Lamotte MF. Management of gestational diabetes. Journal de Gynecologie Obstetrique et Biologie de la Reproduction 2010;39(8):S251-S263. Off topic
Ref ID: 5367

(2397) Jacqueminet S, Jannot-Lamotte MF. Therapeutic management of gestational diabetes. Diabetes and Metabolism 2010;36(6):658-71. Review article
Ref ID: 5366

(2398) Jago R, Baranowski T. Non-curricular approaches for increasing physical activity in youth: a review. [Review] [48 refs]. Preventive Medicine 2004 July;39(1):157-63. Review article
Ref ID: 1616

(2399) Jago R, Baranowski T, Yoo S, Cullen KW, Zakeri I, Watson K, Himes JH, Pratt C, Sun W, Pruitt LA, Matheson DM. Relationship between physical activity and diet among African-American girls. Obesity Research 2004 September;12 Suppl:55S-63S.:55S-63S. Not an exercise intervention study, Observational study
Ref ID: 323

(2400) Jago R, Baranowski T, Baranowski JC, Thompson D, Cullen KW, Watson K, Liu Y. Fit for Life Boy Scout badge: outcome evaluation of a troop and Internet intervention. Preventive Medicine 2006 March;42(3):181-7. Not All Participants were Overweight and/or Obese
Ref ID: 1404

(2401) Jago R, Jonker ML, Missaghian M, Baranowski T. Effect of 4 weeks of Pilates on the body composition of young girls. Preventive Medicine 2006 March;42(3):177-80. Not All Participants were Overweight and/or Obese
Ref ID: 291

(2402) Jago R, McMurray RG, Drews KL, Moe EL, Murray T, Pham TH, Venditti EM, Volpe SL. HEALTHY intervention: fitness, physical activity, and metabolic syndrome results. Medicine and Science in Sports and Exercise 2011 August;43(8):1513-22. Multiple interventions
Ref ID: 2597

(2403) Jago R, Mendoza JA, Chen T, Baranowski T. Longitudinal associations between BMI, waist circumference, and cardiometabolic risk in US youth: monitoring implications. Obesity (Silver Spring) 2013 March;21(3):E271-E279. Inappropriate Study Design
Ref ID: 5900

(2404) Jago R, Drews KL, Otvos JD, Foster GD, Marcus MD, Buse JB, Mietus-Snyder M, Willi SM. Effect of relative weight group change on nuclear magnetic resonance spectroscopy derived lipoprotein particle size and concentrations among adolescents. Journal of Pediatrics 2014 May;164(5):1091-8. Inappropriate Study Design
Ref ID: 5901

(2405) Jagomagi G, Jurimae T. The influence of anthropometrical and flexibility parameters on the results of breaststroke swimming. Anthropologischer Anzeiger 2005 June;63(2):213-9. Off topic
Ref ID: 1502

(2406) Jahari AB, Saco PC, Husaini MA, Pollitt E. Effects of an energy and micronutrient supplement on motor development and motor activity in undernourished children in Indonesia. European Journal of Clinical Nutrition 2000;54 Suppl 2:S60-S68. Diet Intervention or Supplement Study
Ref ID: 4845

(2407) Jakicic JM, Otto AD, Lang W, Semler L, Winters C, Polzien K, Mohr KI. The effect of physical activity on 18-month weight change in overweight adults. Obesity 2011 January;19(1):100-9. Study limited to adults
Ref ID: 2598

(2408) Jalava M, Sillanpaa M. Physical activity, health-related fitness, and health experience in adults with childhood-onset epilepsy: a controlled study. Epilepsia

1997 April;38(4):424-9. Study limited to adults
Ref ID: 2144

(2409) Jallad RS, Liberman B, Vianna CB, Vieira ML, Ramires JA, Knoepfelmacher M. Effects of growth hormone replacement therapy on metabolic and cardiac parameters, in adult patients with childhood-onset growth hormone deficiency. Growth Hormone and Igf Research 2003 April;13(2-3):81-8. Drug intervention study
Ref ID: 1748

(2410) Jamaty C, Bailey B, Larocque A, Notebaert E, Sanogo K, Chauny JM. Lipid emulsions in the treatment of acute poisoning: a systematic review of human and animal studies. Clinical Toxicology (15563650) 2010 January;48(1):1-27. Off topic
Ref ID: 3637

(2411) James FW, Kaplan S, Schwartz DC, Chou TC, Sandker MJ, Naylor V. Response to exercise in patients after total surgical correction of Tetralogy of Fallot. Circulation 1976 October;54(4):671-9. Off topic
Ref ID: 2389

(2412) James RA, Hertz-Picciotto I, Willman E, Keller JA, Charles MJ. Determinants of serum polychlorinated biphenyls and organochlorine pesticides measured in women from the Child Health and Development Study cohort, 1963-1967. Environmental Health Perspectives 2002;110(7):617-24. Off topic
Ref ID: 5368

(2413) James S, Vorster HH, Venter CS, Kruger HS, Nell TA, Veldman FJ, Ubbink JB. Nutritional status influences plasma fibrinogen concentration: evidence from the THUSA survey. Thrombosis Research 2000 June 1;98(5):383-94. Cohort Study
Ref ID: 1977

(2414) James WP. Achieving weight-loss maintenance. Postgrad Med 2001 June;109(6 Suppl):19-28. Not an exercise intervention study, Drug intervention study
Ref ID: 396

(2415) Janczyk W, Socha P, Lebensztejn D, Wierzbicka A, Mazur A, Neuhoff-Murawska J, Matusik P. Omega-3 fatty acids for treatment of non-alcoholic fatty liver disease: design and rationale of randomized controlled trial. BMC Pediatrics 2013;13:85. Inappropriate Study Design
Ref ID: 5902

(2416) Janicke DM, Sallinen BJ, Perri MG, Lutes LD, Silverstein JH, Huerta MG, Guion LA. Sensible treatment of obesity in rural youth (STORY): design and methods. Contemporary Clinical Trials 2008 March;29(2):270-80. Description versus conduct of study
Ref ID: 233

(2417) Janicke DM, Lim CS, Perri MG, Bobroff LB, Mathews AE, Brumback BA, Dumont-Driscoll M, Silverstein JH. The Extension Family Lifestyle Intervention Project (E-FLIP for Kids): design and methods. Contemporary Clinical Trials 2011 January;32(1):50-8. Description versus conduct of study
Ref ID: 1100

(2418) Janicke DM. Treatment of pediatric obesity using a parent-only approach: a case example. Health Psychology 2013 March;32(3):345-50. Inappropriate Study Design
Ref ID: 5903

(2419) Janicke DM, Lim CS, Mathews AE, Shelnutt KP, Boggs SR, Silverstein JH, Brumback BA. The community-based healthy-lifestyle intervention for rural preschools (CHIRP) study: design and methods. Contemporary Clinical Trials 2013 March;34(2):187-95. Inappropriate Study Design
Ref ID: 5904

(2420) Jankowicz-Szymanska A, Mikolajczyk E, Wojtanowski W. The effect of physical training on static balance in young people with intellectual disability. Research in Developmental Disabilities 2012 March;33(2):675-81. Not All Participants were Overweight and/or Obese
Ref ID: 2600

(2421) Jankowski CM, Ben-Ezra V, Gozansky WS, Scheaffer SE. Effects of oral contraceptives on glucoregulatory responses to exercise. Metabolism: Clinical and Experimental 2004 March;53(3):348-52. Drug intervention study
Ref ID: 1662

(2422) Jannini SN, Dória-Filho U, Damiani D, Silva CAA. Dor músculo-esquelética em adolescentes obesos. Musculoskeletal pain in obese adolescents. Jornal de Pediatria 2011 August;87(4):329-35. Cross-sectional study
Ref ID: 4228

(2423) Jansen E, Mulkens S, Jansen A. Tackling childhood overweight: treating parents exclusively is effective. International Journal of Obesity 2011;35:501-9. Behavior Modification Intervention
Ref ID: 4846

(2424) Jansen H, Postma A, Stolk RP, Kamps WA. Acute lymphoblastic leukemia and obesity: increased energy intake or decreased physical activity? Supportive Care in Cancer 2009 January;17(1):103-6. Off topic
Ref ID: 819

(2425) Jansen W, Raat H, Zwanenburg EJ, Reuvers I, van WR, Brug J. A school-based intervention to reduce overweight and inactivity in children aged 6-12 years: study design of a randomized controlled trial. BMC Public Health 2008 July 25;8:257.:257. No exercise only group
Ref ID: 164

(2426) Jansen W, Borsboom G, Meima A, Zwanenburg EJ, Mackenbach JP, Raat H, Brug J. Effectiveness of a primary school-based intervention to reduce overweight. International Journal of Pediatric Obesity 2011 June;6(2-2):e70-e77. Not All Participants were Overweight and/or Obese
Ref ID: 2601

(2427) Jansi LB. Effect of oil massage on changes in weight and neurobehavioural response of low birth weight babies. Nursing Journal of India 99(11):256-8, 2008 Nov 2008;(11):256-8. Off topic
Ref ID: 3008

(2428) Janssen IM, Swank DJ, Boonstra O, Knipscheer BC, Klinkenbijl JH, van GH. Randomized clinical trial of ultrasonic versus electrocautery dissection of the gallbladder in laparoscopic cholecystectomy. British Journal of Surgery 2003 July;90(7):799-803. Off topic
Ref ID: 1733

(2429) Janssen M, Toussaint HM, Van WM, Verhagen EA. PLAYgrounds: effect of a PE playground program in primary schools on PA levels during recess in 6 to 12 year old children. Design of a prospective controlled trial. BMC Public Health 2011;11:282. Description versus conduct of study
Ref ID: 2602

(2430) Jaramillo García CM, Lopera Calderón MC, Zuluaga De Cadena Á, Manrique RD. Factores relacionados con la aparición de estrías atróficas en mujeres adolescentes de dos establecimientos educativos privados de la ciudad de Medellín, 1997-1998. Related factors with atrophic stretch marks in adolescent female students from two private educational establishments from the city of Medellin, 1997-1999. CES Medicine 2009 June;23(1,supl):69-79. Off topic
Ref ID: 4229

(2431) Jarus T, Gol D. The effect of kinesthetic stimulation on the acquisition and retention of a gross motor skill by children with and without sensory integration disorders. Physical and Occupational Therapy in Pediatrics 1994;14:59-73. No non-intervention control group
Ref ID: 4847

(2432) Jarvela LS, Niinikoski H, Lahteenmaki PM, Heinonen OJ, Kapanen J, Arola M, Kemppainen J. Physical activity and fitness in adolescent and young adult long-term survivors of childhood acute lymphoblastic leukaemia. Journal of Cancer Survivorship 2010 December;4(4):339-45. Study not limited to children and adolescents
Ref ID: 2603

(2433) Jarvis SS, Pawelczyk JA. Identification of the human electrical impedance indifferent point: a surrogate for the volume indifferent point? European Journal

of Applied Physiology 2009 November;107(4):473-80. Off topic
Ref ID: 662

(2434) JÃ¸rgensen JOL, Vahl N, Hansen TB, SkjÃ¦rbÃ¦k C, Fisker S, Ã˜rskov H, Hagen C, Christiansen J. Determinants of serum insulin-like growth factor I in growth hormone deficient adults as compared to healthy subjects. Clinical Endocrinology 1998 April;48(4):479-86. Study limited to adults
Ref ID: 557

(2435) JÃ¤ggin N, Gerber S, Schatzmann U. General anaesthesia, analgesia and pain associated with the castration of newborn piglets. Acta Veterinaria Scandinavica 2006 January 2;48:S12-S13. Animal study
Ref ID: 3638

(2436) Jeanes Y, Hart K. Current literature. Journal of Human Nutrition and Dietetics 2010 October;23(5):552-6. Abstract
Ref ID: 3639

(2437) Jeck-Thole S, Wagner W. Betahistine: A Retrospective Synopsis of Safety Data. Drug Safety 2006 August;29(11):1049. Retrospective study
Ref ID: 3640

(2438) Jelalian E, Saelens BE. Empirically supported treatments in pediatric psychology: pediatric obesity. [Review] [97 refs]. Journal of Pediatric Psychology 1999 June;24(3):223-48. Review article
Ref ID: 2014

(2439) Jelalian E, Mehlenbeck R, Lloyd-Richardson EE, Birmaher V, Wing RR. 'Adventure therapy' combined with cognitive-behavioral treatment for overweight adolescents. International Journal of Obesity (London) 2006 January;30(1):31-9. No comparative control group
Ref ID: 299

(2440) Jelalian E, Wember YM, Bungeroth H, Birmaher V. Practitioner review: bridging the gap between research and clinical practice in pediatric obesity. [Review] [98 refs]. Journal of Child Psychology and Psychiatry and Allied Disciplines 2007 February;48(2):115-27. Review article
Ref ID: 1274

(2441) Jelalian E, Hart CN, Mehlenbeck RS, Lloyd-Richardson EE, Kaplan JD, Flynn-O'Brien KT, Wing RR. Predictors of attrition and weight loss in an adolescent weight control program. Obesity (Silver Spring) 2008 June;16(6):1318-23. No comparative control group
Ref ID: 188

(2442) Jelalian E, Lloyd-Richardson EE, Mehlenbeck RS, Hart CN, Flynn-O'Brien K, Kaplan J, Neill M, Wing RR. Behavioral weight control treatment with supervised exercise or peer-enhanced adventure for overweight adolescents.

Journal of Pediatrics 2010 December;157(6):923-8. No comparative control group
Ref ID: 29

(2443) Jelsma JG, van Poppel MN, Galjaard S, Desoye G, Corcoy R, Devlieger R, van AA, Timmerman D, Jans G, Harreiter J, Kautzky-Willer A, Damm P, Mathiesen ER, Jensen DM, Andersen L, Dunne F, Lapolla A, Di CG, Bertolotto A, Wender-Oegowska E, Zawiejska A, Blumska K, Hill D, Rebollo P, Snoek FJ et al. DALI: Vitamin D and lifestyle intervention for gestational diabetes mellitus (GDM) prevention: an European multicentre, randomised trial - study protocol. BMC Pregnancy Childbirth 2013;13:142. Inappropriate Study Design
Ref ID: 5905

(2444) Jenkins DG, Palmer J, Spillman D. The influence of dietary carbohydrate on performance of supramaximal intermittent exercise. European Journal of Applied Physiology and Occupational Physiology 1993;67(4):309-14. Diet Intervention Study
Ref ID: 2249

(2445) Jenkins I, Djuric Z, Darga L, DiLaura NM, Magnan M, Hryniuk WM. Relationship of psychiatric diagnosis and weight loss maintenance in obese breast cancer survivors. Obesity Research 2003 November;11(11):1369-75. Study limited to adults
Ref ID: 350

(2446) Jenkins NT, Landers RQ, Thakkar SR, Fan X, Brown MD, Prior SJ, Spangenburg EE, Hagberg JM. Prior endurance exercise prevents postprandial lipaemia-induced increases in reactive oxygen species in circulating CD31+ cells. Journal of Physiology 2011 November 15;589(Pt:22):22-53. Study limited to adults
Ref ID: 2605

(2447) Jenni OG, Molinari L, Caflisch JA, Largo RH. Sleep duration from ages 1 to 10 years: Variability and stability in comparison with growth. Pediatrics 2007;120(4):e769-E776. Off topic
Ref ID: 5369

(2448) Jennings-Aburto N, Nava F, Bonvecchio A, Safdie M, González-Casanova I, Gust T, Rivera J. Physical activity during the school day in public primary schools in Mexico City. La actividad física durante la jornada escolar en escuelas primarias públicas en la Ciudad de México. Salud Pública de México 2009 April;51(2):141-7. Observational study
Ref ID: 4230

(2449) Jenovesi JF, Bracco MM, Colugnati FAB, Taddei JAdAC. Perfil de atividade física em escolares da rede pública de diferentes estados nutricionais. Revista Brasileira de Ciência e Movimento 2003;11(4):57-62. Not an exercise

intervention study
Ref ID: 4231

(2450) Jenovesi JF, Bracco MM, Colugnati FAB, Taddei JAdAC. Evolução no nível de atividade física de escolares observados pelo período de 1 ano. Revista Brasileira de Ciência e Movimento 2004;12(1):19-24. Not an exercise intervention study
Ref ID: 4232

(2451) Jensen BB. Prevention of boar taint in pig production. Factors affecting the level of skatole. Acta Veterinaria Scandinavica 2006 January 2;48:S6-4. Animal study
Ref ID: 3641

(2452) Jensen CD, Aylward BS, Steele RG. Predictors of attendance in a practical clinical trial of two pediatric weight management interventions. Obesity (Silver Spring) 2012 November;20(11):2250-6. Inappropriate Intervention
Ref ID: 5906

(2453) Jensen PB, Hansen TB, Oxhoj H, Froberg K, Ekelund B, Nielsen FT, Pedersen FB. What are the clinical benefits of correcting the catabolic state in haemodialysis patients? British Journal of Clinical Practice Supplement 1996 August;85:47-51. Off topic
Ref ID: 2152

(2454) Jeon JY, Lee PJ, Sato SI. Use of the standard rubber ball as an impact source with heavyweight concrete floors. Journal of the Acoustical Society of America 2009;126(1):167-78. Off topic
Ref ID: 5370

(2455) Ji CY, Chen TJ. Empirical changes in the prevalence of overweight and obesity among Chinese students from 1985 to 2010 and corresponding preventive strategies. Biomedical and Environmental Sciences 2013 January;26(1):1-12. Inappropriate Study Design
Ref ID: 5907

(2456) Jiang J, Rosenqvist U, Wang H, Greiner T, Ma Y, Toschke AM. Risk factors for overweight in 2- to 6-year-old children in Beijing, China. International Journal of Pediatric Obesity 2006;1(2):103-8. Prevalence study
Ref ID: 1301

(2457) Jiang J, Xia X, Greiner T, Wu G, Lian G, Rosenqvist U. The effects of a 3-year obesity intervention in schoolchildren in Beijing. Child: Care, Health and Development 2007 September;33(5):641-6. No exercise only group
Ref ID: 220

(2458) Jimenez-Pavon D, Ortega FB, Ruiz JR, Chillon P, Castillo R, Artero EG, Martinez-Gomez D, Vicente-Rodriguez G, Rey-Lopez JP, Gracia LA, Noriega

MJ, Moreno LA, Gonzalez-Gross M. Influence of socioeconomic factors on fitness and fatness in Spanish adolescents: the AVENA study. International Journal of Pediatric Obesity 2010 December;5(6):467-73. Cross-sectional study
Ref ID: 2607

(2459) Jimenez-Pavon D, Castillo MJ, Moreno LA, Kafatos A, Manios Y, Kondaki K, Ghin B, Zaccaria M, de HS, Widhalm K, Moln RD, Sj Str MM, Gonz Lez-Gross M, Ruiz JR. Fitness and fatness are independently associated with markers of insulin resistance in European adolescents; the HELENA study. International Journal of Pediatric Obesity 2011 August;6(3-4):253-60. Cross-sectional study
Ref ID: 2606

(2460) Jin F, Nieman DC, Shanely RA, Knab AM, Austin MD, Sha W. The variable plasma quercetin response to 12-week quercetin supplementation in humans. European Journal of Clinical Nutrition 2010 July;64(7):692-7. Diet Intervention or Supplement Study
Ref ID: 497

(2461) Jing J, Li XH, Feng LY, Wu Q, Wang ZC, Zeng SP, Gu YP, Cai XM. Massage and motion training for growth and development of infants. World Journal of Pediatrics 2007;3:295-9. Subjects less than 2 years old
Ref ID: 4848

(2462) Johannesson E, Simren M, Strid H, Bajor A, Sadik R. Physical activity improves symptoms in irritable bowel syndrome: a randomized controlled trial. American Journal of Gastroenterology 2011 May;106(5):915-22. Study limited to adults
Ref ID: 1108

(2463) Johnson-Askew WL, Fisher RA, Yaroch AL. Decision making in eating behavior: State of the science and recommendations for future research. Annals of Behavioral Medicine 2009;38:S88-S92. Review article
Ref ID: 5371

(2464) Johnson-Down L, O'Loughlin J, Koski KG, Gray-Donald K. High prevalence of obesity in low income and multiethnic schoolchildren: a diet and physical activity assessment. Journal of Nutrition 1997 December;127(12):2310-5. Cross-sectional study
Ref ID: 2117

(2465) Johnson CC, Nicklas TA, Arbeit ML, Harsha DW, Mott DS, Hunter SM, Wattigney W, Berenson GS. Cardiovascular intervention for high-risk families: the Heart Smart Program. Southern Medical Journal 1991;84:1305-12. Lifestyle Intervention
Ref ID: 4849

(2466) Johnson CC, Osganian SK, Budman SB, Lytle LA, Barrera EP, Bonura SR, Wu MC, Nader PR. CATCH: family process evaluation in a multicenter trial. Health Education Quarterly Supplement 2:S91-106, 1994 1994;S91-106. Multiple

interventions
Ref ID: 2247

(2467) Johnson CC, Murray DM, Elder JP, Jobe JB, Dunn AL, Kubik M, Voorhees C, Schachter K. Depressive symptoms and physical activity in adolescent girls. Medicine and Science in Sports and Exercise 2008 May;40(5):818-26. Cross-sectional study
Ref ID: 969

(2468) Johnson DE, Guthrie D, Smyke AT, Koga SF, Fox NA, Zeanah CH, Nelson CA. Growth and associations between auxology, caregiving environment, and cognition in socially deprived Romanian children randomized to foster vs ongoing institutional care. Archives of Pediatrics and Adolescent Medicine 2010;164:507-16. Subjects less than 2 years old
Ref ID: 4850

(2469) Johnson LC, Fisher G, Silvester LJ, Hofheins CC. Anabolic steroid: effects on strength, body weight, oxygen uptake and spermatogenesis upon mature males. Medicine and Science in Sports 1972;4(1):43-5. Drug intervention study
Ref ID: 2609

(2470) Johnson RK, Hildreth HG, Contompasis SH, Goran MI. Total energy expenditure in adults with cerebral palsy as assessed by doubly labeled water. Journal of the American Dietetic Association 1997;97(9):966-70. Study limited to adults
Ref ID: 5372

(2471) Johnson RK, Russ J, Goran MI. Physical activity related energy expenditure in children by doubly labeled water as compared with the Caltrac accelerometer. International Journal of Obesity 1998;22(11):1046-52. Not an exercise intervention study
Ref ID: 5373

(2472) Johnson SS, Paiva AL, Cummins CO, Johnson JL, Dyment SJ, Wright JA, Prochaska JO, Prochaska JM, Sherman K. Transtheoretical model-based multiple behavior intervention for weight management: effectiveness on a population basis. Preventive Medicine 2008 March;46(3):238-46. Study limited to adults
Ref ID: 210

(2473) Johnson WG, Hinkle LK, Carr RE, Anderson DA, Lemmon CR, Engler LB, Bergeron KC. Dietary and exercise interventions for juvenile obesity: long-term effect of behavioral and public health models. Obesity Research 1997 May;5(3):257-61. No exercise only group
Ref ID: 425

(2474) Johnston BC, Kanters S, Bandayrel K, Wu P, Naji F, Siemieniuk RA, Ball GD, Busse JW, Thorlund K, Guyatt G, Jansen JP, Mills EJ. Comparison of weight

loss among named diet programs in overweight and obese adults: a meta-analysis. Journal of the American Medical Association 2014 September 3;312(9):923-33. Inappropriate Study Design
Ref ID: 5908

(2475) Johnston CA, Tyler C, McFarlin BK, Poston WS, Haddock CK, Reeves R, Foreyt JP. Weight loss in overweight Mexican American children: a randomized, controlled trial. Pediatrics 2007 December;120(6):e1450-e1457. No exercise only group, No comparative control group
Ref ID: 456

(2476) Johnston CA, Tyler C, Fullerton G, Poston WS, Haddock CK, McFarlin B, Reeves RS, Foreyt JP. Results of an intensive school-based weight loss program with overweight Mexican American children. International Journal of Pediatric Obesity 2007;2(3):144-52. No exercise only group, No comparative control group
Ref ID: 211

(2477) Johnston CA, Tyler C, Fullerton G, McFarlin BK, Poston WS, Haddock CK, Reeves RS, Foreyt JP. Effects of a school-based weight maintenance program for Mexican-American children: results at 2 years.[Erratum appears in Obesity (Silver Spring). 2010 Mar;18(3):647 Note: Fullerton, Ginny [added]]. Obesity 2010 March;18(3):542-7. No exercise only group, No comparative control group
Ref ID: 580

(2478) Johnston CA, Tyler C, Fullerton G, McFarlin BK, Poston WS, Haddock CK, Reeves RS, Foreyt JP. Effects of a school-based weight maintenance program for Mexican-American children: results at 2 years. Obesity (Silver Spring) 2010 March;18(3):542-7. No exercise only group, No comparative control group
Ref ID: 102

(2479) Johnston CA, Tyler C, Palcic JL, Stansberry SA, Gallagher MR, Foreyt JP. Smaller weight changes in standardized body mass index in response to treatment as weight classification increases. Journal of Pediatrics 2011 April;158(4):624-7. Multiple interventions
Ref ID: 2611

(2480) Johnston CA, Fullerton G, Moreno JP, Tyler C, Foreyt JP. Evaluation of treatment effects in obese children with co-morbid medical or psychiatric conditions. Georgian Medical News (196-197):93-100, 2011 Jul-Aug 2011 July;(196-197):93-100. Not a randomized controlled trial (RCT)
Ref ID: 2610

(2481) Johnston CA, Moreno JP, Gallagher MR, Wang J, Papaioannou MA, Tyler C, Foreyt JP. Achieving long-term weight maintenance in Mexican-American adolescents with a school-based intervention. Journal of Adolescent Health

2013 September;53(3):335-41. Inappropriate Intervention
Ref ID: 5909

(2482) Jolliffe CJ, Janssen I. Vascular risks and management of obesity in children and adolescents. Vasc Health Risk Manage 2006;2(2):171-87. Review article
Ref ID: 3244

(2483) Jones D, Hoelscher DM, Kelder SH, Hergenroeder A, Sharma SV. Increasing physical activity and decreasing sedentary activity in adolescent girls - The Incorporating More Physical Activity and Calcium in Teens (IMPACT) Study. International Journal of Behavioral Nutrition and Physical Activity 2008;5:42. Cohort Study
Ref ID: 4851

(2484) Jones G, Ding CH, Glisson M, Hynes K, Ma DQ, Cicuttini F. Knee articular cartilage development in children: A longitudinal study of the effect of sex, growth, body composition, and physical activity. Pediatric Research 2003;54(2):230-6. Off topic
Ref ID: 5374

(2485) Jones LM, Legge M, Goulding A. Factor analysis of the metabolic syndrome in spinal cord-injured men. Metabolism: Clinical and Experimental 2004 October;53(10):1372-7. Study limited to adults
Ref ID: 1587

(2486) Jones ME, Bashford GM, Mann JM. Weight bearing and velocity in trans-tibial and trans-femoral amputees. Prosthetics and Orthotics International 1997 December;21(3):183-6. Review article
Ref ID: 2114

(2487) Jones PJ, Hoffer D, Jones D. Clinical nutrition: 7. Functional foods--more than just nutrition. CMAJ: Canadian Medical Association Journal 2002 June 11;166(12):1555. Review article
Ref ID: 3642

(2488) Jones RA, Okely AD, Collins CE, Morgan PJ, Steele JR, Warren JM, Baur LA, Cliff DP, Burrows T, Cleary J. The HIKCUPS trial: a multi-site randomized controlled trial of a combined physical activity skill-development and dietary modification program in overweight and obese children. BMC Public Health 2007;7:15. Description versus conduct of study
Ref ID: 1279

(2489) Jones RA, Warren JM, Okely AD, Collins CE, Morgan PJ, Cliff DP, Burrows T, Cleary J, Baur LA. Process evaluation of the Hunter Illawarra Kids Challenge Using Parent Support study: a multisite randomized controlled trial for the management of child obesity. Health Promotion Practice 2010 November;11(6):917-27. Secondary analysis
Ref ID: 2612

(2490) Jones RA, Riethmuller A, Hesketh K, Trezise J, Batterham M, Okely AD. Promoting fundamental movement skill development and physical activity in early childhood settings: a cluster randomized controlled trial. Pediatric Exercise Science 2011 November;23(4):600-15. Not All Participants were Overweight and/or Obese
Ref ID: 2613

(2491) Jones TE, Basilio JL, Brophy PM, McCammon MR, Hickner RC. Long-term exercise training in overweight adolescents improves plasma peptide YY and resistin. Obesity 2009 June;17(6):1189-95. Off topic
Ref ID: 722

(2492) Jonsson F, Johanson G. Physiologically based modeling of the inhalation kinetics of styrene in humans using a bayesian population approach. Toxicology and Applied Pharmacology 2002 February 15;179(1):35-49. Off topic
Ref ID: 1845

(2493) Jordão ISC, Kac G. Determinantes da retenção de peso pós-parto segundo a cor da pele em mulheres do Rio de Janeiro, Brasil. Revista Panamericana de Salud Pública 2005 December;18(6):403-11. Cohort Study
Ref ID: 4233

(2494) Jorgensen JO, Pedersen SA, Thuesen L, Jorgensen J, Ingemann-Hansen T, Skakkebaek NE, Christiansen JS. Beneficial effects of growth hormone treatment in GH-deficient adults. Lancet 1989 June 3;1(8649):1221-5. Drug intervention study
Ref ID: 2323

(2495) Jouret B, Ahluwalia N, Dupuy M, Cristini C, Negre-Pages L, Grandjean H, Tauber M. Prevention of overweight in preschool children: results of kindergarten-based interventions. International Journal of Obesity (London) 2009 October;33(10):1075-83. All groups not randomized
Ref ID: 95

(2496) Jovanovic L. Achieving euglycaemia in women with gestational diabetes Mellitus - Current options for screening, diagnosis and treatment. Drugs 2004;64(13):1401-17. Off topic
Ref ID: 5375

(2497) Jover M, Mellier D. Influence of knowledge in postural anticipation in children during development. Annee Psychologique 2005;105(4):553-72. Off topic
Ref ID: 5376

(2498) Judge LW, Bellar D, Judge M. Efficacy of potentiation of performance through overweight implement throws on male and female high-school weight throwers. Journal of Strength and Conditioning Research 2010 July;24(7):1804-9. Not an

exercise intervention study
Ref ID: 39

(2499) Juhasz I, Gyore I, Csende Z, Racz L, Tihanyi J. Creatine supplementation improves the anaerobic performance of elite junior fin swimmers. Acta Physiologica Hungarica 2009 September;96(3):325-36. Diet Intervention or Supplement Study
Ref ID: 688

(2500) Jun HS, Kim IK, Lee HJ, Lee HJ, Kang JH, Kim JR, Shin HD, Song J. Effects of UCP2 and UCP3 Variants on the Manifestation of Overweight in Korean Children. Obesity 2009;17(2):355-62. Off topic
Ref ID: 5377

(2501) Jung K, Maaser R, Ingenillem M, Post C, Woltermann N, Landwehr L, Schmidt J. [A comprehensive sports program geared to children--illustrated on untrained obese 9-13 year old children]. [German]. Medizinische Welt 1978 February 24;29(8):301-9. No non-intervention control group
Ref ID: 2384

(2502) Jung SH, Kim J, Davis JM, Blair SN, Cho HC. Association among basal serum BDNF, cardiorespiratory fitness and cardiovascular disease risk factors in untrained healthy Korean men. European Journal of Applied Physiology 2011 February;111(2):303-11. Study limited to adults
Ref ID: 2614

(2503) Jurado Ortiz LE, Uribe Escalante MT, Montoya Arboleda AC, Otálvaro Alvarez CM, Quintana Guerra AM. Factores de riesgo cardiovascular en docentes universitarios. Med UPB 2006 October;25(2):185-98. Not a randomized controlled trial (RCT)
Ref ID: 4234

(2504) Jurg ME, Kremers SP, Candel MJ, van der Wal MF, de Meij JS. A controlled trial of a school-based environmental intervention to improve physical activity in Dutch children: JUMP-in, kids in motion. Health Promotion International 2006 December;21(4):320-30. Not a randomized controlled trial (RCT)
Ref ID: 1329

(2505) Juul A, Vahl N, Jorgensen JO, Christiansen JS, Sneppen SB, Feldt-Rasmussen U, Skakkebaek NE. Consequences of stopping growth hormone (GH) therapy in young GH deficient patients with childhood onset disease. Growth Hormone and Igf Research 1998 February;8:Suppl-9. Drug intervention study
Ref ID: 2110

(2506) Juvonen R, Bloigu A, Peitso A, Silvennoinen-Kassinen S, Saikku P, Leinonen M, Hassi J, Harju T. Training improves physical fitness and decreases CRP also in asthmatic conscripts. Journal of Asthma 2008 April;45(3):237-42. Not All

Participants were Overweight and/or Obese
Ref ID: 970

(2507) Jürimäe T, Sudi K, Payerl D, Leppik A, Jürimäe J, Müller R, Tafeit E. Relationships between bioelectric impedance and subcutaneous adipose tissue thickness measured by LIPOMETER and skinfold calipers in children. European Journal of Applied Physiology 2003;90:178-84. Off topic
Ref ID: 4852

(2508) Kaaresen PI, Rønning JA, Ulvund SE, Dahl LB. A randomized, controlled trial of the effectiveness of an early-intervention program in reducing parenting stress after preterm birth. Pediatrics 2006;118:e9-19. Off topic
Ref ID: 4853

(2509) KaÅˆovskÃ½ P, BareÅ¡ M, Severa S, Richardson A, Kraus J, Severa S, Turconi AC, Bonikowski M, Jasinski M, Jozwiak M, Lukaszewska A, Tymecka-Maciag I, Wendorff J, Benetin J, LisÃ½ L, Cardo E, Macaya A, Pascual SIP, Poo P, Fontan D, Morton R. Long-term efficacy and tolerability of 4-monthly versus yearly botulinum toxin type A treatment for lower-limb spasticity in children with cerebral palsy. Developmental Medicine and Child Neurology 2009;51(6):436-45. Drug intervention study
Ref ID: 3245

(2510) Kabir I, Rahman MM, Haider R, Mazumder RN, Khaled MA, Mahalanabis D. Increased height gain of children fed a high-protein diet during convalescence from shigellosis: a six-month follow-Up study. Journal of Nutrition 1998;128:1688-91. Diet Intervention Study
Ref ID: 4854

(2511) Kaczor JJ, Ziolkowski W, Popinigis J, Tarnopolsky MA. Anaerobic and aerobic enzyme activities in human skeletal muscle from children and adults. Pediatric Research 2005;57(3):331-5. Not an exercise intervention study
Ref ID: 5378

(2512) Kagawa M, Tahara Y, Moji K, Nakao R, Aoyagi K, Hills AP. Secular changes in growth among Japanese children over 100 years (1900-2000). Asia Pacific Journal of Clinical Nutrition 2011;20(2):180-9. Off topic
Ref ID: 5379

(2513) Kai ML, Yeong YL, Chee KC, Rasool AH. An Open-Label Pilot Study to Assess the Efficacy and Safety of Virgin Coconut Oil in Reducing Visceral Adiposity. ISRN Pharmacology 2011 January;1-7. Diet Intervention or Supplement Study
Ref ID: 3643

(2514) Kaimal S, Thappa DM. Diet in dermatology: Revisited. Indian Journal of Dermatology, Venereology and Leprology 2010 March;76(2):103-15. Diet Intervention Study
Ref ID: 3644

(2515) Kain B, Burrows Argote R, Caballero B, Henry F, Berríos Carrasola X, Reeder B, Manrique Espinoza M, Moreno M. Taller 4: programas de prevención y tratamiento de la obesidad. In: Albala Brevis C, Kain B, Burrows Argote R, Díaz Bustos E, editors. Obesidad: un desafío pendiente.Santiago de Chile: Universitaria; 2000. p. 289-308.Not an exercise intervention study
Ref ID: 4235

(2516) Kain B, Olivares C, Romo M, Leyton D, Vio D, Cerda R, González P, Giadalah A, Albala B. Estado nutricional y resistencia aeróbica en escolares de educación básica: línea base de un Proyecto de Promoción de la Salud. Revista médica de Chile 2004 November;132(11):1395-402. Prevalence study
Ref ID: 4236

(2517) Kain B, Vio dR, Leyton D, Cerda R, Olivares C, Uauy D, Albala B. Estrategia de promoción de la salud en escolares de educación básica municipalizada de la comuna de Casablanca, Chile. Revista Chilena de Nutrición 2005 August;32(2):126-32. Diet & Exercise intervention
Ref ID: 4237

(2518) Kain B, Uauy D, Leyton D, Cerda R, Olivares C, Vio D. Efectividad de una intervención en educación alimentaria y actividad física para prevenir obesidad en escolares de la ciudad de Casablanca, Chile (2003-2004). Revista médica de Chile 2008 January;136(1):22-30. Diet & Exercise intervention
Ref ID: 537

(2519) Kain J, Uauy R, Albala, Vio F, Cerda R, Leyton B. School-based obesity prevention in Chilean primary school children: methodology and evaluation of a controlled study. International Journal of Obesity and Related Metabolic Disorders 2004 April;28(4):483-93. Diet & Exercise intervention
Ref ID: 1656

(2520) Kain J, Leyton B, Cerda R, Vio F, Uauy R. Two-year controlled effectiveness trial of a school-based intervention to prevent obesity in Chilean children. Public Health Nutrition 2009 September;12(9):1451-61. Not a randomized controlled trial (RCT)
Ref ID: 701

(2521) Kain J, Vio F, Albala C. Obesity trends and determinant factors in Latin America. Cadernos de Saúde Pública 2003;19(supl.1):S77-S86. Review article
Ref ID: 729

(2522) Kain J, Concha F, Salazar G, Leyton B, Rodríguez MdP, Ceballos X, Vio F. Prevención de obesidad en preescolares y escolares de escuelas municipales de una comuna de Santiago de Chile: proyecto piloto 2006. Obesity prevention in preschool and schoolchildren attending public schools from a district of Santiago, Chile: pilot project 2006. Archivos Latinoamericanos de Nutrición

2009 June;59(2):139-46. Multiple interventions
Ref ID: 4238

(2523) Kain J, Leyton B, Concha F, Salazar G, Lobos L, Vio F. Estrategia de prevención de obesidad en escolares: Efecto de un programa aplicado a sus profesores (2007-2008). Effect of counselling school teachers on healthy lifestyle on the impact of a program to reduce childhood obesity. Revista médica de Chile 2010 February;138(2):181-7. Multiple interventions
Ref ID: 4239

(2524) Kakkis ED, Muenzer J, Tiller GE, Waber L, Belmont J, Passage M, Izykowski B, Phillips J, Doroshow R, Walot I, Hoft R, Neufeld EF. Enzyme-replacement therapy in mucopolysaccharidosis I. New England Journal of Medicine 2001 January 18;344(3):182-8. Off topic
Ref ID: 1936

(2525) Kalantar-Zadeh K, Mehrotra R, Fouque D, Kopple JD. Metabolic Acidosis and Malnutrition-Inflammation Complex Syndrome in Chronic Renal Failure. Seminars in Dialysis 2004 November;17(6):455-65. Off topic
Ref ID: 3645

(2526) Kalavainen MP, Korppi MO, Nuutinen OM. Clinical efficacy of group-based treatment for childhood obesity compared with routinely given individual counseling. International Journal of Obesity (London) 2007 October;31(10):1500-8. No exercise only group
Ref ID: 241

(2527) Kalavi FN, Muroki NM, Omwega AM, Mwadime RK. Effect of tempe-yellow maize porridge and milk-yellow maize porridge on growth rate, diarrhoea and duration of rehabilitation of malnourished children. East African medical journal 1996;73:427-31. Off topic
Ref ID: 4856

(2528) Kalivianakis M, Minich DM, Bijleveld CMA, van Aalderen WMC, Stellaard F, Laseur M, Vonk RJ, Verkade HJ. Fat malabsorption in cystic fibrosis patients receiving enzyme replacement therapy is due to impaired intestinal uptake of long-chain fatty acids. American Journal of Clinical Nutrition 1999;69(1):127-34. Off topic
Ref ID: 5380

(2529) Kalogeropoulos S, Petrogiannopoulos C, Gagos S, Kampas N, Kalogeropoulos G. The influence of 5-year therapy with tibolone on the lipid profile in postmenopausal women with mild hypercholesterolemia. Gynecological Endocrinology 2004 April;18(4):227-32. Study limited to adults
Ref ID: 3646

(2530) Kamath CC, Vickers KS, Ehrlich A, McGovern L, Johnson J, Singhal V, Paulo R, Hettinger A, Erwin PJ, Montori VM. Clinical review: behavioral interventions

to prevent childhood obesity: a systematic review and metaanalyses of randomized trials. [Review] [45 refs]. Journal of Clinical Endocrinology and Metabolism 2008 December;93(12):4606-15. Review article
Ref ID: 846

(2531) Kamnik R, Shi JQ, Murray-Smith R, Bajd T. Nonlinear modeling of FES-supported standing-up in paraplegia for selection of feedback sensors. IEEE Transactions on Neural Systems and Rehabilitation Engineering 2005 March;13(1):40-52. Off topic
Ref ID: 1528

(2532) Kanaley JA, Dall R, Moller N, Nielsen SC, Christiansen JS, Jensen MD, Jorgensen JO. Acute exposure to GH during exercise stimulates the turnover of free fatty acids in GH-deficient men. Journal of Applied Physiology 2004 February;96(2):747-53. Drug intervention study
Ref ID: 1672

(2533) Kanazawa A, Miyasita C, Okada E, Kobayashi S, Washino N, Sasaki S, Yoshioka E, Mizutani F, Chisaki Y, Saijo Y, Kishi R. Blood persistent organochlorine pesticides in pregnant women in relation to physical and environmental variables in The Hokkaido study on environment and children's health. Science of the Total Environment 2012;426:73-82. Off topic
Ref ID: 5381

(2534) Kang HS, Gutin B, Barbeau P, Owens S, Lemmon CR, Allison J, Litaker MS, Le NA. Physical training improves insulin resistance syndrome markers in obese adolescents. Medicine and Science in Sports and Exercise 2002 December;34(12):1920-7. No exercise only group
Ref ID: 371

(2535) Kann L, Kinchen SA, Williams BI, Ross JG, Lowry R, Grunbaum JA, Kolbe LJ. Youth risk behavior surveillance - United States, 1999. Journal of School Health 2000;70(7):271-85. Survey or questionnaire
Ref ID: 5382

(2536) Kann L, TellJohann, Wooley SF. Health Education: Results From the School Health Policies and Programs Study 2006. Journal of School Health 2007 October;77(8):408-34. Survey or questionnaire
Ref ID: 3825

(2537) Kanner AM. To Comply with AED Therapy . What Patients Are Not Told! Epilepsy Currents 2009 September;9(5):139-40. Retrospective study
Ref ID: 3647

(2538) Kannisto M, Alaranta H, Merikanto J, Kroger H, Karkkainen J. Bone mineral status after pediatric spinal cord injury. Spinal Cord 1998;36(9):641-6. Off topic
Ref ID: 5383

(2539) Kanovsky P, Bares M, Severa S, Richardson A, Dysport Paediatric Limb Spasticity Study Group. Long-term efficacy and tolerability of 4-monthly versus yearly botulinum toxin type A treatment for lower-limb spasticity in children with cerebral palsy. Developmental Medicine and Child Neurology 2009 June;51(6):436-45. Drug intervention study
Ref ID: 732

(2540) Kapellen TM, Wolf J, Rosenbauer J, Stachow R, Ziegler R, Szczepanski R, Holl RW. Changes in the Use of Analogue Insulins in 37206 Children and Adolescents with Type 1 Diabetes in 275 German and Austrian Centers during the Last Twelve Years. Experimental and Clinical Endocrinology and Diabetes 2009;117(7):329-35. Drug intervention study
Ref ID: 5384

(2541) Karabulutlu O, Reis N. The investigation of life styles adopted by women living in Erzurum as regards cervical cancer risk. Healthmed 2011;5(6):1625-31. Off topic
Ref ID: 5385

(2542) Karam JG, McFarlane SI. Prevention of type 2 DM: Implications for adolescents and young adults. Pediatric Endocrinology Reviews 2008;5(SUPPL. 4):980-8. Review article
Ref ID: 3246

(2543) Karanja N, Lutz T, Ritenbaugh C, Maupome G, Jones J, Becker T, Aickin M. The TOTS community intervention to prevent overweight in American Indian toddlers beginning at birth: a feasibility and efficacy study. Journal of Community Health 2010;35:667-75. Subjects less than 2 years old
Ref ID: 4858

(2544) Karanja N, Aickin M, Lutz T, Mist S, Jobe JB, Maupome G, Ritenbaugh C. A community-based intervention to prevent obesity beginning at birth among American Indian children: study design and rationale for the PTOTS study. Journal of Primary Prevention 2012 August;33(4):161-74. Inappropriate Study Design
Ref ID: 5910

(2545) Karasek M, Stawerska R, Smyczynska J, Lewinski A. Increased melatonin concentrations in children with growth hormone deficiency. Journal of Pineal Research 2007;42(2):119-24. Off topic
Ref ID: 5386

(2546) Kardinaal AF, Hoorneman G, Vaananen K, Charles P, Ando S, Maggiolini M, Charzewska J, Rotily M, Deloraine A, Heikkinen J, Juvin R, Schaafsma G. Determinants of bone mass and bone geometry in adolescent and young adult women. Calcified Tissue International 2000 February;66(2):81-9. Off topic
Ref ID: 1992

(2547) Kargarfard M, Kelishadi R, Ziaee V, Ardalan G, Halabchi F, Mazaheri R, Poursafa P, Hayatbakhsh MR. The impact of an after-school physical activity program on health-related fitness of mother/daughter pairs: CASPIAN study. Preventive Medicine 2012 March;54(3-4):219-23. Not a randomized controlled trial (RCT)
Ref ID: 2615

(2548) Karila C, Saulnier JP, Elie C, Taupin P, Scheinmann P, Le BM, Waernessycle S, de BJ. [Exercise alveolar hypoventilation in long-term survivors of bronchopulmonary dysplasia]. Revue des Maladies Respiratoires 2008;25:303-12. Off topic
Ref ID: 4859

(2549) Karl JP, Young AJ, Rood JC, Montain SJ. Independent and combined effects of eating rate and energy density on energy intake, appetite, and gut hormones. Obesity (Silver Spring) 2013 March;21(3):E244-E252. Inappropriate Intervention
Ref ID: 5911

(2550) Karpoff L, Vinet A, Schuster I, Oudot C, Goret L, Dauzat M, Obert P, Perez-Martin A. Abnormal vascular reactivity at rest and exercise in obese boys. European Journal of Clinical Investigation 2009 February;39(2):94-102. Not a randomized controlled trial (RCT)
Ref ID: 801

(2551) Karthaus M, Rosenthal C, Ganser A. Prophylaxis and treatment of chemo- and radiotherapy-induced oral mucositis â€“ are there new strategies? Bone Marrow Transplantation 1999 November 15;24(10):1095. Off topic
Ref ID: 3648

(2552) Karvetti RL, Hakala P. A seven-year follow-up of a weight reduction programme in Finnish primary health care. European Journal of Clinical Nutrition 1992 October;46(10):743-52. Study limited to adults
Ref ID: 436

(2553) Kashiwazaki H, Dejima Y, Oriasrivera J, Coward WA. Energy-Expenditure Determined by the Doubly Labeled Water Method in Bolivian Aymara Living in A High-Altitude Agropastoral Community. American Journal of Clinical Nutrition 1995;62(5):901-10. Not an exercise intervention study
Ref ID: 5387

(2554) Katch V, Becque MD, Marks C, Moorehead C, Rocchini A. Basal metabolism of obese adolescents: inconsistent diet and exercise effects. American Journal of Clinical Nutrition 1988 September;48(3):565-9. No exercise only group
Ref ID: 451

(2555) Kathirvel S, Shende D, Madan R. Comparison of anti-emetic effects of ondansetron, metoclopromide or a combination of both in children undergoing

surgery for strabismus. European journal of anaesthesiology 1999;16:761-5. Drug intervention study
Ref ID: 4860

(2556) Kato A, Kodani Y, Shimada H, Sasaki T, Hayakawa F, Kohyama K. Effects of Fish Collagen Peptides on Physical Properties of Mixed Gels Containing Konjac Glucomannan and Kappa-Carrageenan. Journal of the Japanese Society for Food Science and Technology-Nippon Shokuhin Kagaku Kogaku Kaishi 2011;58(6):252-8. Off topic
Ref ID: 5388

(2557) Kato K, Silva MJ, Needham LL, Calafat AM. Determination of total phthalates in urine by isotope-dilution liquid chromatography-tandem mass spectrometry. Journal of Chromatography B-Analytical Technologies in the Biomedical and Life Sciences 2005;814(2):355-60. Off topic
Ref ID: 5389

(2558) Kattelmann KK, Conti K, Ren C. The medicine wheel nutrition intervention: a diabetes education study with the Cheyenne River Sioux Tribe. Journal of the American Dietetic Association 2009 September;109(9):1532-9. Study limited to adults, Not an exercise intervention study
Ref ID: 98

(2559) Katz DL. School-based interventions for health promotion and weight control: not just waiting on the world to change. Annual Review of Public Health 2009 April 29;30:253-72. Review article
Ref ID: 690

(2560) Katz DL, Cushman D, Reynolds J, Njike V, Treu JA, Walker J, Smith E, Katz C. Putting physical activity where it fits in the school day: preliminary results of the ABC (Activity Bursts in the Classroom) for fitness program. Prev Chronic Dis 2010 July;7(4`):A82. No exercise only group, Not All Participants were Overweight and/or Obese
Ref ID: 37

(2561) Katz DL, Katz CS, Treu JA, Reynolds J, Njike V, Walker J, Smith E, Michael J. Teaching healthful food choices to elementary school students and their parents: the Nutrition DetectivesTM program. Journal of School Health 2011 January;81(1):21-8. Diet Intervention Study
Ref ID: 2617

(2562) Katz DL. Unfattening our children: forks over feet. International Journal of Obesity 2011;35(1):33-7. Review article
Ref ID: 5390

(2563) Katz ES, Moore RH, Rosen CL, Mitchell RB, Amin R, Arens R, Muzumdar H, Chervin RD, Marcus CL, Paruthi S, Willging P, Redline S. Growth after adenotonsillectomy for obstructive sleep apnea: an RCT. Pediatrics 2014

August;134(2):282-9. Inappropriate Intervention
Ref ID: 5912

(2564) Katzmarzyk PT, Leon AS, Rankinen T, Gagnon J, Skinner JS, Wilmore JH, Rao DC, Bouchard C. Changes in blood lipids consequent to aerobic exercise training related to changes in body fatness and aerobic fitness. Metabolism: Clinical and Experimental 2001 July;50(7):841-8. Study limited to adults
Ref ID: 1909

(2565) Kaufman C, Kelly AS, Kaiser DR, Steinberger J, Dengel DR. Aerobic-exercise training improves ventilatory efficiency in overweight children. Pediatric Exercise Science 2007 February;19(1):82-92. Same subjects as another study already included
Ref ID: 238

(2566) Kaufman FR. Obesity and type 2 diabetes in children and youth. Curr Opin Endocrinol Diabetes 2006;13(4):332-7. Review article
Ref ID: 3247

(2567) Kawachi I, Troisi RJ, Rotnitzky AG, Coakley EH, Colditz GA. Can Physical Activity Minimize Weight Gain in Women after Smoking Cessation? American Journal of Public Health 1996 July;86(7):999. Study limited to adults
Ref ID: 3826

(2568) Kawai M, Rosen CJ. Insulin-like growth factor-I and bone: lessons from mice and men. Pediatric Nephrology 2009 July;24(7):1277-85. Animal study
Ref ID: 3649

(2569) Kawata Y, Togashi K, Masuda H, Soya H, Katsuki A, Oshida Y, Sumida Y, Iguchi M. Impact of a diet and exercise program for obese children on serum ghrelin levels. Japanese Journal of Physical Fitness and Sports Medicine 2007;56(4):419-27. Diet & Exercise intervention
Ref ID: 5391

(2570) Kayser B, Marconi C, Amatya T, Basnyat B, Colombini A, Broers B, Cerretelli P. The metabolic and ventilatory response to exercise in Tibetans born at low altitude. Respiration Physiology 1994 September;98(1):15-26. Off topic
Ref ID: 2225

(2571) Kayser B, Hoppeler H, Desplanches D, Marconi C, Broers B, Cerretelli P. Muscle ultrastructure and biochemistry of lowland Tibetans. Journal of Applied Physiology 1996 July;81(1):419-25. Off topic
Ref ID: 2164

(2572) Kazdin AE. Current (lack of) status of theory in child and adolescent psychotherapy research. Journal of Clinical Child Psychology 1999 December;28(4):533. Off topic
Ref ID: 525

(2573) KÃ¼bler J, Hecker-Barth G. Meta-Analysis of Placebo-Controlled Trials of Aprotinin Assessing the Relative Risk of Reoperations in Patients Undergoing Coronary Artery Bypass Graft Surgery. Clinical Drug Investigation 2000 March;19(3):167-71. Review article
Ref ID: 521

(2574) Kean S. Effects on oxygen saturation levels of handling premature infants within the concepts of kinaesthetic infant handling: pilot study. Intensive and Critical Care Nursing 15(4):214-25, 1999 Aug 1999;(4):214-25. Off topic
Ref ID: 3015

(2575) Kecskemethy HH, Herman D, May R, Paul K, Bachrach SJ, Henderson RC. Quantifying weight bearing while in passive standers and a comparison of standers. Developmental Medicine and Child Neurology 2008 July;50(7):520-3. Off topic
Ref ID: 923

(2576) Keegan TH, Milne RL, Andrulis IL, Chang ET, Sangaramoorthy M, Phillips KA, Giles GG, Goodwin PJ, Apicella C, Hopper JL, Whittemore AS, John EM. Past recreational physical activity, body size, and all-cause mortality following breast cancer diagnosis: results from the Breast Cancer Family Registry. Breast Cancer Research and Treatment 2010 September;123(2):531-42. Off topic
Ref ID: 477

(2577) Keehbauch J, Miguel GS, Drapiza L, Pepe J, Bogue R, Smith-Dixon A. Increased documentation and management of pediatric obesity following implementation of an EMR upgrade and education. Clinical Pediatrics 2012 January;51(1):31-8. Off topic
Ref ID: 2618

(2578) Keel PK, Haedt A. Evidence-Based Psychosocial Treatments for Eating Problems and Eating Disorders. Journal of Clinical Child and Adolescent Psychology 2008 January;37(1):39-61. Review article
Ref ID: 3827

(2579) Keele-Smith R, Leon T. Evaluation of individually tailored interventions on exercise adherence. Western Journal of Nursing Research 2003 October;25(6):623-40. Study limited to adults
Ref ID: 353

(2580) Keenan JM. Treatment of patients with lipid disorders in the primary care setting: New treatment guidelines and their implications. Southern Medical Journal 2003 March;96(3):266. Review article
Ref ID: 3650

(2581) Keenan K, Hipwell A, Chung T, Stepp S, Stouthamer-Loeber M, Loeber R, McTigue K. The Pittsburgh girls study: Overview and initial findings. Journal of Clinical Child and Adolescent Psychology 2010 July;39(4):506-21. Longitudinal

Study
Ref ID: 3828

(2582) Keenan MA, Lee GA, Tuckman AS, Esquenazi A. Improving calf muscle strength in patients with spastic equinovarus deformity by transfer of the long toe flexors to the Os calcis. Journal of Head Trauma Rehabilitation 14(2):163-75, 1999 Apr 1999;(2):163-75. Off topic
Ref ID: 3018

(2583) Keitner G. Professionals must reemphasize families' importance in treatment. (cover story). Brown University Child and Adolescent Behavior Letter 2010 August;26(8):1-7. Editorial or letter or comment
Ref ID: 3829

(2584) Kelishadi R, Ardalan G, Gheiratmand R, Majdzadeh R, Delavari A, Heshmat R, Gouya MM, Razaghi EM, Motaghian M, Mokhtari MR, Barekati H, Arabi MS, CASPIAN Study Group. Blood pressure and its influencing factors in a national representative sample of Iranian children and adolescents: the CASPIAN Study. European Journal of Cardiovascular Prevention and Rehabilitation 2006 December;13(6):956-63. Prevalence study
Ref ID: 1320

(2585) Kelishadi R, Razaghi EM, Gouya MM, Ardalan G, Gheiratmand R, Delavari A, Motaghian M, Ziaee V, Siadat ZD, Majdzadeh R, Heshmat R, Barekati H, Arabi MS, Heidarzadeh A, Shariatinejad K, CASPIAN Study Group. Association of physical activity and the metabolic syndrome in children and adolescents: CASPIAN Study. Hormone Research 2007;67(1):46-52. Survey or questionnaire
Ref ID: 1278

(2586) Kelishadi R, Hashemipour M, Mohammadifard N, Alikhassy H, Adeli K. Short- and long-term relationships of serum ghrelin with changes in body composition and the metabolic syndrome in prepubescent obese children following two different weight loss programmes. Clin Endocrinol (Oxf) 2008 November;69(5):721-9. No comparative control group
Ref ID: 193

(2587) Kelishadi R, Hashemi M, Mohammadifard N, Asgary S, Khavarian N. Association of changes in oxidative and proinflammatory states with changes in vascular function after a lifestyle modification trial among obese children. Clinical Chemistry 2008 January;54(1):147-53. Cross-sectional study
Ref ID: 1015

(2588) Kelishadi R, Hashemipour M, Sarrafzadegan N, Mohammadifard N, Alikhasy H, Beizaei M, Sajjadi F, Poursafa P, Amin Z, Ghatreh-Samani S, Khavarian N, Siadat ZD. Effects of a lifestyle modification trial among phenotypically obese metabolically normal and phenotypically obese metabolically abnormal

adolescents in comparison with phenotypically normal metabolically obese adolescents. Maternal and Child Nutrition 2010 July 1;6(3):275-86. Lifestyle Intervention
Ref ID: 2619

(2589) Kelishadi R, Malekahmadi M, Hashemipour M, Soghrati M, Soghrati M, Mirmoghtadaee P, Ghatrehsamani S, Poursafa P, Khavarian N. Can a Trial of Motivational Lifestyle Counseling be Effective for Controlling Childhood Obesity and the Associated Cardiometabolic Risk Factors? Pediatrics and Neonatology 2012;53(2):90-7. Lifestyle Intervention
Ref ID: 5392

(2590) Kelishadi R, Motlagh ME, Roomizadeh P, Abtahi SH, Qorbani M, Taslimi M, Heshmat R, Aminaee T, Ardalan G, Poursafa P, Karimi M. First report on path analysis for cardiometabolic components in a nationally representative sample of pediatric population in the Middle East and North Africa (MENA): the CASPIAN-III Study. Annals of Nutrition and Metabolism 2013;62(3):257-65. Inappropriate Study Design
Ref ID: 5913

(2591) Kelishadi R, Sadri G, Tavasoli AA, Kahbazi M, Roohafza HR, Sadeghi M, Khosravi A, Sabet B, Amani A, Ansari R, Alikhassy H. Cumulative prevalence of risk factors for atherosclerotic cardiovascular diseases in Iranian adolescents: IHHP-HHPC
A prevalência cumulativa de fatores de risco para doença cardiovascular em adolescentes iranianos: IHHP-HHPC. Jornal de Pediatria 2005 December;81(6):447-53. Cross-sectional study
Ref ID: 672

(2592) Kell RT, Bhambhani Y. Cardiorespiratory and hemodynamic responses during repetitive incremental lifting and lowering in healthy males and females. European Journal of Applied Physiology 2003 September;90(1-2):1-9. Study limited to adults
Ref ID: 1716

(2593) Kellens I, Cannizzaro F, Gouilly P, Crielaard JM. [Inspiratory muscles strength training in recreational athletes]. [French]. Revue des Maladies Respiratoires 2011 May;28(5):602-8. Off topic
Ref ID: 2620

(2594) Keller A, Klossek A, Gausche R, Hoepffner W, Kiess W, Keller E. [Prevention for obesity in childhood]. Deutsche Medizinische Wochenschrift 2009;134:13-8. Diet & Exercise intervention
Ref ID: 4862

(2595) Keller A, Klossek A, Gausche R, Hoepffner W, Kiess W, Keller E. [Selective primary obesity prevention in children]. Deutsche medizinische Wochenschrift

2009 January;134(1-2):13-8. No exercise only group
Ref ID: 143

(2596) Keller C, Trevino RP. Effects of two frequencies of walking on cardiovascular risk factor reduction in Mexican American women. Res Nurs Health 2001 October;24(5):390-401. Study limited to adults
Ref ID: 389

(2597) Keller C, Records K, Ainsworth B, Belyea M, Permana P, Coonrod D, Vega-Lopez S, Nagle-Williams A. Madres para la Salud: design of a theory-based intervention for postpartum Latinas. Contemporary Clinical Trials 2011 May;32(3):418-27. Study limited to adults
Ref ID: 2621

(2598) Keller C, Todd M, Ainsworth B, Records K, Vega-Lopez S, Permana P, Coonrod D, Nagle WA. Overweight, obesity, and neighborhood characteristics among postpartum Latinas. Journal of Obesity 2013;2013:916468. Inappropriate Population
Ref ID: 5914

(2599) Kelley GA, Kelley KS. Aerobic exercise and resting blood pressure in women: a meta-analytic review of controlled clinical trials. Journal of Womens Health and Gender-Based Medicine 1999 July;8(6):787-803. Review article
Ref ID: 2002

(2600) Kelley GA, Kelley KS. Effects of aerobic exercise on non-high-density lipoprotein cholesterol in children and adolescents: a meta-analysis of randomized controlled trials. [Review] [34 refs][Erratum appears in Prog Cardiovasc Nurs. 2009 Mar;24(1):44]. Progress in Cardiovascular Nursing 2008;23(3):128-32. Review article
Ref ID: 850

(2601) Kelley GA, Kelley KS. Effects of exercise in the treatment of overweight and obese children and adolescents: a systematic review of meta-analyses. Journal of Obesity 2013;2013:783103. Inappropriate Study Design
Ref ID: 5915

(2602) Kelly AS, Steinberger J, Olson TP, Dengel DR. In the absence of weight loss, exercise training does not improve adipokines or oxidative stress in overweight children. Metabolism 2007 July;56(7):1005-9. Same subjects as another study already included
Ref ID: 235

(2603) Kelly CM, Burnett AF, Newton MJ. The effect of strength training on three-kilometer performance in recreational women endurance runners. Journal of Strength and Conditioning Research 2008 March;22(2):396-403. Study limited to adults
Ref ID: 938

(2604) Kelly EB, Parra-Medina D, Pfeiffer KA, Dowda M, Conway TL, Webber LS, Jobe JB, Going S, Pate RR. Correlates of physical activity in black, Hispanic, and white middle school girls. Journal of Physical Activity and Health 2010 March;7(2):184-93. Survey or questionnaire
Ref ID: 530

(2605) Kelly SA, Melnyk BM. Systematic review of multicomponent interventions with overweight middle adolescents: implications for clinical practice and research. [Review] [56 refs]. Worldviews on Evidence-Based Nursing 2008;5(3):113-35. Review article
Ref ID: 838

(2606) Kemp C, Pienaar AE. Relationship between the body composition and motor and physical competence of Grade 1 learners in South Africa. Journal of Sports Medicine and Physical Fitness 2013 December;53(6):635-43. Inappropriate Study Design
Ref ID: 5916

(2607) Kemper KJ, Cassileth B, Ferris T. Holistic Pediatrics: A Research Agenda. Pediatrics 1999 April 2;103(4):902. Off topic
Ref ID: 3651

(2608) Kempf K, Dirk M, Kolb H, Hebestreit A, Bittner G, Martin S. [The Da Vinci Medical-mental motivation program for supporting lifestyle changes in patients with type 2 diabetes]. [German]. Deutsche Medizinische Wochenschrift 2012 February;137(8):362-7. Not an exercise intervention study
Ref ID: 2622

(2609) Kempton MJ, Ettinger U, Foster R, Williams SC, Calvert GA, Hampshire A, Zelaya FO, O'Gorman RL, McMorris T, Owen AM, Smith MS. Dehydration affects brain structure and function in healthy adolescents. Human Brain Mapping 2011 January;32(1):71-9. Off topic
Ref ID: 2623

(2610) Kennedy LA, Milton B, Bundred P. Lay food and health worker involvement in community nutrition and dietetics in England: roles, responsibilities and relationship with professionals. Journal of Human Nutrition and Dietetics 2008 June;21(3):210-24. Off topic
Ref ID: 3652

(2611) Kent JD, Blader JC. Effects of late-afternoon methylphenidate administration on behavior and sleep in. Pediatrics 1995 August;96(2):320-5. Drug intervention study
Ref ID: 3653

(2612) Keochkerian D, Chlif M, Delanaud S, Gauthier R, Maingourd Y, Ahmaidi S. Timing and driving components of the breathing strategy in children with cystic

fibrosis during exercise. Pediatric Pulmonology 2005;40(5):449-56. Off topic
Ref ID: 5393

(2613) Kerksick CM, Rasmussen C, Lancaster S, Starks M, Smith P, Melton C, Greenwood M, Almada A, Kreider R. Impact of differing protein sources and a creatine containing nutritional formula after 12 weeks of resistance training. Nutrition 2007 September;23(9):647-56. Diet Intervention or Supplement Study
Ref ID: 1189

(2614) Kerksick CM, Wilborn CD, Campbell WI, Harvey TM, Marcello BM, Roberts MD, Parker AG, Byars AG, Greenwood LD, Almada AL, Kreider RB, Greenwood M. The effects of creatine monohydrate supplementation with and without D-pinitol on resistance training adaptations. Journal of Strength and Conditioning Research 2009 December;23(9):2673-82. Diet Intervention or Supplement Study
Ref ID: 634

(2615) Kerr J, Patrick K, Norman G, Stein MB, Calfas K, Zabinski M, Robinson A. Randomized control trial of a behavioral intervention for overweight women: impact on depressive symptoms. Depression and Anxiety 2008;25(7):555-8. Study limited to adults
Ref ID: 237

(2616) Kerr J, Norman GJ, Adams MA, Ryan S, Frank L, Sallis JF, Calfas KJ, Patrick K. Do neighborhood environments moderate the effect of physical activity lifestyle interventions in adults? Health and Place 2010 September;16(5):903-8. Primary outcome(s) not assessed
Ref ID: 42

(2617) Kerr MP, Baker GA, Brodie MJ. A randomized, double-blind, placebo-controlled trial of topiramate in adults with epilepsy and intellectual disability: impact on seizures, severity, and quality of life. Epilepsy and Behavior 2005;7:472-80. Study limited to adults
Ref ID: 4863

(2618) Kesebir S, Aksoy AE. Ureme Hormonlari ve Duygudurum Bozukluklari (Turkish). Current Approaches in Psychiatry / Psikiyatride Guncel Yaklasimlar 2010 September;2(3):281-307. Off topic
Ref ID: 3654

(2619) Kesten JM, Griffiths PL, Cameron N. A systematic review to determine the effectiveness of interventions designed to prevent overweight and obesity in pre-adolescent girls. [Review]. Obesity Reviews 2011 December;12(12):997-1021. Review article
Ref ID: 2624

(2620) Kesztyus D, Schreiber A, Wirt T, Wiedom M, Dreyhaupt J, Brandstetter S, Koch B, Wartha O, Muche R, Wabitsch M, Kilian R, Steinacker JM. Economic

evaluation of URMEL-ICE, a school-based overweight prevention programme comprising metabolism, exercise and lifestyle intervention in children. European Journal of Health Economics 2013 April;14(2):185-95. Inappropriate Intervention
Ref ID: 5917

(2621) Ketola E, Sipila R, Makela M. Effectiveness of individual lifestyle interventions in reducing cardiovascular disease and risk factors. Annals of Medicine 2000 May;32(4):239-51. Review article
Ref ID: 1975

(2622) Ketola E, Makela M, Klockars M. Individualised multifactorial lifestyle intervention trial for high-risk cardiovascular patients in primary care. British Journal of General Practice 2001 April;51(465):291-4. Lifestyle Intervention
Ref ID: 1905

(2623) Kettaneh A, Heude B, Romon M, Oppert JM, Borys JM, Balkau B, Ducimetiere P, Charles MA. High plasma leptin predicts an increase in subcutaneous adiposity in children and adults. European Journal of Clinical Nutrition 2007;61(6):719-26. Cohort Study
Ref ID: 5394

(2624) Khadilkar VV, Pandit DS, Khadilkar AV, Chiplonkar SA, Kinare AS. Diet and exercise intervention, with special reference to micronutrients, reduces cardiometabolic risk in overweight children. Indian Journal of Endocrinology and Metabolism 2012 January;16(1):124-33. Diet & Exercise intervention
Ref ID: 3655

(2625) Khalaf A, Ekblom O, Kowalski J, Berggren V, Westergren A, Al-Hazzaa H. Female university students' physical activity levels and associated factors--a cross-sectional study in southwestern Saudi Arabia. International Journal of Environmental Research and Public Health 2013 August;10(8):3502-17. Inappropriate Population
Ref ID: 5918

(2626) Khambalia AZ, Dickinson S, Hardy LL, Gill T, Baur LA. A synthesis of existing systematic reviews and meta-analyses of school-based behavioural interventions for controlling and preventing obesity. [Review]. Obesity Reviews 2012 March;13(3):214-33. Review article
Ref ID: 2625

(2627) Khan I, Yasmin R. Ivermectin in the treatment of scabies. Journal of Pakistan Association of Dermatologists 2007;17:78-83. Off topic
Ref ID: 4864

(2628) Khan NA, Raine LB, Drollette ES, Scudder MR, Pontifex MB, Castelli DM, Donovan SM, Evans EM, Hillman CH. Impact of the FITKids physical activity intervention on adiposity in prepubertal children. Pediatrics 2014

April;133(4):e875-e883. Inappropriate Population
Ref ID: 5919

(2629) Khandelwal M. GDM: Postpartum management to reduce long-term risks. Current Diabetes Reports 2008;8(4):287-93. Review article
Ref ID: 3248

(2630) Kherkheulidze M, Kavlashvili N, Kandelaki E, Manjavidze T. Evaluation of nutritional knowledge of second grade school children and assessment of their dietary intake. Georgian Medical News 2012 November;(212):58-64. Inappropriate Intervention
Ref ID: 5920

(2631) Khor GL, Sharif ZM. Dual forms of malnutrition in the same households in Malaysia--a case study among Malay rural households. Asia Pacific Journal of Clinical Nutrition 2003;12(4):427-37. Off topic
Ref ID: 1687

(2632) Kieler H, Haglund B, Waldenström U, Axelsson O. Routine ultrasound screening in pregnancy and the children's subsequent growth, vision and hearing. British Journal of Obstetrics and Gynaecology 1997;104:1267-72. Off topic
Ref ID: 4865

(2633) Kielgast U, Krarup T, Holst JJ, Madsbad S. Four weeks of treatment with liraglutide reduces insulin dose without loss of glycemic control in type 1 diabetic patients with and without residual beta-cell function. Diabetes Care 2011 July;34(7):1463-8. Drug intervention study
Ref ID: 1025

(2634) Kiessling SG, Chishti A. Management of pediatric hypertension. Therapy 2009;6(1):51-63. Review article
Ref ID: 3249

(2635) Kilaru A, Griffiths PL, Ganapathy S, Ghosh S. Community-based nutrition education for improving infant growth in rural Karnataka. Indian Pediatrics 2005;42:425-32. Diet Intervention Study
Ref ID: 4866

(2636) Killen JD, Telch MJ, Robinson TN, Maccoby N, Taylor CB, Farquhar JW. Cardiovascular disease risk reduction for tenth graders. A multiple-factor school-based approach. Journal Of The American Medical Association 1988 September 23;260(12):1728-33. Not All Participants were Overweight and/or Obese
Ref ID: 2627

(2637) Killen JD, Robinson TN, Telch MJ, Saylor KE, Maron DJ, Rich T, Bryson S. The Stanford Adolescent Heart Health Program. Health Education Quarterly

1989;16(2):263-83. Not All Participants were Overweight and/or Obese
Ref ID: 2317

(2638) Killen JD, Taylor CB, Hammer LD, Litt I, Wilson DM, Rich T, Hayward C, Simmonds B, Kraemer H, Varady A. An attempt to modify unhealthful eating attitudes and weight regulation practices of young adolescent girls. International Journal of Eating Disorders 1993 May;13(4):369-84. Multiple interventions
Ref ID: 2263

(2639) Killough G, Battram D, Kurtz J, Mandich G, Francis L, He M. Pause-2-Play: a pilot schoolbased obesity prevention program. Pause-2-Play: um programa piloto escolar de prevenção de obesidade. Revista Brasileira de Saúde Materno Infantil 2010 September;10(3):303-11. Behavior Modification Intervention
Ref ID: 4240

(2640) Kim CW, Kim BT, Park KH, Kim KM, Lee DJ, Yang SW, Joo NS. Effects of short-term chromium supplementation on insulin sensitivity and body composition in overweight children: randomized, double-blind, placebo-controlled study. Journal of Nutritional Biochemistry 2011 November;22(11):1030-4. Diet Intervention or Supplement Study
Ref ID: 2628

(2641) Kim HB, Stebbins CL, Chai JH, Song JK. Taekwondo training and fitness in female adolescents. Journal of Sports Sciences 2011 January;29(2):133-8. Not All Participants were Overweight and/or Obese
Ref ID: 2629

(2642) Kim HD, Park JS. [The effect of an exercise program on body composition and physical fitness in obese female college students]. Taehan Kanho Hakhoe Chi 2006 February;36(1):5-14. Study not limited to children and adolescents
Ref ID: 283

(2643) Kim JR, Kiefe CI, Liu K, Williams OD, Jacobs DR, Jr., Oberman A. Heart rate and subsequent blood pressure in young adults: the CARDIA study. Hypertension 1999 February;33(2):640-6. Longitudinal Study
Ref ID: 2048

(2644) Kim S, Koniak-Griffin D, Flaskerud JH, Guarnero PA. The impact of lay health advisors on cardiovascular health promotion: using a community-based participatory approach. Journal of Cardiovascular Nursing 2004 May;19(3):192-9. Survey or questionnaire, Lifestyle Intervention
Ref ID: 1619

(2645) Kim TI, Shin YH, White-Traut RC. Multisensory intervention improves physical growth and illness rates in Korean orphaned newborn infants. Research in Nursing and Health 2003 December;26(6):424-33. Subjects less than 2 years

old
Ref ID: 1685

(2646) Kim YH, Yang YO. [Effects of walking exercise on metabolic syndrome risk factors and body composition in obese middle school girls]. Taehan Kanho Hakhoe Chi 2005 August;35(5):858-67. Not a randomized controlled trial (RCT)
Ref ID: 297

(2647) Kimm SY, Barton BA, Obarzanek E, McMahon RP, Sabry ZI, Waclawiw MA, Schreiber GB, Morrison JA, Similo S, Daniels SR. Racial divergence in adiposity during adolescence: The NHLBI Growth and Health Study. Pediatrics 2001 March;107(3):E34. Prospective Study
Ref ID: 1927

(2648) Kimm SY, Glynn NW, Kriska AM, Barton BA, Kronsberg SS, Daniels SR, Crawford PB, Sabry ZI, Liu K. Decline in physical activity in black girls and white girls during adolescence. New England Journal of Medicine 2002 September 5;347(10):709-15. Prospective Study
Ref ID: 1802

(2649) Kimm SY, Glynn NW, Obarzanek E, Kriska AM, Daniels SR, Barton BA, Liu K. Relation between the changes in physical activity and body-mass index during adolescence: a multicentre longitudinal study. Lancet 2005 July 23;366(9482):301-7. Prospective Study
Ref ID: 1489

(2650) Kimmel PL. Psychosocial factors in dialysis patients. Kidney International 2001 April;59(4):1599-613. Off topic
Ref ID: 3656

(2651) King JA, Wasse LK, Broom DR, Stensel DJ. Influence of brisk walking on appetite, energy intake, and plasma acylated ghrelin. Medicine and Science in Sports and Exercise 2010 March;42(3):485-92. Acute study
Ref ID: 576

(2652) King L, Hill AJ. Magazine adverts for healthy and less healthy foods: effects on recall but not hunger or food choice by pre-adolescent children. Appetite 2008 July;51(1):194-7. Diet Intervention Study
Ref ID: 964

(2653) King WC, Belle SH, Eid GM, Dakin GF, Inabnet WB, Mitchell JE, Patterson EJ, Courcoulas AP, Flum DR, Chapman WH, Wolfe BM, Longitudinal Assessment of Bariatric Surgery Study. Physical activity levels of patients undergoing bariatric surgery in the Longitudinal Assessment of Bariatric Surgery study. Surgery for Obesity and Related Diseases 2008 November;4(6):721-8. Off topic
Ref ID: 853

(2654) Kinnunen TI, Pasanen M, Aittasalo M, Fogelholm M, Weiderpass E, Luoto R. Reducing postpartum weight retention--a pilot trial in primary health care. Nutrition Journal 2007;6:21. Off topic
Ref ID: 1144

(2655) Kinnunen TI, Aittasalo M, Koponen P, Ojala K, Mansikkamaki K, Weiderpass E, Fogelholm M, Luoto R. Feasibility of a controlled trial aiming to prevent excessive pregnancy-related weight gain in primary health care. BMC Pregnancy and Childbirth 2008;8:37. Off topic
Ref ID: 895

(2656) Kinnunen TI, Raitanen J, Aittasalo M, Luoto R. Preventing excessive gestational weight gain--a secondary analysis of a cluster-randomised controlled trial. European Journal of Clinical Nutrition 2012 December;66(12):1344-50. Inappropriate Study Design
Ref ID: 5921

(2657) Kinsey AW, Eddy WR, Madzima TA, Panton LB, Arciero PJ, Kim JS, Ormsbee MJ. Influence of night-time protein and carbohydrate intake on appetite and cardiometabolic risk in sedentary overweight and obese women. British Journal of Nutrition 2014 August;112(3):320-7. Inappropriate Population
Ref ID: 5922

(2658) Kipping RR, Payne C, Lawlor DA. Randomised controlled trial adapting US school obesity prevention to England. Archives of Disease in Childhood 2008 June;93(6):469-73. No exercise only group
Ref ID: 201

(2659) Kipping RR, Howe LD, Jago R, Campbell R, Wells S, Chittleborough CR, Mytton J, Noble SM, Peters TJ, Lawlor DA. Effect of intervention aimed at increasing physical activity, reducing sedentary behaviour, and increasing fruit and vegetable consumption in children: active for Life Year 5 (AFLY5) school based cluster randomised controlled trial. British Medical Journal 2014;348:g3256. Inappropriate Intervention
Ref ID: 5923

(2660) Kirby A, Woodward A, Jackson S. Benefits of omega-3 supplementation for schoolchildren: review of the current evidence. British Educational Research Journal 2010 October;36(5):699-732. Review article
Ref ID: 3830

(2661) Kirchengast S, Marosi A. Gender Differences in Body Composition, Physical Activity, Eating Behavior and Body Image among Normal Weight Adolescents - An Evolutionary. Collegium Antropologicum 2008;32(4):1079-86. Cross-sectional study
Ref ID: 5395

(2662) Kirk EP, Washburn RA, Bailey BW, LeCheminant JD, Donnelly JE. Six months of supervised high-intensity low-volume resistance training improves strength independent of changes in muscle mass in young overweight men. Journal of Strength and Conditioning Research 2007 February;21(1):151-6. Study limited to adults
Ref ID: 250

(2663) Kirk S, Scott BJ, Daniels SR. Pediatric obesity epidemic: treatment options. [Review] [37 refs]. Journal of the American Dietetic Association 2005 May;105(5:Suppl 1):Suppl-51. Review article
Ref ID: 1520

(2664) Kirk S, Brehm B, Saelens BE, Woo JG, Kissel E, D'Alessio D, Bolling C, Daniels SR. Role of carbohydrate modification in weight management among obese children: a randomized clinical trial. Journal of Pediatrics 2012 August;161(2):320-7. Inappropriate Intervention
Ref ID: 5924

(2665) Kirkwood BR, Manu A, Tawiah AC, ten AG, Gyan T, Weobong B, Lewandowski RE, Soremekun S, Danso S, Pitt C, Hanson K, Owusu AS, Hill Z. Newhints cluster randomised trial to evaluate the impact on neonatal mortality in rural Ghana of routine home visits to provide a package of essential newborn care interventions in the third trimester of pregnancy and the first week of life: trial protocol. Trials 2010;11:58. Off topic
Ref ID: 4867

(2666) Kirvela O, Stern RC, Askanazi J, Doershuk CF, Rothkopf MM, Katz DP. Long-term parenteral nutrition in cystic fibrosis. Nutrition 1993 March;9(2):119-26. Diet Intervention Study
Ref ID: 2266

(2667) Kishnani PS, Nicolino M, Voit T, Rogers RC, Tsai AC, Waterson J, Herman GE, Amalfitano A, Thurberg BL, Richards S, Davison M, Corzo D, Chen YT. Chinese hamster ovary cell-derived recombinant human acid alpha-glucosidase in infantile-onset Pompe disease. Journal of Pediatrics 2006 July;149(1):89-97. Subjects less than 2 years old
Ref ID: 1368

(2668) Kiss MAPD, Vallejo Cuellar L, Machida J, Carvalho PRd, Rodrigues RL. Tempo de resistência em esteira: atletismo. Revista Paulo de Educação Física 1988 May;2(2):49-53. Not a randomized controlled trial (RCT)
Ref ID: 4241

(2669) Kissel JT, Scott CB, Reyna SP, Crawford TO, Simard LR, Krosschell KJ, Acsadi G, Elsheik B, Schroth MK, D'Anjou G, LaSalle B, Prior TW, Sorenson S, Maczulski JA, Bromberg MB, Chan GM, Swoboda KJ, Project Cure Spinal Muscular Atrophy Investigators' Network. Sma carnival trial part ii: A

prospective, single-armed trial of l-carnitine and valproic acid in ambulatory children with spinal muscular atrophy. PLoS ONE [Electronic Resource] 2011;6(7):e21296. Drug intervention study
Ref ID: 2630

(2670) Kissileff HR, Pi-Sunyer FX, Segal K, Meltzer S, Foelsch PA. Acute effects of exercise on food intake in obese and nonobese women. American Journal of Clinical Nutrition 52(2):240-5, 1990 Aug 1990;(2):240-5. Acute study
Ref ID: 3030

(2671) Kitler ME. European Society Of Cardiology Congress 2010: 28 August 1 September 2010; Stockholm, Sweden. Pharmaceutical Medicine 2010;24(5):295-300. Review article
Ref ID: 3250

(2672) Kitzman-Ulrich H, Wilson DK, St George SM, Lawman H, Segal M, Fairchild A. The integration of a family systems approach for understanding youth obesity, physical activity, and dietary programs. [Review]. Clinical Child and Family Psychology Review 2010 September;13(3):231-53. Review article
Ref ID: 476

(2673) Kivimaki M, Ferrie JE, Brunner E, Head J, Shipley MJ, Vahtera J, Marmot MG. Justice at work and reduced risk of coronary heart disease among employees: the Whitehall II Study. Archives of Internal Medicine 2005 October 24;165(19):2245-51. Cohort Study
Ref ID: 1459

(2674) Kjendlie PL, Ingjer F, Madsen O, Stallman RK, Stray-Gundersen J. Differences in the energy cost between children and adults during front crawl swimming. European Journal of Applied Physiology 2004 April;91(4):473-80. Off topic
Ref ID: 1660

(2675) Kjendlie PL, Ingjer F, Stallman RK, Stray-Gundersen J. Factors affecting swimming economy in children and adults. European Journal of Applied Physiology 2004 October;93(1-2):65-74. Off topic
Ref ID: 1571

(2676) Klar J, Asling B, Carlsson B, Ulvsback M, Dellsen A, Strom C, Rhedin M, Forslund A, Anneren G, Ludvigsson JF, Dahl N. RAR-related orphan receptor A isoform 1 (RORa1) is disrupted by a balanced translocation t(4;15)(q22.3;q21.3) associated with severe obesity. European Journal of Human Genetics 2005;13(8):928-34. Case-Control / Case Study
Ref ID: 5396

(2677) Klasson-Heggebo L, Andersen LB, Wennlof AH, Sardinha LB, Harro M, Froberg K, Anderssen SA. Graded associations between cardiorespiratory fitness, fatness, and blood pressure in children and adolescents. British Journal

of Sports Medicine 2006 September 25;40(1):25-9. Cross-sectional study
Ref ID: 1432

(2678) Klein JD, Graff CA, Santelli JS, Hedberg VA, Allan MJ, Elster AB. Developing quality measures for adolescent care: validity of adolescents' self-reported receipt of preventive services. Health Services Research 1999 April;34(1:Pt 2):t-404. Off topic
Ref ID: 2035

(2679) Kleinsteuber S. K, Rocco P, Herrera C, Vainzof M, Birke L, Yáñez Z, Flandes J, Zatz M, Carvallo de Saint Quentin P, Avaria Benapres MdlA. Mialgias post ejercicios como forma de presentación de una distrofinopatía. Revista médica de Chile 2000 July;128(7):772-7. Case-Control / Case Study
Ref ID: 774

(2680) Klentrou P, Hay J, Plyley M. Habitual physical activity levels and health outcomes of Ontario youth. European Journal of Applied Physiology 2003 June;89(5):460-5. Cross-sectional study
Ref ID: 1740

(2681) Klepper SE. Effects of an eight-week physical conditioning program on disease signs and symptoms in children with chronic arthritis. Arthritis Care and Research 1999;12:52-60. Not All Participants were Overweight and/or Obese
Ref ID: 4868

(2682) Klesges LM, Baranowski T, Beech B, Cullen K, Murray DM, Rochon J, Pratt C. Social desirability bias in self-reported dietary, physical activity and weight concerns measures in 8- to 10-year-old African-American girls: results from the Girls Health Enrichment Multisite Studies (GEMS). Preventive Medicine 2004 May;38:Suppl-87. Cross-sectional study
Ref ID: 1646

(2683) Klesges RC, Obarzanek E, Klesges LM, Stockton MB, Beech BM, Murray DM, Lanctot JQ, Sherrill-Mittleman DA. Memphis Girls health Enrichment Multi-site Studies (GEMS): Phase 2: design and baseline. Contemporary Clinical Trials 2008 January;29(1):42-55. No exercise only group
Ref ID: 232

(2684) Klesges RC, Obarzanek E, Kumanyika S, Murray DM, Klesges LM, Relyea GE, Stockton MB, Lanctot JQ, Beech BM, McClanahan BS, Sherrill-Mittleman D, Slawson DL. The Memphis Girls' health Enrichment Multi-site Studies (GEMS): an evaluation of the efficacy of a 2-year obesity prevention program in African American girls. Archives of Pediatric Adolescent Medicine 2010 November;164(11):1007-14. No exercise only group, No comparative control group
Ref ID: 15

(2685) Klijn PH, Oudshoorn A, van der Ent CK, van der Net J, Kimpen JL, Helders PJ. Effects of anaerobic training in children with cystic fibrosis: a randomized controlled study. Chest 2004 April;125(4):1299-305. Not All Participants were Overweight and/or Obese
Ref ID: 1644

(2686) Klimentidis YC, Dulin-Keita A, Casazza K, Willig AL, Allison DB, Fernandez JR. Genetic admixture, social-behavioural factors and body composition are associated with blood pressure differently by racial-ethnic group among children. Journal of Human Hypertension 2012;26(2):98-107. Cross-sectional study
Ref ID: 5397

(2687) Kline CE, Crowley EP, Ewing GB, Burch JB, Blair SN, Durstine JL, Davis JM, Youngstedt SD. The effect of exercise training on obstructive sleep apnea and sleep quality: a randomized controlled trial. Sleep 2011 December;34(12):1631-40. Study limited to adults
Ref ID: 1031

(2688) Kline CE, Ewing GB, Burch JB, Blair SN, Durstine JL, Davis JM, Youngstedt SD. Exercise training improves selected aspects of daytime functioning in adults with obstructive sleep apnea. Journal of Clinical Sleep Medicine 2012;8(4):357-65. Inappropriate Population
Ref ID: 5925

(2689) Klingenberg L, Chaput JP, Holmback U, Jennum P, Astrup A, Sjodin A. Sleep restriction is not associated with a positive energy balance in adolescent boys. American Journal of Clinical Nutrition 2012 August;96(2):240-8. Inappropriate Outcomes
Ref ID: 5926

(2690) Kluft J, Beker L, Castagnino M, Gaiser J, Chaney H, Fink RJ. A comparison of bronchial drainage treatments in cystic fibrosis. Pediatric Pulmonology 1996;22:271-4. Off topic
Ref ID: 4869

(2691) Knapp PE, Storer TW, Herbst KL, Singh AB, Dzekov C, Dzekov J, LaValley M, Zhang A, Ulloor J, Bhasin S. Effects of a supraphysiological dose of testosterone on physical function, muscle performance, mood, and fatigue in men with HIV-associated weight loss. American Journal of Physiology - Endocrinology and Metabolism 2008 June;294(6):E1135-E1143. Study limited to adults
Ref ID: 943

(2692) Knobloch K, Joest B, Vogt PM. Cellulite and extracorporeal Shockwave therapy (CelluShock-2009)--a randomized trial. BMC Women's Health 2010;10:29.

Study limited to adults
Ref ID: 2633

(2693) Knoll N, Volckmar AL, Putter C, Scherag A, Kleber M, Hebebrand J, Hinney A, Reinehr T. The fatty acid amide hydrolase (FAAH) gene variant rs324420 AA/AC is not associated with weight loss in a 1-year lifestyle intervention for obese children and adolescents. Hormone and Metabolic Research 2012 January;44(1):75-7. Lifestyle Intervention
Ref ID: 2634

(2694) Knols RH, de Bruin ED, Uebelhart D, Aufdemkampe G, Schanz U, Stenner-Liewen F, Hitz F, Taverna C, Aaronson NK. Effects of an outpatient physical exercise program on hematopoietic stem-cell transplantation recipients: a randomized clinical trial. Bone Marrow Transplantation 2011 September;46(9):1245-55. Study limited to adults
Ref ID: 2635

(2695) Knowlden AP, Sharma M. Systematic review of family and home-based interventions targeting paediatric overweight and obesity. [Review]. Obesity Reviews 2012 June;13(6):499-508. Review article
Ref ID: 2636

(2696) Knudsen FU, Paerregaard A, Andersen R, Andresen J. Long term outcome of prophylaxis for febrile convulsions. Archives of Disease in Childhood 1996;74:13-8. Off topic
Ref ID: 4870

(2697) Knudsen FU, Paerregaard A, Andersen R, Andresen J. [Long-term prognosis in febrile convulsions with and without prophylaxis]. Ugeskrift for laeger 1997;159:3598-602. Duplicate
Ref ID: 945

(2698) Knuth AG, Malta DC, Dumith SC, Pereira CA, Morais Neto OL, Temporão JG, Penna G, Hallal PC. Prática de atividade física e sedentarismo em brasileiros: resultados da Pesquisa Nacional por Amostra de Domicílios (PNAD) 2008. Practice of physical activity and sedentarism among Brazilians: results of the National Household Sample Survey - 2008. Ciência and Saúde Coletiva 2011 September;16(9):3697-705. Survey or questionnaire
Ref ID: 4242

(2699) Knutsen SF, Knutsen R. The Tromso survey: The family intervention study - The effect of intervention on some coronary risk factors and dietary habits, a 6-year follow-up. Preventive Medicine 1991;20(2):197-212. Follow-up Study
Ref ID: 3251

(2700) Knutson LM, Bushman B, Young JC, Ward G. Age Expansion of the Thirty-Second Walk Test Norms for Children. Pediatric Physical Therapy

2009;21(3):235-43. Off topic
Ref ID: 5398

(2701) Koda YKL, Vidolin E. Familial hyperamylasemia. Revista do Hospital das Clínicas Universidade de São Paulo 2002 April;57(2):77-82. Case-Control / Case Study
Ref ID: 4243

(2702) Koff SR. Dance Education as an Aspect of Movement and Mobility in Everyday Living. Quest 2005 February 1;57(1):148-53. Off topic
Ref ID: 3904

(2703) Koiou E, Tziomalos K, Katsikis I, Kandaraki EA, Kalaitzakis E, Delkos D, Vosnakis C, Panidis D. Weight loss significantly reduces serum lipocalin-2 levels in overweight and obese women with polycystic ovary syndrome. Gynecological Endocrinology 2012 January;28(1):20-4. Off topic
Ref ID: 2637

(2704) Koiou E, Tziomalos K, Katsikis I, Delkos D, Tsourdi EA, Panidis D. Disparate effects of pharmacotherapy on plasma plasminogen activator inhibitor-1 levels in women with the polycystic ovary syndrome. Hormones (Athens) 2013 October;12(4):559-66. Inappropriate Population
Ref ID: 5927

(2705) Koivisto VA, Stevens LK, Mattock M, Ebeling P, Muggeo M, Stephenson J, Idzior-Walus B. Cardiovascular disease and its risk factors in IDDM in Europe. EURODIAB IDDM Complications Study Group. Diabetes Care 1996 July;19(7):689-97. Cross-sectional study
Ref ID: 2170

(2706) Kokalas N, Petridou A, Nikolaidis MG, Mougios V. Effect of aerobic exercise on lipaemia and its fatty acid profile after a meal of moderate fat content in eumenorrhoeic women. British Journal Of Nutrition 2005 November;94(5):698-704. Acute study
Ref ID: 1453

(2707) Kokkvoll A, Grimsgaard S, Odegaard R, Flaegstad T, Njolstad I. Single versus multiple-family intervention in childhood overweight--Finnmark Activity School: a randomised trial. Archives of Disease in Childhood 2014 March;99(3):225-31. Inappropriate Intervention
Ref ID: 5928

(2708) Kokorowski PJ, Routh JC. Metabolic and Malignant Consequences of Intestinal Augmentation of the Urinary Tract in Children. Current Medical Literature: Urology 2011 June;17(2):29-38. Off topic
Ref ID: 3657

(2709) Kol IO, Egilmez H, Kaygusuz K, Gursoy S, Mimaroglu C. Open-label, prospective, randomized comparison of propofol and sevoflurane for laryngeal mask anesthesia for magnetic resonance imaging in pediatric patients. Clinical Therapeutics 2008;30:175-81. Drug intervention study
Ref ID: 4871

(2710) Kolle E, Torstveit MK, Sundgot-Borgen J. Bone mineral density in Norwegian premenopausal women. Osteoporosis International 2005 August;16(8):914-20. Inappropriate Outcomes
Ref ID: 1495

(2711) Kollias A, Antonodimitrakis P, Grammatikos E, Chatziantonakis N, Grammatikos EE, Stergiou GS. Trends in high blood pressure prevalence in Greek adolescents. Journal of Human Hypertension 2009;23(6):385-90. Cross-sectional study
Ref ID: 5399

(2712) Kolotkin RL, Crosby RD, Williams GR. Health-related quality of life varies among obese subgroups. Obesity Research 2002 August;10(8):748-56. Survey or questionnaire
Ref ID: 1803

(2713) Kondo T, Kobayashi I, Murakami M. Effect of exercise on circulating adipokine levels in obese young women. Endocrine Journal 2006 April;53(2):189-95. Study limited to adults
Ref ID: 1056

(2714) Kones R. Rosuvastatin, inflammation, C-reactive protein, JUPITER, and primary prevention of cardiovascular disease--a perspective. [Review]. Drug Design, Development and Therapy 2010;4:383-413. Review article
Ref ID: 2638

(2715) Kong AS, Sussman AL, Yahne C, Skipper BJ, Burge MR, Davis SM. School-based health center intervention improves body mass index in overweight and obese adolescents. Journal of Obesity 2013;2013:575016. Inappropriate Intervention
Ref ID: 5929

(2716) Konig JS, Elmadfa I. Plasma copper concentration as marker of copper intake from food. Annals of Nutrition and Metabolism 2000;44(3):129-34. Diet Intervention or Supplement Study
Ref ID: 5400

(2717) Kontulainen SA, Kannus PA, Pasanen ME, Sievänen HT, Heinonen AO, Oja P, Vuori I. Does previous participation in high-impact training result in residual bone gain in growing girls? One year follow-up of a 9-month jumping intervention. International Journal of Sports Medicine 2002;23:575-81. Follow-

up Study
Ref ID: 4872

(2718) Korinthenberg R. Chronic inflammatory demyelinating polyradiculoneuropathy in children and their response to treatment. Neuropediatrics 1999 August;30(4):190-6. Retrospective study
Ref ID: 2061

(2719) Korner A, Neef M, Friebe D, Erbs S, Kratzsch J, Dittrich K, Bluher S, Kapellen TM, Kovacs P, Stumvoll M, Bluher M, Kiess W. Vaspin is related to gender, puberty and deteriorating insulin sensitivity in children. International Journal of Obesity 2011;35(4):578-86. Diet Intervention or Supplement Study
Ref ID: 5401

(2720) Korsten-Reck U, Wolfarth B, Bonk M, Keul J, Berg A. [The Freiburg Intervention Trial for Obesity in Chidren (FITOC)]. [German]. Zeitschrift fur Arztliche Fortbildung und Qualitatssicherung 2000 September;94(8):677-81. Multiple interventions
Ref ID: 1948

(2721) Korsten-Reck U, Kromeyer-Hauschild K, Korsten K, Baumstark MW, Dickhuth HH, Berg A. Frequency of secondary dyslipidemia in obese children. Vascular Health and Risk Management 2008;4(5):1089-94. Cross-sectional study
Ref ID: 825

(2722) Kosinski A, Grzybiak M, Nowinski J, Dabrowska-Kugacka A, Lewicka E, Raczak G, Kozlowski D. Morphological remarks regarding the structure of conduction system in the right ventricle. Kardiologia Polska 2012;70(5):472-7. Off topic
Ref ID: 5402

(2723) Kothari R, Cualing H, Balachander T. Neural network analysis of flow cytometry immunophenotype data. IEEE Transactions on Bio Medical Engineering 1996;43:803-10. Off topic
Ref ID: 4873

(2724) Kousoulis P, Skrepetis K, Efthimiou I, Ferentinos G, Diamantopoulos I, Papadopoulos G. Does G Force Increase the Incidence of Varicocele in Air Force Pilot Cadets? Urologia Internationalis 2010;84(1):73-7. Off topic
Ref ID: 5403

(2725) Kovacs GT, Oh J, Kovacs J, Tonshoff B, Hunziker EB, Zapf J, Mehls O. Growth promoting effects of growth hormone and IGF-I are additive in experimental uremia. Kidney International 1996;49(5):1413-21. Animal study
Ref ID: 5404

(2726) Kovacs VA, Fajcsak Z, Gabor A, Martos E. School-based exercise program improves fitness, body composition and cardiovascular risk profile in

overweight/obese children. Acta Physiologica Hungarica 2009 September;96(3):337-47. No control group (NC)
Ref ID: 687

(2727) Kovalskys I, Indart RP, Amigo MP, De Gregorio MJ, Rausch HC, Karner M. Food intake and anthropometric evaluation in school-aged children of Buenos Aires. Archivos Argentinos de Pediatría 2013 January;111(1):9-14. Inappropriate Outcomes
Ref ID: 5930

(2728) Kraemer WJ, Ratamess N, Fry AC, Triplett-McBride T, Koziris LP, Bauer JA, Lynch JM, Fleck SJ. Influence of resistance training volume and periodization on physiological and performance adaptations in collegiate women tennis players. American Journal of Sports Medicine 2000 September;28(5):626-33. Study limited to adults
Ref ID: 1956

(2729) Kraemer WJ, Mazzetti SA, Nindl BC, Gotshalk LA, Volek JS, Bush JA, Marx JO, Dohi K, Gomez AL, Miles M, Fleck SJ, Newton RU, Hakkinen K. Effect of resistance training on women's strength/power and occupational performances. Medicine and Science in Sports and Exercise 2001 June;33(6):1011-25. Study limited to adults
Ref ID: 1913

(2730) Kraemer WJ, Joseph MF, Volek JS, Hoffman JR, Ratamess NA, Newton RU, Fragala MS, French DN, Rubin MA, Scheett TP, McGuigan MR, Thomas GA, Gomez AL, Hakkinen K, Maresh CM. Endogenous opioid peptide responses to opioid and anti-inflammatory medications following eccentric exercise-induced muscle damage. Peptides 2010 January;31(1):88-93. Drug intervention study
Ref ID: 593

(2731) Krafft CE, Schwarz NF, Chi L, Weinberger AL, Schaeffer DJ, Pierce JE, Rodrigue AL, Yanasak NE, Miller PH, Tomporowski PD, Davis CL, McDowell JE. An 8-month randomized controlled exercise trial alters brain activation during cognitive tasks in overweight children. Obesity (Silver Spring) 2014 January;22(1):232-42. Inappropriate Outcomes
Ref ID: 5932

(2732) Krafft CE, Pierce JE, Schwarz NF, Chi L, Weinberger AL, Schaeffer DJ, Rodrigue AL, Camchong J, Allison JD, Yanasak NE, Liu T, Davis CL, McDowell JE. An eight month randomized controlled exercise intervention alters resting state synchrony in overweight children. Neuroscience 2014 January 3;256:445-55. Inappropriate Outcomes
Ref ID: 5931

(2733) Kramer KL, Greaves RD. Synchrony Between Growth and Reproductive Patterns in Human Females: Early Investment in Growth Among Pume

Foragers. American Journal of Physical Anthropology 2010;141(2):235-44. Off topic
Ref ID: 5405

(2734) Kramer PA. The costs of human locomotion: Maternal investment in child transport. American Journal of Physical Anthropology 1998;107(1):71-85. Off topic
Ref ID: 5406

(2735) Kratenova J, Zejglicova K, Maly M, Filipova V. Prevalence and Risk Factors of Poor Posture in School Children in the Czech Republic. Journal of School Health 2007 March 1;77(3):131-7. Cross-sectional study
Ref ID: 3905

(2736) Kraus JF, Schaffer KB, Rice T, Maroosis J, Harper J. A field trial of back belts to reduce the incidence of acute low back injuries in New York City home attendants. International Journal of Occupational and Environmental Health 2002 April;8(2):97-104. Off topic
Ref ID: 1824

(2737) Kraybill EN, Bose CL, Corbet AJ, Garcia PJ, Asbill D, Edwards K, Long W. Double-blind evaluation of developmental and health status to age 2 years of infants weighing 700 to 1350 grams treated prophylactically at birth with a single dose of synthetic surfactant or air placebo. The Journal of Pediatrics 1995;126:S33-S42. Subjects less than 2 years old
Ref ID: 995

(2738) Kreiter SR, Schwartz RP, Kirkman HN, Charlton PA, Calikoglu AS, Davenport ML. Nutritional rickets in African American breast-fed infants. Journal of Pediatrics 2000;137(2):153-7. Off topic
Ref ID: 5407

(2739) Kreuser F, Kromeyer-Hauschild K, Gollhofer A, Korsten-Reck U, Rottger K. "Obese equals lazy?" analysis of the association between weight status and physical activity in children. Journal of Obesity 2013;2013:437017. Inappropriate Study Design
Ref ID: 5933

(2740) Kreuter MW, Strecher VJ. Do tailored behavior change messages enhance the effectiveness of health risk appraisal? Results from a randomized trial. Health Education Research 1996 March;11(1):97-105. Study limited to adults
Ref ID: 2172

(2741) Krieger JW, Crowe M, Blank SE. Chronic glutamine supplementation increases nasal but not salivary IgA during 9 days of interval training. Journal of Applied Physiology 2004 August;97(2):585-91. Diet Intervention or Supplement Study
Ref ID: 1610

(2742) Kriemler S, Hebestreit H, Mikami S, Bar-Or T, Ayub BV, Bar-Or O. Impact of a single exercise bout on energy expenditure and spontaneous physical activity of obese boys. Pediatric Research 1999 July;46(1):40-4. Acute study
Ref ID: 2013

(2743) Kriemler S, Hebestreit H, Bar-Or O. Temperature-related overestimation of energy expenditure, based on heart-rate monitoring in obese boys. European Journal of Applied Physiology 2002 July;87(3):245-50. Acute study
Ref ID: 1811

(2744) Kriemler S, Manser-Wenger S, Zahner L, Braun-Fahrlander C, Schindler C, Puder JJ. Reduced cardiorespiratory fitness, low physical activity and an urban environment are independently associated with increased cardiovascular risk in children. Diabetologia 2008 August;51(8):1408-15. Cross-sectional study
Ref ID: 177

(2745) Kriemler S, Zahner L, Puder JJ, Braun-Fahrlander C, Schindler C, Farpour-Lambert NJ, Kranzlin M, Rizzoli R. Weight-bearing bones are more sensitive to physical exercise in boys than in girls during pre- and early puberty: a cross-sectional study. Osteoporosis International 2008 December;19(12):1749-58. Cross-sectional study
Ref ID: 870

(2746) Kriemler S, Zahner L, Schindler C, Meyer U, Hartmann T, Hebestreit H, Brunner-La Rocca HP, van MW, Puder JJ. Effect of school based physical activity programme (KISS) on fitness and adiposity in primary schoolchildren: cluster randomised controlled trial. British Medical Journal 2010 February 23;340:c785. doi: 10.1136/bmj.c785.:c785. Not All Participants were Overweight and/or Obese
Ref ID: 57

(2747) Kriska A, Delahanty L, Edelstein S, Amodei N, Chadwick J, Copeland K, Galvin B, El GL, Haymond M, Kelsey M, Lassiter C, Mayer-Davis E, Milaszewski K, Syme A. Sedentary behavior and physical activity in youth with recent onset of type 2 diabetes. Pediatrics 2013 March;131(3):e850-e856. Inappropriate Study Design
Ref ID: 5934

(2748) Krogh-Madsen R, Pedersen M, Solomon TP, Knudsen SH, Hansen LS, Karstoft K, Lehrskov-Schmidt L, Pedersen KK, Thomsen C, Holst JJ, Pedersen BK. Normal physical activity obliterates the deleterious effects of a high-caloric intake. Journal of Applied Physiology (1985) 2014 February 1;116(3):231-9. Inappropriate Intervention
Ref ID: 5935

(2749) Krogh J, Strohle A, Westrin A, Klausen T, Jorgensen MB, Nordentoft M. N-terminal pro-atrial natriuretic peptide response to acute exercise in depressed

patients and healthy controls. Psychoneuroendocrinology 2011 June;36(5):656-63. Acute study
Ref ID: 2639

(2750) Krueger PM, Rogers RG, Ridao-Cano C, Hummer RA. To Help or To Harm? Food stamp receipt and mortality risk prior to the 1996 Welfare Reform Act. Social Forces 2004 June;82(4):1573-99. Off topic
Ref ID: 3832

(2751) Kruel LFM, Tartaruga LAP, Coertjens M, Oliveira AS, Ribas LR, Tartaruga MP. Influência das variáveis antropométricas na economia de corrida e no comprimento de passada em corredoras de rendimento. Motriz Revista de Educação Física (Improv) 2007 March;13(1):1-6. Off topic
Ref ID: 4244

(2752) Kruger A, Wissing MP, Towers GW, Doak CM. Sex differences independent of other psycho-sociodemographic factors as a predictor of body mass index in black South African adults. Journal of Health, Population and Nutrition 2012 March;30(1):56-65. Cross-sectional study
Ref ID: 2640

(2753) Krummel DA, Semmens E, Boury J, Gordon PM, Larkin KT. Stages of change for weight management in postpartum women. Journal of the American Dietetic Association 2004 July;104(7):1102-8. Study limited to adults
Ref ID: 328

(2754) Kruzich LA, Marquis GS, Wilson CM, Stephensen CB. HIV-infected US youth are at high risk of obesity and poor diet quality: a challenge for improving short- and long-term health outcomes. Journal of the American Dietetic Association 2004 October;104(10):1554-60. Cross-sectional study
Ref ID: 1585

(2755) Kubicky RA, Faerber EN, de Chadarevian JP, Wu SF, Rezvani I, De Luca F. An Adolescent With a Mediastinal Mass, Diagnosed With Graves Disease and Thymic Hyperplasia. Pediatrics 2010;125(2):E433-E437. Case-Control / Case Study
Ref ID: 5408

(2756) Kuhl ES, Clifford LM, Bandstra NF, Filigno SS, Yeomans-Maldonado G, Rausch JR, Stark LJ. Examination of the association between lifestyle behavior changes and weight outcomes in preschoolers receiving treatment for obesity. Health Psychology 2014 January;33(1):95-8. Inappropriate Intervention
Ref ID: 5936

(2757) Kuhn P, Zores C, Astruc D, Dufour A, Casper C. Sensory system development and the physical environment of infants born very preterm. Archives de Pediatrie 2011;18:S92-S102. Review article
Ref ID: 5409

(2758) Kulaputana O, Thanakomsirichot S, Anomasiri W. Ginseng supplementation does not change lactate threshold and physical performances in physically active Thai men. Journal of the Medical Association of Thailand 2007 June;90(6):1172-9. Diet Intervention or Supplement Study
Ref ID: 1201

(2759) Kulkarni S, Ramakrishnan U, Dearden KA, Marsh DR, Ha TT, Tran TD, Pachon H. Greater length-for-age increases the odds of attaining motor milestones in Vietnamese children aged 5-18 months. Asia Pacific Journal of Clinical Nutrition 2012;21(2):241-6. Secondary analysis
Ref ID: 2641

(2760) Kulpa PJ, White BM, Visscher R. Aerobic exercise in pregnancy. American Journal of Obstetrics and Gynecology 1987 June;156(6):1395-403. Off topic
Ref ID: 2339

(2761) Kumahara H, Schutz Y, Ayabe M, Yoshioka M, Yoshitake Y, Shindo M, Ishii K, Tanaka H. The use of uniaxial accelerometry for the assessment of physical-activity-related energy expenditure: a validation study against whole-body indirect calorimetry. British Journal Of Nutrition 2004;91(2):235-43. Off topic
Ref ID: 5410

(2762) Kumanyika SK, Obarzanek E, Robinson TN, Beech BM. Phase 1 of the Girls health Enrichment Multi-site Studies (GEMS): conclusion. Ethnicity and Disease 2003;13(1:Suppl 1):Suppl-91. Behavior Modification Intervention
Ref ID: 1750

(2763) Kumanyika SK, Story M, Beech BM, Sherwood NE, Baranowski JC, Powell TM, Cullen KW, Owens AS. Collaborative planning for formative assessment and cultural appropriateness in the Girls health Enrichment Multi-site Studies (GEMS): a retrospection. Ethnicity and Disease 2003;13(1:Suppl 1):Suppl-29. Description of study from review or magazine or etc. (not the actual study)
Ref ID: 1754

(2764) Kumar R. Approved and Investigational Uses of Modafinil: An Evidence-Based Review. Drugs 2008 July;68(11):1803-39. Review article
Ref ID: 3658

(2765) Kuo HT, Lin HC, Tsai CH, Chouc IC, Yeh TF. A follow-up study of preterm infants given budesonide using surfactant as a vehicle to prevent chronic lung disease in preterm infants. The Journal of Pediatrics 2010;156:537-41. Follow-up Study
Ref ID: 4874

(2766) Kupari M, Koskinen P, Virolainen J, Hekali P, Keto P. Prevalence and predictors of audible physiological third heart sound in a population sample aged 36 to 37 years. Circulation 1994 March;89(3):1189-95. Off topic
Ref ID: 2246

(2767) Kuroda K, Saitoh I, Inada E, Takemoto Y, Iwasaki T, Iwase Y, Yamada C, Shinkai M, Matsumoto Y, Hasegawa H, Yamasaki Y, Hayasaki H. Head motion may help mouth opening in children. Archives of Oral Biology 2011;56(1):102-7. Off topic
Ref ID: 5411

(2768) Kussuki MOM, João SMA, Cunha ACPd. Caracterização postural da coluna de crianças obesas de 7 a 10 anos. Fisioterapia em Movimento 2007 March;20(1):77-81. Not an exercise intervention study
Ref ID: 4245

(2769) Kusters DM, Vissers MN, Wiegman A, Kastelein JJP, Hutten BA. Treatment of dyslipidaemia in childhood. Expert Opinion on Pharmacotherapy 2010;11(5):739-53. Review article
Ref ID: 3252

(2770) Kutsal YG, Atalay A, Arslan S, Basaran A, Canturk F, Cindas A, Eryavuz M, Irdesel J, Karadavut KI, Kirazli Y, Sindel D, Senel K, Guler-Uysal F, Yildirim K. Awareness of osteoporotic patients. Osteoporosis International 2005 February;16(2):128-33. Off topic
Ref ID: 1548

(2771) Kuusela AL, Marenk M, Sandahl G, Sanderud J, Nikolajev K, Persson B, Olsson H. Comparative study using oral solutions of bambuterol once daily or terbutaline three times daily in 2-5-year-old children with asthma. Pediatric Pulmonology 2000;29(3):194-201. Drug intervention study
Ref ID: 3253

(2772) Kuzma M, Payer J. [Growth hormone deficiency, its influence on bone mineral density and risk of osteoporotic fractures]. [Review] [45 refs] [Slovak]. Casopis Lekaru Ceskych 2010;149(5):211-6. Review article
Ref ID: 489

(2773) Kuznetsova T, Staessen JA, Olszanecka A, Ryabikov A, Stolarz K, Malyutina S, Fagard R, Kawecka-Jaszcz K, Nikitin Y, European Project On Genes in Hypertension (EPOGH) Investigators. Maternal and paternal influences on left ventricular mass of offspring. Hypertension 2003 January;41(1):69-74. Off topic
Ref ID: 1780

(2774) Kwapiszewski RM, Lee WA. A pilot program to identify and reverse childhood obesity in a primary care clinic. Clinical Pediatrics 2011 July;50(7):630-5. Not a randomized controlled trial (RCT)
Ref ID: 2642

(2775) Kwiterovich PO. Clinical and laboratory assessment of cardiovascular risk in children: Guidelines for screening, evaluation, and treatment. Journal of Clinical Lipidology 2008;2(4):248-66. Review article
Ref ID: 3254

(2776) Kwon MS, Hwang KS. [Effects of an exercise program on body composition, cardiopulmonary function, and physical fitness for obese children]. [Korean]. Daehan Ganho Haghoeji 2007 June;37(4):568-75. Not a randomized controlled trial (RCT)
Ref ID: 1204

(2777) Kyle TM, Brener ND, Kann L, Ross JG, Roberts AM, Iachani R, Robb WH, McManus T. Methods: School Health Policies and Programs Study 2006. Journal of School Health 2007 October;77(8):398-407. Survey or questionnaire
Ref ID: 3833

(2778) Kynde I, Heitmann BL, Bygbjerg IC, Andersen LB, Helge JW. Hypoadiponectinemia in overweight children contributes to a negative metabolic risk profile 6 years later. Metabolism: Clinical and Experimental 2009;58:1817-24. Cross-sectional study
Ref ID: 4877

(2779) Laaksonen DE, Lakka HM, Lynch J, Lakka TA, Niskanen L, Rauramaa R, Salonen JT, Kauhanen J. Cardiorespiratory fitness and vigorous leisure-time physical activity modify the association of small size at birth with the metabolic syndrome. Diabetes Care 2003 July;26(7):2156-64. Cohort Study
Ref ID: 1735

(2780) Laan DJ, Leidy HJ, Lim E, Campbell WW. Effects and reproducibility of aerobic and resistance exercise on appetite and energy intake in young, physically active adults. Applied Physiology, Nutrition, and Metabolism = Physiologie Appliquee, Nutrition et Metabolisme 2010 December;35(6):842-7. Study limited to adults
Ref ID: 2643

(2781) Labayen I, Moreno LA, Marti A, Gonzalez-Lamuno D, Warnberg J, Ortega FB, Bueno G, Nova E, Ruiz JR, Garagorri JM, Martinez JA, Garcia-Fuentes M, Bueno M, Avena Study Group. Effect of the Ala12 allele in the PPARgamma-2 gene on the relationship between birth weight and body composition in adolescents: the AVENA study. Pediatric Research 2007 November;62(5):615-9. Off topic
Ref ID: 1132

(2782) Labayen I, Moreno LA, Ruiz JR, Gonzalez-Gross M, Warnberg J, Breidenassel C, Ortega FB, Marcos A, Bueno M, Avena Study Group. Small birth weight and later body composition and fat distribution in adolescents: the Avena study. Obesity 2008 July;16(7):1680-6. Cross-sectional study
Ref ID: 926

(2783) Labayen I, Ortega FB, Sjostrom M, Nilsson TK, Olsson LA, Ruiz JR. Association of Common Variants of UCP2 Gene With Low-Grade Inflammation in Swedish Children and Adolescents; The European Youth Heart Study.

Pediatric Research 2009;66(3):350-4. Off topic
Ref ID: 5412

(2784) Labayen I, Ortega FB, Ruiz JR, Loit HM, Harro J, Villa I, Veidebaum T, Sjostrom M. Association of exclusive breastfeeding duration and fibrinogen levels in childhood and adolescence: the European Youth Heart Study. Archives of Pediatrics and Adolescent Medicine 2012 January;166(1):56-61. Off topic
Ref ID: 2644

(2785) Lach E. Reduction of subcutaneous fat and improvement in cellulite appearance by dual-wavelength, low-level laser energy combined with vacuum and massage. Journal of Cosmetic and Laser Therapy 2008 December;10(4):202-9. Off topic
Ref ID: 836

(2786) Lachance C, Chessex P, Fouron JC, Widness JA, Bard H. Myocardial, Erythropoietic, and Metabolic Adaptations to Anemia of Prematurity. Journal of Pediatrics 1994;125(2):278-82. Off topic
Ref ID: 5413

(2787) Lago-Penas C, Casais L, Dellal A, Rey E, Dominguez E. Anthropometric and Physiological Characteristics of Young Soccer Players According to Their Playing Positions: Relevance for Competition Success. Journal of Strength and Conditioning Research 2011;25(12):3358-67. Off topic
Ref ID: 5414

(2788) Lai A, Chen W, Helm K. Effects of visfatin gene polymorphism RS4730153 on exercise-induced weight loss of obese children and adolescents of Han Chinese. International Journal of Biological Sciences 2013;9(1):16-21. Inappropriate Outcomes
Ref ID: 5937

(2789) Lai AR, Tashima KT, Taylor LE. Antiretroviral Medication Considerations for Individuals Coinfected with HIV and Hepatitis C Virus. AIDS Patient Care and STDs 2006 October;20(10):678-92. Off topic
Ref ID: 3659

(2790) Lakshman R, Forouhi N, Luben R, Bingham S, Khaw K, Wareham N, Ong KK. Association between age at menarche and risk of diabetes in adults: results from the EPIC-Norfolk cohort study. Diabetologia 2008 May;51(5):781-6. Cohort Study
Ref ID: 978

(2791) Lalau JD. Lactic Acidosis Induced by Metformin Incidence, Management and Prevention. Drug Safety 2010 September;33(9):727-40. Review article
Ref ID: 3660

(2792) Lamari N, Marino LC, Cordeiro JA, Pellegrini AM. Flexibilidade anterior do tronco no adolecente após o pico da velocidade de crescimento em estatura. Acta Ortopédica Brasileira 2007;15(1):25-9. Cross-sectional study
Ref ID: 636

(2793) Lambertucci RH, Puggina EF, Pithon-Curi TC. Efeitos da atividade física em condições patológicas. Revista Brasileira de Ciência e Movimento 2006;14(1):67-74. Review article
Ref ID: 4246

(2794) Lambiase M. Treating pediatric overweight through reductions in sedentary behavior: a review of the literature. [Review] [37 refs]. Journal of Pediatric Health Care 23(1):29-36, 2009 Jan-Feb 2009;(1):29-36. Review article
Ref ID: 3034

(2795) Lambiase MJ, Barry HM, Roemmich JN. Effect of a simulated active commute to school on cardiovascular stress reactivity. Medicine and Science in Sports and Exercise 2010;42:1609-16. Acute study
Ref ID: 4878

(2796) Lambourne K, Washburn RA, Gibson C, Sullivan DK, Goetz J, Lee R, Smith BK, Mayo MS, Donnelly JE. Weight management by phone conference call: a comparison with a traditional face-to-face clinic. Rationale and design for a randomized equivalence trial. Contemporary Clinical Trials 2012 September;33(5):1044-55. Inappropriate Study Design
Ref ID: 5938

(2797) Lambourne K, Washburn RA, Lee J, Betts JL, Thomas DT, Smith BK, Gibson CA, Sullivan DK, Donnelly JE. A 6-month trial of resistance training with milk supplementation in adolescents: effects on body composition. International Journal of Sport Nutrition and Exercise Metabolism 2013 August;23(4):344-56. Inappropriate Intervention
Ref ID: 5939

(2798) Lamina S, Musa DI. Ergogenic effect of varied doses of coffee-caffeine on maximal aerobic power of young African subjects. African Health Sciences 2009 December;9(4):270-4. Diet Intervention or Supplement Study
Ref ID: 611

(2799) Lammers AE, Hislop AA, Flynn Y, Haworth SG. The 6-minute walk test: normal values for children of 4-11 years of age. Archives of Disease in Childhood 2008 June;93(6):464-8. Prospective Study
Ref ID: 951

(2800) Lamont HS, Cramer JT, Bemben DA, Shehab RL, Anderson MA, Bemben MG. Effects of a 6-week periodized squat training program with or without whole-body vibration on jump height and power output following acute vibration exposure. Journal of Strength and Conditioning Research 2009

November;23(8):2317-25. Study limited to adults
Ref ID: 647

(2801) Lana A, del Valle MO, Lopez S, Faya-Ornia G, Lopez ML. Study protocol of a randomized controlled trial to improve cancer prevention behaviors in adolescents and adults using a web-based intervention supplemented with SMS. BMC Public Health 2013;13:357. Inappropriate Study Design
Ref ID: 5940

(2802) Lancarotte I, Nobre MR, Zanetta R, Polydoro M. Estilo de vida e saúde cardiovascular em adolescentes de escolas do município de São Paulo. Lifestyle and cardiovascular health in school adolescents from São Paulo. Arquivos Brasileiros de Cardiologia 2010 July;95(1):61-9. Cross-sectional study
Ref ID: 4247

(2803) Landaeta-Jiménez M, Pérez BM, Arroyo Barahona E, Salazar Loggiodice M. Crecimiento físico y corpulencia en niños y jóvenes nadadores venezolanos. Archivos Venezolanos de Puericultura y Pediatría 2008 December;71(4):131-9. Cross-sectional study
Ref ID: 4248

(2804) Landaeta Jiménez M, Macias Tomei C. Estado nutricional y crecimiento físico en niños mayores lactantes y preescolares del estado Vargas. Archivos Venezolanos de Puericultura y Pediatría 2003 September;66(3):30-42. Cross-sectional study
Ref ID: 683

(2805) Landsbergis PA, Schnall PL, Deitz DK, Warren K, Pickering TG, Schwartz JE. Job strain and health behaviors: results of a prospective study. American Journal of Health Promotion 1998 March;12(4):237-45. Cross-sectional study
Ref ID: 2095

(2806) Lane NE, Hochberg MC, Pressman A, Scott JC, Nevitt MC. Recreational physical activity and the risk of osteoarthritis of the hip in elderly women. Journal of Rheumatology 1999 April;26(4):849-54. Cross-sectional study
Ref ID: 2029

(2807) Langford R, Lunn P, Panter-Brick C. Hand-Washing, Subclinical Infections, and Growth: A Longitudinal Evaluation of an Intervention in Nepali Slums. American Journal of Human Biology 2011;23(5):621-9. Off topic
Ref ID: 5415

(2808) Lanningham-Foster LM, Jensen TB, McCrady SK, Nysse LJ, Foster RC, Levine JA. Laboratory measurement of posture allocation and physical activity in children. Medicine and Science in Sports and Exercise 2005 October;37(10):1800-5. Off topic
Ref ID: 1457

(2809) Lansigan F, Foss FM. Current and Emerging Treatment Strategies for Cutaneous T-cell Lymphoma. Drugs 2010 February 12;70(3):273-86. Off topic
Ref ID: 3661

(2810) Lantz H, Bratteby LE, Fors H, Sandhagen B, Sjostrom L, Samuelson G. Body composition in a cohort of Swedish adolescents aged 15, 17 and 20.5 years. Acta Paediatrica 2008 December;97(12):1691-7. Cross-sectional study
Ref ID: 851

(2811) Lapillonne A, Razafimahefa H, Rigourd V, Granier M. Nutrition of the preterm infant. Archives de Pediatrie 2011;18(3):313-23. Off topic
Ref ID: 5416

(2812) Larguía AM, Enriquez DS, Pensotti A. Fallecidos inmediato al parto. Impacto sobre los componentes de la mortalidad perinatal. Revista del Hospital Materno Infantil Ramón Sardá 2004;23(2):70-4. Off topic
Ref ID: 4249

(2813) LaRowe TL, Wubben DP, Cronin KA, Vannatter SM, Adams AK. Development of a culturally appropriate, home-based nutrition and physical activity curriculum for Wisconsin American Indian families. Preventing Chronic Disease 2007 October;4(4):A109. Diet & Exercise intervention
Ref ID: 1165

(2814) LaRowe TL, Adams AK, Jobe JB, Cronin KA, Vannatter SM, Prince RJ. Dietary intakes and physical activity among preschool-aged children living in rural American Indian communities before a family-based healthy lifestyle intervention. Journal of the American Dietetic Association 2010 July;110(7):1049-57. Not an exercise intervention study
Ref ID: 31

(2815) Larrier YI, Bakerson MA, Linton JM, Walker LR, Woolford SJ. The Role of School Counselors in the Childhood Obesity Epidemic. Journal of School Counseling 2011 January 1;9(3). Review article
Ref ID: 3906

(2816) Larsson P, Henriksson-Larsen K. Combined metabolic gas analyser and dGPS analysis of performance in cross-country skiing. Journal of Sports Sciences 2005 August;23(8):861-70. Off topic
Ref ID: 1469

(2817) Lask B, Fosson A, Rolfe U, Thomas S. Zinc deficiency and childhood-onset anorexia nervosa. Journal of Clinical Psychiatry 1993 February;54(2):63-6. Diet Intervention or Supplement Study
Ref ID: 2272

(2818) Laskowski R, Antosiewicz J. Increased adaptability of young judo sportsmen after protein supplementation. Journal of Sports Medicine and Physical Fitness

2003 September;43(3):342-6. Diet Intervention or Supplement Study
Ref ID: 1701

(2819) Lass N, Kleber M, Winkel K, Wunsch R, Reinehr T. Effect of lifestyle intervention on features of polycystic ovarian syndrome, metabolic syndrome, and intima-media thickness in obese adolescent girls. Journal of Clinical Endocrinology and Metabolism 2011 November;96(11):3533-40. Lifestyle Intervention
Ref ID: 2645

(2820) Latham MC, Stephenson LS, Kurz KM, Kinoti SN. Metrifonate or praziquantel treatment improves physical fitness and appetite of Kenyan schoolboys with Schistosoma haematobium and hookworm infections. American Journal of Tropical Medicine and Hygiene 1990 August;43(2):170-9. Drug intervention study
Ref ID: 2308

(2821) Latif H, Watson K, Nguyen N, Thompson D, Baranowski J, Jago R, Cullen KW, Baranowski T. Effects of goal setting on dietary and physical activity changes in the Boy Scout badge projects. Health Education and Behavior 2011 October;38(5):521-9. Diet & Exercise intervention
Ref ID: 2646

(2822) Latt E, Jurimae J, Haljaste K, Cicchella A, Purge P, Jurimae T. Physical Development and Swimming Performance During Biological Maturation in Young Female Swimmers. Collegium Antropologicum 2009;33(1):117-22. Off topic
Ref ID: 5417

(2823) Lau FC, Bagchi M, Sen C, Roy S, Bagchi D. Nutrigenomic analysis of diet-gene interactions of functional supplements for weight management. Current Genomics 2008;9(4):239-51. Review article
Ref ID: 3255

(2824) Lauber RP, Sheard NF. The American Heart Association dietary guidelines for 2000: A summary report. Nutrition Reviews 2001;59(9):298-306. Review article
Ref ID: 3256

(2825) Laude M. Assessment of nutritional status, cognitive development, and mother-child interaction in Central American refugee children. Revista Panamericana de Salud Pública 1999 September;6(3):164-71. Cross-sectional study
Ref ID: 4250

(2826) Laumer U, Bauer M, Fichter M, Milz H. [Therapeutic effects of the Feldenkrais method "awareness through movement" in patients with eating disorders]. [German]. Psychotherapie, Psychosomatik, Medizinische Psychologie 1997 May;47(5):170-80. Not a randomized controlled trial (RCT)
Ref ID: 2135

(2827) Laurson KR, Eisenmann JC, Welk GJ, Wickel EE, Gentile DA, Walsh DA. Evaluation of youth pedometer-determined physical activity guidelines using receiver operator characteristic curves. Preventive Medicine 2008 May;46(5):419-24. Study less than 4 weeks
Ref ID: 963

(2828) Laurson KR. Designing and evaluating clinical cutpoints for childhood obesity. United States -- Iowa: Iowa State University; 2008.
Cross-sectional study
Ref ID: 5104

(2829) Lavelle HV, Mackay DF, Pell JP. Systematic review and meta-analysis of school-based interventions to reduce body mass index. Journal of Public Health (Oxford) 2012 August;34(3):360-9. Inappropriate Study Design
Ref ID: 5941

(2830) Lavender G, Bird SR. Effect of sodium bicarbonate ingestion upon repeated sprints. British Journal of Sports Medicine 1989 March;23(1):41-5. Diet Intervention or Supplement Study
Ref ID: 2319

(2831) Lavrador MSF, Abbes PT, Escrivão MAMS, Taddei JAdAC. Riscos cardiovasculares em adolescentes com diferentes graus de obesidade. Cardiovascular risks in adolescents with different degrees of obesity. Riesgos cardiovasculares en adolescentes con diferentes grados de obesidad. Arquivos Brasileiros de Cardiologia 2011 March;96(3):205-11. Cross-sectional study
Ref ID: 4251

(2832) Law M, Russell D, Pollock N, Rosenbaum P, Walter S, King G. A comparison of intensive neurodevelopmental therapy plus casting and a regular occupational therapy program for children with cerebral palsy. Developmental Medicine and Child Neurology 1997;39:664-70. Rehabilitation study
Ref ID: 4879

(2833) Lawani MM, Alihonou E, Akplogan B, Poumarat G, Okou L, Adjadi N. [Effect of antenatal gymnastics on childbirth: a study on 50 sedentary women in the Republic of Benin during the second and third quarters of pregnancy]. Santé 2003;13:235-41. Study limited to adults
Ref ID: 4880

(2834) Lawlor DA, Riddoch CJ, Page AS, Andersen LB, Wedderkopp N, Harro M, Stansbie D, Smith GD. Infant feeding and components of the metabolic syndrome: findings from the European Youth Heart Study. Archives of Disease in Childhood 2005 June;90(6):582-8. Cross-sectional study
Ref ID: 1513

(2835) Lawlor DA, Smith GD, Kelly A, Sattar N, Ebrahim S. Leptin and coronary heart disease risk: prospective case control study of British women. Obesity 2007 July;15(7):1694-701. Case-Control / Case Study, Prospective Study
Ref ID: 1198

(2836) Lawlor DA, Jago R, Noble SM, Chittleborough CR, Campbell R, Mytton J, Howe LD, Peters TJ, Kipping RR. The Active for Life Year 5 (AFLY5) school based cluster randomised controlled trial: study protocol for a randomized controlled trial. Trials [Electronic Resource] 2011;12:181. Diet & Exercise intervention
Ref ID: 2647

(2837) Lawlor DA, Smith GD, Ebrahim S. Socioeconomic Position and Hormone Replacement Therapy Use: Explaining the Discrepancy in Evidence From Observational and Randomized Controlled Trials. American Journal of Public Health 2004 December;94(12):2149-54. Cross-sectional study
Ref ID: 3834

(2838) Lawlor DA, Batty GD, Morton SMB, Clark H, Macintyre S, Leon DA. Childhood Socioeconomic Position, Educational Attainment, and Adult Cardiovascular Risk Factors: The Aberdeen Children of the 1950s Cohort Study. American Journal of Public Health 2005 July;95(7):1245-51. Cohort Study
Ref ID: 3835

(2839) Lawman HG, Wilson DK, Van Horn ML, Zarrett N. The role of motivation in understanding social contextual influences on physical activity in underserved adolescents in the ACT Trial: a cross-sectional study. Childhood Obesity 2012 December;8(6):542-50. Inappropriate Study Design
Ref ID: 5942

(2840) Lawrence-Wright MB, Boyne MS, Osmond C, Fraser RA, Soares-Wynter S, Thame M, Reid M, Taylor-Bryan C, Forrester TE. The effect of feto-maternal size and childhood growth on left ventricular mass and arterial stiffness in Afro-Caribbean children. Journal of Human Hypertension 2011;25(7):457-64. Cohort Study
Ref ID: 5418

(2841) Laws R. A new evidence-based model for weight management in primary care: the Counterweight Programme. Journal of Human Nutrition and Dietetics 2004 June;17(3):191-208. Study not limited to children and adolescents
Ref ID: 333

(2842) Lawson JA, Rennie DC, Dosman JA, Cammer AL, Senthilselvan A. Obesity, diet, and activity in relation to asthma and wheeze among rural dwelling children and adolescents. Journal of Obesity 2013;2013:315096. Inappropriate Study Design
Ref ID: 5943

(2843) Lazarou C, Panagiotakos DB, Matalas AL. Lifestyle factors are determinants of children's blood pressure levels: the CYKIDS study. Journal of Human Hypertension 2009;23(7):456-63. Cross-sectional study
Ref ID: 5419

(2844) Lazzer S, Boirie Y, Montaurier C, Vernet J, Meyr M, Vermorel M. A weight reduction program preserves fat-free mass but not metabolic rate in obese adolescents. Obesity Research 2004;12(2):233-40. Diet & Exercise intervention
Ref ID: 5420

(2845) Lazzer S, Vermorel M, Montaurier C, Meyer M, Boirie Y. Changes in adipocyte hormones and lipid oxidation associated with weight loss and regain in severely obese adolescents. International Journal of Obesity 2005;29(10):1184-91. Lifestyle Intervention
Ref ID: 5421

(2846) Lazzer S, Lafortuna C, Busti C, Galli R, Agosti F, Sartorio A. Effects of low- and high-intensity exercise training on body composition and substrate metabolism in obese adolescents. Journal of Endocrinological Investigation 2011 January;34(1):45-52. Study less than 4 weeks
Ref ID: 2648

(2847) Le KA, Ventura EE, Fisher JQ, Davis JN, Weigensberg MJ, Punyanitya M, Hu HH, Nayak KS, Goran MI. Ethnic differences in pancreatic fat accumulation and its relationship with other fat depots and inflammatory markers. Diabetes Care 2011 February;34(2):485-90. Off topic
Ref ID: 1065

(2848) le R, I, le RK, Comulada WS, Greco EM, Desmond KA, Mbewu N, Rotheram-Borus MJ. Home visits by neighborhood Mentor Mothers provide timely recovery from childhood malnutrition in South Africa: results from a randomized controlled trial. Nutrition Journal 2010;9:56. Off topic
Ref ID: 4881

(2849) Leach RA, Yates JM. Nutrition and youth soccer for childhood overweight: a pilot novel chiropractic health education intervention. Journal of Manipulative and Physiological Therapeutics 2008 July;31(6):434-41. Diet & Exercise intervention
Ref ID: 897

(2850) Leader B, Baca QJ, Golan DE. Protein therapeutics: a summary and pharmacological classification. Nature Reviews Drug Discovery 2008 January;7(1):21-39. Review article
Ref ID: 3662

(2851) Leal Junior EC, Lopes-Martins RA, Baroni BM, De MT, Taufer D, Manfro DS, Rech M, Danna V, Grosselli D, Generosi RA, Marcos RL, Ramos L, Bjordal JM. Effect of 830 nm low-level laser therapy applied before high-intensity exercises

on skeletal muscle recovery in athletes. Lasers in Medical Science 2009 November;24(6):857-63. Off topic
Ref ID: 661

(2852) Leal Júnior EC, Pinto FM, Cortez PJO, Fagundes AdA. Comparaçäo da funçäo pulmonar entre jovens atletas e sedentários. Fisioterapia em Movimento 2003 March;16(1):47-50. Not an exercise intervention study
Ref ID: 4252

(2853) Leal DT, Fialho FA, Dias IMÁV, Nascimento Ld, Arruda WC. Diabetes na infância e adolescência: O enfrentamento da doença no cotidiano da família. Diabetes in childhood and adolescence: The facing of the illness in the everyday life of the family. HU Revista 2009 December;35(4). Off topic
Ref ID: 4253

(2854) Leal ML, Lamas L, Aoki MS, Ugrinowitsch C, Ramos MS, Tricoli V, Moriscot AS. Effect of different resistance-training regimens on the WNT-signaling pathway. European Journal of Applied Physiology 2011 October;111(10):2535-45. Study limited to adults
Ref ID: 2650

(2855) Leandro C, Guerra S, Duarte JA, Mota J. Maturaçäo, composiçäo corporal e aptidäo cardiorrespiratória em crianças e adolescentes na área do grande Porto, Portugal. Revista Brasileira de Saúde Materno Infantil 2001 December;1(3):249-56. Cross-sectional study
Ref ID: 762

(2856) Leasure JL, Giddabasappa A, Chaney S, Johnson JE, Pothakos K, Lau YS, Fox DA. Low-level human equivalent gestational lead exposure produces sex-specific motor and coordination abnormalities and late-onset obesity in year-old mice. Environmental Health Perspectives 2008;116(3):355-61. Animal study
Ref ID: 5422

(2857) Leatherdale ST, Wong SL. Modifiable characteristics associated with sedentary behaviours among youth. International Journal of Pediatric Obesity 2008;3(2):93-101. Cross-sectional study
Ref ID: 958

(2858) Leatherdale ST, Pouliou T, Church D, Hobin E. The association between overweight and opportunity structures in the built environment: a multi-level analysis among elementary school youth in the PLAY-ON study. International Journal of Public Health 2011 June;56(3):237-46. Cross-sectional study
Ref ID: 2651

(2859) Leão AS, Lima SO, Albuquerque Junior RLCd. Avaliação do crescimento físico em escolares de 7 a 10 anos da rede pública de ensino do município de Aracaju-SE. Evaluation of the physical growth in students from 7 to 10 years of public education network of the city of Aracaju-SE. Revista Brasileira de

Ciências da Saúde 2010;14(1). Cross-sectional study
Ref ID: 4254

(2860) Leão LSC, Araújo LM, Moraes LTLP, Assis AM. Prevalência de obesidade em escolares de Salvador, Bahia. Arquivos Brasileiros de Endocrinologia and Metabologia 2003 April;47(2):151-7. Cross-sectional study
Ref ID: 4255

(2861) Lebenthal Y, Gat-Yablonski G, Shtaif B, Padoa A, Phillip M, Lazar L. Effect of sex hormone administration on circulating ghrelin levels in peripubertal children. Journal of Clinical Endocrinology and Metabolism 2006 January;91(1):328-31. Drug intervention study
Ref ID: 1429

(2862) Lebrun CM, Petit MA, McKenzie DC, Taunton JE, Prior JC. Decreased maximal aerobic capacity with use of a triphasic oral contraceptive in highly active women: a randomised controlled trial. British Journal of Sports Medicine 2003 August;37(4):315-20. Drug intervention study
Ref ID: 1729

(2863) LeCheminant JD, Jacobsen DJ, Bailey BW, Mayo MS, Hill JO, Smith BK, Donnelly JE. Effects of long-term aerobic exercise on EPOC. International Journal of Sports Medicine 2008 January;29(1):53-8. Study limited to adults
Ref ID: 1016

(2864) LeCheminant JD, Heden T, Smith J, Covington NK. Comparison of energy expenditure, economy, and pedometer counts between normal weight and overweight or obese women during a walking and jogging activity. European Journal of Applied Physiology 2009 July;106(5):675-82. Study limited to adults
Ref ID: 122

(2865) Leclair E, Borel B, Baguet G, Berthoin S, Mucci P, Thevenet D, Reguem SC. Reproducibility of measurement of muscle deoxygenation in children during exercise. Pediatric Exercise Science 2010;22(2):183-94. Off topic
Ref ID: 5423

(2866) Leclerc KM. The role of exercise in reducing coronary heart disease and associated risk factors. [Review] [60 refs]. Journal - Oklahoma State Medical Association 1992 June;85(6):283-90. Review article
Ref ID: 2278

(2867) Lee CD, Jacobs DR, Jr., Schreiner PJ, Iribarren C, Hankinson A. Abdominal obesity and coronary artery calcification in young adults: the Coronary Artery Risk Development in Young Adults (CARDIA) Study. American Journal of Clinical Nutrition 2007 July;86(1):48-54. Cross-sectional study
Ref ID: 1203

(2868) Lee DH, Jacobs DR, Jr., Gross M, Kiefe CI, Roseman J, Lewis CE, Steffes M. Gamma-glutamyltransferase is a predictor of incident diabetes and hypertension: the Coronary Artery Risk Development in Young Adults (CARDIA) Study. Clinical Chemistry 2003 August;49(8):1358-66. Study limited to adults
Ref ID: 1731

(2869) Lee H, Contento IR, Koch P. Using a systematic conceptual model for a process evaluation of a middle school obesity risk-reduction nutrition curriculum intervention: choice, control & change. Journal of Nutrition Education and Behavior 2013 March;45(2):126-36. Inappropriate Study Design
Ref ID: 5944

(2870) Lee HK. The effects of infant massage on weight, height, and mother-infant interaction. Daehan Ganho Haghoeji 2006 December;36(8):1331-9. Subjects less than 2 years old
Ref ID: 1309

(2871) Lee JM, Appugliese D, Kaciroti N, Corwyn RF, Bradley RH, Lumeng JC. Weight status in young girls and the onset of puberty. Pediatrics 2007;119(3):E624-E630. Longitudinal Study
Ref ID: 5424

(2872) Lee PD, Pivarnik JM, Bukar JG, Muurahainen N, Berry PS, Skolnik PR, Nerad JL, Kudsk KA, Jackson L, Ellis KJ, Gesundheit N. A randomized, placebo-controlled trial of combined insulin-like growth factor I and low dose growth hormone therapy for wasting associated with human immunodeficiency virus infection.[Erratum appears in Journal of Clinical Endocrinology and Metabolism 1996 Oct;81(10):3696]. Journal of Clinical Endocrinology and Metabolism 1996 August;81(8):2968-75. Off topic
Ref ID: 2174

(2873) Lee S, Bacha F, Hannon T, Kuk JL, Boesch C, Arslanian S. Effects of aerobic versus resistance exercise without caloric restriction on abdominal fat, intrahepatic lipid, and insulin sensitivity in obese adolescent boys: a randomized, controlled trial. Diabetes 2012 November;61(11):2787-95. Inappropriate Intervention
Ref ID: 5945

(2874) Lee S, Deldin AR, White D, Kim Y, Libman I, Rivera-Vega M, Kuk JL, Sandoval S, Boesch C, Arslanian S. Aerobic exercise but not resistance exercise reduces intrahepatic lipid content and visceral fat and improves insulin sensitivity in obese adolescent girls: a randomized controlled trial. American Journal of Physiology, Endocrinology and Metabolism 2013 November 15;305(10):E1222-E1229. Inappropriate Intervention
Ref ID: 5946

(2875) Lee S, Burns SF, White D, Kuk JL, Arslanian S. Effects of acute exercise on postprandial triglyceride response after a high-fat meal in overweight black and white adolescents. International Journal of Obesity (London) 2013 July;37(7):966-71. Inappropriate Intervention
Ref ID: 5948

(2876) Lee S, Kuk JL. Changes in fat and skeletal muscle with exercise training in obese adolescents: comparison of whole-body MRI and dual energy X-ray absorptiometry. Obesity (Silver Spring) 2013 October;21(10):2063-71. Inappropriate Intervention
Ref ID: 5947

(2877) Lee SJ, Choi EJ, Kwon JS. A naturalistic multicenter trial of a 12-week weight management program for overweight and obese patients with schizophrenia or schizoaffective disorder. Journal of Clinical Psychiatry 2008 April;69(4):555-62. Diet & Exercise intervention
Ref ID: 947

(2878) Lee WTK, Leung SSF, Leung DMY, Tsang HSY, Lau J, Cheng JCY. A randomized double-blind controlled calcium supplementation trial, and bone and height acquisition in children. British Journal of Nutrition 1995;74(1):125-39. Diet Intervention or Supplement Study
Ref ID: 3257

(2879) Lee YS, Poh LKS, Kek BLK, Loke KY. The role of melanocortin 3 receptor gene in childhood obesity. Diabetes 2007;56(10):2622-30. Off topic
Ref ID: 5425

(2880) Leeners B, Rath W, Kuse S, Neumaier-Wagner P. Breast-feeding in women with hypertensive disorders in pregnancy. Journal of Perinatal Medicine 2005;33(6):553-60. Survey or questionnaire
Ref ID: 5426

(2881) Leermakers EA, Anglin K, Wing RR. Reducing postpartum weight retention through a correspondence intervention. International Journal of Obesity and Related Metabolic Disorders 1998 November;22(11):1103-9. Study limited to adults
Ref ID: 419

(2882) Legato MJ, Gelzer A, Goland R, Ebner SA, Rajan S, Villagra V, Kosowski M, Writing Group for The Partnership for Gender-Specific Medicine. Gender-specific care of the patient with diabetes: review and recommendations. [Review] [194 refs]. Gender Medicine 2006 June;3(2):131-58. Review article
Ref ID: 1369

(2883) Legg S, Jacobs K. Ergonomics for schools. Work-A Journal of Prevention Assessment and Rehabilitation 2008;31(4):489-93. Off topic
Ref ID: 5427

(2884) Legg SJ, Laurs E, Hedderley DI. How safe is cycling with a schoolbag? Ergonomics 2003;46:859-69. Acute study
Ref ID: 4882

(2885) Legido J, Gisbert JP, Pajares JM, Mate J. Bone metabolism changes in patients with inflammatory bowel disease. Revista Espanola de Enfermedades Digestivas 2005;97(11):815-29. Review article
Ref ID: 3258

(2886) Leguire LE, Rogers GL, Walson PD, Bremer DL, McGregor ML. Occlusion and levodopa-carbidopa treatment for childhood amblyopia. Journal of the American Association for Pediatric Ophthalmology 1998;2:257-64. Off topic
Ref ID: 4883

(2887) Lehmkuhl M, Malone M, Justice B, Trone G, Pistilli E, Vinci D, Haff EE, Kilgore JL, Haff GG. The effects of 8 weeks of creatine monohydrate and glutamine supplementation on body composition and performance measures. Journal of Strength and Conditioning Research 2003 August;17(3):425-38. Diet Intervention or Supplement Study
Ref ID: 1721

(2888) Lehouck A, Carremans C, De Bent K, Decramer M, Janssens W. Alveolar and bronchial exhaled nitric oxide in chronic obstructive pulmonary disease. Respiratory Medicine 2010;104(7):1020-6. Case-Control / Case Study
Ref ID: 5428

(2889) Lehtonen-Veromaa M, Mottonen T, Irjala K, Nuotio I, Leino A, Viikari J. A 1-year prospective study on the relationship between physical activity, markers of bone metabolism, and bone acquisition in peripubertal girls. Journal of Clinical Endocrinology and Metabolism 2000 October;85(10):3726-32. Prospective Study
Ref ID: 1952

(2890) Leibel RL, Hirsch J, Appel BE, Checani GC. Energy-intake required to maintain body-weight is not affected by wide variation in diet composition. American Journal of Clinical Nutrition 1992;55(2):350-5. Retrospective study
Ref ID: 5429

(2891) Leidy HJ, Dougherty KA, Frye BR, Duke KM, Williams NI. Twenty-four-hour ghrelin is elevated after calorie restriction and exercise training in non-obese women. Obesity (Silver Spring) 2007 February;15(2):446-55. Study limited to adults
Ref ID: 251

(2892) Leidy HJ, Ortinau LC, Douglas SM, Hoertel HA. Beneficial effects of a higher-protein breakfast on the appetitive, hormonal, and neural signals controlling energy intake regulation in overweight/obese, "breakfast-skipping," late-adolescent girls. American Journal of Clinical Nutrition 2013 April;97(4):677-88.

Inappropriate Intervention
Ref ID: 5949

(2893) Leifke E, Korner HC, Link TM, Behre HM, Peters PE, Nieschlag E. Effects of testosterone replacement therapy on cortical and trabecular bone mineral density, vertebral body area and paraspinal muscle area in hypogonadal men. European Journal of Endocrinology 1998 January;138(1):51-8. Study limited to adults
Ref ID: 2103

(2894) Leijs MM, Koppe JG, Olie K, van Aalderen WMC, de Voogt P, Vulsma T, Westra M, Ten Tusscher GW. Delayed initiation of breast development in girls with higher prenatal dioxin exposure; a longitudinal cohort study. Chemosphere 2008;73(6):999-1004. Cohort Study
Ref ID: 5430

(2895) Leino M, Porkka KV, Raitakari OT, Laitinen S, Taimela S, Viikari JS. Influence of parental occupation on coronary heart disease risk factors in children. The Cardiovascular Risk in Young Finns Study. International Journal of Epidemiology 1996 December;25(6):1189-95. Cross-sectional study
Ref ID: 2150

(2896) Leis A. The conundrum of control group selection. Focus on alternative and complementary therapies 2011;16(3):239-40. Review article
Ref ID: 3259

(2897) Leissner KB, Mahmood FU. Physiology and pathophysiology at high altitude: considerations for the anesthesiologist. Journal of Anesthesia 2009 November;23(4):543-53. Review article
Ref ID: 3663

(2898) Leite N, Milano GE, Cieslak F, Lopes WA, Rodacki A, Radominski RB. Effects of physical exercise and nutritional guidance on metabolic syndrome in obese adolescents. Revista Brasileira de Fisioterapia 2009;13:73-81. No control group (NC)
Ref ID: 459

(2899) Leite N, Milano GE, Cieslak F, Stefanello JMF, Radominski RB. Aptidão cardiorrespiratória, perfil lipídico e metabólico em adolescentes obesos e não-obesos. Cardiorespiratory fitness, lipid and metabolic profile in obese and non-obese adolescents. Revista Brasileira de Educação Física e Esporte 2009 September;23(3):275-82. Not All Participants were Overweight and/or Obese
Ref ID: 4256

(2900) Leite N, Lazarotto L, Cavazza JF, Lopes MdFA, Bento PCB, Torres R, Heyde ME, Cieslak F, Milano GE. Efeitos de exercícios aquáticos e orientação nutricional na composição corporal de crianças e adolescentes obesos. Revista Brasileira de Cineantropometria e Desempenho Humano 2010 August;12(4).

No non-intervention control group
Ref ID: 4257

(2901) Leites GT, Sehl PL, Cunha GS, Detoni FA, Meyer F. Responses of obese and lean girls exercising under heat and thermoneutral conditions. Journal of Pediatrics 2013 May;162(5):1054-60. Inappropriate Study Design
Ref ID: 5950

(2902) Lemmon CR, Ludwig DA, Howe CA, Ferguson-Smith A, Barbeau P. Correlates of adherence to a physical activity program in young African-American girls. Obesity 2007 March;15(3):695-703. Inappropriate Outcomes
Ref ID: 1260

(2903) Lemos LFC, David AC, Teixeira CS, Mota CB. Obesidade infantil e suas relações com o equilíbrio corporal. Acta Fisiátrica 2009;16(3). Review article
Ref ID: 4258

(2904) Lenk S. Proceedings of the 13th Annual meeting of the German Society of Andrology. Andrologia 2002 April;34(2):123-53. Abstract
Ref ID: 3664

(2905) Lenoir G, Antypkin YG, Miano A, Moretti P, Zanda M, Varoli G, Preti PAM, Aryayev NL. Efficacy, Safety, and Local Pharmacokinetics of Highly Concentrated Nebulized Tobramycin in Patients with Cystic Fibrosis Colonized with Pseudomonas aeruginosa. Pediatrics Drugs 2007 June 2;9:11-20. Off topic
Ref ID: 3665

(2906) Leon AS, Sanchez OA. Response of blood lipids to exercise training alone or combined with dietary intervention. [Review] [86 refs]. Medicine and Science in Sports and Exercise 33(6 Suppl):S502-15; discussion S528-9, 2001 Jun 2001;discussion S528-9, 2001 Jun.:-9. Review article
Ref ID: 3041

(2907) Leon AS, Gaskill SE, Rice T, Bergeron J, Gagnon J, Rao DC, Skinner JS, Wilmore JH, Bouchard C. Variability in the response of HDL cholesterol to exercise training in the HERITAGE Family Study. International Journal of Sports Medicine 2002 January;23(1):1-9. Study limited to adults
Ref ID: 1859

(2908) Leonard WR, Spencer GJ, Galloway VA, Osipova L. Declining growth status of indigenous Siberian children in post-Soviet Russia. Human Biology 2002;74(2):197-209. Cross-sectional study
Ref ID: 5431

(2909) Leopold K, Wechsler JG. [Obesity: gradual-schedule therapy and long-term results]. [German]. MMW Fortschritte der Medizin 2001 December 13;143(51-

52):I-VIII. Diet Intervention or Supplement Study
Ref ID: 1864

(2910) León HB, Viramontes JA, Sánchez MED, García CMR. Valoración antropométrica de la composición corporal de Bailarines de ballet: un estudio longitudinal. Revista Brasileira de Cineantropometria e Desempenho Humano 2008 June;10(2):115-22. Longitudinal Study
Ref ID: 4259

(2911) Leppik IE. Three New Drugs for Epilepsy: Levetiracetam, Oxcarbazepine, and Zonisamide. Journal of Child Neurology 2002 January 2;17:S53-S57. Review article
Ref ID: 3666

(2912) Leskinen T, Waller K, Mutikainen S, Aaltonen S, Ronkainen PH, Alen M, Sipila S, Kovanen V, Perhonen M, Pietilainen KH, Cheng S, Suominen H, Kainulainen H, Kaprio J, Kujala UM. Effects of 32-year leisure time physical activity discordance in twin pairs on health (TWINACTIVE study): aims, design and results for physical fitness. Twin Research and Human Genetics 2009 February;12(1):108-17. Not an exercise intervention study
Ref ID: 796

(2913) Lessen BS. Effect of oral stimulation on feeding progression in preterm infants. United States -- Illinois: University of Illinois at Chicago; 2008.
Subjects less than 2 years old
Ref ID: 5105

(2914) Leung ML, Chung PK, Leung RW. An assessment of the validity and reliability of two perceived exertion rating scales among Hong Kong children. Perceptual and Motor Skills 2002 December;95(3:Pt 2):t-62. Study less than 4 weeks
Ref ID: 1786

(2915) Levers-Landis CE, Redline S. Pediatric sleep apnea - Implications of the epidemic of childhood overweight. American Journal of Respiratory and Critical Care Medicine 2007;175(5):436-41. Review article
Ref ID: 5432

(2916) Levine J, Gussow JD, Hastings D, Eccher A. Authors' financial relationships with the food and beverage industry and their published positions on the fat substitute olestra. American Journal of Public Health 2003 April;93(4):664-9. Off topic
Ref ID: 3836

(2917) Levy-Shiff R, Vakil E, Dimitrovsky L, Abramovitz M, Shahar N, Har-Even D, Gross S, Lerman M, Levy I, Sirota L, Fish B. Medical, cognitive, emotional, and behavioral outcomes in school-age children conceived by in-vitro fertilization. Journal of Clinical Child Psychology 1998;27(3):320-9. Off topic
Ref ID: 5433

(2918) Lewis CE, Caan B, Funkhouser E, Hilner JE, Bragg C, Dyer A, Raczynski JM, Savage PJ, Armstrong MA, Friedman GD. Inconsistent associations of caffeine-containing beverages with blood pressure and with lipoproteins. The CARDIA Study. Coronary Artery Risk Development in Young Adults. American Journal of Epidemiology 1993 October 1;138(7):502-7. Study limited to adults
Ref ID: 2254

(2919) Lewis CE, Funkhouser E, Raczynski JM, Sidney S, Bild DE, Howard BV. Adverse effect of pregnancy on high density lipoprotein (HDL) cholesterol in young adult women. The CARDIA Study. Coronary Artery Risk Development in Young Adults. American Journal of Epidemiology 1996 August 1;144(3):247-54. Study limited to adults
Ref ID: 2176

(2920) LEWIS JDN, THOMAS LV, WEIR WILL. The potential of probiotic fermented milk products in reducing risk of antibiotic-associated diarrhoea and Clostridium difficile disease. International Journal of Dairy Technology 2009 November;62(4):461-71. Off topic
Ref ID: 3667

(2921) Lê KA, Ventura EE, Fisher JQ, Davis JN, Weigensberg MJ, Punyanitya M, Hu HH, Nayak KS, Goran MI. Ethnic differences in pancreatic fat accumulation and its relationship with other fat depots and inflammatory markers. Diabetes Care 2011;34:485-90. Not an exercise intervention study
Ref ID: 4884

(2922) Li J, Li H, Wang S. [Effects of calcium supplementation on bone mineral accretion in adolescents]. Journal of Hygiene Research 2002;31:363-6. Diet Intervention or Supplement Study
Ref ID: 4885

(2923) Li JX, Hong Y, Robinson PD. The effect of load carriage on movement kinematics and respiratory parameters in children during walking. European Journal of Applied Physiology 2003 September;90(1-2):35-43. Off topic
Ref ID: 1715

(2924) Li L, Li K, Ushijima H. Moderate-vigorous physical activity and body fatness in Chinese urban school children. Pediatrics International 2007 April;49(2):280-5. Longitudinal Study
Ref ID: 1247

(2925) Li M, Dibley MJ, Yan H. School environment factors were associated with BMI among adolescents in Xi'an City, China. BMC Public Health 2011;11:792. Cross-sectional study
Ref ID: 2652

(2926) Li X, Samei E, Segars WP, Sturgeon GM, Colsher JG, Frush DP. Patient-specific dose estimation for pediatric chest CT. Medical Physics

2008;35(12):5821-8. Off topic
Ref ID: 5434

(2927) Li Y, Wang WJ, Crompton RH, Gunther MM. Free vertical moments and transverse forces in human walking and their role in relation to arm-swing. Journal of Experimental Biology 2001;204(1):47-58. Off topic
Ref ID: 5435

(2928) Li Y, Hu X, Zhang Q, Liu A, Fang H, Hao L, Duan Y, Xu H, Shang X, Ma J, Xu G, Du L, Li Y, Guo H, Li T, Ma G. The nutrition-based comprehensive intervention study on childhood obesity in China (NISCOC): a randomised cluster controlled trial. BMC Public Health 2010;10:229. Not All Participants were Overweight and/or Obese
Ref ID: 2653

(2929) Li YP, Hu XQ, Schouten EG, Liu AL, Du SM, Li LZ, Cui ZH, Wang D, Kok FJ, Hu FB, Ma GS. Report on childhood obesity in China (8): effects and sustainability of physical activity intervention on body composition of Chinese youth. Biomedical and Environmental Sciences 2010 June;23(3):180-7. Not All Participants were Overweight and/or Obese
Ref ID: 26

(2930) Li YC, Wu SK, Cairney J, Hsieh CY. Motor Coordination and Health-Related Physical Fitness of Children with Developmental Coordination Disorder: A Three-Year Follow-up Study. Research in Developmental Disabilities: A Multidisciplinary Journal 2011 January 1;32(6-):2993-3002. Longitudinal Study
Ref ID: 3907

(2931) Liang MTC, Norris S. Effects of Skin Blood-Flow and Temperature on Bioelectric Impedance After Exercise. Medicine and Science in Sports and Exercise 1993;25(11):1231-9. Acute study
Ref ID: 5436

(2932) Liao HF, Liu YC, Liu WY, Lin YT. Effectiveness of loaded sit-to-stand resistance exercise for children with mild spastic diplegia: a randomized clinical trial. Archives of Physical Medicine and Rehabilitation 2007;88:25-31. Not All Participants were Overweight and/or Obese
Ref ID: 4886

(2933) Liao Y, Chang SH, Miyashita M, Stensel D, Chen JF, Wen LT, Nakamura Y. Associations between health-related physical fitness and obesity in Taiwanese youth. Journal of Sports Sciences 2013;31(16):1797-804. Inappropriate Study Design
Ref ID: 5951

(2934) Liao Y, Liao J, Durand CP, Dunton GF. Which type of sedentary behaviour intervention is more effective at reducing body mass index in children? A meta-analytic review. Obesity Reviews 2014 March;15(3):159-68. Inappropriate

Study Design
Ref ID: 5952

(2935) Liberona Z, Castillo V, Rozowski N. Suficiencia de la dieta y composición corporal en un grupo de niños de 11-14 años de dos clubes deportivos en Santiago de Chile. Adequacy of the diet and body composition in a group of 11-14 years old children from two sport clubs in Santiago de Chile. Revista Chilena de Nutrición 2010 June;37(2):145-54. Not an exercise intervention study
Ref ID: 4260

(2936) Libman IM, Arslanian SA. Prevention and treatment of type 2 diabetes in youth. Hormone Research 2007;67(1):22-34. Review article
Ref ID: 3261

(2937) Licea Puig ME, Bustamante Teijido M, Lemane Pérez M. Diabetes tipo 2 en niños y adolescentes: aspectos clínico-epidemiológicos, patogénicos y terapéuticos. Revista Cubana de Endocrinología 2008 April;19(1). Review article
Ref ID: 4261

(2938) Liddell C, Morris C. Fuel poverty and human health: A review of recent evidence. Energy Policy 2010;38(6):2987-97. Review article
Ref ID: 5437

(2939) Lien AS, Cho YH, Tsai JL. [Effectiveness evaluation of healthy lifestyle interventions in childhood obesity prevention: a systematic review]. Hu Li Za Zhi 2013 August;60(4):33-42. Inappropriate Study Design
Ref ID: 5953

(2940) Lien N, Bjelland M, Bergh IH, Grydeland M, Anderssen SA, Ommundsen Y, Andersen LF, Henriksen HB, Randby JS, Klepp KI. Design of a 20-month comprehensive, multicomponent school-based randomised trial to promote healthy weight development among 11-13 year olds: The Health In Adolescents study. Scandinavian Journal of Public Health 2010 November;38(5 Suppl):38-51. No exercise only group
Ref ID: 10

(2941) Lilova M, Kaplan BS, Meyers KEC. Recombinant human growth hormone therapy in autosomal recessive polycystic kidney disease. Pediatric Nephrology 2003;18(1):57-61. Off topic
Ref ID: 5438

(2942) Lim HH, Marriott DA, Potter PD, Clayton KD. Comparison of two methods of training student physical therapists to score the gross motor function measure. Pediatric Physical Therapy 2000;12:127-32. Off topic
Ref ID: 4887

(2943) Lim S, Choi SH, Jeong IK, Kim JH, Moon MK, Park KS, Lee HK, Kim YB, Jang HC. Insulin-sensitizing effects of exercise on adiponectin and retinol-binding protein-4 concentrations in young and middle-aged women. Journal of Clinical Endocrinology and Metabolism 2008 June;93(6):2263-8. Study limited to adults
Ref ID: 942

(2944) Lim SS, Norman RJ, Clifton PM, Noakes M. Psychological effects of prescriptive vs general lifestyle advice for weight loss in young women. Journal of the American Dietetic Association 2009 November;109(11):1917-21. Study limited to adults, No exercise only group
Ref ID: 86

(2945) Lim SS, Norman RJ, Clifton PM, Noakes M. The effect of comprehensive lifestyle intervention or metformin on obesity in young women. Nutrition Metabolism and Cardiovascular Diseases 2011 April;21(4):261-8. Drug intervention study, Lifestyle Intervention
Ref ID: 2655

(2946) Lima AHRdA, Forjaz CLdM, Silva GQdM, Menêses AL, Silva AJMR, Ritti-Dias RM. Efeito agudo da intensidade do exercício de força na modulação autonômica cardíaca pós-exercício. Acute effect of resistance exercise intensity in cardiac autonomic modulation after exercise. Efecto agudo de intensidad del ejercicio de fuerza en la modulación autónoma del corazón post-ejercicio. Arquivos Brasileiros de Cardiologia 2011 June;96(6):498-503. Acute study
Ref ID: 4262

(2947) Lima MAdA, Oliveira MAA, Ferreira HdS. Confiabilidade dos dados antropométricos obtidos em crianças atendidas na Rede Básica de Saúde de Alagoas. Reliability of anthropometric data obtained in children seen at the Primary Public Healthcare Service Network in Alagoas, Brazil. Revista Brasileira de Eidemiologia 2010 March;13(1):69-82. Off topic
Ref ID: 4263

(2948) Limbers CA, Turner EA, Varni JW. Promoting healthy lifestyles: Behavior modification and motivational interviewing in the treatment of childhood obesity. Journal of Clinical Lipidology 2008;2(3):169-78. Review article
Ref ID: 3262

(2949) Limbers CA, Ripperger SJ, Heffer RW, Varni JW. Patient-reported pediatric quality of life inventory and trade; 4.0 generic core scales in pediatric patients with attention-deficit/hyperactivity disorder and comorbid psychiatric disorders: feasibility, reliability, and validity. Value in Health 2011;14:521-30. Survey or questionnaire
Ref ID: 4888

(2950) Lin J, Lai X, Qin J, Song F, Zhang Y, Yao P, Yang X, Liu L. Effect of beta-carotene supplementation on health and growth of vitamin A deficient children

in China rural villages: a randomized controlled trial. European e-Journal of Clinical Nutrition and Metabolism 2009;4:e17-e21. Diet Intervention or Supplement Study
Ref ID: 4889

(2951) Lin PH, Proschan MA, Bray GA, Fernandez CP, Hoben K, Most-Windhauser M, Karanja N, Obarzanek E, DASH Collaborative Research Group. Estimation of energy requirements in a controlled feeding trial. American Journal of Clinical Nutrition 2003 March;77(3):639-45. Diet Intervention Study
Ref ID: 1773

(2952) Lin YC, Lyle RM, McCabe LD, McCabe GP, Weaver CM, Teegarden D. Dairy calcium is related to changes in body composition during a two-year exercise intervention in young women. Journal of the American College of Nutrition 2000 November;19(6):754-60. Study limited to adults
Ref ID: 399

(2953) Lindahl B, Nilsson TK, Asplund K, Hallmans G. Intense nonpharmacological intervention in subjects with multiple cardiovascular risk factors: decreased fasting insulin levels but only a minor effect on plasma plasminogen activator inhibitor activity. Metabolism: Clinical and Experimental 1998 April;47(4):384-90. Diet & Exercise intervention
Ref ID: 2096

(2954) Linde JA, Nygaard KE, MacLehose RF, Mitchell NR, Harnack LJ, Cousins JM, Graham DJ, Jeffery RW. HealthWorks: results of a multi-component group-randomized worksite environmental intervention trial for weight gain prevention. International Journal of Behavioral Nutrition and Physical Activity 2012;9:14. Study limited to adults
Ref ID: 2656

(2955) Linden C, Ahlborg HG, Besjakov J, Gardsell P, Karlsson MK. A school curriculum-based exercise program increases bone mineral accrual and bone size in prepubertal girls: two-year data from the pediatric osteoporosis prevention (POP) study. Journal of Bone and Mineral Research 2006 June;21(6):829-35. Cohort Study
Ref ID: 1387

(2956) Lindquist S, Hernell O. Lipid digestion and absorption in early life: an update. Current Opinion in Clinical Nutrition and Metabolic Care 2010;13(3):314-20. Review article
Ref ID: 5439

(2957) Lindstrom J, Absetz P, Hemio K, Peltomaki P, Peltonen M. Reducing the risk of type 2 diabetes with nutrition and physical activity - efficacy and implementation of lifestyle interventions in Finland. Public Health Nutrition 2010

June;13(6A):993-9. No exercise only group
Ref ID: 41

(2958) Linetzky B, Morello P, Virgolini M, Ferrante D. Resultados de la primera encuesta nacional de salud escolar: Argentina, 2007. Results from the first national school health survey: Argentina, 2007. Archivos Argentinos de Pediatría 2011 April;109(2):111-6. Survey or questionnaire
Ref ID: 4264

(2959) Linne Y, Dye L, Barkeling B, Rossner S. Weight development over time in parous women--the SPAWN study--15 years follow-up. International Journal of Obesity and Related Metabolic Disorders 2003 December;27(12):1516-22. Follow-up Study
Ref ID: 1699

(2960) Lioret S, Campbell KJ, Crawford D, Spence AC, Hesketh K, McNaughton SA. A parent focused child obesity prevention intervention improves some mother obesity risk behaviors: the Melbourne inFANT program. International Journal of Behavioral Nutrition and Physical Activity 2012;9:100. Inappropriate Population
Ref ID: 5954

(2961) Liou YM, Liou TH, Chang LC. Obesity among adolescents: sedentary leisure time and sleeping as determinants. Journal of Advanced Nursing 2010 June;66(6):1246-56. Cross-sectional study
Ref ID: 513

(2962) Lipp MEN, Saraiva JFK, Afiune Neto A, Diament J, Rivera IR, Silva MAMd. Aspectos psicológicos na prevenção da aterosclerose na infância e na adolescência. Psychological aspects of preventing atherosclerosis in children and adolescents. Revista de Ciências Médicas (Campinas) 2006;15(6):515-24. Review article
Ref ID: 4265

(2963) Lippa NC, Sanderson SC. Impact of information about obesity genomics on the stigmatization of overweight individuals: an experimental study. Obesity (Silver Spring) 2012 December;20(12):2367-76. Inappropriate Intervention
Ref ID: 5955

(2964) Lippo BRdS, Silva IMd, Aca CRP, Lira PICd, Silva GAPd, Motta MEFA. Fatores determinantes de inatividade física em adolescentes de área urbana. Determinants of physical inactivity among urban adolescents. Jornal de Pediatria 2010 December;86(6):520-4. Case-Control / Case Study
Ref ID: 4267

(2965) Littlewood JM, Wolfe SP. Control of Malabsorption in Cystic Fibrosis. Pediatrics Drugs 2000 May;2(3):205-22. Off topic
Ref ID: 517

(2966) Liu K, Ruth KJ, Flack JM, Jones-Webb R, Burke G, Savage PJ, Hulley SB. Blood pressure in young blacks and whites: relevance of obesity and lifestyle factors in determining differences. The CARDIA Study. Coronary Artery Risk Development in Young Adults. Circulation 1996 January 1;93(1):60-6. Study limited to adults
Ref ID: 2185

(2967) Liu Y, Wang J, Zhang R, Zhang Y, Xu Q, Zhang J, Zhang Y, Zheng Z, Yu X, Jing H, Nosaka N, Kasai M, Aoyama T, Wu J, Xue C. A good response to oil with medium- and long-chain fatty acids in body fat and blood lipid profiles of male hypertriglyceridemic subjects. Asia Pacific Journal of Clinical Nutrition 2009;18(3):351-8. Diet Intervention or Supplement Study
Ref ID: 670

(2968) Liu Z, Sun F, Li J, Han Y, Wei Q, Liu C. Application of acupuncture and moxibustion for keeping shape. Journal of Traditional Chinese Medicine 1998 December;18(4):265-71. Off topic
Ref ID: 2064

(2969) Liusuwan RA, Widman LM, Abresch RT, Johnson AJ, McDonald CM. Behavioral intervention, exercise, and nutrition education to improve health and fitness (BENEfit) in adolescents with mobility impairment due to spinal cord dysfunction. Journal of Spinal Cord Medicine 2007;30:Suppl-26. Behavior Modification Intervention
Ref ID: 1166

(2970) Livingston BP, Segal RL, Song A, Hopkins K, English AW, Manning CC. Functional activation of the extensor carpi radialis muscles in humans. Archives of Physical Medicine and Rehabilitation 2001;82(9):1164-70. Off topic
Ref ID: 5440

(2971) Livolsi JM, Adams GM, Laguna PL. The effect of chromium picolinate on muscular strength and body composition in women athletes. Journal of Strength and Conditioning Research 2001 May;15(2):161-6. Study limited to adults
Ref ID: 1877

(2972) Lizana Arce PJ, Almagià Flores AA, Simpson Lelievre MC, Binvignat Gutiérrez O, Ivanovic Marincovich D, Berral de la Rosa FJ. Aproximación a la tendencia secular del estado nutricional y composición corporal en escolares de enseñanza secundaria, V Región, Chile: 1985-2010. Approximation to the secular tendency of the nutritional state and body composition of high school students, V Region, Chile: 1985-2010. International Journal of Morphology 2011 June;29(2):473-8. Cohort Study
Ref ID: 4268

(2973) Llargues E, Franco R, Recasens A, Nadal A, Vila M, Perez MJ, Manresa JM, Recasens I, Salvador G, Serra J, Roure E, Castells C. Assessment of a school-based intervention in eating habits and physical activity in school children: the AVall study. Journal of Epidemiology and Community Health 2011 October;65(10):896-901. Diet & Exercise intervention
Ref ID: 1051

(2974) Lloyd-Richardson EE, Jelalian E, Sato AF, Hart CN, Mehlenbeck R, Wing RR. Two-year follow-up of an adolescent behavioral weight control intervention. Pediatrics 2012 August;130(2):e281-e288. Inappropriate Study Design
Ref ID: 5956

(2975) Lloyd M, Burghardt A, Ulrich DA, Angulo-Barroso R. Physical Activity and Walking Onset in Infants with Down Syndrome. Adapted Physical Activity Quarterly 2010 January 1;27(1):1-16. Subjects less than 2 years old
Ref ID: 3908

(2976) Lobanco CM, Vedovato GM, Cano C, Bastos DHM. Fidedignidade de rótulos de alimentos comercializados no município de São Paulo, SP. Fidedignidad de rótulos de alimentos comercializados en municipio de Sao Paulo, Sureste de Brasil. Reliability of food labels from products marketed in the city of São Paulo, Southeastern Brazil. Revista de Saúde Pública 2009 June;43(3):499-505. Off topic
Ref ID: 4269

(2977) Lobelo F, Liese AD, Liu J, Mayer-Davis EJ, D'Agostino RB, Jr., Pate RR, Hamman RF, Dabelea D. Physical activity and electronic media use in the SEARCH for diabetes in youth case-control study. Pediatrics 2010 June;125(6):e1364-e1371. Case-Control / Case Study
Ref ID: 518

(2978) Lobet S, Detrembleur C, Francq B, Hermans C. Natural progression of blood-induced joint damage in patients with haemophilia: clinical relevance and reproducibility of three-dimensional gait analysis. Haemophilia 2010 September;16(5):813-21. Off topic
Ref ID: 3668

(2979) Lobstein T, Baur L, Uauy R. Obesity in children and young people: A crisis in public health. Obesity Reviews Suppl 2004;5(1):4-104. Review article
Ref ID: 3263

(2980) LodÃ©n M. The clinical benefit of moisturizers. Journal of the European Academy of Dermatology and Venereology 2005 November;19(6):672-88. Off topic
Ref ID: 3669

(2981) LodÃ©n M. Role of Topical Emollients and Moisturizers in the Treatment of Dry Skin Barrier Disorders. American Journal of Clinical Dermatology 2003

November;4(11):771-88. Off topic
Ref ID: 3670

(2982) Lofshult D. dieting & eating disorders in teens. IDEA Fitness Journal 2006 October;3(9):74. Description of study from review or magazine or etc. (not the actual study)
Ref ID: 3890

(2983) Lofshult D. to vitamin B or not to vitamin B. IDEA Fitness Journal 2006 October;3(9):74. Off topic
Ref ID: 3889

(2984) Lofshult D. resource to make kids media savvy. IDEA Fitness Journal 2006 October;3(9):74. Description of study from review or magazine or etc. (not the actual study)
Ref ID: 3891

(2985) Logan JG, Cook JI, Stanczyk NM, Weeks ENI, Welham SJ, Luntz AJ. To bite or not to bite! A questionnaire-based survey assessing why some people are bitten more than others by midges. BMC Public Health 2010;10. Off topic
Ref ID: 5441

(2986) Logue EE, Bourguet CC, Palmieri PA, Scott ED, Matthews BA, Dudley P, Chipman KJ. The better weight-better sleep study: a pilot intervention in primary care. American Journal of Health Behavior 2012 March;36(3):319-34. Study limited to adults
Ref ID: 2658

(2987) Lohman TG. Measurement of Body Composition in Children. Journal of Physical Education, Recreation and Dance 1982 October 1;53(7):67-70. Review article
Ref ID: 3909

(2988) Lohman TG, Ring K, Pfeiffer K, Camhi S, Arredondo E, Pratt C, Pate R, Webber LS. Relationships among fitness, body composition, and physical activity. Medicine and Science in Sports and Exercise 2008 June;40(6):1163-70. Cross-sectional study
Ref ID: 949

(2989) Lombard C, Deeks A, Jolley D, Teede HJ. Preventing weight gain: the baseline weight related behaviors and delivery of a randomized controlled intervention in community based women. BMC Public Health 2009;9:2. Study limited to adults
Ref ID: 804

(2990) Lombard C, Deeks A, Jolley D, Ball K, Teede H. A low intensity, community based lifestyle programme to prevent weight gain in women with young children: cluster randomised controlled trial. British Medical Journal 2010 July 13;341:c3215. doi: 10.1136/bmj.c3215.:c3215. Study not limited to children and

adolescents
Ref ID: 32

(2991) Lombard CB, Deeks AA, Ball K, Jolley D, Teede HJ. Weight, physical activity and dietary behavior change in young mothers: short term results of the HeLP-her cluster randomized controlled trial. Nutrition Journal 2009;8:17. Study limited to adults
Ref ID: 749

(2992) Lomelí C, Mendoza-González C, Méndez A, Lorenzo JA, Buendía A, Férez-Santander SM, Attie F. Hipertensión arterial sistémica en el niño y adolescente. Hypertension in children and adolescence. Archivos de Cardiología de México 2008 June;78(supl.2):S2-82. Review article
Ref ID: 4270

(2993) London B, Albert C, Anderson ME, Giles WR, Van Wagoner DR, Balk E, Billman GE, Chung M, Lands W, Leaf A, McAnulty J, Martens JR, Costello RB, Lathrop DA. Omega-3 fatty acids and cardiac arrhythmias: Prior studies and recommendations for future research - A report from the national heart, lung, and blood institute and office of dietary supplements omega-3 fatty acids and their role in cardiac arrhythmogenesis workshop. Circulation 2007;116(10):e320-e335. Review article
Ref ID: 3264

(2994) Long SJ, Hart K, Morgan LM. The ability of habitual exercise to influence appetite and food intake in response to high- and low-energy preloads in man. British Journal of Nutrition 2002 May;87(5):517-23. Study limited to adults
Ref ID: 380

(2995) Longworth B, Fary R, Hopper D. Prevalence and predictors of adolescent idiopathic scoliosis in adolescent ballet dancers. Archives of Physical Medicine and Rehabilitation 2014 September;95(9):1725-30. Inappropriate Study Design
Ref ID: 5957

(2996) Loomba R, Sirlin CB, Schwimmer JB, Lavine JE. Advances in pediatric nonalcoholic fatty liver disease. Hepatology 2009;50(4):1282-93. Off topic
Ref ID: 3265

(2997) Lopes AA, Soares RPS, Maeda NY. A mathematical framework for group analysis of von Willebrand factor multimeric composition following luminography. Brazilian Journal of Medical and Biological Research 2002 November;35(11):1259-63. Off topic
Ref ID: 4271

(2998) Lopes FL, Pereira FM, Reboredto MM, Castro TM, Vianna JM, Novo Júnior JM, Silva LP. Redução da variabilidade da freqüência cardíaca em indivíduos de meia-idade e o efeito do treinamento de força. Revista Brasileira de

Fisioterapia 2007 April;11(2):113-9. Study limited to adults
Ref ID: 620

(2999) Lopes PCS, Prado SRLdA, Colombo P. Fatores de risco associados à obesidade e sobrepeso em crianças em idade escolar. Risk factors associated with obesity and overweight in school children. Factores de riesgo asociados a la obesidad y sobrepeso de niños en edad escolar. Revista Brasileira de Enfermagem 2010 February;63(1):73-8. Survey or questionnaire
Ref ID: 4272

(3000) Lopes WA, Rosário N, Leite N. Broncoespasmo induzido pelo exercício em adolescentes asmáticos obesos e não-obesos. Exercise-induced bronchospasm in obese and non-obese asthmatic adolescents. Revista Paulista de Pediatria 2010 March;28(1):36-40. Cross-sectional study
Ref ID: 4273

(3001) Lopez Stewart G, Tambascia M, Rosas Guzmán J, Etchegoyen F, Ortega Carrión J, Artemenko S. Control of type 2 diabetes mellitus among general practitioners in private practice in nine countries of Latin America. Revista Panamericana de Salud Pública 2007 July;22(1):12-20. Cross-sectional study
Ref ID: 4274

(3002) Lopez GA, Rodriguez G, I, Almagro Martin-Lomena P, Garofano GR, Fernandez Cano EM, Maldonado BA. [To find out the life habits and risk factors of adolescents seen in the Health Centres of two semi-urban populations using a structured open response clinical interview]. [Spanish]. Atencion Primaria 2011 April;43(4):176-82. Cross-sectional study
Ref ID: 2659

(3003) Lopez KN, Knudson JD. Obesity: From the Agricultural Revolution to the Contemporary Pediatric Epidemic. Congenital Heart Disease 2012;7(2):189-99. Review article
Ref ID: 5442

(3004) Lopez P, Ledoux M, Garrel DR. Increased thermogenic response to food and fat oxidation in female athletes: relationship with VO(2 max). American Journal of Physiology - Endocrinology and Metabolism 2000 September;279(3):E601-E607. Off topic
Ref ID: 1962

(3005) Lopez SG, Tambascia M, Rosas GJ, Etchegoyen F, Ortega CJ, Artemenko S. Control of type 2 diabetes mellitus among general practitioners in private practice in nine countries of Latin America. Pan American Journal of Public Health 2007 July;22(1):12-20. Cross-sectional study
Ref ID: 1155

(3006) Lorenzo J, Ives JC, Sforzo GA. Knowledge and imagery of contractile mechanisms do not improve muscle strength. Perceptual and Motor Skills 2003

August;97(1):141-6. Off topic
Ref ID: 1703

(3007) Loria-Kohen V, Fernandez-Fernandez C, Bermejo LM, Morencos E, Romero-Moraleda B, Gomez-Candela C. Effect of different exercise modalities plus a hypocaloric diet on inflammation markers in overweight patients: a randomised trial. Clinical Nutrition 2013 August;32(4):511-8. Inappropriate Population
Ref ID: 5958

(3008) Lott MJ, Galloway SD. Fluid balance and sodium losses during indoor tennis match play. International Journal of Sport Nutrition and Exercise Metabolism 2011 December;21(6):492-500. Off topic
Ref ID: 2660

(3009) Lottenberg SA, Glezer A, Turatti LA. Síndrome metabólica: identificando fatores de risco: [revisão]. Metabolic syndrome: identifying the risk factors: [review]. Jornal de Pediatria 2007 November;83(5,supl):S204-S208. Review article
Ref ID: 4275

(3010) Loucks AB, Verdun M, Heath EM. Low energy availability, not stress of exercise, alters LH pulsatility in exercising women. Journal of Applied Physiology 1998 January;84(1):37-46. Off topic
Ref ID: 2105

(3011) Lourenco AE, Santos RV, Orellans JDY, Coimbra CEA. Nutrition transition in Amazonia: Obesity and socioeconomic change in the Surui Indians from Brazil. American Journal of Human Biology 2008;20(5):564-71. Off topic
Ref ID: 5443

(3012) Love-Osborne K, Sheeder J, Zeitler P. Addition of metformin to a lifestyle modification program in adolescents with insulin resistance. Journal of Pediatrics 2008 June;152(6):817-22. No exercise only group, Drug intervention study
Ref ID: 182

(3013) Love AL, Billett HH. Obesity, bariatric surgery, and iron deficiency: true, true, true and related. [Review] [69 refs]. American Journal of Hematology 2008 May;83(5):403-9. Review article
Ref ID: 967

(3014) Lovelady CA, Garner KE, Moreno KL, Williams JP. The effect of weight loss in overweight, lactating women on the growth of their infants. New England Journal of Medicine 2000 February 17;342(7):449-53. Study not limited to children and adolescents
Ref ID: 404

(3015) Lovelady CA, Williams JP, Garner KE, Moreno KL, Taylor ML, Leklem JE. Effect of energy restriction and exercise on vitamin B-6 status of women during

lactation. Medicine and Science in Sports and Exercise 33(4):512-8, 2001 Apr 2001;(4):512-8. Study limited to adults
Ref ID: 3046

(3016) Lovell RJ, Kirke I, Siegler J, McNaughton LR, Greig MP. Soccer half-time strategy influences thermoregulation and endurance performance. Journal of Sports Medicine and Physical Fitness 2007 September;47(3):263-9. Off topic
Ref ID: 1195

(3017) Lovely R, Hossain J, Ramsey JP, Komakula V, George D, Farrell DH, Balagopal PB. Obesity-related increased gamma' fibrinogen concentration in children and its reduction by a physical activity-based lifestyle intervention: a randomized controlled study. Journal of Pediatrics 2013 August;163(2):333-8. Inappropriate Intervention
Ref ID: 5959

(3018) Loveman E, Frampton GK, Shepherd J, Picot J, Cooper K, Bryant J, Welch K, Clegg A. The clinical effectiveness and cost-effectiveness of long-term weight management schemes for adults: a systematic review. [Review]. Health Technology Assessment (Winchester, England) 2011 January;15(2):1-182. Review article
Ref ID: 2661

(3019) Lowe MR, Butryn ML, Thomas JG, Coletta M. Meal replacements, reduced energy density eating, and weight loss maintenance in primary care patients: a randomized controlled trial. Obesity (Silver Spring) 2014 January;22(1):94-100. Inappropriate Intervention
Ref ID: 5960

(3020) Lozada BB. Dismenorrea en atletas. Revista Argentina de Medicina del Deporte 1992;15(48):71-2. Review article
Ref ID: 4276

(3021) López Díaz Zd. Cirugía neonatal maxilofacial: 10 años de experiencia 1994 a 2005. Revista Cubana de Pediatria 2007 September;79(3). Subjects less than 2 years old
Ref ID: 4277

(3022) López E. MÁ. Etapas del cambio conductual ante la ingesta de frutas y verduras, control de peso y ejercicio físico de estudiantes de la Universidad del Desarrollo, sede Concepción, Chile. Revista Chilena de Nutrición 2008 September;35(3):215-24. Cross-sectional study
Ref ID: 4278

(3023) López S. A, Sotomayor S. L, Álvarez G, Céspedes A, Poblete A, Escobar C, Vásquez M. Rendimiento aeróbico en niños obesos de 6 a 10 años.
Aerobic performance in obese children of 6 to 10 years old. Revista Chilena de

Pediatría 2009 October;80(5):444-50. Cross-sectional study
Ref ID: 4279

(3024) Lu MC, Kotelchuck M, Culhane JF, Hobel CJ, Klerman LV, Thorp J. Preconception care between pregnancies: The content of internatal care. Maternal and Child Health Journal 2006;10(SUPPL. 7):107-22. Off topic
Ref ID: 3266

(3025) Lu SR, Su J, Xiang QY, Zhang FY, Wu M. Active transport and health outcomes: findings from a population study in Jiangsu, China. Journal of Environmental and Public Health 2013;2013:624194. Inappropriate Study Design
Ref ID: 5961

(3026) Lu Y, Wang X, Li Q, Li J, Yan Y. Tolerability and safety of topiramate in Chinese patients with epilepsy : an open-label, long-term, prospective study. Clinical Drug Investigation 2007;27:683-90. Drug intervention study
Ref ID: 4891

(3027) Lubans DR, Sheaman C, Callister R. Exercise adherence and intervention effects of two school-based resistance training programs for adolescents. Preventive Medicine 2010 January;50(1-2):56-62. Not All Participants were Overweight and/or Obese
Ref ID: 596

(3028) Lubans DR, Morgan PJ, Callister R, Collins CE, Plotnikoff RC. Exploring the mechanisms of physical activity and dietary behavior change in the program x intervention for adolescents. Journal of Adolescent Health 2010 July;47(1):83-91. No exercise only group
Ref ID: 38

(3029) Lubans DR, Morgan PJ, Dewar D, Collins CE, Plotnikoff RC, Okely AD, Batterham MJ, Finn T, Callister R. The Nutrition and Enjoyable Activity for Teen Girls (NEAT girls) randomized controlled trial for adolescent girls from disadvantaged secondary schools: rationale, study protocol, and baseline results. BMC Public Health 2010 October 28;10:652.:652. Description versus conduct of study, No exercise only group
Ref ID: 16

(3030) Lubans DR, Morgan PJ, Aguiar EJ, Callister R. Randomized controlled trial of the Physical Activity Leaders (PALs) program for adolescent boys from disadvantaged secondary schools. Preventive Medicine 2011 March;52(3-4):239-46. Not All Participants were Overweight and/or Obese
Ref ID: 2664

(3031) Lubans DR, Morgan P, Callister R, Plotnikoff RC, Eather N, Riley N, Smith CJ. Test-retest reliability of a battery of field-based health-related fitness measures

for adolescents. Journal of Sports Sciences 2011 April;29(7):685-93. Off topic
Ref ID: 2663

(3032) Lubans DR, Plotnikoff RC, Morgan PJ, Dewar D, Costigan S, Collins CE. Explaining dietary intake in adolescent girls from disadvantaged secondary schools. A test of Social Cognitive Theory. Appetite 2012 April;58(2):517-24. Diet Intervention Study
Ref ID: 2665

(3033) Lubans DR, Morgan PJ, Callister R. Potential moderators and mediators of intervention effects in an obesity prevention program for adolescent boys from disadvantaged schools. Journal of Science and Medicine in Sport 2012 November;15(6):519-25. Inappropriate Intervention
Ref ID: 5962

(3034) Luca-Moretti M, Grandi A, Luca E, Muratori G, Nofroni MG, Mucci MP, Gambetta P, Stimolo R, Drago P, Giudice G, Tamburlin N, Karbalai M, Valente C, Moras G. Master Amino acid Pattern as sole and total substitute for dietary proteins during a weight-loss diet to achieve the body's nitrogen balance equilibrium. Advances in Therapy 2003 September;20(5):270-81. Diet Intervention or Supplement Study
Ref ID: 1677

(3035) Luca-Moretti M, Grandi A, Luca E, Muratori G, Nofroni MG, Mucci MP, Gambetta P, Stimolo R, Drago P, Giudice G, Tamburlin N. Master Amino acid Pattern as substitute for dietary proteins during a weight-loss diet to achieve the body's nitrogen balance equilibrium with essentially no calories. Advances in Therapy 2003 September;20(5):282-91. Diet Intervention or Supplement Study
Ref ID: 1676

(3036) Lucareli PR, Lima MO, Lima FP, de Almeida JG, Brech GC, D'Andrea Greve JM. Gait analysis following treadmill training with body weight support versus conventional physical therapy: a prospective randomized controlled single blind study. Spinal Cord 2011 September;49(9):1001-7. Off topic
Ref ID: 2666

(3037) Luchetta M, Kayenne C, Formiga MR, Tudella E. Antecedentes obstétricos/neonatais e desenvolvimento motor-adaptativo de crianças pré-termo e com baixo peso entre 24 e 36 meses de idade. Fisioterapia Brasileira 2007 August;8(4):255-60. Subjects less than 2 years old
Ref ID: 4280

(3038) Luckner H, Moss JR, Gericke CA. Effectiveness of interventions to promote healthy weight in general populations of children and adults: a meta-analysis. European Journal of Public Health 2012 August;22(4):491-7. Inappropriate Study Design
Ref ID: 5963

(3039) Ludvigsson J, Huus K, Eklov K, Klintstrom R, Lahdenpera A. Fasting plasma glucose levels in healthy preschool children: effects of weight and lifestyle. Acta Paediatrica 2007 May;96(5):706-9. Prospective Study
Ref ID: 1242

(3040) Ludwig DS. Weight loss strategies for adolescents: a 14-year-old struggling to lose weight. Journal of the American Medical Association 2012 February 1;307(5):498-508. Case-Control / Case Study
Ref ID: 2667

(3041) Luepker RV, Perry CL, McKinlay SM, Nader PR, Parcel GS, Stone EJ, Webber LS, Elder JP, Feldman HA, Johnson CC. Outcomes of a field trial to improve children's dietary patterns and physical activity. The Child and Adolescent Trial for Cardiovascular Health. CATCH collaborative group. Journal of the American Medical Association 1996 March 13;275(10):768-76. Not All Participants were Overweight and/or Obese
Ref ID: 2182

(3042) Luger EJ, Nissan M, Karpf A, Steinberg EL, Dekel S. Patterns of weight distribution under the metatarsal heads. Journal of Bone and Joint Surgery - British Volume 1999 March;81(2):199-202. Off topic
Ref ID: 1107

(3043) Luiz AMAG, Gorayeb R, Liberatore Júnior RDR, Domingos NAM. Depressão, ansiedade e competência social em crianças obesas. Estudos de Psicologia (Natal) 2005 April;10(1):35-9. Review article, Duplicate
Ref ID: 646

(3044) Luiz AMAG, Gorayeb R, Liberatore Júnior RDR, Domingos NAM. Depressão, ansiedade, competência social e problemas comportamentais em crianças obesas. Estudos de Psicologia (Natal) 2005 December;10(3):371-5. Review article
Ref ID: 4281

(3045) Lukaski HC, Siders WA. Validity and accuracy of regional bioelectrical impedance devices to determine whole-body fatness. Nutrition 2003;19(10):851-7. Off topic
Ref ID: 5444

(3046) Lumeng JC, Appugliese D, Cabral HJ, Bradley RH, Zuckerman B. Neighborhood safety and overweight status in children. Archives of Pediatrics and Adolescent Medicine 2006;160(1):25-31. Cross-sectional study
Ref ID: 5446

(3047) Lumeng JC, Rahnama S, Appugliese D, Kaciroti N, Bradley RH. Television exposure and overweight risk in preschoolers. Archives of Pediatrics and Adolescent Medicine 2006;160(4):417-22. Cross-sectional study
Ref ID: 5445

(3048) Lunardi CC, Moreira CM, Santos DLd. Colesterolemia, trigliceridemia e excesso de peso em escolares de Santa Maria, RS, Brasil. Blood lipids abnormalities and overweight prevalence in students of Santa Maria, RS, Brazil. Revista Brasileira de Medicina do Esporte 2010 August;16(4):250-3. Cross-sectional study
Ref ID: 4282

(3049) Lundervold A, Taxt T, Ersland L, Fenstad AM. Volume distribution of cerebrospinal fluid using multispectral MR imaging. Medical Image Analysis 2000;4(2):123-36. Off topic
Ref ID: 5447

(3050) Lustig RH, Hinds PS, Ringwald-Smith K, Christensen RK, Kaste SC, Schreiber RE, Rai SN, Lensing SY, Wu S, Xiong X. Octreotide therapy of pediatric hypothalamic obesity: a double-blind, placebo-controlled trial. Journal of Clinical Endocrinology and Metabolism 2003 June;88(6):2586-92. Not an exercise intervention study
Ref ID: 363

(3051) Luszczynska A, Sobczyk A, Abraham C. Planning to lose weight: randomized controlled trial of an implementation intention prompt to enhance weight reduction among overweight and obese women. Health Psychology 2007 July;26(4):507-12. Study limited to adults
Ref ID: 228

(3052) Lutter CK, Mora JO, Habicht JP, Rasmussen KM, Robson DS, Herrera MG. Age-specific responsiveness of weight and length to nutritional supplementation. American Journal of Clinical Nutrition 1990 March;51(3):359-64. Subjects less than 2 years old
Ref ID: 2315

(3053) Lyle RM, Weaver CM, Sedlock DA, Rajaram S, Martin B, Melby CL. Iron status in exercising women: the effect of oral iron therapy vs increased consumption of muscle foods. American Journal of Clinical Nutrition 1992 December;56(6):1049-55. Study limited to adults
Ref ID: 2279

(3054) Lyseng-Williamson KA, Yang LPH. Topiramate: A Review of its Use in the Treatment of Epilepsy. Drugs 2007 September;67(15):2231-56. Review article
Ref ID: 3671

(3055) Lytle LA. Lessons from the Child and Adolescent Trial for Cardiovascular Health (CATCH): interventions with children. [Review] [36 refs]. Current Opinion in Lipidology 1998 February;9(1):29-33. Review article
Ref ID: 2101

(3056) Lytle LA, Murray DM, Perry CL, Story M, Birnbaum AS, Kubik MY, Varnell S. School-based approaches to affect adolescents' diets: results from the TEENS

study. Health Education and Behavior 2004;31:270-87. Diet Intervention Study
Ref ID: 4892

(3057) Lytle LA, Pasch KE, Farbakhsh K. The relationship between sleep and weight in a sample of adolescents. Obesity 2011 February;19(2):324-31. Cross-sectional study
Ref ID: 1094

(3058) Ma J, Strub P, Camargo CA, Jr., Xiao L, Ayala E, Gardner CD, Buist AS, Haskell WL, Lavori PW, Wilson SR. The Breathe Easier through Weight Loss Lifestyle (BE WELL) Intervention: a randomized controlled trial. BMC Pulmonary Medicine 2010 March 24;10:16.:16. Study not limited to children and adolescents
Ref ID: 54

(3059) Maahs D, de Serna DG, Kolotkin RL, Ralston S, Sandate J, Qualls C, Schade DS. Randomized, double-blind, placebo-controlled trial of orlistat for weight loss in adolescents. Endocrine Practice 2006 January;12(1):18-28. No exercise only group, Drug intervention study
Ref ID: 281

(3060) Macaulay M, Pettersson L, Fader M, Brooks R, Cottenden A. A multicenter evaluation of absorbent products for children with incontinence and disabilities. Journal of Wound, Ostomy, and Continence Nursing 2004;31:235-44. Off topic
Ref ID: 4893

(3061) Macdonald HM, Kontulainen SA, Khan KM, McKay HA. Is a school-based physical activity intervention effective for increasing tibial bone strength in boys and girls? Journal of Bone and Mineral Research 2007;22:434-46. Not All Participants were Overweight and/or Obese
Ref ID: 4894

(3062) MacEneaney OJ, Harrison M, O'Gorman DJ, Pankratieva EV, O'Connor PL, Moyna NM. Effect of prior exercise on postprandial lipemia and markers of inflammation and endothelial activation in normal weight and overweight adolescent boys. European Journal of Applied Physiology 2009 July;106(5):721-9. Acute study, Not a randomized controlled trial (RCT)
Ref ID: 120

(3063) Machado FA, Denadai BS. Influência das variáveis antropométricas nas respostas cardiorrespiratórias de crianças durante o esforço. Influence of anthropometrics variables on the cardiorespiratory responses of children during exercise. Revista Brasileira de Cineantropometria e Desempenho Humano 2011 October;13(5):378-83. Acute study
Ref ID: 4283

(3064) Machado HdS, Campos Wd, Silva SGd. Relaçäo entre composiçäo corporal e a performance de padröes motores fundamentais em escolares. Revista

Brasileira de Atividade Física e Saúde 2002;7(1):63-70. Cross-sectional study
Ref ID: 4284

(3065) Machado M, Pereira R, Jorge FS, Knifis F, Hackney A. Creatine supplementation: effects on blood creatine kinase activity responses to resistance exercise and creatine kinase activity measuremen.
A suplementação de creatina: efeitos sobre o sangue de creatina quinase respostas ao exercício de resistência e de medida da atividade da creatina quinase. Brazilian Journal of Pharmaceutical Sciences 2009 December;45(4):751-7. Diet Intervention or Supplement Study
Ref ID: 4285

(3066) Machado Z, Krebs RJ. Crescimento físico de escolares da ilha de Santa Catarina. Revista Brasileira de Cineantropometria e Desempenho Humano 2001;3(1). Cross-sectional study
Ref ID: 4286

(3067) Machado Z, Krebs RJ, Prestes JD, Santos MBd, Santos JOLd, Nobre GC, Ramalho MHdS. Crescimento físico e estado nutricional de escolares: estudo comparativo - 1997 e 2009. Comparative study of physical growth and nutritional status of schoolchildren (1997 and 2009). Revista Brasileira de Crescimento e Desenvolvimento Humano 2011 June;13(3):216-22. Cross-sectional study
Ref ID: 4287

(3068) Machann J, Stefan N, Schick F. H-1 MR spectroscopy of skeletal muscle, liver and bone marrow. European Journal of Radiology 2008;67(2):275-84. Off topic
Ref ID: 5448

(3069) Macias-Cervantes MH, Malacara JM, Garay-Sevilla ME, Diaz-Cisneros FJ. Effect of recreational physical activity on insulin levels in Mexican/Hispanic children. European Journall of Pediatrics 2009 October;168(10):1195-202. Not All Participants were Overweight and/or Obese
Ref ID: 139

(3070) Macias BR, Murthy G, Chambers H, Hargens AR. Asymmetric loads and pain associated with backpack carrying by children. Journal of Pediatric Orthopedics 2008 July;28(5):512-7. Off topic
Ref ID: 930

(3071) MacKelvie KJ, McKay HA, Khan KM, Crocker PR. A school-based exercise intervention augments bone mineral accrual in early pubertal girls. Journal of Pediatrics 2001 October;139(4):501-8. Not All Participants were Overweight and/or Obese
Ref ID: 1888

(3072) MacKelvie KJ, McKay HA, Petit MA, Moran O, Khan KM. Bone mineral response to a 7-month randomized controlled, school-based jumping

intervention in 121 prepubertal boys: associations with ethnicity and body mass index. Journal of Bone and Mineral Research 2002 May;17(5):834-44. Not All Participants were Overweight and/or Obese
Ref ID: 1830

(3073) MacKelvie KJ, Khan KM, Petit MA, Janssen PA, McKay HA. A school-based exercise intervention elicits substantial bone health benefits: a 2-year randomized controlled trial in girls. Pediatrics 2003 December;112(6:Pt 1):t. Not All Participants were Overweight and/or Obese
Ref ID: 1691

(3074) MacKelvie KJ, Petit MA, Khan KM, Beck TJ, McKay HA. Bone mass and structure are enhanced following a 2-year randomized controlled trial of exercise in prepubertal boys. Bone 2004 April;34(4):755-64. Inappropriate Outcomes
Ref ID: 1655

(3075) MacKelvie KJ, Meneilly GS, Elahi D, Wong ACK, Barr SI, Chanoine JP. Regulation of appetite in lean and obese adolescents after exercise: Role of acylated and desacyl ghrelin. Journal of Clinical Endocrinology and Metabolism 2007;92(2):648-54. Diet Intervention or Supplement Study
Ref ID: 5449

(3076) Mackie HW, Stevenson JM, Reid SA, Legg SJ. The effect of simulated school load carriage configurations on shoulder strap tension forces and shoulder interface pressure. Applied Ergonomics 2005;36(2):199-206. Off topic
Ref ID: 5450

(3077) MacLaughlin HL, Cook SA, Kariyawasam D, Roseke M, van NM, Macdougall IC. Nonrandomized trial of weight loss with orlistat, nutrition education, diet, and exercise in obese patients with CKD: 2-year follow-up. American Journal of Kidney Diseases 2010 January;55(1):69-76. Not a randomized controlled trial (RCT)
Ref ID: 606

(3078) MacMillan K. Evaluación del estado nutricional, hábitos de alimentación y actividad física en escolares de 1º básico de Isla de Pascua. Revista Chilena de Nutrición 2005 December;32(3):232-7. Survey or questionnaire
Ref ID: 4289

(3079) MacMillan K. Valoración de hábitos de alimentación, actividad física y condición nutricional en estudiantes de la Pontificia Universidad Católica de Valparaíso. Evaluation of eating habits and physical activity patterns and nutritional status in Valparaiso Catholic University students. Revista Chilena de Nutrición 2007 December;34(4):330-6. Survey or questionnaire
Ref ID: 4290

(3080) Madanmohan, Mahadevan SK, Balakrishnan S, Gopalakrishnan M, Prakash ES. Effect of six weeks yoga training on weight loss following step test, respiratory pressures, handgrip strength and handgrip endurance in young healthy subjects. Indian Journal of Physiology and Pharmacology 2008 April;52(2):164-70. Not a randomized controlled trial (RCT)
Ref ID: 834

(3081) Maddison R, Foley L, Mhurchu CN, Jull A, Jiang Y, Prapavessis H, Rodgers A, Vander HS, Hohepa M, Schaaf D. Feasibility, design and conduct of a pragmatic randomized controlled trial to reduce overweight and obesity in children: The electronic games to aid motivation to exercise (eGAME) study. BMC Public Health 2009 May;%19;9:146.:146. Description versus conduct of study
Ref ID: 116

(3082) Maddison R, Foley L, Ni MC, Jiang Y, Jull A, Prapavessis H, Hohepa M, Rodgers A. Effects of active video games on body composition: a randomized controlled trial. American Journal of Clinical Nutrition 2011 July;94(1):156-63. Met criteria but could not retrieve data
Ref ID: 2670

(3083) Maddison R, Mhurchu CN, Foley L, Epstein L, Jiang Y, Tsai M, Dewes O, Heke I. Screen-time weight-loss intervention targeting children at home (SWITCH): a randomized controlled trial study protocol. BMC Public Health 2011;11:524. Description versus conduct of study
Ref ID: 2669

(3084) Madic D, Maric D, Obradovic B, Obradovic J, Fratric F, Buncic V, Popovic B, Tumin D, Varga J, Pantovic M. Effects of swimming training on body composition and bone mineral density of prepubertal boys. Healthmed 2011;5(6):2237-42. Not a randomized controlled trial (RCT)
Ref ID: 5451

(3085) Madrid V, Torrejón G, Rivera F, Madrid P. Ciclosporosis: informe de un caso clínico en Concepción, Chile. Revista médica de Chile 1998 May;126(5):559-62. Off topic
Ref ID: 4291

(3086) Madrigal Loría A, González Urrutia AR. Estado nutricional de niños con síndrome down del Centro Nacional de Educación Especial de Costa Rica. Revista Costarricense de Salud Pública 2009 December;18(2):72-8. Off topic
Ref ID: 4292

(3087) Madsen J, Sallis JF, Rupp JW, Senn KL, Patterson TL, Atkins CJ, Nader PR. Relationship between self-monitoring of diet and exercise change and subsequent risk factor changes in children and adults. Patient Education and

Counseling 1993 June;21(1-2):61-9. Diet & Exercise intervention
Ref ID: 2258

(3088) Madsen K, Thompson H, Adkins A, Crawford Y. School-community partnerships: A cluster-randomized trial of an after-school soccer program. JAMA Pediatrics 2013 April;167(4):321-6. Inappropriate Population
Ref ID: 5964

(3089) Madsen KA. School-based body mass index screening and parent notification: a statewide natural experiment. Archives of Pediatrics and Adolescent Medicine 2011 November;165(11):987-92. Cross-sectional study
Ref ID: 2671

(3090) Maeda S, Miyauchi T, Goto K, Matsuda M. Alteration of plasma endothelin-1 by exercise at intensities lower and higher than ventilatory threshold. Journal of Applied Physiology 1994 September;77(3):1399-402. Off topic
Ref ID: 2223

(3091) Maeda S, Miyauchi T, Sakane M, Saito M, Maki S, Goto K, Matsuda M. Does endothelin-1 participate in the exercise-induced changes of blood flow distribution of muscles in humans? Journal of Applied Physiology 1997 April;82(4):1107-11. Off topic
Ref ID: 2146

(3092) Mafart B. Anthropological study of Holocene human footprints of the sebkra El Azrag (Islamic Republic of Mauritania). Anthropologie 2006;110(5):766-87. Off topic
Ref ID: 5453

(3093) Maffeis C, Pinelli L, Zaffanello M, Schena F, Iacumin P, Schutz Y. Daily Energy-Expenditure in Free-Living Conditions in Obese and Nonobese Children - Comparison of Doubly Labeled Water ((H2O)-H-2-O-18) Method and Heart-Rate Monitoring. International Journal of Obesity 1995;19(9):671-7. Cross-sectional study
Ref ID: 5454

(3094) Maffeis C, Zaffanello M, Schutz Y. Relationship between physical inactivity and adiposity in prepubertal boys. Journal of Pediatrics 1997;131(2):288-92. Cross-sectional study
Ref ID: 5455

(3095) Maffiuletti NA, De CA, Agosti F, Ottolini S, Moro D, Genchi M, Massarini M, Lafortuna CL, Sartorio A. Effect of a 3-week body mass reduction program on body composition, muscle function and motor performance in pubertal obese boys and girls. Journal of Endocrinological Investigation 2004 October;27(9):813-20. Study less than 4 weeks
Ref ID: 1561

(3096) Mafla AC. Adolescencia: cambios bio-psicosociales y salud oral. Colombia Médica 2008 March;39(1):41-57. Review article
Ref ID: 4293

(3097) Magarey AM, Perry RA, Baur LA, Steinbeck KS, Sawyer M, Hills AP, Wilson G, Lee A, Daniels LA. A parent-led family-focused treatment program for overweight children aged 5 to 9 years: the PEACH RCT. Pediatrics 2011;127:214-22. Lifestyle Intervention
Ref ID: 4895

(3098) Maggio AB, Aggoun Y, Martin XE, Marchand LM, Beghetti M, Farpour-Lambert NJ. Long-term follow-up of cardiovascular risk factors after exercise training in obese children. International Journal of Pediatric Obesity 2011 June;6(2-2):e603-e610. Follow-up Study
Ref ID: 2672

(3099) Magnago TSBdS, Lisboa MTL, Griep RH, Kirchhof ALC, Camponogara S, Nonnenmacher CdQ, Vieira LB. Condições de trabalho, características sociodemográficas e distúrbios musculoesqueléticos em trabalhadores de enfermagem. Condiciones de trabajo, características sociodemográficas y disturbios músculo-esqueléticos en trabajadores de enfermería. Nursing workers: Work conditions, social-demographic characteristics and skeletal muscle disturbances. Acta paul enferm 2010 April;23(2):187-93. Off topic
Ref ID: 4294

(3100) Magnusson KT, Sveinsson T, Arngrimsson SA, Johannsson E. Predictors of fatness and physical fitness in nine-year-old Icelandic school children. International Journal of Pediatric Obesity 2008;3(4):217-25. Cross-sectional study
Ref ID: 855

(3101) Magnusson KT, Hrafnkelsson H, Sigurgeirsson I, Johannsson E, Sveinsson T. Limited effects of a 2-year school-based physical activity intervention on body composition and cardiorespiratory fitness in 7-year-old children. Health Education Research 2012 June;27(3):484-94. Diet & Exercise intervention
Ref ID: 2673

(3102) Maguiña M. Estrategias nutricionales en el tratamiento de la obesidad en niños y adolescentes. Renut 2008 December;2(6):260-6. Diet Intervention Study
Ref ID: 4295

(3103) Maguire CM, Veen S, Sprij AJ, Le CS, Wit JM, Walther FJ, Leiden-Developmental CP. Effects of basic developmental care on neonatal morbidity, neuromotor development, and growth at term age of infants who were born at <32 weeks. Pediatrics 2008;121:e239-e245. Subjects less than 2 years old
Ref ID: 4896

(3104) Mahadevan M, Graff L. Prospective randomized study of analgesic use for ED patients with right lower quadrant abdominal pain. The American journal of emergency medicine 2000;18:753-6. Off topic
Ref ID: 4897

(3105) Maharaj VR, Dookie T, Ince S, Marsang BL, Rambocas N, Chin M, McDougall L, Teelucksingh S. Knowledge, attitudes and practices of anabolic steroid usage among gym users in Trinidad. West Indian Medical Journal 2000 March;49(1):55-8. Off topic
Ref ID: 4296

(3106) Maher-Edwards G, Dixon R, Hunter J, Gold M, Hopton G, Jacobs G, Hunter J, Williams P. SB-742457 and donepezil in Alzheimer disease: a randomized, placebo-controlled study. International Journal of Geriatric Psychiatry 2011 May;26(5):536-44. Off topic
Ref ID: 3672

(3107) Maher C, Olds TS, Eisenmann JC, Dollman J. Screen time is more strongly associated than physical activity with overweight and obesity in 9- to 16-year-old Australians. Acta Paediatrica 2012 November;101(11):1170-4. Inappropriate Study Design
Ref ID: 5965

(3108) Maher CA, Williams MT, Olds T, Lane AE. An internet-based physical activity intervention for adolescents with cerebral palsy: a randomized controlled trial. Developmental Medicine and Child Neurology 2010;52:448-55. Not All Participants were Overweight and/or Obese
Ref ID: 4898

(3109) Maier IB, Stricker L, Ozel Y, Wagnerberger S, Bischoff SC, Bergheim I. A low fructose diet in the treatment of pediatric obesity: A pilot study. Pediatrics International 2011;53(3):303-8. Diet Intervention Study
Ref ID: 5456

(3110) Maitino E. Fatores de risco da doença coronária em escolares de ensino fundamental. Salusvita 2001;20(2):37-62. Cross-sectional study
Ref ID: 4297

(3111) Maïano C, Ninot G, Erraïs B. Effects of alternated sport competition in perceived competence for adolescent males with mild to moderate mental retardation. International journal of rehabilitation research Internationale Zeitschrift für Rehabilitationsforschung Revue internationale de recherches de réadaptation 2001;24:51-8. Inappropriate Outcomes
Ref ID: 4899

(3112) Majumdar I, Paul P, Talib VH, Ranga S. The effect of iron therapy on the growth of iron-replete and iron-deplete childrenJournal of Tropical Pediatrics

2003;49(2):84-8. Diet Intervention or Supplement Study
Ref ID: 3267

(3113) Maki KC, Curry LL, Carakostas MC, Tarka SM, Reeves MS, Farmer MV, McKenney JM, Toth PD, Schwartz SL, Lubin BC, Dicklin MR, Boileau AC, Bisognano JD. The hemodynamic effects of rebaudioside A in healthy adults with normal and low-normal blood pressure. Food and Chemical Toxicology 2008 July;46:Suppl-6. Study limited to adults
Ref ID: 927

(3114) Makimura H, Stanley TL, Sun N, Hrovat MI, Systrom DM, Grinspoon SK. The Association of Growth Hormone Parameters with Skeletal Muscle Phosphocreatine Recovery in Adult Men. Journal of Clinical Endocrinology and Metabolism 2011;96(3):817-23. Study limited to adults
Ref ID: 5457

(3115) Makkes S, Halberstadt J, Renders CM, Bosmans JE, van der Baan-Slootweg OH, Seidell JC. Cost-effectiveness of intensive inpatient treatments for severely obese children and adolescents in the Netherlands; a randomized controlled trial (HELIOS). BMC Public Health 2011;11:518. Description versus conduct of study
Ref ID: 2674

(3116) Malavolti M, Pietrobelli A, Dugoni M, Poli M, Romagnoli E, De Cristofaro P, Battistini NC. A new device for measuring resting energy expenditure (REE) in healthy subjects. Nutrition Metabolism and Cardiovascular Diseases 2007;17(5):338-43. Off topic
Ref ID: 5458

(3117) Malina RM, Eisenmann JC, Cumming SP, Ribeiro B, Aroso J. Maturity-associated variation in the growth and functional capacities of youth football (soccer) players 13-15 years. European Journal of Applied Physiology 2004 May;91(5-6):555-62. Cross-sectional study
Ref ID: 1643

(3118) Malina RM, Morano PJ, Barron M, Miller SJ, Cumming SP, Kontos AP, Little BB. Overweight and obesity among youth participants in American football. Journal of Pediatrics 2007 October;151(4):378-82. Cross-sectional study
Ref ID: 1162

(3119) Mallan KM, Nambiar S, Magarey AM, Daniels LA. Satiety responsiveness in toddlerhood predicts energy intake and weight status at four years of age. Appetite 2014 March;74:79-85. Inappropriate Study Design
Ref ID: 5966

(3120) Mallion JM, Siche JP, LacourciÃ¨re Y. ABPM comparison of the antihypertensive profiles of the selective angiotensin II receptor antagonists telmisartan and losartan in patients with mild-to-moderate hypertension. Journal

of Human Hypertension 1999 October;13(10):657. Drug intervention study
Ref ID: 3673

(3121) Mallios PL. Faulkner's Indians, or The Poetics of Cannibalism. Faulkner Journal 18[1/2], 143. 2002. Faulkner Journal. Off topic,
Ref ID: 3674

(3122) Malloy J, Capparelli E, Gottschalk M, Guan X, Kothare P, Fineman M. Pharmacology and tolerability of a single dose of exenatide in adolescent patients with type 2 diabetes mellitus being treated with metformin: a randomized, placebo-controlled, single-blind, dose-escalation, crossover study. Clinical Therapeutics 2009 April;31(4):806-15. Drug intervention study
Ref ID: 752

(3123) Maloney AE, Bethea TC, Kelsey KS, Marks JT, Paez S, Rosenberg AM, Catellier DJ, Hamer RM, Sikich L. A pilot of a video game (DDR) to promote physical activity and decrease sedentary screen time. Obesity 2008 September;16(9):2074-80. Not All Participants were Overweight and/or Obese
Ref ID: 826

(3124) Malta DC, Moura EC, Morais Neto OLd. Gender and schooling inequalities in risk and protective factors for chronic diseases among Brazilian adults, through telephone survey. Desigualdades de sexo e escolaridade em fatores de risco e proteção para doenças crônicas em adultos Brasileiros, por meio de inquéritos telefônicos. Revista Brasileira de Eidemiologia 2011 September;14(supl.1):125-35. Survey or questionnaire
Ref ID: 4298

(3125) Malta DC, Oliveira MRd, Moura ECd, Silva SA, Zouain CS, Santos FPd, Morais Neto OLd, Penna GdO. Fatores de risco e proteção para doenças crônicas não transmissíveis entre beneficiários da saúde suplementar: resultados do inquérito telefônico Vigitel, Brasil, 2008. Prevalence of risk health behavior among members of private health insurance plans: results from the 2008 national telephone survey Vigitel, Brazil. Ciência and Saúde Coletiva 2011 March;16(3):2011-22. Survey or questionnaire
Ref ID: 4299

(3126) Maltais D, Bar-Or O, Pierrynowski M, Galea V. Repeated treadmill walks affect physiologic responses in children with cerebral palsy. Medicine and Science in Sports and Exercise 2003 October;35(10):1653-61. Not a randomized controlled trial (RCT)
Ref ID: 1709

(3127) Maltais D, Unnithan V, Wilk B, Bar-Or O. Responses of children with cerebral palsy to arm-crank exercise in the heat. Medicine and Science in Sports and Exercise 2004 February;36(2):191-7. Off topic
Ref ID: 1667

(3128) Mamalakis G, Kafatos A, Manios Y, Kalogeropoulos N, Andrikopoulos N. Adipose fat quality vs. quantity: relationships with children's serum lipid levels. Preventive Medicine 2001 December;33(6):525-35. Not an exercise intervention study, Observational study
Ref ID: 391

(3129) ManeJ, Pedresa E, Loren V, Gassull MA, Espadaler J, Cune J, Audivert S, Bonachera MA, Cabre. A mixture of Lactobacillus plantarum CECT 7315 and CECT 7316 enhances systemic immunity in elderly subjects. A dose-response, double-blind, placebo-controlled, randomized pilot trial. Nutricion Hospitalaria 2011 January;26(1):228-35. Diet Intervention or Supplement Study
Ref ID: 3675

(3130) Manca D, LiMuller ASM, Bell RW. Application of a predictive approach to estimate exposure to non-smoking urban sub-populations to background levels of benzene in Ontario. Human and Ecological Risk Assessment 1997;3(3):415-37. Off topic
Ref ID: 5459

(3131) Mancini M, Strazzullo P. Energy balance and blood-pressure regulation. Update and future perspectives. [Review] [23 refs]. Journal of Clinical Hypertension 1986 June;2(2):148-53. Review article
Ref ID: 2347

(3132) Manios Y, Kafatos A, Mamalakis G. The effects of a health education intervention initiated at first grade over a 3 year period: physical activity and fitness indices. Health Education Research 1998 December;13(4):593-606. Cohort Study
Ref ID: 2066

(3133) Manios Y, Moschandreas J, Hatzis C, Kafatos A. Evaluation of a health and nutrition education program in primary school children of Crete over a three-year period. Preventive Medicine 1999 February;28(2):149-59. Diet & Exercise intervention
Ref ID: 2047

(3134) Manios Y, Moschandreas J, Hatzis C, Kafatos A. Health and nutrition education in primary schools of Crete: Changes in chronic disease risk factors following a 6-year intervention programme. British Journal of Nutrition 2002;88:315-24. Diet & Exercise intervention
Ref ID: 4900

(3135) Mann JI. Can dietary intervention produce long-term reduction in insulin resistance?. [Review] [24 refs]. British Journal of Nutrition 2000 March;83:Suppl-72. Review article
Ref ID: 1973

(3136) Manning S, Younis RT. Management of post-tympanostomy tube otorrhea in children. ENT: Ear, Nose and Throat Journal 2006 October 2;85:8-11. Off topic
Ref ID: 3676

(3137) Manolio TA, Savage PJ, Burke GL, Hilner JE, Liu K, Orchard TJ, Sidney S, Oberman A. Correlates of fasting insulin levels in young adults: the CARDIA study. Journal of Clinical Epidemiology 1991;44(6):571-8. Study limited to adults
Ref ID: 2300

(3138) Manolio TA, Burke GL, Savage PJ, Sidney S, Gardin JM, Oberman A. Exercise blood pressure response and 5-year risk of elevated blood pressure in a cohort of young adults: the CARDIA study. American Journal of Hypertension 1994 March;7(3):234-41. Study limited to adults
Ref ID: 2243

(3139) Manrique-Hurtado H, Acosta-Chacaltana M, Aro-Guardia P, Solís-Villanueva J. Diabetes mellitus tipo 2 en adolescentes: reporte de tres casos. Revista de la Sociedad Peruana de Medicina Interna 2011 June;24(2):81-5. Case-Control / Case Study
Ref ID: 4300

(3140) Manson JE, Bassuk SS, Stampfer MJ. Does Vitamin E Supplementation Prevent Cardiovascular Events? Journal of Women's Health (15409996) 2003 March;12(2):123. Diet Intervention or Supplement Study
Ref ID: 3677

(3141) Mantovani RM, Viana MdFS, Cunha SB, Moura LCRd, Oliveira JMd, Carvalho FFd, Castro JC, Silva ACS. Obesidade na infância e adolescência. Childhood and adolescent obesity. Revista Médica de Minas Gerais 2008 November;18(4,supl.1):S107-S118. Review article
Ref ID: 4301

(3142) Manuela M. Xenobiotics as Stress Factors for Living Organisms. Seria de Monografii de Biochimie Cuantica si Interactii Specifice 2006 June;6:1-90. Off topic
Ref ID: 3678

(3143) Manzur AY, Muntoni F. The management of Duchenne muscular dystrophy. Current Paediatrics 2002;12(4):261-8. Off topic
Ref ID: 3268

(3144) Marani F, Oliveira F, Guedes DP. Indicadores comportamentais associados à prática de atividade físicae saúde em escolares do ensino médio. Revista Brasileira de Ciência e Movimento 2006;14(4):63-70. Survey or questionnaire
Ref ID: 4302

(3145) Marani F, Oliveira ARd, Guedes DP. Indicadores comportamentais associados à prática de atividade física e saúde em escolares do ensino médio. Revista Brasileira de Ciência e Movimento 2007;15(2):39-46. Duplicate
Ref ID: 4303

(3146) Marant C, Arnould B, Marrel A, Spizak CÃ, Colombel JFdr, Faure P, Hagege H, Lemann M, Nahon Sp, Tucat G, Vandromme L, Thibout E, Goldfarb GÃ. Assessing patients' satisfaction with anti-TNFÎ± treatment in Crohn's disease: qualitative steps of the development of a new questionnaire. Clinical and Experimental Gastroenterology 2011 January;4:173-80. Off topic
Ref ID: 3679

(3147) Marceau K, Ram N, Houts RM, Grimm KJ, Susman EJ. Individual Differences in Boys' and Girls' Timing and Tempo of Puberty: Modeling Development With Nonlinear Growth Models. Developmental Psychology 2011;47(5):1389-409. Off topic
Ref ID: 5460

(3148) Marcellin P. Hepatitis B and hepatitis C in 2009. Liver International 2009 January 2;29:1-8. Off topic
Ref ID: 3680

(3149) Marchant WA, Walker I. Anaesthetic management of the child with sickle cell disease. Paediatric Anaesthesia 2003 July;13(6):473-89. Off topic
Ref ID: 3681

(3150) Marcos-Daccarett NJ, Núñez-Rocha GM, Martínez AM, Santos-Ayarzagoitia M, Decanini-Arcaute H. Obesidad como Factor de Riesgo para Trastornos Metabólicos en Adolescentes Mexicanos, 2005. Revista de Salud Pública 2007 June;9(2):180-93. Cross-sectional study
Ref ID: 4304

(3151) Marcovecchio M, Mohn A, Chiarelli F. Type 2 diabetes mellitus in children and adolescents. Journal of Endocrinological Investigation 2005;28(9):853-63. Review article
Ref ID: 3269

(3152) Marcu M, Trivin C, Souberbielle JC, Brauner R. Factors influencing the growth hormone peak and plasma insulin-like growth factor I in young adults with pituitary stalk interruption syndrome. BMC Endocrine Disorders 2008 January;8:1-6. Off topic
Ref ID: 3682

(3153) Marcus BH, Lewis BA, King TK, Albrecht AE, Hogan J, Bock B, Parisi AF, Abrams DB. Rationale, design, and baseline data for Commit to Quit II: an evaluation of the efficacy of moderate-intensity physical activity as an aid to smoking cessation in women. Preventive Medicine 2003 April;36(4):479-92.

Study limited to adults
Ref ID: 1764

(3154) Marcus C, Nyberg G, Nordenfelt A, Karpmyr M, Kowalski J, Ekelund U. A 4-year, cluster-randomized, controlled childhood obesity prevention study: STOPP. International Journal of Obesity (London) 2009 April;33(4):408-17. No exercise only group
Ref ID: 132

(3155) Mardis AL. Current knowledge of the health effects of sugar intake. Family Economics and Nutrition Review 2001 March;13(1):87. Off topic
Ref ID: 3837

(3156) Mardynskii I, Lopatin VF, Bizer VA. [Local ultrasound hyperthermia as a component of radiotherapy for osteogenic sarcoma of tubular bones in children and adolescents]. [Russian]. Voprosy Onkologii 2007;53(5):584-8. Off topic
Ref ID: 1123

(3157) Maresh CM, Bergeron MF, Kenefick RW, Castellani JW, Hoffman JR, Armstrong LE. Effect of overhydration on time-trial swim performance. Journal of Strength and Conditioning Research 2001 November;15(4):514-8. Off topic
Ref ID: 1873

(3158) Maricich SM, Azizi P, Jones JY, Morriss MC, Hunter JV, Smith EO, Miller G. Myelination as assessed by conventional MR imaging is normal in young children with idiopathic developmental delay. American Journal of Neuroradiology 2007;28(8):1602-5. Off topic
Ref ID: 5461

(3159) MARIJE C, V. Effects of Ingestion of Bicarbonate, Citrate, Lactate, and Chloride on Sprint Running. Medicine and Science in Sports and Exercise 2004 July;36(7):1239-43. Diet Intervention or Supplement Study
Ref ID: 3838

(3160) Marild S, Gronowitz E, Forsell C, Dahlgren J, Friberg P. A controlled study of lifestyle treatment in primary care for children with obesity. Pediatric Obesity 2013 June;8(3):207-17. Inappropriate Intervention
Ref ID: 5967

(3161) Markovic G, Jaric S. Movement performance and body size: the relationship for different groups of tests. European Journal of Applied Physiology 2004 June;92(1-2):139-49. Off topic
Ref ID: 1621

(3162) Markovic G, Jaric S. Scaling of muscle power to body size: the effect of stretch-shortening cycle. European Journal of Applied Physiology 2005 September;95(1):11-9. Off topic
Ref ID: 1445

(3163) Marliss EB, Kreisman SH, Manzon A, Halter JB, Vranic M, Nessim SJ. Gender differences in glucoregulatory responses to intense exercise. Journal of Applied Physiology 2000 February;88(2):457-66. Off topic
Ref ID: 1990

(3164) Marques de AT, Avesani CM, Brasileiro RS, de Abreu Carvalhaes JT. Resting energy expenditure of children and adolescents undergoing hemodialysis. Journal of Renal Nutrition 2008 May;18(3):312-9. Cross-sectional study
Ref ID: 972

(3165) Marques AH, Silverman MN, Sternberg EM. Glucocorticoid Dysregulations and Their Clinical Correlates. Annals of the New York Academy of Sciences 2009 October 20;1179(1):1-18. Review article
Ref ID: 3683

(3166) Marques RdMB, Fornés NS, Stringhini MLF. Fatores socioeconômicos, demográficos, nutricionais e de atividade física no controle glicêmico de adolescentes portadores de diabetes melito tipo 1. Socioeconomic, demographic, nutritional, and physical activity factors in the glycemic control of adolescents with type 1 diabetes mellitus. Arquivos Brasileiros de Endocrinologia and Metabologia 2011 April;55(3):194-202. Cross-sectional study
Ref ID: 4305

(3167) Marquez SS, Perry AC, Kaplan TA, Halberstein RA, Signorile JF. Physical and psychological changes with vigorous exercise in sedentary primigravidae. Medicine and Science in Sports and Exercise 2000;32:58-62. Study limited to adults
Ref ID: 4901

(3168) Marsh AB, DiPonio L, Yamakawa K, Khurana S, Haig AJ. Changes in posture and perceived exertion in adolescents wearing backpacks with and without abdominal supports. American Journal of Physical Medicine and Rehabilitation 85(6):509-15, 2006 Jun 2006;(6):509-15. Off topic
Ref ID: 3054

(3169) Marsh S, Foley LS, Wilks DC, Maddison R. Family-based interventions for reducing sedentary time in youth: a systematic review of randomized controlled trials. Obesity Reviews 2014 February;15(2):117-33. Inappropriate Study Design
Ref ID: 5968

(3170) Marshall JD, Bouffard M. Obesity and movement competency in children. Adapted Physical Activity Quarterly 1994;11:297-305. Not All Participants were Overweight and/or Obese
Ref ID: 1027

(3171) Marshall JD, Bouffard M. The effects of quality daily physical education on movement competency in obese versus nonobese children. Adapted Physical Activity Quarterly 1997;14:222-37. Not All Participants were Overweight and/or Obese
Ref ID: 4902

(3172) Marson SM, Wei G, Wasserman D. A Reliability Analysis of Goal Attainment Scaling (GAS) Weights. American Journal of Evaluation 2009;30(2):203-16. Off topic
Ref ID: 5462

(3173) Marti--Carvajal AJ, Pena-Marti GE, Comunian-Carrasco G, Marti-Pena AJ. Interventions for treating painful sickle cell crisis during pregnancy. Cochrane Database of Systematic Reviews 2009;(1). Off topic
Ref ID: 3270

(3174) Martelo S. Efeitos da educação nutricional associada à prática de exercício físico supervisionado sobre indicadores da composição corporale marcadores bioquímicos em adolescentes com excesso de peso. Effects of nutritional ducation associated with exercise on body composition and biochemical parameters in adolescents with excess weight. Nutrire Revista da Sociedade Brasileira de Alimentação e Nutrição 2009 December;34(3). Diet & Exercise intervention
Ref ID: 4306

(3175) Martin-Almendros MI, Martinez-Gonzalez MA, De Irala-Estevez J, Gibney M, Kearney J, Martinez JA. [The perceptions of the adult Spanish population of the factors determinative of health]. [Spanish]. Atencion Primaria 1999 November 30;24(9):514-22. Cross-sectional study
Ref ID: 2056

(3176) Martin A, Saunders DH, Shenkin SD, Sproule J. Lifestyle intervention for improving school achievement in overweight or obese children and adolescents. Cochrane Database of Systematic Reviews 2014;3:CD009728. Inappropriate Study Design
Ref ID: 5969

(3177) Martin CK, Han H, Anton SD, Greenway FL, Smith SR. Effect of valproic acid on body weight, food intake, physical activity and hormones: results of a randomized controlled trial. Journal of Psychopharmacology 2009 September;23(7):814-25. Drug intervention study
Ref ID: 697

(3178) Martin CK, Redman LM, Zhang J, Sanchez M, Anderson CM, Smith SR, Ravussin E. Lorcaserin, a 5-HT(2C) receptor agonist, reduces body weight by decreasing energy intake without influencing energy expenditure. Journal of Clinical Endocrinology and Metabolism 2011 March;96(3):837-45. Drug

intervention study
Ref ID: 1080

(3179) Martin J, Chater A, Lorencatto F. Effective behaviour change techniques in the prevention and management of childhood obesity. International Journal of Obesity (London) 2013 October;37(10):1287-94. Inappropriate Study Design
Ref ID: 5970

(3180) Martin RJ, Dore E, Hautier CA, Van PE, Bedu M. Short-term peak power changes in adolescents of similar anthropometric characteristics. Medicine and Science in Sports and Exercise 2003 August;35(8):1436-40. Not an exercise intervention study
Ref ID: 1727

(3181) Martin S. [Nonpharmacological diabetes therapy]. [Review] [80 refs] [German]. Medizinische Klinik 990 January;101(12):973-89. Review article
Ref ID: 1314

(3182) Martine LG, Fonseca LMM, Scochi CGS. The participation of parents in the care of premature children in a neonatal unit: Meanings attributed by the health team. Revista Latino-Americana de Enfermagem 2007;15(2):239-46. Off topic
Ref ID: 5463

(3183) Martinez-Andrade GO, Cespedes EM, Rifas-Shiman SL, Romero-Quechol G, Gonzalez-Unzaga MA, Benitez-Trejo MA, Flores-Huerta S, Horan C, Haines J, Taveras EM, Perez-Cuevas R, Gillman MW. Feasibility and impact of Creciendo Sanos, a clinic-based pilot intervention to prevent obesity among preschool children in Mexico City. BMC Pediatrics 2014;14:77. Inappropriate Intervention
Ref ID: 5971

(3184) Martinez-Gomez D, Gomez-Martinez S, Puertollano MA, Nova E, Warnberg J, Veiga OL, Marti A, Campoy C, Garagorri JM, Azcona C, Vaquero MP, Redondo-Figuero C, Delgado M, Martinez JA, Garcia-Fuentes M, Moreno LA, Marcos A, EVASYON Study Group. Design and evaluation of a treatment programme for Spanish adolescents with overweight and obesity. The EVASYON Study. BMC Public Health 2009;9:414. Cohort Study
Ref ID: 642

(3185) Martinez-Gomez D, Ortega FB, Ruiz JR, Vicente-Rodriguez G, Veiga OL, Widhalm K, Manios Y, Beghin L, Valtuena J, Kafatos A, Molnar D, Moreno LA, Marcos A, Castillo MJ, Sjostrom M, HELENA Study Group. Excessive sedentary time and low cardiorespiratory fitness in European adolescents: the HELENA study. Archives of Disease in Childhood 2011 March;96(3):240-6. Cross-sectional study
Ref ID: 2676

(3186) Martinez-Gomez D, Gomez-Martinez S, Ruiz JR, Diaz LE, Ortega FB, Widhalm K, Cuenca-Garcia M, Manios Y, De VT, Molnar D, Huybrechts I, Breidenassel C, Gottrand F, Plada M, Moreno S, Ferrari M, Moreno LA, Sjostrom M, Marcos A, HELENA Study Group. Objectively-measured and self-reported physical activity and fitness in relation to inflammatory markers in European adolescents: the HELENA Study. Atherosclerosis 2012 March;221(1):260-7. Cross-sectional study
Ref ID: 2677

(3187) Martinez-Vizcaino V, Salcedo AF, Franquelo GR, Solera MM, Sanchez LM, Serrano MS, Lopez GE, Rodriguez AF. Assessment of an after-school physical activity program to prevent obesity among 9- to 10-year-old children: a cluster randomized trial. International Journal of Obesity (London) 2008 January;32(1):12-22. Not All Participants were Overweight and/or Obese
Ref ID: 216

(3188) Martinez J, Merino S. Host-parasite interactions under extreme climatic conditions. Current Zoology 2011 June;57(3):390-405. Off topic
Ref ID: 3684

(3189) Martino F, Martino E, Morrone F, Carnevali E, Forcone R, Niglio T. Effect of dietary supplementation with glucomannan on plasma total cholesterol and low density lipoprotein cholesterol in hypercholesterolemic children. Nutrition, Metabolism and Cardiovascular Diseases 2005;15(3):174-80. Diet Intervention or Supplement Study
Ref ID: 3271

(3190) Martins EJ, Santos DAd, Thomé MR, Vieira FF, Pereira WM, Ferreira LAB, Kerppers II. Avaliação podal de crianças do ensino fundamental de uma escola pública. Terapia Manual 2009 April;7(30):117-22. Off topic
Ref ID: 4307

(3191) Martins GB, Moreira AA, Viana FdO. Reconstrução de lesões de partes moles do calcanhar com o uso de retalhos fasciocutâneos. Revista Brasileira de Cirurgia Plástica 2009 March;24(1):104-9. Off topic
Ref ID: 4308

(3192) Martins MdCdC, Ricarte IF, Rocha CHL, Maia RB, Silva VBd, Veras AB, Souza Filho MDd. Pressão arterial, excesso de peso e nível de atividade física em estudantes de universidade pública. Blood pressure, excess weight and level of physical activity in students of a public university. Arquivos Brasileiros de Cardiologia 2010 August;95(2):192-9. Cross-sectional study
Ref ID: 4309

(3193) Martins N, Williams DC, Ratan RA, Harrison K. Virtual muscularity: A content analysis of male video game characters. Body Image 2011;8(1):43-51. Off topic
Ref ID: 5464

(3194) Martínez-Aguilar MadlL, Flores-Peña Y, Rizo-Baeza MadlM, Aguilar-Hernández RM, Vázquez-Galindo L, Gutiérres-Sánchez G. 7th to 9th grade obese adolescents' perceptions about obesity in Tamaulipas, Mexico. Percepções da obesidade de adolescentes obesos, estudantes do 7º ao 9º grau residentes em Tamaulipas, México. Percepciones de la obesidad de adolescentes obesos estudiantes del 7º al 9º grado residentes en Tamaulipas, México. Revista Latino-Americana de Enfermagem 2010 February;18(1):48-53. Not a randomized controlled trial (RCT)
Ref ID: 4310

(3195) Martínez-López E, Grajales IC. Efectividad de un programa de promoción de la salud en la disminución de costos médicos asistenciales. A health promotion programme's effectiveness in reducing medical care costs. Revista de Salud Pública 2010 December;12(6):938-49. Off topic
Ref ID: 4311

(3196) Martínez Sotolongo B, Martínez Brito I. Comportamiento de la caries dental en escolares obesos y normopesos de 8 a 13 años. Behavior of dental caries in 8 to 13 years-old obese and normal-weight students. Revista Médica Electrónica 2010 June;32(3). Off topic
Ref ID: 4312

(3197) Martínez CA, Ibáñez JO, Paterno CA, Roig Bustamante MSd, Itatí Heitz M, Kriskovich Juré JO, Bonis GR, Cáceres LC. Sobrepeso y obesidad en niños y adolescentes de la ciudad de Corrrientes. Asociación con factores de riesgo cardiovascular. Medicina (B Aires) 2001;61(3):308-14. Cross-sectional study
Ref ID: 764

(3198) Martínez E, Saldarriaga JF, Sepúlveda FÉ. Actividad física en Medellín: Desafío para la promoción de la salud. Revista Facultad Nacional de Salud Pública 2008 December;26(2):118-22. Survey or questionnaire
Ref ID: 4313

(3199) Martínez JG, Fonseca LMM, Scochi CGS. The participation of parents in the care of premature children in a neonatal unit: Meanings attributed by the health team. Revista Latino-Americana de Enfermagem 2007 April;15(2):239-46. Not a randomized controlled trial (RCT)
Ref ID: 4314

(3200) Martínez V, V, Salcedo AF, Franquelo GR, Solera MM, Sánchez LM, Serrano MS, López GE, Rodríguez AF. Assessment of an after-school physical activity program to prevent obesity among 9- to 10-year-old children: a cluster randomized trial. International Journal of Obesity 2008;32:12-22. Not All Participants were Overweight and/or Obese
Ref ID: 4903

(3201) Martyn JA, Goudsouzian NG, Chang Y, Szyfelbein SK, Schwartz AE, Patel SS. Neuromuscular effects of mivacurium in 2- to 12-yr-old children with burn injury. Anesthesiology 2000;92:31-7. Off topic
Ref ID: 4904

(3202) Maruf AA, Hossain MD, Ahmed M, Samsad IA. Procedural sedation in children for magnetic resonance imaging--comparison between ketamine diazepam combination with midazolam fentanyl combination. Mymensingh Medical Journal 2010;19:60-5. Off topic
Ref ID: 4905

(3203) Marx JO, Gordon SE, Vos NH, Nindl BC, Gomez AL, Volek JS, Pedro J, Ratamess N, Newton RU, French DN, Rubin MR, Hakkinen K, Kraemer WJ. Effect of alkalosis on plasma epinephrine responses to high intensity cycle exercise in humans. European Journal of Applied Physiology 2002 May;87(1):72-7. Diet Intervention or Supplement Study
Ref ID: 1828

(3204) Maryuali A, Uzcátegui LR, Paoli M, Petrosino P, Milano M, Contreras A. Tumor de células de Leydig, presentación con pubertad precoz. Revista Venezolana de Endocrinología y Metabolismo 2007 January;5(1):26-9. Off topic
Ref ID: 4315

(3205) Mascarenhas LPG, Salgueirosa FdM, Nunes GF, Martins PA, Stabelini Neto A, Campos Wd. Relação entre diferentes índices de atividade física e preditores de adiposidade em adolescentes de ambos os sexos. Revista Brasileira de Medicina do Esporte 2005 August;11(4):214-8. Cross-sectional study
Ref ID: 4316

(3206) Mascarenhas LPG, Stabelini Neto A, Bozza R, Campos Wd. Comportamento do consumo máximo de oxigênio e da composição corporal durante o processo maturacional em adolescentes do sexo masculino participantes de treinamento de futebol. Revista Brasileira de Ciência e Movimento 2006;14(2):49-56. Cross-sectional study
Ref ID: 4317

(3207) Mascarenhas LPG, Stabelini Neto A, Vasconcelos IQAd, Smolarek AdC, Bozza R, Ulbrich AZ, Campos Wd. Efeitos de duas intensidades de treinamento aeróbio na composição corporal e na potência aeróbia e anaeróbia de meninos pré-púberes. Revista Brasileira de Educação Física e Esporte 2008 March;22(1):81-9. Not a randomized controlled trial (RCT)
Ref ID: 4318

(3208) Mastalir ET, Kalil RAK, Horowitz ESK, Wender O, Sant'anna JR, Prates PR, Nesralla IA. Desfechos clínicos tardios da cirurgia de Fontan em pacientes com atresia tricúspide. Late clinical outcomes of the Fontan operation in patients with tricuspid atresia. Arquivos Brasileiros de Cardiologia 2002 July;79(1):51-

60. Off topic
Ref ID: 4319

(3209) Mastaloudis A, Traber MG, Carstensen K, Widrick JJ. Antioxidants did not prevent muscle damage in response to an ultramarathon run. Medicine and Science in Sports and Exercise 2006 January;38(1):72-80. Diet Intervention or Supplement Study
Ref ID: 1430

(3210) Masters AM, McGreevy PD. Dogkeeping practices as reported by readers of an Australian dog enthusiast magazine. Australian Veterinary Journal 2008;86(1-2):18-25. Off topic
Ref ID: 5465

(3211) Mathai S, Fernandez A, Mondkar J, Kanbur W. Effects of tactile-kinesthetic stimulation in preterms: a controlled trial. Indian Pediatrics 2001;38:1091-8. Subjects less than 2 years old
Ref ID: 4906

(3212) Mathieu F, Begaux F, Lan ZY, Suetens C, Hinsenkamp M. Clinical manifestations of Kashin-Beck disease in Nyemo Valley, Tibet. International ORTHOPAEDICS 1997;21:151-6. Off topic
Ref ID: 4907

(3213) Matias TS, Rolim MKSB, Kretzer FL, Schmoelz CP, Andrade A. Satisfação corporal associada a prática de atividade física na adolescência. Motriz Revista de Educação Física (Improv) 2010 June;16(2):370-8. Survey or questionnaire
Ref ID: 4320

(3214) Matsuno VM, Camargo MR, Palma GC, Alveno D, Barela AM. Análise do uso de suporte parcial de peso corporal em esteira e em piso fixo durante o andar de crianças com paralisia cerebral. Analysis of partial body weight support during treadmill and overground walking of children with cerebral palsy. Revista Brasileira de Fisioterapia 2010 October;14(5):404-10. Off topic
Ref ID: 4321

(3215) Matsuo H, Katayama K, Ishida K, Muramatsu T, Miyamura M. Effect of menstrual cycle and gender on ventilatory and heart rate responses at the onset of exercise. European Journal of Applied Physiology 2003 September;90(1-2):100-8. Off topic
Ref ID: 1714

(3216) Matsuo T, Saitoh S, Suzuki M. Effects of the menstrual cycle on excess postexercise oxygen consumption in healthy young women. Metabolism: Clinical and Experimental 1999 March;48(3):275-7. Off topic
Ref ID: 2044

(3217) Mattai AK, Hill JL, Lenroot RK. Treatment of early-onset schizophrenia. Curr Opin Psychiatry 2010;23(4):304-10. Review article
Ref ID: 3272

(3218) Mattern-Baxter K. Effects of Partial Body Weight Supported Treadmill Training on Children with Cerebral Palsy. Pediatric Physical Therapy 2009;21(1):12-22. Review article
Ref ID: 5466

(3219) Mattes RD, Maone T, Wager-Page S, Beauchamp G, Bernbaum J, Stallings V, Pereira GR, Gibson E, Russell P, Bhutani V. Effects of sweet taste stimulation on growth and sucking in preterm infants. Journal of Obstetric, Gynecologic, and Neonatal Nursing 25(5):407-14, 1996 Jun 1996;(5):407-14. Off topic
Ref ID: 3057

(3220) Matteucci MC, Calzolari A, Pompei E, Principato F, Turchetta A, Rizzoni G. Abnormal hypertensive response during exercise test in normotensive transplanted children and adolescents. Nephron 1996;73(2):201-6. Off topic
Ref ID: 5467

(3221) Matthews RD, Neumayer L. Inguinal Hernia in the 21st Century: An Evidence-Based Review. Current Problems in Surgery 2008;45(4):261-312. Review article
Ref ID: 3273

(3222) Mattiello R, Sarria EE, Stein R, Fischer GB, Mocelin HT, Barreto SSM, Lima JAB, Brandenburg D. Avaliação funcional durante o exercício em crianças e adolescentes com bronquiolite obliterante pós-infecciosa. Functional capacity assessment during exercise in children and adolescents with post-infectious bronchiolitis obliterans. Jornal de Pediatria 2008 August;84(4):337-43. Off topic
Ref ID: 4322

(3223) Mattila VM, Sillanpaa PJ, Salo T, Laine HJ, Maenpaa H, Pihlajamaki H. Can orthotic insoles prevent lower limb overuse injuries? A randomized-controlled trial of 228 subjects. Scandinavian Journal of Medicine and Science in Sports 2011 December;21(6):804-8. Off topic
Ref ID: 2678

(3224) Mattocks C, Ness A, Deere K, Tilling K, Leary S, Blair SN, Riddoch C. Early life determinants of physical activity in 11 to 12 year olds: cohort study. British Medical Journal 2008 January 5;336(7634):26-9. Cohort Study
Ref ID: 1013

(3225) Maturi MS, Afshary P, Abedi P. Effect of physical activity intervention based on a pedometer on physical activity level and anthropometric measures after childbirth: a randomized controlled trial. BMC Pregnancy and Childbirth 2011;11:103. Study limited to adults
Ref ID: 2679

(3226) Matusitz J, McCormick J. Sedentarism: The Effects of Internet Use on Human Obesity in the United States. Social Work in Public Health 2012;27(3):250-69. Not an exercise intervention study
Ref ID: 5468

(3227) Matvienko O, Ahrabi-Fard I. The effects of a 4-week after-school program on motor skills and fitness of kindergarten and first-grade students. American Journal of Health Promotion 2010 May;24(5):299-303. Not a randomized controlled trial (RCT)
Ref ID: 533

(3228) Matyka KA, Malik S. Management of the obese child - Application of NICE guidelines 2006. British Journal of Diabetes and Vascular Disease 2008;8(4):178-82. Review article
Ref ID: 3274

(3229) Maugans TA, Farley C, Altaye M, Leach J, Cecil KM. Pediatric Sports-Related Concussion Produces Cerebral Blood Flow Alterations. Pediatrics 2012;129(1):28-37. Off topic
Ref ID: 5469

(3230) Mauras N, Pescovitz OH, Allada V, Messig M, Wajnrajch MP, Lippe B, Transition Study Group. Limited efficacy of growth hormone (GH) during transition of GH-deficient patients from adolescence to adulthood: a phase III multicenter, double-blind, randomized two-year trial. Journal of Clinical Endocrinology and Metabolism 2005 July;90(7):3946-55. Drug intervention study
Ref ID: 1498

(3231) Mauras N, Beck RW, Kollman C, Chase HP, Tsalikian E, Fox LA, Weinzimer SA, Xing DY, Ruedy KJ, Steffes MW, Borland TM, Singh R, Tamborlane WV. Impaired overnight counterregulatory hormone responses to spontaneous hypoglycemia in children with type 1 diabetes. Pediatric Diabetes 2007;8(4):199-205. Acute study
Ref ID: 5470

(3232) Mauras N, Delgiorno C, Hossain J, Bird K, Killen K, Merinbaum D, Weltman A, Damaso L, Balagopal P. Metformin use in children with obesity and normal glucose tolerance--effects on cardiovascular markers and intrahepatic fat. Journal of Pediatric Endocrinology 2012;25(1-2):33-40. Drug intervention study
Ref ID: 2680

(3233) Mauriello LM, Ciavatta MM, Paiva AL, Sherman KJ, Castle PH, Johnson JL, Prochaska JM. Results of a multi-media multiple behavior obesity prevention program for adolescents. Preventive Medicine 2010 December;51(6):451-6. Lifestyle Intervention
Ref ID: 1103

(3234) May PA, Gossage JP, Brooke LE, Marais AS, Hendricks LS, Snell CL, Croxford JA, Viljoen DL. Maternal Risk Factors for Fetal Alcohol Syndrome in the Western Cape Province of South Africa: A Population-Based Study. American Journal of Public Health 2005 July;95(7):1190-9. Case-Control / Case Study
Ref ID: 3839

(3235) Mayo-Wilson E. Reporting Implementation in Randomized Trials: Proposed Additions to the Consolidated Standards of Reporting Trials Statement. American Journal of Public Health 2007 April;97(4):630-3. Review article
Ref ID: 3840

(3236) Mazaro IAR, Zanolli MdL, Antonio MÂ, Morcillo AM, Zambon MP. Obesidade e fatores de risco cardiovascular em estudantes de Sorocaba, SP. Obesity and cardiovascular risk factors in school children from Sorocaba, SP. Revista da Associação Médica Brasileira (1992) 2011 December;57(6):674-80. Prevalence study
Ref ID: 4323

(3237) Mazza C, Evangelista P, Figueroa A, Kovalskys I, Digón P, López S, Scaiola E, Perez N, Dieuzeide G. Estudio clínico del síndrome metabólico en niños y adolescentes de Argentina. Clinical study of metabolic syndrome in children and adolescents of Argentina. Revista Argentina de Salud Pública 2011 March;2(6):25-33. Cross-sectional study
Ref ID: 4324

(3238) Mazzeo SE, Kelly NR, Stern M, Gow RW, Cotter EW, Thornton LM, Evans RK, Bulik CM. Parent skills training to enhance weight loss in overweight children: evaluation of NOURISH. Eating Behaviors 2014 April;15(2):225-9. Inappropriate Intervention
Ref ID: 5972

(3239) Mazzetti S, Douglass M, Yocum A, Harber M. Effect of explosive versus slow contractions and exercise intensity on energy expenditure. Medicine and Science in Sports and Exercise 2007 August;39(8):1291-301. Study limited to adults
Ref ID: 1171

(3240) Mazzetti SA, Kraemer WJ, Volek JS, Duncan ND, Ratamess NA, Gomez AL, Newton RU, Hakkinen K, Fleck SJ. The influence of direct supervision of resistance training on strength performance. Medicine and Science in Sports and Exercise 2000 June;32(6):1175-84. Study limited to adults
Ref ID: 1974

(3241) Márquez Arabia JJ, Suárez GR, Márquez Tróchez C. Intervenciones en el estilo de vida basadas en ejercicio en niños y adolescentes con síndrome metabólico. Revista de la Facultad de Medicina (Caracas) 2010;33(2):96-104.

Review article
Ref ID: 4325

(3242) Moller J, Jorgensen JOL, Marqversen JÃ, Frandsen E, Christiansen JS. Insulin-like growth factor I administration induces fluid and sodium retention in healthy adults: possible involvement of renin and atrial natriuretic factor. Clinical Endocrinology 2000 February;52(2):181-6. Drug intervention study
Ref ID: 523

(3243) Moble T, Kleimann M, Rehbein F, Pfeiffer C. Media use and school achievement--boys at risk? British Journal of Developmental Psychology 2010 September;28(3):699-725. Cross-sectional study
Ref ID: 3685

(3244) McAllister TW, Kim E. Agitation, aggression, and disinhibition syndromes after traumatic brain injury. NeuroRehabilitation 2002 December;17(4):297. Off topic
Ref ID: 3686

(3245) McAnulty SR, Hosick PA, McAnulty LS, Quindry JC, Still L, Hudson MB, Dibarnardi AN, Milne GL, Morrow JD, Austin MD. Effect of pharmacological lowering of plasma urate on exercise-induced oxidative stress. Applied Physiology, Nutrition, and Metabolism = Physiologie Appliquee, Nutrition et Metabolisme 2007 December;32(6):1148-55. Drug intervention study
Ref ID: 1131

(3246) McArthur LH, Holbert D, Pena M. Development and application of rapid assessment diet and physical activity indexes, which suggest high consumption of energy-dense foods and inadequate exercise among adolescents from 6 Latin American cities: a pilot study. Nutrition Research 2008;28(9):590-9. Off topic
Ref ID: 5471

(3247) McBeth JM, Earl-Boehm JE, Cobb SC, Huddleston WE. Hip Muscle Activity During 3 Side-Lying Hip-Strengthening Exercises in Distance Runners. Journal of Athletic Training 2012 January;47(1):15-23. Off topic
Ref ID: 3841

(3248) McBride JM, Triplett-McBride T, Davie AJ, Abernethy PJ, Newton RU. Characteristics of titin in strength and power athletes. European Journal of Applied Physiology 2003 February;88(6):553-7. Off topic
Ref ID: 1775

(3249) McCabe MP, Ricciardelli LA, Stanford J, Holt K, Keegan S, Miller L. Where is all the pressure coming from? Messages from mothers and teachers about preschool children's appearance, diet and exercise. European Eating Disorders Review 2007 May;15(3):221-30. Not a randomized controlled trial (RCT)
Ref ID: 1190

(3250) McCallum Z, Wake M, Gerner B, Harris C, Gibbons K, Gunn J, Waters E, Baur LA. Can Australian general practitioners tackle childhood overweight/obesity? Methods and processes from the LEAP (Live, Eat and Play) randomized controlled trial. Journal of Paediatrics and Child Health 2005 September;41(9-10):488-94. No exercise only group
Ref ID: 300

(3251) McCallum Z, Wake M, Gerner B, Baur LA, Gibbons K, Gold L, Gunn J, Harris C, Naughton G, Riess C, Sanci L, Sheehan J, Ukoumunne OC, Waters E. Outcome data from the LEAP (Live, Eat and Play) trial: a randomized controlled trial of a primary care intervention for childhood overweight/mild obesity. International Journal of Obesity (London) 2007 April;31(4):630-6. No exercise only group
Ref ID: 257

(3252) McCann DJ, Adams WC. A dimensional paradigm for identifying the size-independent cost of walking. Medicine and Science in Sports and Exercise 2002 June;34(6):1009-17. Off topic
Ref ID: 1820

(3253) McCarthy PL, Sznajderman SD, Lustman FK, Baron MA, Fink HD, Czarkowski N, Bauchner H, Forsyth BC, Cicchetti DV. Mothers' clinical judgment: a randomized trial of the Acute Illness Observation Scales. The Journal of Pediatrics 1990;116:200-6. Off topic
Ref ID: 4908

(3254) McCarton CM, Brooks GJ, Wallace IF, Bauer CR, Bennett FC, Bernbaum JC, Broyles RS, Casey PH, McCormick MC, Scott DT, Tyson J, Tonascia J, Meinert CL. Results at age 8 years of early intervention for low-birth-weight premature infants. The Infant Health and Development Program. Journal of the American Medical Association 1997;277:126-32. Subjects less than 2 years old
Ref ID: 950

(3255) McCormick DP, Ramirez M, Caldwell S, Ripley AW, Wilkey D. YMCA program for childhood obesity: a case series. Clinical Pediatrics (Philadelphia) 2008 September;47(7):693-7. Not a randomized controlled trial (RCT), Lifestyle Intervention
Ref ID: 457

(3256) McCormick MC, Brooks GJ, Buka SL, Goldman J, Yu J, Salganik M, Scott DT, Bennett FC, Kay LL, Bernbaum JC, Bauer CR, Martin C, Woods ER, Martin A, Casey PH. Early intervention in low birth weight premature infants: Results at 18 years of age for the infant health and development program. Pediatrics 2006;117:771-80. Prospective Study
Ref ID: 4909

(3257) McCormick RK. Osteoporosis: Integrating Biomarkers and Other Diagnostic Correlates into the Management of Bone Fragility. Alternative Medicine Review 2007 June;12(2):113-45. Review article
Ref ID: 3687

(3258) McCrindle BW, Urbina EM, Dennison BA, Jacobson MS, Steinberger J, Rocchini AP, Hayman LL, Daniels SR, American Heart Association Atherosclerosis HaOiYC, American Heart Association Council of Cardiovascular Disease in the Young, American Heart Association Council on Cardiovascular Nursing. Drug therapy of high-risk lipid abnormalities in children and adolescents: A scientific statement from the American Heart Association Atherosclerosis, Hypertension, and Obesity in Youth Committee, Council of Cardiovascular Disease in the Young, with the Council on Cardiovascular Nursing. Circulation 2007 April 10;115(14):1948-67. Review article
Ref ID: 1251

(3259) McCrindle BW, Manlhiot C. Elevated atherogenic lipoproteins in childhood: Risk, prevention, and treatment. Journal of Clinical Lipidology 2008;2(3):138-46. Review article
Ref ID: 3275

(3260) McCrory C, Layte R. Breastfeeding and risk of overweight and obesity at nine-years of age. Social Science and Medicine 2012;75(2):323-30. Retrospective study
Ref ID: 5472

(3261) McCrory MA, Nommsen-Rivers LA, Mole PA, Lonnerdal B, Dewey KG. Randomized trial of the short-term effects of dieting compared with dieting plus aerobic exercise on lactation performance. American Journal of Clinical Nutrition 69(5):959-67, 1999 May 1999;(5):959-67. Study limited to adults
Ref ID: 3062

(3262) McCrory MA. Does dieting during lactation put infant growth at risk?. [Review] [26 refs]. Nutrition Reviews 2001 January;59(1:Pt 1):t-21. Review article
Ref ID: 1924

(3263) McCrory MA. Does dieting during lactation put infant growth at risk? Nutrition Reviews 2001 January;59(1 Pt 1):18-21. Study not limited to children and adolescents, No exercise only group
Ref ID: 398

(3264) Mcdade TW, Reyes-Garcia V, Tanner S, Huanca T, Leonard WR. Maintenance versus growth: Investigating the costs of immune activation among children in lowland Bolivia. American Journal of Physical Anthropology 2008;136(4):478-84. Off topic
Ref ID: 5473

(3265) Mcdade TW, Chyu L, Duncan GJ, Hoyt LT, Doane LD, Adam EK. Adolescents' expectations for the future predict health behaviors in early adulthood. Social Science and Medicine 2011;73(3):391-8. Survey or questionnaire
Ref ID: 5474

(3266) McDermott AY, Kaplan L, Flier JS, Drucker DJ, Malhotra A, Berkowitz RI, Jakicic J, Wadden T, Herron DM, Shikora S, Schwartz M, Kushner RF. Conference coverage: Selected presentations from the 20th Annual Practical Approaches to the Treatment of Obesity; June 22-24, 2006. MedGenMed: Medscape General Medicine 2006;8(4). Abstract
Ref ID: 3276

(3267) McDonagh MS, Selph S, Ozpinar A, Foley C. Systematic review of the benefits and risks of metformin in treating obesity in children aged 18 years and younger. JAMA Pediatrics 2014 February;168(2):178-84. Inappropriate Study Design
Ref ID: 5973

(3268) McDuffie JR, Calis KA, Uwaifo GI, Sebring NG, Fallon EM, Hubbard VS, Yanovski JA. Three-month tolerability of orlistat in adolescents with obesity-related comorbid conditions. Obesity Research 2002 July;10(7):642-50. Drug intervention study
Ref ID: 1815

(3269) McDuffie JR, Yanovski JA. Treatment of childhood and adolescent obesity. Endocrinologist 2004;14(3):138-43. Review article
Ref ID: 3277

(3270) McFarlin BK, Johnston CJ, Carpenter KC, Davidson T, Moreno JL, Strohacker K, Breslin WL, Foreyt JP. A one-year school-based diet/exercise intervention improves non-traditional disease biomarkers in Mexican-American children. Maternal and Child Nutrition 2013 October;9(4):524-32. Inappropriate Intervention
Ref ID: 5974

(3271) McGaffey A, Hughes K, Fidler SK, D'Amico FJ, Stalter MN. Can Elvis Pretzley and the Fitwits improve knowledge of obesity, nutrition, exercise, and portions in fifth graders? International Journal of Obesity 2010 July;34(7):1134-42. Educational intervention
Ref ID: 492

(3272) McGovern L, Johnson JN, Paulo R, Hettinger A, Singhal V, Kamath C, Erwin PJ, Montori VM. Treatment of pediatric obesity: A systematic review and meta-analysis of randomized trials. Journal of Clinical Endocrinology and Metabolism 2008;93(12):4600-5. Review article
Ref ID: 3278

(3273) McGovern L, Johnson JN, Paulo R, Hettinger A, Singhal V, Kamath C, Erwin PJ, Montori VM. Clinical review: treatment of pediatric obesity: a systematic review and meta-analysis of randomized trials. [Review] [23 refs]. Journal of Clinical Endocrinology and Metabolism 2008 December;93(12):4600-5. Review article
Ref ID: 845

(3274) McGowan L, Cooke LJ, Gardner B, Beeken RJ, Croker H, Wardle J. Healthy feeding habits: efficacy results from a cluster-randomized, controlled exploratory trial of a novel, habit-based intervention with parents. American Journal of Clinical Nutrition 2013 September;98(3):769-77. Inappropriate Outcomes
Ref ID: 5975

(3275) Mcgraw JE, Waller DP. Fish ingestion and congener specific polychlorinated biphenyl and p,p '-dichlorodiphenyldichloroethylene serum concentrations in a great lakes cohort of pregnant African American women. Environment International 2009;35(3):557-65. Diet Intervention Study
Ref ID: 5475

(3276) McGuigan MR, Tatasciore M, Newton RU, Pettigrew S. Eight weeks of resistance training can significantly alter body composition in children who are overweight or obese. Journal of Strength and Conditioning Research 2009 January;23(1):80-5. No control group (NC)
Ref ID: 458

(3277) McGuine TA, Sullivan JC, Bernhardt DT. Creatine supplementation in high school football players. Clinical Journal of Sport Medicine 2001 October;11(4):247-53. Diet Intervention or Supplement Study
Ref ID: 1866

(3278) McGuine TA, Hetzel S, Wilson J, Brooks A. The effect of lace-up ankle braces on injury rates in high school football players. American Journal of Sports Medicine 2012 January;40(1):49-57. Off topic
Ref ID: 2682

(3279) McGuire DK, Abdullah SM, See R, Snell PG, McGavock J, Szczepaniak LS, Ayers CR, Drazner MH, Khera A, De Lemos JA. Randomized comparison of the effects of rosiglitazone vs. placebo on peak integrated cardiovascular performance, cardiac structure, and function. European Heart Journal 2010;31(18):2262-70. Drug intervention study
Ref ID: 3279

(3280) McGuire J. What is problem solving? A review of theory, research and applications. Criminal Behaviour and Mental Health 2001 December;11(4):210. Review article
Ref ID: 3688

(3281) Mchutchison JG. Hepatitis C advances in antiviral therapy: What is accepted treatment now? Journal of Gastroenterology and Hepatology 2002 April;17(4):431-41. Off topic
Ref ID: 3689

(3282) McKay H, Tsang G, Heinonen A, MacKelvie K, Sanderson D, Khan KM. Ground reaction forces associated with an effective elementary school based jumping intervention. British Journal of Sports Medicine 2005 January;39(1):10-4. Off topic
Ref ID: 1554

(3283) McKay HA, Petit MA, Schutz RW, Prior JC, Barr SI, Khan KM. Augmented trochanteric bone mineral density after modified physical education classes: a randomized school-based exercise intervention study in prepubescent and early pubescent children. Journal of Pediatrics 2000 February;136(2):156-62. Not All Participants were Overweight and/or Obese
Ref ID: 1989

(3284) McKay HA, MacLean L, Petit M, MacKelvie OK, Janssen P, Beck T, Khan KM. 'Bounce at the Bell': a novel program of short bouts of exercise improves proximal femur bone mass in early pubertal children. British Journal of Sports Medicine 2005;39:521-6. CT
Ref ID: 4910

(3285) McKay WP, Chilibeck PD, Daku BL, Lett B. Quantifying the mechanical work of resting quadriceps muscle tone. European Journal of Applied Physiology 2010 March;108(4):641-8. Off topic
Ref ID: 587

(3286) McManus AM, Armstrong N, Williams CA. Effect of training on the aerobic power and anaerobic performance of prepubertal girls. Acta Paediatrica 1997 May;86(5):456-9. Not All Participants were Overweight and/or Obese
Ref ID: 2138

(3287) McMeekin S, Jansen E, Mallan K, Nicholson J, Magarey A, Daniels L. Associations between infant temperament and early feeding practices. A cross-sectional study of Australian mother-infant dyads from the NOURISH randomised controlled trial. Appetite 2013 January;60(1):239-45. Inappropriate Study Design
Ref ID: 5976

(3288) McMillan AG, Auman NL, Collier DN, Williams DSB. Frontal Plane Lower Extremity Biomechanics During Walking in Boys Who Are Overweight Versus Healthy Weight. Pediatric Physical Therapy 2009;21(2):187-93. Not All Participants were Overweight and/or Obese
Ref ID: 5476

(3289) McMurray RG, Harrell JS, Bangdiwala SI, Bradley CB, Deng S, Levine A. A school-based intervention can reduce body fat and blood pressure in young adolescents. Journal of Adolescent Health 2002 August;31(2):125-32. Not All Participants were Overweight and/or Obese
Ref ID: 1807

(3290) McMurray RG, Harrell JS, Creighton D, Wang Z, Bangdiwala SI. Influence of physical activity on change in weight status as children become adolescents. International Journal of Pediatric Obesity 2008;3(2):69-77. Longitudinal Study
Ref ID: 959

(3291) McMurray RG, Ward DS, Elder JP, Lytle LA, Strikmiller PK, Baggett CD, Young DR. Do overweight girls overreport physical activity? American Journal of Health Behavior 2008 September;32(5):538-46. Not an exercise intervention study
Ref ID: 1003

(3292) McMurray RG, Bassin S, Jago R, Bruecker S, Moe EL, Murray T, Mazzuto SL, Volpe SL. Rationale, design and methods of the HEALTHY study physical education intervention component. International Journal of Obesity (London) 2009 August;33 Suppl 4:S37-43.:S37-S43. No exercise only group
Ref ID: 108

(3293) McMurray RG, Ondrak KS. Effects of being overweight on ventilatory dynamics of youth at rest and during exercise. European Journal of Applied Physiology 2011 February;111(2):285-92. Not All Participants were Overweight and/or Obese
Ref ID: 2683

(3294) McNaughton L, Cedaro R. Sodium citrate ingestion and its effects on maximal anaerobic exercise of different durations. European Journal of Applied Physiology and Occupational Physiology 1992;64(1):36-41. Diet Intervention or Supplement Study
Ref ID: 2293

(3295) McNaughton LR, Lovell RJ, Siegler J, Midgley AW, Moore L, Bentley DJ. The effects of caffeine ingestion on time trial cycling performance. International Journal of Sports Physiology and Performance 2008 June;3(2):157-63. Diet Intervention or Supplement Study
Ref ID: 824

(3296) McNeil DA, Wilson BN, Siever JE, Ronca M, Mah JK. Connecting children to recreational activities: results of a cluster randomized trial. American Journal of Health Promotion 2009 July;23(6):376-87. Not All Participants were Overweight and/or Obese
Ref ID: 718

(3297) McNevin NH, Coraci L, Schafer J. Gait in adolescent cerebral palsy: the effect of partial unweighting. Archives of Physical Medicine and Rehabilitation 2000 April;81(4):525-8. Case-Control / Case Study
Ref ID: 1983

(3298) McVeigh JA, Norris SA, Cameron N, Pettifor JM. Associations between physical activity and bone mass in black and white South African children at age 9 yr. Journal of Applied Physiology 2004;97:1006-12. Cross-sectional study
Ref ID: 4911

(3299) McVey G, Gusella J, Tweed S, Ferrari M. A controlled evaluation of web-based training for teachers and public health practitioners on the prevention of eating disorders. Eating Disorders 2009;17:1-26. Off topic
Ref ID: 4912

(3300) Medeiros CCM, Cardoso MAA, Pereira RAR, Alves GTdA, França ISXd, Coura AS, Carvalho DFd. Estado nutricional e hábitos de vida em escolares. Nutritional status and habits of life in school children. Revista Brasileira de Crescimento e Desenvolvimento Humano 2011;21(3):789-97. Prevalence study
Ref ID: 4326

(3301) Mediano MF, Barbosa JS, Sichieri R, Pereira RA. [Effects of exercise on insulin sensitivity in obese women submitted to a weight loss program: a clinical trial]. [Portuguese]. Arquivos Brasileiros de Endocrinologia e Metabologia 2007 August;51(6):993-9. Study limited to adults
Ref ID: 1153

(3302) Mediano MFF, Barbosa JSdO, Sichieri R, Pereira RA. Efeito do exercício físico na sensibilidade à insulina em mulheres obesas submetidas a programa de perda de peso: Um ensaio clínico. Arquivos Brasileiros de Endocrinologia and Metabologia 2007 August;51(6):993-9. Study limited to adults
Ref ID: 4327

(3303) Mehrotra D, Pradhan R, Mohammad S, Jaiswara C. Random control trial of dermis-fat graft and interposition of temporalis fascia in the management of temporomandibular ankylosis in children. British Journal of Oral and Maxillofacial Surgery 2008 October;46(7):521-6. Off topic
Ref ID: 890

(3304) Mehta SR, Kashyap AS, Das S. Diabetes mellitus in India: The modern scourge. Medical Journal Armed Forces India 2009;65(1):50-4. Review article
Ref ID: 3280

(3305) Mei Z, Serdula MK, Liu JM, Flores-Ayala RC, Wang L, Ye R, Grummer-Strawn LM. Iron-containing micronutrient supplementation of Chinese women with no or mild anemia during pregnancy improved iron status but did not affect perinatal anemia. Journal of Nutrition 2014 June;144(6):943-8. Inappropriate

Population
Ref ID: 5977

(3306) Mekhmoukh A, Chapelot D, Bellisle F. Influence of environmental factors on meal intake in overweight and normal-weight male adolescents. A laboratory study. Appetite 2012;59(1):90-5. Diet Intervention Study
Ref ID: 5477

(3307) Melby CL, Osterberg KL, Resch A, Davy B, Johnson S, Davy K. Effect of carbohydrate ingestion during exercise on post-exercise substrate oxidation and energy intake. International Journal of Sport Nutrition and Exercise Metabolism 2002 September;12(3):294-309. Diet Intervention Study
Ref ID: 1793

(3308) Meléndez I, Olivares C, Lera M, Mediano S F. Etapas Del Cambio, Motivaciones Y Barreras Relacionadas Con el Consumo de Frutas Y Verduras Y la Actividad Física en Madres De Preescolares Atendidas En Centros De Atención Primaria De Salud. Stages of change, motivations and barriers related to the consumption of fruit and vegetables and physical activity in mothers of preschool children attending primary health center. Revista Chilena de Nutrición 2011 December;38(4):466-75. Off topic
Ref ID: 4328

(3309) Mellecker RR, McManus AM. Measurement of resting energy expenditure in healthy children. Journal of Parenteral and Enteral Nutrition 2009;33:640-5. Off topic
Ref ID: 4913

(3310) Mellin LM, Slinkard LA, Irwin CE, Jr. Adolescent obesity intervention: validation of the SHAPEDOWN program. Journal of the American Dietetic Association 1987 March;87(3):333-8. Lifestyle Intervention
Ref ID: 2343

(3311) Mello AD, Marcon SS, Hulsmeyer AP, Cattai GB, Ayres CS, Santana RG. Prevalência de sobrepeso e obesidade em crianças de seis a dez anos de escolas municipais de área urbana. Prevalence of overweight and obesity in six to ten year-old students from urban county schools. Revista Paulista de Pediatria 2010 March;28(1):48-54. Cross-sectional study
Ref ID: 4329

(3312) Mello ED, Luft VC, Meyer F. Atendimento ambulatorial individualizado versus programa de educação em grupo: qual oferece mais mudança de hábitos alimentares e de atividade física em crianças obesas? Jornal de Pediatria 2004 December;80(6):468-74. No non-intervention control group
Ref ID: 4330

(3313) Melnyk BM, Small L, Morrison-Beedy D, Strasser A, Spath L, Kreipe R, Crean H, Jacobson D, Kelly S, O'Haver J. The COPE Healthy Lifestyles TEEN

program: feasibility, preliminary efficacy, & lessons learned from an after school group intervention with overweight adolescents. Journal of Pediatric Health Care 2007 September;21(5):315-22. Behavior Modification Intervention
Ref ID: 1168

(3314) Melnyk BM, Jacobson D, Kelly S, O'Haver J, Small L, Mays MZ. Improving the mental health, healthy lifestyle choices, and physical health of Hispanic adolescents: a randomized controlled pilot study. Journal of School Health 2009 December;79(12):575-84. No exercise only group
Ref ID: 83

(3315) Melnyk BM, Jacobson D, Kelly S, Belyea M, Shaibi G, Small L, O'Haver J, Marsiglia FF. Promoting healthy lifestyles in high school adolescents: a randomized controlled trial. American Journal of Preventive Medicine 2013 October;45(4):407-15. Inappropriate Intervention
Ref ID: 5978

(3316) Melo VLC, Serra PJ, Cunha CdF. Obesidade infantil - Impactos psicossociais. Childhood obesity – Psychosocial impacts. Revista Médica de Minas Gerais 2010 September;20(3). Review article
Ref ID: 4331

(3317) Meltzer LJ, Moore M. Sleep disruptions in parents of children and adolescents with chronic illnesses: Prevalence, causes, and consequences. Journal of Pediatric Psychology 2008 April;33(3):279-91. Review article
Ref ID: 3842

(3318) Melvin AJ, Mohan KM, Arcuino LAM, Edelstein RE, Frenkel LM. Clinical, virologic and immunologic responses of children with advanced human immunodeficiency virus type 1 disease treated with protease inhibitors. Pediatric Infectious Disease Journal 1997;16(10):968-74. Off topic
Ref ID: 5478

(3319) Mendell JR, Moxley RT, Griggs RC, Brooke MH, Fenichel GM, Miller JP, King W, Signore L, Pandya S, Florence J. Randomized, double-blind six-month trial of prednisone in Duchenne's muscular dystrophy. New England Journal of Medicine 1989 June 15;320(24):1592-7. Drug intervention study
Ref ID: 2322

(3320) Mendes RR, Pires I, Oliveira A, Tirapegui J. Effects of creatine supplementation on the performance and body composition of competitive swimmers. Journal of Nutritional Biochemistry 2004 August;15(8):473-8. Diet Intervention or Supplement Study
Ref ID: 1602

(3321) Mendonça Araujo D, Aparecida Pascucci de Souza Sande L, Fernanda Rodrigues Martinho Fernandes L, Pereira K. Comportamento de lactentes em decúbito lateral e a influência da adição de peso no punho. Infants' behavior in

the side-lying and the influence of and additional load to the wrist. Conscientiae Saúde (Impr) 2011 March;10(1). Subjects less than 2 years old
Ref ID: 4332

(3322) Mendoza JA, Watson K, Baranowski T, Nicklas TA, Uscanga DK, Hanfling MJ. The walking school bus and children's physical activity: a pilot cluster randomized controlled trial. Pediatrics 2011 September;128(3):e537-e544. Not All Participants were Overweight and/or Obese
Ref ID: 1038

(3323) Mendoza JA, Nicklas TA, Liu Y, Stuff J, Baranowski T. General versus central adiposity and relationship to pediatric metabolic risk. Metabolic Syndrome and Related Disorders 2012 April;10(2):128-36. Secondary analysis
Ref ID: 2685

(3324) Mendy FO. Functionality, resilience, maintenance and polyunsaturated fatty acids. Ocl-Oleagineux Corps Gras Lipides 2001;8(4):321-7. Off topic
Ref ID: 5479

(3325) Meng L, Xu H, Liu A, van RJ, Bemelmans W, Hu X, Zhang Q, Du S, Fang H, Ma J, Xu G, Li Y, Guo H, Du L, Ma G. The costs and cost-effectiveness of a school-based comprehensive intervention study on childhood obesity in China. PLoS ONE 2013;8(10):e77971. Inappropriate Population
Ref ID: 5979

(3326) Menghetti E, Di FG, Mucedola G, Montaleone M, Carratelli TJ, Agolini D, Martino F, Marulli P. Follow-up of obese child. Rivista Europea Per Le Scienze Mediche e Farmacologiche 1995 March;17(2-3):77-80. Follow-up Study
Ref ID: 2196

(3327) Mentzel HJ, Wünsche K, Malich A, Böttcher J, Vogt S, Kaiser WA. [The effect of sports activities in children and adolescents on the calcaneus--an investigation with quantitative ultrasound]. RöFo : Fortschritte auf dem Gebiete der Röntgenstrahlen und der Nuklearmedizin 2005;177:524-9. Off topic
Ref ID: 4914

(3328) Mercier J, Vago P, Ramonatxo M, Bauer C, Prefaut C. Effect of aerobic training quantity on the VO2 max of circumpubertal swimmers. International Journal of Sports Medicine 1987 February;8(1):26-30. No non-intervention control group
Ref ID: 2341

(3329) Merino de Méndez G. Manejo de las dislipidemias en niños y adolescentes: [revisión]. Archivos Venezolanos de Puericultura y Pediatría 2007 December;70(4):130-5. Review article
Ref ID: 4333

(3330) Mero AA, Keskinen KL, Malvela MT, Sallinen JM. Combined creatine and sodium bicarbonate supplementation enhances interval swimming. Journal of

Strength and Conditioning Research 2004 May;18(2):306-10. Diet Intervention or Supplement Study
Ref ID: 1628

(3331) Merrilees MJ, Smart EJ, Gilchrist NL, Frampton C, Turner JG, Hooke E, March RL, Maguire P. Effects of diary food supplements on bone mineral density in teenage girls. European Journal of Nutrition 2000 December;39(6):256-62. Diet Intervention or Supplement Study
Ref ID: 1939

(3332) Mesa JL, Ortega FB, Ruiz JR, Castillo MJ, Tresaco B, Carreno F, Moreno LA, Gutierrez A, Bueno M, Avena Study Group. Anthropometric determinants of a clustering of lipid-related metabolic risk factors in overweight and non-overweight adolescents--influence of cardiorespiratory fitness. The Avena study. Annals of Nutrition and Metabolism 2006;50(6):519-27. Cross-sectional study
Ref ID: 1304

(3333) Meshram II, Arlappa N, Balkrishna N, Rao KM, Laxmaiah A, Brahmam GN. Prevalence of hypertension, its correlates and awareness among adult tribal population of Kerala state, India. Journal of Postgraduate Medicine 2012 October;58(4):255-61. Inappropriate Population
Ref ID: 5980

(3334) Messina T, Genco A, Favaro R, Maselli R, Torchia F, Guidi F, Razza R, Aloi N, Piattelli M, Lorenzo M. Intragastric balloon positioning and removal: sedation or general anesthesia? Surgical Endoscopy 2011 December;25(12):3811-4. Off topic
Ref ID: 2686

(3335) Messner SF, Eqsenfeld R. Political Restraint of the Market and Levels of Criminal Homicide: A Cross-National Application of Institutional Anomie Theory. Social Forces 1997 June;75(4):1393-416. Off topic
Ref ID: 3843

(3336) Metallinos-Katsaras ES, Freedson PS, Fulton JE, Sherry B. The association between an objective measure of physical activity and weight status in preschoolers. Obesity 2007 March;15(3):686-94. Cross-sectional study
Ref ID: 1261

(3337) Metcalf B, Henley W, Wilkin T. Effectiveness of intervention on physical activity of children: systematic review and meta-analysis of controlled trials with objectively measured outcomes (EarlyBird 54). British Medical Journal 2012;345:e5888. Inappropriate Study Design
Ref ID: 5981

(3338) Mettler S, Lamprecht-Rusca F, Stoffel-Kurt N, Wenk C, Colombani PC. The influence of the subjects' training state on the glycemic index. European

Journal of Clinical Nutrition 2007 January;61(1):19-24. Off topic
Ref ID: 1294

(3339) Mettler S, Mitchell N, Tipton KD. Increased protein intake reduces lean body mass loss during weight loss in athletes. Medicine and Science in Sports and Exercise 2010 February;42(2):326-37. Diet Intervention or Supplement Study
Ref ID: 605

(3340) Metzler MM, Higgins DL, Beeker CG, Freudenberg N, Lantz PM, Senturia KD, Elsinger AA, Viruell-Fuentes EA, Gheisar B, Palermo AG, Softley D. Addressing Urban Health in Detroit, New York City, and Seattle Through Community-Based Participatory Research Partnerships. American Journal of Public Health 2003 May;93(5):803-11. Off topic
Ref ID: 3844

(3341) Meucci M, Cook C, Curry CD, Guidetti L, Baldari C, Collier SR. Effects of supervised exercise program on metabolic function in overweight adolescents. World Journal of Pediatrics 2013 November;9(4):307-11. Inappropriate Population
Ref ID: 5982

(3342) Meulepas MA, Braspenning JC, de Grauw WJ, Lucas AE, Wijkel D, Grol RP. Patient-oriented intervention in addition to centrally organised checkups improves diabetic patient outcome in primary care. Quality and Safety in Health Care 2008 October;17(5):324-8. Off topic
Ref ID: 880

(3343) Meydani M. Vitamin E. Lancet 1995 January 21;345(8943):170. Review article
Ref ID: 3690

(3344) Meyer EC, Coll CT, Lester BM, Boukydis CF, McDonough SM, Oh W. Family-based intervention improves maternal psychological well-being and feeding interaction of preterm infants. Pediatrics 1994;93:241-6. Subjects less than 2 years old
Ref ID: 1019

(3345) Meyer F, Baror O, Macdougall D, Heigenhauser GJF. Sweat Electrolyte Loss During Exercise in the Heat - Effects of Gender and Maturation. Medicine and Science in Sports and Exercise 1992;24(7):776-81. Off topic
Ref ID: 5480

(3346) Meyer U, Romann M, Zahner L, Schindler C, Puder JJ, Kraenzlin M, Rizzoli R, Kriemler S. Effect of a general school-based physical activity intervention on bone mineral content and density: a cluster-randomized controlled trial. Bone 2011;48:792-7. Not All Participants were Overweight and/or Obese
Ref ID: 4915

(3347) Meyer U, Roth R, Zahner L, Gerber M, Puder JJ, Hebestreit H, Kriemler S. Contribution of physical education to overall physical activity. Scandinavian Journal of Medicine and Science in Sports 2013 October;23(5):600-6. Inappropriate Study Design
Ref ID: 5983

(3348) Meyer U, Schindler C, Zahner L, Ernst D, Hebestreit H, van MW, Rocca HP, Probst-Hensch N, Puder JJ, Kriemler S. Long-term effect of a school-based physical activity program (KISS) on fitness and adiposity in children: a cluster-randomized controlled trial. PLoS ONE 2014;9(2):e87929. Inappropriate Study Design
Ref ID: 5984

(3349) Meylan C, Malatesta D. Effects of in-season plyometric training within soccer practice on explosive actions of young players. Journal of Strength and Conditioning Research 2009 December;23(9):2605-13. CT
Ref ID: 632

(3350) Meyrowitsch DW, Simonsen PE. Mass diethylcarbamazine chemotherapy for control of bancroftian filariasis: comparative efficacy of standard treatment and two semi-annual single-dose treatments. Transactions of the Royal Society of Tropical Medicine and Hygiene 1996;90:69-73. Drug intervention study
Ref ID: 4916

(3351) Méndez Ribas J. Amenorreas por pérdida de peso. Revista de la Sociedad Chilena de Obstetricia y Ginecologia Infantil y de la Adolescencia 1994;1(2):63-5. Off topic
Ref ID: 4334

(3352) Michigan A, Johnson TV, Master VA. Review of the relationship between C-reactive protein and exercise. [Review]. Molecular Diagnosis and Therapy 2011 October 1;15(5):265-75. Review article
Ref ID: 2687

(3353) Micklesfield LK, Levitt NS, Carstens MT, Dhansay MA, Norris SA, Lambert EV. Early life and current determinants of bone in South African children of mixed ancestral origin. Annals of Human Biology 2007 November;34(6):647-55. Off topic
Ref ID: 1126

(3354) Middleman AB, Vazquez I, Durant RH. Eating patterns, physical activity, and attempts to change weight among adolescents. Journal of Adolescent Health 1998 January;22(1):37-42. Survey or questionnaire
Ref ID: 2106

(3355) Middleton LE, Barnes DE, Lui LY, Yaffe K. Physical activity over the life course and its association with cognitive performance and impairment in old age. Journal of the American Geriatrics Society 2010 July;58(7):1322-6. Cross-

sectional study
Ref ID: 485

(3356) Mieskonen S, Eronen M, Malmberg LP, Turpeinen M, Kari MA, Hallman M. Controlled trial of dexamethasone in neonatal chronic lung disease: an 8-year follow-up of cardiopulmonary function and growth. Acta Paediatrica 2003;92:896-904. Subjects less than 2 years old
Ref ID: 4917

(3357) Migliaro ER, Contreras P, Bech S, Etxagibel A, Castro M, Ricca R, Vicente K. Relative influence of age, resting heart rate and sedentary life style in short-term analysis of heart rate variability. Brazilian Journal of Medical and Biological Research 2001 April;34(4):493-500. Not a randomized controlled trial (RCT)
Ref ID: 4335

(3358) Mijailovic V, Micic D, Mijailovic M. [Effects of a one-year weight reduction program and physical activity on obesity and comorbid conditions]. [Serbian]. Medicinski Pregled 2004 January;57(1-2):55-9. Diet & Exercise intervention
Ref ID: 1595

(3359) Milano GE, Rodacki A, Radominski RB, Leite N. Escala de VO2pico em adolescentes obesos e não-Obesos por diferentes métodos. Scale of VO2peak in obese and non-obese adolescents by different methods. Escala de VO2pico en adolescentes obesos y no obesos por diferentes métodos. Arquivos Brasileiros de Cardiologia 2009 December;93(6):598-602. Case-Control / Case Study
Ref ID: 4336

(3360) Milano GE, Leite N. Implicações práticas no nível de condicionamento cardiorrespiratório em criança e adolescentes obesos. Practical implications of the level cardiorrespiratory conditioning in children and adolescents obese. Motriz Revista de Educação Física (Improv) 2009 June;15(2):414-26. Review article
Ref ID: 4337

(3361) Miles MP, Keller JM, Kordick LK, Kidd JR. Basal, circadian, and acute inflammation in normal versus overweight men. Medicine and Science in Sports and Exercise 2012 December;44(12):2290-8. Inappropriate Population
Ref ID: 5985

(3362) Milgrom H, Fick RB, Su JQ, Reimann JD, Bush RK, Watrous ML, Metzger WJ. Treatment of allergic asthma with monoclonal anti-IgE antibody. rhuMAb-E25 Study Group. New England Journal of Medicine 1999;341:1966-73. Drug intervention study
Ref ID: 905

(3363) Milgrom H, Berger W, Nayak A, Gupta N, Pollard S, McAlary M, Taylor AF, Rohane P. Treatment of childhood asthma with anti-immunoglobulin E antibody (omalizumab). Pediatrics 2001;108(2). Drug intervention study
Ref ID: 3281

(3364) Milic DJ, Zivic SS, Bogdanovic DC, Karanovic ND, Golubovic ZV. Risk factors related to the failure of venous leg ulcers to heal with compression treatment. Journal of Vascular Surgery 2009 May;49(5):1242-7. Off topic
Ref ID: 763

(3365) Millán K, Morera M, Vargas C. Consejería a adolescentes: descripción epidemiológica y motivos de consulta. Revista Médica de Chile 2007 April;135(4):457-63. Retrospective study
Ref ID: 622

(3366) Miller BF, Fattor JA, Jacobs KA, Horning MA, Suh SH, Navazio F, Brooks GA. Metabolic and cardiorespiratory responses to "the lactate clamp". American Journal of Physiology - Endocrinology and Metabolism 2002 November;283(5):E889-E898. Off topic
Ref ID: 1798

(3367) Miller CD, Laskowski ER, Suman VJ. Effect of corrective rearfoot orthotic devices on ground reaction forces during ambulation. Mayo Clinic Proceedings 1996 August;71(8):757-62. Off topic
Ref ID: 2175

(3368) Miller J. Advances in pediatric obesity research. Pediatric Health 2008;2(1):71-7. Review article
Ref ID: 3282

(3369) Miller JL, Goldstone AP, Couch JA, Shuster J, He GJ, Driscoll DJ, Liu YJ, Schmalfuss IM. Pituitary abnormalities in Prader-Willi syndrome and early onset morbid obesity. American Journal of Medical Genetics Part A 2008;146A(5):570-7. Off topic
Ref ID: 5481

(3370) Miller JT, Btaiche IF. Oxandrolone in pediatric patients with severe thermal burn injury. Annals of Pharmacotherapy 2008;42(9):1310-5. Review article
Ref ID: 3283

(3371) Miller KS, Maxwell KD, Fasula AM, Parker JT, Zackery S, Wyckoff SC. Pre-Risk HIV-Prevention Paradigm Shift: The Feasibility and Acceptability of the Parents Matter! Program in HIV Risk Communities. Public Health Reports 2010;125:38-46. Off topic
Ref ID: 5482

(3372) Miller S, Hall DO, Clayton CB, Nelson R. Chest physiotherapy in cystic fibrosis: a comparative study of autogenic drainage and the active cycle of breathing

techniques with postural drainage. Thorax 1995;50:165-9. Off topic
Ref ID: 4919

(3373) Miller TL. A hospital-based exercise program to improve body composition, strength, and abdominal adiposity in 2 HIV-infected children. Aids Reader 2007;17(9):450-+. Case-Control / Case Study
Ref ID: 5483

(3374) Miller TL, Somarriba G, Kinnamon DD, Weinberg GA, Friedman LB, Scott GB. The effect of a structured exercise program on nutrition and fitness outcomes in human immunodeficiency virus-infected children. AIDS Research and Human Retroviruses 2010 March;26(3):313-9. Not a randomized controlled trial (RCT)
Ref ID: 564

(3375) Millikan RC, Newman B, Tse CK, Moorman PG, Conway K, Smith LV, Labbok MH, Geradts J, Bensen JT, Jackson S, Nyante S, Livasy C, Carey L, Earp HS, Perou CM. Epidemiology of basal-like breast cancer. Breast Cancer Research and Treatment 2008;109(1):123-39. Off topic
Ref ID: 5484

(3376) Millimet DL, Tchernis R, Husain M. School Nutrition Programs and the Incidence of Childhood Obesity. Journal of Human Resources 2010 June 1;45(3):640-54. Cross-sectional study
Ref ID: 3910

(3377) Millson D, Tepper SJ. Abstracts and Citations. Headache: The Journal of Head and Face Pain 43[6], 686-692. 2003. Abstract,
Ref ID: 3691

(3378) Milne FH, Judge DS. Brothers delay menarche and the onset of sexual activity in their sisters. Proceedings of the Royal Society B-Biological Sciences 2011;278(1704):417-23. Off topic
Ref ID: 5485

(3379) Minatto G, Roberto Régis R, Achour Junior A, Santos KD. Idade, maturação sexual, variáveis antropométricas e composição corporal: Influências na flexibilidade. Revista Brasileira de Cineantropometria e Desempenho Humano 2010 June;12(3). Cross-sectional study
Ref ID: 4338

(3380) Minehan MR, Riley MD, Burke LM. Effect of flavor and awareness of kilojoule content of drinks on preference and fluid balance in team sports. International Journal of Sport Nutrition and Exercise Metabolism 2002 March;12(1):81-92. Diet Intervention or Supplement Study
Ref ID: 1832

(3381) Minussi L, Mohrdieck R, Bercini M, Ranieri T, Sanseverino MTV, Momino W, Callegari-Jacques SM, Schuler-Faccini L. Prospective evaluation of pregnant

women vaccinated against rubella in southern Brazil. Reproductive Toxicology 2008;25(1):120-3. Off topic
Ref ID: 5486

(3382) Mishra G, Kok H, Ecob R, Cooper R, Hardy R, Kuh D. Cessation of Hormone Replacement Therapy After Reports of Adverse Findings From Randomized Controlled Trials: Evidence From a British Birth Cohort. American Journal of Public Health 2006 July;96(7):1219-25. Off topic
Ref ID: 3845

(3383) Misquiatti ARN, Cristovão MP, Brito MC. Percurso e resultados da terapia fonoaudiológica na síndrome de Prader-Willi (SPW): Relato de caso. Trajectory and outcomes of speech language therapy in the Prader-Willi syndrome (PWS): Case report. Jornal da Sociedade Brasileira de Fonoaudiologia 2011 March;23(1):77-81. Case-Control / Case Study
Ref ID: 4339

(3384) Miszko TA, Cress ME, Slade JM, Covey CJ, Agrawal SK, Doerr CE. Effect of strength and power training on physical function in community-dwelling older adults. The Journals of Gerontology Series A, Biological Sciences and Medical Sciences 2003;58:171-5. Study limited to adults
Ref ID: 4920

(3385) Mitchell BM, Gutin B, Kapuku G, Barbeau P, Humphries MC, Owens S, Vemulapalli S, Allison J. Left ventricular structure and function in obese adolescents: relations to cardiovascular fitness, percent body fat, and visceral adiposity, and effects of physical training. Pediatrics 2002 May;109(5):E73. No exercise only group, No comparative control group
Ref ID: 382

(3386) Mitchell JB, Costill DL, Houmard JA, Flynn MG, Fink WJ, Beltz JD. Effects of carbohydrate ingestion on gastric emptying and exercise performance. Medicine and Science in Sports and Exercise 1988 April;20(2):110-5. Diet Intervention or Supplement Study
Ref ID: 2688

(3387) Mitchell JB, Rowe JR, Shah M, Barbee JJ, Watkins AM, Stephens C, Simmons S. Effect of prior exercise on postprandial triglycerides in overweight young women after ingesting a high-carbohydrate meal. International Journal of Sport Nutrition and Exercise Metabolism 2008 February;18(1):49-65. Study limited to adults
Ref ID: 197

(3388) Mitchell JB, Phillips MD, Yellott RC, Currie LM. Resistance and aerobic exercise: the influence of mode on the relationship between IL-6 and glucose tolerance in young men who are obese. Journal of Strength and Conditioning

Research 2011 June;25(6):1529-37. Acute study
Ref ID: 2689

(3389) Mitchell RMS, Byrne MF, Baillie J. Pancreatitis. Lancet 2003 April 26;361(9367):1447. Off topic
Ref ID: 3692

(3390) Miura MS, Saleh C, de AM, Assmann M, Lima LH, Lubianca Neto JF. Topical clindamycin in post-adenotonsillectomy analgesia in children: a double-blind, randomized clinical trial. Otolaryngology - Head and Neck Surgery 2009 October;141(4):509-15. Off topic
Ref ID: 669

(3391) Miyamura M, Ishida K, Itoh H, Ohkuwa T. Relationship between maximal pulmonary ventilation and arterialized venous blood potassium and dopamine concentrations obtained at exhaustion in man. Japanese Journal of Physiology 1998 February;48(1):17-23. Off topic
Ref ID: 2097

(3392) Miyashita M, Burns SF, Stensel DJ. Accumulating short bouts of brisk walking reduces postprandial plasma triacylglycerol concentrations and resting blood pressure in healthy young men. American Journal of Clinical Nutrition 2008 November;88(5):1225-31. Acute study
Ref ID: 863

(3393) Mo-suwan L, Pongprapai S, Junjana C, Puetpaiboon A. Effects of a controlled trial of a school-based exercise program on the obesity indexes of preschool children. American Journal of Clinical Nutrition 1998 November;68(5):1006-11. Not All Participants were Overweight and/or Obese
Ref ID: 420

(3394) Mobini S, Chambers LC, Yeomans MR. Effects of hunger state on flavour pleasantness conditioning at home: Flavour-nutrient learning vs. flavour-flavour learning. Appetite 2007;48(1):20-8. Off topic
Ref ID: 5487

(3395) Mobley CC. Lifestyle interventions for "diabesity": The state of the science. [Review] [24 refs]. Compendium of Continuing Education in Dentistry 211 February;25(3):207-8. Review article
Ref ID: 1563

(3396) Modolo VB, Mello MTd, Gimenez PRBd, Tufik S, Antunes HK. Dependência de exercício físico: Humor, qualidade de vida em atletas amadores e profissionais. Physical exercise dependence: Mood, quality of life in amateur and professional athletes. Revista Brasileira de Medicina do Esporte 2009 October;15(5):355-9. Off topic
Ref ID: 4340

(3397) Mogul HR, Lee PDK, Whitman BY, Zipf WB, Frey M, Myers S, Cahan M, Pinyerd B, Southren AL. Growth hormone treatment of adults with Prader-Willi syndrome and growth hormone deficiency improves lean body mass, fractional body fat, and serum triiodothyronine without glucose impairment: Results from the United States multicenter trial. Journal of Clinical Endocrinology and Metabolism 2008;93(4):1238-45. Study limited to adults
Ref ID: 5488

(3398) Moheeb H, Wali YA, El-Sayed MS. Physical fitness indices and anthropometrics profiles in schoolchildren with sickle cell trait/disease. American Journal of Hematology 2007 February;82(2):91-7. Cross-sectional study
Ref ID: 1285

(3399) Moholdt TT, Salvesen K, Ingul CB, Vik T, Oken E, Morkved S. Exercise Training in Pregnancy for obese women (ETIP): study protocol for a randomised controlled trial. Trials [Electronic Resource] 2011;12:154. Study limited to adults
Ref ID: 2690

(3400) Mok E, Letellier G, Cuisset JM, Denjean A, Gottrand F, Alberti C, Hankard R. Lack of functional benefit with glutamine versus placebo in Duchenne muscular dystrophy: a randomized crossover trial. PLoS ONE [Electronic Resource] 2009;4(5):e5448. Drug intervention study
Ref ID: 757

(3401) Molenaar EA, van Ameijden EJ, Vergouwe Y, Grobbee DE, Numans ME. Effect of nutritional counselling and nutritional plus exercise counselling in overweight adults: a randomized trial in multidisciplinary primary care practice. Family Practice 2010 April;27(2):143-50. Study limited to adults
Ref ID: 72

(3402) Molero-Conejo E, Morales LM, Fernández V, Raleigh X, Gómez ME, Semprún-Fereira M, Campos G, Ryder E. Lean adolescents with increased risk for metabolic syndrome. Archivos Latinoamericanos de Nutrición 2003 March;53(1):39-46. Cross-sectional study
Ref ID: 4341

(3403) Molina-Carballo A, Fernandez-Tardaguila E, Uberos-Fernandez J, Seiquer I, Contreras-Chova F, Munoz-Hoyos A. Longitudinal study of the simultaneous secretion of melatonin and leptin during normal puberty. Hormone Research 2007;68(1):11-9. Longitudinal Study
Ref ID: 5489

(3404) Molina MdCB, Faria CPd, Montero MP, Cade NV, Mill JG. Fatores de risco cardiovascular em crianças de 7 a 10 anos de área urbana, Vitória, Espírito Santo, Brasil. Cardiovascular risk factors in 7-to-10-year-old children in Vitória,

Espírito Santo State, Brazil. Cadernos de Saúde Pública 2010 May;26(5):909-17. Cross-sectional study
Ref ID: 4342

(3405) Moliner-Urdiales D, Ruiz JR, Ortega FB, Rey-Lopez JP, Vicente-Rodriguez G, Espana-Romero V, Munguia-Izquierdo D, Castillo MJ, Sjostrom M, Moreno LA, HELENA Study Group. Association of objectively assessed physical activity with total and central body fat in Spanish adolescents; the HELENA Study. International Journal of Obesity 2009 October;33(10):1126-35. Cross-sectional study
Ref ID: 664

(3406) Moliner-Urdiales D, Ruiz JR, Vicente-Rodriguez G, Ortega FB, Rey-Lopez JP, Espana-Romero V, Casajus JA, Molnar D, Widhalm K, Dallongeville J, Gonzalez-Gross M, Castillo MJ, Sjostrom M, Moreno LA, HELENA Study Group. Associations of muscular and cardiorespiratory fitness with total and central body fat in adolescents: the HELENA study. British Journal of Sports Medicine 2011 February;45(2):101-8. Cross-sectional study
Ref ID: 2691

(3407) Moller A, Masharawi Y. The effect of first ballet classes in the community on various postural parameters in young girls. Physical Therapy in Sport 2011 November;12(4):188-93. Longitudinal Study
Ref ID: 2692

(3408) Molnar BE, Gortmaker SL, Bull FC, Buka SL. Unsafe to play? Neighborhood disorder and lack of safety predict reduced physical activity among urban children and adolescents. American Journal of Health Promotion 2004;18(5):378-86. Cross-sectional study
Ref ID: 5490

(3409) Molnar D, Torok K, Erhardt E, Jeges S. Safety and efficacy of treatment with an ephedrine/caffeine mixture. The first double-blind placebo-controlled pilot study in adolescents. International Journal of Obesity and Related Metabolic Disorders 2000 December;24(12):1573. Diet Intervention or Supplement Study
Ref ID: 3693

(3410) Monasta L, Batty GD, Macaluso A, Ronfani L, Lutje V, Bavcar A, van Lenthe FJ, Brug J, Cattaneo A. Interventions for the prevention of overweight and obesity in preschool children: a systematic review of randomized controlled trials. [Review]. Obesity Reviews 2011 May;12(5):e107-e118. Review article
Ref ID: 2693

(3411) Monge-Rojas R. Serum lipids and lipoprotein levels in Costa Rican 13-18 year-old teenagers. Archivos Latinoamericanos de Nutrición 2001 September;51(3):236-43. Cross-sectional study
Ref ID: 4343

(3412) Monte CMGd, Ashworth A, Sa MLB, Diniz RLP. Effectiveness of nutrition centers in Ceara state, northeastern Brazil. Revista Panamericana de Salud Pública 1998 December;4(6):375-82. Diet Intervention Study
Ref ID: 4344

(3413) Monteiro SM, Jancey J, Howat P, Burns S, Jones C, Dhaliwal SS, McManus A, Hills AP, Anderson AS. The protocol of a randomized controlled trial for playgroup mothers: Reminder on Food, Relaxation, Exercise, and Support for Health (REFRESH) Program. BMC Public Health 2011;11:648. Description versus conduct of study
Ref ID: 2694

(3414) Montezuma T, Antônio FI, Silva ACJdSR, Sá MFSd, Ferriani RA, Ferreira CHJ. Assessment of symptoms of urinary incontinence in women with polycystic ovary syndrome. Clinics 2011;66(11):1911-5. Off topic
Ref ID: 4345

(3415) Montgomery DL, França NMd, Matsudo VKR. Uma comparaçäo das características físicas entre escolares brasileiros e canadenses de 7 a 18 anos. Revista Brasileira de Ciência e Movimento 1989 October;3(4):16-22. Cross-sectional study
Ref ID: 4346

(3416) Moore DB. Obesity clinical trials in youth: concepts and challenges. Ethnicity and Disease 2002;12(4):S3. Review article
Ref ID: 1788

(3417) Moore JB, Pawloski LR, Goldberg P, Kyeung MO, Stoehr A, Baghi H. Childhood obesity study: a pilot study of the effect of the nutrition education program Color My Pyramid. Journal of School Nursing 2009 June;25(3):230-9. Not a randomized controlled trial (RCT)
Ref ID: 754

(3418) Moore MJ, White GL, Moore DL. Association of relative backpack weight with reported pain, pain sites, medical utilization, and lost school time in children and adolescents. Journal of School Health 77(5):232-9, 2007 May 2007;(5):232-9. Off topic
Ref ID: 3075

(3419) Moore MS, Dodd CJ, Welsman JR, Armstrong N. Short-term appetite and energy intake following imposed exercise in 9- to 10-year-old girls. Appetite 2004 October;43(2):127-34. Diet Intervention or Supplement Study
Ref ID: 1584

(3420) Moore SM, Borawski EA, Cuttler L, Levers-Landis CE, Love TE. IMPACT: a multi-level family and school intervention targeting obesity in urban youth. Contemporary Clinical Trials 2013 November;36(2):574-86. Inappropriate

Intervention
Ref ID: 5986

(3421) Mora Garda G, Ramos Clason E, Anaya Lorduy F, Malambo García D, Gómez Alegría C, Gómez Camargo D. Body Mass Index and its correlation with pulmonary function in patients with cystic fibrosis from Cartagena (Colombia). Indice de Masa Corporal y su correlación con la función pulmonar en pacientes con fibrosis quística en Cartagena (Colombia). Salud Uninorte 2011 January;27(1):22-9. Cross-sectional study
Ref ID: 4347

(3422) Moraes-Macêdo M, Roquetti-Fernandes P, Fernandes-Filho J. Tabelas de referências das qualidades físicas básicas de meninos de 9 a 14 anos. Basic physical attribute reference (anthropometric) tables for 9 to 14 year-old children. Tablas de referencia de capacidades físicas básicas en niños de 9 a 14 años. Revista de Salud Pública 2011 August;13(4):654-62. Off topic
Ref ID: 4348

(3423) Moraes AC, Falcao MC. Lifestyle factors and socioeconomic variables associated with abdominal obesity in Brazilian adolescents. Annals of Human Biology 2013 January;40(1):1-8. Inappropriate Study Design
Ref ID: 5987

(3424) Moraes ACFd, Fernandes CAM, Elias RGM, Nakashima ATA, Reichert FF, Falcão MC. Prevalência de inatividade física e fatores associados em adolescentes. Prevalence of physical inactivity and associated factors in adolescents. Revista da Associação Médica Brasileira (1992) 2009;55(5):523-8. Cross-sectional study
Ref ID: 4349

(3425) Moraes SAd, Beltrán Rosas J, Mondini L, Freitas ICMd. Prevalência de sobrepeso e obesidade e fatores associados em escolares de área urbana de Chilpancingo, Guerrero, México, 2004. Cadernos de Saúde Pública 2006 June;22(6):1289-301. Cross-sectional study
Ref ID: 4350

(3426) Morais MB, Ferrari AA, Fisberg M. Effect of oral iron therapy on physical growth. Revista Paulista de Medicina 1993 December;111(6):439-44. Diet Intervention or Supplement Study
Ref ID: 811

(3427) Morales-Aguirre JJ, Sanchez-Ruiz M, Linares-Salas VH, Nandi -Lozano ME, Villalobos-Acosta CP, Cashat-Cruz M, Avila-Figueroa C. Condicion nutricia y apoyo alimentario en ninos con VIH. (Spanish). Boletin Medico del Hospital Infantil de Mexico 2002 April;59(4):250. Off topic
Ref ID: 3694

(3428) Morales-Ruán MdC, Hernández-Prado B, Gómez-Acosta LM, Shamah-Levy T, Cuevas-Nasu L. Obesity, overweight, screen time and physical activity in Mexican adolescents. Obesidad, sobrepeso, tiempo frente a la pantalla y actividad física en adolescentes mexicanos. Salud Pública de México 2009;51(supl.4):S613-S620. Cross-sectional study
Ref ID: 4351

(3429) Morales del Valle Z, Vázquez Calzada JL. Apgar score and infant mortality in Puerto Rico. Puerto Rico Health Sciences Journal 1994 September;13(3):175-81. Subjects less than 2 years old
Ref ID: 4352

(3430) Moran CN, Vassilopoulos C, Tsiokanos A, Jamurtas AZ, Bailey MES, Wilson RH, Pitsiladis YP. Effects of interaction between angiotensin I-converting enzyme polymorphisms and lifestyle on adiposity in adolescent Greeks. Obesity Research 2005;13(9):1499-504. Cross-sectional study
Ref ID: 5491

(3431) Moreau D, Kalaboka S, Choquet M, Annesi-Maesano I. Asthma, obesity, and eating behaviors according to the diagnostic and statistical manual of mental disorders IV in a large population-based sample of adolescents. American Journal of Clinical Nutrition 2009 May;89(5):1292-8. Survey or questionnaire
Ref ID: 767

(3432) MOREAU RICH. Hepatorenal syndrome in patients with cirrhosis. Journal of Gastroenterology and Hepatology 2002 July;17(7):739-47. Off topic
Ref ID: 3695

(3433) Moreira GC, Paiva Neto JVd, Godoy MRPd, Cipullo JP. Hipertensão arterial: Aspectos farmacológicos e terapêuticos. Revista da Sociedade Brasileira de Clínica Médica 2007 June;5(3):92-9. Study limited to adults
Ref ID: 4353

(3434) Moreira MEL, Goldani MZ. Child is the father of man: new challenges for child health. Ciencia and Saude Coletiva 2010;15(2):321-7. Review article
Ref ID: 5492

(3435) Moreira PVL, Freitas CHSdM. Educação em saúde nos cenários de prática dos estudantes de nutrição - Relato de experiência. Health education in the practice of nutrition undergradates - Experience report. Revista de APS 2010 December;13(4). Off topic
Ref ID: 4354

(3436) Morencos E, Romero B, Peinado AB, Gonzalez-Gross M, Fernandez C, Gomez-Candela C, Benito PJ. Effects of dietary restriction combined with different exercise programs or physical activity recommendations on blood lipids in overweight adults. Nutricion Hospitalaria 2012 November;27(6):1916-

27. Inappropriate Population
Ref ID: 5988

(3437) Moreno-Moraga J, Valero-Altes T, Riquelme AM, Isarria-Marcosy MI, de la Torre JR. Body contouring by non-invasive transdermal focused ultrasound. Lasers in Surgery and Medicine 2007 April;39(4):315-23. Off topic
Ref ID: 1240

(3438) Moreno González M, Manrique Espinoza M, Guzmán Bondiek S, Maiz Gurruchaga A, Patiño Z, Valdés Corbalán R, Feuchtmann Sáez C. Cambios en los factores de riesgo metabólicos en pacientes obesos en tratamiento. Revista médica de Chile 2000 February;128(2):193-200. Retrospective study
Ref ID: 4355

(3439) Moreno JP, Kelley ML, Landry DN, Paasch V, Terlecki MA, Johnston CA, Foreyt JP. Development and validation of the Family Health Behavior Scale. International Journal of Pediatric Obesity 2011 June;6(2-2):e480-e486. Off topic
Ref ID: 2695

(3440) Moreno LA, Joyanes M, Mesana MI, Gonzalez-Gross M, Gil CM, Sarria A, Gutierrez A, Garaulet M, Perez-Prieto R, Bueno M, Marcos A. Harmonization of anthropometric measurements for a multicenter nutrition survey in Spanish adolescents. Nutrition 2003;19(6):481-6. Off topic
Ref ID: 5493

(3441) Moreno LA. Interventions to improve cardiovascular risk factors in obese children. Journal of Pediatric Gastroenterology and Nutrition 2006 October;43(4):433-5. Editorial or letter or comment
Ref ID: 1341

(3442) Moreno LA, Bel-Serrat S, Santaliestra-Pasias AM, Rodriguez G. Obesity prevention in children. World Review of Nutrition and Dietetics 2013;106:119-26. Inappropriate Study Design
Ref ID: 5989

(3443) Morgan CM, Tanofsky-Kraff M, Wilfley DE, Yanovski JA. Childhood obesity. [Review] [131 refs]. Child and Adolescent Psychiatric Clinics of North America 2002 April;11(2):257-78. Review article
Ref ID: 1812

(3444) Morgan PJ, Lubans DR, Collins CE, Warren JM, Callister R. The SHED-IT randomized controlled trial: evaluation of an Internet-based weight-loss program for men. Obesity (Silver Spring) 2009 November;17(11):2025-32. Study limited to adults
Ref ID: 128

(3445) Morgan PJ, Collins CE, Plotnikoff RC, McElduff P, Burrows T, Warren JM, Young MD, Berry N, Saunders KL, Aguiar EJ, Callister R. The SHED-IT

community trial study protocol: a randomised controlled trial of weight loss programs for overweight and obese men. BMC Public Health 2010 November 16;10:701.:701. Study limited to adults
Ref ID: 11

(3446) Morgan PJ, Collins CE, Plotnikoff RC, Cook AT, Berthon B, Mitchell S, Callister R. Efficacy of a workplace-based weight loss program for overweight male shift workers: the Workplace POWER (Preventing Obesity Without Eating like a Rabbit) randomized controlled trial. Preventive Medicine 2011 May;52(5):317-25. Study limited to adults
Ref ID: 1045

(3447) Morgan PJ, Lubans DR, Plotnikoff RC, Callister R, Burrows T, Fletcher R, Okely AD, Young MD, Miller A, Clay V, Lloyd A, Collins CE. The 'Healthy Dads, Healthy Kids' community effectiveness trial: study protocol of a community-based healthy lifestyle program for fathers and their children. BMC Public Health 2011;11:876. Description versus conduct of study
Ref ID: 2697

(3448) Morgan PJ, Lubans DR, Callister R, Okely AD, Burrows TL, Fletcher R, Collins CE. The 'Healthy Dads, Healthy Kids' randomized controlled trial: efficacy of a healthy lifestyle program for overweight fathers and their children. International Journal of Obesity 2011 March;35(3):436-47. Lifestyle Intervention
Ref ID: 2699

(3449) Morinobu T, Murata T, Takaya R, Tamai H. Nutritional status of beta-carotene, alpha-tocopherol and retinol in obese children. International Journal for Vitamin and Nutrition Research 2002;72(3):119-23. Diet Intervention or Supplement Study
Ref ID: 5494

(3450) Morio B, Beaufrere B, Montaurier C, Verdier E, Ritz P, Fellmann N, Boirie Y, Vermorel M. Gender differences in energy expended during activities and in daily energy expenditure of elderly people. American Journal of Physiology-Endocrinology and Metabolism 1997;273(2):E321-E327. Study limited to adults
Ref ID: 5495

(3451) Morley R, Lucas A. Randomized diet in the neonatal period and growth performance until 7.5-8 y of age in preterm children. American Journal of Clinical Nutrition 2000;71:822-8. Subjects less than 2 years old
Ref ID: 4921

(3452) Moroño M, Díaz L, Fleitas O, Amador M, García T. Indice de aptitud fisica: sus cambios durante la reducción de peso en adolescentes obesos. Revista Cubana de Pediatria 1986 April;58(2):166-74. Not a randomized controlled trial (RCT)
Ref ID: 4356

(3453) Morris AK, Boyle DIR. Adherence to insulin treatment, glycaemic control, and ketoacidosis in insulin-dependent diabetes me. Lancet 1997 November 22;350(9090):1505. Off topic
Ref ID: 3696

(3454) Morris FL, Payne WR, Wark JD. Prospective decrease in progesterone concentrations in female lightweight rowers during the competition season compared with the off season: a controlled study examining weight loss and intensive exercise. British Journal of Sports Medicine 1999 December;33(6):417-22. Off topic
Ref ID: 2059

(3455) Morrison JA, Payne G, Barton BA, Khoury PR, Crawford P. Mother-daughter correlations of obesity and cardiovascular disease risk factors in black and white households: the NHLBI Growth and Health Study. American Journal of Public Health 1994 November;84(11):1761-7. Cross-sectional study
Ref ID: 2232

(3456) Morrison JA, Glueck CJ, Wang P. Preteen insulin levels interact with caloric intake to predict increases in obesity at ages 18 to 19 years: a 10-year prospective study of black and white girls. Metabolism: Clinical and Experimental 2010 May;59(5):718-27. Prospective Study
Ref ID: 546

(3457) Mortimore IL, Whittle AT, Douglas NJ. Comparison of nose and face mask CPAP therapy for sleep apnoea. Thorax 1998;53(4):290-2. Off topic
Ref ID: 3284

(3458) Mory PB, Crispim F, Kasamatsu T, Gabbay MAL, Dib SA, Moisés RS. Atypical generalized lipoatrophy and severe insulin resistance due to a heterozygous LMNA p. T10I mutation. Lipoatrofia generalizada atípica e resistência insulínica grave devido à mutação p. T10I em heterozigose no gene LMNA. Arquivos Brasileiros de Endocrinologia and Metabologia 2008 November;52(8):1252-6. Case-Control / Case Study
Ref ID: 4357

(3459) Mosher PE, Nash MS, Perry AC, LaPerriere AR, Goldberg RB. Aerobic circuit exercise training: Effect on adolescents with well-controlled insulin-dependent diabetes mellitus. Archives of Physical Medicine and Rehabilitation 1998;79(6):652-7. CT
Ref ID: 5496

(3460) Mosso C, Santander V, Pettinelli R, Valdés G, Celis B, Espejo S F, Navarro M, Sepúlveda V. Evaluación de una intervención en actividad física en niños con síndrome de Down. Evaluation of a physical activity intervention among children with Down's syndrome. Revista Chilena de Pediatría 2011

August;82(4):311-8. Not All Participants were Overweight and/or Obese
Ref ID: 4358

(3461) Mota DM, Barros AJD, Matijasevich A, Santos IS. Avaliação longitudinal do controle esfincteriano em uma coorte de crianças Brasileiras. Longitudinal study of sphincter control in a cohort of Brazilian children. Jornal de Pediatria 2010 October;86(5):429-34. Off topic
Ref ID: 4359

(3462) Mota J, Flores L, Flores L, Ribeiro JC, Santos MP. Relationship of single measures of cardiorespiratory fitness and obesity in young schoolchildren. American Journal of Human Biology 2006 May;18(3):335-41. Cross-sectional study
Ref ID: 1395

(3463) Moukarzel AA, Sabri MT. Gastric physiology and function: Effects of fruit juices. Journal of the American College of Nutrition 1996;15(5):S18-S25. Diet Intervention Study
Ref ID: 5497

(3464) Moura EC, Malta DC, Morais Neto OLd, Monteiro CA. Prevalence and social distribution of risk factors for chronic noncommunicable diseases in Brazil. Prevalencia y distribución social de los factores de riesgo de enfermedades crónicas no transmisibles en Brasil. Revista Panamericana de Salud Pública 2009 July;26(1):17-22. Off topic
Ref ID: 4360

(3465) Moura EC, Silva SAd, Malta DC, Morais Neto OL. Fatores de risco e proteção para doenças crônicas: vigilância por meio de inquérito telefônico, VIGITEL, Brasil, 2007t. Risk and protective factors for chronic non-communicable diseases: the VIGITEL telephone disease surveillance system, Brazil, 2007. Cadernos de Saúde Pública 2011 March;27(3):486-96. Off topic
Ref ID: 4362

(3466) Moura EC, Claro RM, Bernal R, Ribeiro J, Malta DC, Morais Neto O. A feasibility study of cell phone and landline phone interviews for monitoring of risk and protection factors for chronic diseases in Brazil. Exequibilidade do uso de entrevistas por telefone celular e por telefone fixo no monitoramento de fatores de risco e proteção para doenças crônicas. Cadernos de Saúde Pública 2011 February;27(2):277-86. Off topic
Ref ID: 4361

(3467) Moura JARd, Rech CR, Fonseca PHSd, Zinn JL. Validação de equações para a estimativa da densidade corporal em atletas de futebol categoria sub-20. Revista Brasileira de Cineantropometria e Desempenho Humano 2003;5(2). Off topic
Ref ID: 567

(3468) Moura JARd, Lunardi CC, Zinn JL. Efeito agudo do treinamento resistido com pesos sobre o peso hidrostático corporal e percentual de gordura. Revista Brasileira de Cineantropometria e Desempenho Humano 2004 November;6(2):45-52. Study limited to adults
Ref ID: 679

(3469) Moya MP, Sanchez LM, Lopez BJ, Escribano SF, Notario PB, Salcedo AF, Martinez V, V. [Cost-effectiveness of an intervention to reduce overweight and obesity in 9-10-year-olds. The Cuenca study]. [Spanish]. Gaceta Sanitaria 2011 May;25(3):198-204. Not All Participants were Overweight and/or Obese
Ref ID: 2700

(3470) Moyer-Mileur LJ, Brunstetter V, McNaught TP, Gill G, Chan GM. Daily physical activity program increases bone mineralization and growth in preterm very low birth weight infants. Pediatrics 2000 November;106(5):1088-92. Subjects less than 2 years old
Ref ID: 1949

(3471) Moyer-Mileur LJ, Xie B, Ball SD, Pratt T. Bone mass and density response to a 12-month trial of calcium and vitamin D supplement in preadolescent girls. J Journal of Musculoskeletal and Neuronal Interactions 2003;3(1):63-70. Diet Intervention or Supplement Study
Ref ID: 3285

(3472) Moyer-Mileur LJ, Ball SD, Brunstetter VL, Chan GM. Maternal-administered physical activity enhances bone mineral acquisition in premature very low birth weight infants. Journal of Perinatology 2008 June;28(6):432-7. Subjects less than 2 years old
Ref ID: 946

(3473) Moyer-Mileur LJ, Ransdell L, Bruggers CS. Fitness of children with standard-risk acute lymphoblastic leukemia during maintenance therapy: response to a home-based exercise and nutrition program. Journal of Pediatric Hematology/Oncology 2009 April;31(4):259-66. Diet & Exercise intervention
Ref ID: 775

(3474) Mozaffari KH, Shakiba M, Eftekhari MH, Fatehi F. Effects of zinc supplementation on physical growth in 2-5-year-old children. Biological Trace Element Research 2009;128:118-27. Diet Intervention or Supplement Study
Ref ID: 4923

(3475) Mozen D, Cradic S, Lehwald H. Establishing a before School Activity Program. Strategies: A Journal for Physical and Sport Educators 2010 March 1;23(4):24-7. Not a randomized controlled trial (RCT)
Ref ID: 3911

(3476) Mølgaard C, Thomsen BL, Michaelsen KF. Effect of habitual dietary calcium intake on calcium supplementation in 12-14-y-old girls. American Journal of

Clinical Nutrition 2004;80:1422-7. Diet Intervention or Supplement Study
Ref ID: 4924

(3477) Mujika I, Santisteban J, Castagna C. In-season effect of short-term sprint and power training programs on elite junior soccer players. Journal of Strength and Conditioning Research 2009 December;23(9):2581-7. Off topic
Ref ID: 631

(3478) Muktabhant B, Lumbiganon P, Ngamjarus C, Dowswell T. Interventions for preventing excessive weight gain during pregnancy. [Review]. Cochrane Database of Systematic Reviews 2012;4:CD007145. Review article
Ref ID: 2701

(3479) Muller-Ehmsen J, Braun D, Schneider T, Pfister R, Worm N, Wielckens K, Scheid C, Frommolt P, Flesch M. Decreased number of circulating progenitor cells in obesity: beneficial effects of weight reduction. European Heart Journal 2008 June;29(12):1560-8. Diet & Exercise intervention
Ref ID: 937

(3480) Muller HL, Muller-Stover S, Gebhardt U, Kolb R, Sorensen N, Handwerker G. Secondary narcolepsy may be a causative factor of increased daytime sleepiness in obese childhood craniopharyngioma patients. Journal of Pediatric Endocrinology 2006 April;19:Suppl-9. Off topic
Ref ID: 1392

(3481) Mulligan K, Harris DR, Monte D, Stoszek S, Emmanuel P, Hardin DS, Kapogiannis BG, Worrell C, Meyer WA, III, Sleasman J, Wilson CM, Aldrovandi GM, Adolescent TN. Obesity and dyslipidemia in behaviorally HIV-infected young women: Adolescent Trials Network study 021. Clinical Infectious Diseases 2010 January 1;50(1):106-14. Cross-sectional study
Ref ID: 608

(3482) Mun S, Decker EA, McClements DJ. Influence of emulsifier type on in vitro digestibility of lipid droplets by pancreatic lipase. Food Research International 2007;40(6):770-81. Off topic
Ref ID: 5498

(3483) Mungrue K, Fyzul A, Ramroop S, Persad T, Asgarali A. Are teenagers at risk for developing cardiovascular disease in later life? International Journal of Adolescent Medicine and Health 2013;25(1):75-80. Inappropriate Study Design
Ref ID: 5990

(3484) Munguba MC, Valdes MT, da Silva CA. The application of an occupational therapy nutrition education programme for children who are obese. Occupational Therapy International 2008;15(1):56-70. Diet Intervention Study
Ref ID: 991

(3485) Munguia-Izquierdo D, Legaz-Arrese A. Exercise in warm water decreases pain and improves cognitive function in middle-aged women with fibromyalgia. Clinical and Experimental Rheumatology 2007 November;25(6):823-30. Study limited to adults
Ref ID: 1122

(3486) Munoz JA, Garcia C, Quilez JL, Andugar MA. Effect of vitamin C on lipoproteins in healthy adults. Annales de Medecine Interne 1994;145(1):13-9. Study limited to adults
Ref ID: 2244

(3487) Muñoz S, Soltero I, Onorato E, Pietri C, Zambrano F. Parametros morfologicos y funcionales del ventriculo izquierdo (masa, grosor parietal y stress parietal telesistolico) en escolares con diferentes niveles de presionarterial, en reposo y durante el ejercicio maximo. Acta Científica Venezolana 1990;41(2):106-13. Off topic
Ref ID: 4363

(3488) Murat I, Bernière J, Constant I. Evaluation of the efficacy of a forced-air warmer (Bair Hugger) during spinal surgery in children. Journal of Clinical Anesthesia 1994;6:425-9. Off topic
Ref ID: 1018

(3489) Muratori LM, Reilmann R, Gordon AM. Coordination of fingertip forces during precision grasping in multiple system atrophy. Neuropsychologia 2003;41(11):1498-508. Off topic
Ref ID: 5499

(3490) Murdoch SD, Bazzarre TL, Snider IP, Goldfarb AH. Differences in the effects of carbohydrate food form on endurance performance to exhaustion. International Journal of Sport Nutrition 1993 March;3(1):41-54. Diet Intervention or Supplement Study
Ref ID: 2262

(3491) Murnan J, Price JH, Telljohann SK, Dake JA, Boardley D. Parents' perceptions of curricular issues affecting children's weight in elementary schools. Journal of School Health 2006 December;76(10):502-11. Survey or questionnaire
Ref ID: 1328

(3492) Murphy JL, Wootton SA. Nutritional management in cystic fibrosis--and alternative perspective in gastrointestinal function. Disability and Rehabilitation 1998 June;20(6/7):226. Diet Intervention Study
Ref ID: 554

(3493) Murray CJL, Lauer JA, Hutubessy RCW, Niessen L, Tomijima N, Rodgers A, Lawes CMM, Evans DB. Effectiveness and costs of interventions to lower systolic blood pressure and cholesterol: a global and regional analysis on reduction of cardiovascular-disease risk. Lancet 2003 March 3;361(9359):717.

Review article
Ref ID: 3697

(3494) Murray DM, Catellier DJ, Hannan PJ, Treuth MS, Stevens J, Schmitz KH, Rice JC, Conway TL. School-level intraclass correlation for physical activity in adolescent girls. Medicine and Science in Sports and Exercise 2004 May;36(5):876-82. Not All Participants were Overweight and/or Obese
Ref ID: 1633

(3495) Murray RD. The phenotype of adults with partial growth hormone deficiency. Hormone Research 2005;64:Suppl-7. Off topic
Ref ID: 1452

(3496) Murta AMG, Lessa AdC, Santos AS, Murta NMG, Cambraia RP. Cognição, motricidade, autocuidados, linguagem e socialização no desenvolvimento de crianças em creche. Cognition, motor activity, self care, language and socialization during children development in day care. Revista Brasileira de Crescimento e Desenvolvimento Humano 2011;21(2):220-9. Survey or questionnaire
Ref ID: 4364

(3497) Mustila T, Raitanen J, Keskinen P, Saari A, Luoto R. Pragmatic controlled trial to prevent childhood obesity in maternity and child health care clinics: pregnancy and infant weight outcomes (the VACOPP Study). BMC Pediatrics 2013;13:80. Inappropriate Study Design
Ref ID: 5991

(3498) Muth ND, Chatterjee A, Williams D, Cross A, Flower K. Making an IMPACT: effect of a school-based pilot intervention. North Carolina Medical Journal 2008 November;69(6):432-40. No exercise only group
Ref ID: 133

(3499) Mutlu A, Krosschell K, Spira DG. Treadmill training with partial body-weight support in children with cerebral palsy: a systematic review. [Review] [42 refs][Erratum appears in Developmental Medicine and Child Neurology . 2009 Sep;51(9):761]. Developmental Medicine and Child Neurology 2009 April;51(4):268-75. Review article
Ref ID: 777

(3500) Mutsaerts MA, Groen H, ter Bogt NC, Bolster JH, Land JA, Bemelmans WJ, Kuchenbecker WK, Hompes PG, Macklon NS, Stolk RP, van d, V, Maas JW, Klijn NF, Kaaijk EM, Oosterhuis GJ, Bouckaert PX, Schierbeek JM, van Kasteren YM, Nap AW, Broekmans FJ, Brinkhuis EA, Koks CA, Burggraaff JM, Blankhart AS, Perquin DA et al. The LIFESTYLE study: costs and effects of a structured lifestyle program in overweight and obese subfertile women to reduce the need for fertility treatment and improve reproductive outcome. A randomised controlled trial. BMC Womens Health 2010 June 25;10:22.:22.

Study not limited to children and adolescents
Ref ID: 35

(3501) Muzaffar H, Chapman-Novakofski K, Castelli DM, Scherer JA. The HOT (Healthy Outcome for Teens) project. Using a web-based medium to influence attitude, subjective norm, perceived behavioral control and intention for obesity and type 2 diabetes prevention. Appetite 2014 January;72:82-9. Inappropriate Study Design
Ref ID: 5992

(3502) Myers SE, Carrel AL, Whitman BY, Allen DB. Physical effects of growth hormone treatment in children with Prader-Willi syndrome. Acta paediatrica Supplement 1999;88:112-4. Drug intervention study
Ref ID: 4925

(3503) Myers SE, Carrel AL, Whitman BY, Allen DB. Sustained benefit after 2 years of growth hormone on body composition, fat utilization, physical strength and agility, and growth in Prader-Willi syndrome. Journal of Pediatrics 2000 July;137(1):42-9. Drug intervention study
Ref ID: 1972

(3504) Myers SE, Whitman BY, Carrel AL, Moerchen V, Bekx MT, Allen DB. Two years of growth hormone therapy in young children with Prader-Willi syndrome: physical and neurodevelopmental benefits. American Journal of Medical Genetics Part A 2007 March 1;143(5):443-8. Drug intervention study
Ref ID: 1266

(3505) Myers TA, Crowther JH. Is self-objectification related to interoceptive awareness? An examination of potential mediating pathways to disordered eating attitudes. Psychology of Women Quarterly 2008 June;32(2):172-80. Off topic
Ref ID: 3846

(3506) Nadal I, Santacruz A, Marcos A, Warnberg J, Garagorri M, Moreno LA, Martin-Matillas M, Campoy C, Marti A, Moleres A, Delgado M, Veiga OL, Garcia-Fuentes M, Redondo CG, Sanz Y. Shifts in clostridia, bacteroides and immunoglobulin-coating fecal bacteria associated with weight loss in obese adolescents. International Journal of Obesity 2009;33(7):758-67. Diet & Exercise intervention
Ref ID: 5500

(3507) Nader NS, Kumar S. Type 2 diabetes mellitus in children and adolescents: Where do we stand with drug treatment and behavioral management? Current Diabetes Reports 2008;8(5):383-8. Review article
Ref ID: 3286

(3508) Nader PR, Sallis JF, Patterson TL, Abramson IS, Rupp JW, Senn KL, Atkins CJ, Roppe BE, Morris JA, Wallace JP. A family approach to cardiovascular risk

reduction: results from the San Diego Family Health Project. Health Education Quarterly 1989;16(2):229-44. Diet & Exercise intervention
Ref ID: 2318

(3509) Nader PR, Stone EJ, Lytle LA, Perry CL, Osganian SK, Kelder S, Webber LS, Elder JP, Montgomery D, Feldman HA, Wu M, Johnson C, Parcel GS, Luepker RV. Three-year maintenance of improved diet and physical activity: the CATCH cohort. Child and Adolescent Trial for Cardiovascular Health. Archives of Pediatrics and Adolescent Medicine 1999 July;153(7):695-704. Follow-up Study
Ref ID: 2012

(3510) Nader PR, Bradley RH, Houts RM, McRitchie SL, O'Brien M. Moderate-to-vigorous physical activity from ages 9 to 15 years. Journal of the American Medical Association 2008;300(3):295-305. Longitudinal Study
Ref ID: 5501

(3511) Nagai N, Moritani T. Effect of physical activity on autonomic nervous system function in lean and obese children. International Journal of Obesity and Related Metabolic Disorders 2004 January;28(1):27-33. Not All Participants were Overweight and/or Obese
Ref ID: 1673

(3512) Nagel G, Wabitsch M, Galm C, Berg S, Brandstetter S, Fritz M, Klenk J, Peter R, Prokopchuk D, Steiner R, Stroth S, Wartha O, Weiland SK, Steinacker J. Determinants of obesity in the Ulm Research on Metabolism, Exercise and Lifestyle in Children (URMEL-ICE). European Journal of Pediatrics 2009 October;168(10):1259-67. Cross-sectional study
Ref ID: 111

(3513) Nahas MV, Barros MVGD, Florindo AA, Farias Júnior JCD, Hallal PC, Konrad L, Barros SSHD, Assis MAAD. Reprodutibilidade e validade do questionário saúde na boa para avaliar atividade física e hábitos alimentares em escolares do ensino médio. Revista Brasileira de Atividade Física e Saúde 2007;12(3). Survey or questionnaire
Ref ID: 4365

(3514) Naheed A, Walker-Fischer CL, Mondal D, Ahmed S, Arifeen SE, Yunus M, Black RE, Baqui AH. Zinc therapy for diarrhoea improves growth among Bangladeshi infants 6 to 11 months of age. Journal of Pediatric Gastroenterology and Nutrition 2009;48:89-93. Off topic
Ref ID: 4926

(3515) Najm W, Lie D. Herbals used for diabetes, obesity, and metabolic syndrome. Primary Care: Clinics in Office Practice 2010;37(2):237-54. Review article
Ref ID: 3287

(3516) Nakagawa TH, Muniz TB, Baldon RM, Maciel CD, Amorim CF, Serrão FV. Electromyographic preactivation pattern of the gluteus medius during weight-

bearing functional tasks in women with and without anterior knee pain. Padrão de pré-ativação eletromiográfica do glúteo médio durante atividades funcionais com descarga de peso em mulheres com e sem dor anterior do joelho. Revista Brasileira de Fisioterapia 2011 February;15(1):59-65. Study limited to adults
Ref ID: 4366

(3517) Nantel J, Brochu M, Prince F. Locomotor strategies in obese and non-obese children. Obesity 2006;14(10):1789-94. Not All Participants were Overweight and/or Obese
Ref ID: 5502

(3518) Naranjo AA, Rodriguez AY, Llera RE, Aroche R. Diabetes risk in a Cuban primary care setting in persons with no known glucose abnormalities. MEDICC Review 2013 April;15(2):16-9. Inappropriate Study Design
Ref ID: 5993

(3519) Narici M, Ferretti G, Susta D, Faglia G, Sartorio A. Maximum anaerobic performance of childhood-onset GH-deficient adults. Growth Hormone and Igf Research 1999 August;9(4):228-35. Study limited to adults
Ref ID: 1996

(3520) Nascimento TBRd, Pereira DC, Glaner MF. Prevalência de indicadores de aptidão física associada à saúde em escolares. Motriz Revista de Educação Física (Improv) 2010 June;16(2):387-94. Prevalence study
Ref ID: 4367

(3521) Nascimento VG, Salvador EP, Silva JPCd, Bertoli CJ, Blake MdT, Leone C. Overweight in preschool children: analysis of a possible intervention. Excesso de peso em pré-escolares: análise de uma intervenção possível. Revista Brasileira de Crescimento e Desenvolvimento Humano 2012;22(1):11-6. Diet & Exercise intervention
Ref ID: 4368

(3522) Naslund GK, Fredrikson M, Hellenius ML, de FU. Determinants of compliance in men enrolled in a diet and exercise intervention trial: a randomized, controlled study. Patient Education and Counseling 1996 December;29(3):247-56. Study limited to adults
Ref ID: 2151

(3523) Nassis GP, Sidossis LS. Methods for assessing body composition, cardiovascular and metabolic function in children and adolescents: implications for exercise studies. Current Opinion in Clinical Nutrition and Metabolic Care 2006;9(5):560-7. Review article
Ref ID: 5503

(3524) Natale R, Scott SH, Messiah SE, Schrack MM, Uhlhorn SB, Delamater A. Design and methods for evaluating an early childhood obesity prevention

program in the childcare center setting. BMC Public Health 2013;13:78. Inappropriate Study Design
Ref ID: 5994

(3525) Naufel M, Bordon M, Aquino T, Ribeiro E, Abreu Carvalhaes Jo. Plasma levels of acylated and total ghrelin in pediatric patients with chronic kidney disease. Pediatric Nephrology 2010 December;25(12):2477-82. Cross-sectional study
Ref ID: 3698

(3526) Nava B, Pérez G, Herrera HA, Hernández H. Hábitos Alimentarios, Actividad Física Y Su Relación Con El Estado. Nutricional-antropométrico de preescolares. Anthropometric-Nutritional assessment, dietary habits and physical activity in preschool children. Revista Chilena de Nutrición 2011 September;38(3):301-12. Survey or questionnaire
Ref ID: 4369

(3527) Naylor LH, Watts K, Sharpe JA, Jones TW, Davis EA, Thompson A, George K, Ramsay JM, O'Driscoll G, Green DJ. Resistance training and diastolic myocardial tissue velocities in obese children. Medicine and Science in Sports and Exercise 2008 December;40(12):2027-32. CT, Not a randomized controlled trial (RCT)
Ref ID: 460

(3528) Näslund GK, Fredrikson M, Hellénius ML, de FU. Determinants of compliance in men enrolled in a diet and exercise intervention trial: a randomized, controlled study. Patient Education And Counseling 1996;29:247-56. Study limited to adults
Ref ID: 965

(3529) Needham LL, Wang RY. Analytic considerations for measuring environmental chemicals in breast milk. Environmental Health Perspectives 2002;110(6):A317-A324. Off topic
Ref ID: 5504

(3530) Needham RA, Morse CI, Degens H. The acute effect of different warm-up protocols on anaerobic performance in elite youth soccer players. Journal of Strength and Conditioning Research 2009 December;23(9):2614-20. Off topic
Ref ID: 633

(3531) Nees K. [Differences in eating behavior of men and women. Fish for the lady, meat for the gentleman?]. [German]. MMW Fortschritte der Medizin 2008 November 6;150(45):18. Diet Intervention Study
Ref ID: 847

(3532) Neidhart G, Pabelick C, Kuhn I, Leuwer M, Vettermann J. [Effect of halothane, enflurane and isoflurane on the pharmacodynamics of mivacurium in children]. Anästhesiologie , Intensivmedizin , Notfallmedizin , Schmerztherapie : AINS

1996;31:293-7. Drug intervention study
Ref ID: 4927

(3533) Neils CM, Udermann BE, Brice GA, Winchester JB, McGuigan MR. Influence of contraction velocity in untrained individuals over the initial early phase of resistance training. Journal of Strength and Conditioning Research 2005 November;19(4):883-7. Study limited to adults
Ref ID: 1449

(3534) Nelson KM. Designing healthier communities through the input of children. Journal of Public Health Management and Practice 2008;14(3):266-71. Not an exercise intervention study
Ref ID: 5505

(3535) Nelson MC, Gordon-Larsen P, North KE, Adair LS. Body mass index gain, fast food, and physical activity: Effects of shared environments over time. Obesity 2006;14(4):701-9. Longitudinal Study
Ref ID: 5506

(3536) Nelson P, Poon T, Guan X, Schnabel C, Wintle M, Fineman M. The incretin mimetic exenatide as a monotherapy in patients with type 2 diabetes. Diabetes Technology and Therapeutics 2007 August;9(4):317-26. Drug intervention study
Ref ID: 1181

(3537) Nemet D, Dolfin T, Litmanowitz I, Shainkin KR, Lis M, Eliakim A. Evidence for exercise-induced bone formation in premature infants. International Journal of Sports Medicine 2002;23:82-5. Subjects less than 2 years old
Ref ID: 4929

(3538) Nemet D, Connolly PH, Pontello-Pescatello AM, Rose-Gottron C, Larson JK, Galassetti P, Cooper DM. Negative energy balance plays a major role in the IGF-I response to exercise training. Journal of Applied Physiology 2004 January;96(1):276-82. Diet Intervention or Supplement Study
Ref ID: 1675

(3539) Nemet D, Barkan S, Epstein Y, Friedland O, Kowen G, Eliakim A. Short- and long-term beneficial effects of a combined dietary-behavioral-physical activity intervention for the treatment of childhood obesity. Pediatrics 2005 April;115(4):e443-e449. No exercise only group
Ref ID: 311

(3540) Nemet D, Berger-Shemesh E, Wolach B, Eliakim A. A combined dietary-physical activity intervention affects bone strength in obese children and adolescents. International Journal of Sports Medicine 2006 August;27(8):666-71. Not a randomized controlled trial (RCT), No exercise only group
Ref ID: 273

(3541) Nemet D, Barzilay-Teeni N, Eliakim A. Treatment of childhood obesity in obese families. Journal of Pediatric Endocrinology and Metabolism 2008 May;21(5):461-7. No exercise only group
Ref ID: 165

(3542) Nemet D, Geva D, Eliakim A. Health promotion intervention in low socioeconomic kindergarten children. Journal of Pediatrics 2011 May;158(5):796-801. Diet & Exercise intervention
Ref ID: 2702

(3543) Nemet D, Ben-Haim I, Pantanowits M, Eliakim A. Effects of a combined intervention for treating severely obese prepubertal children. Journal of Pediatric Endocrinology and Metabolism 2013;26(1-2):91-6. Inappropriate Intervention
Ref ID: 5996

(3544) Nemet D, Oren S, Pantanowitz M, Eliakim A. Effects of a multidisciplinary childhood obesity treatment intervention on adipocytokines, inflammatory and growth mediators. Hormone Research in Paediatrics 2013;79(6):325-32. Inappropriate Intervention
Ref ID: 5995

(3545) Nemoseck T, Kern M. The effects of high-impact and resistance exercise on urinary calcium excretion. International Journal of Sport Nutrition and Exercise Metabolism 2009 April;19(2):162-71. Off topic
Ref ID: 743

(3546) Nespoli L, Verri A, Nosetti L, Salvatoni A, Berini J, Chierici V, Niespolo AC, Cremante A. Sleep Disordered Breathing in Prader-Willi patients treated with rh GH. Journal of Intellectual Disability Research 2008 October;52(10):815. Off topic
Ref ID: 3847

(3547) Nessier V, Monsalvo C, Picech C, Pacheco S. J, Palma C, Martínez G, Ojeda O, Rosa DL, Casini B, Ibáñez S. T. Percepción de usuarios del programa de recuperación del niño en riesgo nutricional nutrir más del área urbana de la ciudad de Santa Fe, Argentina. Users' perception of the recovery program of the child in nutritional risk. Revista Chilena de Nutrición 2010 March;37(1):51-9. Survey or questionnaire
Ref ID: 4370

(3548) Nettlefold L, McKay HA, Warburton DE, McGuire KA, Bredin SS, Naylor PJ. The challenge of low physical activity during the school day: at recess, lunch and in physical education.[Erratum appears in British Journal of Sports Medicine. 2011 Aug;45(10):819]. British Journal of Sports Medicine 2011 August;45(10):813-9. Cross-sectional study
Ref ID: 2703

(3549) Neuhouser ML, Schwarz Y, Wang C, Breymeyer K, Coronado G, Wang CY, Noar K, Song X, Lampe JW. A low-glycemic load diet reduces serum C-reactive protein and modestly increases adiponectin in overweight and obese adults. Journal of Nutrition 2012 February;142(2):369-74. Diet Intervention Study
Ref ID: 2704

(3550) Neumann CG, Bwibo NO, Murphy SP, Sigman M, Whaley S, Allen LH, Guthrie D, Weiss RE, Demment MW. Animal source foods improve dietary quality, micronutrient status, growth and cognitive function in Kenyan school children: background, study design and baseline findings. Journal of Nutrition 2003 November;133(11:Suppl 2):Suppl-3949S. Diet Intervention Study
Ref ID: 1688

(3551) Neumann CG, Murphy SP, Gewa C, Grillenberger M, Bwibo NO. Meat supplementation improves growth, cognitive, and behavioral outcomes in Kenyan children. Journal of Nutrition 2007 April;137(4):1119-23. Diet Intervention Study
Ref ID: 1259

(3552) Neumark-Sztainer D, Story M, Resnick MD, Blum RW. Psychosocial concerns and weight control behaviors among overweight and nonoverweight Native American adolescents. Journal of the American Dietetic Association 1997 June;97(6):598-604. Cross-sectional study
Ref ID: 2139

(3553) Neumark-Sztainer D, Story M, Hannan PJ, Rex J. New Moves: a school-based obesity prevention program for adolescent girls. Preventive Medicine 2003 July;37(1):41-51. Not All Participants were Overweight and/or Obese
Ref ID: 361

(3554) Neumark-Sztainer D, Story M, Hannan PJ, Tharp T, Rex J. Factors associated with changes in physical activity: a cohort study of inactive adolescent girls. Archives of Pediatric Adolescent Medicine 2003 August;157(8):803-10. No exercise only group
Ref ID: 358

(3555) Neumark-Sztainer D, Haines J, Robinson-O'Brien R, Hannan PJ, Robins M, Morris B, Petrich CA. 'Ready. Set. ACTION!' A theater-based obesity prevention program for children: A feasibility study. Health Education Research 2009 June;24(3):407-20. No exercise only group
Ref ID: 170

(3556) Neumark-Sztainer DR, Friend SE, Flattum CF, Hannan PJ, Story MT, Bauer KW, Feldman SB, Petrich CA. New moves-preventing weight-related problems in adolescent girls a group-randomized study. American Journal of Preventive

Medicine 2010 November;39(5):421-32. No exercise only group
Ref ID: 17

(3557) Neves PMJ, Torcato AC, Urquieta AS, Kleiner AF. Importância do tratamento e prevenção da obesidade infantil. Childhood obesity the importance of the treatment and prevention. Arquivos de Ciências da Saúde 2010 September;17(3):150-3. Review article
Ref ID: 4371

(3558) Nevill A, Rowland T, Goff D, Martel L, Ferrone L. Scaling or normalising maximum oxygen uptake to predict 1-mile run time in boys. European Journal of Applied Physiology 2004 July;92(3):285-8. Off topic
Ref ID: 1612

(3559) Nevill AM, Holder RL, Baxter-Jones A, Round JM, Jones DA. Modeling developmental changes in strength and aerobic power in children. Journal of Applied Physiology 1998 March;84(3):963-70. Off topic
Ref ID: 2102

(3560) Newall H, Myles N, Ward PB, Samaras K, Shiers D, Curtis J. Efficacy of metformin for prevention of weight gain in psychiatric populations: A review. International Clinical Psychopharmacology 2012;27(2):69-75. Review article
Ref ID: 3288

(3561) Newsom SA, Paxton RJ, Rynn GM, Bell C. Influence of ascorbic acid on the thermic effect of feeding in overweight and obese adult humans. Obesity (Silver Spring) 2008 August;16(8):1749-54. Study limited to adults, Not an exercise intervention study
Ref ID: 179

(3562) Newton RL, Jr., Han H, Anton SD, Martin CK, Stewart TM, Lewis L, Champagne CM, Sothern M, Ryan D, Williamson DA. An environmental intervention to prevent excess weight gain in African-American students: a pilot study. American Journal of Health Promotion 2010 May;24(5):340-3. Diet & Exercise intervention
Ref ID: 532

(3563) Nguyen B, Kornman KP, Baur LA. A review of electronic interventions for prevention and treatment of overweight and obesity in young people. [Review]. Obesity Reviews 2011 May;12(5):e298-e314. Review article
Ref ID: 2706

(3564) Nguyen B, Shrewsbury VA, O'Connor J, Steinbeck KS, Lee A, Hill AJ, Shah S, Kohn MR, Torvaldsen S, Baur LA. Twelve-month outcomes of the loozit randomized controlled trial: a community-based healthy lifestyle program for overweight and obese adolescents. Archives of Pediatrics and Adolescent Medicine 2012 February;166(2):170-7. Lifestyle Intervention
Ref ID: 2707

(3565) Nguyen DM, El-Serag HB. The Epidemiology of Obesity. Gastroenterology Clinics of North America 2010;39(1):1-+. Review article
Ref ID: 5507

(3566) Nguyen PV, Hong TK, Hoang T, Nguyen DT, Robert AR. High prevalence of overweight among adolescents in Ho Chi Minh City, Vietnam. BMC Public Health 2013;13:141. Inappropriate Study Design
Ref ID: 5997

(3567) Nhantumbo L, Maia J, Saranga S, Fermino R, Prista A. Efeitos da idade, do sexo e da área geográfica no crescimento somático e aptidão física nas crianças e jovens rurais de Calanga, Moçambique. Revista Brasileira de Educação Física e Esporte 2007 December;21(4):271-89. Cross-sectional study
Ref ID: 4372

(3568) Nhantumbo L, Maia J, Saranga S, Prista A. Atividade física em crianças e jovens residentes em uma comunidade rural moçambicana: Efeitos da idade, sexo e estado nutricional. Revista Panamericana de Salud Pública 2008 March;23(3):171-8. Cross-sectional study
Ref ID: 4373

(3569) Ni H, Hu B, Ji SC, Yang SP, Lin YQ, Wang JN, Fu SH. [Meta analysis of the effects of passive swimming exercise training on the body mass of neonates]. Zhongguo Linchuang Kangfu 2005;9:166-8. Review article
Ref ID: 4932

(3570) Ni MC, Maddison R, Jiang Y, Jull A, Prapavessis H, Rodgers A. Couch potatoes to jumping beans: a pilot study of the effect of active video games on physical activity in children. International Journal of Behavioral Nutrition and Physical Activity 2008;5:8. Not All Participants were Overweight and/or Obese
Ref ID: 4933

(3571) Ni MC, Roberts V, Maddison R, Dorey E, Jiang Y, Jull A, Tin TS. Effect of electronic time monitors on children's television watching: pilot trial of a home-based intervention. Preventive Medicine 2009 November;49(5):413-7. Not All Participants were Overweight and/or Obese
Ref ID: 649

(3572) Nicacio SLdSM, Ribeiro AF. Atividade motora e o estado nutricional de lactentes hospitalizados com sibilância recorrente. Motor function and nutritional status in hospitalized infants with recurrent acute wheezing. Pediatria (São Paulo) 2010 September;32(3):184-90. Off topic
Ref ID: 4374

(3573) Nichols DL, Sanborn CF, Love AM. Resistance training and bone mineral density in adolescent females. Journal of Pediatrics 2001 October;139(4):494-

500. Not All Participants were Overweight and/or Obese
Ref ID: 1889

(3574) Nichols DL, Sanborn CF, Essery EV, Clark RA, Letendre JD. Impact of curriculum-based bone loading and nutrition education program on bone accrual in children. Pediatric Exercise Science 2008;20:411-25. Diet & Exercise intervention
Ref ID: 4934

(3575) Nichols JF, Rauh MJ, Barrack MT, Barkai HS. Bone mineral density in female high school athletes: interactions of menstrual function and type of mechanical loading. Bone 2007 September;41(3):371-7. Off topic
Ref ID: 1185

(3576) Nichols SD, Boyne MS, Thame M, Osmond C, Wilks RJ, Bennett FI, McFarlane AN, Young RE, Forrester TE. Cold-induced elevation of forearm vascular resistance is inversely related to birth weight. Journal of Human Hypertension 2005;19:309-14. Off topic
Ref ID: 4935

(3577) Nicholson AJ, Francis BM, Mulholland EK, Moulden AL, Oberklaid F. Health Screening of International Adoptees - Evaluation of A Hospital Based Clinic. Medical Journal of Australia 1992;156(6):377-9. Off topic
Ref ID: 5508

(3578) Nickols-Richardson SM, Miller LE, Wootten DF, Ramp WK, Herbert WG. Concentric and eccentric isokinetic resistance training similarly increases muscular strength, fat-free soft tissue mass, and specific bone mineral measurements in young women. Osteoporosis International 2007 June;18(6):789-96. Study limited to adults
Ref ID: 1239

(3579) Nicolao ALA, Pedrinelli A, Zogaib PSM, Orbetelli R, Barros Neto TLd. Influência da maturação sexual no limiar de lactato em jogadoras de futebol. Influence of sexual maturation in lactate threshold in female soccer players. Revista Brasileira de Medicina do Esporte 2010 October;16(5):335-8. Off topic
Ref ID: 4375

(3580) Nicolino M, Byrne B, Wraith JE, Leslie N, Mandel H, Freyer DR, Arnold GL, Pivnick EK, Ottinger CJ, Robinson PH, Loo JC, Smitka M, Jardine P, Tato L, Chabrol B, McCandless S, Kimura S, Mehta L, Bali D, Skrinar A, Morgan C, Rangachari L, Corzo D, Kishnani PS. Clinical outcomes after long-term treatment with alglucosidase alfa in infants and children with advanced Pompe disease. Genetics in Medicine 2009 March;11(3):210-9. Off topic
Ref ID: 788

(3581) Niederer I, Kriemler S, Zahner L, Burgi F, Ebenegger V, Hartmann T, Meyer U, Schindler C, Nydegger A, Marques-Vidal P, Puder JJ. Influence of a lifestyle

intervention in preschool children on physiological and psychological parameters (Ballabeina): study design of a cluster randomized controlled trial. BMC Public Health 2009 March 31;9:94.:94. Description versus conduct of study, No exercise only group
Ref ID: 130

(3582) Niederer I, Kriemler S, Gut J, Hartmann T, Schindler C, Barral J, Puder JJ. Relationship of aerobic fitness and motor skills with memory and attention in preschoolers (Ballabeina): a cross-sectional and longitudinal study. BMC Pediatrics 2011;11:34. Cross-sectional study
Ref ID: 2708

(3583) Niederer I, Burgi F, Ebenegger V, Marques-Vidal P, Schindler C, Nydegger A, Kriemler S, Puder JJ. Effects of a lifestyle intervention on adiposity and fitness in overweight or low fit preschoolers (Ballabeina). Obesity (Silver Spring) 2013 March;21(3):E287-E293. Inappropriate Intervention
Ref ID: 5998

(3584) Niemuth PE, Johnson RJ, Myers MJ, Thieman TJ. Hip muscle weakness and overuse injuries in recreational runners. Clinical Journal of Sport Medicine 2005 January;15(1):14-21. Off topic
Ref ID: 1550

(3585) Nienaber C, Pieters M, Kruger SH, Stonehouse W, Vorster HH. Overfatness, stunting and physical inactivity are determinants of plasminogen activator inhibitor-1activity, fibrinogen and thrombin-antithrombin complex in African adolescents. Blood Coagulation Fibrinolysis 2008 July;19(5):361-8. Cross-sectional study
Ref ID: 924

(3586) Nieto R, Dinerstein A, Solana C, Pérez G, Basualdo N, Benítez A, Raviolo R, Balanian N, Brundi M, Larguía AM. Premio Sardá 2003. Nutrición y crecimiento en pacientes prematuros con riesgo de desarrollar enfermedad pulmonar crónica. Revista del Hospital Materno Infantil Ramón Sardá 2004;23(2):75-82. Subjects less than 2 years old
Ref ID: 4376

(3587) Nieves JW, Melsop K, Curtis M, Kelsey JL, Bachrach LK, Greendale G, Sowers MF, Sainani KL. Nutritional factors that influence change in bone density and stress fracture risk among young female cross-country runners. Physical Medicine and Rehabilitation 2010;2(8):740-50. Prospective Study
Ref ID: 472

(3588) Nijs G, Tuyttens FAM, Millet S, Van Oeckel MJ, Warnants N, De Brabander DL, Sonck B. Early and reliable detection of boar taint and its genetic predisposition. Acta Veterinaria Scandinavica 2006 January 2;48:5-2. Animal

study
Ref ID: 3699

(3589) Nik AM, Langmaid S, Wright AJ. Digestibility and beta-carotene release from lipid nanodispersions depend on dispersed phase crystallinity and interfacial properties. Food and Function 2012;3(3):234-45. Off topic
Ref ID: 5509

(3590) Nitsche H, Nitsche M, Sudi K, TschoP M, Zotter H, Weinhandl G, Froehlich-Reiterer E, Gallistl S, Pirker M, Borkenstein M. Ghrelin - An indicator for fat oxidation in obese children and adolescents during a weight reduction program. Journal of Pediatric Endocrinology and Metabolism 2007;20(6):719-23. Diet & Exercise intervention
Ref ID: 5510

(3591) Nixon PA, Washburn LK, Mudd LM, Webb HH, O'Shea TM. Aerobic fitness and physical activity levels of children born prematurely following randomization to postnatal dexamethasone. The Journal of Pediatrics 2011;158:65-70. Follow-up Study
Ref ID: 4936

(3592) Nkansah-Amankra S, Walker AD. The Relation between Adolescent Self Assessment of Health and Risk Behaviours: Could a Global Measure of Health Provide Indications of Health Risk Exposures? Health Education Journal 2012 January 1;71(1):39-52. Cross-sectional study
Ref ID: 3912

(3593) Nnoruka EN. Successful treatment of scabies with oral ivermectin in Nigeria. Tropical Doctor 2001;31:15-8. Off topic
Ref ID: 4937

(3594) Noakes M, Clifton P. The role of diet in cardiovascular health. A review of the evidence. Australian Journal of Nutrition and Dietetics 1999 September 2;56(3):S3-S22. Review article
Ref ID: 3700

(3595) Nobili V, Manco M, Devito R, Ciampalini P, Piemonte F, Marcellini M. Effect of vitamin E on aminotransferase levels and insulin resistance in children with non-alcoholic fatty liver disease. Alimentary Pharmacology and Therapeutics 2006 December;24(11-12):1553-61. Diet Intervention or Supplement Study
Ref ID: 1310

(3596) Nobili V, Manco M, Devito R, Di C, V, Comparcola D, Sartorelli MR, Piemonte F, Marcellini M, Angulo P. Lifestyle intervention and antioxidant therapy in children with nonalcoholic fatty liver disease: a randomized, controlled trial. Hepatology 2008 July;48(1):119-28. Lifestyle Intervention
Ref ID: 921

(3597) Nobili V, Manco M, Ciampalini P, Alisi A, Devito R, Bugianesi E, Marcellini M, Marchesini G. Metformin use in children with nonalcoholic fatty liver disease: an open-label, 24-month, observational pilot study. Clinical Therapeutics 2008 June;30(6):1168-76. No exercise only group
Ref ID: 167

(3598) Nogueira FdAM, Sichieri R. Associação entre consumo de refrigerantes, sucos e leite, com o índice de massa corporal em escolares da rede pública de Niterói, Rio de Janeiro, Brasil. Association between consumption of soft drinks, fruit juice, and milk and body mass index among public school students in Niterói, Rio de Janeiro State, Brazil. Cadernos de Saúde Pública 2009 December;25(12):2715-24. Cross-sectional study
Ref ID: 4377

(3599) Nogueira RC, Weeks BK, Beck BR. Exercise to improve pediatric bone and fat: a systematic review and meta-analysis. Medicine and Science in Sports and Exercise 2014 March;46(3):610-21. Inappropriate Study Design
Ref ID: 5999

(3600) Nordin NA, Leonard JH, Thye NC. Work-related injuries among physiotherapists in public hospitals: a Southeast Asian picture. Clinics 2011;66(3):373-8. Off topic
Ref ID: 4378

(3601) Nordmann H. Low calorie foods, beverages and sweeteners Can they really contribute to a healthier future? (Part 1). Agro Food Industry Hi-Tech 2012;23(1):27-9. Diet Intervention Study
Ref ID: 5511

(3602) Nordstrom A, Neovius MG, Rossner S, Nordstrom P. Postpubertal development of total and abdominal percentage body fat: an 8-year longitudinal study. Obesity 2008 October;16(10):2342-7. Longitudinal Study
Ref ID: 876

(3603) North Carolina Child Advocacy Inst. R, North Carolina State Dept.of Health and Human Services R. North Carolina Child Health Report Card, 2000. 2000 Jan 1. Review article
Ref ID: 3913

(3604) Nova A, Russo A, Sala E. Long-term management of obesity in paediatric office practice: Experimental evaluation of two different types of intervention. Ambulatory Child Health 2001;7:239-47. Behavior Modification Intervention
Ref ID: 4938

(3605) Nova A, Sala Monza E, Bettinardi A, Biolchini A, Bovolato P, Brivio L, Cazzaniga R, Corbetta A, Crespi L, Fasani G, Gussoni C, Lietti G, Limonta M, Meregalli G, Narducci M, Scotti L, Vignati B, Zanetto F. Obesity in Italian

children. Occhio Clinical Pediatrics 2002;6(8):16-8. Prospective Study
Ref ID: 3289

(3606) Novaes JFd, Lamounier JA, Franceschini SdCC, Priore SE. Fatores ambientais associados ao sobrepeso infantil. Revista de Nutrição 2009 October;22(5):661-73. Cross-sectional study
Ref ID: 4379

(3607) Novotny R, Going S, Teegarden D, Van LM, McCabe G, McCabe L, Daida YG, Boushey CJ, ACT Research Team. Hispanic and Asian pubertal girls have higher android/gynoid fat ratio than whites. Obesity 2007 June;15(6):1565-70. Cross-sectional study
Ref ID: 1219

(3608) Nowaczyk MJM, Whelan DT, Heshka TW, Hill RE. Smith-Lemli-Opitz syndrome: a treatable inherited error of metabolism causing mental retardation. Canadian Medical Association Journal 1999 July 27;161(2):165-70. Off topic
Ref ID: 3701

(3609) Nowicka P, Hoglund P, Pietrobelli A, Lissau I, Flodmark CE. Family Weight School treatment: 1-year results in obese adolescents. International Journal of Pediatric Obesity 2008;3(3):141-7. Lifestyle Intervention
Ref ID: 841

(3610) Nowicka P, Lanke J, Pietrobelli A, Apitzsch E, Flodmark CE. Sports camp with six months of support from a local sports club as a treatment for childhood obesity. Scandinavian Journal of Public Health 2009 November;37(8):793-800. Not a randomized controlled trial (RCT)
Ref ID: 655

(3611) Nunes APdOB, Rios ACdS, Cunha GAd, Barretto ACP, Negrão CE. Efeitos de um programa de exercício físico não-supervisionado e acompanhado a distância, via internet, sobre a pressão arterial e composição corporal em indivíduos normotensos e pré-hipertensos. Arquivos Brasileiros de Cardiologia 2006 April;86(4):289-96. Study limited to adults
Ref ID: 4380

(3612) Nunes MdO, Rubira MC, Rubira APFDA, Nascimento ACPd, Paula Júnior ARd, Osório RAL. Variabilidade da frequência cardíaca em mulheres com hipermobilidade articular. Heart rate variability in women with joint hipermobility. Fisioterapia e Pesquisa 2011 September;18(3):241-6. Off topic
Ref ID: 4381

(3613) Nunes MMdA, Figueiroa JN, Alves JGB. Excesso de peso, atividade física e hábitos alimentares entre adolescentes de diferentes classes econômicas em Campina Grande (PB). Revista da Associação Médica Brasileira (1992) 2007;53(2):130-4. Cross-sectional study
Ref ID: 4382

(3614) Nurse J, Basher D, Bone A, Bird W. An ecological approach to promoting population mental health and well-being - A response to the challenge of climate change. Perspectives in Public Health 2010;130(1):27-33. Off topic
Ref ID: 5512

(3615) Nuutinen O, Knip M. Predictors of weight reduction in obese children. European Journal of Clinical Nutrition 1992 November;46(11):785-94. CT
Ref ID: 2280

(3616) Núñez-Rivas HP, Monge-Rojas R, Gríos-Dávila C, Elizondo-Ureña AM, Rojas-Chavarría A. La violencia física, psicológica, emocional y sexual durante el embarazo: riesgo reproductivo predictor de bajo peso al nacer en Costa Rica. Revista Panamericana de Salud Pública 2003 August;14(2):75-83. Survey or questionnaire
Ref ID: 4383

(3617) Nybacka A, Carlstrom K, Fabri F, Hellstrom PM, Hirschberg AL. Serum antimullerian hormone in response to dietary management and/or physical exercise in overweight/obese women with polycystic ovary syndrome: secondary analysis of a randomized controlled trial. Fertility and Sterility 2013 October;100(4):1096-102. Inappropriate Population
Ref ID: 6000

(3618) Nyberg G, Sundblom E, Norman A, Elinder LS. A healthy school start - parental support to promote healthy dietary habits and physical activity in children: design and evaluation of a cluster-randomised intervention. BMC Public Health 2011;11:185. Description versus conduct of study
Ref ID: 2709

(3619) O'Brien A, McDonald J, Haines J. An approach to improve parent participation in a childhood obesity prevention program. Canadian Journal of Dietetic Practice and Research 2013;74(3):143-5. Inappropriate Population
Ref ID: 6001

(3620) O'Brien K, Cokkinides V, Jemal A, Cardinez CJ, Murray T, Samuels A, Ward E, Thun MJ. Cancer statistics for Hispanics, 2003. Ca-A Cancer Journal for Clinicians 2003;53(4):208-26. Off topic
Ref ID: 5513

(3621) O'Brien K, Tynan AM, Nixon S, Glazier RH. Effects of progressive resistive exercise in adults living with HIV/AIDS: systematic review and meta-analysis of randomized trials. [Review] [28 refs]. AIDS Care 2008 July;20(6):631-53. Review article
Ref ID: 931

(3622) O'Brien KS, Puhl RM, Latner JD, Mir AS, Hunter JA. Reducing anti-fat prejudice in preservice health students: a randomized trial. Obesity 2010

November;18(11):2138-44. Educational intervention
Ref ID: 2710

(3623) O'Brien M, Nader PR, Houts RM, Bradley R, Friedman SL, Belsky J, Susman E. The ecology of childhood overweight: a 12-year longitudinal analysis. International Journal of Obesity 2007 September;31(9):1469-78. Longitudinal Study
Ref ID: 1177

(3624) O'Connor DM, Crowe MJ. Effects of beta-hydroxy-beta-methylbutyrate and creatine monohydrate supplementation on the aerobic and anaerobic capacity of highly trained athletes. Journal of Sports Medicine and Physical Fitness 2003 March;43(1):64-8. Off topic
Ref ID: 1765

(3625) O'Connor DM, Crowe MJ. Effects of six weeks of beta-hydroxy-beta-methylbutyrate (HMB) and HMB/creatine supplementation on strength, power, and anthropometry of highly trained athletes. Journal of Strength and Conditioning Research 2007 May;21(2):419-23. Off topic
Ref ID: 1228

(3626) O'Connor TM, Hilmers A, Watson K, Baranowski T, Giardino AP. Feasibility of an obesity intervention for paediatric primary care targeting parenting and children: Helping HAND. Child: Care, Health and Development 2013 January;39(1):141-9. Inappropriate Intervention
Ref ID: 6002

(3627) O'Dea JA, Abraham S. Improving the body image, eating attitudes, and behaviors of young male and female adolescents: a new educational approach that focuses on self-esteem. International Journal of Eating Disorders 2000;28:43-57. Educational intervention
Ref ID: 844

(3628) O'Dea JA, Nguyen Hoang TD, Dibley MJ. Plateau in obesity and overweight in a cross sectional study of low, middle and high socioeconomic status schoolchildren between 2004 and 2009. International Journal of Public Health 2011 December;56(6):663-7. Cross-sectional study
Ref ID: 2711

(3629) O'Dell KK, Morse AN, Crawford SL, Howard A. Vaginal pressure during lifting, floor exercises, jogging, and use of hydraulic exercise machines. International Urogynecology Journal 2007 December;18(12):1481-9. Off topic
Ref ID: 1145

(3630) O'Donohue W, Ferguson KE. Evidence-Based Practice in Psychology and Behavior Analysis. Behavior Analyst Today 2006 July;7(3):335-50. Review article
Ref ID: 3848

(3631) O'Dougherty M, Dallman A, Turcotte L, Patterson J, Napolitano MA, Schmitz KH. Barriers and motivators for strength training among women of color and Caucasian women. Women and Health 2008;47(2):41-62. Study limited to adults
Ref ID: 904

(3632) O'Dougherty M, Kurzer MS, Schmitz KH. Shifting motivations: Young women's reflections on physical activity over time and across contexts. Health Education and Behavior 2010 August;37(4):547-67. Study limited to adults
Ref ID: 475

(3633) O'Malley G, Clarke M, Burls A, Murphy S, Murphy N, Perry IJ. A smartphone intervention for adolescent obesity: study protocol for a randomised controlled non-inferiority trial. Trials 2014;15:43. Inappropriate Study Design
Ref ID: 6003

(3634) O'Malley SP. The Australian experiment: the use of evidence based medicine for the reimbursement of surgical and diagnostic procedures (1998-2004). Australia and New Zealand Health Policy (ANZHP) 2006 January;3:3-23. Off topic
Ref ID: 3702

(3635) O'Mara AJ, Marsh HW, Craven RG, Debus RL. Do Self-Concept Interventions Make a Difference? A Synergistic Blend of Construct Validation and Meta-Analysis. Educational Psychologist 2006;41(3):181-206. Review article
Ref ID: 3849

(3636) O'Nunain S, Ruskin J. Cardiac arrest. Lancet 1993 June 26;341(8861):1641. Off topic
Ref ID: 3703

(3637) O'Shea TM, Kothadia JM, Klinepeter KL, Goldstein DJ, Jackson BG, Weaver RG, Dillard RG. Randomized placebo-controlled trial of a 42-day tapering course of dexamethasone to reduce the duration of ventilator dependency in very low birth weight infants: outcome of study participants at 1-year adjusted age. Pediatrics 1999;104:15-21. Subjects less than 2 years old
Ref ID: 891

(3638) Obeid J, Larche MJ, Timmons BW. Optimizing the Wingate Anaerobic Cycling Test for youth with juvenile idiopathic arthritis. Pediatric Exercise Science 2011 August;23(3):303-10. Off topic
Ref ID: 2712

(3639) Oblacinska A, Wojciechowska A, Wroclawska M. [Preliminary evaluation of health behavior in obese students from a selected group of Warsaw adolescents]. [Polish]. Medycyna Wieku Rozwojowego 1999 April;3(2):303-13. Survey or questionnaire
Ref ID: 2053

(3640) Obregon AM, Diaz E, Santos JL. Effect of the melanocortin-3 receptor Thr6Lys and Val81Ile genetic variants on body composition and substrate oxidation in Chilean obese children. Journal of Physiology and Biochemistry 2012;68(1):71-6. Cross-sectional study
Ref ID: 5514

(3641) Ocampo CE, Pradilla A, Méndez F. Impacto de un depósito de residuos sólidos en el crecimiento físico infantil. Colombia Médica 2008 September;39(3):253-9. Off topic
Ref ID: 4384

(3642) Ochoa MC, Azcona C, Biebermann H, Brumm H, Razquin C, Wermter AK, Martinez JA, Hebebrand J, Hinney A, Moreno-Aliaga MJ, Marti A. A novel mutation Thr162Arg of the melanocortin 4 receptor gene in a Spanish children and adolescent population. Clinical Endocrinology 2007;66(5):652-8. Cross-sectional study
Ref ID: 5516

(3643) Ochoa MC, Santos JL, Azcona C, Moreno-Aliaga MJ, Martinez-Gonzalez MA, Martinez JA, Marti A. Association between obesity and insulin resistance with UCP2-UCP3 gene variants in Spanish children and adolescents. Molecular Genetics and Metabolism 2007;92(4):351-8. Case-Control / Case Study
Ref ID: 5515

(3644) Oganov RG, Savel'ev VS, Shal'nova SA, Kirienko AI, Zolotukhin IA. [Risk factors of chronic venous insufficiency of the lower extremities and possibilities of its medication in therapeutic practice]. [Russian]. Terapevticheskii Arkhiv 2006;78(4):68-72. Off topic
Ref ID: 1377

(3645) Oh H, Taylor AH. A brisk walk, compared with being sedentary, reduces attentional bias and chocolate cravings among regular chocolate eaters with different body mass. Appetite 2013 December;71:144-9. Inappropriate Population
Ref ID: 6004

(3646) Ohkawara K, Tanaka S, Miyachi M, Ishikawa-Takata K, Tabata I. A dose-response relation between aerobic exercise and visceral fat reduction: systematic review of clinical trials. [Review] [50 refs][Erratum appears in International Journal of Obesity (London). 2008 Feb;32(2):395]. International Journal of Obesity 2007 December;31(12):1786-97. Review article
Ref ID: 1138

(3647) Ohkawara K, Cornier MA, Kohrt WM, Melanson EL. Effects of increased meal frequency on fat oxidation and perceived hunger. Obesity (Silver Spring) 2013 February;21(2):336-43. Inappropriate Intervention
Ref ID: 6005

(3648) Ohms VR, Escudero P, Lammers K, ten Cate C. Zebra finches and Dutch adults exhibit the same cue weighting bias in vowel perception. Animal Cognition 2012;15(2):155-61. Off topic
Ref ID: 5517

(3649) Ohrig E, Geiss HC, Haas GM, Schwandt P. The Prevention Education Program (PEP) Nuremberg: design and baseline data of a family oriented intervention study. International Journal of Obesity and Related Metabolic Disorders 2001;25 Suppl 1:S89-S92. Prevalence study
Ref ID: 4939

(3650) Ohuchi H, Ohashi H, Park J, Hayashi J, Miyazaki A, Echigo S. Abnormal postexercise cardiovascular recovery and its determinants in patients after right ventricular outflow tract reconstruction. Circulation 2002;106(22):2819-26. Off topic
Ref ID: 5518

(3651) Okano G, Takeda H, Morita I, Katoh M, Mu Z, Miyake S. Effect of pre-exercise fructose ingestion on endurance performance in fed men. Medicine and Science in Sports and Exercise 1988 April;20(2):105-9. Diet Intervention or Supplement Study
Ref ID: 2713

(3652) Okazaki K, Hayase H, Ichinose T, Mitono H, Doi T, Nose H. Protein and carbohydrate supplementation after exercise increases plasma volume and albumin content in older and young men. Journal of Applied Physiology 2009 September;107(3):770-9. Diet Intervention or Supplement Study
Ref ID: 685

(3653) Okely AD, Collins CE, Morgan PJ, Jones RA, Warren JM, Cliff DP, Burrows TL, Colyvas K, Steele JR, Baur LA. Multi-site randomized controlled trial of a child-centered physical activity program, a parent-centered dietary-modification program, or both in overweight children: the HIKCUPS study. Journal of Pediatrics 2010 September;157(3):388-94, 394. No comparative control group
Ref ID: 45

(3654) Oken E, Ning Y, Rifas-Shiman SL, Radesky JS, Rich-Edwards JW, Gillman MW. Associations of physical activity and inactivity before and during pregnancy with glucose tolerance. Obstetrics and Gynecology 2006 November;108(5):1200-7. Cohort Study
Ref ID: 1335

(3655) Okudan N, Gokbel H. The effects of creatine supplementation on performance during the repeated bouts of supramaximal exercise. Journal of Sports Medicine and Physical Fitness 2005 December;45(4):507-11. Diet Intervention or Supplement Study
Ref ID: 1436

(3656) Okuro RT, Morcillo AM, Sakano E, Schivinski CIS, Ribeiro MÂGO, Ribeiro JD. Exercise capacity, respiratory mechanics and posture in mouth breathers. Capacidade ao exercício, mecânica respiratória e postura em respiradores bucais. Brazilian Journal of Otorhinolaryngology 2011 October;77(5):656-62. Off topic
Ref ID: 4385

(3657) Okuro RT, Morcillo AM, Ribeiro MÂGO, Sakano E, Conti PBM, Ribeiro JD. Respiração bucal e anteriorização da cabeça: Efeitos na biomecânica respiratória e na capacidade de exercício em crianças. Mouth breathing and forward head posture: Effects on respiratory biomechanics and exercise capacity in children. Jornal Brasileiro de Pneumologia 2011 August;37(4):471-9. Off topic
Ref ID: 4386

(3658) Oldham-Cooper RE, Hardman CA, Nicoll CE, Rogers PJ, Brunstrom JM. Playing a computer game during lunch affects fullness, memory for lunch, and later snack intake. American Journal of Clinical Nutrition 2011 February;93(2):308-13. Not an exercise intervention study
Ref ID: 5

(3659) Olds DL. Home Visitation for Pregnant-Women and Parents of Young-Children. American Journal of Diseases of Children 1992;146(6):704-8. Off topic
Ref ID: 5519

(3660) Olds T, Tomkinson G, Leger L, Cazorla G. Worldwide variation in the performance of children and adolescents: an analysis of 109 studies of the 20-m shuttle run test in 37 countries. Journal of Sports Sciences 2006 October;24(10):1025-38. Review article
Ref ID: 1325

(3661) Olesen SC, Butterworth P, Jacomb P, Tait RJ. Personal factors influence use of cervical cancer screening services: epidemiological survey and linked administrative data address the limitations of previous research. BMC Health Services Research 2012;12. Off topic
Ref ID: 5520

(3662) Oliva RR, Tous RM, Gil BB, Longo AG, Pereira Cunill JL, Garcia Luna PP. [Impact of a brief educational intervention about nutrition and healthy lifestyles to school students given by a healthcare provider]. Nutricion Hospitalaria 2013 September;28(5):1567-73. Inappropriate Intervention
Ref ID: 6006

(3663) Olivares C, Albala Brevis C, García B, Jofré C. Publicidad televisiva y preferencias alimentarias en escolares de la Región Metropolitana. Revista médica de Chile 1999 July;127(7):791-9. Survey or questionnaire
Ref ID: 4387

(3664) Olivares C, Bustos Z, Moreno H, Lera M, Cortez F. Actitudes y practicas sobre alimentacion y actividad física en niños obesos y sus madres en santiago, chile. Revista Chilena de Nutrición 2006 August;33(2):170-9. Survey or questionnaire
Ref ID: 656

(3665) Olivares C, Bustos Z, Lera M, Zelada ME. Estado nutricional, consumo de alimentos y actividad física en escolares mujeres de diferente nivel socioeconómico de Santiago de Chile. Revista Médica de Chile 2007 January;135(1):71-8. Cross-sectional study
Ref ID: 4388

(3666) Olivares S, Morón C, Kain J, Zacarías I, Andrade M, Lera L, Díaz N, Vio F. Propuesta metodológica para incorporar la educación en nutrición en la enseñanza básica: la experiencia de Chile. Archivos Latinoamericanos de Nutrición 2004 June;54(supl.1):33-9. Diet Intervention or Supplement Study
Ref ID: 4389

(3667) Oliveira EO, Velásquez-Meléndez G, Kac G. Fatores demográficos e comportamentais associados à obesidade abdominal em usuárias de centro de saúde de Belo Horizonte, Minas Gerais, Brasil. Revista de Nutrição 2007 August;20(4):361-9. Cross-sectional study
Ref ID: 4390

(3668) Oliveira ERNd, Lopes AdS. Avaliação da composição corporal, hábitos alimentares e prática de atividade física em alunos da 8a série do ensino fundamental da cidade de Maringá. Arquivos de Ciências da Saúde da UNIPAR 2001 December;5(3):239-47. Cross-sectional study
Ref ID: 4391

(3669) Oliveira EdAM, Anjos LAd. Medidas antropométricas segundo aptidão cardiorrespiratória em militares da ativa, Brasil. Revista de Saúde Pública 2008 April;42(2):217-23. Study limited to adults
Ref ID: 4392

(3670) Oliveira EFVd, Gama SGNd, Silva CMFPd. Gravidez na adolescência e outros fatores de risco para mortalidade fetal e infantil no Município do Rio de Janeiro, Brasil. Teenage pregnancy and other risk factors for fetal and infant mortality in the city of Rio de Janeiro, Brazil. Cadernos de Saúde Pública 2010 March;26(3):567-78. Off topic
Ref ID: 4393

(3671) Oliveira FPd, Bosi MLM, Vigário PdS, Vieira RdS. Comportamento alimentar e imagem corporal em atletas. Eating behavior and body image in athletes. Revista Brasileira de Medicina do Esporte 2003 December;9(6):348-64. Off topic
Ref ID: 4394

(3672) Oliveira GFd, Oliveira TRRd, Rodrigues FF, Corrêa LF, Ikejiri AT, Casulari LA. Prevalência de diabetes melito e tolerância à glicose diminuída nos indígenas da Aldeia Jaguapiru, Brasil. Prevalence of diabetes mellitus and impaired glucose tolerance in indigenous people from Aldeia Jaguapiru, Brazil. Revista Panamericana de Salud Pública 2011 May;29(5):315-21. Prevalence study
Ref ID: 4395

(3673) Oliveira GSd, Tenório SB, Cumino DO, Gomes DBG, Namba EN, Maidana JL, Rocha LEMd. Hemodiluição normovolêmica aguda em crianças submetidas a artrodese de coluna vertebral pela via posterior. Acute normovolemic hemodilution in children submitted to posterior spinal fusion. Revista Brasileira de Anestesiologia 2004 February;54(1):84-90. Off topic
Ref ID: 712

(3674) Oliveira KdJFd, Koury JC, Donangelo CM. Micronutrientes e capacidade antioxidante em adolescentes sedentários e corredores. Revista de Nutrição 2007 April;20(2):171-9. Not All Participants were Overweight and/or Obese
Ref ID: 4396

(3675) Oliveira NKRd, Lima RAd, Mélo EN, Santos CM, Barros SSH, Barros MVGd. Reprodutibilidade de questionário para medida da atividade física e comportamento sedentário em crianças pré-escolares. Reliability of a questionnaire to assess physical activity and sedentary behavior in preschool-aged children. Revista Brasileira de Atividade Física e Saúde 2011 July;16(3). Off topic
Ref ID: 4397

(3676) Oliveira TRPRd, Cunha CdF, Ferreira RA. Características de adolescentes atendidos em ambulatório de obesidade: conhecer para intervir. Characteristics of adolescents assisted in obesity outpatient service: know them to intervene. Nutrire Revista da Sociedade Brasileira de Alimentação e Nutrição 2010 August;35(2). Cross-sectional study
Ref ID: 4398

(3677) Oliveira TCd, Silva AAMd, Santos CdJNd, Silva JS, Conceição SIOd. Atividade física e sedentarismo em escolares da rede pública e privada de ensino em São Luís. Physical activity and sedentary lifestyle among children from private and public schools in Northern Brazil. Actividad física y sedentarismo en escolares de la red pública y privada de enseñanza en Sao Luis, Norte de Brasil. Revista de Saúde Pública 2010 December;44(6):996-1004. Cross-sectional study
Ref ID: 4399

(3678) Oliver GD, Plummer H. Ground reaction forces, kinematics, and muscle activations during the windmill softball pitch. Journal of Sports Sciences 2011 July;29(10):1071-7. Off topic
Ref ID: 2714

(3679) Olmedilla Zafra A, Ortega Toro E. Incidencia de la práctica de actividad física sobre la ansiedad y depresión en mujeres: Perfiles de riesgo. Effect of physical activity on anxiety and depression among women: Risk profiles. Universitas Psychologica 2009 April;8(1):105-16. Off topic
Ref ID: 4400

(3680) Olmo Palma I. El recién nacido con bajo peso: preparación a las madres para su atención en el hogar. Revista Cubana de Enfermería 1992 June;8(1):19-26. Off topic
Ref ID: 4401

(3681) Olmstead MC, Martin A, Brien JF, Reynolds JN. Chronic prenatal ethanol exposure increases disinhibition and perseverative responding in the adult guinea pig. Behavioural Pharmacology 2009;20(5-6):554-7. Animal study
Ref ID: 5521

(3682) Olney DK, Pollitt E, Kariger PK, Khalfan SS, Ali NS, Tielsch JM, Sazawal S, Black R, Allen LH, Stoltzfus RJ. Combined iron and folic acid supplementation with or without zinc reduces time to walking unassisted among Zanzibari infants 5- to 11-mo old. Journal of Nutrition 2006;136:2427-34. Subjects less than 2 years old
Ref ID: 4941

(3683) Olsen NJ, Buch-Andersen T, Handel MN, Ostergaard LM, Pedersen J, Seeger C, Stougaard M, Traerup M, Livemore K, Mortensen EL, Holst C, Heitmann BL. The Healthy Start project: a randomized, controlled intervention to prevent overweight among normal weight, preschool children at high risk of future overweight. BMC Public Health 2012;12:590. Inappropriate Population
Ref ID: 6007

(3684) Olszanecka-Glinianowicz M, Zahorska-Markiewicz B, Kocelak P, Janowska J, Holecki M, Semik-Grabarczyk E. [The effect of weight loss on serum concentration of interleukine-6 (IL-6) and insulin resistance]. [Polish]. Endokrynologia Polska 2006 March;57(2):131-5. Study limited to adults
Ref ID: 1384

(3685) Olvera N, McCarley KE, Leung P, McLeod J, Rodriguez AX. Assessing physical activity preferences in Latino and white preadolescents. Pediatric Exercise Science 2009 November;21(4):400-12. Cross-sectional study
Ref ID: 615

(3686) Olvera N, Scherer R, McLeod J, Graham M, Knox B, Hall K, Butte NF, Bush JA, Smith DW, Bloom J. BOUNCE: an exploratory healthy lifestyle summer intervention for girls. American Journal of Health Behavior 2010 March;34(2):144-55. No exercise only group
Ref ID: 609

(3687) Olvera N, Bush JA, Sharma SV, Knox BB, Scherer RL, Butte NF. BOUNCE: a community-based mother-daughter healthy lifestyle intervention for low-income Latino families. Obesity (Silver Spring) 2010 February;18 Suppl 1:S102-4.:S102-S104. No exercise only group
Ref ID: 67

(3688) Omasu F, Kitagawa J, Koyama K, Asakawa K, Yokouchi J, Ando D, Nakahara Y. The influence of VDR genotype and exercise on ultrasound parameters in young adult Japanese women. Journal of Physiological Anthropology and Applied Human Science 2004 March;23(2):49-55. Study limited to adults
Ref ID: 1650

(3689) Onnerfalt J, Erlandsson LK, Orban K, Broberg M, Helgason C, Thorngren-Jerneck K. A family-based intervention targeting parents of preschool children with overweight and obesity: conceptual framework and study design of L. BMC Public Health 2012;12:879. Inappropriate Study Design
Ref ID: 6008

(3690) Oostdam N, van-Poppel MN, Eekhoff EM, Wouters MG, van MW. Design of FitFor2 study: the effects of an exercise program on insulin sensitivity and plasma glucose levels in pregnant women at high risk for gestational diabetes. BMC Pregnancy and Childbirth 2009;9:1. Study limited to adults
Ref ID: 4942

(3691) Oosting A, van VN, Kegler D, Schipper L, Abrahamse-Berkeveld M, Ringler S, Verkade HJ, van der Beek EM. Effect of dietary lipid structure in early postnatal life on mouse adipose tissue development and function in adulthood. British Journal of Nutrition 2014 January 28;111(2):215-26. Inappropriate Population
Ref ID: 6009

(3692) Ordonez FJ, Fornieles-Gonzalez G, Camacho A, Rosety MA, Rosety I, Diaz AJ, Rosety-Rodriguez M. Anti-inflammatory effect of exercise, via reduced leptin levels, in obese women with Down syndrome. International Journal of Sport Nutrition and Exercise Metabolism 2013 June;23(3):239-44. Inappropriate Population
Ref ID: 6010

(3693) Orenstein DM, Hovell MF, Mulvihill M, Keating KK, Hofstetter CR, Kelsey S, Morris K, Nixon PA. Strength vs aerobic training in children with cystic fibrosis: a randomized controlled trial. Chest 2004 October;126(4):1204-14. Not All Participants were Overweight and/or Obese
Ref ID: 1581

(3694) Orenstein SR, Gremse DA, Pantaleon CD, Kling DF, Rotenberg KS. Nizatidine for the treatment of pediatric gastroesophageal reflux symptoms: an open-label, multiple-dose, randomized, multicenter clinical trial in 210 children. Clinical

Therapeutics 2005;27:472-83. Drug intervention study
Ref ID: 4943

(3695) Oriá RB, Patrick PD, Oriá MOB, Lorntz B, Thompson MR, Azevedo OGR, Lobo RNB, Pinkerton RF, Guerrant RL, Lima AAM. ApoE polymorphisms and diarrheal outcomes in Brazilian shanty town children. Brazilian Journal of Medical and Biological Research 2010 March;43(3):249-56. Off topic
Ref ID: 4402

(3696) Orsano VSM, Tibana RA, Prestes J. Relação da avaliação funcional com indicadores antropométricos de obesidade em adolescentes de Teresina, PI. Revista Brasileira de Cardiologia 2012 April;25(2):111-7. Cross-sectional study
Ref ID: 4403

(3697) Ortega FB, Tresaco B, Ruiz JR, Moreno LA, Martin-Matillas M, Mesa JL, Warnberg J, Bueno M, Tercedor P, Gutierrez A, Castillo MJ, Avena Study Group. Cardiorespiratory fitness and sedentary activities are associated with adiposity in adolescents. Obesity 2007 June;15(6):1589-99. Cross-sectional study
Ref ID: 1218

(3698) Ortega FB, Artero EG, Ruiz JR, Vicente-Rodriguez G, Bergman P, Hagstromer M, Ottevaere C, Nagy E, Konsta O, Rey-Lopez JP, Polito A, Dietrich S, Plada M, Beghin L, Manios Y, Sjostrom M, Castillo MJ, HELENA Study Group. Reliability of health-related physical fitness tests in European adolescents. The HELENA Study. International Journal of Obesity 2008 November;32:Suppl-57. Off topic
Ref ID: 857

(3699) Ortega FB, Artero EG, Ruiz JR, Espana-Romero V, Jimenez-Pavon D, Vicente-Rodriguez G, Moreno LA, Manios Y, Beghin L, Ottevaere C, Ciarapica D, Sarri K, Dietrich S, Blair SN, Kersting M, Molnar D, Gonzalez-Gross M, Gutierrez A, Sjostrom M, Castillo MJ, HELENA s. Physical fitness levels among European adolescents: the HELENA study. British Journal of Sports Medicine 2011 January;45(1):20-9. Cross-sectional study
Ref ID: 2715

(3700) Ortega L, Blain Llanes E, Medina A, Rodríguez T, Gaanam Z, Romano C. Tricoberozoares gástrico como hallazgo incidental. GEN 2003 June;57(2):118-9. Case-Control / Case Study
Ref ID: 4404

(3701) Ortega P, Leal Montiel JY, Amaya D, Chávez CJ. Anemia y depleción de las reservas de hierro en adolescentes de sexo femenino no embarazadas. Revista Chilena de Nutrición 2009 June;36(2):111-9. Off topic
Ref ID: 4405

(3702) Ortiz Silva O, Felipe Huarte I, Alonso Cordero ME, Alvarez Rodríguez A, Barrios Rodríguez JC. Influencia del bajo peso al nacer en el estado de salud durante el primer año. Revista Cubana de Pediatria 1997 August;69(2):145-50. Retrospective study
Ref ID: 4406

(3703) Orwoll ES, Bauer DC, Vogt TM, Fox KM. Axial bone mass in older women. Study of Osteoporotic Fractures Research Group. Annals of Internal Medicine 1996 January 15;124(2):187-96. Off topic
Ref ID: 2184

(3704) Osganian SK, Ebzery MK, Montgomery DH, Nicklas TA, Evans MA, Mitchell PD, Lytle LA, Snyder MP, Stone EJ, Zive MM, Bachman KJ, Rice R, Parcel GS. Changes in the nutrient content of school lunches: results from the CATCH Eat Smart Food service Intervention. Preventive Medicine 1996 July;25(4):400-12. Diet Intervention Study
Ref ID: 2167

(3705) Ost LG, Gotestam KG. Behavioral and pharmacological treatments for obesity: an experimental comparison. Addictive Behaviors 1976;1(4):331-8. Behavior Modification Intervention
Ref ID: 2387

(3706) Ostbye T, Krause KM, Brouwer RJ, Lovelady CA, Morey MC, Bastian LA, Peterson BL, Swamy GK, Chowdhary J, McBride CM. Active Mothers Postpartum (AMP): rationale, design, and baseline characteristics. Journal of Women's Health 2008 December;17(10):1567-75. Off topic
Ref ID: 849

(3707) Ostbye T, Zucker NL, Krause KM, Lovelady CA, Evenson KR, Peterson BL, Bastian LA, Swamy GK, West DG, Brouwer RJ. Kids and adults now! Defeat Obesity (KAN-DO): rationale, design and baseline characteristics. Contemporary Clinical Trials 2011 May;32(3):461-9. Description versus conduct of study
Ref ID: 1048

(3708) Ostbye T, Krause KM, Stroo M, Lovelady CA, Evenson KR, Peterson BL, Bastian LA, Swamy GK, West DG, Brouwer RJ, Zucker NL. Parent-focused change to prevent obesity in preschoolers: results from the KAN-DO study. Preventive Medicine 2012 September;55(3):188-95. Inappropriate Intervention
Ref ID: 6011

(3709) Ostbye T, Peterson BL, Krause KM, Swamy GK, Lovelady CA. Predictors of postpartum weight change among overweight and obese women: results from the Active Mothers Postpartum study. Journal of Women's Health 2012 February;21(2):215-22. Study limited to adults
Ref ID: 2717

(3710) Osteyee JL, Banner W. Effects of two dosing regimens of intravenous ranitidine on gastric pH in critically ill children. American Journal of Critical Care 1994;3:267-72. Drug intervention study
Ref ID: 1017

(3711) Ostlund G, Wahlin A, Sunnerhagen KS, Borg K. Vitality among Swedish patients with post-polio: a physiological phenomenon. Journal of Rehabilitation Medicine 2008 October;40(9):709-14. Cross-sectional study
Ref ID: 881

(3712) Ostrom NK, Parsons JP, Eid NS, Craig TJ, Stoloff S, Hayden ML, Colice GL. Exercise-induced bronchospasm, asthma control, and obesity. Allergy and Asthma Proceedings 2013 July;34(4):342-8. Inappropriate Study Design
Ref ID: 6012

(3713) Osunkwo I. An update on the recent literature on sickle cell bone disease. Current Opinion in Endocrinology, Diabetes and Obesity 2013 December;20(6):539-46. Inappropriate Outcomes
Ref ID: 6013

(3714) Ouedraogo HZ, Dramaix-Wilmet Ml, Zeba AN, Hennart P, Donnen P. Effect of iron or multiple micronutrient supplements on the prevalence of anaemia among anaemic young children of a malaria-endemic area: a randomized double-blind trial. Tropical Medicine and International Health 2008 October;13(10):1257-66. Off topic
Ref ID: 3704

(3715) Oude LH, Baur L, Jansen H, Shrewsbury VA, O'Malley C, Stolk RP, Summerbell CD. Interventions for treating obesity in children. [Review] [273 refs][Update of Cochrane Database of Systematic Reviews. 2003;(3):CD001872; PMID: 12917914]. Cochrane Database of Systematic Reviews (1):CD001872, 2009 2009;(1):CD001872. Review article
Ref ID: 808

(3716) Oudiz RJ, Barst RJ, Hansen JE, Sun XG, Garofano R, Wu X, Wasserman K. Cardiopulmonary exercise testing and six-minute walk correlations in pulmonary arterial hypertension. American Journal of Cardiology 2006 January 1;97(1):123-6. Drug intervention study
Ref ID: 1431

(3717) Ouma GO, Jonas RA, Usman MH, Mohler ER. Targets and delivery methods for therapeutic angiogenesis in peripheral artery disease. Vascular Medicine 2012 August;17(3):174-92. Review article
Ref ID: 3705

(3718) Owens S, Gutin B, Allison J, Riggs S, Ferguson M, Litaker M, Thompson W. Effect of physical training on total and visceral fat in obese children. Medicine and Science in Sports and Exercise 1999 January;31(1):143-8. Primary

outcome(s) not assessed
Ref ID: 417

(3719) Owens SG, Garner JC, III, Loftin JM, van BN, Ermin K. Changes in physical activity and fitness after 3 months of home Wii FitTM use. Journal of Strength and Conditioning Research 2011 November;25(11):3191-7. No control group (NC)
Ref ID: 2718

(3720) Oyeyemi AL, Ishaku CM, Deforche B, Oyeyemi AY, De B, I, Van DD. Perception of built environmental factors and physical activity among adolescents in Nigeria. International Journal of Behavioral Nutrition and Physical Activity 2014;11:56. Inappropriate Study Design
Ref ID: 6014

(3721) Ozener B, Duyar I. The effect of labour on somatotype of males during the adolescent growth period. Homo-Journal of Comparative Human Biology 2008;59(2):161-72. Off topic
Ref ID: 5522

(3722) Ozkan B, Bereket A, Turan S, Keskin S. Addition of orlistat to conventional treatment in adolescents with severe obesity. European Journal of Pediatrics 2004 December;163(12):738-41. No exercise only group, Drug intervention study
Ref ID: 325

(3723) Ozmen T, Ryildirim NU, Yuktasir B, Beets MW. Effects of school-based cardiovascular-fitness training in children with mental retardation. Pediatric Exercise Science 2007 May;19(2):171-8. Not All Participants were Overweight and/or Obese
Ref ID: 1205

(3724) Ozmun JC, Mikesky AE, Surburg PR. Neuromuscular adaptations following prepubescent strength training. Medicine and Science in Sports and Exercise 1994;26:510-4. Not All Participants were Overweight and/or Obese
Ref ID: 1026

(3725) Ozores Suárez FJ. Ecocardiografía transesofágica en pacientes pediátricos. Revista Cubana de Pediatria 2007 December;79(4). Off topic
Ref ID: 4407

(3726) Østbye T, Krause KM, Brouwer RJ, Lovelady CA, Morey MC, Bastian LA, Peterson BL, Swamy GK, Chowdhary J, McBride CM. Active Mothers Postpartum (AMP): rationale, design, and baseline characteristics. Journal of Women's Health 2008;17:1567-75. Off topic
Ref ID: 4944

(3727) Padilha PdC, Rocha HFd, Alves N, Peres WA. Prevalência de doença hepática não-alcoólica em crianças e adolescentes obesos: uma revisão sistemática. Prevalence of nonalcoholic fatty liver disease in obese children and adolescents: a systematic review. Revista Paulista de Pediatria 2010 December;28(4):387-93. Review article
Ref ID: 4408

(3728) Padilla Vargas G, Roselló Araya M, Guzmán Padilla S, Aráuz Hernández A, Solórzano Alfaro N. Percepción del peso corporal y estrategias utilizadas para controlarlo, en adultos costarricenses. Revista Costarricense de Salud Pública 2007 December;16(31):48-54. Cross-sectional study
Ref ID: 4409

(3729) Paez S, Maloney A, Kelsey K, Wiesen C, Rosenberg A. Parental and environmental factors associated with physical activity among children participating in an active video game. Pediatric Physical Therapy 2009;21(3):245-53. Primary outcome(s) not assessed
Ref ID: 100

(3730) Page A, Cooper AR, Stamatakis E, Foster LJ, Crowne EC, Sabin M, Shield JP. Physical activity patterns in nonobese and obese children assessed using minute-by-minute accelerometry. International Journal of Obesity 2005 September;29(9):1070-6. Cross-sectional study
Ref ID: 1486

(3731) Page RC, Harnden KE, Walravens NK, Onslow C, Sutton P, Levy JC, Hockaday DT, Turner RC. 'Healthy living' and sulphonylurea therapy have different effects on glucose tolerance and risk factors for vascular disease in subjects with impaired glucose tolerance. Quarterly Journal of Medicine 1993 March;86(3):145-54. Drug intervention study, Diet & Exercise intervention
Ref ID: 2267

(3732) Paglialonga F, Edefonti A. Nutrition assessment and management in children on peritoneal dialysis. Pediatric Nephrology 2009;24(4):721-30. Diet Intervention Study
Ref ID: 5523

(3733) Pahkala K, Heinonen OJ, Lagstrom H, Hakala P, Sillanmaki L, Kaitosaari T, Viikari J, Aromaa M, Simell O. Parental and childhood overweight in sedentary and active adolescents. Scandinavian Journal of Medicine and Science in Sports 2010 February;20(1):74-82. Not a randomized controlled trial (RCT), Observational study
Ref ID: 149

(3734) Pahkala K, Heinonen OJ, Lagstrom H, Hakala P, Hakanen M, Hernelahti M, Ruottinen S, Sillanmaki L, Ronnemaa T, Viikari J, Raitakari OT, Simell O. Clustered metabolic risk and leisure-time physical activity in adolescents: effect

of dose? British Journal of Sports Medicine 2012 February;46(2):131-7. Cross-sectional study
Ref ID: 2719

(3735) Pahkala K, Hernelahti M, Heinonen OJ, Raittinen P, Hakanen M, Lagstrom H, Viikari JS, Ronnemaa T, Raitakari OT, Simell O. Body mass index, fitness and physical activity from childhood through adolescence. British Journal of Sports Medicine 2013 January;47(2):71-7. Inappropriate Study Design
Ref ID: 6015

(3736) Paineau DL, Beaufils F, Boulier A, Cassuto DA, Chwalow J, Combris P, Couet C, Jouret B, Lafay L, Laville M, Mahe S, Ricour C, Romon M, Simon C, Tauber M, Valensi P, Chapalain V, Zourabichvili O, Bornet F. Family dietary coaching to improve nutritional intakes and body weight control: a randomized controlled trial. Archives of Pediatrics and Adolescent Medicine 2008 January;162(1):34-43. Diet Intervention Study
Ref ID: 1012

(3737) Painter P, Krasnoff J, Mathias R. Exercise capacity and physical fitness in pediatric dialysis and kidney transplant patients. Pediatric Nephrology 2007;22(7):1030-9. Off topic
Ref ID: 5524

(3738) Pakalnis A, Gladstein J. Headaches and hormones. Seminars in Pediatric Neurology 2010;17(2):100-4. Off topic
Ref ID: 3290

(3739) Pal S, Ellis V. The chronic effects of whey proteins on blood pressure, vascular function, and inflammatory markers in overweight individuals. Obesity (Silver Spring) 2010 July;18(7):1354-9. Study limited to adults, Not an exercise intervention study
Ref ID: 85

(3740) Palaniappan L, Carnethon MR, Wang Y, Hanley AJ, Fortmann SP, Haffner SM, Wagenknecht L, Insulin Resistance AS. Predictors of the incident metabolic syndrome in adults: the Insulin Resistance Atherosclerosis Study. Diabetes Care 2004 March;27(3):788-93. Study limited to adults
Ref ID: 1663

(3741) Palatini P, Graniero GR, Mormino P, Nicolosi L, Mos L, Visentin P, Pessina AC. Relation between physical training and ambulatory blood pressure in stage I hypertensive subjects. Results of the HARVEST Trial. Hypertension and Ambulatory Recording Venetia Study. Circulation 1994 December;90(6):2870-6. Study limited to adults
Ref ID: 2229

(3742) Palatini P, Pessina AC, Graniero GR, Canali C, Mormino P, Dorigatti F, Accurso V, Michieletto M, Ferrarese E, Vriz O. [The relationship between

overweight, life style and casual and 24-hour pressures in a population of male subjects with mild hypertension. The results of the HARVEST study]. [Italian]. Giornale Italiano di Cardiologia 1995 August;25(8):977-89. Study limited to adults
Ref ID: 2197

(3743) Palencia D, Mendoza CJ, Torres J, Echandía CA. Kangaroo mother program: physical growth and morbidity in a cohort of children, followed from 40 weeks of postconceptional age until first year. Colombia Médica 2009 September;40(3):292-9. Subjects less than 2 years old
Ref ID: 4410

(3744) Palhares D. Obesidade infantil: como podemos ser eficazes? Jornal de Pediatria 2004 October;80(5):432-3. Editorial or letter or comment
Ref ID: 696

(3745) Palmer GS, Clancy MC, Hawley JA, Rodger IM, Burke LM, Noakes TD. Carbohydrate ingestion immediately before exercise does not improve 20 km time trial performance in well trained cyclists. International Journal of Sports Medicine 1998 August;19(6):415-8. Diet Intervention or Supplement Study
Ref ID: 2079

(3746) Palmer JA, Palmer LK, Michiels K, Thigpen B. Effects of type of exercise on depression in recovering substance abusers. Perceptual and Motor Skills 1995 April;80(2):523-30. Not a randomized controlled trial (RCT)
Ref ID: 2200

(3747) Palmer MA, Capra S, Baines SK. Association between eating frequency, weight, and health. [Review] [60 refs]. Nutrition Reviews 2009 July;67(7):379-90. Review article
Ref ID: 730

(3748) Palmer MS, Logan HM, Spriet LL. On-ice sweat rate, voluntary fluid intake, and sodium balance during practice in male junior ice hockey players drinking water or a carbohydrate-electrolyte solution. Applied Physiology, Nutrition, and Metabolism = Physiologie Appliquee, Nutrition et Metabolisme 2010 June;35(3):328-35. Off topic
Ref ID: 508

(3749) Palmieri B, Sblendorio V, Ferrari A, Pietrobelli A. Duchenne muscle activity evaluation and muscle function preservation: is it possible a prophylactic strategy? Obesity Reviews 2008;9(2):121-39. Off topic
Ref ID: 5525

(3750) Pan CY. The efficacy of an aquatic program on physical fitness and aquatic skills in children with and without autism spectrum disorders. Research in Autism Spectrum Disorders 2011;5(1):657-65. Not All Participants were

Overweight and/or Obese
Ref ID: 3291

(3751) Panagiotakos DB, Pitsavos C, Chrysohoou C, Stefanadis C. The epidemiology of Type 2 diabetes mellitus in Greek adults: the ATTICA study. Diabetic Medicine 2005 November;22(11):1581-8. Study limited to adults
Ref ID: 1460

(3752) Panagiotakos DB, Pitsavos CH, Zampelas AD, Chrysohoou CA, Stefanadis CI. Dairy products consumption is associated with decreased levels of inflammatory markers related to cardiovascular disease in apparently healthy adults: the ATTICA study. Journal of the American College of Nutrition 2010 August;29(4):357-64. Study limited to adults
Ref ID: 2720

(3753) Pandey JS, Kumar R, Devotta S. Health risks of NO2, SPM and SO2 in Delhi (India). Atmospheric Environment 2005;39(36):6868-74. Off topic
Ref ID: 5526

(3754) Pandit K, Mukhopadhyay P, Ghosh S, Chowdhury S. Natriuretic peptides: Diagnostic and therapeutic use. Indian Journal of Endocrinology and Metabolism 2011 October 2;S345-S353. Off topic
Ref ID: 3706

(3755) Pangrazio K, V, Kaczynski R, Shunock M. Early treatment outcome assessed by the Peer Assessment Rating index. American Journal of Orthodontics and Dentofacial Orthopedics 1999;115:544-50. Off topic
Ref ID: 869

(3756) Paoli DS, Abbes PT, Lavrador MSF, Escrivão MAMS, Taddei JAd. Prevenção e tratamento da obesidade na infância: Atividade física e hábitos alimentares. Pediatria Moderna 2009 October;45(5). Review article
Ref ID: 4411

(3757) Papadaki A, Linardakis M, Larsen TM, van-Baak MA, Lindroos AK, Pfeiffer AF, Martinez JA, Handjieva DT, Kunesová M, Holst C, Astrup A, Saris WH, Kafatos A, -DiOGenes-Study-Group. The effect of protein and glycemic index on children's body composition: The DiOGenes randomized study. Pediatrics 2010;126:e1143-e1152. Diet Intervention Study
Ref ID: 4945

(3758) Papailiou A, Sullivan E, Cameron JL. Behaviors in rhesus monkeys (Macaca mulatta) associated with activity counts measured by accelerometer. American Journal of Primatology 2008;70(2):185-90. Animal study
Ref ID: 5527

(3759) Papastergiou M. Exploring the potential of computer and video games for health and physical education: A literature review. Computers and Education

2009;53(3):603-22. Review article
Ref ID: 5528

(3760) Paradis G, Levesque L, Macaulay AC, Cargo M, McComber A, Kirby R, Receveur O, Kishchuk N, Potvin L. Impact of a diabetes prevention program on body size, physical activity, and diet among Kanien'keha:ka (Mohawk) children 6 to 11 years old: 8-year results from the Kahnawake Schools Diabetes Prevention Project. Pediatrics 2005 February;115(2):333-9. Not a randomized controlled trial (RCT), Follow-up Study
Ref ID: 463

(3761) Parat S, Moriette G, Delaperche MF, Escourrou P, Denjean A, Gaultier C. Long-term pulmonary functional outcome of bronchopulmonary dysplasia and premature birth. Pediatric Pulmonology 1995;20:289-96. Off topic
Ref ID: 1001

(3762) Pardee PE, Lavine JE, Schwimmer JB. Diagnosis and treatment of pediatric nonalcoholic steatohepatitis and the implications for bariatric surgery. Seminars in Pediatric Surgery 2009;18(3):144-51. Off topic
Ref ID: 3292

(3763) Pare M, Dugas C. Developmental changes in prehension during childhood. Experimental Brain Research 1999;125(3):239-47. Off topic
Ref ID: 5529

(3764) Parente EB, Guazzelli I, Ribeiro MM, Silva AG, Halpern A, Villares SM. [Obese children lipid profile: effects of hypocaloric diet and aerobic physical exercise]. Arquivos Brasileiros de Endocrinologia and Metabologia 2006 June;50(3):499-504. No exercise only group, No comparative control group
Ref ID: 271

(3765) Parikh S, Guo DH, Pollock NK, Petty K, Bhagatwala J, Gutin B, Houk C, Zhu H, Dong Y. Circulating 25-hydroxyvitamin D concentrations are correlated with cardiometabolic risk among American black and white adolescents living in a year-round sunny climate. Diabetes Care 2012 May;35(5):1133-8. Off topic
Ref ID: 2721

(3766) Park HS, Kim MW, Shin ES. Effect of weight control on hepatic abnormalities in obese patients with fatty liver. Journal of Korean Medical Science 1995 December;10(6):414-21. Study limited to adults
Ref ID: 2187

(3767) Park MI, Camilleri M, O'Connor H, Oenning L, Burton D, Stephens D, Zinsmeister AR. Effect of different macronutrients in excess on gastric sensory and motor functions and appetite in normal-weight, overweight, and obese humans. American Journal of Clinical Nutrition 2007 February;85(2):411-8. Study limited to adults
Ref ID: 252

(3768) Park TG, Hong HR, Lee J, Kang HS. Lifestyle plus exercise intervention improves metabolic syndrome markers without change in adiponectin in obese girls. Annals of Nutrition and Metabolism 2007;51(3):197-203. No exercise only group
Ref ID: 234

(3769) Parks EP, Zemel B, Moore RH, Berkowitz RI. Change in body composition during a weight loss trial in obese adolescents. Pediatric Obesity 2014 February;9(1):26-35. Inappropriate Intervention
Ref ID: 6016

(3770) Parlesak A, Kromker D. Obesity - a social and physical risk. Journal der Deutschen Dermatologischen Gesellschaft 2008;6(6):442-50. Review article
Ref ID: 5530

(3771) Parreira SLS, Resende MBD, Peduto MDC, Marie SKN, Carvalho MS, Reed UC. Quantification of muscle strength and motor ability in patients with Duchenne muscular dystrophy on steroid therapy. Arquivos de Neuro-Psiquiatria 2007 June;65(2A):245-50. Off topic
Ref ID: 4412

(3772) Parrish AM, Iverson D, Russell K, Yeatman H. Observing children's playground activity levels at 13 Illawarra primary schools using CAST2. Journal of Physical Activity and Health 2009;6:Suppl-96. Observational study
Ref ID: 627

(3773) Parsons TJ, Prentice A, Smith EA, Cole TJ, Compston JE. Bone mineral mass consolidation in young British adults. Journal of Bone and Mineral Research 1996 February;11(2):264-74. Off topic
Ref ID: 2165

(3774) Parsons WB, Jr. Controlled-release diethylpropion hydrochloride used in a program for weight reduction. Clinical Therapeutics 1981;3(5):329-35. Drug intervention study
Ref ID: 2374

(3775) Partsch H. [Thrombophlebitis: bed rest or walking exercise?]. [German]. Wiener Medizinische Wochenschrift 1999;149(2-4):50-3. Off topic
Ref ID: 2015

(3776) Paschoal MA, Trevizan PF, Scodeler NF. Variabilidade da frequência cardíaca, lípides e capacidade física de crianças obesas e não-obesas. Variabilidad de la frecuencia cardiaca, lípidos y capacidad física de niños obesos y no obesos. Heart rate variability, blood lipids and physical capacity of obese and non-obese children. Arquivos Brasileiros de Cardiologia 2009 September;93(3):239-46. Not All Participants were Overweight and/or Obese
Ref ID: 4413

(3777) Paschoal MA, Fontana CC. Método do limiar de variabilidade da frequência cardíaca aplicado em pré-adolescentes obesos e não obesos. Method of heart rate variability threshold applied in obese and non-obese pre-adolescents. Método del umbral de variabilidad de la frecuencia cardíaca aplicado en preadolescentes obesos y no obesos. Arquivos Brasileiros de Cardiologia 2011 June;96(6):450-6. Not All Participants were Overweight and/or Obese
Ref ID: 4414

(3778) Passot S, Fonseca F, Alarcon-Lorca M, Rolland D, Marin M. Physical characterisation of formulations for the development of two stable freeze-dried proteins during both dried and liquid storage. European Journal of Pharmaceutics and Biopharmaceutics 2005;60(3):335-48. Off topic
Ref ID: 5531

(3779) Pastucha D, Filipcikova R, Bezdickova M, Blazkova Z, Oborna I, Brezinova J, Machalek L, Sovova E, Cajka V, Bajorek J. Clinical anatomy aspects of functional 3D training - case study. Biomedical Papers-Olomouc 2012;156(1):63-9. Case-Control / Case Study
Ref ID: 5532

(3780) Pate RR, Ward DS, Saunders RP, Felton G, Dishman RK, Dowda M. Promotion of physical activity among high-school girls: a randomized controlled trial. American Journal of Public Health 2005 September;95(9):1582-7. Not All Participants were Overweight and/or Obese
Ref ID: 304

(3781) Pate RR, O'Neill JR. After-school interventions to increase physical activity among youth. [Review] [19 refs]. British Journal of Sports Medicine 2009 January;43(1):14-8. Review article
Ref ID: 816

(3782) Pate RR, Sallis JF, Ward DS, Stevens J, Dowda M, Welk GJ, Young DR, Jobe JB, Strikmiller PK. Age-related changes in types and contexts of physical activity in middle school girls. American Journal of Preventive Medicine 2010 November;39(5):433-9. Cross-sectional study
Ref ID: 2722

(3783) Pate RR, McIver K, Dowda M, Brown WH, Addy C. Directly Observed Physical Activity Levels in Preschool Children. Journal of School Health 2008 August;78(8):438-44. Observational study
Ref ID: 3850

(3784) Paterlini ACCR, Boemer MR. A reinserção escolar na área de oncologia infantil: Avanços y Perspectivas. The scholastic reintegration into the oncology pediatric: Advances and Perspectives. Revista Eletrônica de Enfermagem 2008;10(4). Off topic
Ref ID: 4415

(3785) Patman S, Sanderson D, Blackmore M. Physiotherapy following cardiac surgery: is it necessary during the intubation period? Australian Journal of Physiotherapy 2001;47(1):7-16. Off topic
Ref ID: 1895

(3786) Patrat C, Wolf JP, Epelboin S, Hugues JN, Olivennes F, Granet P, Zorn JR, Jouannet P. Pregnancies, growth and development of children conceived by subzonal injection of spermatozoa. Human Reproduction 1999;14(9):2404-10. Off topic
Ref ID: 5533

(3787) Patrick K, Sallis JF, Prochaska JJ, Lydston DD, Calfas KJ, Zabinski MF, Wilfley DE, Saelens BE, Brown DR. A multicomponent program for nutrition and physical activity change in primary care: PACE+ for adolescents. Archives of Pediatrics and Adolescent Medicine 2001 August;155(8):940-6. Behavior Modification Intervention
Ref ID: 1901

(3788) Patrick K, Norman GJ, Calfas KJ, Sallis JF, Zabinski MF, Rupp J, Cella J. Diet, physical activity, and sedentary behaviors as risk factors for overweight in adolescence. Archives of Pediatric Adolescent Medicine 2004 April;158(4):385-90. Not an exercise intervention study, Cross-sectional study
Ref ID: 337

(3789) Patrick K, Calfas KJ, Norman GJ, Zabinski MF, Sallis JF, Rupp J, Covin J, Cella J. Randomized controlled trial of a primary care and home-based intervention for physical activity and nutrition behaviors: PACE+ for adolescents. Archives of Pediatrics and Adolescent Medicine 2006 February;160(2):128-36. Behavior Modification Intervention
Ref ID: 1424

(3790) Patrick K, Norman GJ, Davila EP, Calfas KJ, Raab F, Gottschalk M, Sallis JF, Godbole S, Covin JR. Outcomes of a 12-month technology-based intervention to promote weight loss in adolescents at risk for type 2 diabetes. Journal of Diabetes Science and Technology 2013 May;7(3):759-70. Inappropriate Intervention
Ref ID: 6017

(3791) Patrick K, Marshall SJ, Davila EP, Kolodziejczyk JK, Fowler JH, Calfas KJ, Huang JS, Rock CL, Griswold WG, Gupta A, Merchant G, Norman GJ, Raab F, Donohue MC, Fogg BJ, Robinson TN. Design and implementation of a randomized controlled social and mobile weight loss trial for young adults (project SMART). Contemporary Clinical Trials 2014 January;37(1):10-8. Inappropriate Study Design
Ref ID: 6018

(3792) Patterson R, Potteiger JA. A comparison of normal versus low dietary carbohydrate intake on substrate oxidation during and after moderate intensity exercise in women. European Journal of Applied Physiology 2011 December;111(12):3143-50. Diet Intervention or Supplement Study
Ref ID: 2723

(3793) Pawlowski B, Jasienska G. Women's preferences for sexual dimorphism in height depend on menstrual cycle phase and expected duration of relationship. Biological Psychology 2005;70(1):38-43. Off topic
Ref ID: 5534

(3794) Pawlowski B, Jasienska G. Women's body morphology and preferences for sexual partners' characteristics. Evolution and Human Behavior 2008;29(1):19-25. Off topic
Ref ID: 5535

(3795) Payne AN, Chassard C, Banz Y, Lacroix C. The composition and metabolic activity of child gut microbiota demonstrate differential adaptation to varied nutrient loads in an in vitro model of colonic fermentation. Fems Microbiology Ecology 2012;80(3):608-23. Off topic
Ref ID: 5536

(3796) Pearson HA. The ever-changing content of Pediatrics over fifty years. Pediatrics 1998 July 2;102(1):168. Review article
Ref ID: 3707

(3797) Pedra CAC, Pedra SF, Esteves CA, Chamiê F, Ramos S, Pontes Júnior SC, Tress JC, Braga SLN, Latson LA, Fontes VF. Experiência inicial no Brasil com a prótese Helex para oclusão percutânea de defeitos interatriais. Initial experience in Brazil with the Helex septal occluder for percutaneous occlusion of atrial septal defects. Arquivos Brasileiros de Cardiologia 2003 November;81(5):435-52. Off topic
Ref ID: 4416

(3798) Pedrinola F, Cavaliere H, Lima N, Medeiros-Neto G. Is DL-fenfluramine a potentially helpful drug therapy in overweight adolescent subjects? Obesity Research 1994 January;2(1):1-4. Drug intervention study
Ref ID: 2217

(3799) Pedrosa C, Oliveira BM, Albuquerque I, Simoes-Pereira C, Vaz-de-Almeida MD, Correia F. Markers of metabolic syndrome in obese children before and after 1-year lifestyle intervention program. European Journal of Nutrition 2011 September;50(6):391-400. Lifestyle Intervention
Ref ID: 2724

(3800) Pedrosa C, Oliveira BM, Albuquerque I, Simoes-Pereira C, Vaz-de-Almeida MD, Correia F. Metabolic syndrome, adipokines and ghrelin in overweight and obese schoolchildren: results of a 1-year lifestyle intervention programme.

European Journal of Pediatrics 2011 April;170(4):483-92. Lifestyle Intervention
Ref ID: 2725

(3801) Pedrozo WR, Bonneau GA, Castillo Rascon MS, Marín G. Prevalencia de obesidad y síndrome metabólico en adolescentes de la ciudad de Posadas, Misiones. Prevalence of obesity and metabolic syndrome in adolescents from Posadas, Misiones, Argentina. Revista Argentina de Endocrinología y Metabolismo 2008 September;45(4):131-41. Cross-sectional study
Ref ID: 4417

(3802) Pedrozo W, Rascón MC, Bonneau G, Pianesi MId, Olivera CC, Aragón SJd, Ceballos B, Gauvry G. Síndrome metabólico y factores de riesgo asociados con el estilo de vida de adolescentes de una ciudad de Argentina, 2005. Revista Panamericana de Salud Pública 2008 September;24(3):149-60. Prevalence study
Ref ID: 4418

(3803) Peeters BWMM, Tonnaer JADM, Groen MB, Broekkamp CLE, Van Der Voort HAA, Schoonen WGFJ, Smets RJM, Vanderheyden PML, Gebhard R, Ruigt GSF. Short Review. Stress: The International Journal on the Biology of Stress 2004 December;7(4):233-41. Off topic
Ref ID: 3708

(3804) Peixoto MDRG, Monego ET, Alexandre VP, Souza RGMD, Moura ECD. Monitoramento por entrevistas telefônicas de fatores de risco para doenças crônicas: Experiência de Goiânia, Goiás, Brasil. Cadernos de Saúde Pública 2008 June;24(6):1323-33. Survey or questionnaire
Ref ID: 4419

(3805) Peker Y, Durak E, Ozgurbuz U. Intragastric balloon treatment for obesity: prospective single-center study findings. Obesity Facts 2010;3(2):105-8. Off topic
Ref ID: 528

(3806) Pekhlivanov B, Mitkov M, Kavurdzhikova S. [Clinical, hormonal and biochemical changes after treatment with metformin and weight reduction in women with polycystic ovary syndrome]. [Bulgarian]. Akusherstvo i Ginekologiia 2006;45(6):29-35. Drug intervention study
Ref ID: 1315

(3807) Pelayo P, Mujika I, Sidney M, Chatard JC. Blood lactate recovery measurements, training, and performance during a 23-week period of competitive swimming. European Journal of Applied Physiology and Occupational Physiology 1996;74(1-2):107-13. Not a randomized controlled trial (RCT)
Ref ID: 2156

(3808) Pelegrini A, Petroski EL. Excesso de peso em adolescentes: Prevalência e fatores associados. Revista Brasileira de Atividade Física e Saúde 2007;12(3). Prevalence study
Ref ID: 4420

(3809) Pelegrini A, Petroski EL, Coqueiro RdS, Gaya ACA. Overweight and obesity in brazilian schoolchildren aged 10 to 15 years: data from a Brazilian sports projection. Sobrepeso e obesidade em escolares brasileiros de 10 a 15 anos: Dados do projeto esporte Brasil. Archivos Latinoamericanos de Nutrición 2008 December;58(4):343-9. Cross-sectional study
Ref ID: 4421

(3810) Pelegrini A, Silva RCRd, Petroski EL. Relação entre o tempo em frente à TV gasto calórico em adolescentes com diferentes percentuais de gordura corporal. Revista Brasileira de Cineantropometria e Desempenho Humano 2008 March;10(1). Cross-sectional study
Ref ID: 4422

(3811) Pelegrini A, Silva DA, Petroski EL, Gaya AC. Sobrepeso e obesidade em escolares brasileiros de sete a nove anos: Dados do projeto esporte Brasil. Revista Paulista de Pediatria 2010 September;28(3):290-5. Cross-sectional study
Ref ID: 4423

(3812) Pella D, Otsuka K, Singh RB. Metabolic syndrome: A disease of the brain. Open Nutraceuticals Journalournal 2011;4:107-18. Review article
Ref ID: 3293

(3813) Pellegrini CA, Duncan JM, Moller AC, Buscemi J, Sularz A, DeMott A, Pictor A, Pagoto S, Siddique J, Spring B. A smartphone-supported weight loss program: design of the ENGAGED randomized controlled trial. BMC Public Health 2012;12:1041. Inappropriate Study Design
Ref ID: 6019

(3814) Pelletier DL, Frongillo EA. Changes in child survival are strongly associated with changes in malnutrition in developing countries. Journal of Nutrition 2003;133(1):107-19. Off topic
Ref ID: 5537

(3815) Pellett TL, Henschel-Pellett HA, Harrison JM. Influence of ball weight on junior high school girls' volleyball performance. Perceptual and Motor Skills 1994;78:1379-84. Off topic
Ref ID: 4946

(3816) Peltola H, Ukkonen P, Saxen H, Stass H. Single-dose and steady-state pharmacokinetics of a new oral suspension of ciprofloxacin in children. Pediatrics 1998 April;101(4:Pt 1):t-62. Drug intervention study
Ref ID: 2100

(3817) Pempek TA, Calvert SL. Tipping the balance: use of advergames to promote consumption of nutritious foods and beverages by low-income African American children. Archives of Pediatrics and Adolescent Medicine 2009 July;163(7):633-7. Diet Intervention Study
Ref ID: 725

(3818) Pena M, Barta L, Simon G, Regoly-Merei A, Tichy M, Bedo M. [Effect of caffeine on the physical achievement of obese children]. [Hungarian]. Orvosi Hetilap 1982 January 24;123(4):207-9. Diet Intervention or Supplement Study
Ref ID: 2371

(3819) Peneau S, Thibault H, Meless D, Soulie D, Carbonel P, Roinsol D, Longueville E, Serog P, Deheeger M, Bellisle F, Maurice-Tison S, Rolland-Cachera MF. Anthropometric and behavioral patterns associated with weight maintenance after an obesity treatment in adolescents. Journal of Pediatrics 2008 May;152(5):678-84. Follow-up Study
Ref ID: 973

(3820) Pennington RL. Causes of early human population growth. American Journal of Physical Anthropology 1996;99(2):259-74. Off topic
Ref ID: 5538

(3821) Penny ME, Marin RM, Duran A, Peerson JM, Lanata CF, Lönnerdal B, Black RE, Brown KH. Randomized controlled trial of the effect of daily supplementation with zinc or multiple micronutrients on the morbidity, growth, and micronutrient status of young Peruvian children. American Journal of Clinical Nutrition 2004;79:457-65. Diet Intervention or Supplement Study
Ref ID: 4947

(3822) Peña M. The effects of sedentarism and physical activity on children's health. West Indian Medical Journal 2002 March 10;51(Supl.1):46-8. Review article
Ref ID: 736

(3823) Peralta LR, Jones RA, Okely AD. Promoting healthy lifestyles among adolescent boys: the Fitness Improvement and Lifestyle Awareness Program RCT. Preventive Medicine 2009 June;48(6):537-42. No exercise only group, No comparative control group
Ref ID: 124

(3824) Pereira Da SD, Martinez De OJ, Negreiro F. Observational study of vaginal pH in healthy Portuguese women. Minerva Ginecologica 2011 April;63(2):203-12. Off topic
Ref ID: 2726

(3825) Pereira CH, Ferreira DdS, Copetti GL, Guimarães LC, Barbacena MM, Liggeri N, Castro OG, Lobato S, David ACd. Aptidão física em escolares de uma unidade de ensino da rede pública de Brasília-DF. Physical fitness among schoolchildren in a teaching unit of the public unit of Brasilia-DF. Revista

Brasileira de Atividade Física e Saúde 2011 July;16(3). Cross-sectional study
Ref ID: 4424

(3826) Pereira FM, Ribeiro MÂGdO, Ribeiro AF, Toro AADC, Hessel G, Ribeiro JD. Desempenho funcional de pacientes com fibrose cística e indivíduos saudáveis no teste de caminhada de seis minutos. Functional performance on the six-minute walk test in patients with cystic fibrosis. Jornal Brasileiro de Pneumologia 2011 December;37(6):735-44. Off topic
Ref ID: 4425

(3827) Perez AJ, Carletti L. Identificação do limiar anaeróbio ventilatório em crianças e adolescentes: revisão da literatura. Identifying ventilatory anaerobic threshold in children and adolescents: A literature review. Revista Brasileira de Cineantropometria e Desempenho Humano 2012;14(3):343-52. Review article
Ref ID: 4426

(3828) Perez MO, Ciambelli GS, Nigri AA, Vieira MW, Costa CD. Doença celíaca associada à tireoidite de Hashimoto e síndrome de Noonan. Celiac disease associated with Hashimoto's thyroiditis and Noonan syndrome. Revista Paulista de Pediatria 2010 December;28(4):398-404. Case-Control / Case Study
Ref ID: 4427

(3829) Perezescamilla R, Pollitt E. Growth Improvements in Children Above 3 Years of Age - the Cali Study. Journal of Nutrition 1995;125(4):885-93. Secondary analysis
Ref ID: 5539

(3830) Perichart-Perera O, Balas-Nakash M, Ortiz-Rodríguez V, Morán-Zenteno JA, Guerrero-Ortiz JL, Vadillo-Ortega F. Programa para mejorar marcadores de riesgo cardiovascular en escolares mexicanos. Salud Pública de México 2008 June;50(3):218-26. Not All Participants were Overweight and/or Obese
Ref ID: 4428

(3831) Perim RR, Signorelli GR, Myers J, Arena R, Araújo CGSd. The slope of the oxygen pulse curve does not depend on the maximal heart rate in elite soccer players. Clinics 2011;66(5):829-35. Off topic
Ref ID: 4429

(3832) Perkins KA, Sexton JE. Influence of aerobic fitness, activity level, and smoking history on the acute thermic effect of nicotine. Physiology and Behavior 1995 June;57(6):1097-102. Off topic
Ref ID: 2202

(3833) Perrella MM, Noriyuki PS, Rossi L. Avaliação da perda hídrica durante treino intenso de rugby. Revista Brasileira de Medicina do Esporte 2005 August;11(4):229-32. Off topic
Ref ID: 4430

(3834) Perrin EM, Vann JC, Lazorick S, Ammerman A, Teplin S, Flower K, Wegner SE, Benjamin JT. Bolstering confidence in obesity prevention and treatment counseling for resident and community pediatricians. Patient Education and Counseling 2008 November;73(2):179-85. Survey or questionnaire
Ref ID: 827

(3835) Perrin EM, Rothman RL, Sanders LM, Skinner AC, Eden SK, Shintani A, Throop EM, Yin HS. Racial and ethnic differences associated with feeding- and activity-related behaviors in infants. Pediatrics 2014 April;133(4):e857-e867. Inappropriate Population
Ref ID: 6020

(3836) Perry AC, Rosenblatt ES, Kempner L, Feldman BB, Paolercio MA, Van Bemden AL. The effects of an exercise physiology program on physical fitness variables, body satisfaction, and physiology knowledge. Journal of Strength and Conditioning Research 2002 May;16(2):219-26. Not a randomized controlled trial (RCT)
Ref ID: 1835

(3837) Perry CM, Wagstaff AJ. Recombinant mammalian cell-derived somatropin - A review of its pharmacological properties and therapeutic potential in the management of wasting associated with HIV infection. BioDrugs 1997;8(5):394-414. Off topic
Ref ID: 5540

(3838) Perusse L, Collier G, Gagnon J, Leon AS, Rao DC, Skinner JS, Wilmore JH, Nadeau A, Zimmet PZ, Bouchard C. Acute and chronic effects of exercise on leptin levels in humans. Journal of Applied Physiology 1997 July;83(1):5-10. Study limited to adults
Ref ID: 2133

(3839) Peter R, Luzio SD, Dunseath G, Miles A, Hare B, Backx K, Pauvaday V, Owens DR. Effects of exercise on the absorption of insulin glargine in patients with type 1 diabetes. Diabetes Care 2005 March;28(3):560-5. Drug intervention study
Ref ID: 1540

(3840) Peterson JL, Puhl RM, Luedicke J. An experimental assessment of physical educators' expectations and attitudes: the importance of student weight and gender. Journal of School Health 2012 September;82(9):432-40. Inappropriate Population
Ref ID: 6021

(3841) Peterson KE, Dubowitz T, Stoddard AM, Troped PJ, Sorensen G, Emmons KM. Social context of physical activity and weight status in working-class populations. Journal of Physical Activity and Health 2007 October;4(4):381-96.

Inappropriate Outcomes
Ref ID: 1118

(3842) Peterson KE, Fox MK. Addressing the epidemic of childhood obesity through school-based interventions: what has been done and where do we go from here?. [Review] [110 refs]. Journal of Law, Medicine and Ethics 2007;35(1):113-30. Review article
Ref ID: 1264

(3843) Petibois C, Cazorla G, Poortmans JR, Deleris G. Biochemical aspects of overtraining in endurance sports: the metabolism alteration process syndrome. / Aspects biochimiques du surentrainement dans les sports d ' endurance: le syndrome du processus d ' alteration du metabolisme. Sports Medicine 2003;33(2):83-94. Off topic
Ref ID: 3892

(3844) Petit MA, McKay HA, MacKelvie KJ, Heinonen A, Khan KM, Beck TJ. A randomized school-based jumping intervention confers site and maturity-specific benefits on bone structural properties in girls: a hip structural analysis study. Journal of Bone and Mineral Research 2002;17:363-72. Prospective Study
Ref ID: 4948

(3845) Petrelluzzi KFS, Kawamura M, Paschoal MA. Avaliação funcional cardiovascular de crianças sedentárias obesas e não-obesas. Revista de Ciências Médicas (Campinas) 2004 June;13(2):127-36. Not All Participants were Overweight and/or Obese
Ref ID: 4431

(3846) Petrofsky J, Batt J, Berk L, Schweichler C, Arai D, Conas C, Newell J, Case K, Keener K, Bains G. The effect of an exercise and diet program on fitness, posture and self image in women. Journal of Applied Research 2010;10(1):40-51. Diet & Exercise intervention
Ref ID: 3294

(3847) Petroski EL, Silva RJ, Pelegrini A. Crescimento físico e estado nutricional de crianças e adolescentes da região de Cotinguiba, Sergipe. Revista Paulista de Pediatria 2008 September;26(3):206-11. Cross-sectional study
Ref ID: 4432

(3848) Petroski EL, Pelegrini A. Associação entre o estilo de vida dos pais e a composição corporal dos filhos adolescentes. Association of parental lifestyle with body composition of their adolescent children. Revista Paulista de Pediatria 2009 March;27(1):48-52. Cross-sectional study
Ref ID: 4433

(3849) Petroski EL, Silva AFd, Rodrigues AB, Pelegrini A. Aptidão física relacionada a saúde em adolescentes brasileiros residentes em áreas de médio/baixo índice

de desenvolvimento humano. Health-related physical fitness in Brazilian adolescents from areas having a medium/low Human Development Index. Capacidades físicas relacionadas con la salud de adolescentes brasileños residentes en áreas con índice de desarrollo humano medio/bajo. Revista de Salud Pública 2011 April;13(2):219-28. Cross-sectional study
Ref ID: 4434

(3850) Pettersen SA, Fredriksen PM, Ingjer E. The correlation between peak oxygen uptake (VO2peak) and running performance in children and adolescents. aspects of different units. Scandinavian Journal of Medicine and Science in Sports 2001 August;11(4):223-8. Cross-sectional study
Ref ID: 1904

(3851) Petty J, Oliver C, Moss J, Howlin P, Tunnicliffe P, Griffith G, Hastings R. Investigating sleep architecture in Angelman, Cri du Chat and Cornelia de Lange syndromes. Journal of Intellectual Disability Research 2008 October;52(10):815. Off topic
Ref ID: 3851

(3852) Petty KH, Davis CL, Tkacz J, Young-Hyman D, Waller JL. Exercise effects on depressive symptoms and self-worth in overweight children: a randomized controlled trial. Journal of Pediatric Psychology 2009 October;34(9):929-39. Same subjects as another study already included
Ref ID: 134

(3853) Pezzetta OM, Lopes ADS, Pires Neto CS. Indicadores de aptidão física relacionados à saúde em escolares do sexo masculino. Revista Brasileira de Cineantropometria e Desempenho Humano 2003;5(2). Cross-sectional study
Ref ID: 4435

(3854) Pérez-Gil SE, Paz C, Romero G. Cuerpo, imagen y saberes alimentarios en infantes oaxaqueños, México: un primer acercamiento. Corpo, imagem e saberes alimentares em infantes em Oaxaca, México: Uma primeira aproximação. Body, image and nutritious knowledge in Oaxaca, Mexico, infants: A first approximation. Revista Latinoamericana de Ciencias Sociales, Niñez y Juventud 2011 December;9(2):847-68. Survey or questionnaire
Ref ID: 4436

(3855) Pérez Miret H, Consuegra R, Porro Y, Moroño M, Amador M. Indices de hidroxiprolina y excreción de creatinina en niños y adolescentes obsesos; cambios a las 2 semanas de tratamiento con dieta hipocalórica y ejercicio. Revista Cubana de Pediatria 1986 August;58(4):419-25. Study less than 4 weeks
Ref ID: 4437

(3856) Pérez Villasante L, Raigada Mares J, Collins Estrada A, Mauricio Alza S, Felices Parodi A, Jiménez Castro S, Casas Castañeda J. Efectividad de un

programa educativo en estilos de vida saludables sobre la reducción de sobrepeso y obesidad en el Colegio Robert M. Smith; Huaraz, Ancash, Perú. Acta Médica Peruana 2008 December;25(4):204-9. Lifestyle Intervention
Ref ID: 4438

(3857) Pérez A, Reininger BM, Aguirre Flores MI, Sanderson M, Roberts RE. Physical activity and overweight among adolescents on the Texas-Mexico border. Revista Panamericana de Salud Pública 2006 April;19(4):244-52. Survey or questionnaire
Ref ID: 4439

(3858) Phelan S, Wing RR, Loria CM, Kim Y, Lewis CE. Prevalence and predictors of weight-loss maintenance in a biracial cohort: results from the coronary artery risk development in young adults study. American Journal of Preventive Medicine 2010 December;39(6):546-54. Study limited to adults
Ref ID: 1066

(3859) Phelan S, Phipps MG, Abrams B, Darroch F, Grantham K, Schaffner A, Wing RR. Does behavioral intervention in pregnancy reduce postpartum weight retention? Twelve-month outcomes of the Fit for Delivery randomized trial. American Journal of Clinical Nutrition 2014 February;99(2):302-11. Inappropriate Population
Ref ID: 6022

(3860) Philibert A, Bouchard M, Mergler D. Neuropsychiatric Symptoms, Omega-3, and Mercury Exposure in Freshwater Fish-Eaters. Archives of Environmental and Occupational Health 2008;63(3):143-53. Off topic
Ref ID: 3709

(3861) Phillips DIW, Syddall HE, Cooper C, Hanson MA. Association of Adult Height and Leg Length with Fasting Plasma Cortisol Concentrations: Evidence for an Effect of Normal Variation in Adrenocortical Activity on Growth. American Journal of Human Biology 2008;20(6):712-5. Study limited to adults
Ref ID: 5541

(3862) Phillips SM, Turner AP, Gray S, Sanderson MF, Sproule J. Ingesting a 6% carbohydrate-electrolyte solution improves endurance capacity, but not sprint performance, during intermittent, high-intensity shuttle running in adolescent team games players aged 12-14 years. European Journal of Applied Physiology 2010 July;109(5):811-21. Diet Intervention or Supplement Study
Ref ID: 506

(3863) Phillips SM, Turner AP, Sanderson MF, Sproule J. Beverage carbohydrate concentration influences the intermittent endurance capacity of adolescent team games players during prolonged intermittent running. European Journal of Applied Physiology 2012 March;112(3):1107-16. Diet Intervention or

Supplement Study
Ref ID: 2729

(3864) Phillips SM, Turner AP, Sanderson MF, Sproule J. Carbohydrate gel ingestion significantly improves the intermittent endurance capacity, but not sprint performance, of adolescent team games players during a simulated team games protocol. European Journal of Applied Physiology 2012 March;112(3):1133-41. Diet Intervention or Supplement Study
Ref ID: 2728

(3865) Pianosi PT, Fisk M. Cardiopulmonary exercise performance in prematurely born children. Pediatric Research 2000 May;47(5):653-8. Off topic
Ref ID: 1978

(3866) Piccinelli M, Pini S, Bellantuono C, Wilkinson G. Efficacy of drug treatment in obsessive-compulsive disorder. A meta-analytic review. The British Journal of Psychiatry: The Journal of Mental Science 1995;166:424-43. Review article
Ref ID: 998

(3867) Pichler G, Urlesberger B, Jirak P, Zotter H, Muller W. Forearm oxygen consumption and forearm blood flow in healthy children and adolescents measured by near infrared spectroscopy. Journal of Physiological Sciences 2006;56(3):191-4. Off topic
Ref ID: 5542

(3868) Piedboeuf B, Jones S, Orrbine E, Filler G. Are the career choices of paediatric residents meeting the needs of academic centres in Canada? Paediatrics and Child Health 2012;17(1):21-4. Off topic
Ref ID: 5543

(3869) Piek JP, Straker LM, Jensen L, Dender A, Barrett NC, McLaren S, Roberts C, Reid C, Rooney R, Packer T, Bradbury G, Elsley S. Rationale, design and methods for a randomised and controlled trial to evaluate "Animal Fun" - a program designed to enhance physical and mental health in young children. BMC Pediatrics 2010 November 4;10:78.:78. Description versus conduct of study
Ref ID: 13

(3870) Pienaar AE. Kinderkinetics: An investment in the total well-being of children. South African Journal for Research in Sport Physical Education and Recreation 2009;31(1):49-67. Description versus conduct of study
Ref ID: 5544

(3871) Pierre S, Eschenhagen T, Geisslinger G, Scholich K. Capturing adenylyl cyclases as potential drug targets. Nature Reviews Drug Discovery 2009 April;8(4):321-35. Drug intervention study
Ref ID: 3710

(3872) Pietrobelli A, Tosi C, Olivieri F, Lubrano L, Fuiano N. From fatness to leaness: Where did we go wrong? International Journal of Pediatric Obesity 2011;6:42-5. Review article
Ref ID: 5545

(3873) Pignatelli P, Basili S. Nutraceuticals in the Early Infancy. Cardiovascular Therapeutics 2010 August;28(4):236-45. Review article
Ref ID: 3711

(3874) Piguel X, Abraham P, Bouhours-Nouet N, Gatelais F, Dufresne S, Rouleau S, Coutant R. Impaired aerobic exercise adaptation in children and adolescents with craniopharyngioma is associated with hypothalamic involvement. European Journal of Endocrinology 2012 February;166(2):215-22. Off topic
Ref ID: 2731

(3875) Piko B. Health-related predictors of self-perceived health in a student population: the importance of physical activity. Journal of Community Health 2000 April;25(2):125-37. Survey or questionnaire
Ref ID: 1982

(3876) Pikosky MA, Gaine PC, Martin WF, Grabarz KC, Ferrando AA, Wolfe RR, Rodriguez NR. Aerobic exercise training increases skeletal muscle protein turnover in healthy adults at rest. Journal of Nutrition 2006 February;136(2):379-83. Study limited to adults
Ref ID: 1428

(3877) Pileggi C, Carbone V, Nobile CG, Pavia M. Blood pressure and related cardiovascular disease risk factors in 6-18 year-old students in Italy. Journal of Paediatrics and Child Health 2005 July;41(7):347-52. Not an exercise intervention study, Observational study
Ref ID: 307

(3878) Pillay WR, Kan YM, Crinnion JN, Abbott KC, Hypolite IO, Agodoa LY, Kjeldsen SE, Dahlof B, Devereaux RB, Little P, Barnett J, Barnsley L, Paueksakon P, Revelo MP, Ma LJ, Booth C, Preston R, Clark G, Leertouwer TC. Hypertension and Systemic Disease. Kidney 2003 January;12(1):21. Off topic
Ref ID: 3712

(3879) Pincelli AI, Bragato R, Scacchi M, Branzi G, Osculati G, Viarengo R, Leonetti G, Cavagnini F. Three weekly injections (TWI) of low-dose growth hormone (GH) restore low normal circulating IGF-I concentrations and reverse cardiac abnormalities associated with adult onset GH deficiency (GHD). Journal of Endocrinological Investigation 2003 May;26(5):420-8. Drug intervention study
Ref ID: 1726

(3880) Pineda Pérez EJ, Gutiérrez Baró EH. Control de la obesidad en niños con síndrome de Down. The obesity control in children with Down syndrome. Revista Cubana de Medicina General Integral 2011 June;27(2). Educational

intervention
Ref ID: 4440

(3881) Pineda AG, Diaz MVP, Guzman MDPG. Preanesthetic medication with midazolam in children: A study comparing rectal and intramuscular administration. Medicacion Preanestesica con Midazolam en Ninos: Estudio Comparativo Via Rectal O Intramuscular. Anestesia en Mexico 1997;9:54-62. Drug intervention study
Ref ID: 4950

(3882) Pinhas-Hamiel O, Zeitler P. Clinical presentation and treatment of type 2 diabetes in children. Pediatric Diabetes 2007;8(SUPPL. 9):16-27. Review article
Ref ID: 3295

(3883) Pinheiro KAT, Horta BL, Pinheiro RT, Horta LL, Terres NG, Silva RAd. Common mental disorders in adolescents: A population based cross-sectional study. Revista Brasileira de Psiquiatria 2007 September;29(3):241-5. Cross-sectional study
Ref ID: 4441

(3884) Pinho RAd, Petroski EL. Adiposidade corporal e nível de atividade física em adolescentes. Revista Brasileira de Cineantropometria e Desempenho Humano 1999;1(1):60-8. Cross-sectional study
Ref ID: 4442

(3885) Pinkoski C, Chilibeck PD, Candow DG, Esliger D, Ewaschuk JB, Facci M, Farthing JP, Zello GA. The effects of conjugated linoleic acid supplementation during resistance training. Medicine and Science in Sports and Exercise 2006 February;38(2):339-48. Diet Intervention or Supplement Study
Ref ID: 1406

(3886) Pinnelli A, Fiori F. [Obesity among the adult population in Italy]. [Italian]. Epidemiologia e Prevenzione 2011 March;35(2):111-7. Study limited to adults
Ref ID: 2732

(3887) Pinto LLC, Schwartz IVD, Puga ACS, Vieira TA, Munoz MV, Giugliani R. Prospective study of 11 Brazilian patients with mucopolysaccharidosis II. Jornal de Pediatria 2006 August;82(4):273-8. Prevalence study
Ref ID: 658

(3888) Pinto MCM, Oliveira AdC. Occurrence of child obesity in preschool children in a São Paulo day-care center. Ocorrência da obesidade infantil em pré-escolares de uma creche de São Paulo. Einstein (São Paulo) 2009;7(2):170-5. Survey or questionnaire
Ref ID: 4443

(3889) Pinto M, Seclén S, Cabello E. Diabetes tipo 2 en niños: reporte de caso. Type 2 diabetes in children: a case report. Revista Médica Herediana 2010 June;21(2):103-6. Case-Control / Case Study
Ref ID: 4444

(3890) Pinto SL, Silva RdCR, Priore SE, Assis AMO, Pinto EdJ. Prevalência de pré-hipertensão e de hipertensão arterial e avaliação de fatores associados em crianças e adolescentes de escolas públicas de Salvador, Bahia, Brasil. Prevalence of pre-hypertension and arterial hypertension and evaluation of associated factors in children and adolescents in public schools in Salvador, Bahia State, Brazil. Cadernos de Saúde Pública 2011 June;27(6):1065-75. Prevalence study
Ref ID: 4445

(3891) Piñeros M, Pardo C. Actividad física en adolescentes de cinco ciudades colombianas: resultados de la Encuesta Mundial de Salud a Escolares. Physical activity in adolescents of five Colombian cities: Results of the Global Youth Health Survey. Revista de Salud Pública 2010 December;12(6):903-14. Survey or questionnaire
Ref ID: 4446

(3892) Pipes TV. Variable resistance versus constant resistance strength training in adult males. European Journal of Applied Physiology and Occupational Physiology 1978 July 17;39(1):27-35. Study limited to adults
Ref ID: 2383

(3893) Pires ACL, Moreira PVL. Aspectos Antropométricos, Nutricionais e de Atividade Física em Escolares do Bairro do Grotão, João Pessoa/PB. Anthropometric, Nutritional and physical activity aspects in schoolchildren from the District of Grotão, Joao Pessoa / PB. Revista Brasileira de Ciências da Saúde 2011 December;15(3). Cross-sectional study
Ref ID: 4447

(3894) Pistilli EE, Devaney JM, Gordish-Dressman H, Bradbury MK, Seip RL, Thompson PD, Angelopoulos TJ, Clarkson PM, Moyna NM, Pescatello LS, Visich PS, Zoeller RF, Gordon PM, Hoffman EP. Interleukin-15 and interleukin-15R alpha SNPs and associations with muscle, bone, and predictors of the metabolic syndrome. Cytokine 2008 July;43(1):45-53. Inappropriate Outcomes
Ref ID: 932

(3895) Pitetti KH, Rendoff AD, Grover T, Beets MW. The efficacy of a 9-month treadmill walking program on the exercise capacity and weight reduction for adolescents with severe autism. Journal of Autism and Developmental Disorders 2007 July;37(6):997-1006. Not a randomized controlled trial (RCT)
Ref ID: 1208

(3896) Place I, Englert Y. A prospective longitudinal study of the physical, psychomotor, and intellectual development of singleton children up to 5 years who were conceived by intracytoplasmic sperm injection compared with children conceived spontaneously and by in vitro fertilization. Fertility and sterility 2003;80:1388-97. Cohort Study
Ref ID: 4951

(3897) Plachta-Danielzik S, Pust S, Asbeck I, Czerwinski-Mast M, Langnase K, Fischer C, Bosy-Westphal A, Kriwy P, Muller MJ. Four-year follow-up of school-based intervention on overweight children: the KOPS study. Obesity (Silver Spring) 2007 December;15(12):3159-69. Not a randomized controlled trial (RCT), No exercise only group
Ref ID: 205

(3898) Plasschaert F, Jones K, Forward M. The effect of simulating weight gain on the energy cost of walking in unimpaired children and children with cerebral palsy. Archives of Physical Medicine and Rehabilitation 2008;89:2302-8. Off topic
Ref ID: 4953

(3899) Platat C, Perrin AE, Oujaa M, Wagner A, Haan MC, Schlienger JL, Simon C. Diet and physical activity profiles in French preadolescents. The British Journal of Nutrition 2006;96:501-7. Survey or questionnaire
Ref ID: 4954

(3900) Ploin D, Chapuis FR, Stamm D, Robert J, David L, Chatelain PG, Dutau G, Floret D. High-Dose Albuterol by Metered-Dose Inhaler Plus a Spacer Device Versus Nebulization in Preschool Children with Recurrent Wheezing. Pediatrics 2000 August;106(2):311. Drug intervention study
Ref ID: 509

(3901) Plonka M, Toton-Morys A, Adamski P, Suder A, Bielanski W, Dobrzanska MJ, Kaminska A, Piorecka B, Glodzik J. Association of the Physical Activity with Leptin Blood Serum Level, Body Mass Indices and Obesity in Schoolgirls. Journal of Physiology and Pharmacology 2011;62(6):647-56. Survey or questionnaire
Ref ID: 5546

(3902) Plourde MÃ, Cunnane SC. Extremely limited synthesis of long chain polyunsaturates in adults: implications for their dietary essentiality and use as supplements. Applied Physiology, Nutrition and Metabolism 2007 August;32(4):619-34. Diet Intervention Study
Ref ID: 3713

(3903) Po'e EK, Heerman WJ, Mistry RS, Barkin SL. Growing Right Onto Wellness (GROW): a family-centered, community-based obesity prevention randomized controlled trial for preschool child-parent pairs. Contemporary Clinical Trials

2013 November;36(2):436-49. Inappropriate Study Design
Ref ID: 6023

(3904) Podpalov VP, Deev AD, Sivakov VP, Rozum LA. [Prognostic significance of parameters of heart rate variability as a risk factor of development of hypertension]. [Russian]. Kardiologiia 2006;46(1):39-42. Off topic
Ref ID: 1415

(3905) Poehlman ET, Dvorak RV, DeNino WF, Brochu M, Ades PA. Effects of resistance training and endurance training on insulin sensitivity in nonobese, young women: a controlled randomized trial. Journal of Clinical Endocrinology and Metabolism 2000 July;85(7):2463-8. Not All Participants were Overweight and/or Obese
Ref ID: 1970

(3906) Poeta LS, Duarte MdFdS, Giuliano IdCB. Qualidade de vida relacionada à saúde de crianças obesas. Health-related quality of life of obese children. Revista da Associação Médica Brasileira (1992) 2010;56(2):168-72. Cross-sectional study
Ref ID: 4448

(3907) Poeta LS, Duarte MdFdS, Giuliano IdCB, Farias Junior JCd. Intervenção interdisciplinar na composição corporal e em testes de aptidão física de crianças obesa. Interdisciplinary intervention on body composition and physical fitness tests in obese children. Revista Brasileira de Cineantropometria e Desempenho Humano 2012;14(2):134-43. Not a randomized controlled trial (RCT)
Ref ID: 4449

(3908) Pohle-Krauza RJ, McCarroll ML, Pannikottu K, Latta TN, DiNuoscio DR, Volsko TA, Gothard MD, Krauza ML. Body mass index moderates the effects of portable oxygen transport modality on 6-minute walk distance in patients with COPD. Journal of Cardiopulmonary Rehabilitation and Prevention 2014 January;34(1):62-8. Inappropriate Population
Ref ID: 6024

(3909) Polak F, Morton R, Ward C, Wallace WA, Doderlein L, Siebel A. Double-blind comparison study of two doses of botulinum toxin A injected into calf muscles in children with hemiplegic cerebral palsy. Developmental Medicine and Child Neurology 44(8):551-5, 2002 Aug 2002;(8):551-5. Off topic
Ref ID: 3100

(3910) Pollitt E, Saco PC, Jahari A, Husaini MA, Huang J. Effects of an energy and micronutrient supplement on mental development and behavior under natural conditions in undernourished children in Indonesia. European Journal of Clinical Nutrition 2000;54 Suppl 2:S80-S90. Diet Intervention or Supplement

Study
Ref ID: 4955

(3911) Polo R, Galindo MJ, Martinez E, Alvarez J, Arevalo JM, Asensi V, Canoves D, Cancer E, Collazos J, Estrada V, Gomez-Candela C, Johnston S, Locutura J, Lopez-Aldeguer J, Lozano F, Miralles C, Munoz-Sanz A, Ortega E, Pascua J, Pedrol E, Pulido F, Martin MS, Sanz J, Viciana P, Chamorro L. Recommendations of the Study Group for Metabolic Alterations/Secretariat for the National AIDS Plan (GEAM/SPNS) on the management of metabolic and morphologic alterations in patients with HIV infection. Enfermedades Infecciosas y Microbiologia Clinica 2006;24(2):96-117. Off topic
Ref ID: 5547

(3912) Polzien KM, Jakicic JM, Tate DF, Otto AD. The efficacy of a technology-based system in a short-term behavioral weight loss intervention. Obesity 2007 April;15(4):825-30. Diet & Exercise intervention
Ref ID: 1250

(3913) Pommerenck Martínez C, Guardado Alvarez MdC, Pérez Bravo ME, Suárez A. Efectos del entrenamiento en mujeres que realizan trabajo sedentario. Revista Cubana de Higiene y Epidemiología 1985 June;23(2):114-23. Study limited to adults
Ref ID: 4450

(3914) Pongcharoen T, Ramakrishnan U, DiGirolamo AM, Winichagoon P, Flores R, Singkhornard J, Martorell R. Influence of Prenatal and Postnatal Growth on Intellectual Functioning in School-aged Children. Archives of Pediatrics and Adolescent Medicine 2012;166(5):411-6. Follow-up Study
Ref ID: 5548

(3915) Pontes JF, Ferreira GMH, Sena-Evangelista KCMd, Dourado Junior ME, Fregonezi G. Força muscular respiratória e perfil postural e nutricional em crianças com doenças neuromusculares. Respiratory muscle strenght, nutritional and postural profile in children with neuromuscular diseases. Fisioterapia em Movimento 2012 June;25(2):253-61. Off topic
Ref ID: 4451

(3916) Pop C, Garabagiu D, Barbulescu B, Daraban D, Schisser G, Schiopu M, Zabreanu M, Rosu R, Fanea A, Popa A, Vlaicu R. [The primary prevention of arterial hypertension. A meta-analysis of controlled clinical studies]. [Romanian]. Revista Medico-Chirurgicala a Societatii de Medici Si Naturalisti Din Iasi 1999 January;103(1-2):77-87. Review article
Ref ID: 2055

(3917) Pope RP, Herbert RD, Kirwan JD, Graham BJ. A randomized trial of preexercise stretching for prevention of lower-limb injury. Medicine and Science

in Sports and Exercise 2000 February;32(2):271-7. Both groups exercised
Ref ID: 1987

(3918) Pope SK, Shue VM, Beck C. Will A Healthy Lifestyle Help Prevent Alzheimer's Disease? Annual Review of Public Health 2003 April;24(1):111. Off topic
Ref ID: 3714

(3919) Popow C, Haschke F, Haber P, Schuster E, Salzer HR. [Influence of sports on development of 10 and 11-year-old boys. II. Spiroergometry]. [German]. Klinische Padiatrie 1984 January;196(1):9-13. Both groups exercised
Ref ID: 2364

(3920) Poprzecki Sa, Zajac A, Chalimoniuk M, Waskiewicz Z, Langfort JÃ. Modification of blood antioxidant status and lipid profile in response to high-intensity endurance exercise after low doses of Ï‰-3 polyunsaturated fatty acids supplementation in healthy volunteers. International Journal of Food Sciences and Nutrition 2009 August 2;60:67-79. Diet Intervention or Supplement Study
Ref ID: 3715

(3921) Portal S, Zadik Z, Rabinowitz J, Pilz-Burstein R, Adler-Portal D, Meckel Y, Cooper DM, Eliakim A, Nemet D. The effect of HMB supplementation on body composition, fitness, hormonal and inflammatory mediators in elite adolescent volleyball players: a prospective randomized, double-blind, placebo-controlled study. European Journal of Applied Physiology 2011 September;111(9):2261-9. Diet Intervention or Supplement Study
Ref ID: 2733

(3922) Porter LS. The impact of physical-physiological activity on infants' growth and development. Nursing Research 1972 May;21(3):210-9. Subjects less than 2 years old
Ref ID: 2734

(3923) Portman RJ, McNiece KL, Swinford RD, Braun MC, Samuels JA. Pediatric hypertension: Diagnosis, evaluation, management, and treatment for the primary care physician. Current Problems in Pediatric and Adolescent Health Care 2005;35(7):262-94. Review article
Ref ID: 3296

(3924) Porto MCV, Brito IC, Calfa AD, Amoras M, Villela N, Araújo LM. Perfil do obeso classe III do ambulatório de obesidade de um hospital universitário de Salvador, Bahia. Arquivos Brasileiros de Endocrinologia and Metabologia 2002 December;46(6):668-73. Retrospective study
Ref ID: 742

(3925) Posada Johnson G. Diabetes knowledge and physical activity increase through child to parent communication. Perspectivas en Nutrición Humana 2006 June;(15):11-29. Educational intervention
Ref ID: 4452

(3926) Posada Lima E, Esquivel Lauzurique M, Rubén Quesada M. Tendencia secular en el desarrollo físico de los niños y adolescentes en Guantánamo. Revista Cubana de Alimentación y Nutrición 1989 April;3(1):9-17. Cross-sectional study
Ref ID: 823

(3927) Poston WS, Haddock CK, Olvera NE, Suminski RR, Reeves RS, Dunn JK, Hanis CL, Foreyt JP. Evaluation of a culturally appropriate intervention to increase physical activity. American Journal of Health Behavior 2001 July;25(4):396-406. Study limited to adults
Ref ID: 395

(3928) Poti JM, Duffey KJ, Popkin BM. The association of fast food consumption with poor dietary outcomes and obesity among children: is it the fast food or the remainder of the diet? American Journal of Clinical Nutrition 2014 January;99(1):162-71. Inappropriate Study Design
Ref ID: 6025

(3929) Potteiger JA, Jacobsen DJ, Donnelly JE, Hill JO. Glucose and insulin responses following 16 months of exercise training in overweight adults: the Midwest Exercise Trial. Metabolism 2003 September;52(9):1175-81. Study limited to adults
Ref ID: 355

(3930) Poulsen AA, Desha L, Ziviani J, Griffiths L, Heaslop A, Khan A, Leong GM. Fundamental movement skills and self-concept of children who are overweight. International Journal of Pediatric Obesity 2011 June;6(2-2):e464-e471. Diet & Exercise intervention
Ref ID: 2735

(3931) Powers SW, Jones JS, Ferguson KS, Piazza WC, Daines C, Acton JD. Randomized clinical trial of behavioral and nutrition treatment to improve energy intake and growth in toddlers and preschoolers with cystic fibrosis. Pediatrics 2005;116:1442-50. Behavior Modification Intervention
Ref ID: 4956

(3932) Poyastro PA, Thornton LM, Plotonicov KH, Tozzi F, Klump KL, Berrettini WH, Brandt H, Crawford S, Crow S, Fichter MM, Goldman D, Halmi KA, Johnson C, Kaplan AS, Keel P, LaVia M, Mitchell J, Rotondo A, Strober M, Treasure J, Woodside DB, Von HA, Hamer R, Kaye WH, Bulik CM. Patterns of menstrual disturbance in eating disorders. International Journal of Eating Disorders 2007 July;40(5):424-34. Off topic
Ref ID: 1217

(3933) Pozzato C, Verduci E, Scaglioni S, Radaelli G, Salvioni M, Rovere A, Cornalba G, Riva E, Giovannini M. Liver fat change in obese children after a 1-year nutrition-behavior intervention. Journal of Pediatric Gastroenterology and

Nutrition 2010 September;51(3):331-5. Diet Intervention Study
Ref ID: 2736

(3934) Pozzobon ME, Glaner MF, Carlet R. Crescimento físico e gordura corporal em meninos de diferentes níveis econômicos. Pensar a Prática (Impresso) 2010 August;13(2):1-14. Retrospective study
Ref ID: 4453

(3935) Prabhakaran B, Dowling EA, Branch JD, Swain DP, Leutholtz BC. Effect of 14 weeks of resistance training on lipid profile and body fat percentage in premenopausal women. British Journal of Sports Medicine 1999 June;33(3):190-5. Study limited to adults
Ref ID: 2016

(3936) Pradhan S, Ghosh D, Srivastava NK, Kumar A, Mittal B, Pandey CM, Singh U. Prednisolone in Duchenne muscular dystrophy with imminent loss of ambulation. Journal of Neurology 2006 October;253(10):1309-16. Drug intervention study
Ref ID: 1331

(3937) Prado DM, Silva AG, Trombetta IC, Ribeiro MM, Nicolau CM, Guazzelli IC, Matos LN, Negrao CE, Villares SM. Weight loss associated with exercise training restores ventilatory efficiency in obese children. International Journal of Sports Medicine 2009 November;30(11):821-6. No exercise only group, All groups not randomized
Ref ID: 99

(3938) Prado DM, Silva AG, Trombetta IC, Ribeiro MM, Guazzelli IC, Matos LN, Santos MS, Nicolau CM, Negrao CE, Villares SM. Exercise training associated with diet improves heart rate recovery and cardiac autonomic nervous system activity in obese children. International Journal of Sports Medicine 2010 December;31(12):860-5. Diet & Exercise intervention
Ref ID: 2737

(3939) Prado WL, Oyama LM, Lofrano-Prado MC, de PA, Stella SG, Nascimento CM, Carnier J, Caranti DA, Tock L, Tufik S, de Mello MT, Damaso AR. Alterations in downstream mediators involved in central control of eating behavior in obese adolescents submitted to a multidisciplinary therapy. Journal of Adolescent Health 2011 September;49(3):300-5. Diet Intervention Study
Ref ID: 2738

(3940) Prado WLd, Siegfried A, Dâmaso AR, Carnier J, Piano Ad, Siegfried W. Efeitos da terapia multidisciplinar de longo prazo sobre a composição corporal de adolescentes internados com obesidade severa. Effects of long-term multidisciplinary inpatient therapy on body composition of severely obese adolescents. Jornal de Pediatria 2009 June;85(3):243-8. Lifestyle Intervention
Ref ID: 4454

(3941) Prakash A, Wagstaff AJ. Domperidone: A Review of its Use in Diabetic Gastropathy. Drugs 1998 September;56(3):429-45. Drug intervention study
Ref ID: 549

(3942) Prapavessis H, McNair PJ. Effects of instruction in jumping technique and experience jumping on ground reaction forces. Journal of Orthopaedic and Sports Physical Therapy 1999 June;29(6):352-6. Off topic
Ref ID: 2018

(3943) Prapavessis H, Cameron L, Baldi JC, Robinson S, Borrie K, Harper T, Grove JR. The effects of exercise and nicotine replacement therapy on smoking rates in women. Addictive Behaviors 2007 July;32(7):1416-32. Study limited to adults
Ref ID: 1241

(3944) Prasad SE, Howley S, Murphy KC. Psychiatric disorders in people with 22q11.2 Deletion Syndrome: A population-based prevalence study in Ireland. Journal of Intellectual Disability Research 2008 October;52(10):816. Off topic
Ref ID: 3852

(3945) Prasad S, Steer C. Switching from Neurostimulant Therapy to Atomoxetine in Children and Adolescents with Attention-Deficit Hyperactivity Disorder: Clinical Approaches and Review of Current Available Evidence. Pediatrics Drugs 2008 February;10(1):39. Review article
Ref ID: 3716

(3946) Prati SRA, Prati ARC. Níveis de aptidão física e análise de tendências posturais em bailarinas clássicas. Revista Brasileira de Cineantropometria e Desempenho Humano 2006 April;8(1). Cross-sectional study
Ref ID: 581

(3947) Pratt CA, Boyington J, Esposito L, Pemberton VL, Bonds D, Kelley M, Yang S, Murray D, Stevens J. Childhood Obesity Prevention and Treatment Research (COPTR): interventions addressing multiple influences in childhood and adolescent obesity. Contemporary Clinical Trials 2013 November;36(2):406-13. Inappropriate Study Design
Ref ID: 6026

(3948) Preiss D, Sattar N. Non-alcoholic fatty liver disease: An overview of prevalence, diagnosis, pathogenesis and treatment considerations. Clinical Science 2008;115(5-6):141-50. Off topic
Ref ID: 3297

(3949) Prentice A. Should lactating women exercise? Nutrition Reviews 52(10):358-60, 1994 Oct 1994;(10):358-60. Study limited to adults
Ref ID: 3103

(3950) Prentice A, Ginty F, Stear SJ, Jones SC, Laskey MA, Cole TJ. Calcium supplementation increases stature and bone mineral mass of 16- to 18-year-old

boys. Journal of Clinical Endocrinology and Metabolism 2005 June;90(6):3153-61. Diet Intervention or Supplement Study
Ref ID: 1512

(3951) Prestes J, Frollini AB, de LC, Donatto FF, Foschini D, de Cassia MR, Figueira A, Jr., Fleck SJ. Comparison between linear and daily undulating periodized resistance training to increase strength. Journal of Strength and Conditioning Research 2009 December;23(9):2437-42. Study limited to adults
Ref ID: 630

(3952) Price JH, Desmond SM, Ruppert ES, Stelzer CM. Pediatricians' perceptions and practices regarding childhood obesity. American Journal of Preventive Medicine 1989 March;5(2):95-103. Survey or questionnaire
Ref ID: 2320

(3953) Prins RG, Brug J, van EP, Oenema A. Effectiveness of YouRAction, an intervention to promote adolescent physical activity using personal and environmental feedback: a cluster RCT. PLoS ONE [Electronic Resource] 2012;7(3):e32682. Not All Participants were Overweight and/or Obese
Ref ID: 2739

(3954) Prior RL, Wu X. Anthocyanins: Structural characteristics that result in unique metabolic patterns and biological activities*. Free Radical Research 2006 October;40(10):1014-28. Drug intervention study
Ref ID: 3717

(3955) Pritzlaff-Roy CJ, Widemen L, Weltman JY, Abbott R, Gutgesell M, Hartman ML, Veldhuis JD, Weltman A. Gender governs the relationship between exercise intensity and growth hormone release in young adults. Journal of Applied Physiology 2002 May;92(5):2053-60. Study limited to adults
Ref ID: 1840

(3956) Probst JÃ, Lawler P. Around the World. Modern Athlete and Coach 2011 October;49(4):41-3. Off topic
Ref ID: 3893

(3957) Prochaska JJ, Sallis JF. A randomized controlled trial of single versus multiple health behavior change: promoting physical activity and nutrition among adolescents. Health Psychology 2004 May;23(3):314-8. Not All Participants were Overweight and/or Obese
Ref ID: 1641

(3958) Procter-Gray E, Cobb KL, Crawford SL, Bachrach LK, Chirra A, Sowers M, Greendale GA, Nieves JW, Kent K, Kelsey JL. Effect of oral contraceptives on weight and body composition in young female runners. Medicine and Science in Sports and Exercise 2008 July;40(7):1205-12. Drug intervention study
Ref ID: 929

(3959) Przkora R, Herndon DN, Suman OE. The effects of oxandrolone and exercise on muscle mass and function in children with severe burns. Pediatrics 2007 January;119(1):e109-e116. Drug intervention study
Ref ID: 1288

(3960) Puciato D, Mynarski W, Rozpara M, Borysiuk Z, Szygula R. Motor development of children and adolescents aged 8-16 years in view of their somatic build and objective quality of life of their families. Journal of Human Kinetics 2011;28:45-53. Cross-sectional study
Ref ID: 5549

(3961) Pudel V. What does humans motivate to observe a healthy diet? Part 2: Principles of marketing applied to dietary counselling, and limits to counselling. Ernahrungs-Umschau 2007;54(7):373-+. Diet Intervention Study
Ref ID: 5550

(3962) Puder JJ, Marques-Vidal P, Schindler C, Zahner L, Niederer I, Burgi F, Ebenegger V, Nydegger A, Kriemler S. Effect of multidimensional lifestyle intervention on fitness and adiposity in predominantly migrant preschool children (Ballabeina): cluster randomised controlled trial. British Medical Journal 2011;343:d6195. Lifestyle Intervention
Ref ID: 1079

(3963) Puder JJ, Schindler C, Zahner L, Kriemler S. Adiposity, fitness and metabolic risk in children: a cross-sectional and longitudinal study. International Journal of Pediatric Obesity 2011 June;6(2-2):e297-e306. Cross-sectional study
Ref ID: 2741

(3964) Puhl CH, Pereira LDC, Grisard N, Hallal ALC. Morbimortalidade do recém-nascido de mãe adolescente. ACM Arquivos Catarinense de Medicina 2007;36(3):52-8. Off topic
Ref ID: 586

(3965) Punnett L, Pruss-Ustun A, Nelson DI, Fingerhut MA, Leigh J, Tak S, Phillips S. Estimating the global burden of low back pain attributable to combined occupational exposures. American Journal of Industrial Medicine 2005;48(6):459-69. Off topic
Ref ID: 5551

(3966) Punthakee Z, Delvin EE, O'Loughlin J, Paradis G, Levy E, Platt RW, Lambert M. Adiponectin, adiposity, and insulin resistance in children and adolescents. Journal of Clinical Endocrinology and Metabolism 2006;91(6):2119-25. Cross-sectional study
Ref ID: 5552

(3967) Quadros TMBd, Gordia AP, Pires Neto CS, Leite MdL, Campos Wd, Kalinowski FG. Crescimento físico de escolares da rede particular de ensino do município de Ponta Grossa, PR. Revista Brasileira de Cineantropometria e Desempenho

Humano 2006 September;8(3). Cross-sectional study
Ref ID: 4455

(3968) Quinn L. Type 2 diabetes - Epidemiology, pathophysiology, and diagnosis. Nursing Clinics of North America 2001;36(2):175-+. Review article
Ref ID: 5553

(3969) Quinn SM, Baur LA, Garnett SP, Cowell CT. Treatment of clinical insulin resistance in children: a systematic review. [Review]. Obesity Reviews 2010 October;11(10):722-30. Review article
Ref ID: 2742

(3970) Quintana LM, Heinz LN, Portes LA, Alfieri FM. Influência do nível de atividade física na dismenorréia. Influence of physical activity in dysmenorrhea. Revista Brasileira de Atividade Física e Saúde 2010 June;15(2). Off topic
Ref ID: 4456

(3971) Rabbia F, Grosso T, Cat GG, Conterno A, De VB, Mulatero P, Chiandussi L, Veglio F. Assessing resting heart rate in adolescents: determinants and correlates. Journal of Human Hypertension 2002;16:327-32. Cross-sectional study
Ref ID: 4957

(3972) Rabelo LM, Viana RM, Schimith MA, Patin RV, Valverde MA, Denadai RC, Cleary AP, Lemes S, Fisberg M, Martinez TLdR. Fatores de risco para doença aterosclerótica em estudantes de uma Universidade privada em Säo Paulo – Brasil. Risk factors for atherosclerosis in students of a private University in Säo Paulo - Brazil. Arquivos Brasileiros de Cardiologia 1999 May;72(5):569-80. Cross-sectional study
Ref ID: 4457

(3973) Racette SB, Schoeller DA, Kushner RF. Comparison of heart-rate and physical-activity recall with doubly labeled water in obese women. Medicine and Science In Sports and Exercise 1995;27(1):126-33. Study limited to adults
Ref ID: 5555

(3974) Racette SB, Schoeller DA, Kushner RF, Neil KM. Exercise enhances dietary compliance during moderate energy restriction in obese women. American Journal of Clinical Nutrition 1995;62(2):345-9. Study limited to adults
Ref ID: 5554

(3975) Racil G, Ben OO, Hammouda O, Kallel A, Zouhal H, Chamari K, Amri M. Effects of high vs. moderate exercise intensity during interval training on lipids and adiponectin levels in obese young females. European Journal of Applied Physiology 2013 October;113(10):2531-40. Inappropriate Outcomes
Ref ID: 6027

(3976) Rafferty K, Watson P, Lappe JM. The selection and prevalence of natural and fortified calcium food sources in the diets of adolescent girls. Journal of Nutrition Education And Behavior 2011;43:96-102. Diet Intervention Study
Ref ID: 4958

(3977) Rafiq N, Younossi ZM. Effects of weight loss on nonalcoholic fatty liver disease. Seminars in Liver Disease 2008;28(4):427-33. Off topic
Ref ID: 3299

(3978) Raitakari OT, Porkka KV, Rasanen L, Ronnemaa T, Viikari JS. Clustering and six year cluster-tracking of serum total cholesterol, HDL-cholesterol and diastolic blood pressure in children and young adults. The Cardiovascular Risk in Young Finns Study. Journal of Clinical Epidemiology 1994 October;47(10):1085-93. Cross-sectional study
Ref ID: 2220

(3979) Raitakari OT, Porkka KV, Taimela S, Telama R, Rasanen L, Viikari JS. Effects of persistent physical activity and inactivity on coronary risk factors in children and young adults. The Cardiovascular Risk in Young Finns Study. American Journal of Epidemiology 1994 August 1;140(3):195-205. Survey or questionnaire
Ref ID: 2241

(3980) Raizman DJ, Montgomery DH, Osganian SK, Ebzery MK, Evans MA, Nicklas TA, Zive MM, Hann BJ, Snyder MP, Clesi AL. CATCH: food service program process evaluation in a multicenter trial. Health Education Quarterly 1994;Suppl 2:S51-S71. Off topic
Ref ID: 4959

(3981) Rakusan K, Flanagan MF, Geva T, Southern J, Vanpraagh R. Morphometry of Human Coronary Capillaries During Normal Growth and the Effect of Age in Left-Ventricular Pressure-Overload Hypertrophy. Circulation 1992;86(1):38-46. Off topic
Ref ID: 5556

(3982) Ralph AP, Ardian M, Wiguna A, Maguire GP, Becker NG, Drogumuller G, Wilks MJ, Waramori G, Tjitra E, Sandjaja, Kenagalem E, Pontororing GJ, Anstey NM, Kelly PM. A simple, valid, numerical score for grading chest x-ray severity in adult smear-positive pulmonary tuberculosis. Thorax 2010 October;65(10):863-9. Off topic
Ref ID: 2743

(3983) Ramalho AC, de Lourdes LM, Nunes F, Cambui Z, Barbosa C, Andrade A, Viana A, Martins M, Abrantes V, Aragao C, Temistocles M. The effect of resistance versus aerobic training on metabolic control in patients with type-1 diabetes mellitus. Diabetes Research and Clinical Practice 2006

June;72(3):271-6. Not a randomized controlled trial (RCT)
Ref ID: 1390

(3984) Ramalho A, Gardotti CM, Borges MBF, Oliveira MD, Palavras M, Nunes P, Sampaio SMD. Imagem corporal na adolescência. Junguiana 2007;(25):133-42. Off topic
Ref ID: 4458

(3985) Ramalho MHdS, Santos JOLd, Soares AdA, Machado Z, Maria WB, Nazario PF, Nobre GC. Crescimento físico de crianças e adolescentes de três cidades brasileiras. Physical growth in children and adolescents of three Brazilian cities. Journal of the Health Sciences Institute 2011 December;29(4). Cross-sectional study
Ref ID: 4459

(3986) Raman A, Ritchie LD, Lustig RH, Fitch MD, Hudes ML, Fleming SE. Insulin resistance is improved in overweight African American boys but not in girls following a one-year multidisciplinary community intervention program. Journal of Pediatric Endocrinology 2010 January;23(1-2):109-20. Not a randomized controlled trial (RCT)
Ref ID: 539

(3987) Ramirez-Marrero FA, Smith BA, Sherman WM, Kirby TE. Comparison of methods to estimate physical activity and energy expenditure in African American children. International Journal of Sports Medicine 2005 June;26(5):363-71. Off topic
Ref ID: 1518

(3988) Ramírez CM, Bravo J, Díaz Y, Mora AM, Mestre M, Piñeiro G. Ingesta de energía y algunos nutrientes en un grupo de levantadores de pesas. Revista Cubana de Alimentación y Nutrición 1990 April;4(1):120-5. Diet Intervention Study
Ref ID: 4460

(3989) Ramírez I, Bellabarba A, Paoli-Valeri M, Arata-Bellabarba G. Frecuencia de obesidad y sobrepeso en escolares de la zona urbana de Mérida-Venezuela. Revista Venezolana de Endocrinología y Metabolismo 2004 September;2(3):16-21. Observational study
Ref ID: 4461

(3990) Ramos Bermúdez S, Alzate Salazar DA, Ayala Zuluaga JE, Franco Jiménez AM, Sánchez Valencia JA. Perfil de fitness de los estudiantes de la Universidad de Caldas. Perfil de fitness dos estudantes da Universidade de Caldast. Hacia Promocion de la salud 2009 June;14(1):23-34. Observational study
Ref ID: 4462

(3991) Ramos Parrací CA, Monje Mahecha J, López Laiseca JD, Figueroa Calderón CJ. Influencia de la cátedra de educación física frente a los estilos de vida de la

población escolar del departamento del Huila. Influence of the physical education subject area vs lifestyles among school-age population in the department of Huila. Educacion Fisica Deporte 2010 June;29(1):67-74. Cross-sectional study
Ref ID: 4463

(3992) Ramos E, Md SGT, Conde JG, Baez-Cordova JA, Guzman-Villar B, Lopategui-Corsino E, Frontera WR. Anaerobic Power and Muscle Strength in Human Immunodeficiency Virus-Positive Preadolescents. Physical Medicine and Rehabilitation 2012;4(3):171-5. Cross-sectional study
Ref ID: 5557

(3993) Ramos R, Cruz F, Pérez M, Salvatierra M, Robles C, Koletzko BUV, Decsi T, Campoy C. Predicción del desarrollo mental a los 20 meses de edad por medio de la evaluación del desarrollo psicomotor a los seis meses de vida en niños sanos. Psychomotor development at six months predicts mental development at 20 months in healthy children. Salud Mental 2008 February;31(1):53-61. Subjects less than 2 years old
Ref ID: 4464

(3994) Ramstrand N, Andersson CB, Rusaw D. Effects of an unstable shoe construction on standing balance in children with developmental disabilities: a pilot study. Prosthetics and Orthotics International 2008 December;32(4):422-33. Off topic
Ref ID: 867

(3995) Ranby KW, Aiken LS, Mackinnon DP, Elliot DL, Moe EL, McGinnis W, Goldberg L. A mediation analysis of the ATHENA intervention for female athletes: prevention of athletic-enhancing substance use and unhealthy weight loss behaviors. Journal of Pediatric Psychology 2009 November;34(10):1069-83. Off topic
Ref ID: 650

(3996) Rand CM, Auinger P, Klein JD, Weitzman M. Preventive counseling at adolescent ambulatory visits. Journal of Adolescent Health 2005;37(2):87-93. Off topic
Ref ID: 5558

(3997) Randerath W, Bauer M, Blau A, Fietze I, Galetke W, Hein H, Maurer JT, Orth M, Rasche K, Ruhle KH, Sanner B, Stuck BA, Verse T. Relevance of non-cpap treatment options in the therapy of the obstructive sleep apnoea syndrome. (English). Somnologie 2006 May;10(2):68-98. Off topic
Ref ID: 3718

(3998) Ranheim B, Haga HA. Local anaesthesia for pigs subject to castration. Acta Veterinaria Scandinavica 2006 January 2;48:S13. Animal study
Ref ID: 3719

(3999) Rani MA, Sathiyasekaran BW. Behavioural determinants for obesity: a cross-sectional study among urban adolescents in India. Journal of Preventive Medicine and Public Health 2013 July;46(4):192-200. Inappropriate Study Design
Ref ID: 6028

(4000) Rankin JW, Goldman LP, Puglisi MJ, Nickols-Richardson SM, Earthman CP, Gwazdauskas FC. Effect of post-exercise supplement consumption on adaptations to resistance training. Journal of the American College of Nutrition 2004 August;23(4):322-30. Diet Intervention or Supplement Study
Ref ID: 1600

(4001) Ransdell LB, Taylor A, Oakland D, Schmidt J, Moyer ML, Shultz B. Daughters and mothers exercising together: effects of home- and community-based programs. Medicine and Science in Sports and Exercise 2003;35:286-96. Not All Participants were Overweight and/or Obese
Ref ID: 4960

(4002) Raphaelli CdO, Azevedo MR, Hallal PC. Associação entre comportamentos de risco à saúde de pais e adolescentes em escolares de zona rural de um município do Sul do Brasil. Association between health risk behaviors in parents and adolescents in a rural area in southern Brazil. Cadernos de Saúde Pública 2011 December;27(12):2429-40. Cross-sectional study
Ref ID: 4465

(4003) Rappaport EB, Daskalakis C, Sendecki JA. Using routinely collected growth data to assess a school-based obesity prevention strategy. International Journal of Obesity (London) 2013 January;37(1):79-85. Inappropriate Intervention
Ref ID: 6029

(4004) Raschka C, Zanellato S. [Sports anthropology investigation on female participants of the German Sports-Aerobic-Masters compared to non-sports participating young females]. [German]. Anthropologischer Anzeiger 2003 December;61(4):461-72. Off topic
Ref ID: 1682

(4005) Rasmussen LG, Larsen TM, Mortensen PK, Due A, Astrup A. Effect on 24-h energy expenditure of a moderate-fat diet high in monounsaturated fatty acids compared with that of a low-fat, carbohydrate-rich diet: a 6-mo controlled dietary intervention trial. American Journal of Clinical Nutrition 2007 April;85(4):1014-22. Study limited to adults
Ref ID: 243

(4006) Ratamess NA, Kraemer WJ, Volek JS, Rubin MR, Gomez AL, French DN, Sharman MJ, McGuigan MM, Scheett T, Hakkinen K, Newton RU, Dioguardi F. The effects of amino acid supplementation on muscular performance during

resistance training overreaching. Journal of Strength and Conditioning Research 2003 May;17(2):250-8. Diet Intervention or Supplement Study
Ref ID: 1743

(4007) Raudsepp L, Viira R. Changes in physical activity in adolescent girls: a latent growth modelling approach. Acta Paediatrica 2008 May;97(5):647-52. Survey or questionnaire
Ref ID: 979

(4008) Rauh K, Kunath J, Rosenfeld E, Kick L, Ulm K, Hauner H. Healthy living in pregnancy: a cluster-randomized controlled trial to prevent excessive gestational weight gain - rationale and design of the GeliS study. BMC Pregnancy Childbirth 2014;14:119. Inappropriate Population
Ref ID: 6030

(4009) Rausch HC, Kovalskys I, De Gregorio MJ. Gender differences and a school-based obesity prevention program in Argentina: a randomized trial. Revista Panamericana de Salud Pública 2013 August;34(2):75-82. Inappropriate Intervention
Ref ID: 6031

(4010) Ravussin E, Valencia ME, Esparza J, Bennett PH, Schulz LO. Effects of a traditional lifestyle on obesity in Pima Indians. Diabetes Care 1994 September;17(9):1067-74. Study limited to adults
Ref ID: 2230

(4011) Ravussin E, Gautier JF. Metabolic predictors of weight gain. International Journal of Obesity 1999;23:37-41. Review article
Ref ID: 5559

(4012) Raymond EG, Tafari N, Troendle JF, Clemens JD. Development of a practical screening tool to identify preterm, low-birthweight neonates in Ethiopia. Lancet 1994;344:524-7. Off topic
Ref ID: 4961

(4013) Raynor HA, Kilanowski CK, Esterlis I, Epstein LH. A cost-analysis of adopting a healthful diet in a family-based obesity treatment program. Journal of the American Dietetic Association 2002 May;102(5):645-56. No exercise only group
Ref ID: 381

(4014) Raynor HA, Van Walleghen EL, Bachman JL, Looney SM, Phelan S, Wing RR. Dietary energy density and successful weight loss maintenance. Eating Behaviors 2011 April;12(2):119-25. Secondary analysis
Ref ID: 1060

(4015) Raynor HA, Osterholt KM, Hart CN, Jelalian E, Vivier P, Wing RR. Efficacy of U.S. paediatric obesity primary care guidelines: two randomized trials. Pediatric

Obesity 2012 February;7(1):28-38. Diet Intervention Study
Ref ID: 2745

(4016) Raz I, Hanefeld M, Xu L, Caria C, Williams-Herman D, Khatami H, Sitagliptin S. Efficacy and safety of the dipeptidyl peptidase-4 inhibitor sitagliptin as monotherapy in patients with type 2 diabetes mellitus. Diabetologia 2006 November;49(11):2564-71. Drug intervention study
Ref ID: 1336

(4017) RÃ¶szer Ts, Ricote M. PPARs in the Renal Regulation of Systemic Blood Pressure. PPAR Research 2010 January;1-11. Off topic
Ref ID: 3720

(4018) Rech RR, Halpern R, Costanzi CB, Bergmann MLdA, Alli LR, Airton Pozo de M, Trentin L, Brum LR. Prevalência de obesidade em escolares de 7 a 12 anos de uma cidade Serrana do RS, Brasil. Revista Brasileira de Cineantropometria e Desempenho Humano 2010;12(2). Cross-sectional study
Ref ID: 4466

(4019) Rector RS, Warner SO, Liu Y, Hinton PS, Sun GY, Cox RH, Stump CS, Laughlin MH, Dellsperger KC, Thomas TR. Exercise and diet induced weight loss improves measures of oxidative stress and insulin sensitivity in adults with characteristics of the metabolic syndrome. American Journal of Physiology - Endocrinology and Metabolism 2007 August;293(2):E500-E506. Study limited to adults
Ref ID: 1192

(4020) Reddy BSN, Kochhar AM, Anitha M, Bamezai R. Bloom's syndrome - a first report from India. International Journal of Dermatology 2000;39(10):760-3. Case-Control / Case Study
Ref ID: 5560

(4021) Redman LM, Elkind-Hirsch K, Ravussin E. Aerobic exercise in women with polycystic ovary syndrome improves ovarian morphology independent of changes in body composition. Fertility and Sterility 2011 June 30;95(8):2696-9. Prospective Study
Ref ID: 1022

(4022) Redman LM, Kraus WE, Bhapkar M, Das SK, Racette SB, Martin CK, Fontana L, Wong WW, Roberts SB, Ravussin E. Energy requirements in nonobese men and women: results from CALERIE. American Journal of Clinical Nutrition 2014 January;99(1):71-8. Inappropriate Population
Ref ID: 6032

(4023) Reed JA, Einstein G, Hahn E, Hooker SP, Gross VP, Kravitz J. Examining the impact of integrating physical activity on fluid intelligence and academic performance in an elementary school setting: a preliminary investigation. Journal of Physical Activity and Health 2010 May;7(3):343-51. Not All

Participants were Overweight and/or Obese
Ref ID: 510

(4024) Reed KE, Warburton DE, Macdonald HM, Naylor PJ, McKay HA. Action Schools! BC: a school-based physical activity intervention designed to decrease cardiovascular disease risk factors in children. Preventive Medicine 2008 June;46(6):525-31. Not All Participants were Overweight and/or Obese
Ref ID: 935

(4025) Reed MD, McCombie BE, Sivillo AE, Thorne PS, Welsh MJ, March TH, McDonald JD, Seilkop SK, Zabner J, Durairaj L. Safety assessment of nebulized xylitol in beagle dogs. Inhalation Toxicology 2012;24(6):365-72. Animal study
Ref ID: 5561

(4026) Reed ME, Ben-Ezra V, Biggerstaff KD, Nichols DL. The effects of two bouts of high- and low-volume resistance exercise on glucose tolerance in normoglycemic women. Journal of Strength and Conditioning Research 2012 January;26(1):251-60. Study limited to adults
Ref ID: 2747

(4027) Rees L, Shaw V. Nutrition in children with CRF and on dialysis. Pediatric Nephrology 2007 October;22(10):1689-702. Diet Intervention Study
Ref ID: 3721

(4028) Reger B, Wootan MG, Booth BS, Smith H. 1% or less: a community-based nutrition campaign. Public Health Reports 1998;113:410-9. Diet Intervention Study
Ref ID: 4962

(4029) Rego RA, Berardo FA, Rodrigues SS, Oliveira ZM, Oliverira MB, Vasconcellos C, Aventurato LV, Moncau JE, Ramos LR. [Risk factors for chronic non-communicable diseases: a domiciliary survey in the municipality of Sao Paulo, SP (Brazil). Methodology and preliminary results]. [Portuguese]. Revista de Saude Publica 1990 August;24(4):277-85. Off topic
Ref ID: 2302

(4030) Reid CM, Maher T, Jennings GL, Heart Project Steering Committee. Substituting lifestyle management for pharmacological control of blood pressure: A pilot study in Australian general practice. Blood Pressure 2000;9(5):267-74. Lifestyle Intervention
Ref ID: 1942

(4031) Reid S, Hamer P, Alderson J, Lloyd D. Neuromuscular adaptations to eccentric strength training in children and adolescents with cerebral palsy. Developmental Medicine and Child Neurology 2010 April;52(4):358-63. Not All Participants were Overweight and/or Obese
Ref ID: 543

(4032) Reilly ET, Freeman RM, Waterfield MR, Waterfield AE, Steggles P, Pedlar F. Prevention of postpartum stress incontinence in primigravidae with increased bladder neck mobility: a randomised controlled trial of antenatal pelvic floor exercises. BJOG: An International Journal of Obstetrics and Gynaecology 2002 January;109(1):68-76. Off topic
Ref ID: 1847

(4033) Reilly JJ, McDowell ZC. Physical activity interventions in the prevention and treatment of paediatric obesity: systematic review and critical appraisal. [Review] [48 refs]. Proceedings of the Nutrition Society 2003 August;62(3):611-9. Review article
Ref ID: 1684

(4034) Reilly JJ, Kelly L, Montgomery C, Williamson A, Fisher A, McColl JH, Lo CR, Paton JY, Grant S. Physical activity to prevent obesity in young children: cluster randomised controlled trial. British Medical Journal 2006 November 18;333(7577):1041. Not All Participants were Overweight and/or Obese
Ref ID: 267

(4035) Reimers TM, Brown KM, Van HL, Stevens V, Obarzanek E, Hartmuller VW, Snetselaar L, von-Almen TK, Chiostri J. Maternal acceptability of a dietary intervention designed to lower children's intake of saturated fat and cholesterol: the Dietary Intervention Study in Children (DISC). Journal of the American Dietetic Association 1998;98:31-4. Diet Intervention Study
Ref ID: 4963

(4036) Reimund JM, Rahmi G, Escalin G, Pinna G, Finck G, Muller CD, Duclos B, Baumann R. Efficacy and safety of an olive oil-based intravenous fat emulsion in adult patients on home parenteral nutrition. Alimentary Pharmacology and Therapeutics 2005 February 15;21(4):445-54. Diet Intervention or Supplement Study
Ref ID: 3722

(4037) Reinehr T, de SG, Roth CL, Andler W. Androgens before and after weight loss in obese children. Journal of Clinical Endocrinology and Metabolism 2005 October;90(10):5588-95. Cross-sectional study
Ref ID: 1466

(4038) Reinehr T, Kersting M, Wollenhaupt A, Alexy U, Kling B, Strobele K, Andler W. [Evaluation of the training program "OBELDICKS" for obese children and adolescents]. [German]. Klinische Padiatrie 2005 January;217(1):1-8. Lifestyle Intervention
Ref ID: 1552

(4039) Reinehr T, de SG, Wabitsch M. Changes of cardiovascular risk factors in obese children effects of inpatient and outpatient interventions. Journal of Pediatric Gastroenterology and Nutrition 2006 October;43(4):506-11. Not a randomized

controlled trial (RCT)
Ref ID: 465

(4040) Reinehr T, de SG, Andler W. Hyperthyrotropinemia in obese children is reversible after weight loss and is not related to lipids. Journal of Clinical Endocrinology and Metabolism 2006 August;91(8):3088-91. Cross-sectional study
Ref ID: 464

(4041) Reinehr T, de SG, Toschke AM, Andler W. Long-term follow-up of cardiovascular disease risk factors in children after an obesity intervention. American Journal of Clinical Nutrition 2006 September;84(3):490-6. Not a randomized controlled trial (RCT)
Ref ID: 466

(4042) Reinehr T, Temmesfeld M, Kersting M, de SG, Toschke AM. Four-year follow-up of children and adolescents participating in an obesity intervention program. International Journal of Obesity 2007 July;31(7):1074-7. Follow-up Study
Ref ID: 1209

(4043) Reinehr T, Roth CL. Fetuin-A and its relation to metabolic syndrome and fatty liver disease in obese children before and after weight loss. Journal of Clinical Endocrinology and Metabolism 2008;93(11):4479-85. Follow-up Study
Ref ID: 5562

(4044) Reinehr T, de SG, Roth CL. Obestatin and ghrelin levels in obese children and adolescents before and after reduction of overweight. Clinical Endocrinology 2008 February;68(2):304-10. Not All Participants were Overweight and/or Obese
Ref ID: 2748

(4045) Reinehr T, Stoffel-Wagner B, Roth CL. Retinol-binding protein 4 and its relation to insulin resistance in obese children before and after weight loss. Journal of Clinical Endocrinology and Metabolism 2008 June;93(6):2287-93. Lifestyle Intervention, Follow-up Study
Ref ID: 453

(4046) Reinehr T, Widhalm K, l'Allemand D, Wiegand S, Wabitsch M, Holl RW, APV-Wiss STudy Group and German Competence Net Obesity. Two-year follow-up in 21,784 overweight children and adolescents with lifestyle intervention. Obesity 2009 June;17(6):1196-9. Follow-up Study
Ref ID: 720

(4047) Reinehr T, Kleber M, Toschke AM. Lifestyle intervention in obese children is associated with a decrease of the metabolic syndrome prevalence. Atherosclerosis 2009 November;207(1):174-80. Lifestyle Intervention
Ref ID: 648

(4048) Reinehr T, Kleber M, de SG, Andler W. Leptin concentrations are a predictor of overweight reduction in a lifestyle intervention. International Journal of Pediatric Obesity 2009;4(4):215-23. Lifestyle Intervention
Ref ID: 643

(4049) Reinehr T, Roth CL, Enriori PJ, Masur K. Changes of dipeptidyl peptidase IV (DPP-IV) in obese children with weight loss: relationships to peptide YY, pancreatic peptide, and insulin sensitivity. Journal of Pediatric Endocrinology 2010 January;23(1-2):101-8. Lifestyle Intervention
Ref ID: 540

(4050) Reinehr T, Roth CL. A new link between skeleton, obesity and insulin resistance: relationships between osteocalcin, leptin and insulin resistance in obese children before and after weight loss. International Journal of Obesity 2010;34(5):852-8. Not All Participants were Overweight and/or Obese
Ref ID: 5563

(4051) Reinehr T, Schaefer A, Winkel K, Finne E, Toschke AM, Kolip P. An effective lifestyle intervention in overweight children: findings from a randomized controlled trial on "Obeldicks light". Clinical Nutrition 2010 June;29(3):331-6. No exercise only group
Ref ID: 68

(4052) Reinehr T, Kleber M, Toschke AM. Former small for gestational age (SGA) status is associated to changes of insulin resistance in obese children during weight loss. Pediatric Diabetes 2010 September;11(6):431-7. Follow-up Study
Ref ID: 469

(4053) Reinehr T, Woelfle J, Wunsch R, Roth CL. Fibroblast growth factor 21 (FGF-21) and its relation to obesity, metabolic syndrome, and nonalcoholic fatty liver in children: a longitudinal analysis. Journal of Clinical Endocrinology and Metabolism 2012 June;97(6):2143-50. Lifestyle Intervention
Ref ID: 2749

(4054) Reinehr T. Lifestyle intervention in childhood obesity: changes and challenges. Nature Reviews Endocrinology 2013 October;9(10):607-14. Inappropriate Study Design
Ref ID: 6033

(4055) Reiner Å, Catapano AL, De Backer G, Graham I, Taskinen MR, Wiklund O, Agewall S, Alegria E, Chapman MJ, Durrington P, Erdine S, Halcox J, Hobbs R, Kjekshus J, Filardi PP, Riccardi G, Storey RF, Wood D. ESC/EAS Guidelines for the management of dyslipidaemias. European Heart Journal 2011;32(14):1769-818. Off topic
Ref ID: 3300

(4056) Reis HFCd, Ladeia AMT, Passos EC, Santos FGdO, Wasconcellos LTd, Correia LCL, Menezes MS, Santos RDG, Bomfim VGd, Rocha MdS.

Prevalência e variáveis associadas à inatividade física em indivíduos de alto e baixo nível socioeconômico. Prevalence and variables associated with physical inactivity in individuals with high and low socioeconomic status. Prevalencia y variables asociadas a la inactividad física en individuos de alto y bajo nivel socioeconómico. Arquivos brasileiros de cardiologia 2009 March;92(3):203-8. Survey or questionnaire
Ref ID: 4467

(4057) Reis VAdB, Azevedo COEd, Rossi L. Perfil antropométrico e taxa de sudorese no futebol juvenil. Revista Brasileira de Cineantropometria e Desempenho Humano 2009 April 29;11(2):134-41. Off topic
Ref ID: 4468

(4058) Reisler G, Tauber T, Afriat R, Bortnik O, Goldman M. Sibutramine as an adjuvant therapy in adolescents suffering from morbid obesity. Israel Medical Association Journal: Imaj 2006 January;8(1):30-2. Drug intervention study
Ref ID: 1425

(4059) Remes V, Poussa M, Lonnqvist T, Puusa A, Tervahartiala P, Helenius I, Peltonen J. Walking ability in patients with diastrophic dysplasia: a clinical, electroneurophysiological, treadmill, and MRI analysis. Journal of Pediatric Orthopedics 2004 September;24(5):546-51. Off topic
Ref ID: 1601

(4060) Rennie KL, Livingstone MB, Wells JC, McGloin A, Coward WA, Prentice AM, Jebb SA. Association of physical activity with body-composition indexes in children aged 6-8 y at varied risk of obesity. American Journal of Clinical Nutrition 2005 July;82(1):13-20. Cross-sectional study
Ref ID: 1499

(4061) Resende AC, Argimon IIdL. Obesidade infantil: aspectos psicológicos que transitam no âmbito sociocultural e familiar, prevenção e tratamento. Temas sobre Desenvolvimento 2008 June;16(92):85-91. Review article
Ref ID: 4469

(4062) Resnick B. Prescribing an Exercise Program and Motivating Older Adults to Comply. Educational Gerontology 2001 April;27(3/4):209-26. Study limited to adults
Ref ID: 3853

(4063) Resnick EA, Bishop M, O'Connell A, Hugo B, Isern G, Timm A, Ozonoff A, Geller AC. The CHEER study to reduce BMI in Elementary School students: a school-based, parent-directed study in Framingham, Massachusetts. Journal of School Nursing 2009 October;25(5):361-72. No exercise only group
Ref ID: 110

(4064) Resnicow K, Taylor R, Baskin M, McCarty F. Results of go girls: a weight control program for overweight African-American adolescent females. Obesity

Research 2005 October;13(10):1739-48. No exercise only group, No comparative control group
Ref ID: 293

(4065) Restiffe AP, Gherpelli JL. Differences in walking attainment ages between low-risk preterm and healthy full-term infants. Diferenças na idade de aquisição da marcha entre lactentes pré-termo de baixo risco e a termo saudáveis. Arquivos de Neuro-Psiquiatria 2012 August;70(8):593-8. Subjects less than 2 years old
Ref ID: 4470

(4066) Restrepo M. Percepciones frente a la alimentación y nutrición del escolar. Perspectivas en Nutrición Humana 2007 June;9(1):23-35. Not a randomized controlled trial (RCT)
Ref ID: 4471

(4067) Rexhepi A, Brestovci B. Differences in bodily growth between young footballers and basketball players. Diferencias en el crecimiento corporal entre futbolistas y jugadores de baloncesto jóvenes. International Journal of Morphology 2010 June;28(2):415-20. Not a randomized controlled trial (RCT)
Ref ID: 4472

(4068) Reyes Baez G, Gonzalez AR, Gomez Raspaldo G, Blasini I, Rivera CE. Comparative study of the effectiveness of thyroxine and steroids on reduction of neonatal morbidity: outcome at 20 months follow-up. Puerto Rico Health Sciences Journal 2004 March;23(1):9-12. Subjects less than 2 years old
Ref ID: 707

(4069) Reyes J, Díaz B, Lera M, BURROWS A. Ingesta y metabolismo energético en una muestra de adolescentes chilenos con sobrepeso y obesidad.
Intake and energy metabolism in a sample of overweight and obese Chilean adolescents. Revista Médica de Chile 2011 April;139(4):425-31. Diet Intervention Study
Ref ID: 4473

(4070) Reyes JF, Vargas R, Kumar D, Cullen EI, Perdomo CA, Pratt RD. Steady-state pharmacokinetics, pharmacodynamics and tolerability of donepezil hydrochloride in hepatically impaired patients. British Journal of Clinical Pharmacology 2004;58 Suppl 1:9-17. Drug intervention study
Ref ID: 4965

(4071) Rezende DFd, Scarpelli RAB, Souza GFd, Costa JOd, Scarpelli AMB, Scarpelli PA, Carvalho GBd, D'agostini HM, Pedrosa JC. Prevalência da hipertensäo arterial sistêmica em escolares de 7 a 14 anos do município de Barbacena, Minas Gerais, em 1999. Prevalence of systemic hypertension in students aged 7 to 14 years in the municipality of Barbacena, in the State of Minas Gerais, in 1999. Arquivos Brasileiros de Cardiologia 2003 October;81(4):375-86.

Prevalence study
Ref ID: 4474

(4072) Rezvanian H, Hashemipour M, Kelishadi R, Tavakoli N, Poursafa P. A randomized, triple masked, placebo-controlled clinical trial for controlling childhood obesity. World Journal of Pediatrics 2010 November;6(4):317-22. Drug intervention study
Ref ID: 2750

(4073) Rêgo ALV, Chiara VL. Nutrição e excesso de massa corporal: fatores de risco cardiovascular em adolescentes. Revista de Nutrição 2006 December;19(6):705-12. Cross-sectional study
Ref ID: 4475

(4074) Rhea MR, Ball SD, Phillips WT, Burkett LN. A comparison of linear and daily undulating periodized programs with equated volume and intensity for strength. Journal of Strength and Conditioning Research 2002 May;16(2):250-5. Study limited to adults
Ref ID: 1833

(4075) Rhodes ET, Wolfsdorf JI, Cuthbertson DD, Feldman HA, Ludwig DS, -Study-Group. Effect of low-dose insulin treatment on body weight and physical development in children and adolescents at risk for type 1 diabetes. Diabetes Care 2005;28:1948-53. Secondary analysis
Ref ID: 4966

(4076) Ribaya-Mercado JD. Influence of Dietary Fat on Î²-Carotene Absorption and Bioconversion into Vitamin A. Nutrition Reviews 2002 April;60(4):104. Diet Intervention Study
Ref ID: 3723

(4077) Ribeiro LF, Lima MC, Gobatto CA. Changes in physiological and stroking parameters during interval swims at the slope of the d-t relationship. Journal of Science and Medicine in Sport 2010 January;13(1):141-5. Off topic
Ref ID: 590

(4078) Ribeiro MM, Silva AG, Santos NS, Guazzelle I, Matos LN, Trombetta IC, Halpern A, Negrao CE, Villares SM. Diet and exercise training restore blood pressure and vasodilatory responses during physiological maneuvers in obese children. Circulation 2005 April 19;111(15):1915-23. No exercise only group, No comparative control group
Ref ID: 310

(4079) Ribeiro RQC, Lotufo PA, Lamounier JA, Oliveira RG, Soares JF, Botter DA. Fatores adicionais de risco cardiovascular associados ao excesso de peso em crianças e adolescentes: o estudo do coração de Belo Horizonte. Arquivos Brasileiros de Cardiologia 2006 June;86(6):408-18. Cross-sectional study
Ref ID: 665

(4080) Ribeiro SML, Santos ZAd, Silva RJd, Louzada E, Donato Junior J, Tirapegui J. Leptina: aspectos sobre o balanço energético, exercício físico e amenorréia do esforço. Arquivos Brasileiros de Endocrinologia and Metabologia 2007 February;51(1):11-24. Review article
Ref ID: 4476

(4081) Ricardo DR, Araújo CGSd. Teste de sentar-levantar: influência do excesso de peso corporal em adulto. Revista Brasileira de Medicina do Esporte 2001 April;7(2):45-52. Study limited to adults
Ref ID: 4477

(4082) Richardson NJ, Rogers PJ, Elliman NA. Conditioned flavour preferences reinforced by caffeine consumed after lunch. Physiology and Behavior 1996;60(1):257-63. Diet Intervention or Supplement Study
Ref ID: 5564

(4083) Richter M, Zech S. [Intraoperative pedography]. Operative Orthopädie und Traumatologie 2010;22:44-51. Off topic
Ref ID: 4967

(4084) Riddoch CJ, Leary SD, Ness AR, Blair SN, Deere K, Mattocks C, Griffiths A, Davey SG, Tilling K. Prospective associations between objective measures of physical activity and fat mass in 12-14 year old children: the Avon Longitudinal Study of Parents and Children (ALSPAC). British Medical Journal 2009;339:b4544. Cohort Study
Ref ID: 638

(4085) Ridgers ND, Stratton G, Fairclough SJ. Assessing physical activity during recess using accelerometry. Preventive Medicine 2005 July;41(1):102-7. Not a randomized controlled trial (RCT)
Ref ID: 1510

(4086) Ridgers ND, Carter LM, Stratton G, McKenzie TL. Examining children's physical activity and play behaviors during school playtime over time. Health Education Research 2011 August;26(4):586-95. Not a randomized controlled trial (RCT)
Ref ID: 2751

(4087) Ridgway CL, Brage S, Anderssen S, Sardinha LB, Andersen LB, Ekelund U. Fat-free mass mediates the association between birth weight and aerobic fitness in youth. International Journal of Pediatric Obesity 2011 June;6(2-2):e590-e596. Cohort Study
Ref ID: 2752

(4088) Riedel BW, Robinson LA, Klesges RC, McLain AB. What motivates adolescent smokers to make a quit attempt? Drug and Alcohol Dependence 2002;68:167-74. Off topic
Ref ID: 4968

(4089) Rieder J, Khan UI, Heo M, Mossavar-Rahmani Y, Blank AE, Strauss T, Viswanathan N, Wylie-Rosett J. Evaluation of a community-based weight management program for predominantly severely obese, difficult-to-reach, inner-city minority adolescents. Childhood Obesity 2013 August;9(4):292-304. Inappropriate Intervention
Ref ID: 6034

(4090) Rieth MA, Moreira MB, Fuchs FD, Moreira LB, Fuchs SC. Fruits and vegetables intake and characteristics associated among adolescents from Southern Brazil. Nutrition Journal 2012;11:95. Inappropriate Intervention
Ref ID: 6035

(4091) Rigamonti AE, Agosti F, De CA, Marazzi N, Lafortuna CL, Cella SG, Muller EE, Sartorio A. Changes in plasma levels of ghrelin, leptin, and other hormonal and metabolic parameters following standardized breakfast, lunch, and physical exercise before and after a multidisciplinary weight-reduction intervention in obese adolescents. Journal of Endocrinological Investigation 2010 October;33(9):633-9. Study less than 4 weeks
Ref ID: 2753

(4092) Rigol JL, Espírito Santo LCd. Perfil das gestantes adolescentes atendidas em consulta de enfermagem. Revista Gaúcha de Enfermagem 2001 July;22(2):122-40. Review article
Ref ID: 738

(4093) Rigon F, Bianchin L, Bernasconi S, Bona G, Bozzola M, Buzi F, Cicognani A, De SC, De S, V, Radetti G, Tato L, Tonini G, Perissinotto E. Update on age at menarche in Italy: toward the leveling off of the secular trend. Journal of Adolescent Health 2010 March;46(3):238-44. Off topic
Ref ID: 584

(4094) Rinaldi AE, Pereira AF, Macedo CS, Mota JF, Burini RC. Contribuições das práticas alimentares e inatividade física para o excesso de peso infantil: [revisão]. Revista Paulista de Pediatria 2008 September;26(3):271-7. Review article
Ref ID: 4478

(4095) Ripsin CM, Kang H, Urban RJ. Management of blood glucose in type 2 diabetes mellitus. American Family Physician 2009 January 1;79(1):29-36. Review article
Ref ID: 813

(4096) Risica PM, Gans KM, Kumanyika S, Kirtania U, Lasater TM. SisterTalk: final results of a culturally tailored cable television delivered weight control program for Black women. International Journal of Behavioral Nutrition and Physical Activity 2013;10:141. Inappropriate Population
Ref ID: 6036

(4097) Ritchie LD, Spector P, Stevens MJ, Schmidt MM, Schreiber GB, Striegel-Moore RH, Wang MC, Crawford PB. Dietary patterns in adolescence are related to adiposity in young adulthood in black and white females. Journal of Nutrition 2007 February;137(2):399-406. Cohort Study
Ref ID: 1283

(4098) Ritchie LD, Sharma S, Ikeda JP, Mitchell RA, Raman A, Green BS, Hudes ML, Fleming SE. Taking Action Together: a YMCA-based protocol to prevent type-2 diabetes in high-BMI inner-city African American children. Trials [Electronic Resource] 2010;11:60. Lifestyle Intervention
Ref ID: 502

(4099) Riva G, Bacchetta M, Baruffi M, Molinari E. Virtual reality-based multidimensional therapy for the treatment of body image disturbances in obesity: a controlled study. CyberPsychology and Behavior 2001 August;4(4):511-26. Study limited to adults
Ref ID: 392

(4100) Rivera-Gallardo MT, Ma del Socorro PC, Barriguete-Melendez JA. [Eating disorders as risk factors for osteoporosis]. [Review] [121 refs] [Spanish]. Salud Publica de Mexico 2005 July;47(4):308-18. Review article
Ref ID: 1458

(4101) Rivera IR, Silva MAMd, Silva RDTA, Oliveira BAVd, Carvalho ACC. Atividade física, horas de assistência à TV e composição corporal em crianças e adolescents. Physical inactivity, TV-watching hours and body composition in children and adolescents. Arquivos Brasileiros de Cardiologia 2010 August;95(2):159-65. Cross-sectional study
Ref ID: 4479

(4102) Rivera JA, Ruel MT, Santizo MC, Lönnerdal B, Brown KH. Zinc supplementation improves the growth of stunted rural Guatemalan infants. Journal of Nutrition 1998;128:556-62. Diet Intervention or Supplement Study
Ref ID: 4969

(4103) Rivera JA, Muñoz-Hernández O, Rosas-Peralta M, Aguilar-Salinas CA, Popkin BM, Willett WC. Consumo de bebidas para una vida saludable: recomendaciones para la población mexicana. Salud Pública de México 2008 April;50(2):173-95. Review article
Ref ID: 4480

(4104) Rivera MF, Rivera IC. Conductas alimentarias y actividad física en niños escolares de Tegucigalpa. Revista Médica Hondureña 2010 June;78(2):65-9. Cross-sectional study
Ref ID: 4481

(4105) Rivero González M, Cabrera Panizo R, Luis Álvarez MdC, Pantoja Pereda O. Alteraciones metabólicas en pacientes obesos y su asociación con la acantosis

nigricans. Metabolic alterations in obese patients and its association with the acanthosis nigricans. Revista Cubana de Pediatria 2012 March;84(1):1-10. Longitudinal Study
Ref ID: 4482

(4106) Riveros Medina MA, Arias Padilla L, Acosta Rodríguez ER, Amaya Alejo SL. Aproximaciones teóricas de los efectos del entrenamiento pliométrico en agua y tierra sobre fuerza muscular y densidad mineral ósea. Revista Colombiana de Rehabilitación 2010 October;9:24-31. Review article
Ref ID: 4483

(4107) Robbins LB, Gretebeck KA, Kazanis AS, Pender NJ. Girls on the move program to increase physical activity participation. Nursing Research 2006 May;55(3):206-16. Primary outcome(s) not assessed
Ref ID: 278

(4108) Roberts CK, Barnard RJ. Effects of exercise and diet on chronic disease. [Review] [424 refs]. Journal of Applied Physiology 2005 January;98(1):3-30. Review article
Ref ID: 1555

(4109) Roberts CK, Chen AK, Barnard RJ. Effect of a short-term diet and exercise intervention in youth on atherosclerotic risk factors. Atherosclerosis 2007 March;191(1):98-106. Diet & Exercise intervention
Ref ID: 1273

(4110) Roberts CK, Croymans DM, Aziz N, Butch AW, Lee CC. Resistance training increases SHBG in overweight/obese, young men. Metabolism 2013 May;62(5):725-33. Inappropriate Population
Ref ID: 6037

(4111) Roberts L, Jones TW, Fournier PA. Exercise training and glycemic control in adolescents with poorly controlled type 1 diabetes mellitus. Journal of Pediatric Endocrinology 2002 May;15(5):621-7. No non-intervention control group
Ref ID: 1827

(4112) Roberts SP, Stokes KA, Trewartha G, Hogben P, Doyle J, Thompson D. Effect of combined carbohydrate-protein ingestion on markers of recovery after simulated rugby union match-play. Journal of Sports Sciences 2011 September;29(12):1253-62. Diet Intervention or Supplement Study
Ref ID: 2754

(4113) Robertson W, Stewart-Brown S, Stallard N, Petrou S, Griffiths F, Thorogood M, Simkiss D, Lang R, Reddington K, Poole F, Rye G, Khan KA, Hamborg T, Kirby J. Evaluation of the effectiveness and cost-effectiveness of Families for Health V2 for the treatment of childhood obesity: study protocol for a randomized controlled trial. Trials 2013;14:81. Inappropriate Study Design
Ref ID: 6038

(4114) Robin AL, Siegel PT, Koepke T, Moye AW, Tice S. Family therapy versus individual therapy for adolescent females with anorexia nervosa. Journal of Developmental and Behavioral Pediatrics 1994 April;15(2):111-6. Off topic
Ref ID: 2240

(4115) Robin AL, Siegel PT, Moye A. Family versus individual therapy for anorexia: impact on family conflict. International Journal of Eating Disorders 1995 May;17(4):313-22. Off topic
Ref ID: 2204

(4116) Robinson M, Eberl S, Tomlinson C, Daviskas E, Regnis JA, Bailey DL, Torzillo PJ, Menache M, Bye PT. Regional mucociliary clearance in patients with cystic fibrosis. Journal of Aerosol Medicine 2000;13(2):73-86. Off topic
Ref ID: 1965

(4117) Robinson TN. Reducing children's television viewing to prevent obesity: a randomized controlled trial. Journal of the American Medical Association 1999 October 27;282(16):1561-7. Not an exercise intervention study
Ref ID: 406

(4118) Robinson TN, Killen JD, Kraemer HC, Wilson DM, Matheson DM, Haskell WL, Pruitt LA, Powell TM, Owens AS, Thompson NS, Flint-Moore NM, Davis GJ, Emig KA, Brown RT, Rochon J, Green S, Varady A. Dance and reducing television viewing to prevent weight gain in African-American girls: the Stanford GEMS pilot study. Ethnicity and Disease 2003;13(1 Suppl 1):S65-S77. No exercise only group
Ref ID: 365

(4119) Robinson TN, Kraemer HC, Matheson DM, Obarzanek E, Wilson DM, Haskell WL, Pruitt LA, Thompson NS, Haydel KF, Fujimoto M, Varady A, McCarthy S, Watanabe C, Killen JD. Stanford GEMS phase 2 obesity prevention trial for low-income African-American girls: design and sample baseline characteristics. Contemporary Clinical Trials 2008 January;29(1):56-69. Description versus conduct of study
Ref ID: 229

(4120) Robinson TN, Matheson DM, Kraemer HC, Wilson DM, Obarzanek E, Thompson NS, Alhassan S, Spencer TR, Haydel KF, Fujimoto M, Varady A, Killen JD. A randomized controlled trial of culturally tailored dance and reducing screen time to prevent weight gain in low-income African American girls: Stanford GEMS. Archives of Pediatrics and Adolescent Medicine 2010 November;164(11):995-1004. Not All Participants were Overweight and/or Obese
Ref ID: 2755

(4121) Robinson TN, Matheson D, Desai M, Wilson DM, Weintraub DL, Haskell WL, McClain A, McClure S, Banda JA, Sanders LM, Haydel KF, Killen JD. Family,

community and clinic collaboration to treat overweight and obese children: Stanford GOALS-A randomized controlled trial of a three-year, multi-component, multi-level, multi-setting intervention. Contemporary Clinical Trials 2013 November;36(2):421-35. Inappropriate Intervention
Ref ID: 6039

(4122) Robroek SJ, van den Berg TI, Plat JF, Burdorf A. The role of obesity and lifestyle behaviours in a productive workforce. Occupational and Environmental Medicine 2011 February;68(2):134-9. Study limited to adults
Ref ID: 2756

(4123) Rocchini AP, Katch V, Schork A, Kelch RP. Insulin and blood pressure during weight loss in obese adolescents. Hypertension 1987 September;10(3):267-73. Lifestyle Intervention
Ref ID: 2338

(4124) Rocchini AP, Katch V, Anderson J, Hinderliter J, Becque D, Martin M, Marks C. Blood pressure in obese adolescents: effect of weight loss. Pediatrics 1988 July;82(1):16-23. No exercise only group
Ref ID: 454

(4125) Rochefort GY, Rocher E, Aveline PC, Garnero P, Bab I, Chappard C, Jaffre C, Benhamou CL. Osteocalcin-insulin relationship in obese children: a role for the skeleton in energy metabolism. Clinical Endocrinology 2011;75(2):265-70. Inappropriate Outcomes
Ref ID: 5565

(4126) Rochon J, Klesges RC, Story M, Robinson TN, Baranowski T, Obarzanek E, Mitchell M. Common design elements of the Girls health Enrichment Multi-site Studies (GEMS). Ethnicity and Disease 2003;13(1:Suppl 1):Suppl-14. Description versus conduct of study
Ref ID: 1755

(4127) Rock CL, Flatt SW, Sherwood NE, Karanja N, Pakiz B, Thomson CA. Effect of a free prepared meal and incentivized weight loss program on weight loss and weight loss maintenance in obese and overweight women: a randomized controlled trial. Journal of the American Medical Association 2010 October 27;304(16):1803-10. Study limited to adults
Ref ID: 20

(4128) Rock M. Diabetes Portrayals in North American Print Media: A Qualitative and Quantitative Analysis. American Journal of Public Health 2005 October;95(10):1832-8. Off topic
Ref ID: 3854

(4129) Rockwell JA, Rankin JW, Toderico B. Creatine supplementation affects muscle creatine during energy restriction. Medicine and Science in Sports and Exercise

2001 January;33(1):61-8. Diet Intervention or Supplement Study
Ref ID: 1934

(4130) Rodearmel SJ, Wyatt HR, Barry MJ, Dong F, Pan D, Israel RG, Cho SS, McBurney MI, Hill JO. A family-based approach to preventing excessive weight gain. Obesity 2006 August;14(8):1392-401. Diet & Exercise intervention
Ref ID: 1346

(4131) Rodearmel SJ, Wyatt HR, Stroebele N, Smith SM, Ogden LG, Hill JO. Small changes in dietary sugar and physical activity as an approach to preventing excessive weight gain: the America on the Move family study. Pediatrics 2007 October;120(4):e869-e879. No exercise only group
Ref ID: 215

(4132) Rodgers WM, Hall CR, Wilson PM, Berry TR. Do nonexercisers also share the positive exerciser stereotype?: An elicitation and comparison of beliefs about exercisers. Journal of Sport and Exercise Psychology 2009 February;31(1):3-17. Off topic
Ref ID: 780

(4133) Rodrigues AN, Moyses MR, Bissoli NS, Pires JGP, Abreu GR. Cardiovascular risk factors in a population of Brazilian schoolchildren. Brazilian Journal of Medical and Biological Research 2006 December;39(12):1637-42. Cross-sectional study
Ref ID: 4484

(4134) Rodrigues AN, Perez AJ, Carletti L, Bissoli NS, Abreu GR. Aptidão cardiorrespiratória e associações com fatores de risco cardiovascular em adolescentes. Jornal de Pediatria 2007 October;83(5):429-35. Cross-sectional study
Ref ID: 600

(4135) Rodrigues ESR, Cheik NC, Mayer AF. Nível de atividade física e tabagismo em universitários. Nivel de actividad física y tabaquismo en universitarios. Revista de Saúde Pública 2008 August;42(4):672-8. Study limited to adults
Ref ID: 4485

(4136) Rodrigues FR, Brandão MJN. Anestesia regional para cesariana em gestantes obesas: estudo retrospective. Regional anesthesia for cesarean section in obese pregnant women: a retrospective study. Anestesia regional para cesárea en embarazadas obesas: estudio retrospectivo. Revista Brasileira de Anestesiologia 2011 February;61(1):17-20. Off topic
Ref ID: 4486

(4137) Rodrigues JC, Takahashi A, Olmos FM, Souza JBd, Bussamra MH, Cardieri JM. Efeito do índice de massa corpórea na gravidade da asma e na reatividade brônquica induzida pelo exercício em crianças asmáticas com sobrepeso e obesas. Effect of body mass index on asthma severity and exercise-induced

bronchial reactivity in overweight and obese asthmatic children. Revista Paulista de Pediatria 2007 September;25(3):207-13. Acute study
Ref ID: 4487

(4138) Rodriguez D, Audrain-McGovern J. Physical activity, global physical self-concept, and adolescent smoking. Annals of Behavioral Medicine 2005 December;30(3):251-9. Cross-sectional study
Ref ID: 1441

(4139) Rodriguez E, Diaz C. Iron, copper and zinc levels in urine: relationship to various individual factors. Journal of Trace Elements in Medicine and Biology 1995 December;9(4):200-9. Off topic
Ref ID: 2186

(4140) Rodríguez Anzardo BR, Martínez Fuentes A, González Medina O. Evaluación antropométrica de un grupo de adolescentes obesos, antes y después de tratamiento. Revista Cubana de Pediatria 1986 August;58(4):427-34. Diet & Exercise intervention
Ref ID: 831

(4141) Rodríguez Fernández OM, Sanchén Casas A, Ramírez Campins A. Fibrosis quística: presentación de un caso. Cystic fibrosis: a case report. Archivo Médico de Camagüey 2011 April;15(2). Case-Control / Case Study
Ref ID: 4488

(4142) Rodríguez JM, Arias J, Garcia C, Torres J, Balibrea JL. Major abdominal surgery and clearance of lipid emulsions. Nutrición Hospitalaria 1994;9:304-10. Off topic
Ref ID: 4970

(4143) Rodríguez O. Situación nutricional del escolar y adolescente en Chile. Revista Chilena de Pediatría 2007 October;78(5):523-33. Diet Intervention Study
Ref ID: 4489

(4144) Roehrig M, Thompson JK, Cafri G. Effects of dieting-related messages on psychological and weight control variables. International Journal of Eating Disorders 2008 March;41(2):164-73. Diet Intervention Study
Ref ID: 993

(4145) Roelen CAM, deVries WR, Koppeschaar HPF, Vervoorn C, Thijssen JHH, Blankenstein MA. Plasma insulin-like growth factor-I and high affinity growth hormone-binding protein levels increase after two weeks of strenuous physical training. International Journal of Sports Medicine 1997;18(4):238-41. Study less than 4 weeks
Ref ID: 5566

(4146) Roemling C, Qaim M. Obesity trends and determinants in Indonesia. Appetite 2012;58(3):1005-13. Review article
Ref ID: 5567

(4147) Roemmich JN, Gurgol CM, Epstein LH. Influence of an interpersonal laboratory stressor on youths' choice to be physically active. Obesity Research 2003 September;11(9):1080-7. Not an exercise intervention study
Ref ID: 356

(4148) Roemmich JN, Gurgol CM, Epstein LH. Open-loop feedback increases physical activity of youth. Medicine and Science in Sports and Exercise 2004 April;36(4):668-73. No comparative control group
Ref ID: 338

(4149) Roemmich JN, Lambiase M, Salvy SJ, Horvath PJ. Protective effect of interval exercise on psychophysiological stress reactivity in children. Psychophysiology 2009 July;46(4):852-61. Off topic
Ref ID: 723

(4150) Rofey DL, Hull EE, Phillips J, Vogt K, Silk JS, Dahl RE. Utilizing Ecological Momentary Assessment in pediatric obesity to quantify behavior, emotion, and sleep. Obesity 2010 June;18(6):1270-2. Lifestyle Intervention
Ref ID: 522

(4151) Roffey DM, Luscombe ND, Byrne NM, Hills AP, Bellon M, Tsopelas C, Kirkwood ID, Wittert GA. Use of [14C]-sodium bicarbonate/urea to measure physical activity induced increases in total energy expenditure in free-living healthy males. Asia Pacific Journal of Clinical Nutrition 2005;14(1):83-90. Study limited to adults
Ref ID: 1541

(4152) Rogers LQ, Hopkins-Price P, Vicari S, Markwell S, Pamenter R, Courneya KS, Hoelzer K, Naritoku C, Edson B, Jones L, Dunnington G, Verhulst S. Physical activity and health outcomes three months after completing a physical activity behavior change intervention: persistent and delayed effects. Cancer Epidemiology, Biomarkers and Prevention 2009 May;18(5):1410-8. Study limited to adults
Ref ID: 756

(4153) Rogers LQ, Hopkins-Price P, Vicari S, Pamenter R, Courneya KS, Markwell S, Verhulst S, Hoelzer K, Naritoku C, Jones L, Dunnington G, Lanzotti V, Wynstra J, Shah L, Edson B, Graff A, Lowy M. A randomized trial to increase physical activity in breast cancer survivors. Medicine and Science in Sports and Exercise 2009 April;41(4):935-46. Study limited to adults
Ref ID: 785

(4154) Rogers MM, Peoples S, Sorenson JR. Translating research into MCH service: comparison of a pilot project and a large-scale resource mothers program.

Public Health Reports 1995;110:563-9. Off topic
Ref ID: 1007

(4155) Rogers VW, Hart PH, Motyka E, Rines EN, Vine J, Deatrick DA. Impact of Let's Go! 5-2-1-0: a community-based, multisetting childhood obesity prevention program. Journal of Pediatric Psychology 2013 October;38(9):1010-20. Inappropriate Study Design
Ref ID: 6040

(4156) Rogol AD. Growth hormone and the adolescent athlete: What are the data for its safety and efficacy as an ergogenic agent? Growth Hormone and Igf Research 2009;19(4):294-9. Drug intervention study
Ref ID: 5568

(4157) Rohr UD, Gocan AG, Bachg D, Schindler AE. Cancer protection of soy resembles cancer protection during pregnancy. Hormone Molecular Biology and Clinical Investigation 2010 December 5;3(2):391-409. Off topic
Ref ID: 3724

(4158) Rojas Rojas M. Desarrollo kinesiológico: la interpretación de un proceso para facilitar el movimiento corporal humano. Kinesiological development: Interpretation of a process to enhance body movement. Revista Ciencias de la Salud (Bogota) 2006 June;4(1):59-72. Review article
Ref ID: 4490

(4159) Rojas J, Uauy D. Necesidad de prevenir la obesidad sin abandonar la protección de los niños con riesgo a desnutrir. Revista Chilena de Nutrición 1999 April;26(1):35-9. Diet Intervention Study
Ref ID: 4491

(4160) Rojas P, Uauy D. Evolución de las normas de alimentación y nutrición del programa alimentario y cambios en el estado nutricional de preescolares beneficiarios de la JUNJI en las últimas 3 décadas. Revista Chilena de Nutrición 2006 April;33(1):91-101. Diet Intervention Study
Ref ID: 4492

(4161) Rolland-Cachera MF, Thibault H, Souberbielle JC, Soulie D, Carbonel P, Deheeger M, Roinsol D, Longueville E, Bellisle F, Serog P. Massive obesity in adolescents: dietary interventions and behaviours associated with weight regain at 2 y follow-up. International Journal of Obesity and Related Metabolic Disorders 2004 April;28(4):514-9. Not an exercise intervention study
Ref ID: 340

(4162) Romagnoli C, Zecca E, Luciano R, Torrioli G, Tortorolo G. Controlled trial of early dexamethasone treatment for the prevention of chronic lung disease in preterm infants: a 3-year follow-up. Pediatrics 2002;109:e85. Subjects less than 2 years old
Ref ID: 4972

(4163) Romanelli G, Giustina A, Cravarezza P, Caldonazzo A, Agabiti-Rosei E, Giustina G. Albuminuria induced by exercise in hypertensive type I and type II diabetic patients: a randomised, double-blind study on the effects of acute administration of captopril and nifedipine. Journal of Human Hypertension 1991 June;5(3):167-73. Study limited to adults
Ref ID: 438

(4164) Romanzini M, Reichert FF, Lopes AdS, Petroski ÉL, Farias Júnior JCd. Prevalência de fatores de risco cardiovascular em adolescentes. Cadernos de Saúde Pública 2008 November;24(11):2573-81. Prevalence study
Ref ID: 4493

(4165) Romanzini M, Petroski EL, Reichert FF. Limiares de acelerômetros para a estimativa da intensidade da atividade física em crianças e adolescentes: uma revisão sistemática. Accelerometers thresholds to estimate physical activity intensity in children and adolescents: a systematic review. Revista Brasileira de Cineantropometria e Desempenho Humano 2012;14(1):101-13. Review article
Ref ID: 4494

(4166) Romero AJ, Robinson TN, Kraemer HC, Erickson SJ, Haydel KF, Mendoza F, Killen JD. Are perceived neighborhood hazards a barrier to physical activity in children? Archives of Pediatrics and Adolescent Medicine 2001 October;155(10):1143-8. Not an exercise intervention study
Ref ID: 1893

(4167) Romero A, Slater B, Florindo AA, Latorre MdRDdO, Cezar C, Silva MVd. Determinantes do índice de massa corporal em adolescentes de escolas públicas de Piracicaba, São Paulo. Determining of body mass index in adolescents from public schools in Piracicaba, São Paulo State. Ciência and Saúde Coletiva 2010 January;15(1):141-9. Cross-sectional study
Ref ID: 4495

(4168) Romero MB, Morencos E, Peinado AB, Bermejo L, Gomez CC, Benito PJ. Can the exercise mode determine lipid profile improvements in obese patients? Nutricion Hospitalaria 2013 May;28(3):607-17. Inappropriate Population
Ref ID: 6041

(4169) Roncesvalles MN, Woollacott MH, Brown N, Jensen JL. An emerging postural response: is control of the hip possible in the newly walking child? Journal of Motor Behavior 2004 June;36(2):147-59. Off topic
Ref ID: 1632

(4170) Ronnberg AK, Nilsson K. Interventions during pregnancy to reduce excessive gestational weight gain: a systematic review assessing current clinical evidence using the Grading of Recommendations, Assessment, Development and Evaluation (GRADE) system. [Review]. International Journal of Obstetrics and

Gynaecology 2010 October;117(11):1327-34. Review article
Ref ID: 2759

(4171) Rono K, Stach-Lempinen B, Klemetti MM, Kaaja RJ, Poyhonen-Alho M, Eriksson JG, Koivusalo SB. Prevention of gestational diabetes through lifestyle intervention: study design and methods of a Finnish randomized controlled multicenter trial (RADIEL). BMC Pregnancy Childbirth 2014;14:70. Inappropriate Population
Ref ID: 6042

(4172) Ronque ER, Cyrino ES, Mortatti AL, Moreira A, Avelar A, Carvalho FO, Arruda Md. Relação entre aptidão cardiorrespiratória e indicadores de adiposidade corporal em adolescents. Relationship between cardiorespiratory fitness and indicators of body adiposity in adolescents. Revista Paulista de Pediatria 2010 September;28(3):296-302. Cross-sectional study
Ref ID: 4496

(4173) Ronque ERV, Cyrino ES, Dórea V, Serassuelo Júnior H, Galdi EHG, Arruda Md. Diagnóstico da aptidão física em escolares de alto nível socioeconômico: avaliação referenciada por critérios de saúde. Revista Brasileira de Medicina do Esporte 2007 April;13(2):71-6. Cross-sectional study
Ref ID: 4497

(4174) Rooney BL, Gritt LR, Havens SJ, Mathiason MA, Clough EA. Growing healthy families: family use of pedometers to increase physical activity and slow the rate of obesity. Wisconsin Medical Journal 2005 July;104(5):54-60. Inappropriate Study Design
Ref ID: 301

(4175) Roongpisuthipong C, Panpakdee O, Boontawee A, Kulapongse S, Tanphaichitr V. Possible thermogenesis with dexfenfluramine. Journal of the Medical Association of Thailand 1999 February;82(2):150-9. Not an exercise intervention study, Drug intervention study
Ref ID: 415

(4176) Rosa CSdC, Messias KP, Rômulo A, Silva CBd, Monteiro HL, Júnior Freitas IF. Atividade física habitual de crianças e adolescentes mensurada por pedômetro e sua relação com índices nutricionais. Usual physical activity in children and adolescents measured by pedometer and its association with nutritional indicators. Revista Brasileira de Cineantropometria e Desempenho Humano 2011;13(1). Cross-sectional study
Ref ID: 4498

(4177) Rosa FJBdl, Puerto JRG, Montaner BHV, Rosa CJBdl, Benitez PC. Estudio de la composición corporal em escolares de 10 a 14 años. Revista Brasileira de Cineantropometria e Desempenho Humano 2001;3(1). Cross-sectional study
Ref ID: 4499

(4178) Rosa MLG, Fonseca VM, Oigman G, Mesquita ET. Pré-hipertensão arterial e pressão de pulso aumentada em adolescentes: prevalência e fatores associados. Arquivos Brasileiros de Cardiologia 2006 July;87(1):46-53. Cross-sectional study
Ref ID: 663

(4179) Rosado CI, Marin ALV, Martinez JA, Cabrerizo L, Gargallo M, Lorenzo H, Quiles J, Planas M, Polanco I, de Avila DR, Russolillo J, Farre R, Villares JMM, Riobo P, Salas-Salvado J. Importance of water in the hydration of the spanish population: Fesnad 2010 document. Nutricion Hospitalaria 2011;26(1):27-36. Off topic
Ref ID: 5569

(4180) Rosado JL, del RA, Montemayor K, Garcia OP, Caamano MC. An increase of cereal intake as an approach to weight reduction in children is effective only when accompanied by nutrition education: a randomized controlled trial. Nutrition Journal 2008 September 10;7:28.:28. Not an exercise intervention study
Ref ID: 158

(4181) Rosal MC, Ockene IS, Restrepo A, White MJ, Borg A, Olendzki B, Scavron J, Candib L, Welch G, Reed G. Randomized trial of a literacy-sensitive, culturally tailored diabetes self-management intervention for low-income latinos: latinos en control. Diabetes Care 2011 April;34(4):838-44. Off topic
Ref ID: 1054

(4182) Rosario R, Araujo A, Oliveira B, Padrao P, Lopes O, Teixeira V, Moreira A, Barros R, Pereira B, Moreira P. Impact of an intervention through teachers to prevent consumption of low nutrition, energy-dense foods and beverages: a randomized trial. Preventive Medicine 2013 July;57(1):20-5. Inappropriate Intervention
Ref ID: 6043

(4183) Rosas-Vargas MA, del-Valle-López CA, del-Río-Navarro BE, Sienra-Monge JJ. [Changes in lung function after a bronchial provocation test with cockroach antigens]. Revista alergia Mexico 2003;50:13-6. Off topic
Ref ID: 4973

(4184) Rose DE, Farmer MM, Yano EM, Washington DL. Racial/ethnic differences in cardiovascular risk factors among women veterans. Journal of General Internal Medicine 2013 July;28 Suppl 2:S524-S528. Inappropriate Study Design
Ref ID: 6044

(4185) Rose JB, Martin TM, Corddry DH, Zagnoev M, Kettrick RG. Ondansetron reduces the incidence and severity of poststrabismus repair vomiting in children. Anesthesia and Analgesia 1994;79:486-9. Off topic
Ref ID: 1028

(4186) Rosen L, Manor O, Engelhard D, Zucker D. In Defense of the Randomized Controlled Trial for Health Promotion Research. American Journal of Public Health 2006 July;96(7):1181-6. Review article
Ref ID: 3855

(4187) Rosenbaum M, Nonas C, Weil R, Horlick M, Fennoy I, Vargas I, Kringas P. School-based intervention acutely improves insulin sensitivity and decreases inflammatory markers and body fatness in junior high school students. Journal of Clinical Endocrinology and Metabolism 2007 February;92(2):504-8. No exercise only group
Ref ID: 262

(4188) Rosenberg DE, Sallis JF, Conway TL, Cain KL, McKenzie TL. Active transportation to school over 2 years in relation to weight status and physical activity. Obesity (Silver Spring) 2006 October;14(10):1771-6. Not a randomized controlled trial (RCT), Observational study
Ref ID: 265

(4189) Rosenberg DE, Norman GJ, Sallis JF, Calfas KJ, Patrick K. Covariation of adolescent physical activity and dietary behaviors over 12 months. Journal of Adolescent Health 2007 November;41(5):472-8. Lifestyle Intervention
Ref ID: 1150

(4190) Rosenberger PH, Dhabhar FS, Epel E, Jokl P, Ickovics JR. Sex differences in factors influencing recovery from arthroscopic knee surgery. Clinical Orthopaedics and Related Research 2010 December;468(12):3399-405. Off topic
Ref ID: 1106

(4191) Rosenbloom AL. Increasing Incidence of Type 2 Diabetes in Children and Adolescents: Treatment Considerations. Pediatrics Drugs 2002 April;4(4):209-21. Review article
Ref ID: 3725

(4192) Rosenkranz RR, Behrens TK, Dzewaltowski DA. A group-randomized controlled trial for health promotion in Girl Scouts: healthier troops in a SNAP (Scouting Nutrition & Activity Program). BMC Public Health 2010 February;%19;10:81.:81. No exercise only group
Ref ID: 58

(4193) Rosenkranz RR, Welk GJ, Hastmann TJ, Dzewaltowski DA. Psychosocial and demographic correlates of objectively measured physical activity in structured and unstructured after-school recreation sessions. Journal of Science and Medicine in Sport 2011 July;14(4):306-11. Cross-sectional study
Ref ID: 2762

(4194) Rosenkranz SK, Rosenkranz RR, Hastmann TJ, Harms CA. High-intensity training improves airway responsiveness in inactive nonasthmatic children:

evidence from a randomized controlled trial. Journal of Applied Physiology 2012 April;112(7):1174-83. Not All Participants were Overweight and/or Obese
Ref ID: 2763

(4195) Rosenstock J, Bergenstal R, Defronzo RA, Hirsch IB, Klonoff D, Boss AH, Kramer D, Petrucci R, Yu W, Levy B, Study Group. Efficacy and safety of Technosphere inhaled insulin compared with Technosphere powder placebo in insulin-naive type 2 diabetes suboptimally controlled with oral agents. Diabetes Care 2008 November;31(11):2177-82. Drug intervention study
Ref ID: 872

(4196) Rosero C, Castro E, Jhayya T. Efectos de la fluticazona (Ft) en niños con asma inducida por ejeercicio: Servicio de Neumología del H. Eugenio Espejo. Revista del Hospital Eugenio Espejo 2003 May;8(1):25-7. Drug intervention study
Ref ID: 4500

(4197) Rosety-Rodriguez M, Rosety I, Fornieles-Gonzalez G, Diaz A, Rosety M, Ordonez FJ. A 12-week aerobic training programme reduced plasmatic allantoin in adolescents with Down syndrome. British Journal of Sports Medicine 2010;44(9):685-7. Not a randomized controlled trial (RCT)
Ref ID: 5570

(4198) Rosseneu M, Cambien F, Vinaimont N, Nicaud V, De BG. Biomarkers of dietary fat composition in young adults with a parental history of premature coronary heart disease compared with controls. The EARS Study. Atherosclerosis 1994 August;108(2):127-36. Diet Intervention Study
Ref ID: 2231

(4199) Rossetti MB, Britto RR, Norton RdC. Prevenção primária de doenças cardiovasculares na obesidade infantojuvenil: efeito anti-inflamatório do exercício físico. Early prevention of cardiovascular diseases in juvenile obesity: the anti-inflammatory effect of physical exercise. Revista Brasileira de Medicina do Esporte 2009 December;15(6):472-5. Review article
Ref ID: 4501

(4200) Rossi CE, Albernaz DO, Vasconcelos FdAGd, Assis MAAd, Di Pietro PF. Influência da televisão no consumo alimentar e na obesidade em crianças e adolescentes: uma revisão sistemática. Television influence on food intake and obesity in children and adolescents: a systematic review. Revista de Nutrição 2010 August;23(4):607-20. Review article
Ref ID: 4502

(4201) Roth B, Munsch S, Meyer AH. [Long-term evaluation of a psychological training for obese children and their parents (TAKE)]. [German]. Praxis der Kinderpsychologie und Kinderpsychiatrie 2011;60(4):304-21. Lifestyle Intervention
Ref ID: 2764

(4202) Roth K, Mauer S, Obinger M, Ruf KC, Graf C, Kriemler S, Lenz D, Lehmacher W, Hebestreit H. Prevention through Activity in Kindergarten Trial (PAKT): a cluster randomised controlled trial to assess the effects of an activity intervention in preschool children. BMC Public Health 2010;10:410. Not All Participants were Overweight and/or Obese
Ref ID: 2765

(4203) Rothe T, Kohl C, Mansfeld HJ. [Controlled study of the effect of sports training on cardiopulmonary functions in asthmatic children and adolescents]. [German]. Pneumologie 1990 September;44(9):1110-4. Study less than 4 weeks
Ref ID: 2303

(4204) Rowe KS. Double-blind randomized controlled trial to assess the efficacy of intravenous gammaglobulin for the management of chronic fatigue syndrome in adolescents. Journal of Psychiatric Research 1997;31:133-47. Drug intervention study
Ref ID: 4975

(4205) Rowland T, Goff D, Martel L, Ferrone L, Kline G. Normalization of maximal cardiovascular variables for body size in premenarcheal girls. Pediatric Cardiology 2000 September;21(5):429-32. Off topic
Ref ID: 1959

(4206) Rowland TW, Unnithan VB, MacFarlane NG, Gibson NG, Paton JY. Clinical manifestations of the 'athlete's heart' in prepubertal male runners. International Journal of Sports Medicine 1994 November;15(8):515-9. Not an exercise intervention study
Ref ID: 2222

(4207) Rowlands AV, Ingledew DK, Powell SM, Eston RG. Interactive effects of habitual physical activity and calcium intake on bone density in boys and girls. Journal of Applied Physiology 2004 October;97(4):1203-8. Not an exercise intervention study
Ref ID: 1590

(4208) Rowley SD, Donaldson G, Lilleby K, Bensinger WI, Appelbaum FR. Experiences of donors enrolled in a randomized study of allogeneic bone marrow or peripheral blood stem cell transplantation. Blood 2001;97:2541-8. Off topic
Ref ID: 4976

(4209) Roxas M. The Role of Enzyme Supplementation in Digestive Disorders. Alternative Medicine Review 2008 December;13(4):307-14. Off topic
Ref ID: 3726

(4210) Roy SK, Bilkes F, Islam K, Ara G, Tanner P, Wosk I, Rahman AS, Chakraborty B, Jolly SP, Khatun W. Impact of pilot project of Rural Maintenance Programme

(RMP) on destitute women: CARE, Bangladesh. Food and Nutrition Bulletin 2008;29:67-75. Off topic
Ref ID: 4977

(4211) Rozenek R, Ward P, Long S, Garhammer J. Effects of high-calorie supplements on body composition and muscular strength following resistance training. Journal of Sports Medicine and Physical Fitness 2002 September;42(3):340-7. Diet Intervention or Supplement Study
Ref ID: 1817

(4212) Ruben AR. Undernutrition and Obesity in Indigenous Children: Epidemiology, Prevention, and Treatment. Pediatric Clinics of North America 2009;56(6):1285-302. Review article
Ref ID: 3301

(4213) Ruben RM, Molinari MA, Bibbee CA, Childress MA, Harman MS, Reed KP, Haff GG. The acute effects of an ascending squat protocol on performance during horizontal plyometric jumps. Journal of Strength and Conditioning Research 2010 February;24(2):358-69. Off topic
Ref ID: 591

(4214) Rubin DA, McMurray RG, Harrell JS, Hackney AC, Thorpe DE, Haqq AM. The association between insulin resistance and cytokines in adolescents: the role of weight status and exercise. Metabolism-Clinical and Experimental 2008;57(5):683-90. Cross-sectional study
Ref ID: 5571

(4215) Rubin DA, Wilson KS, Wiersma LD, Weiss JW, Rose DJ. Rationale and design of active play @ home: a parent-led physical activity program for children with and without disability. BMC Pediatrics 2014;14:41. Inappropriate Study Design
Ref ID: 6045

(4216) Rudelle S, Ferruzzi MG, Cristiani I, Moulin J, Mace K, Acheson KJ, Tappy L. Effect of a thermogenic beverage on 24-hour energy metabolism in humans. Obesity 2007 February;15(2):349-55. Diet Intervention or Supplement Study
Ref ID: 1277

(4217) Ruggenenti P, Gaspari F, Cannata A, Carrara F, Cella C, Ferrari S, Stucchi N, Prandini S, Ene-Iordache B, Diadei O, Perico N, Ondei P, Pisani A, Buongiorno E, Messa P, Dugo M, Remuzzi G. Measuring and Estimating GFR and Treatment Effect in ADPKD Patients: Results and Implications of a Longitudinal Cohort Study. PLoS ONE 2012 February;7(2):1-12. Cohort Study
Ref ID: 3727

(4218) Rugiero P, Walton L, Prieto P, Bravo A, Núñez M, Márquez N, Mühlhausen M. Efecto del masaje terapéutico en prematuros del Servicio de Neonatología del Hospital San José. Revista Chilena de Obstetricia y Ginecología

2008;73(4):257-62. Subjects less than 2 years old
Ref ID: 4503

(4219) Ruiz JR, Ortega FB, Loit HM, Veidebaum T, Sjostrom M. Body fat is associated with blood pressure in school-aged girls with low cardiorespiratory fitness: the European Youth Heart Study. Journal of Hypertension 2007 October;25(10):2027-34. Cross-sectional study
Ref ID: 1163

(4220) Ruiz JR, Labayen I, Ortega FB, Legry V, Moreno LA, Dallongeville J, Martinez-Gomez D, Bokor S, Manios Y, Ciarapica D, Gottrand F, de HS, Molnar D, Sjostrom M, Meirhaeghe A, HELENA Study Group. Attenuation of the effect of the FTO rs9939609 polymorphism on total and central body fat by physical activity in adolescents: the HELENA study. Archives of Pediatrics and Adolescent Medicine 2010 April;164(4):328-33. Cross-sectional study
Ref ID: 560

(4221) Ruiz R, Gesell SB, Buchowski MS, Lambert W, Barkin SL. The relationship between hispanic parents and their preschool-aged children's physical activity. Pediatrics 2011 May;127(5):888-95. Not All Participants were Overweight and/or Obese
Ref ID: 1021

(4222) Rukavina PB, Li W, Shen B, Sun H. A service learning based project to change implicit and explicit bias toward obese individuals in kinesiology pre-professionals. Obesity Facts 2010;3(2):117-26. Off topic
Ref ID: 527

(4223) Rump P, Verstappen F, Gerver WJ, Hornstra G. Body composition and cardiorespiratory fitness indicators in prepubescent boys and girls. International Journal of Sports Medicine 2002 January;23(1):50-4. Cohort Study
Ref ID: 1858

(4224) Runge M, Rittweger J, Russo CR, Schiessl H, Felsenberg D. Is muscle power output a key factor in the age-related decline in physical performance? A comparison of muscle cross section, chair-rising test and jumping power. Clinical Physiology and Functional Imaging 2004 November;24(6):335-40. Study limited to adults
Ref ID: 1574

(4225) Rush E, Reed P, McLennan S, Coppinger T, Simmons D, Graham D. A school-based obesity control programme: Project Energize. Two-year outcomes. British Journal Of Nutrition 2012 February;107(4):581-7. Diet & Exercise intervention
Ref ID: 2767

(4226) Rush E, Reed PW, Simmons D, Coppinger T, McLennan S, Graham D. Baseline measures for a school-based obesity control programme: Project

Energize: differences by ethnicity, rurality, age and school socio-economic status. Journal of Paediatrics and Child Health 2013 April;49(4):E324-E331. Inappropriate Intervention
Ref ID: 6046

(4227) Rush E, McLennan S, Obolonkin V, Vandal AC, Hamlin M, Simmons D, Graham D. Project Energize: whole-region primary school nutrition and physical activity programme; evaluation of body size and fitness 5 years after the randomised controlled trial. British Journal of Nutrition 2014 January 28;111(2):363-71. Inappropriate Intervention
Ref ID: 6047

(4228) Rush EC, Chhichhia P, Kilding AE, Plank LD. Water turnover in children and young adults. European Journal of Applied Physiology 2010 December;110(6):1209-14. Off topic
Ref ID: 2768

(4229) Russo A, Franceschi S, La VC, Dal ML, Montella M, Conti E, Giacosa A, Falcini F, Negri E. Body size and colorectal-cancer risk. International Journal of Cancer 1998 October 5;78(2):161-5. Off topic
Ref ID: 2080

(4230) Russo GH, Bellia CA, Bodas AW. Exercise-induced asthma (EIA): its prevention with the combined use of ipratropium bromide and fenoterol. Respiration 1986;50:Suppl-61. Off topic
Ref ID: 2345

(4231) Rutenfranz J, Macek M, Lange AK, Bell RD, Vavra J, Radvansky J, Klimmer F, Kylian H. The relationship between changing body height and growth related changes in maximal aerobic power. European Journal of Applied Physiology and Occupational Physiology 1990;60(4):282-7. Off topic
Ref ID: 2311

(4232) Rutsztein G, Murawski B, Elizathe L, Arana F, Armatta AM, Leonardelli E. Trastornos alimentarios en mujeres adolescentes: un estudio comparativo entre pacientes, estudiantes de danza y estudiantes de escuelas. Revista Colombiana de Psiquiatría 2010 June;39(2):329-46. Survey or questionnaire
Ref ID: 4504

(4233) Rutters F, Nieuwenhuizen AG, Lemmens SG, Born JM, Westerterp-Plantenga MS. Hypothalamic-pituitary-adrenal (HPA) axis functioning in relation to body fat distribution. Clinical Endocrinology 2010 June;72(6):738-43. Study limited to adults
Ref ID: 505

(4234) Rutters F, Nieuwenhuizen AG, Lemmens SG, Bouwman F, Mariman E, Westerterp-Plantenga MS. Associations between anthropometrical measurements, body composition, single-nucleotide polymorphisms of the

hypothalamus/pituitary/adrenal (HPA) axis and HPA axis functioning. Clinical Endocrinology 2011 June;74(6):679-86. Study limited to adults
Ref ID: 2769

(4235) Ruzicka T, Bieber T, Schopf E, Chipps BE, Sampson HA, Sicherer SH, Teuber SS, Brown RL, Haapanen LAD, Jones SM, Nelson HS, Lahr J, Rule R, Bock A, Leung D, Sachs MJ, Casimir G, Cuvelier P, Allard S, Duchateau J. Best articles relevant to pediatric allergy and immunology. Pediatrics 1998 August 2;102(2):441. Off topic
Ref ID: 550

(4236) Ruzyllo W, Ponikowski P, Wilkins A, Polish Angina Treatment Pattern Program. Clinical characteristics and methods of treatment of patients with stable coronary heart disease in the primary care settings--the results of the Polish, Multicentre Angina Treatment Pattern (ATP) study. International Journal of Clinical Practice 2004 December;58(12):1127-33. Off topic
Ref ID: 1562

(4237) Rydell SA, French SA, Fulkerson JA, Neumark SD, Gerlach AF, Story M, Christopherson KK. Use of a Web-based component of a nutrition and physical activity behavioral intervention with Girl Scouts. Journal of the American Dietetic Association 2005;105:1447-50. Diet & Exercise intervention
Ref ID: 4978

(4238) Rynders C, Weltman A, Delgiorno C, Balagopal P, Damaso L, Killen K, Mauras N. Lifestyle intervention improves fitness independent of metformin in obese adolescents. Medicine and Science in Sports and Exercise 2012 May;44(5):786-92. Diet & Exercise intervention
Ref ID: 2770

(4239) Rynders CA, Angadi SS, Weltman NY, Gaesser GA, Weltman A. Oxygen uptake and ratings of perceived exertion at the lactate threshold and maximal fat oxidation rate in untrained adults. European Journal of Applied Physiology 2011 September;111(9):2063-8. Off topic
Ref ID: 2771

(4240) Ryttig KR, Lammert O, Nielsen E, Garby L, Poulsen K. The effect of a soluble dietary fibre supplement on 24-hour energy expenditure during a standardized physical activity programme. International Journal of Obesity 1990 May;14(5):451-5. Diet Intervention or Supplement Study
Ref ID: 2309

(4241) Saad KR, Colombo AS, Ribeiro AP, Joao SM. Reliability of photogrammetry in the evaluation of the postural aspects of individuals with structural scoliosis. Journal of Bodywork and Movement Therapies 2012 April;16(2):210-6. Off topic
Ref ID: 2772

(4242) Saam F, Leidinger B, Tibesku CO. [The influence of cryotherapy of the ankle on static balance]. [German]. Sportverletzung Sportschaden 2008 March;22(1):45-51. Off topic
Ref ID: 983

(4243) Saavedra JM, Escalante Y, Garcia-Hermoso A. Improvement of aerobic fitness in obese children: a meta-analysis. [Review]. International Journal of Pediatric Obesity 2011 August;6(3-4):169-77. Review article
Ref ID: 2773

(4244) Sabet SR, Kargar M, Kave MH, Tabatabaee H. [The effect of dietary behavior modification on anthropometric indices in obese adolescent female students]. Iranian Journal of Pediatrics 2008;18:71-6. Behavior Modification Intervention
Ref ID: 4979

(4245) Sabia RV, Santos JEd, Ribeiro RPP. Efeito da atividade física associada à orientacão alimentar em adolescentes obesos: comparacão entre o exercício aeróbio e anaeróbio. Effect of physical activity associated with nutritional orientation for obese adolescents: comparison between aerobic and anaerobic exercise. Revista Brasileira de Medicina do Esporte 2004 October;10(5):349-61. Diet & Exercise intervention
Ref ID: 4505

(4246) Sabuncu T, Ucar E, Birden F, Yasar O. The effect of 1-yr sibutramine treatment on glucose tolerance, insulin sensitivity and serum lipid profiles in obese subjects. Diabetes, Nutrition and Metabolism - Clinical and Experimental 2004 April;17(2):103-7. Drug intervention study
Ref ID: 1611

(4247) Sabzghabaee AM, Khayam I, Kelishadi R, Ghannadi A, Soltani R, Badri S, Shirani S. Effect of Zizyphus jujuba fruits on dyslipidemia in obese adolescents: a triple-masked randomized controlled clinical trial. Medical Archives 2013;67(3):156-9. Inappropriate Intervention
Ref ID: 6048

(4248) Sacchetti R, Ceciliani A, Garulli A, Dallolio L, Beltrami P, Leoni E. Effects of a 2-year school-based intervention of enhanced physical education in the primary school. Journal of School Health 2013 September;83(9):639-46. Inappropriate Population
Ref ID: 6049

(4249) Sachdev PS. The current status of tardive dyskinesia. Australian and New Zealand Journal of Psychiatry 2000 June;34(3):355-69. Off topic
Ref ID: 3728

(4250) Sacher PM, Kolotourou M, Chadwick PM, Cole TJ, Lawson MS, Lucas A, Singhal A. Randomized controlled trial of the MEND program: a family-based community intervention for childhood obesity. Obesity (Silver Spring) 2010

February;18 Suppl 1:S62-8.:S62-S68. No exercise only group
Ref ID: 66

(4251) Saelens BE, Epstein LH. Behavioral engineering of activity choice in obese children. International Journal of Obesity and Related Metabolic Disorders 1998 March;22(3):275-7. Reward given for exercise, No comparative control group
Ref ID: 423

(4252) Saelens BE, Sallis JF, Wilfley DE, Patrick K, Cella JA, Buchta R. Behavioral weight control for overweight adolescents initiated in primary care. Obesity Research 2002 January;10(1):22-32. No exercise only group
Ref ID: 386

(4253) Saelens BE, Grow HM, Stark LJ, Seeley RJ, Roehrig H. Efficacy of increasing physical activity to reduce children's visceral fat: a pilot randomized controlled trial. International Journal of Pediatric Obesity 2011 April;6(2):102-12. Behavior Modification Intervention
Ref ID: 1058

(4254) Safarinejad MR. Effect of omega-3 polyunsaturated fatty acid supplementation on semen profile and enzymatic anti-oxidant capacity of seminal plasma in infertile men with idiopathic oligoasthenoteratospermia: a double-blind, placebo-controlled, randomised study. Andrologia 2011 February;43(1):38-47. Off topic
Ref ID: 3729

(4255) Sagedal LR, Overby NC, Lohne-Seiler H, Bere E, Torstveit MK, Henriksen T, Vistad I. Study protocol: fit for delivery - can a lifestyle intervention in pregnancy result in measurable health benefits for mothers and newborns? A randomized controlled trial. BMC Public Health 2013;13:132. Inappropriate Population
Ref ID: 6050

(4256) Saha I, Paul B, Dasgupta A, Ghosh P. Variations of adolescent blood pressure by multifactorial analysis in an urban slum of Kolkata. Journal of the Indian Medical Association 2008;106(9):571-2. Cross-sectional study
Ref ID: 822

(4257) Sahota P, Rudolf MC, Dixey R, Hill AJ, Barth JH, Cade J. Randomised controlled trial of primary school based intervention to reduce risk factors for obesity. British Medical Journal 2001 November 3;323(7320):1029-32. No exercise only group
Ref ID: 393

(4258) Sahota P, Rudolf MC, Dixey R, Hill AJ, Barth JH, Cade J. Evaluation of implementation and effect of primary school based intervention to reduce risk factors for obesity. British Medical Journal 2001 November 3;323(7320):1027-9.

No exercise only group
Ref ID: 394

(4259) Saieh Andonie C. Hipertensión arterial en pediatría. Revista Médica de Clínica Las Condes 1999 October;10(3):114-6. Review article
Ref ID: 4506

(4260) Saito K, Tatsumi M. [Effect of dietary therapy in a school health program for obese children]. [Japanese]. Nippon Koshu Eisei Zasshi - Japanese Journal of Public Health 1994 August;41(8):693-705. Diet Intervention Study
Ref ID: 2234

(4261) Sakamoto FY, Marcon SS, Oliveira AAB, Nardo Junior N. Relação da hipertensão, sobrepeso e aptidão física em estudantes do ensino médio, Maringá-PR. Ciência Cuidado e Saúde 2007 September;6(3):285-90. Prevalence study
Ref ID: 4507

(4262) Sakha K, Behbahan AG. Training for perfect breastfeeding or metoclopramide: which one can promote lactation in nursing mothers? Breastfeeding Medicine 2008;3:120-3. Off topic
Ref ID: 4980

(4263) Saksvig BI, Gittelsohn J, Harris SB, Hanley AJ, Valente TW, Zinman B. A pilot school-based healthy eating and physical activity intervention improves diet, food knowledge, and self-efficacy for native Canadian children. Journal of Nutrition 2005 October;135(10):2392-8. Diet Intervention Study
Ref ID: 1471

(4264) Sakzenian VM, Maestá N, Castanho KF, Michelin E, Orsatti FL, Moraes JEd, Sales MD, Buscariolo FF, Burini RC. Suplementação de proteína do soro do leite nacomposição corporal de jovens praticantes detreinamento para hipertrofi a muscular. Effect of supplementation with whey protein onbody composition of young bodybuilders training for muscle hypertrophy. Nutrire Revista da Sociedade Brasileira de Alimentação e Nutrição 2009 December;34(3). Diet Intervention or Supplement Study
Ref ID: 4508

(4265) Salameh P, Barbour B. Obesity-associated distress in Lebanese adolescents: an exploratory look at a large cohort of students. Eastern Mediterranean Health Journal 2011 December;17(12):949-59. Cross-sectional study
Ref ID: 2775

(4266) Salas A, Gattas Z, Ceballos S X, Burrows A. Tratamiento integral de la obesidad infantil: Efecto de una intervención psicológica. Effects of psychological support as an adjunct to a weight reducing program among obese children. Revista médica de Chile 2010 October;138(10):1217-25.

Retrospective study
Ref ID: 4509

(4267) Salbe AD, Nicolson M, Ravussin E. Total energy expenditure and the level of physical activity correlate with plasma leptin concentrations in five-year-old children. Journal of Clinical Investigation 1997;99(4):592-5. Cross-sectional study
Ref ID: 5572

(4268) Salcedo Aguilar F, Martinez-Vizcaino V, Sanchez LM, Solera MM, Franquelo GR, Serrano MS, Lopez-Garcia E, Rodriguez-Artalejo F. Impact of an after-school physical activity program on obesity in children. Journal of Pediatrics 2010 July;157(1):36-42. Same subjects as another study already included, Not All Participants were Overweight and/or Obese
Ref ID: 55

(4269) Salehpour A, Shidfar F, Hosseinpanah F, Vafa M, Razaghi M, Hoshiarrad A, Gohari M. Vitamin D3 and the risk of CVD in overweight and obese women: a randomised controlled trial. British Journal of Nutrition 2012 November 28;108(10):1866-73. Inappropriate Population
Ref ID: 6051

(4270) Sales-Nobre FS, Jornada-Krebs R, Valentini NC. Práticas de lazer, nível de atividade física e aptidão física de moças e rapazes brasileiros. Brazilian girls' and Boys' leisure practices, physical activity level and physical fitness. Actividad física y prácticas de recreación en jueves de Brasil. Revista de Salud Pública 2009 October;11(5):713-23. Survey or questionnaire
Ref ID: 4510

(4271) Sales-Peres SHdC, Goya S, Sant'Anna RMdF, Silva HM, Sales-Peres AdC, Silva RPRd, Lauris JRP, Bastos JRdM. Prevalência de sobrepeso e obesidade e fatores associados em adolescentes na região centro-oeste do estado de São Paulo (SP, Brasil). Prevalence of overweight and obesity, and associated factors in adolescents, at the central west area of the state São Paulo (SP, Brazil). Ciência and Saúde Coletiva 2010 October;15(supl.2):3175-84. Prevalence study
Ref ID: 4511

(4272) Salinas C, Vio dR. Programas de salud y nutrición sin política de estado: el caso de la promoción de salud escolar en chile. Health and nutrition programs without a state policy: the case of school health promotion in chile. Revista Chilena de Nutrición 2011 June;38(2):100-16. Review article
Ref ID: 4512

(4273) Sallis JF, McKenzie TL, Alcaraz JE, Kolody B, Hovell MF, Nader PR. Project SPARK. Effects of physical education on adiposity in children. Annals of the New York Academy of Sciences 1993 October 29;699:127-36. Not All

Participants were Overweight and/or Obese
Ref ID: 2252

(4274) Sallis JF, Broyles SL, Frankspohrer G, Berry CC, Davis TB, Nader PR. Childs home-environment in relation to the mothers adiposity. International Journal of Obesity 1995;19(3):190-7. Cross-sectional study
Ref ID: 5573

(4275) Sallis JF, McKenzie TL, Conway TL, Elder JP, Prochaska JJ, Brown M, Zive MM, Marshall SJ, Alcaraz JE. Environmental interventions for eating and physical activity: a randomized controlled trial in middle schools. American Journal of Preventive Medicine 2003 April;24(3):209-17. Diet & Exercise intervention
Ref ID: 1763

(4276) Salminen M, Vahlberg T, Ojanlatva A, Kivelä SL. Effects of a controlled family-based health education/counseling intervention. American Journal of Health Behavior 2005;29:395-406. Educational intervention
Ref ID: 4981

(4277) Salmon J, Ball K, Crawford D, Booth M, Telford A, Hume C, Jolley D, Worsley A. Reducing sedentary behaviour and increasing physical activity among 10-year-old children: overview and process evaluation of the 'Switch-Play' intervention. Health Promotion International 2005 March;20(1):7-17. Primary outcome(s) not assessed
Ref ID: 316

(4278) Salmon J, Ball K, Hume C, Booth M, Crawford D. Outcomes of a group-randomized trial to prevent excess weight gain, reduce screen behaviours and promote physical activity in 10-year-old children: switch-play. International Journal of Obesity (London) 2008 April;32(4):601-12. No exercise only group
Ref ID: 200

(4279) Salmon J, Brown H, Hume C. Effects of strategies to promote children's physical activity on potential mediators. [Review] [31 refs]. International Journal of Obesity 2009 April;33:Suppl-73. Review article
Ref ID: 769

(4280) Salmon J, Arundell L, Hume C, Brown H, Hesketh K, Dunstan DW, Daly RM, Pearson N, Cerin E, Moodie M, Sheppard L, Ball K, Bagley S, Paw MC, Crawford D. A cluster-randomized controlled trial to reduce sedentary behavior and promote physical activity and health of 8-9 year olds: the Transform-Us! study. BMC Public Health 2011;11:759. Description versus conduct of study
Ref ID: 2776

(4281) Salmun LM, Herron JM, Banfield C, Padhi D, Lorber R, Affrime MB. The pharmacokinetics, electrocardiographic effects, and tolerability of loratadine syrup in children aged 2 to 5 years. Clinical Therapeutics 2000;22:613-21. Drug

intervention study
Ref ID: 4982

(4282) Salvatore S, Vandenplas Y. Gastroesophageal reflux and cow milk allergy: Is There a Link? Pediatrics 2002 November;110(5):972. Off topic
Ref ID: 3730

(4283) Salvy SJ, Roemmich JN, Bowker JC, Romero ND, Stadler PJ, Epstein LH. Effect of peers and friends on youth physical activity and motivation to be physically active. Journal of Pediatric Psychology 2009 March;34(2):217-25. Not a randomized controlled trial (RCT)
Ref ID: 171

(4284) Samaras TT, Storms LH. Impact of height and weight on life-span. Bulletin of the World Health Organization 1992;70(2):259-67. Off topic
Ref ID: 5574

(4285) Samaras TT, Elrick H, Storms LH. Height, health and growth hormone. Acta Paediatrica 1999;88(6):602-9. Off topic
Ref ID: 5575

(4286) Samtani MN, Vermeulen A, Stuyckens K. Population pharmacokinetics of intramuscular paliperidone palmitate in patients with schizophrenia. Clinical Pharmacokinetics 2009 September;48(9):585-600. Off topic
Ref ID: 3731

(4287) Sanders LM, Perrin EM, Yin HS, Bronaugh A, Rothman RL. "Greenlight study": a controlled trial of low-literacy, early childhood obesity prevention. Pediatrics 2014 June;133(6):e1724-e1737. Inappropriate Population
Ref ID: 6052

(4288) Sanders SW, Buchi KN, Goddard MS, Lang JK, Tolman KG. Single-dose pharmacokinetics and tolerance of a cholesteryl sulfate complex of amphotericin-b administered to healthy-volunteers. Antimicrobial Agents and Chemotherapy 1991;35(6):1029-34. Drug intervention study
Ref ID: 5576

(4289) Sanderson DJ. The influence of cadence and power output on the biomechanics of force application during steady-rate cycling in competitive and recreational cyclists. Journal of Sports Sciences 1991;9(2):191-203. Off topic
Ref ID: 2298

(4290) Sandfort TGM, Bakker F, Vanwesenbeeck I, Schellevis FoG. Sexual orientation and mental and physical health status: findings from a Dutch population survey. American Journal of Public Health 2006 June;96(6):1119-25. Study limited to adults
Ref ID: 3856

(4291) Sandstedt E, Fasth A, Fors H, Beckung E. Bone health in children and adolescents with juvenile idiopathic arthritis and the influence of short-term physical exercise. Pediatric Physical Therapy 2012;24(2):155-61. Not All Participants were Overweight and/or Obese
Ref ID: 5577

(4292) SanGiovanni JP, Parra-Cabrera S, Colditz GA, Berkey CS, Dwyer JT. Meta-analysis of Dietary Essential fatty acids and long-chain polyunsaturated fatty acids as they relate to visual resolution acuity in healthy preterm infants. Pediatrics 2000 June;105(6):1292. Review article
Ref ID: 515

(4293) Sangtawesin C, Sangtawesin V, Lertsutthiwong W, Kanjanapattanakul W, Khorana M, Ayudhaya JK. Prophylaxis of symptomatic patent ductus arteriosus with oral ibuprofen in very low birth weight infants. Journal of the Medical Association of Thailand 2008;91 Suppl 3:S28-S34. Drug intervention study
Ref ID: 4983

(4294) Sanguanrungsirikul S, Somboonwong J, Nakhnahup C, Pruksananonda C. Energy expenditure and physical activity of obese and non-obese Thai children. Journal of the Medical Association of Thailand 2001 June;84:Suppl-20. Cross-sectional study
Ref ID: 1898

(4295) Sankaranarayanan K, Mondkar JA, Chauhan MM, Mascarenhas BM, Mainkar AR, Salvi RY. Oil massage in neonates: an open randomized controlled study of coconut versus mineral oil. Indian Pediatrics 2005 September;42(9):877-84. Off topic
Ref ID: 1467

(4296) Santagostino A, Garbaccio G, Pagliaro P, Girotto M, Camisasca G, Bolis V, Pistorio A. A national policentric study for the definition of a reference range for normal values of peripheral blood lymphocyte subsets in healthy adults. European Journal of Histochemistry 1997;41:Suppl-6. Off topic
Ref ID: 2112

(4297) Santana SE, Groeneveld IF, Gonzalez-Saiz L, Lopez-Mojares LM, Villa-Asensi JR, Barrio Gonzalez MI, Fleck SJ, Perez M, Lucia A. Intrahospital weight and aerobic training in children with cystic fibrosis: a randomized controlled trial. Medicine and Science in Sports and Exercise 2012 January;44(1):2-11. Not All Participants were Overweight and/or Obese
Ref ID: 1050

(4298) Santiago Antonio DI, Rodriguez AF. Tratamiento farmacologico de la caquexia-anorexia relacionada con el cancer. (Spanish). Medicina Interna de Mexico 2002 March;18(2):85. Off topic
Ref ID: 3732

(4299) Santiago SQ, Silva MdL, Davidson J, Aristóteles LRd. Avaliação da força muscular respiratória em crianças e adolescentes com sobrepeso/obesos. Evaluation of respiratory muscle strength in overweight/obese children and adolescents. Revista Paulista de Pediatria 2008 June;26(2):146-50. Cross-sectional study
Ref ID: 4513

(4300) Santoro JR, Daneluzzi JC, Barbieri MA, Ricco RG. Obesidade na infância. In: Woiski JR, editor. Nutrição e Dietética em Pediatria.s.l: Atheneu; 1988. p. 251-62.
Subjects less than 2 years old
Ref ID: 829

(4301) Santos ALd, Carvalho ALd, Garcia Júnior JR. Obesidade infantil e uma proposta de Educação Física preventiva. Motriz Revista de Educação Física (Improv) 2007;13(3):203-13. Review article
Ref ID: 4514

(4302) Santos CM, Wanderley Junior RdS, Barros SSH, Farias Junior JCd, Barros MVGd. Prevalencia e fatores associados a inatividade fisica nos deslocamentos para escola em adolescents. Prevalence of physical inactivity and associated factors among adolescents commuting to school. Cadernos de Saúde Pública 2010 July;26(7):1419-30. Prevalence study
Ref ID: 4515

(4303) Santos DA, Matias CN, Monteiro CP, Silva AM, Rocha PM, Minderico CS, Bettencourt SL, Laires MJ. Magnesium intake is associated with strength performance in elite basketball, handball and volleyball players. Magnesium Research 2011 December;24(4):215-9. Diet Intervention or Supplement Study
Ref ID: 2777

(4304) Santos DMdV, Chaves RNd, Souza MCd, Seabra A, Garganta R, José António Ribeiro M. Taxas de sucesso na aptidão física. Efeitos da idade, sexo, actividade física, sobrepeso e obesidade. Passing rates on physical fitness. Effects of age, gender, physical activity, overweight and obesity. Revista Brasileira de Cineantropometria e Desempenho Humano 2010;12(5). Prevalence study
Ref ID: 4516

(4305) Santos FM, Rodrigues RG, Trindade-Filho EM. [Physical exercise versus exercise program using electrical stimulation devices for home use]. [Portuguese]. Revista de Saude Publica 2008 February;42(1):117-22. Study limited to adults
Ref ID: 1008

(4306) Santos FS, Rangel LGG, Saucedo GP, Rosales GV, Novales MGM. Hypertriglyceridemia and hypercholesterolemia in human immunodeficiency

virus-1-infected children treated with protease inhibitors. Archives of Medical Research 2006;37(1):129-32. Drug intervention study
Ref ID: 5578

(4307) Santos FdMMd, Borges MC, Correia MITD, Telles RW, Lanna CCD. Avaliação do estado nutricional e da atividade física em pacientes com lúpus eritematoso sistêmico. Assessment of nutritional status and physical activity in systemic lupus erythematosus patients. Revista Brasileira de Reumatologia 2010 December;50(6):631-8. Study limited to adults
Ref ID: 4517

(4308) Santos GLd, Bueno TB, Tudella E, Dionísio J. Preferência podal em lactentes com síndrome de Down: fatores extrínsecos e intrínsecos. Foot preference in infants with Down syndrome: extrinsic and intrinsic factors. Fisioterapia e Pesquisa 2012 June;19(2):171-7. Off topic
Ref ID: 4518

(4309) Santos I, Victora CG, Martines J, Gonçalves H, Gigante DP, Valle NJ, Pelto G. Nutrition counseling increases weight gain among Brazilian children. Journal of Nutrition 2001;131:2866-73. Diet Intervention Study
Ref ID: 4984

(4310) Santos JSd, Oliveira TMd, Piza Filho JT, Ono EYS, Hirooka EY. Avaliação da ingestão de derivados de trigo em Londrina, PR, por meio de questionário de frequência de consumo de alimentos. Wheat intake assessment in Londrina, Paraná, Brazil through food consumption frequency questionnaire. Nutrire Revista da Sociedade Brasileira de Alimentação e Nutrição 2009 December;34(3). Diet Intervention Study
Ref ID: 4519

(4311) Santos LMd, Santos DNd, Bastos ACS, Assis AMO, Prado MS, Barreto ML. Determinants of early cognitive development: hierarchical analysis of a longitudinal study. Cadernos de Saúde Pública 2008 February;24(2):427-37. Off topic
Ref ID: 4520

(4312) Santos LRdC, Rabinovich EP. Situações familiares na obesidade exógena infantil do filho único. Saúde Soc 2011 June;20(2):507-21. Not a randomized controlled trial (RCT)
Ref ID: 4521

(4313) Santos MAMd, Leandro CG, Guimarães FJdS. Composição corporal e maturação somática de meninas atletas e não-atletas de natação da cidade do Recife, Brasil. Revista Brasileira de Saúde Materno Infantil 2007 April;7(2):175-81. Not a randomized controlled trial (RCT)
Ref ID: 4522

(4314) Santos MGd, Pegoraro M, Sandrini F, Macuco EC. Fatores de risco no desenvolvimento da aterosclerose na infância e adolescência. Risk factors for the development of atherosclerosis in childhood and adolescence. Arquivos brasileiros de cardiologia 2008 April;90(4):301-8. Review article
Ref ID: 4523

(4315) Santos PL. Morbidade referida, situação vacinal e acesso a serviços de saúde por pré-escolares. Reported morbidity, vaccination status and access to health services in early childhood. Medicina (Ribeirão Preto) 2009 June;42(2):127-34. Off topic
Ref ID: 4524

(4316) Santos RG, Durksen A, Rabbanni R, Chanoine JP, Lamboo MA, Mayer T, McGavock JM. Effectiveness of peer-based healthy living lesson plans on anthropometric measures and physical activity in elementary school students: a cluster randomized trial. JAMA Pediatrics 2014 April;168(4):330-7. Inappropriate Population
Ref ID: 6053

(4317) Saquib N, Pierce JP, Saquib J, Flatt SW, Natarajan L, Bardwell WA, Patterson RE, Stefanick ML, Thomson CA, Rock CL, Jones LA, Gold EB, Karanja N, Parker BA. Poor physical health predicts time to additional breast cancer events and mortality in breast cancer survivors. Psycho-Oncology 2011 March;20(3):252-9. Off topic
Ref ID: 1067

(4318) Sardinha LB, Baptista F, Ekelund U. Objectively measured physical activity and bone strength in 9-year-old boys and girls. Pediatrics 2008 September;122(3):e728-e736. Cross-sectional study
Ref ID: 894

(4319) Saris WHM, Antoine JM, Brouns F, Fogelholm M, Gleeson M, Hespel P, Jeukendrup AE, Maughan RJ, Pannemans D, Stich V. PASSCLAIM - Physical performance and fitness. European Journal of Nutrition 2003 March 2;42(1):i50. Review article
Ref ID: 3733

(4320) Sarraf ZN, Sadri G, Malek AH, Baghaei M, Mohammadi FN, Shahrokhi S, Tolooie H, Poormoghaddas M, Sadeghi M, Tavassoli A, Rafiei M, Kelishadi R, Rabiei K, Bashardoost N, Boshtam M, Asgary S, Naderi G, Changiz T, Yousefie A. Isfahan Healthy Heart Programme: a comprehensive integrated community-based programme for cardiovascular disease prevention and control. Design, methods and initial experience. Acta Cardiologica 2003;58:309-20. Study limited to adults
Ref ID: 4985

(4321) Sarton-Miller I. Noninvasive assessment of energy expenditure in children. American Journal of Human Biology 2006 September;18(5):600-9. Cross-sectional study
Ref ID: 1356

(4322) Sartorio A, Lafortuna CL, Marinone PG, Tavani A, La VC, Bosetti C. Short-term effects of two integrated, non-pharmacological body weight reduction programs on coronary heart disease risk factors in young obese patients. Diabetes, Nutrition and Metabolism 2003 August;16(4):262-5. No comparative control group
Ref ID: 341

(4323) Sasaki H, Maeda J, Usui S, Ishiko T. Effect of sucrose and caffeine ingestion on performance of prolonged strenuous running. International Journal of Sports Medicine 1987 August;8(4):261-5. Diet Intervention or Supplement Study
Ref ID: 2337

(4324) Sash SF. Letter: Treating obesity in schoolboys. South African Medical Journal Suid-Afrikaanse Tydskrif Vir Geneeskunde 1976 May 15;50(21):797. Editorial or letter or comment
Ref ID: 2391

(4325) Sastry BK, Narasimhan C, Reddy NK, Raju BS. Clinical efficacy of sildenafil in primary pulmonary hypertension: a randomized, placebo-controlled, double-blind, crossover study. Journal of the American College of Cardiology 2004 April 7;43(7):1149-53. Drug intervention study
Ref ID: 1654

(4326) Satel S. Is Caffeine Addictive? A Review of the Literature. American Journal of Drug and Alcohol Abuse 2006 November;32(4):493-502. Review article
Ref ID: 3734

(4327) Sato KM, Osiecki R, Campos Wd, Coelho RW, Silva SGd. Curvas de percentis para índice de massa corporal em escolares da rede de ensino pública de Curitiba-PR. Revista Brasileira de Atividade Física e Saúde 2002;7(2):43-52. Cross-sectional study
Ref ID: 4525

(4328) Saucedo-Molina TdJ, Unikel Santoncini C. Conductas alimentarias de riesgo, interiorización del ideal estético de delgadez e índice de masa corporal en estudiantes hidalguenses de preparatoria y licenciatura de una institución privada. Disordered eating, internalization of the body thin-ideal and body mass index in high school and college students from a private institution in Hidalgo, Mexico. Salud Mental 2010 February;33(1):11-9. Survey or questionnaire
Ref ID: 4526

(4329) Saugy M, Robinson N, Saudan C, Baume N, Avois L, Mangin P. Human growth hormone doping in sport. British Journal of Sports Medicine 2006 July 2;40:i35-

i39. Review article
Ref ID: 3894

(4330) Saunders LE, Green JM, Petticrew MP, Steinbach R, Roberts H. What are the health benefits of active travel? A systematic review of trials and cohort studies. PLoS ONE 2013;8(8):e69912. Inappropriate Study Design
Ref ID: 6054

(4331) Saunders TJ, Chaput JP, Goldfield GS, Colley RC, Kenny GP, Doucet E, Tremblay MS. Prolonged sitting and markers of cardiometabolic disease risk in children and youth: a randomized crossover study. Metabolism 2013 October;62(10):1423-8. Inappropriate Intervention
Ref ID: 6055

(4332) Saunders TJ, Chaput JP, Goldfield GS, Colley RC, Kenny GP, Doucet E, Tremblay MS. Children and youth do not compensate for an imposed bout of prolonged sitting by reducing subsequent food intake or increasing physical activity levels: a randomised cross-over study. British Journal of Nutrition 2014 February;111(4):747-54. Inappropriate Intervention
Ref ID: 6056

(4333) Sauseng W, Nagel B, Gamillscheg A, Aigner R, Borkenstein M, Zotter H. Acylated ghrelin increases after controlled short-time exercise in school-aged children. Scandinavian Journal of Medicine and Science in Sports 2011;21(6):E100-E105. Acute study
Ref ID: 5579

(4334) Savastano S, Di SC, Angrisani L, Orio F, Longobardi S, Lombardi G, Colao A. Growth hormone treatment prevents loss of lean mass after bariatric surgery in morbidly obese patients: results of a pilot, open, prospective, randomized, controlled study. Journal of Clinical Endocrinology and Metabolism 2009 March;94(3):817-26. Study limited to adults
Ref ID: 145

(4335) Savine R, Sonksen P. Growth hormone - hormone replacement for the somatopause?. [Review] [40 refs]. Hormone Research 2000;53:Suppl-41. Review article
Ref ID: 1961

(4336) Savino F, Bonfante G, Madon E. [Use of natural vitamin supplements in children during convalescence and in children with athletic activities]. [Review] [48 refs] [Italian]. Minerva Pediatrica 1999 January;51(1-2):1-9. Review article
Ref ID: 2025

(4337) Savino F, Bonfante G, Madon E. Use of vitamin supplements in children during convalescence and in children performing sports. Minerva Pediatrica 1999;51:1-9. Diet Intervention or Supplement Study
Ref ID: 4988

(4338) Savino P. Obesidad y enfermedades no transmisibles relacionadas con la nutrición. Revista Colombiana de Cirugía 2011 September;26(3):180-95. Review article
Ref ID: 4527

(4339) Savoye M, Shaw M, Dziura J, Tamborlane WV, Rose P, Guandalini C, Goldberg-Gell R, Burgert TS, Cali AM, Weiss R, Caprio S. Effects of a weight management program on body composition and metabolic parameters in overweight children: a randomized controlled trial. Journal of the American Medical Association 2007 June 27;297(24):2697-704. No exercise only group
Ref ID: 230

(4340) Savoye M, Nowicka P, Shaw M, Yu S, Dziura J, Chavent G, O'Malley G, Serrecchia JB, Tamborlane WV, Caprio S. Long-term results of an obesity program in an ethnically diverse pediatric population. Pediatrics 2011 March;127(3):402-10. Lifestyle Intervention, No exercise only group
Ref ID: 444

(4341) Sawada S, Tanaka H, Funakoshi M, Shindo M, Kono S, Ishiko T. 5-year prospective-study on blood-pressure and maximal oxygen-uptake. Clinical and Experimental Pharmacology and Physiology 1993;20(7-8):483-7. Study limited to adults
Ref ID: 5580

(4342) Sawczuk M, Maciejewska A, Cieszczyk P, Eider J. The role of genetic research in sport. Science and Sports 2011;26(5):251-8. Off topic
Ref ID: 5581

(4343) Sayeg-Porto MA, Oliveira HP, Cunha AJ, Miranda G, Guimarães MM, Oliveira WA, dos-Santos DM. Linear growth and zinc supplementation in children with short stature. Journal of Pediatric Endocrinology and Metabolism 2000;13:1121-8. Diet Intervention or Supplement Study
Ref ID: 4989

(4344) Sayers A, Timpson NJ, Sattar N, Deanfield J, Hingorani AD, Davey-Smith G, Tobias JH. Adiponectin and its association with bone mass accrual in childhood. Journal of Bone and Mineral Research 2010;25(10):2212-20. Longitudinal Study
Ref ID: 5582

(4345) Sazawal S, Dhingra U, Dhingra P, Hiremath G, Sarkar A, Dutta A, Menon VP, Black RE. Micronutrient fortified milk improves iron status, anemia and growth among children 1-4 years: a double masked, randomized, controlled trial. PLoS ONE 2010;5:e12167. Diet Intervention or Supplement Study
Ref ID: 4990

(4346) Sá Silva SPd, Sandre-Pereira G, Salles-Costa R. Fatores sociodemográficos e atividade física de lazer entre homens e mulheres de Duque de Caxias/RJ.

Socio-demographic factors and leisure-time physical activity among men and women of Duque de Caxias / RJ. Ciência and Saúde Coletiva 2011 November;16(11):4491-501. Study limited to adults
Ref ID: 4528

(4347) Sá NNBd, Moura EC. Fatores associados à carga de doenças da síndrome metabólica entre adultos brasileiros. Factors associated with the burden of metabolic syndrome diseases among Brazilian adults. Cadernos de Saúde Pública 2010 September;26(9):1853-62. Study limited to adults
Ref ID: 4529

(4348) Sáenz-Soto NE, Gallego EC. Efecto de intervención física sobre alimentación y actividad física en adolescentes mexicanos con obesidadi. Texto and Contexto Enfermagem 2004 March;13(1):17-25. Diet & Exercise intervention
Ref ID: 4530

(4349) Slupski W, Trocha M, Szelag A, Rutkowska M. Perspektywy leczniczych zastosowań kannabinoidów w chorobach wątroby i trzustki (Polish). Gastroenterologia Polska / Gastroenterology 2010 September;17(5):374-80. Off topic
Ref ID: 3735

(4350) Sbrocco T, Nedegaard RC, Stone JM, Lewis EL. Behavioral choice treatment promotes continuing weight loss: preliminary results of a cognitive-behavioral decision-based treatment for obesity. Journal of Consulting and Clinical Psychology 1999 April;67(2):260-6. Study not limited to children and adolescents
Ref ID: 410

(4351) Sbruzzi G, Eibel B, Barbiero SM, Petkowicz RO, Ribeiro RA, Cesa CC, Martins CC, Marobin R, Schaan CW, Souza WB, Schaan BD, Pellanda LC. Educational interventions in childhood obesity: a systematic review with meta-analysis of randomized clinical trials. Preventive Medicine 2013 May;56(5):254-64. Inappropriate Study Design
Ref ID: 6057

(4352) Schaad UB, Stoupis C, Wedgwood J, Tschaeppeler H, Vock P. Clinical, radiologic and magnetic-resonance monitoring for skeletal toxicity in pediatric-patients with cystic-fibrosis receiving a 3-month course of ciprofloxacin. Pediatric Infectious Disease Journal 1991;10(10):723-9. Off topic
Ref ID: 5583

(4353) Schaefer A, Winkel K, Finne E, Kolip P, Reinehr T. An effective lifestyle intervention in overweight children: one-year follow-up after the randomized controlled trial on "Obeldicks light". Clinical Nutrition 2011 October;30(5):629-33. Follow-up Study
Ref ID: 2780

(4354) Schauble N, Reichwald K, Grassl W, Bechstein H, Muller HC, Scherag A, Geller F, Utting M, Siegfried W, Goldschmidt H, Blundell J, Lawton C, Alam R, Whybrow S, Stubbs J, Platzer M, Hebebrand J, Hinney A. Human galanin (GAL) and galanin 1 receptor (GALR1) variations are not involved in fat intake and early onset obesity. Journal of Nutrition 2005;135(6):1387-92. Off topic
Ref ID: 5584

(4355) Scheen AJ, Rorive M, Letiexhe M. [Physical exercise for preventing obesity, promoting weight loss and maintaining weight management]. [Review] [11 refs] [French]. Revue Medicale de Liege 2001 April;56(4):244-7. Review article
Ref ID: 1910

(4356) Scheen AJ, Finer N, Hollander P, Jensen MD, Van Gaal LF. Efficacy and tolerability of rimonabant in overweight or obese patients with type 2 diabetes: a randomised controlled study. Lancet 2006 November 11;368(9548):1660-72. Study limited to adults, No exercise only group
Ref ID: 260

(4357) Scheett TP, Nemet D, Stoppani J, Maresh CM, Newcomb R, Cooper DM. The effect of endurance-type exercise training on growth mediators and inflammatory cytokines in pre-pubertal and early pubertal males. Pediatric Research 2002;52:491-7. Not All Participants were Overweight and/or Obese
Ref ID: 4991

(4358) Scheffler C, Ketelhut K, Mohasseb I. Does physical education modify the body composition?--results of a longitudinal study of pre-school children. Anthropologischer Anzeiger 2007 June;65(2):193-201. Longitudinal Study
Ref ID: 1179

(4359) Scheid JL, De Souza MJ, Leidy HJ, Williams NI. Ghrelin but not peptide YY is related to change in body weight and energy availability. Medicine and Science in Sports and Exercise 2011 November;43(11):2063-71. Study limited to adults
Ref ID: 2781

(4360) Scheiwe A, Hardy R, Watt RG. Four-year follow-up of a randomized controlled trial of a social support intervention on infant feeding practices. Maternal and Child Nutrition 2010;6:328-37. Follow-up Study
Ref ID: 4992

(4361) Schelling S, Munsch S, Meyer AH, Newark P, Biedert E, Margraf J. Increasing the motivation for physical activity in obese patients. International Journal of Eating Disorders 2009 March;42(2):130-8. Study not limited to children and adolescents
Ref ID: 155

(4362) Scherer TA, Barandun J, Martinez E, Wanner A, Rubin EM. Effect of high-frequency oral airway and chest wall oscillation and conventional chest physical therapy on expectoration in patients with stable cystic fibrosis. Chest

1998;113:1019-27. Off topic
Ref ID: 917

(4363) Scherwitz LW, Perkins LL, Chesney MA, Hughes GH, Sidney S, Manolio TA. Hostility and health behaviors in young adults: the CARDIA Study. Coronary Artery Risk Development in Young Adults Study. American Journal of Epidemiology 1992 July 15;136(2):136-45. Study limited to adults
Ref ID: 2284

(4364) Schibler A, von der HR, Birrer P, Mullis PE. Prospective randomised treatment with recombinant human growth hormone in cystic fibrosis. Archives of Disease in Childhood 2003 December;88(12):1078-81. Drug intervention study
Ref ID: 1689

(4365) Schiff M, Benit P, Coulibaly A, Loublier S, El-Khoury R, Rustin P. Mitochondrial response to controlled nutrition in health and disease. Nutrition Reviews 2011 February;69(2):65-75. Review article
Ref ID: 3736

(4366) Schlienger JL, Goichot B. [Growth hormone: a magical potion?]. [Review] [47 refs] [French]. Revue de Medecine Interne 1998 April;19(4):279-85. Review article
Ref ID: 2078

(4367) Schlotz W, Jones A, Phillips NMM, Godfrey KM, Phillips DIW. Size at birth and motor activity during stress in children aged 7 to 9 years. Pediatrics 2007;120(5):E1237-E1244. Off topic
Ref ID: 5585

(4368) Schlumpf M, Eiholzer U, Gygax M, Schmid S, van dS, I, l'Allemand D. A daily comprehensive muscle training programme increases lean mass and spontaneous activity in children with Prader-Willi syndrome after 6 months. Journal of Pediatric Endocrinology 2006 January;19(1):65-74. Not a randomized controlled trial (RCT)
Ref ID: 1412

(4369) Schlüssel MM, Souza EBd, Reichenheim ME, Kac G. Physical activity during pregnancy and maternal-child health outcomes: a systematic literature review. Atividade física na gestação e desfechos da saúde materno-infantil: uma revisão sistemática da literatura. Cadernos de Saúde Pública 2008;24(supl.4):s531-s544. Review article
Ref ID: 4531

(4370) Schmidt-Trucksass A. [The metabolic syndrome and sports]. [Review] [0 refs] [German]. MMW Fortschritte der Medizin 2006 September 21;148(38):30-2. Review article
Ref ID: 1339

(4371) Schmidt B, Schulz C, Moebus S, Seiwert M, Kolossa-Gehring M, Jockel KH. Concept for a German national birth cohort for environmental health research. Bundesgesundheitsblatt-Gesundheitsforschung-Gesundheitsschutz 2012;55(6-7):852-7. Off topic
Ref ID: 5586

(4372) Schmidt JB, Gregersen NT, Pedersen SD, Arentoft JL, Ritz C, Schwartz TW, Holst JJ, Astrup A, Sjodin A. Effects of PYY3-36 and GLP-1 on energy intake, energy expenditure, and appetite in overweight men. American Journal of Physiology, Endocrinology and Metabolism 2014 June 1;306(11):E1248-E1256. Inappropriate Population
Ref ID: 6058

(4373) Schmidt OA, Stahl U, Brueck M. Posthepatic obstructive jaundice caused by primary extragonadal germ-cell tumor in a patient with glucose-6-phosphatase dehydrogenase deficiency. Deutsche Medizinische Wochenschrift 2010;135(44):2181-5. Off topic
Ref ID: 5587

(4374) Schmidt SM, Ballke EH, Nuske F, Leistikow G, Wiersbitzky SK. [Effect of ambulatory sports therapy on bronchial asthma in children]. [German]. Pneumologie 1997 August;51(8):835-41. Off topic
Ref ID: 2124

(4375) Schmidt T, Wijga A, Von Zur MA, Brabant G, Wagner TO. Changes in cardiovascular risk factors and hormones during a comprehensive residential three month kriya yoga training and vegetarian nutrition. Acta Physiologica Scandinavica Supplementum 1997;640:158-62. Diet & Exercise intervention
Ref ID: 2118

(4376) Schmidt WC, Ling CX. A decision-tree model of balance scale development. Machine Learning 1996;24(3):203-30. Off topic
Ref ID: 5588

(4377) Schmincke HU, Rausch J, Kutterolf S, Freundt A. Walking through volcanic mud: the 2,100 year-old Acahualinca footprints (Nicaragua) II: the Acahualinca people, environmental conditions and motivation. International Journal of Earth Sciences 2010;99:S279-S292. Off topic
Ref ID: 5589

(4378) Schmitz KH, Jacobs DR, Jr., Leon AS, Schreiner PJ, Sternfeld B. Physical activity and body weight: associations over ten years in the CARDIA study. Coronary Artery Risk Development in Young Adults. International Journal of Obesity and Related Metabolic Disorders 2000 November;24(11):1475-87. Study limited to adults
Ref ID: 1943

(4379) Schmitz KH, Schreiner PJ, Jacobs DR, Leon AS, Liu K, Howard B, Sternfeld B. Independent and interactive effects of apolipoprotein E phenotype and cardiorespiratory fitness on plasma lipids. Annals of Epidemiology 2001 February;11(2):94-103. Study limited to adults
Ref ID: 1930

(4380) Schmitz KH, Lytle LA, Phillips GA, Murray DM, Birnbaum AS, Kubik MY. Psychosocial correlates of physical activity and sedentary leisure habits in young adolescents: the Teens Eating for Energy and Nutrition at School study. Preventive Medicine 2002;34:266-78. Cross-sectional study
Ref ID: 4993

(4381) Schnabel D, Grasemann C, Staab D, Wollmann H, Ratjen F, German Cystic Fibrosis Growth Hormone Study Group. A multicenter, randomized, double-blind, placebo-controlled trial to evaluate the metabolic and respiratory effects of growth hormone in children with cystic fibrosis. Pediatrics 2007 June;119(6):e1230-e1238. Drug intervention study
Ref ID: 1225

(4382) Schneider M, Dunton GF, Bassin S, Graham DJ, Eliakim AF, Cooper DM. Impact of a school-based physical activity intervention on fitness and bone in adolescent females. Journal of Physical Activity and Health 2007 January;4(1):17-29. Not All Participants were Overweight and/or Obese
Ref ID: 1236

(4383) Schneider M, Hall WJ, Hernandez AE, Hindes K, Montez G, Pham T, Rosen L, Sleigh A, Thompson D, Volpe SL, Zeveloff A, Steckler A. Rationale, design and methods for process evaluation in the HEALTHY study. International Journal of Obesity (London) 2009 August;33 Suppl 4:S60-7.:S60-S67. Describes other aspects of the study
Ref ID: 105

(4384) Schneider P, Meyer F. O papel do exercício fisico na composição corporal e na taxa metabólica basal de meninos adolescentes obesos. Revista Brasileira de Ciência e Movimento 2007;15(1):101-7. Review article
Ref ID: 4532

(4385) Schoeller DA. Limitations in the assessment of dietary energy intake by self-report. [Review] [30 refs]. Metabolism: Clinical and Experimental 1995;44:18-22. Review article
Ref ID: 4994

(4386) Schoeller DA. Recent advances from application of doubly labeled water to measurement of human energy expenditure. Journal of Nutrition 1999;129(10):1765-8. Review article
Ref ID: 5590

(4387) Schofield L, Mummery WK, Schofield G. Effects of a controlled pedometer-intervention trial for low-active adolescent girls. Medicine and Science in Sports and Exercise 2005 August;37(8):1414-20. Not All Participants were Overweight and/or Obese
Ref ID: 1480

(4388) Schonfeld-Warden N, Warden CH. Pediatric obesity. An overview of etiology and treatment. [Review] [157 refs]. Pediatric Clinics of North America 1997 April;44(2):339-61. Review article
Ref ID: 2143

(4389) Schooling CM, Jiang CQ, Lam TH, Zhang WS, Cheng KK, Leung GM. Life-course origins of social inequalities in metabolic risk in the population of a developing country. American Journal of Epidemiology 2008 February 15;167(4):419-28. Study limited to adults
Ref ID: 1000

(4390) Schou AJ, Heuck C, Wolthers OD. Differential effects of short-term prednisolone treatment on peripheral and abdominal subcutaneous thickness in children assessed by ultrasound. Steroids 2003;68(6):525-31. Drug intervention study
Ref ID: 3302

(4391) Schöller GM, Kakuda TN, De SG, Woodfall B, Berckmans C, Peeters M, Hoetelmans RM. Effects of hepatic impairment on the steady-state pharmacokinetics of etravirine 200 mg BID: an open-label, multiple-dose, controlled Phase I study in adults. Clinical Therapeutics 2010;32:328-37. Drug intervention study
Ref ID: 4995

(4392) Schranz N, Tomkinson G, Olds T. What is the effect of resistance training on the strength, body composition and psychosocial status of overweight and obese children and adolescents? A Systematic review and meta-analysis. Sports Medicine 2013 September;43(9):893-907. Inappropriate Study Design
Ref ID: 6059

(4393) Schroeder DG, Pachón H, Dearden KA, Kwon CB, Ha TT, Lang TT, Marsh DR. An integrated child nutrition intervention improved growth of younger, more malnourished children in northern Viet Nam. Food and Nutrition Bulletin 2002;23:53-61. Diet Intervention Study
Ref ID: 4996

(4394) Schroeder ET, Hawkins SA, Jaque SV. Musculoskeletal adaptations to 16 weeks of eccentric progressive resistance training in young women. Journal of Strength and Conditioning Research 2004 May;18(2):227-35. Study limited to adults
Ref ID: 1626

(4395) Schubert R, Leupold M. Advantages and disadvantages of vegetarian diets. Ernahrungs-Umschau 2001;48(3):96-+. Diet Intervention Study
Ref ID: 5591

(4396) Schuler G. Primary prevention: Physical activity. Zeitschrift fur Kardiologie 2004;93:8-15. Review article
Ref ID: 5592

(4397) Schulman S, Lindmarker P, Johnsson H. Influence of changes in lifestyle on fibrinolytic parameters and recurrence rate in patients with venous thromboembolism. Blood Coagulation and Fibrinolysis 1995 June;6(4):311-6. Off topic
Ref ID: 2198

(4398) Schulte J, Osborne J, Benson JW, Cooke R, Drayton M, Murphy J, Rennie J, Speidel B. Developmental outcome of the use of etamsylate for prevention of periventricular haemorrhage in a randomised controlled trial. Archives of disease in Childhood Fetal and Neonatal Edition 2005;90:F31-F35. Subjects less than 2 years old
Ref ID: 4998

(4399) Schum J, Blumenstock G, Weber K, Schweizer R, Pfaff C, Schurr N, Ranke MB, Binder G, Ehehalt S, DISKUS-Study Group. Variants of the FTO gene in obese children and their impact on body composition and metabolism before and after lifestyle intervention. Experimental and Clinical Endocrinology and Diabetes 2012 March;120(3):128-31. Longitudinal Study
Ref ID: 2782

(4400) Schuster MA, Elliott MN, Kanouse DE, Wallander JL, Tortolero SR, Ratner JA, Klein DJ, Cuccaro PM, Davies SL, Banspach SW. Racial and ethnic health disparities among fifth-graders in three cities. New England Journal of Medicine 2012 August 23;367(8):735-45. Inappropriate Outcomes
Ref ID: 6060

(4401) Schwab KO. [Evidence-based procedures for the treatment of diseases associated with childhood obesity]. Bundesgesundheitsblatt Gesundheitsforschung Gesundheitsschutz 2013 April;56(4):551-4. Inappropriate Study Design
Ref ID: 6061

(4402) Schwartz RS, Jaeger LF, Veith RC, Lakshminarayan S. The effect of diet or exercise on plasma norepinephrine kinetics in moderately obese young men. Int J Obes 1990 January;14(1):1-11. Study limited to adults
Ref ID: 441

(4403) Schweitzer PB, Miqüelluti D. Estudo do padrão postural de jogadores de futebol da categoria infantil. Fisioterapia Brasileira 2005 December;6(6):419-

23. Off topic
Ref ID: 4533

(4404) Schwingshandl J, Sudi K, Eibl B, Wallner S, Borkenstein M. Effect of an individualised training programme during weight reduction on body composition: a randomised trial. Archives of Disease in Childhood 1999 November;81(5):426-8. No exercise only group, No comparative control group
Ref ID: 407

(4405) Scolapio JS, Lankisch M, Raimondo M. Nutritional support in pancreatitis. Scandinavian Journal of Gastroenterology 2000 October;35(10):1010-5. Off topic
Ref ID: 3737

(4406) Scott LJ, Goa KL. Galantamine: A review of its use in Alzheimer's disease. Drugs 2000 November;60(5):1095-122. Review article
Ref ID: 3738

(4407) Scott MB, Skoner DP. Short-term and long-term safety of budesonide inhalation suspension in infants and young children with persistent asthma. The Journal of Allergy and Clinical Immunology 1999;104:200-9. Drug intervention study
Ref ID: 4999

(4408) Scott MM, Cohen DA, Evenson KR, Elder J, Catellier D, Ashwood JS, Overton A. Weekend schoolyard accessibility, physical activity, and obesity: the Trial of Activity in Adolescent Girls (TAAG) study. Preventive Medicine 2007 May;44(5):398-403. Not a randomized controlled trial (RCT)
Ref ID: 1234

(4409) Scott RA, Bailey MES, Moran CN, Wilson RH, Fuku N, Tanaka M, Tsiokanos A, Jamurtas AZ, Grammatikaki E, Moschonis G, Manios Y, Pitsiladis YP. FTO genotype and adiposity in children: physical activity levels influence the effect of the risk genotype in adolescent males. European Journal of Human Genetics 2010;18(12):1339-43. Cross-sectional study
Ref ID: 5593

(4410) Scranton RE, Gaziano JM, Rutty D, Ezrokhi M, Cincotta A. A randomized, double-blind, placebo-controlled trial to assess safety and tolerability during treatment of type 2 diabetes with usual diabetes therapy and either Cycloset or placebo. BMC Endocrine Disorders 2007 January;7:3-7. Drug intervention study
Ref ID: 3739

(4411) Scrutinio D, Bellotto F, Lagioia R, Passantino A. Physical activity for coronary heart disease: cardioprotective mechanisms and effects on prognosis. [Review] [103 refs]. Monaldi Archives for Chest Disease 2005 June;64(2):77-87. Review article
Ref ID: 1435

(4412) Seedat YK, Croasdale MA, Milne FJ, Opie LH, Pinkney-Atkinson VJ, Rayner BL, Veriava Y. South African hypertension guideline 2006. South African Medical Journal 2006;96(4 II):337-62. Review article
Ref ID: 3303

(4413) Segura DdCA, Nascimento FCd, Sarro TCdA, Navarro M, Guimarães LF, Orssato L, Pires JRM. Efeitos das técnicas de reabilitação cardiorrespiratória em crianças portadoras da síndrome de Prader-Willi. Effects of cardiorrespiratory rehabilitation techniques in children suffering from Prader-Willi syndrome. Arquivos de Ciências da Saúde da UNIPAR 2009 August;13(2). Not a randomized controlled trial (RCT)
Ref ID: 4534

(4414) Seidel B, Krebs DE. Base of support is not wider in chronic ataxic and unsteady patients. Journal of Rehabilitation Medicine 2002 November;34(6):288-92. Off topic
Ref ID: 3740

(4415) Sekerel BE, Saraçlar Y, Etikan I, Kalayci O. Comparison of two different dose regimens of nedocromil sodium with placebo in the management of childhood asthma. Journal of investigational Allergology and Clinical Immunology 1999;9:293-8. Drug intervention study
Ref ID: 862

(4416) Selewski DT, Collier DN, MacHardy J, Gross HE, Pickens EM, Cooper AW, Bullock S, Earls MF, Pratt KJ, Scanlon K, McNeill JD, Messer KL, Lu Y, Thissen D, DeWalt DA, Gipson DS. Promising insights into the health related quality of life for children with severe obesity. Health and Quality of Life Outcomes 2013;11:29. Inappropriate Study Design
Ref ID: 6062

(4417) Sellers EAC, Moore K, Dean HJ. Clinical Management of Type 2 Diabetes in Indigenous Youth. Pediatric Clinics of North America 2009;56(6):1441-59. Review article
Ref ID: 3304

(4418) Selsby JT, Beckett KD, Kern M, Devor ST. Swim performance following creatine supplementation in Division III athletes. Journal of Strength and Conditioning Research 2003 August;17(3):421-4. Diet Intervention or Supplement Study
Ref ID: 1722

(4419) Selvadurai HC, Blimkie CJ, Meyers N, Mellis CM, Cooper PJ, Van Asperen PP. Randomized controlled study of in-hospital exercise training programs in children with cystic fibrosis. Pediatric Pulmonology 2002 March;33(3):194-200. Not All Participants were Overweight and/or Obese
Ref ID: 1848

(4420) Senbanjo IO, Oshikoya KA. Physical activity and body mass index of school children and adolescents in Abeokuta, Southwest Nigeria. World Journal of Pediatrics 2010 August;6(3):217-22. Cross-sectional study
Ref ID: 474

(4421) Seo DC, Sa J. A Meta-analysis of obesity interventions among U.S. minority children. Journal of Adolescent Health 2010;46(4):309-23. Review article
Ref ID: 3305

(4422) Serbescu C, Flora D, Hantiu I, Greene D, Laurent BC, Courteix D. Effect of a six-month training programme on the physical capacities of Romanian schoolchildren. Acta Paediatrica 2006 October;95(10):1258-65. Not All Participants were Overweight and/or Obese
Ref ID: 1348

(4423) Serrano E, Leiferman J, Dauber S. Self-efficacy and health behaviors toward the prevention of diabetes among high risk individuals living in Appalachia. Journal of Community Health 2007 April;32(2):121-33. Survey or questionnaire
Ref ID: 1214

(4424) Serrano SQ, Vasconcelos MGLd, Silva GAPd, Cerqueira MMOd, Pontes CM. Percepção do adolescente obeso sobre as repercussões da obesidade em sua saúde. Percepción de los adolescentes obesos respecto de los efectos de la obesidad en su salud. Obese adolescents' perceptions about the repercussions of obesity on their health. Revista da Escola de Enfermagem da USP 2010 March;44(1):25-31. Not an exercise intervention study
Ref ID: 4535

(4425) Sewani-Rusike CR, Mudambo KS, Tendaupenyu G, Dzuda C, Tafirenyika A, Zenda E. Effects of the Zimbabwe Defence Forces training programme on body composition and reproductive hormones in male army recruits.[Erratum appears in Central African Journal of Medicine 2000 May;46(5):140]. Central African Journal of Medicine 2000 February;46(2):27-31. Study limited to adults
Ref ID: 1938

(4426) Shackelford TK, Schmitt DP, Buss DM. Universal dimensions of human mate preferences. Personality and Individual Differences 2005;39(2):447-58. Off topic
Ref ID: 5594

(4427) Shaffer ML, Hiriote S. Analysis of time-to-event and duration outcomes in neonatal clinical trials with twin births. Contemporary Clinical Trials 2009;30(2):150-4. Subjects less than 2 years old
Ref ID: 5595

(4428) Shaffer SE, Tepper BJ. Effects of learned flavor cues on single meal and daily food-intake in humans. Physiology and Behavior 1994;55(6):979-86. Diet

Intervention Study
Ref ID: 5596

(4429) Shah S, White M, Uryniak T, O'Brien CD. The functionality of a budesonide/formoterol pressurized metered-dose inhaler with an integrated actuation counter. Allergy and Asthma Proceedings 2010;31:40-8. Drug intervention study
Ref ID: 5000

(4430) Shaibi GQ, Davis JN, Weigensberg MJ, Goran MI. Improving insulin resistance in obese youth: choose your measures wisely. International Journal of Pediatric Obesity 2011 June;6(2-2):e290-e296. No non-intervention control group
Ref ID: 2783

(4431) Shalev RS. Developmental dyscalculia. Journal of Child Neurology 2004;19(10):765-71. Off topic
Ref ID: 5597

(4432) Shalitin S, Ashkenazi-Hoffnung L, Yackobovitch-Gavan M, Nagelberg N, Karni Y, Hershkovitz E, Loewenthal N, Shtaif B, Gat-Yablonski G, Phillip M. Effects of a twelve-week randomized intervention of exercise and/or diet on weight loss and weight maintenance, and other metabolic parameters in obese preadolescent children. Hormone Research 2009;72(5):287-301. No comparative control group
Ref ID: 89

(4433) Shalitin S, Yackobovitch-Gavan M, Phillip M. Prevalence of thyroid dysfunction in obese children and adolescents before and after weight reduction and its relation to other metabolic parameters. Hormone Research 2009;71(3):155-61. Prevalence study
Ref ID: 802

(4434) Shamah LT, Morales RC, Amaya CC, Salazar CA, Jimenez AA, Mendez GH, I. Effectiveness of a diet and physical activity promotion strategy on the prevention of obesity in Mexican school children. BMC Public Health 2012;12:152. Diet & Exercise intervention
Ref ID: 2784

(4435) Shank RP, Maryanoff BE. Molecular Pharmacodynamics, Clinical Therapeutics, and Pharmacokinetics of Topiramate. CNS Neuroscience and Therapeutics 2008;14(2):120-42. Drug intervention study
Ref ID: 3741

(4436) Shankaran S, Woldt E, Nelson J, Bedard M, Delaney B, V. Antenatal phenobarbital therapy and neonatal outcome. II: Neurodevelopmental outcome at 36 months. Pediatrics 1996;97:649-52. Drug intervention study
Ref ID: 5001

(4437) Shankaran S, Bann CM, Bauer CR, Lester BM, Bada HS, Das A, Higgins RD, Poole WK, LaGasse LL, Hammond J, Woldt E. Prenatal cocaine exposure and BMI and blood pressure at 9 years of age. Journal of Hypertension 2010 June;28(6):1166-75. Off topic
Ref ID: 531

(4438) Sharifah WW, Nur HH, Ruzita AT, Roslee R, Reilly JJ. The Malaysian Childhood Obesity Treatment Trial (MASCOT). Malaysian Journal of Nutrition 2011 August;17(2):229-36. Diet Intervention Study
Ref ID: 2785

(4439) Sharma AK, Singh S, Meena S, Kannan AT. Impact of NGO run mid day meal program on nutrition status and growth of primary school children. Indian Journal of Pediatrics 2010;77:763-9. Diet Intervention Study
Ref ID: 5002

(4440) Sharma M. School-based interventions for childhood and adolescent obesity. [Review] [51 refs]. Obesity Reviews 2006 August;7(3):261-9. Review article
Ref ID: 1367

(4441) Sharman IM, Down MG, Sen RN. The effects of vitamin E and training on physiological function and athletic performance in adolescent swimmers. British Journal Of Nutrition 1971 September;26(2):265-76. Diet Intervention or Supplement Study
Ref ID: 2786

(4442) Sharrock KCB, Kuzawa CW, Leonard WR, Tanner S, Reyes-Garcia VE, Vadez V, Huanca T, Mcdade TW. Developmental changes in the relationship between leptin and adiposity among Tsimane children and adolescents. American Journal of Human Biology 2008;20(4):392-8. Cross-sectional study
Ref ID: 5598

(4443) Sheikh S, Bakshi SS, Pahwa SG. Outcome and survival in HIV-infected infants with Pneumocystis carinii pneumonia and respiratory failure. Pediatric Aids and HIV Infection-Fetus to Adolescent 1996;7(3):155-63. Subjects less than 2 years old
Ref ID: 5599

(4444) Shelton D, Le GK, Norton L, Stanton-Cook S, Morgan J, Masterman P. Randomised controlled trial: A parent-based group education programme for overweight children. Journal of Paediatrics and Child Health 2007 December;43(12):799-805. Not an exercise intervention study
Ref ID: 218

(4445) Shen B, Reinhart-Lee T, Janisse H, Brogan K, Danford C, Jen KL. African American preschool children's physical activity levels in Head Start. Research Quarterly for Exercise and Sport 2012 June;83(2):168-74. Cross-sectional

study
Ref ID: 2787

(4446) Shepard J, Carlson JS. An empirical evaluation of school-based prevention programs that involve parents. Psychology in the Schools 2003 November;40(6):641. Review article
Ref ID: 3857

(4447) Shephard RJ, Lavallee H. Effects of enhanced physical education on lung volumes of primary school children. Journal of Sports Medicine and Physical Fitness 1996 September;36(3):186-94. Not All Participants were Overweight and/or Obese
Ref ID: 2153

(4448) Sherwood NE, Taylor WC, Treuth M, Klesges LM, Baranowski T, Zhou A, Pratt C, McClanahan B, Robinson TN, Pruitt L, Miller W. Measurement characteristics of activity-related psychosocial measures in 8- to 10-year-old African-American girls in the Girls Health Enrichment Multisite Study (GEMS). Preventive Medicine 2004 May;38:Suppl-8. Off topic
Ref ID: 1648

(4449) Sherwood NE, Levy RL, Langer SL, Senso MM, Crain AL, Hayes MG, Anderson JD, Seburg EM, Jeffery RW. Healthy Homes/Healthy Kids: a randomized trial of a pediatric primary care-based obesity prevention intervention for at-risk 5-10 year olds. Contemporary Clinical Trials 2013 September;36(1):228-43. Inappropriate Intervention
Ref ID: 6064

(4450) Sherwood NE, French SA, Veblen-Mortenson S, Crain AL, Berge J, Kunin-Batson A, Mitchell N, Senso M. NET-Works: Linking families, communities and primary care to prevent obesity in preschool-age children. Contemporary Clinical Trials 2013 November;36(2):544-54. Inappropriate Intervention
Ref ID: 6063

(4451) Shikora SA, Bergenstal R, Bessler M, Brody F, Foster G, Frank A, Gold M, Klein S, Kushner R, Sarwer DB. Implantable gastric stimulation for the treatment of clinically severe obesity: results of the SHAPE trial. Surgery for Obesity and Related Diseases 2009 January;5(1):31-7. Diet Intervention Study
Ref ID: 805

(4452) Shin L, Bregman H, Frazier J, Noyes N. An overview of obesity in children with psychiatric disorders taking atypical antipsychotics. [Review] [113 refs]. Harvard Review of Psychiatry 2008;16(2):69-79. Review article
Ref ID: 971

(4453) Shinohara A, Takakura J, Yamane A, Suzuki M. Effect of the classic 1-week glycogen-loading regimen on fat-loading in rats and humans. Journal of

Nutritional Science and Vitaminology 2010;56(5):299-304. Animal study
Ref ID: 2788

(4454) Shipp A, Lawrence G, Gentry R, McDonald T, Bartow H, Bounds J, Macdonald N, Clewell H, Allen B, Van Landingham C. Acrylamide: Review of toxicity data and dose-response analyses for cancer and noncancer effects. Critical Reviews in Toxicology 2006 July;36(6/7):481-608. Review article
Ref ID: 3742

(4455) Shonkoff JP, Richter L, van der Gaag J, Bhutta ZA. An integrated scientific framework for child survival and early childhood development. Pediatrics 2012;129(2):E460-E472. Off topic
Ref ID: 5600

(4456) Showell NN, Fawole O, Segal J, Wilson RF, Cheskin LJ, Bleich SN, Wu Y, Lau B, Wang Y. A systematic review of home-based childhood obesity prevention studies. Pediatrics 2013 July;132(1):e193-e200. Inappropriate Study Design
Ref ID: 6065

(4457) Shrestha BP, Bhandari B, Manandhar DS, Osrin D, Costello A, Saville N. Community interventions to reduce child mortality in Dhanusha, Nepal: study protocol for a cluster randomized controlled trial. Trials 2011;12:136. Off topic
Ref ID: 5003

(4458) Shrewsbury VA, O'Connor J, Steinbeck KS, Stevenson K, Lee A, Hill AJ, Kohn MR, Shah S, Torvaldsen S, Baur LA. A randomised controlled trial of a community-based healthy lifestyle program for overweight and obese adolescents: the Loozit study protocol. BMC Public Health 2009 April 29;9:119.:119. Description versus conduct of study, No comparative control group
Ref ID: 123

(4459) Shulruf B, Morton S, Goodyear-Smith F, O'Loughlin C, Dixon R. Designing Multidisciplinary longitudinal studies of human development - Analyzing past research to inform methodology. Evaluation and the Health Professions 2007;30(3):207-28. Review article
Ref ID: 5601

(4460) Shultz SP, Sitler MR, Tierney RT, Hillstrom HJ, Song JS. Effects of pediatric obesity on joint kinematics and kinetics during 2 walking cadences. Archives of Physical Medicine and Rehabilitation 2009;90(12):2146-54. Survey or questionnaire
Ref ID: 5602

(4461) Siceloff ER, Coulon SM, Wilson DK. Physical activity as a mediator linking neighborhood environmental supports and obesity in African Americans in the path trial. Health Psychology 2014 May;33(5):481-9. Inappropriate Study

Design
Ref ID: 6066

(4462) Sichieri R, Souza RA. [Strategies for obesity prevention in children and adolescents]. [Portuguese]. Cadernos de Saude Publica 2008;24:Suppl-23. Review article
Ref ID: 907

(4463) Sichieri R, Souza RAd. Estratégias para prevenção da obesidade em crianças e adolescentes. Cadernos de Saúde Pública 2008;24(supl.2):s209-s234. Review article
Ref ID: 4536

(4464) Sichieri R, Moura ECd. Análise multinível das variações no índice de massa corporal entre adultos, Brasil, 2006. Análisis multinivel de las variaciones en el índice de masa corporal entre adultos, Brasil, 2006. A multilevel analysis of variations in body mass index among adults, Brazil, 2006. Revista de Saúde Pública 2009 November;43(supl.2):90-7. Study limited to adults
Ref ID: 4537

(4465) Siconolfi SF, Lasater TM, McKinlay S, Boggia P, Carleton RA. Physical fitness and blood pressure: the role of age. American Journal of Epidemiology 1985 September;122(3):452-7. Off topic
Ref ID: 2357

(4466) Sidiropoulou MP, Fotiadou EG, Tsimaras VK, Zakas AP, Angelopoulou NA. The effect of interval training in children with exercise-induced asthma competing in soccer. Journal of Strength and Conditioning Research 2007;21:446-50. Not a randomized controlled trial (RCT)
Ref ID: 5004

(4467) Sidney K, Jette M. Characteristics of women performing strength training: comparison of participants and dropouts. Journal of Sports Medicine and Physical Fitness 1992 March;32(1):84-95. Study limited to adults
Ref ID: 2286

(4468) Sidney S, Sternfeld B, Gidding SS, Jacobs DR, Jr., Bild DE, Oberman A, Haskell WL, Crow RS, Gardin JM. Cigarette smoking and submaximal exercise test duration in a biracial population of young adults: the CARDIA study. Medicine and Science in Sports and Exercise 1993 August;25(8):911-6. Study limited to adults
Ref ID: 2256

(4469) Siebler J, Galle PR. Treatment of nonalcoholic fatty liver disease. [Review] [77 refs]. World Journal of Gastroenterology 2006 April 14;12(14):2161-7. Review article
Ref ID: 1397

(4470) Siega-Riz AM, El GL, Mobley C, Gillis B, Stadler D, Hartstein J, Volpe SL, Virus A, Bridgman J, HEALTHY study group. The effects of the HEALTHY study intervention on middle school student dietary intakes. International Journal of Behavioral Nutrition and Physical Activity 2011;8:7. Diet Intervention Study
Ref ID: 2789

(4471) Siegel EH, Stoltzfus RJ, Kariger PK, Katz J, Khatry SK, LeClerq SC, Pollitt E, Tielsch JM. Growth indices, anemia, and diet independently predict motor milestone acquisition of infants in south central Nepal. Journal of Nutrition 2005;135:2840-4. Subjects less than 2 years old
Ref ID: 5005

(4472) Siegrist M, Hanssen H, Lammel C, Haller B, Halle M. A cluster randomised school-based lifestyle intervention programme for the prevention of childhood obesity and related early cardiovascular disease (JuvenTUM 3). BMC Public Health 2011;11:258. Lifestyle Intervention
Ref ID: 2790

(4473) Siegrist M, Lammel C, Haller B, Christle J, Halle M. Effects of a physical education program on physical activity, fitness, and health in children: the JuvenTUM project. Scandinavian Journal of Medicine and Science in Sports 2013 June;23(3):323-30. Inappropriate Study Design
Ref ID: 6067

(4474) Siekmeier R, Scharnagl H, Kostner GM, Grammer T, Stojakovic T, Marz W. Variation of Lp(a) plasma concentrations in health and disease. Open Clinical Chemistry Journal 2010 January;3:72-89. Off topic
Ref ID: 3743

(4475) Siemann M. Test of a simple-model of transitive inference using a nonverbal form of presentation. Zeitschrift fur Experimentelle und Angewandte Psychologie 1994;41(4):584-616. Off topic
Ref ID: 5603

(4476) Sies H, Stahl W. Nutritional protection against skin damage from sunlight. Annual Review of Nutrition 2004 August;24(1):173-200. Review article
Ref ID: 3744

(4477) Sigmund E, Sigmundova D. Longitudinal 2-year follow-up on the effect of a non-randomised school-based physical activity intervention on reducing overweight and obesity of Czech children aged 10-12 years. International Journal of Environmental Research and Public Health 2013 August;10(8):3667-83. Inappropriate Study Design
Ref ID: 6068

(4478) Sigmundova D, El AW, Sigmund E. Neighbourhood environment correlates of physical activity: a study of eight Czech regional towns. International Journal of Environmental Research and Public Health [Electronic Resource] 2011

February;8(2):341-57. Survey or questionnaire
Ref ID: 2791

(4479) Sijtsma A, Schierbeek H, Goris AH, Joosten KF, van K, I, Corpeleijn E, Sauer PJ. Validation of the TracmorD triaxial accelerometer to assess physical activity in preschool children. Obesity (Silver Spring) 2013 September;21(9):1877-83. Inappropriate Outcomes
Ref ID: 6069

(4480) Silman AJ, O'Neill TW, Cooper C, Kanis J, Felsenberg D. Influence of physical activity on vertebral deformity in men and women: results from the European Vertebral Osteoporosis Study. Journal of Bone and Mineral Research 1997 May;12(5):813-9. Study limited to adults
Ref ID: 2142

(4481) Silva AJ, Machado R, V, Guidetti L, Bessone AF, Mota P, Freitas J, Baldari C. Effect of creatine on swimming velocity, body composition and hydrodynamic variables. Journal of Sports Medicine and Physical Fitness 2007 March;47(1):58-64. Diet Intervention or Supplement Study
Ref ID: 1262

(4482) Silva AM, Santos DA, Matias CN, Rocha PM, Petroski EL, Minderico CS, Sardinha LB. Changes in regional body composition explain increases in energy expenditure in elite junior basketball players over the season. European Journal of Applied Physiology 2012;112(7):2727-37. Cross-sectional study
Ref ID: 5604

(4483) Silva D, Matos A, Magalhaes T, Martins V, Ricardo L, Almeida H. [Prevalence of hypertension in Portuguese adolescents in Lisbon, Portugal]. Revista Portuguesa de Cardiologia 2012 December;31(12):789-94. Inappropriate Study Design
Ref ID: 6070

(4484) Silva DAS, Smith-Menezes A, Almeida-Gomes M, Sousa TFd. Estágios de mudanças de comportamento para atividade física em estudantes de uma cidade do Brasili. Stages of behavioral change regarding physical activity in students from a Brazilian town. Estados de cambio en actividad física en estudiantes de una ciudad de Brasil. Revista de Salud Pública 2010 August;12(4):623-34. Cross-sectional study
Ref ID: 4538

(4485) Silva DA, Pelegrini A, Grigollo LR, Silva AFd, Petroski EL. Diferenças e similaridades dos estagios de mudança de comportamento para atividade fisica em adolescentes de duas areas brasileiras. Differences and similarities in stages of behavioral change related to physical activity in adolescents from two regions of Brazil. Revista Paulista de Pediatria 2011 June;29(2):193-201.

Cross-sectional study
Ref ID: 4539

(4486) Silva DAS, Petroski EL, Pelegrini A. Da evidência à intervenção: programa de exercício físico para adolescentes com excesso de peso em Florianópolis, SC. Revista Brasileira de Atividade Física e Saúde 2009;14(2). Not a randomized controlled trial (RCT)
Ref ID: 4540

(4487) Silva DAS, Lima JdO, Silva RJdS, Prado RL. Nível de atividade física e comportamento sedentário em escolares. Physical activity level and sedentary behavior among students. Revista Brasileira de Cineantropometria e Desempenho Humano 2009;11(3):299-306. Cross-sectional study
Ref ID: 4541

(4488) Silva DAS, Pelegrini A, Petroski EL, Gaya ACA. Comparação do crescimento de crianças e adolescentes brasileiros com curvas de referência para crescimento físico: dados do Projeto Esporte Brasil. Comparison between the growth of Brazilian children and adolescents and the reference growth charts: data from a Brazilian project. Jornal de Pediatria 2010 April;86(2):115-20. Cross-sectional study
Ref ID: 4542

(4489) Silva ÉdC, Morais FDd, Formiga CK, Viana FP. Síndrome da duplicação do cromossomo 11: relato de caso. Temas Sobre Desenvolvimento 2010 June;17(99):127-31. Off topic
Ref ID: 4543

(4490) Silva HGV, Chiara VL, Barros ME, Rêgo AL, Ferreira A, Pitasi BA, Mattos T. Diagnóstico do estado nutricional de escolares: comparação entre critério nacional e internacional. Diagnosing the nutritional status of schoolchildren: a comparison between Brazilian and international criteria. Jornal de Pediatria 2008 December;84(6):550-5. Diet Intervention Study
Ref ID: 4544

(4491) Silva KSd, Rozenberg R, Bonan C, Chuva VCC, Costa SFd, Gomes MAdSM. Gravidez recorrente na adolescência e vulnerabilidade social no Rio de Janeiro (RJ, Brasil): uma análise de dados do Sistema de Nascidos Vivos. Repeated pregnancy among adolescents and social vulnerability in Rio de Janeiro (RJ, Brazil): data analysis of Information System on Live Births. Ciência and Saúde Coletiva 2011 May;16(5):2485-93. Off topic
Ref ID: 4545

(4492) Silva KS, Lopes AS. Excesso de peso, pressão arterial e atividade física no deslocamento à escola. Excess weight, arterial pressure and physical activity in commuting to school: correlations. Arquivos Brasileiros de Cardiologia 2008

August;91(2):93-101. Survey or questionnaire
Ref ID: 4546

(4493) Silva KSd, Lopes AdS, Silva FMd. Atividade física no deslocamento à escola e no tempo livre em crianças e adolescentes da cidade de João Pessoa, PB, Brasil. Revista Brasileira de Ciência e Movimento 2007;15(3):61-70. Survey or questionnaire
Ref ID: 4547

(4494) Silva KSd, Lopes AdS, Silva FMd. Comportamentos sedentários associados ao excesso de peso corporal. Revista Brasileira de Educação Física e Esporte 2007 June;21(2):135-41. Survey or questionnaire
Ref ID: 4548

(4495) Silva KSd, Nahas MV, Hoefelmann LP, Lopes AdS, Oliveira ESd. Associações entre atividade física, índice de massa corporal e comportamentos sedentários em adolescentes. Revista Brasileira de Eidemiologia 2008 March;11(1):159-68. Survey or questionnaire
Ref ID: 4549

(4496) Silva KSd, Nahas MV, Peres KG, Lopes AdS. Fatores associados à atividade física, comportamento sedentário e participação na Educação Física em estudantes do Ensino Médio em Santa Catarina, Brasil. Factors associated with physical activity, sedentary behavior, and participation in physical education among high school students in Santa Catarina State, Brazil. Cadernos de Saúde Pública 2009 October;25(10):2187-200. Survey or questionnaire
Ref ID: 4550

(4497) Silva LJd, Andrade DR, Oliveira LC, Araújo TLd, Silva AP, Matsudo VKR. Associação entre shuttle run e shuttle run com bola e sua relação com o desempenho do passe no futebol. Revista Brasileira de Ciência e Movimento 2006;14(3):7-14. Cross-sectional study
Ref ID: 4551

(4498) Silva LO, Silva PLd, Nogueira AMOC, Silva MB, Luz GCP, Narciso FV, Carvalho EMd, Cheik NC. Avaliação do broncoespasmo induzido pelo exercício avaliado pelo peak flow meter em adolescentes obesos. Evaluation of exercise-induced bronchospasm assessed by peak flow meter in obese adolescents. Revista Brasileira de Medicina do Esporte 2011 December;17(6):393-6. Off topic
Ref ID: 4552

(4499) Silva LMd, Lins RG, Xidis LJC, Oliveira MCd, Ribeiro MCM. Leishmaniose visceral associada a linfoma de Hodgkin. Brasília Médica 2007;44(3):211-4. Case-Control / Case Study
Ref ID: 4553

(4500) Silva M, Collipal L, Martínez C, Torres I. Análisis del IMC y somatotipo en una muestra de adolescentes con sobrepeso y obesidad en Temuco-Chile. International Journal of Morphology 2008 September;26(3):707-11. Cross-sectional study
Ref ID: 4554

(4501) Silva MdS, Teixeira PC, Matsudo S, Matsudo V. Relação do tempo de TV e aptidão física de escolares de uma região de baixo nível sócio-econômico. Revista Brasileira de Ciência e Movimento 2007;15(4):21-30. Survey or questionnaire
Ref ID: 4555

(4502) Silva MPd, Gasparotto GdS, Smolarek AdC, Dellagrana RA, Mascarenhas LPG, Campos Wd, Silva MPd, Pinheiro ML, Bonfim AL. Comportamento sedentário relacionado ao sobrepeso e à obesidade em crianças e adolescentes. Pensar a Prática (Impresso) 2010 August;13(2):1-15. Survey or questionnaire
Ref ID: 4556

(4503) Silva PVC, Costa Júnior ÁL. Efeitos da atividade física para a saúde de crianças e adolescents. The effects of physical activity on the health of children and adolescents. Psicologia Argumento 2011 March;29(64):41-50. Review article
Ref ID: 4558

(4504) Silva QHd, Pedro FL, Kirsten VR. Satisfação corporal e características de lipodistrofia em crianças e adolescentes com HIV/AIDS em uso de terapia antirretroviral de alta potência. Body satisfaction and lipodystrophy characteristics in HIV/AIDS children and teenagers undergoing highly active antiretroviral therapy. Revista Paulista de Pediatria 2011 September;29(3):357-63. Cross-sectional study
Ref ID: 4559

(4505) Silva RR, Siqueira RV, Silva ACd, Andrade GF, Monteiro CdSM, Grasselli CdSM, Martino HSD. Prevalência de parasitoses e estado nutricional de pré-escolares de centros educacionais municipais no sul de Minas GeraisPrevalence of parasitic diseases. Prevalence of parasitic diseases and nutritional status of preschool children in municipal educational centers in the south of Minas Gerais state. Nutrire Revista da Sociedade Brasileira de Alimentação e Nutrição 2010 April;35(1). Off topic
Ref ID: 4560

(4506) Silva R, Malina RM. Sobrepeso, atividade física e tempo de televisão entre adolescentes de Niterói, Rio de Janeiro, Brasil. Revista Brasileira de Ciência e Movimento 2003;11(4):63-6. Survey or questionnaire
Ref ID: 4561

(4507) Silva SRG, Martins JL, Seixas S, Silva DCGd, Lemos SPP, Lemos PVB. Defeitos congênitos e exposição a agrotóxicos no Vale do São Francisco. Congenital defects and exposure to pesticides in São Francisco Valley. Revista Brasileira de Ginecologia e Obstetrícia 2011 January;33(1):20-6. Off topic
Ref ID: 4562

(4508) Silva SPd, Maia JAR. Classificação morfológica de volibolistas do sexo feminino em escalões de formação. Revista Brasileira de Cineantropometria e Desempenho Humano 2003;5(2). Cross-sectional study
Ref ID: 4563

(4509) Silva SPd, Santos ACdS, Silva HMd, Costa CLA, Nobre GC. Aptidão cardiorespiratória e composição corporal em crianças e adolescentes. Motriz Revista de Educação Física (Improv) 2010 September;16(3):664-71. Cross-sectional study
Ref ID: 4564

(4510) Silva SSPd, Maia ÂdC. Adversidade na infância, características psicológicas e problemas de saúde física: comparação entre obesos e não obesos. Adverse childhood experiences, psychological characteristics and physical health problems: comparison among obese and non-obese. Revista de Psiquiatria Clínica (São Paulo) 2011;38(5):194-200. Off topic
Ref ID: 4565

(4511) Silva TS, Longui CA, Faria CD, Rocha MN, Melo MR, Faria TG, de Souza e Almeida JA, Hayashi LF, Kater CE. Impact of prolonged physical training on the pituitary glucocorticoid sensitivity determined by very low dose intravenous dexamethasone suppression test. Hormone and Metabolic Research 2008 October;40(10):718-21. Study limited to adults
Ref ID: 887

(4512) Silveira EF, Silva MCd. Conhecimento sobre atividade física dos estudantes de uma cidade do sul do Brasil. Knowledge about physical activity of students in a city in southern Brazil. Motriz Revista de Educação Física (Improv) 2011 September;17(3):456-67. Cross-sectional study
Ref ID: 4566

(4513) Silveira JAC, Taddei JAAC, Guerra PH, Nobre MRC. A efetividade de intervenções de educação nutricional nas escolas para prevenção e redução do ganho excessivo de peso em crianças e adolescentes: uma revisão sistemáticai. Effectiveness of school-based nutrition education interventions to prevent and reduce excessive weight gain in children and adolescents: a systematic review. Jornal de Pediatria 2011 October;87(5):382-92. Review article
Ref ID: 4567

(4514) Silverman M, Andrea T. Time course of effect of disodium cromoglycate on exercise-induced asthma. Archives of Disease in Childhood 1972 June;47(253):419-22. Drug intervention study
Ref ID: 2792

(4515) Simão M, Hayashida M, Santos CBd, Cesarino EJ, Nogueira MS. Hypertension among undergraduate students from Lubango, Angola. La hipertensión arterial entre universitarios de la ciudad de Lubango, Angola. Hipertensão arterial entre universitários da cidade de Lubango, Angola. Revista Latino-Americana de Enfermagem 2008 August;16(4):672-8. Prevalence study
Ref ID: 4568

(4516) Simmons A, Mavoa HM, Bell AC, de Court, Schaaf D, Schultz J, Swinburn BA. Creating community action plans for obesity prevention using the ANGELO (Analysis Grid for Elements Linked to Obesity) Framework. Health Promotion International 2009 December;24(4):311-24. Off topic
Ref ID: 645

(4517) Simon C, Wagner A, DiVita C, Rauscher E, Klein-Platat C, Arveiler D, Schweitzer B, Triby E. Intervention centred on adolescents' physical activity and sedentary behaviour (ICAPS): concept and 6-month results. International Journal of Obesity and Related Metabolic Disorders 2004 November;28 Suppl 3:S96-S103.:S96-S103. Same subjects as another study already included, Not All Participants were Overweight and/or Obese
Ref ID: 322

(4518) Simon C, Wagner A, Platat C, Arveiler D, Schweitzer B, Schlienger JL, Triby E. ICAPS: a multilevel program to improve physical activity in adolescents. Diabetes and Metabolism 2006 February;32(1):41-9. Primary outcome(s) not assessed
Ref ID: 282

(4519) Simon C, Schweitzer B, Oujaa M, Wagner A, Arveiler D, Triby E, Copin N, Blanc S, Platat C. Successful overweight prevention in adolescents by increasing physical activity: a 4-year randomized controlled intervention. International Journal of Obesity (London) 2008 October;32(10):1489-98. Not All Participants were Overweight and/or Obese
Ref ID: 169

(4520) Simon JA, Morrison JA, Similo SL, McMahon RP, Schreiber GB. Correlates of high-density lipoprotein cholesterol in Black girls and White girls: the NHLBI Growth and Health Study. American Journal of Public Health 1995 December;85(12):1698-702. Cross-sectional study
Ref ID: 2194

(4521) Simons-Morton BG, Parcel GS, Baranowski T, Forthofer R, O'Hara NM. Promoting physical activity and a healthful diet among children: Results of a

school-based intervention study. American Journal of Public Health 1991;81:986-91. Lifestyle Intervention
Ref ID: 5006

(4522) Simões D, Meneses RF. Auto-conceito em crianças com e sem obesidade. Psicologia Reflexão e Crítica 2007;20(2):246-51. Survey or questionnaire
Ref ID: 4569

(4523) Simpson D, Perry CM. Atomoxetine. Pediatrics Drugs 2003 June;5(6):407. Drug intervention study
Ref ID: 3745

(4524) Simurina T, Mikulandra S, Mraovic, B, Sonicki Z, Kovacic, M, Dzelalija B, Rudic, M. The effect of propofol and fentanyl as compared with sevoflurane on postoperative vomiting in children after adenotonsillectomy. Collegium Antropologicum 2006;30:343-7. Drug intervention study
Ref ID: 5007

(4525) Sinclair WH, Crowe MJ, Spinks WL, Leicht AS. Thermoregulatory responses of junior lifesavers wearing protective clothing. Journal of Science and Medicine in Sport 2008 November;11(6):542-8. Off topic
Ref ID: 871

(4526) Singh AS, Chin APM, Kremers SP, Visscher TL, Brug J, van MW. Design of the Dutch Obesity Intervention in Teenagers (NRG-DOiT): systematic development, implementation and evaluation of a school-based intervention aimed at the prevention of excessive weight gain in adolescents. BMC Public Health 2006;6:304. Diet & Exercise intervention
Ref ID: 1308

(4527) Singh AS, Chin APM, Brug J, van MW. Short-term effects of school-based weight gain prevention among adolescents. Archives of Pediatrics and Adolescent Medicine 2007 June;161(6):565-71. Not All Participants were Overweight and/or Obese
Ref ID: 1223

(4528) Singh AS, Chin APM, Brug J, van MW. Dutch obesity intervention in teenagers: effectiveness of a school-based program on body composition and behavior. Archives of Pediatric Adolescent Medicine 2009 April;163(4):309-17. No exercise only group
Ref ID: 127

(4529) Singh AS, Kang G, Ramachandran A, Sarkar R, Peter P, Bose A. Locally made ready to use therapeutic food for treatment of malnutrition a randomized controlled trial. Indian Pediatrics 2010;47:679-86. Diet Intervention Study
Ref ID: 5008

(4530) Singh KP, Periyandavar I, Rajadhyaksha GC, Jayaram S, Mishra AB, Kinagi S, Sharma A, Baliga VP. Evaluation of the efficacy, safety and tolerability of miglitol in adult Indian patients with uncomplicated type 2 diabetes mellitus. Journal of the Indian Medical Association 2007 June;105(6):344. Drug intervention study
Ref ID: 1117

(4531) Singhal N, Misra A, Shah P, Gulati S, Bhatt S, Sharma S, Pandey RM. Impact of intensive school-based nutrition education and lifestyle interventions on insulin resistance, Î²-cell function, disposition index, and subclinical inflammation among asian indian adolescents: A controlled intervention study. Metabolic Syndrome and Related Disorders 2011;9(2):143-50. Lifestyle Intervention
Ref ID: 3307

(4532) Siqueira FCV, Nahas MV, Facchini LA, Piccini RX, Tomasi E, Thumé E, Silveira DSd, Hallal PC. Atividade física em profissionais de saúde do Sul e Nordeste do Brasil. Physical activity among health professionals from South and Northeast Brazil. Cadernos de Saúde Pública 2009 September;25(9):1917-28. Cross-sectional study
Ref ID: 4570

(4533) Siqueira PP, Alves JG. Fatores associados ao excesso de peso em crianças de uma favela do Nordeste brasileiro. Variables associated with overweight in children from a shantytown in the Northeast of Brazil. Revista Paulista de Pediatria 2009 September;27(3):251-7. Cross-sectional study
Ref ID: 4571

(4534) Siqueira RSd, Monteiro CA. Amamentação na infância e obesidade na idade escolar em famílias de alto nível socioeconômico. Revista de Saúde Pública 2007 February;41(1):5-12. Cross-sectional study
Ref ID: 4572

(4535) Sivakumar B, Vijayaraghavan K, Vazir S, Balakrishna N, Shatrugna V, Sarma KV, Nair KM, Raghuramulu N, Krishnaswamy K. Effect of micronutrient supplement on health and nutritional status of schoolchildren: study design. Nutrition 2006;22:S1-S7. Diet Intervention or Supplement Study
Ref ID: 5009

(4536) Sjodin A, Gasteyger C, Nielsen AL, Raben A, Mikkelsen JD, Jensen JK, Meier D, Astrup A. The effect of the triple monoamine reuptake inhibitor tesofensine on energy metabolism and appetite in overweight and moderately obese men. International Journal of Obesity 2010 November;34(11):1634-43. Drug intervention study
Ref ID: 2794

(4537) Oblacinska A, Wojciechowska A, Wroclawska M. [Preliminary evaluation of health behavior in obese students from a selected group of Warsaw adolescents]. Medycyna Wieku Rozwojowego 1999;3:303-13. Survey or questionnaire
Ref ID: 5010

(4538) Skelton JA, Irby MB, Grzywacz JG, Miller G. Etiologies of obesity in children: Nature and nurture. Pediatric Clinics of North America 2011;58(6):1333-+. Review article
Ref ID: 5605

(4539) Skinner JS, Jaskolski A, Jaskolska A, Krasnoff J, Gagnon J, Leon AS, Rao DC, Wilmore JH, Bouchard C, HERITAGE Family. Age, sex, race, initial fitness, and response to training: the HERITAGE Family Study. Journal of Applied Physiology 2001 May;90(5):1770-6. Study limited to adults
Ref ID: 1922

(4540) Skjaervo G, Stokke BG, Roskaft E. The rarity of twins: a result of an evolutionary battle between mothers and daughters-or do they agree? Behavioral Ecology and Sociobiology 2009;63(8):1133-40. Off topic
Ref ID: 5606

(4541) Skouteris H, McCabe M, Swinburn B, Hill B. Healthy eating and obesity prevention for preschoolers: a randomised controlled trial. BMC Public Health 2010;10:220. Description versus conduct of study
Ref ID: 2795

(4542) Skoyles JR. Human balance, the evolution of bipedalism and dysequilibrium syndrome. Medical Hypotheses 2006;66(6):1060-8. Off topic
Ref ID: 5607

(4543) Skura CL, Fowler EG, Wetzel GT, Graves M, Spencer MJ. Albuterol increases lean body mass in ambulatory boys with Duchenne or Becker muscular dystrophy. Neurology 2008 January 8;70(2):137-43. Drug intervention study
Ref ID: 1011

(4544) Slama G, Rossi F, Bellisle F, Fiquet P, Chappuis AS, Desplanque N, Laffitte A. Low-fat (41%) butter use decreases butter lipid intake over 4-week trials in healthy persons. Appetite 1995;25:127-31. Diet Intervention Study
Ref ID: 5011

(4545) Slaman J, Roebroeck ME, van MJ, van der Slot WM, Reinders-Messelink HA, Lindeman E, Stam HJ, van den Berg-Emons RJ. Learn 2 Move 16-24: effectiveness of an intervention to stimulate physical activity and improve physical fitness of adolescents and young adults with spastic cerebral palsy; a randomized controlled trial. BMC Pediatric 2010;10:79. Description versus conduct of study
Ref ID: 2796

(4546) Slamberova R, Riley MA, Vathy I. Cross-generational effect of prenatal morphine exposure on neurobehavioral development of rat pups. Physiological Research 2005;54(6):655-60. Animal study
Ref ID: 5608

(4547) Slawta J, Bentley J, Smith J, Kelly J, Syman-Degler L. Promoting healthy lifestyles in children: a pilot program of be a fit kid. Health Promotion Practice 2008 July;9(3):305-12. No control group (NC), Not a randomized controlled trial (RCT)
Ref ID: 455

(4548) Slemenda CW, Reister TK, Hui SL, Miller JZ, Christian JC, Johnston CC. Influences on skeletal mineralization in children and adolescents: evidence for varying effects of sexual maturation and physical activity. The Journal of Pediatrics 1994;125:201-7. Observational study
Ref ID: 5012

(4549) Slentz CA, Bateman LA, Willis LH, Shields AT, Tanner CJ, Piner LW, Hawk VH, Muehlbauer MJ, Samsa GP, Nelson RC, Huffman KM, Bales CW, Houmard JA, Kraus WE. Effects of aerobic vs. resistance training on visceral and liver fat stores, liver enzymes, and insulin resistance by HOMA in overweight adults from STRRIDE AT/RT. American Journal of Physiology - Endocrinology and Metabolism 2011 November;301(5):E1033-E1039. Study limited to adults
Ref ID: 1082

(4550) Slevin E, Truesdale-Kennedy M, McConkey R, Livingstone B, Fleming P. Obesity and overweight in intellectual and non-intellectually disabled children. J Journal of Intellectual Disability Research 2014 March;58(3):211-20. Inappropriate Study Design
Ref ID: 6071

(4551) Sloan RP, McCreath H, Tracey KJ, Sidney S, Liu K, Seeman T. RR interval variability is inversely related to inflammatory markers: the CARDIA study. Molecular Medicine 2007 March;13(3-4):178-84. Off topic
Ref ID: 1207

(4552) Slootmaker SM, Chin APM, Schuit AJ, Seidell JC, van MW. Promoting physical activity using an activity monitor and a tailored web-based advice: design of a randomized controlled trial [ISRCTN93896459]. BMC Public Health 2005;5:134. Description versus conduct of study
Ref ID: 1437

(4553) Slootmaker SM, Chinapaw MJ, Schuit AJ, Seidell JC, van MW. Feasibility and effectiveness of online physical activity advice based on a personal activity monitor: randomized controlled trial. Journal of Medical Internet Research 2009;11(3):e27. Study limited to adults
Ref ID: 700

(4554) Slusser W, Staten K, Stephens K, Liu L, Yeh C, Armstrong S, DeUgarte DA, Haemer M. Payment for obesity services: examples and recommendations for stage 3 comprehensive multidisciplinary intervention programs for children and adolescents. Pediatrics 2011 September;128:Suppl-85. Review article
Ref ID: 2798

(4555) Smeets T, Kremers SP, Brug J, de VH. Effects of tailored feedback on multiple health behaviors.[Erratum appears in Annals of Behavioral Medicine. 2007 Jul-Aug;34(1):104]. Annals of Behavioral Medicine 2007 April;33(2):117-23. Multiple interventions
Ref ID: 1246

(4556) Smith AE, Lockwood CM, Moon JR, Kendall KL, Fukuda DH, Tobkin SE, Cramer JT, Stout JR. Physiological effects of caffeine, epigallocatechin-3-gallate, and exercise in overweight and obese women. Applied Physiology Nutrition and Metabolism 2010 October;35(5):607-16. Study limited to adults
Ref ID: 18

(4557) Smith EM, Capogrossi KL, Estabrooks PA. School wellness policies: Effects of using standard templates. American Journal of Preventive Medicine 2012 September;43(3):304-8. Inappropriate Outcomes
Ref ID: 6072

(4558) Smith JJ, Morgan PJ, Plotnikoff RC, Dally KA, Salmon J, Okely AD, Finn TL, Lubans DR. Smart-phone obesity prevention trial for adolescent boys in low-income communities: The ATLAS RCT. Pediatrics 2014 September;134(3):e723-e731. Inappropriate Intervention
Ref ID: 6073

(4559) Smith JJ, Morgan PJ, Plotnikoff RC, Dally KA, Salmon J, Okely AD, Finn TL, Babic MJ, Skinner G, Lubans DR. Rationale and study protocol for the 'active teen leaders avoiding screen-time' (ATLAS) group randomized controlled trial: an obesity prevention intervention for adolescent boys from schools in low-income communities. Contemporary Clinical Trials 2014 January;37(1):106-19. Inappropriate Study Design
Ref ID: 6074

(4560) Smith LH, Holloman C. Comparing the effects of teen mentors to adult teachers on child lifestyle behaviors and health outcomes in Appalachia. Journal of School Nursing 2013 October;29(5):386-96. Inappropriate Comparison Group
Ref ID: 6075

(4561) Smolak L, Levine MP, Schermer F. A controlled evaluation of an elementary school primary prevention program for eating problems. Journal of Psychosomatic Research 1998 March;44(3-4):339-53. Diet & Exercise intervention
Ref ID: 2093

(4562) Snethen JA, Broome ME, Cashin SE. Effective weight loss for overweight children: a meta-analysis of intervention studies. Journal of Pediatric Nursing 2006 February;21(1):45-56. Review article
Ref ID: 1427

(4563) Snider IP, Bazzarre TL, Murdoch SD, Goldfarb A. Effects of coenzyme athletic performance system as an ergogenic aid on endurance performance to exhaustion. International Journal of Sport Nutrition 1992 September;2(3):272-86. Diet Intervention or Supplement Study
Ref ID: 2276

(4564) So HK, Sung RYT, Li AM, Choi KC, Nelson EAS, Yin J, Ng PC, Fok TF. Higher exercise frequency associated with lower blood pressure in Hong Kong adolescents: a population-based study. Journal of Human Hypertension 2010;24(10):646-51. Cross-sectional study
Ref ID: 5609

(4565) Soares LD, Petroski EL. Prevalência, fatores etiológicos e tratamento da obesidade infantil. Revista Brasileira de Cineantropometria e Desempenho Humano 2003;5(1). Subjects less than 2 years old
Ref ID: 4573

(4566) Soares MJ, Shetty PS. Intra-individual variations in resting metabolic rates of human subjects. Human Nutrition - Clinical Nutrition 1986 September;40(5):365-9. Study limited to adults
Ref ID: 2346

(4567) Sobko T, Svensson V, Ek A, Ekstedt M, Karlsson H, Johansson E, Cao Y, Hagstromer M, Marcus C. A randomised controlled trial for overweight and obese parents to prevent childhood obesity--Early STOPP (STockholm Obesity Prevention Program). BMC Public Health 2011;11:336. Behavior Modification Intervention
Ref ID: 2800

(4568) Sobol-Goldberg S, Rabinowitz J, Gross R. School-based obesity prevention programs: a meta-analysis of randomized controlled trials. Obesity (Silver Spring) 2013 December;21(12):2422-8. Inappropriate Study Design
Ref ID: 6076

(4569) Soemantri AG, Hapsari DE, Susanto JC, Rohadi W, Tamam M, Irawan PW, Sugianto A. Daily and weekly iron supplementation and physical growth of school age Indonesian children. The Southeast Asian Journal of Tropical Medicine and Public Health 1997;28 Suppl 2:69-74. Diet Intervention or Supplement Study
Ref ID: 5013

(4570) Sofuoglu A, Kiymet N, Kavcar P, Sofuoglu SC. Polycyclic and nitro musks in indoor air: a primary school classroom and a women's sport center. Indoor Air

2010;20(6):515-22. Off topic
Ref ID: 5610

(4571) Sohanpal SK, Deb S, Thomas C, Soni R, Lenotre L, Unwin G. The effectiveness of antidepressant medication in the management of behaviour problems in adults with intellectual disabilities: a systematic review. Journal of Intellectual Disability Research 2007 October;51(10):750-65. Review article
Ref ID: 3858

(4572) Soler M, Matz J, Townley R, Buhl R, O'Brien J, Fox H, Thirlwell J, Gupta N, Della Cioppa G. The anti-IgE antibody omalizumab reduces exacerbations and steroid requirement in allergic asthmatics. European Respiratory Journal 2001;18(2):254-61. Review article
Ref ID: 3308

(4573) Solimani F. Developmental outcome of low-birth-weight premature infants. Iranian Journal of Pediatrics 2007;17:125-35. Subjects less than 2 years old
Ref ID: 5611

(4574) Sollerhed AC, Apitzsch E, Rastam L, Ejlertsson G. Factors associated with young children's self-perceived physical competence and self-reported physical activity. Health Education Research 2008 February;23(1):125-36. Survey or questionnaire
Ref ID: 1009

(4575) Solomons NW. All That Glitters Is Not Iron (Deficiency): Revisiting the Question of Why Anemic Individuals Are Anemic. Nutrition Reviews 2002 March;60(3):91. Off topic
Ref ID: 3746

(4576) Son SM, Park SH, Moon HK, Lee E, Ahn SH, Cho YW, Byun WM, Jang SH. Diffusion tensor tractography can predict hemiparesis in infants with high risk factors. Neuroscience Letters 2009;451(1):94-7. Off topic
Ref ID: 5612

(4577) Sondergaard SB, Verdich C, Astrup A, Bratholm P, Christensen NJ. Obese male subjects show increased resting forearm venous plasma noradrenaline concentration but decreased 24-hour sympathetic activity as evaluated by thrombocyte noradrenaline measurements. International Journal of Obesity and Related Metabolic Disorders 1999 August;23(8):810-5. Inappropriate Outcomes
Ref ID: 2001

(4578) Song YF, Lin J, Li YQ, He XY, Xu B, Hao L, Song J. [Analysis of risk factors about stress urinary incontinence in female]. [Chinese]. Chung-Hua Fu Chan Ko Tsa Chih [Chinese Journal of Obstetrics and Gynecology] 2003 December;38(12):737-40. Off topic
Ref ID: 1680

(4579) Sonksen PH, Cuneo RC, Salomon F, McGauley G, Wiles CM, Wilmshurst P, Byrne C, Hesp R, Lowy C, Weissberger A. Growth hormone therapy in adults with growth hormone deficiency. Acta Paediatrica Scandinavica - Supplement 1991;379:139-46. Drug intervention study
Ref ID: 2295

(4580) Sonnenborn U, Schulze JÃ. The non-pathogenic Escherichia coli strain Nissle 1917 â€“ features of a versatile probiotic. Microbial Ecology in Health and Disease 2009 September;21(3/4):122-58. Off topic
Ref ID: 3747

(4581) Sonneville KR, Gortmaker SL. Total energy intake, adolescent discretionary behaviors and the energy gap. International Journal of Obesity 2008 December;32:Suppl-27. Observational study
Ref ID: 839

(4582) Sorensen JG, Stoddard AM, Dubowitz T, Barbeau EM, Bigby J, Emmons KM, Berkman LF, Peterson KE. the influence of social context on changes in fruit and vegetable consumption: Results of the healthy directions studies. American Journal of Public Health 2007 July;97(7):1216-27. Diet Intervention Study
Ref ID: 3859

(4583) Sosa ES, Groeneveld IF, Gonzalez-Saiz L, Lopez-Mojares LM, Villa-Asensi JR, Gonzalez MI, Fleck SJ, Perez M, Lucia A. Intrahospital weight and aerobic training in children with cystic fibrosis: a randomized controlled trial. Medicine and Science in Sports and Exercise 2012 January;44(1):2-11. Not All Participants were Overweight and/or Obese
Ref ID: 2801

(4584) Sothern, Udall JN, Jr., Suskind RM, Vargas A, Blecker U. Weight loss and growth velocity in obese children after very low calorie diet, exercise, and behavior modification. Acta Paediatrica 2000 September;89(9):1036-43. Behavior Modification Intervention
Ref ID: 1950

(4585) Sothern MS, von Almen TK, Schumacher H, Zelman M, Farris RP, Carlisle L, Udall JN, Jr., Suskind RM. An effective multidisciplinary approach to weight reduction in youth. Annals of the New York Academy of Sciences 1993 October 29;699:292-4. Not a randomized controlled trial (RCT)
Ref ID: 2250

(4586) Sothern MS, von Almen TK, Schumacher HD, Suskind RM, Blecker U. A multidisciplinary approach to the treatment of childhood obesity. Delaware Medical Journal 1999 June;71(6):255-61. Behavior Modification Intervention
Ref ID: 2008

(4587) Soto I, Mericq G. Restricción del crecimiento fetal e insulinorresistencia: Nuevos hallazgos y revisión de la literatura. Revista médica de Chile 2005

January;133(1):97-104. Subjects less than 2 years old
Ref ID: 4574

(4588) Southard DR, Southard BH. Promoting physical activity in children with MetaKenkoh. Clinical and Investigative Medicine 2006 October;29(5):293-7. Not All Participants were Overweight and/or Obese
Ref ID: 258

(4589) Souza CdO, Silva RdCR. Fatores associados ao excesso de peso em crianças e adolescentes brasileiros: revisão. Factors associated with excess weight in brazilian children and adolescents: review. Nutrire Revista da Sociedade Brasileira de Alimentação e Nutrição 2009 December;34(3). Review article
Ref ID: 4575

(4590) Souza CdO, Silva RdCR, Assis AMO, Fiaccone RL, Pinto EdJ, Moraes LTLPd. Associação entre inatividade física e excesso de peso em adolescentes de Salvador, Bahia – Brasil. Association between physical inactivity and overweight among adolescents in Salvador, Bahia - Brazil. Revista Brasileira de Eidemiologia 2010 September;13(3):468-75. Cross-sectional study
Ref ID: 4576

(4591) Souza EAd, Barbosa Filho VC, Nogueira JAD, Azevedo Júnior MRd. Atividade física e alimentação saudável em escolares brasileiros: revisão de programas de intervenção. Physical activity and healthy eating in Brazilian students: a review of intervention programs. Cadernos de Saúde Pública 2011 August;27(8):1459-71. Review article
Ref ID: 4577

(4592) Souza M, Chaves R, Santos D, Fermino R, Garganta R, Seabra A, Maia J. Agregação familiar na adiposidade do tronco: um estudo em famílias nucleares portuguesas. Familial aggregation on trunkal fat: a study with Portuguese families. Revista Brasileira de Educação Física e Esporte 2011 March;25(1):153-61. Off topic
Ref ID: 4578

(4593) Sowunmi A, Falade CO, Oduola AM, Ogundahunsi OA, Fehintola FA, Gbotosho GO, Larcier P, Salako LA. Cardiac effects of halofantrine in children suffering from acute uncomplicated falciparum malaria. Transactions of the Royal Society of Tropical Medicine and Hygiene 1998;92:446-8. Drug intervention study
Ref ID: 916

(4594) Spaccarotella KJ, Andzel WD. The effects of low fat chocolate milk on postexercise recovery in collegiate athletes. Journal of Strength and Conditioning Research 2011 December;25(12):3456-60. Diet Intervention Study
Ref ID: 2802

(4595) Specker B, Binkley T, Fahrenwald N. Increased periosteal circumference remains present 12 months after an exercise intervention in preschool children. Bone 2004;35:1383-8. Off topic
Ref ID: 5015

(4596) Specker BL, Mulligan L, Ho M. Longitudinal study of calcium intake, physical activity, and bone mineral content in infants 6-18 months of age. Journal of Bone and Mineral Research 1999;14:569-76. Subjects less than 2 years old
Ref ID: 5016

(4597) Specker BL, Johannsen N, Binkley T, Finn K. Total body bone mineral content and tibial cortical bone measures in preschool children. J Bone Miner Res 2001 December;16(12):2298-305. No exercise only group, Observational study
Ref ID: 388

(4598) Specker BL. Influence of rapid growth on skeletal adaptation to exercise. [Review] [25 refs]. Journal of Musculoskeletal and Neuronal Interactions 2006 April;6(2):147-53. Review article
Ref ID: 1373

(4599) Spence AC, McNaughton SA, Lioret S, Hesketh KD, Crawford DA, Campbell KJ. A health promotion intervention can affect diet quality in early childhood. Journal of Nutrition 2013 October;143(10):1672-8. Inappropriate Population
Ref ID: 6078

(4600) Spencer MS, Rosland AM, Kieffer EC, Sinco BR, Valerio M, Palmisano G, Anderson M, Guzman JR, Heisler M. Effectiveness of a community health worker intervention among african american and Latino adults with type 2 diabetes: a randomized controlled trial. American Journal of Public Health 2011 December;101(12):2253-60. Study limited to adults
Ref ID: 3860

(4601) Spencer TJ, Abikoff HB, Connor DF, Biederman J, Pliszka SR, Boellner S, Read SC, Pratt R. Efficacy and safety of mixed amphetamine salts extended release (adderall XR) in the management of oppositional defiant disorder with or without comorbid attention-deficit/hyperactivity disorder in school-aged children and adolescents: A 4-week, multicenter, randomized, double-blind, parallel-group, placebo-controlled, forced-dose-escalation study. Clinical Therapeutics 2006;28:402-18. Drug intervention study
Ref ID: 5017

(4602) Speroni KG, Earley C, Atherton M. Evaluating the effectiveness of the Kids Living Fit program: a comparative study. Journal of School Nursing 2007;23:329-36. Diet & Exercise intervention
Ref ID: 5018

(4603) Speroni KG, Tea C, Earley C, Niehoff V, Atherton M. Evaluation of a pilot hospital-based community program implementing fitness and nutrition

education for overweight children. Journal for Specialists in Pediatric Nursing: JSPN 2008 July;13(3):144-53. Diet & Exercise intervention
Ref ID: 914

(4604) Spettigue W, Buchholz A, Henderson K, Feder S, Moher D, Kourad K, Gaboury I, Norris M, Ledoux S. Evaluation of the efficacy and safety of olanzapine as an adjunctive treatment for anorexia nervosa in adolescent females: a randomized, double-blind, placebo-controlled trial. BMC Pediatrics 2008;8:4. Drug intervention study
Ref ID: 5019

(4605) Spiegel SA, Foulk D. Reducing overweight through a multidisciplinary school-based intervention. Obesity (Silver Spring) 2006 January;14(1):88-96. No exercise only group
Ref ID: 286

(4606) Spijker AT, van Rossum EFC. Glucocorticoid receptor polymorphisms in major depression. Annals of the New York Academy of Sciences 2009 October 20;1179(1):199-215. Off topic
Ref ID: 3748

(4607) Spinola e Castro AM. Interventions for preventing obesity in children. Sao Paulo Medical Journal 2014;132(2):128-9. Inappropriate Study Design
Ref ID: 6079

(4608) Spitz B, Abramson J. When health policy is the problem: A report from the field. Journal of Health Politics, Policy and Law 2005 June;30(3):327-65. Off topic
Ref ID: 3749

(4609) Springer AE, Kelder SH, Byrd-Williams CE, Pasch KE, Ranjit N, Delk JE, Hoelscher DM. Promoting energy-balance behaviors among ethnically diverse adolescents: overview and baseline findings of The Central Texas CATCH Middle School Project. Health Education and Behavior 2013 October;40(5):559-70. Inappropriate Study Design
Ref ID: 6080

(4610) Springer C, Barstow TJ, Wasserman K, Cooper DM. Oxygen-Uptake and Heart-rate responses during hypoxic exercise in children and adults. Medicine and Science in Sports and Exercise 1991;23(1):71-9. Off topic
Ref ID: 5613

(4611) Spruijt-Metz D, Nguyen-Michel ST, Goran MI, Chou CP, Huang TT. Reducing sedentary behavior in minority girls via a theory-based, tailored classroom media intervention. International Journal of Pediatric Obesity 2008;3(4):240-8. Not All Participants were Overweight and/or Obese
Ref ID: 854

(4612) Spruijt-Metz D, Belcher B, Anderson D, Lane CJ, Chou CP, Salter-Venzon D, Davis JN, Hsu YW, Neuhouser ML, Richey JM, McKenzie TL, McClain A, Goran MI, Weigensberg MJ. A high-sugar/low-fiber meal compared with a low-sugar/high-fiber meal leads to higher leptin and physical activity levels in overweight Latina females. Journal of the American Dietetic Association 2009 June;109(6):1058-63. Not an exercise intervention study, Acute study
Ref ID: 115

(4613) Sreevatsava M, Narayan KM, Cunningham SA. Evidence for interventions to prevent and control obesity among children and adolescents: its applicability to India. Indian Journal of Pediatrics 2013 March;80 Suppl 1:S115-S122. Inappropriate Study Design
Ref ID: 6081

(4614) St George SM, Wilson DK, Lawman HG, Van Horn ML. Weight status as a moderator of the relationship between motivation, emotional social support, and physical activity in underserved adolescents. Journal of Pediatric Psychology 2013 May;38(4):387-97. Inappropriate Intervention
Ref ID: 6082

(4615) Stabelini Neto A, Mascarenhas LPG, Bozza R, Ulbrich AZ, Vasconcelos IQAd, Campos Wd. VO2máx e composição corporal durante a puberdade: comparação entre praticantes e não praticantes de treinamento sistematizado de futebol. Revista Brasileira de Cineantropometria e Desempenho Humano 2007 June;9(2). Cross-sectional study
Ref ID: 4579

(4616) Stabelini Neto A, Bozza R, Ulbrich AZ, Vasconcelos ÍQ, Mascarenhas LPG, Boguszewski MC, Campos Wd. Fatores de risco para aterosclerose associados à aptidão cardiorrespiratória e ao IMC em adolescentes. Arquivos Brasileiros de Endocrinologia and Metabologia 2008 August;52(6):1024-30. Cross-sectional study
Ref ID: 4580

(4617) Stabile M, Allin S. The economic costs of childhood disability. Future of Children 2012;22(1):65-96. Off topic
Ref ID: 5614

(4618) Staffileno BA, Minnick A, Coke LA, Hollenberg SM. Blood pressure responses to lifestyle physical activity among young, hypertension-prone African-American women. Journal of Cardiovascular Nursing 2007 March;22(2):107-17. Study limited to adults
Ref ID: 248

(4619) Staiano AE, Abraham AA, Calvert SL. Adolescent exergame play for weight loss and psychosocial improvement: a controlled physical activity intervention.

Obesity (Silver Spring) 2013 March;21(3):598-601. Inappropriate Outcomes
Ref ID: 6083

(4620) Stalvey MS, Anbar RD, Konstan MW, Jacobs JR, Bakker B, Lippe B, Geller DE. A multi-center controlled trial of growth hormone treatment in children with cystic fibrosis. Pediatric Pulmonology 2012 March;47(3):252-63. Drug intervention study
Ref ID: 2803

(4621) Stanforth PR, Gagnon J, Rice T, Bouchard C, Leon AS, Rao DC, Skinner JS, Wilmore JH. Reproducibility of resting blood pressure and heart rate measurements. The HERITAGE Family Study. Annals of Epidemiology 2000 July;10(5):271-7. Study limited to adults
Ref ID: 1964

(4622) Stanicka S, Vondra K, Pelikanova T, Vicek P, Hill M, Zamrazil V. Insulin sensitivity and counter-regulatory hormones in hypothyroidism and during thyroid hormone replacement therapy. Clinical Chemistry and Laboratory Medicine 2005 July;43(7):715-20. Drug intervention study
Ref ID: 3750

(4623) Staniford L, Breckon J, Copeland R. Treatment of childhood obesity: A systematic review. Journal of Child and Family Studies 2012 August;21(4):545-64. Review article
Ref ID: 3861

(4624) Stanley T, Misra M. Polycystic ovary syndrome in obese adolescents. Current Opinion in Endocrinology, Diabetes and Obesity 2008;15(1):30-6. Review article
Ref ID: 3309

(4625) Stanton R, Reaburn PR, Humphries B. The effect of short-term Swiss ball training on core stability and running economy. Journal of Strength and Conditioning Research 2004 August;18(3):522-8. CT
Ref ID: 1597

(4626) Stark LJ, Mulvihill MM, Powers SW, Jelalian E, Keating K, Creveling S, Byrnes-Collins B, Harwood I, Passero MA, Light M, Miller DL, Hovell MF. Behavioral intervention to improve calorie intake of children with cystic fibrosis: Treatment versus wait list control. Journal of Pediatric Gastroenterology and Nutrition 1996 April;22(3):240-53. Diet Intervention Study
Ref ID: 2178

(4627) Stark LJ, Janicke DM, McGrath AM, Mackner LM, Hommel KA, Lovell D. Prevention of Osteoporosis: A randomized clinical trial to increase calcium intake in children with juvenile rheumatoid arthritis. Journal of Pediatric Psychology 2005 July;30(5):377-86. Diet Intervention or Supplement Study
Ref ID: 3862

(4628) Stark TW, Tvoric B, Walker B, Noonan D, Sibla J. Ice hockey players using a weighted implement when training on the ice: a randomized control trial. Research Quarterly for Exercise and Sport 2009;80:54-61. Off topic
Ref ID: 5021

(4629) Starling RD, Toth MJ, Carpenter WH, Matthews DE, Poehlman ET. Energy requirements and physical activity in free-living older women and men: a doubly labeled water study. Journal of Applied Physiology 1998 September;85(3):1063-9. Study limited to adults
Ref ID: 2082

(4630) Steele RG, Wu YP, Cushing CC, Jensen CD. Evaluation of child health matters: a web-based tutorial to enhance school nurses' communications with families about weight-related health. Journal of School Nursing 2013 April;29(2):151-60. Inappropriate Study Design
Ref ID: 6084

(4631) Stefani GP, Montoni JD, Lopes RB, Bricks LF, Cunha AC. Tireoidite linfocítica crônica na adolescência: Relato de caso. Pediatria (São Paulo) 2007;29(1):59-64. Case-Control / Case Study
Ref ID: 4581

(4632) Stefanutti C, Lucani G, Vivenzio A, Di GS. Diet only and diet plus simvastatin in the treatment of heterozygous familial hypercholesterolemia in childhood. Drugs Under Experimental and Clinical Research 1999;25(1):23-8. Diet Intervention Study
Ref ID: 2026

(4633) Stein LL, Dong MH, Loomba R. Insulin sensitizers in nonalcoholic fatty liver disease and steatohepatitis: Current status. Advances in Therapy 2009;26(10):893-907. Off topic
Ref ID: 3310

(4634) Steinbeck K. Obesity: the science behind the management. Internal Medicine Journal 2002;32(5-6):237-41. Review article
Ref ID: 5615

(4635) Steinbeck K. Treatment options. Best Practice and Research Clinical Endocrinology and Metabolism 2005;19(3 SPEC. ISS.):455-69. Review article
Ref ID: 3311

(4636) Steinkamp G, Schmitt-Grohe S, Doring G, Staab D, Pfrunder D, Beck G, Schubert R, Zielen S. Once-weekly azithromycin in cystic fibrosis with chronic pseudomonas Aeruginosa infection. Respiratory Medicine 2008;102(11):1643-53. Off topic
Ref ID: 3312

(4637) Stella SG, Vilar AP, Lacroix C, Fisberg M, Santos RF, Mello MT, Tufik S. Effects of type of physical exercise and leisure activities on the depression scores of obese Brazilian adolescent girls. Brazilian Journal of Medical and Biological Research 2005 November;38(11):1683-9. Not a randomized controlled trial (RCT)
Ref ID: 677

(4638) Stella SG, Fernandez AC, VILAR AP, Lacroix C, Fisberg M, Melo MT, Tufik S. Estudo comparativo das capacidades aeróbica e anaeróbica de adolescentes com obesidade severa da cidade de Säo Paulo. Revista Brasileira de Ciência e Movimento 2003 January;11(1):23-8. Cross-sectional study
Ref ID: 4582

(4639) Stenlof K, Cefalu WT, Kim KA, Alba M, Usiskin K, Tong C, Canovatchel W, Meininger G. Efficacy and safety of canagliflozin monotherapy in subjects with type 2 diabetes mellitus inadequately controlled with diet and exercise. Diabetes, Obesity and Metabolism 2013 April;15(4):372-82. Inappropriate Intervention
Ref ID: 6085

(4640) Stephens BR, Cole AS, Mahon AD. The influence of biological maturation on fat and carbohydrate metabolism during exercise in males. International Journal of Sport Nutrition and Exercise Metabolism 2006 April;16(2):166-79. Off topic
Ref ID: 1383

(4641) Stephens RJ, Bassel C, Sandor P. Olanzapine in the treatment of aggression and tics in children with Tourette's syndrome--a pilot study. Journal of Child and Adolescent Psychopharmacology 2004;14:255-66. Off topic
Ref ID: 5022

(4642) Stephenson LS, Latham MC, Adams EJ, Kinoti SN, Pertet A. Physical fitness, growth and appetite of Kenyan school boys with hookworm, Trichuris trichiura and Ascaris lumbricoides infections are improved four months after a single dose of albendazole. Journal of Nutrition 1993;123:1036-46. Off topic
Ref ID: 5023

(4643) Stergioulas A, Tripolitsioti A, Messinis D, Bouloukos A, Nounopoulos C. The effects of endurance training on selected coronary risk factors in children. Acta Paediatrica 1998;87:401-4. Not a randomized controlled trial (RCT)
Ref ID: 5024

(4644) Stergioulas A, Baltopoulos P, Papadopoulou T. Acute physical exercise in children and the levels of prostacyclin and Thromboxane in urine. Acta Medica Bulgarica 2001;28:125-34. Acute study
Ref ID: 5025

(4645) Stergioulas AT, Filippou DK. Effects of physical conditioning on lipids and arachidonic acid metabolites in untrained boys: a longitudinal study. Applied

Physiology, Nutrition, and Metabolism = Physiologie Appliquee, Nutrition et Metabolisme 2006 August;31(4):432-41. Off topic
Ref ID: 1360

(4646) Stern AH. Reevaluation of the Reference Dose for Methylmercury and Assessment of Current Exposure Levels. Risk Analysis 1993;13(3):355-64. Off topic
Ref ID: 5616

(4647) Sternberg A, Muzumdar H, Dinkevich E, Quintos JB, Austin-Leon G, Owens T, Murphy C, Dapul G, Rao M. The Downstart Program: a hospital-based pediatric healthy lifestyle program for obese and morbidly obese minority youth. Pediatric Endocrinology Reviews 2006 December;3:Suppl-9. Behavior Modification Intervention
Ref ID: 1307

(4648) Sterner Y, Torn C, Lee HS, Larsson H, Winkler C, McLeod W, Lynch K, Simell O, Ziegler A, Schatz D, Hagopian W, Rewers M, She JX, Krischer JP, Akolkar B, Lernmark A. Country-specific birth weight and length in type 1 diabetes high-risk HLA genotypes in combination with prenatal characteristics. Journal of Perinatology 2011;31(12):764-9. Off topic
Ref ID: 5617

(4649) Sterpa A, Pappini A, Picciotti M, Sommariva D, Chiumello G. [Changes of the lipid and protein profile in the obese child in diet therapy (with and without added fiber)]. [Italian]. Pediatria Medica e Chirurgica 1985 May;7(3):419-22. Diet Intervention Study
Ref ID: 2354

(4650) Stevens DA, Kan VL, Judson MA, Morrison VA, Dummer S, Denning DW, Bennett JE, Walsh TJ, Patterson TF, Pankey GA. Practice guidelines for diseases caused by aspergillus. Clinical Infectious Diseases 2000 April 4;30(4):696. Off topic
Ref ID: 3751

(4651) Stevens J, Story M, Becenti A, French SA, Gittelsohn J, Going SB, Juhaeri, Levin S, Murray DM. Weight-related attitudes and behaviors in fourth grade American Indian children. Obesity Research 1999 January;7(1):34-42. Survey or questionnaire
Ref ID: 2049

(4652) Stevens J, Story M, Ring K, Murray DM, Cornell CE, Juhaeri, Gittelsohn J. The impact of the Pathways intervention on psychosocial variables related to diet and physical activity in American Indian schoolchildren. Preventive Medicine 2003 December;37(6 Pt 2):S70-S79. No exercise only group
Ref ID: 345

(4653) Stevenson DK, Verter J, Fanaroff AA, Oh W, Ehrenkranz RA, Shankaran S, Donovan EF, Wright LL, Lemons JA, Tyson JE, Korones SB, Bauer CR, Stoll BJ. Sex differences in outcomes of very low birthweight infants: the newborn male disadvantage. Archives of Disease in Childhood 2000;83(3):F182-F185. Subjects less than 2 years old
Ref ID: 5618

(4654) Stevenson EJ, Williams C, Mash LE, Phillips B, Nute ML. Influence of high-carbohydrate mixed meals with different glycemic indexes on substrate utilization during subsequent exercise in women. American Journal of Clinical Nutrition 2006 August;84(2):354-60. Study limited to adults
Ref ID: 1361

(4655) Stevenson EJ, Astbury NM, Simpson EJ, Taylor MA, Macdonald IA. Fat oxidation during exercise and satiety during recovery are increased following a low-glycemic index breakfast in sedentary women. Journal of Nutrition 2009 May;139(5):890-7. Study limited to adults
Ref ID: 768

(4656) Stewart L, Chapple J, Hughes AR, Poustie V, Reilly JJ. The use of behavioural change techniques in the treatment of paediatric obesity: qualitative evaluation of parental perspectives on treatment. Journal of Human Nutrition and Dietetics 2008;21:464-73. Study limited to adults
Ref ID: 5026

(4657) Steyn NP, Lambert EV, Tabana H. Nutrition interventions for the prevention of type 2 diabetes. Proceedings of the Nutrition Society 2009;68(1):55-70. Review article
Ref ID: 3313

(4658) Steyn NP, Lambert EV, Tabana H. Conference on "Multidisciplinary approaches to nutritional problems". Symposium on "Diabetes and health". Nutrition interventions for the prevention of type 2 diabetes. [Review] [73 refs]. Proceedings of the Nutrition Society 2009 February;68(1):55-70. Review article
Ref ID: 799

(4659) Stice E, Chase A, Stormer S, Appel A. A randomized trial of a dissonance-based eating disorder prevention program. International Journal of Eating Disorders 2001 April;29(3):247-62. Off topic
Ref ID: 1926

(4660) Stice E, Rohde P, Shaw H, Marti CN. Efficacy trial of a selective prevention program targeting both eating disorders and obesity among female college students: 1- and 2-year follow-up effects. Journal of Consulting and Clinical Psychology 2013 February;81(1):183-9. Inappropriate Population
Ref ID: 6086

(4661) Stieven Filho E, Sampaio EB, Namba M, Silva JLVd, Albano M, Rocha LEMd, Agulham MÂ, Cunha LAMd. É possível prever o comprimento de tendões flexores do joelho por antropometria? It is possible to predict the length of knee flexor tendons by anthropometry? Revista do Colégio Brasileiro de Cirurgiões 2010 August;37(4):274-8. Off topic
Ref ID: 4583

(4662) Stillman MJ, Kaniecki RG, Taylor FR. Abstracts and citations. Headache: The Journal of Head and Face Pain 49[2], 314-324. 2009. Abstract,
Ref ID: 3752

(4663) Stinton C, Elison S, Udwin O, Howlin P. Physical and mental health of adults with Williams syndrome. Journal of Intellectual Disability Research 2008 October;52(10):813. Off topic
Ref ID: 3863

(4664) Stob NR, Bell C, van Baak MA, Seals DR. Thermic effect of food and beta-adrenergic thermogenic responsiveness in habitually exercising and sedentary healthy adult humans. Journal of Applied Physiology 2007 August;103(2):616-22. Study limited to adults
Ref ID: 1191

(4665) Stock S, Miranda C, Evans S, Plessis S, Ridley J, Yeh S, Chanoine JP. Healthy Buddies: a novel, peer-led health promotion program for the prevention of obesity and eating disorders in children in elementary school. Pediatrics 2007 October;120(4):e1059-e1068. Prospective Study
Ref ID: 1160

(4666) Stockbrugger BA, Haennel RG. Validity and reliability of a medicine ball explosive power test. Journal of Strength and Conditioning Research 2001 November;15(4):431-8. Off topic
Ref ID: 1874

(4667) Stockmyer C, Kuester S, Ramsey D, Dietz WH. National Nutrition Summit, May 30, 2000: Results of the obesity discussion groups. Obesity Research 2001;9(4):41S-52S. Review article
Ref ID: 5619

(4668) Stockton MB, Lanctot JQ, McClanahan BS, Klesges LM, Klesges RC, Kumanyika S, Sherrill-Mittleman D. Self-perception and body image associations with body mass index among 8-10-year-old African American girls. Journal of Pediatric Psychology 2009 November;34(10):1144-54. Not an exercise intervention study
Ref ID: 126

(4669) Stokes KA, Gilbert KL, Hall GM, Andrews RC, Thompson D. Different responses of selected hormones to three types of exercise in young men. European Journal of Applied Physiology 2013 March;113(3):775-83.

Inappropriate Population
Ref ID: 6087

(4670) Stoltzfus RJ, Albonico M, Tielsch JM, Chwaya HM, Savioli L. School-based deworming program yields small improvement in growth of Zanzibari school children after one year. Journal of Nutrition 1997;127(11):2187-93. Off topic
Ref ID: 5620

(4671) Stone EJ, Osganian SK, McKinlay SM, Wu MC, Webber LS, Luepker RV, Perry CL, Parcel GS, Elder JP. Operational design and quality control in the CATCH multicenter Trial. Preventive Medicine 1996 July;25(4):384-99. Cohort Study
Ref ID: 2168

(4672) Stone EJ, Norman JE, Davis SM, Stewart D, Clay TE, Caballero B, Lohman TG, Murray DM. Design, implementation, and quality control in the Pathways American-Indian multicenter trial. Preventive Medicine 2003 December;37(6 Pt 2):S13-S23. No exercise only group
Ref ID: 349

(4673) Stone HH, Hooper CA, Kolb LD, Geheber CE, Dawkins EJ. Antibiotic prophylaxis in gastric, biliary and colonic surgery. Annals of Surgery 1976 October;184(4):443-52. Off topic
Ref ID: 2386

(4674) Stone HH, Morris ES, Geheber CE, Kolb LD, Dunlop WE. Clinical comparison of cefotaxime with gentamicin plus clindamycin in the treatment of peritonitis and other soft-tissue infections. Reviews of Infectious Diseases 1982 September;4:Suppl-43. Drug intervention study
Ref ID: 2368

(4675) Stone MH, Sanborn K, Smith LL, O'Bryant HS, Hoke T, Utter AC, Johnson RL, Boros R, Hruby J, Pierce KC, Stone ME, Garner B. Effects of in-season (5 weeks) creatine and pyruvate supplementation on anaerobic performance and body composition in American football players. International Journal of Sport Nutrition 1999 June;9(2):146-65. Diet Intervention or Supplement Study
Ref ID: 2023

(4676) Storen O, Helgerud J, Stoa EM, Hoff J. Maximal strength training improves running economy in distance runners. Medicine and Science in Sports and Exercise 2008 June;40(6):1087-92. Not All Participants were Overweight and/or Obese
Ref ID: 948

(4677) Storer TW, Magliano L, Woodhouse L, Lee ML, Dzekov C, Dzekov J, Casaburi R, Bhasin S. Testosterone dose-dependently increases maximal voluntary strength and leg power, but does not affect fatigability or specific tension. Journal of Clinical Endocrinology and Metabolism 2003 April;88(4):1478-85.

Study limited to adults
Ref ID: 1760

(4678) Storm DS, Boland MG, Gortmaker SL, He Y, Skurnick J, Howland L, Oleske JM. Protease inhibitor combination therapy, severity of illness, and quality of life among children with perinatally acquired HIV-1 infection. Pediatrics 2005;115(2):E173-E182. Off topic
Ref ID: 5621

(4679) Story M, Lytle LA, Birnbaum AS, Perry CL. Peer-led, school-based nutrition education for young adolescents: feasibility and process evaluation of the TEENS study. The Journal of School Health 2002;72:121-7. Diet Intervention Study
Ref ID: 5027

(4680) Story M, Sherwood NE, Himes JH, Davis M, Jacobs DR, Jr., Cartwright Y, Smyth M, Rochon J. An after-school obesity prevention program for African-American girls: the Minnesota GEMS pilot study. Ethnicity and Disease 2003;13(1 Suppl 1):S54-S64. No exercise only group
Ref ID: 366

(4681) Story M, Hannan PJ, Fulkerson JA, Rock BH, Smyth M, Arcan C, Himes JH. Bright Start: Description and main outcomes from a group-randomized obesity prevention trial in American Indian children. Obesity (Silver Spring) 2012 November;20(11):2241-9. Inappropriate Intervention
Ref ID: 6088

(4682) Stouch BC, Strayer D, Carter W. Cardiac toxicity in Chronic Fatigue Syndrome: Results from a randomized 40-week multicenter double-blind placebo control trial of rintatolimod. Journal of Applied Research 2010;10(3):80-7. Off topic
Ref ID: 3314

(4683) Stovitz SD, Pereira MA, Vazquez G, Lytle LA, Himes JH. The interaction of childhood height and childhood BMI in the prediction of young adult BMI. Obesity (Silver Spring) 2008 October;16(10):2336-41. Observational study
Ref ID: 160

(4684) Straker LM, Abbott RA, Piek JP, Pollock CM, Davies PS, Smith AJ. Rationale, design and methods for a randomised and controlled trial to investigate whether home access to electronic games decreases children's physical activity. BMC Public Health 2009;9:212. Description versus conduct of study
Ref ID: 5028

(4685) Straker LM, Campbell AC, Jensen LM, Metcalf DR, Smith AJ, Abbott RA, Pollock CM, Piek JP. Rationale, design and methods for a randomised and controlled trial of the impact of virtual reality games on motor competence, physical activity, and mental health in children with developmental coordination

disorder. BMC Public Health 2011;11:654. Description versus conduct of study
Ref ID: 2804

(4686) Stratton G, Ridgers ND, Fairclough SJ, Richardson DJ. Physical activity levels of normal-weight and overweight girls and boys during primary school recess. Obesity 2007 June;15(6):1513-9. Cross-sectional study
Ref ID: 1220

(4687) Stratton P, Tuomala RE, Abboud R, Rodriguez E, Rich K, Pitt J, Diaz C, Hammill H, Minkoff H. Obstetric and newborn outcomes in a cohort of HIV-infected pregnant women: A report of the women and infants transmission study. Journal of Acquired Immune Deficiency Syndromes and Human Retrovirology 1999;20(2):179-86. Off topic
Ref ID: 5622

(4688) Strauss RS, Rodzilsky D, Burack G, Colin M. Psychosocial correlates of physical activity in healthy children. Archives of Pediatrics and Adolescent Medicine 2001 August;155(8):897-902. Survey or questionnaire
Ref ID: 1902

(4689) Streeter SA, McBurney DH. Waist-hip ratio and attractiveness - New evidence and a critique of "a critical test". Evolution and Human Behavior 2003;24(2):88-98. Off topic
Ref ID: 5623

(4690) Streuling I, Beyerlein A, von KR. Can gestational weight gain be modified by increasing physical activity and diet counseling? A meta-analysis of interventional trials. American Journal of Clinical Nutrition 92(4):678-87, 2010 Oct 2010;(4):678-87. Review article
Ref ID: 3135

(4691) Stroescu V, Dragan J, Simionescu L, Stroescu OV. Hormonal and metabolic response in elite female gymnasts undergoing strenuous training and supplementation with SUPRO Brand Isolated Soy Protein. Journal of Sports Medicine and Physical Fitness 2001 March;41(1):89-94. Diet Intervention or Supplement Study
Ref ID: 1919

(4692) Strohmeyer S. The Biomechanical Implications of Obesity in K-12 Learners. Journal of Physical Education, Recreation and Dance (JOPERD) 2007 October 1;78(8):40-2. Review article
Ref ID: 3914

(4693) Stuart GR, Hopkins WG, Cook C, Cairns SP. Multiple Effects of Caffeine on Simulated High-Intensity Team-Sport Performance. Medicine and Science in Sports and Exercise 2005 November;37(11):1998-2005. Diet Intervention or Supplement Study
Ref ID: 3864

(4694) Sturnieks DL, Besier TF, Mills PM, Ackland TR, Maguire KF, Stachowiak GW, Podsiadlo P, Lloyd DG. Knee joint biomechanics following arthroscopic partial meniscectomy. Journal of Orthopaedic Research 2008 August;26(8):1075-80. Off topic
Ref ID: 915

(4695) Styne DM, Skaikh U. Childhood obesity - Is there hope for therapy and prevention? Pediatric Endocrinology Reviews 2009;6(3):372-4. Review article
Ref ID: 3315

(4696) Suarez CL, Moreno Villares JM, Martinez S, V, Aranceta BJ, Dalmau SJ, Gil HA, Lama MR, Martin Mateos MA, Pavon BP. [Calcium intake and bone mineral density in a group of Spanish school-children]. [Spanish]. Anales de Pediatria 2011 January;74(1):3-9. Cross-sectional study
Ref ID: 2805

(4697) Suarez EC. Relations of trait depression and anxiety to low lipid and lipoprotein concentrations in healthy young adult women. Psychosomatic Medicine 1999 May;61(3):273-9. Study limited to adults
Ref ID: 2021

(4698) Suarez EC. Plasma interleukin-6 is associated with psychological coronary risk factors: moderation by use of multivitamin supplements. Brain, Behavior, and Immunity 2003 August;17(4):296-303. Diet Intervention or Supplement Study
Ref ID: 1736

(4699) Suárez Castillo N, Guerrero Ramírez A, Rodríguez Oropesa KM, Flores Martínez C, Tadeo Oropesa I. Prevalencia de obesidad en un círculo infantile. Obesity prevalence in a nursery. Revista Cubana de Pediatria 2010 June;82(2). Diet Intervention Study
Ref ID: 4584

(4700) Subbotsky E. Understanding the distinction between sensations and physical properties of objects by children and adults. International Journal of Behavioral Development 1997;20(2):321-47. Off topic
Ref ID: 5624

(4701) Subramaniam GA, Ives ML, Stitzer ML, Dennis ML. The added risk of opioid problem use among treatment-seeking youth with marijuana and/or alcohol problem use. Addiction 2010;105(4):686-98. Off topic
Ref ID: 5625

(4702) Suchdev PS, Davis SM, Bartoces M, Ruth LJ, Worrell CM, Kanyi H, Odero K, Wiegand RE, Njenga SM, Montgomery JM, Fox LM. Soil-transmitted helminth infection and nutritional status among urban slum children in Kenya. American Journal of Tropical Medicine and Hygiene 2014 February;90(2):299-305. Inappropriate Study Design
Ref ID: 6089

(4703) Sudi KM, Gallistl S, Borkenstein MH, Payerl D, Aigner R, Moller R, Tafeit E. Effects of weight loss on leptin, sex hormones, and measures of adiposity in obese children. Endocrine 2001 April;14(3):429-35. Study less than 4 weeks
Ref ID: 1907

(4704) Sudi KM, Gallistl S, Trobinger M, Payerl D, Aigner R, Borkenstein MH. The effects of changes in body mass and subcutaneous fat on the improvement in metabolic risk factors in obese children after short-term weight loss. Metabolism: Clinical and Experimental 2001 November;50(11):1323-9. Study less than 4 weeks
Ref ID: 1882

(4705) Suessmuth S, Freihorst J, Gappa M. Low-dose theophylline in childhood asthma: A placebo-controlled, double-blind study. Pediatric Allergy and Immunology 2003;14(5):394-400. Drug intervention study
Ref ID: 3316

(4706) Sugawara N, Ohba T, Nakai K, Kakita A, Nakamura T, Suzuki K, Kameo S, Shimada M, Kurokawa N, Satoh C, Satoh H. Effects of perinatal coexposure to methylmercury and polychlorinated biphenyls on neurobehavioral development in mice. Archives of Toxicology 2008;82(6):387-97. Animal study
Ref ID: 5626

(4707) Sugimoto N, Ichikawa M, Siriliang B, Nakahara S, Jimba M, Wakai S. Herbal medicine use and quality of life among people living with HIV/AIDS in northeastern Thailand. AIDS Care 2005 February;17(2):252-62. Off topic
Ref ID: 3753

(4708) Sui Z, Moran LJ, Dodd JM. Physical activity levels during pregnancy and gestational weight gain among women who are overweight or obese. Health Promotion Journal of Australia 2013 December;24(3):206-13. Inappropriate Population
Ref ID: 6090

(4709) Suling M, Hebestreit A, Peplies J, Bammann K, Nappo A, Eiben G, Alvira JM, Verbestel V, Kovacs E, Pitsiladis YP, Veidebaum T, Hadjigeorgiou C, Knof K, Ahrens W, IDEFICS Consortium. Design and results of the pretest of the IDEFICS study. International Journal of Obesity 2011 April;35:Suppl-44. Survey or questionnaire
Ref ID: 2806

(4710) Sullivan PB, Juszczak E, Bachlet AM, Lambert B, Vernon-Roberts A, Grant HW, Eltumi M, McLean L, Alder N, Thomas AG. Gastrostomy tube feeding in children with cerebral palsy: A prospective, longitudinal study. Developmental Medicine and Child Neurology 2005 February;47(2):77-85. Off topic
Ref ID: 1544

(4711) Suman OE, Spies RJ, Celis MM, Mlcak RP, Herndon DN. Effects of a 12-wk resistance exercise program on skeletal muscle strength in children with burn injuries. Journal of Applied Physiology 2001 September;91(3):1168-75. Not All Participants were Overweight and/or Obese
Ref ID: 1899

(4712) Suman OE, Thomas SJ, Wilkins JP, Mlcak RP, Herndon DN. Effect of exogenous growth hormone and exercise on lean mass and muscle function in children with burns. Journal of Applied Physiology 2003 June;94(6):2273-81. Drug intervention study
Ref ID: 1746

(4713) Suman OE, Mlcak RP, Herndon DN. Effects of exogenous growth hormone on resting pulmonary function in children with thermal injury. The Journal of Burn Care and Rehabilitation 2004;25:287-93. Drug intervention study
Ref ID: 5029

(4714) Suman OE, Herndon DN. Effects of cessation of a structured and supervised exercise conditioning program on lean mass and muscle strength in severely burned children. Archives of Physical Medicine and Rehabilitation 2007 December;88(12:Suppl 2):Suppl-9. Not All Participants were Overweight and/or Obese
Ref ID: 1139

(4715) Suminski RR, Petosa R. Stages of change among ethnically diverse college students. Journal of American College Health 2002 July;51(1):26. Off topic
Ref ID: 3865

(4716) Summerbell CD, Ashton V, Campbell KJ, Edmunds L, Kelly S, Waters E. Interventions for treating obesity in children. [Review] [104 refs][Update in Cochrane Database of Systematic Reviews. 2009;(1):CD001872; PMID: 19160202]. Cochrane Database of Systematic Reviews (3):CD001872, 2003 2003;(3):CD001872. Review article
Ref ID: 1723

(4717) Summerbell CD, Waters E, Edmunds LD, Kelly S, Brown T, Campbell KJ. Interventions for preventing obesity in children. [Review] [116 refs][Update of Cochrane Database of Systematic Reviews. 2002;(2):CD001871; PMID: 12076426]. Cochrane Database of Systematic Reviews (3):CD001871, 2005 2005;(3):CD001871. Review article
Ref ID: 1494

(4718) Sun C, Pezic A, Tikellis G, Ponsonby AL, Wake M, Carlin JB, Cleland V, Dwyer T. Effects of school-based interventions for direct delivery of physical activity on fitness and cardiometabolic markers in children and adolescents: A systematic review of randomized controlled trials. Obesity Reviews 2013

October;14(10):818-38. Inappropriate Study Design
Ref ID: 6091

(4719) Sun G, Gagnon J, Chagnon YC, Perusse L, Despres JP, Leon AS, Wilmore JH, Skinner JS, Borecki I, Rao DC, Bouchard C. Association and linkage between an insulin-like growth factor-1 gene polymorphism and fat free mass in the HERITAGE Family Study. International Journal of Obesity and Related Metabolic Disorders 1999 September;23(9):929-35. Off topic
Ref ID: 1999

(4720) Sundaresan PR, Madan MK, Kelvie SL, Weintraub M. Platelet alpha-2 adrenoceptors and the menstrual cycle. Clinical Pharmacology Therapeutics 1985 March;37(3):337-42. Off topic
Ref ID: 2358

(4721) Sunder M. The empirical relationship between longevity and physical stature may be obscured by unobserved genetic diversity in the optimal metabolic rate. Medical Hypotheses 2005;64(6):1225-8. Off topic
Ref ID: 5627

(4722) Sundgot-Borgen J, Rosenvinge JH, Bahr R, Schneider LS. The effect of exercise, cognitive therapy, and nutritional counseling in treating bulimia nervosa. Medicine and Science in Sports and Exercise 2002 February;34(2):190-5. Study limited to adults
Ref ID: 1852

(4723) Sundquist J. Ethnicity, social class and health. A population-based study on the influence of social factors on self-reported illness in 223 Latin American refugees, 333 Finnish and 126 south European labour migrants and 841 Swedish controls. Social Science and Medicine 1995 March;40(6):777-87. Off topic
Ref ID: 2211

(4724) Sundram F, Murphy DG, Murphy KC. White matter microstructure in children with velocardiofacial syndrome: A diffusion tensor imaging and voxel based morphometry study. Journal of Intellectual Disability Research 2008 October;52(10):812. Off topic
Ref ID: 3866

(4725) Sunehag AL, Toffolo G, Treuth MS, Butte NF, Cobelli C, Bier DM, Haymond MW. Effects of dietary macronutrient content on glucose metabolism in children. Journal of Clinical Endocrinology and Metabolism 2002;87:5168-78. Diet Intervention Study
Ref ID: 5030

(4726) Sung-Chan P, Sung YW, Zhao X, Brownson RC. Family-based models for childhood-obesity intervention: a systematic review of randomized controlled

trials. Obesity Reviews 2013 April;14(4):265-78. Inappropriate Study Design
Ref ID: 6092

(4727) Sung RY, Yu CW, Chang SK, Mo SW, Woo KS, Lam CW. Effects of dietary intervention and strength training on blood lipid level in obese children. Archives of Disease in Childhood 2002 June;86(6):407-10. No exercise only group, No comparative control group
Ref ID: 379

(4728) Sunnegardh J, Bratteby LE, Hagman U, Samuelson G, Sjolin S. Physical activity in relation to energy intake and body fat in 8- and 13-year-old children in Sweden. Acta Paediatrica Scandinavica 1986 November;75(6):955-63. Longitudinal Study
Ref ID: 2344

(4729) Sunnegardh J, Bratteby LE. Maximal oxygen uptake, anthropometry and physical activity in a randomly selected sample of 8 and 13 year old children in Sweden. European Journal of Applied Physiology and Occupational Physiology 1987;56(3):266-72. Cross-sectional study
Ref ID: 2340

(4730) Suñé FR, Dias-da-Costa JS, Olinto MTA, Pattussi MP. Prevalência e fatores associados para sobrepeso e obesidade em escolares de uma cidade no Sul do Brasil. Cadernos de Saúde Pública 2007 June;23(6):1361-71. Cross-sectional study
Ref ID: 4585

(4731) Super CM, Herrera MG, Mora JO. Long-term effects of food supplementation and psychosocial intervention on the physical growth of Colombian infants at risk of malnutrition. Child development 1990;61:29-49. Off topic
Ref ID: 1077

(4732) Suri R, Metcalfe C, Lees B, Grieve R, Flather M, Normand C, Thompson S, Bush A, Wallis C. Comparison of hypertonic saline and alternate-day or daily recombinant human deoxyribonuclease in children with cystic fibrosis: A randomised trial. Lancet 2001;358:1316-21. Drug intervention study
Ref ID: 5031

(4733) Suri R, Metcalfe C, Wallis C, Bush A. Assessing the usefulness of outcomes measured in a cystic fibrosis treatment trial. Respiratory Medicine 2007;101:254-60. Drug intervention study
Ref ID: 5032

(4734) Surkan PJ, Gottlieb BR, McCormick MC, Hunt A, Peterson KE. Impact of a health promotion intervention on maternal depressive symptoms at 15 months postpartum. Maternal and Child Health Journal 2012 January;16(1):139-48. Study limited to adults
Ref ID: 1030

(4735) Suskind RM, Sothern MS, Farris RP, von Almen TK, Schumacher H, Carlisle L, Vargas A, Escobar O, Loftin M, Fuchs G. Recent advances in the treatment of childhood obesity. Annals of the New York Academy of Sciences 1993 October 29;699:181-99. Review article
Ref ID: 2251

(4736) Sverdloff HR. Obesidad: una epidemia del siglo XXI. Boletín de la Asociación Argentina de Odontología para Niños 2002 December;31(2/3):11-4. Review article
Ref ID: 4586

(4737) Swan PD, Howley ET. Substrate utilization during prolonged exercise in obese women differing in body fat distribution. International Journal of Obesity and Related Metabolic Disorders 1994 April;18(4):263-8. Study limited to adults
Ref ID: 433

(4738) Swanson JM, Elliott GR, Greenhill LL, Wigal T, Arnold LE, Vitiello B, Hechtman L, Epstein JN, Pelham WE, Abikoff HB, Newcorn JH, Molina BS, Hinshaw SP, Wells KC, Hoza B, Jensen PS, Gibbons RD, Hur K, Stehli A, Davies M, March JS, Conners CK, Caron M, Volkow ND. Effects of stimulant medication on growth rates across 3 years in the MTA follow-up. Journal of the American Academy of Child and Adolescent Psychiatry 2007;46:1015-27. Drug intervention study
Ref ID: 5033

(4739) Swearingen D, Pennick M, Shojaei A, Lyne A, Fiske K. A phase I, randomized, open-label, crossover study of the single-dose pharmacokinetic properties of guanfacine extended-release 1-, 2-, and 4-mg tablets in healthy adults. Clinical Therapeutics 2007;29:617-25. Drug intervention study
Ref ID: 5034

(4740) Swiedler SJ, Beck M, Bajbouj M, Giugliani R, Schwartz I, Harmatz P, Wraith JE, Roberts J, Ketteridge D, Hopwood JJ, Guffon N, Sa Miranda MC, Teles EL, Berger KI, Piscia-Nichols C. Threshold effect of urinary glycosaminoglycans and the walk test as indicators of disease progression in a survey of subjects with Mucopolysaccharidosis VI (Maroteaux-Lamy syndrome). American Journal of Medical Genetics Part A 2005 April 15;134A(2):144-50. Off topic
Ref ID: 1533

(4741) Swillen A, Berghs I, Schoeters A, Fryns JP, Hellinkcx W, Devriendt K. Parental perception of sleep behaviour and sleep disorders in children with VCFS and their siblings. Journal of Intellectual Disability Research 2008 October;52(10):815. Off topic
Ref ID: 3867

(4742) Swinburn B. Obesity prevention in children and adolescents. [Review] [73 refs]. Child and Adolescent Psychiatric Clinics of North America 2009

January;18(1):209-23. Review article
Ref ID: 821

(4743) Swinburn BA, Jolley D, Kremer PJ, Salbe AD, Ravussin E. Estimating the effects of energy imbalance on changes in body weight in children. American Journal of Clinical Nutrition 2006 April;83(4):859-63. Diet Intervention Study
Ref ID: 1399

(4744) Swinburn BA, Sacks G, Lo SK, Westerterp KR, Rush EC, Rosenbaum M, Luke A, Schoeller DA, DeLany JP, Butte NF, Ravussin E. Estimating the changes in energy flux that characterize the rise in obesity prevalence. American Journal of Clinical Nutrition 2009 June;89(6):1723-8. Diet Intervention Study
Ref ID: 747

(4745) Swinburn BA, Millar L, Utter J, Kremer P, Moodie M, Mavoa H, Snowdon W, McCabe MP, Malakellis M, de Court, Waqa G, Fotu KF, Roberts G, Scragg R. The Pacific Obesity Prevention in Communities project: project overview and methods. Obesity Reviews 2011 November;12:Suppl-11. Description versus conduct of study
Ref ID: 2809

(4746) Swoboda KJ, Scott CB, Crawford TO, Simard LR, Reyna SP, Krosschell KJ, Acsadi G, Elsheik B, Schroth MK, D'Anjou G, LaSalle B, Prior TW, Sorenson SL, Maczulski JA, Bromberg MB, Chan GM, Kissel JT. SMA CARNI-VAL Trial Part I: Double-blind, randomized, placebo-controlled trial of l-carnitine and valproic acid in spinal muscular atrophy. PLoS ONE 2010 August;5(8):1-13. Drug intervention study
Ref ID: 3754

(4747) Symon BG, Marley JE, Martin AJ, Norman ER. Effect of a consultation teaching behaviour modification on sleep performance in infants: a randomised controlled trial. The Medical Journal of Australia 2005;182:215-8. Subjects less than 2 years old
Ref ID: 5035

(4748) Szczepaniak-Chichel L, Tykarski A. [Treatment of arterial hypertension in pregnancy in relation to current guidelines of the Polish Society of Arterial Hypertension from 2011]. Ginekologia Polska 2012 October;83(10):778-83. Inappropriate Study Design
Ref ID: 6093

(4749) Szczypaczewska M, Nazar K, Kaciuba-Uscilko H. Glucose tolerance and insulin response to glucose load in body builders. International Journal of Sports Medicine 1989 February;10(1):34-7. Study limited to adults
Ref ID: 2324

(4750) Szendrodi J, Rose B, Schloot NC, Roden M. Update on the molecular fundamentals of nutrition. Diabetologe 2009;5(6):432-+. Diet Intervention Study
Ref ID: 5628

(4751) Szopa J, Jaworski J, Zychowska M. Genetic and environmental influences on somatic and functional development of children from Polish rural population. Biology of Sport 2001;18(3):209-24. Cross-sectional study
Ref ID: 5629

(4752) Szymanski DJ, Szymanski JM, Schade RL, Bradford TJ, McIntyre JS, DeRenne C, Madsen NH. The relation between anthropometric and physiological variables and bat velocity of high-school baseball players before and after 12 weeks of training.[Erratum appears in Journal of Strength and Conditioning Research. 2011 Jan;25(1):286]. Journal of Strength and Conditioning Research 2010 November;24(11):2933-43. Not All Participants were Overweight and/or Obese
Ref ID: 2811

(4753) Tabak RG, Jones E, Jacobs JA, Dobbs T, Sutton V, Dove C, Brownson RC. Policy perceptions related to physical activity and healthy eating in Mississippi. Journal of Public Health Management and Practice 2013 May;19(3 Suppl 1):S97-S104. Inappropriate Study Design
Ref ID: 6094

(4754) Taber DR, Stevens J, Murray DM, Elder JP, Webber LS, Jobe JB, Lytle LA. The effect of a physical activity intervention on bias in self-reported activity. Annals of Epidemiology 2009 May;19(5):316-22. Cross-sectional study
Ref ID: 771

(4755) Tadjalli-mehr K, Becker N, Rahu M, Stengrevics A, Kurtinaitis J, Hakama M. Randomized trial with fruits and vegetables in prevention of cancer. Acta Oncologica 2003 June;42(4):287. Diet Intervention Study
Ref ID: 3755

(4756) Taha D. Hyperlipidemia in children with type 2 diabetes mellitus. [Review] [17 refs]. Journal of Pediatric Endocrinology 2002 April;15:Suppl-7. Review article
Ref ID: 1826

(4757) Tak YR, An JY, Kim YA, Woo HY. [The effects of a physical activity-behavior modification combined intervention(PABM-intervention) on metabolic risk factors in overweight and obese elementary school children]. [Korean]. Daehan Ganho Haghoeji 2007 October;37(6):902-13. Behavior Modification Intervention
Ref ID: 1143

(4758) Takahashi S, Imai N, Nabae K, Wake K, Kawai H, Wang JQ, Watanabe S, Kawabe M, Fujiwara O, Ogawa K, Tamano S, Shirai T. Lack of adverse effects of whole-body exposure to a mobile telecommunication electromagnetic field on

the rat fetus. Radiation Research 2010;173(3):362-72. Animal study
Ref ID: 5630

(4759) Takahashi Y, Fukusato T. Pediatric nonalcoholic fatty liver disease: overview with emphasis on histology. World Journal of Gastroenterology 2010 November 14;16(42):5280-5. Off topic
Ref ID: 1109

(4760) Takeda A, Nakamura Y, Aoki Y. Enzyme-linked-immunosorbent-assay for the detection of cathepsin-kininogen complexes in human plasma. Journal of Immunological Methods 1992;147(2):217-23. Off topic
Ref ID: 5631

(4761) Takei K, Dale S, Charles H, Sasaki A, Nakajima S. Absorption and excretion of colestilan in healthy subjects. Clinical Pharmacokinetics 2010 January;49(1):47-52. Drug intervention study
Ref ID: 3756

(4762) Takeuchi H, Kasai M, Taguchi N, Tsuji H, Suzuki M. Effect of triacylglycerols containing medium- and long-chain fatty acids on serum triacylglycerol levels and body fat in college athletes. Journal of Nutritional Science and Vitaminology (Tokyo) 2002 April;48(2):109-14. Study not limited to children and adolescents
Ref ID: 374

(4763) Takeuchi T, Tanaka D, Saikawa N, Satoh H, Iwasaki J, Inoue M, Narui K, Iikura Y. Growth and endocrine function during school age in very low-birth weight infants. Pediatrics International 2001;43(2):128-33. Follow-up Study
Ref ID: 5632

(4764) Takito MY, Benício MHD, Latorre MdRDdO. Postura materna durante a gestação e sua influência sobre o peso ao nascer. Revista de Saúde Pública 2005 June;39(3):325-32. Cohort Study
Ref ID: 681

(4765) Tamam S, Bellissimo N, Patel BP, Thomas SG, Anderson GH. Overweight and obese boys reduce food intake in response to a glucose drink but fail to increase intake in response to exercise of short duration. Applied Physiology, Nutrition, and Metabolism = Physiologie Appliquee, Nutrition et Metabolisme 2012 June;37(3):520-9. Diet Intervention or Supplement Study
Ref ID: 2813

(4766) Tamers SL, Okechukwu C, Allen J, Yang M, Stoddard A, Tucker-Seeley R, Sorensen G. Are social relationships a healthy influence on obesogenic behaviors among racially/ethnically diverse and socio-economically disadvantaged residents? Preventive Medicine 2013 January;56(1):70-4. Inappropriate Study Design
Ref ID: 6095

(4767) Tan-Ting AM, Llido L. Outcome of a hospital based multidisciplinary weight loss program in obese Filipino children. Nutrition 2011 January;27(1):50-4. Behavior Modification Intervention
Ref ID: 2814

(4768) Tan B, Aziz AR, Spurway NC, Toh C, Mackie H, Xie W, Wong J, Fuss FK, Teh KC. Indicators of maximal hiking performance in Laser sailors. European Journal of Applied Physiology 2006 September;98(2):169-76. Off topic
Ref ID: 1351

(4769) Tan D, Zwar NA, Dennis SM, Vagholkar S. Weight management in general practice: what do patients want? Medical Journal of Australia 2006 July 17;185(2):73-5. Survey or questionnaire
Ref ID: 1374

(4770) Tan KL, He JF, Qu YT, Xie MZ, Lei XM, Dai FY. [Observation on therapeutic effect of moxibustion and exercise for children with short stature of deficience of the kidney essence]. [Chinese]. Zhongguo Zhenjiu 2009 August;29(8):613-5. Diet Intervention or Supplement Study
Ref ID: 637

(4771) Tan ZA, Tong ML, Huang P, Deng JY, Hu YF, Zhang JZ. Effects of early interventions to behavior development in infancy among premature and infants with low body mass at birth. Zhongguo Linchuang Kangfu 2004;8:2992-3. Subjects less than 2 years old
Ref ID: 5037

(4772) Tanaka C, Sakuma H. Human figure drawing size and body image in preschool children from a self-physique perspective. Perceptual and Motor Skills 2004;99(2):691-700. Off topic
Ref ID: 5633

(4773) Tanchoco CC, Cruz AJ, Rogaccion JM, Casem RS, Rodriguez MP, Orense CL, Hermosura LC. Diet supplemented with MCT oil in the management of childhood diarrhea. Asia Pacific Journal of Clinical Nutrition 2007;16:286-92. Off topic
Ref ID: 5038

(4774) Taras H. Nutrition and Student Performance at School. Journal of School Health 2005 August;75(6):199-213. Review article
Ref ID: 3868

(4775) Tarro L, Llaurado E, Albaladejo R, Morina D, Arija V, Sola R, Giralt M. A primary-school-based study to reduce the prevalence of childhood obesity--the EdAl (Educacio en Alimentacio) study: a randomized controlled trial. Trials 2014;15:58. Inappropriate Intervention
Ref ID: 6096

(4776) Tassitano RM, Bezerra J, Tenório MCM, Colares V, Barros MVGd, Hallal PC. Atividade física em adolescentes brasileiros: uma revisão sistemática. Revista Brasileira de Cineantropometria e Desempenho Humano 2007 March;9(1):55-60. Review article
Ref ID: 4587

(4777) Tassitano RM, Barros MVGd, Tenório MCM, Bezerra J, Hallal PC. Prevalência e fatores associados ao sobrepeso e à obesidade em adolescentes, estudantes de escolas de Ensino Médio de Pernambuco, Brasil. Prevalence of overweight and obesity and associated factors among public high school students in Pernambuco State, Brazil. Cadernos de Saúde Pública 2009 December;25(12):2639-52. Survey or questionnaire
Ref ID: 4588

(4778) Tassone F, Hagerman PJ, Hagerman RJ. Newborn screening in Fragile X syndrome. Journal of Intellectual Disability Research 2008 October;52(10):814. Off topic
Ref ID: 3869

(4779) Tataranni PA, Larson DE, Snitker S, Ravussin E. Thermic effect of food in humans: methods and results from use of a respiratory chamber. American Journal of Clinical Nutrition 1995 May;61(5):1013-9. Diet Intervention Study
Ref ID: 2213

(4780) Tavares JdS, Melo ASdO, Amorim MMRd, Benício MHD, Takito MY, Cardoso MAA. Associação entre o padrão de atividade física materna, ganho ponderal gestacional e peso ao nascer em uma coorte de 118 gestantes no município de Campina Grande, Nordeste do Brasil. Association between maternal physical activity, gestational weight gain and birth weight in a cohort of 118 pregnant women in Campina Grande, Northeast of Brazil. Revista da Associação Médica Brasileira (1992) 2009;55(3):335-41. Cohort Study
Ref ID: 4589

(4781) Taveras EM, Marshall R, Horan CM, Gillman MW, Hacker K, Kleinman KP, Koziol R, Price S, Simon SR. Rationale and design of the STAR randomized controlled trial to accelerate adoption of childhood obesity comparative effectiveness research. Contemporary Clinical Trials 2013 January;34(1):101-8. Inappropriate Study Design
Ref ID: 6097

(4782) Taveras EM, Marshall R, Horan CM, Gillman MW, Hacker K, Kleinman KP, Koziol R, Price S, Rifas-Shiman SL, Simon SR. Improving children's obesity-related health care quality: process outcomes of a cluster-randomized controlled trial. Obesity (Silver Spring) 2014 January;22(1):27-31. Inappropriate Intervention
Ref ID: 6098

(4783) Tavichakorntrakool R, Sriboonlue P, Prasongwattana V, Puapairoj A, Yenchitsomanus PT, Sinchaikul S, Chen ST, Wongkham C, Thongboonkerd V. Metabolic enzymes, antioxidants, and cytoskeletal proteins are significantly altered in vastus lateralis muscle of k-depleted cadaveric subjects. Journal of Proteome Research 2009;8(5):2586-93. Off topic
Ref ID: 5634

(4784) Tawfeek HI, Najim NH, Al MS. Efficacy of an infant formula containing anti-Escherichia coli colostral antibodies from hyperimmunized cows in preventing diarrhea in infants and children: a field trial. International journal of infectious diseases : International Journal of Infectious Diseases 2003;7:120-8. Off topic
Ref ID: 5039

(4785) Taylor AW, McDonell E, Brassard L. The effects of an arm ergometer training programme on wheelchair subjects. Paraplegia 1986 April;24(2):105-14. Not All Participants were Overweight and/or Obese
Ref ID: 2351

(4786) Taylor AW. Application of science and medicine to sport. 1975. Springfield, Illinois. Charles C. Thomas, Publisher. Off topic
Ref ID: 3915

(4787) Taylor BJ, Heath AL, Galland BC, Gray AR, Lawrence JA, Sayers RM, Dale K, Coppell KJ, Taylor RW. Prevention of Overweight in Infancy (POI.nz) study: a randomised controlled trial of sleep, food and activity interventions for preventing overweight from birth. BMC Public Health 2011;11:942. Subjects less than 2 years old
Ref ID: 2816

(4788) Taylor NJ, Sahota P, Sargent J, Barber S, Loach J, Louch G, Wright J. Using intervention mapping to develop a culturally appropriate intervention to prevent childhood obesity: the HAPPY (Healthy and Active Parenting Programme for Early Years) study. International Journal of Behavioral Nutrition and Physical Activity 2013;10:142. Inappropriate Intervention
Ref ID: 6099

(4789) Taylor P, Worrell C, Steinberg SM, Hazra R, Jankelevich S, Wood LV, Zwerski S, Yarchoan R, Zeichner S. Natural history of lipid abnormalities and fat redistribution among human immunodeficiency virus-infected children receiving long-term, protease inhibitor-containing, highly active antiretroviral therapy regimens. Pediatrics 2004;114(2):235-42. Off topic
Ref ID: 5635

(4790) Taylor RW, McAuley KA, Barbezat W, Strong A, Williams SM, Mann JI. APPLE Project: 2-y findings of a community-based obesity prevention program in primary school age children. American Journal of Clinical Nutrition 2007

September;86(3):735-42. Diet & Exercise intervention
Ref ID: 1169

(4791) Taylor RW, Brown D, Dawson AM, Haszard J, Cox A, Rose EA, Taylor BJ, Meredith-Jones K, Treacy L, Ross J, William SM. Motivational interviewing for screening and feedback and encouraging lifestyle changes to reduce relative weight in 4-8 year old children: design of the MInT study. BMC Public Health 2010;10:271. Description versus conduct of study
Ref ID: 2817

(4792) Taylor WC, Baranowski T, Klesges LM, Ey S, Pratt C, Rochon J, Zhou A. Psychometric properties of optimism and pessimism: results from the Girls' Health Enrichment Multisite Studies. Preventive Medicine 2004 May;38 Suppl:S69-77.:S69-S77. No exercise only group
Ref ID: 335

(4793) Tondevold E. Danish Orthopedic Society. Acta Orthopaedica Scandinavica 70, 1. 4-2-1999. Off topic,
Ref ID: 541

(4794) Teixeira CS, Pereira ÉF. Aptidão física, idade e estado nutricional em militares. Physical fitness, age and nutritional status of military personnel. Arquivos Brasileiros de Cardiologia 2010 April;94(4):438-43. Study limited to adults
Ref ID: 4590

(4795) Tejada Villar P, Cortez Marino M, Munayco Magallanes A. Enfermedad periodontal como factor de riesgo para enfermedades obstructivas crónicas (EPOC). Kiru 2005 December;2(2):66-73. Off topic
Ref ID: 4591

(4796) Tejani A, Alexander S, Ettenger R, Lerner G, Zimmerman J, Kohaut E, Briscoe DM. Safety and pharmacokinetics of ascending single doses of sirolimus (Rapamune, rapamycin) in pediatric patients with stable chronic renal failure undergoing dialysis. Pediatric Transplantation 2004;8:151-60. Drug intervention study
Ref ID: 5040

(4797) Tek C, Guloksuz S, Srihari VH, Reutenauer EL. Investigating the safety and efficacy of naltrexone for anti-psychotic induced weight gain in severe mental illness: study protocol of a double-blind, randomized, placebo-controlled trial. BMC Psychiatry 2013;13:176. Inappropriate Intervention
Ref ID: 6100

(4798) Telford RD, Catchpole EA, Deakin V, Hahn AG, Plank AW. The effect of 7 to 8 months of vitamin/mineral supplementation on athletic performance. International Journal of Sport Nutrition 1992 June;2(2):135-53. Diet Intervention or Supplement Study
Ref ID: 2277

(4799) Telford RD, Cunningham RB, Fitzgerald R, Olive LS, Prosser L, Jiang X, Telford RM. Physical education, obesity, and academic achievement: a 2-year longitudinal investigation of Australian elementary school children. American Journal of Public Health 2012 February;102(2):368-74. Longitudinal Study
Ref ID: 2818

(4800) Telford RD, Cunningham RB, Waring P, Telford RM, Olive LS, Abhayaratna WP. Physical education and blood lipid concentrations in children: the LOOK randomized cluster trial. PLoS ONE 2013;8(10):e76124. Inappropriate Population
Ref ID: 6101

(4801) Telles S, Naveen VK, Balkrishna A, Kumar S. Short term health impact of a yoga and diet change program on obesity. Medical Science Monitor 2010 January;16(1):CR35-CR40. Diet & Exercise intervention
Ref ID: 607

(4802) Temple JL, Johnson K, Recupero K, Suders H. Nutrition labels decrease energy intake in adults consuming lunch in the laboratory. Journal of the American Dietetic Association 2010 July;110(7):1094-7. Study limited to adults, Not an exercise intervention study
Ref ID: 30

(4803) Tenório MCM, Barros MVGd, Tassitano RM, Bezerra J, Hallal PC. Atividade física e comportamento sedentário em adolescentes estudantes do ensino médio. Physical activity and sedentary behavior among adolescent high school students. Revista Brasileira de Eidemiologia 2010 March;13(1):105-17. Cross-sectional study
Ref ID: 4592

(4804) Terasawa E, Kurian JR, Keen KL, Shiel NA, Colman RJ, Capuano SV. Body Weight impact on puberty: effects of high-calorie diet on puberty onset in female rhesus monkeys. Endocrinology 2012;153(4):1696-705. Animal study
Ref ID: 5636

(4805) Terbrack HG, Gurtler KH, Huls G, BittnerDersch P, Klor HU, Lindemann H. Human fecal pancreatic elastase in children. Monatsschrift Kinderheilkunde 1996;144(9):901-5. Off topic
Ref ID: 5637

(4806) Terzi M, Kocamanoglu B, Guz H, Onar M. The eating attitudes in multiple sclerosis patients. Journal of Neurological Sciences 2009 September;26(3):311-7. Off topic
Ref ID: 3757

(4807) Tessmer CS, Silva MC, Pinho MN, Gazalle FK, Fassa AG. Insatisfação corporal em freqüentadores de academia. Revista Brasileira de Ciência e

Movimento 2006;14(1):7-12. Cross-sectional study
Ref ID: 4593

(4808) Tetens I, Hels O, Khan NI, Thilsted SH, Hassan N. Rice-based diets in rural Bangladesh: how do different age and sex groups adapt to seasonal changes in energy intake? American Journal of Clinical Nutrition 2003;78(3):406-13. Diet Intervention Study
Ref ID: 5638

(4809) Tetens I, Alinia S. The role of fruit consumption in the prevention of obesity. Journal of Horticultural Science and Biotechnology 2009;47-51. Diet Intervention Study
Ref ID: 5639

(4810) Thakor HG, Kumar P, Desai VK. Effect of physical and mental activity on blood pressure. Indian Journal of Pediatrics 2004 April;71(4):307-12. Cross-sectional study
Ref ID: 1639

(4811) Thayyil S, Cleary JO, Sebire NJ, Scott RJ, Chong K, Gunny R, Owens CM, Olsen OE, Offiah AC, Parks HG, Chitty LS, Price AN, Yousry TA, Robertson NJ, Lythgoe MF, Taylor AM. Post-mortem examination of human fetuses: a comparison of whole-body high-field MRI at 9.4 T with conventional MRI and invasive autopsy. Lancet 2009;374(9688):467-75. Off topic
Ref ID: 5640

(4812) Theim KR, Sinton MM, Goldschmidt AB, Van Buren DJ, Doyle AC, Saelens BE, Stein RI, Epstein LH, Wilfley DE. Adherence to behavioral targets and treatment attendance during a pediatric weight control trial. Obesity (Silver Spring) 2013 February;21(2):394-7. Inappropriate Intervention
Ref ID: 6102

(4813) Thein-Nissenbaum JM, Rauh MJ, Carr KE, Loud KJ, McGuine TA. Menstrual irregularity and musculoskeletal injury in female high school athletes. Journal of Athletic Training 2012 January;47(1):74-82. Off topic
Ref ID: 3870

(4814) Theodorou AS, Havenetidis K, Zanker CL, O'Hara JP, King RF, Hood C, Paradisis G, Cooke CB. Effects of acute creatine loading with or without carbohydrate on repeated bouts of maximal swimming in high-performance swimmers. Journal of Strength and Conditioning Research 2005 May;19(2):265-9. Diet Intervention or Supplement Study
Ref ID: 1514

(4815) Theron JJ, Oosthuizen JM, Rautenbach MM. Effect of physical exercise on plasma melatonin levels in normal volunteers. South African Medical Journal Suid-Afrikaanse Tydskrif Vir Geneeskunde 1984 December 1;66(22):838-41.

Acute study
Ref ID: 2360

(4816) Théodore F, Bonvecchio A, Blanco I, Irizarry L, Nava A, Carriedo A. Significados culturalmente construidos para el consumo de bebidas azucaradas entre escolares de la Ciudad de México. Culturally constructed meanings for consumption of sweetened beverages among schoolchildren in Mexico City. Revista Panamericana de Salud Pública 2011 October;30(4):327-34. Diet Intervention Study
Ref ID: 4594

(4817) Thien V, Thomas A, Markin D, Birmingham CL. Pilot study of a graded exercise program for the treatment of anorexia nervosa. International Journal of Eating Disorders 2000 July;28(1):101-6. Not All Participants were Overweight and/or Obese
Ref ID: 1981

(4818) Thivel D, Isacco L, Lazaar N, Aucouturier J, Ratel S, Dore E, Meyer M, Duche P. Effect of a 6-month school-based physical activity program on body composition and physical fitness in lean and obese schoolchildren. European Journal of Pediatrics 2011 November;170(11):1435-43. Not All Participants were Overweight and/or Obese
Ref ID: 2819

(4819) Thivel D, Isacco L, Rousset S, Boirie Y, Morio B, Duche P. Intensive exercise: a remedy for childhood obesity? Physiology and Behavior 2011 February 1;102(2):132-6. Acute study
Ref ID: 2820

(4820) Thivel D, Isacco L, Montaurier C, Boirie Y, Duche P, Morio B. The 24-h energy intake of obese adolescents is spontaneously reduced after intensive exercise: a randomized controlled trial in calorimetric chambers. PLoS ONE [Electronic Resource] 2012;7(1):e29840. Diet Intervention Study
Ref ID: 2821

(4821) Thivel D, Metz L, Aucouturier J, Brakoniecki K, Duche P, Morio B. The effects of imposed sedentary behavior and exercise on energy intake in adolescents with obesity. Journal of Developmental and Behavioral Pediatrics 2013 October;34(8):616-22. Inappropriate Outcomes
Ref ID: 6103

(4822) Thivel D, Metz L, Julien A, Morio B, Duche P. Obese but not lean adolescents spontaneously decrease energy intake after intensive exercise. Physiology and Behavior 2014 January 17;123:41-6. Inappropriate Study Design
Ref ID: 6104

(4823) Thomas-Dobersen DA, Butler-Simon N, Fleshner M. Evaluation of a weight management intervention program in adolescents with insulin-dependent

diabetes mellitus. Journal of the American Dietetic Association 1993 May;93(5):535-40. Behavior Modification Intervention
Ref ID: 2260

(4824) Thomas CM, Pierzga JM, Kenney WL. Aerobic training and cutaneous vasodilation in young and older men. Journal of Applied Physiology 1999 May;86(5):1676-86. Study limited to adults
Ref ID: 2028

(4825) Thomas D, Vydelingum V, Lawrence J. E-mail contact as an effective strategy in the maintenance of weight loss in adults. Journal of Human Nutrition and Dietetics 2011 February;24(1):32-8. Not an exercise intervention study, No exercise only group
Ref ID: 7

(4826) Thomas DR. Anorexia: Aetiology, Epidemiology and management in older people. Drugs and Aging 2009 July;26(7):557-70. Off topic
Ref ID: 3758

(4827) Thomas H. Obesity prevention programs for children and youth: why are their results so modest?. [Review] [53 refs]. Health Education Research 2006 December;21(6):783-95. Review article
Ref ID: 1323

(4828) Thomas JV, Mezzasalma DFC, Teixeira AM, Campos LNR, Luescher JL, Beserra ICR, Guimarães MM. Deficiência de hormônio do crescimento, hipotireoidismo e cromossomo 18 em anel: relato de caso. Growth hormone deficiency, hypothyroidism and ring chromosome 18: case report. Arquivos Brasileiros de Endocrinologia y Metabologia 2006 October;50(5):951-6. Case-Control / Case Study
Ref ID: 4595

(4829) Thomas K, Morris P, Stevenson E. Improved endurance capacity following chocolate milk consumption compared with 2 commercially available sport drinks. Applied Physiology, Nutrition, and Metabolism = Physiologie Appliquee, Nutrition et Metabolisme 2009 February;34(1):78-82. Diet Intervention or Supplement Study
Ref ID: 794

(4830) Thomas NE, Cooper SM, Williams SP, Baker JS, Davies B. Relationship of fitness, fatness, and coronary-heart-disease risk factors in 12- to 13-year-olds. Pediatric Exercise Science 2007 February;19(1):93-101. Cross-sectional study
Ref ID: 1221

(4831) Thomas NE, Baker JS, Graham MR, Cooper SM, Davies B. C-reactive protein in schoolchildren and its relation to adiposity, physical activity, aerobic fitness and habitual diet. British Journal of Sports Medicine 2008 May;42(5):357-60.

Cross-sectional study
Ref ID: 961

(4832) Thomas SS, Buckon CE, Schwartz MH, Russman BS, Sussman MD, Aiona MD. Variability and minimum detectable change for walking energy efficiency variables in children with cerebral palsy. Developmental Medicine and Child Neurology 2009 August;51(8):615-21. Off topic
Ref ID: 708

(4833) Thomas TR, Adeniran SB, Etheridge GL. Effects of different running programs on VO2 max, percent fat, and plasma lipids. Canadian Journal of Applied Sport Sciences - Journal Canadien des Sciences Appliquees au Sport 1984 June;9(2):55-62. Study limited to adults
Ref ID: 2363

(4834) Thomley BS, Ray SH, Cha SS, Bauer BA. Effects of a brief, comprehensive, yoga-based program on quality of life and biometric measures in an employee population: a pilot study. Explore: The Journal of Science and Healing 2011 January;7(1):27-9. Not a randomized controlled trial (RCT)
Ref ID: 2823

(4835) Thompson A, Damyanovich A, Madapallimattam A, Mikalus D, Allard J, Jeejeebhoy KN. P-31-nuclear magnetic resonance studies of bioenergetic changes in skeletal muscle in malnourished human adults. American Journal of Clinical Nutrition 1998;67(1):39-43. Off topic
Ref ID: 5641

(4836) Thompson D, Jago R, Baranowski T, Watson K, Zakeri I, Cullen KW, Story M, Sherwood NE, Pruitt LA, Matheson DM. Covariability in diet and physical activity in African-American girls. Obesity Research 2004 September;12 Suppl:46S-54S.:46S-54S. Not an exercise intervention study, Observational study
Ref ID: 324

(4837) Thompson D, Baranowski T, Cullen K, Watson K, Canada A, Bhatt R, Liu Y, Zakeri I. Food, Fun and Fitness Internet program for girls: influencing log-on rate. Health Education Research 2008 April;23(2):228-37. No exercise only group, No comparative control group
Ref ID: 231

(4838) Thompson JL, Davis SM, Gittelsohn J, Going S, Becenti A, Metcalfe L, Stone E, Harnack L, Ring K. Patterns of physical activity among American Indian children: an assessment of barriers and support. Journal of Community Health 2001 December;26(6):423-45. Survey or questionnaire
Ref ID: 1871

(4839) Thompson JL, Allen P, Helitzer DL, Qualls C, Whyte AN, Wolfe VK, Herman CJ. Reducing diabetes risk in American Indian women. American Journal of

Preventive Medicine 2008 March;34(3):192-201. Study limited to adults
Ref ID: 989

(4840) Thomsen C, Rasmussen O. Comparison of the effects of a monosaturated fat diet and a high carbohydrate diet on. European Journal of Clinical Nutrition 1999 October;53(10):818. Diet Intervention Study
Ref ID: 3759

(4841) Thomson RL, Buckley JD, Noakes M, Clifton PM, Norman RJ, Brinkworth GD. The effect of a hypocaloric diet with and without exercise training on body composition, cardiometabolic risk profile, and reproductive function in overweight and obese women with polycystic ovary syndrome. Journal of Clinical Endocrinology and Metabolism 2008 September;93(9):3373-80. Study limited to adults
Ref ID: 176

(4842) Thurmond RL, Gelfand EW, Dunford PJ. The role of histamine H1 and H4 receptors in allergic inflammation: the search for new antihistamines. Nature Reviews Drug Discovery 2008 January;7(1):41-53. Off topic
Ref ID: 3760

(4843) Thyagarajan B, Meyer A, Smith LJ, Beckett WS, Williams OD, Gross MD, Jacobs DR, Jr. Serum carotenoid concentrations predict lung function evolution in young adults: the Coronary Artery Risk Development in Young Adults (CARDIA) study. American Journal of Clinical Nutrition 2011 November;94(5):1211-8. Cohort Study
Ref ID: 2824

(4844) Tian H, Guo X, Wang X, He Z, Sun R, Ge S, Zhang Z. Chromium picolinate supplementation for overweight or obese adults. Cochrane Database of Systematic Reviews 2013;11:CD010063. Inappropriate Population
Ref ID: 6105

(4845) Tibana R, Aguiar F, Crispim G, Andrade D, Oliveira H, Silva RA. Perfil lipídico, composição corporal, pressão sanguínea arterial e aptidão física de crianças e adolescentes praticantes de futebol da Vila Telebrasília, Distrito Federal. Lipid profile, body composition, arterial blood pressure and physical fitness in children and teenagers recreational soccer players at vila telebrasília, Distrito Federal. Brasília Médica 2010 November;47(3). Cross-sectional study
Ref ID: 4596

(4846) Tibana RA, Balsamo S, Prestes J. Associação entre força muscular relativa e pressão arterial de repouso em mulheres sedentárias. Association between muscle strength and at-rest blood pressure among sedentary women. Revista Brasileira de Cardiologia 2011 June;24(3):163-8. Cross-sectional study
Ref ID: 4597

(4847) Tiedjen U, Stachow R, Haring J, Warschburger P, Petermann F. [Multimodal treatment for children with obesity]. Pravention und Rehabilitation 2004;16:49-58. Behavior Modification Intervention
Ref ID: 5041

(4848) Tigbe WW, Briggs AH, Lean ME. A patient-centred approach to estimate total annual healthcare cost by body mass index in the UK Counterweight programme. International Journal of Obesity (London) 2013 August;37(8):1135-9. Inappropriate Study Design
Ref ID: 6106

(4849) Timmler T, Wierusz K, Markuszewski J, niak W. [The hip joints of preterm neonates in sonographic evaluation]. Chirurgia Narzadów Ruchu i Ortopedia polska 2005;70:301-5. Off topic
Ref ID: 5042

(4850) Timmons BW, Bar-Or O, Riddell MC. Oxidation rate of exogenous carbohydrate during exercise is higher in boys than in men. Journal of Applied Physiology 2003 January;94(1):278-84. Acute study
Ref ID: 1782

(4851) Timmons BW, Bar-Or O. RPE during prolonged cycling with and without carbohydrate ingestion in boys and men. Medicine and Science in Sports and Exercise 2003 November;35(11):1901-7. Acute study
Ref ID: 1705

(4852) Timmons BW, Bar-Or O, Riddell MC. Influence of age and pubertal status on substrate utilization during exercise with and without carbohydrate intake in healthy boys. Applied Physiology, Nutrition, and Metabolism = Physiologie Appliquee, Nutrition et Metabolisme 2007 June;32(3):416-25. Diet Intervention or Supplement Study
Ref ID: 1232

(4853) Tint GS, Seller M, Hughesbenzie R, Batta AK, Shefer S, Genest D, Irons M, Elias E, Salen G. Markedly increased tissue concentrations of 7-dehydrocholesterol combined with low-levels of cholesterol are characteristic of the smith-lemli-opitz syndrome. Journal of Lipid Research 1995;36(1):89-95. Off topic
Ref ID: 5642

(4854) Tipton MJ, Golden FS, Higenbottam C, Mekjavic IB, Eglin CM. Temperature dependence of habituation of the initial responses to cold-water immersion. European Journal of Applied Physiology and Occupational Physiology 1998 August;78(3):253-7. Off topic
Ref ID: 2084

(4855) Tiryaki GR, Atterbom HA. The effects of sodium bicarbonate and sodium citrate on 600 m running time of trained females. Journal of Sports Medicine and

Physical Fitness 1995 September;35(3):194-8. Diet Intervention or Supplement Study
Ref ID: 2188

(4856) Tjonna AE, Stolen TO, Bye A, Volden M, Slordahl SA, Odegard R, Skogvoll E, Wisloff U. Aerobic interval training reduces cardiovascular risk factors more than a multitreatment approach in overweight adolescents. Clinical Science (Lond) 2009 February;116(4):317-26. No comparative control group
Ref ID: 163

(4857) Tkacz J, Young-Hyman D, Boyle CA, Davis CL. Aerobic exercise program reduces anger expression among overweight children. Pediatric Exercise Science 2008 November;20(4):390-401. Same subjects as another study already included
Ref ID: 138

(4858) Tobe H, Tanaka S, Koda M, Satake T, Hosoi T, Orimo H. Effects of bone mineral content and density on accuracy of body fat measurement by underwater weighing. Japanese Journal of Physical Fitness and Sports Medicine 1996;45(5):503-9. Off topic
Ref ID: 5643

(4859) Tobias JD. Continuous infusion of rocuronium in a paediatric intensive care unit. Canadian Journal of Anaesthesia = Journal Canadien d'Anesthésie 1996;43:353-7. Drug intervention study
Ref ID: 5043

(4860) Tobias JH, Steer CD, Mattocks CG, Riddoch C, Ness AR. Habitual levels of physical activity influence bone mass in 11-year-old children from the United Kingdom: findings from a large population-based cohort. Journal of Bone and Mineral Research 2007 January;22(1):101-9. Longitudinal Study
Ref ID: 1293

(4861) TODAY Study Group, Zeitler P, Epstein L, Grey M, Hirst K, Kaufman F, Tamborlane W, Wilfley D. Treatment options for type 2 diabetes in adolescents and youth: a study of the comparative efficacy of metformin alone or in combination with rosiglitazone or lifestyle intervention in adolescents with type 2 diabetes. Pediatric Diabetes 2007 April;8(2):74-87. Description versus conduct of study
Ref ID: 1245

(4862) TODAY Study Group. Design of a family-based lifestyle intervention for youth with type 2 diabetes: the TODAY study. [Review]. International Journal of Obesity 2010 February;34(2):217-26. Review article
Ref ID: 585

(4863) Todd MK, Reis-Bergan MJ, Sidman CL, Flohr JA, Jameson-Walker K, Spicer-Bartolau T, Wildeman K. Effect of a family-based intervention on electronic

media use and body composition among boys aged 8--11 years: a pilot study. Journal of Child Health Care 2008 December;12(4):344-58. No exercise only group
Ref ID: 146

(4864) Toftager M, Christiansen LB, Kristensen PL, Troelsen J. SPACE for physical activity--a multicomponent intervention study: study design and baseline findings from a cluster randomized controlled trial. BMC Public Health 2011;11:777. Not All Participants were Overweight and/or Obese
Ref ID: 2825

(4865) Toh SH, Guelfi KJ, Wong P, Fournier PA. Energy expenditure and enjoyment of small-sided soccer games in overweight boys. Human Movement Science 2011 June;30(3):636-47. Off topic
Ref ID: 2826

(4866) Tojo R, Leis R, Castro JR, Pombo M. Causas y consecuencias de la opulencia en la edad pediátrica. Revista Chilena de Nutrición 1999 August;26(2):175-84. Review article
Ref ID: 4598

(4867) Tolaizadeh AH, Maraghi S, Jelowdar A, Peyvasteh M. Human toxocariasis: A report of 3 cases. Pakistan Journal of Medical Sciences 2007;23(5):782-4. Case-Control / Case Study
Ref ID: 5644

(4868) Tolfrey K, Jones AM, Campbell IG. Lipid-lipoproteins in children: An exercise dose-response study. Medicine and Science in Sports and Exercise 2004 March;36(3):418-27. Not All Participants were Overweight and/or Obese
Ref ID: 1645

(4869) Tomé TH, Valentini NC. Benefícios da atividade física sistemática em parâmetros psicológicos do praticante: um estudo sobre ansiedade e agressividade. Revista da Educação Física 2006 December;17(2):123-30. Survey or questionnaire
Ref ID: 4599

(4870) Tomkinson GR, Clark AJ, Blanchonette P. Secular changes in body dimensions of Royal Australian Air Force aircrew (1971-2005). Ergonomics 2010;53(8):994-1005. Off topic
Ref ID: 5645

(4871) Tompkins CL, Soros A, Sothern MS, Vargas A. Effects of physical activity on diabetes management and lowering risk for type 2 diabetes. American Journal of Health Education 2009 September 1;40(5):286-90. Review article
Ref ID: 3916

(4872) Tomporowski PD, Davis CL, Lambourne K, Gregoski M, Tkacz J. Task switching in overweight children: effects of acute exercise and age. Journal of Sport and Exercise Psychology 2008 October;30(5):497-511. Acute study, Not an exercise intervention study
Ref ID: 154

(4873) Tone CM, Cardoza DM, Carpenter RH, Draghia-Akli R. Long-term effects of plasmid-mediated growth hormone releasing hormone in dogs. Cancer Gene Therapy 2004 May;11(5):389-96. Animal study
Ref ID: 3761

(4874) Tonnessen E, Shalfawi SA, Haugen T, Enoksen E. The effect of 40-m repeated sprint training on maximum sprinting speed, repeated sprint speed endurance, vertical jump, and aerobic capacity in young elite male soccer players. Journal of Strength and Conditioning Research 2011 September;25(9):2364-70. Off topic
Ref ID: 2827

(4875) Tonson A, Ratel S, Le Fur Y, Vilmen C, Cozzone PJ, Bendahan D. Muscle energetics changes throughout maturation: a quantitative P-31-MRS analysis. Journal of Applied Physiology 2010;109(6):1769-78. Off topic
Ref ID: 5646

(4876) Tonstad S, Knudtzon J, Sivertsen M, Refsum H, Ose L. Efficacy and safety of cholestyramine therapy in peripubertal and prepubertal children with familial hypercholesterolemia. Journal of Pediatrics 1996 July;129(1):42-9. Drug intervention study
Ref ID: 2173

(4877) Tonstad S. Identifying obese women most at risk from cardiovascular disease. International Journal of Obesity 2007;31:Suppl-25. Review article
Ref ID: 1147

(4878) Toplak H, Hamann A, Moore R, Masson E, Gorska M, Vercruysse F, Sun X, Fitchet M. Efficacy and safety of topiramate in combination with metformin in the treatment of obese subjects with type 2 diabetes: a randomized, double-blind, placebo-controlled study. International Journal of Obesity (London) 2007 January;31(1):138-46. Study limited to adults
Ref ID: 279

(4879) Topping DL, Bird AR. Food, nutrients and digestive health. Australian Journal of Nutrition and Dietetics 1999 September 2;56(3):S22-S34. Off topic
Ref ID: 3762

(4880) Torres-Duran PV, Ferreira-Hermosillo A, Ramos-Jimenez A, Hernandez-Torres RP, Juarez-Oropeza MA. Effect of Spirulina maxima on Postprandial Lipemia in Young Runners: A Preliminary Report. Journal of Medicinal Food

2012;15(8):753-7. Drug intervention study
Ref ID: 5647

(4881) Torres-Mejía G, Guzmán Pineda R, Téllez-Rojo MM, Lazcano-Ponce E. Peak bone mass and bone mineral density correlates for 9 to 24 year-old Mexican women, using corrected BMD. Pico mineral óseo y factores asociados a la densidad mineral ósea en mujeres mexicanas de 9 a 24 años de edad usando densidad mineral ósea corregida. Salud Pública de México 2009;51(supl.1):s84-s92. Cross-sectional study
Ref ID: 4600

(4882) Torres J, Palencia D, Sánchez DM, García J, Rey H, Echandía CA. Programa madre canguro: primeros resultados de una cohorte de niños seguidos desde la unidad neonatal hasta la semana 40 de edad postconcepcional. Kangaroomother program: results of follow-up at 40 weeks of postconceptional age. Colombia Médica 2006 June;37(2):96-101. Subjects less than 2 years old
Ref ID: 4601

(4883) Torres L, Hernandez JLJ, Almeida GBd, Gomide LB, Ambrósio V, Fernandes MIM. Avaliação clínica, nutricional e espirométrica de pacientes com fibrose cística após implantação de atendimento multidisciplinary. Clinical, nutritional and spirometric evaluation of patients with cystic fibrosis after the implementation of multidisciplinary treatment. Jornal Brasileiro de Pneumologia 2010 December;36(6):731-7. Retrospective study
Ref ID: 4602

(4884) Torres VE, Harris PC. Autosomal dominant polycystic kidney disease: The last 3 years. Kidney International 2009 July 15;76(2):149-68. Off topic
Ref ID: 3763

(4885) Torstveit MK, Sundgot-Borgen J. Low bone mineral density is two to three times more prevalent in non-athletic premenopausal women than in elite athletes: a comprehensive controlled study. British Journal of Sports Medicine 2005 July;39(5):282-7. Study limited to adults
Ref ID: 1522

(4886) Torun B, Viteri FE. Influence of exercise on linear growth. European Journal of Clinical Nutrition 1994;48:S186-S189. Animal study
Ref ID: 5044

(4887) Toruner EK, Savaser S. A controlled evaluation of a school-based obesity prevention in Turkish school children. Journal of School Nursing 2010;26:473-82. Behavior Modification Intervention
Ref ID: 5045

(4888) Torún B. Incremento de la actividad física mediante mejoría del estado nutricional. Archivos Latinoamericanos de Nutrición 1989 September;39(3):308,

309-8, 326. Review article
Ref ID: 4603

(4889) Tosetto AP, Simeão Júnior CA. Obesidade e sintomas de depressão, ansiedadee desesperança em mulheres sedentáriase não sedentárias. Obesity and symptoms of depression, anxiety and hopelessnessin sedentary and non-sedentary women. Medicina (Ribeirão Preto) 2008 December;41(4):497-507. Study limited to adults
Ref ID: 4604

(4890) Toubekis AG, Tsami AP, Tokmakidis SP. Critical velocity and lactate threshold in young swimmers. International Journal of Sports Medicine 2006 February;27(2):117-23. Off topic
Ref ID: 1416

(4891) Toulabi T, Khosh Niyat NM, Amini F, Nazari H, Mardani M. The influence of a behavior modification interventional program on body mass index in obese adolescents. Journal of the Formosan Medical Association 2012 March;111(3):153-9. Behavior Modification Intervention
Ref ID: 2828

(4892) Toussaint M. Patrones de dieta y actividad física en la patogénesis de la obesidad en el escolar urbano. Boletín Médico del Hospital Infantil de México 2000 November;57(11):650-62. Review article
Ref ID: 4605

(4893) Tovar Mojica G, Gutiérrez Poveda J, Ibáñez Pinilla M, Lobelo F. Sobrepeso, inactividad física y baja condición física en un colegio de Bogotá Colombia. Relationship between overweight, physical activity and physical fitness in school-aged boys in Bogotá Colombia. Archivos Latinoamericanos de Nutrición 2008 September;58(3):265-73. Prevalence study
Ref ID: 4606

(4894) Tovee MJ, Brown JE, Jacobs D. Maternal waist-to-hip ratio does not predict child gender. Proceedings of the Royal Society of London Series B-Biological Sciences 2001;268(1471):1007-10. Off topic
Ref ID: 5648

(4895) Townsend J, Wilkes H, Haines A, Jarvis M. Adolescent smokers seen in general practice: Health, lifestyle, physical measurements, and response to antismoking advice.[Erratum appears in British Medical Journal 1991 Nov 9;303(6811):1176]. British Medical Journal 1991 October 19;303(6808):947-50. Off topic
Ref ID: 2296

(4896) Townsend L, Findling RL. Modifying the risk of atypical antipsychotics in the treatment of juvenile-onset schizophrenia. Expert Opinion on Pharmacotherapy

2010;11(2):195-205. Review article
Ref ID: 3318

(4897) Toyoda Y, Yamaguchi M, Yoshimura N, Oka S, Okita Y. Cardioprotective effects and the mechanisms of terminal warm blood cardioplegia in pediatric cardiac surgery. Journal of Thoracic and Cardiovascular Surgery 2003 June;125(6):1242-51. Off topic
Ref ID: 1737

(4898) Tracer DP. Somatic versus reproductive energy allocation in Papua New Guinea: Life history theory and public health policy. American Journal of Human Biology 2002;14(5):621-6. Off topic
Ref ID: 5649

(4899) Trachtman H, Futterweit S, Schwob N, Maesaka J, Valderrama E. Recombinant Human Growth-Hormone Exacerbates Chronic Puromycin Aminonucleoside Nephropathy in Rats. Kidney International 1993;44(6):1281-8. Animal study
Ref ID: 5650

(4900) Trang NH, Hong TK, Van Der Ploeg HP, Hardy LL, Kelly PJ, Dibley MJ. Longitudinal physical activity changes in adolescents: Ho Chi Minh City Youth Cohort. Medicine and Science in Sports and Exercise 2012 August;44(8):1481-9. Inappropriate Study Design
Ref ID: 6107

(4901) Trapnell BC, Strausbaugh SD, Woo MS, Tong SY, Silber SA, Mulberg AE, Leitz G. Efficacy and safety of PANCREAZE® for treatment of exocrine pancreatic insufficiency due to cystic fibrosis. Journal of Cystic Fibrosis 2011;10(5):350-6. Drug intervention study
Ref ID: 3319

(4902) Trapp EG, Chisholm DJ, Freund J, Boutcher SH. The effects of high-intensity intermittent exercise training on fat loss and fasting insulin levels of young women. International Journal of Obesity (London) 2008 April;32(4):684-91. Study limited to adults
Ref ID: 207

(4903) Trappe SW, Costill DL, Vukovich MD, Jones J, Melham T. Aging among elite distance runners: a 22-yr longitudinal study. Journal of Applied Physiology 1996 January;80(1):285-90. Study limited to adults
Ref ID: 2169

(4904) Trasande L, Cronk C, Durkin M, Weiss M, Schoeller D, Gall E, Hewitt J, Carrel A, Landrigan P, Gillman M. Environment and obesity in the National Children's Study. Ambiente e obesidade no National Children's Study. Ciência and Saúde Coletiva 2010 January;15(1):195-210. Review article
Ref ID: 4607

(4905) Travi MIC, Bastos PRHd, Pontes ERJC. Prevalência de sobrepeso, obesidade e circunferência abdominal alterada em escolares de 6 a 11 anos de idade em Campo Grande/MS. Prevalence of overweight, obesity and altered abdominal circumference in school children aged 6 to 11 in Campo Grande/MS. Revista Brasileira em Promoção da Saúde (Impr) 2011 March;24(1). Prevalence study
Ref ID: 4608

(4906) Trejo Ortiz PM, Jasso Chairez S, Mollinedo Montaño FE, Lugo Balderas LG. Relación entre actividad física y obesidad en escolares. Relation between the physical activity and obesity in schoolchildren. Revista Cubana de Medicina General Integral 2012 March;28(1):34-41. Prevalence study
Ref ID: 4609

(4907) Treuth MS, Figueroa-Colon R, Hunter GR, Weinsier RL, Butte NF, Goran MI. Energy expenditure and physical fitness in overweight vs non-overweight prepubertal girls. International Journal of Obesity 1998;22(5):440-7. Not an exercise intervention study
Ref ID: 5651

(4908) Treuth MS, Sunehag AL, Trautwein LM, Bier DM, Haymond MW, Butte NF. Metabolic adaptation to high-fat and high-carbohydrate diets in children and adolescents. American Journal of Clinical Nutrition 2003 February;77(2):479-89. Diet Intervention Study
Ref ID: 1777

(4909) Treuth MS, Sherwood NE, Butte NF, McClanahan B, Obarzanek E, Zhou A, Ayers C, Adolph A, Jordan J, Jacobs DR, Rochon J. Validity and reliability of activity measures in African-American girls for GEMS. Medicine and Science in Sports and Exercise 2003 March;35(3):532-9. Off topic
Ref ID: 1769

(4910) Treuth MS, Catellier DJ, Schmitz KH, Pate RR, Elder JP, McMurray RG, Blew RM, Yang S, Webber L. Weekend and weekday patterns of physical activity in overweight and normal-weight adolescent girls. Obesity 2007 July;15(7):1782-8. Cross-sectional study
Ref ID: 1197

(4911) Trevino RP, Yin Z, Hernandez A, Hale DE, Garcia OA, Mobley C. Impact of the Bienestar school-based diabetes mellitus prevention program on fasting capillary glucose levels: a randomized controlled trial.[Erratum appears in Archives of Pediatric Adolescent Medicine. 2005 Apr;159(4):341]. Archives of Pediatrics and Adolescent Medicine 2004 September;158(9):911-7. Diet & Exercise intervention
Ref ID: 1591

(4912) Trinh A, Campbell M, Ukoumunne OC, Gerner B, Wake M. Physical activity and 3-year BMI change in overweight and obese children. Pediatrics 2013

February;131(2):e470-e477. Inappropriate Study Design
Ref ID: 6108

(4913) Trinity JD, Pahnke MD, Lee JF, Coyle EF. Interaction of hyperthermia and heart rate on stroke volume during prolonged exercise. Journal of Applied Physiology 2010 September;109(3):745-51. Drug intervention study
Ref ID: 2829

(4914) Trivers R, Manning JT, Thornhill R, Singh D, McGuire H. Jamaican Symmetry Project: Long-term study of fluctuating asymmetry in rural Jamaican children. Human Biology 1999;71(3):417-30. Off topic
Ref ID: 5652

(4915) Trochim WM, Cabrera DA, Milstein B, Gallagher RS, Leischow SJ. Practical challenges of systems thinking and modeling in public health. American Journal of Public Health 2006 March;96(3):538-46. Off topic
Ref ID: 3871

(4916) Troncon JK, Gomes JP, Guerra-Júnior G, Lalli CA. Prevalência de obesidade em crianças de uma escola pública e de um ambulatório geral de pediatria de hospital universitário. Obesity prevalence among students of a public school and a pediatric out-patient clinic of a university hospital. Revista Paulista de Pediatria 2007 December;25(4):305-10. Survey or questionnaire
Ref ID: 4610

(4917) Troncoso G, Núñez Villegas I, Guzmán Bondiek S. Protocolo de atención kinésica en pacientes portadores de obesidad mórbida sometidos a bypass gástrico. Revista Chilena de Cirugía 2002 August;54(4):437-43. Off topic
Ref ID: 4611

(4918) Trost SG, Fees B, Dzewaltowski D. Feasibility and efficacy of a "move and learn" physical activity curriculum in preschool children. Journal of Physical Activity and Health 2008 January;5(1):88-103. Primary outcome(s) not assessed, Not All Participants were Overweight and/or Obese
Ref ID: 204

(4919) Trost SG, Loprinzi PD, Moore R, Pfeiffer KA. Comparison of accelerometer cut points for predicting activity intensity in youth. Medicine and Science in Sports and Exercise 2011;43:1360-8. Off topic
Ref ID: 5049

(4920) Trost SG, Sundal D, Foster GD, Lent MR, Vojta D. Effects of a pediatric weight management program with and without active video games a randomized trial. JAMA Pediatrics 2014 May;168(5):407-13. Inappropriate Intervention
Ref ID: 6109

(4921) Trowbridge FL, Sofka D, Holt K, Barlow SE. Management of child and adolescent obesity: Study design and practitioner characteristics. Pediatrics

2002;110(1):205-9. Survey or questionnaire
Ref ID: 5653

(4922) Tsai CT, Chang WD, Kao MJ, Wang CJ, Lai PT. Changes in blood pressure and related autonomic function during cervical traction in healthy women. Orthopedics 2011 July;34(7):e295-e301. Off topic
Ref ID: 1097

(4923) Tsai PY, Boonpleng W, McElmurry BJ, Park CG, McCreary L. Lessons learned in using TAKE 10! with Hispanic children. Journal of School Nursing 25(2):163-72, 2009 Apr 2009;(2):163-72. Survey or questionnaire
Ref ID: 3154

(4924) Tsalikian E, Mauras N, Beck RW, Tamborlane WV, Janz KF, Chase HP, Wysocki T, Weinzimer SA, Buckingham BA, Kollman C, Xing D, Ruedy KJ, -Diabetes-Research-In-Children-Network-Direcnet-Study-Group. Impact of exercise on overnight glycemic control in children with type 1 diabetes mellitus. Journal of Pediatrics 2005;147:528-34. Acute study
Ref ID: 5050

(4925) Tsang TW, Kohn M, Chow CM, Singh MF. A randomized controlled trial of Kung Fu training for metabolic health in overweight/obese adolescents: the "martial fitness" study. Journal of Pediatric Endocrinology and Metabolism 2009 July;22(7):595-607. No comparative control group
Ref ID: 93

(4926) Tsang TW, Kohn MR, Chow CM, Singh MF. Self-perception and attitude toward physical activity in overweight/obese adolescents: the "martial fitness" study. Research in Sports Medicine 2013;21(1):37-51. Inappropriate Outcomes
Ref ID: 6110

(4927) Tsao TH, Yang JB, Yang CB, Hsu CH, Liou TL. The soluble leptin receptor of regular exercisers. International Journal of Sports Medicine 2007;28(9):732-5. Not All Participants were Overweight and/or Obese
Ref ID: 5654

(4928) Tsaoussoglou M, Bixler EO, Calhoun S, Chrousos GP, Sauder K, Vgontzas AN. Sleep-disordered breathing in obese children is associated with prevalent excessive daytime sleepiness, inflammation, and metabolic abnormalities. Journal of Clinical Endocrinology and Metabolism 2010;95(1):143-50. Off topic
Ref ID: 5655

(4929) Tseng ML, Ho CC, Chen SC, Huang YC, Lai CH, Liaw YP. A simple method for increasing levels of high-density lipoprotein cholesterol: a pilot study of combination aerobic- and resistance-exercise training. International Journal of Sport Nutrition and Exercise Metabolism 2013 June;23(3):271-81. Inappropriate Population
Ref ID: 6111

(4930) Tsiaras V, Zafeiridis A, Dipla K, Patras K, Georgoulis A, Kellis S. Prediction of peak oxygen uptake from a maximal treadmill test in 12- to 18-year-old active male adolescents. Pediatric Exercise Science 2010 November;22(4):624-37. Off topic
Ref ID: 2830

(4931) Tsiros MD, Sinn N, Coates AM, Howe PR, Buckley JD. Treatment of adolescent overweight and obesity. [Review] [80 refs]. European Journal of Pediatrics 2008 January;167(1):9-16. Review article
Ref ID: 1020

(4932) Tsiros MD, Sinn N, Brennan L, Coates AM, Walkley JW, Petkov J, Howe PR, Buckley JD. Cognitive behavioral therapy improves diet and body composition in overweight and obese adolescents. American Journal of Clinical Nutrition 2008 May;87(5):1134-40. No exercise only group
Ref ID: 185

(4933) Tsivgoulis SD, Papagelopoulos PJ, Efstathopoulos N, Papadakis NC, Kampanis NA, Christakis DG, Katonis PG. Accelerometry for evaluation of gait pattern in healthy soccer athletes. Journal of International Medical Research 2009 November;37(6):1692-700. Off topic
Ref ID: 613

(4934) Tsolakis CK, Vagenas GK, Dessypris AG. Strength adaptations and hormonal responses to resistance training and detraining in preadolescent males. Journal of Strength and Conditioning Research 2004;18:625-9. Not All Participants were Overweight and/or Obese
Ref ID: 5051

(4935) Tsuji LJS, Martin ID, Martin ES, LeBlanc A, Dumas P. Spring-harvested game birds in the Western James Bay region of Northern Ontario, Canada: the amount of organochlorines in matched samples of breast muscle, skin, and abdominal fat. Environmental Monitoring and Assessment 2008;146(1-3):91-104. Animal study
Ref ID: 5656

(4936) Tsunawake N, Tahara Y, Moji K, Muraki S, Minowa K, Yukawa K. Body composition and physical fitness of female volleyball and basketball players of the Japan inter-high school championship teams. Journal of Physiological Anthropology and Applied Human Science 2003 July;22(4):195-201. Off topic
Ref ID: 1719

(4937) Tucker LA, Seljaas GT, Hager RL. Body fat percentage of children varies according to their diet composition. Journal of the American Dietetic Association 1997;97(9):981-6. Diet Intervention Study
Ref ID: 5657

(4938) Tucker SJ, Lanningham-Foster LM, Murphy JN, Thompson WG, Weymiller AJ, Lohse C, Levine JA. Effects of a worksite physical activity intervention for hospital nurses who are working mothers. AAOHN Journal 2011 September;59(9):377-86. Study limited to adults
Ref ID: 2831

(4939) Tuckman BW, Hinkle JS. An experimental study of the physical and psychological effects of aerobic exercise on schoolchildren. Health Psychology 1986;5(3):197-207. Not All Participants were Overweight and/or Obese
Ref ID: 2349

(4940) Tur J, Alos M, Iglesias L, Luque L, Colom A, Escudero A, Martinez D, Pagan A, Ugarriza E, Frontera M, Nicola G, Palomero A, Tofe S, Urgeles JR, Barcelo MA, Couce M, De La Pena M, Fiol M, Cortes B, Teres E, Tumbarello A, Alvarez C, Salinas R, Pereg V, Gonzalez X et al. [TRAMOMTANA (Multidisciplinary treatment of morbid obesity: Medication, behavioral therapy, nutritional support, and physical activity). From question to reality in an investigator-initiated clinical trial (II)]. [Review] [Spanish]. Endocrinology and Nutrition 2011 June;58(6):299-307. Review article
Ref ID: 2832

(4941) Turgut G, Kaptanoglu B, Turgut S, Genc O, Tekinturk S. Influence of acute exercise on urinary protein, creatinine, insulin-like growth factor-I (IGF-I) and IGF binding protein-3 concentrations in children. Tohoku Journal of Experimental Medicine 2003;201(3):165-70. Acute study
Ref ID: 5658

(4942) Turley KR, Wilmore JH. Cardiovascular responses to submaximal exercise in 7- to 9-yr-old boys and girls. Medicine and Science in Sports and Exercise 1997 June;29(6):824-32. Acute study
Ref ID: 2131

(4943) Turley KR. The chemoreflex: adult versus child comparison. Medicine and Science in Sports and Exercise 2005 March;37(3):418-25. Acute study
Ref ID: 1539

(4944) Turley KR, DeSisso T, Gerst JW. Effects of caffeine on physiological responses to exercise: Boys versus men. Pediatric Exercise Science 2007;19(4):481-92. Diet Intervention or Supplement Study
Ref ID: 3320

(4945) Turner-McGrievy GM, Beets MW, Moore JB, Kaczynski AT, Barr-Anderson DJ, Tate DF. Comparison of traditional versus mobile app self-monitoring of physical activity and dietary intake among overweight adults participating in an mHealth weight loss program. Journal of the American Medical Informatics Association 2013 May 1;20(3):513-8. Inappropriate Population
Ref ID: 6112

(4946) Turnin MC, Tauber MT, Couvaras O, Jouret B, Bolzonella C, Bourgeois O, Buisson JC, Fabre D, Cance-Rouzaud A, Tauber JP, Hanaire-Broutin H. Evaluation of microcomputer nutritional teaching games in 1,876 children at school. Diabetes and Metabolism 2001 September;27(4:Pt 1):t-64. Diet Intervention Study
Ref ID: 1897

(4947) Twisk JW, Snel J, Kemper HC, van MW. Changes in daily hassles and life events and the relationship with coronary heart disease risk factors: A 2-year longitudinal study in 27-29-year-old males and females. Journal of Psychosomatic Research 1999 March;46(3):229-40. Longitudinal Study
Ref ID: 2038

(4948) Tylavsky FA, Holliday K, Danish R, Womack C, Norwood J, Carbone L. Fruit and vegetable intakes are an independent predictor of bone size in early pubertal children. American Journal of Clinical Nutrition 2004 February;79(2):311-7. Diet Intervention Study
Ref ID: 1670

(4949) Tyson JE, Perez A, Zanartu J. Human lactational response to oral thyrotropin releasing hormone. Journal of Clinical Endocrinology and Metabolism 1976 October;43(4):760-8. Off topic
Ref ID: 2388

(4950) Uauy R, Mize CE, Castillo-Duran C. Fat intake during childhood: metabolic responses and effects on growth. American Journal of Clinical Nutrition 2000 November;72(5 Suppl):1354S-60S. Not an exercise intervention study
Ref ID: 401

(4951) Uckun-Kitapci A, Haqq AM, Purnell JQ, Newcomb K, Gulkesen H, Underwood LE. Serum ghrelin concentrations are increased in children with growth hormone insensitivity and decrease during long-term insulinlike growth factor-I treatment. Journal of Investigative Medicine 2008;56(1):26-31. Drug intervention study
Ref ID: 5659

(4952) Ueno T, Sugawara H, Sujaku K, Hashimoto O, Tsuji R, Tamaki S, Torimura T, Inuzuka S, Sata M, Tanikawa K. Therapeutic effects of restricted diet and exercise in obese patients with fatty liver. Journal of Hepatology 1997 July;27(1):103-7. Diet & Exercise intervention
Ref ID: 2129

(4953) Ukkola O, Gagnon J, Rankinen T, Thompson PA, Hong Y, Leon AS, Rao DC, Skinner JS, Wilmore JH, Bouchard C. Age, body mass index, race and other determinants of steroid hormone variability: the HERITAGE Family Study. European Journal of Endocrinology 2001 July;145(1):1-9. Study limited to

adults
Ref ID: 1911

(4954) Uli N, Sundararajan S, Cuttler L. Treatment of childhood obesity. Current Opinion in Endocrinology, Diabetes and Obesity 2008;15(1):37-47. Review article
Ref ID: 3321

(4955) Ulrich DA, Burghardt AR, Lloyd M, Tiernan C, Hornyak JE. Physical activity benefits of learning to ride a two-wheel bicycle for children with Down syndrome: a randomized trial. Physical Therapy 2011 October;91(10):1463-77. Not All Participants were Overweight and/or Obese
Ref ID: 2833

(4956) Ulrich G, Bartsch P, Friedmann-Bette B. Total haemoglobin mass and red blood cell profile in endurance-trained and non-endurance-trained adolescent athletes. European Journal of Applied Physiology 2011 November;111(11):2855-64. Off topic
Ref ID: 2834

(4957) Uludag IF, Sener U, Zorlu Y, Koseoglu MH, Aydin TK. Serum leptin levels in epileptic patients treated with topiramate and valproic acid. Turkish Journal of Neurology 2011 March;17(1):17-31. Off topic
Ref ID: 3764

(4958) Umhau JC, Dauphinais KM, Patel SH, Nahrwold DA, Hibbeln JR, Rawlings RR, George DT. The relationship between folate and docosahexaenoic acid in men. European Journal of Clinical Nutrition 2006 March;60(3):352-7. Retrospective study
Ref ID: 3765

(4959) Unal M, Unal DO, Baltaci AK, Mogulkoc R, Kayserilioglu A. Investigation of serum leptin levels in professional male football players and healthy sedentary males. Neuroendocrinology Letters 2005 April;26(2):148-51. Off topic
Ref ID: 1521

(4960) Unger M, Faure M, Frieg A. Strength training in adolescent learners with cerebral palsy: a randomized controlled trial. Clinical Rehabilitation 2006;20:469-77. Not All Participants were Overweight and/or Obese
Ref ID: 5052

(4961) Urban C, Schwinger W, Benesch M, Lackner H, Kerbl R, Gili R, Patzold U, Burdach S. Feasibility of peripheral blood stem cell (PBSC) and peripheral blood mononuclear cell (PBMNC) separation in children with a body weight below 20 KG. Medical and Pediatric Oncology 1997;29(2):115-20. Off topic
Ref ID: 5660

(4962) Urizar GG, Hurtz SQ, Ahn DK, King AC, Albright CL, Atienza AA. Influence of maternal stress on successful participation in a physical activity intervention: the IMPACT Project. Women and Health 2005;42:63-82. Study limited to adults
Ref ID: 5053

(4963) Urushihara H, Fukuhara S, Tai S, Morita S, Chihara K. Heterogeneity in responsiveness of perceived quality of life to body composition changes between adult- and childhood-onset Japanese hypopituitary adults with GH deficiency during GH replacement. European journal of endocrinology / European Federation of Endocrine Societies 2007;156:637-45. Study limited to adults
Ref ID: 5054

(4964) Urzúa M, Avendaño H, Díaz C, Checura D. Calidad de vida y conductas alimentarias de riesgo en la preadolescencia. Quality of life and eating behaviors in preadolescence. Revista Chilena de Nutrición 2010 September;37(3):282-92. Cross-sectional study
Ref ID: 4612

(4965) Utter J, Scragg R, Schaaf D, Fitzgerald E, Wilson N. Correlates of body mass index among a nationally representative sample of New Zealand children. International Journal of Pediatric Obesity 2007;2(2):104-13. Survey or questionnaire
Ref ID: 1172

(4966) Vahl N, Juul A, Jorgensen JO, Orskov H, Skakkebaek NE, Christiansen JS. Continuation of growth hormone (GH) replacement in GH-deficient patients during transition from childhood to adulthood: a two-year placebo-controlled study. Journal of Clinical Endocrinology and Metabolism 2000 May;85(5):1874-81. Drug intervention study
Ref ID: 1976

(4967) Vahlkvist S, Inman MD, Pedersen S. Effect of asthma treatment on fitness, daily activity and body composition in children with asthma. Allergy 2010 November;65(11):1464-71. Not All Participants were Overweight and/or Obese
Ref ID: 2835

(4968) Vaiksaar S, Jurimae J, Maestu J, Purge P, Kalytka S, Shakhlina L, Jurimae T. No effect of menstrual cycle phase on fuel oxidation during exercise in rowers. European Journal of Applied Physiology 2011 June;111(6):1027-34. Off topic
Ref ID: 2836

(4969) Vaivre DL, Oriot D, Blossier P, Py A, Kasolter PM, Zwang J. The effect of multimodal stimulation and cutaneous application of vegetable oils on neonatal development in preterm infants: a randomized controlled trial. Child:Care, Health and Development 2009;35:96-105. Off topic
Ref ID: 5055

(4970) Vajda I, Meszaros J, Meszaros Z, Prokai A, Sziva A, Photiou A, Zsidegh P. Effects of 3 hours a week of physical activity on body fat and cardio-respiratory parameters in obese boys. Acta Physiologica Hungarica 2007 September;94(3):191-8. Not a randomized controlled trial (RCT)
Ref ID: 1167

(4971) Valdés EF. Disfunción endotelial y enfermedad cardiovascular. Revista de la Asociación Médica Argentina 1997;110(3):51-7. Review article
Ref ID: 4613

(4972) Valdimarsson O, Linden C, Johnell O, Gardsell P, Karlsson MK. Daily physical education in the school curriculum in prepubertal girls during 1 year is followed by an increase in bone mineral accrual and bone width--data from the prospective controlled Malmo pediatric osteoporosis prevention study. Calcified Tissue International 2006 February;78(2):65-71. Not All Participants were Overweight and/or Obese
Ref ID: 1417

(4973) Valdivia AB, Cartagena LC, Sarria NE, Távara IS, Seabra AFT, Silva RMGd, Maia JAR. Coordinación motora: influencia de la edad, sexo, estatus socio-económico y niveles de adiposidad en niños peruanos. Revista Brasileira de Cineantropometria e Desempenho Humano 2008 March;10(1). Cross-sectional study
Ref ID: 4614

(4974) Valea I, Tinto H, Drabo MK, Huybregts L, Henry MC, Roberfroid D, Guiguemde RT, Kolsteren P, D'Alessandro U. Intermittent preventive treatment of malaria with sulphadoxine- pyrimethamine during pregnancy in Burkina Faso: Effect of adding a third dose to the standard two-dose regimen on low birth weight, anaemia and pregnancy outcomes. Malaria Journal 2010;9:324. Off topic
Ref ID: 5057

(4975) Valente H, Padez C, Mourao I, Rosado V, Moreira P. Prevalence of nutritional inadequacy among portuguese children. Acta Medica Portuguesa 2010;23(3):365-70. Prevalence study
Ref ID: 5661

(4976) Valentin-Gudiol M, Mattern-Baxter K, Girabent-Farres M, Bagur-Calafat C, Hadders-Algra M, Angulo-Barroso RM. Treadmill interventions with partial body weight support in children under six years of age at risk of neuromotor delay. [Review]. Cochrane Database of Systematic Reviews (12):CD009242, 2011 2011;(12):CD009242. Review article
Ref ID: 1063

(4977) Valentino LA, Hakobyan N, Kazarian T, Jabbar KJ, Jabbar AA. Experimental haemophilic synovitis: rationale and development of a murine model of human

factor VIII deficiency. Haemophilia 2004;10(3):280-7. Off topic
Ref ID: 5662

(4978) van-Egmond FA, Bräuer W, Goldschmidt H, Hoff EH, Oepen J, Zimmermann E. [Effects of a programme for structured outpatient follow-up care after inpatient rehabilitation of obese children and adolescents--a multicentre, randomized study]. Die Rehabilitation 2006;45:40-51. Counseling Intervention
Ref ID: 5058

(4979) van Baak MA, van ME, Astrup AV, Finer N, Van Gaal LF, Hilsted J, Kopelman PG, Rossner S, James WP, Saris WH. Leisure-time activity is an important determinant of long-term weight maintenance after weight loss in the Sibutramine Trial on Obesity Reduction and Maintenance (STORM trial). American Journal of Clinical Nutrition 2003 August;78(2):209-14. Study limited to adults
Ref ID: 359

(4980) van Dam RM, Seidell JC. Carbohydrate intake and obesity. European Journal of Clinical Nutrition 2007 December 2;61:S75-S99. Diet Intervention Study
Ref ID: 3766

(4981) Van De Vijver E, Desager K, Mulberg AE, Staelens S, Verkade HJ, Bodewes FA, Malfroot A, Hauser B, Sinaasappel M, Van Biervliet S, Behm M, Pelckmans P, Callens D, Veereman-Wauters G. Treatment of infants and toddlers with cystic fibrosis-related pancreatic insufficiency and fat malabsorption with pancrelipase MT. Journal of Pediatric Gastroenterology and Nutrition 2011;53(1):61-4. Off topic
Ref ID: 3322

(4982) van den Berg-Emons RJ, van Baak MA, Speth L, Saris WH. Physical training of school children with spastic cerebral palsy: effects on daily activity, fat mass and fitness. International Journal of Rehabilitation Research 1998 June;21(2):179-94. Not All Participants were Overweight and/or Obese
Ref ID: 2067

(4983) van den Berg P, Thompson JK. Self-schema and social comparison explanations of body dissatisfaction: a laboratory investigation. Body Image 2007 March;4(1):29-38. Off topic
Ref ID: 1125

(4984) van der Heijden GJ, Sauer PJ, Sunehag AL. Twelve weeks of moderate aerobic exercise without dietary intervention or weight loss does not affect 24-h energy expenditure in lean and obese adolescents. American Journal of Clinical Nutrition 2010 March;91(3):589-96. Not All Participants were Overweight and/or Obese
Ref ID: 582

(4985) van der Heijden GJ, Wang ZJ, Chu ZD, Sauer PJ, Haymond MW, Rodriguez LM, Sunehag AL. A 12-week aerobic exercise program reduces hepatic fat accumulation and insulin resistance in obese, Hispanic adolescents.[Erratum appears in Obesity (Silver Spring). 2010 May;18(5):1062]. Obesity 2010 February;18(2):384-90. Not All Participants were Overweight and/or Obese
Ref ID: 597

(4986) van der Pligt P, Willcox J, Hesketh KD, Ball K, Wilkinson S, Crawford D, Campbell K. Systematic review of lifestyle interventions to limit postpartum weight retention: implications for future opportunities to prevent maternal overweight and obesity following childbirth. Obesity Reviews 2013 October;14(10):792-805. Inappropriate Study Design
Ref ID: 6113

(4987) van der Ploeg AT, Clemens PR, Corzo D, Escolar DM, Florence J, Groeneveld GJ, Herson S, Kishnani PS, Laforet P, Lake SL, Lange DJ, Leshner RT, Mayhew JE, Morgan C, Nozaki K, Park DJ, Pestronk A, Rosenbloom B, Skrinar A, van Capelle CI, van der Beek NA, Wasserstein M, Zivkovic SA. A randomized study of alglucosidase alfa in late-onset Pompe's disease. New England Journal of Medicine 2010 April 15;362(15):1396-406. Off topic
Ref ID: 551

(4988) van Egmond-Frohlich A, Brauer W, Goldschmidt H, Hoff-Emden H, Oepen J, Zimmermann E. [Effects of a programme for structured outpatient follow-up care after inpatient rehabilitation of obese children and adolescents--a multicentre, randomized study]. Rehabilitation (Stuttg) 2006 February;45(1):40-51. No exercise only group
Ref ID: 287

(4989) Van Koningsveld R, Schmitz PIM, Van Der Meche FGA, Visser LH, Meulstee J, Van Doorn PA. Effect of methylprednisolone when added to standard treatment with intravenous immunoglobulin for Guillain-Barre syndrome: Randomised trial. Lancet 2004;363(9404):192-6. Drug intervention study
Ref ID: 3323

(4990) Van Loan MD, Keim NL, Barbieri TF, Mayclin PL. The effects of endurance exercise with and without a reduction of energy intake on fat-free mass and the composition of fat-free mass in obese women. European Journal of Clinical Nutrition 1994 June;48(6):408-15. Study limited to adults
Ref ID: 2237

(4991) van Mil E, Westerterp KR, Kester ADM, Curfs LMG, Gerver WJM, Schrander-Stumpel C, Saris WHM. Activity related energy expenditure in children and adolescents with Prader-Willi syndrome. International Journal of Obesity 2000;24(4):429-34. Not a randomized controlled trial (RCT)
Ref ID: 5663

(4992) Van Mil EG, Westerterp KR, Kester AD, Delemarre-van de Waal HA, Gerver WJ, Saris WH. The effect of sibutramine on energy expenditure and body composition in obese adolescents. Journal of Clinical Endocrinology and Metabolism 2007 April;92(4):1409-14. No exercise only group, No comparative control group
Ref ID: 253

(4993) Van Schrojenstein Lantman-de Valk, Veenstra M, Straetmans J, Metsemakers J, Curfs L. Health management for people with intellectual disabilities in primary care; impact on basic physical functions. Journal of Intellectual Disability Research 2008 October;52(10):812. Off topic
Ref ID: 3872

(4994) van Sluijs EMF, Twisk JWR, Calfas KJ, van Poppel MNM, Chin AP, van Mechelen W. Effect of a tailored physical activity intervention delivered in general practice settings: Results of a randomized controlled trial. American Journal of Public Health 2005 October;95(10):1825-31. Study limited to adults
Ref ID: 3873

(4995) van Stralen MM, te Velde SJ, van NF, Brug J, Grammatikaki E, Maes L, De B, I, Verbestel V, Galcheva S, Iotova V, Koletzko BV, von KR, Bayer O, Kulaga Z, Serra-Majem L, Sanchez-Villegas A, Ribas-Barba L, Manios Y, Chinapaw MJ, ToyBox-study group. Weight status of European preschool children and associations with family demographics and energy balance-related behaviours: a pooled analysis of six European studies. [Review]. Obesity Reviews 2012 March;13:Suppl-41. Review article
Ref ID: 2838

(4996) Van CE, Gubbels J, De B, I, Cardon G. Feasibility and validity of accelerometer measurements to assess physical activity in toddlers. International Journal of Behavioral Nutrition and Physical Activity 2011;8:67. Off topic
Ref ID: 2839

(4997) Van d, V, Desager K, Mulberg AE, Staelens S, Verkade HJ, Bodewes FA, Malfroot A, Hauser B, Sinaasappel M, Van BS, Behm M, Pelckmans P, Callens D, Veereman WG. Treatment of infants and toddlers with cystic fibrosis-related pancreatic insufficiency and fat malabsorption with pancrelipase MT. Journal of Pediatric Gastroenterology and Nutrition 2011;53:61-4. Drug intervention study
Ref ID: 5060

(4998) Van DD, Sallis JF, Cardon G, Deforche B, Adams MA, Geremia C, De B, I. Associations of neighborhood characteristics with active park use: an observational study in two cities in the USA and Belgium. International Journal of Health Geographics 2013;12:26. Inappropriate Study Design
Ref ID: 6114

(4999) van GA, Renders CM, Veldhuis L, Looman CW, Hirasing RA, Raat H. Promotion of a healthy lifestyle among 5-year-old overweight children: health behavior outcomes of the 'Be active, eat right' study. BMC Public Health 2014;14:59. Inappropriate Intervention
Ref ID: 6115

(5000) van KE, Shimizu M, Wansink B. Food compensation: do exercise ads change food intake? International Journal of Behavioral Nutrition and Physical Activity 2011;8:6. Not an exercise intervention study
Ref ID: 2840

(5001) Van LL, Claessens AL, Vlietinck R, Derom C, Beunen G. Influence of weight-bearing exercises on bone acquisition in prepubertal monozygotic female twins: a randomized controlled prospective study. Calcified Tissue International 2003 June;72(6):666-74. Not All Participants were Overweight and/or Obese
Ref ID: 1708

(5002) Van LW, Verloigne M, De B, I, Brug J, Bjelland M, Lien N, Maes L. Does parental involvement make a difference in school-based nutrition and physical activity interventions? A systematic review of randomized controlled trials. International Journal of Public Health 2012 August;57(4):673-8. Inappropriate Study Design
Ref ID: 6116

(5003) van ME, Schoeber N, Calvert RE, Bar-Or O. Optimization of force in the Wingate Test for children with a neuromuscular disease. Medicine and Science in Sports and Exercise 1996 September;28(9):1087-92. Off topic
Ref ID: 2158

(5004) Vance ML. The Gordon Wilson Lecture. Growth hormone replacement in adults and other uses. [Review] [33 refs]. Transactions of the American Clinical and Climatological Association 1998;109:87-96. Review article
Ref ID: 2092

(5005) Vandenplas Y, Belli D, Benhamou P, Cadranel S, Cezard JP, Cucchiara S, Dupont C, Faure C, Gottrand F, Hassall E, Heymans H, Kneepkens CMF, Sandhu B. A critical appraisal of current management practices for infant regurgitation: Recommendations of a working party. European Journal of Pediatrics 1997 May;156(5):343. Off topic
Ref ID: 3767

(5006) Vanderford ML, Meyers MC, Skelly WA, Stewart CC, Hamilton KL. Physiological and sport-specific skill response of olympic youth soccer athletes. Journal of Strength and Conditioning Research 2004 May;18(2):334-42. Off topic
Ref ID: 1627

(5007) Vandongen R, Jenner DA, Thompson C, Taggart AC, Spickett EE, Burke V, Beilin LJ, Milligan RA, Dunbar DL. A controlled evaluation of a fitness and nutrition intervention program on cardiovascular health in 10- to 12-year-old children. Preventive Medicine 1995 January;24(1):9-22. Not All Participants were Overweight and/or Obese
Ref ID: 2212

(5008) Vanhelst J, Mikulovic J, Fardy P, Bui-Xuan G, Marchand F, Beghin L, Theunynck D. Effects of a multidisciplinary rehabilitation program on pediatric obesity: the CEMHaVi program. International Journal of Rehabilitation Research 2011 June;34(2):110-4. Not a randomized controlled trial (RCT)
Ref ID: 2841

(5009) Vanhelst J, Beghin L, Duhamel A, Bergman P, Sjostrom M, Gottrand F. Comparison of uniaxial and triaxial accelerometry in the assessment of physical activity among adolescents under free-living conditions: the HELENA study. BMC Medical Research Methodology 2012;12. Off topic
Ref ID: 5664

(5010) Vanhelst J, Fardy PS, Salleron J, Beghin L. The six-minute walk test in obese youth: reproducibility, validity, and prediction equation to assess aerobic power. Disability and Rehabilitation 2013 March;35(6):479-82. Inappropriate Study Design
Ref ID: 6117

(5011) Vann LH, Stanford FC, Durkin MW, Hanna A, Knight LM, Stallworth JR. "Moving and losing": A pilot study incorporating physical activity to decrease obesity in the pediatric population. Journal of the South Carolina Medical Association 2013 December;109(4):116-20. Inappropriate Outcomes
Ref ID: 6118

(5012) Varela MT, Duarte C, Salazar IC, Lema LF, Tamayo JA. Actividad física y sedentarismo en jóvenes universitarios de Colombia: prácticas, motivos y recursos para realizarlas. Physical activity and sedentary behavior in college youth from Colombian cities: practices, motivations, and resources. Colombia Médica 2011 September 26;42(3):269-77. Survey or questionnaire
Ref ID: 4615

(5013) Vargas Soler JA, Carrizosa Moog J, Alfaro Velásquez JM, Balthazar González V, Cornejo Ochoa JW, Cadavid AM, Jaramillo MC. Alteraciones del desarrollo físico y sexual en varones prepúberes y púberes con epilepsia, Hospital Universitario San Vicente de Paúl, Medellín, Colombia, 2005-2006. Disorders of physical and sexual development in prepubertal and pubertal males with epilepsy, at Hospital Universitario San Vicente de Paul Medellin 2005-2006. Latreia 2008 September;21(3):219-28. Off topic
Ref ID: 4616

(5014) Varo JJ, Martinez-Gonzalez MA, De Irala-Estevez J, Kearney J, Gibney M, Martinez JA. Distribution and determinants of sedentary lifestyles in the European Union. International Journal of Epidemiology 2003 February;32(1):138-46. Survey or questionnaire
Ref ID: 1759

(5015) Vasconcelos DF, Junqueira Júnior LF, Sanchez Osella OF. Comparaçäo ecodopplercardiográfica da dinâmica valvar entre atletas ciclistas, corredores e futebolistas e indivíduos sedentários. Arquivos Brasileiros de Cardiologia 1993 September;61(3):161-4. Off topic
Ref ID: 4617

(5016) Vasconcelos IQAd, Stabelini Neto A, Mascarenhas LPG, Bozza R, Ulbrich AZ, Campos Wd, Bertin RL. Fatores de risco cardiovascular em adolescentes com diferentes níveis de gasto energético. Cardiovascular risk factors in adolescents with different levels of energy expenditure. Arquivos Brasileiros de Cardiologia 2008 October;91(4):227-33. Survey or questionnaire
Ref ID: 4618

(5017) Vasconcelos JSd, Riet-Correa F, Dantas AF, Medeiros RMT, Galiza GJ, Oliveira DM, Pessoa AFA. Intoxicação por Mascagnia rigida (Malpighiaceae) em ovinos e caprinos. Pesquisa Veterinária Brasileira 2008 October;28(10):521-6. Animal study
Ref ID: 4619

(5018) Vasques DG, Duarte MdFdS, Lopes AdS. Morfologia de atletas juvenis de handebol. Revista Brasileira de Cineantropometria e Desempenho Humano 2007 June;9(2). Off topic
Ref ID: 4620

(5019) Vasquez F, Andrade M, Rodriguez MP, Salazar G. [Effect of educational nutrition program on the energy and macronutrients intake of preschoolers attending Junji day care centres in the eastern sector of Santiago, Chile]. [Spanish]. Archivos Latinoamericanos de Nutricion 2008 September;58(3):241-8. Diet Intervention Study
Ref ID: 832

(5020) Vaz CES, Guarniero R, Santana PJd, Dal Molin É, Morandini PH. Fixação interna de fragmento osteocondral originado de osteocondrite dissecante do joelho. Internal fixation of osteochondral fragment originated fro dissecting knee osteochondritis. Acta Ortopédica Brasileira 2008;16(4):247-9. Off topic
Ref ID: 4621

(5021) Vaz M, Pauline M, Unni US, Parikh P, Thomas T, Bharathi AV, Avadhany S, Muthayya S, Mehra R, Kurpad AV. Micronutrient supplementation improves physical performance measures in Asian Indian school-age children. Journal of

Nutrition 2011;141:2017-23. Diet Intervention Study
Ref ID: 5061

(5022) Vásquez V, Salazar R. Patrón de actividad física en un grupo de preescolares obesos asistentes a jardines infantiles de JUNJI, evaluado con sensor de movimiento. Revista Chilena de Nutrición 2005 August;32(2):110-7. Off topic
Ref ID: 4623

(5023) Vásquez V, Cardona H, Andrade S. M, Salazar R. Balance de energía, composición corporal y actividad física en preescolares eutróficos y obesos. Revista Chilena de Pediatría 2005 May;76(3):266-74. Cross-sectional study
Ref ID: 4624

(5024) Vásquez V, Cardona H, Andrade S. M. Balance de energía, composición corporal y actividad física en preescolares eutróficos y obesos. Revista de la Sociedad Boliviana de Pediatría 2007;46(3):199-208. Review article
Ref ID: 4625

(5025) Vázquez–Antona CA. La prevención primaria de la enfermedad cardiovascular inicia en la infancia. The primary prevention of the cardiovascular disease begins in the infancy. Archivos de Cardiología de México 2007 March;77(1):7-10. Review article
Ref ID: 4626

(5026) Vectore C, Alvarenga VC, Gomide Júnior S. Construção e validação de uma escala de comportamentos mediacionais de educadores infantis. Construcción y validación de una escala de comportamientos mediadotes de educadores infantiles. Psicologia Escolar e Educacional 2006 June;10(1):53-68. Off topic
Ref ID: 4627

(5027) Veiga OL, Gomez-Martinez S, Martinez-Gomez D, Villagra A, Calle ME, Marcos A, AFINOS Study Group. Physical activity as a preventive measure against overweight, obesity, infections, allergies and cardiovascular disease risk factors in adolescents: AFINOS Study protocol. BMC Public Health 2009;9:475. Description versus conduct of study
Ref ID: 616

(5028) Velasquez-Mieyer P, Neira CP, Nieto R, Cowan PA. Obesity and cardiometabolic syndrome in children. Therapeutic Advances in Cardiovascular Disease 2007;1(1):61-82. Review article
Ref ID: 3325

(5029) Velayati AA, Boloorsaze MR, Farnia P, Mohammadi F, Karam MB, Soheyla Z, Masjedi MR. Mycobacterium gastri causing disseminated infection in children of same family. Pediatric Pulmonology 2005;39(3):284-7. Off topic
Ref ID: 5665

(5030) Veldhuis L, Struijk MK, Kroeze W, Oenema A, Renders CM, Bulk-Bunschoten AM, Hirasing RA, Raat H. 'Be active, eat right', evaluation of an overweight prevention protocol among 5-year-old children: Design of a cluster randomised controlled trial. BMC Public Health 2009 June 8;9:177.:177. Description versus conduct of study
Ref ID: 114

(5031) Velez A, Golem DL, Arent SM. The impact of a 12-week resistance training program on strength, body composition, and self-concept of Hispanic adolescents. Journal of Strength and Conditioning Research 2010 April;24(4):1065-73. Not All Participants were Overweight and/or Obese
Ref ID: 51

(5032) Velkoska E, Morris MJ, Burns P, Weisinger RS. Leptin reduces food intake but does not alter weight regain following food deprivation in the rat. International Journal of Obesity and Related Metabolic Disorders 2003 January;27(1):48. Animal study
Ref ID: 3768

(5033) Vellar OD, Hermansen L. Physical performance and hematological parameters with special reference to hemoglobin and maximal oxygen uptake. Acta Medica Scandinavica - Supplementum 1971;522:1-40. Off topic
Ref ID: 2842

(5034) Venancio SI, Almeida Hd. Método Mãe Canguru: aplicação no Brasil, evidências científicas e impacto sobre o aleitamento materno. Jornal de Pediatria 2004 November;80(5,supl):s173-s180. Off topic
Ref ID: 689

(5035) Venditti EM, Elliot DL, Faith MS, Firrell LS, Giles CM, Goldberg L, Marcus MD, Schneider M, Solomon S, Thompson D, Yin Z. Rationale, design and methods of the HEALTHY study behavior intervention component. International Journal of Obesity (London) 2009 August;33 Suppl 4:S44-51.:S44-S51. Describes other aspects of the study
Ref ID: 106

(5036) Ventura E, Davis J, Byrd-Williams C, Alexander K, McClain A, Lane CJ, Spruijt-Metz D, Weigensberg M, Goran M. Reduction in risk factors for type 2 diabetes mellitus in response to a low-sugar, high-fiber dietary intervention in overweight Latino adolescents. Archives of Pediatrics and Adolescent Medicine 2009 April;163(4):320-7. Diet Intervention Study
Ref ID: 773

(5037) Vera L, Salvi C, Figueroa O, Soto de Sanabria I, López A. Evaluación nutricional y seguimiento de niños y adolescentes obesos en una consulta especializada. Archivos Venezolanos de Puericultura y Pediatría 2005

September;68(3):122-30. Retrospective study
Ref ID: 4628

(5038) Verbeken S, Braet C, Goossens L, van der Oord S. Executive function training with game elements for obese children: a novel treatment to enhance self-regulatory abilities for weight-control. Behaviour Research and Therapy 2013 June;51(6):290-9. Inappropriate Intervention
Ref ID: 6119

(5039) Verbestel V, De Henauw S, Maes L, Haerens L, Marild S, Eiben G, Lissner L, Moreno LA, Frauca NL, Barba G, Kovacs E, Konstabel K, Tornaritis M, Gallois K, Hassel H, De Bourdeaudhuij I. Using the intervention mapping protocol to develop a community-based intervention for the prevention of childhood obesity in a multi-centre European project: the IDEFICS intervention. International Journal of Behavioral Nutrition and Physical Activity 2011;8. Description versus conduct of study
Ref ID: 5666

(5040) Verdolini K, Min Y, Titze IR, Lemke J, Brown K, Jiang J, Fisher K. Biological mechanisms underlying voice changes due to dehydration. Journal of Speech, Language and Hearing Research 2002 April;45(2):268. Off topic
Ref ID: 3874

(5041) Verghese ST, McGill WA, Patel RI, Sell JE, Midgley FM, Ruttimann UE. Ultrasound-guided internal jugular venous cannulation in infants: a prospective comparison with the traditional palpation method. Anesthesiology 1999;91:71-7. Off topic
Ref ID: 906

(5042) Verhulst SL, Franckx H, Van GL, De BW, Desager K. The effect of weight loss on sleep-disordered breathing in obese teenagers. Obesity 2009 June;17(6):1178-83. Diet Intervention Study
Ref ID: 721

(5043) Verrips GHW, Stuifbergen MC, Den-Ouden AL, Bonsel GJ, Gemke RJB, Paneth N, Verloove-Vanhorick SP. Measuring health status using the Health Utilities Index: Agreement between raters and between modalities of administration. Journal of Clinical Epidemiology 2001;54:475-81. Off topic
Ref ID: 5062

(5044) Verschuren O, Ketelaar M, Gorter JW, Helders PJ, Takken T. Relation between physical fitness and gross motor capacity in children and adolescents with cerebral palsy. Developmental Medicine and Child Neurology 2009 November;51(11):866-71. Cross-sectional study
Ref ID: 667

(5045) Verstraeten R, Roberfroid D, Lachat C, Leroy JL, Holdsworth M, Maes L, Kolsteren PW. Effectiveness of preventive school-based obesity interventions

in low- and middle-income countries: a systematic review. American Journal of Clinical Nutrition 2012 August;96(2):415-38. Inappropriate Study Design
Ref ID: 6120

(5046) Vicente-Rodriguez G, Dorado C, Perez-Gomez J, Gonzalez-Henriquez JJ, Calbet JA. Enhanced bone mass and physical fitness in young female handball players. Bone 2004 November;35(5):1208-15. Cross-sectional study
Ref ID: 1573

(5047) Vicente-Rodriguez G, Urzanqui A, Mesana MI, Ortega FB, Ruiz JR, Ezquerra J, Casajus JA, Blay G, Blay VA, Gonzalez-Gross M, Moreno LA, AVENA-Zaragoza Study Group. Physical fitness effect on bone mass is mediated by the independent association between lean mass and bone mass through adolescence: a cross-sectional study. Journal of Bone and Mineral Metabolism 2008;26(3):288-94. Cross-sectional study
Ref ID: 955

(5048) Viebig RF, Valero MP, Araújo F, Yamada AT, Mansur AJ. Perfil de saúde cardiovascular de uma população adulta da região metropolitana de São Paulo. Arquivos Brasileiros de Cardiologia 2006 May;86(5):353-60. Study limited to adults
Ref ID: 4629

(5049) Vieira ÉLR, Katz CRT, Colares V. Indicadores de maus-tratos em crianças e adolescentes para uso na prática da odontopediatria. Indicators of child and adolescent maltreatment in pediatric dentistry practice. Odontologia Clínico-Científica 2008 June;7(2):113-8. Off topic
Ref ID: 4630

(5050) Vieira LF, Amorim HZ, Amorim AC, Rocha PGMd, Vieira JLL. Distúrbios de atitudes alimentares e sua relação com o crescimento físico de atletas paranaenses de Ginástica Rítmica. Eating disorders and the relationship with the physical growth in Paranaenses rythm gymnastic athletes. Motriz Revista de Educação Física (Improv) 2009 September;15(3):552-61. Cross-sectional study
Ref ID: 4632

(5051) Vieira LF, Teixeira CA, Silveira JMd, Teixeira CL, Oliveira Filho A, Rorato WR. Crianças e desempenho motor: um estudo associativo. Motriz Revista de Educação Física (Improv) 2009 December;15(4):804-9. Cross-sectional study
Ref ID: 4631

(5052) Vielma M, Sánchez M, Valeri L, Villarroel V, Arata-Bellabarba G, Paoli M. Marcadores inflamatorios en diabetes mellitus Tipo 1: Relación con perfil lipídico, control metabólico y duración de la enfermedad. Revista Venezolana de Endocrinología y Metabolismo 2010 June;8(2):46-54. Cross-sectional study
Ref ID: 4633

(5053) Vigil L, Gutiérrez R, Cáceres W, Collantes H, Beas J. Salud ocupacional en el trabajo de estiba: los trabajadores de mercados mayoristas de Huancayo, 2006. Revista Peruana de Medicina Experimental y Salud Publica 2007 December;24(4):336-42. Off topic
Ref ID: 4634

(5054) Vilar GE, Rodriguez De MA, Gra OB, Arus SE, Llanio NR, Calzadilla BL, Yasells GA, Del RA, V. Clinical trial: a nutritional supplement Viusid, in combination with diet and exercise, in patients with nonalcoholic fatty liver disease. Alimentary Pharmacology and Therapeutics 2009 November 15;30(10):999-1009. Diet Intervention or Supplement Study
Ref ID: 657

(5055) Villalobos Rodríguez I. Prevención del síndrome metabólico en adolescentes. Gaceta Médica de Caracas 2009 June;117(2):145-50. Diet & Exercise intervention
Ref ID: 4635

(5056) Villamor E, Mbise R, Spiegelman D, Hertzmark E, Fataki M, Peterson KE, Ndossi G, Fawzi WW. Vitamin A supplements ameliorate the adverse effect of HIV-1, malaria, and diarrheal infections on child growth. Pediatrics 2002;109:E6. Off topic
Ref ID: 5063

(5057) Villani RG, Gannon J, Self M, Rich PA. L-carnitine supplementation combined with aerobic training does not promote weight loss in moderately obese women. / La supplementation en L-carnitine associee a un entrainement aerobie ne favorise pas la perte de poids chez les femmes moderement obeses. International Journal of Sport Nutrition and Exercise Metabolism 2000 June;10(2):199-207. Study limited to adults
Ref ID: 3895

(5058) Villard LC, Ryden L, Stahle A. Predictors of healthy behaviours in Swedish school children. European Journal of Cardiovascular Prevention and Rehabilitation 2007 June;14(3):366-72. Survey or questionnaire
Ref ID: 1216

(5059) Villareal R. A randomized, open-label, intervention study to evaluate the effect of diet composition on weight gain in obese, type 2 diabetes patients receiving intensive insulin therapy. United States -- California: TUI University; 2008.¬ Diet Intervention Study
Ref ID: 5106

(5060) Vimaleswaran KS, Franks PW, Brage S, Sardinha LB, Andersen LB, Wareham NJ, Ekelund U, Loos RJ. Absence of association between the INSIG2 gene polymorphism (rs7566605) and obesity in the European Youth Heart Study

(EYHS). Obesity 2009 July;17(7):1453-7. Cross-sectional study
Ref ID: 735

(5061) Vinagre CG, Ficker ES, Finazzo C, Alves MJ, de AK, Irigoyen MC, Negrao CE, Maranhao RC. Enhanced removal from the plasma of LDL-like nanoemulsion cholesteryl ester in trained men compared with sedentary healthy men. Journal of Applied Physiology 2007 October;103(4):1166-71. Study limited to adults
Ref ID: 1156

(5062) Vincelet C, Tabone MD, Berthier M, Bonnefoi MC, Chevallier B, Lemaire JP, Dommergues JP, Groupe de pediatrie general. [How are personal child health records completed? A multicentric evaluation study]. [French]. Archives de Pediatrie 2003 May;10(5):403-9. Off topic
Ref ID: 1732

(5063) Vincent HK, Bourguignon CM, Vincent KR, Weltman AL, Bryant M, Taylor AG. Antioxidant supplementation lowers exercise-induced oxidative stress in young overweight adults. Obesity (Silver Spring) 2006 December;14(12):2224-35. Study limited to adults
Ref ID: 254

(5064) Vinet A, Le GD, Bouges S, Bernard PL, Poulain M, Varray A, Micallef JP. Prediction of VO(2peak) in wheelchair-dependent athletes from the adapted Leger and Boucher test. Spinal Cord 2002 October;40(10):507-12. Off topic
Ref ID: 1799

(5065) Vinter CA, Jensen DM, Ovesen P, Beck-Nielsen H, Jorgensen JS. The LiP (Lifestyle in Pregnancy) study: a randomized controlled trial of lifestyle intervention in 360 obese pregnant women. Diabetes Care 2011 December;34(12):2502-7. Study limited to adults
Ref ID: 2843

(5066) Virmani Ashr, Binienda Zk, Ali Sf, Gaetani Fran. Metabolic syndrome in drug abuse. Annals of the New York Academy of Sciences 2007 December 11;1122:50-68. Off topic
Ref ID: 3769

(5067) Vislocky LM, Pikosky MA, Rubin KH, Vega-Lopez S, Gaine PC, Martin WF, Zern TL, Lofgren IE, Fernandez ML, Rodriguez NR. Habitual consumption of eggs does not alter the beneficial effects of endurance training on plasma lipids and lipoprotein metabolism in untrained men and women. Journal of Nutritional Biochemistry 2009 January;20(1):26-34. Diet Intervention Study
Ref ID: 820

(5068) Vispute SS, Smith JD, LeCheminant JD, Hurley KS. The effect of abdominal exercise on abdominal fat. Journal of Strength and Conditioning Research 2011 September;25(9):2559-64. Study limited to adults
Ref ID: 2844

(5069) Vitola BE, Deivanayagam S, Stein RI, Mohammed BS, Magkos F, Kirk EP, Klein S. Weight loss reduces liver fat and improves hepatic and skeletal muscle insulin sensitivity in obese adolescents. Obesity 2009 September;17(9):1744-8. Diet Intervention Study
Ref ID: 682

(5070) Vivian EM. Type 2 diabetes in children and adolescents--the next epidemic?. [Review] [57 refs]. Current Medical Research and Opinion 2006 February;22(2):297-306. Review article
Ref ID: 1421

(5071) Vohr BR, Stephens BE, Higgins RD, Bann CM, Hintz SR, Das A, Newman JE, Peralta-Carcelen M, Yolton K, Dusick AM, Evans PW, Goldstein RF, Ehrenkranz RA, Pappas A, Adams-Chapman I, Wilson-Costello DE, Bauer CR, Bodnar A, Heyne RJ, Vaucher YE, Dillard RG, Acarregui MJ, McGowan EC, Myers GJ, Fuller J. Are Outcomes of Extremely Preterm Infants Improving? Impact of Bayley Assessment on Outcomes. Journal of Pediatrics 2012;161(2):222-+. Subjects less than 2 years old
Ref ID: 5667

(5072) Vokurka S, Bystricka E, Scudlova J, Mazur E, Visokaiova M, Vasilieva E, Brandejsova R, Chvojkova I, Vrabcova M, Vitkova J, Mjartanova D, Vodickova M, Bockova J, Streinerova K. The risk factors for oral mucositis and the effect of cryotherapy in patients after the BEAM and HD-l-PAM 200[NON-BREAKING SPACE]mg/m(2) autologous hematopoietic stem cell transplantation. European Journal of Oncology Nursing 15(5):508-12, 2011 Dec 2011;(5):508-12. Off topic
Ref ID: 3171

(5073) Volek JS, Gomez AL, Scheett TP, Sharman MJ, French DN, Rubin MR, Ratamess NA, McGuigan MM, Kraemer WJ. Increasing fluid milk favorably affects bone mineral density responses to resistance training in adolescent boys. Journal of the American Dietetic Association 2003 October;103(10):1353-6. Diet Intervention Study
Ref ID: 1710

(5074) Volkl TMK, Haas B, Beier C, Simm D, Dorr HG. Catch-down growth during infancy of children born small (SGA) or appropriate (AGA) for gestational age with short-statured parents. Journal of Pediatrics 2006;148(6):747-52. Off topic
Ref ID: 5668

(5075) Volkman KG, Stergiou N, Stuberg W, Blanke D, Stoner J. Factors Affecting Functional Reach Scores in Youth with Typical Development. Pediatric Physical Therapy 2009;21(1):38-44. Off topic
Ref ID: 5669

(5076) Volpi SCP, Rugolo LMSS, Peraçoli JC, Corrente JE. Aquisição de habilidades motoras até a marcha independente em prematuros de muito baixo peso.

Acquisition of motor abilities up to independent walking in very low birth weight preterm infants. Jornal de Pediatria 2010 April;86(2):143-8. Subjects less than 2 years old
Ref ID: 4636

(5077) Volterrani M, Giustina A, Manelli F, Cicoira MA, Lorusso R, Giordano A. Role of growth hormone in chronic heart failure: therapeutic implications. [Review] [59 refs]. Italian Heart Journal 2000 November;1(11):732-8. Review article
Ref ID: 1946

(5078) von Allworden HN, Horn S, Kahl J, Feldheim W. The influence of lecithin on plasma choline concentrations in triathletes and adolescent runners during exercise. European Journal of Applied Physiology and Occupational Physiology 1993;67(1):87-91. Diet Intervention or Supplement Study
Ref ID: 2255

(5079) Voorhees CC, Catellier DJ, Ashwood JS, Cohen DA, Rung A, Lytle L, Conway TL, Dowda M. Neighborhood socioeconomic status and non school physical activity and body mass index in adolescent girls. Journal of Physical Activity and Health 2009 November;6(6):731-40. Prevalence study
Ref ID: 617

(5080) Vorgerd M, Grehl T, Jager M, Muller K, Freitag G, Patzold T, Bruns N, Fabian K, Tegenthoff M, Mortier W, Luttmann A, Zange J, Malin JP. Creatine therapy in myophosphorylase deficiency (McArdle disease): a placebo-controlled crossover trial. Archives of Neurology 2000 July;57(7):956-63. Diet Intervention or Supplement Study
Ref ID: 1971

(5081) Vorgerd M, Zange J, Kley R, Grehl T, Husing A, Jager M, Muller K, Schroder R, Mortier W, Fabian K, Malin JP, Luttmann A. Effect of high-dose creatine therapy on symptoms of exercise intolerance in McArdle disease: double-blind, placebo-controlled crossover study. Archives of Neurology 2002 January;59(1):97-101. Diet Intervention or Supplement Study
Ref ID: 1855

(5082) Vos MB, McClain CJ. Nutrition and nonalcoholic fatty liver disease in children. Current Gastroenterology Reports 2008;10(3):308-15. Review article
Ref ID: 3327

(5083) Vos RC, Wit JM, Pijl H, Kruyff CC, Houdijk EC. The effect of family-based multidisciplinary cognitive behavioral treatment in children with obesity: study protocol for a randomized controlled trial. Trials [Electronic Resource] 2011;12:110. Behavior Modification Intervention
Ref ID: 2845

(5084) Vos RC, Huisman SD, Houdijk EC, Pijl H, Wit JM. The effect of family-based multidisciplinary cognitive behavioral treatment on health-related quality of life

in childhood obesity. Quality of Life Research 2012 November;21(9):1587-94. Inappropriate Intervention
Ref ID: 6121

(5085) Vriz O, Mos L, Frigo G, Sanigi C, Zanata G, Pegoraro F, Palatini P, HARVEST S, I. Effects of physical exercise on clinic and 24-hour ambulatory blood pressure in young subjects with mild hypertension. Journal of Sports Medicine and Physical Fitness 2002 March;42(1):83-8. Study limited to adults
Ref ID: 1849

(5086) Wadden TA, Hollander P, Klein S, Niswender K, Woo V, Hale PM, Aronne L. Weight maintenance and additional weight loss with liraglutide after low-calorie-diet-induced weight loss: the SCALE Maintenance randomized study. International Journal of Obesity (London) 2013 November;37(11):1443-51. Inappropriate Intervention
Ref ID: 6122

(5087) Wadwa RP. Noninvasive measures of cardiovascular changes in diabetes mellitus. Current Opinion in Endocrinology, Diabetes and Obesity 2007;14(4):263-8. Review article
Ref ID: 3328

(5088) Wafa SW, Talib RA, Hamzaid NH, McColl JH, Rajikan R, Ng LO, Ramli AH, Reilly JJ. Randomized controlled trial of a good practice approach to treatment of childhood obesity in Malaysia: Malaysian Childhood Obesity Treatment Trial (MASCOT). International Journal of Pediatric Obesity 2011 June;6(2-2):e62-e69. Behavior Modification Intervention
Ref ID: 2846

(5089) Wagener J, Nehls-Lowe B, Wisconsin State Dept.of Public Instruction M. Wisconsin youth risk behavior and HIV/AIDS prevention education: Survey results, 1991. Bulletin No. 93253. 1992 Jan 1.
Survey or questionnaire
Ref ID: 3917

(5090) Wagener TL, Fedele DA, Mignogna MR, Hester CN, Gillaspy SR. Psychological effects of dance-based group exergaming in obese adolescents. Pediatric Obesity 2012 October;7(5):e68-e74. Inappropriate Outcomes
Ref ID: 6123

(5091) Wagenmakers R, Stevens M, Groothoff JW, Zijlstra W, Bulstra SK, van BJ, van Raaij JJ, van dA-S, I. Physical activity behavior of patients 1 year after primary total hip arthroplasty: A prospective multicenter cohort study. Physical Therapy 2011 March;91(3):373-80. Cohort Study
Ref ID: 2847

(5092) Wagner A, Simon C, Oujaa M, Platat C, Schweitzer B, Arveiler D. Adiponectin is associated with lipid profile and insulin sensitivity in French adolescents.

Diabetes and Metabolism 2008;34(5):465-71. Cross-sectional study
Ref ID: 5670

(5093) Wagner MO, Kastner J, Petermann F, Jekauc D, Worth A, Bos K. The impact of obesity on developmental coordination disorder in adolescence. Research in Developmental Disabilities 2011;32(5):1970-6. Cross-sectional study
Ref ID: 5671

(5094) Wake M, Gold L, McCallum Z, Gerner B, Waters E. Economic evaluation of a primary care trial to reduce weight gain in overweight/obese children: The LEAP trial. Ambulatory Pediatrics 2008 September;8(5):336-41. No exercise only group
Ref ID: 156

(5095) Wake M, Baur LA, Gerner B, Gibbons K, Gold L, Gunn J, Levickis P, McCallum Z, Naughton G, Sanci L, Ukoumunne OC. Outcomes and costs of primary care surveillance and intervention for overweight or obese children: the LEAP 2 randomised controlled trial. British Medical Journal 2009 September 3;339:b3308. doi: 10.1136/bmj.b3308.:b3308. No exercise only group
Ref ID: 97

(5096) Wake M, Lycett K, Clifford SA, Sabin MA, Gunn J, Gibbons K, Hutton C, McCallum Z, Arnup SJ, Wittert G. Shared care obesity management in 3-10 year old children: 12 month outcomes of HopSCOTCH randomised trial. British Medical Journal 2013;346:f3092. Inappropriate Intervention
Ref ID: 6124

(5097) Wald AB, Uli NK. Pharmacotherapy in pediatric obesity: Current agents and future directions. Reviews in Endocrine and Metabolic Disorders 2009;10(3):205-14. Review article
Ref ID: 3329

(5098) Walders-Abramson N, Wamboldt FS, Curran-Everett D, Zhang L. Encouraging physical activity in pediatric asthma: a case-control study of the wonders of walking (WOW) program. Pediatric Pulmonology 2009 September;44(9):909-16. Case-Control / Case Study
Ref ID: 684

(5099) Walia KS, Khan EA, Ko DH, Raza SS, Khan YN. Side effects of antiepileptics: A review. Pain Practice 2004 September;4(3):194-203. Review article
Ref ID: 3770

(5100) Waling M, Lind T, Hernell O, Larsson C. A one-year intervention has modest effects on energy and macronutrient intakes of overweight and obese Swedish children. Journal of Nutrition 2010 October;140(10):1793-8. No exercise only group
Ref ID: 25

(5101) Walker LS, Bemben MG, Bemben DA, Knehans AW. Chromium picolinate effects on body composition and muscular performance in wrestlers. Medicine and Science in Sports and Exercise 30(12):1730-7, 1998 Dec 1998;(12):1730-7. Diet Intervention or Supplement Study
Ref ID: 3172

(5102) Walker R, Thomas M, Goodman D, Campbell S, Chadban S. Combination therapy with tacrolimus and mycophenolate mofetil: effects of early and late minimization of mycophenolate mofetil after renal transplant. Clinical Transplantation 2008 September;22(5):594-602. Drug intervention study
Ref ID: 3771

(5103) Walker SP, Grantham McGregor SM, Williams S, Himes JH. Adolescent girls school achievement: Nutrition, health and social factors. Cajanus 1998;31(1):21-34. Cross-sectional study
Ref ID: 4637

(5104) Walker WA, Goulet O, Morelli L, Antoine JM. Progress in the science of probiotics: from cellular microbiology and applied immunology to clinical nutrition. European Journal of Nutrition 2006 July 2;45:1-18. Off topic
Ref ID: 3772

(5105) Wallace JD, Abbott-Johnson WJ, Crawford DHG, Barnard R, Potter JM, Cuneo RC. GH treatment in adults with chronic liver disease: A randomized, double-blind, placebo-controlled, cross-over study. Journal of Clinical Endocrinology and Metabolism 2002;87(6):2751-9. Drug intervention study
Ref ID: 3330

(5106) Walldius G, Jungner I. REVIEW Apolipoprotein B and apolipoprotein A-I: risk indicators of coronary heart disease and targets for lipid-modifying therapy. Journal of Internal Medicine 2004 February;255(2):188-205. Review article
Ref ID: 3773

(5107) Walldius GÃ, Jungner I. The apoB/apoA-I ratio: a strong, new risk factor for cardiovascular disease and a target for lipid-lowering therapy: A review of the evidence. Journal of Internal Medicine 2006 May;259(5):493-519. Review article
Ref ID: 3774

(5108) Wallen EF, Mullersdorf M, Christensson K, Malm G, Ekblom O, Marcus C. High prevalence of cardio-metabolic risk factors among adolescents with intellectual disability.[Erratum appears in Acta Paediatrica. 2012 Feb;101(2):218]. Acta Paediatrica 2009 May;98(5):853-9. Cross-sectional study
Ref ID: 1089

(5109) Wallman K, Plant LA, Rakimov B, Maiorana AJ. The effects of two modes of exercise on aerobic fitness and fat mass in an overweight population. Research

in Sports Medicine 2009;17(3):156-70. No comparative control group
Ref ID: 96

(5110) Wallymahmed ME, Morgan C, Gill GV, MacFarlane IA. Aerobic fitness and hand grip strength in Type 1 diabetes: relationship to glycaemic control and body composition. Diabetic Medicine 2007 November;24(11):1296-9. Cross-sectional study
Ref ID: 1149

(5111) Walpole B, Dettmer E, Morrongiello B, McCrindle B, Hamilton J. Motivational interviewing as an intervention to increase adolescent self-efficacy and promote weight loss: Methodology and design. BMC Public Health 2011;11:459. Description versus conduct of study
Ref ID: 5064

(5112) Walpole B, Dettmer E, Morrongiello BA, McCrindle BW, Hamilton J. Motivational interviewing to enhance self-efficacy and promote weight loss in overweight and obese adolescents: A randomized controlled trial. Journal of Pediatric Psychology 2013 October;38(9):944-53. Inappropriate Intervention
Ref ID: 6125

(5113) Walsh AD, Lioret S, Cameron AJ, Hesketh KD, McNaughton SA, Crawford D, Campbell KJ. The effect of an early childhood obesity intervention on father's obesity risk behaviors: the Melbourne InFANT Program. International Journal of Behavioral Nutrition and Physical Activity 2014;11:18. Inappropriate Population
Ref ID: 6126

(5114) Walsh M, Laptook A, Kazzi SN, Engle WA, Yao Q, Rasmussen M, Buchter S, Heldt G, Rhine W, Higgins R, Poole K, -National-Institute-of-Child-Health-and-Human-Development-Neonatal-Research-Network. A cluster-randomized trial of benchmarking and multimodal quality improvement to improve rates of survival free of bronchopulmonary dysplasia for infants with birth weights of less than 1250 grams. Pediatrics 2007;119:876-90. Subjects less than 2 years old
Ref ID: 5065

(5115) Walters KL, LaMarr J, Levy RL, Pearson C, Maresca T, Mohammed SA, Simoni JM, Evans-Campbell T, Fredriksen-Goldsen K, Fryberg S, Jobe JB. Project hli?dx(w)/Healthy Hearts Across Generations: development and evaluation design of a tribally based cardiovascular disease prevention intervention for American Indian families. Journal of Primary Prevention 2012 August;33(4):197-207. Inappropriate Population
Ref ID: 6127

(5116) Walther C, Gaede L, Adams V, Gelbrich G, Leichtle A, Erbs S, Sonnabend M, Fikenzer K, Korner A, Kiess W, Bruegel M, Thiery J, Schuler G. Effect of increased exercise in school children on physical fitness and endothelial

progenitor cells: A prospective randomized trial. Circulation 2009 December 1;120(22):2251-9. Not All Participants were Overweight and/or Obese
Ref ID: 82

(5117) Walther C, Mende M, Gaede L, Muller U, Machalica K, Schuler G. [Effects of daily physical exercise at school on cardiovascular risk--results of a 2-year cluster-randomized study]. [German]. Deutsche Medizinische Wochenschrift 2011 November;136(46):2348-54. Not All Participants were Overweight and/or Obese
Ref ID: 1096

(5118) Wang CJ, McGlynn EA, Brook RH, Leonard CH, Piecuch RE, Hsueh SI, Schuster MA. Quality-of-care indicators for the neurodevelopmental follow-up of very low birth weight children: Results of an expert panel process. Pediatrics 2006;117(6):2080-92. Review article
Ref ID: 5672

(5119) Wang CL, Liang L, Fu JF, Zou CC, Hong F, Xue JZ, Lu JR, Wu XM. Effect of lifestyle intervention on non-alcoholic fatty liver disease in Chinese obese children. World Journal of Gastroenterology 2008 March 14;14(10):1598-602. No exercise only group
Ref ID: 189

(5120) Wang L, Dalton WT, III, Schetzina KE, Fulton-Robinson H, Holt N, Ho AL, Tudiver F, Wu T. Home food environment, dietary intake, and weight among overweight and obese children in Southern Appalachia. Southern Medical Journal 2013 October;106(10):550-7. Inappropriate Intervention
Ref ID: 6128

(5121) Wang LY, Gutin B, Barbeau P, Moore JB, Hanes J, Jr., Johnson MH, Cavnar M, Thornburg J, Yin Z. Cost-effectiveness of a school-based obesity prevention program. Journal of School Health 2008 December;78(12):619-24. No exercise only group
Ref ID: 151

(5122) Wang Y, Tussing L, Odoms-Young A, Braunschweig C, Flay B, Hedeker D, Hellison D. Obesity prevention in low socioeconomic status urban African-american adolescents: Study design and preliminary findings of the HEALTH-KIDS Study. European Journal of Clinical Nutrition 2006 January;60(1):92-103. Description versus conduct of study
Ref ID: 303

(5123) Wang Y, Liang H, Chen X. Measured body mass index, body weight perception, dissatisfaction and control practices in urban, low-income African American adolescents. BMC Public Health 2009 June 12;9:183.:183. Cross-sectional study
Ref ID: 112

(5124) Wang ZM, Heymsfield SB, Chen Z, Zhu SK, Pierson RN. Estimation of percentage body fat by dual-energy x-ray absorptiometry: Evaluation by in vivo human elemental composition. Physics in Medicine and Biology 2010;55(9):2619-35. Off topic
Ref ID: 5673

(5125) Wansink B, Shimizu M, Brumberg A. Association of nutrient-dense snack combinations with calories and vegetable intake. Pediatrics 2013 January;131(1):22-9. Inappropriate Intervention
Ref ID: 6129

(5126) Wapner RJ, Sorokin Y, Mele L, Johnson F, Dudley DJ, Spong CY, Peaceman AM, Leveno KJ, Malone F, Caritis SN, Mercer B, Harper M, Rouse DJ, Thorp JM, Ramin S, Carpenter MW, Gabbe SG, -National-Institute-of-Child-Health-and-Human-Development-Maternal-Fetal-Medicine-Units-Network. Long-term outcomes after repeat doses of antenatal corticosteroids. New England Journal of Medicine 2007;357:1190-8. Off topic
Ref ID: 5067

(5127) Ward DS, Benjamin SE, Ammerman AS, Ball SC, Neelon BH, Bangdiwala SI. Nutrition and physical activity in child care: results from an environmental intervention. American Journal of Preventive Medicine 2008 October;35(4):352-6. No exercise only group
Ref ID: 161

(5128) Ward DS, Vaughn AE, Bangdiwala KI, Campbell M, Jones DJ, Panter AT, Stevens J. Integrating a family-focused approach into child obesity prevention: Rationale and design for the My Parenting SOS study randomized control trial. BMC Public Health 2011;11:431. Description versus conduct of study
Ref ID: 2850

(5129) Ward KA, Roberts SA, Adams JE, Lanham NS, Mughal MZ. Calcium supplementation and weight bearing physical activity--do they have a combined effect on the bone density of pre-pubertal children? Bone 2007;41:496-504. Diet Intervention or Supplement Study
Ref ID: 5068

(5130) Ward KD, Vander Weg MW, Klesges RC, Kovach KW, Elrod MC, DeBon M, Haddock CK, Talcott GW, Lando HA. Characteristics of highly physically active smokers in a population of young adult military recruits. Addictive Behaviors 2003 October;28(8):1405-18. Cross-sectional study
Ref ID: 1712

(5131) Ward LM, Rauch F, Whyte MP, D'Astous J, Gates PE, Grogan D, Lester EL, McCall RE, Pressly TA, Sanders JO, Smith PA, Steiner RD, Sullivan E, Tyerman G, Smith-Wright DL, Verbruggen N, Heyden N, Lombardi A, Glorieux FH. Alendronate for the treatment of pediatric osteogenesis imperfecta: A

randomized placebo-controlled study. Journal of Clinical Endocrinology and Metabolism 2011;96(2):355-64. Drug intervention study
Ref ID: 3331

(5132) Ward NI, Soulsbury KA. The influence of the chemical additive tartrazine on the. Journal of Nutritional Medicine 1990 January;1(1):51. Drug intervention study
Ref ID: 3775

(5133) Ward P. The effect of an anabolic steroid on strength and lean body mass. Medicine and Science in Sports 1973;5(4):277-82. Drug intervention study
Ref ID: 2851

(5134) Ward SA, Tomezsko JL, Holsclaw DS, Paolone AM. Energy expenditure and substrate utilization in adults with cystic fibrosis and diabetes mellitus. American Journal of Clinical Nutrition 1999;69(5):913-9. Study limited to adults
Ref ID: 5674

(5135) Wardle J, Guthrie C, Sanderson S, Birch L, Plomin R. Food and activity preferences in children of lean and obese parents. International Journal of Obesity 2001;25(7):971-7. Survey or questionnaire
Ref ID: 5675

(5136) Wardle J, Brodersen NH, Boniface D. School-based physical activity and changes in adiposity. International Journal of Obesity 2007 September;31(9):1464-8. Longitudinal Study
Ref ID: 1176

(5137) Wareham NJ, van Sluijs EM, Ekelund U. Physical activity and obesity prevention: a review of the current evidence. [Review] [65 refs][Erratum appears in Proceedings of the Nutrition Society . 2005 Nov;64(4):581-4]. Proceedings of the Nutrition Society 2005 May;64(2):229-47. Review article
Ref ID: 1503

(5138) Warlaumont AS, Oller DK, Buder EH, Dale R, Kozma R. Data-driven automated acoustic analysis of human infant vocalizations using neural network tools. Journal of the Acoustical Society of America 2010;127(4):2563-77. Off topic
Ref ID: 5676

(5139) Warner JO, Goldsworthy SJ. Ketotifen in childhood allergic disease. [Review] [50 refs]. Clinical Allergy 1982 October;12:Suppl-7. Review article
Ref ID: 2369

(5140) Warren JM, Henry CJ, Simonite V. Low glycemic index breakfasts and reduced food intake in preadolescent children. Pediatrics 2003;112:e414. Diet Intervention Study
Ref ID: 5069

(5141) Warren JM, Henry CJ, Lightowler HJ, Bradshaw SM, Perwaiz S. Evaluation of a pilot school programme aimed at the prevention of obesity in children. Health Promotion International 2003 December;18(4):287-96. Not All Participants were Overweight and/or Obese
Ref ID: 344

(5142) Warren MW, Maples WR. The anthropometry of contemporary commercial cremation. Journal of Forensic Sciences 1997;42(3):417-23. Off topic
Ref ID: 5677

(5143) Warrington G, Ryan C, Murray F, Duffy P, Kirwan JP. Physiological and metabolic characteristics of elite tug of war athletes. British Journal of Sports Medicine 2001 December;35(6):396-401. Study limited to adults
Ref ID: 1875

(5144) Warwick PM, Garrow JS. The effect of addition of exercise to a regime of dietary restriction on weight loss, nitrogen balance, resting metabolic rate and spontaneous physical activity in three obese women in a metabolic ward. International Journal of Obesity 1981;5(1):25-32. Study limited to adults
Ref ID: 2373

(5145) Washburn RA, Jacobsen DJ, Sonko BJ, Hill JO, Donnelly JE. The validity of the Stanford Seven-Day Physical Activity Recall in young adults. Medicine and Science in Sports and Exercise 2003 August;35(8):1374-80. Off topic
Ref ID: 1728

(5146) Wasielewski NJ, Parker TM, KKotsko KM. Evaluation of electromyographic biofeedback for the quadriceps femoris: A systematic review. Journal of Athletic Training 2011 September;46(5):543-54. Review article
Ref ID: 3875

(5147) Wasser S, Ettrich KU, Schmidt KD, Selle D, Theile H. Influence of tyrosine administration onto cognitive ability in children with phenylketonuria. Fallstudien zum einfluss von tyrosingaben bei phenylketonurischen kindern auf kognitive prozesse. Klin Padiatr 1992;204:417-21. Drug intervention study
Ref ID: 1057

(5148) Waters E, de Silva-Sanigorski A, Hall BJ, Brown T, Campbell KJ, Gao Y, Armstrong R, Prosser L, Summerbell CD. Interventions for preventing obesity in children. [Review][Update of Psychosomatic Medicine. 2008 Apr;70(3):363-71; PMID: 18378876]. Cochrane Database of Systematic Reviews 2011;12:CD001871. Review article
Ref ID: 2852

(5149) Waters E, de Silva-Sanigorski A, Hall BJ, Brown T, Campbell KJ, Gao Y, Armstrong R, Prosser L, Summerbell CD. Interventions for preventing obesity in children. [Review]. Cochrane Database of Systematic Reviews (12):CD001871,

2011 2011;(12):CD001871. Review article
Ref ID: 1064

(5150) Watkins WE, Pollitt E. Effect of removing Ascaris on the growth of Guatemalan schoolchildren. Pediatrics 1996;97:871-6. Off topic
Ref ID: 5070

(5151) Watras AC, Buchholz AC, Close RN, Zhang Z, Schoeller DA. The role of conjugated linoleic acid in reducing body fat and preventing holiday weight gain. International Journal of Obesity (London) 2007 March;31(3):481-7. Study limited to adults
Ref ID: 272

(5152) Watson AW. Quantification of the influence of body fat content on selected physical performance variables in adolescent boys. Irish Journal of Medical Science 1988 December;157(12):383-4. Not a randomized controlled trial (RCT)
Ref ID: 2328

(5153) Watson G, Casa DJ, Fiala KA, Hile A, Roti MW, Healey JC, Armstrong LE, Maresh CM. Creatine use and exercise heat tolerance in dehydrated men. Journal of Athletic Training 2006 January;41(1):18-29. Study limited to adults
Ref ID: 3876

(5154) Watson K, Roberts B, Chow C, Goryakin Y, Rotman D, Gasparishvili A, Haerpfer C, McKee M. Micro- and meso-level influences on obesity in the former Soviet Union: a multi-level analysis. European Journal of Public Health 2013 April;23(2):291-8. Inappropriate Population
Ref ID: 6130

(5155) Watson RR, Moriguchi S, Jackson JC, Werner L, Wilmore JH, Freund BJ. Modification of cellular immune functions in humans by endurance exercise training during beta-adrenergic blockade with atenolol or propranolol. Medicine and Science in Sports and Exercise 1986 February;18(1):95-100. Drug intervention study
Ref ID: 2353

(5156) Watt RG, Tull KI, Hardy R, Wiggins M, Kelly Y, Molloy B, Dowler E, Apps J, McGlone P. Effectiveness of a social support intervention on infant feeding practices: Randomised controlled trial. Journal of Epidemiology and Community Health 2009;63:156-62. Subjects less than 2 years old
Ref ID: 5071

(5157) Watterberg KL, Shaffer ML, Mishefske MJ, Leach CL, Mammel MC, Couser RJ, Abbasi S, Cole CH, Aucott SW, Thilo EH, Rozycki HJ, Lacy CB. Growth and neurodevelopmental outcomes after early low-dose hydrocortisone treatment in extremely low birth weight infants. Pediatrics 2007;120:40-8. Subjects less than

2 years old
Ref ID: 5072

(5158) Watts K, Jones TW, Davis EA, Green D. Exercise training in obese children and adolescents: current concepts. [Review] [75 refs]. Sports Medicine 2005;35(5):375-92. Review article
Ref ID: 1517

(5159) Wässer S, Ettrich KU, Schmidt KD, Selle D, Theile H. [Case studies of the effect of tyrosine administration in children with phenylketonuria on cognitive processes]. Klinische Pädiatrie 1992;204:417-21. Case-Control / Case Study
Ref ID: 5073

(5160) Weaver CM, Teegarden D, Lyle RM, McCabe GP, McCabe LD, Proulx W, Kern M, Sedlock D, Anderson DD, Hillberry BM, Peacock M, Johnston CC. Impact of exercise on bone health and contraindication of oral contraceptive use in young women. Medicine and Science in Sports and Exercise 2001 June;33(6):873-80. Study limited to adults
Ref ID: 1914

(5161) Webb AL, Villamor E. Update: Effects of Antioxidant and Non-Antioxidant Vitamin Supplementation on Immune Function. Nutrition Reviews 2007 May;65(5):181-217. Review article
Ref ID: 3776

(5162) Webb GP. A critical survey of methods used to investigate links. Journal of Biological Education (Society of Biology) 1992;26(4):263. Off topic
Ref ID: 577

(5163) Webber LS, Osganian SK, Feldman HA, Wu M, McKenzie TL, Nichaman M, Lytle LA, Edmundson E, Cutler J, Nader PR, Luepker RV. Cardiovascular risk factors among children after a 2 1/2-year intervention-The CATCH Study. Preventive Medicine 1996 July;25(4):432-41. No exercise only group, Not All Participants were Overweight and/or Obese
Ref ID: 429

(5164) Webber LS, Catellier DJ, Lytle LA, Murray DM, Pratt CA, Young DR, Elder JP, Lohman TG, Stevens J, Jobe JB, Pate RR, TAAG Collaborative Research Group. Promoting physical activity in middle school girls: Trial of Activity for Adolescent Girls. American Journal of Preventive Medicine 2008 March;34(3):173-84. Not All Participants were Overweight and/or Obese
Ref ID: 990

(5165) Weber JL, Cunningham SL, Skipper B, Lytle L, Stevens J, Gittelsohn J, Anliker J, Heller K, Pablo JL. Portion-size estimation training in second- and third-grade American Indian children. American Journal of Clinical Nutrition 1999;69:782S-7S. Diet Intervention Study
Ref ID: 889

(5166) Weber T, Schulz G, Beyer J, Geiling H, Cordes U, Diederich C, Krause U. The influence of penbutolol and placebo on blood sugar levels and insulin consumption in the glucose-controlled insulin infusion system ("artificial endocrine pancreas"). Klinische Wochenschrift 1990 October 3;68(19):951-8. Drug intervention study
Ref ID: 2305

(5167) Wedig J, Christian MS, Hoberman A, Diener RM, Thomas-Wedig R. A study to develop methodology for feeding 24-hour-old neonatal swine for 3 weeks. International Journal of Toxicology 2002;21(5):361-70. Animal study
Ref ID: 5678

(5168) Weeks BK, Young CM, Beck BR. Eight months of regular in-school jumping improves indices of bone strength in adolescent boys and Girls: the POWER PE study. Journal of Bone and Mineral Research 2008 July;23(7):1002-11. Not All Participants were Overweight and/or Obese
Ref ID: 933

(5169) Wehby GL, Castilla EE, Goco N, Rittler M, Cosentino V, Javois L, McCarthy AM, Bobashev G, Litavecz S, Mariona A, Dutra G, Lopez-Camelo JS, Orioli IM, Murray JC. Description of the methodology used in an ongoing pediatric care interventional study of children born with cleft lip and palate in South America [NCT00097149]. BMC Pediatrics 2006;6:9. Off topic
Ref ID: 5074

(5170) Wehmeier PM, Schacht A, Dittmann RW, Helsberg K, Schneider FC, Lehmann M, Bullinger M, Ravens SU. Effect of atomoxetine on quality of life and family burden: results from a randomized, placebo-controlled, double-blind study in children and adolescents with ADHD and comorbid oppositional defiant or conduct disorder. Quality of Life Research 2011;20:691-702. Drug intervention study
Ref ID: 5075

(5171) Weickert MO, Pfeiffer AF. [Preventing type 2 diabetes: what does dietary fiber achieve?]. [Review] [0 refs] [German]. MMW Fortschritte der Medizin 2005 April 28;147(17):28-30. Review article
Ref ID: 1516

(5172) Weigel C, Kokocinski K, Lederer P, Dotsch J, Rascher W, Knerr I. Childhood obesity: concept, feasibility, and interim results of a local group-based, long-term treatment program. Journal of Nutrition Education and Behavior 2008 November;40(6):369-73. No exercise only group
Ref ID: 153

(5173) Weinstock J. A Review of Exercise as Intervention for Sedentary Hazardous Drinking College Students: Rationale and Issues. Journal of American College

Health 2010 May;58(6):539-44. Review article
Ref ID: 3877

(5174) Weintraub DL, Tirumalai EC, Haydel KF, Fujimoto M, Fulton JE, Robinson TN. Team sports for overweight children: the Stanford Sports to Prevent Obesity Randomized Trial (SPORT). Archives of Pediatric Adolescent Medicine 2008 March;162(3):232-7. Inappropriate Intervention
Ref ID: 191

(5175) Weintraub M, Ginsberg G, Stein EC, Sundaresan PR, Schuster B, O'Connor P, Byrne LM. Phenylpropanolamine OROS (Acutrim) vs. placebo in combination with caloric restriction and physician-managed behavior modification. Clinical Pharmacology and Therapeutics 1986 May;39(5):501-9. Drug intervention study
Ref ID: 2352

(5176) Weiping Q, Bauman WA, Cardozo C. Bone and muscle loss after spinal cord injury: organ interactions Bone and muscle after SCI Qin et al. Annals of the New York Academy of Sciences 2010 November 15;1211(1):66-84. Off topic
Ref ID: 3777

(5177) Weise C, Heunemann C, Loddenkemper C, Herz U, van Tol EAF, Worm M. Dietary docosahexaenoic acid in combination with arachidonic acid ameliorates allergen-induced dermatitis in mice. Pediatric Allergy and Immunology 2011 August;22(5):497-504. Animal study
Ref ID: 3778

(5178) Weise M, Eisenhofer G, Merke DP. Pubertal and gender-related changes in the sympathoadrenal system in healthy children. Journal of Clinical Endocrinology and Metabolism 2002;87(11):5038-43. Off topic
Ref ID: 5679

(5179) Weisskopf MG, Anderson HA, Hanrahan LP, Kanarek MS, Falk CM, Steenport DM, Draheim LA. Maternal exposure to Great Lakes sport-caught fish and dichlorodiphenyl dichloroethylene, but not polychlorinated biphenyls, is associated with reduced birth weight. Environmental Research 2005;97(2):149-62. Off topic
Ref ID: 5680

(5180) Weisstaub G, Hertrampf E, de Romana DL, Salazar G, Bugueno C, Castillo-Duran C. Plasma zinc concentration, body composition and physical activity in obese preschool children. Biological Trace Element Research 2007;118(2):167-74. Cross-sectional study
Ref ID: 5681

(5181) Weisstaub SG, Araya Q. Recuperación nutricional: un desafío pendiente. Revista médica de Chile 2003;131(2):213-9. Review article
Ref ID: 728

(5182) Wells JCK. Body composition in childhood: effects of normal growth and disease. Proceedings of the Nutrition Society 2003;62(2):521-8. Review article
Ref ID: 5682

(5183) Wells JCK. Parent-offspring conflict theory, signaling of need, and weight gain in early life. Quarterly Review of Biology 2003 June;78(2):169. Off topic
Ref ID: 3779

(5184) Welsman JR, Armstrong N. Longitudinal changes in submaximal oxygen uptake in 11- to 13-year-olds. Journal of Sports Sciences 2000 March;18(3):183-9. Longitudinal Study
Ref ID: 1985

(5185) Wen LM, Baur LA, Rissel C, Wardle K, Alperstein G, Simpson JM. Early intervention of multiple home visits to prevent childhood obesity in a disadvantaged population: a home-based randomised controlled trial (Healthy Beginnings Trial). BMC Public Health 2007 May 10;7:76.:76. Description versus conduct of study
Ref ID: 239

(5186) Wen LM, Merom D, Rissel C, Simpson JM. Weight status, modes of travel to school and screen time: a cross-sectional survey of children aged 10-13 years in Sydney. Health Promotion Journal of Australia 2010 April;21(1):57-63. Not a randomized controlled trial (RCT)
Ref ID: 48

(5187) Wen LM, Baur LA, Rissel C, Flood V, Simpson JM, Hayes A, Hardy LL, Wardle K. Healthy Beginnings Trial Phase 2 study: follow-up and cost-effectiveness analysis. Contemporary Clinical Trials 2012 March;33(2):396-401. Follow-up Study
Ref ID: 2853

(5188) Werf MJV, Benthem BHBV, Ameijden EJCV. Prevalence, incidence and risk factors of anaemia in HIV-positive and HIV-negative drug users. Addiction 2000 March;95(3):383-92. Off topic
Ref ID: 3896

(5189) Wergel-Kolmert U, Wohlfart B. Day-to-day variation in oxygen consumption and energy expenditure during submaximal treadmill walking in female adolescents. Clinical Physiology 1999 March;19(2):161-8. Acute study
Ref ID: 2033

(5190) Werk Rd, Vieira AZ, Nuñez PRM, Habitante CA, Silva JVPd. Aptidão física relacionada à saúde de crianças de uma escola estadual de campo grande/ms. Health related physical fitness on children in a public school in campo grande/ms. Aptitud física relacionada a la salud de niños de una escuela de la estadual de campo grande/ms. Ciência Cuidado e Saúde 2009 March;8(1):42-

7. Cross-sectional study
Ref ID: 4638

(5191) Wernstedt P, Sjostedt C, Ekman I, Du H, Thuomas KA, Areskog NH, Nylander E. Adaptation of cardiac morphology and function to endurance and strength training. A comparative study using MR imaging and echocardiography in males and females. Scandinavian Journal of Medicine and Science in Sports 2002 February;12(1):17-25. Cross-sectional study
Ref ID: 1836

(5192) Wertlieb D. Converging Trends in Family Research and Pediatrics: Recent findings for the American Academy of Pediatrics Task Force on the Family. Pediatrics 2003 June 2;111(6):1572. Review article
Ref ID: 3780

(5193) Wessely S, Nickson J, Cox B. Symptoms of low blood pressure: A population study. British Medical Journal 1990 August 18;301(6748):362-5. Cross-sectional study
Ref ID: 2307

(5194) West K, Wallen M, Follett J. Acapella vs. PEP mask therapy: A randomised trial in children with cystic fibrosis during respiratory exacerbation. Physiotherapy Theory and Practice 2010;26:143-9. Off topic
Ref ID: 5076

(5195) West KP, LeClerq SC, Shrestha SR, Wu LS, Pradhan EK, Khatry SK, Katz J, Adhikari R, Sommer A. Effects of vitamin A on growth of vitamin A-deficient children: field studies in Nepal. Journal of Nutrition 1997;127:1957-65. Diet Intervention or Supplement Study
Ref ID: 5077

(5196) West SD, Kohler M, Nicoll DJ, Stradling JR. The effect of continuous positive airway pressure treatment on physical activity in patients with obstructive sleep apnoea: A randomised controlled trial. Sleep Medicine 2009 October;10(9):1056-8. Study limited to adults
Ref ID: 676

(5197) West ST, Shores KA, Mudd LM. Association of available parkland, physical activity, and overweight in America's largest cities. Journal of Public Health Management and Practice 2012 September;18(5):423-30. Inappropriate Study Design
Ref ID: 6131

(5198) Westerstahl M, Barnekow-Bergkvist M, Hedberg G, Jansson E. Secular trends in sports: participation and attitudes among adolescents in Sweden from 1974 to 1995. Acta Paediatrica 2003;92(5):602-9. Survey or questionnaire
Ref ID: 5684

(5199) Westerstahl M, Barnekow-Bergkvist M, Hedberg G, Jansson E. Secular trends in body dimensions and physical fitness among adolescents in Sweden from 1974 to 1995. Scandinavian Journal of Medicine and Science in Sports 2003;13(2):128-37. Longitudinal Study
Ref ID: 5683

(5200) Westerterp-Plantenga MS, Lejeune MP, Nijs I, van OM, Kovacs EM. High protein intake sustains weight maintenance after body weight loss in humans. International Journal of Obesity and Related Metabolic Disorders 2004 January;28(1):57-64. Not an exercise intervention study
Ref ID: 342

(5201) Westgarth C, Heron J, Ness AR, Bundred P, Gaskell RM, Coyne KP, German AJ, McCune S, Dawson S. Family Pet Ownership during Childhood: Findings from a UK Birth Cohort and Implications for Public Health Research. International Journal of Environmental Research and Public Health 2010;7(10):3704-29. Off topic
Ref ID: 5685

(5202) Westgarth C, Liu JH, Heron J, Ness AR, Bundred P, Gaskell RM, German AJ, McCune S, Dawson S. Dog Ownership during Pregnancy, Maternal Activity, and Obesity: A Cross-Sectional Study. PLoS ONE 2012;7(2). Off topic
Ref ID: 5686

(5203) Westhof G, Deerberg JC, Schad W, Zimmermann RC, Hatzmann H. Do Macromorphological Features of the Human Placenta Influence Somatic and Psychomotor Development of the Newborn and Early Infant? A Historic Question Revisited. Gynecologic and Obstetric Investigation 2010;69(4):251-9. Subjects less than 2 years old
Ref ID: 5687

(5204) Weston AR, Karamizrak O, Smith A, Noakes TD, Myburgh KH. African runners exhibit greater fatigue resistance, lower lactate accumulation, and higher oxidative enzyme activity. Journal of Applied Physiology 1999 March;86(3):915-23. Acute study
Ref ID: 2042

(5205) Westrup B, Hellström WL, Stjernqvist K, Lagercrantz H. No indications of increased quiet sleep in infants receiving care based on the newborn individualized developmental care and assessment program (NIDCAP). Acta Paediatrica 2002;91:318-22. Subjects less than 2 years old
Ref ID: 5078

(5206) Wetter LA, Payne JH, Kirshenbaum G, Podoll EF, Bachinsky T, Way LW. The ultrasonic dissector facilitates laparoscopic cholecystectomy. Archives of Surgery 1198 September;127(10):1195-8. Off topic
Ref ID: 2283

(5207) Weyand PG, Smith BR, Puyau MR, Butte NF. The mass-specific energy cost of human walking is set by stature. Journal of Experimental Biology 2010 December 1;213(Pt:23):23-9. Off topic
Ref ID: 2854

(5208) Whaley SE, McGregor S, Jiang L, Gomez J, Harrison G, Jenks E. A WIC-based intervention to prevent early childhood overweight. Journal of Nutrition Education and Behavior 2010 May;42(3:Suppl):Suppl-51. Longitudinal Study
Ref ID: 547

(5209) Whipple J. The effect of parent training in music and multimodal stimulation on parent-neonate interactions in the neonatal intensive care unit. Journal of Music Therapy 2000;37:250-68. Off topic
Ref ID: 5079

(5210) Whisenhunt BL, Williamson DA, Drab-Hudson DL, Walden H. Intervening with coaches to promote awareness and prevention of weight pressures in cheerleaders. Eating And Weight Disorders 2008 June;13(2):102-10. Off topic
Ref ID: 922

(5211) White-Traut RC, Nelson MN, Silvestri JM, Patel M, Vasan U, Han BK, Cunningham N, Burns K, Kopischke K, Bradford L. Developmental intervention for preterm infants diagnosed with periventricular leukomalacia. Research in Nursing and Health 1999;22:131-43. Subjects less than 2 years old
Ref ID: 5080

(5212) White-Traut RC, Nelson MN, Silvestri JM, Patel M, Berbaum M, Gu GG, Rey PM. Developmental patterns of physiological response to a multisensory intervention in extremely premature and high-risk infants. Journal of Obstetric, Gynecologic, and Neonatal Nursing 2004;33:266-75. Subjects less than 2 years old
Ref ID: 5081

(5213) White A, Pollack LA, Smith JL, Thompson T, Underwood JM, Fairley T. Racial and ethnic differences in health status and health behavior among breast cancer survivors--Behavioral Risk Factor Surveillance System, 2009. Journal of Cancer Survivorship 2013 March;7(1):93-103. Inappropriate Study Design
Ref ID: 6132

(5214) White KM, Lyle RM, Flynn MG, Teegarden D, Donkin SS. The acute effects of dairy calcium intake on fat metabolism during exercise and endurance exercise performance. International Journal of Sport Nutrition and Exercise Metabolism 2006 December;16(6):565-79. Diet Intervention or Supplement Study
Ref ID: 1302

(5215) White KM, Bauer SJ, Hartz KK, Baldridge M. Changes in body composition with yogurt consumption during resistance training in women. International Journal of Sport Nutrition and Exercise Metabolism 2009 February;19(1):18-33. Diet

Intervention or Supplement Study
Ref ID: 761

(5216) White MA, Martin PD, Newton RL, Walden HM, York-Crowe EE, Gordon ST, Ryan DH, Williamson DA. Mediators of weight loss in a family-based intervention presented over the internet. Obesity Research 2004 July;12(7):1050-9. No exercise only group
Ref ID: 326

(5217) White R, Agouris I, Selbie RD, Kirkpatrick M. The variability of force platform data in normal and cerebral palsy gait. Clinical biomechanics 1999;14:185-92. Off topic
Ref ID: 901

(5218) White VO, Gardner JM. Presence of anorexia nervosa and bulimia nervosa in Jamaica. West Indian Medical Journal 2002 March;51(1):32-4. Off topic
Ref ID: 737

(5219) Whitehead JR, Eklund RC, Williams AC. Using skinfold calipers while teaching body fatness-related concepts: cognitive and affective outcomes. Journal of Science and Medicine in Sport 2003 December;6(4):461-76. Off topic
Ref ID: 1681

(5220) Whitlock EP, O'Connor EA, Williams SB, Beil TL, Lutz KW. Effectiveness of weight management interventions in children: A targeted systematic review for the USPSTF. Pediatrics 2010;125(2):e396-e418. Review article
Ref ID: 3332

(5221) Whitman B, Carrel A, Bekx T, Weber C, Allen D, Myers S. Growth hormone improves body composition and motor development in infants with Prader-Willi syndrome after six months. Journal of Pediatric Endocrinology and Metabolism 2004 April;17(4):591-600. Not an exercise intervention study
Ref ID: 329

(5222) Whitman BY, Myers S, Carrel A, Allen D. A treatment/control group study of growth hormone treatment: Impact on behavior - A preliminary look. Endocrinologist 2000;10:31S-7S. Drug intervention study
Ref ID: 5082

(5223) Whitman BY, Myers S, Carrel A, Allen D. The behavioral impact of growth hormone treatment for children and adolescents with Prader-Willi syndrome: a 2-year, controlled study. Pediatrics 2002 February;109(2):E35. Not an exercise intervention study
Ref ID: 384

(5224) Whitt-Glover MC, Ham SA, Yancey AK. Instant Recess[REGISTERED]: a practical tool for increasing physical activity during the school day. Progress in Community Health Partnerships 2011;5(3):289-97. Not All Participants were

Overweight and/or Obese
Ref ID: 2855

(5225) Whittemore R, Jeon S, Grey M. An internet obesity prevention program for adolescents. Journal of Adolescent Health 2013 April;52(4):439-47. Inappropriate Intervention
Ref ID: 6133

(5226) Whittemore R, Melkus GD, Grey N. Self-report of depressed mood and depression in women with type 2 diabetes. Issues in Mental Health Nursing 2004 April;25(3):243-60. Study limited to adults
Ref ID: 3781

(5227) WHO Multicentre Growth Reference Study Group. Relationship between physical growth and motor development in the WHO Child Growth Standards. Acta Paediatrica Supplement 2006 April;450:96-101. Longitudinal Study
Ref ID: 1379

(5228) Whyte LJ, Gill JM, Cathcart AJ. Effect of 2 weeks of sprint interval training on health-related outcomes in sedentary overweight/obese men. Metabolism: Clinical and Experimental 2010 October;59(10):1421-8. Study less than 4 weeks
Ref ID: 2856

(5229) Wickham EP, Stern M, Evans RK, Bryan DL, Moskowitz WB, Clore JN, Laver JH. Prevalence of the metabolic syndrome among obese adolescents enrolled in a multidisciplinary weight management program: clinical correlates and response to treatment. Metabolic Syndrome and Related Disorders 2009 June;7(3):179-86. Lifestyle Intervention
Ref ID: 750

(5230) Wiebe PN, Blimkie CJ, Farpour-Lambert N, Briody J, Marsh D, Kemp A, Cowell C, Howman-Giles R. Effects of single-leg drop-landing exercise from different heights on skeletal adaptations in prepubertal girls: a randomized controlled study. Pediatric Exercise Science 2008 May;20(2):211-28. Not All Participants were Overweight and/or Obese
Ref ID: 928

(5231) Wiegman A, Hutten BA, de GE, Rodenburg J, Bakker HD, Buller HR, Sijbrands EJ, Kastelein JJ. Efficacy and safety of statin therapy in children with familial hypercholesterolemia: a randomized controlled trial. Journal of the American Medical Association 2004 July 21;292(3):331-7. Drug intervention study
Ref ID: 1605

(5232) Wijesuriya M, Gulliford M, Vasantharajah L, Viberti G, Gnudi L, Karalliedde J. DIABRISK-SL prevention of cardio-metabolic disease with life style modification in young urban Sri Lankan's--study protocol for a randomized controlled trial.

Trials 2011;12:209. Description versus conduct of study
Ref ID: 5084

(5233) Wiksten-Almstrã–Mer Mari, Hirschberg Aln, Hagenfeldt Kers. Reduced bone mineral density in adult women diagnosed with menstrual disorders during adolescence. Acta Obstetricia et Gynecologica Scandinavica 2009 May;88(5):543-9. Study limited to adults
Ref ID: 3782

(5234) Wilder N, Gilders R, Hagerman F, Deivert RG. The effects of a 10-week, periodized, off-season resistance-training program and creatine supplementation among collegiate football players. Journal of Strength and Conditioning Research 2002 August;16(3):343-52. Diet Intervention or Supplement Study
Ref ID: 1804

(5235) Wilk B, Bar OO. Effect of drink flavor and NaCl on voluntary drinking and hydration in boys exercising in the heat. Journal of Applied Physiology 1996;80:1112-7. Diet Intervention or Supplement Study
Ref ID: 5085

(5236) Wilk B, Timmons BW, Bar-Or O. Voluntary fluid intake, hydration status, and aerobic performance of adolescent athletes in the heat. Applied Physiology, Nutrition, and Metabolism = Physiologie Appliquee, Nutrition et Metabolisme 2010 December;35(6):834-41. Diet Intervention or Supplement Study
Ref ID: 2857

(5237) Wilk KE, Andrews JR, Arrigo CA, Keirns MA, Erber DJ. The strength characteristics of internal and external rotator muscles in professional baseball pitchers. American Journal of Sports Medicine 1993 January;21(1):61-6. Off topic
Ref ID: 2273

(5238) Wilkinson PW, Parkin JM, Pearlson G, Strong M, Sykes P. Energy intake and physical activity in obese children. British Medical Journal 1977 March 19;1(6063):756. Cross-sectional study
Ref ID: 2385

(5239) Wilks DC, Sharp SJ, Ekelund U, Thompson SG, Mander AP, Turner RM, Jebb SA, Lindroos AK. Objectively measured physical activity and fat mass in children: A bias-adjusted meta-analysis of prospective studies. PLoS ONE 2011;6(2). Review article
Ref ID: 5688

(5240) Willardson JM, Bressel E. Predicting a 10 repetition maximum for the free weight parallel squat using the 45 degrees angled leg press. Journal of Strength and Conditioning Research 2004 August;18(3):567-71. Off topic
Ref ID: 1598

(5241) Willeboordse M, van de Kant KD, de Laat MN, van Schayck OC, Mulkens S, Dompeling E. Multifactorial intervention for children with asthma and overweight (Mikado): study design of a randomised controlled trial. BMC Public Health 2013;13:494. Inappropriate Study Design
Ref ID: 6134

(5242) Williams AG. Effects of basic training in the British Army on regular and reserve army personnel. Journal of Strength and Conditioning Research 2005 May;19(2):254-9. Study limited to adults
Ref ID: 1515

(5243) Williams AJ, Henley WE, Williams CA, Hurst AJ, Logan S, Wyatt KM. Systematic review and meta-analysis of the association between childhood overweight and obesity and primary school diet and physical activity policies. International Journal of Behavioral Nutrition and Physical Activity 2013;10:101. Inappropriate Study Design
Ref ID: 6135

(5244) Williams CL, Carter BJ, Arnold CB, Wynder EL. Chronic disease risk factors among children. The 'Know Your Body' study. Journal of Chronic Diseases 1979;32(7):505-13. Not a randomized controlled trial (RCT)
Ref ID: 2382

(5245) Williams CL, Bollella MC, Strobino BA, Spark A, Nicklas TA, Tolosi LB, Pittman BP. "Healthy-start": outcome of an intervention to promote a heart healthy diet in preschool children. Journal of the American College of Nutrition 2002;21:62-71. Diet Intervention Study
Ref ID: 5086

(5246) Williams G, Clark R, Schache A, Fini NA, Moore L, Morris ME, McCrory PR. Training conditions influence walking kinematics and self-selected walking speed in patients with neurological impairments. Journal of Neurotrauma 2011 February;28(2):281-7. Off topic
Ref ID: 2858

(5247) Williams NA, Coday M, Somes G, Tylavsky FA, Richey PA, Hare M. Risk factors for poor attendance in a family-based pediatric obesity intervention program for young children. Journal of Developmental and Behavioral Pediatrics 2010 November;31(9):705-12. Not an exercise intervention study, Primary outcome(s) not assessed
Ref ID: 12

(5248) Williamson DA, Copeland AL, Anton SD, Champagne C, Han H, Lewis L, Martin C, Newton RL, Jr., Sothern M, Stewart T, Ryan D. Wise Mind project: A school-based environmental approach for preventing weight gain in children. Obesity (Silver Spring) 2007 April;15(4):906-17. No exercise only group
Ref ID: 242

(5249) Williamson DA, Champagne CM, Harsha DW, Han H, Martin CK, Newton RL, Jr., Sothern MS, Stewart TM, Webber LS, Ryan DH. Effect of an environmental school-based obesity prevention program on changes in body fat and body weight: A randomized trial. Obesity (Silver Spring) 2012 August;20(8):1653-61. Inappropriate Intervention
Ref ID: 6136

(5250) Williford HN, Blessing DL, Duey WJ, Barksdale JM, Wang N, Olson MS, Teel S. Exercise training in black adolescents: changes in blood lipids and Vo2max. Ethnicity and Disease 1996;6:279-85. Not All Participants were Overweight and/or Obese
Ref ID: 976

(5251) Willoughby DS. Effects of heavy resistance training on myostatin mRNA and protein expression. Medicine and Science in Sports and Exercise 2004 April;36(4):574-82. Study limited to adults
Ref ID: 1653

(5252) Willoughby DS, Stout JR, Wilborn CD. Effects of resistance training and protein plus amino acid supplementation on muscle anabolism, mass, and strength. Amino Acids 2007;32(4):467-77. Diet Intervention or Supplement Study
Ref ID: 1231

(5253) Willoughby KL, Dodd KJ, Shields N, Foley S. Efficacy of partial body weight-supported treadmill training compared with overground walking practice for children with cerebral palsy: A randomized controlled trial. Archives of Physical Medicine and Rehabilitation 2010 March;91(3):333-9. Off topic
Ref ID: 569

(5254) Willschke H, Marhofer P, Bösenberg A, Johnston S, Wanzel O, Sitzwohl C, Kettner S, Kapral S. Epidural catheter placement in children: Comparing a novel approach using ultrasound guidance and a standard loss-of-resistance technique. British Journal of Anaesthesia 2006;97:200-7. Off topic
Ref ID: 5087

(5255) Wilmore JH, Despres JP, Stanforth PR, Mandel S, Rice T, Gagnon J, Leon AS, Rao D, Skinner JS, Bouchard C. Alterations in body weight and composition consequent to 20 wk of endurance training: The HERITAGE Family Study. American Journal of Clinical Nutrition 1999 September;70(3):346-52. Study limited to adults
Ref ID: 2005

(5256) Wilmore JH, Green JS, Stanforth PR, Gagnon J, Rankinen T, Leon AS, Rao DC, Skinner JS, Bouchard C. Relationship of changes in maximal and submaximal aerobic fitness to changes in cardiovascular disease and non-insulin-dependent diabetes mellitus risk factors with endurance training: the HERITAGE Family Study. Metabolism: Clinical and Experimental 2001

November;50(11):1255-63. Study limited to adults
Ref ID: 1883

(5257) Wilmot EG, Davies MJ, Edwardson CL, Gorely T, Khunti K, Nimmo M, Yates T, Biddle SJ. Rationale and study design for a randomised controlled trial to reduce sedentary time in adults at risk of type 2 diabetes mellitus: Project stand (Sedentary Time ANd diabetes). BMC Public Health 2011;11:908. Description versus conduct of study
Ref ID: 2859

(5258) Wilson AJ, Prapavessis H, Jung ME, Cramp AG, Vascotto J, Lenhardt L, Shoemaker JK, Watson M, Robinson T, Clarson CL. Lifestyle modification and metformin as long-term treatment options for obese adolescents: study protocol. BMC Public Health 2009 November 30;9:434.:434. No comparative control group
Ref ID: 77

(5259) Wilson AJ, Jung ME, Cramp A, Simatovic J, Prapavessis H, Clarson C. Effects of a group-based exercise and self-regulatory intervention on obese adolescents' physical activity, social cognitions, body composition and strength: a randomized feasibility study. Journal of Health Psychology 2012 November;17(8):1223-37. Inappropriate Comparison Group
Ref ID: 6137

(5260) Wilson CJ, Robbins LJ, Murphy JM, Chang AB. Is a longer time interval between recombinant human deoxyribonuclease (dornase alfa) and chest physiotherapy better? A multi-center, randomized crossover trial. Pediatric Pulmonology 2007;42:1110-6. Drug intervention study
Ref ID: 5088

(5261) Wilson C, Gilliland S, Cullen T, Moore K, Roubideaux Y, Valdez L, Vanderwagen W, Acton K. Diabetes outcomes in the Indian health system during the era of the special diabetes program for Indians and the Government Performance and Results Act. American Journal of Public Health 2005 September;95(9):1518-22. Cross-sectional study
Ref ID: 3878

(5262) Wilson DK, Trumpeter NN, St George SM, Coulon SM, Griffin S, Lee Van HM, Lawman HG, Wandersman A, Egan B, Forthofer M, Goodlett BD, Kitzman-Ulrich H, Gadson B. An overview of the "Positive Action for Today's Health" (PATH) trial for increasing walking in low income, ethnic minority communities. Contemporary Clinical Trials 2010 November;31(6):624-33. Study not limited to children and adolescents
Ref ID: 24

(5263) Wilson DK, Lawman HG, Segal M, Chappell S. Neighborhood and parental supports for physical activity in minority adolescents. American Journal of

Preventive Medicine 2011 October;41(4):399-406. Review article
Ref ID: 1088

(5264) Wilson DM, Wang Y, Cullen KW, Baranowski T, Himes JH, Gross M, McClanahan BS, Robinson TN. Assessing weight-related biochemical cardiovascular risk factors in African-American girls. Obesity Research 2004 September;12:Suppl-83S. Cross-sectional study
Ref ID: 1576

(5265) Wilson DM, Abrams SH, Aye T, Lee PDK, Lenders C, Lustig RH, Osganian SV, Feldman HA, Fechner P, Robinson T, Buckingham B, Esrey T, McNeil K, Sorensen B, Wilson K, Davis J, Klish W, Abrams S, Holt P, Edwards C, Howard L, Gitelman S, Wertz M, Breland J, Lihatsh T et al. Metformin extended release treatment of adolescent obesity: A 48-week randomized, double-blind, placebo-controlled trial with 48-week follow-up. Archives of Pediatric Adolescent Medicine 2010;164(2):116-23. Drug intervention study
Ref ID: 3333

(5266) Wilson RW, Snyder AC, Dorman JC. Analysis of seated and standing triple Wingate tests. Journal of Strength and Conditioning Research 2009 May;23(3):868-73. Off topic
Ref ID: 759

(5267) Wiltheiss GA, Lovelady CA, West DG, Brouwer RJ, Krause KM, Ostbye T. Diet quality and weight change among overweight and obese postpartum women enrolled in a behavioral intervention program. Journal of the Academy of Nutrition and Dietetics 2013 January;113(1):54-62. Inappropriate Population
Ref ID: 6138

(5268) Winchester P, Smith P, Foreman N, Mosby JM, Pacheco F, Querry R, Tansey K. A prediction model for determining over ground walking speed after locomotor training in persons with motor incomplete spinal cord injury. Journal of Spinal Cord Medicine 2009;32(1):63-71. Off topic
Ref ID: 790

(5269) Wing YK, Hui SH, Pak WM, Ho CK, Cheung A, Li AM, Fok TF. A controlled study of sleep related disordered breathing in obese children. Archives of Disease in Childhood 2003;88:1043-7. Off topic
Ref ID: 5089

(5270) Wingen AM, Mehls O. Nutrition in children with preterminal chronic renal failure. Myth or important therapeutic aid? Pediatric Nephrology 2002 February;17(2):111. Off topic
Ref ID: 470

(5271) Winking J, Gurven M, Kaplan H. Father death and adult success among the Tsimane: implications for marriage and divorce. Evolution and Human Behavior

2011;32(2):79-89. Off topic
Ref ID: 5689

(5272) Winkler S, Pico C, Ahrens W. Physiological mechanisms in the development of adiposity. Bundesgesundheitsblatt-Gesundheitsforschung-Gesundheitsschutz 2010;53(7):681-9. Review article
Ref ID: 5690

(5273) Winner P, Rothner AD, Putnam DG, Asgharnejad M. Demographic and migraine characteristics of adolescents with migraine: Glaxo Wellcome clinical trials' database. Headache 2003;43(5):451-7. Drug intervention study
Ref ID: 3334

(5274) Winner P. Overview of pediatric headache. Current Treatment Options in Neurology 2004;6(6):471-87. Off topic
Ref ID: 3335

(5275) Winzenberg T, Shaw K, Fryer J, Jones G. Calcium supplements in healthy children do not affect weight gain, height, or body composition. Obesity 2007;15(7):1789-98. Diet Intervention or Supplement Study
Ref ID: 5691

(5276) Wirz M, Zemon DH, Rupp R, Scheel A, Colombo G, Dietz V, Hornby TG. Effectiveness of automated locomotor training in patients with chronic incomplete spinal cord injury: a multicenter trial. Archives of Physical Medicine and Rehabilitation 2005 April;86(4):672-80. Case-Control / Case Study
Ref ID: 1526

(5277) Withagen A, Kappers AML, Vervloed MPJ, Knoors H, Verhoeven L. Haptic object matching by blind and sighted adults and children. Acta Psychologica 2012;139(2):261-71. Off topic
Ref ID: 5692

(5278) Withers RT, LaForgia J, Pillans RK, Shipp NJ, Chatterton BE, Schultz CG, Leaney F. Comparisons of two-, three-, and four-compartment models of body composition analysis in men and women. Journal of Applied Physiology 1998 July;85(1):238-45. Off topic
Ref ID: 2087

(5279) Wojtyla A, Kapka-Skrzypczak L, Bilinski P, Paprzycki P. Physical activity among women at reproductive age and during pregnancy (Youth Behavioural Polish Survey - YBPS and Pregnancy-related Assessment Monitoring Survay - PrAMS) - epidemiological population studies in Poland during the period 2010-2011. Annals of Agricultural and Environmental Medicine 2011;18(2):365-74. Survey or questionnaire
Ref ID: 5693

(5280) Wolfe S. The nutritional management of cystic fibrosis. Current medical literature: Pediatrics 2003 May;16(1):31. Diet Intervention Study
Ref ID: 3783

(5281) Wolin KY, Ruiz JR, Tuchman H, Lucia A. Exercise in adult and pediatric hematological cancer survivors: An intervention review. [Review] [76 refs]. Leukemia 2010 June;24(6):1113-20. Review article
Ref ID: 516

(5282) Wolowczuk I, Verwaerde C, Viltart O, Delanoye A, Delacre M, Pot B, Grangette C. Feeding our immune system: Impact on metabolism. Clinical and Developmental Immunology 2008 January;1-19. Review article
Ref ID: 3784

(5283) Wolter JM, Bowler SD, Nolan PJ, McCormack JG. Home intravenous therapy in cystic fibrosis: a prospective randomized trial examining clinical, quality of life and cost aspects. European Respiratory Journal 1997 April;10(4):896-900. Drug intervention study
Ref ID: 2141

(5284) Wolthers OD, Heuck C. Short-term treatment with prednisolone has no lasting effect on serum leptin. Scandinavian Journal of Clinical and Laboratory Investigation 2002 June;62(4):321-4. Drug intervention study
Ref ID: 3785

(5285) Wong SC, Smyth A, McNeill E, Galloway PJ, Hassan K, McGrogan P, Ahmed SF. The growth hormone insulin-like growth factor 1 axis in children and adolescents with inflammatory bowel disease and growth retardation. Clinical Endocrinology 2010 August;73(2):220-8. Retrospective study
Ref ID: 3786

(5286) Wong WW. Energy-expenditure of female adolescents. Journal of the American College of Nutrition 1994;13(4):332-7. Off topic
Ref ID: 5694

(5287) Wong WW, Nicolson M, Stuff JE, Butte NF, Ellis KJ, Hergenroeder AC, Hill RB, Smith EO. Serum leptin concentrations in Caucasian and African-American girls. Journal of Clinical Endocrinology and Metabolism 1998;83(10):3574-7. Cross-sectional study
Ref ID: 5695

(5288) Woo J, Sea MM, Tong P, Ko GT, Lee Z, Chan J, Chow FC. Effectiveness of a lifestyle modification programme in weight maintenance in obese subjects after cessation of treatment with Orlistat. Journal of Evaluation in Clinical Practice 2007 December;13(6):853-9. No exercise only group
Ref ID: 209

(5289) Woo KS, Chook P, Yu CW, Sung RY, Qiao M, Leung SS, Lam CW, Metreweli C, Celermajer DS. Effects of diet and exercise on obesity-related vascular dysfunction in children. Circulation 2004 April 27;109(16):1981-6. No exercise only group, No comparative control group
Ref ID: 336

(5290) Wood LG, Fitzgerald DA, Gibson PG, Cooper DM, Garg ML. Increased plasma fatty acid concentrations after respiratory exacerbations are associated with elevated oxidative stress in cystic fibrosis patients.[Erratum appears in American Journal of Clinical Nutrition 2002 Oct;76(4):907]. American Journal of Clinical Nutrition 75(4):668-75, 2002 Apr 2002;(4):668-75. Off topic
Ref ID: 3184

(5291) Wood PD. Impact of experimental manipulation of energy intake and expenditure on body composition. [Review] [12 refs]. Critical Reviews in Food Science and Nutrition 1993;33(4-5):369-73. Review article
Ref ID: 2257

(5292) Woodcock KA, Oliver C, Humphreys GW. The relationship between a deficit in attention switching and specific behaviours in Prader-Willi syndrome. Journal of Intellectual Disability Research 2008 October;52(10):812. Off topic
Ref ID: 3879

(5293) Woodhouse CRJ. Myelomeningocele: Neglected aspects. Pediatric Nephrology 2008;23(8):1223-31. Off topic
Ref ID: 3336

(5294) Woodhouse LJ, Asa SL, Thomas SG, Ezzat S. Measures of submaximal aerobic performance evaluate and predict functional response to growth hormone (GH) treatment in GH-deficient adults. Journal of Clinical Endocrinology and Metabolism 1999 December;84(12):4570-7. Drug intervention study
Ref ID: 2058

(5295) Woolf K, Bidwell WK, Carlson AG. The effect of caffeine as an ergogenic aid in anaerobic exercise. International Journal of Sport Nutrition and Exercise Metabolism 2008 August;18(4):412-29. Diet Intervention or Supplement Study
Ref ID: 898

(5296) Woolf K, Bidwell WK, Carlson AG. Effect of caffeine as an ergogenic aid during anaerobic exercise performance in caffeine naive collegiate football players. Journal of Strength and Conditioning Research 2009 August;23(5):1363-9. Diet Intervention or Supplement Study
Ref ID: 702

(5297) Woolf PJ, Fu LL, Basu A. vProtein: Identifying optimal amino acid complements from plant-based foods. PLoS ONE 2011;6(4). Diet Intervention Study
Ref ID: 5696

(5298) Woollett LA, Wang Y, Buckley DD, Yao L, Chin S, Granholm N, Jones PJH, Setchell KDR, Tso P, Heubi JE. Micellar solubilisation of cholesterol is essential for absorption in humans. Gut 2006;55(2):197-204. Off topic
Ref ID: 5697

(5299) Wooten JS, Biggerstaff KD, Ben-Ezra V. A single 1-h session of moderate-intensity aerobic exercise does not modify lipids and lipoproteins in normolipidemic obese women. Applied Physiology, Nutrition, and Metabolism = Physiologie Appliquee, Nutrition et Metabolisme 2011 October;36(5):715-22. Acute study
Ref ID: 2862

(5300) Wootton PT, Flavell DM, Montgomery HE, World M, Humphries SE, Talmud PJ. Lipoprotein-associated phospholipase A2 A379V variant is associated with body composition changes in response to exercise training. Nutrition, Metabolism and Cardiovascular Diseases 2007 January;17(1):24-31. Not a randomized controlled trial (RCT)
Ref ID: 256

(5301) Worobey J, Vetrini NR, Rozo EM. Mechanical measurement of infant activity: A cautionary note. Infant Behavior and Development 2009;32(2):167-72. Subjects less than 2 years old
Ref ID: 5698

(5302) Wortman J. Health promotion when the 'vaccine' does not work. [Review] [34 refs]. Health Promotion Journal of Australia 2006 August;17(2):91-6. Review article
Ref ID: 1357

(5303) Wraith JE, Vecchio D, Jacklin E, Abel L, Chadha-Boreham H, Luzy C, Giorgino R, Patterson MC. Miglustat in adult and juvenile patients with Niemann-Pick disease type C: long-term data from a clinical trial. Molecular Genetics and Metabolism 2010 April;99(4):351-7. Drug intervention study
Ref ID: 571

(5304) Wright HE, Selkirk GA, McLellan TM. HPA and SAS responses to increasing core temperature during uncompensable exertional heat stress in trained and untrained males. European Journal of Applied Physiology 2010 March;108(5):987-97. Off topic
Ref ID: 578

(5305) Wright JA, Phillips BD, Watson BL, Newby PK, Norman GJ, Adams WG. Randomized trial of a family-based, automated, conversational obesity treatment program for underserved populations. Obesity (Silver Spring) 2013 September;21(9):E369-E378. Inappropriate Intervention
Ref ID: 6139

(5306) Wright K, Norris K, Newman GJ, Suro Z. Improving healthy dietary behaviors, nutrition knowledge, and self-efficacy among underserved school children with parent and community involvement. Childhood Obesity 2012 August;8(4):347-56. Inappropriate Intervention
Ref ID: 6140

(5307) Wright K, Giger JN, Norris K, Suro Z. Impact of a nurse-directed, coordinated school health program to enhance physical activity behaviors and reduce body mass index among minority children: A parallel-group, randomized control trial. Int J Nurs Stud 2013 June;50(6):727-37. Inappropriate Intervention
Ref ID: 6141

(5308) Wright OR, Netzel GA, Sakzewski AR. A randomized, double-blind, placebo-controlled trial of the effect of dried purple carrot on body mass, lipids, blood pressure, body composition, and inflammatory markers in overweight and obese adults: the QUENCH trial. Canadian Journal of Physiology and Pharmacology 2013 June;91(6):480-8. Inappropriate Intervention
Ref ID: 6142

(5309) Wu MY, Bowtell JL, Williams CA. Glycaemic index of meals affects appetite sensation but not energy balance in active males. European Journal of Nutrition 2014 February;53(1):309-19. Inappropriate Intervention
Ref ID: 6143

(5310) Wyatt KM, Lloyd JJ, Abraham C, Creanor S, Dean S, Densham E, Daurge W, Green C, Hillsdon M, Pearson V, Taylor RS, Tomlinson R, Logan S. The Healthy Lifestyles Programme (HeLP), a novel school-based intervention to prevent obesity in school children: study protocol for a randomised controlled trial. Trials 2013;14:95. Inappropriate Study Design
Ref ID: 6144

(5311) Wynne K, Park AJ, Small CJ, Patterson M, Ellis SM, Murphy KG, Wren AM, Frost GS, Meeran K, Ghatei MA, Bloom SR. Subcutaneous oxyntomodulin reduces body weight in overweight and obese subjects: a double-blind, randomized, controlled trial. Diabetes 2005 August;54(8):2390-5. Not an exercise intervention study
Ref ID: 306

(5312) Xavier RM, Xavier MM, Cartafina RA, Magalhães FO, Nunes AA, Santos VMd. Prevalência de hipertensão arterial em escolares vinculados à universidadede uberaba (UNIUBE). Brasília Médica 2007;44(3):169-72. Cross-sectional study
Ref ID: 4639

(5313) Xu F, Wang X, Ware RS, Tse LA, Wang Z, Hong X, Chan EY, Li J, Wang Y. A school-based comprehensive lifestyle intervention among Chinese kids against Obesity (CLICK-Obesity) in Nanjing City, China: the baseline data. Asia Pacific

Journal of Clinical Nutrition 2014;23(1):48-54. Inappropriate Intervention
Ref ID: 6145

(5314) Xu KS, Yan TB, Mai JN. [Effects of botulinum toxin guided by electric stimulation on spasticity in ankle plantar flexor of children with cerebral palsy: a randomized trial]. [Chinese]. Zhonghua Erke Zazhi 2006 December;44(12):913-7. Drug intervention study
Ref ID: 1306

(5315) Xu L, Dubois L, Burnier D, Girard M, Prud'homme D. Parental overweight/obesity, social factors, and child overweight/obesity at 7 years of age. Pediatrics International 2011;53(6):826-31. Cohort Study
Ref ID: 5699

(5316) Xu X, Pennell ML, Lu B, Murray DM. Efficient Bayesian joint models for group randomized trials with multiple observation times and multiple outcomes. Statistics in Medicine 2012 October 30;31(24):2858-71. Inappropriate Outcomes
Ref ID: 6146

(5317) Xue FS, Luo LK, Tong SY, Liao X, Deng XM, An G. Study of the safe threshold of apneic period in children during anesthesia induction. Journal of Clinical Anesthesia 1996;8:568-74. Drug intervention study
Ref ID: 987

(5318) Xujun M, Ezzeldin HH, Diasio RB. Histone Deacetylase Inhibitors. Drugs 2009 October;69(14):1911-34. Review article
Ref ID: 3787

(5319) Yack HJ, Riley LM, Whieldon TR. Anterior tibial translation during progressive loading of the ACL-deficient knee during weight-bearing and nonweight-bearing isometric exercise. Journal of Orthopaedic and Sports Physical Therapy 1994 November;20(5):247-53. Off topic
Ref ID: 2224

(5320) Yackobovitch-Gavan M, Nagelberg N, Phillip M, Ashkenazi-Hoffnung L, Hershkovitz E, Shalitin S. The influence of diet and/or exercise and parental compliance on health-related quality of life in obese children. Nutrition Research 2009 June;29(6):397-404. No comparative control group
Ref ID: 103

(5321) Yalçin SS, Yurdakök K, Tezcan I, Oner L. Effect of glutamine supplementation on diarrhea, interleukin-8 and secretory immunoglobulin A in children with acute diarrhea. Journal of Pediatric Gastroenterology and Nutrition 2004;38:494-501. Off topic
Ref ID: 5093

(5322) Yanagiya T, Kanehisa H, Kouzaki M, Kawakami Y, Fukunaga T. Effect of gender on mechanical power output during repeated bouts of maximal running in trained teenagers. International Journal of Sports Medicine 2003 May;24(4):304-10. Acute study
Ref ID: 1742

(5323) Yancey AK, McCarthy WJ, Taylor WC, Merlo A, Gewa C, Weber MD, Fielding JE. The Los Angeles Lift Off: a sociocultural environmental change intervention to integrate physical activity into the workplace. Preventive Medicine 2004 June;38(6):848-56. Study limited to adults
Ref ID: 330

(5324) Yancy WS, Jr., Olsen MK, Guyton JR, Bakst RP, Westman EC. A low-carbohydrate, ketogenic diet versus a low-fat diet to treat obesity and hyperlipidemia: a randomized, controlled trial. Annals of Internal Medicine 2004 May 18;140(10):769-77. Study not limited to children and adolescents
Ref ID: 331

(5325) Yang JJ, Xing HJ, Xiao HL, Li Q, Li M, Wang SJ. [Effects of acupuncture combined with diet adjustment and aerobic exercise on weight and waist-hip ratio in simple obesity patients]. Zhongguo Zhen Jiu 2010 July;30(7):555-8. No comparative control group, No exercise only group
Ref ID: 22

(5326) Yang JJ, Xing HJ, Wang SJ, Xiao HL, Li M, Li Q. [Effects of acupuncture combined with dietary adjustments and aerobic exercise on body weight, body mass index and serum leptin level in simple obesity patients]. Zhen Ci Yan Jiu 2010 December;35(6):453-7. No exercise only group, No comparative control group
Ref ID: 1

(5327) Yang S, Smith B, Graham G. Healthy Video Gaming: Oxymoron or Possibility? Innovate: Journal of Online Education 2008 April 1;4(4). Survey or questionnaire
Ref ID: 3918

(5328) Yang VK, Freeman LM, Rush JE. Comparisons of morphometric measurements and serum insulin-like growth factor concentration in healthy cats and cats with hypertrophic cardiomyopathy. American Journal of Veterinary Research 2008;69(8):1061-6. Animal study
Ref ID: 5700

(5329) Yaniv M, Becker T, Goldwirt M, Khamis S, Steinberg DM, Weintroub S. Prevalence of bowlegs among child and adolescent soccer players. Clinical Journal of Sport Medicine 2006;16(5):392-6. Cross-sectional study
Ref ID: 5701

(5330) Yanovski JA. How effective is sibutramine for the treatment of overweight adolescents? Nature Clinical Practice Endocrinology and Metabolism 2007;3(2):82-3. Drug intervention study
Ref ID: 3337

(5331) Yanovski SZ, Yanovski JA. Obesity. [Review] [108 refs]. New England Journal of Medicine 2002 February 21;346(8):591-602. Review article
Ref ID: 1846

(5332) Yarasheski KE, Cade WT, Overton ET, Mondy KE, Hubert S, Laciny E, Bopp C, Lassa-Claxton S, Reeds DN. Exercise training augments the peripheral insulin-sensitizing effects of pioglitazone in HIV-infected adults with insulin resistance and central adiposity. American Journal of Physiology, Endocrinology and Metabolism 2011 January;300(1):E243-E251. Study limited to adults
Ref ID: 19

(5333) Yarborough BJ, DeBar LL, Wu P, Pearson J, Stevens VJ. Responding to pediatric providers' perceived barriers to adolescent weight management. Clinical Pediatrics (Philadelphia) 2012 November;51(11):1063-70. Inappropriate Population
Ref ID: 6147

(5334) Yeomans MR, Weinberg L, James S. Effects of palatability and learned satiety on energy density influences on breakfast intake in humans. Physiology and Behavior 2005;86(4):487-99. Diet Intervention Study
Ref ID: 5702

(5335) Yeomans MR, Leitch M, Gould NJ, Mobini S. Differential hedonic, sensory and behavioral changes associated with flavor-nutrient and flavor-flavor learning. Physiology and Behavior 2008;93(4-5):798-806. Diet Intervention Study
Ref ID: 5703

(5336) Yeomans MR, Mobini S, Bertenshaw EJ, Gould NJ. Acquired liking for sweet-paired odours is related to the disinhibition but not restraint factor from the Three Factor Eating Questionnaire. Physiology and Behavior 2009;96(2):244-52. Diet Intervention Study
Ref ID: 5704

(5337) Yeomans MR. Understanding Individual Differences in Acquired Flavour Liking in Humans. Chemosensory Perception 2010;3(1):34-41. Diet Intervention Study
Ref ID: 5705

(5338) Yeomans MR. Flavour-nutrient learning in humans: An elusive phenomenon? Physiology and Behavior 2012;106(3):345-55. Diet Intervention Study
Ref ID: 5706

(5339) Yeste D, Garcia-Reyna N, Gussinyer S, Marhuenda C, Clemente M, Albisu M, Gussinyer M, Carrascosa A. Present trends in childhood and adolescent

obesity treatment. Revista Espanola de Obesidad 2008;6(3):139-52. Review article
Ref ID: 3338

(5340) Yiallouros PK, Savva SC, Kolokotroni O, Behbod B, Zeniou M, Economou M, Chadjigeorgiou C, Kourides YA, Tornaritis MJ, Lamnisos D, Middleton N, Milton DK. Low serum high-density lipoprotein cholesterol in childhood is associated with adolescent asthma. Clinical and Experimental Allergy 2012;42(3):423-32. Cross-sectional study
Ref ID: 5707

(5341) Yildirim M, Arundell L, Cerin E, Carson V, Brown H, Crawford D, Hesketh KD, Ridgers ND, te Velde SJ, Chinapaw MJ, Salmon J. What helps children to move more at school recess and lunchtime? Mid-intervention results from Transform-Us! cluster-randomised controlled trial. British Journal of Sports Medicine 2014 February;48(3):271-7. Inappropriate Intervention
Ref ID: 6148

(5342) Yilmaz Y, Younossi ZM. Obesity-associated nonalcoholic fatty liver disease. Clin Liver Dis 2014 February;18(1):19-31. Inappropriate Study Design
Ref ID: 6149

(5343) Yin FZ, Lu Q, Wang SY, Ma CM, Lou DH, Liu BW, Liu XL. The study of insulin resistance and soluble intercellular adhesion molecule-1 in normotensive adolescents with a family history of hypertension. Journal of Human Hypertension 2009;23(6):402-6. Cross-sectional study
Ref ID: 5708

(5344) Yin HS, Sanders LM, Rothman RL, Shustak R, Eden SK, Shintani A, Cerra ME, Cruzatte EF, Perrin EM. Parent health literacy and "obesogenic" feeding and physical activity-related infant care behaviors. Journal of Pediatrics 2014 March;164(3):577-83. Inappropriate Population
Ref ID: 6150

(5345) Yin J, Zhang Q, Liu A, Du W, Wang X, Hu X, Ma G. Calcium supplementation for 2 years improves bone mineral accretion and lean body mass in Chinese adolescents. Asia Pacific Journal of Clinical Nutrition 2010;19(2):152-60. Diet Intervention or Supplement Study
Ref ID: 536

(5346) Yin Z, Moore JB, Johnson MH, Barbeau P, Cavnar M, Thornburg J, Gutin B. The Medical College of Georgia Fitkid project: the relations between program attendance and changes in outcomes in year 1. International Journal of Obesity (London) 2005 September;29 Suppl 2:S40-5.:S40-S45. No exercise only group, Not All Participants were Overweight and/or Obese
Ref ID: 290

(5347) Yin Z, Gutin B, Johnson MH, Hanes J, Jr., Moore JB, Cavnar M, Thornburg J, Moore D, Barbeau P. An environmental approach to obesity prevention in children: Medical College of Georgia FitKid Project year 1 results. Obesity Research 2005 December;13(12):2153-61. No exercise only group, Not All Participants were Overweight and/or Obese
Ref ID: 289

(5348) Yin Z, Hanes J, Jr., Moore JB, Humbles P, Barbeau P, Gutin B. An after-school physical activity program for obesity prevention in children: the Medical College of Georgia FitKid Project. Evaluation and the Health Professions 2005 March;28(1):67-89. Description versus conduct of study
Ref ID: 315

(5349) Yin Z, Parra-Medina D, Cordova A, He M, Trummer V, Sosa E, Gallion KJ, Sintes-Yallen A, Huang Y, Wu X, Acosta D, Kibbe D, Ramirez A. Miranos! Look at us, we are healthy! An environmental approach to early childhood obesity prevention. Childhood Obesity 2012 October;8(5):429-39. Inappropriate Study Design
Ref ID: 6151

(5350) Yiu-ming W, Suk-tak C, Kwok-wing T, Ng GVF. Two modes of weight training programs and patellar stabilization. Journal of Athletic Training 2009 May;44(3):264-71. Study limited to adults
Ref ID: 3880

(5351) Yokota M, Berglund LG, Bathalon GP. Female anthropometric variability and their effects on predicted thermoregulatory responses to work in the heat. International Journal of Biometeorology 2012 March;56(2):379-85. Off topic
Ref ID: 2866

(5352) Yoneyama SM, Silva TLNd, Baptista JdS, Mayer WP, Paganotti MT, Costa PF. Eficiência do treino de marcha em suporte parcial de peso no equilíbrio de pacientes hemiparéticos. Revista de Medicina (Sao Paulo) 2009 June;88(2):80-6. Study limited to adults
Ref ID: 4640

(5353) Yoong SL, Wolfenden L, Finch M, Williams A, Dodds P, Gillham K, Wyse R. A randomised controlled trial of an active telephone-based recruitment strategy to increase childcare-service staff attendance at a physical activity and nutrition training workshop. Health Promotion Journal of Australia 2013 December;24(3):224-6. Inappropriate Population
Ref ID: 6152

(5354) Yoshinaga M, Shimago A, Koriyama C, Nomura Y, Miyata K, Hashiguchi J, Arima K. Rapid increase in the prevalence of obesity in elementary school children. International Journal of Obesity and Related Metabolic Disorders 2004

April;28(4):494-9. Prevalence study
Ref ID: 1657

(5355) Yoshioka M, Doucet E, St-Pierre S, Almeras N, Richard D, Labrie A, Despres JP, Bouchard C, Tremblay A. Impact of high-intensity exercise on energy expenditure, lipid oxidation and body fatness. International Journal of Obesity and Related Metabolic Disorders 2001 March;25(3):332. Study limited to adults
Ref ID: 3788

(5356) Young DR, Phillips JA, Yu T, Haythornthwaite JA. Effects of a life skills intervention for increasing physical activity in adolescent girls. Archives of Pediatrics and Adolescent Medicine 2006 December;160(12):1255-61. Not All Participants were Overweight and/or Obese
Ref ID: 1317

(5357) Young DR, Felton GM, Grieser M, Elder JP, Johnson C, Lee JS, Kubik MY. Policies and opportunities for physical activity in middle school environments. Journal of School Health 2007 January;77(1):41-7. Survey or questionnaire
Ref ID: 1287

(5358) Young JC. Meal size and frequency: effect on potentiation of the thermal effect of food by prior exercise. European Journal of Applied Physiology and Occupational Physiology 1995;70(5):437-41. Diet Intervention Study
Ref ID: 2201

(5359) Young MD, Collins CE, Callister R, Plotnikoff RC, Doran CM, Morgan PJ. The SHED-IT weight loss maintenance trial protocol: A randomised controlled trial of a weight loss maintenance program for overweight and obese men. Contemporary Clinical Trials 2014 January;37(1):84-97. Inappropriate Population
Ref ID: 6153

(5360) Young TK. Obesity, central fat patterning, and their metabolic correlates among the inuit of the central Canadian Arctic. Human Biology 1996 April;68(2):245-63. Study limited to adults
Ref ID: 2162

(5361) Yount KM, DiGirolamo AM, Ramakrishnan U. Impacts of domestic violence on child growth and nutrition: A conceptual review of the pathways of influence. Social Science and Medicine 2011;72(9):1534-54. Review article
Ref ID: 5709

(5362) Yu-Hua T, Cypess AM, Kahn CR. Cellular bioenergetics as a target for obesity therapy. Nature Reviews Drug Discovery 2010 June;9(6):465-82. Review article
Ref ID: 3789

(5363) Yu C, Zhao S, Zhao X. [Treatment of simple obesity in children with photo-acupuncture]. [Chinese]. Zhongguo Zhong Xi Yi Jie He Za Zhi Zhongguo

Zhongxiyi Jiehe Zazhi/Chinese Journal of Integrated Traditional and Western Medicine/Zhongguo Zhong Xi Yi Jie He Xue Hui, Zhongguo Zhong Yi Yan Jiu Yuan Zhu Ban 1998 June;18(6):348-50. Off topic
Ref ID: 2109

(5364) Yu CC, Sung RY, So RC, Lui KC, Lau W, Lam PK, Lau EM. Effects of strength training on body composition and bone mineral content in children who are obese. Journal of Strength and Conditioning Research 2005 August;19(3):667-72. No exercise only group, No comparative control group
Ref ID: 305

(5365) Yu CC, Sung RY, Hau KT, Lam PK, Nelson EA, So RC. The effect of diet and strength training on obese children's physical self-concept. Journal of Sports Medicine and Physical Fitness 2008 March;48(1):76-82. No exercise only group, No comparative control group
Ref ID: 203

(5366) Yu CC, Li AM, Chan KO, Chook P, Kam JT, Au CT, So RC, Sung RY, McManus AM. Orlistat improves endothelial function in obese adolescents: a randomised trial. Journal of Paediatrics and Child Health 2013 November;49(11):969-75. Inappropriate Intervention
Ref ID: 6154

(5367) Yu DK, Nordbrock E, Hutcheson SJ, Lewis EW, Sullivan W, Bhargava VO, Weir SJ. Population pharmacokinetics of teicoplanin in patients with endocarditis. Journal of Pharmacokinetics and Biopharmaceutics 1995 February;23(1):25-39. Drug intervention study
Ref ID: 2190

(5368) Yurdakök K, Ozmert E, Yalçin SS, Laleli Y. Vitamin A supplementation in acute diarrhea. Journal of Pediatric Gastroenterology and Nutrition 2000;31:234-7. Off topic
Ref ID: 5094

(5369) Zacny JP. Characterizing the subjective,psychomotor, and physiological effects of a hydrocodone combination product (Hycodan)in non-drug-abusing volunteers. Psychopharmacology 2003 January;165(2):146. Drug intervention study
Ref ID: 3790

(5370) Zadeh LA. From computing with numbers to computing with words - From manipulation of measurements to manipulation of perceptions. Ieee Transactions on Circuits and Systems I-Regular Papers 1999;46(1):105-19. Off topic
Ref ID: 5710

(5371) Zafeiridis A, Dalamitros A, Dipla K, Manou V, Galanis N, Kellis S. Recovery during high-intensity intermittent anaerobic exercise in boys, teens, and men.

Medicine and Science in Sports and Exercise 2005 March;37(3):505-12. Acute study
Ref ID: 1538

(5372) Zahner L, Puder JJ, Roth R, Schmid M, Guldimann R, Puhse U, Knopfli M, Braun-Fahrlander C, Marti B, Kriemler S. A school-based physical activity program to improve health and fitness in children aged 6-13 years ("Kinder-Sportstudie KISS"): study design of a randomized controlled trial [ISRCTN15360785]. BMC Public Health 2006;6:147. Not All Participants were Overweight and/or Obese
Ref ID: 1372

(5373) Zahner L, Muehlbauer T, Schmid M, Meyer U, Puder JJ, Kriemler S. Association of sports club participation with fitness and fatness in children. Medicine and Science in Sports and Exercise 2009 February;41(2):344-50. Cross-sectional study
Ref ID: 140

(5374) Zalilah MS, Khor GL, Mirnalini K, Norimah AK, Ang M. Dietary intake, physical activity and energy expenditure of Malaysian adolescents. Singapore Medical Journal 2006 June;47(6):491-8. Cross-sectional study
Ref ID: 1388

(5375) Zaman S, Ashraf RN, Martines J. Training in complementary feeding counselling of healthcare workers and its influence on maternal behaviours and child growth: a cluster-randomized controlled trial in Lahore, Pakistan. Journal of Health, Population, and Nutrition 2008;26:210-22. Off topic
Ref ID: 5095

(5376) Zambon MP, Antonio MÂ, Mendes RT, Filho B. Hipotireoidismo adquirido tratado como obesidade exógena: a importância do controle do crescimento. Acquired hypothyroidism treated as exogen obesity: the importance of growth follow-up. Revista Paulista de Pediatria 2009 March;27(1):106-9. Case-Control / Case Study
Ref ID: 4641

(5377) Zamora S JD, Laclé M. Evaluación del gasto energético y actividad física en escolares eutróficos, con sobrepeso u obesidad. Assessment of energy expenditure and physical activity in eutrophic, overweight or obese school students using Actihearts #8482; monitors. Revista Chilena de Pediatría 2012 April;83(2):134-45. Not All Participants were Overweight and/or Obese
Ref ID: 4642

(5378) Zamora J, Jara P, Vergara E, Garrido N. Ejercicio y actividad enzimática en jóvenes deportistas y sedentarios. Revista Chilena de Tecnología Médica 1986;9(2):458-61. Acute study
Ref ID: 4643

(5379) Zanchetta LM, Barros MBdA, César CLG, Carandina L, Goldbaum M, Alves MCGP. Inatividade física e fatores associados em adultos, São Paulo, Brasil Physical inactivity and associated factors in adults, São Paulo, Brazilien. Revista Brasileira de Eidemiologia 2010 September;13(3):387-99. Study limited to adults
Ref ID: 4644

(5380) Zanconato S, Riedy G, Cooper DM. Calf Muscle Cross-sectional area and peak oxygen-uptake and work rate in children and adults. American Journal of Physiology 1994;267(3):R720-R725. Off topic
Ref ID: 5711

(5381) Zanella AM, Nakazone MA, Pinhel MAS, Souza DRS. Lipid profile, apolipoprotein A-I and oxidative stress in professional footballers, sedentary individuals, and their relatives. Perfil lipídico, apolipoproteína A-I e estresse oxidativo em jogadores de futebol profissionais, indivíduos sedentários e seus familiares. Arquivos Brasileiros de Endocrinologia and Metabologia 2011 March;55(2):121-6. Study limited to adults
Ref ID: 4645

(5382) Zang Y, Zhao Y, Yang Q, Pan Y, Li N, Liu T. A randomised trial on pubertal development and health in China. Journal of Clinical Nursing 2011;20:3081-91. Educational intervention
Ref ID: 5096

(5383) Zapico AG, Benito PJ, Gonzalez-Gross M, Peinado AB, Morencos E, Romero B, Rojo-Tirado MA, Cupeiro R, Szendrei B, Butragueno J, Bermejo M, Alvarez-Sanchez M, Garcia-Fuentes M, Gomez-Candela C, Bermejo LM, Fernandez-Fernandez C, Calderon FJ. Nutrition and physical activity programs for obesity treatment (PRONAF study): Methodological approach of the project. BMC Public Health 2012;12:1100. Inappropriate Population
Ref ID: 6155

(5384) Zask A, Adams JK, Brooks LO, Hughes DF. Tooty Fruity Vegie: an obesity prevention intervention evaluation in Australian preschools. Health Promotion Journal of Australia 2012 April;23(1):10-5. Diet Intervention Study
Ref ID: 2867

(5385) Zatz M, Betti RT, Frota-Pessoa O. Treatment of Duchenne muscular dystrophy with growth hormone inhibitors. American Journal of Medical Genetics 1986 July;24(3):549-66. Drug intervention study
Ref ID: 2350

(5386) Zeitler P, Pinhas-Hamiel O. Prevention and screening for type 2 diabetes in youth. Endocrine Research 2007;33(1-2):73-91. Review article
Ref ID: 3339

(5387) Zelkowitz P, Feeley N, Shrier I, Stremler R, Westreich R, Dunkley D, Steele R, Rosberger Z, Lefebvre F, Papageorgiou A. The Cues and Care Trial: A randomized controlled trial of an intervention to reduce maternal anxiety and improve developmental outcomes in very low birthweight infants. BMC Pediatrics 2008;8:38. Study limited to adults
Ref ID: 5097

(5388) Zelkowitz P, Feeley N, Shrier I, Stremler R, Westreich R, Dunkley D, Steele R, Rosberger Z, Lefebvre F, Papageorgiou A. The cues and care randomized controlled trial of a neonatal intensive care unit intervention: effects on maternal psychological distress and mother-infant interaction. Journal of Developmental and Behavioral Pediatrics 2011;32:591-9. Study limited to adults
Ref ID: 5098

(5389) Zeno SA, Deuster PA, Davis JL, Kim-Dorner SJ, Remaley AT, Poth M. Diagnostic criteria for metabolic syndrome: Caucasians versus African-Americans. Metabolic Syndrome and Related Disorders 2010 April;8(2):149-56. Study limited to adults
Ref ID: 559

(5390) Zhang H, Zhang K, Mu Y, Yao N, Sclabassi RJ, Sun MG. Weight measurement using image-based pose analysis. Progress in Natural Science 2008;18(12):1507-12. Off topic
Ref ID: 5712

(5391) Zhang X, Chen K, Qu P, Liu YX, Li TY. Effect of biscuits fortified with different doses of vitamin A on indices of vitamin A status, haemoglobin and physical growth levels of pre-school children in Chongqing. Public Health Nutrition 2010;13:1462-71. Diet Intervention Study
Ref ID: 5099

(5392) Zhang XL. [Effect of behavior modification in controlling children's obesity]. Zhongguo Linchuang Kangfu 2005;9:166-7. Behavior Modification Intervention
Ref ID: 5100

(5393) Zhang Y, Cooke R. Using a combined motivational and volitional intervention to promote exercise and healthy dietary behaviour among undergraduates. Diabetes Research and Clinical Practice 2012 February;95(2):215-23. Study limited to adults
Ref ID: 2868

(5394) Zhang YX, Wang SR. Differences in development and the prevalence of obesity among children and adolescents in different socioeconomic status districts in Shandong, China. Annals of Human Biology 2012;39(4):290-6. Prevalence study
Ref ID: 5713

(5395) Zhao DC, Ji CY, Ma J, Liao WK, Zhang B, Hu PJ. [Analysis of motor performance status in students of Han nationality in fifteen provinces in China]. [Chinese]. Chung-Hua Yu Fang i Hsueh Tsa Chih [Chinese Journal of Preventive Medicine] 2005 November;39(6):385-7. Cross-sectional study
Ref ID: 1444

(5396) Zhao J, Li J, Hamer-Maansson JE, Andersson T, Fulmer R, Illueca M, Lundborg P. Pharmacokinetic properties of esomeprazole in children aged 1 to 11 years with symptoms of gastroesophageal reflux disease: a randomized, open-label study. Clinical Therapeutics 2006;28:1868-76. Drug intervention study
Ref ID: 5101

(5397) Zhao J, Xiao P, Guo Y, Liu YJ, Pei YF, Yang TL, Pan F, Chen Y, Shen H, Zhao LJ, Papasian CJ, Drees BM, Hamilton JJ, Deng HY, Recker RR, Deng HW. Bivariate genome linkage analysis suggests pleiotropic effects on chromosomes 20p and 3p for body fat mass and lean mass. Genetics Research 2008;90(3):259-68. Off topic
Ref ID: 5714

(5398) Zhu H, Luo HL, Meng H, Zhang GJ. Effect of vitamin E supplementation on development of reproductive organs in Boer goat. Animal Reproduction Science 2009;113(1-4):93-101. Animal study
Ref ID: 5715

(5399) Zhu HD, Wang XL, Gutin B, Davis CL, Keeton D, Thomas J, Stallmann-Jorgensen I, Mooken G, Bundy V, Snieder H, van der Harst P, Dong YB. Leukocyte telomere length in healthy Caucasian and African-American adolescents: Relationships with race, sex, adiposity, adipokines, and physical activity. Journal of Pediatrics 2011;158(2):215-20. Cross-sectional study
Ref ID: 5716

(5400) Zhu K, Zhang Q, Foo LH, Trube A, Ma G, Hu X, Du X, Cowell CT, Fraser DR, Greenfield H. Growth, bone mass, and vitamin D status of Chinese adolescent girls 3 y after withdrawal of milk supplementation. American Journal of Clinical Nutrition 2006 March;83(3):714-21. Diet Intervention or Supplement Study
Ref ID: 1409

(5401) Zhu N, Jacobs DR, Jr., Sidney S, Sternfeld B, Carnethon M, Lewis CE, Shay CM, Sood A, Bouchard C. Fat mass modifies the association of fat-free mass with symptom-limited treadmill duration in the Coronary Artery Risk Development in Young Adults (CARDIA) Study. American Journal of Clinical Nutrition 2011 August;94(2):385-91. Study limited to adults
Ref ID: 2869

(5402) Zhu Q, Bingham GP. Human readiness to throw: the size-weight illusion is not an illusion when picking the best objects to throw. Evolution and Human

Behavior 2011;32(4):288-93. Off topic
Ref ID: 5717

(5403) Zhu WL, Feng NP, Ma J, Wang Y, Ye GJ. [The effect of dietary intervention on lipidemia in school-aged children]. Zhonghua liu xing bing xue za zhi = Zhonghua liuxingbingxue zazhi 2003;24:794-7. Diet Intervention Study
Ref ID: 5102

(5404) Ziegler TR, Evans ME, Fernandez-Estivariz C, Jones DP. Trophic and cytoprotective nutrition for intestinal adaptation,mucosal repair, and barrier function. Annual Review of Nutrition 2003 August;23(1):229-61. Diet Intervention Study
Ref ID: 3791

(5405) Zietz B, Schnabl S, Nerlich M, Schoelmerich J, Schaeffler A. Nutritional composition in different training stages in young female athletes (swimming) and association with leptin, IGF-1 and estradiol. Experimental and Clinical Endocrinology and Diabetes 2009 June;117(6):283-8. Survey or questionnaire
Ref ID: 739

(5406) Zilberman DE, Albala DM. Complications of Robotic Surgery. AUANews 2009 September;14(9):11-20. Off topic
Ref ID: 3792

(5407) Zive MM, McKay H, Frankspohrer GC, Broyles SL, Nelson JA, Nader PR. Infant-feeding practices and adiposity in 4-year-old Anglo-Americans and Mexican-Americans. American Journal of Clinical Nutrition 1992;55(6):1104-8. Diet Intervention Study
Ref ID: 5718

(5408) Zohrabian A. Clinical and economic considerations of antiobesity treatment: A review of orlistat. Clinical Outcomes Research 2010;2(1):63-74. Review article
Ref ID: 3340

(5409) Zoorob R, Buchowski MS, Beech BM, Canedo JR, Chandrasekhar R, Akohoue S, Hull PC. Healthy families study: design of a childhood obesity prevention trial for Hispanic families. Contemporary Clinical Trials 2013 July;35(2):108-21. Inappropriate Study Design
Ref ID: 6156

(5410) Zorbas YG, Kakurin VJ, Kuznetsov NA, Yarullin VL, Andreyev ID, Charapakhin KP. Phosphate deposition capacity of athletes during hypokinesia, phosphate loading, and ambulation. Biological Trace Element Research 2002 March;85(3):211-26. Off topic
Ref ID: 1842

(5411) Zorbas YG, Kakuris KK, Federenko YF, Deogenov VA. Utilization of magnesium during hypokinesia and magnesium supplementation in healthy

subjects. Nutrition 2010 November;26(11-12):1134-8. Diet Intervention or Supplement Study
Ref ID: 2870

(5412) Zouhal H, Jabbour G, Youssef H, Flaa A, Moussa E, Groussard C, Jacob C. Obesity and catecholamine responses to maximal exercise in adolescent girls. European Journal of Applied Physiology 2010 September;110(2):247-54. Acute study
Ref ID: 468

(5413) Zuanassi Macari M, Misael Cavenaghi F, Chinali Komesu M, Orive Lunardi L, Sala MA, Novaes Júnior AB, Grisi MFdM, Taba Júnior M, Scombatti de Souza SL. Immune Cells Depletion During Wound Healing as a Long-Term Effect of Undernutrition. Depleción de Células Inmunocitarias Durante la Curación de una Herida como Efecto de Desnutrición Postnatal. International Journal of Morphology 2005;23(1):25-32. Off topic
Ref ID: 4646

(5414) Zuiani GR, Cavali PTM, Santos MAM, Rossato AJ, Lehoczki MA, Risso Neto MÍ, Veiga IG, Pasqualini W, Landim É. Uso da prótese vertical expansível de titânio para costela no tratamento da cifose congênita em portadores de mielomeningocele torácica. Uso de la prótesis vertical expansible de titanio para costilla en el tratamiento de la cifosis congénita en portadores de mielomeningocele torácico. Use of vertical expandable prosthetic of titanium for the rib for treating congenital kyphosis in thoracic meningomyelocele patients. Coluna/Columna 2009 September;8(3):286-96. Off topic
Ref ID: 4647

(5415) Zunquin G, Theunynck D, Sesboue B, Arhan P, Bougle D. Effects of puberty on the glucido-lipidic balance during obese children's exercise. Applied Physiology Nutrition and Metabolism-Physiologie Appliquee Nutrition et Metabolisme 2006;31(4):442-8. Acute study
Ref ID: 5719

(5416) Zwiauer KF. Prevention and treatment of overweight and obesity in children and adolescents. [Review] [113 refs]. European Journal of Pediatrics 2000 September;159:Suppl-68. Review article
Ref ID: 1958

Supplementary File 4. Study level risk of bias.

Study	Random assignment	Allocation concealment	Blinding (participants/ personnel)	Blinding (outcome assessment)	Incomplete outcome data	Selective reporting	Participants inactive
Alberga et al. [61]	low	Low	high	unclear	high	low	unclear
Alves et al. [62]	low	unclear	high	unclear	low	low	unclear
Elloumi et al. [63]	low	unclear	high	unclear	unclear	low	high
Farpour-Lambert et al. [64]	low	Low	high	low	low	low	low
Hagstromer et al. [65]	low	unclear	high	unclear	high	low	high
Karacabey [66]	low	unclear	high	unclear	unclear	low	low
Kelly et al. [67]	low	unclear	high	unclear	unclear	low	unclear
Kim et al. [68]	low	unclear	high	unclear	unclear	low	low
Kim et al. [69]	low	unclear	high	unclear	high	low	unclear
Meyer et al. [70]	low	unclear	high	unclear	high	low	low
Murphy et al. [71]	low	unclear	high	unclear	unclear	low	unclear
Saygin & Ozturk [72]	low	unclear	high	unclear	unclear	low	unclear
Shaibi et al. [73]	low	unclear	high	unclear	high	low	unclear
Sigal et al. [74]	low	low	high	unclear	low	low	low
Song et al. [75]	low	unclear	high	low	high	low	low
Sun et al. [76]	low	unclear	high	unclear	low	low	unclear

Tan et al. [77]	high	high	high	unclear	low	low	unclear
Watts et al. [78]	low	unclear	high	unclear	unclear	low	unclear
Watts et al. [79]	low	unclear	high	unclear	unclear	low	unclear
Wong et al. [80]	low	unclear	high	unclear	low	low	unclear

Notes: Item-by-item risk of bias results for each study using the Cochrane Risk of Bias Assessment Instrument. Low, low risk of bias, High, high risk of bias, Unclear, unclear risk of bias.

Supplementary File 5. Mixed–effects meta-regression results for changes in BMI in kg/m^2.

Variable	ES (#)	$\beta_1 \pm$ SE	CI (95%)	*Z(p)*
Study Characteristics				
- Year of publication	22	-0.016 ± 0.094	-0.201,0.169	-0.17(0.86)
- Country[a]	UAC	UAC	UAC	UAC
- Control group type[b]	22	0.189±0.764	-1.310,1.687	0.25(0.80)
- Random sequencing[c]	UAC	UAC	UAC	UAC
- Allocation concealment[c]	UAC	UAC	UAC	UAC
- Blinding (part. & pers.) [c]	UAC	UAC	UAC	UAC
- Blinding (outcome asses.) [c]	UAC	UAC	UAC	UAC
- Outcome reporting[c]	14	-0.082±0.534	-1.130,0.965	-0.15(0.88)
Selective reporting[c]	UAC	UAC	UAC	UAC
Previously active[c]	UAC	UAC	UAC	UAC
Sample size estimates[d]	22	0.741±0.618	-0.470,1.951	1.20(0.23)
Type of analysis[e]	15	-0.175±0.506	-1.167,0.8170	-0.35(0.73)
Funding[d]	UAC	UAC	UAC	UAC
Dropouts (%)	11	-0.027±0.022	-0.070,0.016	-1.22(0.22)
Adverse events[d]	UAC	UAC	UAC	UAC
Participant Characteristics				
- Age (years)	21	-0.0003 ± 0.118	-0.231, 0.230	-0.001(0.99)
- Gender[f]	22	UAC	UAC	UAC
- Race/Ethnicity[g]	UAC	UAC	UAC	UAC
- Physical activity changes[h]	UAC	UAC	UAC	UAC
- Pubertal stage[i]	UAC	UAC	UAC	UAC
- Initial BMI (kg/m^2)	19	-0.107±0.093	-0.2885,0.074	-1.16(0.25)
Exercise Characteristics				
- Length (weeks)	22	-0.019±0.053	-0.123,0.085	-0.36(0.72)

- Frequency (days/week)	22	-0.034±0.273	-0.570,0.502	-0.12(0.90)
- Intensity[j]	11	0.946±1.107	-1.224,3.115	0.85(0.39)
- Duration (min/session)[k]	18	-0.023±0.021	-0.065,0.018	-1.09(0.27)
- Minutes/week [k]	18	-0.005±0.006	-0.017,0.007	-0.85(0.39)
- Minutes/week (adj.) [k]	UAC	UAC	UAC	UAC
- Total minutes[k]	18	-0.0003±0.003	-0.0008,0.0002	-1.07(0.28)
- Total minutes (adj.)[k]	UAC	UAC	UAC	UAC
- Compliance (%)	4	0.009±0.020	-0.031,0.049	0.44(0.66)
- Exercise supervision[l]	UAC	UAC	UAC	UAC
- Exercise location[m]	UAC	UAC	UAC	UAC
- Type of participation[n]	22	0.415±0.755	-1.064,1.895	0.55(0.58)
- Type of exercise[o]	UAC	UAC	UAC	UAC

Notes: ES (#), total number of effect sizes in model; SE, standard error; CI, confidence interval; Z, Z-score; p, alpha value; $\beta_{1,}$ beta for predictors; [a], Other versus USA (reference); [b],Other versus Non-intervention (reference); UAC, unable to calculate because of insufficient sample sizes (<4 results for continuous variables or <4 results per group for categorical variables; [c], Low versus high risk (reference); [d], Yes versus no (reference); [e], Per-protocol versus intention-to-treat (reference);[f], Males versus females (reference); [g], Non-hispanic whites versus other (reference); [h], Increase and decrease versus no change (reference); [i], prepubertal and pubertal versus postpubertal (reference); [j], moderate versus high (reference); [k], limited to aerobic exercise as an intervention; [l], supervised and unsupervised versus both (reference); *, [m], Facility and home versus both (reference); [n], Self versus both (reference); [o], Aerobic and weight training versus both (reference).

Supplementary File 6. GRADE results.

Quality assessment							No of patients		Effect		Quality	Importance
No of studies	Design	Risk of bias	Inconsistency	Indirectness	Imprecision	Other considerations		Control	Relative (95% CI)	Absolute		
**Changes in BMI in kilogram meters squared (follow-up mean 13.4 weeks; measured with: Calculated based on the assessment of height and weight; Better indicated by higher values)**												
20	randomised trials	serious[1]	serious[2]	no serious indirectness	no serious imprecision	strong association[3]	575	396	-	MD 1.08 lower (0.52 to 1.64 lower)	⊕⊕⊕O MODERATE	CRITICAL

[1] (1) Allocation concealment rated as unclear in 16 of 20 studies (80%) and high in 1 (5%), (2) all 20 studies considered to be at a high risk of bias with respect to blinding of participants and personnel because it's impossible to blind participants to an exercise intervention, (3) attrition bias classified as high in 6 studies (30%) and unclear in 8 (40%), (4) wthether participants were physically inactive prior to enrollment rated as unclear in 12 studies (60%) and high in 2 (10%).

[2] Used a random-effects model for analysis but overall I-squared statistic for inconsistency was 90.9% (95% confidence interval = 87.6 to 93.4) and diversity statistic was 91.5%, Both demonstrate large inconsistency and diversity.

[3] Despite some of the biases observed as well as overlapping prediction intervals and lack of information on adverse events, the overall findings and trial sequential analysis results suggest that it is highly unlikley that the direction of the overall effect of exercise on BMI in overweight and obese children and adolescents would change, and that in general, exercise is a safe acvtivity for this population group to particpate in.
